# Global Prevalence of Overweight and Obesity in Children and Adolescents: A Systematic Review and Meta-Analysis

**DOI:** 10.1001/jamapediatrics.2024.1576

**Published:** 2024-06-10

**Authors:** Xinyue Zhang, Jiaye Liu, Yinyun Ni, Cheng Yi, Yiqiao Fang, Qingyang Ning, Bingbing Shen, Kaixiang Zhang, Yang Liu, Lin Yang, Kewei Li, Yong Liu, Rui Huang, Zhihui Li

**Affiliations:** 1 Division of Thyroid Surgery, Department of General Surgery, Laboratory of Thyroid and Parathyroid Diseases, Frontiers Science Center for Disease-Related Molecular Network, West China Hospital, Sichuan University, Chengdu, China; 2 Department of Nuclear Medicine, West China Hospital, Sichuan University, Chengdu, China; 3 Department of Respiratory and Critical Care Medicine, Frontiers Science Center for Disease-related Molecular Network, Center of Precision Medicine, Precision Medicine Key Laboratory of Sichuan Province, West China Hospital, Sichuan University, Chengdu, China; 4 Frontiers Medical Center, Tianfu Jincheng Laboratory, Sichuan University, Chengdu, China; 5 Department of Obstetrics and Gynecology, The Second Affiliated Hospital, Chongqing Medical University, Chongqing, China; 6 Department of Gynecology, The First Affiliated Hospital, Chongqing Medical University, Chongqing, China; 7 Department of Pediatrics, West China Hospital, Sichuan University, Chengdu, China; 8 Division of Gastrointestinal Surgery, Department of General Surgery, West China Hospital, Sichuan University, Chengdu, Sichuan, China

## Abstract

**Importance:**

Overweight and obesity in childhood and adolescence is a global health issue associated with adverse outcomes throughout the life course.

**Objective:**

To estimate worldwide prevalence of overweight and obesity in children and adolescents from 2000 to 2023 and to assess potential risk factors for and comorbidities of obesity.

**Data Sources:**

MEDLINE, Web of Science, Embase, and Cochrane.

**Study Selection:**

The inclusion criteria were: (1) studies provided adequate information, (2) diagnosis based on body mass index cutoffs proposed by accepted references, (3) studies performed on general population between January 2000 and March 2023, (4) participants were younger than 18 years.

**Data Extraction and Synthesis:**

The current study was performed in accordance with the Meta-analysis of Observational Studies in Epidemiology guidelines. DerSimonian-Laird random-effects model with Free-Tukey double arcsine transformation was used for data analysis. Sensitivity analysis, meta-regression, and subgroup analysis of obesity among children and adolescents were conducted.

**Main Outcomes and Measures:**

Prevalence of overweight and obesity among children and adolescents assessed by World Health Organization, International Obesity Task Force, the US Centers for Disease Control and Prevention, or other national references.

**Results:**

A total of 2033 studies from 154 different countries or regions involving 45 890 555 individuals were included. The overall prevalence of obesity in children and adolescents was 8.5% (95% CI 8.2-8.8). We found that the prevalence varied across countries, ranging from 0.4% (Vanuatu) to 28.4% (Puerto Rico). Higher prevalence of obesity among children and adolescents was reported in countries with Human Development Index scores of 0.8 or greater and high-income countries or regions. Compared to 2000 to 2011, a 1.5-fold increase in the prevalence of obesity was observed in 2012 to 2023. Substantial differences in rates of obesity were noted when stratified by 11 risk factors. Children and adolescents with obesity had a high risk of depression and hypertension. The pooled estimates of overweight and excess weight in children and adolescents were 14.8% (95% CI 14.5-15.1) and 22.2% (95% CI 21.6-22.8), respectively.

**Conclusions and Relevance:**

This study’s findings indicated 1 of 5 children or adolescents experienced excess weight and that rates of excess weight varied by regional income and Human Development Index. Excess weight among children and adolescents was associated with a mix of inherent, behavioral, environmental, and sociocultural influences that need the attention and committed intervention of primary care professionals, clinicians, health authorities, and the general public.

## Key Points

**Question:**

What is the global prevalence of overweight and obesity in children and adolescents?

**Findings:**

In this systematic review and meta-analysis, we found high prevalence of overweight and obesity in children and adolescents. Various possible risk factors were identified, including inherent, dietary, and environmental factors.

**Meaning:**

These findings suggest that excess weight commonly occurrs in children and adolescents, indicating a need for more control measures incorporating behavioral, environmental, and sociocultural factors.

## Teaser

This systematic review and meta-analysis estimates global prevalence of overweight and obesity in children and adolescents from 2000 to 2023 and evaluates potential risk factors associated therewith.

## Introduction

Overweight and obesity in children and adolescents is an emerging worldwide health concern. Estimates of the prevalence have shown heterogeneity across countries and regions, typically demonstrating a growing trend.^1,2,3,4^ The Global Burden of Disease Obesity Collaborators^5^ reported an overall prevalence of 5.0% for childhood obesity, with 107.7 million children having obesity globally in 2015, and data from the World Obesity Federation^6^ indicate that the rising trend has not yet been stopped, as it estimated that 158 million children and adolescents aged 5 to 19 years would experience obesity in 2020, 206 million in 2025, and 254 million in 2030. Awareness is growing that the epidemiological burden of childhood obesity has posed incremental expenses for both individuals and society.^7^

Obesity could result from multidimensional biological, behavioral, and environmental causes, and unbalanced diet and sedentary habits appearing to be the main drivers.^8,9,10^ Since obesity is a disease in and of itself, managing it becomes more difficult when it coexists with other pathological illnesses including diabetes, cardiovascular disease, and psychological disorders.^11^ Furthermore, childhood overweight and obesity have been shown to persist into adulthood,^12^ and their related adverse outcomes include not only certain health conditions in childhood, but also a greater risk and earlier onset of chronic disorders in later life.^13,14,15^ Hence, there is a demand for routine surveillance of weight status in children and adolescents.

There has been a dearth of studies into the prevalence of obesity among children and adolescents from global perspective since the Non-Communicable Diseases Risk Factor Collaboration^16^ reported an estimation of 5.6% of girls and 7.8% of boys with obesity in 2016. The present study pooled a larger and more recent set of national surveys than previously reported to estimate global prevalence as well as risk factors and comorbidities associated with overweight and obesity among children and adolescents under 18 years old from 2000 to 2023.

## Methods

### Search Strategy and Selection Criteria

The study followed the Meta-analysis of Observational Studies in Epidemiology (MOOSE) reporting guideline. A comprehensive literature search were performed in MEDLINE, Web of Science, Embase, and Cochrane databases between January 1, 2000, and March 31, 2023. The search strategy was structured to include terms pertaining to “overweight,” “obesity,” “excess weight,” “children,” “adolescent,” and “prevalence.” eTable 1 in Supplement 1 contains a full list of the search terms used. The study protocol was registered in PROSPERO (CRD42023483885).

Predefined inclusion criteria were cohort studies, case-control trials, and randomized clinical trials that (1) reported the prevalence of obesity, overweight, and excess weight (overweight and obesity) assessed by body mass index (BMI, calculated as weight in kilograms divided by height in meters squared) cutoffs in children and adolescents younger than 18 years; (2) were conducted in the general population (defined as apparently healthy children or adolescents from school, community, or national demographic census); (3) used standardized instruments, self-reported questionnaires, or clinically structured interviews for assessment of overweight and obesity; and (4) completed data collection between January 2000 and March 2023. We excluded studies of hospitalized patients or a mix of hospitalized and general populations. Title and abstract screening were done by X.Z, J.L, K.L, and C.Y based on the selection criteria. If articles seemed relevant, then the full text was assessed for inclusion.

### Data Extraction and Quality Assessment

Researchers reviewed and extracted data from included studies by using a data extraction form that included country or region, geographic region, publication year, study period, income of country or region, Human Development Index (HDI) of the country or region, study design, sample source, diagnostic reference, sample size, study quality, risk factors, and comorbidities. We also included race and ethnicity in subgroup analyses for comprehensive assessment, and the categories in this study were in accordance with our data sources, using a 4-level variable (Asian, Black, Hispanic, and White). Initial data extraction was done by X.Z, J.L, K.L, and C.Y. For quality assurance, data collected from all the included studies were validated by a second team member (Y.F, Q.N, B.S, or Y.N) for accuracy and completeness against the original source. All discrepancies were reviewed and resolved either by consensus or by a third team member if consensus was not reached. When duplicate data were identified, the duplicate with the smallest sample size or shortest duration of follow-up was excluded. We assessed the quality of included studies using an assessment scale based on the Joanna Briggs Institute Tool in accordance with previous published studies.^15,16^ Studies scoring 1 to 3 were defined as low quality, 4 to 6 as average quality, and 7 to 9 as high quality. Studies were not excluded regardless of their quality score to increase transparency and to ensure all available evidence in this area was reported.

### Statistical Analysis

All data analysis was performed using R version 4.0.0 (R Foundation) with the meta and metafor statistical packages. A 95% CI was estimated using the Wilson score method, and the pooled prevalence was calculated using the DerSimonian-Laird random-effects model with Free-Tukey double arcsine transformation. Heterogeneity among the included studies was evaluated through the Cochran *Q* and *I*^2^ statistics. Given the anticipated heterogeneity in global data, a random-effects model was used to estimate the prevalence of obesity, overweight, and excess weight. Sensitivity analyses were conducted by performing a set of leave-1-out diagnostic tests focusing on the significant heterogeneity associated with obesity where individual studies were systematically removed from the meta-analysis and the pooled-effect estimate recalculated. The results were then verified by using a build-in function in metafor. As sensitivity analysis was unable to decrease the heterogeneity, meta-regression was performed by using a mixed-effects model. Univariable and multivariable meta-regression (multimodel inference) were performed by using the dmetar package in synthesizing evidence from multiple studies and exploring heterogeneity. The random-effects weighting method was used for assigning weights in meta-regression. To assess the potential confounding effects of heterogeneity, subgroup analyses were conducted. Characteristics of participants were compared with the prevalence of obesity to determine the pooled estimates of risk factors and comorbidities. IQR was defined as the difference between the first and the third quartile. *P* < .05 was considered as significant difference.

## Results

**Figure 1.**
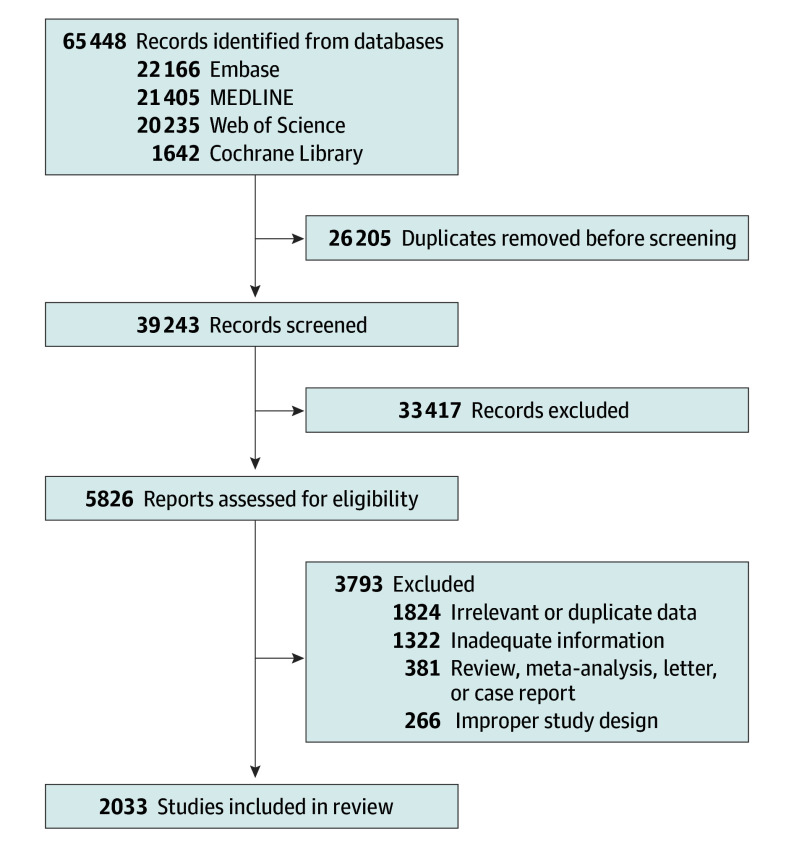
Flow Diagram of Study Selection Process

The search identified 65 448 records, 39 243 of which were retained after removing duplicates. Titles and abstracts were screened, resulting in the exclusion of 33 417 ineligible records. Full texts of the remaining 5826 records were assessed for eligibility, and 3793 were excluded. Overall, 2033 eligible studies involving 45 890 555 children and adolescents from 154 countries or regions were included in the final analysis (Figure 1).

### Study Characteristics and Risk of Bias

The characteristics and quality assessment score of all 2033 included studies are presented in eTables 2-5 in Supplement 1. The sample size ranged from 30 to 3 190 300 participants. The cross-sectional design was used in most of the included research. The mean or median age and sex of participants was reported in 737 and 1090 studies. The median (IQR) age was 10.0 (7.1-12.5) years, and the median (IQR) proportion of participants who were female was 49.64% (48.1-51.5).

### Prevalence of Obesity Among Children and Adolescents

The prevalence of obesity in children and adolescents was reported by 1668 studies comprising 44 414 245 individuals from 152 countries or regions (eTable 3 in Supplement 1). A total of 4 519 587 participants were diagnosed as having obesity with a pooled prevalence of 8.5% (95% CI, 8.2-8.8; *I*^2^, 99.9%). To gain a deeper understanding of the heterogeneity, we conducted a sensitivity analysis by performing a set of leave-1-out diagnostic tests (eTables 6-7 in Supplement 1). After removing the outliers, the pooled estimate of obesity for children and adolescents was 8.3% (95% CI, 8.0-8.6; *I*^2^, 99.9%). To further explore the source of heterogeneity, meta-regression analysis was performed. Our univariate meta-regression model indicated that country or region (*R*^2^, 66.6%; *P* < .001), geographic region (*R*^2^, 46.8%; *P* < .001), diagnostic reference (*R*^2^, 0; *P* < .001), HDI level (R^2^, 41.9%; *P* < .001), sample size (*R*^2^, 0.01%; *P* < .001), sample source (*R*^2^, 2.4%; *P* < .001), and publication year (*R*^2^, 1.4%; *P* = .02) were associated with heterogeneity, while study design was not (*R*^2^ = 4.4%; *P* = .63) (eTable 8 in Supplement 1). By performing multivariable meta-regression, it was found that the geographic region, income level of the country or region, sample sources, diagnostic reference, and sample size showed the highest predictor importance of 99.99% (eTable 9 in Supplement 1).

In subgroup analyses, prevalence of obesity varied substantially across different countries and regions, from 0.4% (Vanuatu, 95% CI, 0.1-0.8) to 28.4% (Puerto Rico, 95% CI, 23.6-33.4). Stratified data by geographic regions, the highest obesity prevalence was found in Polynesia with an estimated rate of 19.5% (95% CI, 16.1-23.1), and the lowest prevalence appeared in Middle Africa (2.4%; 95% CI, 1.8-3.0). The prevalence of obesity in countries and regions with HDI scores of 0.8 or greater was 9.5% (95% CI, 9.2-9.8), whereas countries and regions with HDI scores lower than 0.8 showed a significantly lower prevalence of 7.6% (95% CI, 7.3-7.9; *P* < .001). Likewise, there was a positive association between income of countries and regions and prevalence of children and adolescents’ obesity, with high-income countries showing the highest prevalence (9.3%; 95% CI, 9.0-9.6) and low-income countries exhibiting the lowest (3.6%; 95% CI, 2.5-4.8; *P* < .001). We also discovered significant disparity among race and ethnicity, with the highest prevalence appearing in the Hispanic population (23.55; 95% CI, 20.66-26.56) and the lowest appearing in the Asian population (10.0%; 95% CI 8.73-11.29; *P* < .001). Regarding sample sources, participants from medical institutions presented the highest prevalence of 13.6% (95% CI, 12.2-15.1), although sample sources drawn from databases contained most participants. Considering the diagnostic references for assessing obesity, 466 studies used the World Health Organization reference^17^ (8.6%; 95% CI, 7.9-9.3), 807 used the International Obesity Task Force reference^18^ (5.4%; 95% CI, 5.1-5.7), 453 used the US Centers for Disease Control and Prevention reference^19^ (14.5%; 95% CI, 13.6-15.3), and 282 studies used various national references (9.7%; 95% CI, 9.0-10.3). A pattern of decreased prevalence was found in studies having more than 5000 participants (7.7%; 95% CI, 7.1-8.2) than those with fewer than 5000 participants (8.7%; 95% CI, 8.4-9.1; *P* < .001). Moreover, studies performed from 2000 to 2011 showed significantly lower rates (7.1%; 95% CI, 6.8-7.3) than those performed from 2012 to 2023 (11.3%; 95% CI, 10.8-11.8; *P* < .001) (Table 1; eTable 10 in Supplement 1.

**Table 1.** Subgroup Analysis for Obesity Prevalence Among Children and Adolescents

Subgroup	Studies, No.	Events, No.	Total, No.	Prevalence (95% CI)	*P* value	*I*^2^, %
Country						
Albania	2	671	8069	8.72 (6.67-11.03)	<.001	90.1
Algeria	3	119	3837	3.53 (1.82-5.76)	<.001	89.0
Argentina	13	2516	25 261	11.53 (9.22-14.07)	<.001	95.8
Australia	57	14 105	220 141	5.96 (5.39-6.55)	<.001	96.6
Austria	4	315	8940	3.66 (1.78-6.15)	<.001	96.4
Bahamas	1	279	1308	21.33 (19.15-23.59)	<.001	NA
Bahrain	3	295	3350	9.74 (5.48-15.04)	<.001	94.4
Bangladesh	8	966	18 088	7.76 (3.96-12.68)	<.001	98.9
Barbados	1	214	1504	14.23 (12.51-16.04)	<.001	NA
Belgium	15	870	39 466	2.22 (1.48-3.10)	<.001	96.2
Benin	2	33	3398	1.30 (0.11-3.62)	<.001	92.8
Bhutan	1	2	392	0.51 (0.01-1.53)	<.001	NA
Bolivia	4	446	7020	3.87 (1.45-7.35)	<.001	97.0
Bosnia and Herzegovina	2	619	6108	10.52 (4.05-19.54)	<.001	99.0
Botswana	1	35	707	4.95 (3.46-6.68)	<.001	NA
Brazil	92	31 043	333 397	8.65 (7.59-9.77)	<.001	99.1
Brunei Darussalam	1	319	1824	17.49 (15.78-19.27)	<.001	NA
Bulgaria	6	1130	14 734	6.29 (2.53-11.57)	<.001	99.2
Burkina Faso	3	185	8431	2.20 (0.66-4.57)	<.001	94.9
Burundi	1	52	3493	1.49 (1.11-1.92)	<.001	NA
Cameroon	7	334	13 385	2.32 (1.74-2.97)	<.001	79.2
Canada	47	380 348	3 478 991	10.43 (9.26-11.66)	<.001	99.5
Chile	13	4894	173 378	15.94 (10.03-22.91)	<.001	99.7
China mainland	148	410 959	5 986 764	7.77 (7.11-8.45)	<.001	99.9
Colombia	6	889	25 937	4.70 (3.02-6.73)	<.001	96.5
Comoros	1	173	2699	6.41 (5.52-7.37)	<.001	NA
Congo	2	286	12 922	1.95 (0.81-3.56)	<.001	96.7
Costa Rica	1	49 128	347 366	14.14 (14.03-14.26)	<.001	NA
Cote d’Ivoire	2	71	4545	1.76 (0.72-3.22)	<.001	88.1
Croatia	8	1486	19 623	5.77 (2.28-10.71)	<.001	99.3
Cyprus	8	1522	21 867	6.54 (5.42-7.76)	<.001	90.7
Czech	7	1488	48 743	4.35 (1.87-7.78)	<.001	99.4
Denmark	12	2047	68 426	3.07 (1.58-5.02)	<.001	99.3
Djibouti	2	226	3249	6.93 (3.75-11)	<.001	94.2
Dominican Republic	1	117	954	12.26 (10.26-14.42)	<.001	NA
East Timor	1	20	1631	1.23 (0.74-1.82)	<.001	NA
Ecuador	4	1435	12 962	12.28 (4.03-24.12)	<.001	98.9
Egypt	10	1608	15 845	13.33 (10.72-16.17)	<.001	95.2
El Salvador	1	10 087	111 991	9.01 (8.84-9.18)	<.001	NA
Estonia	4	188	10 275	2.09 (0.97-3.60)	<.001	94.4
Ethiopia	11	336	18 012	2.70 (1.61-4.06)	<.001	94.7
Fiji	3	684	12 257	6.07 (4.52-7.84)	<.001	89.8
Finland	8	838	31 278	2.88 (2.45-3.34)	<.001	76.5
France	23	5411	162 311	3.93 (3.17-4.76)	<.001	97.9
French Polynesis	1	420	1902	22.08 (20.25-23.97)	<.001	NA
Gabon	1	129	3482	3.70 (3.10-4.36)	<.001	NA
Gambia	1	114	3360	3.39 (2.81-4.03)	<.001	NA
Georgia	2	281	3226	8.60 (7.65-9.61)	<.001	0
Germany	39	7349	168 736	4.35 (3.74-5.01)	<.001	97.3
Ghana	13	686	20 425	7.16 (4.06-11.04)	<.001	98.7
Greece	59	33 519	418 004	8.19 (7.54-8.86)	<.001	97.5
Greenland	2	34	1501	2.18 (1.30-3.28)	<.001	39.8
Guatemala	1	71	363	19.56 (15.63-23.81)	<.001	NA
Guinea	1	80	3216	2.49 (1.98-3.06)	<.001	NA
Honduras	1	112	2554	4.39 (3.62-5.22)	<.001	NA
Hong Kong	11	13 180	256 924	5.32 (4.59-6.11)	<.001	96.0
Hungary	11	3129	43 224	6.30 (4.36-8.58)	<.001	98.7
Iceland	4	438	14 284	2.67 (1.66-3.90)	<.001	92.4
India	89	14 355	318 874	5.63 (4.92-6.39)	<.001	98.7
Indonesia	12	10 682	186 391	10.18 (8.71-11.76)	<.001	98.0
Iran	79	169 562	460 7462	8.28 (7.83-8.75)	<.001	99.6
Iraq	5	617	21 340	5.09 (2.86-7.91)	<.001	98.4
Ireland	15	4289	65 512	5.78 (5.00-6.60)	<.001	93.5
Israel	9	24 855	612 186	6.41 (4.82-8.22)	<.001	99.8
Italy	55	30 477	282 659	8.49 (6.77-10.38)	<.001	99.6
Jamaica	1	107	1061	10.08 (8.34-11.97)	<.001	NA
Japan	14	3071	86 053	3.9 0(2.84-5.12)	<.001	98.5
Jordan	12	1508	14 367	9.09 (6.49-12.08)	<.001	97.1
Kazakhstan	2	241	6388	2.51 (0-9.86)	<.001	99.4
Kenya	4	143	2826	5.48 (3.91-7.29)	<.001	70.5
Kiribati	1	117	1582	7.40 (6.16-8.74)	<.001	NA
Kuwait	11	4506	64 261	20.49 (11.68-31.01)	<.001	99.8
Kyrgyzstan	1	161	5958	2.70 (2.31-3.13)	<.001	NA
Laos	1	36	1644	2.19 (1.53-2.96)	<.001	NA
Latvia	5	537	13 122	2.85 (0.32-7.59)	<.001	99.2
Lebanon	7	1440	20 131	6.84 (5.32-8.52)	<.001	92.2
Liberia	1	55	3259	1.69 (1.27-2.16)	<.001	NA
Libya	4	1146	9251	10.22 (7.54-13.26)	<.001	90.3
Lithuania	5	912	19 529	3.82 (0.90-8.66)	<.001	99.5
Luxemburg	1	90	3904	2.31 (1.86-2.80)	<.001	NA
Macedonia	4	508	11 931	5.86 (1.98-11.60)	<.001	99.2
Malawi	2	298	7134	2.74 (0.01-9.72)	<.001	99.3
Malaysia	20	14 446	126 080	10.90 (9.84-12.01)	<.001	96.1
Mali	1	101	4591	2.20 (1.79-2.65)	<.001	NA
Malta	5	970	6904	12.67 (9.28-16.51)	<.001	94.7
Mauritania	1	69	2028	3.40 (2.65-4.24)	<.001	NA
Mauritius	3	235	2996	6.87 (4.09-10.29)	<.001	87.7
Mexico	37	11 205	69 829	16.56 (14.05-19.22)	<.001	98.7
Mongolia	1	67	3707	1.81 (1.40-2.26)	<.001	NA
Montenegro	3	583	6999	9.26 (4.09-16.22)	<.001	98.3
Morocco	5	1241	14 974	7.78 (2.87-14.80)	<.001	99.4
Mozambique	1	408	9721	4.20 (3.81-4.60)	<.001	NA
Multiple countries	15	12178	210 258	6.36 (4.31-8.78)	<.001	99.7
Namibia	2	79	3781	2.09 (1.65-2.57)	<.001	0
Nepal	7	212	7945	3.36 (0.80-7.48)	<.001	98.0
the Netherlands	25	7303	252 778	3.23 (2.38-4.19)	<.001	99.2
New Zealand	10	36 378	226 167	15.33 (10.95-20.28)	<.001	99.8
Niger	1	179	5123	3.49 (3.01-4.01)	<.001	NA
Nigeria	13	1672	49 525	4.02 (2.67-5.62)	<.001	98.0
Norway	18	1187	53 178	2.37 (2.06-2.70)	<.001	78.2
Pakistan	12	1515	24 011	10.37 (7.89-13.15)	<.001	96.8
Palestine	8	488	7463	5.68 (3.19-8.82)	<.001	96.2
Peru	4	35 815	2 341 760	6.25 (2.06-12.47)	<.001	99.4
Philippines	1	173	6162	2.81 (2.41-3.24)	<.001	NA
Poland	39	3675	94 598	4.32 (3.55-5.15)	<.001	97.1
Portugal	38	9655	121 395	8.39 (7.22-9.64)	<.001	98.2
Puerto Rico	4	1339	5211	28.35 (23.57-33.39)	<.001	90.6
Qatar	3	700	11 824	9.13 (5.22-13.99)	<.001	98.1
Republic of Marshall Islands	1	167	3271	5.11 (4.38-5.89)	<.001	NA
Romania	10	6096	55 265	6.46 (3.56-10.15)	<.001	99.6
Russia	5	358	19 758	2.28 (0.63-4.88)	<.001	98.9
Rwanda	1	95	4116	2.31 (1.87-2.79)	<.001	NA
Samoa	1	467	2418	19.31 (17.76-20.91)	<.001	NA
San Marino	1	37	303	12.21 (8.75-16.15)	<.001	NA
Saudi Arabia	29	9734	72 356	16.93 (13.7-20.42)	<.001	99.3
Senegal	1	49	6062	0.81 (0.60-1.05)	<.001	NANA
Serbia	10	2035	32 643	8.21 (6.18-10.49)	<.001	97.7
Seychelles	4	1759	30 478	6.60 (3.51-10.52)	<.001	98.3
Sierra Leone	1	446	4698	9.49 (8.67-10.35)	<.001	NA
Singapore	3	649	9870	6.55 (6.07-7.05)	<.001	0
Slovakia	2	55	5078	1.23 (0.44-2.39)	<.001	87.6
Slovenia	11	2501	46 166	4.97 (3.78-6.32)	<.001	97.4
Solomon Islands	1	38	1421	2.67 (1.89-3.58)	<.001	NA
South Africa	24	2565	45 509	6.20 (4.49-8.16)	<.001	98.3
South Korea	26	633 630	5 644 482	8.39 (7.68-9.14)	<.001	99.8
Spain	54	392 129	2 821 506	9.28 (8.27-10.33)	<.001	99.5
Sri Lanka	4	667	15 077	3.34 (1.53-5.80)	<.001	94.7
Sudan	3	168	2344	7.11 (2.84-13.07)	<.001	95.4
Suriname	1	167	1453	11.49 (9.9-13.19)	<.001	NA
Sweden	33	12 744	1 131 530	3.18 (2.45-4.01)	<.001	99.4
Switzerland	10	965	31 991	3.24 (2.08-4.64)	<.001	97.5
Syria	3	1231	7292	10.99 (3.39-22.19)	<.001	99.1
Taiwan	21	8752	70 568	12.05 (9.99-14.28)	<.001	98.6
Tajikistan	1	42	2822	1.49 (1.07-1.97)	<.001	NA
Tanzania	7	505	12 740	4.88 (3.35-6.67)	<.001	92.8
Thailand	15	3775	42 954	9.80 (7.75-12.07)	<.001	98.1
Togo	2	41	3862	1.26 (0.46-2.44)	<.001	76.7
Tonga	3	998	4602	18.33 (12.1-25.52)	<.001	96.8
Trinidad and Tobago	2	450	2699	13.05 (5.47-23.22)	<.001	95.4
Tunisia	2	123	2138	5.75 (4.80-6.78)	<.001	0
Turkey	55	10 879	152 633	6.97 (5.79-8.25)	<.001	98.9
Turkmenistan	1	1055	9768	10.80 (10.19-11.42)	<.001	NA
Uganda	1	55	4212	1.31 (0.98-1.67)	<.001	NA
Ukraine	5	1406	40 633	2.56 (1.11-4.57)	<.001	99.0
United Arab Emirates	7	5422	31 845	15.62 (13.86-17.45)	<.001	82.1
United Kingdom	53	163 750	1 294 718	7.63 (6.40-8.95)	<.001	99.6
US	262	1 849 465	10, 411 152	18.57 (18.03-19.12)	<.001	99.8
Vanuatu	1	4	1119	0.36 (0.08-0.81)	<.001	NA
Vietnam	13	2617	30 800	6.91 (3.85-10.77)	<.001	99.2
Yemen	1	975	10 924	8.93 (8.40-9.47)	<.001	NA
Zambia	1	397	11 677	3.40 (3.08-3.74)	<.001	NA
Zimbabwe	2	207	5379	5.05 (1.44-10.64)	<.001	97.3
Geographic region						
Southern Europe	248	474 682	3 770 379	8.42 (7.84-9.01)	<.001	99.6
Northern Africa	27	4405	48 389	9.22 (7.32-11.3)	<.001	98.2
South America	133	76 759	2 914 148	9.38 (8.24-10.59)	<.001	99.8
Australia and New Zealand	67	50 483	446 308	6.99 (5.74-8.36)	<.001	99.6
Western Europe	124	24 042	687 349	3.79 (3.38-4.22)	<.001	98.7
Caribbean	10	2506	12 737	19.22 (15.1-23.7)	<.001	97.3
Western Asia	152	62 734	1 040 310	9.94 (9.03-10.88)	<.001	99.5
Southern Asia	125	18 163	391 407	5.79 (5.17-6.45)	<.001	98.6
Western Africa	43	3781	122 523	3.95 (3.13-4.87)	<.001	98.2
Southern Africa	27	2679	49 997	5.76 (4.24-7.50)	<.001	98.2
South-Eastern Asia	146	202 279	5 014 818	8.71 (8.26-9.17)	<.001	99.6
Eastern Europe	85	17 337	322 033	4.58 (3.75-5.50)	<.001	99.3
Eastern Africa	34	4384	105 992	4.12 (3.30-5.04)	<.001	97.9
Middle Africa	10	749	29 789	2.36 (1.83-2.96)	<.001	89.1
Northern America	311	2 229 847	13 891 644	17.17 (16.59-17.75)	<.001	99.9
Eastern Asia	221	1 069 659	12 048 498	7.78 (7.24-8.32)	<.001	99.9
Central America	41	70 603	532 103	15.85 (14.23-17.55)	<.001	99.3
Northern Europe	157	186 930	2 701 852	4.55 (3.57-5.63)	<.001	99.9
Melanesia	5	726	14 797	3.79 (1.84-6.40)	<.001	97.5
Polynesia	5	1885	8922	19.45 (16.06-23.07)	<.001	94.2
Central Asia	12	2004	37 676	4.28 (2.46-6.58)	<.001	98.9
Micronesia	10	772	12 316	5.80 (3.95-7.98)	<.001	95.4
Not applicable	15	12 178	210 258	6.36 (4.31-8.78)	<.001	99.7
HDI						
<0.8	1047	871 742	17 166 470	7.56 (7.28-7.85)	<.001	99.8
≥0.8	946	3 635 667	27 037 517	9.50 (9.19-9.82)	<.001	99.9
Not applicable	15	12 178	210 258	6.36 (4.31-8.78)	<.001	99.7
Country or region income						
High income	1129	3 692 176	28 815 921	9.29 (8.95-9.64)	<.001	99.9
Upper-middle income	495	588 196	9 709 961	8.50 (8.02-8.99)	<.001	99.9
Lower-middle income	333	221 821	5 562 640	6.35 (6.09-6.62)	<.001	99.2
Low income	36	5216	115 465	3.60 (2.54-4.83)	<.001	99.1
Not applicable	15	12 178	210 258	6.36 (4.31-8.78)	<.001	99.7
Race and ethnicity^a^						
Asian	23	9414	91 834	9.97 (8.73-11.29)	<.001	91.6
Black	53	21 917	129 800	16.64 (14.06-19.39)	<.001	99.2
Hispanic	35	250 747	1 141 081	23.55 (20.66-26.56)	<.001	99.9
White	66	61 705	505 895	12.28 (11.19-13.42)	<.001	98.8
Sample source						
Database	681	3 079 529	27 464 011	7.40 (7.01-7.79)	<.001	99.9
School	1026	1 073 740	8 985 888	8.66 (8.23-9.10)	<.001	99.8
Community	192	233 742	6 721 847	8.90 (8.32-9.50)	<.001	99.8
Medical institution	109	132 576	1 242 499	13.59 (12.18-15.05)	<.001	99.8
Study design						
Cross-sectional	1855	3 797 742	38 296 246	8.38 (8.10-8.67)	.004	99.9
Longitudinal	44	492 403	3 216 207	9.70 (8.15-11.36)	.004	99.9
Cohort	86	64 556	1 618 514	9.53 (7.82-11.40)	.004	99.9
Randomized clinical trial	12	2161	16 279	10.99 (8.06-14.30)	.004	97.3
Prospective	10	162 687	1 266 740	9.98 (5.27-15.96)	.004	99.9
Case-control	1	38	259	14.67 (10.61-19.26)	.004	NA
Diagnostic reference						
WHO	466	730 348	8 703 325	8.59 (7.94-9.26)	<.001	99.9
IOTF	807	661 187	8 215 613	5.41 (5.11-5.73)	<.001	99.7
CDC	453	1 877 278	14 592 246	14.46 (13.63-15.32)	<.001	100.0
National reference	282	1 250 774	12 903 061	9.67 (9.02-10.33)	<.001	99.9
Sample size						
≤5000	1558	195 777	2 356 611	8.74 (8.41-9.07)	<.001	98.8
>5000	450	4 323 810	42 057 634	7.67 (7.12-8.23)	<.001	100.0
Study period						
2000-2011	1232	1 318 508	21 086 914	7.05 (6.80-7.32)	<.001	99.8
2012-2023	681	211 0031	15 956 095	11.31 (10.81-11.81)	<.001	99.9

Abbreviations: CDC, US Centers for Disease Control and Prevention; HDI, Human Development Index; IOTF, International Obesity Task Force; NA, not applicable; WHO, World Health Organization.

^a^ Race and ethnicity data were collected via in accordance with the data sources and reported for comprehensive assessment.

### Analysis of Risk Factors Associated With Obesity Among Children and Adolescents

To gain a more comprehensive view of obesity in children and adolescents, further analysis regarding potential risk factors were performed (Table 2; eTable 11 in Supplement 1). Results indicated that a significant difference in the prevalence of obesity was found in the pooled estimate by age (0-5, 6-12, or 13-18 years; 8.5% vs 9.4% vs 6.9%, respectively; *P* < .001), sex (male or female; 9.4% vs 7.5%, respectively; *P* < .001), school type (public or private; 6.5% vs 11.6%, respectively; *P* < .001), maternal weight status (obesity or nonobesity; 15.9% vs 8.1%, respectively; *P* = .001), breakfast (having breakfast daily or usually skipping breakfast; 7.1% vs 10.0%, respectively; *P* = .03), numbers of meals per day (>3 or ≤3; 3.3% vs 11.6%, respectively; *P* = .008), hours of playing on the computer per day (≥2 or <2 hours; 11.9% vs 5.5%, respectively; *P* = .01), maternal smoking in pregnancy (smoking or never; 7.7% vs 4.7%, respectively; *P* = .006), birth weight (low, normal, or high; 6.2% vs 9.2% vs 12.8%, respectively; *P* = .005), physical activity (regular or irregular; 7.7% vs 12.1%, respectively; *P* = .006), and nightly sleep duration (<10 or ≥10 hours; 13.7% vs 7.2%, respectively; *P* = .03). Minimal differences were observed among other factors.

**Table 2.** Analysis of Risk Factors Associated With Obesity in Children and Adolescents

Risk factor	Studies, No.	Events, No.	Total, No.	Prevalence (95% CI)	*P* value	*I*^2^, %
Age, y						
0-5	246	524 593	7 839 060	8.46 (7.64-9.32)	<.001	99.9
6-12	816	931 446	8 322 894	9.36 (8.88-9.85)		99.8
13-18	515	10,22 690	10 787 040	6.92 (6.51-7.34)		99.8
Sex						
Male	1070	955 224	9 909 202	9.38 (8.95-9.81)	<.001	99.8
Female	1093	616 419	9 365 604	7.50 (7.17-7.83)		99.8
Residential location						
Rural	74	39 680	1 388 709	6.25 (5.12-7.48)	.14	99.8
Urban	83	1 146 682	3 005 017	8.12 (6.75-9.60)		99.9
Suburban	14	9979	152 193	7.94 (4.72-11.90)		99.8
School type						
Public	42	5583	121 865	6.53 (4.94-8.32)	<.001	99.0
Private	40	4622	84 325	11.63 (9.64-13.78)		98.5
Paternal weight status						
Obesity	13	1677	8378	12.22 (6.94-18.69)	.11	98.4
Nonobesity	13	3241	32 375	7.06 (3.97-10.93)		99.3
Maternal weight status						
Obesity	26	3362	22 637	15.92 (12.24-19.97)	.001	98.2
Nonobesity	26	14 385	146 859	8.06 (5.38-11.23)		99.7
Maternal diabetes						
With diabetes	6	233	7351	13.29 (3.80-26.87)	.32	96.1
Without diabetes	6	7503	945 998	7.52 (2.48-14.93)		99.7
Gestational diabetes						
With gestational diabetes	5	411	2004	17.83 (9.89-27.44)	.43	95.1
Without gestational diabetes	5	3612	19 981	13.83 (8.32-20.45)		99.1
Paternal diabetes						
With diabetes	2	254	13 044	11.43 (0-49.08)	.73	98.8
Without diabetes	2	6800	931 876	6.20 (0-30.41)		99.3
Maternal education						
Less than secondary	30	2098	22 838	8.81 (6.12-11.87)	.63	97.4
Secondary	29	6942	49 769	9.83 (7.79-12.06)		97.4
Tertiary	33	7916	47 855	11.60 (9.04-14.43)		98.6
Paternal education						
Less than secondary	22	941	13 490	6.78 (4.83-9.01)	.52	92.7
Secondary	21	1204	16 386	8.73 (6.71-10.98)		93.4
Tertiary	20	1495	28 047	8.27 (5.66-11.32)		97.8
Mother occupation						
Employed	20	5601	44 643	9.80 (6.99-13.02)	.79	98.3
Unemployed	20	2537	26 267	8.32 (5.16-12.05)		98.6
Father occupation						
Employed	7	882	18 430	6.50 (3.67-10.05)	.85	98.1
Unemployed	7	71	1069	5.42 (1.87-10.42)		85.7
Parental marriage status						
Married or cohabiting	4	4050	580 120	2.43 (1.28-3.92)	.92	99.1
Never married, widowed, or divorced	4	3328	391 895	1.35 (0.17-3.22)		92.1
Caregivers						
Parents	2	58	662	10.09 (4.79-16.98)	.79	67.1
Grandparents	2	35	217	6.80 (0-35.40)		93.7
No. of children in the family						
1	12	2525	14 718	10.32 (5.50-16.39)	.28	98.9
>1	12	3292	38 658	6.88 (3.78-10.78)		99.4
Birth order						
First born	7	923	24 389	5.28 (2.63-8.77)	.63	98.9
Not first born	7	924	29 578	4.41 (2.60-6.66)		98.1
Socioeconomic status of family						
Low	33	4691	96 335	6.91 (5.15-8.90)	.71	98.4
Middle	30	5498	114 863	7.96 (6.52-9.53)		98.0
High	30	5510	109 564	7.56 (6.13-9.12)		96.6
Way of going to school						
Walk	12	399	6295	6.40 (3.98-9.31)	.16	93.9
Bike	4	48	832	5.94 (2.92-9.87)		74.1
Car	9	335	3014	11.29 (6.66-16.92)		94.5
Breakfast						
Daily	24	3902	54 224	7.08 (5.20-9.23)	.03	98.7
Usually skipped	24	1332	14 133	9.96 (7.94-12.16)		92.6
No. of meals per day						
>3	4	75	2870	3.26 (1.32-5.97)	.008	88.3
≤3	5	169	1823	11.64 (5.77-19.16)		94.6
Watching TV while eating						
Usually	6	364	1640	28.17 (5.37-59.71)	.56	99.3
Never	6	162	1819	18.29 (4.48-38.35)		98.5
Hours of watching TV per d						
≥2 h	19	5970	42 021	18.61 (13.58-24.23)	.06	99.3
<2 h	19	10 419	87 570	12.57 (9.16-16.44)		98.9
Hours of playing on the computer per d						
≥2 h	3	593	4466	11.88 (8.20-16.12)	.01	89.9
<2 h	3	1025	13 258	5.48 (2.94-8.73)		96.6
Passive smoking						
Exposed	8	3431	30 454	9.68 (6.02-14.09)	.27	98.5
Not exposed	8	3902	70 516	6.50 (3.11-11.01)		99.7
Maternal smoking in pregnancy						
Smoking	22	855	11 101	7.66 (5.75-9.80)	.006	92.5
Never	22	3297	66 990	4.70 (3.50-6.07)		98.3
Gestational weight gain						
Inadequate	4	807	6561	11.09 (6.49-16.70)	.63	96.3
Adequate	4	2538	17 038	11.19 (6.21-17.37)		98.5
Excessive	4	5553	31 198	15.85 (7.75-26.12)		99.6
Maternal age at birth, y						
≥25	7	1294	37 181	5.75 (2.90-9.47)	.37	99.0
<25	7	1077	16 680	7.95 (4.73-11.90)		98.3
Term of delivery						
Premature	14	4025	25 546	10.92 (8.45-13.66)	.65	95.4
Full term	14	38 255	260 305	10.32 (8.09-12.77)		99.6
Type of delivery						
Cesarean	14	1451	17 265	9.94 (7.51-12.67)	.29	96.3
Vaginal	14	1714	21 852	8.35 (6.79-10.05)		94.1
Birth weight						
Low (≤2499 g)	17	1958	18 413	6.22 (4.26-8.50)	.005	95.8
Normal (2500-4000 g)	17	37 096	251 645	9.16 (7.14-11.40)		99.6
High (≥4001 g)	15	5345	24 954	12.82 (9.56-16.46)		97.6
Duration of breastfeeding						
≥3 mo	10	2553	21 255	8.51 (5.00-12.82)	.68	98.9
<3 mo	7	1768	18 803	9.37 (8.26-10.55)		79.5
Breastfeeding amount						
None (full formula)	12	5113	38 862	10.45 (7.25-14.13)	.29	99.0
Mixed	8	9030	83 335	10.07 (6.95-13.67)		99.5
Full (no formula)	8	2521	31 312	7.88 (6.05-9.93)		96.3
Antibiotic exposure						
Exposed	6	29 079	218 911	8.66 (5.15-12.96)	.84	99.1
Not exposed	6	20 079	160 943	7.51 (2.38-14.94)		99.6
Physical activity						
Regular exercise	21	3118	32 947	7.65 (5.65-9.92)	.006	97.7
No regular exercise	21	2742	24 289	12.08 (9.88-14.48)		96.3
Nightly sleep duration						
<10 h	14	4217	28 615	13.68 (8.99-19.15)	.03	99.2
≥10 h	10	1404	16 849	7.23 (4.42-10.66)		98.2

### Comorbidities of Obesity Among Children and Adolescents

Eight comorbidities associated with obesity among children and adolescents were investigated (Table 3; eTable 12 in Supplement 1). There were 26 studies reporting on hypertension in children and adolescents with obesity, with a pooled rate of 28.0% (95% CI, 20.2-36.6). In addition, 13 studies documented dental caries (17.9%; 95% CI, 12.6-23.8), 8 included vitamin D deficiency (11.6% 95% CI, 5.4-19.9), 7 included asthma (18.8%; 95% CI, 12.5-26.2), 3 reported on diabetes (1.2%; 95% CI, 0.2-3.0), 3 included flatfoot (26.1%; 95% CI, 6.7-52.2), 2 reported on anxiety (25.1%; 95% CI, 0-94.2), and 2 included depression (35.2%; 95% CI, 0.4-87.0).

**Table 3.** Analysis of Comorbidities for Obesity in Children and Adolescents

Comorbidity	Studies, No.	Events, No.	Total, No.	Prevalence (95% CI)	*I*^2^, %
Hypertension	26	5583	195,22	28.02 (20.16-36.61)	99.2
Dental caries	13	36 472	531 470	17.88 (12.61-23.83)	99.8
Vitamin D deficiency	8	705	4546	11.63 (5.36-19.86)	98.3
Asthma	7	844	7696	18.84 (12.46-26.16)	97.7
Diabetes	3	62	5916	1.23 (0.23-2.98)	95.1
Flatfoot	3	69	354	26.08 (6.69-52.19)	95.8
Anxiety	2	316	1173	25.08 (0-94.18)	99.9
Depression	2	379	1166	35.24 (0.44-86.90)	99.7

### Prevalence of Overweight and Excess Weight in Children and Adolescents

**Figure 2.**
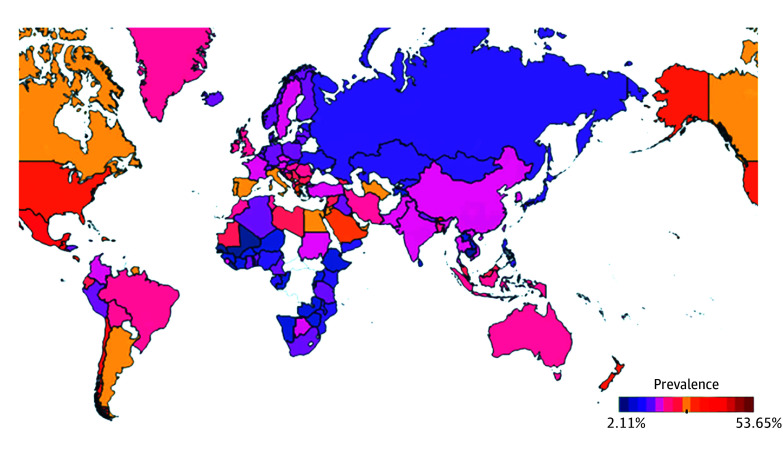
Global Prevalence of Excess Weight in Children and Adolescents

We further performed analyses on the prevalence of overweight and excess weight in children and adolescents. In total, 5 621 782 participants were diagnosed as having overweight with a pooled prevalence of 14.8% (95% CI, 14.5-15.1; *I*^2^, 99.8%), and 5 621 782 participants were diagnosed as having excess weight with a pooled prevalence of 22.2% (95% CI, 21.6-22.8; *I*^2^, 100.0%) (Figure 2). Details on subgroup analyses for overweight and excess weight are listed in eTables 13-14 in Supplement 1.

## Discussion

This systematic review and meta-analysis provided a comprehensive analysis of the global epidemiology of overweight and obesity from 2000 to 2023 in children and adolescents younger than 18 years. The overall prevalence of pediatric obesity, overweight, and excess weight was 8.5%, 14.8%, and 22.2%, respectively. According to our findings, there were notable regional variations, with Polynesia exhibiting the highest prevalence across all 3 categories and Middle and Western Africa displaying the lowest rates. Furthermore, a number of factors demonstrated a noteworthy association with the prevalence of pediatric obesity, including age, sex, school type, maternal obesity, having breakfast, number of meals per day, hours of playing on the computer per day, maternal smoking in pregnancy, birth weight, regular exercise, and sleep duration. Besides, children and adolescents with obesity are more likely to experience mental and physical comorbidities, such as depression and hypertension.

The Non-Communicable Diseases Risk Factor Collaboration^16^ provided data on global prevalence of obesity in children and adolescents aged 5 to 19 years from 1975 to 2016 and found the prevalence had grown for both boys and girls, from 0.9% to 7.8% and 0.7% to 5.6%, respectively. Their key finding was that, although the prevalence of obesity in high-income nations had plateaued around the year 2000, in other parts of Asia it was still rising. Our findings reconfirmed that obesity was more common in boys than girls. More importantly, we found a sharply increased prevalence of obesity from 2012 to 2023 to 2000 to 2011. Even though obesity is growing more widespread globally, there are still notable regional differences to be aware of. According to previous studies, Polynesia, the Caribbean, Northern America, and Central America have the highest rates of obesity (above 15%).^16,20^ Apart from the fact that many countries in these regions, such as the US, are well developed, which may contribute to the high prevalence of childhood obesity, it is noteworthy that most of these regions are adjacent to each other geographically, indicating that the genetic traits and unique diet habits of the habitants may also be potential drivers. Interestingly, the lowest prevalence (under 4%) appeared in Western European, Middle Africa, Melanesia, and Western Africa, covering highly developed countries as well as a large number of the least-developed countries. While the prevalence in Western Europe may be attributed to the quality of the health care system and health-conscious lifestyle choices, the similar prevalence in Middle Africa, Melanesia, and Western Africa were mainly due to their poverty. Furthermore, current findings revealed that pediatric obesity prevalence was closely linked to country development and national or regional income, which is in line with prior research.^20^ Notably, even among nations in similar economic strata, there are differences in the estimates of prevalence. For example, the prevalence of pediatric obesity in the US is 18.6%, while that in Japan, another high-income country, is 3.9%. Differences in dietary habits may play a role in this disparity. European countries and the US often embrace a diet preference of processed food, which are typically abundant in unhealthy fats, added sugars, and refined carbohydrates. In contrast, diets rich in whole grains and vegetables, which are generally regarded as healthier options, have historically been prioritized in Southeast Asian countries.

Prevalence of obesity in children and adolescents shows disparities across different ages. Our results revealed a lower prevalence of obesity in adolescents than that of preschool and school-age children, which is largely in accordance with prior studies.^20^ This decline in obesity prevalence could be mainly attributed to the hormone shifts as boys and girls approach puberty.^21^ Besides, teenagers tend to be more conscious about their appearance, thus making more effort toward weight control. Furthermore, heavier pressure from middle and high school could partly contribute to weight loss in adolescents.

Early life is a pivotal period for childhood obesity development.^22^ Prior analyses have linked preconception and prenatal environmental exposures to childhood obesity, including high maternal prepregnancy BMI,^23^ gestational weight gain,^24^ gestational diabetes,^25^ and maternal smoking,^26^ potentially through effects on the environment in uterus. The current study determined maternal obesity and smoking in pregnancy as risk factors for childhood and adolescent obesity, while maternal diabetes, gastrointestinal diabetes and gestational weight gain exhibited positive yet modest impact on it. Although prior studies considered paternal obesity to be a risk factor for childhood obesity, our findings revealed otherwise.^27,28^ Furthermore, our results revealed low birthweight was associated with lowest prevalence of obesity. However, Yuan et al^29^ claimed that children weighing less than 1500 g were most likely to be centrally obese. This mismatch may be due to the fact that we used BMI to quantify general obesity, whereas central obesity is measured by sex-specific waist to height ratio. Additionally, different infant feeding strategies, such as breastfeeding duration and formula addition, exhibit varying effects on childhood obesity in several meta-analyses.^30,31,32^ Nevertheless, our findings showed no discernible impact from these parameters.

The rise in prevalence of obesity has been profoundly influenced by environmental and behavioral factors,^33^ including dietary patterns,^34,35^ physical activity level,^36^ and use of technology.^37^ The current study revealed that skipping breakfast was associated with an increased risk of pediatric obesity, which was consistent with previous research.^38^ Surprisingly, having more than 3 meals per day was associated with a lower risk of being obese, which might be explained by the theory that having several small meals throughout a day is healthier than 3 large ones.^39,40^ As previously noted, children with obesity tend to participate in less physical activity than their peers without obesity,^36^ and decreasing levels of exercise as well as increasing sedentary behaviors contribute to obesity development. Our findings also showed that children with regular exercise had a much lower chance of obesity. Moreover, we observed that playing on the computer for more than 2 hours a day was associated with an increase in risk of excess weight, and time spent watching TV also showed a positive correlation, yet not significant. A connection between screen time and obesity in the pediatric population was initially demonstrated in studies of TV viewing,^41,42^ while mobile and gaming devices are gaining more and more attention.^43,44^ Screen exposure may raise the risk of obesity via increased exposure to food marketing, increased mindless eating while watching screens, displacement of time spent in physical activities, reinforcement of sedentary behaviors, and reduced sleep duration.

All body systems can be affected by obesity in the short or long term, depending upon age and obesity severity. Plenty of previous studies have discussed potential comorbidities of multiple system related to childhood obesity.^45,46,47,48,49,50,51^ According to a systematic analysis, children and adolescents with obesity have a 1.4 times higher likelihood of developing prediabetes, 1.7 times higher likelihood of developing asthma, 4.4 times higher likelihood of developing high blood pressure, and 26.1 times higher likelihood of developing fatty liver disease compared to those who are of a healthy weight.^52^ Likewise, our research disclosed high prevalence of comorbidities in children and adolescents with obesity. The highest pooled prevalence was found in depression, which approximately 1 in 3 children with obesity might experience, followed by hypertension, with a pooled prevalence of 28.0%. Compared to previously reported incidence in general population, which is approximately 25% for depression^53^ and 4% for hypertension,^54^ children and adolescents with obesity seemed to be more vulnerable to those health condition. The association between obesity and mentioned comorbidities had been shown to be bidirectional.^55,56^ In the management of childhood and adolescent obesity, it is pivotal that comorbidities are assessed and treated alongside to prevent progression of both.

### Limitations

There are some limitations in the present research. To our knowledge, this is the most comprehensive study to date, covering all geographic regions, but some countries and regions had limited data, making it challenging to accurately estimate. Besides, different criteria for recognizing overweight and obesity in children may influence the accuracy of the estimation. Moreover, limited studies concerning comorbidities were included in our analysis, since we focused on the epidemiology in the literature search process. Additionally, we simply divided the study period into 2 categories, namely 2000 to 2011 and 2012 to 2023, which resulted in less detailed information of the time trajectory of prevalence for childhood and adolescent obesity.

## Conclusions

In conclusion, the current study provided new epidemiological insights of overweight and obesity among children and adolescents worldwide. Our findings indicated high prevalence of overweight and obesity in children and adolescent with a pooled estimation of 8.5% and 14.8%, meaning approximately 1 of every 5 children or adolescents experience excess weight. Various risk factors, including inherent, dietary, and environmental factors, were significantly associated with the prevalence of pediatric obesity. It is noteworthy that children and adolescents with obesity were at high risk of mental and physical comorbidities. Global coordinated action and national control program are paramount to comprehend, prevent, and manage childr and adolescent obesity.

## Notes

Supplement 1. eTable 1. Searching strategy for prevalence of overweight and obesity in children and adolescents eTable 2. Quality assessment for including studies eTable 3. Characteristics of the studies for prevalence of obesity in children and adolescents eTable 4. Characteristics of the studies for prevalence of overweight in children and adolescents eTable 5. Characteristics of the studies for prevalence of excess weight in children and adolescents eTable 6. Sensitivity analysis and leave-one-out results performed in Metafor package eTable 7. Sensitivity analysis performed by using a built-in function eTable 8. Univariate meta-regression eTable 9. Multi-variable meta-regression eTable 10. Subgroup analysis for obesity in children and adolescents eTable 11. Analysis of risk factors for obesity in children and adolescents eTable 12. Analysis of comorbidities for obesity in children and adolescents eTable 13. Subgroup analysis for overweight in children and adolescents eTable 14. Subgroup analysis for excess weight in children and adolescents.

Supplement 2. Data sharing statement
