# Supplementary material for: Global Prevalence of Overweight and Obesity in Children and Adolescents: A Systematic Review and Meta-Analysis
Source: JAMA Pediatr. 2024 Jun 10;178(8):800–13. doi: 10.1001/jamapediatrics.2024.1576 (PMC11165417; doi:10.1001/jamapediatrics.2024.1576)
Supplement: Supplement 1. — eTable 1. Searching strategy for prevalence of overweight and obesity in children and adolescents eTable 2. Quality assessment for including studies eTable 3. Characteristics of the studies for prevalence of obesity in children and adolescents eTable 4. Characteristics of the studies for prevalence of overweight in children and adolescents eTable 5. Characteristics of the studies for prevalence of excess weight in children and adolescents eTable 6. Sensitivity analysis and leave-one-out results performed in Metafor package eTable 7. Sensitivity analysis performed by using a built-in function eTable 8. Univariate meta-regression eTable 9. Multi-variable meta-regression eTable 10. Subgroup analysis for obesity in children and adolescents eTable 11. Analysis of risk factors for obesity in children and adolescents eTable 12. Analysis of comorbidities for obesity in children and adolescents eTable 13. Subgroup analysis for overweight in children and adolescents eTable 14. Subgroup analysis for excess weight in children and adolescents. [file jamapediatr-e241576-s001.pdf]

Supplemental Online Content

Zhang X, Liu J, Ni Y, et al. Global prevalence of overweight and obesity in children and adolescents: a systematic review and meta-analysis. *JAMA Pediatr*. Published online June 10, 2024. doi:10.1001/jamapediatrics.2024.1576

eTable 1. Searching strategy for prevalence of overweight and obesity in children and adolescents

eTable 2. Quality assessment for including studies

eTable 3. Characteristics of the studies for prevalence of obesity in children and adolescents

eTable 4. Characteristics of the studies for prevalence of overweight in children and adolescents

eTable 5. Characteristics of the studies for prevalence of excess weight in children and adolescents

eTable 6. Sensitivity analysis and leave-one-out results performed in Metafor package

eTable 7. Sensitivity analysis performed by using a built-in function

eTable 8. Univariate meta-regression

eTable 9. Multi-variable meta-regression

eTable 10. Subgroup analysis for obesity in children and adolescents

eTable 11. Analysis of risk factors for obesity in children and adolescents

eTable 12. Analysis of comorbidities for obesity in children and adolescents

eTable 13. Subgroup analysis for overweight in children and adolescents

eTable 14. Subgroup analysis for excess weight in children and adolescents.

This supplemental material has been provided by the authors to give readers additional information about their work.

© 2024 Zhang X et al. *JAMA Pediatrics.*

eTable 1. Searching strategy for prevalence of overweight and obesity in children and adolescents.

Embase	22166
Medline Ovid	21405
Web of Science	20235
Cochrane	1642
**Overall**	**65448**

Embase

('childhood obesity'/de OR (((pediatric* OR paediatric* OR childhood* OR child* OR adolescent* OR adolescence* OR infant* OR infantile* OR youth*) NEAR/6 (obese* OR overweight* OR adiposity*))):ab,ti,kw) AND ('epidemiological data'/de OR 'epidemiology'/de OR 'geographic distribution'/de OR 'patient volume'/de OR prevalence/exp OR 'incidence'/de OR geography/de OR 'geographic names'/exp OR 'cross-sectional study'/de OR (epidemiolog* OR ((geograph* OR global*) NEAR/3 (distribut*)) OR (patient* NEAR/3 volume*) OR prevalen* OR incidenc* OR population-based* OR cross-sectional*):ab,ti,kw) NOT ((animal/exp OR animal*:de OR nonhuman/de) NOT ('human'/exp)) NOT ([Conference Abstract]/lim)

Medline Ovid

(Obesity, Pediatric/ OR (((pediatric* OR paediatric* OR childhood* OR child* OR adolescent* OR adolescence* OR infant* OR infantile* OR youth*) ADJ6 (obese* OR overweight* OR adiposity*))).ab,ti,kf.) AND (Epidemiological Monitoring/ OR Epidemiology/ OR Epidemiology.fs. OR exp Incidence/ OR exp Prevalence/ OR Incidence/ OR Geography/ OR exp Geographic Locations/ OR Epidemiologic Studies OR Cross-Sectional Studies/ OR (epidemiolog* OR ((geograph* OR global*) ADJ3 (distribut*)) OR (patient* ADJ3 volume*) OR prevalen* OR incidenc* OR population-based* OR cross-sectional*).ab,ti,kf.) NOT (exp Animals/ NOT Humans/) NOT (news OR congres* OR abstract* OR book* OR chapter* OR dissertation abstract*). pt.

Cochrane

((((pediatric* OR paediatric* OR childhood* OR child* OR adolescent* OR adolescence* OR infant* OR infantile* OR youth*) NEAR/6 (obesity* OR obese* OR overweight* OR adiposity*))):ab,ti,kw) AND ((epidemiolog* OR ((geograph* OR global*) NEAR/3 (distribut*)) OR (patient* NEAR/3 volume*) OR prevalen* OR incidenc* OR (population NEXT/1 based*) OR (cross NEXT/1 sectional*)):ab,ti,kw)

Web of Science

TS=(((((pediatric* OR paediatric* OR childhood* OR child* OR adolescent* OR adolescence* OR infant* OR infantile* OR youth*) NEAR/5 (obesity* OR obese* OR overweight* OR adiposity*)))) AND ((epidemiolog* OR ((geograph* OR global*) NEAR/2 (distribut*)) OR (patient* NEAR/2 volume*) OR prevalen* OR incidenc* OR population-based* OR cross-sectional*)) NOT ((animal* OR rat OR rats OR mouse OR mice OR murine OR dog OR dogs OR canine OR cat OR cats OR feline OR rabbit OR cow OR cows OR bovine OR rodent* OR sheep OR ovine OR pig OR swine OR porcine OR veterinar* OR chick* OR zebrafish* OR baboon* OR nonhuman* OR primate* OR cattle* OR goose OR geese OR duck OR macaque* OR avian* OR bird* OR fish*) NOT (human* OR patient* OR women OR woman OR men OR man))) AND DT=(Article OR Review)

© 2024 Zhang X et al. *JAMA Pediatrics.*

eTable 2. Quality assessment for including studies.

Study	Publication Year	Was the sample frame appropriate to address the target population?	Were study participants sampled in an appropriate way?	Was the sample size adequate?	Were the study subjects and the setting described in detail?	Was the data analysis conducted with sufficient coverage of the identified sample?	Were valid methods used for the identification of the condition?	Was the condition measured in a standard, reliable way for all participants?	Was there appropriate staistical analysis?	Was the response rate adequate, and if not, was the low response rate managed appropriately?	Quality Score
Salas, et al[1]	2021	yes	yes	yes	yes	no	yes	yes	yes	yes	8
Hyska, et al[2]	2014	yes	yes	yes	yes	yes	yes	yes	yes	yes	9
Benmohammed, et al[3]	2020	yes	yes	yes	yes	yes	yes	yes	yes	yes	9
Musaiger, et al[4]	2012	yes	yes	yes	yes	yes	yes	yes	yes	no	8
Fedala, et al[5]	2017	yes	yes	yes	yes	yes	yes	yes	yes	yes	9
Orden, et al[6]	2019	yes	yes	yes	yes	yes	yes	yes	yes	yes	9
Meyer, et al[7]	2013	yes	yes	yes	yes	yes	yes	no	yes	yes	8
Hirschler, et al[8]	2010	yes	yes	yes	yes	yes	yes	yes	no	yes	8
Stray-Pedersen,et al[9]	2009	yes	yes	yes	yes	yes	yes	yes	yes	yes	9
Hirschler, et al[10]	2008	yes	yes	yes	yes	yes	yes	no	yes	yes	8
Hirschler, et al[11]	2008	yes	yes	yes	yes	no	yes	yes	no	yes	7
Hirschler, et al[12]	2006	yes	yes	yes	yes	yes	yes	yes	yes	yes	9
Hirschler, et al[13]	2015	yes	yes	yes	yes	yes	no	yes	no	yes	7
Kovalskys, et al[14]	2011	yes	yes	yes	no	yes	yes	yes	yes	yes	8
Tringler, et al[15]	2012	yes	yes	yes	yes	no	yes	yes	no	yes	7
Catalani, et al[16]	2016	yes	yes	no	yes	yes	yes	yes	yes	yes	8
Gotthelf, et al[17]	2017	yes	yes	yes	no	yes	yes	no	yes	yes	7
Rivero, et al[18]	2018	yes	yes	yes	yes	yes	yes	yes	yes	yes	9
Abbott	2010	yes	no	yes	yes	yes	yes	yes	yes	yes	8

© 2024 Zhang X et al. *JAMA Pediatrics.*

, et al[19]											
Gibson, et al[20]	2007	yes	yes	yes	yes	yes	yes	yes	no	yes	8
Spinks, et al[21]	2007	yes	yes	no	yes	yes	yes	no	yes	yes	7
Crawford, et al[22]	2008	yes	yes	yes	yes	yes	no	yes	yes	yes	8
Franklin, et al[23]	2006	yes	yes	yes	yes	yes	yes	yes	yes	yes	9
Wolfenden, et al[24]	2011	yes	yes	yes	yes	yes	yes	yes	no	yes	8
Cauwenberghe, et al[25]	2012	yes	yes	yes	yes	yes	yes	yes	yes	yes	9
Wake, et al[26]	2013	yes	yes	yes	yes	no	yes	yes	yes	no	7
James, et al[27]	2013	yes	yes	yes	yes	yes	yes	yes	yes	yes	9
Marshall, et al[28]	2012	yes	yes	yes	yes	yes	yes	yes	yes	yes	9
Martin, et al[29]	2012	yes	yes	yes	yes	yes	yes	yes	no	yes	8
O'Dea, et al[30]	2010	yes	yes	yes	yes	no	yes	yes	yes	no	7
O'Dea, et al[31]	2014	yes	no	yes	yes	yes	yes	no	yes	yes	7
Schofield, et al[32]	2009	yes	yes	no	yes	yes	yes	yes	no	yes	7
Gopinath, et al[33]	2012	yes	yes	yes	yes	no	yes	yes	yes	yes	8
Spurrier, et al[34]	2012	yes	yes	yes	yes	yes	no	yes	yes	no	7
Trapp, et al[35]	2011	yes	yes	yes	yes	yes	yes	yes	yes	yes	9
Waters, et al[36]	2008	yes	yes	yes	yes	yes	yes	yes	yes	yes	9
Bell, et al[37]	2018	yes	yes	yes	no	yes	yes	yes	yes	yes	8
Wen, et al[38]	2014	yes	yes	yes	yes	yes	yes	yes	yes	yes	9
Hoare, et al[39]	2019	yes	yes	yes	yes	yes	yes	yes	yes	no	8
Maher, et al[40]	2012	yes	yes	yes	yes	no	yes	yes	yes	yes	8
Hayes, et al[41]	2021	yes	yes	yes	yes	yes	yes	yes	yes	yes	9
Miller, et al[42]	2014	yes	yes	yes	yes	yes	yes	yes	no	no	7
Achat, et al[43]	2014	yes	yes	no	yes	yes	yes	yes	yes	yes	8
O'Dea, et al[44]	2008	yes	yes	yes	yes	no	yes	yes	no	yes	7
O'Sullivan, et al[45]	2015	yes	no	yes	yes	yes	yes	yes	yes	yes	8
Barnes, et al[46]	2021	yes	yes	yes	yes	yes	yes	yes	no	yes	8

© 2024 Zhang X et al. *JAMA Pediatrics*.

Magee, et al[47]	2013	yes	yes	yes	no	yes	yes	no	yes	yes	7
Cox, et al[48]	2012	yes	yes	yes	yes	yes	yes	yes	yes	no	8
Wickramasinghe, et al[49]	2005	yes	yes	yes	yes	yes	yes	yes	yes	yes	9
Telford, et al[50]	2008	yes	no	yes	yes	yes	yes	yes	yes	yes	8
Seach, et al[51]	2010	yes	yes	yes	yes	yes	no	yes	yes	yes	8
Mallan,et al[52]	2017	yes	yes	yes	yes	yes	yes	yes	yes	yes	9
Jansen, et al[53]	2013	yes	yes	yes	yes	yes	yes	yes	no	yes	8
Tai, et al[54]	2009	yes	yes	yes	yes	yes	yes	yes	yes	yes	9
Hoare, et al[55]	2014	yes	yes	no	yes	yes	yes	yes	yes	yes	8
Bergmeier, et al[56]	2014	yes	yes	yes	yes	no	yes	yes	no	yes	7
Hardy, et al[57]	2012	yes	yes	yes	yes	yes	yes	yes	yes	yes	9
Waston, et al[58]	2023	yes	yes	yes	yes	yes	yes	yes	yes	yes	9
Au, et al[59]	2012	yes	yes	yes	yes	yes	yes	yes	yes	yes	9
Aurangzeb, et al[60]	2012	yes	yes	yes	yes	yes	no	yes	no	yes	7
Sanigorski, et al[61]	2005	yes	yes	yes	no	yes	yes	yes	yes	yes	8
Rehor, et al[62]	2002	yes	yes	yes	yes	yes	yes	no	yes	yes	8
Wen, et al[63]	2010	yes	yes	yes	yes	yes	yes	yes	yes	yes	9
Scott, et al[64]	2019	yes	yes	yes	yes	yes	yes	yes	yes	yes	9
Shi, et al[65]	2010	yes	yes	yes	yes	yes	yes	yes	no	no	7
Johns, et al[66]	2010	yes	no	yes	no	yes	no	yes	yes	yes	6
MacFarlane, et al[67]	2009	yes	yes	yes	yes	yes	yes	yes	yes	yes	9
Johns, et al[68]	2009	yes	yes	yes	yes	yes	yes	yes	no	yes	8
Haysom, et al[69]	2009	yes	yes	yes	yes	no	yes	yes	yes	yes	8
Ducher, et al[70]	2009	yes	yes	yes	yes	yes	yes	yes	yes	yes	9
Sutherland, et al[71]	2008	yes	yes	yes	yes	no	yes	yes	yes	yes	8
Denney-Wilson, et al[72]	2008	yes	yes	yes	yes	yes	yes	no	no	yes	7
Fisher, er al[73]	2006	yes	yes	yes	yes	yes	yes	yes	yes	yes	9
Cretikos, et	2008	yes	yes	yes	yes	no	yes	yes	no	yes	7

© 2024 Zhang X et al. *JAMA Pediatrics.*

al[74]											
Cleland, et al[75]	2008	yes	yes	yes	yes	yes	yes	yes	yes	yes	9
Crawford, et al[76]	2006	yes	yes	yes	yes	yes	no	yes	yes	yes	8
Campbell, et al[77]	2006	yes	yes	yes	yes	yes	yes	yes	yes	yes	9
Burke, et al[78]	2006	yes	yes	yes	yes	yes	yes	yes	yes	yes	9
Sanigorski, et al[79]	2007	yes	yes	yes	yes	no	yes	yes	yes	no	7
McLean, et al[80]	2007	yes	yes	yes	yes	yes	yes	yes	yes	yes	9
Leech, et al[81]	2015	yes	yes	yes	yes	yes	yes	yes	yes	yes	9
Cochrane, et al[82]	2015	yes	yes	yes	yes	no	yes	no	yes	yes	7
O'Dea, et al[83]	2014	yes	yes	yes	yes	yes	yes	yes	no	yes	8
Chen, et al[84]	2014	yes	yes	no	yes	yes	yes	yes	yes	yes	8
Keating, et al[85]	2011	yes	yes	yes	yes	no	yes	yes	yes	yes	8
Olds, et al[86]	2011	yes	no	yes	yes	yes	yes	yes	yes	yes	8
Morley, et al[87]	2012	yes	yes	yes	yes	no	yes	yes	no	yes	7
Schultz, et al[88]	2012	yes	yes	yes	yes	yes	yes	yes	yes	yes	9
Fredrickson, et al[89]	2013	yes	no	yes	yes	yes	yes	yes	yes	yes	8
Ruedl, et al[90]	2016	yes	yes	yes	yes	yes	yes	yes	yes	yes	9
Furthner, et al[91]	2017	yes	yes	yes	yes	yes	no	yes	yes	yes	8
Mihrshahi, et al[92]	2017	yes	yes	yes	yes	yes	yes	yes	yes	yes	9
Furthner, et al[93]	2018	yes	yes	yes	yes	yes	no	yes	no	yes	7
Yngve, et al[94]	2008	yes	yes	yes	yes	yes	yes	yes	yes	yes	9
Janssen, et al[95]	2005	yes	yes	yes	no	yes	no	yes	yes	yes	7
Pfeiffer, et al[96]	2006	yes	yes	yes	yes	yes	yes	yes	yes	yes	9
Romano, et al[97]	2022	yes	yes	yes	yes	yes	yes	yes	no	yes	8
Musaiger, et al[98]	2014	yes	yes	yes	yes	yes	yes	no	yes	no	7
Al-Raees, et al[99]	2009	yes	yes	yes	yes	yes	yes	no	yes	yes	8
Al-Sendi,	2003	yes	yes	yes	yes	yes	no	yes	yes	yes	8

© 2024 Zhang X et al. *JAMA Pediatrics*.

et al[100]											
Musaiger, et al[101]	2014	yes	yes	yes	yes	yes	yes	no	yes	yes	8
Anam, et al[102]	2022	yes	yes	yes	no	no	yes	yes	yes	yes	7
Taher, et al[103]	2021	yes	yes	yes	yes	yes	yes	yes	yes	no	8
Sultana, et al[104]	2015	yes	yes	yes	yes	yes	yes	yes	yes	yes	9
Sultana, et al[105]	2016	yes	yes	yes	yes	yes	yes	no	yes	yes	8
Saha, et al[106]	2018	yes	yes	yes	yes	yes	yes	yes	yes	no	8
Islam, et al[107]	2019	yes	yes	yes	yes	yes	yes	no	yes	yes	8
Sultana, et al[108]	2019	yes	yes	yes	yes	yes	yes	no	yes	yes	8
Khan, et al[109]	2020	yes	yes	yes	yes	yes	yes	no	yes	yes	8
Bulbul, et al[110]	2014	yes	yes	no	no	yes	yes	yes	yes	yes	7
Brug, et al[111]	2012	yes	yes	yes	yes	yes	no	yes	yes	yes	8
Usheva, et al[112]	2021	yes	yes	yes	yes	yes	yes	yes	yes	no	8
Júlíusson, et al[113]	2015	yes	yes	yes	yes	yes	yes	yes	no	yes	8
Seghers, et al[114]	2010	yes	yes	yes	yes	yes	no	yes	no	yes	7
Vriendt, et al[115]	2009	yes	yes	yes	no	yes	yes	yes	yes	yes	8
De Coen, et al[116]	2014	yes	yes	yes	yes	yes	no	yes	yes	yes	8
Gysel, et al[117]	2009	yes	yes	no	yes	yes	yes	yes	yes	yes	8
Deforche, et al[118]	2009	yes	yes	yes	yes	yes	yes	yes	no	yes	8
Vissers, et al[119]	2008	yes	yes	yes	yes	yes	yes	yes	yes	yes	9
Huybrechts, et al[120]	2006	yes	yes	no	yes	yes	yes	yes	no	yes	7
Velde, et al[121]	2017	yes	yes	yes	yes	no	yes	yes	yes	yes	8
Manyanga, et al[122]	2014	yes	yes	yes	yes	yes	yes	yes	no	no	7
Norbu, et al[123]	2019	yes	yes	no	yes	yes	yes	no	yes	yes	7
Botti, et al[124]	2010	yes	yes	yes	yes	yes	yes	yes	yes	yes	9
Pe´rez-Cueto, et al[125]	2005	yes	yes	yes	yes	no	yes	yes	yes	yes	8
Benefice, et al[126]	2007	yes	yes	yes	yes	yes	yes	yes	yes	yes	9
Spahić, et al[127]	2019	yes	yes	no	yes	yes	yes	yes	no	yes	7

© 2024 Zhang X et al. *JAMA Pediatrics*.

Hansanbegović, et al[128]	2010	yes	yes	yes	no	yes	no	yes	yes	yes	7
Ramic, et al[129]	2009	yes	yes	yes	yes	yes	yes	yes	no	no	7
Wrotniak, et al[130]	2012	yes	yes	yes	yes	no	yes	yes	no	yes	7
Alexius, et al[131]	2012	yes	yes	yes	yes	yes	yes	no	yes	yes	8
Andaki, et al[132]	2017	yes	yes	yes	yes	yes	no	no	yes	yes	7
Ataíde Lima, et al[133]	2015	yes	yes	yes	yes	yes	yes	yes	no	yes	8
Castilho, et al[134]	2014	yes	yes	yes	yes	yes	yes	yes	yes	yes	9
Costa, et al[135]	2015	yes	yes	no	yes	yes	no	yes	yes	no	6
Ferreira, et al[136]	2015	yes	yes	yes	yes	yes	yes	yes	yes	yes	9
Fraiz, et al[137]	2019	yes	yes	yes	yes	yes	yes	yes	yes	yes	9
Justo, et al[138]	2012	yes	yes	yes	yes	yes	no	yes	yes	yes	8
Guedes, et al[139]	2011	yes	yes	no	yes	yes	yes	yes	yes	yes	8
Kupek, et al[140]	2016	yes	yes	yes	yes	yes	yes	yes	yes	no	8
Moreira, et al[141]	2012	yes	yes	yes	yes	yes	no	yes	yes	yes	8
Nobre, et al[142]	2013	yes	yes	yes	yes	yes	yes	yes	yes	yes	9
Oppitz, et al[143]	2014	yes	yes	yes	yes	yes	yes	yes	yes	yes	9
Pretto, et al[144]	2004	yes	yes	yes	no	yes	yes	yes	yes	no	7
Rmalho, et al[145]	2013	yes	yes	yes	yes	yes	yes	yes	yes	yes	9
Santos, et al[146]	2019	yes	yes	yes	yes	yes	no	yes	yes	yes	8
Sliva, et al[147]	2018	yes	yes	yes	no	yes	no	yes	yes	no	6
Mastroeni, et al[148]	2017	yes	yes	yes	yes	yes	yes	yes	yes	yes	9
Vale, et al[149]	2022	yes	yes	yes	yes	yes	yes	no	yes	yes	8
Pereira, et al[150]	2023	yes	no	yes	yes	yes	yes	yes	yes	no	7
Lopes, et al[151]	2022	yes	yes	yes	yes	yes	yes	yes	yes	yes	9
Santos, et al[152]	2022	yes	yes	no	yes	yes	yes	yes	yes	yes	8
Coelho, et al[153]	2022	yes	yes	yes	yes	no	yes	yes	yes	no	7
Cavalcante,	2022	yes	yes	yes	yes	yes	yes	yes	yes	yes	9

© 2024 Zhang X et al. *JAMA Pediatrics*.

et al[154]											
Blumenberg, et al[155]	2021	yes	no	yes	yes	yes	yes	yes	no	yes	7
Barbiero, et al[156]	2009	yes	no	yes	yes	yes	no	yes	yes	yes	7
Barbosa, et al[157]	2021	yes	yes	yes	yes	yes	yes	yes	yes	no	8
Gemelli, et al[158]	2016	yes	yes	yes	yes	yes	yes	yes	no	yes	8
Cândido, et al[159]	2009	yes	yes	yes	no	no	yes	yes	yes	yes	7
Polderman, et al[160]	2011	yes	yes	yes	yes	yes	yes	yes	no	yes	8
Novaes, et al[161]	2013	yes	yes	no	yes	yes	yes	yes	yes	yes	8
Duncan, et al[162]	2011	yes	yes	yes	yes	no	yes	no	yes	no	6
Halal, et al[163]	2016	yes	yes	yes	yes	yes	yes	yes	no	yes	8
Dalamaria, et al[164]	2021	yes	yes	yes	yes	yes	yes	yes	no	yes	8
Lima, et al[165]	2020	yes	yes	yes	no	yes	yes	yes	yes	yes	8
Ribeiro, et al[166]	2017	yes	yes	yes	yes	yes	yes	yes	yes	yes	9
Nogueira, et al[167]	2020	yes	yes	yes	yes	yes	yes	yes	yes	yes	9
Todendi, et al[168]	2020	yes	yes	yes	yes	yes	yes	yes	yes	yes	9
Goldani, et al[169]	2013	yes	yes	yes	yes	yes	yes	yes	no	yes	8
Caixeta, et al[170]	2020	yes	yes	yes	yes	yes	no	yes	yes	no	7
Kuschnir, et al[171]	2009	yes	yes	yes	yes	yes	yes	yes	yes	yes	9
Ferreira, et al[172]	2008	yes	yes	yes	yes	yes	yes	yes	yes	yes	9
Rodrigues, et al[173]	2006	yes	yes	no	yes	yes	yes	yes	no	yes	7
Amorim, et al[174]	2006	yes	yes	yes	yes	yes	no	no	yes	yes	7
Araújo, et al[175]	2010	yes	yes	yes	yes	yes	yes	yes	yes	no	8
Flores, et al[176]	2013	yes	yes	yes	yes	no	yes	yes	yes	yes	8
Bispo, et al[177]	2015	yes	yes	yes	no	yes	yes	yes	yes	yes	8
Castro, et al[178]	2012	yes	yes	yes	yes	yes	no	yes	yes	no	7
Moraes, et al[179]	2014	yes	yes	yes	yes	yes	yes	yes	yes	no	8

© 2024 Zhang X et al. *JAMA Pediatrics.*

Santana, et al[180]	2013	yes	yes	yes	yes	no	yes	no	yes	yes	7
Lock, et al[181]	2020	yes	yes	yes	yes	no	yes	yes	yes	yes	8
Cruz, et al[182]	2013	yes	yes	yes	yes	yes	yes	yes	yes	yes	9
Moraes, et al[183]	2019	yes	yes	yes	yes	yes	yes	no	yes	yes	8
Christofaro, et al[184]	2016	yes	yes	yes	yes	yes	yes	yes	yes	yes	9
Coutinho, et al[185]	2014	yes	yes	yes	yes	yes	yes	yes	yes	no	8
Pinho, et al[186]	2014	yes	yes	yes	no	yes	yes	yes	yes	yes	8
Guedes, et al[187]	2013	yes	no	yes	yes	no	yes	yes	yes	no	6
Rosini, et al[188]	2015	yes	yes	yes	yes	yes	no	yes	no	yes	7
Neves, et al[189]	2015	yes	yes	yes	yes	yes	yes	yes	yes	yes	9
Santana, et al[190]	2017	yes	yes	yes	yes	yes	no	no	yes	yes	7
Costa, et al[191]	2020	yes	yes	yes	yes	yes	yes	yes	yes	yes	9
Vieira, et al[192]	2015	yes	yes	yes	yes	yes	yes	yes	no	yes	8
Araujo, et al[193]	2018	yes	yes	no	yes	yes	yes	yes	yes	yes	8
Carmo, et al[194]	2018	yes	yes	yes	yes	yes	yes	yes	no	yes	8
Fradkin, et al[195]	2018	yes	yes	yes	no	yes	no	yes	yes	yes	7
Reuter, et al[196]	2018	yes	yes	yes	yes	yes	yes	yes	yes	no	8
Ulbricht, et al[197]	2018	yes	yes	yes	yes	yes	yes	yes	yes	yes	9
Ripka, et al[198]	2017	yes	yes	yes	yes	no	yes	yes	yes	yes	8
Tebar, et al[199]	2018	yes	yes	yes	yes	yes	yes	yes	yes	yes	9
Farias, et al[200]	2019	yes	yes	yes	yes	yes	yes	yes	yes	no	8
Silva, et al[201]	2020	yes	yes	yes	yes	yes	yes	yes	yes	yes	9
Schwertner, et al[202]	2020	yes	yes	yes	yes	yes	yes	yes	yes	yes	9
Dantas, et al[203]	2018	yes	no	yes	yes	yes	yes	yes	yes	yes	8
Christofaro, et al[204]	2016	yes	yes	yes	yes	yes	no	yes	yes	yes	8
Werneck, et al[205]	2018	no	yes	yes	yes	yes	yes	no	yes	yes	7
Assis, et al[206]	2005	yes	yes	yes	no	yes	yes	yes	yes	yes	8
Salas, et al[207]	2018	yes	yes	yes	yes	yes	yes	yes	no	yes	8
Assis,	2019	yes	yes	yes	yes	yes	yes	yes	yes	yes	9

© 2024 Zhang X et al. *JAMA Pediatrics.*

et al[208]											
Dalmaso, et al[209]	2019	no	yes	no	yes	yes	yes	yes	yes	yes	7
Lima, et al[210]	2019	yes	yes	yes	yes	yes	yes	no	yes	yes	8
Porcelli, et al[211]	2019	yes	yes	yes	yes	yes	yes	yes	yes	yes	9
Alves, et al[212]	2020	yes	yes	yes	yes	no	yes	yes	no	yes	7
Folmann, et al[213]	2020	yes	yes	yes	yes	yes	yes	yes	no	yes	8
Hércules, et al[214]	2020	yes	no	yes	yes	yes	no	yes	yes	yes	7
Lucena, et al[215]	2020	yes	yes	yes	yes	yes	yes	yes	no	yes	8
Rocha, et al[216]	2020	yes	yes	yes	yes	yes	yes	yes	yes	yes	9
Rivera, et al[217]	2010	yes	yes	yes	yes	no	yes	yes	yes	yes	8
Gabriel, et al[218]	2010	yes	yes	yes	yes	yes	yes	yes	yes	yes	9
Anwar, et al[219]	2010	yes	yes	yes	yes	no	yes	yes	no	yes	7
Molina, et al[220]	2009	yes	yes	no	yes	yes	yes	yes	yes	yes	8
Fernandes, et al[221]	2009	yes	yes	yes	yes	yes	yes	no	no	yes	7
Alves, et al[222]	2009	yes	yes	yes	no	yes	yes	yes	yes	yes	8
Pelegrini, et al[223]	2008	yes	yes	yes	yes	yes	yes	yes	yes	yes	9
Bertolace, et al[224]	2008	yes	yes	yes	yes	yes	yes	no	yes	yes	8
Oliveira, et al[225]	2007	yes	yes	yes	yes	yes	yes	yes	no	yes	8
da Silva, et al[226]	2007	yes	yes	yes	yes	yes	yes	no	yes	yes	8
Granville-Garcia, et al[227]	2006	yes	yes	yes	yes	yes	yes	yes	yes	yes	9
Lima, et al[228]	2015	yes	yes	yes	yes	yes	no	yes	yes	no	7
Weinmayr, et al[229]	2014	yes	yes	yes	yes	yes	yes	yes	yes	yes	9
Müller, et al[230]	2014	yes	no	yes	yes	yes	no	yes	yes	yes	7
Moreira, et al[231]	2014	yes	yes	yes	yes	no	yes	no	yes	yes	7
Mendonça, et al[232]	2014	yes	yes	yes	yes	yes	yes	yes	no	yes	8
Crispim, et al[233]	2014	yes	yes	yes	yes	yes	yes	yes	yes	yes	9

© 2024 Zhang X et al. *JAMA Pediatrics*.

Menezes, et al[234]	2011	yes	yes	yes	yes	yes	yes	yes	yes	yes	9
Cremm, et al[235]	2012	yes	yes	no	yes	yes	no	yes	yes	no	6
Nascimento, et al[236]	2012	yes	yes	yes	yes	yes	yes	yes	yes	no	8
Novaes, et al[237]	2012	yes	yes	yes	yes	yes	yes	yes	yes	yes	9
Rosaneli, et al[238]	2012	yes	yes	yes	yes	yes	yes	yes	yes	yes	9
Marques, et al[239]	2013	yes	yes	yes	yes	yes	yes	yes	yes	no	8
Reuter, et al[240]	2013	yes	no	yes	yes	yes	yes	yes	yes	yes	8
Andrade, et al[241]	2016	yes	yes	yes	yes	no	yes	no	yes	yes	7
Casonatto, et al[242]	2016	yes	yes	yes	yes	yes	yes	yes	yes	yes	9
Farias Júnior, et al[243]	2016	yes	yes	yes	yes	yes	yes	yes	yes	yes	9
Jardim-Botelho, et al[244]	2016	yes	yes	yes	yes	yes	yes	yes	yes	yes	9
Silva, et al[245]	2016	no	yes	yes	yes	yes	no	yes	yes	yes	7
Araujo, et al[246]	2017	yes	yes	yes	no	no	yes	yes	yes	yes	7
Cuesta, et al[247]	2018	yes	yes	yes	yes	yes	yes	yes	yes	no	8
Dos Santos, et al[248]	2018	yes	yes	no	yes	no	yes	yes	yes	yes	7
Pivatto, et al[249]	2018	yes	yes	yes	yes	yes	yes	no	yes	yes	8
Reuter, et al[250]	2018	yes	yes	yes	yes	yes	yes	yes	yes	yes	9
Mladenova, et al[251]	2015	yes	yes	yes	no	yes	yes	yes	yes	yes	8
Gorog, et al[252]	2011	yes	yes	no	yes	no	yes	yes	yes	yes	7
Wijnhoven, et al[253]	2015	yes	yes	yes	yes	yes	yes	yes	yes	yes	9
Mank, et al[254]	2022	yes	yes	yes	yes	yes	yes	yes	yes	no	8
Daboné, et al[255]	2011	yes	yes	yes	yes	yes	yes	no	yes	yes	8
Gebremedhin, et al[256]	2015	yes	yes	yes	yes	yes	yes	yes	yes	yes	9
Dimaisip-Nabuab, et	2018	yes	yes	yes	yes	yes	yes	yes	no	yes	8

© 2024 Zhang X et al. *JAMA Pediatrics.*

al[257]											
Navti, et al[258]	2021	yes	no	yes	yes	yes	no	yes	yes	yes	7
Navti, et al[259]	2014	yes	yes	yes	yes	yes	no	yes	yes	yes	8
Choukem, et al[260]	2017	yes	yes	yes	yes	yes	no	yes	yes	yes	8
Chelo, et al[261]	2019	yes	yes	yes	no	no	yes	yes	yes	yes	7
Dapi, et al[262]	2009	yes	yes	yes	yes	no	yes	yes	yes	yes	8
Navti, et al[263]	2015	yes	yes	yes	no	yes	yes	no	yes	yes	7
Wamba, et al[264]	2013	yes	yes	yes	yes	yes	yes	yes	yes	yes	9
Navti , et al[265]	2017	yes	yes	yes	yes	yes	yes	yes	yes	no	8
Hulst, et al[266]	2022	yes	yes	yes	no	yes	yes	no	yes	yes	7
Anderson, et al[267]	2022	yes	yes	yes	yes	yes	yes	yes	yes	yes	9
Héroux, et al[268]	2012	yes	yes	yes	yes	yes	no	yes	yes	yes	8
Le, et al[269]	2016	yes	yes	yes	yes	no	yes	yes	yes	yes	8
Seliske, et al[270]	2009	yes	yes	yes	yes	no	yes	no	yes	yes	7
Ball, et al[271]	2019	yes	yes	yes	yes	yes	yes	yes	yes	yes	9
Dubois, et al[272]	2009	yes	yes	yes	yes	yes	yes	no	yes	no	7
Chaput, et al[273]	2006	yes	yes	no	yes	yes	yes	yes	yes	yes	8
Chaput, et al[274]	2011	yes	yes	yes	yes	yes	yes	yes	yes	no	8
Davidson, et al[275]	2016	yes	yes	yes	yes	yes	yes	yes	yes	yes	9
Brault, et al[276]	2015	yes	yes	yes	no	no	yes	yes	no	yes	6
Larsen, et al[277]	2015	yes	yes	yes	yes	yes	yes	yes	yes	yes	9
Azad, et al[278]	2014	yes	yes	yes	yes	yes	yes	yes	yes	yes	9
Bridgman, et al[279]	2018	yes	yes	yes	yes	yes	yes	yes	yes	no	8
Cassidy-Bushrow, et al[280]	2018	yes	yes	yes	no	yes	yes	yes	yes	yes	8
Sheilds, et al[281]	2010	yes	yes	yes	yes	yes	no	yes	yes	yes	8
Medehouenou, et al[282]	2015	yes	yes	yes	yes	yes	yes	yes	yes	yes	9
Wang, et al[283]	2008	yes	yes	yes	yes	yes	no	yes	yes	no	7
Mai, et al[284]	2007	yes	no	yes	yes	no	yes	no	yes	yes	6

© 2024 Zhang X et al. *JAMA Pediatrics*.

Shi, et al[285]	2013	yes	yes	yes	yes	yes	yes	yes	yes	yes	9
Walton, et al[286]	2014	yes	yes	yes	yes	yes	yes	yes	yes	yes	9
Oliver, et al[287]	2005	no	yes	yes	yes	yes	yes	yes	yes	yes	8
MOFFAT, et al[288]	2005	yes	yes	yes	yes	yes	yes	yes	yes	yes	9
Ball, et al[289]	2005	yes	yes	yes	yes	yes	yes	yes	no	no	7
Woodruff, et al[290]	2010	yes	yes	no	yes	yes	yes	yes	yes	yes	8
Twells, et al[291]	2010	yes	yes	yes	yes	yes	yes	yes	yes	no	8
Menon, et al[292]	2019	yes	yes	yes	yes	yes	yes	yes	yes	yes	9
Shay, et al[293]	2020	yes	no	yes	yes	yes	yes	no	yes	yes	7
Simen-Kapeu, et al[294]	2010	yes	yes	yes	yes	yes	yes	yes	yes	yes	9
Leatherdale, et al[295]	2010	yes	yes	yes	yes	yes	no	yes	yes	yes	8
Khaili, et al[296]	2010	yes	yes	no	yes	yes	yes	yes	yes	no	7
Ismailov, et al[297]	2010	yes	yes	yes	yes	yes	yes	yes	yes	yes	9
Galloway, et al[298]	2010	yes	yes	no	yes	yes	yes	yes	yes	no	7
Doan, et al[299]	2010	yes	yes	yes	yes	yes	yes	yes	yes	yes	9
Cairney, et al[300]	2010	yes	yes	yes	yes	yes	yes	yes	yes	yes	9
Bengoecher, et al[301]	2010	yes	yes	yes	yes	yes	yes	yes	yes	yes	9
Wahi, et al[302]	2009	yes	yes	yes	yes	no	yes	yes	yes	yes	8
Vance, et al[303]	2009	yes	yes	yes	yes	no	yes	yes	yes	no	7
Potestio, et al[304]	2009	no	no	yes	yes	yes	yes	yes	yes	yes	7
McGavock, et al[305]	2009	yes	yes	yes	yes	yes	yes	yes	yes	yes	9
Downs, et al[306]	2009	yes	yes	yes	yes	yes	no	yes	yes	yes	8
Spence, et al[307]	2008	yes	no	yes	no	yes	yes	yes	yes	yes	7
Salvadori, et al[308]	2008	yes	yes	yes	yes	yes	yes	yes	yes	yes	9
Edwards, et al[309]	2008	no	yes	yes	yes	yes	yes	yes	yes	yes	8
Bruner, et al[310]	2008	yes	yes	yes	yes	yes	yes	yes	yes	yes	9

© 2024 Zhang X et al. *JAMA Pediatrics.*

Willows, et al[311]	2007	yes	yes	yes	no	yes	yes	no	yes	yes	7
He, et al[312]	2007	yes	yes	yes	yes	yes	yes	yes	yes	yes	9
Bassett, et al[313]	2007	yes	yes	yes	yes	yes	no	yes	yes	yes	8
Ng, et al[314]	2006	no	yes	yes	yes	yes	yes	yes	yes	yes	8
Janssen, et al[315]	2006	yes	yes	yes	yes	yes	yes	no	yes	no	7
Galloway, et al[316]	2006	yes	yes	yes	yes	yes	yes	no	yes	yes	8
Rossiter, et al[317]	2015	yes	yes	yes	yes	yes	yes	yes	yes	yes	9
Borghese, et al[318]	2015	yes	yes	yes	yes	yes	yes	yes	yes	yes	9
Banerjee, et al[319]	2015	yes	yes	yes	no	yes	yes	yes	yes	yes	8
Carson, et al[320]	2014	yes	yes	yes	yes	yes	yes	yes	yes	yes	9
Leatherdale, et al[321]	2013	yes	yes	yes	yes	yes	yes	yes	yes	yes	9
Nyström, et al[322]	2018	yes	yes	yes	yes	yes	no	yes	yes	no	7
Peña-Jorquera, et al[323]	2021	yes	yes	yes	yes	no	yes	yes	yes	yes	8
Agüero, et al[324]	2016	yes	yes	yes	yes	yes	yes	yes	yes	yes	9
Olivares, et al[325]	2004	yes	yes	yes	yes	yes	yes	yes	yes	yes	9
Cediel, et al[326]	2016	yes	yes	yes	no	yes	yes	yes	yes	yes	8
Mardones, et al[327]	2008	yes	yes	yes	yes	yes	yes	no	yes	yes	8
Delgado-Floody, et al[328]	2019	yes	yes	yes	yes	yes	yes	yes	yes	yes	9
Corvalán, et al[329]	2010	yes	yes	yes	yes	yes	yes	yes	yes	no	8
Valenzuela, et al[330]	2015	yes	yes	yes	yes	yes	yes	no	yes	yes	8
Lizana, et al[331]	2015	yes	yes	yes	yes	yes	yes	yes	yes	no	8
Cadenas-Sánchez, et al[332]	2015	no	yes	yes	yes	yes	yes	yes	yes	yes	8
Heitzinger, et al[333]	2014	yes	yes	yes	yes	yes	no	yes	yes	yes	8
Silva, et al[334]	2013	yes	yes	yes	yes	yes	yes	yes	yes	yes	9

© 2024 Zhang X et al. *JAMA Pediatrics.*

Kagawa, et al[335]	2016	yes	yes	no	yes	yes	yes	yes	yes	yes	8
García-Hermoso, et al[336]	2017	yes	yes	yes	yes	yes	yes	yes	no	yes	8
Liu, et al[337]	2016	yes	yes	yes	yes	yes	no	yes	yes	no	7
Li, et al[338]	2015	yes	yes	yes	yes	no	yes	no	yes	yes	7
Tang, et al[339]	2022	yes	yes	yes	yes	yes	yes	yes	yes	no	8
Su, et al[340]	2022	yes	yes	yes	yes	yes	yes	yes	yes	yes	9
Shi, et al[341]	2022	yes	yes	yes	no	yes	yes	yes	no	yes	7
Shi, et al[342]	2022	yes	yes	yes	yes	yes	yes	yes	yes	yes	9
Ma, et al[343]	2022	yes	yes	yes	yes	yes	yes	yes	no	yes	8
Liu, et al[344]	2022	yes	yes	no	yes	yes	yes	no	yes	no	6
Liu, et al[345]	2022	yes	yes	yes	yes	yes	no	yes	yes	yes	8
Li, et al[346]	2022	yes	yes	yes	yes	yes	yes	yes	yes	yes	9
Li, et al[347]	2022	yes	yes	yes	yes	yes	yes	yes	yes	yes	9
Huang, et al[348]	2022	yes	yes	yes	yes	yes	yes	no	yes	no	7
He, et al[349]	2022	yes	yes	yes	yes	yes	yes	yes	yes	yes	9
Guo, et al[350]	2022	yes	yes	yes	yes	yes	yes	yes	yes	no	8
Gong, et al[351]	2022	yes	yes	yes	yes	yes	yes	yes	yes	yes	9
Chen, et al[352]	2022	yes	yes	yes	yes	yes	no	yes	yes	yes	8
Chen, et al[353]	2022	yes	yes	yes	yes	yes	yes	no	yes	yes	8
Chen, et al[354]	2022	yes	no	yes	yes	yes	yes	yes	yes	yes	8
Chang, et al[355]	2022	yes	yes	yes	yes	yes	yes	yes	yes	yes	9
Zhu, et al[356]	2021	yes	yes	yes	yes	yes	yes	no	yes	yes	8
Zhou, et al[357]	2021	yes	yes	no	yes	yes	yes	yes	yes	yes	8
Zheng, et al[358]	2021	yes	yes	yes	yes	yes	yes	yes	yes	yes	9
Zhao, et al[359]	2021	yes	yes	yes	yes	yes	no	yes	yes	yes	8
Zhang, et al[360]	2021	yes	yes	yes	yes	yes	yes	yes	yes	yes	9
Zhang, et al[361]	2021	yes	yes	yes	yes	yes	no	yes	yes	yes	8
Zhang, et al[362]	2021	yes	yes	yes	yes	yes	yes	yes	yes	yes	9
Yuan, et al[363]	2021	yes	yes	yes	no	yes	yes	yes	yes	yes	8
You, et al[364]	2021	yes	yes	yes	yes	yes	yes	no	yes	yes	8
Xu, et al[365]	2021	yes	yes	no	yes	yes	no	yes	yes	yes	7

© 2024 Zhang X et al. *JAMA Pediatrics.*

Wang, et al[366]	2021	yes	yes	yes	yes	yes	yes	yes	yes	yes	9
Wang, et al[367]	2021	yes	yes	yes	yes	yes	yes	yes	yes	yes	9
Sun, et al[368]	2021	yes	yes	yes	yes	yes	yes	no	yes	yes	8
Min, et al[369]	2021	yes	yes	yes	yes	yes	yes	yes	yes	yes	9
Liu, et al[370]	2021	no	yes	yes	yes	yes	yes	yes	yes	yes	8
Zhou, et al[371]	2020	yes	yes	yes	yes	yes	yes	yes	no	yes	8
Zhou, et al[372]	2020	yes	yes	yes	yes	yes	yes	yes	yes	yes	9
Zheng, et al[373]	2020	no	yes	no	yes	yes	yes	yes	yes	yes	7
Xu, et al[374]	2020	yes	yes	yes	yes	yes	yes	yes	yes	yes	9
Tang, et al[375]	2010	yes	yes	yes	yes	no	yes	no	yes	yes	7
Shan, et al[376]	2010	yes	yes	yes	yes	yes	yes	yes	yes	yes	9
Zhang, et al[377]	2014	yes	yes	yes	yes	yes	yes	yes	yes	yes	9
Abdumijit, et al[378]	2022	yes	yes	yes	yes	yes	yes	yes	no	yes	8
Cheng, et al[379]	2019	yes	yes	yes	yes	yes	yes	yes	yes	yes	9
Guo, et al[380]	2020	yes	yes	no	yes	no	yes	yes	yes	yes	7
Li, et al[381]	2014	yes	yes	yes	yes	yes	yes	yes	no	yes	8
Zheng, et al[382]	2021	yes	yes	yes	yes	yes	yes	yes	yes	yes	9
Zhang, et al[383]	2021	yes	yes	yes	yes	yes	yes	yes	no	yes	8
Liu, et al[384]	2019	yes	yes	yes	yes	yes	yes	yes	yes	yes	9
Jiang, et al[385]	2007	yes	yes	yes	yes	no	yes	yes	yes	yes	8
Li, et al[386]	2010	no	yes	yes	yes	yes	yes	yes	yes	yes	8
Li, et al[387]	2014	yes	yes	yes	yes	yes	yes	yes	yes	yes	9
Xu, et al[388]	2015	yes	yes	yes	yes	yes	no	yes	yes	yes	8
Meng, et al[389]	2013	yes	yes	yes	yes	yes	yes	yes	yes	yes	9
Ji, et al[390]	2018	yes	no	yes	yes	yes	yes	yes	no	yes	7
Cui, et al[391]	2010	yes	yes	yes	yes	yes	yes	yes	yes	yes	9
Li, et al[392]	2008	yes	yes	yes	yes	yes	yes	no	yes	yes	8
Zhang, et al[393]	2012	no	yes	yes	yes	yes	yes	yes	yes	yes	8
Ma, et al[394]	2014	yes	yes	yes	yes	yes	yes	yes	yes	yes	9
Zhang, et al[395]	2013	yes	yes	yes	yes	yes	yes	yes	yes	yes	9
Cheng, et al[396]	2020	yes	yes	yes	no	yes	yes	yes	yes	no	7
Zhang, et	2020	no	yes	yes	yes	yes	no	yes	yes	yes	7

© 2024 Zhang X et al. *JAMA Pediatrics.*

al[397]											
Xiao, et al[398]	2020	yes	yes	yes	yes	yes	yes	yes	yes	yes	9
Andegiorgish, et al[399]	2012	yes	yes	yes	yes	yes	yes	yes	yes	yes	9
Yao, et al[400]	2014	no	yes	yes	yes	yes	yes	no	yes	yes	7
Zhou, et al[401]	2010	yes	yes	yes	no	yes	yes	yes	no	yes	7
Li, et al[402]	2013	no	yes	yes	yes	yes	yes	yes	yes	yes	8
Zhang, et al[403]	2013	yes	no	yes	yes	yes	yes	yes	yes	yes	8
Dong, et al[404]	2012	yes	yes	yes	yes	yes	yes	yes	yes	yes	9
Zhou, et al[405]	2021	yes	yes	yes	yes	yes	no	yes	yes	yes	8
Zhao, et al[406]	2017	yes	yes	yes	yes	yes	yes	yes	yes	yes	9
Ma, et al[407]	2011	yes	yes	yes	yes	yes	yes	yes	no	yes	8
Cao, et al[408]	2012	yes	yes	yes	yes	no	yes	yes	no	yes	7
He, et al[409]	2009	no	yes	yes	yes	yes	yes	yes	no	yes	7
Zhang, et al[410]	2012	yes	yes	yes	yes	yes	yes	no	yes	yes	8
He, et al[411]	2017	yes	yes	yes	yes	yes	yes	yes	yes	yes	9
Zou, et al[412]	2022	yes	yes	yes	yes	yes	yes	yes	yes	yes	9
Zheng, et al[413]	2022	yes	yes	yes	no	yes	yes	yes	no	yes	7
Zhang, et al[414]	2022	yes	yes	yes	yes	yes	yes	yes	yes	yes	9
Zhang, et al[415]	2022	yes	yes	yes	yes	yes	yes	yes	yes	yes	9
Zeng, et al[416]	2022	yes	yes	yes	yes	yes	yes	yes	yes	yes	9
You, et al[417]	2022	no	yes	yes	yes	yes	yes	yes	yes	yes	8
Yang, et al[418]	2022	yes	yes	yes	no	no	yes	no	yes	yes	6
Xu, et al[419]	2022	yes	yes	yes	yes	yes	yes	yes	no	yes	8
Wu, et al[420]	2022	yes	yes	yes	yes	yes	yes	yes	no	yes	8
Wang, et al[421]	2022	yes	yes	yes	yes	no	yes	yes	yes	yes	8
Wang, et al[422]	2022	yes	yes	yes	yes	yes	yes	yes	yes	yes	9
Iwata, et al[423]	2003	yes	yes	yes	yes	yes	yes	yes	yes	yes	9
Xiong, et al[424]	2010	no	yes	yes	yes	yes	yes	yes	yes	yes	8
Tan, et al[425]	2018	yes	yes	yes	no	yes	no	yes	yes	yes	7
Tan, et al[426]	2018	yes	yes	yes	yes	yes	yes	yes	yes	yes	9
Wang, et al[427]	2018	yes	yes	yes	yes	yes	yes	yes	yes	yes	9
Zhai, et al[428]	2018	yes	yes	yes	yes	no	yes	yes	no	yes	7
Zhang, et	2018	no	yes	yes	yes	yes	yes	yes	yes	yes	8

© 2024 Zhang X et al. *JAMA Pediatrics*.

al[429]											
Zhang, et al[430]	2018	yes	yes	yes	yes	yes	yes	yes	yes	yes	9
Zhang, et al[431]	2018	yes	yes	yes	no	yes	yes	yes	yes	yes	8
Cai, et al[432]	2019	no	yes	yes	yes	yes	yes	yes	yes	yes	8
Chen , et al[433]	2019	yes	yes	yes	yes	yes	yes	no	yes	yes	8
Lu, et al[434]	2019	yes	no	yes	yes	yes	yes	yes	yes	yes	8
Zhang, et al[435]	2019	yes	yes	yes	yes	no	yes	no	yes	yes	7
Zhao, et al[436]	2019	yes	yes	yes	yes	yes	yes	yes	yes	yes	9
Zou, et al[437]	2019	yes	yes	yes	yes	yes	yes	yes	yes	no	8
Duan, et al[438]	2020	yes	yes	yes	yes	yes	yes	no	yes	yes	8
Ke, et al[439]	2020	yes	yes	yes	yes	yes	yes	yes	yes	yes	9
Liu, et al[440]	2020	yes	yes	yes	yes	yes	yes	yes	yes	yes	9
Qian, et al[441]	2020	no	yes	no	yes	yes	yes	yes	yes	yes	7
Song, et al[442]	2020	yes	yes	yes	yes	yes	yes	yes	no	yes	8
Sun, et al[443]	2020	no	yes	yes	yes	yes	yes	yes	yes	yes	8
Xing, et al[444]	2020	yes	yes	yes	yes	yes	yes	yes	yes	yes	9
Fan, et al[445]	2010	yes	yes	yes	yes	yes	yes	no	yes	yes	8
Wang, et al[446]	2009	yes	yes	yes	yes	yes	yes	yes	yes	yes	9
Xu, et al[447]	2008	yes	yes	no	yes	yes	yes	yes	yes	yes	8
Xu, et al[448]	2008	yes	yes	yes	yes	yes	yes	no	no	yes	7
Li, et al[449]	2008	yes	yes	yes	no	yes	yes	yes	yes	yes	8
Shi, et al[450]	2007	yes	yes	yes	yes	no	yes	no	yes	yes	7
Liu, et al[451]	2007	yes	no	no	yes	yes	yes	yes	yes	yes	7
Yan, et al[452]	2006	yes	yes	yes	yes	yes	yes	yes	no	yes	8
Li, et al[453]	2006	yes	yes	yes	yes	no	yes	yes	yes	no	7
Jiang, et al[454]	2006	yes	yes	yes	no	yes	yes	yes	yes	yes	8
Zhu, et al[455]	2015	yes	yes	yes	yes	yes	yes	yes	yes	yes	9
Yuan, et al[456]	2015	yes	yes	yes	yes	no	yes	yes	yes	no	7
Xu, et al[457]	2015	yes	yes	yes	yes	yes	yes	yes	yes	yes	9
Xiao, et al[458]	2015	yes	yes	yes	yes	yes	yes	yes	yes	yes	9
Wu, et al[459]	2015	yes	yes	yes	no	yes	yes	yes	yes	yes	8
Wan, et al[460]	2015	yes	no	yes	yes	yes	yes	yes	yes	yes	8
Piernas, et al[461]	2015	yes	yes	yes	yes	yes	no	yes	yes	yes	8
Ma, et al[462]	2015	yes	yes	yes	yes	yes	no	no	yes	yes	7
Cao,	2015	yes	yes	yes	no	yes	yes	yes	yes	yes	8

© 2024 Zhang X et al. *JAMA Pediatrics.*

et al[463]											
Wang, et al[464]	2014	yes	yes	yes	yes	yes	yes	yes	yes	yes	9
Li, et al[465]	2014	yes	yes	yes	yes	yes	no	yes	yes	yes	8
He, et al[466]	2014	yes	yes	yes	yes	yes	no	yes	yes	yes	8
Dong, et al[467]	2014	yes	yes	yes	yes	yes	yes	yes	yes	yes	9
Dai, et al[468]	2014	yes	yes	yes	yes	yes	yes	yes	yes	yes	9
Chen, et al[469]	2011	yes	yes	yes	yes	no	yes	yes	yes	yes	8
Xu, et al[470]	2011	yes	no	yes	yes	no	yes	yes	yes	yes	7
Guo, et al[471]	2012	yes	yes	yes	yes	yes	yes	yes	yes	no	8
Jia, et al[472]	2012	yes	yes	yes	yes	no	yes	yes	yes	yes	8
Li, et al[473]	2012	yes	yes	yes	yes	yes	yes	no	yes	yes	8
Yi, et al[474]	2012	yes	yes	no	yes	yes	no	yes	yes	yes	7
Dong, et al[475]	2013	yes	yes	yes	yes	no	yes	yes	yes	yes	8
Chen, et al[476]	2016	yes	yes	yes	no	no	yes	yes	yes	no	6
Lei, et al[477]	2016	yes	yes	yes	yes	yes	yes	no	yes	yes	8
Liu, et al[478]	2016	yes	yes	yes	yes	yes	yes	yes	no	yes	8
Liu, et al[479]	2016	yes	yes	yes	yes	yes	yes	yes	yes	yes	9
Peng, et al[480]	2016	yes	yes	yes	yes	yes	no	yes	yes	yes	8
Wei, et al[481]	2016	yes	yes	yes	yes	yes	yes	yes	no	yes	8
Zhang, et al[482]	2016	yes	yes	no	yes	yes	yes	yes	yes	yes	8
Zhang, et al[483]	2016	yes	yes	yes	yes	yes	yes	no	yes	yes	8
Cai, et al[484]	2017	yes	yes	yes	no	yes	yes	no	yes	yes	7
Liu, et al[485]	2017	yes	yes	yes	yes	yes	yes	yes	no	yes	8
Liu, et al[486]	2017	yes	yes	yes	yes	yes	yes	yes	yes	yes	9
Zhang, et al[487]	2017	yes	no	yes	yes	yes	yes	no	yes	yes	7
Zong, et al[488]	2017	yes	yes	no	yes	no	yes	yes	no	yes	6
Chen, et al[489]	2018	yes	yes	yes	yes	yes	yes	no	yes	yes	8
Dong, et al[490]	2018	yes	yes	yes	yes	yes	yes	no	yes	yes	8
Gong, et al[491]	2018	yes	yes	yes	yes	yes	yes	yes	yes	yes	9
He, et al[492]	2018	yes	yes	yes	yes	yes	yes	no	yes	yes	8
Li, et al[493]	2018	yes	yes	yes	no	yes	yes	yes	no	yes	7
Liu, et al[494]	2018	yes	yes	yes	yes	yes	yes	yes	yes	yes	9
Rutayisire, et al[495]	2018	yes	yes	yes	yes	no	no	yes	yes	yes	7
Contreras, et al[496]	2015	yes	yes	yes	yes	yes	yes	yes	yes	yes	9

© 2024 Zhang X et al. *JAMA Pediatrics*.

Rincón-Pabón, et al[497]	2019	yes	yes	yes	yes	yes	yes	no	yes	yes	8
Flannagan, et al[498]	2020	yes	yes	yes	yes	yes	yes	yes	yes	yes	9
Ramírez-Vélez, et al[499]	2017	yes	yes	yes	yes	yes	yes	yes	yes	yes	9
Martinez-Ospina, et al[500]	2019	yes	yes	yes	yes	yes	yes	yes	yes	yes	9
McDonald, et al[501]	2009	yes	yes	yes	yes	yes	yes	yes	yes	no	8
Gomez, et al[502]	2007	yes	yes	yes	yes	yes	yes	yes	yes	yes	9
Arango, et al[503]	2011	yes	yes	yes	yes	yes	yes	yes	yes	no	8
Monge-Rojas, et al[504]	2022	yes	yes	yes	yes	no	yes	yes	no	yes	7
Gamboa-Gamboa, et al[505]	2021	yes	yes	yes	yes	yes	no	yes	yes	yes	8
Núñez-Rivas, et al[506]	2003	yes	yes	yes	yes	yes	yes	yes	yes	yes	9
Fossou, et al[507]	2020	no	yes	yes	yes	yes	yes	yes	yes	no	7
Juresa, et al[508]	2012	yes	yes	yes	yes	yes	yes	yes	yes	yes	9
Milanović, et al[509]	2020	yes	yes	yes	yes	no	no	yes	yes	no	6
Bili}-Kirin, et al[510]	2014	yes	yes	yes	yes	yes	yes	yes	no	yes	8
Pećin, et al[511]	2013	no	yes	yes	yes	yes	yes	yes	yes	yes	8
Savva, et al[512]	2005	yes	yes	yes	no	yes	yes	yes	no	yes	7
Savva, et al[513]	2008	yes	yes	yes	yes	yes	yes	yes	no	yes	8
Savva, et al[514]	2014	yes	yes	yes	yes	no	yes	yes	yes	yes	8
Lazarou, et al[515]	2008	yes	yes	yes	yes	yes	no	yes	yes	yes	8
Loucaides, et al[516]	2008	yes	yes	no	yes	yes	yes	yes	yes	yes	8
Loucaides, et al[517]	2010	yes	yes	yes	no	yes	yes	yes	no	yes	7
Tornaritis, et al[518]	2014	yes	yes	yes	yes	no	no	yes	no	yes	6
Savva, et al[519]	2014	yes	yes	yes	yes	yes	yes	no	yes	yes	8

© 2024 Zhang X et al. *JAMA Pediatrics.*

Vážná, et al[520]	2022	yes	yes	yes	yes	yes	yes	yes	yes	yes	9
Gouw, et al[521]	2010	yes	yes	yes	yes	yes	yes	yes	yes	yes	9
Brixval, et al[522]	2012	yes	yes	yes	yes	yes	yes	yes	yes	yes	9
Ajslev, et al[523]	2011	yes	yes	yes	yes	yes	yes	yes	no	yes	8
Mor, et al[524]	2015	yes	no	yes	yes	yes	yes	yes	yes	yes	8
Høyer, et al[525]	2014	yes	yes	yes	yes	no	yes	yes	yes	yes	8
Karlsen, et al[526]	2017	yes	no	no	yes	yes	yes	yes	yes	yes	7
Krue, et al[527]	2010	no	yes	yes	yes	yes	yes	yes	yes	yes	8
Rex, et al[528]	2014	yes	yes	yes	yes	no	yes	yes	yes	yes	8
Matthiessen, et al[529]	2014	yes	yes	yes	no	yes	yes	yes	no	yes	7
Klakk, et al[530]	2013	no	yes	yes	yes	yes	yes	yes	yes	yes	8
Kjelgaard, et al[531]	2017	yes	yes	yes	no	no	yes	yes	no	yes	6
Andrade, et al[532]	2014	yes	yes	yes	no	yes	yes	no	yes	yes	7
Abril, et al[533]	2013	yes	yes	yes	yes	yes	yes	yes	yes	yes	9
Freire, et al[534]	2014	no	yes	yes	yes	yes	no	yes	yes	yes	7
Ortiz, et al[535]	2014	yes	yes	yes	yes	yes	yes	yes	no	yes	8
Casaoulla, et al[536]	2017	no	yes	yes	yes	yes	yes	yes	yes	yes	8
Walrod, et al[537]	2018	yes	yes	yes	yes	yes	no	yes	yes	yes	8
Abd El-Aty, et al[538]	2020	yes	yes	yes	yes	yes	yes	yes	yes	yes	9
Abdel Wahed, et al[539]	2017	yes	yes	yes	yes	no	yes	yes	yes	yes	8
Abdelkarim, et al[540]	2017	yes	yes	yes	yes	yes	yes	no	yes	yes	8
Abou-Khadra, et al[541]	2022	yes	yes	yes	yes	yes	yes	no	yes	no	7
El-Sabely, et al[542]	2013	yes	yes	yes	yes	yes	yes	yes	no	yes	8
Badawi, et al[543]	2013	yes	yes	yes	yes	yes	yes	yes	yes	yes	9
Hassan, et al[544]	2008	yes	yes	yes	yes	yes	yes	yes	yes	yes	9
Hassan, et al[545]	2016	yes	yes	yes	yes	yes	yes	yes	no	yes	8
Talat, et al[546]	2016	yes	yes	yes	yes	yes	yes	yes	yes	yes	9

© 2024 Zhang X et al. *JAMA Pediatrics.*

Pérez, et al[547]	2020	no	yes	yes	no	yes	yes	no	yes	yes	6
Worku, et al[548]	2021	yes	yes	yes	yes	yes	yes	yes	yes	no	8
Wakayo, et al[549]	2016	yes	yes	yes	yes	yes	yes	yes	yes	yes	9
Abich, et al[550]	2020	yes	yes	yes	yes	yes	yes	yes	no	yes	8
Tadesse, et al[551]	2017	yes	yes	yes	yes	yes	yes	yes	yes	yes	9
Sorrie, et al[552]	2017	yes	yes	yes	yes	yes	yes	yes	yes	yes	9
Desalew, et al[553]	2017	yes	yes	yes	yes	yes	yes	yes	yes	yes	9
Yetubie, et al[554]	2010	yes	yes	yes	yes	yes	yes	yes	yes	yes	9
Gali, et al[555]	2017	yes	yes	yes	yes	yes	yes	yes	yes	no	8
Askal, et al[556]	2015	yes	yes	yes	no	yes	yes	yes	yes	yes	8
Mitiku, et al[557]	2019	yes	yes	yes	yes	yes	yes	yes	yes	no	8
Mekonnen, et al[558]	2018	yes	yes	yes	yes	yes	yes	yes	yes	yes	9
Pengpid, et al[559]	2015	yes	yes	yes	yes	yes	yes	yes	yes	yes	9
Petersen, et al[560]	2014	yes	yes	yes	yes	yes	yes	yes	yes	yes	9
Sarkkola, et al[561]	2022	yes	yes	no	yes	yes	no	yes	yes	yes	7
Eloranta, et al[562]	2012	yes	yes	yes	yes	yes	yes	yes	yes	yes	9
Laitinen, et al[563]	2012	yes	yes	yes	yes	yes	yes	yes	yes	no	8
Saari, et al[564]	2015	yes	yes	yes	yes	yes	yes	no	yes	yes	8
Parikka, et al[565]	2015	yes	yes	yes	yes	yes	yes	yes	yes	yes	9
Vuorela, et al[566]	2010	yes	yes	yes	yes	yes	yes	yes	yes	yes	9
Veltsista, et al[567]	2010	yes	yes	no	yes	no	no	yes	yes	yes	6
Vanhala, et al[568]	2009	yes	yes	yes	yes	yes	yes	yes	yes	yes	9
Fogelholm, et al[569]	2008	yes	yes	yes	yes	yes	yes	yes	yes	yes	9
Virtanen, et al[570]	2015	yes	yes	yes	yes	yes	yes	yes	no	yes	8
Palomäki, et al[571]	2015	yes	yes	yes	yes	yes	yes	yes	yes	yes	9
Vanhelst, et al[572]	2022	no	yes	no	yes	yes	yes	yes	yes	yes	7
Roth, et al[573]	2022	yes	yes	yes	yes	yes	yes	yes	no	yes	8

© 2024 Zhang X et al. *JAMA Pediatrics*.

Vanhelst, et al[574]	2021	yes	yes	yes	yes	yes	yes	yes	yes	yes	9
Luiggi, et al[575]	2021	yes	yes	yes	yes	yes	yes	yes	yes	no	8
Dupuy, et al[576]	2011	yes	yes	yes	yes	yes	yes	yes	yes	yes	9
Thibault, et al[577]	2013	yes	yes	yes	yes	yes	yes	yes	yes	yes	9
Thibault, et al[578]	2010	yes	no	yes	yes	yes	yes	no	yes	yes	7
Carriere, et al[579]	2015	yes	yes	yes	no	yes	yes	yes	yes	no	7
Salanave, et al[580]	2009	yes	yes	yes	yes	yes	yes	yes	yes	yes	9
Heude, et al[581]	2003	yes	yes	yes	yes	yes	yes	yes	yes	yes	9
Legleye, et al[582]	2014	yes	no	yes	yes	yes	no	yes	yes	yes	7
Chau, et al[583]	2013	yes	yes	yes	yes	yes	yes	yes	yes	yes	9
Jouret, et al[584]	2007	yes	yes	yes	yes	yes	yes	yes	yes	yes	9
Pitrou, et al[585]	2010	yes	yes	yes	yes	yes	yes	yes	no	yes	8
Kleiser, et al[586]	2003	yes	yes	yes	yes	yes	yes	yes	yes	yes	9
Rolland-Cachera, et al[587]	2002	yes	yes	no	yes	yes	yes	yes	yes	yes	8
Tubert-Jeannin, et al[588]	2018	yes	yes	yes	yes	yes	yes	yes	yes	yes	9
Kêkê, et al[589]	2015	yes	yes	yes	yes	yes	yes	yes	yes	yes	9
Vanhelst, et al[590]	2017	yes	yes	yes	yes	yes	yes	yes	yes	yes	9
Wang, et al[591]	2023	yes	yes	yes	yes	no	yes	yes	yes	no	7
Hoebel, et al[592]	2022	no	yes	no	yes	yes	yes	yes	no	yes	6
Zhou, et al[593]	2021	yes	yes	yes	yes	yes	yes	no	no	yes	7
Nguyen, et al[594]	2021	yes	yes	yes	yes	yes	yes	yes	yes	yes	9
Liu, et al[595]	2021	yes	yes	yes	yes	no	yes	yes	yes	yes	8
Willerhausen, et al[596]	2007	yes	yes	yes	yes	yes	yes	yes	no	yes	8
Kesztyüs, et al[597]	2013	yes	yes	yes	yes	no	yes	yes	yes	yes	8
Negal, et al[598]	2009	yes	yes	yes	yes	yes	yes	yes	yes	no	8
Boneberger, et al[599]	2009	yes	yes	yes	yes	yes	yes	yes	yes	yes	9

© 2024 Zhang X et al. *JAMA Pediatrics*.

Kromeyer-Hauschild, et al[600]	2007	yes	no	yes	no	yes	yes	yes	yes	yes	7
Landsberg, et al[601]	2010	yes	yes	yes	no	yes	yes	yes	no	yes	7
Toschke, et al[602]	2005	yes	yes	yes	yes	yes	yes	yes	yes	yes	9
Toschke, et al[603]	2003	yes	yes	yes	yes	yes	yes	yes	yes	yes	9
Kries, et al[604]	2008	yes	yes	yes	yes	yes	yes	yes	yes	yes	9
Raum, et al[605]	2011	yes	yes	yes	yes	yes	yes	yes	yes	no	8
Pei, et al[606]	2013	yes	yes	yes	yes	yes	yes	yes	yes	yes	9
Kleiser, et al[607]	2009	yes	yes	yes	no	yes	yes	yes	no	yes	7
Toschke, et al[608]	2007	no	yes	yes	yes	yes	yes	yes	yes	yes	8
Weyermann, et al[609]	2006	yes	yes	yes	yes	yes	yes	yes	yes	yes	9
Hoffmann, et al[610]	2019	yes	yes	yes	yes	no	yes	yes	no	yes	7
Suchert, et al[611]	2016	yes	yes	yes	yes	yes	yes	yes	no	yes	8
Will, et al[612]	2005	yes	yes	yes	yes	yes	yes	yes	yes	yes	9
Rapp, et al[613]	2005	yes	yes	yes	yes	yes	yes	yes	yes	yes	9
Lamerz, et al[614]	2005	yes	yes	yes	yes	yes	yes	yes	yes	yes	9
Graf, et al[615]	2004	yes	yes	yes	no	yes	yes	yes	yes	yes	8
Graf, et al[616]	2004	yes	yes	yes	no	yes	yes	yes	no	yes	7
Reich, et al[617]	2003	yes	yes	yes	yes	yes	yes	yes	yes	yes	9
Würbach, et al[618]	2009	yes	no	yes	no	yes	yes	yes	yes	no	6
Warschburger, et al[619]	2009	yes	yes	yes	yes	yes	yes	yes	yes	yes	9
Toschke, et al[620]	2009	yes	yes	yes	yes	yes	yes	yes	yes	yes	9
Sporišević, et al[621]	2009	yes	yes	yes	yes	yes	yes	yes	yes	no	8
Koller, et al[622]	2009	yes	yes	yes	yes	yes	yes	no	yes	yes	8
De Toia , et al[623]	2009	yes	yes	yes	yes	yes	yes	yes	yes	yes	9
Bayer, et al[624]	2009	yes	yes	yes	yes	yes	yes	yes	yes	yes	9
Bau, et al[625]	2009	no	yes	yes	no	yes	yes	yes	yes	yes	7
Nagel, et al[626]	2008	yes	yes	yes	yes	yes	yes	yes	yes	no	8
Mauch	2008	yes	yes	yes	yes	no	yes	yes	yes	yes	8

© 2024 Zhang X et al. *JAMA Pediatrics.*

, et al[627]											
Jahnke, et al[628]	2008	yes	yes	yes	yes	yes	yes	yes	yes	yes	9
Herpertz-Dahlmann, et al[629]	2008	yes	yes	yes	yes	yes	yes	yes	yes	yes	9
Willershausen, et al[630]	2007	no	yes	no	yes	yes	yes	yes	yes	yes	7
Kobel, et al[631]	2015	yes	yes	yes	yes	yes	yes	yes	yes	yes	9
Brettschneider, et al[632]	2015	yes	yes	yes	no	yes	yes	yes	yes	yes	8
Akoto, et al[633]	2022	no	yes	yes	yes	yes	yes	no	yes	yes	7
Agaba, et al[634]	2022	yes	yes	yes	yes	yes	yes	yes	yes	yes	9
Aboagye, et al[635]	2022	yes	yes	yes	yes	yes	yes	yes	yes	yes	9
Adom, et al[636]	2019	yes	yes	yes	yes	yes	yes	yes	no	yes	8
Amidu, et al[637]	2013	yes	yes	yes	no	yes	yes	yes	yes	yes	8
Hohammed, et al[638]	2012	no	yes	yes	yes	yes	yes	no	yes	yes	7
Intiful, et al[639]	2013	yes	yes	yes	yes	no	yes	yes	no	yes	7
Obiricorang, et al[640]	2015	yes	yes	yes	yes	yes	yes	yes	yes	yes	9
Aryeetey, et al[641]	2017	yes	yes	yes	yes	yes	yes	no	yes	yes	8
Atsu, et al[642]	2017	yes	yes	yes	yes	yes	yes	yes	yes	yes	9
Annan-Asare, et al[643]	2017	yes	yes	yes	yes	no	no	yes	yes	yes	7
Kwabla, et al[644]	2018	yes	yes	yes	yes	yes	yes	no	yes	yes	8
Gyamfi, et al[645]	2019	yes	yes	yes	yes	yes	yes	yes	no	yes	8
Ganle, et al[646]	2019	yes	yes	yes	yes	no	yes	yes	yes	yes	8
Mogre, et al[647]	2013	yes	yes	yes	yes	yes	yes	yes	yes	yes	9
Makri, et al[648]	2022	yes	yes	yes	no	yes	yes	yes	yes	yes	8
Kostopoulou, et al[649]	2021	yes	yes	yes	yes	yes	yes	yes	yes	yes	9
Pikramenou, et al[650]	2016	no	yes	yes	yes	yes	yes	yes	yes	no	7
Trikaliotis, et al[651]	2011	yes	yes	yes	yes	yes	yes	yes	yes	yes	9

© 2024 Zhang X et al. *JAMA Pediatrics.*

Grigorakis, et al[652]	2016	yes	no	yes	yes	yes	yes	yes	yes	no	7
Kleanthous, et al[653]	2016	yes	yes	yes	yes	yes	no	yes	yes	yes	8
Manios, et al[654]	2013	yes	yes	no	yes	yes	yes	yes	yes	no	7
Manios, et al[655]	2011	yes	yes	no	yes	yes	yes	yes	yes	yes	8
Tokmakidis, et al[656]	2006	no	yes	yes	yes	yes	yes	yes	yes	no	7
Risvas, et al[657]	2012	yes	yes	yes	yes	yes	yes	no	yes	yes	8
Daraki, et al[658]	2015	yes	yes	yes	yes	yes	no	no	yes	yes	7
Lamprokostopoulou, et al[659]	2019	no	yes	yes	yes	yes	yes	yes	yes	yes	8
Kontogianni, et al[660]	2010	yes	yes	no	yes	yes	yes	yes	yes	yes	8
Kosti, et al[661]	2007	yes	yes	yes	yes	yes	yes	yes	yes	no	8
Lagiou, et al[662]	2008	yes	yes	yes	no	yes	no	yes	yes	yes	7
Cassimos, et al[663]	2011	yes	no	yes	yes	yes	yes	yes	yes	yes	8
Krassas, et al[664]	2004	yes	yes	yes	yes	yes	yes	yes	yes	yes	9
Angelopoulos, et al[665]	2006	yes	yes	yes	yes	no	yes	yes	yes	yes	8
Magkos, et al[666]	2006	yes	yes	yes	yes	yes	no	yes	yes	no	7
Magkos, et al[667]	2006	yes	yes	yes	yes	yes	no	yes	yes	yes	8
Papadimitriou, et al[668]	2006	yes	yes	yes	yes	yes	yes	yes	yes	yes	9
Manios, et al[669]	2007	yes	yes	yes	yes	yes	yes	no	no	yes	7
Kamtsios, et al[670]	2008	yes	yes	yes	yes	yes	yes	yes	yes	yes	9
Linardakis, et al[671]	2008	yes	yes	yes	yes	yes	yes	yes	no	yes	8
Hassapidou, et al[672]	2009	yes	yes	yes	yes	yes	yes	no	yes	yes	8
Kollias, et al[673]	2009	yes	yes	yes	yes	yes	no	yes	yes	yes	8
Koroni, et al[674]	2009	yes	yes	yes	yes	yes	yes	yes	yes	no	8
Mavrakanas,	2009	yes	yes	yes	yes	yes	yes	yes	yes	yes	9

© 2024 Zhang X et al. *JAMA Pediatrics.*

et al[675]											
Yannakoulia, et al[676]	2010	no	yes	yes	yes	yes	yes	yes	no	no	6
Hassapidou, et al[677]	2015	yes	no	yes	yes	yes	yes	yes	yes	yes	8
Spathopoulos, et al[678]	2009	yes	yes	yes	yes	no	yes	yes	yes	yes	8
Antonogeorgos, et al[679]	2012	yes	yes	yes	yes	yes	no	yes	no	yes	7
Nassis, et al[680]	2005	no	yes	yes	yes	yes	yes	no	yes	yes	7
Manios, et al[681]	2004	yes	no	yes	yes	yes	yes	yes	yes	yes	8
Poulimeneas, et al[682]	2019	yes	yes	yes	yes	yes	yes	yes	no	yes	8
Vazquez, et al[683]	2019	yes	yes	yes	yes	yes	yes	no	yes	yes	8
Katsagoni, et al[684]	2020	yes	yes	yes	yes	yes	yes	no	yes	no	7
Notara ,et al[685]	2020	yes	yes	no	yes	yes	yes	yes	yes	yes	8
Mirkopoulou, et al[686]	2010	no	yes	yes	yes	yes	yes	yes	yes	yes	8
Lazarou, et al[687]	2010	yes	yes	yes	no	yes	yes	no	yes	yes	7
Antonogeorgos, et al[688]	2010	yes	yes	yes	yes	yes	yes	no	yes	yes	8
Karatzi , et al[689]	2009	no	yes	yes	yes	yes	no	yes	yes	yes	7
Yannakoulia, et al[690]	2008	yes	yes	yes	yes	no	yes	no	no	yes	6
Hassapidou, et al[691]	2006	no	yes	yes	yes	yes	yes	yes	no	yes	7
Vafeiadi, et al[692]	2015	yes	yes	yes	yes	no	yes	yes	yes	yes	8
Sourani, et al[693]	2015	yes	yes	yes	yes	yes	yes	yes	yes	yes	9
Patsopoulou, et al[694]	2015	yes	yes	yes	yes	yes	yes	yes	no	yes	8
Kambas, et al[695]	2015	yes	yes	yes	yes	no	yes	yes	yes	yes	8
Grammatikopoulou, et al[696]	2014	yes	yes	no	yes	yes	no	yes	yes	yes	7
Antonogeorgos, et	2011	yes	yes	yes	yes	yes	yes	yes	no	no	7

© 2024 Zhang X et al. *JAMA Pediatrics*.

al[697]											
Athanasopoulos, et al[698]	2011	yes	yes	yes	yes	no	yes	yes	yes	yes	8
Farajian, et al[699]	2011	yes	yes	yes	yes	yes	yes	yes	no	yes	8
Michalopoulou, et al[700]	2011	yes	yes	yes	yes	yes	yes	yes	yes	yes	9
Jelastopulu, et al[701]	2012	yes	yes	yes	yes	yes	yes	yes	yes	yes	9
Kyriazis, et al[702]	2012	yes	yes	yes	yes	no	yes	yes	no	yes	7
Tambalis, et al[703]	2013	yes	yes	yes	yes	yes	no	yes	yes	yes	8
Poulimeneas, et al[704]	2016	yes	yes	yes	yes	yes	yes	no	no	yes	7
Garoufi, et al[705]	2017	yes	yes	yes	yes	yes	yes	yes	no	yes	8
Koulouvaris, et al[706]	2018	yes	yes	yes	no	yes	yes	yes	yes	no	7
Manios, et al[707]	2018	yes	yes	yes	yes	yes	yes	yes	yes	yes	9
Alvarado, et al[708]	2009	yes	yes	yes	yes	yes	no	yes	yes	yes	8
Torres, et al[709]	2014	yes	yes	yes	no	yes	yes	yes	no	yes	7
Tung, et al[710]	2021	yes	yes	yes	yes	yes	yes	yes	yes	yes	9
Huang, et al[711]	2019	yes	yes	yes	yes	yes	yes	no	yes	yes	8
Wing, et al[712]	2009	yes	yes	yes	yes	no	yes	no	yes	no	6
Wang, et al[713]	2019	no	yes	yes	yes	yes	yes	yes	yes	yes	8
Chan, et al[714]	2013	no	yes	yes	yes	yes	yes	yes	yes	no	7
Wang, et al[715]	2017	yes	yes	yes	yes	no	yes	yes	yes	yes	8
Wong, et al[716]	2005	yes	yes	yes	no	yes	yes	yes	yes	yes	8
Leung, et al[717]	2009	yes	yes	yes	yes	yes	yes	yes	no	yes	8
Ko, et al[718]	2008	no	yes	yes	yes	yes	no	yes	yes	yes	7
Cheung, et al[719]	2007	yes	yes	yes	yes	no	no	yes	yes	yes	7
Knowles, et al[720]	2015	yes	no	yes	yes	no	yes	yes	yes	yes	7
Chan, et al[721]	2014	yes	yes	yes	yes	yes	no	yes	yes	yes	8
Ip, et al[722]	2017	yes	yes	yes	yes	yes	yes	yes	yes	yes	9
Lee, et al[723]	2017	yes	yes	yes	yes	yes	yes	no	yes	yes	8
Baráth, et al[724]	2010	yes	yes	yes	yes	yes	yes	yes	yes	yes	9

© 2024 Zhang X et al. *JAMA Pediatrics*.

Bjørnarå, et al[725]	2014	yes	yes	yes	yes	yes	yes	yes	yes	yes	9
Antal, et al[726]	2009	yes	yes	yes	yes	no	yes	yes	yes	yes	8
Erdei, et al[727]	2018	yes	yes	yes	no	yes	yes	yes	yes	yes	8
Jakab, et al[728]	2018	yes	yes	yes	yes	yes	yes	yes	no	yes	8
Aanesen, et al[729]	2020	yes	yes	yes	yes	yes	yes	yes	yes	yes	9
Sergentanis, et al[730]	2021	yes	yes	yes	yes	yes	yes	yes	yes	yes	9
Hrafnkelsson, et al[731]	2009	yes	yes	yes	yes	yes	yes	no	yes	no	7
Saha, et al[732]	2022	yes	yes	yes	yes	no	yes	yes	no	yes	7
Dabas, et al[733]	2022	yes	yes	yes	yes	yes	yes	no	yes	no	7
Arushi, et al[734]	2022	no	yes	yes	yes	yes	yes	yes	yes	yes	8
Thomas, et al[735]	2021	yes	yes	yes	yes	yes	yes	no	no	yes	7
Seema, et al[736]	2021	yes	yes	yes	yes	yes	yes	yes	yes	yes	9
Moitra, et al[737]	2021	yes	yes	yes	yes	no	yes	no	yes	yes	7
Elangovan, et al[738]	2012	yes	yes	yes	yes	yes	yes	yes	yes	yes	9
Subramaniam, et al[739]	2011	yes	no	yes	yes	yes	yes	yes	yes	yes	8
Shailee, et al[740]	2018	yes	yes	yes	yes	yes	yes	yes	no	no	7
Balaram, et al[741]	2017	yes	yes	yes	yes	yes	yes	yes	yes	yes	9
Goyal, et al[742]	2011	yes	yes	yes	no	yes	yes	no	yes	yes	7
Jain, et al[743]	2010	yes	yes	yes	yes	yes	yes	yes	yes	yes	9
Gautam, et al[744]	2019	yes	yes	yes	yes	yes	yes	yes	yes	yes	9
Bharati, et al[745]	2008	yes	yes	yes	yes	yes	yes	no	yes	yes	8
Kotian, et al[746]	2010	yes	yes	yes	yes	no	yes	yes	yes	yes	8
Warraich, et al[747]	2009	yes	yes	yes	yes	yes	yes	yes	yes	yes	9
Mushtag, et al[748]	2011	yes	yes	yes	no	yes	yes	yes	no	no	6
Kumar, et al[749]	2007	yes	yes	yes	yes	yes	yes	yes	yes	yes	9
Tiwari, et al[750]	2014	yes	yes	yes	yes	yes	yes	yes	yes	no	8
Bansal	2022	yes	yes	yes	yes	yes	yes	no	yes	yes	8

© 2024 Zhang X et al. *JAMA Pediatrics.*

, et al[751]											
Baruah, et al[752]	2018	yes	yes	yes	yes	yes	yes	yes	yes	no	8
Baston, et al[753]	2014	yes	yes	yes	yes	yes	yes	no	yes	yes	8
Khadilkar, et al[754]	2011	yes	yes	yes	yes	yes	yes	no	yes	yes	8
Sultan, et al[755]	2008	no	yes	yes	yes	yes	yes	no	yes	yes	7
Gupta, et al[756]	2011	yes	yes	yes	yes	yes	yes	no	yes	yes	8
Sharma, et al[757]	2007	yes	yes	yes	yes	yes	yes	no	yes	yes	8
Ramachandran, et al[758]	2002	yes	yes	yes	yes	yes	yes	yes	yes	yes	9
Bose, et al[759]	2007	no	yes	yes	yes	yes	yes	yes	yes	yes	8
Mehta, et al[760]	2007	yes	yes	yes	yes	yes	yes	yes	yes	yes	9
Laxmaiah, et al[761]	2007	yes	yes	yes	no	yes	no	yes	yes	yes	7
Raj, et al[762]	2007	yes	yes	yes	yes	yes	yes	yes	no	yes	8
Iyer, et al[763]	2011	no	yes	yes	yes	yes	yes	yes	yes	yes	8
Premanath, et al[764]	2010	no	yes	yes	yes	yes	yes	yes	yes	yes	8
Marwaha, et al[765]	2006	yes	yes	yes	yes	yes	yes	yes	yes	yes	9
Kumar, et al[766]	2008	yes	yes	yes	yes	yes	yes	yes	yes	yes	9
Sood, et al[767]	2007	yes	yes	yes	yes	yes	yes	no	yes	yes	8
Goyal, et al[768]	2010	no	yes	yes	yes	yes	no	yes	yes	yes	7
Ghosh, et al[769]	2011	no	yes	yes	yes	yes	yes	yes	yes	yes	8
Mahajan, et al[770]	2011	yes	yes	yes	yes	yes	yes	no	yes	yes	8
Cherian, et al[771]	2012	yes	yes	yes	yes	yes	no	yes	yes	yes	8
Thakre, et al[772]	2011	yes	no	yes	yes	no	yes	yes	yes	yes	7
Keerthan, et al[773]	2011	yes	yes	no	yes	yes	yes	yes	no	yes	7
Vohra, et al[774]	2011	yes	yes	no	yes	yes	yes	yes	yes	yes	8
Chakraborty, et al[775]	2011	yes	yes	yes	yes	yes	yes	yes	yes	yes	9
Sidhu, et al[776]	2005	yes	yes	no	yes	yes	yes	yes	yes	yes	8
Sidhu, et al[777]	2006	yes	yes	yes	yes	yes	yes	no	no	yes	7
Misra, et al[778]	2011	yes	yes	yes	no	yes	no	yes	yes	yes	7

© 2024 Zhang X et al. *JAMA Pediatrics.*

Jagadesan, et al[779]	2014	yes	yes	yes	no	yes	yes	yes	yes	yes	8
Kaur, et al[780]	2008	yes	yes	yes	yes	yes	yes	yes	no	yes	8
Chhabra, et al[781]	2012	yes	yes	yes	yes	yes	yes	yes	no	yes	8
Chhatwal, et al[782]	2004	yes	yes	yes	yes	yes	yes	yes	yes	yes	9
Saikia, et al[783]	2018	yes	yes	yes	yes	no	yes	yes	no	yes	7
Anebaracy, et al[784]	2019	yes	yes	yes	yes	no	yes	no	yes	yes	7
Chandra, et al[785]	2019	yes	yes	yes	yes	no	yes	yes	yes	yes	8
Minhas, et al[786]	2019	yes	yes	yes	yes	yes	no	yes	yes	yes	8
Mohan, et al[787]	2019	yes	yes	yes	yes	yes	yes	yes	yes	yes	9
Pinni, et al[788]	2019	yes	yes	yes	yes	yes	yes	yes	yes	yes	9
Sharma, et al[789]	2019	yes	yes	yes	yes	yes	yes	yes	yes	yes	9
Swaminathan, et al[790]	2019	yes	yes	yes	no	no	yes	no	yes	yes	6
Singh, et al[791]	2020	yes	yes	yes	yes	yes	no	yes	yes	yes	8
Singh, et al[792]	2020	yes	yes	yes	yes	yes	yes	yes	yes	yes	9
Solanki, et al[793]	2020	no	yes	yes	yes	yes	yes	yes	yes	yes	8
Ramesh, et al[794]	2010	yes	yes	yes	yes	yes	yes	yes	no	yes	8
Sharma, et al[795]	2009	no	yes	yes	yes	yes	yes	yes	yes	yes	8
Rao, et al[796]	2009	no	yes	yes	no	no	yes	yes	yes	yes	6
Ghosh, et al[797]	2009	yes	yes	yes	yes	yes	yes	no	yes	yes	8
Singh, et al[798]	2007	yes	yes	yes	yes	yes	yes	yes	no	yes	8
Senbanjo, et al[799]	2007	no	yes	yes	yes	yes	yes	yes	yes	no	7
Kasi, et al[800]	2015	no	yes	yes	no	no	yes	yes	yes	yes	6
Joseph, et al[801]	2015	yes	yes	yes	yes	yes	yes	yes	yes	yes	9
Faizi, et al[802]	2015	yes	no	yes	yes	yes	yes	no	yes	yes	7
Basha, et al[803]	2015	yes	yes	yes	yes	yes	yes	yes	yes	yes	9
Jani, et al[804]	2014	yes	yes	yes	no	yes	yes	no	no	yes	6
Gregori, et al[805]	2014	yes	yes	yes	yes	yes	yes	yes	no	yes	8
Ghosh	2014	no	yes	yes	no	yes	yes	yes	yes	yes	7

© 2024 Zhang X et al. *JAMA Pediatrics*.

, et al[806]											
Aroor, et al[807]	2014	yes	yes	yes	yes	yes	yes	yes	yes	yes	9
Honne, et al[808]	2012	no	yes	yes	yes	yes	yes	no	yes	yes	7
Sakeenabi, et al[809]	2012	yes	yes	yes	yes	yes	yes	yes	no	yes	8
Chakravathy, et al[810]	2013	yes	yes	yes	yes	yes	no	yes	yes	yes	8
Maiti, et al[811]	2013	yes	yes	yes	no	yes	yes	yes	no	yes	7
Bhargava, et al[812]	2016	yes	yes	yes	yes	yes	yes	yes	yes	yes	9
Deepika, et al[813]	2016	yes	yes	yes	yes	no	yes	no	yes	yes	7
Pawar, et al[814]	2016	yes	yes	yes	yes	yes	yes	yes	yes	no	8
Prasad, et al[815]	2016	no	yes	yes	yes	yes	yes	no	yes	yes	7
Saikia, et al[816]	2016	yes	yes	yes	yes	no	yes	yes	yes	yes	8
Bharati, et al[817]	2017	yes	yes	yes	yes	yes	yes	yes	yes	yes	9
Choudhary, et al[818]	2017	yes	yes	no	yes	yes	yes	no	yes	yes	7
Eshwar, et al[819]	2017	yes	yes	no	yes	no	yes	yes	yes	yes	7
Ganie, et al[820]	2017	no	yes	yes	yes	yes	yes	no	yes	yes	7
Haq, et al[821]	2017	yes	yes	yes	yes	yes	yes	yes	yes	no	8
Kumar, et al[822]	2017	yes	yes	yes	yes	yes	yes	yes	yes	yes	9
Mishra, et al[823]	2017	yes	yes	yes	yes	yes	no	yes	no	yes	7
Shah, et al[824]	2017	yes	yes	yes	no	yes	no	yes	yes	yes	7
Greene-Cramer, et al[825]	2018	yes	yes	yes	yes	yes	yes	yes	yes	no	8
Andriani, et al[826]	2021	yes	yes	yes	yes	yes	yes	yes	yes	yes	9
Sarintohe, et al[827]	2022	yes	yes	yes	yes	yes	yes	yes	yes	yes	9
Windiani, et al[828]	2021	yes	yes	yes	yes	yes	no	yes	yes	yes	8
Aditya, et al[829]	2017	yes	yes	yes	yes	yes	yes	yes	yes	yes	9
Agustina, et al[830]	2021	yes	yes	yes	yes	yes	yes	yes	yes	yes	9
Collins, et al[831]	2008	yes	yes	yes	yes	yes	no	yes	yes	yes	8

© 2024 Zhang X et al. *JAMA Pediatrics*.

Rachmi, et al[832]	2016	yes	yes	yes	yes	yes	yes	yes	yes	yes	9
Yuwanita, et al[833]	2018	yes	yes	yes	yes	yes	yes	no	yes	yes	8
Febriani, et al[834]	2019	no	yes	yes	yes	yes	yes	no	yes	yes	7
Maehara, et al[835]	2019	yes	yes	yes	yes	yes	yes	yes	yes	yes	9
Melinda, et al[836]	2019	yes	yes	yes	yes	yes	yes	yes	yes	no	8
Rizkiriani, et al[837]	2014	yes	yes	no	yes	yes	yes	yes	yes	yes	8
Syahrul, et al[838]	2016	yes	yes	yes	yes	no	no	yes	yes	yes	7
Yulia, et al[839]	2017	yes	yes	yes	yes	yes	yes	yes	yes	yes	9
Kalsum, et al[840]	2018	yes	yes	yes	yes	yes	yes	yes	no	yes	8
Moradi, et al[841]	2016	yes	yes	yes	no	yes	yes	no	yes	yes	7
Soheilipour, et al[842]	2022	yes	yes	yes	yes	yes	yes	yes	yes	no	8
Barati, et al[843]	2022	yes	yes	yes	yes	yes	yes	yes	yes	yes	9
Mohammadi, et al[844]	2021	no	yes	yes	yes	no	yes	yes	yes	yes	7
Entezarmahdi, et al[845]	2021	yes	yes	yes	yes	yes	yes	yes	yes	yes	9
Jari, et al[846]	2015	yes	yes	yes	yes	yes	yes	yes	yes	no	8
Bagherian, et al[847]	2013	yes	yes	yes	no	yes	yes	yes	yes	yes	8
Sadeghi, et al[848]	2011	yes	yes	yes	yes	yes	yes	yes	yes	yes	9
Shahraki, et al[849]	2013	yes	yes	yes	yes	yes	yes	yes	no	yes	8
Agha-Alinejad, et al[850]	2015	yes	yes	yes	yes	yes	yes	yes	yes	yes	9
Javedan, et al[851]	2016	yes	yes	yes	yes	yes	yes	yes	no	yes	8
Fatemeh, et al[852]	2012	yes	yes	yes	yes	yes	yes	yes	yes	yes	9
Ahmadi, et al[853]	2014	yes	yes	yes	yes	yes	no	no	yes	yes	7
Badeli, et al[854]	2016	yes	yes	yes	yes	yes	yes	yes	yes	yes	9
Bahreini, et al[855]	2013	yes	yes	yes	no	no	yes	yes	yes	yes	7
Bahreynian, et al[856]	2015	yes	yes	yes	yes	yes	yes	yes	yes	yes	9

© 2024 Zhang X et al. *JAMA Pediatrics.*

Basiratnia, et al[857]	2013	yes	yes	yes	no	yes	yes	yes	yes	yes	8
Maddah, et al[858]	2010	yes	no	yes	yes	yes	yes	no	yes	yes	7
Kelishadi, et al[859]	2008	yes	yes	no	yes	yes	yes	yes	yes	no	7
Amini, et al[860]	2007	yes	no	yes	yes	yes	yes	yes	no	yes	7
Motlagh, et al[861]	2011	yes	yes	yes	yes	yes	yes	yes	yes	no	8
Ziaoddini, et al[862]	2010	yes	yes	yes	yes	no	yes	yes	yes	yes	8
Ayatollahi, et al[863]	2007	yes	yes	yes	no	yes	yes	yes	yes	yes	8
Khodaverdi, et al[864]	2011	yes	yes	yes	yes	yes	yes	yes	yes	no	8
Kajbaf, et al[865]	2011	yes	yes	yes	yes	yes	yes	yes	yes	yes	9
Vafa, et al[866]	2012	yes	yes	yes	yes	yes	yes	no	no	yes	7
Zarrati, et al[867]	2013	yes	yes	yes	yes	yes	yes	yes	yes	yes	9
Hajian-Tilaki, et al[868]	2012	yes	yes	yes	yes	no	yes	yes	yes	yes	8
Gaeini, et al[869]	2011	yes	yes	yes	yes	yes	yes	no	yes	yes	8
Montazerifar, et al[870]	2009	no	yes	yes	yes	yes	no	yes	yes	yes	7
Heidari, et al[871]	2014	yes	yes	no	yes	yes	yes	yes	yes	yes	8
Hajian-Tilaki, et al[872]	2011	yes	yes	yes	yes	no	no	yes	yes	yes	7
Gargari, et al[873]	2004	yes	yes	yes	yes	yes	yes	no	no	yes	7
Tebesh, et al[874]	2013	no	yes	yes	yes	yes	yes	yes	no	yes	7
Shafaghi, et al[875]	2014	yes	yes	yes	yes	no	yes	yes	yes	yes	8
Moayeri, et al[876]	2006	yes	yes	yes	yes	yes	yes	yes	yes	yes	9
Behzadnia, et al[877]	2012	yes	yes	yes	yes	yes	yes	yes	yes	yes	9
Maddah, et al[878]	2010	yes	yes	yes	yes	yes	yes	yes	yes	yes	9
Saeidlou, et al[879]	2014	yes	yes	yes	yes	yes	no	yes	no	yes	7
Kavehmanesh, et al[880]	2013	yes	yes	yes	yes	no	yes	yes	yes	yes	8
Hajian-Tilaki, et al[881]	2013	yes	yes	yes	yes	yes	yes	yes	yes	yes	9

© 2024 Zhang X et al. *JAMA Pediatrics.*

Mirhosseini, et al[882]	2012	yes	yes	yes	no	yes	yes	yes	no	yes	7
Heshmat, et al[883]	2015	yes	yes	no	yes	yes	yes	yes	yes	yes	8
Sadeghi, et al[884]	2007	yes	yes	yes	yes	yes	yes	yes	yes	yes	9
Zekavat, et al[885]	2014	yes	yes	yes	yes	no	yes	yes	no	yes	7
Ejtahed, et al[886]	2018	yes	yes	yes	yes	yes	yes	yes	yes	no	8
Salehi-Abargouei, et al[887]	2013	yes	yes	yes	no	yes	yes	yes	no	yes	7
Maddah, et al[888]	2009	yes	yes	yes	yes	yes	no	yes	yes	yes	8
Taheri, et al[889]	2013	yes	yes	yes	yes	yes	yes	yes	yes	yes	9
Zakeri, et al[890]	2012	yes	yes	yes	yes	yes	yes	yes	yes	yes	9
Jari, et al[891]	2014	yes	yes	yes	yes	no	yes	yes	no	yes	7
Sedaghat, et al[892]	2019	yes	yes	yes	yes	yes	yes	yes	yes	yes	9
Rafraf, et al[893]	2010	no	yes	yes	yes	yes	yes	no	yes	yes	7
Maddah, et al[894]	2010	yes	yes	yes	yes	yes	yes	yes	yes	yes	9
Maddah, et al[895]	2010	yes	yes	yes	yes	yes	yes	yes	yes	yes	9
Salem, et al[896]	2009	yes	yes	yes	yes	yes	yes	yes	no	yes	8
Maddah, et al[897]	2009	yes	yes	yes	yes	yes	yes	yes	yes	yes	9
Kelishadi, et al[898]	2009	yes	yes	yes	yes	yes	yes	no	yes	yes	8
Fallahzadeh, et al[899]	2009	yes	yes	yes	yes	yes	yes	yes	yes	yes	9
Azita, et al[900]	2009	yes	yes	no	yes	yes	yes	yes	yes	yes	8
Bidad, et al[901]	2008	yes	yes	yes	yes	no	yes	no	yes	yes	7
Rashidi, et al[902]	2007	yes	yes	yes	no	yes	yes	yes	yes	yes	8
Mozaffari, et al[903]	2007	yes	yes	yes	yes	yes	yes	yes	yes	yes	9
Maddah, et al[904]	2007	no	yes	yes	yes	yes	yes	yes	yes	yes	8
Tabriz, et al[905]	2015	yes	yes	yes	yes	yes	yes	no	yes	yes	8
Ghadimi, et al[906]	2015	yes	yes	yes	yes	no	yes	yes	yes	no	7
Jalali-Farah	2014	yes	yes	yes	yes	yes	no	yes	yes	yes	8

© 2024 Zhang X et al. *JAMA Pediatrics.*

ani, et al[907]											
Hatami, et al[908]	2014	no	yes	yes	yes	yes	yes	yes	yes	yes	8
Ahmadi, et al[909]	2014	yes	yes	yes	yes	yes	yes	yes	yes	yes	9
Motlagh, et al[910]	2011	yes	yes	yes	yes	yes	yes	yes	yes	yes	9
Hatami, et al[911]	2013	yes	yes	yes	no	yes	yes	no	yes	yes	7
Hassanzadeh-Rostami, et al[912]	2016	yes	yes	yes	yes	no	yes	yes	yes	yes	8
Keykhaei, et al[913]	2016	no	yes	yes	yes	yes	yes	yes	no	yes	7
Saeidlou, et al[914]	2016	yes	yes	yes	yes	no	yes	yes	yes	yes	8
Salehiniya, et al[915]	2016	yes	yes	yes	yes	yes	yes	yes	yes	yes	9
Darabiyan, et al[916]	2018	yes	yes	yes	yes	no	yes	yes	yes	yes	8
Ghobadi, et al[917]	2018	yes	yes	yes	yes	yes	yes	yes	yes	yes	9
Heshmat, et al[918]	2018	yes	yes	yes	yes	yes	yes	yes	yes	yes	9
Motlagh, et al[919]	2018	yes	yes	yes	yes	yes	yes	yes	yes	no	8
Parastar, et al[920]	2018	yes	yes	yes	yes	yes	yes	yes	yes	yes	9
Abood, et al[921]	2021	yes	yes	yes	yes	yes	no	yes	yes	yes	8
Lafta, et al[922]	2005	yes	yes	yes	yes	yes	yes	yes	yes	yes	9
Amin, et al[923]	2019	yes	no	yes	yes	yes	yes	yes	yes	yes	8
Lafta, et al[924]	2007	yes	yes	yes	yes	yes	yes	no	yes	yes	8
Musaiger, et al[925]	2016	yes	yes	yes	no	yes	yes	yes	yes	yes	8
Reulbach, et al[926]	2013	yes	yes	yes	yes	yes	yes	yes	yes	yes	9
Quinn, et al[927]	2022	yes	yes	yes	yes	yes	yes	no	yes	yes	8
Heslin, et al[928]	2023	yes	yes	yes	yes	yes	yes	yes	yes	yes	9
Barron, et al[929]	2009	yes	yes	yes	yes	no	yes	yes	yes	yes	8
Dowd, et al[930]	2015	yes	yes	yes	yes	yes	yes	no	yes	yes	8
Evans, et al[931]	2011	no	yes	yes	yes	yes	yes	yes	yes	yes	8
Whelton, et al[932]	2007	yes	yes	yes	yes	yes	yes	yes	yes	yes	9

© 2024 Zhang X et al. *JAMA Pediatrics.*

O'Neill, et al[933]	2007	yes	yes	yes	yes	no	yes	yes	yes	yes	8
Kelly, et al[934]	2019	yes	yes	yes	yes	yes	yes	yes	yes	yes	9
McMaster, et al[935]	2005	yes	no	yes	yes	yes	yes	yes	yes	yes	8
Belton, et al[936]	2010	yes	yes	yes	yes	yes	yes	no	yes	yes	8
Perry, et al[937]	2015	yes	yes	yes	yes	yes	yes	no	yes	yes	8
Pinhas-Hamiel, et al[938]	2009	yes	no	yes	yes	yes	no	yes	yes	yes	7
Dayan, et al[939]	2003	yes	yes	yes	yes	yes	yes	yes	yes	yes	9
Soskolne, et al[940]	2018	no	yes	yes	yes	yes	no	yes	yes	yes	7
Weisband, et al[941]	2020	yes	yes	yes	no	yes	yes	yes	yes	yes	8
Haug, et al[942]	2009	yes	yes	yes	yes	yes	yes	yes	yes	yes	9
Meyerovitch, et al[943]	2007	yes	yes	no	yes	yes	yes	yes	yes	yes	8
Goldberg, et al[944]	2014	yes	yes	yes	yes	no	yes	yes	no	yes	7
Kaufman-Shriqui, et al[945]	2013	yes	yes	yes	yes	yes	yes	yes	no	yes	8
Ram, et al[946]	2013	yes	yes	yes	yes	yes	yes	yes	yes	yes	9
Ahrens, et al[947]	2014	yes	no	yes	yes	yes	yes	no	yes	yes	7
Galfo, et al[948]	2022	yes	yes	yes	yes	yes	yes	yes	no	yes	8
Ashi, et al[949]	2019	yes	no	yes	yes	yes	yes	no	yes	yes	7
Costacurta, et al[950]	2011	yes	yes	yes	yes	no	yes	yes	yes	yes	8
Barba, et al[951]	2005	yes	yes	yes	yes	yes	no	yes	yes	yes	8
Barba, et al[952]	2006	no	yes	yes	yes	yes	yes	yes	yes	yes	8
Fioravanti, et al[953]	2018	yes	yes	yes	yes	yes	no	no	yes	yes	7
Ceschia, et al[954]	2016	yes	yes	yes	yes	yes	yes	yes	no	yes	8
Toselli, et al[955]	2014	yes	yes	yes	yes	no	yes	no	yes	yes	7
Caserta, et al[956]	2010	yes	yes	yes	yes	yes	yes	yes	yes	yes	9
Genovesi, et al[957]	2010	yes	yes	yes	no	no	yes	yes	yes	yes	7
Bertoncello,	2008	yes	yes	yes	yes	yes	yes	yes	yes	yes	9

© 2024 Zhang X et al. *JAMA Pediatrics.*

et al[958]											
Albertini, et al[959]	2008	yes	yes	yes	yes	yes	yes	yes	no	yes	8
Maffeis, et al[960]	2006	yes	yes	yes	yes	yes	yes	yes	yes	yes	9
Parrino, et al[961]	2012	yes	yes	yes	yes	yes	yes	no	yes	yes	8
Parrino, et al[962]	2016	yes	yes	yes	yes	no	yes	yes	yes	yes	8
Lombardo, et al[963]	2015	yes	yes	yes	yes	yes	yes	yes	no	yes	8
Cibella, et al[964]	2011	yes	yes	yes	yes	no	yes	yes	yes	yes	8
Ruiz, et al[965]	2016	yes	yes	yes	yes	yes	yes	yes	yes	yes	9
Genovesi, et al[966]	2005	yes	yes	yes	yes	no	yes	yes	yes	yes	8
Valerio, et al[967]	2003	no	yes	yes	yes	yes	yes	yes	yes	yes	8
Pecoraro, et al[968]	2003	yes	yes	yes	yes	no	yes	yes	no	yes	7
Collo, et al[969]	2019	yes	yes	yes	yes	yes	yes	yes	yes	yes	9
Toselli, et al[970]	2010	yes	yes	yes	yes	yes	yes	yes	yes	yes	9
Greco, et al[971]	2020	yes	yes	yes	yes	no	yes	yes	yes	yes	8
Binkin, et al[972]	2010	yes	yes	yes	yes	yes	yes	no	yes	yes	8
Rapa, et al[973]	2009	yes	yes	yes	yes	yes	yes	yes	yes	no	8
Caserta, et al[974]	2009	yes	yes	yes	yes	yes	yes	no	no	yes	7
Bonaccorsi, et al[975]	2009	yes	yes	yes	yes	yes	yes	yes	yes	yes	9
Turconi, et al[976]	2008	yes	yes	yes	yes	yes	yes	yes	yes	no	8
Maffeis, et al[977]	2008	yes	no	yes	yes	yes	yes	yes	yes	yes	8
Lazzeri, et al[978]	2008	yes	yes	yes	yes	no	yes	yes	yes	yes	8
Guida, et al[979]	2008	yes	yes	yes	yes	yes	yes	yes	yes	yes	9
Gualdi-Russo, et al[980]	2008	yes	yes	yes	yes	yes	yes	yes	yes	yes	9
Fuiano, et al[981]	2008	yes	yes	yes	no	yes	yes	no	yes	yes	7
Vidal, et al[982]	2006	yes	yes	yes	yes	yes	yes	yes	yes	yes	9
Menghetti, et al[983]	2015	no	yes	yes	yes	yes	yes	yes	yes	yes	8
Lazzeri, et	2015	yes	yes	yes	yes	no	yes	yes	no	yes	7

© 2024 Zhang X et al. *JAMA Pediatrics*.

al[984]											
Geremia, et al[985]	2015	yes	yes	yes	yes	yes	yes	no	yes	yes	8
Lazzeri, et al[986]	2014	no	yes	yes	yes	yes	yes	yes	yes	yes	8
Bo, et al[987]	2014	yes	no	yes	yes	yes	yes	yes	no	yes	7
Sacchetti, et al[988]	2012	yes	yes	no	yes	yes	no	yes	yes	yes	7
Bracale, et al[989]	2013	yes	yes	yes	yes	yes	yes	yes	yes	no	8
Petracci, et al[990]	2013	no	yes	yes	yes	yes	yes	yes	yes	no	7
Pileggi, et al[991]	2013	yes	yes	yes	yes	yes	yes	yes	yes	no	8
Vijaykumar, et al[992]	2013	yes	yes	yes	no	yes	yes	yes	yes	yes	8
Galfo, et al[993]	2016	yes	yes	yes	yes	yes	yes	no	yes	yes	8
Grassi, et al[994]	2016	yes	yes	yes	yes	yes	yes	yes	yes	no	8
Fox, et al[995]	2009	yes	yes	yes	no	no	no	yes	yes	yes	6
Sung, et al[996]	2009	yes	yes	yes	yes	yes	yes	no	yes	no	7
Tsujiguchi, et al[997]	2018	yes	yes	yes	yes	yes	no	yes	yes	yes	8
Yaguchi-Tanaka, et al[998]	2021	yes	yes	yes	yes	yes	yes	yes	no	no	7
Tani, et al[999]	2021	yes	yes	yes	yes	yes	no	yes	yes	yes	8
Watanabe, et al[1000]	2011	yes	yes	yes	no	yes	yes	yes	yes	yes	8
Yoshitake, et al[1001]	2012	yes	yes	no	yes	yes	yes	yes	yes	yes	8
Oishi, et al[1002]	2021	yes	yes	no	yes	yes	yes	yes	no	yes	7
Kachi, et al[1003]	2015	yes	yes	yes	yes	yes	yes	yes	no	yes	8
Nakano, et al[1004]	2010	yes	yes	yes	yes	yes	no	no	yes	yes	7
Wang, et al[1005]	2007	yes	yes	yes	yes	yes	yes	yes	no	yes	8
Okabe, et al[1006]	2011	yes	yes	yes	yes	yes	yes	no	yes	yes	8
Suzuki, et al[1007]	2009	yes	no	yes	yes	yes	yes	yes	yes	yes	8
Yamashita, et al[1008]	2023	yes	yes	yes	yes	yes	yes	no	yes	yes	8
Shirasawa,	2018	no	yes	yes	yes	yes	yes	yes	yes	yes	8

© 2024 Zhang X et al. *JAMA Pediatrics.*

et al[1009]											
Tomata, et al[1010]	2019	yes	yes	yes	yes	yes	yes	yes	no	yes	8
Wada, et al[1011]	2019	yes	yes	yes	yes	yes	no	yes	yes	yes	8
Ochiai, et al[1012]	2020	yes	yes	yes	yes	yes	yes	no	yes	yes	8
Sasaki, et al[1013]	2010	yes	yes	yes	yes	yes	yes	yes	yes	yes	9
Okuda, et al[1014]	2010	no	yes	yes	yes	yes	yes	yes	yes	yes	8
Ochiai, et al[1015]	2010	yes	yes	yes	yes	yes	yes	yes	no	yes	8
Shirasawa, et al[1016]	2015	yes	yes	yes	yes	yes	yes	yes	yes	no	8
Shinozaki, et al[1017]	2015	yes	yes	yes	yes	yes	no	yes	yes	yes	8
Ochiai, et al[1018]	2015	yes	yes	no	yes	yes	yes	yes	yes	yes	8
Murakami, et al[1019]	2011	yes	yes	yes	yes	yes	yes	no	yes	yes	8
Itoi, et al[1020]	2012	no	yes	yes	yes	yes	yes	yes	yes	yes	8
Mizuta, et al[1021]	2016	no	yes	yes	no	yes	yes	yes	yes	yes	7
Ochiai, et al[1022]	2018	yes	yes	yes	no	yes	yes	no	yes	yes	7
Baker, et al[1023]	2010	yes	yes	yes	yes	yes	yes	yes	yes	no	8
Hamaideh, et al[1024]	2010	no	yes	yes	yes	yes	yes	yes	yes	yes	8
Ibrahim, et al[1025]	2008	yes	yes	yes	no	yes	yes	yes	yes	yes	8
Khader, et al[1026]	2009	yes	yes	yes	yes	yes	yes	yes	no	yes	8
Al-Akour, et al[1027]	2012	yes	yes	yes	no	yes	yes	yes	yes	yes	8
Al-Domi, et al[1028]	2019	yes	yes	yes	no	yes	no	yes	yes	yes	7
Al-Kloub, et al[1029]	2010	yes	yes	yes	yes	yes	yes	yes	yes	yes	9
Tayyem, et al[1030]	2014	yes	yes	yes	yes	yes	yes	yes	yes	yes	9
Musaiger, et al[1031]	2013	yes	yes	yes	yes	yes	yes	yes	yes	no	8
Zayed, et al[1032]	2016	yes	yes	yes	yes	no	yes	yes	yes	yes	8

© 2024 Zhang X et al. *JAMA Pediatrics.*

Facchini, et al[1033]	2007	yes	no	yes	yes	yes	yes	yes	no	yes	7
Tiruneh, er al[1034]	2021	yes	yes	yes	yes	yes	yes	yes	yes	yes	9
Adamo, et al[1035]	2011	yes	no	yes	yes	yes	yes	no	yes	yes	7
Kyallo, et al[1036]	2013	yes	yes	yes	yes	yes	yes	yes	yes	yes	9
Muthuri, et al[1037]	2014	no	yes	yes	yes	yes	yes	no	yes	yes	7
Gewa, et al[1038]	2010	yes	yes	yes	yes	yes	yes	yes	yes	no	8
Abdelalim, et al[1039]	2012	yes	yes	yes	yes	no	yes	yes	yes	yes	8
Alqaoud, et al[1040]	2022	yes	yes	yes	yes	yes	no	yes	yes	no	7
Al-Haifi, et al[1041]	2022	yes	yes	yes	yes	no	yes	no	yes	yes	7
Badr, et al[1042]	2017	no	yes	yes	yes	yes	yes	yes	yes	yes	8
El-Ghaziri, et al[1043]	2011	yes	yes	yes	yes	yes	yes	no	yes	yes	8
El-Bayoumy, et al[1044]	2009	yes	yes	yes	yes	yes	yes	no	yes	no	7
AlRodhan, et al[1045]	2019	yes	yes	yes	yes	yes	yes	yes	yes	no	8
Al-Qaoud, et al[1046]	2009	yes	yes	yes	yes	yes	yes	yes	yes	yes	9
Al-Refaee, et al[1047]	2013	yes	yes	yes	yes	yes	yes	yes	yes	no	8
Musaiger, et al[1048]	2013	yes	yes	yes	yes	yes	yes	yes	no	yes	8
Karklina, et al[1049]	2011	yes	yes	no	yes	yes	yes	yes	yes	yes	8
Nasreddine, et al[1050]	2014	yes	yes	no	yes	yes	yes	yes	yes	yes	8
Chacar, et al[1051]	2011	yes	yes	yes	yes	yes	no	yes	yes	no	7
Jabre, et al[1052]	2005	yes	yes	yes	yes	yes	no	yes	yes	yes	8
Fazah, et al[1053]	2010	yes	yes	yes	yes	yes	yes	yes	yes	yes	9
Chakar, et al[1054]	2006	yes	yes	yes	no	yes	yes	yes	yes	yes	8

© 2024 Zhang X et al. *JAMA Pediatrics.*

Nasreddine, et al[1055]	2017	yes	yes	yes	yes	yes	no	yes	yes	yes	8
Smetanina, et al[1056]	2015	yes	yes	no	yes	yes	yes	yes	yes	yes	8
Grazulevicienе, et al[1057]	2014	yes	yes	yes	yes	yes	yes	no	yes	yes	8
Raufi, et al[1058]	2022	yes	yes	yes	yes	yes	yes	yes	yes	yes	9
Gontarev, et al[1059]	2018	yes	yes	yes	yes	yes	yes	yes	yes	no	8
Myrtaj, et al[1060]	2018	yes	yes	yes	yes	yes	yes	yes	yes	yes	9
Mohamed, et al[1061]	2023	yes	yes	yes	yes	yes	yes	no	yes	yes	8
Lai, et al[1062]	2022	yes	yes	yes	yes	yes	yes	no	yes	yes	8
Al-Sadat, et al[1063]	2016	yes	yes	yes	no	yes	yes	yes	yes	yes	8
Khor, et al[1064]	2011	yes	yes	yes	yes	yes	no	yes	yes	yes	8
Adeyemi, et al[1065]	2014	yes	yes	yes	yes	yes	no	no	yes	yes	7
Ahmad, et al[1066]	2017	yes	yes	yes	yes	yes	yes	yes	yes	yes	9
Tee, et al[1067]	2018	yes	yes	yes	yes	no	yes	yes	yes	yes	8
Kee, et al[1068]	2017	no	yes	yes	yes	yes	yes	yes	yes	yes	8
Cheong, et al[1069]	2019	yes	yes	yes	yes	yes	yes	no	yes	yes	8
Moy, et al[1070]	2004	yes	yes	yes	yes	no	yes	no	yes	yes	7
Mahaletchumy, et al[1071]	2019	yes	yes	yes	yes	yes	no	yes	yes	yes	8
Cheah, et al[1072]	2019	yes	yes	yes	yes	yes	no	no	yes	yes	7
Tan, et al[1073]	2019	yes	yes	yes	yes	yes	yes	yes	yes	yes	9
Muhammad, et al[1074]	2008	yes	no	yes	yes	yes	yes	yes	yes	yes	8
Zalilah, et al[1075]	2006	yes	yes	yes	yes	yes	yes	no	yes	yes	8
Mohd, et al[1076]	2006	yes	yes	yes	yes	yes	yes	yes	yes	no	8
Woon, et	2015	no	yes	yes	yes	yes	yes	yes	yes	yes	8

© 2024 Zhang X et al. *JAMA Pediatrics.*

al[1077]											
Loh, et al[1078]	2015	yes	yes	yes	yes	no	yes	yes	yes	yes	8
Kaartina, et al[1079]	2015	yes	yes	yes	yes	yes	yes	yes	no	yes	8
Zainuddin, et al[1080]	2014	yes	yes	yes	yes	yes	no	yes	yes	yes	8
Su, et al[1081]	2014	yes	yes	yes	yes	yes	no	yes	yes	yes	8
Rezali, et al[1082]	2012	no	yes	yes	yes	yes	yes	no	yes	no	6
Naidu, et al[1083]	2013	yes	yes	yes	yes	yes	yes	yes	yes	yes	9
Poh, et al[1084]	2016	yes	yes	yes	yes	yes	yes	yes	no	yes	8
Shariff, et al[1085]	2016	yes	yes	yes	yes	yes	yes	yes	no	yes	8
Partap, et al[1086]	2017	yes	yes	yes	yes	yes	yes	yes	no	yes	8
Cazzaniga, et al[1087]	2022	yes	no	yes	yes	yes	yes	yes	yes	yes	8
Decelis, et al[1088]	2014	yes	yes	yes	yes	yes	yes	yes	yes	yes	9
Decelis, et al[1089]	2013	yes	yes	no	yes	yes	no	yes	yes	yes	7
Caleyachetty, et al[1090]	2012	yes	yes	yes	yes	yes	no	yes	yes	yes	8
Fokeena, et al[1091]	2012	yes	yes	yes	yes	yes	yes	yes	no	yes	8
Shamah-Levy, et al[1092]	2022	yes	yes	yes	yes	yes	yes	yes	yes	yes	9
Brambila-Paz, et al[1093]	2022	yes	yes	yes	yes	yes	yes	no	yes	yes	8
Aburto, et al[1094]	2015	yes	yes	yes	no	yes	no	no	yes	yes	6
Aguilera-Galaviz, et al[1095]	2019	yes	yes	yes	yes	yes	yes	yes	yes	yes	9
Pérez, et al[1096]	2020	yes	yes	yes	yes	yes	yes	yes	no	yes	8
Irigoyen-Camacho, et al[1097]	2014	yes	yes	yes	yes	yes	yes	yes	yes	yes	9
Lara-Capi, et al[1098]	2018	yes	yes	yes	yes	no	no	yes	yes	yes	7

© 2024 Zhang X et al. *JAMA Pediatrics.*

Patiño-Marín, et al[1099]	2018	yes	yes	yes	yes	yes	yes	yes	yes	yes	9
Sánchez-Pérez, et al[1100]	2010	yes	yes	yes	yes	yes	yes	no	yes	yes	8
Serrano-Piña, et al[1101]	2019	yes	yes	yes	no	yes	yes	yes	yes	yes	8
Vázquez-Nava, et al[1102]	2010	yes	yes	yes	yes	no	yes	no	yes	yes	7
Ávila-Ortiz, et al[1103]	2017	yes	yes	yes	yes	yes	yes	yes	yes	yes	9
Bacardi-Gascón, et al[1104]	2009	yes	yes	yes	yes	yes	yes	yes	yes	yes	9
Bacardi-Gascón, et al[1105]	2012	yes	yes	yes	yes	yes	yes	yes	yes	yes	9
Lopez-Gonzalez, et al[1106]	2020	yes	yes	yes	no	yes	yes	yes	yes	yes	8
Del-Rio-Navarro, et al[1107]	2008	yes	yes	yes	yes	yes	no	no	yes	yes	7
Gómez-Díaz, et al[1108]	2005	yes	yes	yes	yes	no	yes	yes	yes	yes	8
Brewis, et al[1109]	2003	yes	yes	yes	yes	yes	yes	yes	no	yes	8
Vergara-Castañeda, et al[1110]	2010	yes	yes	no	yes	yes	yes	yes	yes	yes	8
Jimenez-Cruz, et al[1111]	2010	yes	yes	yes	yes	yes	no	no	yes	yes	7
Varela-Silva, et al[1112]	2009	yes	yes	yes	yes	yes	yes	yes	no	yes	8
Macías-Rosales, et al[1113]	2009	yes	yes	yes	yes	yes	yes	yes	yes	yes	9
Flores-Huerta	2009	yes	yes	yes	yes	yes	yes	yes	no	yes	8

© 2024 Zhang X et al. *JAMA Pediatrics.*

, et al[1114]											
Ortiz-Hernández, et al[1115]	2008	yes	yes	yes	yes	yes	yes	no	yes	yes	8
Basaldúa, et al[1116]	2008	no	yes	yes	yes	yes	yes	yes	yes	yes	8
Villa-Caballero, et al[1117]	2006	yes	yes	yes	yes	yes	no	no	yes	yes	7
RODRÍGUEZ-FUENTES, et al[1118]	2015	yes	yes	yes	yes	yes	no	yes	yes	yes	8
López-Barrón, et al[1119]	2015	yes	yes	yes	yes	yes	no	yes	yes	yes	8
Flores, et al[1120]	2015	yes	yes	yes	yes	yes	yes	yes	yes	yes	9
Banik, et al[1121]	2014	yes	yes	yes	yes	yes	yes	yes	yes	yes	9
Banik, et al[1122]	2014	yes	yes	yes	yes	no	yes	yes	yes	yes	8
Barrera, et al[1123]	2016	yes	yes	yes	yes	no	yes	yes	yes	yes	8
Caraza, et al[1124]	2016	yes	yes	yes	yes	yes	yes	yes	no	yes	8
Peña, et al[1125]	2018	yes	yes	yes	yes	yes	yes	yes	no	yes	8
Ramírez-Jiménez ,et al[1126]	2018	yes	yes	yes	yes	yes	no	yes	yes	yes	8
Banjari, et al[1127]	2020	yes	yes	no	yes	yes	yes	no	yes	yes	7
Martinovic, et al[1128]	2015	yes	yes	yes	yes	yes	yes	yes	no	yes	8
Mehdad, et al[1129]	2022	yes	yes	yes	yes	yes	yes	yes	no	yes	8
El Moussaoui, et al[1130]	2022	no	yes	yes	yes	yes	yes	no	yes	yes	7
Achouri, et al[1131]	2021	yes	yes	yes	yes	no	yes	yes	yes	yes	8
Dekkaki, et al[1132]	2011	yes	yes	no	yes	yes	yes	yes	yes	yes	8
El Kabbaoui, et al[1133]	2018	yes	yes	yes	yes	yes	yes	yes	yes	yes	9

© 2024 Zhang X et al. *JAMA Pediatrics.*

Qiao, et al[1134]	2015	no	yes	no	yes	yes	yes	no	yes	yes	6
Bishwajt, et al[1135]	2019	yes	yes	yes	no	yes	yes	yes	yes	yes	8
Stival, et al[1136]	2022	yes	no	yes	yes	yes	yes	yes	yes	yes	8
Moschonis, et al[1137]	2022	yes	yes	yes	yes	yes	yes	no	yes	yes	8
Ferrari, et al[1138]	2022	yes	yes	yes	yes	no	yes	yes	yes	yes	8
Collings, et al[1139]	2022	yes	no	yes	yes	yes	yes	no	yes	yes	7
Ho, et al[1140]	2021	yes	yes	yes	yes	no	yes	yes	yes	no	7
Peltzer, et al[1141]	2011	yes	yes	yes	yes	yes	yes	yes	no	yes	8
Vrijheid, et al[1142]	2020	yes	yes	yes	yes	yes	no	yes	yes	yes	8
Roy, et al[1143]	2020	yes	yes	yes	yes	yes	yes	yes	no	yes	8
Ekelund, et al[1144]	2004	yes	yes	yes	yes	yes	yes	no	no	yes	7
McCabe, et al[1145]	2009	yes	yes	yes	yes	yes	yes	yes	yes	yes	9
Velde, et al[1146]	2007	yes	yes	yes	yes	yes	yes	yes	yes	yes	9
Blaženčić-Mladenović, et al[1147]	2006	yes	yes	yes	yes	yes	yes	yes	no	yes	8
Olaya, et al[1148]	2015	yes	yes	yes	yes	yes	yes	yes	yes	yes	9
Katzmarzyk, et al[1149]	2015	yes	yes	yes	yes	yes	no	no	yes	yes	7
Tsitsika, et al[1150]	2016	yes	yes	yes	yes	yes	yes	no	yes	yes	8
Qiao, et al[1151]	2017	yes	yes	yes	yes	yes	yes	yes	no	yes	8
Achaya, et al[1152]	2016	yes	yes	yes	yes	yes	yes	no	yes	yes	8
Piryani, et al[1153]	2016	yes	yes	yes	no	yes	yes	yes	yes	yes	8
Bhattarai, et al[1154]	2019	yes	yes	yes	yes	yes	yes	no	no	yes	7
Karki, et al[1155]	2019	yes	yes	yes	yes	yes	yes	yes	no	yes	8
Koirala, et al[1156]	2015	yes	yes	yes	yes	yes	yes	yes	no	yes	8

© 2024 Zhang X et al. *JAMA Pediatrics.*

Gurung, et al[1157]	2014	yes	yes	yes	no	yes	yes	yes	yes	yes	8
Pandey, et al[1158]	2018	yes	yes	yes	yes	yes	yes	yes	yes	yes	9
Greeff, et al[1159]	2016	yes	yes	yes	no	yes	yes	no	yes	yes	7
Veldhuis, et al[1160]	2012	yes	yes	yes	yes	yes	yes	yes	yes	yes	9
Jabakhanji, et al[1161]	2022	yes	yes	yes	yes	yes	yes	no	yes	yes	8
Vinke, et al[1162]	2021	yes	yes	yes	yes	yes	yes	no	yes	yes	8
Croezen, et al[1163]	2009	yes	yes	yes	yes	yes	yes	no	yes	yes	8
Drukker, et al[1164]	2009	yes	yes	yes	yes	yes	yes	yes	yes	yes	9
Jansen, et al[1165]	2012	yes	yes	yes	no	yes	yes	yes	no	yes	7
Wilde, et al[1166]	2009	yes	yes	yes	yes	yes	yes	yes	yes	yes	9
Jong, et al[1167]	2013	yes	yes	yes	yes	yes	yes	no	yes	yes	8
Jansen, et al[1168]	2006	yes	yes	yes	yes	yes	yes	no	yes	yes	8
Steur, et al[1169]	2011	yes	yes	yes	yes	yes	no	yes	yes	yes	8
Mbakwa, et al[1170]	2016	yes	no	yes	yes	yes	yes	yes	yes	yes	8
Jansen, et al[1171]	2008	no	yes	yes	yes	yes	yes	yes	yes	yes	8
Scholtens, et al[1172]	2009	yes	yes	yes	yes	yes	no	yes	yes	yes	8
Timmermans, et al[1173]	2014	no	yes	yes	yes	yes	yes	yes	yes	yes	8
Vrijkotte, et al[1174]	2020	yes	no	yes	yes	yes	no	yes	yes	yes	7
Wilde, et al[1175]	2019	yes	yes	yes	yes	yes	yes	no	yes	yes	8
Luttikhuis, et al[1176]	2010	no	yes	yes	yes	yes	yes	yes	yes	yes	8
Jansen, et al[1177]	2010	yes	yes	yes	yes	yes	yes	yes	no	yes	8
Bossink-Tuna, et al[1178]	2009	yes	yes	yes	yes	yes	yes	yes	no	yes	8
Snoek, et al[1179]	2007	yes	yes	yes	yes	yes	no	no	yes	yes	7

© 2024 Zhang X et al. *JAMA Pediatrics*.

Scholtens, et al[1180]	2007	yes	yes	yes	no	yes	yes	yes	yes	yes	8
Jansen, et al[1181]	2006	yes	yes	yes	yes	yes	yes	no	yes	yes	8
Wijtzes, et al[1182]	2014	yes	yes	yes	yes	yes	yes	no	yes	yes	8
Bere, et al[1183]	2011	yes	yes	yes	yes	yes	yes	no	yes	no	7
Schwiebbe, et al[1184]	2011	yes	yes	yes	yes	yes	yes	no	yes	yes	8
de Jong, et al[1185]	2012	no	yes	yes	yes	yes	yes	yes	yes	yes	8
Veldwijk, et al[1186]	2012	yes	yes	yes	yes	yes	yes	yes	yes	no	8
Willeboordse, et al[1187]	2013	yes	yes	yes	yes	yes	yes	yes	yes	no	8
Leppers, et al [1188]	2017	yes	yes	yes	yes	yes	yes	yes	no	yes	8
Ridder, et al[1189]	2018	yes	yes	yes	yes	yes	yes	yes	no	yes	8
Utter, et al[1190]	2006	yes	yes	yes	yes	yes	yes	yes	yes	no	8
Hobbs, et al[1191]	2021	yes	yes	yes	yes	yes	yes	yes	no	yes	8
Aung, et al[1192]	2021	yes	yes	yes	yes	yes	yes	yes	yes	yes	9
Chelimo, et al[1193]	2020	yes	yes	yes	yes	yes	yes	yes	yes	no	8
Rockell, et al[1194]	2005	yes	yes	no	yes	yes	yes	no	yes	yes	7
Gordon, et al[1195]	2003	yes	yes	yes	yes	yes	yes	no	yes	yes	8
Utter, et al[1196]	2010	yes	yes	yes	yes	yes	yes	yes	yes	no	8
Quigg, et al[1197]	2010	yes	yes	yes	yes	no	yes	yes	no	yes	7
Rajput, et al[1198]	2014	yes	yes	yes	yes	yes	no	yes	yes	yes	8
Adeomi, et al[1199]	2019	no	yes	yes	yes	yes	yes	yes	yes	yes	8
Adetunji, et al[1200]	2019	yes	yes	yes	yes	yes	yes	yes	no	yes	8
Maruf, et al[1201]	2013	yes	yes	yes	yes	yes	yes	yes	no	yes	8
Ene-obong, et al[1202]	2012	yes	yes	yes	yes	yes	no	yes	yes	yes	8

© 2024 Zhang X et al. *JAMA Pediatrics*.

Omigbodun, et al[1203]	2010	yes	yes	yes	yes	yes	yes	yes	no	yes	8
Opara, et al[1204]	2010	yes	yes	yes	yes	yes	yes	no	yes	yes	8
Senbanjo, et al[1205]	2010	yes	yes	yes	yes	yes	yes	no	no	yes	7
Goon, et al[1206]	2010	yes	yes	yes	no	yes	yes	yes	yes	yes	8
Fetuga, et al[1207]	2011	yes	yes	yes	yes	yes	no	yes	yes	yes	8
Wariri, et al[1208]	2020	yes	yes	yes	yes	yes	yes	yes	no	yes	8
Mezie-Okoye, et al[1209]	2015	yes	yes	yes	yes	yes	yes	yes	yes	no	8
Nwizu, et al[1210]	2011	no	yes	yes	yes	yes	yes	yes	yes	no	7
Musa, et al[1211]	2012	yes	yes	yes	yes	yes	yes	no	yes	yes	8
Oduwole, et al[1212]	2012	yes	yes	yes	yes	yes	yes	yes	no	yes	8
Sadoh, et al[1213]	2016	no	yes	yes	yes	yes	yes	yes	yes	yes	8
Igbokwe, et al[1214]	2017	yes	yes	no	yes	yes	yes	yes	yes	yes	8
Aadland, et al[1215]	2021	yes	yes	yes	yes	no	yes	no	yes	yes	7
Aadland, et al[1216]	2022	no	yes	yes	yes	yes	yes	no	yes	yes	7
Donkor, et al[1217]	2017	yes	yes	yes	yes	yes	yes	yes	no	yes	8
Øvrebø, et al[1218]	2021	yes	yes	yes	yes	yes	yes	no	yes	yes	8
Andersen, et al[1219]	2005	yes	yes	yes	yes	yes	no	yes	no	yes	7
Grydeland, et al[1220]	2014	yes	no	yes	yes	yes	yes	yes	no	yes	7
Bernhardsen, et al[1221]	2019	yes	yes	yes	yes	yes	yes	no	yes	yes	8
Oellingrath, et al[1222]	2010	yes	yes	yes	yes	no	yes	yes	yes	yes	8
Júlíusson, et al[1223]	2010	no	yes	yes	yes	yes	no	yes	yes	yes	7
Kolle, et al[1224]	2009	yes	yes	yes	yes	no	yes	yes	no	yes	7
Gebremariam, et al[1225]	2015	yes	yes	yes	yes	no	yes	yes	yes	yes	8

© 2024 Zhang X et al. *JAMA Pediatrics.*

Fasting, et al[1226]	2009	yes	yes	yes	yes	yes	yes	yes	no	yes	8
Hestetun, et al[1227]	2015	yes	yes	yes	yes	yes	yes	yes	yes	yes	9
Vik, et al[1228]	2010	yes	yes	no	yes	yes	yes	yes	yes	yes	8
Bjelland, et al[1229]	2010	yes	yes	yes	no	yes	yes	yes	yes	yes	8
Grøholt, et al[1230]	2008	yes	yes	yes	yes	yes	yes	yes	yes	no	8
Brannsether, et al[1231]	2014	yes	yes	yes	yes	yes	yes	no	yes	no	7
Tanveer, et al[1232]	2022	yes	yes	yes	yes	yes	yes	no	yes	yes	8
Bekhwani, et al[1233]	2022	yes	yes	yes	yes	yes	yes	no	yes	yes	8
Ahmed, et al[1234]	2013	yes	yes	yes	yes	no	yes	yes	yes	yes	8
Mansoori, et al[1235]	2018	yes	yes	yes	no	yes	yes	yes	yes	yes	8
Anwar, et al[1236]	2010	yes	yes	yes	yes	yes	yes	yes	yes	no	8
Hashmi, et al[1237]	2013	yes	yes	yes	no	yes	no	yes	yes	yes	7
Jafar, et al[1238]	2008	yes	yes	yes	yes	yes	yes	yes	yes	yes	9
Ramzan, et al[1239]	2008	yes	yes	yes	yes	yes	yes	yes	yes	yes	9
Aziz, et al[1240]	2009	yes	yes	yes	yes	yes	no	no	yes	yes	7
Haq, et al[1241]	2010	yes	yes	yes	yes	yes	no	yes	yes	yes	8
Aziz, et al[1242]	2012	yes	yes	yes	yes	yes	yes	yes	yes	yes	9
Hydrie, et al[1243]	2005	yes	yes	yes	yes	yes	yes	yes	yes	no	8
Basit,et al[1244]	2005	no	yes	yes	no	yes	yes	yes	no	yes	6
Ishaque, et al[1245]	2012	yes	yes	yes	yes	yes	yes	yes	no	yes	8
Salah, et al[1246]	2021	yes	yes	yes	yes	no	yes	yes	yes	yes	8
Abudayya, et al[1247]	2007	yes	yes	yes	yes	yes	no	yes	no	yes	7
AbuKishk, et al[1248]	2021	no	yes	yes	yes	yes	yes	yes	yes	yes	8
Badrasawi, et al[1249]	2019	yes	yes	yes	no	yes	yes	yes	yes	yes	8
Al-Lahha	2019	no	yes	yes	yes	yes	yes	yes	yes	yes	8

© 2024 Zhang X et al. *JAMA Pediatrics*.

m, et al[1250]											
Mikki, et al[1251]	2009	yes	yes	yes	yes	yes	yes	yes	no	yes	8
Al Sabbah, et al[1252]	2009	yes	yes	yes	yes	yes	yes	yes	yes	yes	9
Massad, et al[1253]	2016	yes	yes	yes	yes	yes	yes	yes	yes	yes	9
Bartkowiak, et al[1254]	2021	yes	yes	yes	yes	yes	no	yes	yes	yes	8
Carrillo-Larco, et al[1255]	2014	yes	yes	yes	yes	yes	yes	no	yes	yes	8
Echevarría-Castro ,et al[1256]	2020	no	yes	yes	yes	yes	yes	yes	no	yes	7
Preston, et al[1257]	2015	yes	yes	yes	yes	no	yes	yes	yes	yes	8
Hernández-Vásquez, et al[1258]	2016	yes	yes	no	yes	yes	yes	yes	yes	yes	8
Suligowska, et al[1259]	2022	yes	yes	yes	yes	yes	yes	yes	yes	no	8
Potempa-Jeziorowska, et al[1260]	2022	yes	yes	yes	yes	yes	yes	yes	yes	yes	9
Kryst, et al[1261]	2022	yes	yes	yes	yes	yes	no	yes	yes	yes	8
Bryl, et al[1262]	2022	yes	yes	no	yes	yes	no	yes	yes	yes	7
Basiak-Rasała, et al[1263]	2022	yes	yes	yes	yes	yes	yes	yes	yes	yes	9
Wyszyńska, et al[1264]	2021	yes	yes	yes	yes	yes	yes	yes	yes	yes	9
Wieniawski, et al[1265]	2021	yes	yes	yes	no	yes	yes	yes	yes	no	7
Matłosz, et al[1266]	2021	yes	yes	yes	yes	yes	yes	yes	no	yes	8
Żegleń, et al[1267]	2020	yes	yes	yes	yes	yes	yes	yes	yes	yes	9
Szczyrska, et al[1268]	2020	yes	yes	yes	yes	yes	no	yes	yes	yes	8
Tomaszewski	2015	yes	no	yes	yes	yes	yes	yes	no	yes	7

© 2024 Zhang X et al. *JAMA Pediatrics.*

, et al[1269]											
Bac, et al[1270]	2012	yes	yes	no	yes	yes	yes	yes	yes	yes	8
Baran, et al[1271]	2022	yes	yes	yes	yes	yes	yes	yes	yes	yes	9
Kułaga, et al[1272]	2011	yes	yes	yes	no	no	yes	yes	yes	yes	7
Malecka-Tendera, et al[1273]	2005	yes	yes	yes	yes	yes	yes	yes	yes	yes	9
Kowal, et al[1274]	2014	yes	yes	yes	yes	yes	yes	no	no	yes	7
Chrzanowska, et al[1275]	2007	yes	yes	yes	yes	yes	yes	yes	yes	yes	9
Ządzińska, et al[1276]	2013	yes	yes	yes	yes	yes	no	yes	yes	yes	8
Zatoński, et al[1277]	2020	yes	yes	yes	no	yes	yes	yes	yes	yes	8
Woźniacka, et al[1278]	2018	yes	yes	yes	yes	no	yes	no	yes	yes	7
Baran, et al[1279]	2019	yes	yes	yes	yes	yes	yes	no	yes	yes	8
Jodkowska, et al[1280]	2010	yes	yes	yes	yes	yes	yes	yes	yes	yes	9
Zawodniak-Szałapska, et al[1281]	2007	yes	yes	yes	yes	yes	yes	no	yes	yes	8
Klimek-Piotrowska, et al[1282]	2015	yes	yes	yes	yes	yes	no	yes	yes	yes	8
Długosz, et al[1283]	2015	yes	no	yes	yes	yes	yes	yes	yes	yes	8
Stankiewicz, et al[1284]	2014	yes	yes	yes	no	yes	yes	yes	yes	yes	8
Kowalkowska, et al[1285]	2014	yes	no	yes	yes	no	yes	yes	yes	yes	7
Januszek-Trzciąkowska, et al[1286]	2014	yes	yes	yes	yes	yes	yes	yes	yes	yes	9
Golec, et al[1287]	2014	no	yes	yes	yes	no	yes	yes	yes	yes	7
Rutkowski, et	2013	yes	no	yes	yes	yes	yes	yes	yes	yes	8

© 2024 Zhang X et al. *JAMA Pediatrics.*

al[1288]											
Czyż, et al[1289]	2017	yes	yes	yes	yes	yes	yes	yes	no	yes	8
Kantanista, et al[1290]	2017	no	yes	yes	yes	yes	yes	yes	yes	yes	8
Rodrigues, et al[1291]	2022	yes	yes	yes	yes	yes	yes	yes	no	yes	8
Paciência, et al[1292]	2021	yes	yes	yes	yes	yes	yes	yes	no	yes	8
Nazareth, et al[1293]	2021	yes	yes	yes	yes	yes	yes	no	no	yes	7
Abreu, et al[1294]	2019	yes	yes	yes	yes	yes	yes	no	yes	yes	8
Abreu, et al[1295]	2014	yes	yes	yes	yes	yes	yes	yes	no	yes	8
Frias-Bulhosa, et al[1296]	2015	yes	yes	yes	yes	yes	no	yes	yes	yes	8
Abreu, et al[1297]	2014	yes	yes	yes	yes	yes	no	yes	yes	yes	8
Padez, et al[1298]	2005	yes	yes	yes	yes	yes	yes	no	yes	yes	8
Araújo, et al[1299]	2012	yes	yes	yes	yes	yes	no	yes	yes	yes	8
Abreu, et al[1300]	2012	yes	yes	yes	no	yes	yes	yes	yes	yes	8
Silva-Santos, et al[1301]	2017	yes	yes	yes	yes	yes	yes	yes	no	yes	8
Sardinha, et al[1302]	2011	yes	yes	yes	yes	yes	no	yes	no	yes	7
Seabra, et al[1303]	2013	yes	yes	yes	yes	yes	no	yes	yes	yes	8
Rito, et al[1304]	2012	yes	yes	yes	no	yes	yes	yes	no	yes	7
Antunes, et al[1305]	2015	yes	yes	yes	yes	yes	yes	no	yes	yes	8
Ferreira, et al[1306]	2008	no	yes	yes	yes	yes	yes	yes	yes	yes	8
Marques-Vidal, et al[1307]	2008	yes	yes	yes	yes	yes	yes	yes	yes	yes	9
Cabral, et al[1308]	2016	yes	yes	yes	no	yes	yes	yes	yes	yes	8
Ribeiro, et al[1309]	2020	yes	yes	yes	yes	yes	yes	yes	no	yes	8
Mota, et al[1310]	2008	yes	yes	yes	no	yes	yes	yes	yes	yes	8

© 2024 Zhang X et al. *JAMA Pediatrics.*

Minghelli, et al[1311]	2014	yes	yes	yes	yes	yes	yes	no	yes	yes	8
Albuquerque, et al[1312]	2012	yes	no	yes	yes	yes	yes	yes	yes	yes	8
Padez, et al[1313]	2004	yes	yes	yes	yes	no	yes	yes	yes	yes	8
Vale, et al[1314]	2010	yes	yes	yes	yes	yes	yes	yes	yes	no	8
Gama, et al[1315]	2020	yes	no	yes	yes	yes	yes	yes	yes	yes	8
Pereira, et al[1316]	2010	yes	yes	yes	no	yes	yes	yes	yes	yes	8
Padez, et al[1317]	2009	no	yes	no	yes	yes	yes	yes	yes	yes	7
Fonseca, et al[1318]	2009	yes	yes	yes	yes	yes	yes	yes	yes	yes	9
Rebelo, et al[1319]	2008	yes	yes	yes	yes	yes	yes	no	yes	yes	8
Aires, et al[1320]	2008	yes	yes	yes	yes	yes	yes	no	yes	yes	8
Moreira, et al[1321]	2007	yes	yes	yes	yes	no	yes	yes	yes	yes	8
Ribeiro, et al[1322]	2006	yes	no	no	yes	yes	yes	yes	yes	yes	7
Mota, et al[1323]	2006	yes	yes	yes	no	yes	yes	yes	yes	yes	8
Vale, et al[1324]	2015	yes	yes	yes	yes	yes	yes	yes	no	yes	8
Minghelli, et al[1325]	2015	yes	yes	no	yes	yes	yes	yes	yes	yes	8
Pedrosa, et al[1326]	2011	yes	yes	yes	yes	yes	no	yes	yes	yes	8
Valente, et al[1327]	2011	yes	yes	yes	yes	yes	yes	yes	yes	no	8
Vasques, et al[1328]	2012	yes	yes	yes	yes	yes	yes	yes	no	yes	8
Nogueira, et al[1329]	2013	yes	yes	yes	yes	yes	yes	yes	yes	yes	9
Marques, et al[1330]	2016	yes	yes	yes	yes	no	yes	yes	no	yes	7
Marques, et al[1331]	2018	yes	yes	yes	yes	yes	yes	yes	yes	yes	9
Santiago, et al[1332]	2021	yes	yes	yes	yes	yes	yes	yes	yes	yes	9
Rivera-Soto, et al[1333]	2010	yes	yes	yes	yes	yes	yes	no	yes	yes	8
Rodriguez, et al[1334]	2008	yes	yes	yes	yes	yes	yes	no	no	yes	7

© 2024 Zhang X et al. *JAMA Pediatrics.*

Elías-Boneta, et al[1335]	2015	no	yes	yes	yes	yes	yes	yes	yes	yes	8
Torres, et al[1336]	2014	yes	yes	yes	yes	yes	yes	yes	yes	no	8
Cheema, et al[1337]	2022	yes	yes	yes	yes	yes	no	yes	yes	yes	8
Kerkadi, et al[1338]	2019	yes	yes	yes	yes	yes	yes	yes	yes	no	8
Bener, et al[1339]	2005	yes	yes	yes	yes	yes	no	yes	no	yes	7
Bener, et al[1340]	2006	yes	yes	yes	yes	yes	yes	yes	yes	yes	9
Passmore, et al[1341]	2019	yes	yes	yes	yes	yes	yes	no	yes	yes	8
Barbu, et al[1342]	2015	no	yes	yes	yes	yes	yes	yes	yes	yes	8
Pop, et al[1343]	2021	yes	no	yes	yes	yes	yes	yes	yes	yes	8
Valean, et al[1344]	2009	yes	yes	yes	yes	yes	no	yes	yes	yes	8
Mocanu, et al[1345]	2013	yes	yes	yes	no	yes	yes	yes	yes	no	7
Emandi, et al[1346]	2012	yes	yes	yes	yes	yes	yes	yes	yes	yes	9
Cinteza, et al[1347]	2013	yes	yes	yes	no	no	yes	yes	yes	yes	7
Khasnutdinova, et al[1348]	2010	yes	yes	yes	yes	yes	yes	no	yes	yes	8
Choy, et al[1349]	2022	yes	yes	yes	no	yes	yes	yes	yes	yes	8
Choy, et al[1350]	2017	yes	yes	yes	yes	no	yes	yes	yes	yes	8
Abdellatif, et al[1351]	2020	yes	yes	yes	no	yes	yes	yes	yes	yes	8
Mohamed, et al[1352]	2022	yes	yes	yes	yes	yes	yes	yes	no	yes	8
Abu El Qomsan, et al[1353]	2017	yes	yes	yes	yes	yes	yes	yes	yes	no	8
Alghamdi, et al[1354]	2017	yes	yes	yes	yes	yes	yes	no	yes	no	7
Bhayat, et al[1355]	2016	yes	yes	yes	yes	yes	yes	yes	no	yes	8
Farsi, et al[1356]	2017	yes	yes	yes	yes	yes	yes	no	yes	yes	8
Farsi, et al[1357]	2016	yes	yes	yes	yes	yes	yes	yes	no	no	7

© 2024 Zhang X et al. *JAMA Pediatrics.*

Quadri, et al[1358]	2017	yes	yes	yes	yes	yes	yes	no	yes	yes	8
Bahathig, et al[1359]	2021	yes	yes	yes	yes	yes	yes	yes	no	yes	8
Amin, et al[1360]	2008	yes	yes	yes	yes	yes	yes	yes	no	yes	8
Bajamal, et al[1361]	2017	yes	yes	yes	yes	yes	yes	yes	yes	yes	9
Bawazeer, et al[1362]	2009	yes	yes	yes	yes	yes	yes	yes	no	yes	8
Abalkhail, et al[1363]	2002	yes	yes	yes	yes	yes	yes	yes	yes	yes	9
Al-Attas, et al[1364]	2010	yes	yes	no	yes	yes	yes	yes	yes	yes	8
Alwan, et al[1365]	2013	yes	yes	yes	yes	yes	yes	no	yes	yes	8
Al-Almaie, et al[1366]	2005	no	yes	yes	yes	yes	yes	yes	yes	yes	8
Mustafa, et al[1367]	2021	yes	yes	no	yes	no	yes	yes	yes	yes	7
Al-Agha, et al[1368]	2016	yes	yes	yes	yes	no	no	yes	yes	yes	7
Al-Musharaf, et al[1369]	2012	yes	yes	yes	yes	yes	yes	yes	yes	yes	9
Alselaim, et al[1370]	2012	yes	yes	no	yes	yes	yes	no	yes	yes	7
Washi, et al[1371]	2010	yes	yes	yes	yes	yes	no	yes	yes	yes	8
Al-Hussaini, et al[1372]	2019	yes	yes	yes	yes	yes	yes	no	yes	yes	8
Fakeeh, et al[1373]	2019	yes	yes	yes	yes	yes	yes	yes	yes	yes	9
Mouzan, et al[1374]	2010	yes	yes	yes	yes	no	yes	yes	yes	yes	8
Al-Dossary, et al[1375]	2010	yes	yes	yes	yes	yes	yes	yes	no	yes	8
Al-Saeed, et al[1376]	2007	yes	yes	yes	yes	yes	no	yes	yes	yes	8
Al-Muhaimeed, et al[1377]	2015	yes	yes	yes	no	yes	yes	yes	yes	yes	8
Akinpelu, et al[1378]	2014	yes	yes	yes	yes	yes	yes	yes	yes	yes	9
El Mouza	2012	yes	yes	yes	no	yes	no	yes	yes	yes	7

© 2024 Zhang X et al. *JAMA Pediatrics.*

n, et al[1379]											
Al Dahi, et al[1380]	2016	yes	yes	yes	yes	yes	yes	yes	yes	yes	9
Šušnjević, et al[1381]	2021	yes	yes	yes	yes	no	yes	yes	no	yes	7
Djordjic, et al[1382]	2016	yes	yes	yes	yes	yes	no	yes	yes	yes	8
Rakić, et al[1383]	2019	yes	yes	yes	no	yes	yes	yes	yes	yes	8
Bukara-Radujković, et al[1384]	2009	yes	no	yes	yes	yes	yes	yes	yes	yes	8
Markovic, et al[1385]	2015	yes	yes	yes	yes	yes	yes	yes	no	yes	8
Rakic, et al[1386]	2011	yes	yes	yes	no	yes	yes	no	no	yes	6
Srdić, et al[1387]	2012	yes	yes	yes	yes	yes	no	yes	yes	yes	8
Janic, et al[1388]	2013	yes	yes	yes	yes	yes	yes	yes	yes	yes	9
Bjelanovic, et al[1389]	2017	yes	yes	yes	yes	yes	yes	no	yes	yes	8
Halasi, et al[1390]	2018	yes	yes	yes	no	yes	yes	yes	no	yes	7
Chiolero, et al[1391]	2007	no	yes	yes	yes	yes	yes	yes	yes	yes	8
Bovet, et al[1392]	2007	yes	yes	yes	yes	yes	yes	no	yes	yes	8
Bovet, et al[1393]	2010	yes	no	yes	yes	yes	yes	yes	yes	yes	8
Yeo, et al[1394]	2019	yes	yes	yes	yes	yes	yes	yes	no	yes	8
Fu, et al[1395]	2003	no	yes	yes	yes	yes	yes	yes	yes	yes	8
Pwint, et al[1396]	2013	yes	yes	yes	yes	no	yes	yes	yes	yes	8
Sabanayagam, et al[1397]	2009	yes	yes	yes	yes	yes	yes	yes	no	yes	8
Deurenberg-Yap, et al[1398]	2009	yes	no	yes	yes	yes	yes	yes	yes	yes	8
Planinsec, et al[1399]	2004	yes	yes	yes	yes	yes	yes	yes	yes	yes	9
Planinsec, et al[1400]	2009	yes	yes	no	yes	yes	yes	yes	no	yes	7
Sedej, et	2014	yes	yes	yes	yes	yes	yes	yes	yes	no	8

© 2024 Zhang X et al. *JAMA Pediatrics.*

al[1401]											
Sedej, et al[1402]	2016	no	yes	yes	yes	yes	yes	no	yes	yes	7
Symington, et al[1403]	2015	yes	yes	yes	yes	yes	no	yes	yes	yes	8
Kirsten, et al[1404]	2013	yes	yes	yes	yes	yes	yes	yes	yes	yes	9
Nomatshila, et al[1405]	2022	yes	yes	yes	yes	yes	no	yes	yes	yes	8
Engwa, et al[1406]	2022	yes	yes	yes	yes	yes	no	yes	yes	yes	8
Abrahams, et al[1407]	2011	yes	yes	yes	yes	yes	yes	yes	yes	yes	9
Armstrong, et al[1408]	2006	yes	yes	yes	yes	yes	yes	yes	no	yes	8
Baard, et al[1409]	2014	no	yes	yes	yes	yes	yes	yes	yes	yes	8
Moselakgamo, et al[1410]	2015	yes	yes	no	yes	yes	yes	yes	no	yes	7
Oldewage-Theron, et al[1411]	2010	yes	yes	yes	yes	yes	no	yes	yes	no	7
Pedro, et al[1412]	2014	yes	yes	yes	yes	yes	no	yes	yes	yes	8
Pienaar, et al[1413]	2015	yes	yes	yes	yes	no	yes	no	yes	yes	7
Puckree, et al[1414]	2011	yes	yes	yes	yes	yes	yes	yes	yes	yes	9
Tathiah, et al[1415]	2013	yes	yes	no	yes	yes	yes	yes	yes	yes	8
Truter, et al[1416]	2015	yes	yes	yes	yes	yes	yes	no	no	yes	7
Wiles, et al[1417]	2013	yes	yes	yes	yes	yes	yes	yes	no	yes	8
Negash, et al[1418]	2017	yes	yes	yes	yes	no	yes	yes	yes	yes	8
Kimani-Murage, et al[1419]	2011	yes	yes	yes	yes	yes	no	yes	no	yes	7
Reddy, et al[1420]	2012	yes	yes	yes	yes	yes	yes	yes	yes	no	8
Moselakgomo, et al[1421]	2017	yes	yes	yes	yes	yes	yes	yes	no	yes	8
Modjadji, et al[1422]	2019	yes	yes	yes	yes	yes	yes	yes	yes	yes	9

© 2024 Zhang X et al. *JAMA Pediatrics.*

Pretorius, et al[1423]	2019	yes	yes	yes	yes	yes	yes	yes	no	yes	8
Monyeki, et al[1424]	2006	yes	yes	yes	yes	yes	yes	yes	no	yes	8
Kruger, et al[1425]	2006	yes	yes	yes	yes	yes	yes	yes	no	yes	8
Nkeh-Chungag, et al[1426]	2015	yes	yes	yes	yes	yes	yes	yes	no	yes	8
Meko, et al[1427]	2015	yes	yes	yes	yes	yes	no	yes	yes	yes	8
Van Niekerk, et al[1428]	2014	yes	yes	no	yes	no	yes	yes	yes	yes	7
Toriola, et al[1429]	2012	yes	yes	yes	yes	no	yes	yes	yes	yes	8
Craig, et al[1430]	2016	yes	yes	no	yes	yes	yes	yes	yes	yes	8
Choo, et al[1431]	2020	yes	yes	yes	no	yes	yes	yes	yes	yes	8
Park, et al[1432]	2022	yes	yes	yes	yes	yes	yes	yes	yes	yes	9
Kim, et al[1433]	2021	yes	yes	yes	yes	yes	yes	yes	yes	no	8
Park, et al[1434]	2013	yes	yes	yes	yes	yes	no	yes	yes	yes	8
Bae, et al[1435]	2021	yes	yes	yes	no	yes	yes	yes	yes	yes	8
Baek, et al[1436]	2012	yes	yes	yes	yes	yes	yes	no	yes	yes	8
Kim, et al[1437]	2012	yes	yes	yes	yes	no	yes	yes	yes	yes	8
Byun, et al[1438]	2012	yes	yes	yes	yes	yes	yes	yes	yes	yes	9
Lee, et al[1439]	2013	no	yes	yes	yes	yes	no	no	yes	yes	6
Lee, et al[1440]	2013	yes	yes	yes	yes	no	yes	yes	yes	yes	8
Bae, et al[1441]	2010	yes	yes	yes	yes	yes	yes	yes	no	yes	8
Lee, et al[1442]	2013	no	yes	yes	yes	yes	no	yes	yes	yes	7
Choo, et al[1443]	2017	yes	yes	yes	yes	no	yes	yes	no	yes	7
Le, et al[1444]	2020	yes	yes	yes	yes	yes	no	yes	yes	yes	8
Lee, et al[1445]	2012	yes	yes	yes	yes	yes	yes	yes	no	yes	8
Kim, et al[1446]	2005	yes	yes	yes	yes	yes	yes	yes	yes	no	8
Sunwoo, et al[1447]	2020	yes	yes	yes	no	yes	yes	yes	yes	yes	8
Yoo,	2020	yes	yes	yes	yes	yes	yes	no	yes	yes	8

et al[1448]											
Lee, et al[1449]	2010	yes	yes	yes	yes	yes	yes	yes	yes	no	8
Kim, et al[1450]	2010	yes	yes	no	yes	yes	yes	yes	yes	yes	8
Lee, et al[1451]	2009	yes	yes	no	yes	yes	yes	yes	no	yes	7
Chang, et al[1452]	2008	yes	no	yes	no	yes	yes	yes	no	yes	6
Ryu, et al[1453]	2007	yes	yes	yes	yes	yes	yes	yes	yes	yes	9
Yoo, et al[1454]	2006	yes	yes	yes	yes	yes	yes	yes	yes	yes	9
Lee, et al[1455]	2006	yes	yes	yes	no	yes	yes	yes	yes	yes	8
Yoo, et al[1456]	2015	no	yes	yes	yes	yes	yes	yes	yes	yes	8
Lee, et al[1457]	2015	yes	yes	yes	yes	yes	no	yes	yes	yes	8
Kong, et al[1458]	2015	yes	yes	yes	yes	yes	no	yes	yes	yes	8
Noh, et al[1459]	2014	yes	yes	yes	yes	yes	no	yes	yes	yes	8
Oh, et al[1460]	2011	yes	yes	yes	yes	yes	no	yes	yes	yes	8
Yoo, et al[1461]	2011	yes	yes	yes	yes	yes	yes	no	yes	yes	8
Kim, et al[1462]	2012	yes	yes	no	no	yes	yes	yes	yes	yes	7
Choi, et al[1463]	2013	yes	yes	yes	yes	yes	no	yes	yes	yes	8
Kim, et al[1464]	2017	no	yes	yes	yes	yes	yes	yes	yes	yes	8
Cho, et al[1465]	2018	yes	yes	yes	no	yes	yes	yes	yes	yes	8
Ara, et al[1466]	2007	yes	yes	yes	yes	yes	yes	yes	no	yes	8
Sánchez-Cruz, et al[1467]	2013	yes	yes	yes	yes	no	yes	yes	yes	yes	8
Gulías-González, et al[1468]	2014	no	yes	yes	yes	yes	yes	no	yes	yes	7
Iguacel, et al[1469]	2018	yes	yes	yes	yes	yes	yes	yes	no	yes	8
López-Gil, et al[1470]	2022	yes	yes	yes	yes	yes	yes	yes	no	yes	8
Lasarte-Velillas, et al[1471]	2022	yes	yes	yes	yes	yes	yes	yes	no	yes	8
Bont, et al[1472]	2022	yes	yes	yes	yes	no	yes	yes	yes	yes	8

© 2024 Zhang X et al. *JAMA Pediatrics*.

Cartanyà-Hueso, et al[1473]	2022	yes	yes	no	yes	yes	yes	yes	yes	yes	8
Cabeza, et al[1474]	2022	yes	yes	yes	yes	yes	yes	no	yes	yes	8
Aragón-Martín, et al[1475]	2022	no	yes	yes	yes	yes	yes	no	yes	yes	7
Vega-Ramirez, et al[1476]	2021	yes	yes	yes	yes	yes	yes	yes	yes	yes	9
Barja-Fernández, et al[1477]	2018	yes	yes	yes	yes	yes	yes	yes	yes	yes	9
Adelantado-Renau, et al[1478]	2018	yes	yes	yes	yes	yes	yes	yes	yes	no	8
Aguilar, et al[1479]	2010	yes	yes	yes	yes	yes	yes	yes	no	yes	8
Marcos-Pasero, et al[1480]	2019	no	yes	yes	yes	yes	yes	yes	yes	yes	8
Baile, et al[1481]	2020	yes	yes	no	yes	yes	no	yes	yes	yes	7
Bawaked, et al[1482]	2020	yes	yes	yes	yes	yes	yes	no	yes	yes	8
Bazán, et al[1483]	2018	yes	yes	yes	yes	yes	yes	yes	yes	no	8
Bont, et al[1484]	2019	yes	yes	yes	yes	yes	yes	yes	yes	no	8
Tamayo-Ortiz, et al[1485]	2021	yes	yes	yes	yes	yes	no	yes	yes	yes	8
Martín, et al[1486]	2008	yes	yes	yes	yes	yes	yes	yes	yes	yes	9
Llargues, et al[1487]	2011	yes	yes	yes	no	yes	yes	no	yes	yes	7
Santiago, et al[1488]	2013	yes	yes	yes	yes	yes	yes	yes	yes	yes	9
Riaño-Galán, et al[1489]	2017	yes	yes	yes	yes	yes	no	yes	yes	yes	8
Montero, et al[1490]	2005	yes	yes	yes	no	yes	yes	yes	yes	yes	8
Pérez-Bonaventura, et al[1491]	2015	yes	yes	yes	yes	yes	yes	yes	yes	no	8

© 2024 Zhang X et al. *JAMA Pediatrics*.

Garcia-Marcos, et al[1492]	2008	yes	yes	yes	yes	yes	yes	no	yes	yes	8
Garcia-Marcos, et al[1493]	2008	yes	yes	yes	yes	no	yes	no	yes	yes	7
Vázquez, et al[1494]	2010	yes	yes	yes	yes	yes	yes	no	yes	yes	8
Albaladejo, et al[1495]	2019	yes	yes	yes	yes	yes	yes	yes	yes	yes	9
Scholz, et al[1496]	2019	yes	yes	yes	yes	yes	no	yes	yes	yes	8
Esteban-Vasallo, et al[1497]	2020	yes	yes	yes	yes	no	yes	yes	no	yes	7
Martinez-Gomez, et al[1498]	2010	yes	yes	yes	yes	yes	yes	yes	no	yes	8
Bibiloni, et al[1499]	2010	yes	yes	yes	yes	no	yes	yes	no	yes	7
Larrañaga, et al[1500]	2007	yes	yes	yes	yes	yes	yes	no	yes	yes	8
Ayala, et al[1501]	2007	yes	yes	yes	yes	yes	yes	yes	yes	yes	9
Rojo, et al[1502]	2006	yes	yes	yes	yes	yes	no	no	yes	yes	7
Morales-Suárez-Varela, et al[1503]	2015	yes	yes	yes	yes	yes	yes	no	yes	yes	8
Gutiérrez-Zornoza, et al[1504]	2015	yes	yes	yes	yes	yes	no	yes	yes	yes	8
Alonso-Fernández, et al[1505]	2015	yes	yes	yes	yes	yes	no	yes	yes	yes	8
Veses, et al[1506]	2014	yes	yes	no	yes	no	yes	yes	yes	yes	7
Tobarra, et al[1507]	2014	yes	yes	yes	yes	no	yes	yes	yes	yes	8
Nova, et al[1508]	2014	yes	no	yes	yes	yes	yes	yes	yes	yes	8
Dadvand, et al[1509]	2014	yes	yes	yes	yes	yes	no	yes	yes	yes	8
Rodríguez-	2011	no	yes	yes	yes	yes	yes	yes	yes	yes	8

© 2024 Zhang X et al. *JAMA Pediatrics.*

Ramírez, et al[1510]											
Cerrillo, et al[1511]	2012	yes	yes	yes	no	yes	yes	yes	yes	yes	8
Martínez-Vizcaíno , et al[1512]	2012	yes	no	yes	yes	yes	no	yes	yes	yes	7
Navalpotro, et al[1513]	2012	yes	yes	yes	yes	yes	no	yes	yes	yes	8
Jiménez-Ormeño, et al[1514]	2013	yes	yes	yes	no	yes	no	yes	yes	yes	7
Laguna, et al[1515]	2013	yes	yes	yes	no	yes	yes	yes	yes	yes	8
Morales, et al[1516]	2013	yes	yes	yes	yes	yes	yes	no	yes	yes	8
Moreno, et al[1517]	2013	yes	yes	yes	yes	yes	yes	yes	no	yes	8
Pérez-Farinós, et al[1518]	2013	yes	yes	yes	yes	no	yes	yes	yes	yes	8
García-García , et al[1519]	2016	yes	yes	yes	yes	yes	yes	no	no	yes	7
Alvarez Zallo , et al[1520]	2017	yes	yes	yes	yes	yes	yes	no	yes	yes	8
Martín-Espinosa, et al[1521]	2017	yes	yes	yes	yes	yes	no	no	yes	yes	7
Ramos, et al[1522]	2018	yes	yes	yes	yes	yes	no	yes	yes	yes	8
Rathnayake, et al[1523]	2013	yes	yes	yes	yes	yes	yes	yes	no	yes	8
Wickramasinghe, et al[1524]	2013	yes	yes	yes	yes	yes	yes	no	yes	yes	8
Warnakulasuriya, et al[1525]	2019	yes	yes	yes	yes	yes	yes	no	no	yes	7
Wickramasinghe, et al[1526]	2009	yes	yes	yes	yes	yes	no	yes	yes	yes	8
Salman, et al[1527]	2010	yes	yes	yes	yes	yes	yes	yes	no	yes	8
Nagwa, et al[1528]	2011	yes	yes	yes	yes	yes	yes	no	yes	yes	8

© 2024 Zhang X et al. *JAMA Pediatrics.*

Wallby, et al[1529]	2017	yes	yes	yes	yes	yes	yes	yes	yes	yes	9
Hamano, et al[1530]	2017	yes	yes	yes	yes	yes	yes	yes	yes	yes	9
Önnestam, et al[1531]	2022	yes	yes	yes	yes	yes	yes	no	yes	yes	8
Fäldt, et al[1532]	2022	yes	yes	no	yes	yes	yes	yes	yes	yes	8
Sjöberg, et al[1533]	2005	yes	yes	yes	yes	yes	yes	no	yes	yes	8
Norberg, et al[1534]	2012	yes	yes	yes	yes	yes	yes	yes	no	yes	8
Nilsen, et al[1535]	2017	yes	yes	no	yes	yes	yes	no	yes	yes	7
Moraeus, et al[1536]	2014	yes	yes	yes	yes	yes	yes	no	yes	yes	8
Garmy, et al[1537]	2014	yes	yes	yes	yes	yes	yes	no	no	yes	7
Sjöberg, et al[1538]	2008	yes	yes	yes	yes	yes	yes	yes	yes	yes	9
Mårild, et al[1539]	2004	yes	yes	no	yes	yes	yes	yes	yes	yes	8
Mangrio, et al[1540]	2010	yes	yes	yes	yes	yes	yes	no	yes	yes	8
Koch, et al[1541]	2008	yes	yes	yes	yes	yes	yes	yes	yes	no	8
Stenhammar, et al[1542]	2010	yes	yes	yes	yes	yes	yes	yes	yes	yes	9
Börnhorst, et al[1543]	2015	yes	yes	no	yes	yes	yes	yes	yes	no	7
White, et al[1544]	2022	yes	yes	no	yes	yes	yes	no	yes	yes	7
Magnusson, et al[1545]	2005	yes	yes	yes	yes	yes	yes	yes	yes	yes	9
Erling, et al[1546]	2004	yes	yes	yes	yes	yes	yes	yes	yes	no	8
Litsfeldt, et al[1547]	2020	yes	yes	yes	no	yes	yes	yes	yes	yes	8
Lager, et al[1548]	2009	yes	yes	yes	yes	yes	yes	no	yes	yes	8
Guinhouya, et al[1549]	2009	yes	yes	yes	yes	no	yes	yes	yes	yes	8
Bergström, et al[1550]	2009	yes	yes	no	yes	yes	no	yes	yes	yes	7
Blomquist, et al[1551]	2007	yes	yes	yes	yes	no	yes	yes	yes	yes	8

© 2024 Zhang X et al. *JAMA Pediatrics*.

Moraeus, et al[1552]	2015	yes	yes	yes	yes	yes	yes	no	yes	yes	8
Lindkvist, et al[1553]	2015	yes	yes	yes	yes	yes	no	yes	yes	yes	8
Roos, et al[1554]	2014	yes	yes	yes	no	yes	yes	yes	yes	yes	8
Vaezghasemi, et al[1555]	2012	yes	yes	yes	yes	yes	yes	yes	yes	yes	9
Khanolkar, et al[1556]	2013	yes	yes	yes	yes	yes	yes	yes	yes	no	8
Munter, et al[1557]	2016	yes	yes	yes	yes	no	no	yes	yes	yes	7
Winkvist, et al[1558]	2016	yes	yes	yes	yes	yes	no	no	yes	yes	7
Aeberli, et al[1559]	2010	yes	yes	yes	yes	yes	yes	yes	yes	yes	9
Aeberli, et al[1560]	2010	yes	yes	yes	yes	yes	yes	yes	no	yes	8
Jeannot, et al[1561]	2015	yes	yes	yes	yes	yes	yes	yes	yes	no	8
Lasserre, et al[1562]	2007	no	yes	yes	yes	yes	yes	yes	yes	yes	8
Zimmermann, et al[1563]	2004	yes	no	yes	no	yes	yes	yes	yes	yes	7
Köchli, et al[1564]	2019	yes	yes	no	yes	yes	yes	yes	yes	yes	8
Aeberli, et al[1565]	2013	yes	yes	yes	yes	yes	yes	yes	no	yes	8
Bonvin, et al[1566]	2012	yes	yes	yes	yes	yes	yes	yes	yes	yes	9
Nasreddine, et al[1567]	2010	no	yes	yes	yes	no	yes	yes	yes	yes	7
Taguri, et al[1568]	2009	yes	yes	no	yes	yes	yes	yes	yes	yes	8
Chen, et al[1569]	2023	yes	yes	yes	yes	no	yes	yes	yes	yes	8
Yen, et al[1570]	2021	yes	yes	yes	yes	yes	yes	yes	yes	no	8
Wang, et al[1571]	2021	yes	yes	yes	yes	yes	no	yes	yes	yes	8
Chen, et al[1572]	2012	yes	yes	yes	yes	yes	yes	yes	no	yes	8
Chu, et al[1573]	2007	yes	yes	yes	yes	yes	yes	yes	no	yes	8
Liou, et al[1574]	2010	yes	yes	yes	yes	yes	yes	yes	no	yes	8
Ting, et	2012	yes	yes	yes	yes	yes	yes	yes	no	yes	8

© 2024 Zhang X et al. *JAMA Pediatrics*.

al[1575]											
Tsai, et al[1576]	2009	yes	yes	yes	yes	yes	no	yes	no	yes	7
Chen, et al[1577]	2019	yes	yes	yes	yes	yes	yes	yes	yes	yes	9
Lai, et al[1578]	2001	yes	no	yes	yes	yes	yes	yes	yes	yes	8
Yen, et al[1579]	2010	yes	yes	yes	yes	yes	no	yes	yes	yes	8
Pu, et al[1580]	2010	yes	yes	yes	yes	yes	yes	yes	no	yes	8
Chang, et al[1581]	2010	yes	no	yes	no	yes	yes	yes	yes	yes	7
Chen, et al[1582]	2009	yes	yes	yes	yes	yes	no	yes	yes	yes	8
Chen, et al[1583]	2008	yes	yes	yes	yes	yes	no	yes	yes	no	7
Chang, et al[1584]	2015	yes	yes	yes	yes	yes	yes	yes	yes	yes	9
Lee, et al[1585]	2014	yes	yes	yes	yes	yes	yes	yes	yes	yes	9
Hsieh, et al[1586]	2014	yes	yes	yes	yes	no	yes	yes	yes	yes	8
Chen, et al[1587]	2014	yes	yes	yes	yes	yes	no	yes	yes	yes	8
Chiang, et al[1588]	2013	yes	yes	yes	yes	yes	yes	yes	yes	yes	9
Liao, et al[1589]	2013	yes	yes	yes	no	yes	yes	yes	yes	yes	8
Chen, et al[1590]	2016	yes	yes	yes	yes	yes	no	yes	yes	yes	8
Chang, et al[1591]	2018	yes	no	yes	yes	yes	yes	yes	yes	no	7
Mosha, et al[1592]	2021	yes	yes	yes	yes	yes	no	yes	yes	yes	8
Mosha, et al[1593]	2010	yes	yes	yes	yes	yes	yes	yes	yes	yes	9
Mpembeni, et al[1594]	2014	yes	yes	yes	yes	yes	yes	no	yes	yes	8
Mwaikambo, et al[1595]	2015	yes	no	yes	yes	yes	no	yes	no	yes	6
Pangani, et al[1596]	2016	no	yes	yes	yes	yes	yes	yes	yes	yes	8
Chomba, et al[1597]	2019	no	no	yes	yes	yes	yes	yes	yes	yes	7
Shinsugi, et al[1598]	2021	yes	yes	yes	yes	yes	yes	yes	yes	no	8
Rerksuppaphol, et al[1599]	2021	yes	yes	yes	yes	yes	yes	no	no	yes	7

© 2024 Zhang X et al. *JAMA Pediatrics.*

Weeraarchakul, et al[1600]	2017	yes	yes	yes	yes	yes	yes	yes	no	yes	8
Nonbo onyawat, et al[1601]	2019	yes	yes	yes	no	yes	yes	yes	yes	yes	8
Pawloski, et al[1602]	2008	yes	yes	yes	yes	yes	yes	yes	no	yes	8
Manandhar, et al[1603]	2019	yes	yes	yes	yes	yes	yes	yes	yes	no	8
Sengmeuang, et al[1604]	2010	yes	yes	yes	yes	yes	yes	yes	no	yes	8
Rerksuppaphol, et al[1605]	2010	yes	yes	yes	yes	yes	yes	yes	yes	yes	9
Narksawat, et al[1606]	2009	yes	yes	yes	yes	yes	yes	no	yes	yes	8
Rerksuppaphol, et al[1607]	2015	no	yes	yes	yes	yes	no	yes	yes	yes	7
Sukhonthachit, et al[1608]	2014	yes	yes	yes	yes	yes	yes	yes	yes	yes	9
Firestone, et al[1609]	2011	yes	yes	yes	yes	yes	yes	yes	yes	yes	9
Jitnarin, et al[1610]	2011	yes	yes	yes	no	yes	yes	yes	yes	yes	8
Rojroongwasinkul, et al[1611]	2013	yes	yes	yes	yes	yes	yes	no	yes	yes	8
Thasanasuwan, et al[1612]	2016	yes	yes	yes	yes	yes	no	yes	yes	yes	8
Sagbo, et al[1613]	2018	yes	yes	yes	yes	yes	yes	yes	no	yes	8
Smith, et al[1614]	2007	yes	yes	yes	yes	yes	yes	yes	yes	yes	9
Simeon, et al[1615]	2003	no	yes	yes	no	yes	yes	yes	yes	yes	7
Mumena, et al[1616]	2018	yes	yes	no	yes	yes	yes	yes	yes	yes	8
Bouktir, et al[1617]	2011	yes	yes	yes	no	yes	no	yes	yes	yes	7
Ercan, et al[1618]	2012	yes	yes	yes	yes	yes	no	yes	yes	yes	8
Salman, et al[1619]	2022	yes	yes	yes	yes	yes	yes	yes	yes	yes	9
Meydanlioglu, et	2022	yes	no	yes	yes	yes	yes	yes	yes	yes	8

© 2024 Zhang X et al. *JAMA Pediatrics.*

al[1620]											
Gunalan, et al[1621]	2021	yes	no	yes	yes	yes	yes	yes	yes	yes	8
Arslan, et al[1622]	2021	yes	yes	yes	yes	yes	yes	yes	yes	yes	9
Acar Tek, et al[1623]	2017	yes	yes	yes	yes	yes	yes	no	yes	yes	8
Sağlam, et al[1624]	2008	yes	yes	yes	yes	yes	yes	yes	no	yes	8
Sur, et al[1625]	2005	yes	yes	yes	yes	yes	yes	yes	yes	yes	9
Gundogdu, et al[1626]	2008	yes	yes	yes	yes	yes	yes	yes	yes	yes	9
Turkkahraman, et al[1627]	2006	yes	yes	yes	yes	yes	yes	yes	no	yes	8
Nur, et al[1628]	2008	yes	yes	yes	yes	yes	yes	yes	yes	yes	9
Bayat, et al[1629]	2009	yes	yes	yes	yes	yes	yes	yes	no	yes	8
Discigil, et al[1630]	2009	yes	yes	yes	yes	yes	yes	yes	yes	yes	9
Etiler, et al[1631]	2011	yes	yes	yes	no	yes	no	yes	yes	yes	7
Ozmen, et al[1632]	2007	yes	yes	yes	yes	yes	yes	yes	yes	yes	9
Simsek, et al[1633]	2008	yes	yes	yes	yes	yes	yes	yes	no	yes	8
Yuca, et al[1634]	2010	yes	yes	yes	yes	yes	yes	yes	yes	yes	9
Pirinççi, et al[1635]	2010	yes	yes	yes	yes	yes	no	yes	yes	yes	8
Duzova, et al[1636]	2013	yes	yes	yes	yes	yes	yes	yes	yes	yes	9
Demirci, et al[1637]	2013	yes	yes	yes	no	yes	no	yes	yes	yes	7
Polat, et al[1638]	2014	yes	yes	yes	yes	no	yes	yes	yes	yes	8
Gökler, et al[1639]	2015	yes	yes	yes	yes	yes	yes	no	yes	yes	8
Agirbasli, et al[1640]	2011	yes	yes	yes	no	no	yes	yes	yes	yes	7
Manios, et al[1641]	2005	yes	yes	yes	yes	yes	yes	yes	yes	yes	9
Yardim, et al[1642]	2019	yes	yes	yes	yes	yes	yes	yes	yes	no	8
Akbulut, et al[1643]	2014	yes	yes	yes	yes	yes	no	yes	yes	yes	8
Canan, et al[1644]	2014	yes	yes	yes	yes	yes	yes	no	yes	yes	8

© 2024 Zhang X et al. *JAMA Pediatrics*.

Dündar, et al[1645]	2012	yes	yes	yes	yes	yes	no	yes	no	yes	7
Gültekin, et al[1646]	2005	no	yes	yes	yes	yes	yes	yes	yes	yes	8
Uçkun-Kitapçi, et al[1647]	2004	yes	yes	yes	yes	yes	yes	yes	yes	no	8
Oner, et al[1648]	2004	yes	yes	yes	yes	no	yes	yes	yes	yes	8
Ardıç, et al[1649]	2019	yes	yes	yes	yes	yes	no	yes	no	yes	7
Aşut, et al[1650]	2019	yes	yes	yes	yes	no	yes	yes	yes	yes	8
Çelmeli, et al[1651]	2019	no	yes	yes	yes	yes	yes	yes	yes	yes	8
Comba, et al[1652]	2019	yes	yes	yes	yes	yes	yes	yes	yes	yes	9
Deniz, et al[1653]	2019	yes	yes	yes	yes	yes	yes	yes	yes	no	8
Karakus, et al[1654]	2019	yes	yes	yes	yes	yes	yes	yes	yes	yes	9
Arikan, et al[1655]	2020	yes	yes	yes	yes	yes	no	no	yes	yes	7
Esenay, et al[1656]	2010	yes	yes	yes	yes	yes	no	yes	no	yes	7
Ucar, et al[1657]	2009	yes	yes	yes	yes	yes	yes	yes	yes	yes	9
Garipagaoglu, et al[1658]	2009	yes	yes	yes	yes	yes	yes	yes	yes	no	8
Dinç, et al[1659]	2009	yes	yes	yes	yes	yes	yes	yes	yes	yes	9
Önsüz, et al[1660]	2015	yes	yes	no	yes	yes	yes	yes	yes	yes	8
Meseri, et al[1661]	2015	yes	yes	yes	yes	yes	yes	yes	yes	yes	9
Inal, et al[1662]	2015	yes	yes	yes	yes	yes	no	yes	yes	yes	8
Vehapoglu, et al[1663]	2014	no	yes	yes	yes	yes	yes	no	yes	yes	7
Cabar, et al[1664]	2014	yes	yes	yes	yes	yes	yes	yes	yes	no	8
Ayyildiz, et al[1665]	2014	yes	yes	yes	yes	yes	yes	yes	yes	no	8
Calisir, et al[1666]	2011	yes	yes	yes	yes	yes	yes	no	yes	yes	8
Cinar, et al[1667]	2011	yes	yes	yes	yes	yes	yes	yes	yes	yes	9
Akca, et	2016	yes	yes	yes	yes	yes	yes	yes	no	yes	8

© 2024 Zhang X et al. *JAMA Pediatrics.*

al[1668]											
Geckil, et al[1669]	2017	no	yes	yes	yes	yes	yes	yes	yes	yes	8
Gül, et al[1670]	2017	yes	yes	yes	yes	yes	no	yes	yes	yes	8
Eker, et al[1671]	2018	yes	yes	yes	yes	yes	no	yes	yes	yes	8
Haney, et al[1672]	2018	no	yes	yes	no	yes	yes	yes	yes	yes	7
Dereń, et al[1673]	2020	yes	yes	yes	yes	yes	yes	yes	no	yes	8
Dereń, et al[1674]	2018	yes	yes	no	yes	yes	yes	yes	yes	yes	8
AlBlooshi, et al[1675]	2016	yes	yes	yes	no	yes	no	yes	yes	yes	7
Ismail, et al[1676]	2022	no	yes	no	yes	yes	yes	yes	yes	yes	7
Abduelkarem, et al[1677]	2020	yes	yes	yes	yes	yes	yes	no	yes	yes	8
Abdullatif, et al[1678]	2022	yes	yes	yes	yes	no	yes	yes	yes	yes	8
Aburawi, et al[1679]	2019	no	yes	yes	yes	yes	yes	yes	yes	yes	8
Zaal, et al[1680]	2009	yes	yes	yes	yes	yes	yes	yes	yes	no	8
Fatima, et al[1681]	2018	yes	yes	yes	yes	yes	yes	no	yes	yes	8
Adab, et al[1682]	2018	yes	yes	yes	no	yes	yes	yes	yes	yes	8
Sweeting, et al[1683]	2005	yes	yes	no	yes	yes	yes	yes	yes	no	7
Fraser, et al[1684]	2012	yes	yes	yes	yes	yes	no	no	yes	yes	7
Gilliland, et al[1685]	2012	yes	yes	yes	yes	yes	yes	no	yes	yes	8
Uerlich, et al[1686]	2021	yes	yes	yes	yes	yes	yes	yes	yes	no	8
Ralphs, et al[1687]	2021	yes	yes	yes	yes	no	yes	yes	no	yes	7
Adab, et al[1688]	2014	yes	yes	yes	yes	yes	yes	no	yes	yes	8
Bartle, et al[1689]	2013	yes	yes	yes	yes	yes	yes	no	yes	yes	8
Pearce, et al[1690]	2010	yes	yes	yes	yes	no	yes	yes	yes	yes	8
Harding, et al[1691]	2008	yes	yes	yes	yes	yes	yes	yes	yes	no	8
Bonuck, et al[1692]	2015	yes	yes	yes	yes	yes	yes	yes	no	yes	8

© 2024 Zhang X et al. *JAMA Pediatrics*.

Steele, et al[1693]	2009	yes	yes	yes	yes	no	yes	yes	yes	yes	8
Basterfield, et al[1694]	2014	yes	yes	yes	yes	yes	yes	yes	yes	no	8
Griffiths, et al[1695]	2014	yes	yes	yes	yes	yes	yes	no	yes	yes	8
Green, et al[1696]	2021	yes	yes	yes	yes	yes	yes	yes	no	yes	8
Scott, et al[1697]	2016	yes	yes	no	yes	yes	yes	yes	yes	yes	8
Clemente, et al[1698]	2019	yes	yes	yes	yes	yes	yes	no	no	yes	7
Griffiths, et al[1699]	2011	yes	yes	yes	yes	yes	yes	yes	yes	no	8
Tiffin, et al[1700]	2011	yes	yes	no	yes	yes	yes	yes	yes	yes	8
Mendez, et al[1701]	2008	yes	yes	yes	yes	yes	yes	yes	no	no	7
Harrison, et al[1702]	2011	yes	yes	yes	yes	no	yes	yes	yes	no	7
Taylor, et al[1703]	2005	yes	yes	yes	yes	yes	yes	yes	yes	yes	9
Cecil, et al[1704]	2005	yes	yes	yes	yes	yes	yes	yes	yes	yes	9
Fletcher, et al[1705]	2004	yes	yes	no	yes	yes	yes	yes	yes	yes	8
Webster-Gandy, et al[1706]	2003	yes	yes	yes	yes	no	yes	yes	yes	no	7
Warren, et al[1707]	2003	yes	yes	yes	yes	yes	yes	yes	no	yes	8
Whitaker, et al[1708]	2010	yes	yes	yes	no	yes	yes	yes	yes	yes	8
Thomas, et a[1709]	2010	yes	yes	yes	yes	yes	no	yes	yes	yes	8
Skidmore, et al[1710]	2010	yes	yes	no	yes	yes	yes	yes	yes	yes	8
Jennings, et al[1711]	2010	yes	yes	yes	yes	yes	yes	yes	no	yes	8
Edwards, et al[1712]	2010	yes	yes	yes	yes	yes	yes	yes	yes	no	8
Willianmson, et a[1713]	2009	yes	yes	yes	yes	yes	no	yes	yes	yes	8
Webber, et a[1714]	2009	yes	yes	yes	yes	yes	yes	yes	yes	yes	9
Standley, et al[1715]	2009	no	yes	yes	yes	yes	yes	no	yes	yes	7
Nelson, et	2009	yes	yes	no	yes	no	yes	yes	yes	yes	7

© 2024 Zhang X et al. *JAMA Pediatrics.*

al[1716]											
McCullough, et al[1717]	2009	yes	yes	yes	yes	no	yes	yes	yes	yes	8
Fox, et al[1718]	2009	yes	no	yes	yes	yes	yes	yes	yes	yes	8
Fairclough, et al[1719]	2009	yes	yes	yes	yes	no	yes	yes	yes	yes	8
van Sluijs, et al[1720]	2008	no	yes	yes	yes	yes	yes	yes	yes	no	7
Harding, et al[1721]	2008	yes	yes	yes	yes	yes	yes	yes	yes	yes	9
Routh, et al[1722]	2006	yes	yes	yes	yes	no	yes	yes	yes	yes	8
Mutunga, et al[1723]	2006	yes	yes	yes	yes	yes	yes	yes	yes	yes	9
Solmi, et al[1724]	2015	yes	yes	yes	yes	yes	yes	yes	yes	no	8
Jackson, et al[1725]	2015	no	yes	yes	yes	yes	yes	yes	yes	yes	8
Falconer, et al[1726]	2014	yes	yes	yes	yes	no	yes	no	yes	yes	7
Pallan, et al[1727]	2011	yes	yes	yes	yes	yes	yes	yes	yes	no	8
Coulthard, et al[1728]	2016	no	yes	yes	yes	yes	yes	yes	yes	yes	8
Beynon, et al[1729]	2017	no	yes	yes	yes	yes	yes	yes	yes	yes	8
Hudda, et al[1730]	2018	yes	yes	yes	yes	no	yes	yes	yes	yes	8
Wang, et al[1731]	2010	yes	yes	yes	yes	yes	no	yes	yes	no	7
Chiasson, et al[1732]	2016	yes	yes	yes	no	yes	yes	yes	yes	yes	8
Ehrenthal, et al[1733]	2016	yes	yes	yes	yes	yes	yes	no	yes	yes	8
Taveras, et al[1734]	2006	yes	yes	yes	yes	yes	yes	yes	yes	no	8
Weden, et al[1735]	2012	yes	yes	yes	yes	no	no	no	yes	yes	6
Laurson, et al[1736]	2014	yes	yes	yes	yes	yes	yes	yes	yes	yes	9
Vehrs, et al[1737]	2022	yes	yes	yes	yes	yes	yes	yes	yes	no	8
Vazquez, et al[1738]	2022	yes	yes	yes	yes	yes	no	yes	yes	yes	8
Leung, et al[1739]	2011	yes	yes	yes	yes	yes	yes	no	yes	no	7
Shier, et	2012	yes	yes	yes	yes	yes	yes	yes	yes	yes	9

© 2024 Zhang X et al. *JAMA Pediatrics*.

al[1740]											
Salcido, et al[1741]	2022	yes	yes	yes	yes	no	yes	no	yes	yes	7
Odusanya, et al[1742]	2022	yes	yes	no	yes	yes	yes	yes	yes	yes	8
Mayne, et al[1743]	2023	yes	yes	yes	yes	yes	yes	yes	yes	yes	9
Bader, et al[1744]	2013	yes	no	yes	yes	yes	yes	no	yes	yes	7
Burdette, et al[1745]	2004	yes	yes	yes	yes	yes	yes	yes	yes	yes	9
Carroll-Scott, et al[1746]	2013	yes	yes	yes	yes	yes	yes	no	yes	yes	8
Hunt, et al[1747]	2022	yes	yes	yes	yes	yes	yes	yes	yes	yes	9
Fyfe-Johnson, et al[1748]	2022	yes	yes	no	yes	no	yes	yes	yes	yes	7
Flórez, et al[1749]	2022	yes	yes	yes	yes	yes	yes	yes	no	yes	8
Davis, et al[1750]	2009	yes	yes	yes	yes	no	yes	yes	yes	yes	8
Salazar, et al[1751]	2022	no	yes	yes	yes	yes	yes	no	yes	yes	7
Galvez, et al[1752]	2009	yes	yes	yes	yes	yes	yes	yes	yes	no	8
Bejarano, et al[1753]	2022	no	yes	no	yes	yes	yes	yes	no	yes	6
Findling, et al[1754]	2018	yes	yes	yes	yes	yes	yes	yes	yes	no	8
Zhang, et al[1755]	2021	no	yes	yes	yes	yes	yes	yes	yes	yes	8
Kepper, et al[1756]	2016	yes	no	yes	yes	yes	yes	yes	yes	yes	8
Stine, et al[1757]	2021	yes	no	yes	yes	yes	yes	no	yes	yes	7
Reilly, et al[1758]	2021	no	yes	yes	yes	yes	yes	yes	yes	yes	8
Liu, et al[1759]	2007	yes	yes	yes	yes	yes	yes	yes	yes	no	8
Imoisili, et al[1760]	2021	yes	yes	no	yes	yes	yes	yes	yes	yes	8
Mellor, et al[1761]	2011	yes	yes	yes	yes	no	yes	yes	yes	no	7
Ohri-Vachaspati, et al[1762]	2015	yes	yes	yes	yes	yes	yes	yes	no	yes	8
Oreskovic, et	2009	yes	yes	yes	yes	yes	yes	yes	yes	no	8

© 2024 Zhang X et al. *JAMA Pediatrics.*

al[1763]											
Salois, et al[1764]	2012	no	yes	yes	yes	yes	yes	yes	yes	yes	8
Sánchez, et al[1765]	2012	yes	yes	yes	yes	yes	yes	yes	yes	yes	9
Tang, et al[1766]	2014	yes	yes	yes	no	yes	yes	no	yes	yes	7
Wasserman, et al[1767]	2014	yes	yes	yes	no	yes	yes	yes	yes	yes	8
Halfon, et al[1768]	2013	yes	yes	yes	yes	yes	yes	yes	yes	yes	9
Hurt, et al[1769]	2014	no	yes	yes	yes	yes	yes	yes	yes	yes	8
Sacheck, et al[1770]	2011	yes	yes	yes	yes	yes	yes	yes	no	yes	8
Turer, et al[1771]	2013	yes	yes	yes	yes	yes	yes	no	yes	yes	8
Moore, et al[1772]	2016	yes	yes	yes	no	yes	yes	yes	no	yes	7
Lee, etal[1773]	2007	yes	yes	yes	yes	yes	yes	yes	yes	yes	9
Achaya, et al[1774]	2011	yes	yes	yes	yes	yes	yes	yes	no	yes	8
Adachi-Mejia, et al[1775]	2007	yes	yes	yes	yes	yes	yes	yes	yes	yes	9
Adams, et al[1776]	2010	no	yes	yes	yes	yes	yes	yes	yes	yes	8
Adams, et al[1777]	2005	yes	yes	yes	yes	yes	yes	yes	yes	yes	9
Adams, et al[1778]	2019	yes	yes	yes	yes	yes	yes	yes	no	yes	8
Adams, et al[1779]	2013	yes	yes	yes	no	yes	yes	yes	no	yes	7
Ogden, et al[1780]	2018	yes	yes	yes	yes	no	yes	yes	yes	yes	8
Davis, et al[1781]	2011	yes	yes	yes	yes	no	yes	yes	yes	yes	8
Lewis, et al[1782]	2006	no	yes	yes	yes	yes	yes	yes	yes	yes	8
Singh, et al[1783]	2008	yes	yes	yes	yes	yes	yes	yes	no	yes	8
Davis, et al[1784]	2008	no	yes	yes	yes	yes	yes	yes	no	yes	7
Agazzi, et al[1785]	2010	yes	yes	yes	yes	yes	no	yes	yes	yes	8
DeBoer, et al[1786]	2015	yes	yes	yes	yes	yes	yes	yes	yes	no	8
Govindan, et	2013	no	yes	yes	yes	yes	yes	yes	no	yes	7

© 2024 Zhang X et al. *JAMA Pediatrics.*

al[1787]											
Wojcicki, et al[1788]	2011	yes	yes	yes	yes	yes	yes	yes	yes	yes	9
Beck, et al[1789]	2014	yes	yes	yes	yes	yes	no	yes	yes	yes	8
Badon, et al[1790]	2020	yes	yes	yes	yes	yes	yes	yes	yes	yes	9
Bai, et al[1791]	2016	no	yes	yes	no	yes	yes	yes	no	yes	6
Giammattei[1792]	2003	yes	yes	yes	yes	yes	yes	yes	yes	no	8
Barlow, et al[1793]	2007	yes	yes	yes	yes	yes	yes	no	yes	yes	8
Barrera, et al[1794]	2016	yes	yes	yes	no	yes	yes	yes	yes	yes	8
Barroso, et al[1795]	2012	yes	yes	no	yes	yes	yes	yes	yes	no	7
Baxter, et al[1796]	2013	no	yes	yes	yes	yes	yes	yes	yes	yes	8
Baxter, et al[1797]	2011	no	yes	yes	yes	yes	yes	yes	yes	yes	8
Geier, et al[1798]	2007	yes	yes	yes	yes	yes	yes	yes	no	yes	8
Shore, et al[1799]	2008	yes	yes	yes	yes	yes	yes	yes	yes	no	8
Pan, et al[1800]	2013	no	yes	yes	yes	no	yes	yes	yes	yes	7
Kim, et al[1801]	2018	yes	yes	yes	yes	yes	yes	yes	yes	no	8
Seicean, et al[1802]	2007	no	yes	yes	yes	yes	yes	yes	yes	yes	8
Lumeng, et al[1803]	2007	yes	yes	yes	yes	yes	no	yes	yes	yes	8
Miller, et al[1804]	2014	yes	yes	yes	yes	yes	no	yes	yes	yes	8
Silva, et al[1805]	2011	no	yes	no	yes	yes	yes	yes	yes	yes	7
Fiorito, et al[1806]	2006	yes	yes	yes	yes	yes	yes	yes	yes	yes	9
Charvet, et al[1807]	2019	yes	yes	yes	no	yes	yes	yes	yes	yes	8
Haidar, et al[1808]	2019	yes	yes	yes	yes	yes	yes	yes	yes	no	8
Hu, et al[1809]	2018	yes	yes	yes	yes	yes	yes	yes	yes	yes	9
Drake, et al[1810]	2012	yes	yes	yes	no	yes	yes	yes	yes	yes	8
Suglia, et al[1811]	2013	yes	no	yes	yes	yes	no	yes	yes	yes	7
Drake, et	2013	yes	yes	yes	yes	yes	yes	yes	yes	no	8

© 2024 Zhang X et al. *JAMA Pediatrics*.

al[1812]											
Pérez, et al[1813]	2015	yes	yes	yes	yes	yes	yes	yes	no	no	7
Ghosh-Dastidar, et al[1814]	2016	yes	yes	yes	yes	yes	no	yes	yes	yes	8
Berkowitz, et al[1815]	2005	yes	no	yes	yes	yes	yes	yes	yes	yes	8
Gillman, et al[1816]	2008	yes	yes	yes	yes	yes	yes	yes	yes	yes	9
Hinkle, et al[1817]	2012	yes	yes	yes	no	yes	no	yes	yes	yes	7
Kubo, et al[1818]	2016	yes	yes	yes	yes	yes	yes	yes	yes	yes	9
Lindberg, et al[1819]	2012	yes	yes	yes	yes	yes	yes	yes	yes	no	8
Olson, et al[1820]	2010	yes	no	yes	yes	yes	yes	yes	yes	yes	8
Wojcicki, et al[1821]	2015	yes	yes	yes	yes	yes	no	yes	yes	yes	8
Bider-Canfield, et al[1822]	2017	yes	yes	yes	yes	yes	yes	yes	yes	no	8
Pham, et al[1823]	2013	yes	yes	yes	yes	yes	yes	yes	yes	yes	9
Ohri-Vachaspati, et al[1824]	2013	yes	yes	yes	no	yes	yes	yes	no	yes	7
Han, et al[1825]	2020	yes	yes	yes	no	yes	yes	yes	yes	yes	8
Jia, et al[1826]	2019	no	yes	no	yes	yes	no	yes	yes	yes	6
Elbel, et al[1827]	2019	yes	yes	yes	yes	yes	yes	yes	yes	no	8
Reis, et al[1828]	2020	no	yes	yes	no	yes	yes	yes	yes	yes	7
Ohri-Vachaspati, et al[1829]	2021	yes	yes	yes	yes	yes	yes	yes	yes	no	8
Bailey, et al[1830]	2014	yes	yes	yes	yes	yes	yes	yes	yes	yes	9
Ville, et al[1831]	2017	yes	yes	yes	yes	yes	yes	yes	no	yes	8
Theall, et al[1832]	2019	yes	yes	yes	yes	yes	yes	yes	yes	yes	9
Wojcicki, et al[1833]	2016	yes	yes	yes	yes	no	yes	yes	yes	no	7
Zhu, et	2015	yes	yes	yes	yes	yes	yes	yes	yes	yes	9

© 2024 Zhang X et al. *JAMA Pediatrics.*

al[1834]											
Harley, et al[1835]	2013	yes	yes	yes	yes	yes	yes	no	yes	yes	8
Huh, et al[1836]	2012	yes	yes	yes	yes	no	yes	yes	no	yes	7
Wang, et al[1837]	2013	yes	yes	yes	yes	yes	no	yes	yes	yes	8
Peck, et al[1838]	2015	yes	yes	yes	yes	yes	yes	yes	no	yes	8
Miles, et al[1839]	2018	yes	yes	yes	yes	yes	yes	yes	yes	no	8
Kjaer, et al[1840]	2018	yes	yes	yes	yes	yes	yes	yes	yes	yes	9
DuBose, et al[1841]	2006	yes	yes	yes	yes	yes	yes	yes	no	yes	8
Heer, et al[1842]	2013	no	yes	yes	yes	yes	yes	yes	yes	no	7
Oken, et al[1843]	2005	yes	yes	yes	yes	yes	yes	no	yes	yes	8
Braun, et al[1844]	2010	yes	yes	yes	yes	yes	yes	yes	yes	yes	9
Mosli, et al[1845]	2016	yes	yes	yes	yes	yes	yes	yes	yes	no	8
Block, et al[1846]	2018	yes	yes	yes	yes	yes	no	yes	no	yes	7
Assari, et al[1847]	2015	yes	yes	yes	yes	yes	yes	yes	yes	yes	9
Boutelle, et al[1848]	2010	yes	yes	yes	yes	yes	yes	yes	yes	yes	9
Kubzansky, et al[1849]	2012	no	yes	yes	yes	yes	yes	yes	yes	yes	8
McClure, et al[1850]	2010	yes	yes	yes	yes	yes	no	yes	yes	yes	8
Roberts, et al[1851]	2013	yes	yes	yes	yes	no	yes	yes	yes	yes	8
Borges, et al[1852]	2010	yes	yes	yes	yes	yes	yes	no	no	yes	7
Vannucci, et al[1853]	2017	yes	yes	yes	yes	yes	yes	yes	yes	yes	9
Adams, et al[1854]	2005	yes	yes	yes	yes	yes	yes	no	yes	no	7
Hack, et al[1855]	2011	no	yes	yes	yes	yes	yes	yes	yes	yes	8
Vohr, et al[1856]	2018	yes	yes	yes	yes	no	yes	yes	yes	yes	8
Wood, et al[1857]	2018	no	no	yes	yes	yes	yes	yes	no	yes	6
Cottrell, et	2011	yes	yes	yes	yes	yes	no	yes	yes	yes	8

© 2024 Zhang X et al. *JAMA Pediatrics*.

al[1858]											
Vangeeparam, et al[1859]	2011	yes	yes	yes	yes	yes	yes	yes	yes	yes	9
Kwon, et al[1860]	2006	yes	yes	no	yes	yes	yes	yes	yes	yes	8
Musaad, et al[1861]	2009	no	yes	yes	yes	yes	yes	no	yes	yes	7
Anderson, et al[1862]	2014	yes	yes	yes	yes	yes	yes	yes	yes	yes	9
Taveras, et al[1863]	2014	yes	yes	yes	yes	yes	yes	yes	yes	no	8
Scharf, et al[1864]	2015	yes	yes	yes	yes	yes	yes	yes	no	yes	8
Storfer-Isser, et al[1865]	2012	yes	yes	yes	yes	yes	yes	yes	yes	yes	9
Bell, et al[1866]	2010	no	yes	yes	yes	yes	yes	yes	yes	yes	8
Roberts, et al[1867]	2013	yes	yes	yes	yes	yes	no	yes	yes	yes	8
Anderson, et al[1868]	2011	yes	yes	yes	yes	yes	yes	yes	no	yes	8
Lohman, et al[1869]	2009	no	yes	yes	yes	yes	yes	yes	yes	yes	8
Foster, et al[1870]	2008	yes	yes	yes	yes	yes	no	yes	no	yes	7
Chomitz, et al[1871]	2010	yes	yes	yes	yes	yes	yes	yes	no	yes	8
Shriver, et al[1872]	2011	yes	no	yes	yes	yes	yes	no	yes	yes	7
Eagle, et al[1873]	2010	yes	yes	yes	yes	yes	no	yes	yes	yes	8
Lee, et al[1874]	2010	no	yes	yes	yes	yes	yes	yes	yes	yes	8
Kunin-Batson, et al[1875]	2023	yes	yes	yes	yes	yes	yes	yes	no	yes	8
Wu, et al[1876]	2022	yes	yes	no	yes	yes	yes	yes	no	yes	7
Arcan, et al[1877]	2012	yes	no	yes	yes	yes	yes	yes	yes	yes	8
Armstrong, et al[1878]	2012	yes	yes	yes	no	yes	yes	yes	yes	no	7
Stettler, et al[1879]	2005	yes	yes	yes	yes	yes	yes	yes	yes	yes	9
Rappaport, et al[1880]	2005	yes	yes	yes	yes	yes	yes	yes	yes	yes	9
Kim, et al[1881]	2005	yes	yes	yes	yes	yes	yes	yes	yes	yes	9
Keysey, et	2005	yes	yes	yes	yes	yes	yes	yes	yes	no	8

© 2024 Zhang X et al. *JAMA Pediatrics.*

al[1882]											
Flynn, et al[1883]	2005	no	yes	yes	yes	yes	yes	yes	yes	yes	8
Thorpe, et al[1884]	2004	yes	yes	no	yes	yes	yes	yes	yes	yes	8
Patrick, et al[1885]	2004	yes	yes	yes	yes	yes	yes	yes	yes	no	8
Nelson, et al[1886]	2004	yes	yes	yes	yes	yes	yes	yes	yes	yes	9
Mirza, et al[1887]	2004	yes	yes	yes	yes	yes	yes	yes	yes	yes	9
Hoelscher, et al[1888]	2004	yes	yes	no	yes	yes	yes	yes	yes	yes	8
Drobac, et al[1889]	2004	yes	yes	yes	yes	yes	yes	no	yes	yes	8
Davy, et al[1890]	2004	yes	yes	yes	yes	yes	yes	yes	yes	yes	9
Trost, et al[1891]	2003	yes	no	yes	no	yes	yes	yes	yes	yes	7
Jiménez-Cruz, et al[1892]	2003	yes	yes	yes	yes	yes	yes	yes	yes	yes	9
Friedlander, et al[1893]	2003	yes	yes	yes	no	yes	yes	yes	yes	yes	8
Eisenmann, et al[1894]	2003	yes	no	yes	yes	yes	no	yes	no	yes	6
Demerath, et al[1895]	2003	no	yes	yes	yes	yes	yes	yes	yes	yes	8
Rinderknecht, et al[1896]	2002	no	yes	yes	yes	yes	yes	yes	yes	yes	8
Rinderknecht, et al[1897]	2002	yes	yes	yes	yes	yes	yes	yes	no	yes	8
Yang, et al[1898]	2018	yes	yes	yes	yes	yes	yes	yes	yes	yes	9
Hidalgo-Mendez, et al[1899]	2019	yes	yes	yes	yes	yes	yes	yes	no	yes	8
Tschamler, et al[1900]	2010	yes	yes	yes	yes	yes	yes	yes	yes	yes	9
Thundiyil, et al[1901]	2010	yes	yes	yes	yes	yes	yes	yes	yes	no	8
Amram, et al[1902]	2020	yes	yes	yes	yes	yes	yes	yes	yes	no	8
Strickman-Stein,	2010	yes	yes	yes	yes	yes	yes	no	yes	yes	8

© 2024 Zhang X et al. *JAMA Pediatrics.*

et al[1903]											
Shankaran, et al[1904]	2010	yes	yes	yes	yes	yes	yes	no	yes	yes	8
Shabbir, et al[1905]	2010	yes	yes	yes	no	yes	yes	yes	yes	yes	8
Rodriguez, et al[1906]	2010	no	yes	yes	yes	yes	yes	yes	yes	yes	8
Meininger, et al[1907]	2010	yes	yes	yes	yes	yes	yes	yes	yes	yes	9
Hennessy, et al[1908]	2010	yes	yes	yes	no	yes	yes	yes	yes	yes	8
Gungor, et al[1909]	2010	yes	yes	yes	yes	yes	yes	yes	yes	no	8
Grow, et al[1910]	2010	yes	yes	no	yes	yes	yes	yes	yes	yes	8
Farhat, et al[1911]	2010	no	yes	yes	yes	yes	no	yes	yes	yes	7
Elder, et al[1912]	2010	yes	yes	yes	yes	yes	yes	yes	yes	yes	9
Dammann, et al[1913]	2010	yes	yes	yes	yes	yes	yes	yes	yes	yes	9
Bethell, et al[1914]	2010	yes	yes	yes	yes	yes	no	yes	no	yes	7
Beets, et al[1915]	2010	yes	yes	yes	no	yes	yes	yes	yes	yes	8
Bayles, et al[1916]	2010	yes	yes	yes	yes	yes	yes	yes	yes	no	8
Baranowski, et al[1917]	2010	yes	yes	yes	yes	yes	yes	yes	no	yes	8
Babey, et a[1918]	2010	no	yes	yes	yes	yes	yes	yes	yes	yes	8
Villa-Caballero, et al[1919]	2009	yes	yes	no	yes	yes	no	yes	yes	yes	7
Valente, et al[1920]	2009	yes	yes	yes	yes	yes	yes	no	yes	yes	8
Vader, et al[1921]	2009	yes	yes	yes	yes	yes	yes	yes	yes	no	8
Treuth, et al[1922]	2009	yes	yes	yes	yes	yes	no	yes	yes	yes	8
Trent, et al[1923]	2009	yes	yes	yes	yes	yes	yes	yes	yes	yes	9
Smith, et al[1924]	2009	yes	yes	yes	yes	yes	yes	yes	no	yes	8
Santos, et al[1925]	2009	yes	yes	yes	yes	no	yes	yes	yes	yes	8
Rundle, et al[1926]	2009	yes	yes	yes	yes	no	yes	yes	yes	yes	8

© 2024 Zhang X et al. *JAMA Pediatrics.*

Raynor, et al[1927]	2009	yes	yes	yes	yes	yes	no	yes	yes	no	7
Nsiah-Kumi, et al[1928]	2009	yes	yes	yes	yes	no	yes	yes	yes	yes	8
Montgomery-Reagan, et al[1929]	2009	yes	yes	yes	yes	yes	yes	yes	no	yes	8
Martyn-Nemeth, et al[1930]	2009	yes	yes	yes	yes	yes	yes	yes	yes	yes	9
Lim, et al[1931]	2009	yes	yes	yes	yes	yes	yes	yes	no	yes	8
Kubik, et al[1932]	2009	yes	yes	yes	yes	no	yes	yes	yes	yes	8
Krukowski, et al[1933]	2009	yes	yes	yes	yes	yes	yes	yes	yes	no	8
Hillman, et al[1934]	2009	yes	yes	yes	yes	no	yes	yes	yes	yes	8
Harnack, et al[1935]	2009	yes	yes	yes	yes	yes	no	yes	yes	yes	8
Harbaugh, et al[1936]	2009	yes	yes	yes	no	no	yes	yes	yes	yes	7
Franzini, et al[1937]	2009	yes	yes	yes	yes	yes	yes	yes	yes	no	8
Forman, et al[1938]	2009	yes	yes	yes	yes	yes	no	yes	yes	yes	8
Duncan, et al[1939]	2009	yes	yes	yes	yes	yes	yes	yes	no	yes	8
Dorsey, et al[1940]	2009	yes	yes	yes	yes	no	yes	yes	no	yes	7
Dixon, et al[1941]	2009	yes	yes	yes	no	yes	yes	yes	yes	yes	8
De La O, et al[1942]	2009	yes	yes	yes	yes	yes	no	yes	no	yes	7
Chen, et al[1943]	2009	yes	yes	yes	yes	yes	yes	yes	yes	no	8
Asante, et al[1944]	2009	no	yes	yes	yes	yes	yes	yes	yes	yes	8
Anderson, et al[1945]	2009	yes	yes	yes	yes	yes	yes	yes	no	yes	8
West, et al[1946]	2008	no	yes	yes	yes	yes	yes	yes	yes	yes	8
Stovitz, et al[1947]	2008	yes	yes	yes	yes	yes	yes	yes	yes	no	8
Steele, et al[1948]	2008	yes	yes	yes	yes	yes	yes	yes	yes	no	8
Pollack, et	2008	yes	yes	no	yes	yes	yes	yes	yes	yes	8

© 2024 Zhang X et al. *JAMA Pediatrics.*

al[1949]										
Margellos-Anast, et al[1950]	2008	yes	yes	yes	yes	no	yes	yes	yes	yes	8
Laurson, et al[1951]	2008	yes	yes	yes	no	no	yes	yes	yes	yes	7
Kapoor, et al[1952]	2008	yes	yes	yes	yes	yes	no	yes	yes	yes	8
Kalich, et al[1953]	2008	yes	yes	no	yes	yes	yes	yes	yes	yes	8
Irigoyen, et al[1954]	2008	yes	yes	yes	yes	yes	yes	yes	no	yes	8
Eisenmann, et al[1955]	2008	yes	yes	no	yes	yes	yes	yes	yes	yes	8
Eichner, et al[1956]	2008	yes	yes	yes	yes	no	yes	yes	yes	yes	8
Wald, et al[1957]	2007	yes	yes	yes	yes	yes	yes	no	no	yes	7
Roseman, et al[1958]	2007	yes	yes	yes	yes	yes	yes	yes	yes	no	8
Metallinos-Katsaras, et al[1959]	2007	yes	yes	yes	yes	yes	no	yes	yes	yes	8
Martin, et al[1960]	2007	yes	yes	yes	yes	yes	yes	yes	yes	yes	9
Malaty, et al[1961]	2007	yes	yes	yes	yes	yes	no	yes	yes	yes	8
Lynch, et al[1962]	2007	no	yes	yes	yes	yes	yes	yes	yes	yes	8
Hernández-Valero, et al[1963]	2007	yes	yes	yes	yes	yes	no	yes	yes	yes	8
Eisenmann, et al[1964]	2007	yes	yes	yes	yes	yes	no	yes	yes	yes	8
Akridge, et al[1965]	2007	yes	yes	yes	yes	yes	no	yes	yes	yes	8
Zephier, et al[1966]	2006	no	yes	yes	yes	no	yes	yes	yes	yes	7
Nelson, et al[1967]	2006	yes	yes	yes	yes	yes	yes	no	yes	yes	8
Pobutsky, et al[1968]	2006	yes	yes	yes	no	yes	yes	yes	yes	yes	8
Mulvaney, et al[1969]	2006	yes	yes	yes	yes	yes	yes	yes	yes	no	8
Trappmann, et	2015	yes	yes	yes	no	yes	yes	yes	yes	yes	8

© 2024 Zhang X et al. *JAMA Pediatrics.*

al[1970]											
Trapp, et al[1971]	2015	no	yes	yes	yes	yes	yes	no	yes	yes	7
Tomayko, et al[1972]	2015	yes	yes	no	yes	yes	yes	yes	yes	yes	8
Scherrer, et al[1973]	2015	yes	yes	yes	yes	yes	yes	yes	no	yes	8
Park, et al[1974]	2015	yes	yes	yes	yes	yes	yes	yes	yes	no	8
Nobari, et al[1975]	2015	yes	yes	yes	yes	yes	yes	no	yes	yes	8
Nagata, et al[1976]	2015	yes	yes	no	yes	yes	yes	yes	no	yes	7
Moreno, et al[1977]	2015	no	yes	yes	yes	yes	yes	yes	yes	yes	8
Lumeng, et al[1978]	2015	yes	yes	yes	yes	yes	yes	yes	yes	no	8
Li, et al[1979]	2015	yes	yes	yes	yes	yes	no	no	yes	yes	7
Jin, et al[1980]	2015	yes	yes	yes	yes	yes	yes	yes	yes	yes	9
Jackson, et al[1981]	2015	yes	yes	no	yes	yes	yes	yes	yes	yes	8
Gunter, et al[1982]	2015	yes	yes	yes	yes	yes	yes	no	yes	yes	8
Ansari, et al[1983]	2015	yes	yes	yes	yes	yes	yes	yes	yes	yes	9
Weedn, et al[1984]	2014	yes	yes	yes	yes	yes	yes	no	yes	yes	8
Nichols, et al[1985]	2014	yes	yes	yes	yes	yes	yes	no	yes	yes	8
Nguyen, et al[1986]	2014	yes	yes	no	yes	yes	yes	yes	yes	yes	8
Lo, et al[1987]	2014	yes	yes	yes	yes	yes	yes	yes	yes	yes	9
Cui, et al[1988]	2014	yes	yes	yes	yes	yes	yes	yes	yes	yes	9
Dammann, et al[1989]	2011	yes	yes	yes	yes	yes	yes	no	yes	yes	8
Florin, et al[1990]	2011	yes	yes	yes	yes	yes	yes	no	yes	no	7
Hill, et al[1991]	2011	yes	yes	yes	yes	yes	no	yes	yes	yes	8
Isasi, et al[1992]	2011	yes	yes	no	yes	yes	yes	yes	yes	yes	8
Mulasi-Pokhriyal, et al[1993]	2011	yes	yes	yes	no	yes	yes	yes	yes	yes	8
Nervik, et al[1994]	2011	yes	yes	yes	yes	yes	yes	no	yes	yes	8
Pérez, et al[1995]	2011	yes	yes	yes	yes	yes	yes	yes	no	no	7

© 2024 Zhang X et al. *JAMA Pediatrics*.

Spruyt, et al[1996]	2011	yes	yes	yes	yes	yes	yes	yes	yes	no	8
Xanthopoulos, et al[1997]	2011	yes	yes	yes	no	yes	yes	yes	yes	yes	8
Gamble, et al[1998]	2012	yes	yes	yes	yes	yes	yes	yes	yes	no	8
Weedn, et al[1999]	2012	yes	yes	yes	yes	yes	yes	yes	yes	no	8
Dodd, et al[2000]	2013	yes	yes	yes	yes	yes	yes	yes	no	yes	8
Hayes, et al[2001]	2013	yes	yes	yes	no	yes	yes	yes	yes	yes	8
Loth, et al[2002]	2013	yes	yes	yes	yes	yes	yes	yes	no	yes	8
Novotny, et al[2003]	2013	yes	yes	yes	yes	no	yes	no	yes	yes	7
Nunez-Gaunaurd, et al[2004]	2013	yes	yes	yes	yes	yes	yes	yes	no	yes	8
Pan, et al[2005]	2013	yes	yes	yes	yes	yes	no	yes	yes	yes	8
Novotny, et al[2006]	2016	no	yes	yes	yes	yes	yes	yes	yes	yes	8
Stiefel, et al[2007]	2016	no	yes	yes	yes	yes	yes	yes	yes	yes	8
Zeller, et al[2008]	2016	yes	yes	yes	no	yes	yes	yes	yes	yes	8
Novotny, et al[2009]	2017	yes	yes	yes	yes	yes	no	yes	no	yes	7
Sadeghi, et al[2010]	2017	yes	yes	no	yes	yes	yes	yes	yes	yes	8
DuBose, et al[2011]	2018	yes	yes	yes	yes	yes	no	yes	yes	yes	8
Beal, et al[2012]	2020	yes	yes	yes	no	yes	yes	yes	yes	yes	8
Le, et al[2013]	2022	yes	yes	yes	yes	yes	yes	no	yes	yes	8
Nguyen, et al[2014]	2021	yes	yes	yes	yes	yes	yes	yes	yes	yes	9
Dieu, et al[2015]	2007	yes	yes	yes	yes	yes	yes	yes	yes	yes	9
Phan, et al[2016]	2020	yes	yes	yes	yes	yes	no	yes	yes	yes	8
Chuc, et al[2017]	2019	yes	yes	yes	yes	no	yes	yes	yes	yes	8
Huynh, et al[2018]	2019	yes	yes	yes	yes	yes	yes	no	yes	no	7
Pham, et al[2019]	2019	yes	yes	yes	yes	yes	yes	yes	yes	no	8

© 2024 Zhang X et al. *JAMA Pediatrics.*

Trang, et al[2020]	2010	yes	yes	yes	yes	yes	yes	no	yes	yes	8
Mai, et al[2021]	2020	yes	yes	yes	yes	yes	yes	yes	yes	no	8
Pham, et al[2022]	2020	yes	yes	yes	yes	no	yes	yes	yes	yes	8
Nguyen, et al[2023]	2010	yes	yes	yes	yes	yes	yes	yes	yes	no	8
Dieu, et al[2024]	2009	yes	yes	yes	no	yes	yes	yes	no	yes	7
Tang, et al[2025]	2007	yes	yes	yes	yes	yes	yes	no	yes	yes	8
Hong, et al[2026]	2007	yes	yes	yes	yes	yes	yes	yes	yes	yes	9
Trang, et al[2027]	2012	yes	yes	yes	yes	yes	yes	no	yes	yes	8
Nguyen, et al[2028]	2013	yes	yes	yes	no	no	yes	yes	yes	no	6
Hoang, et al[2029]	2018	yes	yes	yes	yes	yes	yes	no	yes	yes	8
Ngan, et al[2030]	2018	yes	yes	yes	yes	yes	yes	no	yes	yes	8
Badi, et al[2031]	2012	yes	yes	yes	yes	yes	yes	yes	yes	yes	9
Raja'a, et al[2032]	2005	yes	yes	no	no	yes	yes	yes	yes	yes	7
Kambondo, et al[2033]	2018	yes	no	yes	yes	yes	yes	yes	yes	yes	8

eTable 3. Characteristics of the studies for prevalence of obesity in children and adolescents.

Study	Country or Region	Publication Year	Study Period	Study Design	Sample Source	Diagnostic Reference	No. of Obesity	Sample Size
Salas, et al (1)	Albania	2021	2015-2017	cross-sectional	database	WHO	224	2259
Hyska, et al	Albania	2014	2013	cross-sectional	school	WHO	447	5810
Benmohammed, et al	Algeria	2020	2007	cross-sectional	school	IOTF	51	1100
Musaiger, et al (1)	Algeria	2012	2010-2011	cross-sectional	school	IOTF	20	459
Fedala, et al	Algeria	2017	2013-2014	cross-sectional	school	IOTF	48	2278
Orden, et al	Argentina	2019	2015-2016	cross-sectional	school	IOTF	166	1366
Meyer, et al	Argentina	2013	2010-2011	cross-sectional	database	IOTF	1351	15541
Moraes, et al (2)	Argentina	2014	2008	cross-sectional	school	IOTF	26	933
Hirschler, et al	Argentina	2010	2005	cross-sectional	school	CDC	220	1564
Stray-Pedersen, et al	Argentina	2009	2004-2005	cross-sectional	database	IOTF	29	669
Hirschler, et al	Argentina	2008	2006	cross-sectional	school	CDC	95	621
Hirschler, et al	Argentina	2008	2006-2007	cross-sectional	community	CDC	164	1027
Hirschler, et al	Argentina	2006	2004	cross-sectional	school	CDC	59	321

© 2024 Zhang X et al. *JAMA Pediatrics*.

Kovalskys, et al	Argentina	2011	2005	cross-sectional	school	CDC	184	1588
Tringler, et al	Argentina	2012	2007-2008	cross-sectional	database	CDC	31	334
Catalani, et al	Argentina	2016	2014	cross-sectional	school	National Reference	100	711
Gotthelf, et al	Argentina	2017	2015	cross-sectional	school	WHO	52	283
Rivero, et al	Argentina	2018	2017	cross-sectional	community	WHO	39	303
Abbott, et al	Australia	2010	2006	cross-sectional	database	IOTF	143	3043
Crawford, et al	Australia	2008	2004	cross-sectional	community	IOTF	24	380
Franklin, et al	Australia	2006	2006	cross-sectional	community	CDC	226	2743
Wake, et al	Australia	2013	2000-2006	cross-sectional	community	IOTF	837	16339
James, et al	Australia	2013	2001-2005	cross-sectional	community	IOTF	747	17008
Marshall, et al	Australia	2012	2005	cross-sectional	community	IOTF	40	691
Martin, et al	Australia	2012	2005	cross-sectional	community	IOTF	21	408
O'Dea, et al (1)	Australia	2010	2000	cross-sectional	community	IOTF	197	3819
O'Dea, et al (2)	Australia	2010	2006	cross-sectional	community	IOTF	336	5524
O'Dea, et al	Australia	2014	2007	longitudinal	school	IOTF	38	939
Schofield, et al	Australia	2009	2002	cross-sectional	school	IOTF	25	415
Gopinath, et al	Australia	2012	2003-2005	cross-sectional	school	IOTF	296	4094
Spurrier, et al	Australia	2012	2009	cross-sectional	school	IOTF	517	11859
Trapp, et al	Australia	2011	2007	cross-sectional	database	IOTF	37	1197
Waters, et al	Australia	2008	2004-2005	cross-sectional	school	IOTF	244	2685
Bell, et al	Australia	2018	2013-2014	cohort	database	WHO	24	953
Maher, et al	Australia	2012	2012	cross-sectional	database	IOTF	143	2200
Hayes, et al	Australia	2021	2004	cohort	database	WHO	813	9225
Miller, et al	Australia	2014	2005-2010	cross-sectional	community	CDC	107	1850
Achat, et al	Australia	2014	2007	cross-sectional	school	IOTF	136	2341
O'Dea, et al	Australia	2008	2006	cross-sectional	school	IOTF	500	7889
O'Sullivan, et al	Australia	2015	2003-2005	cross-sectional	database	IOTF	102	1416
Barnes, et al	Australia	2021	2017	RCT	school	WHO	61	815
Magee, et al	Australia	2013	2006	longitudinal	database	IOTF	334	1833
Cox, et al	Australia	2012	2010	cross-sectional	community	CDC	5	135
Wickramasinghe, et al	Australia	2005	2005	cross-sectional	community	CDC	8	138
Telford, et al	Australia	2008	2007	cross-sectional	database	CDC	85	741
Seach, et al	Australia	2010	2000-2004	cohort	medical institution	IOTF	8	307
Jansen, et al	Australia	2013	2010	cross-sectional	database	IOTF	208	3197
Tai, et al	Australia	2009	2006	cross-sectional	school	IOTF	82	1457
Bergmeier, et al	Australia	2014	2009-2011	longitudinal	community	CDC	6	201
Hardy, et al	Australia	2012	2010	cross-sectional	database	IOTF	56	1141
White, et al (2)	Australia	2022	2022	cohort	database	IOTF	265	3998
Rehor, et al	Australia	2002	2001	cross-sectional	school	IOTF	19	329
Wen, et al	Australia	2010	2006	cross-sectional	school	IOTF	31	964
Shi, et al	Australia	2010	2004-2008	cross-sectional	database	IOTF	260	3495
Haysom, et al	Australia	2009	2003-2004	longitudinal	school	National Reference	145	2266
Haug, et al (2)	Australia	2009	2005-2006	cross-sectional	database	IOTF	95	4509
Sutherland, et al	Australia	2008	2004	cross-sectional	school	IOTF	201	2224
Denney-Wilson, et al	Australia	2008	2004	cross-sectional	database	IOTF	28	496
Fisher, er al	Australia	2006	2002	cross-sectional	school	CDC	13	296
Cretikos, et	Australia	2008	2002-2006	cross-sectional	database	IOTF	1473	12925

© 2024 Zhang X et al. *JAMA Pediatrics*.

al								
Crawford, et al	Australia	2006	2001	cross-sectional	school	IOTF	22	1141
Campbell, et al	Australia	2006	2002	cross-sectional	database	IOTF	9	324
Burke, et al	Australia	2006	2006	cross-sectional	school	IOTF	27	570
Sanigorski, et al	Australia	2007	2003-2004	cross-sectional	community	IOTF	166	2184
Katzmarzyk, et al (2)	Australia	2015	2011-2013	cross-sectional	database	WHO	50	491
Cochrane, et al	Australia	2015	2000-2011	cross-sectional	school	IOTF	2083	31424
O'Dea, et al (1)	Australia	2014	2006	cross-sectional	database	IOTF	576	8702
O'Dea, et al (2)	Australia	2014	2012	cross-sectional	database	IOTF	805	12587
Chen, et al (1)	Australia	2014	2010-2011	cross-sectional	community	IOTF	2	89
Keating, et al	Australia	2011	2005-2006	cross-sectional	database	IOTF	181	2890
Olds, et al	Australia	2011	2007	cross-sectional	database	IOTF	143	2200
Morley, et al	Australia	2012	2009-2010	cross-sectional	school	IOTF	609	12188
Schultz, et al	Australia	2012	2012	cross-sectional	database	WHO	54	996
Fredrickson, et al	Australia	2013	2005-2008	cross-sectional	database	WHO	278	2954
Furthner, et al	Australia	2017	2012-2013	cross-sectional	school	National Reference	164	2916
Furthner, et al	Austria	2018	2012-2013	cross-sectional	school	National Reference	164	2930
Yngve, et al (1)	Austria	2008	2003	cross-sectional	database	IOTF	28	1181
Janssen, et al (1)	Austria	2005	2001-2002	cross-sectional	database	IOTF	76	3994
Pfeiffer, et al	Austria	2006	2006	cross-sectional	school	National Reference	47	835
Romano, et al (1)	Bahamas	2022	2013	cross-sectional	database	WHO	279	1308
Al-Raees, et al	Bahrain	2009	2009	cross-sectional	community	WHO	45	698
Al-Sendi, et al	Bahrain	2003	2000	cross-sectional	school	IOTF	83	506
Musaiger, et al	Bahrain	2014	2003-2005	cross-sectional	school	IOTF	167	2146
Anam, et al	Bangladesh	2022	2019	cross-sectional	school	WHO	74	1044
Taher, et al	Bangladesh	2021	2016-2018	cross-sectional	school	CDC	270	1450
Sultana, et al	Bangladesh	2015	2015	cross-sectional	school	National Reference	12	150
Sultana, et al	Bangladesh	2016	2016	cross-sectional	school	WHO	115	500
Saha, et al	Bangladesh	2018	2014	cross-sectional	school	CDC	16	288
Romano, et al (2)	Bangladesh	2022	2014	cross-sectional	database	WHO	36	2753
Sultana, et al	Bangladesh	2019	2012-2013	cross-sectional	school	IOTF	84	1768
Bulbul, et al	Bangladesh	2014	2009	cross-sectional	school	WHO	359	10135
Romano, et al (3)	Barbados	2022	2011	cross-sectional	database	WHO	214	1504
Brug, et al (1)	Belgium	2012	2010	cross-sectional	database	IOTF	30	1003
Ahrens, et al (4)	Belgium	2014	2007-2008	cross-sectional	database	IOTF	55	2352
Usheva, et al (1)	Belgium	2021	2012	cross-sectional	database	WHO	14	1128
Júlíusson, et al (1)	Belgium	2015	2002-2006	cross-sectional	database	IOTF	334	12200
Yngve, et al (2)	Belgium	2008	2003	cross-sectional	database	IOTF	13	965
Seghers, et al	Belgium	2010	2006-2007	cross-sectional	school	IOTF	18	798
Vriendt, et al	Belgium	2009	2004-2005	cross-sectional	school	IOTF	20	982
Janssen, et	Belgium	2005	2001-2002	cross-sectional	database	IOTF	14	5876

© 2024 Zhang X et al. *JAMA Pediatrics*.

al (2)								
Janssen, et al (3)	Belgium	2005	2001-2002	cross-sectional	database	IOTF	52	3066
Gysel, et al	Belgium	2009	2004-2005	cross-sectional	database	IOTF	116	1576
Haug, et al (3)	Belgium	2009	2005-2006	cross-sectional	database	IOTF	59	3966
Haug, et al (4)	Belgium	2009	2005-2006	cross-sectional	database	IOTF	78	3267
Vissers, et al	Belgium	2008	2008	cross-sectional	school	IOTF	30	994
Huybrechts, et al	Belgium	2006	2006	cross-sectional	school	IOTF	7	297
Velde, et al (1)	Belgium	2017	2010	cross-sectional	database	IOTF	30	996
Manyanga, et al (1)	Benin	2014	2006/2010	cross-sectional	database	WHO	16	2681
Romano, et al (4)	Benin	2022	2016	cross-sectional	database	WHO	17	717
Norbu, et al	Bhutan	2019	2019	cross-sectional	school	CDC	2	392
Botti, et al	Bolivia	2010	2007	cross-sectional	database	WHO	298	3306
Romano, et al (5)	Bolivia	2022	2012	cross-sectional	database	WHO	132	2804
Pe ́rez-Cueto, et al	Bolivia	2005	2003	cross-sectional	school	IOTF	12	525
Benefice, et al	Bolivia	2007	2004-2005	cross-sectional	community	IOTF	4	385
Spahić, et al	Bosnia and Herzegovina	2019	2016-2017	cross-sectional	school	CDC	371	2500
Hansanbegović, et al	Bosnia and Herzegovina	2010	2008-2009	cross-sectional	school	CDC	248	3608
Wrotniak, et al	Botswana	2012	2012	cross-sectional	school	WHO	35	707
Alexius, et al	Brazil	2012	2007	cross-sectional	community	IOTF	40	1048
Andaki, et al (1)	Brazil	2017	2009-2011	cross-sectional	community	IOTF	109	2423
Ataíde Lima, et al	Brazil	2015	2008-2010	cross-sectional	community	WHO	24	203
Castilho, et al	Brazil	2014	2010-2012	cross-sectional	community	WHO	435	3130
Costa, et al	Brazil	2015	2012-2013	cross-sectional	community	WHO	205	1530
Ferreira, et al	Brazil	2015	2012-2013	cross-sectional	community	WHO	179	1338
Fraiz, et al	Brazil	2019	2019	cross-sectional	community	WHO	75	686
Justo, et al	Brazil	2012	2009-2010	cross-sectional	community	WHO	45	901
Guedes, et al	Brazil	2011	2007	cross-sectional	community	WHO	110	5100
Kupek, et al	Brazil	2016	2007	cross-sectional	community	WHO	129	1232
Moreira, et al	Brazil	2012	2007	cross-sectional	community	WHO	20	963
Oppitz, et al	Brazil	2014	2008	cross-sectional	community	WHO	28	1640
Sliva, et al	Brazil	2018	2012-2013	cross-sectional	community	National Reference	169	1125
Vale, et al	Brazil	2022	2015	cross-sectional	database	WHO	1119	9400
Pereira, et al	Brazil	2023	2018-2019	cross-sectional	database	WHO	184	1060
Santos, et al	Brazil	2022	2012-2013	cross-sectional	school	WHO	69	402
Coelho, et al	Brazil	2022	2019	cross-sectional	community	WHO	25	170
Blumenberg, et al	Brazil	2021	2015	longitudinal	community	WHO	108	874
Barbiero, et al	Brazil	2009	2009	cross-sectional	school	WHO	50	511
Barbosa, et al	Brazil	2021	2021	cross-sectional	school	WHO	26	353
Cândido, et al	Brazil	2009	2006	cross-sectional	school	CDC	52	779
Polderman, et al	Brazil	2011	2008	cross-sectional	school	IOTF	37	1002
Novaes, et al	Brazil	2013	2013	cross-sectional	school	WHO	82	769
Duncan, et	Brazil	2011	2011	cross-sectional	school	IOTF	219	3397

© 2024 Zhang X et al. *JAMA Pediatrics*.

al								
Halal, et al	Brazil	2016	2008	longitudinal	community	WHO	201	4231
Goldani, et al	Brazil	2013	2004-2005	cohort	medical institution	IOTF	119	1463
Caixeta, et al	Brazil	2020	2016-2017	cross-sectional	school	CDC	44	486
Ferreira, et al	Brazil	2008	2003	cross-sectional	school	IOTF	11	412
Rodrigues, et al	Brazil	2006	2003-2005	cross-sectional	school	IOTF	21	380
Amorim, et al	Brazil	2006	2003	cross-sectional	school	IOTF	23	1719
Araújo, et al	Brazil	2010	2004-2005	cohort	medical institution	WHO	516	4452
Flores, et al	Brazil	2013	2005-2006	cross-sectional	database	National Reference	990	20514
Bispo, et al	Brazil	2015	2008-2009	cross-sectional	database	WHO	83	1030
Castro, et al	Brazil	2012	2008	cross-sectional	school	CDC	121	1786
Moraes, et al (1)	Brazil	2014	2008	cross-sectional	school	IOTF	57	991
Santana, et al	Brazil	2013	2009	cross-sectional	school	WHO	89	1494
Lock, et al	Brazil	2020	2009-2010	cross-sectional	school	WHO	207	1528
Cruz, et al	Brazil	2013	2010-2011	cross-sectional	school	IOTF	26	523
Moraes, et al	Brazil	2019	2010	longitudinal	database	WHO	125	673
Guedes, et al	Brazil	2013	2011	cross-sectional	school	IOTF	66	1968
Rosini, et al	Brazil	2015	2009	cross-sectional	school	WHO	133	1011
Neves, et al	Brazil	2015	2012	cross-sectional	school	WHO	51	411
Costa, et al	Brazil	2020	2020	cross-sectional	school	WHO	135	1334
Vieira, et al	Brazil	2015	2013	cross-sectional	school	WHO	29	347
Araujo, et al	Brazil	2018	2014	cross-sectional	school	WHO	132	1182
Carmo, et al	Brazil	2018	2014-2016	cross-sectional	school	WHO	18	405
Fradkin, et al	Brazil	2018	2014-2016	cross-sectional	school	CDC	131	1738
Reuter, et al	Brazil	2018	2014-2015	cross-sectional	school	WHO	134	1200
Ripka, et al	Brazil	2017	2015-2016	cross-sectional	school	WHO	21	374
Silva, et al	Brazil	2020	2015	cohort	database	WHO	112	862
Schwertner, et al	Brazil	2020	2020	cross-sectional	school	IOTF	18	330
Dantas, et al	Brazil	2018	2017	cross-sectional	school	IOTF	47	578
Assis, et al	Brazil	2005	2002	cross-sectional	school	IOTF	162	2936
Salas, et al	Brazil	2018	2010	cross-sectional	school	IOTF	160	1211
Assis, et al	Brazil	2019	2011-2012	cross-sectional	school	WHO	92	661
Dalmaso, et al	Brazil	2019	2019	cross-sectional	community	IOTF	41	572
Lima, et al	Brazil	2019	2019	cross-sectional	school	National Reference	56	1169
Porcelli, et al	Brazil	2019	2012	cross-sectional	medical institution	WHO	9	191
Alves, et al	Brazil	2020	2013-2014	cross-sectional	database	WHO	5506	71298
Folmann, et al	Brazil	2020	2013-2014	cross-sectional	school	WHO	298	1715
Hércules, et al	Brazil	2020	2016-2017	cross-sectional	database	WHO	12630	80782
Lucena, et al	Brazil	2020	2020	cross-sectional	community	WHO	164	1487
Rocha, et al	Brazil	2020	2017	cross-sectional	database	WHO	148	2059
Rivera, et al	Brazil	2010	2001	cross-sectional	school	National Reference	56	1253
Gabriel, et al	Brazil	2010	2007-2008	cross-sectional	school	IOTF	300	4964
Anwar, et al	Brazil	2010	2007	cross-sectional	school	CDC	167	988
Molina, et al	Brazil	2009	2007	cross-sectional	school	IOTF	115	1251
Fernandes, et al	Brazil	2009	2007	cross-sectional	school	IOTF	109	1779
Pelegrini, et al	Brazil	2008	2004-2005	cross-sectional	database	IOTF	961	36976
Bertolace, et al	Brazil	2008	2003	cross-sectional	school	CDC	37	421

© 2024 Zhang X et al. *JAMA Pediatrics.*

Oliveira, et al	Brazil	2007	2007	cross-sectional	school	IOTF	31	699
da Silva, et al	Brazil	2007	2002	cross-sectional	community	National Reference	27	471
Granville-Garcia, et al	Brazil	2006	2006	cross-sectional	school	WHO	240	2651
Katzmarzyk, et al (3)	Brazil	2015	2011-2013	cross-sectional	database	WHO	108	493
Weinmayr, et al (1)	Brazil	2014	2004	cross-sectional	database	IOTF	105	953
Müller, et al	Brazil	2014	2008	cross-sectional	community	WHO	741	6360
Moreira, et al	Brazil	2014	2005-2006	cross-sectional	community	WHO	87	1115
Mendonça, et al	Brazil	2014	2010-2011	cross-sectional	school	WHO	87	1168
Crispim, et al	Brazil	2014	2011-2012	cross-sectional	school	WHO	31	276
Menezes, et al	Brazil	2011	2006	cross-sectional	database	WHO	77	940
Nascimento, et al	Brazil	2012	2009	cross-sectional	medical institution	WHO	18	447
Novaes, et al	Brazil	2012	2005-2006	cross-sectional	school	WHO	82	764
Rosaneli, et al	Brazil	2012	2006	cross-sectional	school	WHO	353	5037
Reuter, et al	Brazil	2013	2013	cross-sectional	school	CDC	69	564
Andrade, et al	Brazil	2016	2016	cross-sectional	school	WHO	77	396
Casonatto, et al	Brazil	2016	2002-2005	cross-sectional	database	IOTF	81	978
Jardim-Botelho, et al	Brazil	2016	2009-2012	cross-sectional	community	WHO	11	153
Silva, et al	Brazil	2016	2004	cross-sectional	school	WHO	241	2180
Araujo, et al	Brazil	2017	2017	cross-sectional	community	WHO	23	548
Cuesta, et al	Brazil	2018	2013	cross-sectional	community	WHO	239	1296
Dos Santos, et al	Brazil	2018	2018	cross-sectional	school	IOTF	44	501
Pivatto, et al	Brazil	2018	2016	cross-sectional	school	WHO	61	236
Romano, et al (6)	Brunei Darussalam	2022	2014	cross-sectional	database	WHO	319	1824
Usheva, et al (2)	Bulgaria	2021	2012	cross-sectional	database	WHO	29	874
Salas, et al (2)	Bulgaria	2021	2015-2017	cross-sectional	database	WHO	440	3238
Mladenova, et al	Bulgaria	2015	2012-2014	cross-sectional	school	IOTF	49	878
Gorog, et al (1)	Bulgaria	2011	2011	cross-sectional	database	IOTF	75	1554
Haug, et al (19)	Bulgaria	2009	2005-2006	cross-sectional	database	IOTF	87	4563
Wijnhoven, et al (1)	Bulgaria	2015	2007/2008	cross-sectional	school	WHO	450	3627
Mank, et al	Burkina Faso	2022	2020	cross-sectional	school	WHO	53	1059
Daboné, et al	Burkina Faso	2011	2008-2009	cross-sectional	school	WHO	4	649
Gebremedhin, et al (2)	Burkina Faso	2015	2010	cross-sectional	database	WHO	128	6723
Gebremedhin, et al (1)	Burundi	2015	2010	cross-sectional	database	WHO	52	3493
Navti, et al (1)	Cameroon	2021	2010	cross-sectional	school	WHO	32	1274
Navti, et al (2)	Cameroon	2021	2020	cross-sectional	school	WHO	46	1550
Gebremedhin, et al (3)	Cameroon	2015	2011	cross-sectional	database	WHO	145	5185
Choukem, et al	Cameroon	2017	2013	cross-sectional	school	WHO	39	1343
Chelo, et al	Cameroon	2019	2017-2018	cross-sectional	school	National Reference	5	822

© 2024 Zhang X et al. *JAMA Pediatrics.*

Wamba, et al	Cameroon	2013	2010	cross-sectional	database	IOTF	51	2689
Navti ,et al	Cameroon	2017	2017	cross-sectional	school	WHO	16	522
Hulst, et al	Canada	2022	2001	longitudinal	database	WHO	153	1226
Anderson, et al	Canada	2022	2013-2019	cross-sectional	database	WHO	217	5962
Le, et al	Canada	2016	2011	cross-sectional	database	WHO	160	1331
Seliske, et al	Canada	2009	2005-2006	cross-sectional	database	IOTF	458	7987
Ball, et al	Canada	2019	2010/2017	cross-sectional	database	WHO	10323	161114
Chaput, et al	Canada	2006	2003	cross-sectional	school	IOTF	29	422
Chaput, et al	Canada	2011	2005-2008	cohort	database	CDC	122	550
Davidson, et al	Canada	2016	2009-2011	cross-sectional	community	CDC	40	235
Brault, et al	Canada	2015	2010	cross-sectional	school	IOTF	33	786
Larsen, et al	Canada	2015	2010-2011	cross-sectional	database	IOTF	103	943
Bridgman, et al	Canada	2018	2018	cohort	database	WHO	41	885
Cassidy-Bushrow, et al	Canada	2018	2003-2007	cohort	database	CDC	45	527
Sheilds, et al	Canada	2010	2004	cross-sectional	database	CDC	1083	8661
Medehouenou, et al	Canada	2015	2005-2010	cross-sectional	database	CDC	32	290
Wang, et al	Canada	2008	2003	cross-sectional	database	IOTF	490	4945
Shi, et al	Canada	2013	2007-2009	cross-sectional	database	WHO	121	968
Oliver, et al	Canada	2005	2000-2001	cross-sectional	database	IOTF	360100	3190300
MOFFAT, et al	Canada	2005	2002-2004	cross-sectional	school	CDC	23	266
Woodruff, et al	Canada	2010	2005-2006	cross-sectional	school	IOTF	22	1293
Twells, et al	Canada	2010	2005	cross-sectional	school	CDC	170	1026
Simen-Kapeu, et al	Canada	2010	2008-2010	cross-sectional	school	IOTF	236	3421
Leatherdale, et al	Canada	2010	2007	cross-sectional	school	CDC	122	1264
Khaili, et al	Canada	2010	2005-2007	cross-sectional	community	CDC	59	125
Ismailov, et al	Canada	2010	2005-2006	cross-sectional	school	CDC	1601	25416
Galloway, et al	Canada	2010	2007-2008	cross-sectional	community	CDC	191	376
Doan, et al	Canada	2010	2005	cross-sectional	community	IOTF	599	12170
Cairney, et al	Canada	2010	2005-2007	longitudinal	school	IOTF	210	2278
Bengoechera, et al	Canada	2010	2010	cross-sectional	database	IOTF	297	3159
Wahi, et al	Canada	2009	2009	cross-sectional	community	CDC	10	30
Vance, et al	Canada	2009	2002-2003	cross-sectional	school	IOTF	56	1917
Potestio, et al	Canada	2009	2005-2006	cross-sectional	school	IOTF	305	6772
Downs, et al	Canada	2009	2004-2005	cross-sectional	community	IOTF	69	201
Salvadori, et al	Canada	2008	2004	prospective	database	CDC	77	675
Edwards, et al	Canada	2008	2004-2005	cross-sectional	medical institution	CDC	839	7369
Bruner, et al	Canada	2008	2001-2002	cross-sectional	database	IOTF	233	4851
Willows, et al	Canada	2007	2002	cross-sectional	school	IOTF	222	1044
He, et al	Canada	2007	2001-2003	cross-sectional	school	CDC	45	335
Bassett, et al	Canada	2007	2005	cross-sectional	school	IOTF	2	139
Ng, et al	Canada	2006	2004	cross-sectional	school	IOTF	27	82
Janssen, et al	Canada	2006	2001	cross-sectional	database	IOTF	281	6684
Galloway, et al	Canada	2006	2004	cross-sectional	school	CDC	55	487
Rossiter, et	Canada	2015	2011	cross-sectional	database	IOTF	599	5560

© 2024 Zhang X et al. *JAMA Pediatrics.*

al								
Katzmarzyk, et al (4)	Canada	2015	2011-2013	cross-sectional	database	WHO	63	522
Borghese, et al	Canada	2015	2012-2013	cross-sectional	school	CDC	60	550
Banerjee, et al	Canada	2015	2010	cross-sectional	database	IOTF	57	734
Carson, et al	Canada	2014	2010-2011	cross-sectional	school	IOTF	53	787
Leatherdale, et al	Canada	2013	2007-2008	cross-sectional	database	IOTF	215	2326
Agüero, et al	Chile	2016	2014	cross-sectional	school	National Reference	548	1810
Olivares, et al	Chile	2004	2004	cross-sectional	school	CDC	264	1701
Cediel, et al	Chile	2016	2009-2010	cohort	database	WHO	72	435
Mardones, et al	Chile	2008	2005	cross-sectional	school	CDC	2749	153536
Delgado-Floody, et al	Chile	2019	2019	cross-sectional	school	CDC	129	605
Corvalán, et al	Chile	2010	2006	cross-sectional	school	WHO	43	324
Valenzuela, et al	Chile	2015	2010-2011	cross-sectional	school	CDC	161	1477
Lizana, et al	Chile	2015	2013	cross-sectional	school	CDC	53	206
Cadenas-Sánchez, et al	Chile	2015	2013	cross-sectional	school	WHO	99	434
Heitzinger, et al	Chile	2014	2009-2010	cross-sectional	medical institution	CDC	223	795
Silva, et al	Chile	2013	2007	cross-sectional	community	National Reference	60	453
Kagawa, et al	Chile	2016	2012	cross-sectional	database	WHO	413	11207
García-Hermoso, et al	Chile	2017	2017	cross-sectional	school	IOTF	80	395
Liu, et al	China mainland	2016	2013	cross-sectional	school	National Reference	565	10587
Li, et al	China mainland	2015	2012	cross-sectional	school	WHO	262	2400
Tang, et al	China mainland	2022	2003	cohort	database	WHO	3373	101505
Shi, et al	China mainland	2022	2019-2020	cross-sectional	school	National Reference	3098	105181
Shi, et al	China mainland	2022	2016-2017	cross-sectional	database	National Reference	5355	54269
Liu, et al	China mainland	2022	2019	cross-sectional	community	National Reference	330	7664
Liu, et al	China mainland	2022	2017	cross-sectional	school	National Reference	2318	10753
Li, et al	China mainland	2022	2009-2013	cohort	medical institution	WHO	5492	65056
Li, et al	China mainland	2022	2020	cross-sectional	school	IOTF	1745	18176
Huang, et al	China mainland	2022	2015	cross-sectional	database	IOTF	382	8053
He, et al	China mainland	2022	2019-2020	longitudinal	database	WHO	512	5963
Guo, et al	China mainland	2022	2016	cross-sectional	database	WHO	1567	26120
Chen, et al	China mainland	2022	2014	cross-sectional	database	National Reference	861	17356
Zhu, et al	China mainland	2021	2018	cross-sectional	school	National Reference	1088	12860
Zheng, et al	China mainland	2021	2015-2017	longitudinal	school	National Reference	421	3313
Zhao, et al (1)	China mainland	2021	2002	cross-sectional	school	National Reference	1625	26644
Zhao, et al (2)	China mainland	2021	2018	cross-sectional	school	National Reference	6268	45417
Zhang, et al	China mainland	2021	2016	cross-sectional	community	WHO	2673	110491

© 2024 Zhang X et al. *JAMA Pediatrics*.

Zhang, et al	China mainland	2021	2018	cross-sectional	school	WHO	24	2642
Zhang, et al	China mainland	2021	2017-2019	cross-sectional	database	National Reference	17800	201098
Yuan, et al	China mainland	2021	2016	cross-sectional	school	National Reference	82	768
You, et al	China mainland	2021	2017	cross-sectional	school	National Reference	345	3504
Xu, et al (1)	China mainland	2021	2014	cross-sectional	school	WHO	72	1200
Wang, et al	China mainland	2021	2016-2018	cross-sectional	school	WHO	2321	21571
Wang, et al	China mainland	2021	2014-2016	cross-sectional	database	WHO	1486	8365
Sun, et al	China mainland	2021	2017	longitudinal	database	National Reference	241	1973
Min, et al	China mainland	2021	2018	cross-sectional	school	WHO	179	3373
Liu, et al	China mainland	2021	2021	cross-sectional	school	National Reference	2564	10855
Zhou, et al	China mainland	2020	2016	cross-sectional	school	National Reference	329	2201
Zheng, et al	China mainland	2020	2014-2015	cross-sectional	school	WHO	466	5295
Xu, et al	China mainland	2020	2020	cross-sectional	school	WHO	1497	22681
Shan, et al	China mainland	2010	2004	cross-sectional	school	WHO	1746	21198
Zhang, et al	China mainland	2014	2014	cross-sectional	school	National Reference	164	1488
Abdumijit, et al	China mainland	2022	2021	cross-sectional	community	National Reference	574	4970
Cheng, et al	China mainland	2019	2016-2017	cross-sectional	school	National Reference	97641	1196004
Guo, et al	China mainland	2020	2013-2016	cross-sectional	database	National Reference	6091	40607
Li, et al	China mainland	2014	2009-2010	cross-sectional	school	WHO	45	497
Zheng, et al	China mainland	2021	2019	cross-sectional	school	National Reference	5638	36456
Zhang, et al	China mainland	2021	2021	cross-sectional	database	National Reference	4384	44718
Liu, et al	China mainland	2019	2013-2014	RCT	school	National Reference	371	1889
Jiang, et al	China mainland	2007	2007	RCT	school	IOTF	281	2425
Li, et al	China mainland	2010	2005-2006	RCT	school	WHO	698	4700
Xu, et al	China mainland	2015	2010-2011	RCT	school	National Reference	122	1108
Ji, et al	China mainland	2018	2017	cross-sectional	school	National Reference	11	112
Cui, et al	China mainland	2010	2006	longitudinal	database	IOTF	58	1174
Li, et al	China mainland	2008	2002	cross-sectional	database	IOTF	404	44880
Zhang, et al	China mainland	2012	2003	cross-sectional	community	IOTF	2620	70431
Ma, et al	China mainland	2014	2011	cross-sectional	database	National Reference	1526	36328
Zhang, et al	China mainland	2013	2010	cross-sectional	school	IOTF	1807	42275
Cheng, et al	China mainland	2020	2015-2016	cross-sectional	school	IOTF	379	2201
Andegiorgish, et al	China mainland	2012	2010	cross-sectional	school	National Reference	493	3140
Yao, et al	China mainland	2014	2009-2013	cross-sectional	school	IOTF	2490	67956
Li, et al	China mainland	2013	2009-2011	cross-sectional	medical institution	National Reference	7458	38539
Zhang, et al	China mainland	2013	2013	cohort	community	National Reference	111	1098
Dong, et al	China	2012	2006-2008	cross-sectional	school	CDC	4233	30056

© 2024 Zhang X et al. *JAMA Pediatrics.*

	mainland							
Zhou, et al	China mainland	2021	2011-2016	cross-sectional	medical institution	CDC	920	8441
Zhao, et al	China mainland	2017	2015	cross-sectional	school	National Reference	180	1626
Ma, et al	China mainland	2011	2008-2009	cross-sectional	database	WHO	1195	8653
Cao, et al	China mainland	2012	2009	cross-sectional	school	National Reference	4373	88974
He, et al	China mainland	2009	2006	cross-sectional	school	National Reference	151	2179
Zhang, et al	China mainland	2012	2005	cross-sectional	database	IOTF	318	8568
He, et al	China mainland	2017	2017	cross-sectional	school	National Reference	50	848
Zou, et al	China mainland	2022	2016-2017	cross-sectional	database	National Reference	297	2818
Zhang, et al	China mainland	2022	2019	cross-sectional	database	WHO	1563	14534
Zhang, et al	China mainland	2022	2021	cross-sectional	school	National Reference	525	4412
Zeng, et al	China mainland	2022	2012-2013	cohort	database	IOTF	14	430
Yang, et al	China mainland	2022	2019	longitudinal	database	WHO	856	6047
Xu, et al	China mainland	2022	2009	longitudinal	school	National Reference	475	4538
Wu, et al	China mainland	2022	2009-2011	cross-sectional	database	WHO	5446	47990
Wang, et al	China mainland	2022	2018	cross-sectional	school	National Reference	653	10536
Wang, et al	China mainland	2022	2020	cross-sectional	school	National Reference	1029	9501
Iwata, et al	China mainland	2003	2003	cross-sectional	school	National Reference	112	532
Xiong, et al	China mainland	2010	2003-2004	cross-sectional	school	IOTF	320	7326
Tan, et al	China mainland	2018	2018	cross-sectional	school	IOTF	244	8999
Wang, et al	China mainland	2018	2017	cross-sectional	school	National Reference	615	18403
Zhang, et al (1)	China mainland	2018	2010	cross-sectional	community	IOTF	2546	68020
Zhang, et al (2)	China mainland	2018	2011	cross-sectional	community	IOTF	2219	43928
Zhang, et al (3)	China mainland	2018	2012	cross-sectional	community	IOTF	5896	105153
Zhang, et al (4)	China mainland	2018	2013	cross-sectional	community	IOTF	7956	129830
Zhang, et al (5)	China mainland	2018	2014	cross-sectional	community	IOTF	7188	119692
Zhang, et al (6)	China mainland	2018	2015	cross-sectional	community	IOTF	6946	107587
Zhang, et al	China mainland	2018	2015	cross-sectional	database	IOTF	92	1617
Zhang, et al	China mainland	2018	2014-2017	cross-sectional	school	National Reference	38035	325083
Cai, et al	China mainland	2019	2013	cross-sectional	school	National Reference	5419	47590
Chen ,et al (1)	China mainland	2019	2011	cross-sectional	community	WHO	1081	15757
Chen ,et al (2)	China mainland	2019	2014	cross-sectional	community	WHO	1126	19098
Chen ,et al (3)	China mainland	2019	2017	cross-sectional	community	WHO	1003	21883
Zhang, et al	China mainland	2019	2015	cross-sectional	database	CDC	11877	177419
Zhao, et al	China mainland	2019	2012	cross-sectional	database	National Reference	86	1081
Zou, et al	China mainland	2019	2014	cross-sectional	school	National Reference	214	2639
Duan, et al	China mainland	2020	2016	cross-sectional	medical institution	National Reference	96	1955

© 2024 Zhang X et al. *JAMA Pediatrics.*

Ke, et al	China mainland	2020	2019	cross-sectional	school	IOTF	146	1330
Qian, et al	China mainland	2020	2017	cross-sectional	community	IOTF	1889	12426
Song, et al	China mainland	2020	2016	cross-sectional	database	WHO	117	4488
Sun, et al	China mainland	2020	2012	cross-sectional	school	WHO	181	2185
Xing, et al	China mainland	2020	2012-2013	cross-sectional	database	WHO	1154	6740
Fan, et al	China mainland	2010	2010	cross-sectional	school	National Reference	170	3544
Wang, et al	China mainland	2009	2009	cross-sectional	community	WHO	435	8041
Xu, et al	China mainland	2008	2006	cross-sectional	school	IOTF	146	2020
Shi, et al	China mainland	2007	2002	cross-sectional	school	WHO	40	824
Liu, et al	China mainland	2007	2000	cross-sectional	community	IOTF	2652	263168
Yan, et al	China mainland	2006	2003	cross-sectional	school	National Reference	2671	69627
Li, et al	China mainland	2006	2004	cross-sectional	school	IOTF	64	1792
Jiang, et al	China mainland	2006	2006	cross-sectional	school	IOTF	39	930
Zhu, et al	China mainland	2015	2013	cross-sectional	school	IOTF	82	4788
Yuan, et al	China mainland	2015	2010	cross-sectional	school	National Reference	1438	16580
Xu, et al	China mainland	2015	2007-2011	cross-sectional	community	National Reference	1333	29997
Xiao, et al	China mainland	2015	2006-2014	cross-sectional	school	WHO	2611	145078
Wu, et al	China mainland	2015	2011-2012	cross-sectional	school	IOTF	5079	55536
Piernas, et al (1)	China mainland	2015	2009	cross-sectional	database	IOTF	54	1191
Piernas, et al (2)	China mainland	2015	2011	cross-sectional	database	IOTF	151	1648
Ma, et al (1)	China mainland	2015	2007	cross-sectional	school	National Reference	272	3832
Ma, et al (2)	China mainland	2015	2008	cross-sectional	school	National Reference	815	13141
Ma, et al (3)	China mainland	2015	2009	cross-sectional	school	National Reference	1054	14052
Ma, et al (4)	China mainland	2015	2010	cross-sectional	school	National Reference	1004	13750
Katzmarzyk, et al (5)	China mainland	2015	2011-2013	cross-sectional	database	WHO	123	501
Cao, et al	China mainland	2015	2013	cross-sectional	school	National Reference	736	8760
Wang, et al	China mainland	2014	2009	cross-sectional	school	CDC	4233	30056
He, et al	China mainland	2014	2014	cross-sectional	school	IOTF	2490	67956
Dong, et al	China mainland	2014	2011	cross-sectional	community	National Reference	377	4898
Dai, et al	China mainland	2014	2009-2010	cross-sectional	medical institution	National Reference	1505	18707
Chen, et al (2)	China mainland	2014	2010-2011	cross-sectional	community	IOTF	106	1951
Chen, et al	China mainland	2011	2005	cross-sectional	database	National Reference	8790	231326
Xu, et al	China mainland	2011	2011	cross-sectional	school	National Reference	926	8898
Guo, et al	China mainland	2012	2010-2011	cross-sectional	community	IOTF	273	4262
Jia, et al	China mainland	2012	2010	cross-sectional	school	National Reference	112	702
Li, et al	China mainland	2012	2011	cross-sectional	school	WHO	238	7194
Yi, et al	China	2012	2008-2009	cross-sectional	school	National	277	6740

© 2024 Zhang X et al. *JAMA Pediatrics.*

	mainland					Reference		
Dong, et al	China mainland	2013	2010	cross-sectional	database	National Reference	10259	197191
Chen, et al (1)	China mainland	2016	2009	cross-sectional	medical institution	WHO	196	22576
Chen, et al (2)	China mainland	2016	2012	cross-sectional	medical institution	WHO	228	24816
Chen, et al (3)	China mainland	2016	2015	cross-sectional	medical institution	WHO	332	23837
Lei, et al	China mainland	2016	2013	cross-sectional	database	National Reference	417	3327
Liu, et al	China mainland	2016	2014	cross-sectional	school	WHO	679	9917
Peng, et al	China mainland	2016	2016	cross-sectional	school	National Reference	931	12297
Wei, et al	China mainland	2016	2016	cross-sectional	community	National Reference	129	1928
Zhang, et al	China mainland	2016	2013	cross-sectional	medical institution	CDC	102	470
Zhang, et al	China mainland	2016	2009-2010	cross-sectional	school	WHO	115	1410
Cai, et al	China mainland	2017	2016	cross-sectional	database	National Reference	13396	116615
Liu, et al	China mainland	2017	2015	cross-sectional	database	WHO	4831	45608
Liu, et al	China mainland	2017	2016	cross-sectional	school	WHO	340	4926
Zhang, et al	China mainland	2017	2014	cross-sectional	database	IOTF	1784	17318
Zong, et al	China mainland	2017	2006	cross-sectional	school	National Reference	13073	290043
Dong, et al	China mainland	2018	2014	cross-sectional	database	National Reference	3556	80821
Gong, et al	China mainland	2018	2016-2018	cross-sectional	database	IOTF	97	2795
He, et al	China mainland	2018	2013-2014	prospective	school	National Reference	178	2032
Li, et al	China mainland	2018	2014	prospective	school	National Reference	108	1237
Liu, et al	China mainland	2018	2017	cross-sectional	school	National Reference	623	3670
Contreras, et al	Colombia	2015	2013	cross-sectional	school	WHO	25	603
Rincón-Pabón, et al	Colombia	2019	2010	cross-sectional	database	WHO	546	18177
Ramírez-Vélez, et al	Colombia	2017	2014-2015	cross-sectional	database	IOTF	139	2510
Martinez-Ospina, et al	Colombia	2019	2015	cross-sectional	school	WHO	76	715
McDonald, et al	Colombia	2009	2006	cross-sectional	school	IOTF	54	3075
Katzmarzyk, et al (6)	Colombia	2015	2011-2013	cross-sectional	database	WHO	49	857
Gebremedhin, et al (4)	Comoros	2015	2012	cross-sectional	database	WHO	173	2699
Gebremedhin, et al (5)	Congo	2015	2011/2012	cross-sectional	database	WHO	59	4531
Gebremedhin, et al (6)	Congo	2015	2013/2014	cross-sectional	database	WHO	227	8391
Gamboa-Gamboa, et al	Costa Rica	2021	2016	cross-sectional	database	WHO	49128	347366
Gebremedhin, et al (11)	Côte d'Ivoire	2015	2011/2012	cross-sectional	database	WHO	40	3294
Fossou, et al	Côte d'Ivoire	2020	2018	cross-sectional	school	WHO	31	1251
Salas, et al (3)	Croatia	2021	2015-2017	cross-sectional	database	WHO	351	2601
Banjari, et al (2)	Croatia	2020	2020	cross-sectional	school	IOTF	33	478
Juresa, et al	Croatia	2012	2003-2004	cross-sectional	school	IOTF	73	960
Janssen, et	Croatia	2005	2001-2002	cross-sectional	database	IOTF	75	4145

© 2024 Zhang X et al. *JAMA Pediatrics.*

al (4)								
Milanović, et al	Croatia	2020	2015	cross-sectional	school	WHO	816	5591
Haug, et al (10)	Croatia	2009	2005-2006	cross-sectional	database	IOTF	104	4720
Bili}-Kirin, et al	Croatia	2014	2014	cross-sectional	school	National Reference	9	372
Pećin, et al	Croatia	2013	2013	cross-sectional	school	CDC	25	756
Ahrens, et al (3)	Cyprus	2014	2007-2008	cross-sectional	database	IOTF	247	2942
Savva, et al	Cyprus	2005	2004	cross-sectional	school	IOTF	78	1413
Savva, et al	Cyprus	2008	2002-2003	cross-sectional	school	IOTF	559	7060
Savva, et al	Cyprus	2014	2009-2010	cross-sectional	school	IOTF	251	3090
Lazarou, et al	Cyprus	2008	2008	cross-sectional	school	IOTF	35	823
Loucaides, et al	Cyprus	2008	2007	cross-sectional	school	IOTF	17	247
Tornaritis, et al	Cyprus	2014	2009-2010	cross-sectional	school	IOTF	93	1414
Savva, et al	Cyprus	2014	2001-2003	longitudinal	school	IOTF	242	4878
Vážná, et al	Czech	2022	2021	cross-sectional	school	WHO	512	3517
Salas, et al (4)	Czech	2021	2015-2017	cross-sectional	database	WHO	98	1395
Gorog, et al (2)	Czech	2011	2011	cross-sectional	database	IOTF	30	1508
Gouw, et al	Czech	2010	2001	cross-sectional	database	IOTF	556	30966
Janssen, et al (5)	Czech	2005	2001-2002	cross-sectional	database	IOTF	50	4990
Haug, et al (20)	Czech	2009	2005-2006	cross-sectional	database	IOTF	123	4734
Wijnhoven, et al (2)	Czech	2015	2007/2008	cross-sectional	school	WHO	119	1633
Brixval, et al	Denmark	2012	2002	cross-sectional	database	IOTF	53	4208
Salas, et al (5)	Denmark	2021	2015-2017	cross-sectional	database	WHO	30	935
Yngve, et al (3)	Denmark	2008	2003	cross-sectional	database	IOTF	11	1066
Ajslev, et al	Denmark	2011	2004-2009	cohort	database	IOTF	378	28354
Mor, et al	Denmark	2015	2002-2013	cohort	database	IOTF	309	9886
Høyer, et al (1)	Denmark	2014	2002-2004	cohort	database	WHO	46	525
Janssen, et al (6)	Denmark	2005	2001-2002	cross-sectional	database	IOTF	52	4009
Krue, et al	Denmark	2010	2010	cross-sectional	school	National Reference	974	8694
Haug, et al (27)	Denmark	2009	2005-2006	cross-sectional	database	IOTF	61	4708
Rex, et al	Denmark	2014	2011-2013	cross-sectional	medical institution	IOTF	41	607
Matthiessen, et al	Denmark	2014	2005-2008	cross-sectional	database	IOTF	16	512
Kjelgaard, et al	Denmark	2017	2010	cross-sectional	database	IOTF	76	4922
Manyanga, et al (2)	Djibouti	2014	2014	cross-sectional	database	WHO	89	1711
Taguri, et al (3)	Djibouti	2009	2002	cross-sectional	database	WHO	137	1538
Romano, et al (7)	Dominican Republic	2022	2016	cross-sectional	database	WHO	117	954
Romano, et al (8)	East Timor	2022	2015	cross-sectional	database	WHO	20	1631
Freire, et al	Ecuador	2014	2012	cross-sectional	database	WHO	1257	11534
Ortiz, et al	Ecuador	2014	2008	cross-sectional	community	WHO	41	703
Casaoulla, et al	Ecuador	2017	2015	cross-sectional	school	IOTF	8	427
Walrod, et al	Ecuador	2018	2013-2014	cross-sectional	community	WHO	129	298
Abd El-Aty, et al	Egypt	2020	2016-2017	cross-sectional	community	CDC	470	5458
Abdel Wahed, et al	Egypt	2017	2014-2015	cross-sectional	community	WHO	110	736
Abdelkarim,	Egypt	2017	2014-2015	cross-sectional	community	IOTF	83	676

© 2024 Zhang X et al. *JAMA Pediatrics*.

et al								
Abou-Khadra, et al	Egypt	2022	2018-2019	cross-sectional	school	WHO	72	319
El-Sabely, et al	Egypt	2013	2013	cross-sectional	school	WHO	77	288
Badawi, et al	Egypt	2013	2011	cross-sectional	school	National Reference	115	852
Manyanga, et al (3)	Egypt	2014	2014	cross-sectional	database	WHO	482	5179
Hassan, et al	Egypt	2008	2002-2004	cross-sectional	school	CDC	73	1283
Hassan, et al	Egypt	2016	2013-2016	cross-sectional	medical institution	National Reference	30	154
Talat, et al	Egypt	2016	2014-2015	cross-sectional	school	National Reference	96	900
Pérez, et al	El Salvador	2020	2015-2016	cross-sectional	school	IOTF	10087	111991
Ahrens, et al (2)	Estonia	2014	2007-2008	cross-sectional	database	IOTF	80	2067
Janssen, et al (8)	Estonia	2005	2001-2002	cross-sectional	database	IOTF	38	3752
Haug, et al (29)	Estonia	2009	2005-2006	cross-sectional	database	IOTF	63	4215
Weinmayr, et al (2)	Estonia	2014	2004	cross-sectional	database	IOTF	7	241
Worku, et al	Ethiopia	2021	2018	cross-sectional	school	WHO	40	522
Abich, et al	Ethiopia	2020	2018-2019	cross-sectional	school	CDC	42	823
Tadesse, et al	Ethiopia	2017	2015	cross-sectional	school	WHO	13	462
Sorrie, et al	Ethiopia	2017	2016	cross-sectional	community	WHO	21	500
Desalew, et al	Ethiopia	2017	2016	cross-sectional	school	CDC	26	448
Gebremedhin, et al (7)	Ethiopia	2015	2011	cross-sectional	database	WHO	128	9880
Gali, et al	Ethiopia	2017	2015	cross-sectional	school	WHO	8	510
Askal, et al	Ethiopia	2015	2014	cross-sectional	school	CDC	15	828
Carrillo-Larco, et al (4)	Ethiopia	2014	2014	cohort	database	IOTF	4	1882
Mitiku, et al	Ethiopia	2019	2014	cross-sectional	school	WHO	19	1523
Mekonnen, et al	Ethiopia	2018	2016	cross-sectional	school	CDC	20	634
Romano, et al (9)	Fiji	2022	2016	cross-sectional	database	WHO	126	1537
Pengpid, et al (1)	Fiji	2015	2010-2011	cross-sectional	database	IOTF	92	1773
Petersen, et al	Fiji	2014	2005-2008	cross-sectional	database	IOTF	466	8947
Sarkkola, et al	Finland	2022	2011-2014	cross-sectional	database	IOTF	277	10646
Eloranta, et al	Finland	2012	2007-2009	cross-sectional	database	IOTF	23	510
Janssen, et al (9)	Finland	2005	2001-2002	cross-sectional	database	IOTF	130	5205
Veltsista, et al (1)	Finland	2010	2001	cross-sectional	school	IOTF	186	6468
Vanhala, et al	Finland	2009	2004	cross-sectional	school	IOTF	34	749
Haug, et al (30)	Finland	2009	2005-2006	cross-sectional	database	IOTF	113	4934
Fogelholm, et al	Finland	2008	2003	cross-sectional	school	IOTF	49	2266
Katzmarzyk, et al (7)	Finland	2015	2011-2013	cross-sectional	database	WHO	26	500
Vanhelst, et al	France	2022	2008-2018	cross-sectional	database	IOTF	2860	90250
Roth, et al	France	2022	2017-2020	cross-sectional	school	IOTF	509	19295
Luiggi, et al	France	2021	2019	cross-sectional	school	CDC	35	1038
Thibault, et al	France	2013	2007-2009	cross-sectional	school	IOTF	195	7667
Thibault, et al	France	2010	2004-2005	cross-sectional	school	IOTF	45	2385
Carriere, et	France	2015	2004	cross-sectional	school	IOTF	54	1836

© 2024 Zhang X et al. *JAMA Pediatrics.*

al								
Salanave, et al	France	2009	2007	cross-sectional	school	IOTF	28	1014
Heude, et al	France	2003	2000	cross-sectional	school	IOTF	17	601
Legleye, et al	France	2014	2010	cross-sectional	database	IOTF	7	303
Chau, et al	France	2013	2010	cross-sectional	school	National Reference	166	1270
Jouret, et al	France	2007	2007	cross-sectional	school	IOTF	32	1780
Pitrou, et al	France	2010	2004-2005	cross-sectional	school	IOTF	34	1030
Janssen, et al (10)	France	2005	2001-2002	cross-sectional	database	IOTF	122	7624
Kleiser, et al	France	2003	2001	cross-sectional	school	IOTF	227	4326
Rolland-Cachera, et al	France	2002	2000	cross-sectional	school	IOTF	60	1582
Tubert-Jeannin, et al	France	2018	2011-2012	cross-sectional	school	WHO	386	2676
Haug, et al (5)	France	2009	2005-2006	cross-sectional	database	IOTF	105	6583
Kêkê, et al	France	2015	2009	cross-sectional	school	IOTF	93	1382
Vanhelst, et al (1)	France	2017	2009	cross-sectional	database	IOTF	70	1848
Vanhelst, et al (2)	France	2017	2010	cross-sectional	database	IOTF	75	1818
Vanhelst, et al (3)	France	2017	2011	cross-sectional	database	IOTF	94	2578
Vanhelst, et al (4)	France	2017	2012	cross-sectional	database	IOTF	120	2165
Vanhelst, et al (5)	France	2017	2013	cross-sectional	database	IOTF	77	1260
Romano, et al (10)	French Polynesis	2022	2015	cross-sectional	database	WHO	420	1902
Gebremedhin, et al (8)	Gabon	2015	2012	cross-sectional	database	WHO	129	3482
Gebremedhin, et al (9)	Gambia	2015	2013	cross-sectional	database	WHO	114	3360
Salas, et al (6)	Georgia	2021	2015-2017	cross-sectional	database	WHO	269	3057
Weinmayr, et al (3)	Georgia	2014	2004	cross-sectional	database	IOTF	12	169
Ahrens, et al (6)	Germany	2014	2007-2008	cross-sectional	database	IOTF	103	2194
Hoebel, et al	Germany	2022	2014-2017	cross-sectional	database	National Reference	210	3567
Zhou, et al	Germany	2021	2010-2014	cross-sectional	school	National Reference	930	22678
Usheva, et al (3)	Germany	2021	2012	cross-sectional	database	WHO	14	1104
Sergentanis, et al (3)	Germany	2021	2011-2012	cross-sectional	database	IOTF	46	1621
Nguyen, et al	Germany	2021	2017-2018	cross-sectional	school	National Reference	550	5656
Liu, et al	Germany	2021	2013-2018	cross-sectional	school	WHO	1478	33407
Willerhausen, et al	Germany	2007	2007	cross-sectional	medical institution	National Reference	126	1290
Kesztyüs, et al	Germany	2013	2009	cross-sectional	database	National Reference	4	1730
Negal, et al	Germany	2009	2006	RCT	school	IOTF	38	1063
Boneberger, et al	Germany	2009	2005-2006	cross-sectional	school	IOTF	160	4718
Kromeyer-Hauschild, et al	Germany	2007	2001	cross-sectional	school	IOTF	26	1915
Landsberg, et al	Germany	2010	2000-2005	cross-sectional	database	IOTF	83	1894
Toschke, et al	Germany	2005	2001-2002	cross-sectional	medical institution	IOTF	114	4370
Toschke, et al	Germany	2003	2000-2001	cross-sectional	school	IOTF	125	4706
Kries, et al	Germany	2008	2005	cross-sectional	school	IOTF	230	5899
Raum, et al	Germany	2011	2001-2002	cross-sectional	medical	National	83	1979

© 2024 Zhang X et al. *JAMA Pediatrics.*

					institution	Reference		
Kleiser, et al	Germany	2009	2003-2006	cross-sectional	database	IOTF	709	13395
Toschke, et al	Germany	2007	2001-2002	cross-sectional	school	IOTF	176	5472
Weyermann, et al	Germany	2006	2000-2001	cohort	medical institution	National Reference	72	855
Will, et al	Germany	2005	2002	cross-sectional	school	IOTF	13	523
Rapp, et al	Germany	2005	2002	longitudinal	school	IOTF	96	2140
Lamerz, et al	Germany	2005	2001-2002	cross-sectional	school	National Reference	178	1979
Janssen, et al (11)	Germany	2005	2001-2002	cross-sectional	database	IOTF	83	4878
Graf, et al	Germany	2004	2001	cross-sectional	school	National Reference	32	668
Graf, et al	Germany	2004	2002-2003	cross-sectional	school	National Reference	22	344
Reich, et al	Germany	2003	2003	cross-sectional	school	National Reference	280	2354
Toschke, et al	Germany	2009	2004-2005	cross-sectional	school	IOTF	166	4642
Sporišević, et al	Germany	2009	2007-2008	cross-sectional	community	CDC	20	214
Haug, et al (6)	Germany	2009	2005-2006	cross-sectional	database	IOTF	126	6619
De Toia , et al	Germany	2009	2006-2008	cross-sectional	school	National Reference	43	1228
Bayer, et al	Germany	2009	2004-2006	cross-sectional	database	IOTF	479	12199
Nagel, et al	Germany	2008	2004-2005	cross-sectional	school	IOTF	20	557
Jahnke, et al	Germany	2008	2008	cross-sectional	medical institution	IOTF	15	142
Herpertz-Dahlmann, et al	Germany	2008	2003-2006	cross-sectional	community	National Reference	142	1843
Willershausen, et al	Germany	2007	2007	cross-sectional	school	National Reference	131	2071
Kobel, et al	Germany	2015	2015	cross-sectional	school	National Reference	13	294
Brettschneider, et al	Germany	2015	2009-2012	cross-sectional	database	National Reference	152	4948
Weinmayr, et al (4)	Germany	2014	2004	cross-sectional	database	IOTF	61	1580
Aboagye, et al	Ghana	2022	2022	cross-sectional	school	National Reference	58	423
Adom, et al	Ghana	2019	2019	cross-sectional	school	WHO	39	543
Amidu, et al	Ghana	2013	2012-2013	cross-sectional	school	CDC	30	400
Hohammed, et al	Ghana	2012	2012	cross-sectional	school	WHO	29	270
Intiful, et al	Ghana	2013	2013	cross-sectional	school	WHO	14	124
Manyanga, et al (4)	Ghana	2014	2014	cross-sectional	database	WHO	62	6155
Obiricorang, et al	Ghana	2015	2013-2014	cross-sectional	school	National Reference	7	303
Aryeetey, et al	Ghana	2017	2009-2012	cross-sectional	school	WHO	176	3089
Atsu, et al	Ghana	2017	2011	cross-sectional	database	WHO	48	7328
Annan-Asare, et al	Ghana	2017	2017	cross-sectional	school	National Reference	69	260
Gyamfi, et al	Ghana	2019	2019	cross-sectional	school	WHO	89	1004
Ganle, et al	Ghana	2019	2019	cross-sectional	school	CDC	65	285
Weinmayr, et al (5)	Ghana	2014	2004	cross-sectional	database	IOTF	0	241
Makri, et al	Greece	2022	2018	cross-sectional	database	WHO	202	3816
Usheva, et al (4)	Greece	2021	2012	cross-sectional	database	WHO	80	1647
Sergentanis, et al (7)	Greece	2021	2011-2012	cross-sectional	database	IOTF	49	1516
Kostopoulou, et al	Greece	2021	2018-2019	cross-sectional	database	CDC	424	3504
Pikramenou, et al	Greece	2016	2013	cross-sectional	school	IOTF	71	2180
Trikaliotis, et al	Greece	2011	2011	cross-sectional	school	IOTF	10	361

© 2024 Zhang X et al. *JAMA Pediatrics.*

Grigorakis, et al	Greece	2016	2010-2011	cross-sectional	school	IOTF	10922	124113
Kleanthous, et al	Greece	2016	2009	cross-sectional	school	IOTF	125	1327
Manios, et al	Greece	2013	2007	cross-sectional	database	IOTF	284	2492
Manios, et al	Greece	2011	2005-2006	cross-sectional	school	IOTF	63	481
Tokmakidis, et al	Greece	2006	2006	cross-sectional	school	IOTF	105	709
Lamprokostopoulou, et al	Greece	2019	2007-2010	cross-sectional	school	IOTF	276	919
Kontogianni, et al	Greece	2010	2007	cross-sectional	community	IOTF	113	1170
Kosti, et al	Greece	2007	2004-2005	cross-sectional	database	IOTF	62	2008
Cassimos, et al	Greece	2011	2008-2009	cross-sectional	school	IOTF	53	335
Krassas, et al (1)	Greece	2004	2004	cross-sectional	school	National Reference	101	2468
Angelopoulos, et al	Greece	2006	2003-2004	cross-sectional	school	IOTF	30	312
Magkos, et al	Greece	2006	2002	cross-sectional	community	IOTF	74	620
Magkos, et al	Greece	2006	2006	cross-sectional	school	IOTF	13	198
Papadimitriou, et al	Greece	2006	2003-2004	cross-sectional	school	IOTF	459	4131
Manios, et al	Greece	2007	2003-2004	cross-sectional	database	IOTF	170	2374
Kamtsios, et al	Greece	2008	2008	cross-sectional	school	IOTF	26	775
Linardakis, et al	Greece	2008	2004-2005	cross-sectional	school	IOTF	91	856
Hassapidou, et al	Greece	2009	2006	cross-sectional	school	IOTF	31	266
Kollias, et al	Greece	2009	2009	cross-sectional	school	IOTF	66	797
Koroni, et al	Greece	2009	2009	cross-sectional	school	IOTF	169	1861
Mavrakanas, et al	Greece	2009	2007	cross-sectional	school	IOTF	80	572
Yannakoulia, et al	Greece	2010	2010	cross-sectional	database	IOTF	103	1132
Hassapidou, et al	Greece	2015	2009-2010	cross-sectional	school	IOTF	58	1006
Spathopoulos, et al	Greece	2009	2005-2006	cross-sectional	school	National Reference	334	2715
Antonogeorgos, et al	Greece	2012	2005-2006	cross-sectional	database	IOTF	49	700
Janssen, et al (12)	Greece	2005	2001-2002	cross-sectional	database	IOTF	79	3584
Manios, et al	Greece	2004	2004	cross-sectional	school	IOTF	13	195
Veltsista, et al (2)	Greece	2010	2001	cross-sectional	school	IOTF	63	2842
Poulimeneas, et al	Greece	2019	2019	cross-sectional	community	IOTF	7	172
Vazquez, et al	Greece	2019	2012-2013	cross-sectional	database	CDC	284	2675
Katsagoni, et al	Greece	2020	2014-2015	cross-sectional	database	WHO	13009	174209
Notara,et al	Greece	2020	2014-2016	cross-sectional	school	IOTF	87	1659
Mirkopoulou, et al	Greece	2010	2005	cross-sectional	school	IOTF	2	98
Karatzi , et al	Greece	2009	2004-2005	prospective	school	IOTF	29	754
Haug, et al (11)	Greece	2009	2005-2006	cross-sectional	database	IOTF	89	3566
Vafeiadi, et al	Greece	2015	2007	prospective	community	IOTF	48	689
Sourani, et al	Greece	2015	2011	cross-sectional	community	IOTF	29	352
Patsopoulou, et al	Greece	2015	2015	cross-sectional	school	IOTF	20	451

© 2024 Zhang X et al. *JAMA Pediatrics*.

Kambas, et al	Greece	2015	2011	cross-sectional	database	IOTF	39	250
Weinmayr, et al (6)	Greece	2014	2004	cross-sectional	database	IOTF	70	404
Grammatikopoulou, et al	Greece	2014	2010-2012	cross-sectional	school	IOTF	2786	37344
Antonogeorgos, et al	Greece	2011	2006	cross-sectional	database	National Reference	49	700
Athanasopoulos, et al	Greece	2011	2010	cross-sectional	school	IOTF	19	232
Farajian, et al	Greece	2011	2009	cross-sectional	school	IOTF	560	4786
Michalopoulou, et al	Greece	2011	2009	cross-sectional	school	IOTF	34	532
Jelastopulu, et al	Greece	2012	2009	cross-sectional	school	IOTF	21	200
Kyriazis, et al	Greece	2012	2009-2010	cross-sectional	school	IOTF	173	2374
Tambalis, et al	Greece	2013	2013	cross-sectional	school	IOTF	353	3195
Poulimeneas, et al	Greece	2016	2016	cross-sectional	school	IOTF	420	4833
Garoufi, et al	Greece	2017	2009-2010	cross-sectional	school	National Reference	76	736
Velde, et al (2)	Greece	2017	2010	cross-sectional	database	IOTF	113	1085
Koulouvaris, et al	Greece	2018	2018	cross-sectional	community	WHO	87	463
Manios, et al	Greece	2018	2018	cross-sectional	school	IOTF	267	2263
Janssen, et al (13)	Greenland	2005	2001-2002	cross-sectional	database	IOTF	9	558
Haug, et al (31)	Greenland	2009	2005-2006	cross-sectional	database	IOTF	25	943
Alvarado, et al	Guatemala	2009	2004-2005	cross-sectional	school	CDC	71	363
Gebremedhin, et al (10)	Guinea	2015	2012	cross-sectional	database	WHO	80	3216
Torres, et al	Honduras	2014	2011	cross-sectional	school	WHO	112	2554
Tung, et al	Hong Kong	2021	2014	cohort	database	IOTF	1093	18863
Huang, et al	Hong Kong	2019	2009-2012	cohort	database	IOTF	50	599
Wing, et al	Hong Kong	2009	2003	cross-sectional	school	CDC	253	5159
Wang, et al	Hong Kong	2019	2008-2009	cohort	database	IOTF	109	3614
Chan, et al	Hong Kong	2013	2007-2008	cross-sectional	school	National Reference	120	1614
Wang, et al	Hong Kong	2017	2015	cross-sectional	school	IOTF	41	894
Wong, et al	Hong Kong	2005	2002	cross-sectional	school	IOTF	830	10773
Ko, et al	Hong Kong	2008	2003-2004	cross-sectional	school	IOTF	58	2098
Knowles, et al	Hong Kong	2015	2015	cross-sectional	school	IOTF	36	620
Ip, et al	Hong Kong	2017	2013-2014	cross-sectional	school	IOTF	10351	208280
Lee, et al	Hong Kong	2017	2012-2013	cross-sectional	database	IOTF	239	4410
Brug, et al (2)	Hungary	2012	2012	cross-sectional	database	IOTF	54	1020
Ahrens, et al (7)	Hungary	2014	2007-2008	cross-sectional	database	IOTF	201	3159
Baráth, et al	Hungary	2010	2005-2006	cross-sectional	school	IOTF	943	14290
Bjørnarå, et al	Hungary	2014	2010	cross-sectional	database	National Reference	121	929
Gorog, et al (3)	Hungary	2011	2011	cross-sectional	database	IOTF	50	1661
Janssen, et al (14)	Hungary	2005	2001-2002	cross-sectional	database	IOTF	92	3845
Haug, et al (21)	Hungary	2009	2005-2006	cross-sectional	database	IOTF	93	3214
Antal, et al	Hungary	2009	2005-2006	cross-sectional	school	IOTF	133	1928
Velde, et al (3)	Hungary	2017	2010	cross-sectional	database	IOTF	54	1022
Erdei, et al	Hungary	2018	2016	cross-sectional	school	IOTF	473	5332
Jakab, et al	Hungary	2018	2015-2017	cross-sectional	school	IOTF	915	6824
Aanesen, et al	Iceland	2020	2016-2017	cross-sectional	community	IOTF	193	4360
Sergentanis	Iceland	2021	2011-2012	cross-sectional	database	IOTF	26	1196

© 2024 Zhang X et al. *JAMA Pediatrics.*

, et al (1)								
Yngve, et al (4)	Iceland	2008	2003	cross-sectional	database	IOTF	11	714
Haug, et al (32)	Iceland	2009	2005-2006	cross-sectional	database	IOTF	208	8014
Dabas, et al (1)	India	2022	2008	cross-sectional	school	IOTF	130	3172
Dabas, et al (2)	India	2022	2013	cross-sectional	school	IOTF	293	3007
Dabas, et al (3)	India	2022	2015	cross-sectional	school	IOTF	137	2238
Arushi, et al	India	2022	2020-2021	cross-sectional	school	WHO	76	1620
Thomas, et al	India	2021	2019-2020	cross-sectional	school	WHO	17	440
Seema, et al	India	2021	2016-2018	cross-sectional	school	WHO	26	385
Moitra, et al	India	2021	2021	cross-sectional	school	WHO	81	772
Elangovan, et al	India	2012	2011	cross-sectional	medical institution	CDC	60	510
Subramaniam, et al	India	2011	2011	cross-sectional	school	CDC	58	2033
Shailee, et al	India	2018	2009	cross-sectional	school	CDC	7	1011
Goyal, et al	India	2011	2009-2010	cross-sectional	school	National Reference	76	1159
Jain, et al	India	2010	2003-2004	cross-sectional	school	National Reference	222	2570
Gautam, et al	India	2019	2012	cross-sectional	school	WHO	74	1185
Bharati, et al	India	2008	2005-2006	cross-sectional	school	National Reference	32	2255
Kotian, et al	India	2010	2007	cross-sectional	school	IOTF	43	900
Warraich, et al	India	2009	2009	cross-sectional	school	National Reference	17	284
Mushtag, et al	India	2011	2011	cross-sectional	school	WHO	176	1860
Kumar, et al	India	2007	2007	cross-sectional	school	National Reference	86	1496
Tiwari, et al	India	2014	2010-2011	cross-sectional	school	WHO	10	940
Bansal, et al	India	2022	2022	cross-sectional	school	National Reference	40	859
Baruah, et al	India	2018	2017	cross-sectional	school	National Reference	11	349
Baston, et al	India	2014	2009-2010	cross-sectional	school	WHO	321	2130
Carrillo-Larco, et al (2)	India	2014	2014	cohort	database	IOTF	6	1929
Khadilkar, et al	India	2011	2007-2008	cross-sectional	community	IOTF	951	20243
Sultan, et al	India	2008	2008	cross-sectional	medical institution	WHO	24	172
Gupta, et al	India	2011	2008-2009	cross-sectional	database	IOTF	574	4908
Sharma, et al	India	2007	2007	cross-sectional	school	IOTF	282	4399
Ramachandran, et al	India	2002	2002	cross-sectional	school	IOTF	149	4700
Bose, et al	India	2007	2002	cross-sectional	school	IOTF	22	431
Mehta, et al	India	2007	2002	cross-sectional	school	IOTF	22	414
Laxmaiah, et al	India	2007	2003	cross-sectional	school	IOTF	15	1208
Raj, et al	India	2007	2003-2004	cross-sectional	school	CDC	313	24842
Iyer, et al	India	2011	2004	cross-sectional	school	IOTF	63	1067
Premanath, et al	India	2010	2005-2006	cross-sectional	school	National Reference	1448	43152
Marwaha, et al	India	2006	2006	cross-sectional	school	IOTF	747	21485
Kumar, et al	India	2008	2007	cross-sectional	school	National Reference	6	425
Sood, et al	India	2007	2007	cross-sectional	school	CDC	171	3970
Goyal, et al	India	2010	2010	cross-sectional	school	IOTF	130	5664
Ghosh, et al	India	2011	2011	cross-sectional	school	National Reference	42	753

© 2024 Zhang X et al. *JAMA Pediatrics*.

Mahajan, et al	India	2011	2008-2009	cross-sectional	school	CDC	54	2542
Cherian, et al	India	2012	2009-2010	cross-sectional	school	CDC	67	1634
Thakre, et al	India	2011	2009-2011	cross-sectional	school	CDC	84	1524
Keerthan, et al	India	2011	2011	cross-sectional	school	National Reference	15	500
Vohra, et al	India	2011	2011	cross-sectional	school	CDC	3	407
Chakraborty, et al	India	2011	2008-2009	cross-sectional	school	CDC	181	979
Sidhu, et al	India	2005	2005	cross-sectional	school	National Reference	36	640
Sidhu, et al	India	2006	2006	cross-sectional	school	IOTF	61	1000
Misra, et al	India	2011	2006-2008	cross-sectional	school	IOTF	1072	38296
Jagadesan, et al	India	2014	2014	cross-sectional	school	IOTF	645	18955
Kaur, et al	India	2008	2008	cross-sectional	community	IOTF	470	15996
Chhabra, et al	India	2012	2012	cross-sectional	medical institution	National Reference	189	10000
Saikia, et al	India	2018	2015-2016	cross-sectional	school	WHO	112	1096
Anebaracy, et al	India	2019	2018	cross-sectional	medical institution	National Reference	180	3081
Chandra, et al	India	2019	2018	cross-sectional	school	National Reference	134	544
Minhas, et al	India	2019	2014-2015	cross-sectional	school	CDC	57	1550
Mohan, et al	India	2019	2016-2017	cross-sectional	school	National Reference	125	1959
Sharma, et al	India	2019	2019	cross-sectional	community	CDC	118	1000
Swaminathan, et al	India	2019	2019	cross-sectional	school	CDC	298	2200
Singh, et al	India	2020	2020	cross-sectional	school	National Reference	144	1030
Singh, et al	India	2020	2016	cross-sectional	school	WHO	136	1237
Solanki, et al	India	2020	2011-2013	cross-sectional	school	CDC	487	10037
Ramesh, et al	India	2010	2008-2009	cross-sectional	school	CDC	108	1718
Sharma, et al	India	2009	2009	cross-sectional	medical institution	CDC	54	500
Singh, et al	India	2007	2007	cross-sectional	school	National Reference	60	1083
Senbanjo, et al	India	2007	2007	cross-sectional	community	WHO	14	270
Katzmarzyk, et al (8)	India	2015	2011-2013	cross-sectional	database	WHO	60	553
Kasi, et al	India	2015	2008-2009	cross-sectional	school	IOTF	25	1229
Joseph, et al	India	2015	2013	cross-sectional	school	National Reference	8	300
Basha, et al	India	2015	2015	cross-sectional	school	National Reference	109	1450
Weinmayr, et al (7)	India	2014	2004	cross-sectional	database	IOTF	0	119
Jani, et al	India	2014	2014	cross-sectional	medical institution	WHO	25	111
Ghosh, et al	India	2014	2008-2011	cross-sectional	school	National Reference	68	1061
Aroor, et al	India	2014	2011	cross-sectional	school	CDC	18	755
Honne, et al	India	2012	2012	cross-sectional	school	IOTF	16	463
Sakeenabi, et al	India	2012	2008	cross-sectional	school	National Reference	116	1550
Maiti, et al	India	2013	2010-2011	cross-sectional	school	IOTF	18	1375
Bhargava, et al	India	2016	2013-2014	cross-sectional	school	WHO	68	1266
Deepika, et al	India	2016	2013	cross-sectional	school	IOTF	88	877
Pawar, et al	India	2016	2014-2015	cross-sectional	school	IOTF	62	1828
Prasad, et al	India	2016	2014	cross-sectional	school	National Reference	105	2465
Saikia, et al	India	2016	2015	cross-sectional	school	WHO	73	752
Bharati, et	India	2017	2017	cross-sectional	school	CDC	1158	5216

© 2024 Zhang X et al. *JAMA Pediatrics.*

al								
Choudhary, et al	India	2017	2016-2017	cross-sectional	school	CDC	34	540
Eshwar, et al	India	2017	2015	cross-sectional	school	IOTF	77	1496
Ganie, et al	India	2017	2011-2013	cross-sectional	school	CDC	94	2024
Haq, et al	India	2017	2015	cross-sectional	medical institution	IOTF	15	303
Kumar, et al	India	2017	2017	cross-sectional	community	National Reference	40	1092
Mishra, et al	India	2017	2012-2013	cross-sectional	school	IOTF	10	300
Shah, et al	India	2017	2011-2015	cross-sectional	community	IOTF	206	1034
Andriani, et al	Indonesia	2021	2013	cross-sectional	database	WHO	4221	63237
Windiani, et al	Indonesia	2021	2019	cross-sectional	school	National Reference	61	468
Aditya, et al	Indonesia	2017	2015	cross-sectional	school	CDC	89	384
Agustina, et al	Indonesia	2021	2013	cross-sectional	database	WHO	5505	108890
Collins, et al	Indonesia	2008	2002-2003	cross-sectional	school	CDC	134	1736
Romano, et al (11)	Indonesia	2022	2015	cross-sectional	database	WHO	467	8806
Yuwanita, et al	Indonesia	2018	2015	cross-sectional	school	CDC	31	150
Febriani, et al	Indonesia	2019	2017	cross-sectional	school	WHO	37	145
Maehara, et al	Indonesia	2019	2017	cross-sectional	community	WHO	65	2160
Melinda, et al	Indonesia	2019	2015	cross-sectional	school	CDC	22	107
Rizkiriani, et al	Indonesia	2014	2014	cross-sectional	school	National Reference	13	213
Yulia, et al	Indonesia	2017	2016	cross-sectional	school	WHO	37	95
Moradi, et al	Iran	2016	2015	cross-sectional	school	WHO	290	2506
Soheilipour, et al	Iran	2022	2022	cross-sectional	school	WHO	121	829
Barati, et al	Iran	2022	2022	cross-sectional	school	CDC	219	1091
Mohammadi, et al	Iran	2021	2019	cross-sectional	school	National Reference	120	581
Entezarmahdi, et al	Iran	2021	2017	cross-sectional	community	WHO	34	2432
Jari, et al	Iran	2015	2009-2010	cross-sectional	database	WHO	104	1090
Bagherian, et al	Iran	2013	2009	cross-sectional	school	National Reference	99	400
Sadeghi, et al	Iran	2011	2008	cross-sectional	school	CDC	44	747
Shahraki, et al	Iran	2013	2013	cross-sectional	school	National Reference	62	1213
Agha-Alinejad, et al	Iran	2015	2011	cross-sectional	school	CDC	32	381
Javedan, et al	Iran	2016	2015	cross-sectional	school	WHO	1194	12796
Fatemeh, et al	Iran	2012	2008	cross-sectional	school	CDC	38	500
Ahmadi, et al	Iran	2014	2014	cross-sectional	school	CDC	105	1992
Badeli, et al	Iran	2016	2013-2015	cross-sectional	school	National Reference	72	2072
Bahreini, et al	Iran	2013	2010	cross-sectional	school	WHO	105	3002
Bahreynian, et al	Iran	2015	2011-2012	cross-sectional	school	WHO	1585	13322
Basiratnia, et al	Iran	2013	2010-2011	cross-sectional	school	CDC	140	2000
Maddah, et al	Iran	2010	2006-2007	cross-sectional	school	IOTF	360	6635
Kelishadi, et al	Iran	2008	2003-2004	cross-sectional	database	IOTF	612	21111
Amini, et al	Iran	2007	2000-2001	cross-sectional	school	CDC	39	398
Motlagh, et al	Iran	2011	2008	cross-sectional	database	CDC	29323	862433

© 2024 Zhang X et al. *JAMA Pediatrics.*

Ziaoddini, et al	Iran	2010	2007	cross-sectional	database	CDC	31467	899054
Ayatollahi, et al	Iran	2007	2002-2003	cross-sectional	school	CDC	102	2195
Khodaverdi, et al	Iran	2011	2007	cross-sectional	school	National Reference	35	240
Kajbaf, et al	Iran	2011	2009	cross-sectional	school	IOTF	62	903
Vafa, et al	Iran	2012	2008	cross-sectional	school	WHO	60	511
Zarrati, et al	Iran	2013	2011-2012	cross-sectional	school	WHO	63	1184
Hajian-Tilaki, et al	Iran	2012	2008	cross-sectional	school	CDC	100	1200
Gaeini, et al	Iran	2011	2007-2008	cross-sectional	community	CDC	35	755
Montazerifar, et al	Iran	2009	2005-2006	cross-sectional	school	CDC	9	590
Heidari, et al	Iran	2014	2010	cross-sectional	school	CDC	2865	12946
Hajian-Tilaki, et al	Iran	2011	2006	cross-sectional	school	CDC	58	1000
Gargari, et al	Iran	2004	2001	cross-sectional	school	IOTF	59	1518
Tebesh, et al	Iran	2013	2012-2013	cross-sectional	school	WHO	320	5811
Shafaghi, et al	Iran	2014	2010-2011	cross-sectional	school	WHO	138	1189
Moayeri, et al	Iran	2006	2004-2005	cross-sectional	school	CDC	201	2880
Behzadnia, et al	Iran	2012	2009-2010	cross-sectional	school	National Reference	78	653
Maddah, et al	Iran	2010	2006-2007	cross-sectional	school	IOTF	148	2577
Saeidlou, et al	Iran	2014	2011	cross-sectional	community	WHO	10	902
Kavehmanesh, et al	Iran	2013	2009-2011	case-control	community	National Reference	38	259
Hajian-Tilaki, et al	Iran	2013	2012	cross-sectional	school	CDC	114	760
Mirhosseini, et al	Iran	2012	2007	cross-sectional	school	WHO	16	477
Heshmat, et al	Iran	2015	2009-2010	cross-sectional	school	CDC	501	5625
Sadeghi, et al	Iran	2007	2007	cross-sectional	school	National Reference	425	633
Zekavat, et al	Iran	2014	2008-2009	cross-sectional	school	CDC	58	1156
Ejtahed, et al	Iran	2018	2015	cross-sectional	school	WHO	1615	14002
Salehi-Abargouei, et al	Iran	2013	2013	cross-sectional	school	WHO	63	837
Maddah, et al	Iran	2009	2006	cross-sectional	school	IOTF	109	2091
Taheri, et al	Iran	2013	2012	cross-sectional	school	CDC	142	1541
Zakeri, et al	Iran	2012	2006-2007	cross-sectional	database	CDC	538	8339
Jari, et al	Iran	2014	2009-2010	cross-sectional	database	WHO	486	5528
Sedaghat, et al	Iran	2019	2014-2015	cross-sectional	school	CDC	42	263
Rafraf, et al	Iran	2010	2008	cross-sectional	school	IOTF	28	985
Maddah, et al	Iran	2010	2006-2007	cross-sectional	school	IOTF	503	9046
Salem, et al	Iran	2009	2006-2007	cross-sectional	community	CDC	29	1221
Maddah, et al	Iran	2009	2005-2006	cross-sectional	school	IOTF	106	2255
Kelishadi, et al	Iran	2009	2006-2007	cross-sectional	school	CDC	295	1107
Fallahzadeh, et al	Iran	2009	2006	cross-sectional	school	CDC	104	800
Azita, et al	Iran	2009	2006	cross-sectional	school	National Reference	17	954
Bidad, et al	Iran	2008	2004-2005	cross-sectional	school	CDC	23	358
Rashidi, et al	Iran	2007	2000-2001	cross-sectional	school	CDC	186	2321
Mozaffari, et al	Iran	2007	2002	cross-sectional	school	IOTF	139	1800

© 2024 Zhang X et al. *JAMA Pediatrics.*

Maddah, et al	Iran	2007	2005	cross-sectional	school	IOTF	56	1054
Tabriz, et al	Iran	2015	2009-2013	cross-sectional	community	CDC	66	1151
Ghadimi, et al	Iran	2015	2012	cross-sectional	school	CDC	523	3647
Jalali-Farahani, et al	Iran	2014	2014	cross-sectional	school	WHO	57	465
Ahmadi, et al	Iran	2014	2010	cross-sectional	school	National Reference	18	145
Motlagh, et al (1)	Iran	2011	2007	cross-sectional	community	CDC	29323	862433
Motlagh, et al (2)	Iran	2011	2008	cross-sectional	community	CDC	27379	782244
Motlagh, et al (3)	Iran	2011	2009	cross-sectional	community	CDC	32483	955388
Hatami, et al	Iran	2013	2009-2010	cross-sectional	school	WHO	49	739
Keykhaei, et al	Iran	2016	2012	cross-sectional	school	CDC	129	585
Saeidlou, et al	Iran	2016	2009-2011	prospective	school	CDC	1243	51583
Salehiniya, et al	Iran	2016	2012	cross-sectional	database	WHO	1031	4656
Darabiyan, et al	Iran	2018	2017	cross-sectional	school	CDC	45	239
Ghobadi, et al	Iran	2018	2015-2016	cross-sectional	school	WHO	51	607
Heshmat, et al	Iran	2018	2015	cross-sectional	database	WHO	762	3843
Motlagh, et al	Iran	2018	2015-2016	cross-sectional	community	CDC	225	2444
Parastar, et al	Iran	2018	2016	cross-sectional	community	CDC	37	242
Abood, et al	Iraq	2021	2018-2019	cross-sectional	school	CDC	64	357
Lafta, et al	Iraq	2005	2002	cross-sectional	school	National Reference	111	8300
Amin, et al	Iraq	2019	2019	cross-sectional	school	CDC	168	6418
Lafta, et al	Iraq	2007	2005	cross-sectional	school	IOTF	218	5361
Musaiger, et al (1)	Iraq	2016	2013-2014	cross-sectional	database	IOTF	56	904
Reulbach, et al	Ireland	2013	2007-2008	cross-sectional	school	IOTF	727	8568
Quinn, et al	Ireland	2022	2011-2014	cohort	database	National Reference	296	4144
Heslin, et al (1)	Ireland	2023	2005-2006	cross-sectional	database	IOTF	8	440
Heslin, et al (2)	Ireland	2023	2019-2020	cross-sectional	database	IOTF	27	425
Barron, et al	Ireland	2009	2007	cross-sectional	school	IOTF	66	969
Dowd, et al	Ireland	2015	2006	cohort	community	IOTF	512	7914
Evans, et al	Ireland	2011	2004-2007	cross-sectional	school	IOTF	260	3482
Whelton, et al	Ireland	2007	2001	cross-sectional	school	IOTF	1259	19538
O'Neill, et al	Ireland	2007	2003-2004	cross-sectional	school	IOTF	27	375
Kelly, et al	Ireland	2019	2019	longitudinal	database	National Reference	470	8186
McMaster, et al	Ireland	2005	2001-2002	cross-sectional	school	IOTF	28	328
Janssen, et al (15)	Ireland	2005	2001-2002	cross-sectional	database	IOTF	27	1140
Belton, et al	Ireland	2010	2008	cross-sectional	school	IOTF	17	301
Haug, et al (33)	Ireland	2009	2005-2006	cross-sectional	database	IOTF	34	1566
Perry, et al	Ireland	2015	2007-2008	cross-sectional	school	IOTF	531	8136
Pinhas-Hamiel, et al	Israel	2009	2009	cross-sectional	school	CDC	31	204
Dayan, et al	Israel	2003	2003	cross-sectional	medical institution	National Reference	2897	76732
Janssen, et al (16)	Israel	2005	2001-2002	cross-sectional	database	IOTF	76	4200
Weisband,	Israel	2020	2018	cross-sectional	database	WHO	8131	116774

© 2024 Zhang X et al. *JAMA Pediatrics.*

et al								
Haug, et al (1)	Israel	2009	2005-2006	cross-sectional	database	IOTF	93	4037
Meyerovitch, et al	Israel	2007	2001-2004	cross-sectional	medical institution	CDC	232	949
Goldberg, et al	Israel	2014	2014	cross-sectional	database	National Reference	13195	404922
Kaufman-Shriqui, et al	Israel	2013	2008	cross-sectional	school	WHO	27	238
Ram, et al	Israel	2013	2009	cross-sectional	school	IOTF	173	4130
Ahrens, et al (1)	Italy	2014	2007-2008	cross-sectional	database	IOTF	482	2424
Galfo, et al	Italy	2022	2011-2012	cross-sectional	database	IOTF	29	369
Salas, et al (7)	Italy	2021	2015-2017	cross-sectional	database	WHO	7139	42496
Ashi, et al (1)	Italy	2019	2019	cross-sectional	school	WHO	15	220
Costacurta, et al	Italy	2011	2011	cross-sectional	medical institution	National Reference	14	107
Barba, et al	Italy	2006	2003-2004	cross-sectional	school	IOTF	820	3923
Ceschia, et al	Italy	2016	2016	cross-sectional	school	IOTF	166	2411
Toselli, et al	Italy	2014	2007-2008	cross-sectional	school	IOTF	115	1432
Caserta, et al	Italy	2010	2007-2008	cross-sectional	school	IOTF	82	575
Genovesi, et al	Italy	2010	2004-2006	cross-sectional	school	IOTF	326	5131
Bertoncello, et al	Italy	2008	2004	cross-sectional	school	IOTF	712	12853
Albertini, et al	Italy	2008	2002-2005	cross-sectional	school	IOTF	504	5636
Maffeis, et al	Italy	2006	2002	cross-sectional	school	IOTF	172	2150
Parrino, et al	Italy	2012	2009-2010	cross-sectional	school	IOTF	252	915
Parrino, et al	Italy	2016	2016	cross-sectional	school	IOTF	220	1521
Lombardo, et al	Italy	2015	2010	cross-sectional	database	WHO	8402	41592
Janssen, et al (17)	Italy	2005	2001-2002	cross-sectional	database	IOTF	102	4095
Valerio, et al	Italy	2003	2003	cross-sectional	school	IOTF	67	587
Pecoraro, et al	Italy	2003	2003	cross-sectional	school	IOTF	50	228
Collo, et al	Italy	2019	2016-2017	cross-sectional	medical institution	WHO	6	86
Toselli, et al	Italy	2010	2010	cross-sectional	school	IOTF	27	598
Greco, et al	Italy	2020	2016-2017	cross-sectional	school	IOTF	177	1122
Binkin, et al	Italy	2010	2008	cross-sectional	school	IOTF	6147	50197
Rapa, et al	Italy	2009	2007-2008	cross-sectional	medical institution	National Reference	15	88
Haug, et al (12)	Italy	2009	2005-2006	cross-sectional	database	IOTF	78	3556
Caserta, et al	Italy	2009	2007-2008	cross-sectional	school	IOTF	87	642
Bonaccorsi, et al	Italy	2009	2005	cross-sectional	school	National Reference	10	449
Turconi, et al	Italy	2008	2002	cross-sectional	school	IOTF	15	532
Maffeis, et al	Italy	2008	2003	cross-sectional	school	IOTF	93	1836
Lazzeri, et al (1)	Italy	2008	2002	cross-sectional	school	IOTF	271	3048
Lazzeri, et al (2)	Italy	2008	2006	cross-sectional	school	IOTF	119	1430
Lazzeri, et al (3)	Italy	2008	2004	cross-sectional	school	IOTF	36	1066
Lazzeri, et al (4)	Italy	2008	2006	cross-sectional	school	IOTF	32	997
Lazzeri, et al (5)	Italy	2008	2004	cross-sectional	school	IOTF	39	1185

© 2024 Zhang X et al. *JAMA Pediatrics*.

Lazzeri, et al (6)	Italy	2008	2006	cross-sectional	school	IOTF	35	1086
Lazzeri, et al (7)	Italy	2008	2004	cross-sectional	school	IOTF	15	1160
Lazzeri, et al (8)	Italy	2008	2006	cross-sectional	school	IOTF	28	1078
Guida, et al	Italy	2008	2001	cross-sectional	school	IOTF	111	464
Gualdi-Russo, et al	Italy	2008	2000-2001	cross-sectional	database	IOTF	93	866
Fuiano, et al	Italy	2008	2005	longitudinal	school	National Reference	44	632
Vidal, et al	Italy	2006	2006	cross-sectional	community	IOTF	10	258
Menghetti, et al	Italy	2015	2015	cross-sectional	school	IOTF	134	2007
Lazzeri, et al (1)	Italy	2015	2002	cross-sectional	database	IOTF	149	1485
Lazzeri, et al (2)	Italy	2015	2006	cross-sectional	database	IOTF	140	1405
Lazzeri, et al (3)	Italy	2015	2008	cross-sectional	database	IOTF	102	1375
Lazzeri, et al (4)	Italy	2015	2010	cross-sectional	database	IOTF	102	1424
Lazzeri, et al (5)	Italy	2015	2012	cross-sectional	database	IOTF	100	1494
Geremia, et al	Italy	2015	2015	cross-sectional	school	WHO	49	590
Weinmayr, et al (8)	Italy	2014	2004	cross-sectional	database	IOTF	137	1307
Lazzeri, et al	Italy	2014	2009-2010	cross-sectional	database	IOTF	1275	47583
Sacchetti, et al	Italy	2012	2012	cross-sectional	school	IOTF	48	489
Bracale, et al	Italy	2013	2008	cross-sectional	community	IOTF	657	16588
Petracci, et al	Italy	2013	2003	cross-sectional	database	IOTF	210	4338
Galfo, et al	Italy	2016	2011-2012	cross-sectional	database	IOTF	29	369
Grassi, et al	Italy	2016	2014-2015	cross-sectional	database	IOTF	188	1164
Romano, et al (12)	Jamaica	2022	2017	cross-sectional	database	WHO	107	1061
Xu, et al (2)	Japan	2021	2014	cross-sectional	school	WHO	57	1200
Tani, et al	Japan	2021	2018	cross-sectional	database	WHO	290	5257
Watanabe, et al	Japan	2011	2003	cross-sectional	school	IOTF	30	1765
Oishi, et al	Japan	2021	2018-2019	cross-sectional	school	National Reference	505	7277
Nakano, et al	Japan	2010	2001	longitudinal	school	IOTF	791	16245
Suzuki, et al	Japan	2009	2000-2001	cohort	database	IOTF	58	1302
Yamashita, et al	Japan	2023	2001	longitudinal	database	IOTF	662	32081
Tomata, et al	Japan	2019	2015	cross-sectional	medical institution	IOTF	126	1848
Sasaki, et al	Japan	2010	2008	cross-sectional	community	IOTF	37	449
Okuda, et al	Japan	2010	2006-2008	cross-sectional	school	IOTF	152	3812
Ochiai, et al	Japan	2010	2004-2008	cross-sectional	school	CDC	177	3750
Shinozaki, et al	Japan	2015	2006-2010	cross-sectional	database	IOTF	90	5600
Ochiai, et al	Japan	2015	2004-2009	cross-sectional	school	IOTF	69	2499
Mizuta, et al	Japan	2016	2012-2013	cross-sectional	school	WHO	27	2968
Baker, et al	Jordan	2010	2007	cross-sectional	school	CDC	118	1355
Hamaideh, et al	Jordan	2010	2010	cross-sectional	school	IOTF	52	824
Ibrahim, et al	Jordan	2008	2006	cross-sectional	school	WHO	338	1695
Khader, et al	Jordan	2009	2006	cross-sectional	school	IOTF	118	2131
Al-Akour, et al	Jordan	2012	2009	cross-sectional	school	CDC	112	1433
Al-Domi, et al	Jordan	2019	2009-2011	cross-sectional	school	IOTF	129	767
Al-Kloub, et al	Jordan	2010	2008	cross-sectional	school	IOTF	50	518

© 2024 Zhang X et al. *JAMA Pediatrics.*

Tayyem, et al	Jordan	2014	2009	cross-sectional	database	IOTF	52	735
Musaiger, et al (2)	Jordan	2012	2010-2011	cross-sectional	school	IOTF	69	937
Musaiger, et al	Jordan	2013	2013	cross-sectional	school	IOTF	22	475
Musaiger, et al (2)	Jordan	2016	2013-2014	cross-sectional	database	IOTF	43	795
Zayed, et al	Jordan	2016	2015-2016	cross-sectional	school	CDC	405	2702
Salas, et al (8)	Kazakhstan	2021	2015-2017	cross-sectional	database	WHO	227	3988
Facchini, et al (1)	Kazakhstan	2007	2002-2004	cross-sectional	database	CDC	14	2400
Kyallo, et al	Kenya	2013	2008	cross-sectional	school	WHO	19	321
Muthuri, et al	Kenya	2014	2012	cross-sectional	school	WHO	36	563
Gewa, et al	Kenya	2010	2003	cross-sectional	database	WHO	55	1443
Katzmarzyk, et al (9)	Kenya	2015	2011-2013	cross-sectional	database	WHO	33	499
Pengpid, et al (2)	Kiribati	2015	2010-2011	cross-sectional	database	IOTF	117	1582
Abdelalim, et al	Kuwait	2012	2012	cross-sectional	community	CDC	186	1006
Alqaoud, et al	Kuwait	2022	2007-2019	cross-sectional	database	WHO	1252	47782
Al-Haifi, et al	Kuwait	2022	2019	cross-sectional	school	IOTF	179	706
Badr, et al	Kuwait	2017	2010-2011	cross-sectional	database	CDC	650	2672
El-Ghaziri, et al	Kuwait	2011	2011	cross-sectional	school	CDC	213	499
El-Bayoumy, et al	Kuwait	2009	2006	cross-sectional	school	National Reference	787	5402
AlRodhan, et al	Kuwait	2019	2019	cross-sectional	school	CDC	542	2208
Al-Qaoud, et al	Kuwait	2009	2003-2004	cross-sectional	school	CDC	277	2291
Al-Refaee, et al	Kuwait	2013	2012	cross-sectional	medical institution	CDC	77	361
Musaiger, et al (1)	Kuwait	2013	2010-2011	cross-sectional	database	IOTF	168	628
Musaiger, et al (3)	Kuwait	2016	2013-2014	cross-sectional	database	IOTF	175	706
Salas, et al (9)	Kyrgyzstan	2021	2015-2017	cross-sectional	database	WHO	161	5958
Romano, et al (13)	Laos	2022	2015	cross-sectional	database	WHO	36	1644
Salas, et al (11)	Latvia	2021	2015-2017	cross-sectional	database	WHO	453	5593
Janssen, et al (18)	Latvia	2005	2001-2002	cross-sectional	database	IOTF	15	3091
Haug, et al (34)	Latvia	2009	2005-2006	cross-sectional	database	IOTF	34	3778
Weinmayr, et al (9)	Latvia	2014	2004	cross-sectional	database	IOTF	3	156
Karklina, et al	Latvia	2011	2007-2009	cross-sectional	school	National Reference	32	504
Nasreddine, et al	Lebanon	2014	2009	cross-sectional	community	WHO	63	368
Chacar, et al	Lebanon	2011	2007	cross-sectional	school	IOTF	167	2547
Romano, et al (14)	Lebanon	2022	2017	cross-sectional	database	WHO	228	3347
Jabre, et al	Lebanon	2005	2000	cross-sectional	community	IOTF	17	234
Fazah, et al	Lebanon	2010	2010	cross-sectional	school	IOTF	43	982
Chakar, et al	Lebanon	2006	2002-2003	cross-sectional	school	IOTF	908	12128
Nasreddine, et al	Lebanon	2017	2011-2012	cross-sectional	community	WHO	14	525
Gebremedhin, et al (12)	Liberia	2015	2013	cross-sectional	database	WHO	55	3259
Taguri, et al (5)	Libya	2009	2003	cross-sectional	database	WHO	963	7232

© 2024 Zhang X et al. *JAMA Pediatrics*.

Musaiger, et al (3)	Libya	2012	2010-2011	cross-sectional	school	IOTF	62	630
Musaiger, et al (2)	Libya	2013	2010-2011	cross-sectional	database	IOTF	62	630
Musaiger, et al (4)	Libya	2016	2013-2014	cross-sectional	database	IOTF	59	759
Salas, et al (10)	Lithuania	2021	2015-2017	cross-sectional	database	WHO	357	3431
Smetanina, et al	Lithuania	2015	2008-2010	cross-sectional	school	IOTF	165	3990
Janssen, et al (19)	Lithuania	2005	2001-2002	cross-sectional	database	IOTF	17	4194
Haug, et al (35)	Lithuania	2009	2005-2006	cross-sectional	database	IOTF	34	3830
Wijnhoven, et al (3)	Lithuania	2015	2007/2008	cross-sectional	school	WHO	339	4084
Haug, et al (7)	Luxemburg	2009	2005-2006	cross-sectional	database	IOTF	90	3904
Raufi, et al	Macedonia	2022	2019-2020	cross-sectional	medical institution	CDC	202	1034
Janssen, et al (20)	Macedonia	2005	2001-2002	cross-sectional	database	IOTF	86	3596
Haug, et al (13)	Macedonia	2009	2005-2006	cross-sectional	database	IOTF	103	4911
Myrtaj, et al	Macedonia	2018	2016	cross-sectional	school	IOTF	117	2390
Manyanga, et al (5)	Malawi	2014	2014	cross-sectional	database	WHO	18	2305
Gebremedhin, et al (13)	Malawi	2015	2010	cross-sectional	database	WHO	280	4829
Mohamed, et al	Malaysia	2023	2014-2015	cross-sectional	school	IOTF	98	917
Lai, et al	Malaysia	2022	2019	cross-sectional	school	WHO	331	2221
Al-Sadat, et al	Malaysia	2016	2012	cross-sectional	database	IOTF	116	1350
Khor, et al	Malaysia	2011	2008	cross-sectional	school	WHO	66	402
Adeyemi, et al	Malaysia	2014	2014	cross-sectional	school	IOTF	27	411
Ahmad, et al	Malaysia	2017	2014-2015	cross-sectional	database	WHO	7508	62567
Tee, et al	Malaysia	2018	2013	cross-sectional	school	WHO	1200	8322
Kee, et al	Malaysia	2017	2013/2014	cross-sectional	database	WHO	44	663
Moy, et al	Malaysia	2004	2004	cross-sectional	school	WHO	260	3556
Mahaletchumy, et al	Malaysia	2019	2016	cross-sectional	school	WHO	716	6248
Cheah, et al	Malaysia	2019	2019	cross-sectional	school	WHO	300	1314
Tan, et al	Malaysia	2019	2012	cross-sectional	database	WHO	2414	24339
Mohd, et al	Malaysia	2006	2006	cross-sectional	school	IOTF	50	699
Woon, et al	Malaysia	2015	2015	cross-sectional	school	WHO	44	333
Loh, et al	Malaysia	2015	2013	cross-sectional	school	IOTF	68	646
Kaartina, et al	Malaysia	2015	2011	cross-sectional	school	WHO	44	379
Su, et al	Malaysia	2014	2014	cross-sectional	school	IOTF	116	1327
Poh, et al	Malaysia	2016	2015	cross-sectional	database	WHO	400	3227
Shariff, et al	Malaysia	2016	2016	cross-sectional	community	WHO	60	745
Partap, et al	Malaysia	2017	2013-2014	cross-sectional	database	IOTF	584	6414
Gebremedhin, et al (14)	Mali	2015	2012/2013	cross-sectional	database	WHO	101	4591
Salas, et al (12)	Malta	2021	2015-2017	cross-sectional	database	WHO	536	3115
Decelis, et al	Malta	2014	2012	cross-sectional	database	IOTF	115	811
Janssen, et al (21)	Malta	2005	2001-2002	cross-sectional	database	IOTF	84	1065
Haug, et al (14)	Malta	2009	2005-2006	cross-sectional	database	IOTF	111	1039
Decelis, et al	Malta	2013	2012	cross-sectional	school	IOTF	124	874
Manyanga, et al (6)	Mauritania	2014	2014	cross-sectional	database	WHO	69	2028
Caleyachetty, et al	Mauritius	2012	2006	cross-sectional	school	IOTF	42	841
Fokeena, et al	Mauritius	2012	2012	cross-sectional	school	CDC	13	200

© 2024 Zhang X et al. *JAMA Pediatrics.*

Romano, et al (15)	Mauritius	2022	2017	cross-sectional	database	WHO	180	1955
Shamah-Levy, et al (1)	Mexico	2022	2006	cross-sectional	database	WHO	2346	14990
Shamah-Levy, et al (2)	Mexico	2022	2012	cross-sectional	database	WHO	2453	16351
Shamah-Levy, et al (3)	Mexico	2022	2016	cross-sectional	database	WHO	431	3179
Shamah-Levy, et al (4)	Mexico	2022	2018	cross-sectional	database	WHO	1019	6183
Shamah-Levy, et al (5)	Mexico	2022	2020	cross-sectional	database	WHO	351	1944
Shamah-Levy, et al (6)	Mexico	2022	2021	cross-sectional	database	WHO	482	2569
Brambila-Paz, et al	Mexico	2022	2002	longitudinal	database	WHO	14	3202
Aguilera-Galaviz, et al	Mexico	2019	2016	cross-sectional	school	WHO	10	203
Ashi, et al (2)	Mexico	2019	2019	cross-sectional	school	WHO	46	224
Pérez, et al	Mexico	2020	2018	cross-sectional	school	WHO	105	522
Irigoyen-Camacho, et al	Mexico	2014	2010-2011	cross-sectional	school	IOTF	14	257
Patiño-Marín, et al	Mexico	2018	2013-2016	cross-sectional	school	WHO	355	1527
Sánchez-Pérez, et al	Mexico	2010	2010	longitudinal	school	CDC	15	88
Serrano-Piña, et al	Mexico	2019	2019	cross-sectional	school	National Reference	46	331
Vázquez-Nava, et al	Mexico	2010	2005	cross-sectional	school	CDC	372	1160
Ávila-Ortiz, et al	Mexico	2017	2011-2013	cross-sectional	school	CDC	35	91
Bacardi-Gascón, et al	Mexico	2009	2006-2007	cross-sectional	school	National Reference	465	1684
Lopez-Gonzalez, et al	Mexico	2020	2015-2019	cross-sectional	community	CDC	235	1449
Del-Rio-Navarro, et al	Mexico	2008	2005	cross-sectional	school	CDC	446	1819
Gómez-Díaz, et al	Mexico	2005	2005	cross-sectional	school	IOTF	42	833
Brewis, et al	Mexico	2003	2001	cross-sectional	school	CDC	53	219
Vergara-Castañeda, et al	Mexico	2010	2010	cross-sectional	school	National Reference	9	83
Jimenez-Cruz, et al	Mexico	2010	2009	cross-sectional	medical institution	WHO	82	1685
Macías-Rosales, et al	Mexico	2009	2004	cross-sectional	medical institution	CDC	78	641
Flores-Huerta, et al	Mexico	2009	2005-2006	cross-sectional	school	CDC	363	2029
Ortiz-Hernández, et al	Mexico	2008	2005	cross-sectional	school	CDC	188	1015
Basaldúa, et al	Mexico	2008	2004	cross-sectional	school	IOTF	73	551
Villa-Caballero, et al	Mexico	2006	2001-2002	cross-sectional	school	CDC	263	1172
RODRÍGUEZ-	Mexico	2015	2011	cross-sectional	school	CDC	25	104

© 2024 Zhang X et al. *JAMA Pediatrics.*

FUENTES, et al								
López-Barrón, et al	Mexico	2015	2010-2011	cross-sectional	school	WHO	177	684
Flores, et al	Mexico	2015	2008	cross-sectional	medical institution	WHO	66	164
Banik, et al	Mexico	2014	2008-2009	cross-sectional	school	WHO	26	321
Banik, et al	Mexico	2014	2014	cross-sectional	school	WHO	29	283
Barrera, et al	Mexico	2016	2012-2013	cross-sectional	school	WHO	150	725
Caraza, et al	Mexico	2016	2013-2014	cross-sectional	school	CDC	87	605
Peña, et al	Mexico	2018	2018	cross-sectional	school	WHO	243	849
Ramírez-Jiménez ,et al	Mexico	2018	2011	cross-sectional	community	WHO	11	93
Romano, et al (16)	Mongolia	2022	2013	cross-sectional	database	WHO	67	3707
Salas, et al (13)	Montenegro	2021	2015-2017	cross-sectional	database	WHO	343	2678
Banjari, et al (1)	Montenegro	2020	2020	cross-sectional	school	IOTF	24	224
Martinovic, et al	Montenegro	2015	2012-2013	cross-sectional	school	IOTF	216	4097
El Moussaoui, et al	Morocco	2022	2020	cross-sectional	medical institution	WHO	84	450
Dekkaki, et al	Morocco	2011	2010	cross-sectional	school	WHO	56	1570
Manyanga, et al (7)	Morocco	2014	2014	cross-sectional	database	WHO	207	5756
Taguri, et al (2)	Morocco	2009	2003-2004	cross-sectional	database	WHO	832	5380
El Kabbaoui, et al	Morocco	2018	2014-2015	cross-sectional	school	WHO	62	1818
Gebremedhin, et al (15)	Mozambique	2015	2011	cross-sectional	database	WHO	408	9721
Qiao, et al	Multiple Countries	2015	2011-2013	cross-sectional	database	WHO	639	4502
Bishwajt, et al	Multiple Countries	2019	2014-2018	cross-sectional	database	WHO	5818	132231
Stival, et al (1)	Multiple Countries	2022	2019	cross-sectional	database	WHO	148	3093
Stival, et al (2)	Multiple Countries	2022	2018	cross-sectional	database	WHO	52	2916
Moschonis, et al	Multiple Countries	2022	2016-2018	cross-sectional	database	IOTF	902	12030
Ferrari, et al	Multiple Countries	2022	2014-2015	cross-sectional	database	WHO	51	671
Collings, et al	Multiple Countries	2022	2022	cross-sectional	database	National Reference	2205	15810
Peltzer, et al	Multiple Countries	2011	2003/2007	cross-sectional	school	IOTF	40	5613
Vrijheid, et al	Multiple Countries	2020	2013/2016	cohort	database	WHO	129	1301
Roy, et al	Multiple Countries	2020	2003-2014	cohort	database	WHO	276	3679
Ekelund, et al	Multiple Countries	2004	2004	cross-sectional	school	IOTF	39	1292
Olaya, et al	Multiple Countries	2015	2010	cross-sectional	database	IOTF	255	5206
Katzmarzyk, et al (1)	Multiple Countries	2015	2011-2013	cross-sectional	database	WHO	815	6539
Tsitsika, et al	Multiple Countries	2016	2011-2012	cross-sectional	school	IOTF	183	10287
Qiao, et al	Multiple Countries	2017	2013	cross-sectional	database	WHO	626	5088
Gebremedhin, et al (16)	Namibia	2015	2013	cross-sectional	database	WHO	42	1845
Romano, et al (17)	Namibia	2022	2013	cross-sectional	database	WHO	37	1936
Achaya, et	Nepal	2016	2013	cross-sectional	school	WHO	19	838

© 2024 Zhang X et al. *JAMA Pediatrics*.

al								
Romano, et al (18)	Nepal	2022	2015	cross-sectional	database	WHO	23	4616
Bhattarai, et al	Nepal	2019	2017	cross-sectional	school	CDC	4	510
Karki, et al	Nepal	2019	2017	cross-sectional	school	WHO	41	575
Koirala, et al	Nepal	2015	2013	cross-sectional	school	WHO	111	986
Gurung, et al	Nepal	2014	2010	cross-sectional	school	IOTF	10	300
Pandey, et al	Nepal	2018	2018	cross-sectional	school	WHO	4	120
Greeff, et al	Netherlands	2016	2016	RCT	school	IOTF	19	376
Jabakhanji, et al	Netherlands	2022	2013	cohort	database	IOTF	417	7042
Sergentanis, et al (2)	Netherlands	2021	2011-2012	cross-sectional	database	IOTF	9	727
Croezen, et al	Netherlands	2009	2003	cross-sectional	database	IOTF	244	25176
Drukker, et al	Netherlands	2009	2004-2005	cross-sectional	database	IOTF	44	1411
Jansen, et al	Netherlands	2012	2004-2010	cross-sectional	database	IOTF	48	3157
Wilde, et al	Netherlands	2009	2007	cross-sectional	database	IOTF	547	10308
Yngve, et al (5)	Netherlands	2008	2003	cross-sectional	database	IOTF	5	684
Akbulut, et al (2)	Netherlands	2014	2003-2011	cross-sectional	database	IOTF	39	433
Jansen, et al	Netherlands	2008	2000-2001	cross-sectional	database	IOTF	135	1923
Janssen, et al (22)	Netherlands	2005	2001-2002	cross-sectional	database	IOTF	31	3860
Wilde, et al	Netherlands	2019	2007-2014	cross-sectional	medical institution	IOTF	4167	109769
Luttikhuis, et al	Netherlands	2010	2006	cross-sectional	school	IOTF	16	397
Jansen, et al	Netherlands	2010	2006	cross-sectional	school	IOTF	99	1095
Haug, et al (8)	Netherlands	2009	2005-2006	cross-sectional	database	IOTF	39	3850
Bossink-Tuna, et al	Netherlands	2009	2006	cross-sectional	medical institution	IOTF	5	635
Snoek, et al	Netherlands	2007	2007	cross-sectional	school	IOTF	77	9011
Scholtens, et al	Netherlands	2007	2000-2001	cross-sectional	database	IOTF	23	864
Jansen, et al	Netherlands	2006	2000-2001	cross-sectional	database	IOTF	135	1819
Weinmayr, et al (10)	Netherlands	2014	2004	cross-sectional	database	IOTF	98	2638
Schwiebbe, et al	Netherlands	2011	2008	cross-sectional	school	IOTF	253	2148
Veldwijk, et al	Netherlands	2012	2003-2007	cross-sectional	school	IOTF	518	51856
Willeboordse, et al	Netherlands	2013	2010	cross-sectional	community	National Reference	225	9272
Leppers, et al	Netherlands	2017	2012-2015	cross-sectional	medical institution	IOTF	79	3408
Velde, et al (4)	Netherlands	2017	2010	cross-sectional	database	IOTF	31	919
Utter, et al	New Zealand	2006	2002	cross-sectional	database	IOTF	246	2716
Hobbs, et al	New Zealand	2021	2013-2017	cross-sectional	database	IOTF	3751	9728
Aung, et al	New Zealand	2021	2014-2015	cross-sectional	school	National Reference	2964	27333
Chelimo, et al	New Zealand	2020	2007-2010	cohort	medical institution	IOTF	437	5128
Rockell, et al	New Zealand	2005	2002	cross-sectional	school	IOTF	517	3275
Gordon, et al	New Zealand	2003	2000	cross-sectional	community	IOTF	17	41
Utter, et al	New Zealand	2010	2007	cross-sectional	school	IOTF	897	8796

© 2024 Zhang X et al. *JAMA Pediatrics*.

Quigg, et al	New Zealand	2010	2007	cross-sectional	school	CDC	19	184
Weinmayr, et al (11)	New Zealand	2014	2004	cross-sectional	database	IOTF	25	222
Rajput, et al	New Zealand	2014	2009-2012	cross-sectional	database	WHO	27505	168744
Gebremedhin, et al (17)	Niger	2015	2012	cross-sectional	database	WHO	179	5123
Adeomi, et al	Nigeria	2019	2019	cross-sectional	school	WHO	4	309
Adetunji, et al	Nigeria	2019	2013	cross-sectional	school	WHO	17	1187
Maruf, et al	Nigeria	2013	2009	cross-sectional	school	IOTF	71	9014
Ene-obong, et al	Nigeria	2012	2012	cross-sectional	school	IOTF	44	1599
Opara, et al	Nigeria	2010	2009	cross-sectional	school	WHO	56	985
Gebremedhin, et al (18)	Nigeria	2015	2013	cross-sectional	database	WHO	1097	26758
Goon, et al	Nigeria	2010	2005	cross-sectional	school	CDC	45	2015
Wariri, et al	Nigeria	2020	2015-2017	cross-sectional	community	WHO	63	2100
Mezie-Okoye, et al	Nigeria	2015	2010	cross-sectional	school	CDC	19	220
Musa, et al	Nigeria	2012	2012	cross-sectional	school	IOTF	73	3240
Oduwole, et al	Nigeria	2012	2012	cross-sectional	school	CDC	83	885
Sadoh, et al	Nigeria	2016	2011-2012	cross-sectional	school	CDC	65	353
Igbokwe, et al	Nigeria	2017	2013	cross-sectional	school	WHO	35	860
Aadland, et al	Norway	2021	2015-2016	cross-sectional	community	IOTF	29	1182
Aadland, et al	Norway	2022	2019-2022	cross-sectional	community	IOTF	28	1003
Donkor, et al	Norway	2017	2007	cohort	school	IOTF	74	1864
Øvrebø, et al	Norway	2021	2017	longitudinal	database	IOTF	46	1838
Andersen, et al	Norway	2005	2000	cross-sectional	school	IOTF	38	1489
Bernhardsen, et al	Norway	2019	2013-2015	cohort	database	IOTF	6	445
Oellingrath, et al	Norway	2010	2007	cross-sectional	school	IOTF	37	955
Júlíusson, et al	Norway	2010	2003-2006	cross-sectional	community	IOTF	140	6140
Kolle, et al	Norway	2009	2005	cross-sectional	database	IOTF	13	440
Júlíusson, et al (2)	Norway	2015	2015	cross-sectional	database	IOTF	142	6139
Yngve, et al (6)	Norway	2008	2003	cross-sectional	database	IOTF	8	670
Hestetun, et al	Norway	2015	2010	cross-sectional	school	IOTF	22	744
Janssen, et al (23)	Norway	2005	2001-2002	cross-sectional	database	IOTF	97	4415
Haug, et al (36)	Norway	2009	2005-2006	cross-sectional	database	IOTF	51	3675
Grøholt, et al	Norway	2008	2000-2004	cross-sectional	school	IOTF	299	15966
Weinmayr, et al (12)	Norway	2014	2004	cross-sectional	database	IOTF	25	637
Brannsether, et al	Norway	2014	2003-2006	cross-sectional	database	IOTF	118	4576
Velde, et al (5)	Norway	2017	2010	cross-sectional	database	IOTF	14	1000
Tanveer, et al	Pakistan	2022	2021	cross-sectional	school	CDC	191	3551
Bekhwani, et al	Pakistan	2022	2020-2021	cross-sectional	medical institution	CDC	80	184
Ahmed, et al	Pakistan	2013	2008-2009	cross-sectional	school	WHO	58	501
Mansoori, et al	Pakistan	2018	2012	cross-sectional	school	CDC	96	887
Anwar, et al	Pakistan	2010	2009	cross-sectional	school	WHO	64	293
Hashmi, et	Pakistan	2013	2008-2009	cross-sectional	school	WHO	58	501

© 2024 Zhang X et al. *JAMA Pediatrics.*

al								
Ramzan, et al	Pakistan	2008	2008	cross-sectional	school	CDC	75	1136
Aziz, et al	Pakistan	2009	2007	cross-sectional	school	WHO	24	398
Haq, et al	Pakistan	2010	2010	cross-sectional	school	National Reference	153	3200
Aziz, et al	Pakistan	2012	2006-2009	cross-sectional	school	CDC	664	12837
Basit,et al	Pakistan	2005	2001	cross-sectional	school	National Reference	5	92
Ishaque, et al	Pakistan	2012	2012	cross-sectional	school	WHO	47	431
Abudayya, et al	Palestine	2007	2002	cross-sectional	school	WHO	55	1022
Badrasawi, et al	Palestine	2019	2017	cross-sectional	school	WHO	13	392
Al-Lahham, et al	Palestine	2019	2016	cross-sectional	school	CDC	207	1320
Mikki, et al	Palestine	2009	2007-2008	cross-sectional	school	IOTF	59	1942
Weinmayr, et al (13)	Palestine	2014	2004	cross-sectional	database	IOTF	10	216
Musaiger, et al (3)	Palestine	2013	2010-2011	cross-sectional	database	IOTF	20	477
Massad, et al	Palestine	2016	2009	cross-sectional	school	CDC	86	1484
Musaiger, et al (5)	Palestine	2016	2013-2014	cross-sectional	database	IOTF	38	610
Bartkowiak, et al	Peru	2021	2021	cross-sectional	school	WHO	150	681
Carrillo-Larco, et al (1)	Peru	2014	2002	cohort	database	IOTF	104	1929
Romano, et al (19)	Peru	2022	2010	cross-sectional	database	WHO	68	2359
Hernández-Vásquez, et al	Peru	2016	2015	cross-sectional	community	WHO	35493	2336791
Romano, et al (20)	Philippines	2022	2015	cross-sectional	database	WHO	173	6162
Suligowska, et al	Poland	2022	2017-2019	cross-sectional	database	WHO	29	381
Potempa-Jeziorowska, et al	Poland	2022	2018-2019	cross-sectional	school	WHO	62	908
Kryst, et al (1)	Poland	2022	2009-2010	cross-sectional	school	IOTF	83	1926
Kryst, et al (2)	Poland	2022	2019-2020	cross-sectional	school	IOTF	69	1850
Bryl, et al	Poland	2022	2017-2019	cross-sectional	school	IOTF	28	530
Basiak-Rasała, et al	Poland	2022	2016	cross-sectional	school	WHO	117	2913
Wieniawski, et al	Poland	2021	2021	cross-sectional	school	National Reference	59	690
Usheva, et al (5)	Poland	2021	2012	cross-sectional	database	WHO	30	1334
Sergentanis, et al (4)	Poland	2021	2011-2012	cross-sectional	database	IOTF	23	1190
Salas, et al (14)	Poland	2021	2015-2017	cross-sectional	database	WHO	363	2884
Matłosz, et al	Poland	2021	2018-2019	cross-sectional	school	CDC	51	1172
Żegleń, et al (1)	Poland	2020	2008	cross-sectional	school	IOTF	63	1113
Żegleń, et al (2)	Poland	2020	2018	cross-sectional	school	IOTF	14	1054
Szczyrska, et al	Poland	2020	2007-2016	cross-sectional	database	National Reference	522	12330
Tomaszewski, et al	Poland	2015	2015	cross-sectional	school	IOTF	39	791
Bac, et al	Poland	2012	2008-2009	cross-sectional	community	IOTF	77	1495
Baran, et al	Poland	2022	2015	cross-sectional	school	National Reference	46	749
Kułaga, et	Poland	2011	2007-2009	cross-sectional	database	IOTF	522	15607

© 2024 Zhang X et al. *JAMA Pediatrics.*

al								
Malecka-Tendera, et al	Poland	2005	2001	cross-sectional	school	IOTF	106	2916
Kowal,et al (1)	Poland	2014	2000	cross-sectional	school	IOTF	23	2062
Kowal,et al (2)	Poland	2014	2010	cross-sectional	school	IOTF	62	1970
Chrzanowska, et al	Poland	2007	2000	cross-sectional	school	IOTF	51	2003
Ządzińska, et al	Poland	2013	2002-2004	cross-sectional	community	IOTF	21	812
Gorog, et al (4)	Poland	2011	2011	cross-sectional	database	IOTF	44	1454
Høyer, et al (2)	Poland	2014	2002-2005	cohort	database	WHO	5	92
Zatoński, et al	Poland	2020	2016-2017	cross-sectional	school	National Reference	114	2913
Janssen, et al (24)	Poland	2005	2001-2002	cross-sectional	database	IOTF	65	5909
Woźniacka, et al	Poland	2018	2009-2010	cross-sectional	school	IOTF	211	3405
Baran, et al	Poland	2019	2019	cross-sectional	school	National Reference	10	300
Jodkowska, et al	Poland	2010	2005	cross-sectional	school	IOTF	153	8065
Haug, et al (22)	Poland	2009	2005-2006	cross-sectional	database	IOTF	80	5324
Zawodniak-Szałapska, et al	Poland	2007	2007	cross-sectional	school	National Reference	183	1012
Klimek-Piotrowska, et al	Poland	2015	2012-2013	cross-sectional	school	National Reference	41	970
Długosz, et al	Poland	2015	2010-2011	cross-sectional	school	IOTF	13	553
Stankiewicz, et al	Poland	2014	2014	cross-sectional	community	National Reference	77	1515
Januszek-Trzciąkowska, et al	Poland	2014	2001	cross-sectional	school	IOTF	94	2571
Golec, et al	Poland	2014	2012	cross-sectional	school	WHO	28	305
Rutkowski, et al	Poland	2013	2006-2010	cross-sectional	school	National Reference	71	889
Czyż, et al	Poland	2017	2012-2014	cross-sectional	school	IOTF	26	641
Andaki, et al (2)	Portugal	2017	2009-2015	cross-sectional	community	IOTF	190	4052
Rodrigues, et al (1)	Portugal	2022	2009/2010	cross-sectional	school	IOTF	132	1996
Rodrigues, et al (2)	Portugal	2022	2016/2017	cross-sectional	school	IOTF	82	2077
Salas, et al (15)	Portugal	2021	2015-2017	cross-sectional	database	WHO	683	5992
Paciência, et al	Portugal	2021	2014-2015	cross-sectional	school	WHO	120	845
Abreu, et al	Portugal	2014	2008	cross-sectional	school	IOTF	92	1209
Frias-Bulhosa, et al	Portugal	2015	2015	cross-sectional	school	CDC	15	181
Padez, et al	Portugal	2005	2002-2003	cross-sectional	school	IOTF	510	4511
Araújo, et al	Portugal	2012	2003-2004	cross-sectional	school	CDC	106	1171
Silva-Santos, et al	Portugal	2017	2012	cross-sectional	school	IOTF	45	467
Sardinha, et al	Portugal	2011	2008	cross-sectional	school	IOTF	1147	22048
Seabra, et al	Portugal	2013	2010	cross-sectional	school	IOTF	84	682
Rito, et al	Portugal	2012	2007-2008	cross-sectional	school	IOTF	335	3765
Antunes, et al	Portugal	2015	2006	cross-sectional	database	IOTF	79	1273
Ferreira, et al	Portugal	2008	2004	cross-sectional	school	IOTF	142	1125

© 2024 Zhang X et al. *JAMA Pediatrics.*

Marques-Vidal, et al	Portugal	2008	2000-2002	cross-sectional	school	IOTF	252	5013
Yngve, et al (7)	Portugal	2008	2003	cross-sectional	database	IOTF	48	1176
Cabral, et al	Portugal	2016	2003-2004	cross-sectional	school	CDC	153	1547
Ribeiro, et al	Portugal	2020	2012-2013	cohort	database	WHO	803	5203
Minghelli, et al	Portugal	2014	2014	cross-sectional	school	WHO	105	966
Albuquerque, et al	Portugal	2012	2011	cross-sectional	school	IOTF	153	1433
Padez, et al	Portugal	2004	2002-2003	cross-sectional	school	IOTF	510	4511
Vale, et al	Portugal	2010	2006-2007	cross-sectional	school	IOTF	42	788
Gama, et al	Portugal	2020	2009-2010	cross-sectional	community	IOTF	1420	17277
Pereira, et al	Portugal	2010	2010	cross-sectional	school	IOTF	471	3699
Haug, et al (15)	Portugal	2009	2005-2006	cross-sectional	database	IOTF	107	3566
Fonseca, et al	Portugal	2009	2002	cross-sectional	database	IOTF	170	5470
Rebelo, et al	Portugal	2008	2000-2002	cross-sectional	school	IOTF	307	5083
Aires, et al	Portugal	2008	2008	cross-sectional	school	IOTF	28	636
Mota, et al	Portugal	2006	2006	cross-sectional	school	IOTF	33	255
Wijnhoven, et al (4)	Portugal	2015	2007/2008	cross-sectional	school	WHO	442	3026
Minghelli, et al	Portugal	2015	2015	cross-sectional	school	IOTF	52	966
Katzmarzyk, et al (10)	Portugal	2015	2011-2013	cross-sectional	database	WHO	118	686
Pedrosa, et al	Portugal	2011	2005-2006	cross-sectional	school	IOTF	73	905
Valente, et al	Portugal	2011	2011	cross-sectional	community	IOTF	208	1675
Vasques, et al	Portugal	2012	2008	cross-sectional	school	IOTF	168	1786
Marques, et al	Portugal	2016	2010	cross-sectional	database	IOTF	104	2938
Marques, et al	Portugal	2018	2018	cross-sectional	school	WHO	126	1396
Santiago, et al	Puerto Rico	2021	2014-2017	cross-sectional	school	National Reference	808	3145
Rivera-Soto, et al	Puerto Rico	2010	2010	cross-sectional	school	CDC	67	250
Rodriguez, et al	Puerto Rico	2008	2008	cross-sectional	school	CDC	97	234
Elías-Boneta, et al	Puerto Rico	2015	2010-2011	cross-sectional	school	CDC	367	1582
Cheema, et al	Qatar	2022	2018-2020	cross-sectional	school	WHO	91	459
Bener, et al	Qatar	2005	2003-2004	cross-sectional	school	IOTF	362	7442
Bener, et al	Qatar	2006	2003-2004	cross-sectional	school	IOTF	247	3923
Passmore, et al	Republic of Marshall Islands	2019	2017-2018	cross-sectional	school	CDC	167	3271
Sergentanis, et al (5)	Romania	2021	2011-2012	cross-sectional	database	IOTF	6	1240
Salas, et al (16)	Romania	2021	2015-2017	cross-sectional	database	WHO	647	5885
Barbu, et al	Romania	2015	2010-2011	cross-sectional	school	WHO	99	866
Pop, et al	Romania	2021	2016	cross-sectional	school	WHO	3414	21625
Valean, et al	Romania	2009	2009	cross-sectional	school	National Reference	1015	7904
Mocanu, et al	Romania	2013	2008-2012	cross-sectional	school	IOTF	245	3444
Emandi, et al	Romania	2012	2010-2011	cross-sectional	school	IOTF	260	3626
Cinteza, et al	Romania	2013	2006-2008	cross-sectional	school	CDC	311	4866
Gorog, et al (5)	Romania	2011	2011	cross-sectional	database	IOTF	14	1359
Haug, et al	Romania	2009	2005-2006	cross-sectional	database	IOTF	85	4450

© 2024 Zhang X et al. *JAMA Pediatrics.*

(23)								
Salas, et al (17)	Russia	2021	2015-2017	cross-sectional	database	WHO	188	2001
Janssen, et al (25)	Russia	2005	2001-2002	cross-sectional	database	IOTF	45	7450
Khasnutdinova, et al	Russia	2010	2006	cross-sectional	school	IOTF	21	1066
Haug, et al (24)	Russia	2009	2005-2006	cross-sectional	database	IOTF	61	6833
Facchini, et al (2)	Russia	2007	2002-2004	cross-sectional	database	CDC	43	2408
Gebremedhin, et al (19)	Rwanda	2015	2010	cross-sectional	database	WHO	95	4116
Pengpid, et al (3)	Samoa	2015	2010-2011	cross-sectional	database	IOTF	467	2418
Salas, et al (18)	San Marino	2021	2015-2017	cross-sectional	database	WHO	37	303
Abdellatif, et al	Saudi Arabia	2020	2020	cross-sectional	community	National Reference	112	2247
Mohamed, et al	Saudi Arabia	2022	2022	cross-sectional	school	National Reference	175	1250
Abu El Qomsan, et al	Saudi Arabia	2017	2017	cross-sectional	school	CDC	192	386
Ashi, et al (3)	Saudi Arabia	2019	2019	cross-sectional	school	WHO	73	225
Alghamdi, et al	Saudi Arabia	2017	2015	cross-sectional	school	National Reference	98	610
Bhayat, et al	Saudi Arabia	2016	2014	cross-sectional	school	WHO	121	402
Farsi, et al	Saudi Arabia	2017	2014-2015	cross-sectional	school	National Reference	197	801
Farsi, et al	Saudi Arabia	2016	2014-2015	cross-sectional	school	National Reference	164	915
Quadri, et al	Saudi Arabia	2017	2017	cross-sectional	school	CDC	17	360
Bahathig, et al	Saudi Arabia	2021	2019	cross-sectional	school	WHO	10	399
Amin, et al	Saudi Arabia	2008	2008	cross-sectional	school	IOTF	110	1139
Bajamal, et al	Saudi Arabia	2017	2017	cross-sectional	school	IOTF	30	383
Bawazeer, et al	Saudi Arabia	2009	2007	cross-sectional	school	National Reference	933	5498
Abalkhail, et al	Saudi Arabia	2002	2000	cross-sectional	school	WHO	268	1993
Al-Attas, et al	Saudi Arabia	2010	2010	cross-sectional	medical institution	IOTF	52	148
Alwan, et al	Saudi Arabia	2013	2006	cross-sectional	school	WHO	154	1212
Al-Almaie, et al	Saudi Arabia	2005	2001	cross-sectional	school	WHO	259	1766
Mustafa, et al	Saudi Arabia	2021	2019	cross-sectional	school	WHO	73	300
Al-Agha, et al	Saudi Arabia	2016	2015	cross-sectional	medical institution	CDC	316	365
Alselaim, et al	Saudi Arabia	2012	2001-2009	cohort	medical institution	CDC	55	933
Al-Hussaini, et al	Saudi Arabia	2019	2015	cross-sectional	school	WHO	1439	7931
Fakeeh, et al	Saudi Arabia	2019	2017	cross-sectional	community	WHO	37	298
Mouzan, et al	Saudi Arabia	2010	2005	cross-sectional	community	WHO	1796	19317
Al-Dossary, et al	Saudi Arabia	2010	2006	cross-sectional	medical institution	CDC	1644	7056
Al-Saeed, et al	Saudi Arabia	2007	2003	cross-sectional	school	IOTF	254	2239
Akinpelu, et al	Saudi Arabia	2014	2014	cross-sectional	school	IOTF	23	1903
El Mouzan, et al	Saudi Arabia	2012	2004-2005	cross-sectional	community	CDC	913	11112
Al Dahi, et al	Saudi Arabia	2016	2015-2016	cross-sectional	school	IOTF	44	200
Musaiger, et al (6)	Saudi Arabia	2016	2013-2014	cross-sectional	database	IOTF	175	968

© 2024 Zhang X et al. *JAMA Pediatrics*.

Gebremedhin, et al (20)	Senegal	2015	2010/2011	cross-sectional	database	WHO	49	6062
Šušnjević, et al	Serbia	2021	2013	cross-sectional	database	National Reference	165	1376
Djordjic, et al	Serbia	2016	2015	cross-sectional	school	IOTF	335	4861
Rakić, et al	Serbia	2019	2012-2017	cross-sectional	community	IOTF	63	1592
Bukara-Radujković, et al	Serbia	2009	2004	cross-sectional	school	IOTF	73	1204
Markovic, et al	Serbia	2015	2012-2014	cross-sectional	medical institution	WHO	140	406
Rakic, et al	Serbia	2011	2001-2004	cross-sectional	school	National Reference	131	2650
Srdić, et al	Serbia	2012	2012	cross-sectional	medical institution	CDC	214	2284
Janic, et al	Serbia	2013	2013	cross-sectional	school	IOTF	582	11644
Bjelanovic, et al	Serbia	2017	2013-2014	cross-sectional	school	WHO	320	6444
Halasi, et al	Serbia	2018	2018	cross-sectional	school	IOTF	12	182
Romano, et al (21)	Seychelles	2022	2015	cross-sectional	database	WHO	260	2061
Chiolero, et al	Seychelles	2007	2002-2004	cross-sectional	community	IOTF	812	15612
Bovet, et al	Seychelles	2007	2004	cross-sectional	school	IOTF	162	4343
Bovet, et al	Seychelles	2010	2004-2006	cross-sectional	school	IOTF	525	8462
Gebremedhin, et al (21)	Sierra Leone	2015	2013	cross-sectional	database	WHO	446	4698
Fu, et al	Singapore	2003	2003	cross-sectional	school	IOTF	43	623
Pwint, et al	Singapore	2013	2006-2008	cross-sectional	database	CDC	159	2256
Deurenberg-Yap, et al	Singapore	2009	2009	cross-sectional	school	CDC	447	6991
Gorog, et al (6)	Slovakia	2011	2011	cross-sectional	database	IOTF	25	1390
Haug, et al (25)	Slovakia	2009	2005-2006	cross-sectional	database	IOTF	30	3688
Janssen, et al (27)	Slovenia	2005	2001-2002	cross-sectional	database	IOTF	75	3769
Planinsec, et al	Slovenia	2004	2004	cross-sectional	school	IOTF	28	364
Planinsec, et al	Slovenia	2009	2006	cross-sectional	school	IOTF	371	5613
Haug, et al (16)	Slovenia	2009	2005-2006	cross-sectional	database	IOTF	136	4874
Sedej, et al (1)	Slovenia	2014	2001	cross-sectional	medical institution	IOTF	126	2742
Sedej, et al (2)	Slovenia	2014	2004	cross-sectional	medical institution	IOTF	207	4684
Sedej, et al (3)	Slovenia	2014	2009	cross-sectional	medical institution	IOTF	283	5406
Sedej, et al (1)	Slovenia	2016	2004	cross-sectional	school	IOTF	86	2641
Sedej, et al (2)	Slovenia	2016	2009	cross-sectional	school	IOTF	512	6861
Sedej, et al (3)	Slovenia	2016	2014	cross-sectional	school	IOTF	612	8036
Velde, et al (6)	Slovenia	2017	2010	cross-sectional	database	IOTF	65	1176
Pengpid, et al (4)	Solomon Islands	2015	2010-2011	cross-sectional	database	IOTF	38	1421
Symington, et al	South Africa	2015	2005	cross-sectional	database	IOTF	19	519
Kirsten, et al	South Africa	2013	2013	cross-sectional	school	IOTF	27	638
Abrahams, et al	South Africa	2011	2008	cross-sectional	school	WHO	43	643
Armstrong, et al	South Africa	2006	2001-2004	cross-sectional	school	IOTF	408	10283
Baard, et al	South Africa	2014	2014	cross-sectional	school	IOTF	70	713
Moselakgamo, et al	South Africa	2015	2015	cross-sectional	school	National Reference	8	1361
Oldewage-Theron, et	South Africa	2010	2010	cross-sectional	school	National Reference	4	142

© 2024 Zhang X et al. *JAMA Pediatrics*.

al								
Pedro, et al	South Africa	2014	2009	cross-sectional	database	WHO	11	588
Pienaar, et al	South Africa	2015	2013	longitudinal	database	IOTF	42	574
Tathiah, et al	South Africa	2013	2011	cross-sectional	database	IOTF	35	959
Truter, et al	South Africa	2015	2015	cross-sectional	school	IOTF	18	280
Wiles, et al	South Africa	2013	2013	cross-sectional	school	WHO	85	311
Negash, et al	South Africa	2017	2007-2008	cross-sectional	school	IOTF	114	1559
Reddy, et al (1)	South Africa	2012	2002	cross-sectional	database	IOTF	333	9522
Reddy, et al (2)	South Africa	2012	2008	cross-sectional	database	IOTF	515	9371
Moselakgomo, et al	South Africa	2017	2017	cross-sectional	school	CDC	73	1361
Modjadji, et al	South Africa	2019	2017	cross-sectional	school	WHO	5	508
Pretorius, et al	South Africa	2019	2019	prospective	medical institution	WHO	487	1785
Kruger, et al	South Africa	2006	2000-2001	cross-sectional	school	IOTF	20	1257
Nkeh-Chungag, et al	South Africa	2015	2015	cross-sectional	school	CDC	80	392
Meko, et al	South Africa	2015	2015	cross-sectional	school	WHO	25	414
Katzmarzyk, et al (11)	South Africa	2015	2011-2013	cross-sectional	database	WHO	52	468
Van Niekerk, et al	South Africa	2014	2014	cross-sectional	school	IOTF	33	689
Toriola, et al	South Africa	2012	2010	cross-sectional	school	CDC	58	1172
Park, et al (1)	South Korea	2022	2019	cross-sectional	database	National Reference	288397	2612812
Park, et al (2)	South Korea	2022	2020	cross-sectional	database	National Reference	311161	2568754
Kim, et al	South Korea	2021	2017-2019	cross-sectional	database	WHO	203	2136
Bae, et al	South Korea	2021	2012-2013	cross-sectional	database	National Reference	197	2893
Baek, et al	South Korea	2012	2009	cross-sectional	database	WHO	5979	72399
Kim, et al	South Korea	2012	2012	cohort	database	WHO	5975	72399
Lee, et al	South Korea	2013	2006-2010	cross-sectional	database	National Reference	241	1649
Bae, et al	South Korea	2010	2008	cross-sectional	school	National Reference	58	379
Lee, et al	South Korea	2013	2013	cross-sectional	school	National Reference	48	422
Choo, et al	South Korea	2017	2015	cross-sectional	community	National Reference	20	126
Le, et al (1)	South Korea	2020	2007-2009	cross-sectional	database	National Reference	353	5415
Le, et al (2)	South Korea	2020	2010-2012	cross-sectional	database	National Reference	331	4936
Le, et al (3)	South Korea	2020	2013-2015	cross-sectional	database	National Reference	278	4131
Lee, et al	South Korea	2012	2008	cohort	school	National Reference	69	1030
Kim, et al	South Korea	2005	2002	cross-sectional	school	IOTF	108	1107
Sunwoo, et al	South Korea	2020	2011	cross-sectional	school	National Reference	1375	22906
Lee, et al	South Korea	2010	2005	cross-sectional	database	IOTF	51	928
Chang, et al	South Korea	2008	2008	cross-sectional	school	IOTF	224	4033
Yoo, et al	South Korea	2006	2006	cross-sectional	school	IOTF	103	938
Lee, et al	South Korea	2006	2001	cross-sectional	school	CDC	269	3059
Kong, et al	South Korea	2015	2013	cross-sectional	database	CDC	1856	53769
Oh, et al	South Korea	2011	2007	cross-sectional	database	National Reference	5645	60643
Kim, et al	South Korea	2012	2008	cross-sectional	school	National Reference	109	1644
Choi, et al	South Korea	2013	2010-2011	cross-sectional	community	National Reference	139	2038
Kim, et al	South Korea	2017	2011-2015	cross-sectional	database	National Reference	9528	136739
Cho, et al	South Korea	2018	2007-2014	cross-sectional	database	National	913	7197

© 2024 Zhang X et al. *JAMA Pediatrics.*

						Reference		
Ara, et al	Spain	2007	2007	cross-sectional	school	IOTF	69	1068
Sánchez-Cruz, et al	Spain	2013	2012	cross-sectional	community	WHO	123	978
Gulías-González, et al	Spain	2014	2010	cross-sectional	community	IOTF	213	2301
Ahrens, et al (8)	Spain	2014	2007-2008	cross-sectional	database	IOTF	95	1539
Lasarte-Velillas, et al	Spain	2022	2003-2018	cross-sectional	database	WHO	21517	161335
Bont, et al	Spain	2022	2005-2017	longitudinal	database	WHO	356728	2504568
Cartanyà-Hueso, et al	Spain	2022	2017	cross-sectional	database	IOTF	457	4528
Cabeza, et al	Spain	2022	2022	cross-sectional	school	WHO	49	212
Aragón-Martín, et al	Spain	2022	2018	cross-sectional	school	IOTF	112	864
Vega-Ramirez, et al	Spain	2021	2021	cross-sectional	school	National Reference	32	287
Usheva, et al (6)	Spain	2021	2012	cross-sectional	database	WHO	23	713
Sergentanis, et al (6)	Spain	2021	2011-2012	cross-sectional	database	IOTF	15	1295
Salas, et al (19)	Spain	2021	2015-2017	cross-sectional	database	WHO	1782	10239
Barja-Fernández, et al	Spain	2018	2018	cross-sectional	medical institution	IOTF	176	471
Marcos-Pasero, et al	Spain	2019	2017	longitudinal	school	IOTF	16	201
Baile, et al	Spain	2020	2020	cross-sectional	school	National Reference	46	1197
Bawaked, et al	Spain	2020	2003-2008	longitudinal	database	WHO	133	1480
Bazán, et al	Spain	2018	2011	cross-sectional	database	National Reference	445	3752
Bont, et al	Spain	2019	2012-2013	cross-sectional	database	WHO	421	2660
Tamayo-Ortiz, et al	Spain	2021	2012	cross-sectional	database	WHO	290	1233
Martín, et al	Spain	2008	2004-2006	cross-sectional	database	IOTF	83	1312
Llargues, et al	Spain	2011	2006	RCT	school	IOTF	45	508
Yngve, et al (8)	Spain	2008	2003	cross-sectional	database	IOTF	18	745
Riaño-Galán, et al	Spain	2017	2003-2008	cohort	community	IOTF	67	1044
Montero, et al	Spain	2005	2005	cross-sectional	school	IOTF	27	229
Garcia-Marcos, et al	Spain	2008	2008	cross-sectional	school	IOTF	1689	17145
Garcia-Marcos, et al	Spain	2008	2008	cross-sectional	school	IOTF	128	931
Janssen, et al (28)	Spain	2005	2001-2002	cross-sectional	database	IOTF	111	4445
Vázquez, et al	Spain	2010	2010	cross-sectional	school	IOTF	206	2305
Esteban-Vasallo, et al	Spain	2020	2012	cross-sectional	database	IOTF	97	2914
Martinez-Gomez, et al	Spain	2010	2007-2008	cross-sectional	community	IOTF	3	192
Bibiloni, et al	Spain	2010	2007-2008	cross-sectional	community	WHO	128	1231
Haug, et al (17)	Spain	2009	2005-2006	cross-sectional	database	IOTF	177	7380

© 2024 Zhang X et al. *JAMA Pediatrics*.

Larrañaga, et al	Spain	2007	2004-2005	cross-sectional	community	IOTF	64	1178
Ayala, et al	Spain	2007	2007	cross-sectional	community	CDC	52	154
Rojo, et al	Spain	2006	2003-2004	cross-sectional	database	National Reference	2957	35403
Morales-Suárez-Varela, et al	Spain	2015	2013-2014	cross-sectional	database	CDC	135	710
Alonso-Fernández, et al (1)	Spain	2015	2006	cross-sectional	database	IOTF	278	5108
Alonso-Fernández, et al (2)	Spain	2015	2011-2012	cross-sectional	database	IOTF	270	3265
Weinmayr, et al (14)	Spain	2014	2004	cross-sectional	database	IOTF	141	968
Cerrillo, et al	Spain	2012	2012	cross-sectional	school	IOTF	141	990
Martínez-Vizcaíno, et al (1)	Spain	2012	2004	cross-sectional	school	IOTF	104	1119
Martínez-Vizcaíno, et al (2)	Spain	2012	2010	cross-sectional	school	IOTF	102	1070
Navalpotro, et al	Spain	2012	2006-2007	cross-sectional	database	IOTF	332	4528
Jiménez-Ormeño, et al	Spain	2013	2013	cross-sectional	school	IOTF	103	1032
Laguna, et al	Spain	2013	2013	cross-sectional	database	IOTF	44	761
Morales, et al	Spain	2013	2010-2011	cross-sectional	school	IOTF	116	1158
Moreno, et al	Spain	2013	2008-2009	cross-sectional	database	CDC	449	2316
Pérez-Farinós, et al	Spain	2013	2010-2011	cross-sectional	database	IOTF	843	7659
García-García, et al	Spain	2016	2012	cross-sectional	community	IOTF	129	1317
Alvarez Zallo ,et al	Spain	2017	2017	cross-sectional	database	IOTF	173	8607
Martín-Espinosa, et al	Spain	2017	2013	cross-sectional	school	IOTF	134	1604
Velde, et al (7)	Spain	2017	2010	cross-sectional	database	IOTF	30	1022
Ramos, et al	Spain	2018	2015	cross-sectional	school	WHO	11	235
Rathnayake, et al	Sri Lanka	2013	2013	cross-sectional	medical institution	WHO	13	1087
Wickramasinghe, et al	Sri Lanka	2013	2009-2010	cross-sectional	school	CDC	25	920
Warnakulasuriya, et al	Sri Lanka	2019	2019	cross-sectional	school	WHO	612	12788
Wickramasinghe, et al	Sri Lanka	2009	2004-2005	cross-sectional	school	IOTF	17	282
Salman, et al	Sudan	2010	2010	cross-sectional	school	CDC	30	304
Nagwa, et al	Sudan	2011	2011	cross-sectional	school	WHO	110	1138
Musaiger, et al (7)	Sudan	2016	2013-2014	cross-sectional	database	IOTF	28	902
Romano, et al (22)	Suriname	2022	2016	cross-sectional	database	WHO	167	1453
Ahrens, et al (5)	Sweden	2014	2007-2008	cross-sectional	database	IOTF	41	1824
Wallby, et al	Sweden	2017	2002-2007	cohort	database	IOTF	571	30508
Hamano, et al	Sweden	2017	2005-2010	cohort	database	National Reference	6968	944487
Önnestam, et al	Sweden	2022	2015	longitudinal	medical institution	IOTF	7	314
Fäldt, et al	Sweden	2022	2015-2019	cross-sectional	database	National	744	23214

© 2024 Zhang X et al. *JAMA Pediatrics*.

(1)						Reference		
Fäldt, et al (2)	Sweden	2022	2020-2021	cross-sectional	database	National Reference	719	19171
Sjöberg, et al	Sweden	2005	2004	cross-sectional	database	IOTF	131	4703
Norberg, et al	Sweden	2012	2004	cross-sectional	medical institution	IOTF	47	920
Nilsen, et al	Sweden	2017	2008-2010	cross-sectional	community	WHO	121	2620
Moraeus, et al	Sweden	2014	2008/2010/2013	cross-sectional	school	IOTF	111	3492
Garmy, et al	Sweden	2014	2008-2009	cross-sectional	school	IOTF	92	2891
Sjöberg, et al	Sweden	2008	2000-2005	cross-sectional	school	IOTF	247	8876
Mårild, et al	Sweden	2004	2000	cross-sectional	community	IOTF	138	4730
Yngve, et al (9)	Sweden	2008	2003	cross-sectional	database	IOTF	14	888
Mangrio, et al	Sweden	2010	2003-2008	cross-sectional	medical institution	IOTF	264	8621
Koch, et al	Sweden	2008	2001	cohort	database	IOTF	282	6733
White, et al (1)	Sweden	2022	2022	cohort	database	IOTF	77	3237
Janssen, et al (29)	Sweden	2005	2001-2002	cross-sectional	database	IOTF	49	3507
Litsfeldt, et al	Sweden	2020	2015-2017	cross-sectional	school	IOTF	46	1363
Lager,et al	Sweden	2009	2003-2006	cross-sectional	school	IOTF	319	7584
Haug, et al (38)	Sweden	2009	2005-2006	cross-sectional	database	IOTF	56	3974
Bergström, et al (1)	Sweden	2009	2002-2003	cross-sectional	medical institution	IOTF	193	4407
Blomquist, et al	Sweden	2007	2007	cross-sectional	medical institution	IOTF	200	4407
Wijnhoven, et al (5)	Sweden	2015	2007/2008	cross-sectional	school	WHO	236	3633
Moraeus, et al (1)	Sweden	2015	2008	cross-sectional	school	IOTF	22	833
Moraeus, et al (2)	Sweden	2015	2010	cross-sectional	school	IOTF	26	1085
Moraeus, et al (3)	Sweden	2015	2013	cross-sectional	school	IOTF	24	1134
Lindkvist, et al	Sweden	2015	2008-2012	cross-sectional	community	WHO	89	697
Weinmayr, et al (15)	Sweden	2014	2004	cross-sectional	database	IOTF	31	459
Vaezghasemi, et al	Sweden	2012	2007	cross-sectional	school	IOTF	120	4987
Khanolkar, et al	Sweden	2013	2013	cross-sectional	community	IOTF	377	10628
Winkvist, et al	Sweden	2016	2008	cross-sectional	community	IOTF	274	11222
Bergström, et al (2)	Sweden	2009	2007-2008	cross-sectional	medical institution	IOTF	108	4381
Aeberli, et al (1)	Switzerland	2010	2007	cross-sectional	school	CDC	93	2222
Aeberli, et al (2)	Switzerland	2010	2002	cross-sectional	school	CDC	157	2404
Aeberli, et al	Switzerland	2010	2009	cross-sectional	database	CDC	47	900
Jeannot, et al	Switzerland	2015	2011-2012	cross-sectional	school	IOTF	271	8544
Lasserre, et al	Switzerland	2007	2005-2006	cross-sectional	school	IOTF	89	5207
Janssen, et al (30)	Switzerland	2005	2001-2002	cross-sectional	database	IOTF	54	4190
Zimmermann, et al	Switzerland	2004	2004	cross-sectional	school	CDC	159	2431
Köchli, et al	Switzerland	2019	2019	cross-sectional	database	IOTF	43	1246
Haug, et al (9)	Switzerland	2009	2005-2006	cross-sectional	database	IOTF	38	4251
Velde, et al (8)	Switzerland	2017	2010	cross-sectional	database	IOTF	14	596
Nasreddine, et al	Syria	2010	2010	cross-sectional	school	WHO	67	776

© 2024 Zhang X et al. *JAMA Pediatrics*.

Taguri, et al (1)	Syria	2009	2001	cross-sectional	database	WHO	1100	5454
Musaiger, et al (4)	Syria	2012	2010-2011	cross-sectional	school	IOTF	64	1062
Yen, et al	Taiwan	2021	2019-2020	cross-sectional	school	WHO	103	569
Wang, et al	Taiwan	2021	2004-2017	cross-sectional	school	National Reference	198	1599
Chen, et al	Taiwan	2012	2007/2010	cross-sectional	database	National Reference	1321	7930
Chu, et al	Taiwan	2007	2001-2002	cross-sectional	database	National Reference	289	2405
Liou, et al	Taiwan	2010	2006-2007	cross-sectional	school	IOTF	624	8640
Ting, et al	Taiwan	2012	2010-2011	cross-sectional	school	National Reference	102	859
Tsai, et al	Taiwan	2009	2007	cross-sectional	school	CDC	221	1287
Chen, et al	Taiwan	2019	2010	cohort	database	National Reference	514	2804
Lai, et al	Taiwan	2001	2000	cross-sectional	school	National Reference	273	2011
Yen, et al	Taiwan	2010	2004	cross-sectional	database	IOTF	630	10371
Pu, et al	Taiwan	2010	2003	cross-sectional	database	IOTF	94	1879
Chang, et al	Taiwan	2010	2010	cross-sectional	school	National Reference	266	2083
Chen, et al	Taiwan	2009	2009	cross-sectional	school	IOTF	52	1024
Chen, et al	Taiwan	2008	2008	cross-sectional	school	IOTF	54	866
Chang, et al	Taiwan	2015	2008-2012	cross-sectional	school	National Reference	87	838
Lee, et al	Taiwan	2014	2009	cross-sectional	school	IOTF	804	5254
Hsieh, et al	Taiwan	2014	2010-2011	cross-sectional	school	IOTF	402	2419
Chen, et al	Taiwan	2014	2014	cross-sectional	medical institution	National Reference	12	121
Chiang, et al	Taiwan	2013	2001-2002	cross-sectional	database	National Reference	306	2283
Liao, et al	Taiwan	2013	2010-2011	cross-sectional	database	National Reference	2093	13500
Chen, et al	Taiwan	2016	2010-2011	cross-sectional	database	National Reference	307	1826
Salas, et al (20)	Tajikistan	2021	2015-2017	cross-sectional	database	WHO	42	2822
Mosha, et al	Tanzania	2021	2019	cross-sectional	school	WHO	70	1170
Mosha, et al	Tanzania	2010	2008	cross-sectional	community	National Reference	11	222
Mpembeni, et al	Tanzania	2014	2014	cross-sectional	school	National Reference	23	446
Mwaikambo, et al	Tanzania	2015	2015	cross-sectional	school	IOTF	78	1722
Pangani, et al	Tanzania	2016	2012	cross-sectional	school	WHO	119	1781
Gebremedhin, et al (22)	Tanzania	2015	2010	cross-sectional	database	WHO	181	6948
Chomba, et al	Tanzania	2019	2018	cross-sectional	community	WHO	23	451
Rerksuppaphol, et al	Thailand	2021	2015	cross-sectional	school	WHO	607	3870
Weraarchakul, et al	Thailand	2017	2015	cross-sectional	school	WHO	39	210
Nonboonyawat, et al	Thailand	2019	2016	cross-sectional	school	WHO	127	1749
Pawloski, et al	Thailand	2008	2008	cross-sectional	school	IOTF	32	410
Romano, et al (23)	Thailand	2022	2015	cross-sectional	database	WHO	273	4132
Manandhar, et al	Thailand	2019	2018	cross-sectional	school	WHO	30	440
Sengmeuang, et al	Thailand	2010	2007	cross-sectional	school	National Reference	345	7096
Rerksuppaphol, et al	Thailand	2010	2007	cross-sectional	school	IOTF	133	1140
Narksawat, et al	Thailand	2009	2009	cross-sectional	school	National Reference	54	862
Rerksuppaphol, et al	Thailand	2015	2013	cross-sectional	school	WHO	620	3991
Sukhonthac	Thailand	2014	2012	cross-sectional	school	CDC	145	693

© 2024 Zhang X et al. *JAMA Pediatrics.*

hit, et al								
Firestone, et al	Thailand	2011	2004	cross-sectional	database	CDC	381	4610
Jitnarin, et al	Thailand	2011	2011	cross-sectional	database	IOTF	604	9287
Rojroongwasinkul, et al	Thailand	2013	2011	cross-sectional	community	WHO	243	3119
Thasanasuwan, et al	Thailand	2016	2016	cross-sectional	database	WHO	142	1345
Gebremedhin, et al (23)	Togo	2015	2013/2014	cross-sectional	database	WHO	29	3228
Sagbo, et al	Togo	2018	2015	cross-sectional	school	IOTF	12	634
Romano, et al (24)	Tonga	2022	2017	cross-sectional	database	WHO	488	1946
Smith, et al	Tonga	2007	2000	cross-sectional	school	IOTF	43	445
Pengpid, et al (5)	Tonga	2015	2010-2011	cross-sectional	database	IOTF	467	2211
Romano, et al (25)	Trinidad and Tobago	2022	2017	cross-sectional	database	WHO	421	2363
Mumena, et al	Trinidad and Tobago	2018	2012-2014	prospective	school	WHO	29	336
Boukthir, et al	Tunisia	2011	2007	cross-sectional	school	IOTF	77	1335
Musaiger, et al (8)	Tunisia	2016	2013-2014	cross-sectional	database	IOTF	46	803
Ercan, et al	Turkey	2012	2010-2011	cross-sectional	school	National Reference	678	8848
Salman, et al	Turkey	2022	2014	cross-sectional	school	National Reference	102	1127
Meydanlioglu, et al	Turkey	2022	2017	cross-sectional	school	CDC	611	5160
Salas, et al (21)	Turkey	2021	2015-2017	cross-sectional	database	WHO	117	3658
Gunalan, et al	Turkey	2021	2017	cross-sectional	school	WHO	215	1561
Arslan, et al	Turkey	2021	2016	cross-sectional	school	CDC	1030	9786
Acar Tek, et al	Turkey	2017	2017	cross-sectional	school	WHO	22	1111
Sağlam, et al	Turkey	2008	2008	cross-sectional	school	CDC	538	5368
Sur, et al	Turkey	2005	2001-2002	cross-sectional	school	IOTF	21	1044
Gundogdu, et al	Turkey	2008	2002	cross-sectional	medical institution	CDC	59	1899
Turkkahraman, et al	Turkey	2006	2002-2003	cross-sectional	school	IOTF	88	2465
Nur, et al	Turkey	2008	2004	cross-sectional	school	CDC	2	1020
Bayat, et al	Turkey	2009	2004	cross-sectional	school	CDC	13	610
Discigil, et al	Turkey	2009	2005	cross-sectional	school	CDC	50	1348
Etiler, et al	Turkey	2011	2011	cross-sectional	school	WHO	171	2281
Ozmen, et al	Turkey	2007	2007	cross-sectional	school	IOTF	24	2101
Simsek, et al	Turkey	2008	2005-2006	cross-sectional	school	IOTF	427	6925
Yuca, et al	Turkey	2010	2006-2007	cross-sectional	school	IOTF	198	9048
Pirinçci, et al	Turkey	2010	2007	cross-sectional	school	IOTF	60	3642
Duzova, et al	Turkey	2013	2007-2008	cross-sectional	community	CDC	318	3571
Demirci, et al	Turkey	2013	2011-2012	cross-sectional	community	National Reference	112	1000
Polat, et al	Turkey	2014	2012	cross-sectional	school	CDC	393	2826
Gökler, et al	Turkey	2015	2012	cross-sectional	school	WHO	408	3918
Agirbasli, et al	Turkey	2011	2008	cross-sectional	medical institution	IOTF	75	1746
Manios, et al	Turkey	2005	2001	cross-sectional	school	IOTF	8	510
Krassas, et al (2)	Turkey	2004	2004	cross-sectional	school	National Reference	59	3703
Yardim, et al	Turkey	2019	2015	cross-sectional	school	WHO	483	3291
Akbulut, et al (1)	Turkey	2014	2014	cross-sectional	school	IOTF	55	915

© 2024 Zhang X et al. *JAMA Pediatrics.*

Canan, et al	Turkey	2014	2014	cross-sectional	school	National Reference	16	1938
Dündar, et al	Turkey	2012	2009	cross-sectional	school	CDC	254	2477
Uçkun-Kitapçi, et al	Turkey	2004	2004	cross-sectional	school	National Reference	59	1647
Oner, et al	Turkey	2004	2001	cross-sectional	community	IOTF	18	989
Ardıç, et al	Turkey	2019	2006-2010	cross-sectional	medical institution	National Reference	22	180
Aşut, et al	Turkey	2019	2017	cross-sectional	school	National Reference	24	459
Çelmeli, et al	Turkey	2019	2015	cross-sectional	community	IOTF	165	1687
Comba, et al	Turkey	2019	2017	cross-sectional	school	WHO	110	1684
Deniz, et al	Turkey	2019	2019	cross-sectional	school	WHO	90	1298
Arikan, et al	Turkey	2020	2020	cross-sectional	school	National Reference	599	10781
Ucar, et al	Turkey	2009	2001-2003	cross-sectional	school	IOTF	744	11629
Haug, et al (18)	Turkey	2009	2005-2006	cross-sectional	database	IOTF	61	4680
Garipagaoglu, et al	Turkey	2009	2006	cross-sectional	medical institution	National Reference	184	592
Dinç, et al	Turkey	2009	2009	cross-sectional	school	CDC	43	1346
Önsüz, et al	Turkey	2015	2010	cross-sectional	school	WHO	390	2166
Meseri, et al	Turkey	2015	2012	cross-sectional	school	WHO	84	462
Inal, et al	Turkey	2015	2011	cross-sectional	school	CDC	77	531
Weinmayr, et al (16)	Turkey	2014	2004	cross-sectional	database	IOTF	21	342
Vehapoglu, et al	Turkey	2014	2012-2013	cross-sectional	medical institution	National Reference	389	4990
Cabar, et al	Turkey	2014	2010-2011	cross-sectional	school	National Reference	202	3352
Ayyildiz, et al	Turkey	2014	2010	cross-sectional	school	National Reference	143	868
Cinar, et al	Turkey	2011	2004	cross-sectional	school	WHO	139	451
Akca, et al	Turkey	2016	2011	cross-sectional	school	CDC	79	554
Geckil, et al	Turkey	2017	2012-2013	cross-sectional	school	National Reference	263	3028
Gül, et al	Turkey	2017	2013-2014	cross-sectional	school	National Reference	141	1374
Eker, et al	Turkey	2018	2011-2012	cross-sectional	school	WHO	35	1357
Haney, et al	Turkey	2018	2014-2015	cross-sectional	school	National Reference	190	1289
Salas, et al (22)	Turkmenistan	2021	2015-2017	cross-sectional	database	WHO	1055	9768
Gebremedhin, et al (24)	Uganda	2015	2011	cross-sectional	database	WHO	55	4212
Dereń, et al	Ukraine	2020	2018-2019	cross-sectional	school	WHO	746	18144
Dereń, et al	Ukraine	2018	2018	cross-sectional	school	WHO	577	13739
Høyer, et al (3)	Ukraine	2014	2002-2006	cohort	database	WHO	28	492
Janssen, et al (31)	Ukraine	2005	2001-2002	cross-sectional	database	IOTF	18	3645
Haug, et al (26)	Ukraine	2009	2005-2006	cross-sectional	database	IOTF	37	4613
AlBlooshi, et al	United Arab Emirates	2016	2014-2015	cross-sectional	community	CDC	4673	27078
Abduelkarem, et al	United Arab Emirates	2020	2017	cross-sectional	school	CDC	95	684
Abdullatif, et al	United Arab Emirates	2022	2018-2019	cross-sectional	school	WHO	282	1683
Aburawi, et al	United Arab Emirates	2019	2019	cross-sectional	school	IOTF	134	967
Zaal, et al	United Arab Emirates	2009	2009	cross-sectional	school	WHO	141	661
Musaiger, et al (5)	United Arab Emirates	2012	2010-2011	cross-sectional	school	IOTF	66	505
Fatima, et al	United Arab Emirates	2018	2017-2018	cross-sectional	school	CDC	31	267
Adab, et al	United Kingdom of Great Britain	2018	2005	RCT	school	National Reference	124	1397

© 2024 Zhang X et al. *JAMA Pediatrics*.

	and Northern Ireland							
Sweeting, et al	United Kingdom of Great Britain and Northern Ireland	2005	2005	cohort	database	National Reference	214	2127
Fraser, et al	United Kingdom of Great Britain and Northern Ireland	2012	2004-2008	cohort	database	National Reference	623	4768
Gilliland, et al	United Kingdom of Great Britain and Northern Ireland	2012	2012	cross-sectional	school	WHO	73	966
Uerlich, et al	United Kingdom of Great Britain and Northern Ireland	2021	2014-2015	cross-sectional	database	WHO	7	171
Ralphs, et al	United Kingdom of Great Britain and Northern Ireland	2021	2007-2010	cross-sectional	database	National Reference	335	6410
Adab, et al	United Kingdom of Great Britain and Northern Ireland	2014	2006-2007	RCT	school	National Reference	73	571
Bartle, et al	United Kingdom of Great Britain and Northern Ireland	2013	2013	longitudinal	school	IOTF	21	400
Pearce, et al	United Kingdom of Great Britain and Northern Ireland	2010	2003-2005	cohort	database	IOTF	692	13187
Harding, et al	United Kingdom of Great Britain and Northern Ireland	2008	2002-2003	cross-sectional	school	IOTF	462	5684
Steele, et al	United Kingdom of Great Britain and Northern Ireland	2009	2007	cross-sectional	school	IOTF	95	1862
Basterfield, et al	United Kingdom of Great Britain and Northern Ireland	2014	2006-2007	cross-sectional	database	IOTF	27	425
Griffiths, et al	United Kingdom of Great Britain and Northern	2014	2005-2007	cross-sectional	database	National Reference	2479	13291

© 2024 Zhang X et al. *JAMA Pediatrics.*

	Ireland							
Scott, et al	United Kingdom of Great Britain and Northern Ireland	2016	2000-2013	cohort	database	IOTF	1087	17231
Clemente, et al	United Kingdom of Great Britain and Northern Ireland	2019	2019	cross-sectional	database	WHO	84	1396
Griffiths, et al	United Kingdom of Great Britain and Northern Ireland	2011	2000-2002	cross-sectional	database	IOTF	601	11202
Tiffin, et al	United Kingdom of Great Britain and Northern Ireland	2011	2007	cross-sectional	database	IOTF	305	3961
Harrison, et al	United Kingdom of Great Britain and Northern Ireland	2011	2011	cross-sectional	school	IOTF	90	1724
Taylor, et al	United Kingdom of Great Britain and Northern Ireland	2005	2001	cross-sectional	school	IOTF	211	2482
Janssen, et al (7)	United Kingdom of Great Britain and Northern Ireland	2005	2001-2002	cross-sectional	database	IOTF	184	3601
Janssen, et al (26)	United Kingdom of Great Britain and Northern Ireland	2005	2001-2002	cross-sectional	database	IOTF	64	2133
Janssen, et al (33)	United Kingdom of Great Britain and Northern Ireland	2005	2001-2002	cross-sectional	database	IOTF	152	3170
Cecil, et al	United Kingdom of Great Britain and Northern Ireland	2005	2002-2003	cross-sectional	school	IOTF	150	2454
Fletcher, et al	United Kingdom of Great Britain and Northern Ireland	2004	2000	cross-sectional	school	IOTF	30	424
Webster-Gandy, et al	United Kingdom of Great Britain and Northern Ireland	2003	2003	cross-sectional	school	IOTF	7	188
Warren, et	United	2003	2001	cross-sectional	school	IOTF	2	37

© 2024 Zhang X et al. *JAMA Pediatrics*.

al	Kingdom of Great Britain and Northern Ireland							
Whitaker, et al	United Kingdom of Great Britain and Northern Ireland	2010	2001-2006	cross-sectional	database	IOTF	460	7078
Thomas, et al(1)	United Kingdom of Great Britain and Northern Ireland	2010	2002	cross-sectional	school	IOTF	2	71
Thomas, et al(2)	United Kingdom of Great Britain and Northern Ireland	2010	2007	cross-sectional	school	IOTF	3	83
Skidmore, et al	United Kingdom of Great Britain and Northern Ireland	2010	2007	cross-sectional	school	IOTF	92	1721
Jennings, et al	United Kingdom of Great Britain and Northern Ireland	2010	2010	cross-sectional	school	IOTF	17	84
Edwards, et al	United Kingdom of Great Britain and Northern Ireland	2010	2000-2006	cross-sectional	community	National Reference	2537	33594
Willianmson, et al(1)	United Kingdom of Great Britain and Northern Ireland	2009	2006	cross-sectional	school	CDC	741	2709
Willianmson, et al(2)	United Kingdom of Great Britain and Northern Ireland	2009	2006	cross-sectional	school	IOTF	590	2709
Webber, et al(1)	United Kingdom of Great Britain and Northern Ireland	2009	2006	cross-sectional	database	IOTF	8	239
Webber, et al(2)	United Kingdom of Great Britain and Northern Ireland	2009	2006	cross-sectional	database	IOTF	12	167
Standley, et al	United Kingdom of Great Britain and Northern Ireland	2009	2002	cross-sectional	school	IOTF	294	4167
Nelson, et al	United Kingdom of Great Britain	2009	2003-2005	cross-sectional	school	IOTF	184	4587

© 2024 Zhang X et al. *JAMA Pediatrics*.

	and Northern Ireland							
Haug, et al (28)	United Kingdom of Great Britain and Northern Ireland	2009	2005-2006	cross-sectional	database	IOTF	33	1961
Haug, et al (37)	United Kingdom of Great Britain and Northern Ireland	2009	2005-2006	cross-sectional	database	IOTF	57	2600
Haug, et al (39)	United Kingdom of Great Britain and Northern Ireland	2009	2005-2006	cross-sectional	database	IOTF	116	2910
van Sluijs, et al	United Kingdom of Great Britain and Northern Ireland	2008	2007	cross-sectional	database	IOTF	114	2064
Harding, et al	United Kingdom of Great Britain and Northern Ireland	2008	2008	cross-sectional	database	IOTF	453	5515
Routh, et al	United Kingdom of Great Britain and Northern Ireland	2006	2006	cross-sectional	school	IOTF	18	252
Mutunga, et al	United Kingdom of Great Britain and Northern Ireland	2006	2000	cross-sectional	database	National Reference	308	2016
Solmi, et al	United Kingdom of Great Britain and Northern Ireland	2015	2006-2012	cross-sectional	database	IOTF	1576	29001
Katzmarzyk, et al (12)	United Kingdom of Great Britain and Northern Ireland	2015	2011-2013	cross-sectional	database	WHO	44	478
Jackson, et al	United Kingdom of Great Britain and Northern Ireland	2015	2005-2012	cross-sectional	database	IOTF	373	4979
Falconer, et al	United Kingdom of Great Britain and Northern Ireland	2014	2010-2011	cross-sectional	database	National Reference	203	2737
Pallan, et al	United Kingdom of Great Britain and Northern	2011	2011	cross-sectional	database	National Reference	73	574

© 2024 Zhang X et al. *JAMA Pediatrics.*

	Ireland							
Coulthard, et al	United Kingdom of Great Britain and Northern Ireland	2016	2008-2012	cross-sectional	database	National Reference	297	1620
Beynon, et al	United Kingdom of Great Britain and Northern Ireland	2017	2008-2012	cross-sectional	database	National Reference	1582	11279
Hudda, et al	United Kingdom of Great Britain and Northern Ireland	2018	2012-2013	cross-sectional	database	National Reference	145371	1068261
Wang, et al	United States of America	2010	2005-2006	cross-sectional	database	CDC	966	6939
Chiasson, et al	United States of America	2016	2008-2009	cohort	database	CDC	7548	50589
Ehrenthal, et al	United States of America	2016	2004-2011	cohort	database	CDC	290	2172
Taveras, et al	United States of America	2006	2006	cohort	database	CDC	91	1012
Weden, et al	United States of America	2012	2001-2007	cohort	database	CDC	442	3300
Vehrs, et al	United States of America	2022	2022	cross-sectional	community	CDC	19	332
Vazquez, et al	United States of America	2022	2019	cross-sectional	school	CDC	9	237
Leung, et al	United States of America	2011	2005-2006	cohort	database	CDC	48	353
Shier, et al	United States of America	2012	2004/2007	cross-sectional	database	CDC	1202	6260
Salcido, et al	United States of America	2022	2017-2018	cross-sectional	medical institution	CDC	1307	7270
Odusanya, et al	United States of America	2022	2009-2018	cross-sectional	database	CDC	143	3248
Mayne, et al	United States of America	2023	2019-2020	cross-sectional	medical institution	CDC	12528	81418
Bader, et al	United States of America	2013	2007-2008	cross-sectional	database	CDC	15611	113123
Burdette, et al	United States of America	2004	2004	cross-sectional	database	CDC	634	6907
Carroll-Scott, et al	United States of America	2013	2009	cross-sectional	database	National Reference	303	996
Hunt, et al	United States of America	2022	2009-2013	cohort	database	CDC	107	816
Fyfe-Johnson, et al	United States of America	2022	2012	cross-sectional	database	CDC	4878	17115
Flórez, et al	United States of America	2022	2006-2017	cross-sectional	database	CDC	93035	570172
Davis, et al	United	2009	2002-2005	cross-sectional	database	CDC	63524	529367

© 2024 Zhang X et al. *JAMA Pediatrics.*

	States of America							
Salazar, et al	United States of America	2022	2018-2019	cross-sectional	medical institution	CDC	34	112
Bejarano, et al	United States of America	2022	2015-2016	cross-sectional	database	IOTF	60481	320005
Zhang, et al	United States of America	2021	2017	cross-sectional	database	CDC	1030	4348
Kepper, et al	United States of America	2016	2016	RCT	database	CDC	8	78
Stine, et al	United States of America	2021	2017	cross-sectional	database	CDC	1143	3956
Reilly, et al (1)	United States of America	2021	2008-2009	cross-sectional	school	CDC	109	511
Reilly, et al (2)	United States of America	2021	2015-2016	cross-sectional	school	CDC	182	836
Liu, et al	United States of America	2007	2000	cross-sectional	medical institution	CDC	1648	7334
Imoisili, et al	United States of America	2021	2012-2018	cross-sectional	database	National Reference	126125	759591
Mellor, et al	United States of America	2011	2006	cross-sectional	school	CDC	352	2023
Oreskovic, et al	United States of America	2009	2006	cross-sectional	medical institution	National Reference	1341	6680
Salois, et al	United States of America	2012	2007-2009	cross-sectional	database	CDC	310	2192
Sánchez, et al	United States of America	2012	2007	cross-sectional	database	CDC	203724	926018
Wasserman, et al	United States of America	2014	2008-2009	cross-sectional	school	CDC	2501	12090
Halfon, et al	United States of America	2013	2007	cross-sectional	database	CDC	6928	43297
Hurt, et al	United States of America	2014	2005-2010	cross-sectional	database	CDC	1491	7629
Sacheck, et al	United States of America	2011	2009	cross-sectional	school	CDC	65	263
Turer, et al	United States of America	2013	2003-2006	cross-sectional	database	CDC	2478	12292
Moore, et al	United States of America	2016	2005-2006	cross-sectional	database	National Reference	582	2482
Lee, etal	United States of America	2007	2007	longitudinal	database	CDC	36	309
Achaya, et al	United States of America	2011	2011	cross-sectional	school	CDC	181	719
Adachi-Mejia, et al	United States of America	2007	2002-2003	cross-sectional	school	CDC	523	2343
Adams, et al	United States of America	2010	2010	cross-sectional	community	CDC	106	421
Adams, et al	United States of America	2005	2001-2003	cross-sectional	school	CDC	95	366

© 2024 Zhang X et al. *JAMA Pediatrics.*

Adams, et al	United States of America	2019	2013-2015	cross-sectional	database	National Reference	99	450
Adams, et al	United States of America	2013	2007-2009	cross-sectional	database	CDC	89089	495848
Ogden, et al	United States of America	2018	2013-2016	cross-sectional	database	CDC	648	4219
Davis, et al	United States of America	2011	2003-2006	cross-sectional	database	CDC	1562	7882
Lewis, et al	United States of America	2006	2002	cross-sectional	school	CDC	629	3114
Singh, et al	United States of America	2008	2003-2004	cross-sectional	database	CDC	6931	46707
Davis, et al	United States of America	2008	2008	cross-sectional	school	CDC	29	138
Agazzi, et al	United States of America	2010	2005	cross-sectional	school	CDC	115	535
DeBoer, et al	United States of America	2015	2001	longitudinal	database	CDC	1350	8950
Govindan, et al	United States of America	2013	2004-2011	cross-sectional	school	CDC	292	1714
Wojcicki, et al	United States of America	2011	2006-2007	cohort	medical institution	CDC	45	145
Beck, et al	United States of America	2014	2014	cross-sectional	community	CDC	31	319
Badon, et al	United States of America	2020	2008-2014	cross-sectional	medical institution	CDC	10433	111482
Bai, et al	United States of America	2016	2012	cross-sectional	database	CDC	226	1114
Giammattei	United States of America	2003	2000-2001	cross-sectional	school	CDC	67	385
Barlow, et al	United States of America	2007	2003	cross-sectional	database	CDC	10041	52845
Barrera, et al	United States of America	2016	2005-2007	cross-sectional	database	CDC	142	1181
Baxter, et al	United States of America	2013	2004-2007	cross-sectional	school	CDC	376	1504
Baxter, et al	United States of America	2011	2004-2007	cross-sectional	school	CDC	276	920
Geier, et al	United States of America	2007	2007	cross-sectional	school	National Reference	245	1069
Shore, et al	United States of America	2008	2004-2005	cross-sectional	school	CDC	58	549
Pan, et al	United States of America	2013	2009	cross-sectional	database	CDC	413	3470
Kim, et al	United States of America	2018	2002-2003	longitudinal	school	CDC	347	2318
Seicean, et al	United States of America	2007	2004	cross-sectional	school	CDC	40	509
Lumeng, et al	United States of	2007	2003	cohort	database	CDC	139	785

© 2024 Zhang X et al. *JAMA Pediatrics.*

	America							
Silva, et al	United States of America	2011	2004-2009	cohort	database	CDC	46	304
Fiorito, et al	United States of America	2006	2006	cross-sectional	community	CDC	25	177
Charvet, et al	United States of America	2019	2019	cross-sectional	medical institution	CDC	30	197
Haidar, et al	United States of America	2019	2009-2011	cross-sectional	database	CDC	1498	6716
Hu, et al	United States of America	2018	2015	cross-sectional	database	CDC	2005	15624
Drake, et al	United States of America	2012	2002-2009	longitudinal	community	CDC	223	1718
Suglia, et al	United States of America	2013	2003-2005	cohort	database	CDC	270	1589
Drake, et al	United States of America	2013	2007-2008	cohort	school	CDC	96	479
Pérez, et al (1)	United States of America	2015	2000-2002	cross-sectional	database	CDC	1522	8938
Pérez, et al (2)	United States of America	2015	2004-2005	cross-sectional	database	CDC	2712	15283
Ghosh-Dastidar, et al	United States of America	2016	2013	cross-sectional	database	CDC	51	475
Berkowitz, et al	United States of America	2005	2005	cohort	community	CDC	6	78
Gillman, et al	United States of America	2008	2008	cohort	database	CDC	105	896
Hinkle, et al	United States of America	2012	2006	cohort	database	CDC	644	3600
Kubo, et al	United States of America	2016	2005-2012	cohort	database	CDC	58	417
Lindberg, et al	United States of America	2012	2012	cohort	database	CDC	128	471
Olson, et al	United States of America	2010	2009-2010	cohort	database	CDC	47	321
Wojcicki, et al	United States of America	2015	2005-2009	cohort	database	CDC	204	833
Han, et al	United States of America	2020	2009-2016	longitudinal	school	CDC	112793	486178
Jia, et al	United States of America	2019	2007	longitudinal	database	CDC	1468	7530
Elbel, et al	United States of America	2019	2013	cross-sectional	database	CDC	142995	735192
Reis, et al	United States of America	2020	2020	cross-sectional	school	CDC	67	171
Ohri-Vachaspati, et al	United States of America	2021	2009-2017	cross-sectional	database	CDC	115	449
Bailey, et al	United States of America	2014	2001-2013	cohort	medical institution	CDC	13359	89057

© 2024 Zhang X et al. *JAMA Pediatrics.*

Ville, et al	United States of America	2017	2012-2013	cohort	medical institution	CDC	11	97
Wojcicki, et al	United States of America	2016	2006-2007	cross-sectional	medical institution	CDC	82	200
Harley, et al	United States of America	2013	2000-2010	cohort	database	CDC	106	319
Huh, et al	United States of America	2012	2002-2005	cohort	medical institution	CDC	116	1255
Wang, et al	United States of America	2013	2000-2004	cohort	database	CDC	170	917
Peck, et al	United States of America	2015	2011	cohort	database	CDC	2751	24137
Kjaer, et al	United States of America	2018	2015-2016	cohort	medical institution	CDC	43	102
DuBose, et al	United States of America	2006	2006	cross-sectional	database	CDC	86	375
Heer, et al	United States of America	2013	2008	cross-sectional	school	CDC	173	653
Block, et al	United States of America	2018	2009-2016	cross-sectional	medical institution	CDC	48224	362550
Assari, et al	United States of America	2015	2003	cross-sectional	database	National Reference	287	1170
Boutelle, et al	United States of America	2010	2010	cross-sectional	community	CDC	53	495
Kubzansky, et al	United States of America	2012	2001-2005	cross-sectional	school	CDC	144	1528
McClure, et al	United States of America	2010	2003	cross-sectional	community	CDC	513	4458
Roberts, et al	United States of America	2013	2000	cross-sectional	database	CDC	822	4175
Vannucci, et al	United States of America	2017	2007/2009	cross-sectional	school	CDC	45	368
Adams, et al	United States of America	2005	2000-2004	cohort	database	CDC	56	252
Hack, et al	United States of America	2011	2004	longitudinal	medical institution	CDC	35	296
Vohr, et al	United States of America	2018	2018	cohort	database	CDC	39	388
Wood, et al	United States of America	2018	2012-2014	cohort	database	CDC	103	871
Cottrell, et al	United States of America	2011	2007-2008	cross-sectional	database	CDC	3734	17944
Vangeepuram, et al	United States of America	2011	2004-2007	cross-sectional	database	CDC	133	503
Kwon, et al	United States of America	2006	2002-2004	cross-sectional	database	CDC	188	853
Anderson, et al	United States of America	2014	2007-2011	cohort	database	CDC	1290	15141
Taveras, et al	United States of	2014	2014	cohort	medical institution	CDC	116	1046

© 2024 Zhang X et al. *JAMA Pediatrics*.

	America							
Scharf, et al	United States of America	2015	2001-2006	cohort	database	CDC	1396	8950
Storfer-Isser, et al	United States of America	2012	2002-2006	cohort	database	IOTF	24	313
Bell, et al	United States of America	2010	2002	cohort	database	CDC	300	1920
Roberts, et al	United States of America	2013	2000-2001	cross-sectional	school	CDC	868	4175
Anderson, et al	United States of America	2011	2003	cross-sectional	school	CDC	143	918
Lohman, et al	United States of America	2009	2009	cross-sectional	database	CDC	202	1011
Foster, et al	United States of America	2008	2008	RCT	school	CDC	321	1349
Chomitz, et al	United States of America	2010	2003-2004	cohort	school	CDC	375	1858
Shriver, et al	United States of America	2011	2008	cross-sectional	school	CDC	52	237
Eagle, et al	United States of America	2010	2004-2009	cross-sectional	database	CDC	150	1003
Lee, et al	United States of America	2010	2002	longitudinal	database	National Reference	78	401
Kunin-Batson, et al	United States of America	2023	2012-2014	longitudinal	database	CDC	121	534
Wu, et al	United States of America	2022	2018	longitudinal	database	CDC	6974	46151
White, et al (3)	United States of America	2022	2022	cohort	database	IOTF	401	2545
Arcan, et al	United States of America	2012	2005-2006	cross-sectional	school	CDC	63	413
Armstrong, et al	United States of America	2012	2008-2011	cross-sectional	medical institution	CDC	31	94
Rappaport, et al	United States of America	2005	2002	cross-sectional	community	CDC	603	2621
Janssen, et al (32)	United States of America	2005	2001-2002	cross-sectional	database	IOTF	302	4447
Flynn, et al	United States of America	2005	2003	cross-sectional	school	CDC	630	7048
Thorpe, et al	United States of America	2004	2003	cross-sectional	school	CDC	670	2681
Patrick, et al	United States of America	2004	2001-2002	cross-sectional	medical institution	CDC	242	878
Nelson, et al	United States of America	2004	2001	cross-sectional	database	CDC	121	556
Mirza, et al	United States of America	2004	2002	cross-sectional	medical institution	CDC	116	309
Drobac, et al	United States of America	2004	2001-2002	cross-sectional	medical institution	National Reference	258	997

© 2024 Zhang X et al. *JAMA Pediatrics*.

Davy, et al	United States of America	2004	2004	cross-sectional	school	CDC	66	205
Jiménez-Cruz, et al	United States of America	2003	2001-2002	cross-sectional	school	CDC	228	1200
Friedlander, et al	United States of America	2003	2003	cross-sectional	community	CDC	91	371
Eisenmann, et al	United States of America	2003	2003	cross-sectional	school	IOTF	62	263
Demerath, et al	United States of America	2003	2000	cross-sectional	school	CDC	362	1338
Rinderknecht, et al	United States of America	2002	2000	cross-sectional	community	CDC	64	155
Rinderknecht, et al	United States of America	2002	2002	cross-sectional	school	CDC	617	4240
Yang, et al	United States of America	2018	2014-2015	cross-sectional	school	CDC	9330	41283
Hidalgo-Mendez, et al	United States of America	2019	2019	cross-sectional	medical institution	CDC	48	186
Thundiyil, et al	United States of America	2010	2007	cross-sectional	medical institution	CDC	44	179
Amram, et al	United States of America	2020	2020	cross-sectional	school	CDC	1590	10327
Strickman-Stein, et al	United States of America	2010	2004-2006	cross-sectional	medical institution	CDC	38	200
Shankaran, et al	United States of America	2010	2010	cross-sectional	community	CDC	184	880
Shabbir, et al	United States of America	2010	2006-2007	cross-sectional	school	CDC	153591	670352
Rodriguez, et al	United States of America	2010	2007	cross-sectional	school	CDC	454	2038
Meininger, et al	United States of America	2010	2006	cross-sectional	school	CDC	307	1070
Grow, et al	United States of America	2010	2006	cross-sectional	community	CDC	1293	8616
Farhat, et al	United States of America	2010	2005-2006	cross-sectional	school	CDC	1132	7737
Elder, et al	United States of America	2010	2003-2004	cross-sectional	community	CDC	220	745
Dammann, et al	United States of America	2010	2010	cross-sectional	community	CDC	33	92
Bethell, et al	United States of America	2010	2007	cross-sectional	database	CDC	7233	44101
Beets, et al	United States of America	2010	2006	cross-sectional	database	CDC	495	6603
Bayles, et al	United States of America	2010	2010	cross-sectional	community	CDC	12	3699
Baranowski, et al	United States of America	2010	2010	cross-sectional	school	CDC	300	1551
Babey, et al(1)	United States of	2010	2001	cross-sectional	database	CDC	726	5858

© 2024 Zhang X et al. *JAMA Pediatrics.*

	America							
Babey, et al(2)	United States of America	2010	2003	cross-sectional	database	CDC	497	4010
Babey, et al(3)	United States of America	2010	2005	cross-sectional	database	CDC	572	4029
Babey, et al(4)	United States of America	2010	2007	cross-sectional	database	CDC	484	3638
Villa-Caballero, et al	United States of America	2009	2003-2004	cross-sectional	community	CDC	31	725
Valente, et al	United States of America	2009	2009	cross-sectional	school	CDC	90	562
Vader, et al	United States of America	2009	2000-2002	cross-sectional	school	CDC	2575	11594
Treuth, et al	United States of America	2009	2003-2005	longitudinal	school	CDC	535	3085
Trent, et al	United States of America	2009	2003-2004	cross-sectional	database	CDC	698	2630
Smith, et al	United States of America	2009	2005-2007	cross-sectional	school	CDC	116	251
Santos, et al	United States of America	2009	2006	cross-sectional	school	WHO	174	1195
Rundle, et al	United States of America	2009	2003-2006	cross-sectional	school	CDC	96	437
Nsiah-Kumi, et al	United States of America	2009	2003	cross-sectional	medical institution	National Reference	60	336
Montgomery-Reagan, et al	United States of America	2009	2006-2007	cross-sectional	school	CDC	1114	5306
Martyn-Nemeth, et al	United States of America	2009	2005	cross-sectional	school	CDC	28	102
Lim, et al	United States of America	2009	2002-2003	longitudinal	school	CDC	40	365
Krukowski, et al	United States of America	2009	2003	cross-sectional	school	CDC	214	1071
Hillman, et al	United States of America	2009	2004	cross-sectional	medical institution	CDC	94	397
Harnack, et al	United States of America	2009	2004-2006	cross-sectional	school	CDC	95	593
Harbaugh, et al	United States of America	2009	2005-2009	cross-sectional	school	CDC	258	1250
Franzini, et al	United States of America	2009	2003	cross-sectional	community	CDC	163	650
Dorsey, et al	United States of America	2009	2005	cross-sectional	medical institution	CDC	30	75
Dixon, et al	United States of America	2009	2001	cross-sectional	school	National Reference	49	112
De La O, et al	United States of America	2009	2009	cross-sectional	school	CDC	39	576
Anderson, et al	United States of America	2009	2005	cross-sectional	database	CDC	1573	8550

© 2024 Zhang X et al. *JAMA Pediatrics.*

West, et al	United States of America	2008	2003-2004	cross-sectional	school	CDC	326	1551
Steele, et al	United States of America	2008	2001-2004	cross-sectional	medical institution	CDC	572	3221
Pollack, et al	United States of America	2008	2000-2006	cross-sectional	database	CDC	588	3232
Margellos-Anast, et al	United States of America	2008	2002-2003	cross-sectional	database	CDC	217	501
Laurson, et al	United States of America	2008	2001	longitudinal	community	IOTF	27	268
Kapoor, et al	United States of America	2008	2008	cross-sectional	school	CDC	284	1058
Kalich, et al	United States of America	2008	2003	cross-sectional	school	CDC	147	786
Irigoyen, et al	United States of America	2008	2004-2005	cross-sectional	medical institution	CDC	384	1713
Eisenmann, et al	United States of America	2008	2001	cross-sectional	database	IOTF	1471	12464
Eichner, et al	United States of America	2008	2002-2003	cross-sectional	school	IOTF	416	1706
Wald, et al	United States of America	2007	2004	cross-sectional	medical institution	CDC	140	612
Roseman, et al	United States of America	2007	2004	cross-sectional	school	CDC	607	4049
Metallinos-Katsaras, et al	United States of America	2007	2000	cross-sectional	medical institution	CDC	13	56
Martin, et al	United States of America	2007	2003-2004	cross-sectional	community	CDC	67	212
Malaty, et al	United States of America	2007	2001-2002	cross-sectional	school	CDC	139	552
Lynch, et al	United States of America	2007	2002-2003	cross-sectional	school	CDC	494	2055
Hernández-Valero, et al	United States of America	2007	2001-2003	cross-sectional	community	CDC	118	438
Eisenmann, et al	United States of America	2007	2007	cross-sectional	school	IOTF	132	813
Akridge, et al	United States of America	2007	2004	cross-sectional	medical institution	IOTF	20	107
Zephier, et al	United States of America	2006	2002-2003	cross-sectional	school	CDC	3006	10821
Nelson, et al	United States of America	2006	2003	cross-sectional	database	CDC	95	526
Pobutsky, et al	United States of America	2006	2002-2003	cross-sectional	school	CDC	1469	10199
Mulvaney, et al	United States of America	2006	2006	cross-sectional	school	CDC	59	402
Trapp, et al	United States of America	2015	2010-2011	cross-sectional	medical institution	CDC	65	222
Tomayko, et al	United States of	2015	2007-2012	cross-sectional	database	CDC	6289	53775

© 2024 Zhang X et al. *JAMA Pediatrics*.

	America							
Scherrer, et al	United States of America	2015	2008-2011	cross-sectional	database	CDC	5153	28792
Nobari, et al	United States of America	2015	2011	cross-sectional	database	National Reference	365	2051
Nagata, et al	United States of America	2015	2006-2007	cross-sectional	medical institution	CDC	47	174
Lumeng, et al	United States of America	2015	2005-2013	longitudinal	medical institution	CDC	5166	43748
Katzmarzyk, et al (13)	United States of America	2015	2011-2013	cross-sectional	database	WHO	87	491
Jin, et al	United States of America	2015	2010-2012	cross-sectional	school	CDC	328332	1617400
Jackson, et al	United States of America	2015	2012-2014	cross-sectional	database	CDC	14	102
Gunter, et al	United States of America	2015	2013	cross-sectional	school	CDC	288	1482
Ansari, et al	United States of America	2015	2006	prospective	school	CDC	478	2810
Weedn, et al (1)	United States of America	2014	2005	cross-sectional	community	CDC	4641	35424
Weedn, et al (2)	United States of America	2014	2006	cross-sectional	community	CDC	4496	34061
Weedn, et al (3)	United States of America	2014	2007	cross-sectional	community	CDC	4768	34303
Weedn, et al (4)	United States of America	2014	2008	cross-sectional	community	CDC	4924	35426
Weedn, et al (5)	United States of America	2014	2010	cross-sectional	community	CDC	5737	40121
Nichols, et al (1)	United States of America	2014	2003	cross-sectional	database	National Reference	103	415
Nichols, et al (2)	United States of America	2014	2004	cross-sectional	database	National Reference	130	414
Nichols, et al (3)	United States of America	2014	2005	cross-sectional	database	National Reference	90	358
Nichols, et al (4)	United States of America	2014	2006	cross-sectional	database	National Reference	55	179
Nichols, et al (5)	United States of America	2014	2007	cross-sectional	database	National Reference	53	220
Nichols, et al (6)	United States of America	2014	2008	cross-sectional	database	National Reference	55	182
Nichols, et al (7)	United States of America	2014	2009	cross-sectional	database	National Reference	69	234
Nichols, et al (8)	United States of America	2014	2010	cross-sectional	database	National Reference	85	278
Nichols, et al (9)	United States of America	2014	2011	cross-sectional	database	National Reference	68	183
Nguyen, et al	United States of America	2014	2010	cross-sectional	medical institution	CDC	126	691

© 2024 Zhang X et al. *JAMA Pediatrics.*

Lo, et al	United States of America	2014	2007-2010	cross-sectional	database	CDC	4775	42559
Cui, et al	United States of America	2014	2001-2010	cross-sectional	database	CDC	1414	7031
Dammann, et al	United States of America	2011	2011	cross-sectional	community	CDC	82	257
Florin, et al	United States of America	2011	2003	cross-sectional	database	CDC	1178	11012
Hill, et al	United States of America	2011	2005	cross-sectional	school	CDC	83	649
Isasi, et al	United States of America	2011	2008	cross-sectional	school	CDC	362	1607
Mulasi-Pokhriyal, et al	United States of America	2011	2011	cross-sectional	community	CDC	102	335
Nervik, et al	United States of America	2011	2009	cross-sectional	medical institution	CDC	4	50
Pérez, et al	United States of America	2011	2004-2005	cross-sectional	school	CDC	4860	23190
Spruyt, et al	United States of America	2011	2011	cross-sectional	community	CDC	100	308
Xanthopoulos, et al	United States of America	2011	2011	cross-sectional	school	CDC	293	1212
Gamble, et al	United States of America	2012	2009-2010	cross-sectional	community	CDC	327	1136
Weedn, et al	United States of America	2012	2009	cross-sectional	database	CDC	5364	39151
Dodd, et al	United States of America	2013	2004-2005	cross-sectional	school	CDC	528	2314
Loth, et al	United States of America	2013	2010	cross-sectional	database	CDC	516	2231
Novotny, et al	United States of America	2013	2010	cross-sectional	medical institution	CDC	906	4599
Nunez-Gaunaurd, et al	United States of America	2013	2010-2011	cross-sectional	school	CDC	19	86
Pan, et al	United States of America	2013	2008	prospective	database	CDC	160010	1204839
Novotny, et al	United States of America	2016	2011-2013	cross-sectional	community	CDC	778	5558
Stiefel, et al	United States of America	2016	2009-2013	cross-sectional	medical institution	CDC	1811	7705
Zeller, et al	United States of America	2016	2008-2009	cross-sectional	school	CDC	2401	19678
Novotny, et al	United States of America	2017	2013	cross-sectional	database	CDC	765	5462
Sadeghi, et al	United States of America	2017	2013	cross-sectional	community	CDC	158	609
Pengpid, et al (6)	Vanuatu	2015	2010-2011	cross-sectional	database	IOTF	4	1119
Le, et al	Vietnam	2022	2021	cross-sectional	school	WHO	112	782
Dieu, et al	Vietnam	2007	2005	cross-sectional	school	IOTF	109	670

© 2024 Zhang X et al. *JAMA Pediatrics.*

Carrillo-Larco, et al (3)	Vietnam	2014	2014	cohort	database	IOTF	53	1910
Phan, et al	Vietnam	2020	2018	cross-sectional	community	WHO	240	2788
Pham, et al	Vietnam	2019	2014	cross-sectional	community	WHO	157	821
Trang, et al	Vietnam	2010	2004	cross-sectional	school	IOTF	54	2660
Mai, et al	Vietnam	2020	2014-2015	cross-sectional	school	IOTF	1220	10949
Pham, et al	Vietnam	2020	2016	cross-sectional	school	IOTF	508	2764
Tang, et al	Vietnam	2007	2002	cross-sectional	school	IOTF	9	1504
Hong, et al (1)	Vietnam	2007	2002	cross-sectional	school	IOTF	8	1003
Hong, et al (2)	Vietnam	2007	2004	cross-sectional	school	IOTF	55	2684
Nguyen, et al	Vietnam	2013	2010	cross-sectional	database	WHO	64	1989
Ngan, et al	Vietnam	2018	2012	cross-sectional	school	WHO	28	276
Taguri, et al (4)	Yemen	2009	2003	cross-sectional	database	WHO	975	10924
Gebremedhin, et al (25)	Zambia	2015	2013/2014	cross-sectional	database	WHO	397	11677
Gebremedhin, et al (26)	Zimbabwe	2015	2010/2011	cross-sectional	database	WHO	132	4405
Kambondo, et al	Zimbabwe	2018	2015	cross-sectional	school	IOTF	75	974

eTable 4. Characteristics of the studies fore prevalence of overweight in children and adolescents.

Study	Country or Region	Publication Year	Study Period	Study Design	Sample Source	Diagnostic Reference	No. of Overweight	Sample Size
Salas, et al (1)	Albania	2021	2015-2017	cross-sectional	database	WHO	339	2259
Hyska, et al	Albania	2014	2013	cross-sectional	school	WHO	435	5810
Benmohammed, et al	Algeria	2020	2007	cross-sectional	school	IOTF	179	1100
Musaiger, et al (1)	Algeria	2012	2010-2011	cross-sectional	school	IOTF	59	459
Fedala, et al	Algeria	2017	2013-2014	cross-sectional	school	IOTF	160	2278
Orden, et al	Argentina	2019	2015-2016	cross-sectional	school	IOTF	278	1366
Meyer, et al	Argentina	2013	2010-2011	cross-sectional	database	IOTF	2881	15541
Hirschler, et al	Argentina	2010	2005	cross-sectional	school	CDC	300	1564
Stray-Pedersen,et al	Argentina	2009	2004-2005	cross-sectional	database	IOTF	97	669
Hirschler, et al	Argentina	2008	2006	cross-sectional	school	CDC	108	621
Hirschler, et al	Argentina	2008	2006-2007	cross-sectional	community	CDC	169	1027
Hirschler, et al	Argentina	2006	2004	cross-sectional	school	CDC	61	321
Kovalskys, et al	Argentina	2011	2005	cross-sectional	school	CDC	257	1588
Tringler, et al	Argentina	2012	2007-2008	cross-sectional	database	CDC	47	334
Catalani, et al	Argentina	2016	2014	cross-sectional	school	National Reference	188	711
Gotthelf, et al	Argentina	2017	2015	cross-sectional	school	WHO	78	283
Rivero, et al	Argentina	2018	2017	cross-sectional	community	WHO	85	303
Abbott, et al	Australia	2010	2006	cross-sectional	database	IOTF	481	3043
Crawford, et al	Australia	2008	2004	cross-sectional	community	IOTF	84	380
Franklin, et al	Australia	2006	2006	cross-sectional	community	CDC	359	2743
Wake, et al	Australia	2013	2000-2006	cross-sectional	community	IOTF	2657	16339
James, et al	Australia	2013	2001-2005	cross-sectional	community	IOTF	1987	17008
Marshall, et al	Australia	2012	2005	cross-sectional	community	IOTF	154	691
Martin, et al	Australia	2012	2005	cross-sectional	community	IOTF	85	408
O'Dea, et al (1)	Australia	2010	2000	cross-sectional	community	IOTF	623	3819

© 2024 Zhang X et al. *JAMA Pediatrics.*

O'Dea, et al (2)	Australia	2010	2006	cross-sectional	community	IOTF	1050	5524
O'Dea, et al	Australia	2014	2007	longitudinal	school	IOTF	155	939
Schofield, et al	Australia	2009	2002	cross-sectional	school	IOTF	94	415
Gopinath, et al	Australia	2012	2003-2005	cross-sectional	school	IOTF	705	4094
Spurrier, et al	Australia	2012	2009	cross-sectional	school	IOTF	1685	11859
Trapp, et al	Australia	2011	2007	cross-sectional	database	IOTF	237	1197
Waters, et al	Australia	2008	2004-2005	cross-sectional	school	IOTF	578	2685
Bell, et al	Australia	2018	2013-2014	cohort	database	WHO	86	953
Maher, et al	Australia	2012	2012	cross-sectional	database	IOTF	418	2200
Hayes, et al	Australia	2021	2004	cohort	database	WHO	2239	9225
Miller, et al	Australia	2014	2005-2010	cross-sectional	community	CDC	303	1850
Achat, et al	Australia	2014	2007	cross-sectional	school	IOTF	447	2341
O'Dea, et al	Australia	2008	2006	cross-sectional	school	IOTF	1435	7889
O'Sullivan, et al	Australia	2015	2003-2005	cross-sectional	database	IOTF	257	1416
Barnes, et al	Australia	2021	2017	RCT	school	WHO	168	815
Cox, et al	Australia	2012	2010	cross-sectional	community	CDC	15	135
Wickramasinghe, et al	Australia	2005	2005	cross-sectional	community	CDC	19	138
Telford, et al	Australia	2008	2007	cross-sectional	database	CDC	114	741
Seach, et al	Australia	2010	2000-2004	cohort	medical institution	IOTF	77	307
Jansen, et al	Australia	2013	2010	cross-sectional	database	IOTF	659	3197
Tai, et al	Australia	2009	2006	cross-sectional	school	IOTF	199	1457
Bergmeier, et al	Australia	2014	2009-2011	longitudinal	community	CDC	20	201
Hardy, et al	Australia	2012	2010	cross-sectional	database	IOTF	157	1141
White, et al (2)	Australia	2022	2022	cohort	database	IOTF	661	3998
Rehor, et al	Australia	2002	2001	cross-sectional	school	IOTF	60	329
Wen, et al	Australia	2010	2006	cross-sectional	school	IOTF	172	964
Haug, et al (2)	Australia	2009	2005-2006	cross-sectional	database	IOTF	424	4509
Sutherland, et al	Australia	2008	2004	cross-sectional	school	IOTF	415	2224
Denney-Wilson, et al	Australia	2008	2004	cross-sectional	database	IOTF	91	496
Fisher, er al	Australia	2006	2002	cross-sectional	school	CDC	39	296
Cretikos, et al	Australia	2008	2002-2006	cross-sectional	database	IOTF	2365	12925
Crawford, et al	Australia	2006	2001	cross-sectional	school	IOTF	81	1141
Campbell, et al	Australia	2006	2002	cross-sectional	database	IOTF	54	324
Burke, et al	Australia	2006	2006	cross-sectional	school	IOTF	99	570
Sanigorski, et al	Australia	2007	2003-2004	cross-sectional	community	IOTF	422	2184
Cochrane, et al	Australia	2015	2000-2011	cross-sectional	school	IOTF	5664	31424
Chen, et al (1)	Australia	2014	2010-2011	cross-sectional	community	IOTF	5	89
Keating, et al	Australia	2011	2005-2006	cross-sectional	database	IOTF	583	2890
Olds, et al	Australia	2011	2007	cross-sectional	database	IOTF	418	2200
Morley, et al	Australia	2012	2009-2010	cross-sectional	school	IOTF	2243	12188
Schultz, et al	Australia	2012	2012	cross-sectional	database	WHO	137	996
Fredrickson, et al	Australia	2013	2005-2008	cross-sectional	database	WHO	650	2954
Furthner, et al	Australia	2017	2012-2013	cross-sectional	school	National Reference	494	2916
Furthner, et al	Austria	2018	2012-2013	cross-sectional	school	National Reference	495	2930
Yngve, et al (1)	Austria	2008	2003	cross-sectional	database	IOTF	186	1181
Janssen, et al (1)	Austria	2005	2001-2002	cross-sectional	database	IOTF	435	3994
Pfeiffer, et al	Austria	2006	2006	cross-sectional	school	National	53	835

© 2024 Zhang X et al. *JAMA Pediatrics*.

						Reference		
Romano, et al (1)	Bahamas	2022	2013	cross-sectional	database	WHO	319	1308
Al-Raees, et al	Bahrain	2009	2009	cross-sectional	community	WHO	70	698
Al-Sendi, et al	Bahrain	2003	2000	cross-sectional	school	IOTF	101	506
Musaiger, et al	Bahrain	2014	2003-2005	cross-sectional	school	IOTF	289	2146
Anam, et al	Bangladesh	2022	2019	cross-sectional	school	WHO	187	1044
Taher, et al	Bangladesh	2021	2016-2018	cross-sectional	school	CDC	254	1450
Sultana, et al	Bangladesh	2015	2015	cross-sectional	school	National Reference	84	150
Sultana, et al	Bangladesh	2016	2016	cross-sectional	school	WHO	78	500
Saha, et al	Bangladesh	2018	2014	cross-sectional	school	CDC	25	288
Romano, et al (2)	Bangladesh	2022	2014	cross-sectional	database	WHO	223	2753
Sultana, et al	Bangladesh	2019	2012-2013	cross-sectional	school	IOTF	112	1768
Bulbul, et al	Bangladesh	2014	2009	cross-sectional	school	WHO	977	10135
Romano, et al (3)	Barbados	2022	2011	cross-sectional	database	WHO	266	1504
Brug, et al (1)	Belgium	2012	2010	cross-sectional	database	IOTF	121	1003
Ahrens, et al (4)	Belgium	2014	2007-2008	cross-sectional	database	IOTF	168	2352
Usheva, et al (1)	Belgium	2021	2012	cross-sectional	database	WHO	47	1128
Júlíusson, et al (1)	Belgium	2015	2002-2006	cross-sectional	database	IOTF	1235	12200
Yngve, et al (2)	Belgium	2008	2003	cross-sectional	database	IOTF	88	965
Seghers, et al	Belgium	2010	2006-2007	cross-sectional	school	IOTF	104	798
Vriendt, et al	Belgium	2009	2004-2005	cross-sectional	school	IOTF	130	982
Janssen, et al (2)	Belgium	2005	2001-2002	cross-sectional	database	IOTF	505	5876
Janssen, et al (3)	Belgium	2005	2001-2002	cross-sectional	database	IOTF	313	3066
Gysel, et al	Belgium	2009	2004-2005	cross-sectional	database	IOTF	228	1576
Haug, et al (3)	Belgium	2009	2005-2006	cross-sectional	database	IOTF	309	3966
Haug, et al (4)	Belgium	2009	2005-2006	cross-sectional	database	IOTF	304	3267
Vissers, et al	Belgium	2008	2008	cross-sectional	school	IOTF	98	994
Huybrechts, et al	Belgium	2006	2006	cross-sectional	school	IOTF	30	297
Velde, et al (1)	Belgium	2017	2010	cross-sectional	database	IOTF	121	996
Manyanga, et al (1)	Benin	2014	2006/2010	cross-sectional	database	WHO	284	2681
Romano, et al (4)	Benin	2022	2016	cross-sectional	database	WHO	92	717
Norbu, et al	Bhutan	2019	2019	cross-sectional	school	CDC	138	392
Botti, et al	Bolivia	2010	2007	cross-sectional	database	WHO	466	3306
Romano, et al (5)	Bolivia	2022	2012	cross-sectional	database	WHO	485	2804
Pe´rez-Cueto, et al	Bolivia	2005	2003	cross-sectional	school	IOTF	104	525
Benefice, et al	Bolivia	2007	2004-2005	cross-sectional	community	IOTF	47	385
Spahić, et al	Bosnia and Herzegovina	2019	2016-2017	cross-sectional	school	CDC	359	2500
Hansanbegović, et al	Bosnia and Herzegovina	2010	2008-2009	cross-sectional	school	CDC	428	3608
Wrotniak, et al	Botswana	2012	2012	cross-sectional	school	WHO	87	707
Alexius, et al	Brazil	2012	2007	cross-sectional	community	IOTF	120	1048
Andaki, et al (1)	Brazil	2017	2009-2011	cross-sectional	community	IOTF	313	2423
Ataíde Lima, et al	Brazil	2015	2008-2010	cross-sectional	community	WHO	40	203

© 2024 Zhang X et al. *JAMA Pediatrics*.

Castilho, et al	Brazil	2014	2010-2012	cross-sectional	community	WHO	659	3130
Costa, et al	Brazil	2015	2012-2013	cross-sectional	community	WHO	356	1530
Fraiz, et al	Brazil	2019	2019	cross-sectional	community	WHO	114	686
Guedes, et al	Brazil	2011	2007	cross-sectional	community	WHO	497	5100
Kupek, et al	Brazil	2016	2007	cross-sectional	community	WHO	294	1232
Moreira, et al	Brazil	2012	2007	cross-sectional	community	WHO	63	963
Oppitz, et al	Brazil	2014	2008	cross-sectional	community	WHO	103	1640
Sliva, et al	Brazil	2018	2012-2013	cross-sectional	community	National Reference	195	1125
Pereira, et al	Brazil	2023	2018-2019	cross-sectional	database	WHO	332	1060
Santos, et al	Brazil	2022	2012-2013	cross-sectional	school	WHO	84	402
Coelho, et al	Brazil	2022	2019	cross-sectional	community	WHO	39	170
Blumenberg, et al	Brazil	2021	2015	longitudinal	community	WHO	317	874
Barbiero, et al	Brazil	2009	2009	cross-sectional	school	WHO	91	511
Barbosa, et al	Brazil	2021	2021	cross-sectional	school	WHO	64	353
Cândido, et al	Brazil	2009	2006	cross-sectional	school	CDC	64	779
Polderman, et al	Brazil	2011	2008	cross-sectional	school	IOTF	137	1002
Novaes, et al	Brazil	2013	2013	cross-sectional	school	WHO	144	769
Duncan, et al	Brazil	2011	2011	cross-sectional	school	IOTF	600	3397
Halal, et al	Brazil	2016	2008	longitudinal	community	WHO	302	4231
Caixeta, et al	Brazil	2020	2016-2017	cross-sectional	school	CDC	48	486
Ferreira, et al	Brazil	2008	2003	cross-sectional	school	IOTF	49	412
Rodrigues, et al	Brazil	2006	2003-2005	cross-sectional	school	IOTF	32	380
Amorim, et al	Brazil	2006	2003	cross-sectional	school	IOTF	144	1719
Araújo, et al	Brazil	2010	2004-2005	cohort	medical institution	WHO	515	4452
Flores, et al	Brazil	2013	2005-2006	cross-sectional	database	National Reference	4506	20514
Bispo, et al	Brazil	2015	2008-2009	cross-sectional	database	WHO	142	1030
Santana, et al	Brazil	2013	2009	cross-sectional	school	WHO	131	1494
Lock, et al	Brazil	2020	2009-2010	cross-sectional	school	WHO	335	1528
Cruz, et al	Brazil	2013	2010-2011	cross-sectional	school	IOTF	88	523
Moraes, et al	Brazil	2019	2010	longitudinal	database	WHO	168	673
Guedes, et al	Brazil	2013	2011	cross-sectional	school	IOTF	311	1968
Rosini, et al	Brazil	2015	2009	cross-sectional	school	WHO	213	1011
Neves, et al	Brazil	2015	2012	cross-sectional	school	WHO	82	411
Costa, et al	Brazil	2020	2020	cross-sectional	school	WHO	195	1334
Vieira, et al	Brazil	2015	2013	cross-sectional	school	WHO	71	347
Araujo, et al	Brazil	2018	2014	cross-sectional	school	WHO	159	1182
Carmo, et al	Brazil	2018	2014-2016	cross-sectional	school	WHO	37	405
Fradkin, et al	Brazil	2018	2014-2016	cross-sectional	school	CDC	199	1738
Reuter, et al	Brazil	2018	2014-2015	cross-sectional	school	WHO	199	1200
Ripka, et al	Brazil	2017	2015-2016	cross-sectional	school	WHO	76	374
Silva, et al	Brazil	2020	2015	cohort	database	WHO	222	862
Schwertner, et al	Brazil	2020	2020	cross-sectional	school	IOTF	58	330
Dantas, et al	Brazil	2018	2017	cross-sectional	school	IOTF	105	578
Assis, et al	Brazil	2005	2002	cross-sectional	school	IOTF	487	2936
Salas, et al	Brazil	2018	2010	cross-sectional	school	IOTF	257	1211
Dalmaso, et al	Brazil	2019	2019	cross-sectional	community	IOTF	119	572
Lima, et al	Brazil	2019	2019	cross-sectional	school	National Reference	123	1169
Porcelli, et	Brazil	2019	2012	cross-sectional	medical	WHO	44	191

© 2024 Zhang X et al. *JAMA Pediatrics*.

al					institution			
Alves, et al	Brazil	2020	2013-2014	cross-sectional	database	WHO	11967	71298
Folmann, et al	Brazil	2020	2013-2014	cross-sectional	school	WHO	250	1715
Hércules, et al	Brazil	2020	2016-2017	cross-sectional	database	WHO	16190	80782
Lucena, et al	Brazil	2020	2020	cross-sectional	community	WHO	212	1487
Rocha, et al	Brazil	2020	2017	cross-sectional	database	WHO	223	2059
Rivera, et al	Brazil	2010	2001	cross-sectional	school	National Reference	116	1253
Gabriel, et al	Brazil	2010	2007-2008	cross-sectional	school	IOTF	766	4964
Anwar, et al	Brazil	2010	2007	cross-sectional	school	CDC	157	988
Molina, et al	Brazil	2009	2007	cross-sectional	school	IOTF	183	1251
Fernandes, et al	Brazil	2009	2007	cross-sectional	school	IOTF	299	1779
Pelegrini, et al	Brazil	2008	2004-2005	cross-sectional	database	IOTF	4399	36976
Bertolace, et al	Brazil	2008	2003	cross-sectional	school	CDC	52	421
Oliveira, et al	Brazil	2007	2007	cross-sectional	school	IOTF	64	699
da Silva, et al	Brazil	2007	2002	cross-sectional	community	National Reference	45	471
Weinmayr, et al (1)	Brazil	2014	2004	cross-sectional	database	IOTF	368	953
Moreira, et al	Brazil	2014	2005-2006	cross-sectional	community	WHO	353	1115
Mendonça, et al	Brazil	2014	2010-2011	cross-sectional	school	WHO	169	1168
Crispim, et al	Brazil	2014	2011-2012	cross-sectional	school	WHO	49	276
Nascimento, et al	Brazil	2012	2009	cross-sectional	medical institution	WHO	22	447
Rosaneli, et al	Brazil	2012	2006	cross-sectional	school	WHO	856	5037
Reuter, et al	Brazil	2013	2013	cross-sectional	school	CDC	71	564
Andrade, et al	Brazil	2016	2016	cross-sectional	school	WHO	52	396
Casonatto, et al	Brazil	2016	2002-2005	cross-sectional	database	IOTF	190	978
Jardim-Botelho, et al	Brazil	2016	2009-2012	cross-sectional	community	WHO	37	153
Silva, et al	Brazil	2016	2004	cross-sectional	school	WHO	214	2180
Araujo, et al	Brazil	2017	2017	cross-sectional	community	WHO	45	548
Cuesta, et al	Brazil	2018	2013	cross-sectional	community	WHO	318	1296
Dos Santos, et al	Brazil	2018	2018	cross-sectional	school	IOTF	76	501
Pivatto, et al	Brazil	2018	2016	cross-sectional	school	WHO	51	236
Romano, et al (6)	Brunei Darussalam	2022	2014	cross-sectional	database	WHO	341	1824
Usheva, et al (2)	Bulgaria	2021	2012	cross-sectional	database	WHO	76	874
Salas, et al (2)	Bulgaria	2021	2015-2017	cross-sectional	database	WHO	525	3238
Mladenova, et al	Bulgaria	2015	2012-2014	cross-sectional	school	IOTF	157	878
Gorog, et al (1)	Bulgaria	2011	2011	cross-sectional	database	IOTF	326	1554
Haug, et al (19)	Bulgaria	2009	2005-2006	cross-sectional	database	IOTF	516	4563
Wijnhoven, et al (1)	Bulgaria	2015	2007/2008	cross-sectional	school	WHO	1045	3627
Mank, et al	Burkina Faso	2022	2020	cross-sectional	school	WHO	115	1059
Daboné, et al	Burkina Faso	2011	2008-2009	cross-sectional	school	WHO	11	649
Gebremedhin, et al (2)	Burkina Faso	2015	2010	cross-sectional	database	WHO	175	6723
Gebremedhin, et al (1)	Burundi	2015	2010	cross-sectional	database	WHO	112	3493
Navti, et al	Cameroon	2021	2010	cross-sectional	school	WHO	180	1274

© 2024 Zhang X et al. *JAMA Pediatrics.*

(1)								
Navti, et al (2)	Cameroon	2021	2020	cross-sectional	school	WHO	230	1550
Gebremedhin, et al (3)	Cameroon	2015	2011	cross-sectional	database	WHO	337	5185
Choukem, et al	Cameroon	2017	2013	cross-sectional	school	WHO	129	1343
Chelo, et al	Cameroon	2019	2017-2018	cross-sectional	school	National Reference	55	822
Wamba, et al	Cameroon	2013	2010	cross-sectional	database	IOTF	282	2689
Navti ,et al	Cameroon	2017	2017	cross-sectional	school	WHO	78	522
Hulst, et al	Canada	2022	2001	longitudinal	database	WHO	274	1226
Anderson, et al	Canada	2022	2013-2019	cross-sectional	database	WHO	705	5962
Le, et al	Canada	2016	2011	cross-sectional	database	WHO	323	1331
Seliske, et al	Canada	2009	2005-2006	cross-sectional	database	IOTF	1278	7987
Ball, et al	Canada	2019	2010/2017	cross-sectional	database	WHO	26581	161114
Chaput, et al	Canada	2006	2003	cross-sectional	school	IOTF	64	422
Chaput, et al	Canada	2011	2005-2008	cohort	database	CDC	104	550
Davidson, et al	Canada	2016	2009-2011	cross-sectional	community	CDC	41	235
Brault, et al	Canada	2015	2010	cross-sectional	school	IOTF	103	786
Larsen, et al	Canada	2015	2010-2011	cross-sectional	database	IOTF	145	943
Cassidy-Bushrow, et al	Canada	2018	2003-2007	cohort	database	CDC	45	527
Sheilds, et al	Canada	2010	2004	cross-sectional	database	CDC	1377	8661
Medehouenou, et al	Canada	2015	2005-2010	cross-sectional	database	CDC	38	290
Wang, et al	Canada	2008	2003	cross-sectional	database	IOTF	1162	4945
Shi, et al	Canada	2013	2007-2009	cross-sectional	database	WHO	182	968
Oliver, et al	Canada	2005	2000-2001	cross-sectional	database	IOTF	584600	3190300
MOFFAT, et al	Canada	2005	2002-2004	cross-sectional	school	CDC	50	266
Woodruff, et al	Canada	2010	2005-2006	cross-sectional	school	IOTF	120	1293
Twells, et al	Canada	2010	2005	cross-sectional	school	CDC	196	1026
Simen-Kapeu, et al	Canada	2010	2008-2010	cross-sectional	school	IOTF	740	3421
Leatherdale, et al	Canada	2010	2007	cross-sectional	school	CDC	220	1264
Khaili, et al	Canada	2010	2005-2007	cross-sectional	community	CDC	20	125
Ismailov, et al	Canada	2010	2005-2006	cross-sectional	school	CDC	3634	25416
Galloway, et al	Canada	2010	2007-2008	cross-sectional	community	CDC	102	376
Doan, et al	Canada	2010	2005	cross-sectional	community	IOTF	1805	12170
Cairney, et al	Canada	2010	2005-2007	longitudinal	school	IOTF	649	2278
Bengoecher, et al	Canada	2010	2010	cross-sectional	database	IOTF	368	3159
Wahi, et al	Canada	2009	2009	cross-sectional	community	CDC	5	30
Vance, et al	Canada	2009	2002-2003	cross-sectional	school	IOTF	326	1917
Potestio, et al	Canada	2009	2005-2006	cross-sectional	school	IOTF	785	6772
Downs, et al	Canada	2009	2004-2005	cross-sectional	community	IOTF	60	201
Salvadori, et al	Canada	2008	2004	prospective	database	CDC	122	675
Edwards, et al	Canada	2008	2004-2005	cross-sectional	medical institution	CDC	1014	7369
Bruner, et al	Canada	2008	2001-2002	cross-sectional	database	IOTF	893	4851
Willows, et al	Canada	2007	2002	cross-sectional	school	IOTF	330	1044
He, et al	Canada	2007	2001-2003	cross-sectional	school	CDC	54	335
Bassett, et al	Canada	2007	2005	cross-sectional	school	IOTF	10	139
Ng, et al	Canada	2006	2004	cross-sectional	school	IOTF	24	82
Janssen, et al	Canada	2006	2001	cross-sectional	database	IOTF	1283	6684
Galloway, et	Canada	2006	2004	cross-sectional	school	CDC	89	487

© 2024 Zhang X et al. *JAMA Pediatrics.*

al								
Rossiter, et al	Canada	2015	2011	cross-sectional	database	IOTF	1188	5560
Borghese, et al	Canada	2015	2012-2013	cross-sectional	school	CDC	68	550
Banerjee, et al	Canada	2015	2010	cross-sectional	database	IOTF	148	734
Carson, et al	Canada	2014	2010-2011	cross-sectional	school	IOTF	154	787
Leatherdale, et al	Canada	2013	2007-2008	cross-sectional	database	IOTF	321	2326
Agüero, et al	Chile	2016	2014	cross-sectional	school	National Reference	451	1810
Olivares, et al	Chile	2004	2004	cross-sectional	school	CDC	344	1701
Cediel, et al	Chile	2016	2009-2010	cohort	database	WHO	120	435
Delgado-Floody, et al	Chile	2019	2019	cross-sectional	school	CDC	153	605
Corvalán, et al	Chile	2010	2006	cross-sectional	school	WHO	144	324
Valenzuela, et al	Chile	2015	2010-2011	cross-sectional	school	CDC	270	1477
Lizana, et al	Chile	2015	2013	cross-sectional	school	CDC	49	206
Cadenas-Sánchez, et al	Chile	2015	2013	cross-sectional	school	WHO	126	434
Heitzinger, et al	Chile	2014	2009-2010	cross-sectional	medical institution	CDC	247	795
Silva, et al	Chile	2013	2007	cross-sectional	community	National Reference	116	453
Kagawa, et al	Chile	2016	2012	cross-sectional	database	WHO	1962	11207
Liu, et al	China mainland	2016	2013	cross-sectional	school	National Reference	1059	10587
Li, et al	China mainland	2015	2012	cross-sectional	school	WHO	364	2400
Tang, et al	China mainland	2022	2003	cohort	database	WHO	7657	101505
Shi, et al	China mainland	2022	2019-2020	cross-sectional	school	National Reference	9304	105181
Shi, et al	China mainland	2022	2016-2017	cross-sectional	database	National Reference	6656	54269
Liu, et al	China mainland	2022	2019	cross-sectional	community	National Reference	697	7664
Liu, et al	China mainland	2022	2017	cross-sectional	school	National Reference	1813	10753
Li, et al	China mainland	2022	2020	cross-sectional	school	IOTF	3090	18176
Huang, et al	China mainland	2022	2015	cross-sectional	database	IOTF	899	8053
He, et al	China mainland	2022	2019-2020	longitudinal	database	WHO	546	5963
Guo, et al	China mainland	2022	2016	cross-sectional	database	WHO	3605	26120
Chen, et al	China mainland	2022	2014	cross-sectional	database	National Reference	1734	17356
Zhu, et al	China mainland	2021	2018	cross-sectional	school	National Reference	1780	12860
Zheng, et al	China mainland	2021	2015-2017	longitudinal	school	National Reference	567	3313
Zhao, et al (1)	China mainland	2021	2002	cross-sectional	school	National Reference	1945	26644
Zhao, et al (2)	China mainland	2021	2018	cross-sectional	school	National Reference	5632	45417
Zhang, et al	China mainland	2021	2016	cross-sectional	community	WHO	5738	110491
Zhang, et al	China mainland	2021	2018	cross-sectional	school	WHO	151	2642
Zhang, et al	China mainland	2021	2017-2019	cross-sectional	database	National Reference	31691	201098
Yuan, et al	China mainland	2021	2016	cross-sectional	school	National Reference	96	768
You, et al	China mainland	2021	2017	cross-sectional	school	National Reference	398	3504

© 2024 Zhang X et al. *JAMA Pediatrics*.

Xu, et al (1)	China mainland	2021	2014	cross-sectional	school	WHO	186	1200
Wang, et al	China mainland	2021	2016-2018	cross-sectional	school	WHO	2958	21571
Wang, et al	China mainland	2021	2014-2016	cross-sectional	database	WHO	1867	8365
Sun, et al	China mainland	2021	2017	longitudinal	database	National Reference	570	1973
Min, et al	China mainland	2021	2018	cross-sectional	school	WHO	97	3373
Liu, et al	China mainland	2021	2021	cross-sectional	school	National Reference	1910	10855
Zheng, et al	China mainland	2020	2014-2015	cross-sectional	school	WHO	741	5295
Xu, et al	China mainland	2020	2020	cross-sectional	school	WHO	3428	22681
Shan, et al	China mainland	2010	2004	cross-sectional	school	WHO	2803	21198
Zhang, et al	China mainland	2014	2014	cross-sectional	school	National Reference	212	1488
Abdumijit, et al	China mainland	2022	2021	cross-sectional	community	National Reference	788	4970
Cheng, et al	China mainland	2019	2016-2017	cross-sectional	school	National Reference	141874	1196004
Guo, et al	China mainland	2020	2013-2016	cross-sectional	database	National Reference	5238	40607
Li, et al	China mainland	2014	2009-2010	cross-sectional	school	WHO	76	497
Liu, et al	China mainland	2019	2013-2014	RCT	school	National Reference	294	1889
Jiang, et al	China mainland	2007	2007	RCT	school	IOTF	313	2425
Li, et al	China mainland	2010	2005-2006	RCT	school	WHO	830	4700
Ji, et al	China mainland	2018	2017	cross-sectional	school	National Reference	17	112
Cui, et al	China mainland	2010	2006	longitudinal	database	IOTF	97	1174
Li, et al	China mainland	2008	2002	cross-sectional	database	IOTF	1975	44880
Zhang, et al	China mainland	2012	2003	cross-sectional	community	IOTF	9629	70431
Ma, et al	China mainland	2014	2011	cross-sectional	database	National Reference	3633	36328
Zhang, et al	China mainland	2013	2010	cross-sectional	school	IOTF	5671	42275
Andegiorgish, et al	China mainland	2012	2010	cross-sectional	school	National Reference	394	3140
Yao, et al	China mainland	2014	2009-2013	cross-sectional	school	IOTF	9665	67956
Li, et al	China mainland	2013	2009-2011	cross-sectional	medical institution	National Reference	15038	38539
Zhang, et al	China mainland	2013	2013	cohort	community	National Reference	158	1098
Dong, et al	China mainland	2012	2006-2008	cross-sectional	school	CDC	3704	30056
Zhou, et al	China mainland	2021	2011-2016	cross-sectional	medical institution	CDC	1258	8441
Zhao, et al	China mainland	2017	2015	cross-sectional	school	National Reference	246	1626
Ma, et al	China mainland	2011	2008-2009	cross-sectional	database	WHO	946	8653
Cao, et al	China mainland	2012	2009	cross-sectional	school	National Reference	10296	88974
He, et al	China mainland	2009	2006	cross-sectional	school	National Reference	183	2179
Zhang, et al	China mainland	2012	2005	cross-sectional	database	IOTF	1093	8568
He, et al	China mainland	2017	2017	cross-sectional	school	National Reference	82	848
Zou, et al	China mainland	2022	2016-2017	cross-sectional	database	National Reference	304	2818
Zhang, et al	China	2022	2019	cross-sectional	database	WHO	2752	14534

© 2024 Zhang X et al. *JAMA Pediatrics.*

	mainland							
Zhang, et al	China mainland	2022	2021	cross-sectional	school	National Reference	631	4412
Zeng, et al	China mainland	2022	2012-2013	cohort	database	IOTF	37	430
Yang, et al	China mainland	2022	2019	longitudinal	database	WHO	1342	6047
Xu, et al	China mainland	2022	2009	longitudinal	school	National Reference	519	4538
Wang, et al	China mainland	2022	2018	cross-sectional	school	National Reference	1001	10536
Wang, et al	China mainland	2022	2020	cross-sectional	school	National Reference	1333	9501
Xiong, et al	China mainland	2010	2003-2004	cross-sectional	school	IOTF	1243	7326
Tan, et al	China mainland	2018	2018	cross-sectional	school	IOTF	767	8999
Wang, et al	China mainland	2018	2017	cross-sectional	school	National Reference	1442	18403
Zhang, et al (1)	China mainland	2018	2010	cross-sectional	community	IOTF	8450	68020
Zhang, et al (2)	China mainland	2018	2011	cross-sectional	community	IOTF	6885	43928
Zhang, et al (3)	China mainland	2018	2012	cross-sectional	community	IOTF	17546	105153
Zhang, et al (4)	China mainland	2018	2013	cross-sectional	community	IOTF	22617	129830
Zhang, et al (5)	China mainland	2018	2014	cross-sectional	community	IOTF	20952	119692
Zhang, et al (6)	China mainland	2018	2015	cross-sectional	community	IOTF	19598	107587
Zhang, et al	China mainland	2018	2015	cross-sectional	database	IOTF	212	1617
Zhang, et al	China mainland	2018	2014-2017	cross-sectional	school	National Reference	49413	325083
Cai, et al	China mainland	2019	2013	cross-sectional	school	National Reference	6290	47590
Chen ,et al (1)	China mainland	2019	2011	cross-sectional	community	WHO	1566	15757
Chen ,et al (2)	China mainland	2019	2014	cross-sectional	community	WHO	2117	19098
Chen ,et al (3)	China mainland	2019	2017	cross-sectional	community	WHO	2040	21883
Zhang, et al	China mainland	2019	2015	cross-sectional	database	CDC	21765	177419
Zhao, et al	China mainland	2019	2012	cross-sectional	database	National Reference	115	1081
Zou, et al	China mainland	2019	2014	cross-sectional	school	National Reference	472	2639
Duan, et al	China mainland	2020	2016	cross-sectional	medical institution	National Reference	248	1955
Ke, et al	China mainland	2020	2019	cross-sectional	school	IOTF	286	1330
Song, et al	China mainland	2020	2016	cross-sectional	database	WHO	272	4488
Sun, et al	China mainland	2020	2012	cross-sectional	school	WHO	371	2185
Xing, et al	China mainland	2020	2012-2013	cross-sectional	database	WHO	1068	6740
Fan, et al	China mainland	2010	2010	cross-sectional	school	National Reference	372	3544
Wang, et al	China mainland	2009	2009	cross-sectional	community	WHO	1222	8041
Xu, et al	China mainland	2008	2006	cross-sectional	school	IOTF	385	2020
Shi, et al	China mainland	2007	2002	cross-sectional	school	WHO	72	824
Liu, et al	China mainland	2007	2000	cross-sectional	community	IOTF	16738	263168
Li, et al	China mainland	2006	2004	cross-sectional	school	IOTF	228	1792
Jiang, et al	China mainland	2006	2006	cross-sectional	school	IOTF	100	930

© 2024 Zhang X et al. *JAMA Pediatrics.*

Zhu, et al	China mainland	2015	2013	cross-sectional	school	IOTF	443	4788
Yuan, et al	China mainland	2015	2010	cross-sectional	school	National Reference	1928	16580
Xu, et al	China mainland	2015	2007-2011	cross-sectional	community	National Reference	2757	29997
Xiao, et al	China mainland	2015	2006-2014	cross-sectional	school	WHO	8269	145078
Wu, et al	China mainland	2015	2011-2012	cross-sectional	school	IOTF	10666	55536
Piernas, et al (1)	China mainland	2015	2009	cross-sectional	database	IOTF	116	1191
Piernas, et al (2)	China mainland	2015	2011	cross-sectional	database	IOTF	188	1648
Ma, et al (1)	China mainland	2015	2007	cross-sectional	school	National Reference	372	3832
Ma, et al (2)	China mainland	2015	2008	cross-sectional	school	National Reference	1235	13141
Ma, et al (3)	China mainland	2015	2009	cross-sectional	school	National Reference	1419	14052
Ma, et al (4)	China mainland	2015	2010	cross-sectional	school	National Reference	1416	13750
Wang, et al	China mainland	2014	2009	cross-sectional	school	CDC	3704	30056
He, et al	China mainland	2014	2014	cross-sectional	school	IOTF	9639	67956
Dong, et al	China mainland	2014	2011	cross-sectional	community	National Reference	539	4898
Dai, et al	China mainland	2014	2009-2010	cross-sectional	medical institution	National Reference	3950	18707
Chen, et al (2)	China mainland	2014	2010-2011	cross-sectional	community	IOTF	189	1951
Chen, et al	China mainland	2011	2005	cross-sectional	database	National Reference	18275	231326
Xu, et al	China mainland	2011	2011	cross-sectional	school	National Reference	1019	8898
Guo, et al	China mainland	2012	2010-2011	cross-sectional	community	IOTF	651	4262
Jia, et al	China mainland	2012	2010	cross-sectional	school	National Reference	112	702
Li, et al	China mainland	2012	2011	cross-sectional	school	WHO	1044	7194
Dong, et al	China mainland	2013	2010	cross-sectional	database	National Reference	19211	197191
Chen, et al (1)	China mainland	2016	2009	cross-sectional	medical institution	WHO	284	22576
Chen, et al (2)	China mainland	2016	2012	cross-sectional	medical institution	WHO	754	24816
Chen, et al (3)	China mainland	2016	2015	cross-sectional	medical institution	WHO	1503	23837
Lei, et al	China mainland	2016	2013	cross-sectional	database	National Reference	1187	3327
Liu, et al	China mainland	2016	2014	cross-sectional	school	WHO	447	9917
Peng, et al	China mainland	2016	2016	cross-sectional	school	National Reference	1656	12297
Wei, et al	China mainland	2016	2016	cross-sectional	community	National Reference	195	1928
Zhang, et al	China mainland	2016	2009-2010	cross-sectional	school	WHO	211	1410
Cai, et al	China mainland	2017	2016	cross-sectional	database	National Reference	16421	116615
Liu, et al	China mainland	2017	2015	cross-sectional	database	WHO	7481	45608
Liu, et al	China mainland	2017	2016	cross-sectional	school	WHO	537	4926
Zong, et al	China mainland	2017	2006	cross-sectional	school	National Reference	24653	290043
Gong, et al	China mainland	2018	2016-2018	cross-sectional	database	IOTF	449	2795
He, et al	China mainland	2018	2013-2014	prospective	school	National Reference	230	2032
Li, et al	China	2018	2014	prospective	school	National	128	1237

© 2024 Zhang X et al. *JAMA Pediatrics*.

	mainland					Reference		
Contreras, et al	Colombia	2015	2013	cross-sectional	school	WHO	127	603
Rincón-Pabón, et al	Colombia	2019	2010	cross-sectional	database	WHO	2358	18177
Ramírez-Vélez, et al	Colombia	2017	2014-2015	cross-sectional	database	IOTF	459	2510
Martinez-Ospina, et al	Colombia	2019	2015	cross-sectional	school	WHO	127	715
McDonald, et al	Colombia	2009	2006	cross-sectional	school	IOTF	287	3075
Gebremedhin, et al (4)	Comoros	2015	2012	cross-sectional	database	WHO	266	2699
Gebremedhin, et al (5)	Congo	2015	2011/2012	cross-sectional	database	WHO	159	4531
Gebremedhin, et al (6)	Congo	2015	2013/2014	cross-sectional	database	WHO	319	8391
Gamboa-Gamboa, et al	Costa Rica	2021	2016	cross-sectional	database	WHO	68941	347366
Gebremedhin, et al (11)	Côte d'Ivoire	2015	2011/2012	cross-sectional	database	WHO	96	3294
Fossou, et al	Côte d'Ivoire	2020	2018	cross-sectional	school	WHO	97	1251
Salas, et al (3)	Croatia	2021	2015-2017	cross-sectional	database	WHO	512	2601
Banjari, et al (2)	Croatia	2020	2020	cross-sectional	school	IOTF	53	478
Juresa, et al	Croatia	2012	2003-2004	cross-sectional	school	IOTF	127	960
Janssen, et al (4)	Croatia	2005	2001-2002	cross-sectional	database	IOTF	439	4145
Milanović, et al	Croatia	2020	2015	cross-sectional	school	WHO	1191	5591
Haug, et al (10)	Croatia	2009	2005-2006	cross-sectional	database	IOTF	595	4720
Bili}-Kirin, et al	Croatia	2014	2014	cross-sectional	school	National Reference	24	372
Pećin, et al	Croatia	2013	2013	cross-sectional	school	CDC	19	756
Ahrens, et al (3)	Cyprus	2014	2007-2008	cross-sectional	database	IOTF	442	2942
Savva, et al	Cyprus	2005	2004	cross-sectional	school	IOTF	199	1413
Savva, et al	Cyprus	2008	2002-2003	cross-sectional	school	IOTF	1525	7060
Savva, et al	Cyprus	2014	2009-2010	cross-sectional	school	IOTF	624	3090
Lazarou, et al	Cyprus	2008	2008	cross-sectional	school	IOTF	153	823
Loucaides, et al	Cyprus	2008	2007	cross-sectional	school	IOTF	73	247
Tornaritis, et al	Cyprus	2014	2009-2010	cross-sectional	school	IOTF	210	1414
Savva, et al	Cyprus	2014	2001-2003	longitudinal	school	IOTF	933	4878
Vážná, et al	Czech	2022	2021	cross-sectional	school	WHO	654	3517
Salas, et al (4)	Czech	2021	2015-2017	cross-sectional	database	WHO	194	1395
Gorog, et al (2)	Czech	2011	2011	cross-sectional	database	IOTF	215	1508
Gouw, et al	Czech	2010	2001	cross-sectional	database	IOTF	3356	30966
Janssen, et al (5)	Czech	2005	2001-2002	cross-sectional	database	IOTF	454	4990
Haug, et al (20)	Czech	2009	2005-2006	cross-sectional	database	IOTF	592	4734
Wijnhoven, et al (2)	Czech	2015	2007/2008	cross-sectional	school	WHO	341	1633
Brixval, et al	Denmark	2012	2002	cross-sectional	database	IOTF	409	4208
Salas, et al (5)	Denmark	2021	2015-2017	cross-sectional	database	WHO	99	935
Yngve, et al (3)	Denmark	2008	2003	cross-sectional	database	IOTF	111	1066
Ajslev, et al	Denmark	2011	2004-2009	cohort	database	IOTF	2333	28354
Mor, et al	Denmark	2015	2002-2013	cohort	database	IOTF	768	9886
Høyer, et al (1)	Denmark	2014	2002-2004	cohort	database	WHO	93	525
Janssen, et al (6)	Denmark	2005	2001-2002	cross-sectional	database	IOTF	405	4009

© 2024 Zhang X et al. *JAMA Pediatrics.*

Krue, et al	Denmark	2010	2010	cross-sectional	school	National Reference	1217	8694
Haug, et al (27)	Denmark	2009	2005-2006	cross-sectional	database	IOTF	395	4708
Rex, et al	Denmark	2014	2011-2013	cross-sectional	medical institution	IOTF	96	607
Matthiessen, et al	Denmark	2014	2005-2008	cross-sectional	database	IOTF	70	512
Kjelgaard, et al	Denmark	2017	2010	cross-sectional	database	IOTF	318	4922
Manyanga, et al (2)	Djibouti	2014	2014	cross-sectional	database	WHO	233	1711
Romano, et al (7)	Dominican Republic	2022	2016	cross-sectional	database	WHO	192	954
Romano, et al (8)	East Timor	2022	2015	cross-sectional	database	WHO	77	1631
Freire, et al	Ecuador	2014	2012	cross-sectional	database	WHO	2188	11534
Ortiz, et al	Ecuador	2014	2008	cross-sectional	community	WHO	79	703
Casaoulla, et al	Ecuador	2017	2015	cross-sectional	school	IOTF	42	427
Abd El-Aty, et al	Egypt	2020	2016-2017	cross-sectional	community	CDC	174	5458
Abdelkarim, et al	Egypt	2017	2014-2015	cross-sectional	community	IOTF	76	676
Abou-Khadra, et al	Egypt	2022	2018-2019	cross-sectional	school	WHO	54	319
El-Sabely, et al	Egypt	2013	2013	cross-sectional	school	WHO	60	288
Badawi, et al	Egypt	2013	2011	cross-sectional	school	National Reference	151	852
Manyanga, et al (3)	Egypt	2014	2014	cross-sectional	database	WHO	1144	5179
Hassan, et al	Egypt	2008	2002-2004	cross-sectional	school	CDC	154	1283
Hassan, et al	Egypt	2016	2013-2016	cross-sectional	medical institution	National Reference	17	154
Talat, et al	Egypt	2016	2014-2015	cross-sectional	school	National Reference	180	900
Pérez, et al	El Salvador	2020	2015-2016	cross-sectional	school	IOTF	15881	111991
Ahrens, et al (2)	Estonia	2014	2007-2008	cross-sectional	database	IOTF	217	2067
Janssen, et al (8)	Estonia	2005	2001-2002	cross-sectional	database	IOTF	244	3752
Haug, et al (29)	Estonia	2009	2005-2006	cross-sectional	database	IOTF	337	4215
Weinmayr, et al (2)	Estonia	2014	2004	cross-sectional	database	IOTF	37	241
Worku, et al	Ethiopia	2021	2018	cross-sectional	school	WHO	56	522
Abich, et al	Ethiopia	2020	2018-2019	cross-sectional	school	CDC	93	823
Tadesse, et al	Ethiopia	2017	2015	cross-sectional	school	WHO	19	462
Sorrie, et al	Ethiopia	2017	2016	cross-sectional	community	WHO	48	500
Desalew, et al	Ethiopia	2017	2016	cross-sectional	school	CDC	66	448
Gebremedhin, et al (7)	Ethiopia	2015	2011	cross-sectional	database	WHO	168	9880
Gali, et al	Ethiopia	2017	2015	cross-sectional	school	WHO	60	510
Askal, et al	Ethiopia	2015	2014	cross-sectional	school	CDC	66	828
Carrillo-Larco, et al (4)	Ethiopia	2014	2014	cohort	database	IOTF	9	1882
Mitiku, et al	Ethiopia	2019	2014	cross-sectional	school	WHO	67	1523
Mekonnen, et al	Ethiopia	2018	2016	cross-sectional	school	CDC	56	634
Romano, et al (9)	Fiji	2022	2016	cross-sectional	database	WHO	298	1537
Pengpid, et al (1)	Fiji	2015	2010-2011	cross-sectional	database	IOTF	246	1773
Petersen, et al	Fiji	2014	2005-2008	cross-sectional	database	IOTF	1494	8947
Sarkkola, et al	Finland	2022	2011-2014	cross-sectional	database	IOTF	1352	10646
Eloranta, et	Finland	2012	2007-2009	cross-sectional	database	IOTF	44	510

© 2024 Zhang X et al. *JAMA Pediatrics.*

al								
Janssen, et al (9)	Finland	2005	2001-2002	cross-sectional	database	IOTF	614	5205
Veltsista, et al (1)	Finland	2010	2001	cross-sectional	school	IOTF	686	6468
Vanhala, et al	Finland	2009	2004	cross-sectional	school	IOTF	91	749
Haug, et al (30)	Finland	2009	2005-2006	cross-sectional	database	IOTF	656	4934
Fogelholm, et al	Finland	2008	2003	cross-sectional	school	IOTF	279	2266
Vanhelst, et al	France	2022	2008-2018	cross-sectional	database	IOTF	11114	90250
Roth, et al	France	2022	2017-2020	cross-sectional	school	IOTF	1708	19295
Luiggi, et al	France	2021	2019	cross-sectional	school	CDC	92	1038
Thibault, et al	France	2013	2007-2009	cross-sectional	school	IOTF	753	7667
Thibault, et al	France	2010	2004-2005	cross-sectional	school	IOTF	279	2385
Carriere, et al	France	2015	2004	cross-sectional	school	IOTF	226	1836
Salanave, et al	France	2009	2007	cross-sectional	school	IOTF	132	1014
Heude, et al	France	2003	2000	cross-sectional	school	IOTF	67	601
Legleye, et al	France	2014	2010	cross-sectional	database	IOTF	47	303
Chau, et al	France	2013	2010	cross-sectional	school	National Reference	397	1270
Jouret, et al	France	2007	2007	cross-sectional	school	IOTF	123	1780
Pitrou, et al	France	2010	2004-2005	cross-sectional	school	IOTF	144	1030
Janssen, et al (10)	France	2005	2001-2002	cross-sectional	database	IOTF	762	7624
Kleiser, et al	France	2003	2001	cross-sectional	school	IOTF	982	4326
Rolland-Cachera, et al	France	2002	2000	cross-sectional	school	IOTF	226	1582
Tubert-Jeannin, et al	France	2018	2011-2012	cross-sectional	school	WHO	94	2676
Haug, et al (5)	France	2009	2005-2006	cross-sectional	database	IOTF	579	6583
Kêkê, et al	France	2015	2009	cross-sectional	school	IOTF	224	1382
Vanhelst, et al (1)	France	2017	2009	cross-sectional	database	IOTF	280	1848
Vanhelst, et al (2)	France	2017	2010	cross-sectional	database	IOTF	298	1818
Vanhelst, et al (3)	France	2017	2011	cross-sectional	database	IOTF	409	2578
Vanhelst, et al (4)	France	2017	2012	cross-sectional	database	IOTF	347	2165
Vanhelst, et al (5)	France	2017	2013	cross-sectional	database	IOTF	185	1260
Romano, et al (10)	French Polynesis	2022	2015	cross-sectional	database	WHO	455	1902
Gebremedhin, et al (8)	Gabon	2015	2012	cross-sectional	database	WHO	209	3482
Gebremedhin, et al (9)	Gambia	2015	2013	cross-sectional	database	WHO	67	3360
Salas, et al (6)	Georgia	2021	2015-2017	cross-sectional	database	WHO	477	3057
Weinmayr, et al (3)	Georgia	2014	2004	cross-sectional	database	IOTF	31	169
Ahrens, et al (6)	Germany	2014	2007-2008	cross-sectional	database	IOTF	258	2194
Hoebel, et al	Germany	2022	2014-2017	cross-sectional	database	National Reference	549	3567
Zhou, et al	Germany	2021	2010-2014	cross-sectional	school	National Reference	1270	22678
Usheva, et al (3)	Germany	2021	2012	cross-sectional	database	WHO	61	1104
Sergentanis, et al (3)	Germany	2021	2011-2012	cross-sectional	database	IOTF	209	1621
Liu, et al	Germany	2021	2013-2018	cross-sectional	school	WHO	4595	33407

© 2024 Zhang X et al. *JAMA Pediatrics.*

Willerhausen, et al	Germany	2007	2007	cross-sectional	medical institution	National Reference	153	1290
Kesztyüs, et al	Germany	2013	2009	cross-sectional	database	National Reference	55	1730
Negal, et al	Germany	2009	2006	RCT	school	IOTF	179	1063
Boneberger, et al	Germany	2009	2005-2006	cross-sectional	school	IOTF	649	4718
Kromeyer-Hauschild, et al	Germany	2007	2001	cross-sectional	school	IOTF	217	1915
Landsberg, et al	Germany	2010	2000-2005	cross-sectional	database	IOTF	275	1894
Toschke, et al	Germany	2005	2001-2002	cross-sectional	medical institution	IOTF	450	4370
Toschke, et al	Germany	2003	2000-2001	cross-sectional	school	IOTF	492	4706
Kries, et al	Germany	2008	2005	cross-sectional	school	IOTF	808	5899
Raum, et al	Germany	2011	2001-2002	cross-sectional	medical institution	National Reference	175	1979
Kleiser, et al	Germany	2009	2003-2006	cross-sectional	database	IOTF	2035	13395
Toschke, et al	Germany	2007	2001-2002	cross-sectional	school	IOTF	616	5472
Weyermann, et al	Germany	2006	2000-2001	cohort	medical institution	National Reference	24	855
Will, et al	Germany	2005	2002	cross-sectional	school	IOTF	49	523
Rapp, et al	Germany	2005	2002	longitudinal	school	IOTF	339	2140
Janssen, et al (11)	Germany	2005	2001-2002	cross-sectional	database	IOTF	473	4878
Graf, et al	Germany	2004	2001	cross-sectional	school	National Reference	44	668
Graf, et al	Germany	2004	2002-2003	cross-sectional	school	National Reference	19	344
Reich, et al	Germany	2003	2003	cross-sectional	school	National Reference	308	2354
Sporišević, et al	Germany	2009	2007-2008	cross-sectional	community	CDC	23	214
Haug, et al (6)	Germany	2009	2005-2006	cross-sectional	database	IOTF	728	6619
De Toia , et al	Germany	2009	2006-2008	cross-sectional	school	National Reference	118	1228
Bayer, et al	Germany	2009	2004-2006	cross-sectional	database	IOTF	1744	12199
Nagel, et al	Germany	2008	2004-2005	cross-sectional	school	IOTF	97	557
Jahnke, et al	Germany	2008	2008	cross-sectional	medical institution	IOTF	18	142
Herpertz-Dahlmann, et al	Germany	2008	2003-2006	cross-sectional	community	National Reference	195	1843
Willershausen, et al	Germany	2007	2007	cross-sectional	school	National Reference	218	2071
Kobel, et al	Germany	2015	2015	cross-sectional	school	National Reference	16	294
Brettschneider, et al	Germany	2015	2009-2012	cross-sectional	database	National Reference	292	4948
Weinmayr, et al (4)	Germany	2014	2004	cross-sectional	database	IOTF	268	1580
Aboagye, et al	Ghana	2022	2022	cross-sectional	school	National Reference	47	423
Adom, et al	Ghana	2019	2019	cross-sectional	school	WHO	50	543
Amidu, et al	Ghana	2013	2012-2013	cross-sectional	school	CDC	39	400
Hohammed, et al	Ghana	2012	2012	cross-sectional	school	WHO	43	270
Intiful, et al	Ghana	2013	2013	cross-sectional	school	WHO	15	124
Manyanga, et al (4)	Ghana	2014	2014	cross-sectional	database	WHO	473	6155
Obiricorang, et al	Ghana	2015	2013-2014	cross-sectional	school	National Reference	35	303
Aryeetey, et al	Ghana	2017	2009-2012	cross-sectional	school	WHO	382	3089
Atsu, et al	Ghana	2017	2011	cross-sectional	database	WHO	130	7328
Annan-Asare, et al	Ghana	2017	2017	cross-sectional	school	National Reference	78	260
Gyamfi, et al	Ghana	2019	2019	cross-sectional	school	WHO	135	1004
Ganle, et al	Ghana	2019	2019	cross-sectional	school	CDC	68	285

© 2024 Zhang X et al. *JAMA Pediatrics.*

Weinmayr, et al (5)	Ghana	2014	2004	cross-sectional	database	IOTF	1	241
Makri, et al	Greece	2022	2018	cross-sectional	database	WHO	740	3816
Usheva, et al (4)	Greece	2021	2012	cross-sectional	database	WHO	200	1647
Sergentanis, et al (7)	Greece	2021	2011-2012	cross-sectional	database	IOTF	273	1516
Kostopoulou, et al	Greece	2021	2018-2019	cross-sectional	database	CDC	673	3504
Pikramenou, et al	Greece	2016	2013	cross-sectional	school	IOTF	278	2180
Trikaliotis, et al	Greece	2011	2011	cross-sectional	school	IOTF	26	361
Grigorakis, et al	Greece	2016	2010-2011	cross-sectional	school	IOTF	30780	124113
Kleanthous, et al	Greece	2016	2009	cross-sectional	school	IOTF	368	1327
Manios, et al	Greece	2013	2007	cross-sectional	database	IOTF	770	2492
Manios, et al	Greece	2011	2005-2006	cross-sectional	school	IOTF	135	481
Tokmakidis, et al	Greece	2006	2006	cross-sectional	school	IOTF	183	709
Lamprokostopoulou, et al	Greece	2019	2007-2010	cross-sectional	school	IOTF	124	919
Kontogianni, et al	Greece	2010	2007	cross-sectional	community	IOTF	212	1170
Kosti, et al	Greece	2007	2004-2005	cross-sectional	database	IOTF	326	2008
Cassimos, et al	Greece	2011	2008-2009	cross-sectional	school	IOTF	113	335
Krassas, et al (1)	Greece	2004	2004	cross-sectional	school	National Reference	546	2468
Angelopoulos, et al	Greece	2006	2003-2004	cross-sectional	school	IOTF	107	312
Magkos, et al	Greece	2006	2002	cross-sectional	community	IOTF	166	620
Magkos, et al	Greece	2006	2006	cross-sectional	school	IOTF	60	198
Papadimitriou, et al	Greece	2006	2003-2004	cross-sectional	school	IOTF	1121	4131
Manios, et al	Greece	2007	2003-2004	cross-sectional	database	IOTF	336	2374
Kamtsios, et al	Greece	2008	2008	cross-sectional	school	IOTF	183	775
Linardakis, et al	Greece	2008	2004-2005	cross-sectional	school	IOTF	160	856
Hassapidou, et al	Greece	2009	2006	cross-sectional	school	IOTF	64	266
Kollias, et al	Greece	2009	2009	cross-sectional	school	IOTF	172	797
Koroni, et al	Greece	2009	2009	cross-sectional	school	IOTF	421	1861
Yannakoulia, et al	Greece	2010	2010	cross-sectional	database	IOTF	327	1132
Hassapidou, et al	Greece	2015	2009-2010	cross-sectional	school	IOTF	155	1006
Spathopoulos, et al	Greece	2009	2005-2006	cross-sectional	school	National Reference	403	2715
Antonogeorgos, et al	Greece	2012	2005-2006	cross-sectional	database	IOTF	150	700
Janssen, et al (12)	Greece	2005	2001-2002	cross-sectional	database	IOTF	530	3584
Manios, et al	Greece	2004	2004	cross-sectional	school	IOTF	59	195
Veltsista, et al (2)	Greece	2010	2001	cross-sectional	school	IOTF	369	2842
Poulimeneas, et al	Greece	2019	2019	cross-sectional	community	IOTF	45	172
Vazquez, et al	Greece	2019	2012-2013	cross-sectional	database	CDC	327	2675
Katsagoni, et al	Greece	2020	2014-2015	cross-sectional	database	WHO	39913	174209
Notara,et al	Greece	2020	2014-2016	cross-sectional	school	IOTF	375	1659
Mirkopoulou, et al	Greece	2010	2005	cross-sectional	school	IOTF	29	98
Karatzi , et al	Greece	2009	2004-2005	prospective	school	IOTF	74	754
Haug, et al	Greece	2009	2005-2006	cross-sectional	database	IOTF	563	3566

© 2024 Zhang X et al. *JAMA Pediatrics*.

(11)								
Vafeiadi, et al	Greece	2015	2007	prospective	community	IOTF	99	689
Sourani, et al	Greece	2015	2011	cross-sectional	community	IOTF	88	352
Patsopoulou, et al	Greece	2015	2015	cross-sectional	school	IOTF	96	451
Kambas, et al	Greece	2015	2011	cross-sectional	database	IOTF	19	250
Weinmayr, et al (6)	Greece	2014	2004	cross-sectional	database	IOTF	127	404
Grammatikopoulou, et al	Greece	2014	2010-2012	cross-sectional	school	IOTF	8831	37344
Antonogeorgos, et al	Greece	2011	2006	cross-sectional	database	National Reference	150	700
Athanasopoulos, et al	Greece	2011	2010	cross-sectional	school	IOTF	48	232
Farajian, et al	Greece	2011	2009	cross-sectional	school	IOTF	1412	4786
Michalopoulou, et al	Greece	2011	2009	cross-sectional	school	IOTF	128	532
Jelastopulu, et al	Greece	2012	2009	cross-sectional	school	IOTF	64	200
Kyriazis, et al	Greece	2012	2009-2010	cross-sectional	school	IOTF	567	2374
Tambalis, et al	Greece	2013	2013	cross-sectional	school	IOTF	890	3195
Poulimeneas, et al	Greece	2016	2016	cross-sectional	school	IOTF	1314	4833
Garoufi, et al	Greece	2017	2009-2010	cross-sectional	school	National Reference	234	736
Velde, et al (2)	Greece	2017	2010	cross-sectional	database	IOTF	328	1085
Koulouvaris, et al	Greece	2018	2018	cross-sectional	community	WHO	114	463
Manios, et al	Greece	2018	2018	cross-sectional	school	IOTF	695	2263
Janssen, et al (13)	Greenland	2005	2001-2002	cross-sectional	database	IOTF	107	558
Haug, et al (31)	Greenland	2009	2005-2006	cross-sectional	database	IOTF	151	943
Alvarado, et al	Guatemala	2009	2004-2005	cross-sectional	school	CDC	68	363
Gebremedhin, et al (10)	Guinea	2015	2012	cross-sectional	database	WHO	103	3216
Torres, et al	Honduras	2014	2011	cross-sectional	school	WHO	266	2554
Tung, et al	Hong Kong	2021	2014	cohort	database	IOTF	2214	18863
Wing, et al	Hong Kong	2009	2003	cross-sectional	school	CDC	511	5159
Wang, et al	Hong Kong	2019	2008-2009	cohort	database	IOTF	645	3614
Chan, et al	Hong Kong	2013	2007-2008	cross-sectional	school	National Reference	212	1614
Wang, et al	Hong Kong	2017	2015	cross-sectional	school	IOTF	137	894
Wong, et al	Hong Kong	2005	2002	cross-sectional	school	IOTF	1768	10773
Ko, et al	Hong Kong	2008	2003-2004	cross-sectional	school	IOTF	208	2098
Knowles, et al	Hong Kong	2015	2015	cross-sectional	school	IOTF	108	620
Lee, et al	Hong Kong	2017	2012-2013	cross-sectional	database	IOTF	919	4410
Brug, et al (2)	Hungary	2012	2012	cross-sectional	database	IOTF	200	1020
Ahrens, et al (7)	Hungary	2014	2007-2008	cross-sectional	database	IOTF	349	3159
Baráth, et al	Hungary	2010	2005-2006	cross-sectional	school	IOTF	2401	14290
Bjørnarå, et al	Hungary	2014	2010	cross-sectional	database	National Reference	242	929
Gorog, et al (3)	Hungary	2011	2011	cross-sectional	database	IOTF	215	1661
Janssen, et al (14)	Hungary	2005	2001-2002	cross-sectional	database	IOTF	408	3845
Haug, et al (21)	Hungary	2009	2005-2006	cross-sectional	database	IOTF	395	3214
Antal, et al	Hungary	2009	2005-2006	cross-sectional	school	IOTF	362	1928
Velde, et al (3)	Hungary	2017	2010	cross-sectional	database	IOTF	200	1022
Erdei, et al	Hungary	2018	2016	cross-sectional	school	IOTF	679	5332

© 2024 Zhang X et al. *JAMA Pediatrics.*

Jakab, et al	Hungary	2018	2015-2017	cross-sectional	school	IOTF	448	6824
Aanesen, et al	Iceland	2020	2016-2017	cross-sectional	community	IOTF	583	4360
Sergentanis, et al (1)	Iceland	2021	2011-2012	cross-sectional	database	IOTF	127	1196
Yngve, et al (4)	Iceland	2008	2003	cross-sectional	database	IOTF	104	714
Haug, et al (32)	Iceland	2009	2005-2006	cross-sectional	database	IOTF	938	8014
Dabas, et al (1)	India	2022	2008	cross-sectional	school	IOTF	473	3172
Dabas, et al (2)	India	2022	2013	cross-sectional	school	IOTF	721	3007
Dabas, et al (3)	India	2022	2015	cross-sectional	school	IOTF	476	2238
Arushi, et al	India	2022	2020-2021	cross-sectional	school	WHO	229	1620
Thomas, et al	India	2021	2019-2020	cross-sectional	school	WHO	54	440
Seema, et al	India	2021	2016-2018	cross-sectional	school	WHO	66	385
Moitra, et al	India	2021	2021	cross-sectional	school	WHO	215	772
Elangovan, et al	India	2012	2011	cross-sectional	medical institution	CDC	88	510
Subramaniam, et al	India	2011	2011	cross-sectional	school	CDC	100	2033
Shailee, et al	India	2018	2009	cross-sectional	school	CDC	22	1011
Goyal, et al	India	2011	2009-2010	cross-sectional	school	National Reference	161	1159
Jain, et al	India	2010	2003-2004	cross-sectional	school	National Reference	499	2570
Gautam, et al	India	2019	2012	cross-sectional	school	WHO	128	1185
Bharati, et al	India	2008	2005-2006	cross-sectional	school	National Reference	79	2255
Kotian, et al	India	2010	2007	cross-sectional	school	IOTF	89	900
Warraich, et al	India	2009	2009	cross-sectional	school	National Reference	23	284
Mushtag, et al	India	2011	2011	cross-sectional	school	WHO	316	1860
Tiwari, et al	India	2014	2010-2011	cross-sectional	school	WHO	62	940
Baruah, et al	India	2018	2017	cross-sectional	school	National Reference	19	349
Baston, et al	India	2014	2009-2010	cross-sectional	school	WHO	365	2130
Carrillo-Larco, et al (2)	India	2014	2014	cohort	database	IOTF	25	1929
Khadilkar, et al	India	2011	2007-2008	cross-sectional	community	IOTF	3016	20243
Sultan, et al	India	2008	2008	cross-sectional	medical institution	WHO	44	172
Gupta, et al	India	2011	2008-2009	cross-sectional	database	IOTF	1237	4908
Sharma, et al	India	2007	2007	cross-sectional	school	IOTF	987	4399
Ramachandran, et al	India	2002	2002	cross-sectional	school	IOTF	790	4700
Bose, et al	India	2007	2002	cross-sectional	school	IOTF	76	431
Mehta, et al	India	2007	2002	cross-sectional	school	IOTF	63	414
Laxmaiah, et al	India	2007	2003	cross-sectional	school	IOTF	87	1208
Raj, et al	India	2007	2003-2004	cross-sectional	school	CDC	1227	24842
Iyer, et al	India	2011	2004	cross-sectional	school	IOTF	154	1067
Premanath, et al	India	2010	2005-2006	cross-sectional	school	National Reference	3682	43152
Marwaha, et al	India	2006	2006	cross-sectional	school	IOTF	2483	21485
Kumar, et al	India	2008	2007	cross-sectional	school	National Reference	19	425
Sood, et al	India	2007	2007	cross-sectional	school	CDC	520	3970
Goyal, et al	India	2010	2010	cross-sectional	school	IOTF	688	5664
Ghosh, et al	India	2011	2011	cross-sectional	school	National Reference	86	753
Mahajan, et al	India	2011	2008-2009	cross-sectional	school	CDC	112	2542

© 2024 Zhang X et al. *JAMA Pediatrics.*

Cherian, et al	India	2012	2009-2010	cross-sectional	school	CDC	181	1634
Thakre, et al	India	2011	2009-2011	cross-sectional	school	CDC	137	1524
Keerthan, et al	India	2011	2011	cross-sectional	school	National Reference	13	500
Vohra, et al	India	2011	2011	cross-sectional	school	CDC	17	407
Chakraborty, et al	India	2011	2008-2009	cross-sectional	school	CDC	22	979
Sidhu, et al	India	2005	2005	cross-sectional	school	National Reference	70	640
Sidhu, et al	India	2006	2006	cross-sectional	school	IOTF	133	1000
Misra, et al	India	2011	2006-2008	cross-sectional	school	IOTF	4442	38296
Jagadesan, et al	India	2014	2014	cross-sectional	school	IOTF	2577	18955
Kaur, et al	India	2008	2008	cross-sectional	community	IOTF	1451	15996
Saikia, et al	India	2018	2015-2016	cross-sectional	school	WHO	229	1096
Chandra, et al	India	2019	2018	cross-sectional	school	National Reference	195	544
Minhas, et al	India	2019	2014-2015	cross-sectional	school	CDC	267	1550
Mohan, et al	India	2019	2016-2017	cross-sectional	school	National Reference	187	1959
Sharma, et al	India	2019	2019	cross-sectional	community	CDC	84	1000
Singh, et al	India	2020	2020	cross-sectional	school	National Reference	102	1030
Singh, et al	India	2020	2016	cross-sectional	school	WHO	224	1237
Solanki, et al	India	2020	2011-2013	cross-sectional	school	CDC	930	10037
Ramesh, et al	India	2010	2008-2009	cross-sectional	school	CDC	206	1718
Sharma, et al	India	2009	2009	cross-sectional	medical institution	CDC	111	500
Singh, et al	India	2007	2007	cross-sectional	school	National Reference	43	1083
Senbanjo, et al	India	2007	2007	cross-sectional	community	WHO	23	270
Kasi, et al	India	2015	2008-2009	cross-sectional	school	IOTF	139	1229
Joseph, et al	India	2015	2013	cross-sectional	school	National Reference	41	300
Basha, et al	India	2015	2015	cross-sectional	school	National Reference	262	1450
Weinmayr, et al (7)	India	2014	2004	cross-sectional	database	IOTF	7	119
Ghosh, et al	India	2014	2008-2011	cross-sectional	school	National Reference	187	1061
Aroor, et al	India	2014	2011	cross-sectional	school	CDC	45	755
Honne, et al	India	2012	2012	cross-sectional	school	IOTF	86	463
Sakeenabi, et al	India	2012	2008	cross-sectional	school	National Reference	280	1550
Maiti, et al	India	2013	2010-2011	cross-sectional	school	IOTF	103	1375
Bhargava, et al	India	2016	2013-2014	cross-sectional	school	WHO	129	1266
Deepika, et al	India	2016	2013	cross-sectional	school	IOTF	165	877
Pawar, et al	India	2016	2014-2015	cross-sectional	school	IOTF	207	1828
Prasad, et al	India	2016	2014	cross-sectional	school	National Reference	240	2465
Saikia, et al	India	2016	2015	cross-sectional	school	WHO	169	752
Bharati, et al	India	2017	2017	cross-sectional	school	CDC	532	5216
Choudhary, et al	India	2017	2016-2017	cross-sectional	school	CDC	56	540
Eshwar, et al	India	2017	2015	cross-sectional	school	IOTF	236	1496
Ganie, et al	India	2017	2011-2013	cross-sectional	school	CDC	95	2024
Haq, et al	India	2017	2015	cross-sectional	medical institution	IOTF	20	303
Kumar, et al	India	2017	2017	cross-sectional	community	National Reference	105	1092
Mishra, et al	India	2017	2012-2013	cross-sectional	school	IOTF	19	300
Windiani, et al	Indonesia	2021	2019	cross-sectional	school	National Reference	62	468
Aditya, et al	Indonesia	2017	2015	cross-sectional	school	CDC	44	384

© 2024 Zhang X et al. *JAMA Pediatrics*.

Agustina, et al	Indonesia	2021	2013	cross-sectional	database	WHO	12958	108890
Romano, et al (11)	Indonesia	2022	2015	cross-sectional	database	WHO	969	8806
Febriani, et al	Indonesia	2019	2017	cross-sectional	school	WHO	23	145
Maehara, et al	Indonesia	2019	2017	cross-sectional	community	WHO	176	2160
Rizkiriani, et al	Indonesia	2014	2014	cross-sectional	school	National Reference	36	213
Yulia, et al	Indonesia	2017	2016	cross-sectional	school	WHO	31	95
Moradi, et al	Iran	2016	2015	cross-sectional	school	WHO	604	2506
Soheilipour, et al	Iran	2022	2022	cross-sectional	school	WHO	171	829
Barati, et al	Iran	2022	2022	cross-sectional	school	CDC	185	1091
Mohammadi, et al	Iran	2021	2019	cross-sectional	school	National Reference	111	581
Jari, et al	Iran	2015	2009-2010	cross-sectional	database	WHO	85	1090
Bagherian, et al	Iran	2013	2009	cross-sectional	school	National Reference	53	400
Sadeghi, et al	Iran	2011	2008	cross-sectional	school	CDC	103	747
Shahraki, et al	Iran	2013	2013	cross-sectional	school	National Reference	95	1213
Agha-Alinejad, et al	Iran	2015	2011	cross-sectional	school	CDC	34	381
Javedan, et al	Iran	2016	2015	cross-sectional	school	WHO	3497	12796
Fatemeh, et al	Iran	2012	2008	cross-sectional	school	CDC	53	500
Ahmadi, et al	Iran	2014	2014	cross-sectional	school	CDC	240	1992
Badeli, et al	Iran	2016	2013-2015	cross-sectional	school	National Reference	276	2072
Bahreini, et al	Iran	2013	2010	cross-sectional	school	WHO	169	3002
Bahreynian, et al	Iran	2015	2011-2012	cross-sectional	school	WHO	1292	13322
Basiratnia, et al	Iran	2013	2010-2011	cross-sectional	school	CDC	260	2000
Maddah, et al	Iran	2010	2006-2007	cross-sectional	school	IOTF	871	6635
Kelishadi, et al	Iran	2008	2003-2004	cross-sectional	database	IOTF	2386	21111
Amini, et al	Iran	2007	2000-2001	cross-sectional	school	CDC	64	398
Motlagh, et al	Iran	2011	2008	cross-sectional	database	CDC	110391	862433
Ziaoddini, et al	Iran	2010	2007	cross-sectional	database	CDC	121372	899054
Ayatollahi, et al	Iran	2007	2002-2003	cross-sectional	school	CDC	117	2195
Khodaverdi, et al	Iran	2011	2007	cross-sectional	school	National Reference	33	240
Kajbaf, et al	Iran	2011	2009	cross-sectional	school	IOTF	86	903
Vafa, et al	Iran	2012	2008	cross-sectional	school	WHO	41	511
Zarrati, et al	Iran	2013	2011-2012	cross-sectional	school	WHO	261	1184
Hajian-Tilaki, et al	Iran	2012	2008	cross-sectional	school	CDC	181	1200
Gaeini, et al	Iran	2011	2007-2008	cross-sectional	community	CDC	76	755
Montazerifar, et al	Iran	2009	2005-2006	cross-sectional	school	CDC	52	590
Heidari, et al	Iran	2014	2010	cross-sectional	school	CDC	2222	12946
Hajian-Tilaki, et al	Iran	2011	2006	cross-sectional	school	CDC	123	1000
Gargari, et al	Iran	2004	2001	cross-sectional	school	IOTF	154	1518
Tebesh, et al	Iran	2013	2012-2013	cross-sectional	school	WHO	1246	5811
Shafaghi, et al	Iran	2014	2010-2011	cross-sectional	school	WHO	206	1189
Moayeri, et al	Iran	2006	2004-2005	cross-sectional	school	CDC	499	2880
Behzadnia,	Iran	2012	2009-2010	cross-sectional	school	National	177	653

© 2024 Zhang X et al. *JAMA Pediatrics.*

et al						Reference		
Maddah, et al	Iran	2010	2006-2007	cross-sectional	school	IOTF	480	2577
Saeidlou, et al	Iran	2014	2011	cross-sectional	community	WHO	36	902
Kavehmanesh, et al	Iran	2013	2009-2011	case-control	community	National Reference	72	259
Hajian-Tilaki, et al	Iran	2013	2012	cross-sectional	school	CDC	90	760
Mirhosseini, et al	Iran	2012	2007	cross-sectional	school	WHO	70	477
Heshmat, et al	Iran	2015	2009-2010	cross-sectional	school	CDC	451	5625
Sadeghi, et al	Iran	2007	2007	cross-sectional	school	National Reference	107	633
Zekavat, et al	Iran	2014	2008-2009	cross-sectional	school	CDC	115	1156
Ejtahed, et al	Iran	2018	2015	cross-sectional	school	WHO	1330	14002
Salehi-Abargouei, et al	Iran	2013	2013	cross-sectional	school	WHO	74	837
Maddah, et al	Iran	2009	2006	cross-sectional	school	IOTF	389	2091
Taheri, et al	Iran	2013	2012	cross-sectional	school	CDC	147	1541
Zakeri, et al	Iran	2012	2006-2007	cross-sectional	database	CDC	858	8339
Jari, et al	Iran	2014	2009-2010	cross-sectional	database	WHO	437	5528
Sedaghat, et al	Iran	2019	2014-2015	cross-sectional	school	CDC	32	263
Rafraf, et al	Iran	2010	2008	cross-sectional	school	IOTF	162	985
Maddah, et al	Iran	2010	2006-2007	cross-sectional	school	IOTF	1301	9046
Salem, et al	Iran	2009	2006-2007	cross-sectional	community	CDC	137	1221
Maddah, et al	Iran	2009	2005-2006	cross-sectional	school	IOTF	413	2255
Kelishadi, et al	Iran	2009	2006-2007	cross-sectional	school	CDC	377	1107
Azita, et al	Iran	2009	2006	cross-sectional	school	National Reference	33	954
Bidad, et al	Iran	2008	2004-2005	cross-sectional	school	CDC	50	358
Rashidi, et al	Iran	2007	2000-2001	cross-sectional	school	CDC	348	2321
Mozaffari, et al	Iran	2007	2002	cross-sectional	school	IOTF	239	1800
Maddah, et al	Iran	2007	2005	cross-sectional	school	IOTF	231	1054
Tabriz, et al	Iran	2015	2009-2013	cross-sectional	community	CDC	186	1151
Ghadimi, et al	Iran	2015	2012	cross-sectional	school	CDC	430	3647
Jalali-Farahani, et al	Iran	2014	2014	cross-sectional	school	WHO	122	465
Ahmadi, et al	Iran	2014	2010	cross-sectional	school	National Reference	16	145
Motlagh, et al (1)	Iran	2011	2007	cross-sectional	community	CDC	110391	862433
Motlagh, et al (2)	Iran	2011	2008	cross-sectional	community	CDC	105603	782244
Motlagh, et al (3)	Iran	2011	2009	cross-sectional	community	CDC	104137	955388
Hatami, et al	Iran	2013	2009-2010	cross-sectional	school	WHO	103	739
Keykhaei, et al	Iran	2016	2012	cross-sectional	school	CDC	69	585
Saeidlou, et al	Iran	2016	2009-2011	prospective	school	CDC	5433	51583
Salehiniya, et al	Iran	2016	2012	cross-sectional	database	WHO	561	4656
Darabiyan, et al	Iran	2018	2017	cross-sectional	school	CDC	53	239
Ghobadi, et al	Iran	2018	2015-2016	cross-sectional	school	WHO	55	607
Motlagh, et al	Iran	2018	2015-2016	cross-sectional	community	CDC	370	2444

© 2024 Zhang X et al. *JAMA Pediatrics*.

Parastar, et al	Iran	2018	2016	cross-sectional	community	CDC	149	242
Abood, et al	Iraq	2021	2018-2019	cross-sectional	school	CDC	36	357
Lafta, et al	Iraq	2005	2002	cross-sectional	school	National Reference	388	8300
Lafta, et al	Iraq	2007	2005	cross-sectional	school	IOTF	664	5361
Musaiger, et al (1)	Iraq	2016	2013-2014	cross-sectional	database	IOTF	172	904
Reulbach, et al	Ireland	2013	2007-2008	cross-sectional	school	IOTF	1779	8568
Quinn, et al	Ireland	2022	2011-2014	cohort	database	National Reference	222	4144
Heslin, et al (1)	Ireland	2023	2005-2006	cross-sectional	database	IOTF	67	440
Heslin, et al (2)	Ireland	2023	2019-2020	cross-sectional	database	IOTF	67	425
Barron, et al	Ireland	2009	2007	cross-sectional	school	IOTF	173	969
Dowd, et al	Ireland	2015	2006	cohort	community	IOTF	1532	7914
Evans, et al	Ireland	2011	2004-2007	cross-sectional	school	IOTF	670	3482
Whelton, et al	Ireland	2007	2001	cross-sectional	school	IOTF	3690	19538
O'Neill, et al	Ireland	2007	2003-2004	cross-sectional	school	IOTF	56	375
Kelly, et al	Ireland	2019	2019	longitudinal	database	National Reference	853	8186
McMaster, et al	Ireland	2005	2001-2002	cross-sectional	school	IOTF	55	328
Janssen, et al (15)	Ireland	2005	2001-2002	cross-sectional	database	IOTF	129	1140
Belton, et al	Ireland	2010	2008	cross-sectional	school	IOTF	43	301
Haug, et al (33)	Ireland	2009	2005-2006	cross-sectional	database	IOTF	177	1566
Perry, et al	Ireland	2015	2007-2008	cross-sectional	school	IOTF	1565	8136
Pinhas-Hamiel, et al	Israel	2009	2009	cross-sectional	school	CDC	51	204
Dayan, et al	Israel	2003	2003	cross-sectional	medical institution	National Reference	9202	76732
Janssen, et al (16)	Israel	2005	2001-2002	cross-sectional	database	IOTF	390	4200
Weisband, et al	Israel	2020	2018	cross-sectional	database	WHO	13206	116774
Haug, et al (1)	Israel	2009	2005-2006	cross-sectional	database	IOTF	412	4037
Meyerovitch, et al	Israel	2007	2001-2004	cross-sectional	medical institution	CDC	97	949
Goldberg, et al	Israel	2014	2014	cross-sectional	database	National Reference	45119	404922
Kaufman-Shriqui, et al	Israel	2013	2008	cross-sectional	school	WHO	44	238
Ahrens, et al (1)	Italy	2014	2007-2008	cross-sectional	database	IOTF	545	2424
Galfo, et al	Italy	2022	2011-2012	cross-sectional	database	IOTF	57	369
Salas, et al (7)	Italy	2021	2015-2017	cross-sectional	database	WHO	9604	42496
Ashi, et al (1)	Italy	2019	2019	cross-sectional	school	WHO	31	220
Barba, et al	Italy	2006	2003-2004	cross-sectional	school	IOTF	1018	3923
Ceschia, et al	Italy	2016	2016	cross-sectional	school	IOTF	441	2411
Toselli, et al	Italy	2014	2007-2008	cross-sectional	school	IOTF	304	1432
Caserta, et al	Italy	2010	2007-2008	cross-sectional	school	IOTF	179	575
Genovesi, et al	Italy	2010	2004-2006	cross-sectional	school	IOTF	1025	5131
Bertoncello, et al	Italy	2008	2004	cross-sectional	school	IOTF	2722	12853
Albertini, et al	Italy	2008	2002-2005	cross-sectional	school	IOTF	1053	5636
Maffeis, et al	Italy	2006	2002	cross-sectional	school	IOTF	357	2150
Parrino, et al	Italy	2012	2009-2010	cross-sectional	school	IOTF	125	915
Parrino, et al	Italy	2016	2016	cross-sectional	school	IOTF	410	1521
Lombardo, et al	Italy	2015	2010	cross-sectional	database	WHO	9690	41592
Janssen, et	Italy	2005	2001-2002	cross-sectional	database	IOTF	610	4095

© 2024 Zhang X et al. *JAMA Pediatrics*.

al (17)								
Pecoraro, et al	Italy	2003	2003	cross-sectional	school	IOTF	60	228
Collo, et al	Italy	2019	2016-2017	cross-sectional	medical institution	WHO	9	86
Toselli, et al	Italy	2010	2010	cross-sectional	school	IOTF	130	598
Greco, et al	Italy	2020	2016-2017	cross-sectional	school	IOTF	346	1122
Binkin, et al	Italy	2010	2008	cross-sectional	school	IOTF	11846	50197
Rapa, et al	Italy	2009	2007-2008	cross-sectional	medical institution	National Reference	10	88
Haug, et al (12)	Italy	2009	2005-2006	cross-sectional	database	IOTF	572	3556
Caserta, et al	Italy	2009	2007-2008	cross-sectional	school	IOTF	196	642
Bonaccorsi, et al	Italy	2009	2005	cross-sectional	school	National Reference	80	449
Turconi, et al	Italy	2008	2002	cross-sectional	school	IOTF	94	532
Maffeis, et al	Italy	2008	2003	cross-sectional	school	IOTF	387	1836
Lazzeri, et al (1)	Italy	2008	2002	cross-sectional	school	IOTF	695	3048
Lazzeri, et al (2)	Italy	2008	2006	cross-sectional	school	IOTF	359	1430
Lazzeri, et al (3)	Italy	2008	2004	cross-sectional	school	IOTF	184	1066
Lazzeri, et al (4)	Italy	2008	2006	cross-sectional	school	IOTF	164	997
Lazzeri, et al (5)	Italy	2008	2004	cross-sectional	school	IOTF	160	1185
Lazzeri, et al (6)	Italy	2008	2006	cross-sectional	school	IOTF	160	1086
Lazzeri, et al (7)	Italy	2008	2004	cross-sectional	school	IOTF	139	1160
Lazzeri, et al (8)	Italy	2008	2006	cross-sectional	school	IOTF	184	1078
Guida, et al	Italy	2008	2001	cross-sectional	school	IOTF	135	464
Gualdi-Russo, et al	Italy	2008	2000-2001	cross-sectional	database	IOTF	223	866
Fuiano, et al	Italy	2008	2005	longitudinal	school	National Reference	140	632
Vidal, et al	Italy	2006	2006	cross-sectional	community	IOTF	37	258
Menghetti, et al	Italy	2015	2015	cross-sectional	school	IOTF	546	2007
Lazzeri, et al (1)	Italy	2015	2002	cross-sectional	database	IOTF	327	1485
Lazzeri, et al (2)	Italy	2015	2006	cross-sectional	database	IOTF	365	1405
Lazzeri, et al (3)	Italy	2015	2008	cross-sectional	database	IOTF	304	1375
Lazzeri, et al (4)	Italy	2015	2010	cross-sectional	database	IOTF	303	1424
Lazzeri, et al (5)	Italy	2015	2012	cross-sectional	database	IOTF	285	1494
Geremia, et al	Italy	2015	2015	cross-sectional	school	WHO	96	590
Weinmayr, et al (8)	Italy	2014	2004	cross-sectional	database	IOTF	402	1307
Lazzeri, et al	Italy	2014	2009-2010	cross-sectional	database	IOTF	7194	47583
Sacchetti, et al	Italy	2012	2012	cross-sectional	school	IOTF	121	489
Bracale, et al	Italy	2013	2008	cross-sectional	community	IOTF	2444	16588
Petracci, et al	Italy	2013	2003	cross-sectional	database	IOTF	891	4338
Galfo, et al	Italy	2016	2011-2012	cross-sectional	database	IOTF	57	369
Grassi, et al	Italy	2016	2014-2015	cross-sectional	database	IOTF	146	1164
Romano, et al (12)	Jamaica	2022	2017	cross-sectional	database	WHO	166	1061
Xu, et al (2)	Japan	2021	2014	cross-sectional	school	WHO	112	1200
Tani, et al	Japan	2021	2018	cross-sectional	database	WHO	716	5257
Watanabe, et al	Japan	2011	2003	cross-sectional	school	IOTF	131	1765
Nakano, et	Japan	2010	2001	longitudinal	school	IOTF	2665	16245

© 2024 Zhang X et al. *JAMA Pediatrics.*

al								
Suzuki, et al	Japan	2009	2000-2001	cohort	database	IOTF	217	1302
Yamashita, et al	Japan	2023	2001	longitudinal	database	IOTF	2988	32081
Okuda, et al	Japan	2010	2006-2008	cross-sectional	school	IOTF	209	3812
Shinozaki, et al	Japan	2015	2006-2010	cross-sectional	database	IOTF	517	5600
Ochiai, et al	Japan	2015	2004-2009	cross-sectional	school	IOTF	289	2499
Mizuta, et al	Japan	2016	2012-2013	cross-sectional	school	WHO	161	2968
Baker, et al	Jordan	2010	2007	cross-sectional	school	CDC	213	1355
Hamaideh, et al	Jordan	2010	2010	cross-sectional	school	IOTF	157	824
Ibrahim, et al	Jordan	2008	2006	cross-sectional	school	WHO	279	1695
Khader, et al	Jordan	2009	2006	cross-sectional	school	IOTF	412	2131
Al-Akour, et al	Jordan	2012	2009	cross-sectional	school	CDC	252	1433
Al-Domi, et al	Jordan	2019	2009-2011	cross-sectional	school	IOTF	250	767
Al-Kloub, et al	Jordan	2010	2008	cross-sectional	school	IOTF	91	518
Tayyem, et al	Jordan	2014	2009	cross-sectional	database	IOTF	103	735
Musaiger, et al (2)	Jordan	2012	2010-2011	cross-sectional	school	IOTF	183	937
Musaiger, et al	Jordan	2013	2013	cross-sectional	school	IOTF	83	475
Musaiger, et al (2)	Jordan	2016	2013-2014	cross-sectional	database	IOTF	135	795
Zayed, et al	Jordan	2016	2015-2016	cross-sectional	school	CDC	463	2702
Salas, et al (8)	Kazakhstan	2021	2015-2017	cross-sectional	database	WHO	534	3988
Facchini, et al (1)	Kazakhstan	2007	2002-2004	cross-sectional	database	CDC	98	2400
Kyallo, et al	Kenya	2013	2008	cross-sectional	school	WHO	42	321
Muthuri, et al	Kenya	2014	2012	cross-sectional	school	WHO	81	563
Gewa, et al	Kenya	2010	2003	cross-sectional	database	WHO	202	1443
Pengpid, et al (2)	Kiribati	2015	2010-2011	cross-sectional	database	IOTF	514	1582
Abdelalim, et al	Kuwait	2012	2012	cross-sectional	community	CDC	232	1006
Alqaoud, et al	Kuwait	2022	2007-2019	cross-sectional	database	WHO	4188	47782
Al-Haifi, et al	Kuwait	2022	2019	cross-sectional	school	IOTF	169	706
Badr, et al	Kuwait	2017	2010-2011	cross-sectional	database	CDC	579	2672
El-Ghaziri, et al	Kuwait	2011	2011	cross-sectional	school	CDC	11	499
El-Bayoumy, et al	Kuwait	2009	2006	cross-sectional	school	National Reference	1660	5402
AlRodhan, et al	Kuwait	2019	2019	cross-sectional	school	CDC	335	2208
Al-Qaoud, et al	Kuwait	2009	2003-2004	cross-sectional	school	CDC	184	2291
Al-Refaee, et al	Kuwait	2013	2012	cross-sectional	medical institution	CDC	52	361
Musaiger, et al (1)	Kuwait	2013	2010-2011	cross-sectional	database	IOTF	144	628
Musaiger, et al (3)	Kuwait	2016	2013-2014	cross-sectional	database	IOTF	165	706
Salas, et al (9)	Kyrgyzstan	2021	2015-2017	cross-sectional	database	WHO	429	5958
Romano, et al (13)	Laos	2022	2015	cross-sectional	database	WHO	155	1644
Salas, et al (11)	Latvia	2021	2015-2017	cross-sectional	database	WHO	945	5593
Janssen, et al (18)	Latvia	2005	2001-2002	cross-sectional	database	IOTF	167	3091
Haug, et al (34)	Latvia	2009	2005-2006	cross-sectional	database	IOTF	253	3778
Weinmayr, et al (9)	Latvia	2014	2004	cross-sectional	database	IOTF	20	156
Karklina, et	Latvia	2011	2007-2009	cross-sectional	school	National	85	504

© 2024 Zhang X et al. *JAMA Pediatrics.*

al						Reference		
Nasreddine, et al	Lebanon	2014	2009	cross-sectional	community	WHO	148	368
Chacar, et al	Lebanon	2011	2007	cross-sectional	school	IOTF	523	2547
Romano, et al (14)	Lebanon	2022	2017	cross-sectional	database	WHO	679	3347
Jabre, et al	Lebanon	2005	2000	cross-sectional	community	IOTF	36	234
Fazah, et al	Lebanon	2010	2010	cross-sectional	school	IOTF	166	982
Chakar, et al	Lebanon	2006	2002-2003	cross-sectional	school	IOTF	2957	12128
Nasreddine, et al	Lebanon	2017	2011-2012	cross-sectional	community	WHO	34	525
Gebremedhin, et al (12)	Liberia	2015	2013	cross-sectional	database	WHO	101	3259
Musaiger, et al (3)	Libya	2012	2010-2011	cross-sectional	school	IOTF	139	630
Musaiger, et al (2)	Libya	2013	2010-2011	cross-sectional	database	IOTF	139	630
Musaiger, et al (4)	Libya	2016	2013-2014	cross-sectional	database	IOTF	132	759
Salas, et al (10)	Lithuania	2021	2015-2017	cross-sectional	database	WHO	539	3431
Smetanina, et al	Lithuania	2015	2008-2010	cross-sectional	school	IOTF	503	3990
Janssen, et al (19)	Lithuania	2005	2001-2002	cross-sectional	database	IOTF	197	4194
Haug, et al (35)	Lithuania	2009	2005-2006	cross-sectional	database	IOTF	241	3830
Wijnhoven, et al (3)	Lithuania	2015	2007/2008	cross-sectional	school	WHO	943	4084
Haug, et al (7)	Luxemburg	2009	2005-2006	cross-sectional	database	IOTF	402	3904
Raufi, et al	Macedonia	2022	2019-2020	cross-sectional	medical institution	CDC	140	1034
Janssen, et al (20)	Macedonia	2005	2001-2002	cross-sectional	database	IOTF	378	3596
Haug, et al (13)	Macedonia	2009	2005-2006	cross-sectional	database	IOTF	604	4911
Myrtaj, et al	Macedonia	2018	2016	cross-sectional	school	IOTF	452	2390
Manyanga, et al (5)	Malawi	2014	2014	cross-sectional	database	WHO	213	2305
Gebremedhin, et al (13)	Malawi	2015	2010	cross-sectional	database	WHO	420	4829
Mohamed, et al	Malaysia	2023	2014-2015	cross-sectional	school	IOTF	123	917
Lai, et al	Malaysia	2022	2019	cross-sectional	school	WHO	377	2221
Al-Sadat, et al	Malaysia	2016	2012	cross-sectional	database	IOTF	210	1350
Khor, et al	Malaysia	2011	2008	cross-sectional	school	WHO	72	402
Adeyemi, et al	Malaysia	2014	2014	cross-sectional	school	IOTF	65	411
Ahmad, et al	Malaysia	2017	2014-2015	cross-sectional	database	WHO	9385	62567
Tee, et al	Malaysia	2018	2013	cross-sectional	school	WHO	1164	8322
Kee, et al	Malaysia	2017	2013/2014	cross-sectional	database	WHO	81	663
Mahaletchumy, et al	Malaysia	2019	2016	cross-sectional	school	WHO	999	6248
Cheah, et al	Malaysia	2019	2019	cross-sectional	school	WHO	185	1314
Tan, et al	Malaysia	2019	2012	cross-sectional	database	WHO	3285	24339
Mohd, et al	Malaysia	2006	2006	cross-sectional	school	IOTF	102	699
Woon, et al	Malaysia	2015	2015	cross-sectional	school	WHO	50	333
Loh, et al	Malaysia	2015	2013	cross-sectional	school	IOTF	104	646
Kaartina, et al	Malaysia	2015	2011	cross-sectional	school	WHO	10	379
Su, et al	Malaysia	2014	2014	cross-sectional	school	IOTF	210	1327
Poh, et al	Malaysia	2016	2015	cross-sectional	database	WHO	367	3227
Shariff, et al	Malaysia	2016	2016	cross-sectional	community	WHO	101	745
Partap, et al	Malaysia	2017	2013-2014	cross-sectional	database	IOTF	1700	6414
Gebremedhin, et al (14)	Mali	2015	2012/2013	cross-sectional	database	WHO	129	4591
Salas, et al (12)	Malta	2021	2015-2017	cross-sectional	database	WHO	573	3115
Decelis, et al	Malta	2014	2012	cross-sectional	database	IOTF	165	811

© 2024 Zhang X et al. *JAMA Pediatrics*.

Janssen, et al (21)	Malta	2005	2001-2002	cross-sectional	database	IOTF	186	1065
Haug, et al (14)	Malta	2009	2005-2006	cross-sectional	database	IOTF	192	1039
Decelis, et al	Malta	2013	2012	cross-sectional	school	IOTF	178	874
Manyanga, et al (6)	Mauritania	2014	2014	cross-sectional	database	WHO	424	2028
Caleyachetty, et al	Mauritius	2012	2006	cross-sectional	school	IOTF	146	841
Fokeena, et al	Mauritius	2012	2012	cross-sectional	school	CDC	23	200
Romano, et al (15)	Mauritius	2022	2017	cross-sectional	database	WHO	303	1955
Shamah-Levy, et al (1)	Mexico	2022	2006	cross-sectional	database	WHO	3056	14990
Shamah-Levy, et al (2)	Mexico	2022	2012	cross-sectional	database	WHO	3189	16351
Shamah-Levy, et al (3)	Mexico	2022	2016	cross-sectional	database	WHO	551	3179
Shamah-Levy, et al (4)	Mexico	2022	2018	cross-sectional	database	WHO	1209	6183
Shamah-Levy, et al (5)	Mexico	2022	2020	cross-sectional	database	WHO	388	1944
Shamah-Levy, et al (6)	Mexico	2022	2021	cross-sectional	database	WHO	510	2569
Brambila-Paz, et al	Mexico	2022	2002	longitudinal	database	WHO	87	3202
Aguilera-Galaviz, et al	Mexico	2019	2016	cross-sectional	school	WHO	41	203
Ashi, et al (2)	Mexico	2019	2019	cross-sectional	school	WHO	47	224
Pérez, et al	Mexico	2020	2018	cross-sectional	school	WHO	92	522
Irigoyen-Camacho, et al	Mexico	2014	2010-2011	cross-sectional	school	IOTF	63	257
Patiño-Marín, et al	Mexico	2018	2013-2016	cross-sectional	school	WHO	285	1527
Sánchez-Pérez, et al	Mexico	2010	2010	longitudinal	school	CDC	11	88
Serrano-Piña, et al	Mexico	2019	2019	cross-sectional	school	National Reference	83	331
Vázquez-Nava, et al	Mexico	2010	2005	cross-sectional	school	CDC	165	1160
Ávila-Ortiz, et al	Mexico	2017	2011-2013	cross-sectional	school	CDC	14	91
Bacardi-Gascón, et al	Mexico	2009	2006-2007	cross-sectional	school	National Reference	367	1684
Lopez-Gonzalez, et al	Mexico	2020	2015-2019	cross-sectional	community	CDC	234	1449
Del-Rio-Navarro, et al	Mexico	2008	2005	cross-sectional	school	CDC	418	1819
Gómez-Díaz, et al	Mexico	2005	2005	cross-sectional	school	IOTF	85	833
Vergara-Castañeda, et al	Mexico	2010	2010	cross-sectional	school	National Reference	24	83
Jimenez-Cruz, et al	Mexico	2010	2009	cross-sectional	medical institution	WHO	104	1685
Macías-Rosales, et al	Mexico	2009	2004	cross-sectional	medical institution	CDC	99	641
Flores-	Mexico	2009	2005-2006	cross-sectional	school	CDC	381	2029

© 2024 Zhang X et al. *JAMA Pediatrics.*

Huerta, et al								
Ortiz-Hernández, et al	Mexico	2008	2005	cross-sectional	school	CDC	378	1015
Basaldúa, et al	Mexico	2008	2004	cross-sectional	school	IOTF	134	551
Villa-Caballero, et al	Mexico	2006	2001-2002	cross-sectional	school	CDC	220	1172
RODRÍGUEZ-FUENTES, et al	Mexico	2015	2011	cross-sectional	school	CDC	17	104
López-Barrón, et al	Mexico	2015	2010-2011	cross-sectional	school	WHO	191	684
Flores, et al	Mexico	2015	2008	cross-sectional	medical institution	WHO	63	164
Banik, et al	Mexico	2014	2008-2009	cross-sectional	school	WHO	81	321
Banik, et al	Mexico	2014	2014	cross-sectional	school	WHO	75	283
Barrera, et al	Mexico	2016	2012-2013	cross-sectional	school	WHO	180	725
Caraza, et al	Mexico	2016	2013-2014	cross-sectional	school	CDC	74	605
Peña, et al	Mexico	2018	2018	cross-sectional	school	WHO	252	849
Ramírez-Jiménez ,et al	Mexico	2018	2011	cross-sectional	community	WHO	10	93
Romano, et al (16)	Mongolia	2022	2013	cross-sectional	database	WHO	374	3707
Salas, et al (13)	Montenegro	2021	2015-2017	cross-sectional	database	WHO	546	2678
Banjari, et al (1)	Montenegro	2020	2020	cross-sectional	school	IOTF	44	224
Martinovic, et al	Montenegro	2015	2012-2013	cross-sectional	school	IOTF	721	4097
El Moussaoui, et al	Morocco	2022	2020	cross-sectional	medical institution	WHO	112	450
Dekkaki, et al	Morocco	2011	2010	cross-sectional	school	WHO	80	1570
Manyanga, et al (7)	Morocco	2014	2014	cross-sectional	database	WHO	749	5756
El Kabbaoui, et al	Morocco	2018	2014-2015	cross-sectional	school	WHO	140	1818
Gebremedhin, et al (15)	Mozambique	2015	2011	cross-sectional	database	WHO	749	9721
Bishwajt, et al	Multiple Countries	2019	2014-2018	cross-sectional	database	WHO	20775	132231
Stival, et al (1)	Multiple Countries	2022	2019	cross-sectional	database	WHO	544	3093
Stival, et al (2)	Multiple Countries	2022	2018	cross-sectional	database	WHO	356	2916
Moschonis, et al	Multiple Countries	2022	2016-2018	cross-sectional	database	IOTF	2165	12030
Ferrari, et al	Multiple Countries	2022	2014-2015	cross-sectional	database	WHO	119	671
Collings, et al	Multiple Countries	2022	2022	cross-sectional	database	National Reference	2069	15810
Peltzer, et al	Multiple Countries	2011	2003/2007	cross-sectional	school	IOTF	373	5613
Vrijheid, et al	Multiple Countries	2020	2013/2016	cohort	database	WHO	374	1301
Ekelund, et al	Multiple Countries	2004	2004	cross-sectional	school	IOTF	152	1292
Olaya, et al	Multiple Countries	2015	2010	cross-sectional	database	IOTF	812	5206
Tsitsika, et al	Multiple Countries	2016	2011-2012	cross-sectional	school	IOTF	1092	10287
Gebremedhin, et al (16)	Namibia	2015	2013	cross-sectional	database	WHO	68	1845
Romano, et al (17)	Namibia	2022	2013	cross-sectional	database	WHO	110	1936
Achaya, et al	Nepal	2016	2013	cross-sectional	school	WHO	49	838

© 2024 Zhang X et al. *JAMA Pediatrics*.

Romano, et al (18)	Nepal	2022	2015	cross-sectional	database	WHO	309	4616
Bhattarai, et al	Nepal	2019	2017	cross-sectional	school	CDC	16	510
Karki, et al	Nepal	2019	2017	cross-sectional	school	WHO	107	575
Koirala, et al	Nepal	2015	2013	cross-sectional	school	WHO	144	986
Gurung, et al	Nepal	2014	2010	cross-sectional	school	IOTF	36	300
Pandey, et al	Nepal	2018	2018	cross-sectional	school	WHO	12	120
Greeff, et al	Netherlands	2016	2016	RCT	school	IOTF	65	376
Jabakhanji, et al	Netherlands	2022	2013	cohort	database	IOTF	1322	7042
Sergentanis, et al (2)	Netherlands	2021	2011-2012	cross-sectional	database	IOTF	46	727
Croezen, et al	Netherlands	2009	2003	cross-sectional	database	IOTF	1725	25176
Drukker, et al	Netherlands	2009	2004-2005	cross-sectional	database	IOTF	127	1411
Jansen, et al	Netherlands	2012	2004-2010	cross-sectional	database	IOTF	239	3157
Wilde, et al	Netherlands	2009	2007	cross-sectional	database	IOTF	1426	10308
Yngve, et al (5)	Netherlands	2008	2003	cross-sectional	database	IOTF	57	684
Akbulut, et al (2)	Netherlands	2014	2003-2011	cross-sectional	database	IOTF	134	433
Jansen, et al	Netherlands	2008	2000-2001	cross-sectional	database	IOTF	423	1923
Janssen, et al (22)	Netherlands	2005	2001-2002	cross-sectional	database	IOTF	270	3860
Wilde, et al	Netherlands	2019	2007-2014	cross-sectional	medical institution	IOTF	17733	109769
Luttikhuis, et al	Netherlands	2010	2006	cross-sectional	school	IOTF	20	397
Jansen, et al	Netherlands	2010	2006	cross-sectional	school	IOTF	234	1095
Haug, et al (8)	Netherlands	2009	2005-2006	cross-sectional	database	IOTF	277	3850
Bossink-Tuna, et al	Netherlands	2009	2006	cross-sectional	medical institution	IOTF	41	635
Snoek, et al	Netherlands	2007	2007	cross-sectional	school	IOTF	812	9011
Scholtens, et al	Netherlands	2007	2000-2001	cross-sectional	database	IOTF	93	864
Jansen, et al	Netherlands	2006	2000-2001	cross-sectional	database	IOTF	389	1819
Weinmayr, et al (10)	Netherlands	2014	2004	cross-sectional	database	IOTF	413	2638
Schwiebbe, et al	Netherlands	2011	2008	cross-sectional	school	IOTF	352	2148
Veldwijk, et al	Netherlands	2012	2003-2007	cross-sectional	school	IOTF	4148	51856
Willeboordse, et al	Netherlands	2013	2010	cross-sectional	community	National Reference	1412	9272
Leppers, et al	Netherlands	2017	2012-2015	cross-sectional	medical institution	IOTF	420	3408
Velde, et al (4)	Netherlands	2017	2010	cross-sectional	database	IOTF	107	919
Utter, et al	New Zealand	2006	2002	cross-sectional	database	IOTF	574	2716
Aung, et al	New Zealand	2021	2014-2015	cross-sectional	school	National Reference	3948	27333
Chelimo, et al	New Zealand	2020	2007-2010	cohort	medical institution	IOTF	1051	5128
Rockell, et al	New Zealand	2005	2002	cross-sectional	school	IOTF	789	3275
Utter, et al	New Zealand	2010	2007	cross-sectional	school	IOTF	2111	8796
Quigg, et al	New Zealand	2010	2007	cross-sectional	school	CDC	37	184
Weinmayr, et al (11)	New Zealand	2014	2004	cross-sectional	database	IOTF	52	222
Rajput, et al	New Zealand	2014	2009-2012	cross-sectional	database	WHO	30880	168744
Gebremedhin, et al (17)	Niger	2015	2012	cross-sectional	database	WHO	113	5123
Adeomi, et al	Nigeria	2019	2019	cross-sectional	school	WHO	26	309

© 2024 Zhang X et al. *JAMA Pediatrics*.

Adetunji, et al	Nigeria	2019	2013	cross-sectional	school	WHO	41	1187
Maruf, et al	Nigeria	2013	2009	cross-sectional	school	IOTF	552	9014
Ene-obong, et al	Nigeria	2012	2012	cross-sectional	school	IOTF	182	1599
Gebremedhin, et al (18)	Nigeria	2015	2013	cross-sectional	database	WHO	990	26758
Goon, et al	Nigeria	2010	2005	cross-sectional	school	CDC	54	2015
Wariri, et al	Nigeria	2020	2015-2017	cross-sectional	community	WHO	129	2100
Mezie-Okoye, et al	Nigeria	2015	2010	cross-sectional	school	CDC	33	220
Musa, et al	Nigeria	2012	2012	cross-sectional	school	IOTF	402	3240
Oduwole, et al	Nigeria	2012	2012	cross-sectional	school	CDC	122	885
Sadoh, et al	Nigeria	2016	2011-2012	cross-sectional	school	CDC	54	353
Igbokwe, et al	Nigeria	2017	2013	cross-sectional	school	WHO	73	860
Aadland, et al	Norway	2021	2015-2016	cross-sectional	community	IOTF	187	1182
Aadland, et al	Norway	2022	2019-2022	cross-sectional	community	IOTF	146	1003
Donkor, et al	Norway	2017	2007	cohort	school	IOTF	219	1864
Øvrebø, et al	Norway	2021	2017	longitudinal	database	IOTF	244	1838
Andersen, et al	Norway	2005	2000	cross-sectional	school	IOTF	215	1489
Bernhardsen, et al	Norway	2019	2013-2015	cohort	database	IOTF	53	445
Oellingrath, et al	Norway	2010	2007	cross-sectional	school	IOTF	151	955
Júlíusson, et al	Norway	2010	2003-2006	cross-sectional	community	IOTF	850	6140
Kolle, et al	Norway	2009	2005	cross-sectional	database	IOTF	68	440
Júlíusson, et al (2)	Norway	2015	2015	cross-sectional	database	IOTF	709	6139
Yngve, et al (6)	Norway	2008	2003	cross-sectional	database	IOTF	90	670
Hestetun, et al	Norway	2015	2010	cross-sectional	school	IOTF	104	744
Janssen, et al (23)	Norway	2005	2001-2002	cross-sectional	database	IOTF	455	4415
Haug, et al (36)	Norway	2009	2005-2006	cross-sectional	database	IOTF	331	3675
Grøholt, et al	Norway	2008	2000-2004	cross-sectional	school	IOTF	1625	15966
Weinmayr, et al (12)	Norway	2014	2004	cross-sectional	database	IOTF	106	637
Brannsether, et al	Norway	2014	2003-2006	cross-sectional	database	IOTF	534	4576
Velde, et al (5)	Norway	2017	2010	cross-sectional	database	IOTF	126	1000
Tanveer, et al	Pakistan	2022	2021	cross-sectional	school	CDC	205	3551
Bekhwani, et al	Pakistan	2022	2020-2021	cross-sectional	medical institution	CDC	25	184
Ahmed, et al	Pakistan	2013	2008-2009	cross-sectional	school	WHO	40	501
Mansoori, et al	Pakistan	2018	2012	cross-sectional	school	CDC	169	887
Anwar, et al	Pakistan	2010	2009	cross-sectional	school	WHO	35	293
Hashmi, et al	Pakistan	2013	2008-2009	cross-sectional	school	WHO	98	501
Ramzan, et al	Pakistan	2008	2008	cross-sectional	school	CDC	118	1136
Aziz, et al	Pakistan	2009	2007	cross-sectional	school	WHO	77	398
Ishaque, et al	Pakistan	2012	2012	cross-sectional	school	WHO	75	431
Abudayya, et al	Palestine	2007	2002	cross-sectional	school	WHO	129	1022
Badrasawi, et al	Palestine	2019	2017	cross-sectional	school	WHO	54	392
Al-Lahham, et al	Palestine	2019	2016	cross-sectional	school	CDC	191	1320
Mikki, et al	Palestine	2009	2007-2008	cross-sectional	school	IOTF	285	1942

© 2024 Zhang X et al. *JAMA Pediatrics*.

Weinmayr, et al (13)	Palestine	2014	2004	cross-sectional	database	IOTF	25	216
Musaiger, et al (3)	Palestine	2013	2010-2011	cross-sectional	database	IOTF	60	477
Massad, et al	Palestine	2016	2009	cross-sectional	school	CDC	180	1484
Musaiger, et al (5)	Palestine	2016	2013-2014	cross-sectional	database	IOTF	99	610
Carrillo-Larco, et al (1)	Peru	2014	2002	cohort	database	IOTF	304	1929
Romano, et al (19)	Peru	2022	2010	cross-sectional	database	WHO	418	2359
Romano, et al (20)	Philippines	2022	2015	cross-sectional	database	WHO	487	6162
Suligowska, et al	Poland	2022	2017-2019	cross-sectional	database	WHO	71	381
Potempa-Jeziorowska, et al	Poland	2022	2018-2019	cross-sectional	school	WHO	93	908
Kryst, et al (1)	Poland	2022	2009-2010	cross-sectional	school	IOTF	318	1926
Kryst, et al (2)	Poland	2022	2019-2020	cross-sectional	school	IOTF	311	1850
Bryl, et al	Poland	2022	2017-2019	cross-sectional	school	IOTF	80	530
Basiak-Rasała, et al	Poland	2022	2016	cross-sectional	school	WHO	320	2913
Wieniawski, et al	Poland	2021	2021	cross-sectional	school	National Reference	64	690
Usheva, et al (5)	Poland	2021	2012	cross-sectional	database	WHO	83	1334
Sergentanis, et al (4)	Poland	2021	2011-2012	cross-sectional	database	IOTF	122	1190
Salas, et al (14)	Poland	2021	2015-2017	cross-sectional	database	WHO	519	2884
Matłosz, et al	Poland	2021	2018-2019	cross-sectional	school	CDC	78	1172
Żegleń, et al (1)	Poland	2020	2008	cross-sectional	school	IOTF	133	1113
Żegleń, et al (2)	Poland	2020	2018	cross-sectional	school	IOTF	110	1054
Szczyrska, et al	Poland	2020	2007-2016	cross-sectional	database	National Reference	924	12330
Tomaszewski, et al	Poland	2015	2015	cross-sectional	school	IOTF	122	791
Bac, et al	Poland	2012	2008-2009	cross-sectional	community	IOTF	329	1495
Baran, et al	Poland	2022	2015	cross-sectional	school	National Reference	71	749
Kułaga, et al	Poland	2011	2007-2009	cross-sectional	database	IOTF	2022	15607
Malecka-Tendera, et al	Poland	2005	2001	cross-sectional	school	IOTF	345	2916
Kowal,et al (1)	Poland	2014	2000	cross-sectional	school	IOTF	216	2062
Kowal,et al (2)	Poland	2014	2010	cross-sectional	school	IOTF	220	1970
Chrzanowska, et al	Poland	2007	2000	cross-sectional	school	IOTF	254	2003
Ząḑzińska, et al	Poland	2013	2002-2004	cross-sectional	community	IOTF	96	812
Gorog, et al (4)	Poland	2011	2011	cross-sectional	database	IOTF	242	1454
Høyer, et al (2)	Poland	2014	2002-2005	cohort	database	WHO	12	92
Zatoński, et al	Poland	2020	2016-2017	cross-sectional	school	National Reference	320	2913
Janssen, et al (24)	Poland	2005	2001-2002	cross-sectional	database	IOTF	437	5909
Woźniacka, et al	Poland	2018	2009-2010	cross-sectional	school	IOTF	519	3405
Baran, et al	Poland	2019	2019	cross-sectional	school	National Reference	16	300
Jodkowska,	Poland	2010	2005	cross-sectional	school	IOTF	1008	8065

© 2024 Zhang X et al. *JAMA Pediatrics.*

et al								
Haug, et al (22)	Poland	2009	2005-2006	cross-sectional	database	IOTF	490	5324
Klimek-Piotrowska, et al	Poland	2015	2012-2013	cross-sectional	school	National Reference	99	970
Długosz, et al	Poland	2015	2010-2011	cross-sectional	school	IOTF	78	553
Stankiewicz, et al	Poland	2014	2014	cross-sectional	community	National Reference	136	1515
Golec, et al	Poland	2014	2012	cross-sectional	school	WHO	59	305
Rutkowski, et al	Poland	2013	2006-2010	cross-sectional	school	National Reference	72	889
Czyż, et al	Poland	2017	2012-2014	cross-sectional	school	IOTF	84	641
Andaki, et al (2)	Portugal	2017	2009-2015	cross-sectional	community	IOTF	644	4052
Rodrigues, et al (1)	Portugal	2022	2009/2010	cross-sectional	school	IOTF	342	1996
Rodrigues, et al (2)	Portugal	2022	2016/2017	cross-sectional	school	IOTF	293	2077
Salas, et al (15)	Portugal	2021	2015-2017	cross-sectional	database	WHO	1132	5992
Paciência, et al	Portugal	2021	2014-2015	cross-sectional	school	WHO	172	845
Abreu, et al	Portugal	2014	2008	cross-sectional	school	IOTF	280	1209
Frias-Bulhosa, et al	Portugal	2015	2015	cross-sectional	school	CDC	35	181
Padez, et al	Portugal	2005	2002-2003	cross-sectional	school	IOTF	914	4511
Araújo, et al	Portugal	2012	2003-2004	cross-sectional	school	CDC	175	1171
Silva-Santos, et al	Portugal	2017	2012	cross-sectional	school	IOTF	106	467
Sardinha, et al	Portugal	2011	2008	cross-sectional	school	IOTF	3836	22048
Seabra, et al	Portugal	2013	2010	cross-sectional	school	IOTF	194	682
Rito, et al	Portugal	2012	2007-2008	cross-sectional	school	IOTF	723	3765
Antunes, et al	Portugal	2015	2006	cross-sectional	database	IOTF	245	1273
Ferreira, et al	Portugal	2008	2004	cross-sectional	school	IOTF	259	1125
Marques-Vidal, et al	Portugal	2008	2000-2002	cross-sectional	school	IOTF	930	5013
Yngve, et al (7)	Portugal	2008	2003	cross-sectional	database	IOTF	260	1176
Minghelli, et al	Portugal	2014	2014	cross-sectional	school	WHO	200	966
Albuquerque, et al	Portugal	2012	2011	cross-sectional	school	IOTF	320	1433
Padez, et al	Portugal	2004	2002-2003	cross-sectional	school	IOTF	914	4511
Vale, et al	Portugal	2010	2006-2007	cross-sectional	school	IOTF	169	788
Gama, et al	Portugal	2020	2009-2010	cross-sectional	community	IOTF	3426	17277
Pereira, et al	Portugal	2010	2010	cross-sectional	school	IOTF	274	3699
Haug, et al (15)	Portugal	2009	2005-2006	cross-sectional	database	IOTF	410	3566
Fonseca, et al	Portugal	2009	2002	cross-sectional	database	IOTF	810	5470
Rebelo, et al	Portugal	2008	2000-2002	cross-sectional	school	IOTF	981	5083
Aires, et al	Portugal	2008	2008	cross-sectional	school	IOTF	119	636
Mota, et al	Portugal	2006	2006	cross-sectional	school	IOTF	76	255
Wijnhoven, et al (4)	Portugal	2015	2007/2008	cross-sectional	school	WHO	1138	3026
Minghelli, et al	Portugal	2015	2015	cross-sectional	school	IOTF	178	966
Pedrosa, et al	Portugal	2011	2005-2006	cross-sectional	school	IOTF	181	905
Valente, et al	Portugal	2011	2011	cross-sectional	community	IOTF	423	1675
Vasques, et al	Portugal	2012	2008	cross-sectional	school	IOTF	404	1786
Marques, et al	Portugal	2016	2010	cross-sectional	database	IOTF	437	2938
Marques, et al	Portugal	2018	2018	cross-sectional	school	WHO	190	1396

© 2024 Zhang X et al. *JAMA Pediatrics.*

Rivera-Soto, et al	Puerto Rico	2010	2010	cross-sectional	school	CDC	28	250
Rodriguez, et al	Puerto Rico	2008	2008	cross-sectional	school	CDC	24	234
Elías-Boneta, et al	Puerto Rico	2015	2010-2011	cross-sectional	school	CDC	289	1582
Cheema, et al	Qatar	2022	2018-2020	cross-sectional	school	WHO	107	459
Bener, et al	Qatar	2005	2003-2004	cross-sectional	school	IOTF	1601	7442
Bener, et al	Qatar	2006	2003-2004	cross-sectional	school	IOTF	932	3923
Passmore, et al	Republic of Marshall Islands	2019	2017-2018	cross-sectional	school	CDC	269	3271
Sergentanis, et al (5)	Romania	2021	2011-2012	cross-sectional	database	IOTF	126	1240
Salas, et al (16)	Romania	2021	2015-2017	cross-sectional	database	WHO	1006	5885
Barbu, et al	Romania	2015	2010-2011	cross-sectional	school	WHO	175	866
Pop, et al	Romania	2021	2016	cross-sectional	school	WHO	2989	21625
Valean, et al	Romania	2009	2009	cross-sectional	school	National Reference	656	7904
Mocanu, et al	Romania	2013	2008-2012	cross-sectional	school	IOTF	572	3444
Emandi, et al	Romania	2012	2010-2011	cross-sectional	school	IOTF	659	3626
Cinteza, et al	Romania	2013	2006-2008	cross-sectional	school	CDC	490	4866
Gorog, et al (5)	Romania	2011	2011	cross-sectional	database	IOTF	112	1359
Haug, et al (23)	Romania	2009	2005-2006	cross-sectional	database	IOTF	423	4450
Salas, et al (17)	Russia	2021	2015-2017	cross-sectional	database	WHO	310	2001
Janssen, et al (25)	Russia	2005	2001-2002	cross-sectional	database	IOTF	395	7450
Khasnutdinova, et al	Russia	2010	2006	cross-sectional	school	IOTF	75	1066
Haug, et al (24)	Russia	2009	2005-2006	cross-sectional	database	IOTF	594	6833
Facchini, et al (2)	Russia	2007	2002-2004	cross-sectional	database	CDC	126	2408
Gebremedhin, et al (19)	Rwanda	2015	2010	cross-sectional	database	WHO	305	4116
Pengpid, et al (3)	Samoa	2015	2010-2011	cross-sectional	database	IOTF	788	2418
Salas, et al (18)	San Marino	2021	2015-2017	cross-sectional	database	WHO	64	303
Mohamed, et al	Saudi Arabia	2022	2022	cross-sectional	school	National Reference	227	1250
Abu El Qomsan, et al	Saudi Arabia	2017	2017	cross-sectional	school	CDC	71	386
Ashi, et al (3)	Saudi Arabia	2019	2019	cross-sectional	school	WHO	31	225
Alghamdi, et al	Saudi Arabia	2017	2015	cross-sectional	school	National Reference	104	610
Bhayat, et al	Saudi Arabia	2016	2014	cross-sectional	school	WHO	100	402
Farsi, et al	Saudi Arabia	2017	2014-2015	cross-sectional	school	National Reference	126	801
Farsi, et al	Saudi Arabia	2016	2014-2015	cross-sectional	school	National Reference	163	915
Quadri, et al	Saudi Arabia	2017	2017	cross-sectional	school	CDC	23	360
Bahathig, et al	Saudi Arabia	2021	2019	cross-sectional	school	WHO	52	399
Amin, et al	Saudi Arabia	2008	2008	cross-sectional	school	IOTF	162	1139
Bajamal, et al	Saudi Arabia	2017	2017	cross-sectional	school	IOTF	67	383
Bawazeer, et al	Saudi Arabia	2009	2007	cross-sectional	school	National Reference	926	5498
Abalkhail, et al	Saudi Arabia	2002	2000	cross-sectional	school	WHO	253	1993
Alwan, et al	Saudi Arabia	2013	2006	cross-sectional	school	WHO	259	1212
Al-Almaie, et	Saudi Arabia	2005	2001	cross-sectional	school	WHO	227	1766

© 2024 Zhang X et al. *JAMA Pediatrics.*

al								
Mustafa, et al	Saudi Arabia	2021	2019	cross-sectional	school	WHO	30	300
Al-Agha, et al	Saudi Arabia	2016	2015	cross-sectional	medical institution	CDC	36	365
Al-Hussaini, et al	Saudi Arabia	2019	2015	cross-sectional	school	WHO	1065	7931
Fakeeh, et al	Saudi Arabia	2019	2017	cross-sectional	community	WHO	30	298
Mouzan, et al	Saudi Arabia	2010	2005	cross-sectional	community	WHO	2801	19317
Al-Dossary, et al	Saudi Arabia	2010	2006	cross-sectional	medical institution	CDC	1341	7056
Al-Saeed, et al	Saudi Arabia	2007	2003	cross-sectional	school	IOTF	448	2239
Akinpelu, et al	Saudi Arabia	2014	2014	cross-sectional	school	IOTF	48	1903
El Mouzan, et al	Saudi Arabia	2012	2004-2005	cross-sectional	community	CDC	1124	11112
Al Dahi, et al	Saudi Arabia	2016	2015-2016	cross-sectional	school	IOTF	31	200
Musaiger, et al (6)	Saudi Arabia	2016	2013-2014	cross-sectional	database	IOTF	189	968
Gebremedhin, et al (20)	Senegal	2015	2010/2011	cross-sectional	database	WHO	79	6062
Šušnjević, et al	Serbia	2021	2013	cross-sectional	database	National Reference	194	1376
Djordjic, et al	Serbia	2016	2015	cross-sectional	school	IOTF	788	4861
Rakić, et al	Serbia	2019	2012-2017	cross-sectional	community	IOTF	231	1592
Bukara-Radujković, et al	Serbia	2009	2004	cross-sectional	school	IOTF	147	1204
Markovic, et al	Serbia	2015	2012-2014	cross-sectional	medical institution	WHO	134	406
Rakic, et al	Serbia	2011	2001-2004	cross-sectional	school	National Reference	264	2650
Srdić, et al	Serbia	2012	2012	cross-sectional	medical institution	CDC	308	2284
Janic, et al	Serbia	2013	2013	cross-sectional	school	IOTF	2236	11644
Bjelanovic, et al	Serbia	2017	2013-2014	cross-sectional	school	WHO	688	6444
Halasi, et al	Serbia	2018	2018	cross-sectional	school	IOTF	26	182
Romano, et al (21)	Seychelles	2022	2015	cross-sectional	database	WHO	348	2061
Chiolero, et al	Seychelles	2007	2002-2004	cross-sectional	community	IOTF	1670	15612
Bovet, et al	Seychelles	2007	2004	cross-sectional	school	IOTF	458	4343
Bovet, et al	Seychelles	2010	2004-2006	cross-sectional	school	IOTF	1578	8462
Gebremedhin, et al (21)	Sierra Leone	2015	2013	cross-sectional	database	WHO	348	4698
Pwint, et al	Singapore	2013	2006-2008	cross-sectional	database	CDC	183	2256
Gorog, et al (6)	Slovakia	2011	2011	cross-sectional	database	IOTF	163	1390
Haug, et al (25)	Slovakia	2009	2005-2006	cross-sectional	database	IOTF	288	3688
Janssen, et al (27)	Slovenia	2005	2001-2002	cross-sectional	database	IOTF	486	3769
Planinsec, et al	Slovenia	2004	2004	cross-sectional	school	IOTF	60	364
Planinsec, et al	Slovenia	2009	2006	cross-sectional	school	IOTF	1033	5613
Haug, et al (16)	Slovenia	2009	2005-2006	cross-sectional	database	IOTF	609	4874
Sedej, et al (1)	Slovenia	2014	2001	cross-sectional	medical institution	IOTF	387	2742
Sedej, et al (2)	Slovenia	2014	2004	cross-sectional	medical institution	IOTF	684	4684
Sedej, et al (3)	Slovenia	2014	2009	cross-sectional	medical institution	IOTF	848	5406
Sedej, et al (1)	Slovenia	2016	2004	cross-sectional	school	IOTF	516	2641
Sedej, et al (2)	Slovenia	2016	2009	cross-sectional	school	IOTF	1723	6861

© 2024 Zhang X et al. *JAMA Pediatrics*.

Sedej, et al (3)	Slovenia	2016	2014	cross-sectional	school	IOTF	2115	8036
Velde, et al (6)	Slovenia	2017	2010	cross-sectional	database	IOTF	242	1176
Pengpid, et al (4)	Solomon Islands	2015	2010-2011	cross-sectional	database	IOTF	269	1421
Symington, et al	South Africa	2015	2005	cross-sectional	database	IOTF	43	519
Kirsten, et al	South Africa	2013	2013	cross-sectional	school	IOTF	57	638
Abrahams, et al	South Africa	2011	2008	cross-sectional	school	WHO	92	643
Armstrong, et al	South Africa	2006	2001-2004	cross-sectional	school	IOTF	1213	10283
Baard, et al	South Africa	2014	2014	cross-sectional	school	IOTF	149	713
Moselakgamo, et al	South Africa	2015	2015	cross-sectional	school	National Reference	14	1361
Oldewage-Theron, et al	South Africa	2010	2010	cross-sectional	school	National Reference	17	142
Pedro, et al	South Africa	2014	2009	cross-sectional	database	WHO	37	588
Pienaar, et al	South Africa	2015	2013	longitudinal	database	IOTF	54	574
Tathiah, et al	South Africa	2013	2011	cross-sectional	database	IOTF	81	959
Truter, et al	South Africa	2015	2015	cross-sectional	school	IOTF	43	280
Wiles, et al	South Africa	2013	2013	cross-sectional	school	WHO	83	311
Negash, et al	South Africa	2017	2007-2008	cross-sectional	school	IOTF	243	1559
Reddy, et al (1)	South Africa	2012	2002	cross-sectional	database	IOTF	1562	9522
Reddy, et al (2)	South Africa	2012	2008	cross-sectional	database	IOTF	1893	9371
Moselakgomo, et al	South Africa	2017	2017	cross-sectional	school	CDC	138	1361
Modjadji, et al	South Africa	2019	2017	cross-sectional	school	WHO	17	508
Kruger, et al	South Africa	2006	2000-2001	cross-sectional	school	IOTF	79	1257
Nkeh-Chungag, et al	South Africa	2015	2015	cross-sectional	school	CDC	84	392
Meko, et al	South Africa	2015	2015	cross-sectional	school	WHO	67	414
Van Niekerk, et al	South Africa	2014	2014	cross-sectional	school	IOTF	77	689
Toriola, et al	South Africa	2012	2010	cross-sectional	school	CDC	118	1172
Park, et al (1)	South Korea	2022	2019	cross-sectional	database	National Reference	252743	2612812
Park, et al (2)	South Korea	2022	2020	cross-sectional	database	National Reference	261307	2568754
Kim, et al	South Korea	2021	2017-2019	cross-sectional	database	WHO	207	2136
Baek, et al	South Korea	2012	2009	cross-sectional	database	WHO	7349	72399
Kim, et al	South Korea	2012	2012	cohort	database	WHO	7346	72399
Lee, et al	South Korea	2013	2013	cross-sectional	school	National Reference	49	422
Choo, et al	South Korea	2017	2015	cross-sectional	community	National Reference	17	126
Le, et al (1)	South Korea	2020	2007-2009	cross-sectional	database	National Reference	620	5415
Le, et al (2)	South Korea	2020	2010-2012	cross-sectional	database	National Reference	541	4936
Le, et al (3)	South Korea	2020	2013-2015	cross-sectional	database	National Reference	436	4131
Lee, et al	South Korea	2012	2008	cohort	school	National Reference	110	1030
Kim, et al	South Korea	2005	2002	cross-sectional	school	IOTF	138	1107
Lee, et al	South Korea	2010	2005	cross-sectional	database	IOTF	194	928
Chang, et al	South Korea	2008	2008	cross-sectional	school	IOTF	898	4033
Yoo, et al	South Korea	2006	2006	cross-sectional	school	IOTF	285	938
Kong, et al	South Korea	2015	2013	cross-sectional	database	CDC	6062	53769
Kim, et al	South Korea	2012	2008	cross-sectional	school	National Reference	204	1644
Choi, et al	South Korea	2013	2010-2011	cross-sectional	community	National Reference	219	2038
Cho, et al	South Korea	2018	2007-2014	cross-sectional	database	National Reference	413	7197

© 2024 Zhang X et al. *JAMA Pediatrics*.

Ara, et al	Spain	2007	2007	cross-sectional	school	IOTF	335	1068
Sánchez-Cruz, et al	Spain	2013	2012	cross-sectional	community	WHO	254	978
Gulías-González, et al	Spain	2014	2010	cross-sectional	community	IOTF	547	2301
Ahrens, et al (8)	Spain	2014	2007-2008	cross-sectional	database	IOTF	231	1539
Lasarte-Velillas, et al	Spain	2022	2003-2018	cross-sectional	database	WHO	28618	161335
Bont, et al	Spain	2022	2005-2017	longitudinal	database	WHO	535223	2504568
Cartanyà-Hueso, et al	Spain	2022	2017	cross-sectional	database	IOTF	795	4528
Cabeza, et al	Spain	2022	2022	cross-sectional	school	WHO	57	212
Aragón-Martín, et al	Spain	2022	2018	cross-sectional	school	IOTF	190	864
Vega-Ramirez, et al	Spain	2021	2021	cross-sectional	school	National Reference	75	287
Usheva, et al (6)	Spain	2021	2012	cross-sectional	database	WHO	75	713
Sergentanis, et al (6)	Spain	2021	2011-2012	cross-sectional	database	IOTF	137	1295
Salas, et al (19)	Spain	2021	2015-2017	cross-sectional	database	WHO	2355	10239
Barja-Fernández, et al	Spain	2018	2018	cross-sectional	medical institution	IOTF	170	471
Marcos-Pasero, et al	Spain	2019	2017	longitudinal	school	IOTF	31	201
Baile, et al	Spain	2020	2020	cross-sectional	school	National Reference	54	1197
Bawaked, et al	Spain	2020	2003-2008	longitudinal	database	WHO	317	1480
Bazán, et al	Spain	2018	2011	cross-sectional	database	National Reference	443	3752
Bont, et al	Spain	2019	2012-2013	cross-sectional	database	WHO	702	2660
Tamayo-Ortiz, et al	Spain	2021	2012	cross-sectional	database	WHO	215	1233
Martín, et al	Spain	2008	2004-2006	cross-sectional	database	IOTF	311	1312
Llargues, et al	Spain	2011	2006	RCT	school	IOTF	94	508
Yngve, et al (8)	Spain	2008	2003	cross-sectional	database	IOTF	137	745
Riaño-Galán, et al	Spain	2017	2003-2008	cohort	community	IOTF	135	1044
Montero, et al	Spain	2005	2005	cross-sectional	school	IOTF	59	229
Janssen, et al (28)	Spain	2005	2001-2002	cross-sectional	database	IOTF	725	4445
Vázquez, et al	Spain	2010	2010	cross-sectional	school	IOTF	690	2305
Esteban-Vasallo, et al	Spain	2020	2012	cross-sectional	database	IOTF	271	2914
Martinez-Gomez, et al	Spain	2010	2007-2008	cross-sectional	community	IOTF	16	192
Bibiloni, et al	Spain	2010	2007-2008	cross-sectional	community	WHO	215	1231
Haug, et al (17)	Spain	2009	2005-2006	cross-sectional	database	IOTF	1100	7380
Larrañaga, et al	Spain	2007	2004-2005	cross-sectional	community	IOTF	270	1178
Ayala, et al	Spain	2007	2007	cross-sectional	community	CDC	28	154
Rojo, et al	Spain	2006	2003-2004	cross-sectional	database	National Reference	2787	35403
Morales-Suárez-Varela, et al	Spain	2015	2013-2014	cross-sectional	database	CDC	139	710
Alonso-Fernández, et al (1)	Spain	2015	2006	cross-sectional	database	IOTF	783	5108
Alonso-Fernández,	Spain	2015	2011-2012	cross-sectional	database	IOTF	501	3265

© 2024 Zhang X et al. *JAMA Pediatrics*.

et al (2)								
Weinmayr, et al (14)	Spain	2014	2004	cross-sectional	database	IOTF	289	968
Cerrillo, et al	Spain	2012	2012	cross-sectional	school	IOTF	219	990
Martínez-Vizcaíno , et al (1)	Spain	2012	2004	cross-sectional	school	IOTF	248	1119
Martínez-Vizcaíno , et al (2)	Spain	2012	2010	cross-sectional	school	IOTF	277	1070
Navalpotro, et al	Spain	2012	2006-2007	cross-sectional	database	IOTF	1210	4528
Jiménez-Ormeño, et al	Spain	2013	2013	cross-sectional	school	IOTF	250	1032
Laguna, et al	Spain	2013	2013	cross-sectional	database	IOTF	173	761
Morales, et al	Spain	2013	2010-2011	cross-sectional	school	IOTF	295	1158
Moreno, et al	Spain	2013	2008-2009	cross-sectional	database	CDC	401	2316
Pérez-Farinós, et al	Spain	2013	2010-2011	cross-sectional	database	IOTF	1853	7659
García-García, et al	Spain	2016	2012	cross-sectional	community	IOTF	282	1317
Alvarez Zallo ,et al	Spain	2017	2017	cross-sectional	database	IOTF	873	8607
Martín-Espinosa, et al	Spain	2017	2013	cross-sectional	school	IOTF	188	1604
Velde, et al (7)	Spain	2017	2010	cross-sectional	database	IOTF	219	1022
Ramos, et al	Spain	2018	2015	cross-sectional	school	WHO	60	235
Rathnayake, et al	Sri Lanka	2013	2013	cross-sectional	medical institution	WHO	67	1087
Warnakulas uriya, et al	Sri Lanka	2019	2019	cross-sectional	school	WHO	1322	12788
Salman, et al	Sudan	2010	2010	cross-sectional	school	CDC	41	304
Nagwa, et al	Sudan	2011	2011	cross-sectional	school	WHO	123	1138
Musaiger, et al (7)	Sudan	2016	2013-2014	cross-sectional	database	IOTF	67	902
Romano, et al (22)	Suriname	2022	2016	cross-sectional	database	WHO	256	1453
Ahrens, et al (5)	Sweden	2014	2007-2008	cross-sectional	database	IOTF	160	1824
Wallby, et al	Sweden	2017	2002-2007	cohort	database	IOTF	3421	30508
Önnestam, et al	Sweden	2022	2015	longitudinal	medical institution	IOTF	59	314
Fäldt, et al (1)	Sweden	2022	2015-2019	cross-sectional	database	National Reference	2613	23214
Fäldt, et al (2)	Sweden	2022	2020-2021	cross-sectional	database	National Reference	2271	19171
Sjöberg, et al	Sweden	2005	2004	cross-sectional	database	IOTF	645	4703
Norberg, et al	Sweden	2012	2004	cross-sectional	medical institution	IOTF	130	920
Nilsen, et al	Sweden	2017	2008-2010	cross-sectional	community	WHO	356	2620
Moraeus, et al	Sweden	2014	2008/2010/2013	cross-sectional	school	IOTF	650	3492
Garmy, et al	Sweden	2014	2008-2009	cross-sectional	school	IOTF	372	2891
Sjöberg, et al	Sweden	2008	2000-2005	cross-sectional	school	IOTF	1312	8876
Mårild, et al	Sweden	2004	2000	cross-sectional	community	IOTF	863	4730
Yngve, et al (9)	Sweden	2008	2003	cross-sectional	database	IOTF	110	888
Mangrio, et al	Sweden	2010	2003-2008	cross-sectional	medical institution	IOTF	1008	8621
White, et al (1)	Sweden	2022	2022	cohort	database	IOTF	410	3237
Janssen, et al (29)	Sweden	2005	2001-2002	cross-sectional	database	IOTF	344	3507

© 2024 Zhang X et al. *JAMA Pediatrics.*

Litsfeldt, et al	Sweden	2020	2015-2017	cross-sectional	school	IOTF	157	1363
Lager,et al	Sweden	2009	2003-2006	cross-sectional	school	IOTF	1349	7584
Haug, et al (38)	Sweden	2009	2005-2006	cross-sectional	database	IOTF	358	3974
Bergström, et al (1)	Sweden	2009	2002-2003	cross-sectional	medical institution	IOTF	869	4407
Blomquist, et al	Sweden	2007	2007	cross-sectional	medical institution	IOTF	852	4407
Wijnhoven, et al (5)	Sweden	2015	2007/2008	cross-sectional	school	WHO	843	3633
Moraeus, et al (1)	Sweden	2015	2008	cross-sectional	school	IOTF	125	833
Moraeus, et al (2)	Sweden	2015	2010	cross-sectional	school	IOTF	187	1085
Moraeus, et al (3)	Sweden	2015	2013	cross-sectional	school	IOTF	193	1134
Lindkvist, et al	Sweden	2015	2008-2012	cross-sectional	community	WHO	125	697
Weinmayr, et al (15)	Sweden	2014	2004	cross-sectional	database	IOTF	91	459
Vaezghasemi, et al	Sweden	2012	2007	cross-sectional	school	IOTF	656	4987
Khanolkar, et al	Sweden	2013	2013	cross-sectional	community	IOTF	1400	10628
Winkvist, et al	Sweden	2016	2008	cross-sectional	community	IOTF	1398	11222
Bergström, et al (2)	Sweden	2009	2007-2008	cross-sectional	medical institution	IOTF	726	4381
Aeberli, et al (1)	Switzerland	2010	2007	cross-sectional	school	CDC	244	2222
Aeberli, et al (2)	Switzerland	2010	2002	cross-sectional	school	CDC	309	2404
Aeberli, et al	Switzerland	2010	2009	cross-sectional	database	CDC	111	900
Jeannot, et al	Switzerland	2015	2011-2012	cross-sectional	school	IOTF	1178	8544
Lasserre, et al	Switzerland	2007	2005-2006	cross-sectional	school	IOTF	624	5207
Janssen, et al (30)	Switzerland	2005	2001-2002	cross-sectional	database	IOTF	318	4190
Zimmermann, et al	Switzerland	2004	2004	cross-sectional	school	CDC	471	2431
Köchli, et al	Switzerland	2019	2019	cross-sectional	database	IOTF	122	1246
Haug, et al (9)	Switzerland	2009	2005-2006	cross-sectional	database	IOTF	315	4251
Velde, et al (8)	Switzerland	2017	2010	cross-sectional	database	IOTF	70	596
Nasreddine, et al	Syria	2010	2010	cross-sectional	school	WHO	147	776
Musaiger, et al (4)	Syria	2012	2010-2011	cross-sectional	school	IOTF	214	1062
Yen, et al	Taiwan	2021	2019-2020	cross-sectional	school	WHO	105	569
Chen, et al	Taiwan	2012	2007/2010	cross-sectional	database	National Reference	2443	7930
Chu, et al	Taiwan	2007	2001-2002	cross-sectional	database	National Reference	361	2405
Liou, et al	Taiwan	2010	2006-2007	cross-sectional	school	IOTF	1387	8640
Ting, et al	Taiwan	2012	2010-2011	cross-sectional	school	National Reference	86	859
Tsai, et al	Taiwan	2009	2007	cross-sectional	school	CDC	232	1287
Chen, et al	Taiwan	2019	2010	cohort	database	National Reference	432	2804
Lai, et al	Taiwan	2001	2000	cross-sectional	school	National Reference	228	2011
Yen, et al	Taiwan	2010	2004	cross-sectional	database	IOTF	1572	10371
Pu, et al	Taiwan	2010	2003	cross-sectional	database	IOTF	333	1879
Chang, et al	Taiwan	2010	2010	cross-sectional	school	National Reference	291	2083
Chen, et al	Taiwan	2009	2009	cross-sectional	school	IOTF	153	1024
Chen, et al	Taiwan	2008	2008	cross-sectional	school	IOTF	168	866
Chang, et al	Taiwan	2015	2008-2012	cross-sectional	school	National Reference	118	838
Lee, et al	Taiwan	2014	2009	cross-sectional	school	IOTF	245	5254

© 2024 Zhang X et al. *JAMA Pediatrics.*

Hsieh, et al	Taiwan	2014	2010-2011	cross-sectional	school	IOTF	314	2419
Chen, et al	Taiwan	2014	2014	cross-sectional	medical institution	National Reference	18	121
Chiang, et al	Taiwan	2013	2001-2002	cross-sectional	database	National Reference	325	2283
Liao, et al	Taiwan	2013	2010-2011	cross-sectional	database	National Reference	1786	13500
Chen, et al	Taiwan	2016	2010-2011	cross-sectional	database	National Reference	226	1826
Salas, et al (20)	Tajikistan	2021	2015-2017	cross-sectional	database	WHO	175	2822
Mosha, et al	Tanzania	2021	2019	cross-sectional	school	WHO	105	1170
Mosha, et al	Tanzania	2010	2008	cross-sectional	community	National Reference	12	222
Mpembeni, et al	Tanzania	2014	2014	cross-sectional	school	National Reference	44	446
Mwaikambo, et al	Tanzania	2015	2015	cross-sectional	school	IOTF	175	1722
Pangani, et al	Tanzania	2016	2012	cross-sectional	school	WHO	283	1781
Gebremedhin, et al (22)	Tanzania	2015	2010	cross-sectional	database	WHO	313	6948
Chomba, et al	Tanzania	2019	2018	cross-sectional	community	WHO	57	451
Rerksuppaphol, et al	Thailand	2021	2015	cross-sectional	school	WHO	528	3870
Weraarchakul, et al	Thailand	2017	2015	cross-sectional	school	WHO	22	210
Nonboonyawat, et al	Thailand	2019	2016	cross-sectional	school	WHO	157	1749
Pawloski, et al	Thailand	2008	2008	cross-sectional	school	IOTF	44	410
Romano, et al (23)	Thailand	2022	2015	cross-sectional	database	WHO	558	4132
Manandhar, et al	Thailand	2019	2018	cross-sectional	school	WHO	58	440
Sengmeuang, et al	Thailand	2010	2007	cross-sectional	school	National Reference	672	7096
Rerksuppaphol, et al	Thailand	2010	2007	cross-sectional	school	IOTF	120	1140
Narksawat, et al	Thailand	2009	2009	cross-sectional	school	National Reference	46	862
Rerksuppaphol, et al	Thailand	2015	2013	cross-sectional	school	WHO	551	3991
Sukhonthachit, et al	Thailand	2014	2012	cross-sectional	school	CDC	105	693
Jitnarin, et al	Thailand	2011	2011	cross-sectional	database	IOTF	845	9287
Rojroongwasinkul, et al	Thailand	2013	2011	cross-sectional	community	WHO	238	3119
Gebremedhin, et al (23)	Togo	2015	2013/2014	cross-sectional	database	WHO	55	3228
Sagbo, et al	Togo	2018	2015	cross-sectional	school	IOTF	33	634
Romano, et al (24)	Tonga	2022	2017	cross-sectional	database	WHO	584	1946
Smith, et al	Tonga	2007	2000	cross-sectional	school	IOTF	161	445
Pengpid, et al (5)	Tonga	2015	2010-2011	cross-sectional	database	IOTF	831	2211
Romano, et al (25)	Trinidad and Tobago	2022	2017	cross-sectional	database	WHO	388	2363
Mumena, et al	Trinidad and Tobago	2018	2012-2014	prospective	school	WHO	45	336
Boukthir, et al	Tunisia	2011	2007	cross-sectional	school	IOTF	187	1335
Musaiger, et al (8)	Tunisia	2016	2013-2014	cross-sectional	database	IOTF	127	803
Ercan, et al	Turkey	2012	2010-2011	cross-sectional	school	National Reference	737	8848
Salman, et al	Turkey	2022	2014	cross-sectional	school	National Reference	117	1127
Meydanlioglu, et al	Turkey	2022	2017	cross-sectional	school	CDC	587	5160
Salas, et al (21)	Turkey	2021	2015-2017	cross-sectional	database	WHO	315	3658

© 2024 Zhang X et al. *JAMA Pediatrics*.

Gunalan, et al	Turkey	2021	2017	cross-sectional	school	WHO	223	1561
Arslan, et al	Turkey	2021	2016	cross-sectional	school	CDC	1650	9786
Acar Tek, et al	Turkey	2017	2017	cross-sectional	school	WHO	134	1111
Sağlam, et al	Turkey	2008	2008	cross-sectional	school	CDC	666	5368
Sur, et al	Turkey	2005	2001-2002	cross-sectional	school	IOTF	124	1044
Gundogdu, et al	Turkey	2008	2002	cross-sectional	medical institution	CDC	147	1899
Turkkahraman, et al	Turkey	2006	2002-2003	cross-sectional	school	IOTF	353	2465
Nur, et al	Turkey	2008	2004	cross-sectional	school	CDC	36	1020
Bayat, et al	Turkey	2009	2004	cross-sectional	school	CDC	44	610
Discigil, et al	Turkey	2009	2005	cross-sectional	school	CDC	164	1348
Etiler, et al	Turkey	2011	2011	cross-sectional	school	WHO	267	2281
Ozmen, et al	Turkey	2007	2007	cross-sectional	school	IOTF	189	2101
Simsek, et al	Turkey	2008	2005-2006	cross-sectional	school	IOTF	724	6925
Yuca, et al	Turkey	2010	2006-2007	cross-sectional	school	IOTF	1006	9048
Pirinçci, et al	Turkey	2010	2007	cross-sectional	school	IOTF	481	3642
Duzova, et al	Turkey	2013	2007-2008	cross-sectional	community	CDC	331	3571
Polat, et al	Turkey	2014	2012	cross-sectional	school	CDC	392	2826
Gökler, et al	Turkey	2015	2012	cross-sectional	school	WHO	458	3918
Agirbasli, et al	Turkey	2011	2008	cross-sectional	medical institution	IOTF	265	1746
Manios, et al	Turkey	2005	2001	cross-sectional	school	IOTF	54	510
Krassas, et al (2)	Turkey	2004	2004	cross-sectional	school	National Reference	393	3703
Yardim, et al	Turkey	2019	2015	cross-sectional	school	WHO	697	3291
Akbulut, et al (1)	Turkey	2014	2014	cross-sectional	school	IOTF	238	915
Canan, et al	Turkey	2014	2014	cross-sectional	school	National Reference	75	1938
Dündar, et al	Turkey	2012	2009	cross-sectional	school	CDC	554	2477
Uçkun-Kitapçi, et al	Turkey	2004	2004	cross-sectional	school	National Reference	173	1647
Oner, et al	Turkey	2004	2001	cross-sectional	community	IOTF	109	989
Ardıç, et al	Turkey	2019	2006-2010	cross-sectional	medical institution	National Reference	27	180
Aşut, et al	Turkey	2019	2017	cross-sectional	school	National Reference	55	459
Çelmeli, et al	Turkey	2019	2015	cross-sectional	community	IOTF	392	1687
Comba, et al	Turkey	2019	2017	cross-sectional	school	WHO	229	1684
Deniz, et al	Turkey	2019	2019	cross-sectional	school	WHO	206	1298
Arikan, et al	Turkey	2020	2020	cross-sectional	school	National Reference	799	10781
Ucar, et al	Turkey	2009	2001-2003	cross-sectional	school	IOTF	1150	11629
Haug, et al (18)	Turkey	2009	2005-2006	cross-sectional	database	IOTF	421	4680
Dinç, et al	Turkey	2009	2009	cross-sectional	school	CDC	145	1346
Önsüz, et al	Turkey	2015	2010	cross-sectional	school	WHO	179	2166
Meseri, et al	Turkey	2015	2012	cross-sectional	school	WHO	69	462
Inal, et al	Turkey	2015	2011	cross-sectional	school	CDC	136	531
Weinmayr, et al (16)	Turkey	2014	2004	cross-sectional	database	IOTF	52	342
Vehapoglu, et al	Turkey	2014	2012-2013	cross-sectional	medical institution	National Reference	654	4990
Cabar, et al	Turkey	2014	2010-2011	cross-sectional	school	National Reference	290	3352
Ayyildiz, et al	Turkey	2014	2010	cross-sectional	school	National Reference	161	868
Akca, et al	Turkey	2016	2011	cross-sectional	school	CDC	67	554
Geckil, et al	Turkey	2017	2012-2013	cross-sectional	school	National Reference	407	3028
Eker, et al	Turkey	2018	2011-2012	cross-sectional	school	WHO	171	1357
Haney, et al	Turkey	2018	2014-2015	cross-sectional	school	National Reference	164	1289
Salas, et al (22)	Turkmenistan	2021	2015-2017	cross-sectional	database	WHO	1514	9768

© 2024 Zhang X et al. *JAMA Pediatrics.*

Gebremedhin, et al (24)	Uganda	2015	2011	cross-sectional	database	WHO	198	4212
Dereń, et al	Ukraine	2020	2018-2019	cross-sectional	school	WHO	1833	18144
Dereń, et al	Ukraine	2018	2018	cross-sectional	school	WHO	1841	13739
Høyer, et al (3)	Ukraine	2014	2002-2006	cohort	database	WHO	66	492
Janssen, et al (31)	Ukraine	2005	2001-2002	cross-sectional	database	IOTF	200	3645
Haug, et al (26)	Ukraine	2009	2005-2006	cross-sectional	database	IOTF	332	4613
AlBlooshi, et al	United Arab Emirates	2016	2014-2015	cross-sectional	community	CDC	3407	27078
Abduelkarem, et al	United Arab Emirates	2020	2017	cross-sectional	school	CDC	96	684
Abdullatif, et al	United Arab Emirates	2022	2018-2019	cross-sectional	school	WHO	425	1683
Aburawi, et al	United Arab Emirates	2019	2019	cross-sectional	school	IOTF	153	967
Zaal, et al	United Arab Emirates	2009	2009	cross-sectional	school	WHO	104	661
Musaiger, et al (5)	United Arab Emirates	2012	2010-2011	cross-sectional	school	IOTF	77	505
Fatima, et al	United Arab Emirates	2018	2017-2018	cross-sectional	school	CDC	36	267
Adab, et al	United Kingdom of Great Britain and Northern Ireland	2018	2005	RCT	school	National Reference	171	1397
Gilliland, et al	United Kingdom of Great Britain and Northern Ireland	2012	2012	cross-sectional	school	WHO	163	966
Uerlich, et al	United Kingdom of Great Britain and Northern Ireland	2021	2014-2015	cross-sectional	database	WHO	33	171
Ralphs, et al	United Kingdom of Great Britain and Northern Ireland	2021	2007-2010	cross-sectional	database	National Reference	643	6410
Adab, et al	United Kingdom of Great Britain and Northern Ireland	2014	2006-2007	RCT	school	National Reference	42	571
Bartle, et al	United Kingdom of Great Britain and Northern Ireland	2013	2013	longitudinal	school	IOTF	54	400
Pearce, et al	United Kingdom of Great Britain and Northern Ireland	2010	2003-2005	cohort	database	IOTF	2407	13187
Harding, et al	United Kingdom of Great Britain and Northern Ireland	2008	2002-2003	cross-sectional	school	IOTF	1186	5684
Steele, et al	United Kingdom of	2009	2007	cross-sectional	school	IOTF	332	1862

© 2024 Zhang X et al. *JAMA Pediatrics.*

	Great Britain and Northern Ireland							
Basterfield, et al	United Kingdom of Great Britain and Northern Ireland	2014	2006-2007	cross-sectional	database	IOTF	75	425
Griffiths, et al	United Kingdom of Great Britain and Northern Ireland	2014	2005-2007	cross-sectional	database	National Reference	4360	13291
Clemente, et al	United Kingdom of Great Britain and Northern Ireland	2019	2019	cross-sectional	database	WHO	215	1396
Tiffin, et al	United Kingdom of Great Britain and Northern Ireland	2011	2007	cross-sectional	database	IOTF	760	3961
Harrison, et al	United Kingdom of Great Britain and Northern Ireland	2011	2011	cross-sectional	school	IOTF	289	1724
Taylor, et al	United Kingdom of Great Britain and Northern Ireland	2005	2001	cross-sectional	school	IOTF	682	2482
Janssen, et al (7)	United Kingdom of Great Britain and Northern Ireland	2005	2001-2002	cross-sectional	database	IOTF	479	3601
Janssen, et al (26)	United Kingdom of Great Britain and Northern Ireland	2005	2001-2002	cross-sectional	database	IOTF	279	2133
Janssen, et al (33)	United Kingdom of Great Britain and Northern Ireland	2005	2001-2002	cross-sectional	database	IOTF	529	3170
Cecil, et al	United Kingdom of Great Britain and Northern Ireland	2005	2002-2003	cross-sectional	school	IOTF	454	2454
Fletcher, et al	United Kingdom of Great Britain and Northern Ireland	2004	2000	cross-sectional	school	IOTF	128	424
Webster-Gandy, et al	United Kingdom of Great Britain and	2003	2003	cross-sectional	school	IOTF	14	188

© 2024 Zhang X et al. *JAMA Pediatrics.*

	Northern Ireland							
Warren, et al	United Kingdom of Great Britain and Northern Ireland	2003	2001	cross-sectional	school	IOTF	9	37
Whitaker, et al	United Kingdom of Great Britain and Northern Ireland	2010	2001-2006	cross-sectional	database	IOTF	1353	7078
Thomas, et al(1)	United Kingdom of Great Britain and Northern Ireland	2010	2002	cross-sectional	school	IOTF	21	71
Thomas, et al(2)	United Kingdom of Great Britain and Northern Ireland	2010	2007	cross-sectional	school	IOTF	17	83
Skidmore, et al	United Kingdom of Great Britain and Northern Ireland	2010	2007	cross-sectional	school	IOTF	306	1721
Jennings, et al	United Kingdom of Great Britain and Northern Ireland	2010	2010	cross-sectional	school	IOTF	16	84
Edwards, et al	United Kingdom of Great Britain and Northern Ireland	2010	2000-2006	cross-sectional	community	National Reference	3841	33594
Willianmson, et al(1)	United Kingdom of Great Britain and Northern Ireland	2009	2006	cross-sectional	school	CDC	479	2709
Willianmson, et al(2)	United Kingdom of Great Britain and Northern Ireland	2009	2006	cross-sectional	school	IOTF	606	2709
Standley, et al	United Kingdom of Great Britain and Northern Ireland	2009	2002	cross-sectional	school	IOTF	723	4167
Nelson, et al	United Kingdom of Great Britain and Northern Ireland	2009	2003-2005	cross-sectional	school	IOTF	716	4587
Haug, et al (28)	United Kingdom of Great Britain and Northern Ireland	2009	2005-2006	cross-sectional	database	IOTF	200	1961

© 2024 Zhang X et al. *JAMA Pediatrics.*

Haug, et al (37)	United Kingdom of Great Britain and Northern Ireland	2009	2005-2006	cross-sectional	database	IOTF	341	2600
Haug, et al (39)	United Kingdom of Great Britain and Northern Ireland	2009	2005-2006	cross-sectional	database	IOTF	436	2910
van Sluijs, et al	United Kingdom of Great Britain and Northern Ireland	2008	2007	cross-sectional	database	IOTF	359	2064
Harding, et al	United Kingdom of Great Britain and Northern Ireland	2008	2008	cross-sectional	database	IOTF	1172	5515
Routh, et al	United Kingdom of Great Britain and Northern Ireland	2006	2006	cross-sectional	school	IOTF	50	252
Mutunga, et al	United Kingdom of Great Britain and Northern Ireland	2006	2000	cross-sectional	database	National Reference	278	2016
Solmi, et al	United Kingdom of Great Britain and Northern Ireland	2015	2006-2012	cross-sectional	database	IOTF	4801	29001
Jackson, et al	United Kingdom of Great Britain and Northern Ireland	2015	2005-2012	cross-sectional	database	IOTF	981	4979
Falconer, et al	United Kingdom of Great Britain and Northern Ireland	2014	2010-2011	cross-sectional	database	National Reference	290	2737
Pallan, et al	United Kingdom of Great Britain and Northern Ireland	2011	2011	cross-sectional	database	National Reference	42	574
Coulthard, et al	United Kingdom of Great Britain and Northern Ireland	2016	2008-2012	cross-sectional	database	National Reference	221	1620
Beynon, et al	United Kingdom of Great Britain and Northern Ireland	2017	2008-2012	cross-sectional	database	National Reference	1310	11279
Hudda, et al	United Kingdom of	2018	2012-2013	cross-sectional	database	National Reference	145436	1068261

© 2024 Zhang X et al. *JAMA Pediatrics*.

	Great Britain and Northern Ireland							
Wang, et al	United States of America	2010	2005-2006	cross-sectional	database	CDC	1147	6939
Chiasson, et al	United States of America	2016	2008-2009	cohort	database	CDC	8550	50589
Ehrenthal, et al	United States of America	2016	2004-2011	cohort	database	CDC	368	2172
Taveras, et al	United States of America	2006	2006	cohort	database	CDC	182	1012
Vehrs, et al	United States of America	2022	2022	cross-sectional	community	CDC	41	332
Vazquez, et al	United States of America	2022	2019	cross-sectional	school	CDC	41	237
Leung, et al	United States of America	2011	2005-2006	cohort	database	CDC	50	353
Shier, et al	United States of America	2012	2004/2007	cross-sectional	database	CDC	2253	6260
Salcido, et al	United States of America	2022	2017-2018	cross-sectional	medical institution	CDC	1046	7270
Odusanya, et al	United States of America	2022	2009-2018	cross-sectional	database	CDC	331	3248
Bader, et al	United States of America	2013	2007-2008	cross-sectional	database	CDC	18778	113123
Burdette, et al	United States of America	2004	2004	cross-sectional	database	CDC	1455	6907
Carroll-Scott, et al	United States of America	2013	2009	cross-sectional	database	National Reference	190	996
Hunt, et al	United States of America	2022	2009-2013	cohort	database	CDC	131	816
Fyfe-Johnson, et al	United States of America	2022	2012	cross-sectional	database	CDC	3166	17115
Flórez, et al	United States of America	2022	2006-2017	cross-sectional	database	CDC	105584	570172
Davis, et al	United States of America	2009	2002-2005	cross-sectional	database	CDC	83111	529367
Salazar, et al	United States of America	2022	2018-2019	cross-sectional	medical institution	CDC	22	112
Bejarano, et al	United States of America	2022	2015-2016	cross-sectional	database	IOTF	56961	320005
Kepper, et al	United States of America	2016	2016	RCT	database	CDC	10	78
Stine, et al	United States of America	2021	2017	cross-sectional	database	CDC	744	3956
Reilly, et al (1)	United States of America	2021	2008-2009	cross-sectional	school	CDC	94	511
Reilly, et al (2)	United States of America	2021	2015-2016	cross-sectional	school	CDC	162	836
Liu, et al	United	2007	2000	cross-sectional	medical	CDC	1266	7334

© 2024 Zhang X et al. *JAMA Pediatrics.*

	States of America				institution			
Imoisili, et al	United States of America	2021	2012-2018	cross-sectional	database	National Reference	104091	759591
Oreskovic, et al	United States of America	2009	2006	cross-sectional	medical institution	National Reference	1106	6680
Sánchez, et al	United States of America	2012	2007	cross-sectional	database	CDC	379667	926018
Wasserman, et al	United States of America	2014	2008-2009	cross-sectional	school	CDC	4711	12090
Halfon, et al	United States of America	2013	2007	cross-sectional	database	CDC	6495	43297
Hurt, et al	United States of America	2014	2005-2010	cross-sectional	database	CDC	1227	7629
Sacheck, et al	United States of America	2011	2009	cross-sectional	school	CDC	52	263
Turer, et al	United States of America	2013	2003-2006	cross-sectional	database	CDC	2086	12292
Moore, et al	United States of America	2016	2005-2006	cross-sectional	database	National Reference	413	2482
Lee, etal	United States of America	2007	2007	longitudinal	database	CDC	49	309
Adachi-Mejia, et al	United States of America	2007	2002-2003	cross-sectional	school	CDC	478	2343
Adams, et al	United States of America	2010	2010	cross-sectional	community	CDC	84	421
Adams, et al	United States of America	2005	2001-2003	cross-sectional	school	CDC	70	366
Adams, et al	United States of America	2019	2013-2015	cross-sectional	database	National Reference	81	450
Adams, et al	United States of America	2013	2007-2009	cross-sectional	database	CDC	77112	495848
Davis, et al	United States of America	2011	2003-2006	cross-sectional	database	CDC	1232	7882
Lewis, et al	United States of America	2006	2002	cross-sectional	school	CDC	498	3114
Davis, et al	United States of America	2008	2008	cross-sectional	school	CDC	31	138
Agazzi, et al	United States of America	2010	2005	cross-sectional	school	CDC	100	535
DeBoer, et al	United States of America	2015	2001	longitudinal	database	CDC	1400	8950
Govindan, et al	United States of America	2013	2004-2011	cross-sectional	school	CDC	296	1714
Wojcicki, et al	United States of America	2011	2006-2007	cohort	medical institution	CDC	19	145
Beck, et al	United States of America	2014	2014	cross-sectional	community	CDC	20	319
Bai, et al	United States of America	2016	2012	cross-sectional	database	CDC	195	1114

© 2024 Zhang X et al. *JAMA Pediatrics.*

Giammattei	United States of America	2003	2000-2001	cross-sectional	school	CDC	69	385
Barlow, et al	United States of America	2007	2003	cross-sectional	database	CDC	8772	52845
Baxter, et al	United States of America	2013	2004-2007	cross-sectional	school	CDC	376	1504
Baxter, et al	United States of America	2011	2004-2007	cross-sectional	school	CDC	178	920
Geier, et al	United States of America	2007	2007	cross-sectional	school	National Reference	182	1069
Shore, et al	United States of America	2008	2004-2005	cross-sectional	school	CDC	85	549
Pan, et al	United States of America	2013	2009	cross-sectional	database	CDC	640	3470
Kim, et al	United States of America	2018	2002-2003	longitudinal	school	CDC	320	2318
Seicean, et al	United States of America	2007	2004	cross-sectional	school	CDC	62	509
Silva, et al	United States of America	2011	2004-2009	cohort	database	CDC	108	304
Fiorito, et al	United States of America	2006	2006	cross-sectional	community	CDC	53	177
Charvet, et al	United States of America	2019	2019	cross-sectional	medical institution	CDC	33	197
Haidar, et al	United States of America	2019	2009-2011	cross-sectional	database	CDC	1054	6716
Drake, et al	United States of America	2012	2002-2009	longitudinal	community	CDC	498	1718
Drake, et al	United States of America	2013	2007-2008	cohort	school	CDC	73	479
Pérez, et al (1)	United States of America	2015	2000-2002	cross-sectional	database	CDC	1555	8938
Pérez, et al (2)	United States of America	2015	2004-2005	cross-sectional	database	CDC	2641	15283
Ghosh-Dastidar, et al	United States of America	2016	2013	cross-sectional	database	CDC	89	475
Berkowitz, et al	United States of America	2005	2005	cohort	community	CDC	18	78
Hinkle, et al	United States of America	2012	2006	cohort	database	CDC	659	3600
Kubo, et al	United States of America	2016	2005-2012	cohort	database	CDC	60	417
Lindberg, et al	United States of America	2012	2012	cohort	database	CDC	83	471
Olson, et al	United States of America	2010	2009-2010	cohort	database	CDC	56	321
Wojcicki, et al	United States of America	2015	2005-2009	cohort	database	CDC	133	833
Han, et al	United States of	2020	2009-2016	longitudinal	school	CDC	198847	486178

© 2024 Zhang X et al. *JAMA Pediatrics*.

	America							
Jia, et al	United States of America	2019	2007	longitudinal	database	CDC	2673	7530
Elbel, et al	United States of America	2019	2013	cross-sectional	database	CDC	132040	735192
Reis, et al	United States of America	2020	2020	cross-sectional	school	CDC	61	171
Bailey, et al	United States of America	2014	2001-2013	cohort	medical institution	CDC	16030	89057
Harley, et al	United States of America	2013	2000-2010	cohort	database	CDC	66	319
Huh, et al	United States of America	2012	2002-2005	cohort	medical institution	CDC	213	1255
Wang, et al	United States of America	2013	2000-2004	cohort	database	CDC	312	917
Peck, et al	United States of America	2015	2011	cohort	database	CDC	3707	24137
Kjaer, et al	United States of America	2018	2015-2016	cohort	medical institution	CDC	17	102
DuBose, et al	United States of America	2006	2006	cross-sectional	database	CDC	79	375
Heer, et al	United States of America	2013	2008	cross-sectional	school	CDC	141	653
Block, et al	United States of America	2018	2009-2016	cross-sectional	medical institution	CDC	52774	362550
Boutelle, et al	United States of America	2010	2010	cross-sectional	community	CDC	61	495
Kubzansky, et al	United States of America	2012	2001-2005	cross-sectional	school	CDC	561	1528
McClure, et al	United States of America	2010	2003	cross-sectional	community	CDC	692	4458
Roberts, et al	United States of America	2013	2000	cross-sectional	database	CDC	689	4175
Vannucci, et al	United States of America	2017	2007/2009	cross-sectional	school	CDC	75	368
Adams, et al	United States of America	2005	2000-2004	cohort	database	CDC	47	252
Vohr, et al	United States of America	2018	2018	cohort	database	CDC	47	388
Wood, et al	United States of America	2018	2012-2014	cohort	database	CDC	107	871
Cottrell, et al	United States of America	2011	2007-2008	cross-sectional	database	CDC	2981	17944
Vangeepuram, et al	United States of America	2011	2004-2007	cross-sectional	database	CDC	78	503
Kwon, et al	United States of America	2006	2002-2004	cross-sectional	database	CDC	139	853
Anderson, et al	United States of America	2014	2007-2011	cohort	database	CDC	1896	15141

© 2024 Zhang X et al. *JAMA Pediatrics*.

Taveras, et al	United States of America	2014	2014	cohort	medical institution	CDC	144	1046
Scharf, et al	United States of America	2015	2001-2006	cohort	database	CDC	1423	8950
Storfer-Isser, et al	United States of America	2012	2002-2006	cohort	database	IOTF	63	313
Bell, et al	United States of America	2010	2002	cohort	database	CDC	370	1920
Roberts, et al	United States of America	2013	2000-2001	cross-sectional	school	CDC	760	4175
Lohman, et al	United States of America	2009	2009	cross-sectional	database	CDC	273	1011
Foster, et al	United States of America	2008	2008	RCT	school	CDC	228	1349
Chomitz, et al	United States of America	2010	2003-2004	cohort	school	CDC	312	1858
Shriver, et al	United States of America	2011	2008	cross-sectional	school	CDC	39	237
Lee, et al	United States of America	2010	2002	longitudinal	database	National Reference	58	401
Kunin-Batson, et al	United States of America	2023	2012-2014	longitudinal	database	CDC	137	534
Wu, et al	United States of America	2022	2018	longitudinal	database	CDC	7365	46151
White, et al (3)	United States of America	2022	2022	cohort	database	IOTF	556	2545
Arcan, et al	United States of America	2012	2005-2006	cross-sectional	school	CDC	61	413
Armstrong, et al	United States of America	2012	2008-2011	cross-sectional	medical institution	CDC	10	94
Rappaport, et al	United States of America	2005	2002	cross-sectional	community	CDC	944	2621
Janssen, et al (32)	United States of America	2005	2001-2002	cross-sectional	database	IOTF	814	4447
Flynn, et al	United States of America	2005	2003	cross-sectional	school	CDC	1071	7048
Thorpe, et al	United States of America	2004	2003	cross-sectional	school	CDC	483	2681
Patrick, et al	United States of America	2004	2001-2002	cross-sectional	medical institution	CDC	159	878
Nelson, et al	United States of America	2004	2001	cross-sectional	database	CDC	97	556
Drobac, et al	United States of America	2004	2001-2002	cross-sectional	medical institution	National Reference	219	997
Davy, et al	United States of America	2004	2004	cross-sectional	school	CDC	45	205
Jiménez-Cruz, et al	United States of America	2003	2001-2002	cross-sectional	school	CDC	228	1200
Friedlander, et al	United States of	2003	2003	cross-sectional	community	CDC	74	371

© 2024 Zhang X et al. *JAMA Pediatrics*.

	America							
Eisenmann, et al	United States of America	2003	2003	cross-sectional	school	IOTF	60	263
Demerath, et al	United States of America	2003	2000	cross-sectional	school	CDC	236	1338
Rinderknecht, et al	United States of America	2002	2000	cross-sectional	community	CDC	34	155
Yang, et al	United States of America	2018	2014-2015	cross-sectional	school	CDC	6605	41283
Hidalgo-Mendez, et al	United States of America	2019	2019	cross-sectional	medical institution	CDC	39	186
Thundiyil, et al	United States of America	2010	2007	cross-sectional	medical institution	CDC	18	179
Amram, et al	United States of America	2020	2020	cross-sectional	school	CDC	1625	10327
Strickman-Stein, et al	United States of America	2010	2004-2006	cross-sectional	medical institution	CDC	37	200
Shankaran, et al	United States of America	2010	2010	cross-sectional	community	CDC	140	880
Shabbir, et al	United States of America	2010	2006-2007	cross-sectional	school	CDC	130013	670352
Rodriguez, et al	United States of America	2010	2007	cross-sectional	school	CDC	385	2038
Meininger, et al	United States of America	2010	2006	cross-sectional	school	CDC	191	1070
Farhat, et al	United States of America	2010	2005-2006	cross-sectional	school	CDC	1343	7737
Elder, et al	United States of America	2010	2003-2004	cross-sectional	community	CDC	126	745
Dammann, et al	United States of America	2010	2010	cross-sectional	community	CDC	17	92
Bethell, et al	United States of America	2010	2007	cross-sectional	database	CDC	6747	44101
Beets, et al	United States of America	2010	2006	cross-sectional	database	CDC	891	6603
Bayles, et al	United States of America	2010	2010	cross-sectional	community	CDC	8	3699
Baranowski, et al	United States of America	2010	2010	cross-sectional	school	CDC	275	1551
Babey, et al(1)	United States of America	2010	2001	cross-sectional	database	CDC	943	5858
Babey, et al(2)	United States of America	2010	2003	cross-sectional	database	CDC	658	4010
Babey, et al(3)	United States of America	2010	2005	cross-sectional	database	CDC	645	4029
Babey, et al(4)	United States of America	2010	2007	cross-sectional	database	CDC	524	3638
Villa-Caballero, et al	United States of America	2009	2003-2004	cross-sectional	community	CDC	18	725

© 2024 Zhang X et al. *JAMA Pediatrics*.

Valente, et al	United States of America	2009	2009	cross-sectional	school	CDC	98	562
Vader, et al	United States of America	2009	2000-2002	cross-sectional	school	CDC	2089	11594
Treuth, et al	United States of America	2009	2003-2005	longitudinal	school	CDC	543	3085
Trent, et al	United States of America	2009	2003-2004	cross-sectional	database	CDC	413	2630
Smith, et al	United States of America	2009	2005-2007	cross-sectional	school	CDC	33	251
Santos, et al	United States of America	2009	2006	cross-sectional	school	WHO	355	1195
Rundle, et al	United States of America	2009	2003-2006	cross-sectional	school	CDC	94	437
Nsiah-Kumi, et al	United States of America	2009	2003	cross-sectional	medical institution	National Reference	57	336
Montgomery-Reagan, et al	United States of America	2009	2006-2007	cross-sectional	school	CDC	902	5306
Martyn-Nemeth, et al	United States of America	2009	2005	cross-sectional	school	CDC	20	102
Lim, et al	United States of America	2009	2002-2003	longitudinal	school	CDC	50	365
Krukowski, et al	United States of America	2009	2003	cross-sectional	school	CDC	199	1071
Hillman, et al	United States of America	2009	2004	cross-sectional	medical institution	CDC	68	397
Harnack, et al	United States of America	2009	2004-2006	cross-sectional	school	CDC	151	593
Harbaugh, et al	United States of America	2009	2005-2009	cross-sectional	school	CDC	224	1250
Franzini, et al	United States of America	2009	2003	cross-sectional	community	CDC	104	650
Dorsey, et al	United States of America	2009	2005	cross-sectional	medical institution	CDC	14	75
De La O, et al	United States of America	2009	2009	cross-sectional	school	CDC	53	576
West, et al	United States of America	2008	2003-2004	cross-sectional	school	CDC	248	1551
Steele, et al	United States of America	2008	2001-2004	cross-sectional	medical institution	CDC	543	3221
Pollack, et al	United States of America	2008	2000-2006	cross-sectional	database	CDC	584	3232
Laurson, et al	United States of America	2008	2001	longitudinal	community	IOTF	44	268
Kapoor, et al	United States of America	2008	2008	cross-sectional	school	CDC	184	1058
Kalich, et al	United States of America	2008	2003	cross-sectional	school	CDC	133	786
Irigoyen, et al	United States of	2008	2004-2005	cross-sectional	medical institution	CDC	228	1713

© 2024 Zhang X et al. *JAMA Pediatrics.*

	America							
Eisenmann, et al	United States of America	2008	2001	cross-sectional	database	IOTF	2854	12464
Eichner, et al	United States of America	2008	2002-2003	cross-sectional	school	IOTF	419	1706
Wald, et al	United States of America	2007	2004	cross-sectional	medical institution	CDC	92	612
Roseman, et al	United States of America	2007	2004	cross-sectional	school	CDC	648	4049
Metallinos-Katsaras, et al	United States of America	2007	2000	cross-sectional	medical institution	CDC	8	56
Martin, et al	United States of America	2007	2003-2004	cross-sectional	community	CDC	30	212
Lynch, et al	United States of America	2007	2002-2003	cross-sectional	school	CDC	438	2055
Hernández-Valero, et al	United States of America	2007	2001-2003	cross-sectional	community	CDC	82	438
Eisenmann, et al	United States of America	2007	2007	cross-sectional	school	IOTF	210	813
Akridge, et al	United States of America	2007	2004	cross-sectional	medical institution	IOTF	17	107
Zephier, et al	United States of America	2006	2002-2003	cross-sectional	school	CDC	2103	10821
Nelson, et al	United States of America	2006	2003	cross-sectional	database	CDC	78	526
Pobutsky, et al	United States of America	2006	2002-2003	cross-sectional	school	CDC	1438	10199
Trapp, et al	United States of America	2015	2010-2011	cross-sectional	medical institution	CDC	41	222
Nobari, et al	United States of America	2015	2011	cross-sectional	database	National Reference	293	2051
Nagata, et al	United States of America	2015	2006-2007	cross-sectional	medical institution	CDC	20	174
Lumeng, et al	United States of America	2015	2005-2013	longitudinal	medical institution	CDC	6277	43748
Jackson, et al	United States of America	2015	2012-2014	cross-sectional	database	CDC	12	102
Gunter, et al	United States of America	2015	2013	cross-sectional	school	CDC	277	1482
Ansari, et al	United States of America	2015	2006	prospective	school	CDC	523	2810
Nichols, et al (1)	United States of America	2014	2003	cross-sectional	database	National Reference	74	415
Nichols, et al (2)	United States of America	2014	2004	cross-sectional	database	National Reference	72	414
Nichols, et al (3)	United States of America	2014	2005	cross-sectional	database	National Reference	67	358
Nichols, et al (4)	United States of America	2014	2006	cross-sectional	database	National Reference	39	179

© 2024 Zhang X et al. *JAMA Pediatrics.*

Nichols, et al (5)	United States of America	2014	2007	cross-sectional	database	National Reference	46	220
Nichols, et al (6)	United States of America	2014	2008	cross-sectional	database	National Reference	33	182
Nichols, et al (7)	United States of America	2014	2009	cross-sectional	database	National Reference	32	234
Nichols, et al (8)	United States of America	2014	2010	cross-sectional	database	National Reference	41	278
Nichols, et al (9)	United States of America	2014	2011	cross-sectional	database	National Reference	32	183
Nguyen, et al	United States of America	2014	2010	cross-sectional	medical institution	CDC	129	691
Lo, et al	United States of America	2014	2007-2010	cross-sectional	database	CDC	6265	42559
Cui, et al	United States of America	2014	2001-2010	cross-sectional	database	CDC	1150	7031
Dammann, et al	United States of America	2011	2011	cross-sectional	community	CDC	43	257
Florin, et al	United States of America	2011	2003	cross-sectional	database	CDC	2081	11012
Hill, et al	United States of America	2011	2005	cross-sectional	school	CDC	115	649
Isasi, et al	United States of America	2011	2008	cross-sectional	school	CDC	345	1607
Mulasi-Pokhriyal, et al	United States of America	2011	2011	cross-sectional	community	CDC	54	335
Nervik, et al	United States of America	2011	2009	cross-sectional	medical institution	CDC	8	50
Pérez, et al	United States of America	2011	2004-2005	cross-sectional	school	CDC	4151	23190
Spruyt, et al	United States of America	2011	2011	cross-sectional	community	CDC	53	308
Xanthopoulos, et al	United States of America	2011	2011	cross-sectional	school	CDC	202	1212
Gamble, et al	United States of America	2012	2009-2010	cross-sectional	community	CDC	208	1136
Weedn, et al	United States of America	2012	2009	cross-sectional	database	CDC	6667	39151
Dodd, et al	United States of America	2013	2004-2005	cross-sectional	school	CDC	375	2314
Loth, et al	United States of America	2013	2010	cross-sectional	database	CDC	406	2231
Novotny, et al	United States of America	2013	2010	cross-sectional	medical institution	CDC	593	4599
Nunez-Gaunaurd, et al	United States of America	2013	2010-2011	cross-sectional	school	CDC	20	86
Novotny, et al	United States of America	2016	2011-2013	cross-sectional	community	CDC	800	5558
Stiefel, et al	United States of	2016	2009-2013	cross-sectional	medical institution	CDC	1564	7705

© 2024 Zhang X et al. *JAMA Pediatrics*.

	America							
Zeller, et al	United States of America	2016	2008-2009	cross-sectional	school	CDC	3011	19678
Sadeghi, et al	United States of America	2017	2013	cross-sectional	community	CDC	117	609
Pengpid, et al (6)	Vanuatu	2015	2010-2011	cross-sectional	database	IOTF	129	1119
Le, et al	Vietnam	2022	2021	cross-sectional	school	WHO	169	782
Dieu, et al	Vietnam	2007	2005	cross-sectional	school	IOTF	137	670
Carrillo-Larco, et al (3)	Vietnam	2014	2014	cohort	database	IOTF	117	1910
Phan, et al	Vietnam	2020	2018	cross-sectional	community	WHO	484	2788
Pham, et al	Vietnam	2019	2014	cross-sectional	community	WHO	140	821
Trang, et al	Vietnam	2010	2004	cross-sectional	school	IOTF	312	2660
Mai, et al	Vietnam	2020	2014-2015	cross-sectional	school	IOTF	2612	10949
Pham, et al	Vietnam	2020	2016	cross-sectional	school	IOTF	828	2764
Tang, et al	Vietnam	2007	2002	cross-sectional	school	IOTF	75	1504
Hong, et al (1)	Vietnam	2007	2002	cross-sectional	school	IOTF	67	1003
Hong, et al (2)	Vietnam	2007	2004	cross-sectional	school	IOTF	314	2684
Nguyen, et al	Vietnam	2013	2010	cross-sectional	database	WHO	354	1989
Ngan, et al	Vietnam	2018	2012	cross-sectional	school	WHO	31	276
Gebremedhin, et al (25)	Zambia	2015	2013/2014	cross-sectional	database	WHO	631	11677
Gebremedhin, et al (26)	Zimbabwe	2015	2010/2011	cross-sectional	database	WHO	242	4405
Kambondo, et al	Zimbabwe	2018	2015	cross-sectional	school	IOTF	59	974

eTable 5. Characteristics of the studies for the prevalence of excess weight in children and adolescents.

Study	Country or Region	Publication Year	Study Period	Study Design	Sample Source	Diagnostic Reference	No. of Excess Weight	Sample Size
Salas, et al (1)	Albania	2021	2015-2017	cross-sectional	database	WHO	563	2259
Hyska, et al	Albania	2014	2013	cross-sectional	school	WHO	882	5810
Benmohammed, et al	Algeria	2020	2007	cross-sectional	school	IOTF	230	1100
Musaiger, et al (1)	Algeria	2012	2010-2011	cross-sectional	school	IOTF	79	459
Fedala, et al	Algeria	2017	2013-2014	cross-sectional	school	IOTF	208	2278
Orden, et al	Argentina	2019	2015-2016	cross-sectional	school	IOTF	444	1366
Meyer, et al	Argentina	2013	2010-2011	cross-sectional	database	IOTF	4232	15541
Moraes, et al (2)	Argentina	2014	2008	cross-sectional	school	CDC	26	933
Hirschler, et al	Argentina	2010	2005	cross-sectional	school	IOTF	520	1564
Stray-Pedersen,et al	Argentina	2009	2004-2005	cross-sectional	database	CDC	126	669
Hirschler, et al	Argentina	2008	2006	cross-sectional	school	CDC	203	621
Hirschler, et al	Argentina	2008	2006-2007	cross-sectional	community	CDC	333	1027
Hirschler, et al	Argentina	2006	2004	cross-sectional	school	CDC	120	321
Hirschler, et al	Argentina	2015	2014	cross-sectional	school	CDC	80	271
Kovalskys, et al	Argentina	2011	2005	cross-sectional	school	National Reference	441	1588
Tringler, et al	Argentina	2012	2007-2008	cross-sectional	database	WHO	78	334
Catalani, et al	Argentina	2016	2014	cross-sectional	school	WHO	288	711
Gotthelf, et	Argentina	2017	2015	cross-sectional	school	IOTF	130	283

© 2024 Zhang X et al. *JAMA Pediatrics.*

al								
Rivero, et al	Argentina	2018	2017	cross-sectional	community	IOTF	124	303
Abbott, et al	Australia	2010	2006	cross-sectional	database	CDC	624	3043
Gibson, et al	Australia	2007	2004-2005	cross-sectional	database	IOTF	181	1080
Spinks, et al	Australia	2007	2001-2003	cross-sectional	community	IOTF	104	518
Crawford, et al	Australia	2008	2004	cross-sectional	community	IOTF	108	380
Franklin, et al	Australia	2006	2006	cross-sectional	community	IOTF	585	2743
Wolfenden, et al	Australia	2011	2007	cross-sectional	community	IOTF	128	764
Cauwenberghe, et al	Australia	2012	2008-2009	cohort	community	IOTF	122	703
Wake, et al	Australia	2013	2000-2006	cross-sectional	community	IOTF	3494	16339
James, et al	Australia	2013	2001-2005	cross-sectional	community	IOTF	2734	17008
Marshall, et al	Australia	2012	2005	cross-sectional	community	IOTF	194	691
Martin, et al	Australia	2012	2005	cross-sectional	community	IOTF	106	408
O'Dea, et al (1)	Australia	2010	2000	cross-sectional	community	IOTF	820	3819
O'Dea, et al (2)	Australia	2010	2006	cross-sectional	community	IOTF	1386	5524
O'Dea, et al	Australia	2014	2007	longitudinal	school	WHO	193	939
Schofield, et al	Australia	2009	2002	cross-sectional	school	IOTF	119	415
Gopinath, et al	Australia	2012	2003-2005	cross-sectional	school	WHO	1001	4094
Spurrier, et al	Australia	2012	2009	cross-sectional	school	CDC	2202	11859
Trapp, et al	Australia	2011	2007	cross-sectional	database	IOTF	274	1197
Waters, et al	Australia	2008	2004-2005	cross-sectional	school	IOTF	822	2685
Bell, et al	Australia	2018	2013-2014	cohort	database	IOTF	110	953
Wen, et al	Australia	2014	2007-2010	longitudinal	database	WHO	33	234
Hoare, et al	Australia	2019	2016	cross-sectional	school	CDC	219	809
Maher, et al	Australia	2012	2012	cross-sectional	database	CDC	561	2200
Hayes, et al	Australia	2021	2004	cohort	database	CDC	3052	9225
Miller, et al	Australia	2014	2005-2010	cross-sectional	community	IOTF	410	1850
Achat, et al	Australia	2014	2007	cross-sectional	school	IOTF	583	2341
O'Dea, et al	Australia	2008	2006	cross-sectional	school	IOTF	1935	7889
O'Sullivan, et al	Australia	2015	2003-2005	cross-sectional	database	CDC	359	1416
Barnes, et al	Australia	2021	2017	RCT	school	IOTF	229	815
Magee, et al	Australia	2013	2006	longitudinal	database	IOTF	334	1833
Cox, et al	Australia	2012	2010	cross-sectional	community	IOTF	20	135
Wickramasinghe, et al	Australia	2005	2005	cross-sectional	community	IOTF	27	138
Telford, et al	Australia	2008	2007	cross-sectional	database	IOTF	199	741
Seach, et al	Australia	2010	2000-2004	cohort	medical institution	IOTF	85	307
Mallan,et al	Australia	2017	2008-2009	RCT	medical institution	IOTF	3	100
Jansen, et al	Australia	2013	2010	cross-sectional	database	CDC	867	3197
Tai, et al	Australia	2009	2006	cross-sectional	school	IOTF	281	1457
Hoare, et al	Australia	2014	2012	cross-sectional	database	IOTF	213	800
Bergmeier, et al	Australia	2014	2009-2011	longitudinal	community	IOTF	26	201
Hardy, et al	Australia	2012	2010	cross-sectional	database	IOTF	213	1141
Waston, et al	Australia	2023	2008-2009	longitudinal	school	IOTF	75	476
White, et al (2)	Australia	2022	2022	cohort	database	IOTF	926	3998
Au, et al	Australia	2012	2004-2009	cohort	database	IOTF	890	4335
Aurangzeb, et al	Australia	2012	2006	cross-sectional	medical institution	IOTF	27	157
Sanigorski, et al	Australia	2005	2003-2004	cross-sectional	school	IOTF	455	1681
Rehor, et al	Australia	2002	2001	cross-sectional	school	IOTF	79	329
Wen, et al	Australia	2010	2006	cross-sectional	school	WHO	203	964
Scott, et al	Australia	2019	2014	cross-sectional	school	WHO	898	2407
Shi, et al	Australia	2010	2004-2008	cross-sectional	database	National Reference	260	3495
Johns, et al	Australia	2010	2008	cross-sectional	database	National	385	1414

© 2024 Zhang X et al. *JAMA Pediatrics*.

						Reference		
MacFarlane, et al	Australia	2009	2002-2006	longitudinal	database	IOTF	58	293
Johns, et al	Australia	2009	2004	cross-sectional	database	IOTF	27	138
Haysom, et al	Australia	2009	2003-2004	longitudinal	school	National Reference	145	2266
Haug, et al (2)	Australia	2009	2005-2006	cross-sectional	database	WHO	519	4509
Ducher, et al	Australia	2009	2005	cross-sectional	school	WHO	93	698
Sutherland, et al	Australia	2008	2004	cross-sectional	school	IOTF	616	2224
Denney-Wilson, et al	Australia	2008	2004	cross-sectional	database	IOTF	119	496
Fisher, er al	Australia	2006	2002	cross-sectional	school	WHO	52	296
Cretikos, et al	Australia	2008	2002-2006	cross-sectional	database	CDC	3838	12925
Cleland, et al	Australia	2008	2001-2004	longitudinal	school	National Reference	125	529
Crawford, et al	Australia	2006	2001	cross-sectional	school	WHO	103	1141
Campbell, et al	Australia	2006	2002	cross-sectional	database	CDC	63	324
Burke, et al	Australia	2006	2006	cross-sectional	school	WHO	126	570
Sanigorski, et al	Australia	2007	2003-2004	cross-sectional	community	IOTF	588	2184
McLean, et al	Australia	2007	2005	cross-sectional	medical institution	WHO	12	102
Leech, et al	Australia	2015	2002-2003	longitudinal	school	WHO	26	123
Katzmarzyk, et al (2)	Australia	2015	2011-2013	cross-sectional	database	IOTF	50	491
Cochrane, et al	Australia	2015	2000-2011	cross-sectional	school	IOTF	7747	31424
O'Dea, et al (1)	Australia	2014	2006	cross-sectional	database	WHO	576	8702
O'Dea, et al (2)	Australia	2014	2012	cross-sectional	database	IOTF	805	12587
Chen, et al (1)	Australia	2014	2010-2011	cross-sectional	community	IOTF	7	89
Keating, et al	Australia	2011	2005-2006	cross-sectional	database	IOTF	764	2890
Olds, et al	Australia	2011	2007	cross-sectional	database	IOTF	561	2200
Morley, et al	Australia	2012	2009-2010	cross-sectional	school	IOTF	2852	12188
Schultz, et al	Australia	2012	2012	cross-sectional	database	IOTF	191	996
Fredrickson, et al	Australia	2013	2005-2008	cross-sectional	database	IOTF	928	2954
Ruedl, et al	Australia	2016	2014	cross-sectional	school	IOTF	61	304
Furthner, et al	Australia	2017	2012-2013	cross-sectional	school	IOTF	658	2916
Mihrshahi, et al	Australia	2017	2015	cross-sectional	database	IOTF	1823	7555
Furthner, et al	Austria	2018	2012-2013	cross-sectional	school	IOTF	659	2930
Yngve, et al (1)	Austria	2008	2003	cross-sectional	database	IOTF	214	1181
Janssen, et al (1)	Austria	2005	2001-2002	cross-sectional	database	WHO	511	3994
Pfeiffer, et al	Austria	2006	2006	cross-sectional	school	WHO	100	835
Romano, et al (1)	Bahamas	2022	2013	cross-sectional	database	CDC	598	1308
Musaiger, et al	Bahrain	2014	2006-2007	cross-sectional	school	WHO	247	735
Al-Raees, et al	Bahrain	2009	2009	cross-sectional	community	WHO	115	698
Al-Sendi, et al	Bahrain	2003	2000	cross-sectional	school	IOTF	184	506
Musaiger, et al	Bahrain	2014	2003-2005	cross-sectional	school	IOTF	456	2146
Anam, et al	Bangladesh	2022	2019	cross-sectional	school	CDC	261	1044
Taher, et al	Bangladesh	2021	2016-2018	cross-sectional	school	CDC	524	1450
Sultana, et al	Bangladesh	2015	2015	cross-sectional	school	WHO	96	150
Sultana, et	Bangladesh	2016	2016	cross-sectional	school	IOTF	193	500

© 2024 Zhang X et al. *JAMA Pediatrics*.

al								
Saha, et al	Bangladesh	2018	2014	cross-sectional	school	IOTF	41	288
Romano, et al (2)	Bangladesh	2022	2014	cross-sectional	database	WHO	259	2753
Islam, et al	Bangladesh	2019	2015-2016	cross-sectional	school	WHO	146	976
Sultana, et al	Bangladesh	2019	2012-2013	cross-sectional	school	WHO	196	1768
Khan, et al	Bangladesh	2020	2019	cross-sectional	database	WHO	449	12497
Bulbul, et al	Bangladesh	2014	2009	cross-sectional	school	WHO	1336	10135
Romano, et al (3)	Barbados	2022	2011	cross-sectional	database	WHO	480	1504
Brug, et al (1)	Belgium	2012	2010	cross-sectional	database	WHO	151	1003
Ahrens, et al (4)	Belgium	2014	2007-2008	cross-sectional	database	WHO	223	2352
Usheva, et al (1)	Belgium	2021	2012	cross-sectional	database	National Reference	61	1128
Júlíusson, et al (1)	Belgium	2015	2002-2006	cross-sectional	database	WHO	1569	12200
Yngve, et al (2)	Belgium	2008	2003	cross-sectional	database	WHO	101	965
Seghers, et al	Belgium	2010	2006-2007	cross-sectional	school	WHO	122	798
Vriendt, et al	Belgium	2009	2004-2005	cross-sectional	school	WHO	150	982
De Coen, et al	Belgium	2014	2008-2009	longitudinal	school	WHO	106	568
Janssen, et al (2)	Belgium	2005	2001-2002	cross-sectional	database	WHO	519	5876
Janssen, et al (3)	Belgium	2005	2001-2002	cross-sectional	database	CDC	365	3066
Gysel, et al	Belgium	2009	2004-2005	cross-sectional	database	IOTF	344	1576
Haug, et al (3)	Belgium	2009	2005-2006	cross-sectional	database	WHO	368	3966
Haug, et al (4)	Belgium	2009	2005-2006	cross-sectional	database	IOTF	382	3267
Deforche, et al	Belgium	2009	2006-2007	cross-sectional	school	WHO	59	120
Vissers, et al	Belgium	2008	2008	cross-sectional	school	CDC	128	994
Huybrechts, et al	Belgium	2006	2006	cross-sectional	school	IOTF	37	297
Velde, et al (1)	Belgium	2017	2010	cross-sectional	database	IOTF	151	996
Manyanga, et al (1)	Benin	2014	2006/2010	cross-sectional	database	IOTF	300	2681
Romano, et al (4)	Benin	2022	2016	cross-sectional	database	WHO	109	717
Norbu, et al	Bhutan	2019	2019	cross-sectional	school	National Reference	140	392
Botti, et al	Bolivia	2010	2007	cross-sectional	database	WHO	764	3306
Romano, et al (5)	Bolivia	2022	2012	cross-sectional	database	WHO	617	2804
Pe ́rez-Cueto, et al	Bolivia	2005	2003	cross-sectional	school	WHO	116	525
Benefice, et al	Bolivia	2007	2004-2005	cross-sectional	community	IOTF	51	385
Spahić, et al	Bosnia and Herzegovina	2019	2016-2017	cross-sectional	school	WHO	730	2500
Hansanbegović, et al	Bosnia and Herzegovina	2010	2008-2009	cross-sectional	school	IOTF	676	3608
Ramic, et al	Bosnia and Herzegovina	2009	2009	cross-sectional	school	WHO	117	530
Wrotniak, et al	Botswana	2012	2012	cross-sectional	school	WHO	122	707
Alexius, et al	Brazil	2012	2007	cross-sectional	community	WHO	160	1048
Andaki, et al (1)	Brazil	2017	2009-2011	cross-sectional	community	WHO	422	2423
Ataíde Lima, et al	Brazil	2015	2008-2010	cross-sectional	community	WHO	64	203
Castilho, et al	Brazil	2014	2010-2012	cross-sectional	community	WHO	1094	3130
Costa, et al	Brazil	2015	2012-2013	cross-sectional	community	CDC	561	1530
Ferreira, et al	Brazil	2015	2012-2013	cross-sectional	community	WHO	179	1338

© 2024 Zhang X et al. *JAMA Pediatrics*.

Fraiz, et al	Brazil	2019	2019	cross-sectional	community	WHO	189	686
Justo, et al	Brazil	2012	2009-2010	cross-sectional	community	WHO	45	901
Guedes, et al	Brazil	2011	2007	cross-sectional	community	IOTF	607	5100
Kupek, et al	Brazil	2016	2007	cross-sectional	community	IOTF	423	1232
Moreira, et al	Brazil	2012	2007	cross-sectional	community	IOTF	83	963
Nobre, et al	Brazil	2013	2009-2010	cross-sectional	community	IOTF	40	232
Oppitz, et al	Brazil	2014	2008	cross-sectional	community	IOTF	131	1640
Pretto, et al	Brazil	2004	2002-2011	cohort	medical institution	National Reference	104	616
Rmalho, et al (1)	Brazil	2013	2003	cross-sectional	community	WHO	2	199
Rmalho, et al (2)	Brazil	2013	2010	cross-sectional	community	WHO	25	378
Santos, et al	Brazil	2019	2004-	cohort	medical institution	WHO	461	3135
Sliva, et al	Brazil	2018	2012-2013	cross-sectional	community	WHO	364	1125
Mastroeni, et al	Brazil	2017	2012-2014	cohort	database	WHO	126	305
Vale, et al	Brazil	2022	2015	cross-sectional	database	WHO	1119	9400
Pereira, et al	Brazil	2023	2018-2019	cross-sectional	database	National Reference	516	1060
Lopes, et al	Brazil	2022	2008-2010	cross-sectional	school	IOTF	163	752
Santos, et al	Brazil	2022	2012-2013	cross-sectional	school	CDC	153	402
Coelho, et al	Brazil	2022	2019	cross-sectional	community	IOTF	64	170
Cavalcante, et al	Brazil	2022	2010	cohort	database	IOTF	234	2181
Blumenberg, et al	Brazil	2021	2015	longitudinal	community	IOTF	425	874
Barbiero, et al	Brazil	2009	2009	cross-sectional	school	CDC	141	511
Barbosa, et al	Brazil	2021	2021	cross-sectional	school	IOTF	90	353
Gemelli, et al	Brazil	2016	2014	cross-sectional	school	National Reference	260	727
Cândido, et al	Brazil	2009	2006	cross-sectional	school	IOTF	116	779
Polderman, et al	Brazil	2011	2008	cross-sectional	school	WHO	174	1002
Novaes, et al	Brazil	2013	2013	cross-sectional	school	WHO	226	769
Duncan, et al	Brazil	2011	2011	cross-sectional	school	WHO	819	3397
Halal, et al	Brazil	2016	2008	longitudinal	community	WHO	503	4231
Dalamaria, et al	Brazil	2021	2015	cross-sectional	school	WHO	304	1387
Lima, et al	Brazil	2020	2016	cross-sectional	school	CDC	196	583
Ribeiro, et al	Brazil	2017	2017	cohort	community	WHO	26	378
Nogueira, et al	Brazil	2020	2015-2016	cross-sectional	database	IOTF	86	259
Todendi, et al	Brazil	2020	2020	cross-sectional	database	WHO	362	981
Goldani, et al	Brazil	2013	2004-2005	cohort	medical institution	WHO	119	1463
Caixeta, et al	Brazil	2020	2016-2017	cross-sectional	school	WHO	92	486
Kuschnir, et al	Brazil	2009	2002	cross-sectional	database	WHO	458	2858
Ferreira, et al	Brazil	2008	2003	cross-sectional	school	IOTF	60	412
Rodrigues, et al	Brazil	2006	2003-2005	cross-sectional	school	WHO	53	380
Amorim, et al	Brazil	2006	2003	cross-sectional	school	WHO	167	1719
Araújo, et al	Brazil	2010	2004-2005	cohort	medical institution	WHO	1031	4452
Flores, et al	Brazil	2013	2005-2006	cross-sectional	database	WHO	5496	20514
Bispo, et al	Brazil	2015	2008-2009	cross-sectional	database	IOTF	225	1030
Castro, et al	Brazil	2012	2008	cross-sectional	school	IOTF	121	1786
Moraes, et al (1)	Brazil	2014	2008	cross-sectional	school	IOTF	57	991

© 2024 Zhang X et al. *JAMA Pediatrics.*

Santana, et al	Brazil	2013	2009	cross-sectional	school	WHO	220	1494
Lock, et al	Brazil	2020	2009-2010	cross-sectional	school	WHO	542	1528
Cruz, et al	Brazil	2013	2010-2011	cross-sectional	school	WHO	114	523
Moraes, et al	Brazil	2019	2010	longitudinal	database	WHO	293	673
Christofaro, et al	Brazil	2016	2011	cross-sectional	school	WHO	239	1231
Coutinho, et al	Brazil	2014	2011-2012	cross-sectional	school	WHO	876	2351
Pinho, et al	Brazil	2014	2011	cross-sectional	school	WHO	99	535
Guedes, et al	Brazil	2013	2011	cross-sectional	school	WHO	377	1968
Rosini, et al	Brazil	2015	2009	cross-sectional	school	WHO	346	1011
Neves, et al	Brazil	2015	2012	cross-sectional	school	National Reference	133	411
Santana, et al	Brazil	2017	2012	cross-sectional	school	IOTF	108	392
Costa, et al	Brazil	2020	2020	cross-sectional	school	WHO	330	1334
Vieira, et al	Brazil	2015	2013	cross-sectional	school	WHO	100	347
Araujo, et al	Brazil	2018	2014	cross-sectional	school	WHO	291	1182
Carmo, et al	Brazil	2018	2014-2016	cross-sectional	school	WHO	55	405
Fradkin, et al	Brazil	2018	2014-2016	cross-sectional	school	IOTF	330	1738
Reuter, et al	Brazil	2018	2014-2015	cross-sectional	school	WHO	333	1200
Ulbricht, et al	Brazil	2018	2014-2016	cross-sectional	school	IOTF	224	675
Ripka, et al	Brazil	2017	2015-2016	cross-sectional	school	CDC	97	374
Tebar, et al	Brazil	2018	2018	cross-sectional	school	CDC	280	1011
Farias, et al	Brazil	2019	2015	cross-sectional	school	IOTF	652	2694
Silva, et al	Brazil	2020	2015	cohort	database	IOTF	334	862
Schwertner, et al	Brazil	2020	2020	cross-sectional	school	CDC	76	330
Dantas, et al	Brazil	2018	2017	cross-sectional	school	CDC	152	578
Christofaro, et al	Brazil	2016	2016	cross-sectional	school	CDC	230	1231
Werneck, et al	Brazil	2018	2018	cross-sectional	school	IOTF	251	1209
Assis, et al	Brazil	2005	2002	cross-sectional	school	WHO	649	2936
Salas, et al	Brazil	2018	2010	cross-sectional	school	IOTF	417	1211
Assis, et al	Brazil	2019	2011-2012	cross-sectional	school	CDC	92	661
Dalmaso, et al	Brazil	2019	2019	cross-sectional	community	IOTF	160	572
Lima, et al	Brazil	2019	2019	cross-sectional	school	CDC	179	1169
Porcelli, et al	Brazil	2019	2012	cross-sectional	medical institution	IOTF	53	191
Alves, et al	Brazil	2020	2013-2014	cross-sectional	database	CDC	17473	71298
Folmann, et al	Brazil	2020	2013-2014	cross-sectional	school	CDC	548	1715
Hércules, et al	Brazil	2020	2016-2017	cross-sectional	database	CDC	28820	80782
Lucena, et al	Brazil	2020	2020	cross-sectional	community	CDC	376	1487
Rocha, et al	Brazil	2020	2017	cross-sectional	database	IOTF	371	2059
Rivera, et al	Brazil	2010	2001	cross-sectional	school	IOTF	172	1253
Gabriel, et al	Brazil	2010	2007-2008	cross-sectional	school	IOTF	1066	4964
Anwar, et al	Brazil	2010	2007	cross-sectional	school	CDC	324	988
Molina, et al	Brazil	2009	2007	cross-sectional	school	IOTF	298	1251
Fernandes, et al	Brazil	2009	2007	cross-sectional	school	IOTF	408	1779
Alves, et al	Brazil	2009	2006	cross-sectional	community	IOTF	92	733
Pelegrini, et al	Brazil	2008	2004-2005	cross-sectional	database	CDC	5360	36976
Bertolace, et al	Brazil	2008	2003	cross-sectional	school	CDC	89	421
Oliveira, et al	Brazil	2007	2007	cross-sectional	school	IOTF	95	699
da Silva, et al	Brazil	2007	2002	cross-sectional	community	IOTF	72	471
Granville-Garcia, et al	Brazil	2006	2006	cross-sectional	school	CDC	240	2651
Lima, et al	Brazil	2015	2015	cross-sectional	school	IOTF	64	175

© 2024 Zhang X et al. *JAMA Pediatrics*.

Katzmarzyk, et al (3)	Brazil	2015	2011-2013	cross-sectional	database	IOTF	108	493
Weinmayr, et al (1)	Brazil	2014	2004	cross-sectional	database	IOTF	473	953
Müller, et al	Brazil	2014	2008	cross-sectional	community	CDC	741	6360
Moreira, et al	Brazil	2014	2005-2006	cross-sectional	community	IOTF	440	1115
Mendonça, et al	Brazil	2014	2010-2011	cross-sectional	school	CDC	256	1168
Crispim, et al	Brazil	2014	2011-2012	cross-sectional	school	IOTF	80	276
Menezes, et al	Brazil	2011	2006	cross-sectional	database	IOTF	77	940
Cremm, et al	Brazil	2012	2012	cross-sectional	database	IOTF	196	531
Nascimento, et al	Brazil	2012	2009	cross-sectional	medical institution	National Reference	40	447
Novaes, et al	Brazil	2012	2005-2006	cross-sectional	school	CDC	82	764
Rosaneli, et al	Brazil	2012	2006	cross-sectional	school	WHO	1209	5037
Marques, et al	Brazil	2013	2013	cross-sectional	school	CDC	220	1396
Reuter, et al	Brazil	2013	2013	cross-sectional	school	WHO	140	564
Andrade, et al	Brazil	2016	2016	cross-sectional	school	CDC	129	396
Casonatto, et al	Brazil	2016	2002-2005	cross-sectional	database	CDC	271	978
Farias Júnior, et al (1)	Brazil	2016	2005	cross-sectional	school	WHO	193	2768
Farias Júnior, et al (2)	Brazil	2016	2009	cross-sectional	school	CDC	304	2776
Jardim-Botelho, et al	Brazil	2016	2009-2012	cross-sectional	community	National Reference	48	153
Silva, et al	Brazil	2016	2004	cross-sectional	school	WHO	455	2180
Araujo, et al	Brazil	2017	2017	cross-sectional	community	National Reference	68	548
Cuesta, et al	Brazil	2018	2013	cross-sectional	community	WHO	557	1296
Dos Santos, et al	Brazil	2018	2018	cross-sectional	school	WHO	120	501
Pivatto, et al	Brazil	2018	2016	cross-sectional	school	National Reference	112	236
Reuter, et al	Brazil	2018	2018	cross-sectional	school	National Reference	136	381
Romano, et al (6)	Brunei Darussalam	2022	2014	cross-sectional	database	National Reference	660	1824
Usheva, et al (2)	Bulgaria	2021	2012	cross-sectional	database	National Reference	105	874
Salas, et al (2)	Bulgaria	2021	2015-2017	cross-sectional	database	IOTF	965	3238
Mladenova, et al	Bulgaria	2015	2012-2014	cross-sectional	school	IOTF	206	878
Gorog, et al (1)	Bulgaria	2011	2011	cross-sectional	database	WHO	401	1554
Haug, et al (19)	Bulgaria	2009	2005-2006	cross-sectional	database	WHO	603	4563
Wijnhoven, et al (1)	Bulgaria	2015	2007/2008	cross-sectional	school	National Reference	1495	3627
Roos, et al (9)	Bulgaria	2014	2009	cross-sectional	database	National Reference	177	883
Mank, et al	Burkina Faso	2022	2020	cross-sectional	school	National Reference	168	1059
Daboné, et al	Burkina Faso	2011	2008-2009	cross-sectional	school	National Reference	15	649
Gebremedhin, et al (2)	Burkina Faso	2015	2010	cross-sectional	database	National Reference	303	6723
Gebremedhin, et al (1)	Burundi	2015	2010	cross-sectional	database	WHO	164	3493
Dimaisip-Nabuab, et	Cambodia	2018	2012-2014	prospective	database	WHO	21	624

© 2024 Zhang X et al. *JAMA Pediatrics.*

al (1)								
Navti, et al (1)	Cameroon	2021	2010	cross-sectional	school	National Reference	212	1274
Navti, et al (2)	Cameroon	2021	2020	cross-sectional	school	National Reference	276	1550
Navti, et al	Cameroon	2014	2012	cross-sectional	school	National Reference	97	557
Gebremedhin, et al (3)	Cameroon	2015	2011	cross-sectional	database	WHO	482	5185
Choukem, et al	Cameroon	2017	2013	cross-sectional	school	WHO	168	1343
Chelo, et al	Cameroon	2019	2017-2018	cross-sectional	school	WHO	60	822
Dapi, et al	Cameroon	2009	2009	cross-sectional	school	National Reference	18	581
Navti, et al	Cameroon	2015	2015	cross-sectional	school	WHO	98	557
Wamba, et al	Cameroon	2013	2010	cross-sectional	database	National Reference	333	2689
Navti ,et al	Cameroon	2017	2017	cross-sectional	school	WHO	94	522
Hulst, et al	Canada	2022	2001	longitudinal	database	WHO	427	1226
Anderson, et al	Canada	2022	2013-2019	cross-sectional	database	WHO	922	5962
Héroux, et al (1)	Canada	2012	2009-2010	cross-sectional	database	National Reference	3945	15532
Le, et al	Canada	2016	2011	cross-sectional	database	National Reference	483	1331
Seliske, et al	Canada	2009	2005-2006	cross-sectional	database	National Reference	1736	7987
Ball, et al	Canada	2019	2010/2017	cross-sectional	database	National Reference	36904	161114
Dubois, et al	Canada	2009	2002	cross-sectional	database	WHO	134	1520
Chaput, et al	Canada	2006	2003	cross-sectional	school	National Reference	93	422
Chaput, et al	Canada	2011	2005-2008	cohort	database	IOTF	226	550
Davidson, et al	Canada	2016	2009-2011	cross-sectional	community	WHO	81	235
Brault, et al	Canada	2015	2010	cross-sectional	school	National Reference	136	786
Larsen, et al	Canada	2015	2010-2011	cross-sectional	database	IOTF	248	943
Azad, et al	Canada	2014	2002	cross-sectional	database	IOTF	181	616
Bridgman, et al	Canada	2018	2018	cohort	database	IOTF	41	885
Cassidy-Bushrow, et al	Canada	2018	2003-2007	cohort	database	National Reference	90	527
Sheilds, et al	Canada	2010	2004	cross-sectional	database	IOTF	2460	8661
Medehouenou, et al	Canada	2015	2005-2010	cross-sectional	database	National Reference	70	290
Wang, et al	Canada	2008	2003	cross-sectional	database	IOTF	1652	4945
Mai, et al	Canada	2007	2003-2005	cohort	medical institution	National Reference	231	722
Shi, et al	Canada	2013	2007-2009	cross-sectional	database	National Reference	303	968
Walton, et al	Canada	2014	2014	RCT	database	CDC	49	110
Oliver, et al	Canada	2005	2000-2001	cross-sectional	database	CDC	944700	3190300
MOFFAT, et al	Canada	2005	2002-2004	cross-sectional	school	National Reference	73	266
Ball, et al	Canada	2005	2005	cross-sectional	school	WHO	20	135
Woodruff, et al	Canada	2010	2005-2006	cross-sectional	school	National Reference	142	1293
Twells, et al	Canada	2010	2005	cross-sectional	school	National Reference	366	1026
Menon, et al	Canada	2019	2015	cross-sectional	database	IOTF	2886	9866
Shay, et al	Canada	2020	2008-2010	cross-sectional	database	National Reference	177	1582
Simen-Kapeu, et al	Canada	2010	2008-2010	cross-sectional	school	National Reference	976	3421
Leatherdale, et al	Canada	2010	2007	cross-sectional	school	WHO	342	1264
Khaili, et al	Canada	2010	2005-2007	cross-sectional	community	National Reference	79	125
Ismailov, et al	Canada	2010	2005-2006	cross-sectional	school	IOTF	5235	25416

© 2024 Zhang X et al. *JAMA Pediatrics*.

Galloway, et al	Canada	2010	2007-2008	cross-sectional	community	WHO	293	376
Doan, et al	Canada	2010	2005	cross-sectional	community	National Reference	2404	12170
Cairney, et al	Canada	2010	2005-2007	longitudinal	school	National Reference	859	2278
Bengoecher, et al	Canada	2010	2010	cross-sectional	database	National Reference	665	3159
Wahi, et al	Canada	2009	2009	cross-sectional	community	IOTF	15	30
Vance, et al	Canada	2009	2002-2003	cross-sectional	school	IOTF	382	1917
Potestio, et al	Canada	2009	2005-2006	cross-sectional	school	National Reference	1090	6772
McGavock, et al	Canada	2009	2004-2006	longitudinal	school	IOTF	177	902
Downs, et al	Canada	2009	2004-2005	cross-sectional	community	IOTF	129	201
Spence, et al	Canada	2008	2004	longitudinal	medical institution	IOTF	108	501
Salvadori, et al	Canada	2008	2004	prospective	database	IOTF	199	675
Edwards, et al	Canada	2008	2004-2005	cross-sectional	medical institution	IOTF	1853	7369
Bruner, et al	Canada	2008	2001-2002	cross-sectional	database	IOTF	1126	4851
Willows, et al	Canada	2007	2002	cross-sectional	school	IOTF	552	1044
He, et al	Canada	2007	2001-2003	cross-sectional	school	National Reference	99	335
Bassett, et al	Canada	2007	2005	cross-sectional	school	National Reference	12	139
Ng, et al	Canada	2006	2004	cross-sectional	school	WHO	51	82
Janssen, et al	Canada	2006	2001	cross-sectional	database	WHO	1564	6684
Galloway, et al	Canada	2006	2004	cross-sectional	school	WHO	144	487
Rossiter, et al	Canada	2015	2011	cross-sectional	database	CDC	1787	5560
Katzmarzyk, et al (4)	Canada	2015	2011-2013	cross-sectional	database	National Reference	63	522
Borghese, et al	Canada	2015	2012-2013	cross-sectional	school	National Reference	128	550
Banerjee, et al	Canada	2015	2010	cross-sectional	database	National Reference	205	734
Carson, et al	Canada	2014	2010-2011	cross-sectional	school	IOTF	207	787
Leatherdale, et al	Canada	2013	2007-2008	cross-sectional	database	WHO	536	2326
Nyström, et al	Canada	2018	2014-2017	cross-sectional	community	WHO	3036	8343
Peña-Jorquera, et al	Chile	2021	2017-2019	cross-sectional	school	WHO	618	1181
Agüero, et al	Chile	2016	2014	cross-sectional	school	National Reference	999	1810
Olivares, et al	Chile	2004	2004	cross-sectional	school	WHO	608	1701
Cediel, et al	Chile	2016	2009-2010	cohort	database	IOTF	192	435
Mardones, et al	Chile	2008	2005	cross-sectional	school	WHO	2749	153536
Delgado-Floody, et al	Chile	2019	2019	cross-sectional	school	IOTF	282	605
Corvalán, et al	Chile	2010	2006	cross-sectional	school	IOTF	187	324
Valenzuela, et al	Chile	2015	2010-2011	cross-sectional	school	IOTF	431	1477
Lizana, et al	Chile	2015	2013	cross-sectional	school	IOTF	102	206
Cadenas-Sánchez, et al	Chile	2015	2013	cross-sectional	school	National Reference	225	434
Heitzinger, et al	Chile	2014	2009-2010	cross-sectional	medical institution	National Reference	470	795
Silva, et al	Chile	2013	2007	cross-sectional	community	WHO	176	453
Kagawa, et al	Chile	2016	2012	cross-sectional	database	IOTF	2375	11207
García-Hermoso, et	Chile	2017	2017	cross-sectional	school	IOTF	80	395

© 2024 Zhang X et al. *JAMA Pediatrics*.

al								
Liu, et al	China mainland	2016	2013	cross-sectional	school	IOTF	1624	10587
Li, et al	China mainland	2015	2012	cross-sectional	school	National Reference	626	2400
Tang, et al	China mainland	2022	2003	cohort	database	National Reference	11030	101505
Su, et al	China mainland	2022	2019-2020	cross-sectional	school	National Reference	607	3648
Shi, et al	China mainland	2022	2019-2020	cross-sectional	school	National Reference	12402	105181
Shi, et al	China mainland	2022	2016-2017	cross-sectional	database	CDC	12011	54269
Ma, et al	China mainland	2022	2019	cross-sectional	school	IOTF	2392	13670
Liu, et al	China mainland	2022	2019	cross-sectional	community	National Reference	1027	7664
Liu, et al	China mainland	2022	2017	cross-sectional	school	National Reference	4131	10753
Li, et al	China mainland	2022	2009-2013	cohort	medical institution	IOTF	5492	65056
Li, et al	China mainland	2022	2020	cross-sectional	school	National Reference	4835	18176
Huang, et al	China mainland	2022	2015	cross-sectional	database	National Reference	1281	8053
He, et al	China mainland	2022	2019-2020	longitudinal	database	IOTF	1058	5963
Guo, et al	China mainland	2022	2016	cross-sectional	database	National Reference	5172	26120
Gong, et al	China mainland	2022	2018	cross-sectional	school	WHO	144	629
Chen, et al	China mainland	2022	2019-2020	cross-sectional	database	National Reference	12221	36849
Chen, et al	China mainland	2022	2020	cross-sectional	school	WHO	612	2019
Chen, et al	China mainland	2022	2014	cross-sectional	database	WHO	2595	17356
Chang, et al	China mainland	2022	2009-2018	cohort	medical institution	WHO	3522	23617
Zhu, et al	China mainland	2021	2018	cross-sectional	school	National Reference	2868	12860
Zhou, et al	China mainland	2021	2016-2017	cohort	medical institution	WHO	352	10547
Zheng, et al	China mainland	2021	2015-2017	longitudinal	school	National Reference	988	3313
Zhao, et al (1)	China mainland	2021	2002	cross-sectional	school	National Reference	3570	26644
Zhao, et al (2)	China mainland	2021	2018	cross-sectional	school	WHO	11900	45417
Zhang, et al	China mainland	2021	2016	cross-sectional	community	National Reference	8411	110491
Zhang, et al	China mainland	2021	2018	cross-sectional	school	WHO	175	2642
Zhang, et al	China mainland	2021	2017-2019	cross-sectional	database	WHO	49491	201098
Yuan, et al	China mainland	2021	2016	cross-sectional	school	National Reference	178	768
You, et al	China mainland	2021	2017	cross-sectional	school	IOTF	743	3504
Xu, et al (1)	China mainland	2021	2014	cross-sectional	school	National Reference	258	1200
Wang, et al	China mainland	2021	2016-2018	cross-sectional	school	National Reference	5279	21571
Wang, et al	China mainland	2021	2014-2016	cross-sectional	database	WHO	3353	8365
Sun, et al	China mainland	2021	2017	longitudinal	database	WHO	811	1973
Min, et al	China mainland	2021	2018	cross-sectional	school	IOTF	276	3373
Liu, et al	China mainland	2021	2021	cross-sectional	school	WHO	4474	10855
Zhou, et al	China mainland	2020	2010-2014	cross-sectional	community	IOTF	312	3221

© 2024 Zhang X et al. *JAMA Pediatrics.*

Zhou, et al	China mainland	2020	2016	cross-sectional	school	WHO	329	2201
Zheng, et al	China mainland	2020	2014-2015	cross-sectional	school	WHO	1207	5295
Xu, et al	China mainland	2020	2020	cross-sectional	school	WHO	4925	22681
Tang, et al	China mainland	2010	2007	cross-sectional	school	WHO	143	1144
Shan, et al	China mainland	2010	2004	cross-sectional	school	WHO	4549	21198
Zhang, et al	China mainland	2014	2014	cross-sectional	school	WHO	376	1488
Abdumijit, et al	China mainland	2022	2021	cross-sectional	community	WHO	1362	4970
Cheng, et al	China mainland	2019	2016-2017	cross-sectional	school	IOTF	239515	1196004
Guo, et al	China mainland	2020	2013-2016	cross-sectional	database	IOTF	11329	40607
Li, et al	China mainland	2014	2009-2010	cross-sectional	school	IOTF	121	497
Zheng, et al	China mainland	2021	2019	cross-sectional	school	WHO	5638	36456
Zhang, et al	China mainland	2021	2021	cross-sectional	database	IOTF	4384	44718
Liu, et al	China mainland	2019	2013-2014	RCT	school	National Reference	665	1889
Jiang, et al	China mainland	2007	2007	RCT	school	CDC	594	2425
Li, et al	China mainland	2010	2005-2006	RCT	school	IOTF	1528	4700
Li, et al	China mainland	2014	2012-2013	RCT	school	IOTF	339	921
Xu, et al	China mainland	2015	2010-2011	RCT	school	IOTF	122	1108
Meng, et al	China mainland	2013	2009-2010	RCT	school	IOTF	1772	8301
Ji, et al	China mainland	2018	2017	cross-sectional	school	IOTF	28	112
Cui, et al	China mainland	2010	2006	longitudinal	database	IOTF	155	1174
Li, et al	China mainland	2008	2002	cross-sectional	database	IOTF	2379	44880
Zhang, et al	China mainland	2012	2003	cross-sectional	community	IOTF	12249	70431
Ma, et al	China mainland	2014	2011	cross-sectional	database	WHO	5159	36328
Zhang, et al	China mainland	2013	2010	cross-sectional	school	WHO	7478	42275
Cheng, et al	China mainland	2020	2015-2016	cross-sectional	school	IOTF	379	2201
Zhang, et al	China mainland	2020	2014	cross-sectional	school	IOTF	181	2264
Xiao, et al	China mainland	2020	2013-2014	cross-sectional	school	IOTF	1460	6091
Andegiorgish, et al	China mainland	2012	2010	cross-sectional	school	IOTF	887	3140
Yao, et al	China mainland	2014	2009-2013	cross-sectional	school	WHO	12155	67956
Zhou, et al	China mainland	2010	2007	cross-sectional	school	IOTF	243	1726
Li, et al	China mainland	2013	2009-2011	cross-sectional	medical institution	WHO	22496	38539
Zhang, et al	China mainland	2013	2013	cohort	community	IOTF	269	1098
Dong, et al	China mainland	2012	2006-2008	cross-sectional	school	IOTF	7937	30056
Zhou, et al	China mainland	2021	2011-2016	cross-sectional	medical institution	IOTF	2178	8441
Zhao, et al	China mainland	2017	2015	cross-sectional	school	WHO	426	1626
Ma, et al	China mainland	2011	2008-2009	cross-sectional	database	IOTF	2141	8653
Cao, et al	China	2012	2009	cross-sectional	school	National	14669	88974

© 2024 Zhang X et al. *JAMA Pediatrics*.

	mainland					Reference		
He, et al	China mainland	2009	2006	cross-sectional	school	IOTF	334	2179
Zhang, et al	China mainland	2012	2005	cross-sectional	database	IOTF	1411	8568
He, et al	China mainland	2017	2017	cross-sectional	school	IOTF	132	848
Zou, et al	China mainland	2022	2016-2017	cross-sectional	database	IOTF	601	2818
Zheng, et al	China mainland	2022	2019-2020	cross-sectional	database	WHO	186	1347
Zhang, et al	China mainland	2022	2019	cross-sectional	database	WHO	4315	14534
Zhang, et al	China mainland	2022	2021	cross-sectional	school	WHO	1156	4412
Zeng, et al	China mainland	2022	2012-2013	cohort	database	WHO	51	430
You, et al	China mainland	2022	2018	cohort	database	WHO	728	4513
Yang, et al	China mainland	2022	2019	longitudinal	database	IOTF	2198	6047
Xu, et al	China mainland	2022	2009	longitudinal	school	CDC	994	4538
Wu, et al	China mainland	2022	2009-2011	cross-sectional	database	IOTF	5446	47990
Wang, et al	China mainland	2022	2018	cross-sectional	school	WHO	1654	10536
Wang, et al	China mainland	2022	2020	cross-sectional	school	WHO	2362	9501
Iwata, et al	China mainland	2003	2003	cross-sectional	school	National Reference	112	532
Xiong, et al	China mainland	2010	2003-2004	cross-sectional	school	WHO	1563	7326
Tan, et al	China mainland	2018	2018	cross-sectional	school	CDC	1011	8999
Tan, et al	China mainland	2018	2013-2014	cross-sectional	school	National Reference	1695	19487
Wang, et al	China mainland	2018	2017	cross-sectional	school	National Reference	2057	18403
Zhai, et al	China mainland	2018	2013	cross-sectional	school	IOTF	133	853
Zhang, et al (1)	China mainland	2018	2010	cross-sectional	community	IOTF	10995.38	68020
Zhang, et al (2)	China mainland	2018	2011	cross-sectional	community	IOTF	9103.973	43928
Zhang, et al (3)	China mainland	2018	2012	cross-sectional	community	IOTF	23442.165	105153
Zhang, et al (4)	China mainland	2018	2013	cross-sectional	community	IOTF	30573.256	129830
Zhang, et al (5)	China mainland	2018	2014	cross-sectional	community	WHO	28140.385	119692
Zhang, et al (6)	China mainland	2018	2015	cross-sectional	community	CDC	26544.055	107587
Zhang, et al	China mainland	2018	2015	cross-sectional	database	WHO	304	1617
Zhang, et al	China mainland	2018	2014-2017	cross-sectional	school	WHO	87448	325083
Cai, et al	China mainland	2019	2013	cross-sectional	school	CDC	11709	47590
Chen ,et al (1)	China mainland	2019	2011	cross-sectional	community	WHO	2647	15757
Chen ,et al (2)	China mainland	2019	2014	cross-sectional	community	WHO	3243	19098
Chen ,et al (3)	China mainland	2019	2017	cross-sectional	community	CDC	3043	21883
Lu, et al	China mainland	2019	2019	cross-sectional	school	IOTF	221	687
Zhang, et al	China mainland	2019	2015	cross-sectional	database	WHO	33642	177419
Zhao, et al	China mainland	2019	2012	cross-sectional	database	CDC	201	1081
Zou, et al	China mainland	2019	2014	cross-sectional	school	WHO	686	2639

© 2024 Zhang X et al. *JAMA Pediatrics*.

Duan, et al	China mainland	2020	2016	cross-sectional	medical institution	IOTF	344	1955
Ke, et al	China mainland	2020	2019	cross-sectional	school	IOTF	432	1330
Liu, et al	China mainland	2020	2017	cross-sectional	school	IOTF	175	1123
Qian, et al	China mainland	2020	2017	cross-sectional	community	IOTF	1889	12426
Song, et al	China mainland	2020	2016	cross-sectional	database	IOTF	389	4488
Sun, et al	China mainland	2020	2012	cross-sectional	school	IOTF	552	2185
Xing, et al	China mainland	2020	2012-2013	cross-sectional	database	IOTF	2222	6740
Fan, et al	China mainland	2010	2010	cross-sectional	school	IOTF	542	3544
Wang, et al	China mainland	2009	2009	cross-sectional	community	IOTF	1657	8041
Xu, et al	China mainland	2008	2006	cross-sectional	school	IOTF	531	2020
Xu, et al	China mainland	2008	2004	cross-sectional	school	IOTF	451	6848
Li, et al	China mainland	2008	2004	cross-sectional	school	CDC	292	1792
Shi, et al	China mainland	2007	2002	cross-sectional	school	IOTF	112	824
Liu, et al	China mainland	2007	2000	cross-sectional	community	IOTF	19390	263168
Yan, et al	China mainland	2006	2003	cross-sectional	school	IOTF	2671	69627
Li, et al	China mainland	2006	2004	cross-sectional	school	IOTF	292	1792
Jiang, et al	China mainland	2006	2006	cross-sectional	school	IOTF	139	930
Zhu, et al	China mainland	2015	2013	cross-sectional	school	IOTF	525	4788
Yuan, et al	China mainland	2015	2010	cross-sectional	school	National Reference	3366	16580
Xu, et al	China mainland	2015	2007-2011	cross-sectional	community	IOTF	4090	29997
Xiao, et al	China mainland	2015	2006-2014	cross-sectional	school	IOTF	10880	145078
Wu, et al	China mainland	2015	2011-2012	cross-sectional	school	IOTF	15745	55536
Wan, et al	China mainland	2015	2013	cross-sectional	school	IOTF	552	2025
Piernas, et al (1)	China mainland	2015	2009	cross-sectional	database	IOTF	170	1191
Piernas, et al (2)	China mainland	2015	2011	cross-sectional	database	WHO	339	1648
Ma, et al (1)	China mainland	2015	2007	cross-sectional	school	IOTF	644	3832
Ma, et al (2)	China mainland	2015	2008	cross-sectional	school	IOTF	2050	13141
Ma, et al (3)	China mainland	2015	2009	cross-sectional	school	IOTF	2473	14052
Ma, et al (4)	China mainland	2015	2010	cross-sectional	school	IOTF	2420	13750
Katzmarzyk, et al (5)	China mainland	2015	2011-2013	cross-sectional	database	IOTF	123	501
Cao, et al	China mainland	2015	2013	cross-sectional	school	IOTF	736	8760
Wang, et al	China mainland	2014	2009	cross-sectional	school	IOTF	7937	30056
Li, et al	China mainland	2014	2012	cross-sectional	school	WHO	243	1150
He, et al	China mainland	2014	2014	cross-sectional	school	WHO	12129	67956
Dong, et al	China mainland	2014	2011	cross-sectional	community	WHO	916	4898
Dai, et al	China mainland	2014	2009-2010	cross-sectional	medical institution	WHO	5455	18707
Chen, et al	China	2014	2010-2011	cross-sectional	community	IOTF	295	1951

© 2024 Zhang X et al. *JAMA Pediatrics.*

(2)	mainland							
Chen, et al	China mainland	2011	2005	cross-sectional	database	IOTF	27065	231326
Xu, et al	China mainland	2011	2011	cross-sectional	school	National Reference	1945	8898
Guo, et al	China mainland	2012	2010-2011	cross-sectional	community	National Reference	924	4262
Jia, et al	China mainland	2012	2010	cross-sectional	school	WHO	224	702
Li, et al	China mainland	2012	2011	cross-sectional	school	IOTF	1282	7194
Yi, et al	China mainland	2012	2008-2009	cross-sectional	school	WHO	277	6740
Dong, et al	China mainland	2013	2010	cross-sectional	database	National Reference	29470	197191
Chen, et al (1)	China mainland	2016	2009	cross-sectional	medical institution	National Reference	480	22576
Chen, et al (2)	China mainland	2016	2012	cross-sectional	medical institution	IOTF	982	24816
Chen, et al (3)	China mainland	2016	2015	cross-sectional	medical institution	IOTF	1835	23837
Lei, et al	China mainland	2016	2013	cross-sectional	database	IOTF	1604	3327
Liu, et al	China mainland	2016	2016	cross-sectional	school	IOTF	1180	5781
Liu, et al	China mainland	2016	2014	cross-sectional	school	IOTF	1126	9917
Peng, et al	China mainland	2016	2016	cross-sectional	school	IOTF	2587	12297
Wei, et al	China mainland	2016	2016	cross-sectional	community	IOTF	324	1928
Zhang, et al	China mainland	2016	2013	cross-sectional	medical institution	National Reference	102	470
Zhang, et al	China mainland	2016	2009-2010	cross-sectional	school	IOTF	326	1410
Cai, et al	China mainland	2017	2016	cross-sectional	database	IOTF	29817	116615
Liu, et al	China mainland	2017	2015	cross-sectional	database	National Reference	12312	45608
Liu, et al	China mainland	2017	2016	cross-sectional	school	IOTF	877	4926
Zhang, et al	China mainland	2017	2014	cross-sectional	database	IOTF	1784	17318
Zong, et al	China mainland	2017	2006	cross-sectional	school	IOTF	37726.0184	290043
Chen, et al	China mainland	2018	2015-2016	cross-sectional	school	National Reference	52	222
Dong, et al	China mainland	2018	2014	cross-sectional	database	National Reference	3556	80821
Gong, et al	China mainland	2018	2016-2018	cross-sectional	database	National Reference	546	2795
He, et al	China mainland	2018	2013-2014	prospective	school	CDC	408	2032
Li, et al	China mainland	2018	2014	prospective	school	IOTF	236	1237
Liu, et al	China mainland	2018	2017	cross-sectional	school	National Reference	623	3670
Rutayisire, et al	China mainland	2018	2015	cross-sectional	school	IOTF	2326	8900
Contreras, et al	Colombia	2015	2013	cross-sectional	school	IOTF	152	603
Rincón-Pabón, et al	Colombia	2019	2010	cross-sectional	database	IOTF	2904	18177
Flannagan, et al	Colombia	2020	2006	cross-sectional	database	National Reference	137	723
Ramírez-Vélez, et al	Colombia	2017	2014-2015	cross-sectional	database	National Reference	598	2510
Martinez-Ospina, et al	Colombia	2019	2015	cross-sectional	school	National Reference	203	715
McDonald, et al	Colombia	2009	2006	cross-sectional	school	National Reference	341	3075
Gomez, et al	Colombia	2007	2005	cross-sectional	database	IOTF	1236	11137
Katzmarzyk,	Colombia	2015	2011-2013	cross-sectional	database	National	49	857

© 2024 Zhang X et al. *JAMA Pediatrics*.

et al (6)						Reference		
Arango, et al	Colombia	2011	2008	cross-sectional	school	WHO	88	546
Tiruneh, er al (3)	Comoros	2021	2010-2019	cross-sectional	database	CDC	220	2445
Gebremedhin, et al (4)	Comoros	2015	2012	cross-sectional	database	WHO	439	2699
Gebremedhin, et al (5)	Congo	2015	2011/2012	cross-sectional	database	WHO	218	4531
Gebremedhin, et al (6)	Congo	2015	2013/2014	cross-sectional	database	WHO	546	8391
Monge-Rojas, et al	Costa Rica	2022	2017	cross-sectional	school	National Reference	226	695
Gamboa-Gamboa, et al	Costa Rica	2021	2016	cross-sectional	database	WHO	118069	347366
Núñez-Rivas, et al	Costa Rica	2003	2000-2001	cross-sectional	school	WHO	593	1718
Gebremedhin, et al (11)	Côte d'Ivoire	2015	2011/2012	cross-sectional	database	National Reference	136	3294
Fossou, et al	Côte d'Ivoire	2020	2018	cross-sectional	school	WHO	128	1251
Salas, et al (3)	Croatia	2021	2015-2017	cross-sectional	database	CDC	863	2601
Banjari, et al (2)	Croatia	2020	2020	cross-sectional	school	IOTF	86	478
Juresa, et al	Croatia	2012	2003-2004	cross-sectional	school	WHO	200	960
Janssen, et al (4)	Croatia	2005	2001-2002	cross-sectional	database	WHO	514	4145
Milanović, et al	Croatia	2020	2015	cross-sectional	school	IOTF	2007	5591
Haug, et al (10)	Croatia	2009	2005-2006	cross-sectional	database	CDC	699	4720
Bili}-Kirin, et al	Croatia	2014	2014	cross-sectional	school	IOTF	33	372
Pećin, et al	Croatia	2013	2013	cross-sectional	school	IOTF	44	756
Ahrens, et al (3)	Cyprus	2014	2007-2008	cross-sectional	database	IOTF	689	2942
Savva, et al	Cyprus	2005	2004	cross-sectional	school	IOTF	277	1413
Savva, et al	Cyprus	2008	2002-2003	cross-sectional	school	IOTF	2084	7060
Savva, et al	Cyprus	2014	2009-2010	cross-sectional	school	IOTF	875	3090
Lazarou, et al	Cyprus	2008	2008	cross-sectional	school	IOTF	188	823
Loucaides, et al	Cyprus	2008	2007	cross-sectional	school	IOTF	90	247
Loucaides, et al	Cyprus	2010	2010	cross-sectional	school	IOTF	433	1966
Tornaritis, et al	Cyprus	2014	2009-2010	cross-sectional	school	IOTF	303	1414
Savva, et al	Cyprus	2014	2001-2003	longitudinal	school	IOTF	1175	4878
Vážná, et al	Czech	2022	2021	cross-sectional	school	National Reference	1166	3517
Salas, et al (4)	Czech	2021	2015-2017	cross-sectional	database	IOTF	292	1395
Gorog, et al (2)	Czech	2011	2011	cross-sectional	database	IOTF	245	1508
Gouw, et al	Czech	2010	2001	cross-sectional	database	IOTF	3912	30966
Janssen, et al (5)	Czech	2005	2001-2002	cross-sectional	database	IOTF	504	4990
Haug, et al (20)	Czech	2009	2005-2006	cross-sectional	database	IOTF	715	4734
Wijnhoven, et al (2)	Czech	2015	2007/2008	cross-sectional	school	IOTF	460	1633
Brixval, et al	Denmark	2012	2002	cross-sectional	database	IOTF	462	4208
Salas, et al (5)	Denmark	2021	2015-2017	cross-sectional	database	IOTF	129	935
Yngve, et al (3)	Denmark	2008	2003	cross-sectional	database	IOTF	122	1066
Ajslev, et al	Denmark	2011	2004-2009	cohort	database	IOTF	2711	28354
Mor, et al	Denmark	2015	2002-2013	cohort	database	IOTF	1077	9886
Høyer, et al (1)	Denmark	2014	2002-2004	cohort	database	IOTF	139	525
Karlsen, et al	Denmark	2017	2007-2009	cohort	medical institution	National Reference	70	371

© 2024 Zhang X et al. *JAMA Pediatrics*.

Janssen, et al (6)	Denmark	2005	2001-2002	cross-sectional	database	IOTF	457	4009
Krue, et al	Denmark	2010	2010	cross-sectional	school	IOTF	2191	8694
Haug, et al (27)	Denmark	2009	2005-2006	cross-sectional	database	IOTF	456	4708
Rex, et al	Denmark	2014	2011-2013	cross-sectional	medical institution	IOTF	137	607
Matthiessen, et al	Denmark	2014	2005-2008	cross-sectional	database	IOTF	86	512
Klakk, et al	Denmark	2013	2008	prospective	school	CDC	67	632
Kjelgaard, et al	Denmark	2017	2010	cross-sectional	database	WHO	394	4922
Manyanga, et al (2)	Djibouti	2014	2014	cross-sectional	database	IOTF	322	1711
Taguri, et al (3)	Djibouti	2009	2002	cross-sectional	database	IOTF	137	1538
Romano, et al (7)	Dominican Republic	2022	2016	cross-sectional	database	IOTF	309	954
Romano, et al (8)	East Timor	2022	2015	cross-sectional	database	IOTF	97	1631
Andrade, et al	Ecuador	2014	2009-2012	RCT	school	IOTF	287	1440
Abril, et al	Ecuador	2013	2010-2011	cross-sectional	school	IOTF	243	743
Freire, et al	Ecuador	2014	2012	cross-sectional	database	IOTF	3445	11534
Ortiz, et al	Ecuador	2014	2008	cross-sectional	community	IOTF	120	703
Casaoulla, et al	Ecuador	2017	2015	cross-sectional	school	IOTF	50	427
Walrod, et al	Ecuador	2018	2013-2014	cross-sectional	community	IOTF	129	298
Abd El-Aty, et al	Egypt	2020	2016-2017	cross-sectional	community	National Reference	644	5458
Abdel Wahed, et al	Egypt	2017	2014-2015	cross-sectional	community	IOTF	110	736
Abdelkarim, et al	Egypt	2017	2014-2015	cross-sectional	community	IOTF	159	676
Abou-Khadra, et al	Egypt	2022	2018-2019	cross-sectional	school	IOTF	126	319
El-Sabely, et al	Egypt	2013	2013	cross-sectional	school	IOTF	137	288
Badawi, et al	Egypt	2013	2011	cross-sectional	school	IOTF	266	852
Manyanga, et al (3)	Egypt	2014	2014	cross-sectional	database	IOTF	1626	5179
Hassan, et al	Egypt	2008	2002-2004	cross-sectional	school	IOTF	227	1283
Hassan, et al	Egypt	2016	2013-2016	cross-sectional	medical institution	National Reference	47	154
Talat, et al	Egypt	2016	2014-2015	cross-sectional	school	IOTF	276	900
Pérez, et al	El Salvador	2020	2015-2016	cross-sectional	school	WHO	25968	111991
Ahrens, et al (2)	Estonia	2014	2007-2008	cross-sectional	database	IOTF	297	2067
Janssen, et al (8)	Estonia	2005	2001-2002	cross-sectional	database	IOTF	282	3752
Haug, et al (29)	Estonia	2009	2005-2006	cross-sectional	database	IOTF	400	4215
Weinmayr, et al (2)	Estonia	2014	2004	cross-sectional	database	CDC	44	241
Worku, et al	Ethiopia	2021	2018	cross-sectional	school	WHO	96	522
Tiruneh, er al (2)	Ethiopia	2021	2010-2019	cross-sectional	database	WHO	297	10513
Wakayo, et al	Ethiopia	2016	2013	cross-sectional	school	IOTF	12	174
Abich, et al	Ethiopia	2020	2018-2019	cross-sectional	school	CDC	135	823
Tadesse, et al	Ethiopia	2017	2015	cross-sectional	school	IOTF	32	462
Sorrie, et al	Ethiopia	2017	2016	cross-sectional	community	National Reference	69	500
Desalew, et al	Ethiopia	2017	2016	cross-sectional	school	IOTF	92	448
Gebremedhin, et al (7)	Ethiopia	2015	2011	cross-sectional	database	IOTF	296	9880
Yetubie, et al	Ethiopia	2010	2008	cross-sectional	school	IOTF	6	194
Gali, et al	Ethiopia	2017	2015	cross-sectional	school	IOTF	68	510

© 2024 Zhang X et al. *JAMA Pediatrics*.

Askal, et al	Ethiopia	2015	2014	cross-sectional	school	IOTF	81	828
Carrillo-Larco, et al (4)	Ethiopia	2014	2014	cohort	database	IOTF	13	1882
Mitiku, et al	Ethiopia	2019	2014	cross-sectional	school	IOTF	86	1523
Mekonnen, et al	Ethiopia	2018	2016	cross-sectional	school	IOTF	76	634
Romano, et al (9)	Fiji	2022	2016	cross-sectional	database	National Reference	424	1537
Pengpid, et al (1)	Fiji	2015	2010-2011	cross-sectional	database	IOTF	338	1773
Petersen, et al	Fiji	2014	2005-2008	cross-sectional	database	IOTF	1960	8947
Sarkkola, et al	Finland	2022	2011-2014	cross-sectional	database	IOTF	1629	10646
Eloranta, et al	Finland	2012	2007-2009	cross-sectional	database	IOTF	67	510
Laitinen, et al	Finland	2012	2001-2002	longitudinal	medical institution	IOTF	994	6637
Saari, et al	Finland	2015	2003-2007	cohort	medical institution	IOTF	2094	12062
Parikka, et al	Finland	2015	2007-2009	cross-sectional	database	IOTF	786	4409
Janssen, et al (9)	Finland	2005	2001-2002	cross-sectional	database	IOTF	744	5205
Vuorela, et al	Finland	2010	2010	cross-sectional	school	IOTF	117	606
Veltsista, et al (1)	Finland	2010	2001	cross-sectional	school	IOTF	872	6468
Vanhala, et al	Finland	2009	2004	cross-sectional	school	IOTF	125	749
Haug, et al (30)	Finland	2009	2005-2006	cross-sectional	database	IOTF	769	4934
Fogelholm, et al	Finland	2008	2003	cross-sectional	school	IOTF	328	2266
Virtanen, et al	Finland	2015	2008-2009	cross-sectional	school	IOTF	3014	23182
Palomäki, et al	Finland	2015	2010	cross-sectional	school	WHO	214	1301
Katzmarzyk, et al (7)	Finland	2015	2011-2013	cross-sectional	database	WHO	26	500
Roos, et al (4)	Finland	2014	2009	cross-sectional	database	WHO	146	857
Vanhelst, et al	France	2022	2008-2018	cross-sectional	database	WHO	13974	90250
Roth, et al	France	2022	2017-2020	cross-sectional	school	CDC	2217	19295
Vanhelst, et al (1)	France	2021	2008	cross-sectional	database	CDC	407	2253
Vanhelst, et al (2)	France	2021	2015	cross-sectional	database	CDC	291	2242
Luiggi, et al	France	2021	2019	cross-sectional	school	National Reference	127	1038
Dupuy, et al	France	2011	2006	cross-sectional	database	National Reference	686	6604
Thibault, et al	France	2013	2007-2009	cross-sectional	school	WHO	948	7667
Thibault, et al	France	2010	2004-2005	cross-sectional	school	National Reference	324	2385
Carriere, et al	France	2015	2004	cross-sectional	school	IOTF	280	1836
Salanave, et al	France	2009	2007	cross-sectional	school	National Reference	160	1014
Heude, et al	France	2003	2000	cross-sectional	school	WHO	84	601
Legleye, et al	France	2014	2010	cross-sectional	database	WHO	54	303
Chau, et al	France	2013	2010	cross-sectional	school	National Reference	563	1270
Jouret, et al	France	2007	2007	cross-sectional	school	WHO	155	1780
Pitrou, et al	France	2010	2004-2005	cross-sectional	school	IOTF	178	1030
Ruiz, et al (5)	France	2016	2007	cohort	database	IOTF	88	1139
Janssen, et al (10)	France	2005	2001-2002	cross-sectional	database	WHO	884	7624

© 2024 Zhang X et al. *JAMA Pediatrics.*

Kleiser, et al	France	2003	2001	cross-sectional	school	IOTF	1209	4326
Rolland-Cachera, et al	France	2002	2000	cross-sectional	school	IOTF	286	1582
Tubert-Jeannin, et al	France	2018	2011-2012	cross-sectional	school	IOTF	480	2676
Haug, et al (5)	France	2009	2005-2006	cross-sectional	database	IOTF	684	6583
Kêkê, et al	France	2015	2009	cross-sectional	school	IOTF	317	1382
Vanhelst, et al (1)	France	2017	2009	cross-sectional	database	IOTF	350	1848
Vanhelst, et al (2)	France	2017	2010	cross-sectional	database	CDC	373	1818
Vanhelst, et al (3)	France	2017	2011	cross-sectional	database	IOTF	503	2578
Vanhelst, et al (4)	France	2017	2012	cross-sectional	database	National Reference	467	2165
Vanhelst, et al (5)	France	2017	2013	cross-sectional	database	IOTF	262	1260
Romano, et al (10)	French Polynesia	2022	2015	cross-sectional	database	National Reference	875	1902
Gebremedhin, et al (8)	Gabon	2015	2012	cross-sectional	database	CDC	338	3482
Gebremedhin, et al (9)	Gambia	2015	2013	cross-sectional	database	IOTF	181	3360
Salas, et al (6)	Georgia	2021	2015-2017	cross-sectional	database	National Reference	746	3057
Weinmayr, et al (3)	Georgia	2014	2004	cross-sectional	database	CDC	43	169
Ahrens, et al (6)	Germany	2014	2007-2008	cross-sectional	database	CDC	361	2194
Wang, et al	Germany	2023	2005	cohort	database	CDC	225	1978
Hoebel, et al	Germany	2022	2014-2017	cross-sectional	database	National Reference	759	3567
Zhou, et al	Germany	2021	2010-2014	cross-sectional	school	CDC	2200	22678
Usheva, et al (3)	Germany	2021	2012	cross-sectional	database	CDC	75	1104
Sergentanis, et al (3)	Germany	2021	2011-2012	cross-sectional	database	National Reference	255	1621
Nguyen, et al	Germany	2021	2017-2018	cross-sectional	school	IOTF	550	5656
Liu, et al	Germany	2021	2013-2018	cross-sectional	school	IOTF	6073	33407
Willerhausen, et al	Germany	2007	2007	cross-sectional	medical institution	IOTF	279	1290
Kesztyüs, et al	Germany	2013	2009	cross-sectional	database	IOTF	59	1730
Negal, et al	Germany	2009	2006	RCT	school	WHO	217	1063
Boneberger, et al	Germany	2009	2005-2006	cross-sectional	school	National Reference	809	4718
Kromeyer-Hauschild, et al	Germany	2007	2001	cross-sectional	school	CDC	243	1915
Landsberg, et al	Germany	2010	2000-2005	cross-sectional	database	National Reference	358	1894
Toschke, et al	Germany	2005	2001-2002	cross-sectional	medical institution	CDC	564	4370
Toschke, et al	Germany	2003	2000-2001	cross-sectional	school	National Reference	617	4706
Kries, et al	Germany	2008	2005	cross-sectional	school	WHO	1038	5899
Raum, et al	Germany	2011	2001-2002	cross-sectional	medical institution	CDC	258	1979
Pei, et al	Germany	2013	2005-2008	cohort	database	CDC	646	3116
Kleiser, et al	Germany	2009	2003-2006	cross-sectional	database	CDC	2744	13395
Toschke, et al	Germany	2007	2001-2002	cross-sectional	school	National Reference	792	5472
Weyermann, et al	Germany	2006	2000-2001	cohort	medical institution	WHO	96	855
Hoffmann, et al	Germany	2019	2019	cross-sectional	school	IOTF	17	198
Suchert, et al	Germany	2016	2014	cross-sectional	school	National Reference	299	1228
Will, et al	Germany	2005	2002	cross-sectional	school	National	62	523

© 2024 Zhang X et al. *JAMA Pediatrics*.

						Reference		
Rapp, et al	Germany	2005	2002	longitudinal	school	IOTF	435	2140
Lamerz, et al	Germany	2005	2001-2002	cross-sectional	school	National Reference	178	1979
Janssen, et al (11)	Germany	2005	2001-2002	cross-sectional	database	CDC	556	4878
Graf, et al	Germany	2004	2001	cross-sectional	school	IOTF	76	668
Graf, et al	Germany	2004	2002-2003	cross-sectional	school	National Reference	41	344
Reich, et al	Germany	2003	2003	cross-sectional	school	IOTF	588	2354
Würbach, et al	Germany	2009	2005-2006	cross-sectional	school	WHO	132	1571
Warschburger, et al	Germany	2009	2006	cross-sectional	medical institution	IOTF	27	219
Toschke, et al	Germany	2009	2004-2005	cross-sectional	school	IOTF	166	4642
Sporišević, et al	Germany	2009	2007-2008	cross-sectional	community	National Reference	43	214
Koller, et al	Germany	2009	2004	cross-sectional	school	WHO	933	9067
Haug, et al (6)	Germany	2009	2005-2006	cross-sectional	database	CDC	854	6619
De Toia , et al	Germany	2009	2006-2008	cross-sectional	school	CDC	161	1228
Bayer, et al	Germany	2009	2004-2006	cross-sectional	database	IOTF	2223	12199
Bau, et al	Germany	2009	2006-2007	cross-sectional	database	CDC	26	236
Nagel, et al	Germany	2008	2004-2005	cross-sectional	school	IOTF	117	557
Mauch, et al	Germany	2008	2008	cross-sectional	school	National Reference	456	2871
Jahnke, et al	Germany	2008	2008	cross-sectional	medical institution	IOTF	33	142
Herpertz-Dahlmann, et al	Germany	2008	2003-2006	cross-sectional	community	National Reference	337	1843
Willershausen, et al	Germany	2007	2007	cross-sectional	school	CDC	349	2071
Kobel, et al	Germany	2015	2015	cross-sectional	school	WHO	29	294
Brettschneider, et al	Germany	2015	2009-2012	cross-sectional	database	WHO	444	4948
Weinmayr, et al (4)	Germany	2014	2004	cross-sectional	database	WHO	329	1580
Roos, et al (5)	Germany	2014	2009	cross-sectional	database	WHO	108	567
Akoto, et al	Ghana	2022	2022	cross-sectional	school	National Reference	17	155
Agaba, et al	Ghana	2022	2018	cross-sectional	school	WHO	19	711
Aboagye, et al	Ghana	2022	2022	cross-sectional	school	WHO	105	423
Adom, et al	Ghana	2019	2019	cross-sectional	school	WHO	89	543
Amidu, et al	Ghana	2013	2012-2013	cross-sectional	school	CDC	69	400
Hohammed, et al	Ghana	2012	2012	cross-sectional	school	National Reference	72	270
Intiful, et al	Ghana	2013	2013	cross-sectional	school	WHO	29	124
Manyanga, et al (4)	Ghana	2014	2014	cross-sectional	database	National Reference	535	6155
Obiricorang, et al	Ghana	2015	2013-2014	cross-sectional	school	CDC	42	303
Aryeetey, et al	Ghana	2017	2009-2012	cross-sectional	school	National Reference	558	3089
Atsu, et al	Ghana	2017	2011	cross-sectional	database	CDC	178	7328
Annan-Asare, et al	Ghana	2017	2017	cross-sectional	school	WHO	147	260
Kwabla, et al	Ghana	2018	2016	cross-sectional	school	CDC	3	359
Gyamfi, et al	Ghana	2019	2019	cross-sectional	school	CDC	224	1004
Ganle, et al	Ghana	2019	2019	cross-sectional	school	National Reference	133	285
Weinmayr, et al (5)	Ghana	2014	2004	cross-sectional	database	WHO	1	241
Mogre, et al	Ghana	2013	2011	cross-sectional	school	WHO	34	400
Makri, et al	Greece	2022	2018	cross-sectional	database	CDC	942	3816
Usheva, et al (4)	Greece	2021	2012	cross-sectional	database	IOTF	280	1647

© 2024 Zhang X et al. *JAMA Pediatrics.*

Sergentanis, et al (7)	Greece	2021	2011-2012	cross-sectional	database	IOTF	322	1516
Kostopoulou, et al	Greece	2021	2018-2019	cross-sectional	database	CDC	1097	3504
Pikramenou, et al	Greece	2016	2013	cross-sectional	school	CDC	349	2180
Trikaliotis, et al	Greece	2011	2011	cross-sectional	school	CDC	36	361
Grigorakis, et al	Greece	2016	2010-2011	cross-sectional	school	CDC	41702	124113
Kleanthous, et al	Greece	2016	2009	cross-sectional	school	National Reference	493	1327
Manios, et al	Greece	2013	2007	cross-sectional	database	IOTF	1054	2492
Manios, et al	Greece	2011	2005-2006	cross-sectional	school	WHO	198	481
Tokmakidis, et al	Greece	2006	2006	cross-sectional	school	WHO	288	709
Risvas, et al	Greece	2012	2008-2009	cross-sectional	database	CDC	877	2093
Daraki, et al	Greece	2015	2007-2008	cross-sectional	database	CDC	134	618
Lamprokostopoulou, et al	Greece	2019	2007-2010	cross-sectional	school	CDC	400	919
Kontogianni, et al	Greece	2010	2007	cross-sectional	community	CDC	325	1170
Kosti, et al	Greece	2007	2004-2005	cross-sectional	database	CDC	388	2008
Lagiou, et al	Greece	2008	2003	cross-sectional	school	IOTF	121	633
Cassimos, et al	Greece	2011	2008-2009	cross-sectional	school	WHO	166	335
Krassas, et al (1)	Greece	2004	2004	cross-sectional	school	WHO	647	2468
Angelopoulos, et al	Greece	2006	2003-2004	cross-sectional	school	CDC	137	312
Magkos, et al	Greece	2006	2002	cross-sectional	community	National Reference	240	620
Magkos, et al	Greece	2006	2006	cross-sectional	school	IOTF	73	198
Papadimitriou, et al	Greece	2006	2003-2004	cross-sectional	school	WHO	1580	4131
Manios, et al	Greece	2007	2003-2004	cross-sectional	database	National Reference	506	2374
Kamtsios, et al	Greece	2008	2008	cross-sectional	school	CDC	209	775
Linardakis, et al	Greece	2008	2004-2005	cross-sectional	school	WHO	251	856
Hassapidou, et al	Greece	2009	2006	cross-sectional	school	CDC	95	266
Kollias, et al	Greece	2009	2009	cross-sectional	school	National Reference	238	797
Koroni, et al	Greece	2009	2009	cross-sectional	school	CDC	590	1861
Mavrakanas, et al	Greece	2009	2007	cross-sectional	school	WHO	80	572
Yannakoulia, et al	Greece	2010	2010	cross-sectional	database	WHO	430	1132
Hassapidou, et al	Greece	2015	2009-2010	cross-sectional	school	IOTF	213	1006
Spathopoulos, et al	Greece	2009	2005-2006	cross-sectional	school	CDC	737	2715
Antonogeorgos, et al	Greece	2012	2005-2006	cross-sectional	database	CDC	199	700
Nassis, et al	Greece	2005	2005	cross-sectional	school	WHO	368	1362
Janssen, et al (12)	Greece	2005	2001-2002	cross-sectional	database	CDC	609	3584
Manios, et al	Greece	2004	2004	cross-sectional	school	IOTF	72	195
Veltsista, et al (2)	Greece	2010	2001	cross-sectional	school	IOTF	432	2842
Poulimeneas, et al	Greece	2019	2019	cross-sectional	community	CDC	52	172
Vazquez, et al	Greece	2019	2012-2013	cross-sectional	database	IOTF	611	2675
Katsagoni, et al	Greece	2020	2014-2015	cross-sectional	database	CDC	52922	174209
Notara,et al	Greece	2020	2014-2016	cross-sectional	school	National Reference	462	1659
Mirkopoulou	Greece	2010	2005	cross-sectional	school	CDC	31	98

© 2024 Zhang X et al. *JAMA Pediatrics*.

, et al								
Lazarou, et al	Greece	2010	2005	cross-sectional	school	CDC	192	1140
Antonogeorgos, et al	Greece	2010	2005-2006	cross-sectional	school	IOTF	243	700
Karatzi , et al	Greece	2009	2004-2005	prospective	school	IOTF	103	754
Haug, et al (11)	Greece	2009	2005-2006	cross-sectional	database	CDC	652	3566
Yannakoulia, et al	Greece	2008	2007	cross-sectional	database	CDC	428	1132
Hassapidou, et al	Greece	2006	2006	cross-sectional	school	WHO	131	502
Vafeiadi, et al	Greece	2015	2007	prospective	community	National Reference	147	689
Sourani, et al	Greece	2015	2011	cross-sectional	community	CDC	117	352
Patsopoulou, et al	Greece	2015	2015	cross-sectional	school	CDC	116	451
Kambas, et al	Greece	2015	2011	cross-sectional	database	CDC	58	250
Weinmayr, et al (6)	Greece	2014	2004	cross-sectional	database	WHO	197	404
Roos, et al (8)	Greece	2014	2009	cross-sectional	database	CDC	228	652
Grammatikopoulou, et al	Greece	2014	2010-2012	cross-sectional	school	CDC	11617	37344
Antonogeorgos, et al	Greece	2011	2006	cross-sectional	database	WHO	199	700
Athanasopoulos, et al	Greece	2011	2010	cross-sectional	school	CDC	67	232
Farajian, et al	Greece	2011	2009	cross-sectional	school	WHO	1972	4786
Michalopoulou, et al	Greece	2011	2009	cross-sectional	school	CDC	162	532
Jelastopulu, et al	Greece	2012	2009	cross-sectional	school	CDC	85	200
Kyriazis, et al	Greece	2012	2009-2010	cross-sectional	school	CDC	740	2374
Tambalis, et al	Greece	2013	2013	cross-sectional	school	National Reference	1243	3195
Poulimeneas, et al	Greece	2016	2016	cross-sectional	school	IOTF	1734	4833
Garoufi, et al	Greece	2017	2009-2010	cross-sectional	school	IOTF	310	736
Velde, et al (2)	Greece	2017	2010	cross-sectional	database	IOTF	441	1085
Koulouvaris, et al	Greece	2018	2018	cross-sectional	community	National Reference	201	463
Manios, et al	Greece	2018	2018	cross-sectional	school	IOTF	962	2263
Janssen, et al (13)	Greenland	2005	2001-2002	cross-sectional	database	IOTF	116	558
Haug, et al (31)	Greenland	2009	2005-2006	cross-sectional	database	IOTF	176	943
Alvarado, et al	Guatemala	2009	2004-2005	cross-sectional	school	IOTF	139	363
Gebremedhin, et al (10)	Guinea	2015	2012	cross-sectional	database	IOTF	183	3216
Torres, et al	Honduras	2014	2011	cross-sectional	school	IOTF	378	2554
Tung, et al	Hong Kong	2021	2014	cohort	database	IOTF	3307	18863
Huang, et al	Hong Kong	2019	2009-2012	cohort	database	National Reference	50	599
Wing, et al	Hong Kong	2009	2003	cross-sectional	school	IOTF	764	5159
Wang, et al	Hong Kong	2019	2008-2009	cohort	database	IOTF	754	3614
Chan, et al	Hong Kong	2013	2007-2008	cross-sectional	school	IOTF	332	1614
Wang, et al	Hong Kong	2017	2015	cross-sectional	school	IOTF	178	894
Wong, et al	Hong Kong	2005	2002	cross-sectional	school	IOTF	2598	10773
Leung, et al	Hong Kong	2009	2003	cross-sectional	database	CDC	98	486
Ko, et al	Hong Kong	2008	2003-2004	cross-sectional	school	National Reference	266	2098
Cheung, et al	Hong Kong	2007	2003-2004	cross-sectional	school	IOTF	152	1088
Knowles, et	Hong Kong	2015	2015	cross-sectional	school	WHO	144	620

© 2024 Zhang X et al. *JAMA Pediatrics*.

al								
Chan, et al	Hong Kong	2014	2003-2004	cross-sectional	school	IOTF	57	351
Ip, et al	Hong Kong	2017	2013-2014	cross-sectional	school	CDC	10351	208280
Lee, et al	Hong Kong	2017	2012-2013	cross-sectional	database	National Reference	1158	4410
Brug, et al (2)	Hungary	2012	2012	cross-sectional	database	WHO	254	1020
Ahrens, et al (7)	Hungary	2014	2007-2008	cross-sectional	database	IOTF	550	3159
Baráth, et al	Hungary	2010	2005-2006	cross-sectional	school	IOTF	3344	14290
Bjørnarå, et al	Hungary	2014	2010	cross-sectional	database	WHO	363	929
Gorog, et al (3)	Hungary	2011	2011	cross-sectional	database	WHO	265	1661
Janssen, et al (14)	Hungary	2005	2001-2002	cross-sectional	database	IOTF	500	3845
Haug, et al (21)	Hungary	2009	2005-2006	cross-sectional	database	IOTF	488	3214
Antal, et al	Hungary	2009	2005-2006	cross-sectional	school	IOTF	495	1928
Velde, et al (3)	Hungary	2017	2010	cross-sectional	database	IOTF	254	1022
Erdei, et al	Hungary	2018	2016	cross-sectional	school	IOTF	1152	5332
Jakab, et al	Hungary	2018	2015-2017	cross-sectional	school	IOTF	1363	6824
Aanesen, et al	Iceland	2020	2016-2017	cross-sectional	community	IOTF	776	4360
Sergentanis, et al (1)	Iceland	2021	2011-2012	cross-sectional	database	IOTF	153	1196
Yngve, et al (4)	Iceland	2008	2003	cross-sectional	database	IOTF	115	714
Hrafnkelsso n , et al	Iceland	2009	2006	cross-sectional	school	IOTF	37	267
Haug, et al (32)	Iceland	2009	2005-2006	cross-sectional	database	WHO	1146	8014
Roos, et al (3)	Iceland	2014	2009	cross-sectional	database	IOTF	72	378
Saha, et al	India	2022	2015-2016	cross-sectional	database	IOTF	5130	176255
Dabas, et al (1)	India	2022	2008	cross-sectional	school	WHO	603	3172
Dabas, et al (2)	India	2022	2013	cross-sectional	school	IOTF	1014	3007
Dabas, et al (3)	India	2022	2015	cross-sectional	school	IOTF	613	2238
Arushi, et al	India	2022	2020-2021	cross-sectional	school	IOTF	305	1620
Thomas, et al	India	2021	2019-2020	cross-sectional	school	National Reference	71	440
Seema, et al	India	2021	2016-2018	cross-sectional	school	IOTF	92	385
Moitra, et al	India	2021	2021	cross-sectional	school	IOTF	296	772
Elangovan, et al	India	2012	2011	cross-sectional	medical institution	National Reference	148	510
Subramania m, et al	India	2011	2011	cross-sectional	school	IOTF	158	2033
Shailee, et al	India	2018	2009	cross-sectional	school	IOTF	29	1011
Balaram, et al	India	2017	2012-2015	cross-sectional	school	IOTF	123	1748
Goyal, et al	India	2011	2009-2010	cross-sectional	school	IOTF	237	1159
Jain, et al	India	2010	2003-2004	cross-sectional	school	IOTF	721	2570
Gautam, et al	India	2019	2012	cross-sectional	school	IOTF	202	1185
Bharati, et al	India	2008	2005-2006	cross-sectional	school	IOTF	111	2255
Kotian, et al	India	2010	2007	cross-sectional	school	IOTF	132	900
Warraich, et al	India	2009	2009	cross-sectional	school	IOTF	40	284
Mushtag, et al	India	2011	2011	cross-sectional	school	IOTF	492	1860
Kumar, et al	India	2007	2007	cross-sectional	school	IOTF	86	1496
Tiwari, et al	India	2014	2010-2011	cross-sectional	school	IOTF	72	940
Bansal, et al	India	2022	2022	cross-sectional	school	National Reference	40	859
Baruah, et al	India	2018	2017	cross-sectional	school	IOTF	30	349
Baston, et al	India	2014	2009-2010	cross-sectional	school	IOTF	686	2130
Carrillo-	India	2014	2014	cohort	database	IOTF	31	1929

© 2024 Zhang X et al. *JAMA Pediatrics.*

Larco, et al (2)								
Khadilkar, et al	India	2011	2007-2008	cross-sectional	community	IOTF	3967	20243
Sultan, et al	India	2008	2008	cross-sectional	medical institution	IOTF	68	172
Gupta, et al	India	2011	2008-2009	cross-sectional	database	IOTF	1811	4908
Sharma, et al	India	2007	2007	cross-sectional	school	IOTF	1269	4399
Ramachandran, et al	India	2002	2002	cross-sectional	school	WHO	939	4700
Bose, et al	India	2007	2002	cross-sectional	school	IOTF	98	431
Mehta, et al	India	2007	2002	cross-sectional	school	IOTF	85	414
Laxmaiah, et al	India	2007	2003	cross-sectional	school	IOTF	102	1208
Raj, et al	India	2007	2003-2004	cross-sectional	school	IOTF	1540	24842
Iyer, et al	India	2011	2004	cross-sectional	school	IOTF	217	1067
Premanath, et al	India	2010	2005-2006	cross-sectional	school	IOTF	5130	43152
Marwaha, et al	India	2006	2006	cross-sectional	school	IOTF	3230	21485
Kumar, et al	India	2008	2007	cross-sectional	school	WHO	25	425
Sood, et al	India	2007	2007	cross-sectional	school	WHO	691	3970
Goyal, et al	India	2010	2010	cross-sectional	school	WHO	818	5664
Ghosh, et al	India	2011	2011	cross-sectional	school	IOTF	128	753
Mahajan, et al	India	2011	2008-2009	cross-sectional	school	IOTF	166	2542
Cherian, et al	India	2012	2009-2010	cross-sectional	school	IOTF	248	1634
Thakre, et al	India	2011	2009-2011	cross-sectional	school	IOTF	221	1524
Keerthan, et al	India	2011	2011	cross-sectional	school	IOTF	28	500
Vohra, et al	India	2011	2011	cross-sectional	school	IOTF	20	407
Chakraborty, et al	India	2011	2008-2009	cross-sectional	school	IOTF	203	979
Sidhu, et al	India	2005	2005	cross-sectional	school	WHO	106	640
Sidhu, et al	India	2006	2006	cross-sectional	school	CDC	194	1000
Misra, et al	India	2011	2006-2008	cross-sectional	school	IOTF	5514	38296
Jagadesan, et al	India	2014	2014	cross-sectional	school	WHO	3222	18955
Kaur, et al	India	2008	2008	cross-sectional	community	IOTF	1921	15996
Chhabra, et al	India	2012	2012	cross-sectional	medical institution	CDC	189	10000
Chhatwal, et al	India	2004	2004	cross-sectional	school	IOTF	285	2008
Saikia, et al	India	2018	2015-2016	cross-sectional	school	IOTF	341	1096
Anebaracy, et al	India	2019	2018	cross-sectional	medical institution	IOTF	180	3081
Chandra, et al	India	2019	2018	cross-sectional	school	IOTF	329	544
Minhas, et al	India	2019	2014-2015	cross-sectional	school	IOTF	324	1550
Mohan, et al	India	2019	2016-2017	cross-sectional	school	IOTF	312	1959
Pinni, et al	India	2019	2019	cross-sectional	community	CDC	38	1022
Sharma, et al	India	2019	2019	cross-sectional	community	WHO	202	1000
Swaminathan, et al	India	2019	2019	cross-sectional	school	CDC	298	2200
Singh, et al	India	2020	2020	cross-sectional	school	WHO	246	1030
Singh, et al	India	2020	2016	cross-sectional	school	WHO	360	1237
Solanki, et al	India	2020	2011-2013	cross-sectional	school	WHO	1417	10037
Ramesh, et al	India	2010	2008-2009	cross-sectional	school	IOTF	314	1718
Sharma, et al	India	2009	2009	cross-sectional	medical institution	CDC	165	500
Rao, et al	India	2009	2009	cross-sectional	school	WHO	293	2078
Ghosh, et al	India	2009	2009	cross-sectional	school	IOTF	113	1153
Singh, et al	India	2007	2007	cross-sectional	school	CDC	103	1083
Senbanjo, et al	India	2007	2007	cross-sectional	community	CDC	37	270
Katzmarzyk, et al (8)	India	2015	2011-2013	cross-sectional	database	National Reference	60	553

© 2024 Zhang X et al. *JAMA Pediatrics.*

Kasi, et al	India	2015	2008-2009	cross-sectional	school	CDC	164	1229
Joseph, et al	India	2015	2013	cross-sectional	school	CDC	49	300
Faizi, et al	India	2015	2011-2012	cross-sectional	school	CDC	207	1416
Basha, et al	India	2015	2015	cross-sectional	school	IOTF	371	1450
Weinmayr, et al (7)	India	2014	2004	cross-sectional	database	IOTF	7	119
Jani, et al	India	2014	2014	cross-sectional	medical institution	WHO	25	111
Gregori, et al	India	2014	2014	cross-sectional	database	WHO	377	1680
Ghosh, et al	India	2014	2008-2011	cross-sectional	school	WHO	255	1061
Aroor, et al	India	2014	2011	cross-sectional	school	IOTF	63	755
Honne, et al	India	2012	2012	cross-sectional	school	IOTF	102	463
Sakeenabi, et al	India	2012	2008	cross-sectional	school	IOTF	396	1550
Chakravathy, et al	India	2013	2013	cross-sectional	school	National Reference	157	456
Maiti, et al	India	2013	2010-2011	cross-sectional	school	WHO	121	1375
Bhargava, et al	India	2016	2013-2014	cross-sectional	school	IOTF	197	1266
Deepika, et al	India	2016	2013	cross-sectional	school	WHO	253	877
Pawar, et al	India	2016	2014-2015	cross-sectional	school	IOTF	269	1828
Prasad, et al	India	2016	2014	cross-sectional	school	IOTF	345	2465
Saikia, et al	India	2016	2015	cross-sectional	school	IOTF	242	752
Bharati, et al	India	2017	2017	cross-sectional	school	WHO	1690	5216
Choudhary, et al	India	2017	2016-2017	cross-sectional	school	WHO	90	540
Eshwar, et al	India	2017	2015	cross-sectional	school	IOTF	313	1496
Ganie, et al	India	2017	2011-2013	cross-sectional	school	IOTF	189	2024
Haq, et al	India	2017	2015	cross-sectional	medical institution	IOTF	35	303
Kumar, et al	India	2017	2017	cross-sectional	community	WHO	145	1092
Mishra, et al	India	2017	2012-2013	cross-sectional	school	IOTF	29	300
Shah, et al	India	2017	2011-2015	cross-sectional	community	IOTF	206	1034
Greene-Cramer, et al	India	2018	2007-2009	cross-sectional	school	IOTF	163	551
Andriani, et al	Indonesia	2021	2013	cross-sectional	database	WHO	4221	63237
Sarintohe, et al	Indonesia	2022	2019	cross-sectional	database	IOTF	149	411
Windiani, et al	Indonesia	2021	2019	cross-sectional	school	CDC	123	468
Aditya, et al	Indonesia	2017	2015	cross-sectional	school	IOTF	133	384
Agustina, et al	Indonesia	2021	2013	cross-sectional	database	IOTF	18463	108890
Collins, et al	Indonesia	2008	2002-2003	cross-sectional	school	IOTF	134	1736
Rachmi, et al (1)	Indonesia	2016	2000	cross-sectional	database	WHO	110	939
Rachmi, et al (2)	Indonesia	2016	2007	cross-sectional	database	WHO	216	1311
Romano, et al (11)	Indonesia	2022	2015	cross-sectional	database	IOTF	1436	8806
Yuwanita, et al	Indonesia	2018	2015	cross-sectional	school	WHO	31	150
Febriani, et al	Indonesia	2019	2017	cross-sectional	school	IOTF	60	145
Maehara, et al	Indonesia	2019	2017	cross-sectional	community	WHO	241	2160
Melinda, et al	Indonesia	2019	2015	cross-sectional	school	IOTF	22	107
Rizkiriani, et al	Indonesia	2014	2014	cross-sectional	school	WHO	49	213
Syahrul, et al	Indonesia	2016	2014	cross-sectional	community	WHO	179	877
Yulia, et al	Indonesia	2017	2016	cross-sectional	school	WHO	68	95
Dimaisip-Nabuab, et al (2)	Indonesia	2018	2012-2014	prospective	database	WHO	79	570
Kalsum, et	Indonesia	2018	2015	cross-sectional	school	WHO	35	149

© 2024 Zhang X et al. *JAMA Pediatrics.*

al								
Moradi, et al	Iran	2016	2015	cross-sectional	school	WHO	894	2506
Soheilipour, et al	Iran	2022	2022	cross-sectional	school	IOTF	292	829
Barati, et al	Iran	2022	2022	cross-sectional	school	WHO	404	1091
Mohammadi, et al	Iran	2021	2019	cross-sectional	school	IOTF	231	581
Entezarmahdi, et al	Iran	2021	2017	cross-sectional	community	WHO	34	2432
Jari, et al	Iran	2015	2009-2010	cross-sectional	database	IOTF	189	1090
Bagherian, et al	Iran	2013	2009	cross-sectional	school	WHO	152	400
Sadeghi, et al	Iran	2011	2008	cross-sectional	school	WHO	147	747
Shahraki, et al	Iran	2013	2013	cross-sectional	school	IOTF	157	1213
Agha-Alinejad, et al	Iran	2015	2011	cross-sectional	school	WHO	66	381
Javedan, et al	Iran	2016	2015	cross-sectional	school	WHO	4691	12796
Fatemeh, et al	Iran	2012	2008	cross-sectional	school	IOTF	91	500
Ahmadi, et al	Iran	2014	2014	cross-sectional	school	IOTF	345	1992
Badeli, et al	Iran	2016	2013-2015	cross-sectional	school	IOTF	348	2072
Bahreini, et al	Iran	2013	2010	cross-sectional	school	IOTF	274	3002
Bahreynian, et al	Iran	2015	2011-2012	cross-sectional	school	WHO	2877	13322
Basiratnia, et al	Iran	2013	2010-2011	cross-sectional	school	IOTF	400	2000
Maddah, et al	Iran	2010	2006-2007	cross-sectional	school	CDC	1231	6635
Kelishadi, et al	Iran	2008	2003-2004	cross-sectional	database	WHO	2998	21111
Amini, et al	Iran	2007	2000-2001	cross-sectional	school	WHO	103	398
Motlagh, et al	Iran	2011	2008	cross-sectional	database	WHO	139714	862433
Ziaoddini, et al	Iran	2010	2007	cross-sectional	database	WHO	152839	899054
Ayatollahi, et al	Iran	2007	2002-2003	cross-sectional	school	WHO	219	2195
Khodaverdi, et al	Iran	2011	2007	cross-sectional	school	WHO	68	240
Kajbaf, et al	Iran	2011	2009	cross-sectional	school	WHO	148	903
Vafa, et al	Iran	2012	2008	cross-sectional	school	WHO	101	511
Zarrati, et al	Iran	2013	2011-2012	cross-sectional	school	WHO	324	1184
Hajian-Tilaki, et al	Iran	2012	2008	cross-sectional	school	WHO	281	1200
Gaeini, et al	Iran	2011	2007-2008	cross-sectional	community	WHO	111	755
Montazerifar, et al	Iran	2009	2005-2006	cross-sectional	school	IOTF	61	590
Heidari, et al	Iran	2014	2010	cross-sectional	school	WHO	5087	12946
Hajian-Tilaki, et al	Iran	2011	2006	cross-sectional	school	CDC	181	1000
Gargari, et al	Iran	2004	2001	cross-sectional	school	National Reference	213	1518
Tebesh, et al	Iran	2013	2012-2013	cross-sectional	school	CDC	1566	5811
Shafaghi, et al	Iran	2014	2010-2011	cross-sectional	school	CDC	344	1189
Moayeri, et al	Iran	2006	2004-2005	cross-sectional	school	National Reference	700	2880
Behzadnia, et al	Iran	2012	2009-2010	cross-sectional	school	CDC	255	653
Maddah, et al	Iran	2010	2006-2007	cross-sectional	school	CDC	628	2577
Saeidlou, et al	Iran	2014	2011	cross-sectional	community	IOTF	46	902
Kavehmanesh, et al	Iran	2013	2009-2011	case-control	community	National Reference	110	259
Hajian-Tilaki, et al	Iran	2013	2012	cross-sectional	school	WHO	204	760

© 2024 Zhang X et al. *JAMA Pediatrics.*

Mirhosseini, et al	Iran	2012	2007	cross-sectional	school	CDC	86	477
Heshmat, et al	Iran	2015	2009-2010	cross-sectional	school	CDC	952	5625
Sadeghi, et al	Iran	2007	2007	cross-sectional	school	CDC	532	633
Zekavat, et al	Iran	2014	2008-2009	cross-sectional	school	IOTF	173	1156
Ejtahed, et al	Iran	2018	2015	cross-sectional	school	CDC	2945	14002
Salehi-Abargouei, et al	Iran	2013	2013	cross-sectional	school	CDC	137	837
Maddah, et al	Iran	2009	2006	cross-sectional	school	WHO	498	2091
Taheri, et al	Iran	2013	2012	cross-sectional	school	WHO	289	1541
Zakeri, et al	Iran	2012	2006-2007	cross-sectional	database	WHO	1396	8339
Jari, et al	Iran	2014	2009-2010	cross-sectional	database	WHO	923	5528
Sedaghat, et al	Iran	2019	2014-2015	cross-sectional	school	WHO	74	263
Rafraf, et al	Iran	2010	2008	cross-sectional	school	CDC	190	985
Maddah, et al	Iran	2010	2008-2009	cross-sectional	school	WHO	105	1079
Maddah, et al	Iran	2010	2006-2007	cross-sectional	school	WHO	1804	9046
Salem, et al	Iran	2009	2006-2007	cross-sectional	community	WHO	166	1221
Maddah, et al	Iran	2009	2005-2006	cross-sectional	school	WHO	519	2255
Kelishadi, et al	Iran	2009	2006-2007	cross-sectional	school	IOTF	672	1107
Fallahzadeh, et al	Iran	2009	2006	cross-sectional	school	IOTF	104	800
Azita, et al	Iran	2009	2006	cross-sectional	school	WHO	50	954
Bidad, et al	Iran	2008	2004-2005	cross-sectional	school	WHO	73	358
Rashidi, et al	Iran	2007	2000-2001	cross-sectional	school	WHO	534	2321
Mozaffari, et al	Iran	2007	2002	cross-sectional	school	WHO	378	1800
Maddah, et al	Iran	2007	2005	cross-sectional	school	WHO	287	1054
Tabriz, et al	Iran	2015	2009-2013	cross-sectional	community	WHO	252	1151
Ghadimi, et al	Iran	2015	2012	cross-sectional	school	WHO	953	3647
Jalali-Farahani, et al	Iran	2014	2014	cross-sectional	school	WHO	179	465
Hatami, et al	Iran	2014	2009-2010	cross-sectional	school	IOTF	230	1127
Ahmadi, et al	Iran	2014	2010	cross-sectional	school	WHO	34	145
Motlagh, et al (1)	Iran	2011	2007	cross-sectional	community	National Reference	139714	862433
Motlagh, et al (2)	Iran	2011	2008	cross-sectional	community	IOTF	132982	782244
Motlagh, et al (3)	Iran	2011	2009	cross-sectional	community	WHO	136620	955388
Hatami, et al	Iran	2013	2009-2010	cross-sectional	school	IOTF	152	739
Hassanzadeh-Rostami, et al	Iran	2016	2012-2013	cross-sectional	community	IOTF	956	6738
Keykhaei, et al	Iran	2016	2012	cross-sectional	school	IOTF	198	585
Saeidlou, et al	Iran	2016	2009-2011	prospective	school	WHO	6676	51583
Salehiniya, et al	Iran	2016	2012	cross-sectional	database	WHO	1592	4656
Darabiyan, et al	Iran	2018	2017	cross-sectional	school	WHO	98	239
Ghobadi, et al	Iran	2018	2015-2016	cross-sectional	school	WHO	106	607
Heshmat, et al	Iran	2018	2015	cross-sectional	database	CDC	762	3843
Motlagh, et al	Iran	2018	2015-2016	cross-sectional	community	WHO	595	2444

© 2024 Zhang X et al. *JAMA Pediatrics*.

Parastar, et al	Iran	2018	2016	cross-sectional	community	WHO	186	242
Abood, et al	Iraq	2021	2018-2019	cross-sectional	school	IOTF	100	357
Lafta, et al	Iraq	2005	2002	cross-sectional	school	WHO	499	8300
Amin, et al	Iraq	2019	2019	cross-sectional	school	IOTF	168	6418
Lafta, et al	Iraq	2007	2005	cross-sectional	school	IOTF	882	5361
Musaiger, et al (1)	Iraq	2016	2013-2014	cross-sectional	database	IOTF	227.808	904
Reulbach, et al	Ireland	2013	2007-2008	cross-sectional	school	IOTF	2506	8568
Quinn, et al	Ireland	2022	2011-2014	cohort	database	IOTF	518	4144
Heslin, et al (1)	Ireland	2023	2005-2006	cross-sectional	database	IOTF	75	440
Heslin, et al (2)	Ireland	2023	2019-2020	cross-sectional	database	IOTF	94	425
Barron, et al	Ireland	2009	2007	cross-sectional	school	IOTF	239	969
Dowd, et al	Ireland	2015	2006	cohort	community	IOTF	2044	7914
Evans, et al	Ireland	2011	2004-2007	cross-sectional	school	IOTF	930	3482
Whelton, et al	Ireland	2007	2001	cross-sectional	school	IOTF	4949	19538
O'Neill, et al	Ireland	2007	2003-2004	cross-sectional	school	IOTF	83	375
Kelly, et al	Ireland	2019	2019	longitudinal	database	IOTF	1323	8186
McMaster, et al	Ireland	2005	2001-2002	cross-sectional	school	IOTF	83	328
Janssen, et al (15)	Ireland	2005	2001-2002	cross-sectional	database	IOTF	156	1140
Belton, et al	Ireland	2010	2008	cross-sectional	school	IOTF	60	301
Haug, et al (33)	Ireland	2009	2005-2006	cross-sectional	database	IOTF	211	1566
Perry, et al	Ireland	2015	2007-2008	cross-sectional	school	IOTF	2096	8136
Pinhas-Hamiel, et al	Israel	2009	2009	cross-sectional	school	IOTF	82	204
Dayan, et al	Israel	2003	2003	cross-sectional	medical institution	IOTF	12099	76732
Janssen, et al (16)	Israel	2005	2001-2002	cross-sectional	database	IOTF	466	4200
Soskolne, et al	Israel	2018	2015	cross-sectional	medical institution	IOTF	203	946
Weisband, et al	Israel	2020	2018	cross-sectional	database	National Reference	21337	116774
Haug, et al (1)	Israel	2009	2005-2006	cross-sectional	database	IOTF	505	4037
Meyerovitch, et al	Israel	2007	2001-2004	cross-sectional	medical institution	IOTF	329	949
Goldberg, et al	Israel	2014	2014	cross-sectional	database	IOTF	58314	404922
Kaufman-Shriqui, et al	Israel	2013	2008	cross-sectional	school	National Reference	71	238
Ram, et al	Israel	2013	2009	cross-sectional	school	IOTF	173	4130
Ahrens, et al (1)	Italy	2014	2007-2008	cross-sectional	database	IOTF	1027	2424
Galfo, et al	Italy	2022	2011-2012	cross-sectional	database	IOTF	86	369
Salas, et al (7)	Italy	2021	2015-2017	cross-sectional	database	CDC	16743	42496
Ashi, et al (1)	Italy	2019	2019	cross-sectional	school	IOTF	46	220
Costacurta, et al	Italy	2011	2011	cross-sectional	medical institution	WHO	14	107
Barba, et al	Italy	2005	2000-2002	cross-sectional	school	WHO	883	1859
Barba, et al	Italy	2006	2003-2004	cross-sectional	school	WHO	1838	3923
Fioravanti, et al	Italy	2018	2003-2004	cohort	database	WHO	184	499
Ceschia, et al	Italy	2016	2016	cross-sectional	school	IOTF	607	2411
Toselli, et al	Italy	2014	2007-2008	cross-sectional	school	IOTF	419	1432
Caserta, et al	Italy	2010	2007-2008	cross-sectional	school	WHO	261	575
Genovesi, et al	Italy	2010	2004-2006	cross-sectional	school	CDC	1351	5131
Bertoncello, et al	Italy	2008	2004	cross-sectional	school	WHO	3434	12853
Albertini, et	Italy	2008	2002-2005	cross-sectional	school	CDC	1557	5636

© 2024 Zhang X et al. *JAMA Pediatrics.*

al									
Maffeis, et al	Italy	2006	2002	cross-sectional	school	IOTF	529	2150	
Parrino, et al	Italy	2012	2009-2010	cross-sectional	school	CDC	377	915	
Parrino, et al	Italy	2016	2016	cross-sectional	school	CDC	630	1521	
Lombardo, et al	Italy	2015	2010	cross-sectional	database	WHO	18092	41592	
Cibella, et al	Italy	2011	2005-2006	cross-sectional	school	IOTF	116	708	
Ruiz, et al (1)	Italy	2016	2007	cohort	database	IOTF	131	543	
Janssen, et al (17)	Italy	2005	2001-2002	cross-sectional	database	IOTF	712	4095	
Genovesi, et al	Italy	2005	2005	cross-sectional	school	IOTF	656	2416	
Valerio, et al	Italy	2003	2003	cross-sectional	school	IOTF	67	587	
Pecoraro, et al	Italy	2003	2003	cross-sectional	school	IOTF	110	228	
Collo, et al	Italy	2019	2016-2017	cross-sectional	medical institution	IOTF	15	86	
Toselli, et al	Italy	2010	2010	cross-sectional	school	IOTF	157	598	
Greco, et al	Italy	2020	2016-2017	cross-sectional	school	IOTF	523	1122	
Binkin, et al	Italy	2010	2008	cross-sectional	school	IOTF	17993	50197	
Rapa, et al	Italy	2009	2007-2008	cross-sectional	medical institution	IOTF	25	88	
Haug, et al (12)	Italy	2009	2005-2006	cross-sectional	database	IOTF	650	3556	
Caserta, et al	Italy	2009	2007-2008	cross-sectional	school	IOTF	283	642	
Bonaccorsi, et al	Italy	2009	2005	cross-sectional	school	IOTF	90	449	
Turconi, et al	Italy	2008	2002	cross-sectional	school	IOTF	109	532	
Maffeis, et al	Italy	2008	2003	cross-sectional	school	IOTF	480	1836	
Lazzeri, et al (1)	Italy	2008	2002	cross-sectional	school	IOTF	966	3048	
Lazzeri, et al (2)	Italy	2008	2006	cross-sectional	school	IOTF	478	1430	
Lazzeri, et al (3)	Italy	2008	2004	cross-sectional	school	CDC	220	1066	
Lazzeri, et al (4)	Italy	2008	2006	cross-sectional	school	CDC	196	997	
Lazzeri, et al (5)	Italy	2008	2004	cross-sectional	school	WHO	199	1185	
Lazzeri, et al (6)	Italy	2008	2006	cross-sectional	school	CDC	195	1086	
Lazzeri, et al (7)	Italy	2008	2004	cross-sectional	school	WHO	154	1160	
Lazzeri, et al (8)	Italy	2008	2006	cross-sectional	school	WHO	212	1078	
Guida, et al	Italy	2008	2001	cross-sectional	school	CDC	246	464	
Gualdi-Russo, et al	Italy	2008	2000-2001	cross-sectional	database	WHO	316	866	
Fuiano, et al	Italy	2008	2005	longitudinal	school	WHO	184	632	
Vidal, et al	Italy	2006	2006	cross-sectional	community	WHO	47	258	
Menghetti, et al	Italy	2015	2015	cross-sectional	school	WHO	680	2007	
Lazzeri, et al (1)	Italy	2015	2002	cross-sectional	database	CDC	476	1485	
Lazzeri, et al (2)	Italy	2015	2006	cross-sectional	database	IOTF	505	1405	
Lazzeri, et al (3)	Italy	2015	2008	cross-sectional	database	IOTF	406	1375	
Lazzeri, et al (4)	Italy	2015	2010	cross-sectional	database	IOTF	405	1424	
Lazzeri, et al (5)	Italy	2015	2012	cross-sectional	database	CDC	385	1494	
Geremia, et al	Italy	2015	2015	cross-sectional	school	IOTF	145	590	
Weinmayr, et al (8)	Italy	2014	2004	cross-sectional	database	IOTF	539	1307	
Lazzeri, et al	Italy	2014	2009-2010	cross-sectional	database	WHO	8469	47583	
Bo, et al	Italy	2014	2011-2012	cross-sectional	school	WHO	61	389	
Sacchetti, et al	Italy	2012	2012	cross-sectional	school	WHO	169	489	

© 2024 Zhang X et al. *JAMA Pediatrics*.

Bracale, et al	Italy	2013	2008	cross-sectional	community	WHO	3101	16588
Petracci, et al	Italy	2013	2003	cross-sectional	database	IOTF	1101	4338
Pileggi, et al	Italy	2013	2010-2011	cross-sectional	school	IOTF	199	542
Vijaykumar, et al	Italy	2013	2013	cross-sectional	school	IOTF	153	858
Galfo, et al	Italy	2016	2011-2012	cross-sectional	database	WHO	86	369
Grassi, et al	Italy	2016	2014-2015	cross-sectional	database	National Reference	334	1164
Romano, et al (12)	Jamaica	2022	2017	cross-sectional	database	WHO	273	1061
Fox, et al	Jamaica	2009	2009	cross-sectional	school	IOTF	336	3003
Sung, et al	Japan	2009	2002	cross-sectional	database	WHO	945	5753
Tsujiguchi, et al	Japan	2018	2013	cross-sectional	database	CDC	251	1374
Yaguchi-Tanaka, et al	Japan	2021	2001-2013	longitudinal	database	IOTF	2299	28772
Xu, et al (2)	Japan	2021	2014	cross-sectional	school	IOTF	169	1200
Tani, et al	Japan	2021	2018	cross-sectional	database	National Reference	1006	5257
Watanabe, et al	Japan	2011	2003	cross-sectional	school	IOTF	161	1765
Yoshitake, et al	Japan	2012	2006-2010	cross-sectional	school	IOTF	43	358
Oishi, et al	Japan	2021	2018-2019	cross-sectional	school	National Reference	505	7277
Kachi, et al	Japan	2015	2010	cross-sectional	database	IOTF	85	794
Nakano, et al	Japan	2010	2001	longitudinal	school	IOTF	3456	16245
Wang, et al	Japan	2007	2002	cohort	database	IOTF	1225	7959
Okabe, et al	Japan	2011	2008	cross-sectional	school	IOTF	20292	139117
Suzuki, et al	Japan	2009	2000-2001	cohort	database	IOTF	275	1302
Yamashita, et al	Japan	2023	2001	longitudinal	database	IOTF	3650	32081
Shirasawa, et al	Japan	2018	2011-2012	cross-sectional	school	IOTF	115	880
Tomata, et al	Japan	2019	2015	cross-sectional	medical institution	WHO	126	1848
Wada, et al	Japan	2019	2011-2015	prospective	school	National Reference	199	2596
Ochiai, et al	Japan	2020	2004-2007	prospective	school	IOTF	212	1436
Sasaki, et al	Japan	2010	2008	cross-sectional	community	IOTF	37	449
Okuda, et al	Japan	2010	2006-2008	cross-sectional	school	National Reference	361	3812
Ochiai, et al	Japan	2010	2004-2008	cross-sectional	school	IOTF	177	3750
Shirasawa, et al	Japan	2015	2003-2012	cross-sectional	school	IOTF	1062	8091
Shinozaki, et al	Japan	2015	2006-2010	cross-sectional	database	National Reference	607	5600
Ochiai, et al	Japan	2015	2004-2009	cross-sectional	school	IOTF	358	2499
Murakami, et al	Japan	2011	2004-2005	cross-sectional	database	National Reference	3186	24176
Itoi, et al	Japan	2012	2000	cross-sectional	school	WHO	32	227
Mizuta, et al	Japan	2016	2012-2013	cross-sectional	school	National Reference	188	2968
Ochiai, et al	Japan	2018	2001-2004	prospective	school	IOTF	254	1370
Baker, et al	Jordan	2010	2007	cross-sectional	school	IOTF	331	1355
Hamaideh, et al	Jordan	2010	2010	cross-sectional	school	IOTF	209	824
Ibrahim, et al	Jordan	2008	2006	cross-sectional	school	IOTF	617	1695
Khader, et al	Jordan	2009	2006	cross-sectional	school	WHO	530	2131
Al-Akour, et al	Jordan	2012	2009	cross-sectional	school	WHO	364	1433
Al-Domi, et al	Jordan	2019	2009-2011	cross-sectional	school	IOTF	379	767
Al-Kloub, et al	Jordan	2010	2008	cross-sectional	school	CDC	141	518
Tayyem, et al	Jordan	2014	2009	cross-sectional	database	IOTF	155	735
Musaiger, et	Jordan	2012	2010-2011	cross-sectional	school	CDC	252	937

© 2024 Zhang X et al. *JAMA Pediatrics*.

al (2)								
Musaiger, et al	Jordan	2013	2013	cross-sectional	school	IOTF	105	475
Musaiger, et al (2)	Jordan	2016	2013-2014	cross-sectional	database	IOTF	178.08	795
Zayed, et al	Jordan	2016	2015-2016	cross-sectional	school	IOTF	868	2702
Salas, et al (8)	Kazakhstan	2021	2015-2017	cross-sectional	database	IOTF	761	3988
Facchini, et al (1)	Kazakhstan	2007	2002-2004	cross-sectional	database	IOTF	112	2400
Tiruneh, er al (1)	Kenya	2021	2010-2019	cross-sectional	database	IOTF	792	19249
Adamo, et al	Kenya	2011	2011	cross-sectional	school	IOTF	12	179
Kyallo, et al	Kenya	2013	2008	cross-sectional	school	IOTF	61	321
Muthuri, et al	Kenya	2014	2012	cross-sectional	school	WHO	117	563
Gewa, et al	Kenya	2010	2003	cross-sectional	database	IOTF	257	1443
Katzmarzyk, et al (9)	Kenya	2015	2011-2013	cross-sectional	database	IOTF	33	499
Pengpid, et al (2)	Kiribati	2015	2010-2011	cross-sectional	database	IOTF	631	1582
Abdelalim, et al	Kuwait	2012	2012	cross-sectional	community	IOTF	418	1006
Alqaoud, et al	Kuwait	2022	2007-2019	cross-sectional	database	IOTF	5440	47782
Al-Haifi, et al	Kuwait	2022	2019	cross-sectional	school	IOTF	348	706
Badr, et al	Kuwait	2017	2010-2011	cross-sectional	database	IOTF	1229	2672
El-Ghaziri, et al	Kuwait	2011	2011	cross-sectional	school	IOTF	224	499
El-Bayoumy, et al	Kuwait	2009	2006	cross-sectional	school	IOTF	2447	5402
AlRodhan, et al	Kuwait	2019	2019	cross-sectional	school	IOTF	877	2208
Al-Qaoud, et al	Kuwait	2009	2003-2004	cross-sectional	school	WHO	461	2291
Al-Refaee, et al	Kuwait	2013	2012	cross-sectional	medical institution	IOTF	129	361
Musaiger, et al (1)	Kuwait	2013	2010-2011	cross-sectional	database	IOTF	312	628
Musaiger, et al (3)	Kuwait	2016	2013-2014	cross-sectional	database	IOTF	340.292	706
Salas, et al (9)	Kyrgyzstan	2021	2015-2017	cross-sectional	database	IOTF	590	5958
Romano, et al (13)	Laos	2022	2015	cross-sectional	database	IOTF	191	1644
Dimaisip-Nabuab, et al (3)	Laos	2018	2012-2014	prospective	database	WHO	35	653
Salas, et al (11)	Latvia	2021	2015-2017	cross-sectional	database	CDC	1398	5593
Janssen, et al (18)	Latvia	2005	2001-2002	cross-sectional	database	CDC	182	3091
Haug, et al (34)	Latvia	2009	2005-2006	cross-sectional	database	CDC	287	3778
Weinmayr, et al (9)	Latvia	2014	2004	cross-sectional	database	WHO	23	156
Karklina, et al	Latvia	2011	2007-2009	cross-sectional	school	IOTF	117	504
Nasreddine, et al	Lebanon	2014	2009	cross-sectional	community	IOTF	211	368
Chacar, et al	Lebanon	2011	2007	cross-sectional	school	CDC	690	2547
Romano, et al (14)	Lebanon	2022	2017	cross-sectional	database	IOTF	907	3347
Jabre, et al	Lebanon	2005	2000	cross-sectional	community	WHO	53	234
Fazah, et al	Lebanon	2010	2010	cross-sectional	school	WHO	209	982
Chakar, et al	Lebanon	2006	2002-2003	cross-sectional	school	WHO	3865	12128
Nasreddine, et al	Lebanon	2017	2011-2012	cross-sectional	community	National Reference	48	525
Gebremedhin, et al (12)	Liberia	2015	2013	cross-sectional	database	IOTF	156	3259
Taguri, et al (5)	Libya	2009	2003	cross-sectional	database	IOTF	963	7232
Musaiger, et	Libya	2012	2010-2011	cross-sectional	school	CDC	201	630

© 2024 Zhang X et al. *JAMA Pediatrics.*

al (3)								
Musaiger, et al (2)	Libya	2013	2010-2011	cross-sectional	database	IOTF	201	630
Musaiger, et al (4)	Libya	2016	2013-2014	cross-sectional	database	IOTF	191.268	759
Salas, et al (10)	Lithuania	2021	2015-2017	cross-sectional	database	WHO	896	3431
Smetanina, et al	Lithuania	2015	2008-2010	cross-sectional	school	IOTF	668	3990
Grazulevicie ne, et al	Lithuania	2014	2013	cohort	database	IOTF	111	1453
Janssen, et al (19)	Lithuania	2005	2001-2002	cross-sectional	database	IOTF	214	4194
Haug, et al (35)	Lithuania	2009	2005-2006	cross-sectional	database	CDC	275	3830
Wijnhoven, et al (3)	Lithuania	2015	2007/2008	cross-sectional	school	WHO	1282	4084
Haug, et al (7)	Luxemburg	2009	2005-2006	cross-sectional	database	IOTF	492	3904
Raufi, et al	Macedonia	2022	2019-2020	cross-sectional	medical institution	WHO	342	1034
Janssen, et al (20)	Macedonia	2005	2001-2002	cross-sectional	database	National Reference	464	3596
Haug, et al (13)	Macedonia	2009	2005-2006	cross-sectional	database	CDC	707	4911
Gontarev, et al	Macedonia	2018	2013	cross-sectional	school	WHO	3116	9081
Myrtaj, et al	Macedonia	2018	2016	cross-sectional	school	National Reference	569	2390
Manyanga, et al (5)	Malawi	2014	2014	cross-sectional	database	WHO	231	2305
Gebremedhin, et al (13)	Malawi	2015	2010	cross-sectional	database	National Reference	700	4829
Mohamed, et al	Malaysia	2023	2014-2015	cross-sectional	school	National Reference	221	917
Lai, et al	Malaysia	2022	2019	cross-sectional	school	CDC	708	2221
Al-Sadat, et al	Malaysia	2016	2012	cross-sectional	database	WHO	326	1350
Khor, et al	Malaysia	2011	2008	cross-sectional	school	IOTF	138	402
Adeyemi, et al	Malaysia	2014	2014	cross-sectional	school	IOTF	92	411
Ahmad, et al	Malaysia	2017	2014-2015	cross-sectional	database	National Reference	16893	62567
Tee, et al	Malaysia	2018	2013	cross-sectional	school	WHO	2364	8322
Kee, et al	Malaysia	2017	2013/2014	cross-sectional	database	WHO	125	663
Cheong, et al	Malaysia	2019	2011	cross-sectional	database	WHO	1386	5829
Moy, et al	Malaysia	2004	2004	cross-sectional	school	WHO	260	3556
Mahaletchumy, et al	Malaysia	2019	2016	cross-sectional	school	CDC	1715	6248
Cheah, et al	Malaysia	2019	2019	cross-sectional	school	WHO	485	1314
Tan, et al	Malaysia	2019	2012	cross-sectional	database	WHO	5699	24339
Muhammad, et al	Malaysia	2008	2008	cross-sectional	community	WHO	56	204
Zalilah, et al	Malaysia	2006	2006	cross-sectional	school	CDC	1189	6555
Mohd, et al	Malaysia	2006	2006	cross-sectional	school	IOTF	152	699
Woon, et al	Malaysia	2015	2015	cross-sectional	school	IOTF	94	333
Loh, et al	Malaysia	2015	2013	cross-sectional	school	CDC	172	646
Kaartina, et al	Malaysia	2015	2011	cross-sectional	school	IOTF	54	379
Zainuddin, et al	Malaysia	2014	2012	cross-sectional	school	IOTF	10855	39908
Su, et al	Malaysia	2014	2014	cross-sectional	school	WHO	326	1327
Rezali, et al	Malaysia	2012	2012	cross-sectional	school	National Reference	74	382
Naidu, et al	Malaysia	2013	2006	cross-sectional	database	IOTF	1498	7749
Poh, et al	Malaysia	2016	2015	cross-sectional	database	IOTF	767	3227
Shariff, et al	Malaysia	2016	2016	cross-sectional	community	IOTF	161	745
Partap, et al	Malaysia	2017	2013-2014	cross-sectional	database	WHO	2284	6414
Cazzaniga, et al	Maldives	2022	2018	cross-sectional	school	National Reference	20	145
Gebremedhi	Mali	2015	2012/2013	cross-sectional	database	CDC	230	4591

© 2024 Zhang X et al. *JAMA Pediatrics*.

n, et al (14)								
Salas, et al (12)	Malta	2021	2015-2017	cross-sectional	database	IOTF	1109	3115
Decelis, et al	Malta	2014	2012	cross-sectional	database	WHO	280	811
Janssen, et al (21)	Malta	2005	2001-2002	cross-sectional	database	IOTF	270	1065
Haug, et al (14)	Malta	2009	2005-2006	cross-sectional	database	WHO	303	1039
Decelis, et al	Malta	2013	2012	cross-sectional	school	IOTF	302	874
Manyanga, et al (6)	Mauritania	2014	2014	cross-sectional	database	IOTF	493	2028
Caleyachetty, et al	Mauritius	2012	2006	cross-sectional	school	IOTF	188	841
Fokeena, et al	Mauritius	2012	2012	cross-sectional	school	WHO	36	200
Romano, et al (15)	Mauritius	2022	2017	cross-sectional	database	CDC	483	1955
Shamah-Levy, et al (1)	Mexico	2022	2006	cross-sectional	database	IOTF	5402	14990
Shamah-Levy, et al (2)	Mexico	2022	2012	cross-sectional	database	IOTF	5642	16351
Shamah-Levy, et al (3)	Mexico	2022	2016	cross-sectional	database	IOTF	982	3179
Shamah-Levy, et al (4)	Mexico	2022	2018	cross-sectional	database	IOTF	2228	6183
Shamah-Levy, et al (5)	Mexico	2022	2020	cross-sectional	database	IOTF	739	1944
Shamah-Levy, et al (6)	Mexico	2022	2021	cross-sectional	database	IOTF	992	2569
Brambila-Paz, et al	Mexico	2022	2002	longitudinal	database	IOTF	101	3202
Aburto, et al	Mexico	2015	2011-2012	cross-sectional	database	IOTF	799	2367
Aguilera-Galaviz, et al	Mexico	2019	2016	cross-sectional	school	IOTF	51	203
Ashi, et al (2)	Mexico	2019	2019	cross-sectional	school	IOTF	93	224
Pérez, et al	Mexico	2020	2018	cross-sectional	school	IOTF	197	522
Irigoyen-Camacho, et al	Mexico	2014	2010-2011	cross-sectional	school	IOTF	77	257
Lara-Capi, et al	Mexico	2018	2018	cross-sectional	school	IOTF	271	464
Patiño-Marín, et al	Mexico	2018	2013-2016	cross-sectional	school	IOTF	640	1527
Sánchez-Pérez, et al	Mexico	2010	2010	longitudinal	school	IOTF	26	88
Serrano-Piña, et al	Mexico	2019	2019	cross-sectional	school	IOTF	129	331
Vázquez-Nava, et al	Mexico	2010	2005	cross-sectional	school	WHO	537	1160
Ávila-Ortiz, et al	Mexico	2017	2011-2013	cross-sectional	school	IOTF	49	91
Bacardi-Gascón, et al	Mexico	2009	2006-2007	cross-sectional	school	IOTF	832	1684
Bacardi-Gascón, et al	Mexico	2012	2008-2010	RCT	school	National Reference	241	532
Lopez-Gonzalez, et al	Mexico	2020	2015-2019	cross-sectional	community	National Reference	469	1449
Del-Rio-Navarro, et al	Mexico	2008	2005	cross-sectional	school	WHO	864	1819

© 2024 Zhang X et al. *JAMA Pediatrics.*

Gómez-Díaz, et al	Mexico	2005	2005	cross-sectional	school	IOTF	127	833
Brewis, et al	Mexico	2003	2001	cross-sectional	school	IOTF	53	219
Vergara-Castañeda, et al	Mexico	2010	2010	cross-sectional	school	IOTF	33	83
Jimenez-Cruz, et al	Mexico	2010	2009	cross-sectional	medical institution	WHO	186	1685
Varela-Silva, et al	Mexico	2009	2006	cross-sectional	community	IOTF	68	206
Macías-Rosales, et al	Mexico	2009	2004	cross-sectional	medical institution	IOTF	177	641
Flores-Huerta, et al	Mexico	2009	2005-2006	cross-sectional	school	IOTF	744	2029
Ortiz-Hernández, et al	Mexico	2008	2005	cross-sectional	school	CDC	566	1015
Basaldúa, et al	Mexico	2008	2004	cross-sectional	school	WHO	207	551
Villa-Caballero, et al	Mexico	2006	2001-2002	cross-sectional	school	IOTF	483	1172
RODRÍGUEZ-FUENTES, et al	Mexico	2015	2011	cross-sectional	school	CDC	42	104
López-Barrón, et al	Mexico	2015	2010-2011	cross-sectional	school	WHO	368	684
Flores, et al	Mexico	2015	2008	cross-sectional	medical institution	IOTF	129	164
Banik, et al	Mexico	2014	2008-2009	cross-sectional	school	CDC	107	321
Banik, et al	Mexico	2014	2014	cross-sectional	school	National Reference	104	283
Barrera, et al	Mexico	2016	2012-2013	cross-sectional	school	National Reference	330	725
Caraza, et al	Mexico	2016	2013-2014	cross-sectional	school	WHO	161	605
Peña, et al	Mexico	2018	2018	cross-sectional	school	WHO	495	849
Ramírez-Jiménez ,et al	Mexico	2018	2011	cross-sectional	community	WHO	21	93
Romano, et al (16)	Mongolia	2022	2013	cross-sectional	database	National Reference	441	3707
Salas, et al (13)	Montenegro	2021	2015-2017	cross-sectional	database	National Reference	889	2678
Banjari, et al (1)	Montenegro	2020	2020	cross-sectional	school	National Reference	68	224
Martinovic, et al	Montenegro	2015	2012-2013	cross-sectional	school	National Reference	937	4097
Mehdad, et al	Morocco	2022	2018-2019	cross-sectional	school	National Reference	112	292
El Moussaoui, et al	Morocco	2022	2020	cross-sectional	medical institution	National Reference	196	450
Achouri, et al	Morocco	2021	2021	cross-sectional	school	IOTF	47	271
Dekkaki, et al	Morocco	2011	2010	cross-sectional	school	IOTF	136	1570
Manyanga, et al (7)	Morocco	2014	2014	cross-sectional	database	IOTF	956	5756
Taguri, et al (2)	Morocco	2009	2003-2004	cross-sectional	database	IOTF	832	5380
El Kabbaoui, et al	Morocco	2018	2014-2015	cross-sectional	school	CDC	202	1818
Tiruneh, er al (4)	Mozambique	2021	2010-2019	cross-sectional	database	National Reference	697	9423
Gebremedhin, et al (15)	Mozambique	2015	2011	cross-sectional	database	National Reference	1157	9721
Qiao, et al	Multiple Countries	2015	2011-2013	cross-sectional	database	National Reference	639	4502
Bishwajt, et al	Multiple Countries	2019	2014-2018	cross-sectional	database	IOTF	26593	132231
Stival, et al	Multiple	2022	2019	cross-sectional	database	WHO	692	3093

© 2024 Zhang X et al. *JAMA Pediatrics.*

(1)	Countries							
Stival, et al (2)	Multiple Countries	2022	2018	cross-sectional	database	IOTF	408	2916
Moschonis, et al	Multiple Countries	2022	2016-2018	cross-sectional	database	IOTF	3067	12030
Ferrari, et al	Multiple Countries	2022	2014-2015	cross-sectional	database	WHO	170	671
Collings, et al	Multiple Countries	2022	2022	cross-sectional	database	WHO	4274	15810
Ho, et al	Multiple Countries	2021	2012-2014	cross-sectional	database	IOTF	1098	7108
Peltzer, et al	Multiple Countries	2011	2003/2007	cross-sectional	school	WHO	413	5613
Vrijheid, et al	Multiple Countries	2020	2013/2016	cohort	database	IOTF	503	1301
Roy, et al	Multiple Countries	2020	2003-2014	cohort	database	National Reference	276	3679
Ekelund, et al	Multiple Countries	2004	2004	cross-sectional	school	WHO	191	1292
McCabe, et al	Multiple Countries	2009	2009	cross-sectional	school	IOTF	665	2223
Velde, et al	Multiple Countries	2007	2003	cross-sectional	school	WHO	1951	12538
Blaženčić-Mladenović, et al	Multiple Countries	2006	2006	cross-sectional	school	IOTF	227	1555
Olaya, et al	Multiple Countries	2015	2010	cross-sectional	database	IOTF	1067	5206
Katzmarzyk, et al (1)	Multiple Countries	2015	2011-2013	cross-sectional	database	National Reference	815	6539
Tsitsika, et al	Multiple Countries	2016	2011-2012	cross-sectional	school	WHO	1275	10287
Qiao, et al	Multiple Countries	2017	2013	cross-sectional	database	National Reference	626	5088
Gebremedhin, et al (16)	Namibia	2015	2013	cross-sectional	database	WHO	110	1845
Romano, et al (17)	Namibia	2022	2013	cross-sectional	database	WHO	147	1936
Achaya, et al	Nepal	2016	2013	cross-sectional	school	IOTF	68	838
Piryani, et al	Nepal	2016	2014	cross-sectional	school	IOTF	35	268
Romano, et al (18)	Nepal	2022	2015	cross-sectional	database	IOTF	332	4616
Bhattarai, et al	Nepal	2019	2017	cross-sectional	school	IOTF	20	510
Karki, et al	Nepal	2019	2017	cross-sectional	school	IOTF	148	575
Koirala, et al	Nepal	2015	2013	cross-sectional	school	IOTF	255	986
Gurung, et al	Nepal	2014	2010	cross-sectional	school	IOTF	46	300
Pandey, et al	Nepal	2018	2018	cross-sectional	school	IOTF	16	120
Greeff, et al	Netherlands	2016	2016	RCT	school	IOTF	84	376
Veldhuis, et al	Netherlands	2012	2007-2008	cross-sectional	database	WHO	658	7505
Jabakhanji, et al	Netherlands	2022	2013	cohort	database	IOTF	1739	7042
Vinke, et al	Netherlands	2021	2006-2007	cohort	database	IOTF	167	1001
Sergentanis, et al (2)	Netherlands	2021	2011-2012	cross-sectional	database	CDC	55	727
Croezen, et al	Netherlands	2009	2003	cross-sectional	database	National Reference	1969	25176
Drukker, et al	Netherlands	2009	2004-2005	cross-sectional	database	CDC	171	1411
Jansen, et al	Netherlands	2012	2004-2010	cross-sectional	database	IOTF	287	3157
Wilde, et al	Netherlands	2009	2007	cross-sectional	database	IOTF	1973	10308
Yngve, et al (5)	Netherlands	2008	2003	cross-sectional	database	IOTF	62	684
Jong, et al	Netherlands	2013	2016	cross-sectional	database	IOTF	476	4072
Jansen, et al	Netherlands	2006	2003/2004	cross-sectional	school	IOTF	33	499
Steur, et al	Netherlands	2011	2004-2005	cohort	database	IOTF	235	1687
Akbulut, et al (2)	Netherlands	2014	2003-2011	cross-sectional	database	IOTF	173	433
Mbakwa, et	Netherlands	2016	2000-2010	cohort	database	IOTF	69	768

© 2024 Zhang X et al. *JAMA Pediatrics*.

al								
Jansen, et al	Netherlands	2008	2000-2001	cross-sectional	database	IOTF	558	1923
Scholtens, et al	Netherlands	2009	2002-2004	cohort	database	IOTF	245	2366
Timmermans, et al	Netherlands	2014	2007-2008	cohort	database	CDC	72	1143
Vrijkotte, et al	Netherlands	2020	2003-2004	cohort	database	IOTF	487	3714
Janssen, et al (22)	Netherlands	2005	2001-2002	cross-sectional	database	IOTF	301	3860
Wilde, et al	Netherlands	2019	2007-2014	cross-sectional	medical institution	IOTF	21900	109769
Luttikhuis, et al	Netherlands	2010	2006	cross-sectional	school	IOTF	36	397
Jansen, et al	Netherlands	2010	2006	cross-sectional	school	IOTF	333	1095
Haug, et al (8)	Netherlands	2009	2005-2006	cross-sectional	database	WHO	316	3850
Bossink-Tuna, et al	Netherlands	2009	2006	cross-sectional	medical institution	WHO	46	635
Snoek, et al	Netherlands	2007	2007	cross-sectional	school	WHO	889	9011
Scholtens, et al	Netherlands	2007	2000-2001	cross-sectional	database	CDC	116	864
Jansen, et al	Netherlands	2006	2000-2001	cross-sectional	database	WHO	524	1819
Wijtzes, et al	Netherlands	2014	2002-2006	cross-sectional	community	IOTF	1043	5913
Weinmayr, et al (10)	Netherlands	2014	2004	cross-sectional	database	WHO	511	2638
Roos, et al (2)	Netherlands	2014	2009	cross-sectional	database	IOTF	50	503
Bere, et al	Netherlands	2011	2005-2006	cross-sectional	database	IOTF	496	2558
Schwiebbe, et al	Netherlands	2011	2008	cross-sectional	school	IOTF	605	2148
de Jong, et al	Netherlands	2012	2006	cross-sectional	database	National Reference	475	4072
Veldwijk, et al	Netherlands	2012	2003-2007	cross-sectional	school	National Reference	4666	51856
Willeboordse, et al	Netherlands	2013	2010	cross-sectional	community	IOTF	1637	9272
Leppers, et al	Netherlands	2017	2012-2015	cross-sectional	medical institution	IOTF	499	3408
Velde, et al (4)	Netherlands	2017	2010	cross-sectional	database	WHO	138	919
Ridder, et al	Netherlands	2018	2010-2011	cross-sectional	school	IOTF	202	1084
Utter, et al	New Zealand	2006	2002	cross-sectional	database	IOTF	820	2716
Hobbs, et al	New Zealand	2021	2013-2017	cross-sectional	database	IOTF	3751	9728
Aung, et al	New Zealand	2021	2014-2015	cross-sectional	school	IOTF	6912	27333
Chelimo, et al	New Zealand	2020	2007-2010	cohort	medical institution	IOTF	1488	5128
Rockell, et al	New Zealand	2005	2002	cross-sectional	school	IOTF	1306	3275
Gordon, et al	New Zealand	2003	2000	cross-sectional	community	IOTF	17	41
Utter, et al	New Zealand	2010	2007	cross-sectional	school	IOTF	3008	8796
Quigg, et al	New Zealand	2010	2007	cross-sectional	school	IOTF	56	184
Weinmayr, et al (11)	New Zealand	2014	2004	cross-sectional	database	IOTF	77	222
Rajput, et al	New Zealand	2014	2009-2012	cross-sectional	database	IOTF	58385	168744
Gebremedhin, et al (17)	Niger	2015	2012	cross-sectional	database	IOTF	292	5123
Adeomi, et al	Nigeria	2019	2019	cross-sectional	school	IOTF	30	309
Adetunji, et al	Nigeria	2019	2013	cross-sectional	school	WHO	58	1187
Maruf, et al	Nigeria	2013	2009	cross-sectional	school	IOTF	623	9014
Ene-obong, et al	Nigeria	2012	2012	cross-sectional	school	IOTF	226	1599
Omigbodun, et al	Nigeria	2010	2010	cross-sectional	school	IOTF	38	1503

© 2024 Zhang X et al. *JAMA Pediatrics*.

Opara, et al	Nigeria	2010	2009	cross-sectional	school	WHO	56	985
Senbanjo, et al	Nigeria	2010	2010	cross-sectional	school	IOTF	6	392
Gebremedhin, et al (18)	Nigeria	2015	2013	cross-sectional	database	IOTF	2087	26758
Goon, et al	Nigeria	2010	2005	cross-sectional	school	IOTF	99	2015
Fetuga, et al	Nigeria	2011	2008	cross-sectional	school	IOTF	27	1690
Wariri, et al	Nigeria	2020	2015-2017	cross-sectional	community	IOTF	192	2100
Mezie-Okoye, et al	Nigeria	2015	2010	cross-sectional	school	CDC	52	220
Nwizu, et al	Nigeria	2011	2006	cross-sectional	community	CDC	60	377
Musa, et al	Nigeria	2012	2012	cross-sectional	school	CDC	475	3240
Oduwole, et al	Nigeria	2012	2012	cross-sectional	school	IOTF	205	885
Sadoh, et al	Nigeria	2016	2011-2012	cross-sectional	school	IOTF	119	353
Igbokwe, et al	Nigeria	2017	2013	cross-sectional	school	IOTF	108	860
Aadland, et al	Norway	2021	2015-2016	cross-sectional	community	CDC	216	1182
Aadland, et al	Norway	2022	2019-2022	cross-sectional	community	IOTF	174	1003
Donkor, et al	Norway	2017	2007	cohort	school	IOTF	293	1864
Øvrebø, et al	Norway	2021	2017	longitudinal	database	IOTF	290	1838
Andersen, et al	Norway	2005	2000	cross-sectional	school	WHO	253	1489
Grydeland, et al	Norway	2014	2007	RCT	school	IOTF	170	1324
Bernhardsen, et al	Norway	2019	2013-2015	cohort	database	WHO	59	445
Oellingrath, et al	Norway	2010	2007	cross-sectional	school	National Reference	188	955
Júlíusson, et al	Norway	2010	2003-2006	cross-sectional	community	National Reference	990	6140
Kolle, et al	Norway	2009	2005	cross-sectional	database	IOTF	81	440
Júlíusson, et al (2)	Norway	2015	2015	cross-sectional	database	National Reference	851	6139
Yngve, et al (6)	Norway	2008	2003	cross-sectional	database	CDC	98	670
Gebremariam, et al	Norway	2015	2009	cross-sectional	database	National Reference	91	828
Fasting, et al	Norway	2009	2007	cohort	database	National Reference	63	711
Hestetun, et al	Norway	2015	2010	cross-sectional	school	IOTF	126	744
Janssen, et al (23)	Norway	2005	2001-2002	cross-sectional	database	IOTF	552	4415
Vik, et al	Norway	2010	2005	cross-sectional	school	National Reference	301	2870
Bjelland, et al	Norway	2010	2007	cross-sectional	school	IOTF	209	1481
Haug, et al (36)	Norway	2009	2005-2006	cross-sectional	database	IOTF	382	3675
Grøholt, et al	Norway	2008	2000-2004	cross-sectional	school	National Reference	1924	15966
Weinmayr, et al (12)	Norway	2014	2004	cross-sectional	database	IOTF	131	637
Brannsether, et al	Norway	2014	2003-2006	cross-sectional	database	IOTF	652	4576
Velde, et al (5)	Norway	2017	2010	cross-sectional	database	National Reference	140	1000
Tanveer, et al	Pakistan	2022	2021	cross-sectional	school	National Reference	396	3551
Bekhwani, et al	Pakistan	2022	2020-2021	cross-sectional	medical institution	National Reference	105	184
Ahmed, et al	Pakistan	2013	2008-2009	cross-sectional	school	National Reference	98	501
Mansoori, et al	Pakistan	2018	2012	cross-sectional	school	WHO	265	887
Anwar, et al	Pakistan	2010	2009	cross-sectional	school	WHO	99	293
Hashmi, et al	Pakistan	2013	2008-2009	cross-sectional	school	National Reference	156	501
Jafar, et al	Pakistan	2008	2004-2005	cross-sectional	database	National	95	1675

© 2024 Zhang X et al. *JAMA Pediatrics*.

						Reference		
Ramzan, et al	Pakistan	2008	2008	cross-sectional	school	IOTF	193	1136
Aziz, et al	Pakistan	2009	2007	cross-sectional	school	WHO	101	398
Haq, et al	Pakistan	2010	2010	cross-sectional	school	WHO	153	3200
Aziz, et al	Pakistan	2012	2006-2009	cross-sectional	school	WHO	664	12837
Hydrie, et al	Pakistan	2005	2001-2002	cross-sectional	school	WHO	15	107
Basit,et al	Pakistan	2005	2001	cross-sectional	school	WHO	5	92
Ishaque, et al	Pakistan	2012	2012	cross-sectional	school	WHO	122	431
Salah, et al	Palestine	2021	2013	cross-sectional	school	IOTF	566	2400
Abudayya, et al	Palestine	2007	2002	cross-sectional	school	WHO	184	1022
AbuKishk, et al	Palestine	2021	2019	cross-sectional	community	WHO	56	367
Badrasawi, et al	Palestine	2019	2017	cross-sectional	school	National Reference	67	392
Al-Lahham, et al	Palestine	2019	2016	cross-sectional	school	IOTF	398	1320
Mikki, et al	Palestine	2009	2007-2008	cross-sectional	school	National Reference	344	1942
Al Sabbah, et al	Palestine	2009	2001-2002	cross-sectional	database	WHO	2121	12847
Weinmayr, et al (13)	Palestine	2014	2004	cross-sectional	database	CDC	35	216
Musaiger, et al (3)	Palestine	2013	2010-2011	cross-sectional	database	IOTF	80	477
Massad, et al	Palestine	2016	2009	cross-sectional	school	WHO	266	1484
Musaiger, et al (5)	Palestine	2016	2013-2014	cross-sectional	database	WHO	136.64	610
Bartkowiak, et al	Peru	2021	2021	cross-sectional	school	IOTF	150	681
Carrillo-Larco, et al (1)	Peru	2014	2002	cohort	database	WHO	408	1929
Romano, et al (19)	Peru	2022	2010	cross-sectional	database	IOTF	486	2359
Echevarría-Castro ,et al	Peru	2020	2016	cross-sectional	database	IOTF	357	7935
Preston, et al	Peru	2015	2009	cross-sectional	community	WHO	483	1737
Hernández-Vásquez, et al	Peru	2016	2015	cross-sectional	community	WHO	35493	2336791
Romano, et al (20)	Philippines	2022	2015	cross-sectional	database	IOTF	660	6162
Suligowska, et al	Poland	2022	2017-2019	cross-sectional	database	IOTF	100	381
Potempa-Jeziorowska , et al	Poland	2022	2018-2019	cross-sectional	school	National Reference	155	908
Kryst, et al (1)	Poland	2022	2009-2010	cross-sectional	school	National Reference	401	1926
Kryst, et al (2)	Poland	2022	2019-2020	cross-sectional	school	CDC	380	1850
Bryl, et al	Poland	2022	2017-2019	cross-sectional	school	WHO	108	530
Basiak-Rasała, et al	Poland	2022	2016	cross-sectional	school	WHO	437	2913
Wyszyńska, et al	Poland	2021	2018-2019	cross-sectional	school	CDC	76	676
Wieniawski, et al	Poland	2021	2021	cross-sectional	school	WHO	123	690
Usheva, et al (5)	Poland	2021	2012	cross-sectional	database	CDC	113	1334
Sergentanis, et al (4)	Poland	2021	2011-2012	cross-sectional	database	IOTF	145	1190
Salas, et al (14)	Poland	2021	2015-2017	cross-sectional	database	CDC	882	2884
Matłosz, et al	Poland	2021	2018-2019	cross-sectional	school	IOTF	129	1172
Żegleń, et al (1)	Poland	2020	2008	cross-sectional	school	CDC	196	1113

© 2024 Zhang X et al. *JAMA Pediatrics.*

Żegleń, et al (2)	Poland	2020	2018	cross-sectional	school	CDC	124	1054
Szczyrska, et al	Poland	2020	2007-2016	cross-sectional	database	CDC	1446	12330
Tomaszewski, et al	Poland	2015	2015	cross-sectional	school	WHO	161	791
Bac, et al	Poland	2012	2008-2009	cross-sectional	community	IOTF	406	1495
Baran, et al	Poland	2022	2015	cross-sectional	school	IOTF	117	749
Kułaga, et al	Poland	2011	2007-2009	cross-sectional	database	IOTF	2544	15607
Malecka-Tendera, et al	Poland	2005	2001	cross-sectional	school	IOTF	451	2916
Kowal,et al (1)	Poland	2014	2000	cross-sectional	school	CDC	239	2062
Kowal,et al (2)	Poland	2014	2010	cross-sectional	school	CDC	282	1970
Chrzanowska, et al	Poland	2007	2000	cross-sectional	school	WHO	305	2003
Ządzińska, et al	Poland	2013	2002-2004	cross-sectional	community	IOTF	117	812
Gorog, et al (4)	Poland	2011	2011	cross-sectional	database	IOTF	286	1454
Høyer, et al (2)	Poland	2014	2002-2005	cohort	database	National Reference	17	92
Zatoński, et al	Poland	2020	2016-2017	cross-sectional	school	WHO	434	2913
Janssen, et al (24)	Poland	2005	2001-2002	cross-sectional	database	IOTF	502	5909
Woźniacka, et al	Poland	2018	2009-2010	cross-sectional	school	National Reference	730	3405
Baran, et al	Poland	2019	2019	cross-sectional	school	CDC	26	300
Jodkowska, et al	Poland	2010	2005	cross-sectional	school	National Reference	1161	8065
Haug, et al (22)	Poland	2009	2005-2006	cross-sectional	database	IOTF	570	5324
Zawodniak-Szałapska, et al	Poland	2007	2007	cross-sectional	school	National Reference	183	1012
Klimek-Piotrowska, et al	Poland	2015	2012-2013	cross-sectional	school	National Reference	140	970
Długosz, et al	Poland	2015	2010-2011	cross-sectional	school	IOTF	91	553
Stankiewicz, et al	Poland	2014	2014	cross-sectional	community	WHO	213	1515
Kowalkowska, et al	Poland	2014	2010-2011	cross-sectional	school	WHO	170	1176
Januszek-Trzciąkowska, et al	Poland	2014	2001	cross-sectional	school	National Reference	94	2571
Golec, et al	Poland	2014	2012	cross-sectional	school	IOTF	87	305
Rutkowski, et al	Poland	2013	2006-2010	cross-sectional	school	IOTF	143	889
Czyż, et al	Poland	2017	2012-2014	cross-sectional	school	CDC	110	641
Kantanista, et al	Poland	2017	2017	cross-sectional	school	WHO	511	3249
Andaki, et al (2)	Portugal	2017	2009-2015	cross-sectional	community	WHO	834	4052
Rodrigues, et al (1)	Portugal	2022	2009/2010	cross-sectional	school	CDC	474	1996
Rodrigues, et al (2)	Portugal	2022	2016/2017	cross-sectional	school	IOTF	375	2077
Salas, et al (15)	Portugal	2021	2015-2017	cross-sectional	database	National Reference	1815	5992
Paciência, et al	Portugal	2021	2014-2015	cross-sectional	school	National Reference	292	845
Nazareth, et al	Portugal	2021	2012	cross-sectional	database	National Reference	97	1665
Abreu, et al	Portugal	2019	2011-2012	cross-sectional	school	CDC	135	412
Abreu, et al	Portugal	2014	2008	cross-sectional	school	National Reference	372	1209
Frias-Bulhosa, et	Portugal	2015	2015	cross-sectional	school	WHO	50	181

© 2024 Zhang X et al. *JAMA Pediatrics.*

al								
Abreu, et al	Portugal	2014	2008	cross-sectional	database	National Reference	146	503
Padez, et al	Portugal	2005	2002-2003	cross-sectional	school	WHO	1424	4511
Araújo, et al	Portugal	2012	2003-2004	cross-sectional	school	WHO	281	1171
Abreu, et al	Portugal	2012	2012	longitudinal	database	WHO	312	1001
Silva-Santos, et al	Portugal	2017	2012	cross-sectional	school	WHO	151	467
Sardinha, et al	Portugal	2011	2008	cross-sectional	school	WHO	4983	22048
Seabra, et al	Portugal	2013	2010	cross-sectional	school	IOTF	278	682
Rito, et al	Portugal	2012	2007-2008	cross-sectional	school	IOTF	1058	3765
Antunes, et al	Portugal	2015	2006	cross-sectional	database	CDC	324	1273
Ferreira, et al	Portugal	2008	2004	cross-sectional	school	CDC	401	1125
Marques-Vidal, et al	Portugal	2008	2000-2002	cross-sectional	school	WHO	1182	5013
Yngve, et al (7)	Portugal	2008	2003	cross-sectional	database	IOTF	308	1176
Cabral, et al	Portugal	2016	2003-2004	cross-sectional	school	WHO	153	1547
Ribeiro, et al	Portugal	2020	2012-2013	cohort	database	IOTF	803	5203
Mota, et al	Portugal	2008	2006	cross-sectional	school	CDC	180	886
Minghelli, et al	Portugal	2014	2014	cross-sectional	school	National Reference	305	966
Ruiz, et al (3)	Portugal	2016	2009	cohort	database	WHO	1183	5685
Albuquerque, et al	Portugal	2012	2011	cross-sectional	school	WHO	473	1433
Padez, et al	Portugal	2004	2002-2003	cross-sectional	school	National Reference	1424	4511
Vale, et al	Portugal	2010	2006-2007	cross-sectional	school	National Reference	211	788
Gama, et al	Portugal	2020	2009-2010	cross-sectional	community	IOTF	4846	17277
Pereira, et al	Portugal	2010	2010	cross-sectional	school	IOTF	745	3699
Padez, et al	Portugal	2009	2002-2003	cross-sectional	school	IOTF	1382	4347
Haug, et al (15)	Portugal	2009	2005-2006	cross-sectional	database	IOTF	517	3566
Fonseca, et al	Portugal	2009	2002	cross-sectional	database	IOTF	980	5470
Rebelo, et al	Portugal	2008	2000-2002	cross-sectional	school	National Reference	1288	5083
Aires, et al	Portugal	2008	2008	cross-sectional	school	WHO	147	636
Moreira, et al	Portugal	2007	2002-2003	cross-sectional	school	IOTF	1494	4845
Ribeiro, et al	Portugal	2006	2001	cross-sectional	school	IOTF	222	819
Mota, et al	Portugal	2006	2006	cross-sectional	school	IOTF	109	255
Wijnhoven, et al (4)	Portugal	2015	2007/2008	cross-sectional	school	IOTF	1580	3026
Vale, et al	Portugal	2015	2009-2013	cross-sectional	school	IOTF	231	733
Minghelli, et al	Portugal	2015	2015	cross-sectional	school	IOTF	230	966
Katzmarzyk, et al (10)	Portugal	2015	2011-2013	cross-sectional	database	IOTF	118	686
Roos, et al (6)	Portugal	2014	2009	cross-sectional	database	IOTF	211	703
Pedrosa, et al	Portugal	2011	2005-2006	cross-sectional	school	IOTF	254	905
Valente, et al	Portugal	2011	2011	cross-sectional	community	IOTF	631	1675
Vasques, et al	Portugal	2012	2008	cross-sectional	school	IOTF	572	1786
Nogueira, et al	Portugal	2013	2009	cross-sectional	school	IOTF	505	1885
Marques, et al	Portugal	2016	2010	cross-sectional	database	IOTF	541	2938
Marques, et al	Portugal	2018	2018	cross-sectional	school	IOTF	316	1396
Santiago, et al	Puerto Rico	2021	2014-2017	cross-sectional	school	IOTF	808	3145
Rivera-Soto, et al	Puerto Rico	2010	2010	cross-sectional	school	National Reference	95	250

© 2024 Zhang X et al. *JAMA Pediatrics*.

Rodriguez, et al	Puerto Rico	2008	2008	cross-sectional	school	CDC	121	234
Elías-Boneta, et al	Puerto Rico	2015	2010-2011	cross-sectional	school	IOTF	656	1582
Torres, et al	Puerto Rico	2014	2012-2013	cross-sectional	school	IOTF	41	114
Cheema, et al	Qatar	2022	2018-2020	cross-sectional	school	IOTF	198	459
Kerkadi, et al	Qatar	2019	2013-2014	cross-sectional	school	IOTF	480	1161
Bener, et al	Qatar	2005	2003-2004	cross-sectional	school	IOTF	1963	7442
Bener, et al	Qatar	2006	2003-2004	cross-sectional	school	IOTF	1179	3923
Passmore, et al	Republic of Marshall Islands	2019	2017-2018	cross-sectional	school	IOTF	436	3271
Sergentanis, et al (5)	Romania	2021	2011-2012	cross-sectional	database	IOTF	132	1240
Salas, et al (16)	Romania	2021	2015-2017	cross-sectional	database	IOTF	1653	5885
Barbu, et al	Romania	2015	2010-2011	cross-sectional	school	National Reference	274	866
Pop, et al	Romania	2021	2016	cross-sectional	school	IOTF	6403	21625
Valean, et al	Romania	2009	2009	cross-sectional	school	IOTF	1671	7904
Mocanu, et al	Romania	2013	2008-2012	cross-sectional	school	National Reference	817	3444
Emandi, et al	Romania	2012	2010-2011	cross-sectional	school	National Reference	919	3626
Cinteza, et al	Romania	2013	2006-2008	cross-sectional	school	National Reference	801	4866
Gorog, et al (5)	Romania	2011	2011	cross-sectional	database	National Reference	126	1359
Haug, et al (23)	Romania	2009	2005-2006	cross-sectional	database	National Reference	508	4450
Salas, et al (17)	Russia	2021	2015-2017	cross-sectional	database	CDC	498	2001
Janssen, et al (25)	Russia	2005	2001-2002	cross-sectional	database	CDC	440	7450
Khasnutdinova, et al	Russia	2010	2006	cross-sectional	school	CDC	96	1066
Haug, et al (24)	Russia	2009	2005-2006	cross-sectional	database	CDC	655	6833
Facchini, et al (2)	Russia	2007	2002-2004	cross-sectional	database	CDC	169	2408
Gebremedhin, et al (19)	Rwanda	2015	2010	cross-sectional	database	CDC	400	4116
Choy, et al	Samoa	2022	2015	cohort	database	CDC	32	197
Pengpid, et al (3)	Samoa	2015	2010-2011	cross-sectional	database	CDC	1255	2418
Choy, et al	Samoa	2017	2015	cross-sectional	community	CDC	62	305
Salas, et al (18)	San Marino	2021	2015-2017	cross-sectional	database	CDC	101	303
Abdellatif, et al	Saudi Arabia	2020	2020	cross-sectional	community	CDC	112	2247
Mohamed, et al	Saudi Arabia	2022	2022	cross-sectional	school	CDC	402	1250
Abu El Qomsan, et al	Saudi Arabia	2017	2017	cross-sectional	school	National Reference	263	386
Ashi, et al (3)	Saudi Arabia	2019	2019	cross-sectional	school	CDC	104	225
Alghamdi, et al	Saudi Arabia	2017	2015	cross-sectional	school	CDC	202	610
Bhayat, et al	Saudi Arabia	2016	2014	cross-sectional	school	CDC	221	402
Farsi, et al	Saudi Arabia	2017	2014-2015	cross-sectional	school	CDC	323	801
Farsi, et al	Saudi Arabia	2016	2014-2015	cross-sectional	school	CDC	327	915
Quadri, et al	Saudi Arabia	2017	2017	cross-sectional	school	IOTF	40	360
Bahathig, et al	Saudi Arabia	2021	2019	cross-sectional	school	CDC	62	399
Amin, et al	Saudi Arabia	2008	2008	cross-sectional	school	CDC	272	1139
Bajamal, et al	Saudi Arabia	2017	2017	cross-sectional	school	CDC	97	383
Bawazeer, et al	Saudi Arabia	2009	2007	cross-sectional	school	CDC	1859	5498
Abalkhail, et	Saudi Arabia	2002	2000	cross-sectional	school	CDC	521	1993

© 2024 Zhang X et al. *JAMA Pediatrics.*

al								
Al-Attas, et al	Saudi Arabia	2010	2010	cross-sectional	medical institution	National Reference	52	148
Alwan, et al	Saudi Arabia	2013	2006	cross-sectional	school	National Reference	413	1212
Al-Almaie, et al	Saudi Arabia	2005	2001	cross-sectional	school	CDC	486	1766
Mustafa, et al	Saudi Arabia	2021	2019	cross-sectional	school	CDC	103	300
Al-Agha, et al	Saudi Arabia	2016	2015	cross-sectional	medical institution	CDC	352	365
Al-Musharaf, et al	Saudi Arabia	2012	2010	cross-sectional	medical institution	CDC	124	331
Alselaim, et al	Saudi Arabia	2012	2001-2009	cohort	medical institution	CDC	55	933
Washi, et al	Saudi Arabia	2010	2006	cross-sectional	school	CDC	54	239
Al-Hussaini, et al	Saudi Arabia	2019	2015	cross-sectional	school	National Reference	2504	7931
Fakeeh, et al	Saudi Arabia	2019	2017	cross-sectional	community	CDC	67	298
Mouzan, et al	Saudi Arabia	2010	2005	cross-sectional	community	CDC	4597	19317
Al-Dossary, et al	Saudi Arabia	2010	2006	cross-sectional	medical institution	CDC	2985	7056
Al-Saeed, et al	Saudi Arabia	2007	2003	cross-sectional	school	CDC	702	2239
Al-Muhaimeed, et al	Saudi Arabia	2015	2012	cross-sectional	school	National Reference	98	601
Akinpelu, et al	Saudi Arabia	2014	2014	cross-sectional	school	CDC	71	1903
El Mouzan, et al	Saudi Arabia	2012	2004-2005	cross-sectional	community	CDC	2037	11112
Al Dahi, et al	Saudi Arabia	2016	2015-2016	cross-sectional	school	CDC	75	200
Musaiger, et al (6)	Saudi Arabia	2016	2013-2014	cross-sectional	database	CDC	363.968	968
Gebremedhin, et al (20)	Senegal	2015	2010/2011	cross-sectional	database	CDC	128	6062
Šušnjević, et al	Serbia	2021	2013	cross-sectional	database	CDC	359	1376
Djordjic, et al	Serbia	2016	2015	cross-sectional	school	CDC	1123	4861
Rakić, et al	Serbia	2019	2012-2017	cross-sectional	community	CDC	294	1592
Bukara-Radujković, et al	Serbia	2009	2004	cross-sectional	school	CDC	220	1204
Markovic, et al	Serbia	2015	2012-2014	cross-sectional	medical institution	CDC	274	406
Rakic, et al	Serbia	2011	2001-2004	cross-sectional	school	CDC	395	2650
Srdić, et al	Serbia	2012	2012	cross-sectional	medical institution	CDC	522	2284
Janic, et al	Serbia	2013	2013	cross-sectional	school	CDC	2818	11644
Bjelanovic, et al	Serbia	2017	2013-2014	cross-sectional	school	CDC	1008	6444
Halasi, et al	Serbia	2018	2018	cross-sectional	school	National Reference	38	182
Romano, et al (21)	Seychelles	2022	2015	cross-sectional	database	CDC	608	2061
Chiolero, et al	Seychelles	2007	2002-2004	cross-sectional	community	CDC	2482	15612
Bovet, et al	Seychelles	2007	2004	cross-sectional	school	CDC	620	4343
Bovet, et al	Seychelles	2010	2004-2006	cross-sectional	school	CDC	2103	8462
Gebremedhin, et al (21)	Sierra Leone	2015	2013	cross-sectional	database	CDC	794	4698
Yeo, et al	Singapore	2019	2016-2017	cross-sectional	school	CDC	181	2313
Fu, et al	Singapore	2003	2003	cross-sectional	school	CDC	43	623
Pwint, et al	Singapore	2013	2006-2008	cross-sectional	database	CDC	342	2256
Sabanayagam, et al	Singapore	2009	2001-2004	cross-sectional	database	CDC	179	797
Deurenberg-Yap, et al	Singapore	2009	2009	cross-sectional	school	CDC	447	6991
Gorog, et al (6)	Slovakia	2011	2011	cross-sectional	database	CDC	188	1390

© 2024 Zhang X et al. *JAMA Pediatrics*.

Haug, et al (25)	Slovakia	2009	2005-2006	cross-sectional	database	CDC	318	3688
Janssen, et al (27)	Slovenia	2005	2001-2002	cross-sectional	database	CDC	561	3769
Planinsec, et al	Slovenia	2004	2004	cross-sectional	school	CDC	88	364
Planinsec, et al	Slovenia	2009	2006	cross-sectional	school	CDC	1404	5613
Haug, et al (16)	Slovenia	2009	2005-2006	cross-sectional	database	CDC	745	4874
Sedej, et al (1)	Slovenia	2014	2001	cross-sectional	medical institution	CDC	513	2742
Sedej, et al (2)	Slovenia	2014	2004	cross-sectional	medical institution	CDC	891	4684
Sedej, et al (3)	Slovenia	2014	2009	cross-sectional	medical institution	CDC	1131	5406
Roos, et al (7)	Slovenia	2014	2009	cross-sectional	database	CDC	247	1121
Sedej, et al (1)	Slovenia	2016	2004	cross-sectional	school	CDC	602	2641
Sedej, et al (2)	Slovenia	2016	2009	cross-sectional	school	CDC	2235	6861
Sedej, et al (3)	Slovenia	2016	2014	cross-sectional	school	CDC	2727	8036
Velde, et al (6)	Slovenia	2017	2010	cross-sectional	database	CDC	307	1176
Pengpid, et al (4)	Solomon Islands	2015	2010-2011	cross-sectional	database	CDC	307	1421
Symington, et al	South Africa	2015	2005	cross-sectional	database	CDC	62	519
Kirsten, et al	South Africa	2013	2013	cross-sectional	school	CDC	84	638
Nomatshila, et al	South Africa	2022	2022	cross-sectional	database	CDC	28	209
Engwa, et al	South Africa	2022	2022	cross-sectional	school	CDC	77	540
Abrahams, et al	South Africa	2011	2008	cross-sectional	school	CDC	135	643
Armstrong, et al	South Africa	2006	2001-2004	cross-sectional	school	CDC	1621	10283
Baard, et al	South Africa	2014	2014	cross-sectional	school	CDC	219	713
Moselakgamo, et al	South Africa	2015	2015	cross-sectional	school	CDC	22	1361
Oldewage-Theron, et al	South Africa	2010	2010	cross-sectional	school	CDC	21	142
Pedro, et al	South Africa	2014	2009	cross-sectional	database	CDC	48	588
Pienaar, et al	South Africa	2015	2013	longitudinal	database	CDC	96	574
Puckree, et al	South Africa	2011	2006	cross-sectional	school	CDC	6	120
Tathiah, et al	South Africa	2013	2011	cross-sectional	database	CDC	116	959
Truter, et al	South Africa	2015	2015	cross-sectional	school	CDC	61	280
Wiles, et al	South Africa	2013	2013	cross-sectional	school	CDC	168	311
Negash, et al	South Africa	2017	2007-2008	cross-sectional	school	CDC	357	1559
Kimani-Murage, et al	South Africa	2011	2007	cross-sectional	school	CDC	71	944
Reddy, et al (1)	South Africa	2012	2002	cross-sectional	database	CDC	1895	9522
Reddy, et al (2)	South Africa	2012	2008	cross-sectional	database	CDC	2408	9371
Moselakgomo, et al	South Africa	2017	2017	cross-sectional	school	CDC	211	1361
Modjadji, et al	South Africa	2019	2017	cross-sectional	school	CDC	22	508
Pretorius, et al	South Africa	2019	2019	prospective	medical institution	IOTF	487	1785
Monyeki, et al	South Africa	2006	2000	cross-sectional	school	CDC	26	1884
Kruger, et al	South Africa	2006	2000-2001	cross-sectional	school	CDC	99	1257
Nkeh-Chungag, et al	South Africa	2015	2015	cross-sectional	school	CDC	164	392
Meko, et al	South Africa	2015	2015	cross-sectional	school	CDC	92	414

© 2024 Zhang X et al. *JAMA Pediatrics*.

Katzmarzyk, et al (11)	South Africa	2015	2011-2013	cross-sectional	database	CDC	52	468
Van Niekerk, et al	South Africa	2014	2014	cross-sectional	school	CDC	110	689
Toriola, et al	South Africa	2012	2010	cross-sectional	school	National Reference	176	1172
Craig, et al	South Africa	2016	2016	cross-sectional	school	CDC	180	1310
Choo, et al	South Korea	2020	2017	RCT	school	CDC	37	104
Park, et al (1)	South Korea	2022	2019	cross-sectional	database	IOTF	541140	2612812
Park, et al (2)	South Korea	2022	2020	cross-sectional	database	CDC	572468	2568754
Kim, et al	South Korea	2021	2017-2019	cross-sectional	database	CDC	410	2136
Park, et al	South Korea	2013	2011	cross-sectional	school	CDC	161	939
Bae, et al	South Korea	2021	2012-2013	cross-sectional	database	IOTF	197	2893
Baek, et al	South Korea	2012	2009	cross-sectional	database	CDC	13328	72399
Kim, et al	South Korea	2012	2012	cohort	database	CDC	13321	72399
Byun, et al	South Korea	2012	2005	cross-sectional	database	CDC	141	577
Lee, et al	South Korea	2013	2006-2010	cross-sectional	database	CDC	241	1649
Lee, et al	South Korea	2013	2011	cohort	database	National Reference	27	205
Bae, et al	South Korea	2010	2008	cross-sectional	school	CDC	58	379
Lee, et al	South Korea	2013	2013	cross-sectional	school	CDC	97	422
Choo, et al	South Korea	2017	2015	cross-sectional	community	CDC	37	126
Le, et al (1)	South Korea	2020	2007-2009	cross-sectional	database	IOTF	973	5415
Le, et al (2)	South Korea	2020	2010-2012	cross-sectional	database	CDC	872	4936
Le, et al (3)	South Korea	2020	2013-2015	cross-sectional	database	CDC	714	4131
Lee, et al	South Korea	2012	2008	cohort	school	CDC	179	1030
Kim, et al	South Korea	2005	2002	cross-sectional	school	CDC	246	1107
Sunwoo, et al	South Korea	2020	2011	cross-sectional	school	CDC	1375	22906
Yoo, et al	South Korea	2020	2015	cross-sectional	school	CDC	212	1621
Lee, et al	South Korea	2010	2005	cross-sectional	database	CDC	245	928
Kim, et al	South Korea	2010	2005	cross-sectional	community	CDC	40	124
Lee, et al	South Korea	2009	2006	cross-sectional	school	CDC	687	5443
Chang, et al	South Korea	2008	2008	cross-sectional	school	CDC	1122	4033
Ryu, et al	South Korea	2007	2007	cross-sectional	school	CDC	264	1393
Yoo, et al	South Korea	2006	2006	cross-sectional	school	CDC	388	938
Lee, et al	South Korea	2006	2001	cross-sectional	school	CDC	269	3059
Yoo, et al	South Korea	2015	2006	cross-sectional	school	CDC	360	2004
Lee, et al	South Korea	2015	2015	cross-sectional	school	CDC	599	4895
Kong, et al	South Korea	2015	2013	cross-sectional	database	CDC	7918	53769
Noh, et al	South Korea	2014	2009	cross-sectional	database	CDC	1758	9411
Oh, et al	South Korea	2011	2007	cross-sectional	database	CDC	5645	60643
Yoo, et al	South Korea	2011	2011	cross-sectional	school	CDC	239	717
Kim, et al	South Korea	2012	2008	cross-sectional	school	CDC	313	1644
Choi, et al	South Korea	2013	2010-2011	cross-sectional	community	CDC	358	2038
Kim, et al	South Korea	2017	2011-2015	cross-sectional	database	CDC	9528	136739
Cho, et al	South Korea	2018	2007-2014	cross-sectional	database	CDC	1326	7197
Ara, et al	Spain	2007	2007	cross-sectional	school	CDC	404	1068
Sánchez-Cruz, et al	Spain	2013	2012	cross-sectional	community	CDC	377	978
Gulías-González, et al	Spain	2014	2010	cross-sectional	community	CDC	760	2301
Ahrens, et al (8)	Spain	2014	2007-2008	cross-sectional	database	CDC	326	1539
Iguacel, et al	Spain	2018	2009-2010	cohort	database	CDC	325	1031
López-Gil, et al	Spain	2022	2022	cross-sectional	school	WHO	1106	2890
Lasarte-Velillas, et al	Spain	2022	2003-2018	cross-sectional	database	CDC	50135	161335
Bont, et al	Spain	2022	2005-2017	longitudinal	database	National Reference	891951	2504568
Cartanyà-Hueso, et al	Spain	2022	2017	cross-sectional	database	CDC	1252	4528
Cabeza, et al	Spain	2022	2022	cross-sectional	school	CDC	106	212
Aragón-Martín, et al	Spain	2022	2018	cross-sectional	school	CDC	302	864
Vega-Ramirez, et	Spain	2021	2021	cross-sectional	school	CDC	107	287

© 2024 Zhang X et al. *JAMA Pediatrics*.

al								
Usheva, et al (6)	Spain	2021	2012	cross-sectional	database	CDC	98	713
Sergentanis, et al (6)	Spain	2021	2011-2012	cross-sectional	database	CDC	152	1295
Salas, et al (19)	Spain	2021	2015-2017	cross-sectional	database	CDC	4137	10239
Barja-Fernández, et al	Spain	2018	2018	cross-sectional	medical institution	CDC	346	471
Adelantado-Renau, et al	Spain	2018	2015	longitudinal	school	CDC	33	263
Aguilar, et al	Spain	2010	2004	RCT	school	CDC	124	921
Marcos-Pasero, et al	Spain	2019	2017	longitudinal	school	CDC	47	201
Baile, et al	Spain	2020	2020	cross-sectional	school	CDC	100	1197
Bawaked, et al	Spain	2020	2003-2008	longitudinal	database	CDC	450	1480
Bazán, et al	Spain	2018	2011	cross-sectional	database	IOTF	888	3752
Bont, et al	Spain	2019	2012-2013	cross-sectional	database	CDC	1123	2660
Tamayo-Ortiz, et al	Spain	2021	2012	cross-sectional	database	CDC	505	1233
Martín, et al	Spain	2008	2004-2006	cross-sectional	database	CDC	394	1312
Llargues, et al	Spain	2011	2006	RCT	school	IOTF	139	508
Santiago, et al	Spain	2013	2008	cross-sectional	medical institution	IOTF	878	2814
Yngve, et al (8)	Spain	2008	2003	cross-sectional	database	CDC	155	745
Riaño-Galán, et al	Spain	2017	2003-2008	cohort	community	CDC	202	1044
Montero, et al	Spain	2005	2005	cross-sectional	school	CDC	86	229
Pérez-Bonaventura, et al	Spain	2015	2009-2012	cross-sectional	community	CDC	53	611
Garcia-Marcos, et al	Spain	2008	2008	cross-sectional	school	CDC	1689	17145
Garcia-Marcos, et al	Spain	2008	2008	cross-sectional	school	CDC	128	931
Ruiz, et al (2)	Spain	2016	2001	cohort	database	IOTF	338	1726
Janssen, et al (28)	Spain	2005	2001-2002	cross-sectional	database	IOTF	836	4445
Vázquez, et al	Spain	2010	2010	cross-sectional	school	CDC	896	2305
Albaladejo, et al	Spain	2019	2004-2006	cross-sectional	database	CDC	317	1181
Scholz, et al	Spain	2019	2009-2012	cross-sectional	database	CDC	347	1744
Esteban-Vasallo, et al	Spain	2020	2012	cross-sectional	database	CDC	368	2914
Martinez-Gomez, et al	Spain	2010	2007-2008	cross-sectional	community	National Reference	19	192
Bibiloni, et al	Spain	2010	2007-2008	cross-sectional	community	CDC	343	1231
Haug, et al (17)	Spain	2009	2005-2006	cross-sectional	database	CDC	1277	7380
Larrañaga, et al	Spain	2007	2004-2005	cross-sectional	community	CDC	334	1178
Ayala, et al	Spain	2007	2007	cross-sectional	community	CDC	80	154
Rojo, et al	Spain	2006	2003-2004	cross-sectional	database	CDC	5744	35403
Morales-Suárez-Varela, et al	Spain	2015	2013-2014	cross-sectional	database	National Reference	274	710
Gutiérrez-Zornoza, et al	Spain	2015	2006	cross-sectional	school	National Reference	264	956
Alonso-Fernández, et al (1)	Spain	2015	2006	cross-sectional	database	National Reference	1061	5108
Alonso-Fernández,	Spain	2015	2011-2012	cross-sectional	database	National Reference	771	3265

© 2024 Zhang X et al. *JAMA Pediatrics*.

et al (2)								
Veses, et al	Spain	2014	2000-2002	cross-sectional	database	National Reference	343	1554
Weinmayr, et al (14)	Spain	2014	2004	cross-sectional	database	National Reference	430	968
Tobarra, et al	Spain	2014	2010	cross-sectional	school	National Reference	137	373
Nova, et al	Spain	2014	2007-2008	cross-sectional	database	National Reference	359	2054
Dadvand, et al	Spain	2014	2006	cross-sectional	database	National Reference	628	3178
Rodríguez-Ramírez, et al	Spain	2011	2006	cross-sectional	database	CDC	2104	8252
Cerrillo, et al	Spain	2012	2012	cross-sectional	school	CDC	360	990
Martínez-Vizcaíno , et al (1)	Spain	2012	2004	cross-sectional	school	CDC	352	1119
Martínez-Vizcaíno , et al (2)	Spain	2012	2010	cross-sectional	school	CDC	379	1070
Navalpotro, et al	Spain	2012	2006-2007	cross-sectional	database	CDC	1542	4528
Jiménez-Ormeño, et al	Spain	2013	2013	cross-sectional	school	CDC	353	1032
Laguna, et al	Spain	2013	2013	cross-sectional	database	CDC	217	761
Morales, et al	Spain	2013	2010-2011	cross-sectional	school	CDC	411	1158
Moreno, et al	Spain	2013	2008-2009	cross-sectional	database	CDC	850	2316
Pérez-Farinós, et al	Spain	2013	2010-2011	cross-sectional	database	CDC	2696	7659
García-García, et al	Spain	2016	2012	cross-sectional	community	CDC	411	1317
Alvarez Zallo ,et al	Spain	2017	2017	cross-sectional	database	CDC	1046	8607
Martín-Espinosa, et al	Spain	2017	2013	cross-sectional	school	CDC	322	1604
Velde, et al (7)	Spain	2017	2010	cross-sectional	database	CDC	249	1022
Ramos, et al	Spain	2018	2015	cross-sectional	school	CDC	71	235
Rathnayake, et al	Sri Lanka	2013	2013	cross-sectional	medical institution	CDC	80	1087
Wickramasinghe, et al	Sri Lanka	2013	2009-2010	cross-sectional	school	CDC	25	920
Warnakulasuriya, et al	Sri Lanka	2019	2019	cross-sectional	school	CDC	1934	12788
Wickramasinghe, et al	Sri Lanka	2009	2004-2005	cross-sectional	school	CDC	17	282
Salman, et al	Sudan	2010	2010	cross-sectional	school	CDC	71	304
Nagwa, et al	Sudan	2011	2011	cross-sectional	school	CDC	233	1138
Musaiger, et al (7)	Sudan	2016	2013-2014	cross-sectional	database	CDC	94.71	902
Romano, et al (22)	Suriname	2022	2016	cross-sectional	database	IOTF	423	1453
Ahrens, et al (5)	Sweden	2014	2007-2008	cross-sectional	database	WHO	201	1824
Wallby, et al	Sweden	2017	2002-2007	cohort	database	IOTF	3992	30508
Hamano, et al	Sweden	2017	2005-2010	cohort	database	IOTF	6968	944487
Önnestam, et al	Sweden	2022	2015	longitudinal	medical institution	WHO	66	314
Fäldt, et al (1)	Sweden	2022	2015-2019	cross-sectional	database	WHO	3357	23214
Fäldt, et al (2)	Sweden	2022	2020-2021	cross-sectional	database	IOTF	2990	19171
Sjöberg, et al	Sweden	2005	2004	cross-sectional	database	IOTF	776	4703

© 2024 Zhang X et al. *JAMA Pediatrics*.

Norberg, et al	Sweden	2012	2004	cross-sectional	medical institution	IOTF	177	920
Nilsen, et al	Sweden	2017	2008-2010	cross-sectional	community	IOTF	477	2620
Moraeus, et al	Sweden	2014	2008/2010/2013	cross-sectional	school	IOTF	761	3492
Garmy, et al	Sweden	2014	2008-2009	cross-sectional	school	IOTF	464	2891
Sjöberg, et al	Sweden	2008	2000-2005	cross-sectional	school	WHO	1559	8876
Mårild, et al	Sweden	2004	2000	cross-sectional	community	WHO	1001	4730
Yngve, et al (9)	Sweden	2008	2003	cross-sectional	database	WHO	124	888
Mangrio, et al	Sweden	2010	2003-2008	cross-sectional	medical institution	WHO	1272	8621
Koch, et al	Sweden	2008	2001	cohort	database	IOTF	282	6733
Stenhammar, et al	Sweden	2010	2007	cross-sectional	community	Diagnostic Reference	182	873
Börnhorst, et al	Sweden	2015	2006	cross-sectional	database	WHO	553	2441
White, et al (1)	Sweden	2022	2022	cohort	database	WHO	487	3237
Magnusson, et al	Sweden	2005	2005	cross-sectional	school	IOTF	31	108
Janssen, et al (29)	Sweden	2005	2001-2002	cross-sectional	database	IOTF	393	3507
Erling, et al	Sweden	2004	2004	cross-sectional	school	IOTF	103	960
Litsfeldt, et al	Sweden	2020	2015-2017	cross-sectional	school	IOTF	203	1363
Lager,et al	Sweden	2009	2003-2006	cross-sectional	school	IOTF	1668	7584
Haug, et al (38)	Sweden	2009	2005-2006	cross-sectional	database	CDC	414	3974
Guinhouya, et al	Sweden	2009	2005-2006	cross-sectional	school	IOTF	38	113
Bergström, et al (1)	Sweden	2009	2002-2003	cross-sectional	medical institution	CDC	1062	4407
Blomquist, et al	Sweden	2007	2007	cross-sectional	medical institution	CDC	1052	4407
Wijnhoven, et al (5)	Sweden	2015	2007/2008	cross-sectional	school	CDC	1079	3633
Moraeus, et al (1)	Sweden	2015	2008	cross-sectional	school	CDC	147	833
Moraeus, et al (2)	Sweden	2015	2010	cross-sectional	school	CDC	213	1085
Moraeus, et al (3)	Sweden	2015	2013	cross-sectional	school	National Reference	217	1134
Lindkvist, et al	Sweden	2015	2008-2012	cross-sectional	community	WHO	214	697
Weinmayr, et al (15)	Sweden	2014	2004	cross-sectional	database	WHO	122	459
Roos, et al (1)	Sweden	2014	2009	cross-sectional	database	IOTF	72	652
Vaezghasemi, et al	Sweden	2012	2007	cross-sectional	school	IOTF	776	4987
Khanolkar, et al	Sweden	2013	2013	cross-sectional	community	CDC	1777	10628
Munter, et al (1)	Sweden	2016	2003	cross-sectional	community	IOTF	1138	7728
Munter, et al (2)	Sweden	2016	2011	cross-sectional	community	IOTF	1856	12882
Winkvist, et al	Sweden	2016	2008	cross-sectional	community	IOTF	1672	11222
Bergström, et al (2)	Sweden	2009	2007-2008	cross-sectional	medical institution	IOTF	834	4381
Aeberli, et al (1)	Switzerland	2010	2007	cross-sectional	school	IOTF	337	2222
Aeberli, et al (2)	Switzerland	2010	2002	cross-sectional	school	IOTF	466	2404
Aeberli, et al	Switzerland	2010	2009	cross-sectional	database	IOTF	158	900
Jeannot, et al	Switzerland	2015	2011-2012	cross-sectional	school	IOTF	1449	8544
Lasserre, et al	Switzerland	2007	2005-2006	cross-sectional	school	IOTF	713	5207
Janssen, et al (30)	Switzerland	2005	2001-2002	cross-sectional	database	IOTF	372	4190

© 2024 Zhang X et al. *JAMA Pediatrics*.

Zimmermann, et al	Switzerland	2004	2004	cross-sectional	school	IOTF	630	2431
Köchli, et al	Switzerland	2019	2019	cross-sectional	database	IOTF	165	1246
Haug, et al (9)	Switzerland	2009	2005-2006	cross-sectional	database	WHO	353	4251
Aeberli, et al	Switzerland	2013	2013	cross-sectional	school	IOTF	243	2303
Bonvin, et al	Switzerland	2012	2012	cross-sectional	school	WHO	68	529
Velde, et al (8)	Switzerland	2017	2010	cross-sectional	database	CDC	84	596
Nasreddine, et al	Syria	2010	2010	cross-sectional	school	IOTF	214	776
Taguri, et al (1)	Syria	2009	2001	cross-sectional	database	IOTF	1100	5454
Musaiger, et al (4)	Syria	2012	2010-2011	cross-sectional	school	IOTF	278	1062
Chen, et al	Taiwan	2023	2010	cross-sectional	school	WHO	250	564
Yen, et al	Taiwan	2021	2019-2020	cross-sectional	school	CDC	208	569
Wang, et al	Taiwan	2021	2004-2017	cross-sectional	school	CDC	198	1599
Chen, et al	Taiwan	2012	2007/2010	cross-sectional	database	CDC	3764	7930
Chu, et al	Taiwan	2007	2001-2002	cross-sectional	database	IOTF	650	2405
Liou, et al	Taiwan	2010	2006-2007	cross-sectional	school	IOTF	2011	8640
Ting, et al	Taiwan	2012	2010-2011	cross-sectional	school	IOTF	188	859
Tsai, et al	Taiwan	2009	2007	cross-sectional	school	CDC	453	1287
Chen, et al	Taiwan	2019	2010	cohort	database	IOTF	946	2804
Lai, et al	Taiwan	2001	2000	cross-sectional	school	IOTF	501	2011
Yen, et al	Taiwan	2010	2004	cross-sectional	database	IOTF	2202	10371
Pu, et al	Taiwan	2010	2003	cross-sectional	database	IOTF	427	1879
Chang, et al	Taiwan	2010	2010	cross-sectional	school	IOTF	557	2083
Chen, et al	Taiwan	2009	2009	cross-sectional	school	IOTF	205	1024
Chen, et al	Taiwan	2008	2008	cross-sectional	school	IOTF	222	866
Chang, et al	Taiwan	2015	2008-2012	cross-sectional	school	CDC	205	838
Lee, et al	Taiwan	2014	2009	cross-sectional	school	IOTF	1049	5254
Hsieh, et al	Taiwan	2014	2010-2011	cross-sectional	school	IOTF	716	2419
Chen, et al	Taiwan	2014	2014	cross-sectional	medical institution	IOTF	30	121
Chiang, et al	Taiwan	2013	2001-2002	cross-sectional	database	IOTF	631	2283
Liao, et al	Taiwan	2013	2010-2011	cross-sectional	database	IOTF	3879	13500
Chen, et al	Taiwan	2016	2010-2011	cross-sectional	database	IOTF	533	1826
Chang, et al	Taiwan	2018	2012	cross-sectional	database	IOTF	10318	33942
Salas, et al (20)	Tajikistan	2021	2015-2017	cross-sectional	database	IOTF	217	2822
Mosha, et al	Tanzania	2021	2019	cross-sectional	school	IOTF	175	1170
Mosha, et al	Tanzania	2010	2008	cross-sectional	community	IOTF	23	222
Mpembeni, et al	Tanzania	2014	2014	cross-sectional	school	WHO	67	446
Mwaikambo, et al	Tanzania	2015	2015	cross-sectional	school	WHO	253	1722
Pangani, et al	Tanzania	2016	2012	cross-sectional	school	National Reference	402	1781
Gebremedhin, et al (22)	Tanzania	2015	2010	cross-sectional	database	National Reference	494	6948
Chomba, et al	Tanzania	2019	2018	cross-sectional	community	IOTF	80	451
Shinsugi, et al	Thailand	2021	2015-2016	cross-sectional	database	IOTF	834	9060
Rerksuppaphol, et al	Thailand	2021	2015	cross-sectional	school	National Reference	1135	3870
Weraarchakul, et al	Thailand	2017	2015	cross-sectional	school	WHO	61	210
Nonboonyawat, et al	Thailand	2019	2016	cross-sectional	school	WHO	284	1749
Pawloski, et al	Thailand	2008	2008	cross-sectional	school	IOTF	76	410
Romano, et al (23)	Thailand	2022	2015	cross-sectional	database	IOTF	831	4132
Manandhar, et al	Thailand	2019	2018	cross-sectional	school	WHO	88	440
Sengmeuang, et al	Thailand	2010	2007	cross-sectional	school	CDC	1017	7096
Rerksuppaphol, et al	Thailand	2010	2007	cross-sectional	school	National Reference	253	1140
Narksawat,	Thailand	2009	2009	cross-sectional	school	WHO	100	862

© 2024 Zhang X et al. *JAMA Pediatrics.*

et al								
Rerksuppaphol, et al	Thailand	2015	2013	cross-sectional	school	CDC	1171	3991
Sukhonthachit, et al	Thailand	2014	2012	cross-sectional	school	WHO	250	693
Firestone, et al	Thailand	2011	2004	cross-sectional	database	IOTF	381	4610
Jitnarin, et al	Thailand	2011	2011	cross-sectional	database	WHO	1449	9287
Rojroongwasinkul, et al	Thailand	2013	2011	cross-sectional	community	WHO	481	3119
Thasanasuwan, et al	Thailand	2016	2016	cross-sectional	database	IOTF	142	1345
Gebremedhin, et al (23)	Togo	2015	2013/2014	cross-sectional	database	IOTF	84	3228
Sagbo, et al	Togo	2018	2015	cross-sectional	school	WHO	45	634
Romano, et al (24)	Tonga	2022	2017	cross-sectional	database	IOTF	1072	1946
Smith, et al	Tonga	2007	2000	cross-sectional	school	IOTF	204	445
Pengpid, et al (5)	Tonga	2015	2010-2011	cross-sectional	database	IOTF	1298	2211
Romano, et al (25)	Trinidad and Tobago	2022	2017	cross-sectional	database	IOTF	809	2363
Simeon, et al	Trinidad and Tobago	2003	2003	cross-sectional	school	IOTF	138	1090
Mumena, et al	Trinidad and Tobago	2018	2012-2014	prospective	school	IOTF	74	336
Boukthir, et al	Tunisia	2011	2007	cross-sectional	school	IOTF	264	1335
Musaiger, et al (8)	Tunisia	2016	2013-2014	cross-sectional	database	IOTF	172.645	803
Ercan, et al	Turkey	2012	2010-2011	cross-sectional	school	IOTF	1415	8848
Salman, et al	Turkey	2022	2014	cross-sectional	school	IOTF	219	1127
Meydanlioglu, et al	Turkey	2022	2017	cross-sectional	school	IOTF	1198	5160
Salas, et al (21)	Turkey	2021	2015-2017	cross-sectional	database	IOTF	432	3658
Gunalan, et al	Turkey	2021	2017	cross-sectional	school	WHO	438	1561
Arslan, et al	Turkey	2021	2016	cross-sectional	school	WHO	2680	9786
Acar Tek, et al	Turkey	2017	2017	cross-sectional	school	CDC	156	1111
Sağlam, et al	Turkey	2008	2008	cross-sectional	school	WHO	1204	5368
Sur, et al	Turkey	2005	2001-2002	cross-sectional	school	WHO	145	1044
Gundogdu, et al	Turkey	2008	2002	cross-sectional	medical institution	IOTF	206	1899
Turkkahraman, et al	Turkey	2006	2002-2003	cross-sectional	school	IOTF	441	2465
Nur, et al	Turkey	2008	2004	cross-sectional	school	CDC	38	1020
Bayat, et al	Turkey	2009	2004	cross-sectional	school	CDC	57	610
Discigil, et al	Turkey	2009	2005	cross-sectional	school	WHO	214	1348
Etiler, et al	Turkey	2011	2011	cross-sectional	school	IOTF	438	2281
Ozmen, et al	Turkey	2007	2007	cross-sectional	school	IOTF	213	2101
Simsek, et al	Turkey	2008	2005-2006	cross-sectional	school	WHO	1151	6925
Yuca, et al	Turkey	2010	2006-2007	cross-sectional	school	WHO	1204	9048
Pirinçci, et al	Turkey	2010	2007	cross-sectional	school	WHO	541	3642
Duzova, et al	Turkey	2013	2007-2008	cross-sectional	community	WHO	649	3571
Demirci, et al	Turkey	2013	2011-2012	cross-sectional	community	WHO	112	1000
Polat, et al	Turkey	2014	2012	cross-sectional	school	WHO	785	2826
Gökler, et al	Turkey	2015	2012	cross-sectional	school	WHO	866	3918
Agirbasli, et al	Turkey	2011	2008	cross-sectional	medical institution	WHO	340	1746
Manios, et al	Turkey	2005	2001	cross-sectional	school	National Reference	62	510
Krassas, et al (2)	Turkey	2004	2004	cross-sectional	school	WHO	452	3703
Yardim, et al	Turkey	2019	2015	cross-sectional	school	WHO	1180	3291
Akbulut, et	Turkey	2014	2014	cross-sectional	school	WHO	293	915

© 2024 Zhang X et al. *JAMA Pediatrics.*

al (1)								
Canan, et al	Turkey	2014	2014	cross-sectional	school	WHO	91	1938
Dündar, et al	Turkey	2012	2009	cross-sectional	school	WHO	808	2477
Gültekin, et al	Turkey	2005	2005	cross-sectional	school	WHO	14	591
Uçkun-Kitapçi, et al	Turkey	2004	2004	cross-sectional	school	CDC	232	1647
Oner, et al	Turkey	2004	2001	cross-sectional	community	IOTF	127	989
Ardıç, et al	Turkey	2019	2006-2010	cross-sectional	medical institution	WHO	49	180
Aşut, et al	Turkey	2019	2017	cross-sectional	school	IOTF	79	459
Çelmeli, et al	Turkey	2019	2015	cross-sectional	community	WHO	557	1687
Comba, et al	Turkey	2019	2017	cross-sectional	school	CDC	339	1684
Deniz, et al	Turkey	2019	2019	cross-sectional	school	IOTF	296	1298
Karakus, et al	Turkey	2019	2016	cross-sectional	school	IOTF	59	177
Arikan, et al	Turkey	2020	2020	cross-sectional	school	IOTF	1398	10781
Esenay, et al	Turkey	2010	2010	cross-sectional	school	WHO	161	635
Ucar, et al	Turkey	2009	2001-2003	cross-sectional	school	National Reference	1894	11629
Haug, et al (18)	Turkey	2009	2005-2006	cross-sectional	database	WHO	482	4680
Garipagaoglu, et al	Turkey	2009	2006	cross-sectional	medical institution	WHO	184	592
Dinç, et al	Turkey	2009	2009	cross-sectional	school	WHO	188	1346
Önsüz, et al	Turkey	2015	2010	cross-sectional	school	IOTF	569	2166
Meseri, et al	Turkey	2015	2012	cross-sectional	school	WHO	153	462
Inal, et al	Turkey	2015	2011	cross-sectional	school	IOTF	213	531
Weinmayr, et al (16)	Turkey	2014	2004	cross-sectional	database	WHO	73	342
Vehapoglu, et al	Turkey	2014	2012-2013	cross-sectional	medical institution	WHO	1043	4990
Cabar, et al	Turkey	2014	2010-2011	cross-sectional	school	WHO	492	3352
Ayyildiz, et al	Turkey	2014	2010	cross-sectional	school	WHO	304	868
Calisir, et al	Turkey	2011	2005-2006	cross-sectional	school	WHO	122	460
Cinar, et al	Turkey	2011	2004	cross-sectional	school	WHO	139	451
Akca, et al	Turkey	2016	2011	cross-sectional	school	CDC	146	554
Geckil, et al	Turkey	2017	2012-2013	cross-sectional	school	WHO	670	3028
Gül, et al	Turkey	2017	2013-2014	cross-sectional	school	WHO	141	1374
Eker, et al	Turkey	2018	2011-2012	cross-sectional	school	WHO	206	1357
Haney, et al	Turkey	2018	2014-2015	cross-sectional	school	IOTF	354	1289
Salas, et al (22)	Turkmenistan	2021	2015-2017	cross-sectional	database	IOTF	2569	9768
Gebremedhin, et al (24)	Uganda	2015	2011	cross-sectional	database	IOTF	253	4212
Dereń, et al	Ukraine	2020	2018-2019	cross-sectional	school	IOTF	2579	18144
Dereń, et al	Ukraine	2018	2018	cross-sectional	school	IOTF	2418	13739
Høyer, et al (3)	Ukraine	2014	2002-2006	cohort	database	National Reference	94	492
Janssen, et al (31)	Ukraine	2005	2001-2002	cross-sectional	database	WHO	218	3645
Haug, et al (26)	Ukraine	2009	2005-2006	cross-sectional	database	WHO	369	4613
AlBlooshi, et al	United Arab Emirates	2016	2014-2015	cross-sectional	community	WHO	8080	27078
Ismail, et al	United Arab Emirates	2022	2019-2020	cross-sectional	medical institution	WHO	19	276
Abduelkarem, et al	United Arab Emirates	2020	2017	cross-sectional	school	WHO	191	684
Abdullatif, et al	United Arab Emirates	2022	2018-2019	cross-sectional	school	WHO	707	1683
Aburawi, et al	United Arab Emirates	2019	2019	cross-sectional	school	National Reference	287	967
Zaal, et al	United Arab Emirates	2009	2009	cross-sectional	school	IOTF	245	661
Musaiger, et al (5)	United Arab Emirates	2012	2010-2011	cross-sectional	school	CDC	143	505
Fatima, et al	United Arab Emirates	2018	2017-2018	cross-sectional	school	IOTF	67	267
Adab, et al	United	2018	2005	RCT	school	IOTF	295	1397

© 2024 Zhang X et al. *JAMA Pediatrics.*

	Kingdom of Great Britain and Northern Ireland							
Sweeting, et al	United Kingdom of Great Britain and Northern Ireland	2005	2005	cohort	database	IOTF	214	2127
Fraser, et al	United Kingdom of Great Britain and Northern Ireland	2012	2004-2008	cohort	database	CDC	623	4768
Gilliland, et al	United Kingdom of Great Britain and Northern Ireland	2012	2012	cross-sectional	school	IOTF	236	966
Héroux, et al (2)	United Kingdom of Great Britain and Northern Ireland	2012	2012	cross-sectional	database	National Reference	800	4792
Uerlich, et al	United Kingdom of Great Britain and Northern Ireland	2021	2014-2015	cross-sectional	database	IOTF	40	171
Ralphs, et al	United Kingdom of Great Britain and Northern Ireland	2021	2007-2010	cross-sectional	database	WHO	978	6410
Adab, et al	United Kingdom of Great Britain and Northern Ireland	2014	2006-2007	RCT	school	WHO	115	571
Bartle, et al	United Kingdom of Great Britain and Northern Ireland	2013	2013	longitudinal	school	WHO	75	400
Pearce, et al	United Kingdom of Great Britain and Northern Ireland	2010	2003-2005	cohort	database	WHO	3099	13187
Harding, et al	United Kingdom of Great Britain and Northern Ireland	2008	2002-2003	cross-sectional	school	WHO	1648	5684
Bonuck, et al	United Kingdom of Great Britain and Northern Ireland	2015	2006-2007	longitudinal	community	CDC	211	1899
Steele, et al	United Kingdom of Great Britain	2009	2007	cross-sectional	school	WHO	427	1862

© 2024 Zhang X et al. *JAMA Pediatrics*.

	and Northern Ireland							
Basterfield, et al	United Kingdom of Great Britain and Northern Ireland	2014	2006-2007	cross-sectional	database	IOTF	102	425
Griffiths, et al	United Kingdom of Great Britain and Northern Ireland	2014	2005-2007	cross-sectional	database	WHO	6839	13291
Green, et al	United Kingdom of Great Britain and Northern Ireland	2021	2011-2012	longitudinal	database	WHO	2570	9736
Scott, et al	United Kingdom of Great Britain and Northern Ireland	2016	2000-2013	cohort	database	WHO	1087	17231
Clemente, et al	United Kingdom of Great Britain and Northern Ireland	2019	2019	cross-sectional	database	WHO	299	1396
Griffiths, et al	United Kingdom of Great Britain and Northern Ireland	2011	2000-2002	cross-sectional	database	IOTF	601	11202
Tiffin, et al	United Kingdom of Great Britain and Northern Ireland	2011	2007	cross-sectional	database	WHO	1065	3961
Mendez, et al	United Kingdom of Great Britain and Northern Ireland	2008	2004	cohort	database	WHO	101	369
Ruiz, et al (4)	United Kingdom of Great Britain and Northern Ireland	2016	2005	cohort	database	WHO	2937	14186
Harrison, et al	United Kingdom of Great Britain and Northern Ireland	2011	2011	cross-sectional	school	WHO	379	1724
Taylor, et al	United Kingdom of Great Britain and Northern Ireland	2005	2001	cross-sectional	school	IOTF	893	2482
Janssen, et al (7)	United Kingdom of Great Britain and Northern	2005	2001-2002	cross-sectional	database	IOTF	663	3601

© 2024 Zhang X et al. *JAMA Pediatrics.*

	Ireland							
Janssen, et al (26)	United Kingdom of Great Britain and Northern Ireland	2005	2001-2002	cross-sectional	database	IOTF	343	2133
Janssen, et al (33)	United Kingdom of Great Britain and Northern Ireland	2005	2001-2002	cross-sectional	database	WHO	681	3170
Cecil, et al	United Kingdom of Great Britain and Northern Ireland	2005	2002-2003	cross-sectional	school	WHO	604	2454
Fletcher, et al	United Kingdom of Great Britain and Northern Ireland	2004	2000	cross-sectional	school	WHO	158	424
Webster-Gandy, et al	United Kingdom of Great Britain and Northern Ireland	2003	2003	cross-sectional	school	WHO	21	188
Warren, et al	United Kingdom of Great Britain and Northern Ireland	2003	2001	cross-sectional	school	WHO	11	37
Whitaker, et al	United Kingdom of Great Britain and Northern Ireland	2010	2001-2006	cross-sectional	database	WHO	1813	7078
Thomas, et al(1)	United Kingdom of Great Britain and Northern Ireland	2010	2002	cross-sectional	school	WHO	23	71
Thomas, et al(2)	United Kingdom of Great Britain and Northern Ireland	2010	2007	cross-sectional	school	WHO	20	83
Skidmore, et al	United Kingdom of Great Britain and Northern Ireland	2010	2007	cross-sectional	school	WHO	398	1721
Jennings, et al	United Kingdom of Great Britain and Northern Ireland	2010	2010	cross-sectional	school	National Reference	33	84
Edwards, et al	United Kingdom of Great Britain and Northern Ireland	2010	2000-2006	cross-sectional	community	IOTF	6378	33594
Willianmson,	United	2009	2006	cross-sectional	school	WHO	1220	2709

© 2024 Zhang X et al. *JAMA Pediatrics*.

et al(1)	Kingdom of Great Britain and Northern Ireland							
Willianmson, et al(2)	United Kingdom of Great Britain and Northern Ireland	2009	2006	cross-sectional	school	WHO	1196	2709
Webber, et al(1)	United Kingdom of Great Britain and Northern Ireland	2009	2006	cross-sectional	database	WHO	8	239
Webber, et al(2)	United Kingdom of Great Britain and Northern Ireland	2009	2006	cross-sectional	database	WHO	12	167
Standley, et al	United Kingdom of Great Britain and Northern Ireland	2009	2002	cross-sectional	school	IOTF	1017	4167
Nelson, et al	United Kingdom of Great Britain and Northern Ireland	2009	2003-2005	cross-sectional	school	WHO	900	4587
McCullough, et al	United Kingdom of Great Britain and Northern Ireland	2009	2009	cross-sectional	school	IOTF	58	211
Haug, et al (28)	United Kingdom of Great Britain and Northern Ireland	2009	2005-2006	cross-sectional	database	CDC	233	1961
Haug, et al (37)	United Kingdom of Great Britain and Northern Ireland	2009	2005-2006	cross-sectional	database	CDC	398	2600
Haug, et al (39)	United Kingdom of Great Britain and Northern Ireland	2009	2005-2006	cross-sectional	database	IOTF	552	2910
Fox, et al	United Kingdom of Great Britain and Northern Ireland	2009	2009	cross-sectional	school	IOTF	35	201
Fairclough, et al	United Kingdom of Great Britain and Northern Ireland	2009	2004-2007	cross-sectional	school	CDC	1856	6337
van Sluijs, et al	United Kingdom of Great Britain	2008	2007	cross-sectional	database	CDC	473	2064

© 2024 Zhang X et al. *JAMA Pediatrics*.

	and Northern Ireland							
Harding, et al	United Kingdom of Great Britain and Northern Ireland	2008	2008	cross-sectional	database	CDC	1625	5515
Routh, et al	United Kingdom of Great Britain and Northern Ireland	2006	2006	cross-sectional	school	IOTF	68	252
Mutunga, et al	United Kingdom of Great Britain and Northern Ireland	2006	2000	cross-sectional	database	WHO	586	2016
Solmi, et al	United Kingdom of Great Britain and Northern Ireland	2015	2006-2012	cross-sectional	database	IOTF	6377	29001
Katzmarzyk, et al (12)	United Kingdom of Great Britain and Northern Ireland	2015	2011-2013	cross-sectional	database	CDC	44	478
Jackson, et al	United Kingdom of Great Britain and Northern Ireland	2015	2005-2012	cross-sectional	database	IOTF	1354	4979
Falconer, et al	United Kingdom of Great Britain and Northern Ireland	2014	2010-2011	cross-sectional	database	CDC	493	2737
Pallan, et al	United Kingdom of Great Britain and Northern Ireland	2011	2011	cross-sectional	database	IOTF	115	574
Coulthard, et al	United Kingdom of Great Britain and Northern Ireland	2016	2008-2012	cross-sectional	database	CDC	518	1620
Beynon, et al	United Kingdom of Great Britain and Northern Ireland	2017	2008-2012	cross-sectional	database	CDC	2892	11279
Hudda, et al	United Kingdom of Great Britain and Northern Ireland	2018	2012-2013	cross-sectional	database	CDC	290807	1068261
Wang, et al	United States of America	2010	2005-2006	cross-sectional	database	CDC	2113	6939
Chiasson, et al	United States of	2016	2008-2009	cohort	database	IOTF	16098	50589

© 2024 Zhang X et al. *JAMA Pediatrics*.

	America							
Ehrenthal, et al	United States of America	2016	2004-2011	cohort	database	IOTF	658	2172
Taveras, et al	United States of America	2006	2006	cohort	database	IOTF	273	1012
Weden, et al	United States of America	2012	2001-2007	cohort	database	CDC	442	3300
Laurson, et al	United States of America	2014	2005	cross-sectional	community	IOTF	223	1360
Vehrs, et al	United States of America	2022	2022	cross-sectional	community	IOTF	60	332
Vazquez, et al	United States of America	2022	2019	cross-sectional	school	IOTF	50	237
Leung, et al	United States of America	2011	2005-2006	cohort	database	CDC	98	353
Shier, et al	United States of America	2012	2004/2007	cross-sectional	database	CDC	3455	6260
Salcido, et al	United States of America	2022	2017-2018	cross-sectional	medical institution	IOTF	2353	7270
Odusanya, et al	United States of America	2022	2009-2018	cross-sectional	database	IOTF	474	3248
Mayne, et al	United States of America	2023	2019-2020	cross-sectional	medical institution	CDC	12528	81418
Bader, et al	United States of America	2013	2007-2008	cross-sectional	database	IOTF	34389	113123
Burdette, et al	United States of America	2004	2004	cross-sectional	database	IOTF	2089	6907
Carroll-Scott, et al	United States of America	2013	2009	cross-sectional	database	IOTF	493	996
Hunt, et al	United States of America	2022	2009-2013	cohort	database	CDC	238	816
Fyfe-Johnson, et al	United States of America	2022	2012	cross-sectional	database	IOTF	8044	17115
Flórez, et al	United States of America	2022	2006-2017	cross-sectional	database	CDC	198619	570172
Davis, et al	United States of America	2009	2002-2005	cross-sectional	database	IOTF	146635	529367
Salazar, et al	United States of America	2022	2018-2019	cross-sectional	medical institution	IOTF	56	112
Galvez, et al	United States of America	2009	2009	longitudinal	database	IOTF	139	323
Bejarano, et al	United States of America	2022	2015-2016	cross-sectional	database	National Reference	117442	320005
Findling, et al	United States of America	2018	2012-2013	cross-sectional	database	CDC	780	1720
Zhang, et al	United States of America	2021	2017	cross-sectional	database	WHO	1030	4348
Héroux, et al (3)	United States of America	2012	2012	cross-sectional	database	CDC	2233	6454

© 2024 Zhang X et al. *JAMA Pediatrics.*

Kepper, et al	United States of America	2016	2016	RCT	database	WHO	18	78
Stine, et al	United States of America	2021	2017	cross-sectional	database	CDC	1887	3956
Reilly, et al (1)	United States of America	2021	2008-2009	cross-sectional	school	CDC	203	511
Reilly, et al (2)	United States of America	2021	2015-2016	cross-sectional	school	WHO	344	836
Liu, et al	United States of America	2007	2000	cross-sectional	medical institution	CDC	2914	7334
Imoisili, et al	United States of America	2021	2012-2018	cross-sectional	database	National Reference	230216	759591
Mellor, et al	United States of America	2011	2006	cross-sectional	school	WHO	352	2023
Ohri-Vachaspati, et al	United States of America	2015	2009-2010	cross-sectional	community	National Reference	223	321
Oreskovic, et al	United States of America	2009	2006	cross-sectional	medical institution	WHO	2447	6680
Salois, et al	United States of America	2012	2007-2009	cross-sectional	database	WHO	310	2192
Sánchez, et al	United States of America	2012	2007	cross-sectional	database	National Reference	583391	926018
Tang, et al	United States of America	2014	2008-2009	cross-sectional	database	National Reference	5648	12954
Wasserman, et al	United States of America	2014	2008-2009	cross-sectional	school	National Reference	7212	12090
Halfon, et al	United States of America	2013	2007	cross-sectional	database	National Reference	13423	43297
Hurt, et al	United States of America	2014	2005-2010	cross-sectional	database	IOTF	2718	7629
Sacheck, et al	United States of America	2011	2009	cross-sectional	school	IOTF	117	263
Turer, et al	United States of America	2013	2003-2006	cross-sectional	database	WHO	4564	12292
Moore, et al	United States of America	2016	2005-2006	cross-sectional	database	WHO	995	2482
Lee, etal	United States of America	2007	2007	longitudinal	database	National Reference	85	309
Achaya, et al	United States of America	2011	2011	cross-sectional	school	National Reference	181	719
Adachi-Mejia, et al	United States of America	2007	2002-2003	cross-sectional	school	National Reference	1001	2343
Adams, et al	United States of America	2010	2010	cross-sectional	community	National Reference	190	421
Adams, et al	United States of America	2005	2001-2003	cross-sectional	school	National Reference	165	366
Adams, et al	United States of America	2019	2013-2015	cross-sectional	database	WHO	180	450
Adams, et al	United States of	2013	2007-2009	cross-sectional	database	WHO	166201	495848

© 2024 Zhang X et al. *JAMA Pediatrics*.

	America							
Ogden, et al	United States of America	2018	2013-2016	cross-sectional	database	National Reference	648	4219
Davis, et al	United States of America	2011	2003-2006	cross-sectional	database	National Reference	2794	7882
Lewis, et al	United States of America	2006	2002	cross-sectional	school	National Reference	1127	3114
Singh, et al	United States of America	2008	2003-2004	cross-sectional	database	WHO	6931	46707
Davis, et al	United States of America	2008	2008	cross-sectional	school	WHO	60	138
Agazzi, et al	United States of America	2010	2005	cross-sectional	school	WHO	215	535
DeBoer, et al	United States of America	2015	2001	longitudinal	database	National Reference	2750	8950
Govindan, et al	United States of America	2013	2004-2011	cross-sectional	school	WHO	588	1714
Wojcicki, et al	United States of America	2011	2006-2007	cohort	medical institution	National Reference	64	145
Beck, et al	United States of America	2014	2014	cross-sectional	community	WHO	51	319
Badon, et al	United States of America	2020	2008-2014	cross-sectional	medical institution	WHO	10433	111482
Bai, et al	United States of America	2016	2012	cross-sectional	database	WHO	421	1114
Giammattei	United States of America	2003	2000-2001	cross-sectional	school	National Reference	136	385
Barlow, et al	United States of America	2007	2003	cross-sectional	database	National Reference	18813	52845
Barrera, et al	United States of America	2016	2005-2007	cross-sectional	database	National Reference	142	1181
Barroso, et al	United States of America	2012	2012	longitudinal	medical institution	National Reference	153	372
Baxter, et al	United States of America	2013	2004-2007	cross-sectional	school	WHO	752	1504
Baxter, et al	United States of America	2011	2004-2007	cross-sectional	school	National Reference	454	920
Geier, et al	United States of America	2007	2007	cross-sectional	school	IOTF	427	1069
Shore, et al	United States of America	2008	2004-2005	cross-sectional	school	WHO	143	549
Pan, et al	United States of America	2013	2009	cross-sectional	database	National Reference	1053	3470
Kim, et al	United States of America	2018	2002-2003	longitudinal	school	IOTF	667	2318
Seicean, et al	United States of America	2007	2004	cross-sectional	school	IOTF	102	509
Lumeng, et al	United States of America	2007	2003	cohort	database	IOTF	139	785

© 2024 Zhang X et al. *JAMA Pediatrics.*

Miller, et al	United States of America	2014	2014	cross-sectional	school	National Reference	135	366
Silva, et al	United States of America	2011	2004-2009	cohort	database	IOTF	154	304
Fiorito, et al	United States of America	2006	2006	cross-sectional	community	National Reference	78	177
Charvet, et al	United States of America	2019	2019	cross-sectional	medical institution	IOTF	63	197
Haidar, et al	United States of America	2019	2009-2011	cross-sectional	database	National Reference	2552	6716
Hu, et al	United States of America	2018	2015	cross-sectional	database	National Reference	2005	15624
Drake, et al	United States of America	2012	2002-2009	longitudinal	community	CDC	721	1718
Suglia, et al	United States of America	2013	2003-2005	cohort	database	CDC	270	1589
Drake, et al	United States of America	2013	2007-2008	cohort	school	National Reference	169	479
Pérez, et al (1)	United States of America	2015	2000-2002	cross-sectional	database	WHO	3077	8938
Pérez, et al (2)	United States of America	2015	2004-2005	cross-sectional	database	National Reference	5353	15283
Ghosh-Dastidar, et al	United States of America	2016	2013	cross-sectional	database	National Reference	140	475
Dalton 3rd, et al	United States of America	2014	2012	cross-sectional	school	IOTF	692	1491
Berkowitz, et al	United States of America	2005	2005	cohort	community	National Reference	24	78
Gillman, et al	United States of America	2008	2008	cohort	database	National Reference	105	896
Hinkle, et al	United States of America	2012	2006	cohort	database	WHO	1303	3600
Kubo, et al	United States of America	2016	2005-2012	cohort	database	National Reference	118	417
Lindberg, et al	United States of America	2012	2012	cohort	database	IOTF	211	471
Olson, et al	United States of America	2010	2009-2010	cohort	database	WHO	103	321
Wojcicki, et al	United States of America	2015	2005-2009	cohort	database	National Reference	337	833
Bider-Canfield, et al	United States of America	2017	2011	cohort	medical institution	National Reference	2147	15710
Pham, et al	United States of America	2013	2004-2007	cohort	medical institution	National Reference	493	2093
Ohri-Vachaspati, et al	United States of America	2013	2009-2010	cross-sectional	community	IOTF	267	702
Han, et al	United States of America	2020	2009-2016	longitudinal	school	IOTF	311640	486178
Jia, et al	United States of	2019	2007	longitudinal	database	National Reference	4141	7530

© 2024 Zhang X et al. *JAMA Pediatrics.*

	America							
Elbel, et al	United States of America	2019	2013	cross-sectional	database	IOTF	275035	735192
Reis, et al	United States of America	2020	2020	cross-sectional	school	IOTF	128	171
Ohri-Vachaspati, et al	United States of America	2021	2009-2017	cross-sectional	database	IOTF	115	449
Bailey, et al	United States of America	2014	2001-2013	cohort	medical institution	IOTF	29389	89057
Ville, et al	United States of America	2017	2012-2013	cohort	medical institution	IOTF	11	97
Theall, et al	United States of America	2019	2012-2013	cross-sectional	community	IOTF	29	90
Wojcicki, et al	United States of America	2016	2006-2007	cross-sectional	medical institution	IOTF	82	200
Zhu, et al	United States of America	2015	2001-2005	cross-sectional	school	National Reference	191	766
Harley, et al	United States of America	2013	2000-2010	cohort	database	National Reference	172	319
Huh, et al	United States of America	2012	2002-2005	cohort	medical institution	WHO	329	1255
Wang, et al	United States of America	2013	2000-2004	cohort	database	WHO	482	917
Peck, et al	United States of America	2015	2011	cohort	database	WHO	6458	24137
Miles, et al	United States of America	2018	2010	cross-sectional	database	CDC	1027	2770
Kjaer, et al	United States of America	2018	2015-2016	cohort	medical institution	National Reference	60	102
DuBose, et al	United States of America	2006	2006	cross-sectional	database	National Reference	165	375
Heer, et al	United States of America	2013	2008	cross-sectional	school	National Reference	314	653
Oken, et al	United States of America	2005	2005	cross-sectional	database	IOTF	204	746
Braun, et al	United States of America	2010	2003-2006	cohort	database	WHO	66	411
Mosli, et al	United States of America	2016	2009	cohort	community	WHO	116	273
Block, et al	United States of America	2018	2009-2016	cross-sectional	medical institution	WHO	100998	362550
Assari, et al	United States of America	2015	2003	cross-sectional	database	National Reference	287	1170
Boutelle, et al	United States of America	2010	2010	cross-sectional	community	WHO	114	495
Kubzansky, et al	United States of America	2012	2001-2005	cross-sectional	school	IOTF	705	1528
McClure, et al	United States of America	2010	2003	cross-sectional	community	WHO	1205	4458

© 2024 Zhang X et al. *JAMA Pediatrics*.

Roberts, et al	United States of America	2013	2000	cross-sectional	database	IOTF	1511	4175
Borges, et al	United States of America	2010	2005	cross-sectional	community	IOTF	445	3005
Vannucci, et al	United States of America	2017	2007/2009	cross-sectional	school	IOTF	120	368
Adams, et al	United States of America	2005	2000-2004	cohort	database	IOTF	103	252
Hack, et al	United States of America	2011	2004	longitudinal	medical institution	National Reference	35	296
Vohr, et al	United States of America	2018	2018	cohort	database	National Reference	86	388
Wood, et al	United States of America	2018	2012-2014	cohort	database	WHO	210	871
Cottrell, et al	United States of America	2011	2007-2008	cross-sectional	database	IOTF	6715	17944
Vangeepuram, et al	United States of America	2011	2004-2007	cross-sectional	database	IOTF	211	503
Kwon, et al	United States of America	2006	2002-2004	cross-sectional	database	IOTF	327	853
Musaad, et al	United States of America	2009	2009	cross-sectional	medical institution	National Reference	86	540
Anderson, et al	United States of America	2014	2007-2011	cohort	database	National Reference	3186	15141
Taveras, et al	United States of America	2014	2014	cohort	medical institution	National Reference	260	1046
Scharf, et al	United States of America	2015	2001-2006	cohort	database	National Reference	2819	8950
Storfer-Isser, et al	United States of America	2012	2002-2006	cohort	database	CDC	87	313
Bell, et al	United States of America	2010	2002	cohort	database	IOTF	670	1920
Roberts, et al	United States of America	2013	2000-2001	cross-sectional	school	National Reference	1628	4175
Anderson, et al	United States of America	2011	2003	cross-sectional	school	National Reference	143	918
Lohman, et al	United States of America	2009	2009	cross-sectional	database	IOTF	475	1011
Foster, et al	United States of America	2008	2008	RCT	school	National Reference	549	1349
Chomitz, et al	United States of America	2010	2003-2004	cohort	school	National Reference	687	1858
Shriver, et al	United States of America	2011	2008	cross-sectional	school	IOTF	91	237
Eagle, et al	United States of America	2010	2004-2009	cross-sectional	database	National Reference	150	1003
Lee, et al	United States of America	2010	2002	longitudinal	database	WHO	136	401
Kunin-Batson, et al	United States of	2023	2012-2014	longitudinal	database	National Reference	258	534

© 2024 Zhang X et al. *JAMA Pediatrics.*

	America							
Wu, et al	United States of America	2022	2018	longitudinal	database	WHO	14339	46151
White, et al (3)	United States of America	2022	2022	cohort	database	WHO	957	2545
Arcan, et al	United States of America	2012	2005-2006	cross-sectional	school	WHO	124	413
Armstrong, et al	United States of America	2012	2008-2011	cross-sectional	medical institution	National Reference	41	94
Stettler, et al	United States of America	2005	2001	cross-sectional	medical institution	WHO	569	2474
Rappaport, et al	United States of America	2005	2002	cross-sectional	community	National Reference	1547	2621
Kim, et al	United States of America	2005	2001-2003	cross-sectional	school	National Reference	2212	11845
Keysey, et al	United States of America	2005	2003-2004	cross-sectional	medical institution	WHO	78	364
Janssen, et al (32)	United States of America	2005	2001-2002	cross-sectional	database	National Reference	1116	4447
Flynn, et al	United States of America	2005	2003	cross-sectional	school	WHO	1701	7048
Thorpe, et al	United States of America	2004	2003	cross-sectional	school	WHO	1153	2681
Patrick, et al	United States of America	2004	2001-2002	cross-sectional	medical institution	National Reference	401	878
Nelson, et al	United States of America	2004	2001	cross-sectional	database	IOTF	218	556
Mirza, et al	United States of America	2004	2002	cross-sectional	medical institution	National Reference	116	309
Hoelscher, et al	United States of America	2004	2000-2001	cross-sectional	school	National Reference	1312	6630
Drobac, et al	United States of America	2004	2001-2002	cross-sectional	medical institution	WHO	477	997
Davy, et al	United States of America	2004	2004	cross-sectional	school	WHO	111	205
Trost, et al	United States of America	2003	2003	cross-sectional	school	IOTF	60	245
Jiménez-Cruz, et al	United States of America	2003	2001-2002	cross-sectional	school	WHO	456	1200
Friedlander, et al	United States of America	2003	2003	cross-sectional	community	IOTF	165	371
Eisenmann, et al	United States of America	2003	2003	cross-sectional	school	WHO	122	263
Demerath, et al	United States of America	2003	2000	cross-sectional	school	WHO	598	1338
Rinderknecht, et al	United States of America	2002	2000	cross-sectional	community	WHO	98	155
Rinderknecht, et al	United States of America	2002	2002	cross-sectional	school	WHO	617	4240

© 2024 Zhang X et al. *JAMA Pediatrics*.

Yang, et al	United States of America	2018	2014-2015	cross-sectional	school	WHO	15935	41283
Hidalgo-Mendez, et al	United States of America	2019	2019	cross-sectional	medical institution	WHO	87	186
Tschamler, et al	United States of America	2010	2006	cross-sectional	medical institution	WHO	59	193
Thundiyil, et al	United States of America	2010	2007	cross-sectional	medical institution	IOTF	62	179
Amram, et al	United States of America	2020	2020	cross-sectional	school	IOTF	3215	10327
Strickman-Stein, et al	United States of America	2010	2004-2006	cross-sectional	medical institution	IOTF	75	200
Shankaran, et al	United States of America	2010	2010	cross-sectional	community	WHO	324	880
Shabbir, et al	United States of America	2010	2006-2007	cross-sectional	school	IOTF	283604	670352
Rodriguez, et al	United States of America	2010	2007	cross-sectional	school	National Reference	839	2038
Meininger, et al	United States of America	2010	2006	cross-sectional	school	CDC	498	1070
Hennessy, et al	United States of America	2010	2007	cross-sectional	school	IOTF	75	99
Gungor, et al	United States of America	2010	2010	cross-sectional	medical institution	IOTF	32	129
Grow, et al	United States of America	2010	2006	cross-sectional	community	IOTF	1293	8616
Farhat, et al	United States of America	2010	2005-2006	cross-sectional	school	IOTF	2475	7737
Elder, et al	United States of America	2010	2003-2004	cross-sectional	community	IOTF	346	745
Dammann, et al	United States of America	2010	2010	cross-sectional	community	IOTF	50	92
Bethell, et al	United States of America	2010	2007	cross-sectional	database	IOTF	13980	44101
Beets, et al	United States of America	2010	2006	cross-sectional	database	IOTF	1386	6603
Bayles, et al	United States of America	2010	2010	cross-sectional	community	WHO	20	3699
Baranowski, et al	United States of America	2010	2010	cross-sectional	school	WHO	575	1551
Babey, et al(1)	United States of America	2010	2001	cross-sectional	database	IOTF	1669	5858
Babey, et al(2)	United States of America	2010	2003	cross-sectional	database	IOTF	1155	4010
Babey, et al(3)	United States of America	2010	2005	cross-sectional	database	IOTF	1217	4029
Babey, et al(4)	United States of America	2010	2007	cross-sectional	database	IOTF	1008	3638
Villa-Caballero, et	United States of	2009	2003-2004	cross-sectional	community	WHO	49	725

© 2024 Zhang X et al. *JAMA Pediatrics.*

al	America							
Valente, et al	United States of America	2009	2009	cross-sectional	school	IOTF	188	562
Vader, et al	United States of America	2009	2000-2002	cross-sectional	school	WHO	4664	11594
Treuth, et al	United States of America	2009	2003-2005	longitudinal	school	IOTF	1078	3085
Trent, et al	United States of America	2009	2003-2004	cross-sectional	database	IOTF	1111	2630
Smith, et al	United States of America	2009	2005-2007	cross-sectional	school	IOTF	149	251
Santos, et al	United States of America	2009	2006	cross-sectional	school	WHO	529	1195
Rundle, et al	United States of America	2009	2003-2006	cross-sectional	school	IOTF	190	437
Raynor, et al	United States of America	2009	2004-2005	cross-sectional	medical institution	National Reference	60	185
Nsiah-Kumi, et al	United States of America	2009	2003	cross-sectional	medical institution	IOTF	117	336
Montgomery-Reagan, et al	United States of America	2009	2006-2007	cross-sectional	school	IOTF	2016	5306
Martyn-Nemeth, et al	United States of America	2009	2005	cross-sectional	school	IOTF	48	102
Lim, et al	United States of America	2009	2002-2003	longitudinal	school	IOTF	90	365
Kubik, et al	United States of America	2009	2006	cross-sectional	school	WHO	59	140
Krukowski, et al	United States of America	2009	2003	cross-sectional	school	WHO	413	1071
Hillman, et al	United States of America	2009	2004	cross-sectional	medical institution	WHO	162	397
Harnack, et al	United States of America	2009	2004-2006	cross-sectional	school	WHO	246	593
Harbaugh, et al	United States of America	2009	2005-2009	cross-sectional	school	WHO	482	1250
Franzini, et al	United States of America	2009	2003	cross-sectional	community	IOTF	267	650
Forman, et al	United States of America	2009	2009	cross-sectional	community	CDC	20	43
Duncan, et al	United States of America	2009	2006	cross-sectional	database	IOTF	511	1140
Dorsey, et al	United States of America	2009	2005	cross-sectional	medical institution	WHO	44	75
Dixon, et al	United States of America	2009	2001	cross-sectional	school	WHO	49	112
De La O, et al	United States of America	2009	2009	cross-sectional	school	National Reference	92	576
Chen, et al	United States of America	2009	2004-2005	cross-sectional	community	WHO	24	65

© 2024 Zhang X et al. *JAMA Pediatrics*.

Asante, et al	United States of America	2009	2004	cross-sectional	medical institution	CDC	151	324
Anderson, et al	United States of America	2009	2005	cross-sectional	database	National Reference	1573	8550
West, et al	United States of America	2008	2003-2004	cross-sectional	school	National Reference	574	1551
Stovitz, et al	United States of America	2008	2000-2001	cross-sectional	database	IOTF	215	1302
Steele, et al	United States of America	2008	2001-2004	cross-sectional	medical institution	IOTF	1115	3221
Pollack, et al	United States of America	2008	2000-2006	cross-sectional	database	IOTF	1172	3232
Margellos-Anast, et al	United States of America	2008	2002-2003	cross-sectional	database	IOTF	217	501
Laurson, et al	United States of America	2008	2001	longitudinal	community	IOTF	71	268
Kapoor, et al	United States of America	2008	2008	cross-sectional	school	WHO	468	1058
Kalich, et al	United States of America	2008	2003	cross-sectional	school	CDC	280	786
Irigoyen, et al	United States of America	2008	2004-2005	cross-sectional	medical institution	WHO	612	1713
Eisenmann, et al	United States of America	2008	2001	cross-sectional	database	WHO	4325	12464
Eichner, et al	United States of America	2008	2002-2003	cross-sectional	school	CDC	835	1706
Wald, et al	United States of America	2007	2004	cross-sectional	medical institution	WHO	232	612
Roseman, et al	United States of America	2007	2004	cross-sectional	school	WHO	1255	4049
Metallinos-Katsaras, et al	United States of America	2007	2000	cross-sectional	medical institution	CDC	21	56
Martin, et al	United States of America	2007	2003-2004	cross-sectional	community	IOTF	97	212
Malaty, et al	United States of America	2007	2001-2002	cross-sectional	school	WHO	139	552
Lynch, et al	United States of America	2007	2002-2003	cross-sectional	school	CDC	932	2055

eTable 6. Sensitivity analysis and leave-one-out results performed in Metafor package.

resid	se	z	
1352	0.89792996	0.12673419	7.08514395
842	0.66367257	0.12626314	5.25626537
252	0.49687073	0.1277239	3.89019385
1336	0.48632362	0.12767553	3.80905888
1903	0.45115014	0.12910583	3.49442098

© 2024 Zhang X et al. *JAMA Pediatrics.*

250	0.46108362	0.13293818	3.4684064
1922	0.42194353	0.12719983	3.31717053
490	0.42178075	0.12853916	3.28134047
1000	0.41561114	0.12721815	3.26691712
1207	0.42381726	0.1305522	3.24634325
1916	0.4267488	0.1338556	3.18812806
1310	0.4033362	0.12946107	3.11550187
1822	0.3988162	0.13016961	3.06381969
1875	0.40187452	0.131555	3.05480236
1827	0.41085859	0.13468885	3.05042766
1098	0.39125868	0.13123181	2.98143177
1165	0.37343453	0.1253883	2.9782247
1818	0.3804367	0.13100766	2.90392726
1459	0.36141005	0.12741819	2.83640873
1868	0.36333461	0.1285079	2.82733279
1915	0.38948467	0.13793182	2.82374771
798	0.3785586	0.13539214	2.79601596
1966	0.35968424	0.13066714	2.75267548
1083	0.37367554	0.1358217	2.7512213
1169	0.40506628	0.14746719	2.74682301
1368	0.33145124	0.12779285	2.59366031
1348	0.33893228	0.13192145	2.56919772
1889	0.3471345	0.13573955	2.55735701
259	0.33023346	0.13023318	2.53570902
1823	0.31834839	0.1284663	2.47806933
1480	0.32458438	0.13168788	2.46480081
1082	0.30558981	0.12624343	2.42063941
1977	0.31028846	0.12858489	2.41310207
1337	0.31026181	0.12974528	2.39131475
1870	0.30768895	0.1301652	2.36383413
1970	0.30442674	0.12921718	2.3559308
1861	0.31701194	0.13555641	2.33859792
1959	0.29872324	0.12778586	2.33768619
1647	0.29513438	0.12707558	2.32251057
1932	0.30134629	0.13001728	2.31774026
266	0.31673599	0.13697389	2.31238226
1656	0.29242684	0.1275986	2.29177157
1743	0.28785653	0.12640596	2.27723863
275	0.28618021	0.12595882	2.27201403
1965	0.29006318	0.12894798	2.24945897
1974	0.28855348	0.12835429	2.24810159
619	0.28364173	0.12649432	2.24232779

© 2024 Zhang X et al. *JAMA Pediatrics*.

1790	0.28328563	0.12649356	2.23952615
1782	0.29567321	0.13209848	2.23827868
1961	0.29209692	0.13085943	2.23214267
1339	0.28466896	0.1278723	2.22619718
1888	0.27820861	0.12675219	2.19490175
1963	0.28660913	0.13077703	2.19158619
1752	0.27095728	0.12567749	2.15597314
1748	0.28880477	0.13400572	2.15516753
1964	0.27838468	0.12961413	2.14779572
1979	0.26991474	0.12630354	2.13703232
1885	0.26889354	0.12635856	2.12801999
1942	0.27616456	0.12983878	2.12698047
1745	0.26658299	0.12548818	2.12436732
1103	0.26818032	0.1266005	2.11831948
256	0.32444697	0.1547257	2.09691714
284	0.2618633	0.12668645	2.06701905
1938	0.25855974	0.12553106	2.05972724
607	-0.2646956	0.12950977	-2.0438269
1084	0.25682312	0.12603299	2.03774526
1866	0.25639724	0.12657449	2.02566285
920	0.25614363	0.12652915	2.02438441
1708	0.25386016	0.12581236	2.01776809
1413	0.25311896	0.12600346	2.00882539
1874	0.25060895	0.12619216	1.98593128
1812	0.25231688	0.12754868	1.9782006
1407	0.25432655	0.12861346	1.97744893
514	-0.2480283	0.12598198	-1.9687603
100	-0.2471736	0.12562286	-1.9675847
1924	0.24826978	0.12639084	1.96430199
1618	-0.2476037	0.12642836	-1.9584503
1935	0.24971221	0.12770807	1.95533618
564	-0.2459267	0.12603056	-1.9513255
1005	0.24740544	0.1270315	1.94759132
855	0.24606327	0.12635158	1.94744909
1902	0.24469655	0.12583294	1.94461435
1829	0.24446971	0.12697427	1.92534839
495	0.24771718	0.12886892	1.92224145
1309	0.24857138	0.1293787	1.92126975
1946	0.25138678	0.13105011	1.91824924
1842	0.24400436	0.12742415	1.91489887
1908	0.25728152	0.13484031	1.908046
604	0.24558782	0.1292328	1.90035199

© 2024 Zhang X et al. *JAMA Pediatrics*.

1892	-0.2387087	0.12573136	-1.8985616
720	-0.2388302	0.12597832	-1.8958043
767	-0.2511386	0.13355066	-1.8804743
1869	0.2373362	0.12645897	1.87678418
1990	0.23826832	0.12708927	1.87481064
1097	0.23745622	0.12691257	1.87102217
1308	0.23502235	0.12578059	1.86851044
1771	0.23870103	0.1281575	1.86255998
1991	-0.233519	0.12635611	-1.8481019
1028	-0.232224	0.1257059	-1.8473597
1819	0.23464423	0.12766743	1.83793339
209	0.23787031	0.12961687	1.8351802
1074	-0.2295252	0.12578212	-1.8247842
998	0.2314601	0.12687325	1.82434129
1878	0.23771194	0.13070847	1.81864218
282	0.23668113	0.13021047	1.81768124
1600	0.22801768	0.12598239	1.80991702
1768	0.22938152	0.1268499	1.80829092
1933	0.22958448	0.12726417	1.80399941
1865	0.22698018	0.12584318	1.80367493
1789	0.22717147	0.12613329	1.801043
1914	0.2283076	0.12699648	1.79774748
1770	0.22970889	0.12781688	1.79717179
1010	-0.2259952	0.12579663	-1.7965123
1667	-0.225561	0.12574804	-1.7937534
1133	-0.2254472	0.12569062	-1.793668
1960	0.22938461	0.12822533	1.78891809
1316	-0.2244639	0.12627807	-1.7775369
794	0.23483788	0.13215123	1.77703895
1006	0.2249329	0.12687922	1.77281126
1958	0.22558576	0.12785409	1.76439999
1002	0.22186562	0.12592749	1.76185213
1086	0.22156718	0.12602408	1.75813367
805	0.22481173	0.12794308	1.75712292
1340	0.22264587	0.12671544	1.75705396
1831	0.22178392	0.12632659	1.75563918
751	0.22325925	0.127296	1.75385906
1814	0.22141949	0.12666916	1.74801422
903	0.22087661	0.12652454	1.74572146
1928	0.22001432	0.12606169	1.74529086
403	0.2223614	0.12745172	1.74467166
999	0.21927386	0.1258516	1.74232071

© 2024 Zhang X et al. *JAMA Pediatrics*.

1327	-0.218621	0.12561475	-1.7404088
990	-0.2190649	0.12589575	-1.7400503
1764	0.2247806	0.12920831	1.73967605
1872	0.22230358	0.12813599	1.73490353
108	-0.2183364	0.12585295	-1.7348532
1401	-0.2177955	0.12621295	-1.7256192
1978	0.21764221	0.12630096	1.72320313
2000	-0.2173362	0.12614402	-1.7229215
603	-0.2154053	0.12561989	-1.7147386
1934	0.21595151	0.12596641	1.71435787
1351	0.2202296	0.12875813	1.71041318
1879	0.22375021	0.13091984	1.7090627
110	-0.2173994	0.12800085	-1.6984215
225	-0.2150848	0.12669229	-1.6976944
1852	0.21317531	0.12622182	1.68889432
1088	0.21905548	0.12994843	1.68571091
944	0.21509214	0.12761399	1.68549027
1122	-0.2118039	0.12566468	-1.6854688
1750	0.21176911	0.12571469	1.68452159
218	-0.2136665	0.12701337	-1.6822359
1510	-0.2108388	0.12548884	-1.6801399
315	0.21091923	0.12557797	1.67958783
1962	0.21777144	0.12992951	1.67607373
1987	0.20956097	0.12561675	1.66825665
707	-0.2106923	0.12647119	-1.6659309
1465	0.20995682	0.12629377	1.66244794
1766	0.20899245	0.1258893	1.66012875
1911	0.21233571	0.12797202	1.65923543
1177	-0.2076637	0.12560128	-1.6533565
1357	0.20714412	0.12563077	1.64883266
1036	-0.2071572	0.12592331	-1.6451061
1668	-0.2065651	0.12570774	-1.6432173
1363	-0.2063594	0.12565628	-1.6422527
1149	-0.206393	0.12575002	-1.6412956
1815	0.20592332	0.12549248	1.64092154
1079	0.20670053	0.12614168	1.63863788
1873	0.21151475	0.1292189	1.63687158
1383	-0.2057872	0.12576257	-1.6363155
1311	0.20610489	0.12611958	1.63420214
1146	-0.2072026	0.12694108	-1.6322741
1982	0.20521926	0.12593764	1.62953073
1155	-0.2039697	0.12560449	-1.6239043

© 2024 Zhang X et al. *JAMA Pediatrics.*

1096	0.21865521	0.13469402	1.62334755
1635	-0.2044685	0.12600726	-1.6226726
417	-0.2034081	0.12553845	-1.6202855
1862	0.2037483	0.12587256	1.6186872
2001	-0.2047336	0.12648399	-1.6186523
1883	0.20250311	0.12549488	1.61363644
789	0.20647154	0.12805996	1.61230367
333	-0.2017574	0.12551788	-1.6073998
1329	-0.20183	0.12564158	-1.606395
1791	0.2028809	0.12642301	1.60477837
1029	-0.2017978	0.12575594	-1.6046784
1154	-0.2038023	0.12705495	-1.6040486
1549	-0.2015378	0.1257304	-1.6029365
87	0.20418573	0.12747083	1.60182321
1981	0.2015178	0.12592479	1.60030285
1011	-0.2011439	0.12576006	-1.5994257
418	-0.2007324	0.12553673	-1.5989936
1598	-0.2011103	0.12580499	-1.5985872
1048	0.20178336	0.12625112	1.59826984
741	-0.2043174	0.12791955	-1.5972339
1929	0.20259545	0.12711259	1.59382675
976	-0.2004483	0.12583255	-1.5929766
1134	-0.2029983	0.12743487	-1.5929573
306	-0.2004107	0.12587395	-1.5921539
1828	0.20360685	0.12812316	1.58914949
1453	0.20637829	0.13010256	1.58627386
1877	0.19878901	0.12552072	1.58371465
283	0.20204015	0.12777039	1.5812752
1142	-0.1981283	0.12553833	-1.5782298
1857	0.20009403	0.12734914	1.57122406
1973	0.19810308	0.12611651	1.57079422
1755	0.19727632	0.12563383	1.57024842
606	0.20237652	0.12894211	1.56951453
1160	-0.1966728	0.12551914	-1.566875
388	-0.1962692	0.12550402	-1.5638483
1926	0.19677761	0.12607936	1.56074399
1095	0.19714712	0.12634629	1.56037128
468	-0.1960743	0.12570022	-1.5598569
600	-0.1959018	0.12566256	-1.5589509
1800	0.19529177	0.12564798	1.55427707
1153	-0.1953833	0.12575988	-1.5536217
503	-0.1953765	0.12576664	-1.5534844

© 2024 Zhang X et al. *JAMA Pediatrics*.

1769	0.19558624	0.12592438	1.55320392
494	0.19944524	0.12858179	1.55111574
1884	0.19506609	0.12598829	1.54828751
1900	0.19412652	0.12558648	1.54575973
779	0.19405519	0.12569166	1.54389873
829	0.19316769	0.12557828	1.53822538
872	0.19337123	0.12571519	1.53816925
1324	-0.1934058	0.12623447	-1.5321157
551	0.19273478	0.12602502	1.52933738
234	0.19436544	0.12730011	1.52682855
1760	0.19156935	0.1255036	1.52640524
473	-0.1928506	0.12643523	-1.5252916
1257	-0.1913605	0.12567297	-1.5226862
1843	0.192443	0.1266656	1.51929956
870	0.1927599	0.12719447	1.51547387
1226	0.19238022	0.12695767	1.51530998
1412	-0.1927981	0.12745028	-1.5127316
1837	0.19555799	0.12939088	1.51137381
716	-0.1910284	0.126561	-1.509378
1250	-0.1899023	0.12598825	-1.5073013
1772	0.19231371	0.12769769	1.5060077
1905	0.19195007	0.12776263	1.5023961
1709	0.18902389	0.12587231	1.50171148
191	0.19112467	0.12750878	1.49891377
1622	-0.1886626	0.12598027	-1.4975567
768	0.20075365	0.13414577	1.49653362
1754	0.18919672	0.12669165	1.49336379
837	-0.1888155	0.12660698	-1.4913514
1931	0.21155139	0.14205225	1.48925054
1867	0.18930711	0.12728467	1.48727346
1854	0.1922684	0.129635	1.48315191
1457	-0.1872614	0.12627528	-1.4829618
294	0.1862288	0.12559954	1.48271875
1361	0.19323595	0.13038258	1.48206869
1210	0.19082126	0.12885779	1.48086702
925	0.19219035	0.12979512	1.48072085
424	0.18868733	0.12760783	1.47865012
446	-0.185735	0.12581115	-1.4762997
114	-0.1888089	0.12806816	-1.4742841
1359	-0.1855071	0.12603189	-1.4719058
729	-0.1842542	0.12554997	-1.4675769
1500	-0.1852222	0.12642313	-1.4650973

© 2024 Zhang X et al. *JAMA Pediatrics*.

486	-0.1845218	0.12611962	-1.4630694
471	-0.1838453	0.12574697	-1.4620259
1779	0.18609252	0.12735635	1.46119544
80	0.18370216	0.12626891	1.45484864
515	-0.1834764	0.12616354	-1.4542742
511	-0.1825395	0.12561207	-1.4532003
1546	-0.1825434	0.12574898	-1.4516489
728	-0.1833693	0.1263333	-1.4514722
95	-0.1832703	0.12639149	-1.4500212
479	-0.182296	0.12572302	-1.4499807
1198	-0.1840838	0.12698908	-1.4496036
1673	0.18397458	0.12700774	1.4485304
477	-0.1821546	0.12576	-1.4484303
443	-0.1819934	0.12573151	-1.4474766
1646	-0.1819522	0.12572455	-1.4472293
263	0.18296516	0.12646086	1.44681251
279	0.18391342	0.12714554	1.44647958
1663	-0.1817975	0.1257483	-1.4457252
1753	0.18412114	0.1274439	1.44472307
474	-0.1810309	0.12554747	-1.4419317
89	-0.1814486	0.12587374	-1.4415129
559	-0.1820378	0.12641148	-1.4400415
1984	0.19650219	0.1365453	1.4390989
879	-0.1806676	0.12563269	-1.438062
1004	0.18438884	0.12823814	1.43786279
1141	-0.1823796	0.12687506	-1.4374743
1602	0.18104313	0.12596111	1.43729384
942	-0.1810361	0.12636894	-1.4326
773	-0.180622	0.12623573	-1.4308311
1907	0.17939781	0.12569993	1.42719098
1919	0.17984895	0.12615306	1.42564079
147	-0.179675	0.1260921	-1.4249507
1976	0.17888195	0.12555549	1.42472431
419	-0.1784451	0.12555608	-1.4212383
567	-0.1789515	0.12603353	-1.4198722
359	0.18069976	0.12737059	1.41869295
1243	-0.1793355	0.12645596	-1.4181656
912	0.17828187	0.12576692	1.41755778
1201	-0.1781842	0.12578553	-1.4165715
1525	-0.177756	0.12579881	-1.4130179
1882	0.17867782	0.12664043	1.41090655
1841	0.17706357	0.12556943	1.41008504

© 2024 Zhang X et al. *JAMA Pediatrics*.

1528	-0.1773186	0.12576579	-1.4099114
1593	0.17896366	0.1269426	1.4097998
97	-0.1783494	0.12654319	-1.4093956
803	-0.1774633	0.12592436	-1.4092851
1849	0.17689818	0.12575264	1.40671547
711	-0.1765112	0.12595712	-1.4013594
1761	0.17556011	0.12559745	1.39780001
1205	-0.1761914	0.12650888	-1.3927199
1101	0.17606497	0.12688193	1.38762842
1913	0.17524426	0.12631029	1.38741078
1176	-0.1751641	0.12635377	-1.3862991
103	-0.1740454	0.12576869	-1.3838531
220	-0.1739345	0.12580274	-1.3825971
504	-0.1737865	0.12575405	-1.3819559
802	0.17574871	0.12721846	1.3814718
1575	-0.1738153	0.12587052	-1.3809058
1261	-0.1735577	0.12570502	-1.3806743
1229	-0.1732641	0.12551849	-1.3803871
1193	-0.175891	0.1277352	-1.3769967
1778	0.18210172	0.13251567	1.37419016
1144	-0.1725766	0.12583402	-1.3714623
1175	-0.176228	0.12869787	-1.3693154
482	-0.1718397	0.12572153	-1.3668276
1949	0.17074007	0.12551913	1.36027133
733	-0.173069	0.12784043	-1.3537892
1785	0.1709114	0.12641004	1.35203978
544	-0.1698951	0.12567187	-1.3518942
1415	0.1730126	0.12803247	1.35131814
540	-0.1697283	0.12565133	-1.3507877
922	0.1695165	0.12554337	1.35026248
1076	0.17527365	0.12988143	1.3494897
1632	-0.1697363	0.12578958	-1.3493672
1776	0.16960267	0.12583913	1.34777372
974	-0.1693619	0.1256988	-1.3473627
793	0.1776603	0.13197348	1.34618182
1552	0.16917274	0.12570234	1.34582015
1765	0.1690199	0.12560085	1.3456907
1853	0.16948226	0.12605487	1.34451184
1969	0.16843736	0.12566191	1.34040101
696	-0.1696831	0.12690861	-1.3370498
1758	0.1679897	0.12566967	1.33675606
1414	-0.1688241	0.12631175	-1.3365668

© 2024 Zhang X et al. *JAMA Pediatrics*.

287	0.17108644	0.12801485	1.33645777
828	-0.1697342	0.1271981	-1.3344087
1521	-0.1687748	0.12663862	-1.3327277
1625	-0.1675915	0.12579558	-1.3322526
801	0.16825565	0.1264306	1.33081427
1077	0.1691436	0.12741387	1.32751327
1910	0.16711365	0.12644831	1.32159654
1851	0.16712047	0.12650309	1.32107809
979	0.16648123	0.12610831	1.32014478
1804	0.16829596	0.12758291	1.31911059
1021	-0.1659679	0.12582879	-1.318998
796	0.17730602	0.13447013	1.31855318
579	-0.1656212	0.12572772	-1.3173007
101	-0.1656083	0.12584824	-1.3159363
907	0.16570733	0.12593271	1.31584023
786	0.16639249	0.12648184	1.3155445
1714	-0.1657772	0.1260307	-1.3153718
1545	-0.1653478	0.12571501	-1.3152591
512	-0.1675385	0.12746039	-1.3144359
1631	-0.1675385	0.12746039	-1.3144359
392	-0.1651868	0.12573185	-1.3138022
875	0.16494527	0.1257818	1.31136034
1775	0.16475758	0.12564921	1.3112505
965	-0.1650222	0.12608741	-1.3087923
129	-0.1646517	0.12613049	-1.3054074
667	-0.1661252	0.1272959	-1.3050319
246	-0.1645761	0.12629269	-1.3031323
1983	0.16331764	0.12574037	1.29884814
440	-0.1633134	0.12584886	-1.2976944
1835	0.16318378	0.12576243	1.29755584
1312	0.16563347	0.12767365	1.29731914
1128	-0.1628638	0.12562227	-1.2964562
278	-0.1625941	0.12553207	-1.2952395
467	-0.1623566	0.12555793	-1.2930813
395	-0.1622445	0.12553268	-1.2924481
327	0.16268255	0.12605079	1.29061108
1118	-0.1622553	0.1258672	-1.2890993
1330	-0.1620328	0.12593923	-1.2865951
900	-0.1614592	0.12576347	-1.2838322
1763	0.16131907	0.12565576	1.28381756
451	-0.1614578	0.12576662	-1.2837891
1105	-0.1614821	0.12579496	-1.2836926

© 2024 Zhang X et al. *JAMA Pediatrics*.

538	-0.1613191	0.12608515	-1.2794457
1816	0.16070779	0.12565789	1.27893113
1032	0.16153691	0.12648509	1.27712217
1382	-0.1610079	0.12624161	-1.2753952
1817	0.16008468	0.12552734	1.27529726
1706	0.17426268	0.13680723	1.27378263
857	-0.1610428	0.12656681	-1.272394
1509	-0.1595185	0.12556032	-1.2704531
1202	-0.1594253	0.12559014	-1.2694096
1951	0.16013102	0.12619667	1.26890056
669	0.16251101	0.12823774	1.26726352
1493	0.15945212	0.12595606	1.26593446
340	0.15885193	0.12555269	1.26522128
748	-0.158724	0.12562783	-1.2634465
1638	-0.1596969	0.12653103	-1.2621166
1260	-0.1584221	0.12565192	-1.2608011
1893	0.1589712	0.12616768	1.25999939
513	-0.1596637	0.12672513	-1.2599212
1332	0.15851633	0.12593859	1.25867952
1856	0.16105587	0.12798432	1.25840319
585	-0.1581359	0.12567908	-1.2582514
219	-0.158129	0.12567676	-1.2582198
202	0.16098066	0.12801507	1.25751339
532	-0.1582806	0.12594569	-1.256737
78	-0.1579895	0.12577799	-1.2560982
215	-0.157906	0.12574699	-1.2557439
226	-0.1580047	0.12589878	-1.2550135
1325	-0.1577697	0.12575261	-1.2546038
1737	0.15699494	0.12568721	1.24909249
1131	-0.1572238	0.12604291	-1.2473828
1476	-0.162077	0.13060187	-1.2410008
499	0.16293146	0.13181859	1.23602798
1996	0.15641364	0.12673612	1.23416776
61	-0.1559909	0.12640024	-1.234103
265	-0.1634475	0.13247834	-1.2337678
1239	-0.1558857	0.12636461	-1.2336188
885	-0.1575887	0.12777188	-1.2333601
1403	-0.156655	0.12721213	-1.2314468
1384	-0.1548214	0.12579491	-1.2307442
1787	0.15438949	0.12554865	1.22971843
1496	-0.1543605	0.1256468	-1.2285271
1599	-0.1560714	0.12709126	-1.2280259

© 2024 Zhang X et al. *JAMA Pediatrics*.

1871	0.15469898	0.12635721	1.22429876
1482	0.15510371	0.12692457	1.2220149
466	-0.1541638	0.12619023	-1.2216779
1328	-0.1543892	0.12646222	-1.2208326
1749	0.15309615	0.12553375	1.21956171
1613	-0.1540902	0.12642484	-1.2188285
1997	-0.1532079	0.12590589	-1.2168447
489	-0.1554751	0.12784073	-1.2161623
969	-0.1526105	0.12556323	-1.2154076
2002	-0.1525406	0.12590297	-1.2115723
1615	-0.1528867	0.12648269	-1.2087562
764	-0.15233	0.12634044	-1.2057105
1073	0.15148619	0.125919	1.20304478
1034	-0.1511383	0.12573588	-1.2020304
99	-0.151903	0.12654304	-1.2004055
56	-0.1507755	0.12575417	-1.19897
1881	0.15632111	0.13040521	1.19873367
665	0.1525506	0.12766336	1.1949443
5	-0.1504084	0.12597025	-1.1939997
1688	0.14994437	0.12560763	1.19375213
1925	0.15107124	0.12679291	1.19148018
737	-0.1498887	0.1259253	-1.1902982
128	-0.150471	0.12656376	-1.1888948
85	0.1497924	0.12621804	1.18677492
873	0.1538291	0.12962507	1.18672337
126	-0.1490917	0.12572965	-1.1858121
1109	0.1509932	0.12772528	1.18217161
1534	-0.1493613	0.12640969	-1.1815653
1624	-0.1481539	0.12564507	-1.1791465
526	-0.148501	0.12597373	-1.178825
156	0.14950646	0.12700418	1.17717746
1825	0.14890048	0.12661497	1.17601009
1093	0.14864547	0.12651114	1.17495948
1200	-0.1476677	0.12576079	-1.1741948
931	-0.1477007	0.12581518	-1.1739498
1058	-0.147583	0.12575221	-1.173602
453	-0.1474759	0.12574635	-1.172805
896	-0.1478825	0.12616977	-1.1720914
1715	-0.1475728	0.125918	-1.1719756
996	0.14822331	0.12652011	1.17153951
742	0.14822391	0.12654719	1.17129357
639	-0.1473382	0.12581322	-1.171087

© 2024 Zhang X et al. *JAMA Pediatrics*.

207	0.1475193	0.12630058	1.16800173
1008	-0.1472974	0.12614009	-1.1677284
1918	0.14669028	0.12565121	1.16744024
695	-0.1474693	0.12636561	-1.1670051
641	-0.1467965	0.12588594	-1.1661075
1999	0.14655103	0.12589479	1.16407536
1619	-0.1476359	0.12715728	-1.1610496
1561	0.14592636	0.12589005	1.15915728
1181	-0.1460077	0.1260298	-1.1585175
1584	0.1507825	0.1301807	1.1582554
1726	0.14606327	0.12614861	1.15786663
1184	-0.1456407	0.12584369	-1.1573146
1508	-0.1454357	0.12608169	-1.1535034
1186	0.14799304	0.12832223	1.15329235
1195	-0.1449669	0.12569912	-1.1532851
525	-0.1445661	0.12573897	-1.1497319
735	-0.144442	0.12571306	-1.1489813
1238	-0.14508	0.12628143	-1.1488628
13	0.14767564	0.12859661	1.14836344
42	0.14458775	0.12607838	1.14680843
1130	-0.1444887	0.12607606	-1.1460436
1937	0.15409721	0.1344817	1.14586004
17	0.14777291	0.12900149	1.14551323
1921	0.14412266	0.12584422	1.14524659
902	-0.1440464	0.12578407	-1.1451875
1031	-0.1439786	0.12579251	-1.144572
1197	-0.1438685	0.12569981	-1.1445402
1331	-0.1439084	0.12577943	-1.1441334
1967	0.1451238	0.12696869	1.14298881
1354	0.14341074	0.12566231	1.14123909
1162	-0.1434967	0.12582985	-1.1404024
98	-0.144062	0.12677935	-1.1363209
1132	-0.1437683	0.12672056	-1.1345299
1650	0.1447362	0.12767246	1.13365243
94	-0.1426171	0.12596124	-1.1322301
1262	0.14297448	0.12651741	1.13007759
1072	0.14242257	0.12604878	1.12990046
1362	0.1429347	0.12656179	1.12936697
1554	0.14353614	0.12727448	1.12776843
1649	0.14175045	0.12599686	1.12503162
1524	-0.1414747	0.12584645	-1.1241851
1738	0.14125729	0.12567501	1.12398865

© 2024 Zhang X et al. *JAMA Pediatrics*.

1773	0.14105768	0.12554015	1.12360611
933	-0.1434945	0.12773551	-1.1233719
1939	0.14306257	0.12741562	1.12280243
1478	-0.1411025	0.12567419	-1.1227647
871	-0.140913	0.12555868	-1.1222879
104	-0.1411926	0.1258438	-1.1219667
688	-0.1410939	0.12579812	-1.1215899
1033	-0.1411042	0.12581601	-1.1215124
305	-0.1406197	0.12554855	-1.1200423
1537	-0.1407447	0.1257391	-1.1193391
1012	-0.1473122	0.13174916	-1.1181264
1944	0.14016289	0.12557328	1.116184
1161	-0.1402243	0.12564718	-1.1160163
77	-0.1409005	0.12637995	-1.1148958
113	-0.1420609	0.12742082	-1.1148954
894	-0.1409335	0.12641005	-1.1148918
853	-0.1408053	0.12635257	-1.1143843
1810	0.14015422	0.12581515	1.11396935
1341	0.14094465	0.12662201	1.11311333
1092	0.14026611	0.12602867	1.11296985
1539	-0.1397677	0.12562879	-1.1125452
1533	-0.139959	0.12645455	-1.1067932
1603	0.13926781	0.12596011	1.10565012
878	0.14169908	0.12829484	1.10447996
1540	-0.1387938	0.12576781	-1.1035719
109	-0.1400589	0.12692104	-1.1035121
1511	-0.1419653	0.1286681	-1.1033449
1550	-0.1403422	0.12719921	-1.1033262
1945	0.13903166	0.12602414	1.10321451
312	0.13846257	0.12565874	1.10189361
770	-0.1397409	0.12685211	-1.101605
1920	0.13846388	0.12584862	1.10024153
1264	-0.1400855	0.12732737	-1.1001993
1806	0.13818733	0.12560503	1.10017358
923	-0.1379461	0.12578415	-1.0966888
1906	0.14086181	0.12846537	1.09649633
670	-0.1379498	0.12585049	-1.0961405
522	-0.1378089	0.12573244	-1.0960488
1473	-0.1377722	0.12576513	-1.0954721
648	-0.1377327	0.12582029	-1.0946781
1188	-0.138232	0.12638075	-1.0937744
1468	-0.1386824	0.1268702	-1.0931044

© 2024 Zhang X et al. *JAMA Pediatrics*.

1796	0.13819555	0.12680203	1.08985287
447	-0.1375171	0.12633499	-1.0885115
1191	-0.1371136	0.12608237	-1.087492
1948	0.13867103	0.12755077	1.08718303
531	-0.1364438	0.12567184	-1.085715
536	-0.1391643	0.12878153	-1.0806231
601	-0.1391643	0.12878153	-1.0806231
221	-0.1364647	0.1263213	-1.0802981
1252	-0.135802	0.12603865	-1.0774632
1204	-0.1351958	0.12576019	-1.0750285
186	-0.1348422	0.12556981	-1.0738423
34	-0.1358624	0.12658292	-1.0733073
697	-0.1348074	0.12566714	-1.0727337
1192	-0.1353603	0.12620972	-1.0725027
520	-0.1346504	0.12563652	-1.0717459
210	0.13502972	0.1260866	1.07092836
469	-0.1345919	0.12575327	-1.0702855
1581	-0.1344773	0.12568638	-1.0699432
997	-0.1341846	0.12556408	-1.0686544
381	-0.1343011	0.12576495	-1.0678741
106	-0.137571	0.12884687	-1.0677091
569	-0.1342376	0.12577097	-1.0673179
880	-0.1340659	0.12569843	-1.0665676
454	-0.1365794	0.12818872	-1.0654556
1660	-0.1344098	0.12627492	-1.0644223
1458	0.13371743	0.12563969	1.06429287
576	-0.1356212	0.12743143	-1.0642684
529	-0.1335839	0.12559519	-1.0636068
1757	0.13383276	0.12603379	1.06188
177	0.13355683	0.12612197	1.05894982
1901	0.13299546	0.12586533	1.0566489
943	-0.1335613	0.126464	-1.0561214
570	-0.132781	0.12575553	-1.0558663
132	0.13347905	0.12647856	1.05534916
956	-0.132338	0.1255652	-1.0539386
1253	-0.1334662	0.12676392	-1.0528718
1786	0.13476447	0.12810027	1.05202328
1669	0.13175542	0.12558032	1.0491725
1343	-0.1341092	0.12801283	-1.0476233
444	-0.1313993	0.12566345	-1.0456442
1007	-0.1314218	0.12571186	-1.0454205
653	0.13371506	0.12798179	1.0447975

© 2024 Zhang X et al. *JAMA Pediatrics*.

361	-0.1312222	0.12565553	-1.0443009
337	0.13143141	0.12599545	1.04314408
1059	0.13120974	0.12586322	1.04247885
1395	-0.1315086	0.12624363	-1.0417049
668	-0.1317033	0.12659614	-1.0403422
1532	-0.1318112	0.1267342	-1.0400604
96	-0.1304573	0.12562693	-1.0384501
1560	0.13093894	0.12631543	1.03660291
1302	0.13162268	0.12698656	1.0365087
1156	-0.1312273	0.12669204	-1.0357971
383	0.13000224	0.12569222	1.03429028
133	0.13157706	0.12799489	1.02798685
1519	-0.1290378	0.12565825	-1.0268948
975	-0.1292849	0.12594387	-1.026528
745	-0.1286621	0.12557228	-1.0246063
1805	0.12874698	0.1256566	1.02459385
1178	-0.1292482	0.12616725	-1.0244194
1514	-0.1288135	0.12575782	-1.0242984
679	-0.1290711	0.12601989	-1.0242125
1501	-0.1296343	0.12662338	-1.0237787
1387	-0.1286768	0.12575047	-1.023271
1781	0.12905618	0.12612476	1.02324225
1014	0.13108641	0.12821893	1.02236392
223	-0.1285061	0.12573832	-1.0220125
1952	0.12861766	0.12589924	1.02159202
1994	-0.1286907	0.12606662	-1.020815
1020	-0.1300185	0.12742719	-1.0203358
646	-0.1380017	0.13527769	-1.0201367
1230	-0.1282168	0.12570806	-1.019957
1803	0.12848637	0.12617054	1.01835475
435	0.12811254	0.12581666	1.01824781
1346	0.12799728	0.12572665	1.01806
534	-0.1284433	0.12652454	-1.0151648
145	-0.1291837	0.12793821	-1.0097355
46	-0.1299695	0.12874446	-1.0095155
1189	-0.1275142	0.1265357	-1.0077328
8	-0.1275569	0.12660951	-1.0074825
183	0.12746926	0.12654967	1.00726665
235	0.13044894	0.12970549	1.00573185
560	-0.1266636	0.12616019	-1.0039903
523	-0.1261783	0.1257015	-1.0037934
706	-0.126385	0.12603641	-1.0027659

© 2024 Zhang X et al. *JAMA Pediatrics.*

909	0.12564996	0.12557031	1.00063431
817	-0.1256346	0.12559506	-1.0003151
1574	0.1260621	0.12609087	0.99977178
982	0.12647659	0.12683773	0.99715269
1228	-0.1255952	0.12596938	-0.9970293
689	-0.125425	0.1258575	-0.9965635
851	-0.1259601	0.12655448	-0.9953035
765	-0.1280214	0.12881895	-0.9938085
1671	0.12532166	0.12613738	0.99353307
1520	-0.1248524	0.12575869	-0.9927934
747	-0.1244469	0.12561076	-0.9907343
291	-0.1243087	0.12555805	-0.9900498
535	-0.1254779	0.12719326	-0.9865138
257	-0.1243119	0.12606699	-0.9860782
224	-0.1245144	0.12628773	-0.9859579
1556	0.12380638	0.12567339	0.98514392
934	-0.1254499	0.1274049	-0.9846555
930	0.13377212	0.13633475	0.98120341
1080	0.13377212	0.13633475	0.98120341
533	-0.1236913	0.12609012	-0.9809753
508	-0.1252276	0.12768369	-0.9807642
613	-0.1256987	0.12827376	-0.9799253
1756	0.12302613	0.12554957	0.97990091
1570	0.12340002	0.12595909	0.97968331
437	-0.1225266	0.12560428	-0.9754968
1498	-0.1232639	0.12651954	-0.9742675
2007	-0.1225009	0.12577541	-0.9739652
222	-0.1227722	0.12619016	-0.9729143
62	-0.125039	0.1285812	-0.972452
247	0.12298954	0.12651495	0.9721345
1295	-0.1223114	0.12582858	-0.9720481
1696	-0.1220561	0.12601575	-0.9685778
1182	-0.1220597	0.12602305	-0.9685504
675	-0.121864	0.12582511	-0.9685192
795	-0.1218075	0.12601006	-0.9666491
1071	0.12143358	0.12571007	0.96598133
277	0.12334522	0.12781487	0.96503027
687	-0.1215894	0.12614805	-0.9638628
1199	-0.1221334	0.12688054	-0.962586
1655	0.12179787	0.1266906	0.9613805
93	-0.1216119	0.12653865	-0.9610653
1522	-0.120738	0.12566588	-0.9607862

© 2024 Zhang X et al. *JAMA Pediatrics*.

1221	-0.1208975	0.12606205	-0.959032
1125	-0.1211131	0.1263185	-0.9587918
1254	-0.1210105	0.12623322	-0.9586268
1890	0.12029544	0.12557225	0.95797792
593	-0.1203412	0.12575173	-0.9569746
107	-0.1209913	0.12654587	-0.9561061
69	-0.130174	0.13622107	-0.9556087
105	-0.1208128	0.12654796	-0.9546796
82	0.12120554	0.12750059	0.95062728
1041	0.12163305	0.12799975	0.95026006
1296	-0.1193308	0.12573305	-0.9490802
1746	0.11915019	0.12555199	0.9490107
621	-0.1194881	0.12604596	-0.9479724
1173	0.11891963	0.12555627	0.94714211
475	-0.1189298	0.12565202	-0.9465011
1196	-0.1209544	0.12779143	-0.9464987
1616	-0.1188962	0.12607459	-0.9430625
32	-0.1189374	0.12638031	-0.9411069
740	-0.1200038	0.12752529	-0.9410193
528	-0.1178093	0.1255629	-0.9382496
1993	0.11917306	0.12702705	0.93817071
1143	-0.1183243	0.12625527	-0.9371832
1085	0.11812374	0.12623604	0.93573705
1544	-0.1175866	0.12566849	-0.9356892
1936	0.11861441	0.12676897	0.93567389
725	-0.1175047	0.12576373	-0.9343287
1251	-0.1177679	0.12605632	-0.9342481
1506	-0.1181966	0.12665046	-0.9332504
1517	-0.1171652	0.12583704	-0.9310865
1512	-0.116742	0.12559529	-0.9295094
1518	-0.1169838	0.12589625	-0.9292083
961	0.11729383	0.12640359	0.92793116
227	-0.1182455	0.12744358	-0.9278266
1610	-0.1166206	0.12582439	-0.9268524
505	-0.1199209	0.12960938	-0.9252486
574	-0.1162332	0.1257345	-0.9244338
1648	-0.1161087	0.12629019	-0.9193805
2003	-0.1157735	0.12605247	-0.9184552
904	-0.1152772	0.12555556	-0.9181373
1338	0.11648828	0.12717252	0.91598625
1762	0.11486661	0.12557538	0.91472238
481	-0.1164939	0.12748101	-0.9138138

© 2024 Zhang X et al. *JAMA Pediatrics.*

516	-0.116148	0.12711264	-0.9137407
610	-0.1151592	0.12620818	-0.912454
1391	-0.114848	0.12592978	-0.9120002
939	-0.1153451	0.12654756	-0.9114764
1912	0.11589373	0.12721892	0.91097874
472	-0.1152884	0.12661322	-0.9105561
612	-0.114728	0.12600928	-0.9104729
1899	0.11585483	0.12731018	0.91002014
941	-0.1150808	0.12646652	-0.9099707
53	-0.1151275	0.12658155	-0.9095127
12	0.11486604	0.12651796	0.90790299
413	0.11487744	0.12696217	0.90481626
414	-0.113705	0.1256922	-0.9046303
290	-0.1134635	0.12556378	-0.9036326
1456	-0.1143712	0.12694168	-0.9009746
362	-0.1128802	0.12560835	-0.8986679
850	0.11617351	0.12927616	0.89864602
1248	-0.1127895	0.12561808	-0.8978762
1402	-0.1187213	0.13235602	-0.8969846
1475	-0.1128697	0.12589549	-0.8965353
940	-0.1132691	0.12639134	-0.8961774
731	-0.1125388	0.12557748	-0.8961706
1464	0.1126514	0.12592723	0.89457543
49	-0.1165608	0.13040136	-0.8938618
718	-0.1145592	0.12837133	-0.8924045
539	-0.1128317	0.12651699	-0.8918301
1319	0.11195192	0.12559983	0.8913382
1168	0.11205917	0.12585741	0.89036609
157	-0.1119659	0.12605957	-0.8881987
928	0.11218416	0.12643778	0.88726773
1859	0.1116904	0.12594454	0.88682209
868	-0.1113381	0.12555601	-0.8867603
819	-0.1113365	0.12555612	-0.8867468
866	-0.1113365	0.12555612	-0.8867468
622	0.11377736	0.12848705	0.88551616
2006	-0.111233	0.12564027	-0.885329
746	-0.1111939	0.12560756	-0.8852481
211	-0.1121346	0.12668827	-0.8851223
566	-0.1113073	0.12576586	-0.885036
455	-0.1122162	0.12686415	-0.884538
553	-0.1111535	0.12585105	-0.8832149
338	0.11088996	0.125871	0.88098101

© 2024 Zhang X et al. *JAMA Pediatrics*.

1526	-0.1110901	0.12628334	-0.879689
1583	0.11064339	0.12581144	0.8794382
776	-0.1108665	0.12609864	-0.8792048
353	-0.1123524	0.12784685	-0.878805
1064	-0.1106416	0.12604526	-0.8777926
1220	0.11083767	0.12630623	0.87753135
1429	0.11673836	0.1331918	0.87646807
530	-0.1108808	0.12651048	-0.8764555
1068	0.11008086	0.12562108	0.8762929
938	-0.1107688	0.12648554	-0.8757431
1440	-0.1098904	0.12557415	-0.8751038
1113	-0.1103499	0.12610186	-0.8750856
178	0.10983815	0.12556711	0.87473667
629	-0.1109336	0.1268328	-0.8746447
1163	-0.1106847	0.12663367	-0.8740541
1848	0.10994498	0.12607221	0.87207943
1846	0.10937659	0.12566621	0.87037396
732	-0.10917	0.1256023	-0.869172
432	-0.1089489	0.12591179	-0.8652798
1850	0.10955702	0.12663448	0.86514368
820	-0.1085961	0.12555733	-0.8649125
867	-0.1085946	0.1255575	-0.8648996
1409	-0.1085421	0.12566078	-0.8637705
1592	0.10857595	0.12580461	0.86305226
1174	-0.1085129	0.12575048	-0.8629222
812	-0.1086708	0.12603577	-0.8622215
1219	-0.1104011	0.12806742	-0.8620541
1548	-0.1089169	0.12635245	-0.862009
840	-0.1099311	0.12762408	-0.8613666
276	0.10853346	0.1261393	0.86042546
1573	0.10806048	0.12562934	0.86015321
813	-0.1082404	0.12588761	-0.8598174
652	0.11123022	0.12946856	0.85912916
325	0.10750612	0.12558317	0.85605516
1112	0.10757426	0.12574077	0.8555241
91	-0.107329	0.12565501	-0.8541558
1538	-0.1071981	0.12565051	-0.8531449
1288	0.10714447	0.12574725	0.8520621
586	-0.1075289	0.12636469	-0.8509412
1306	-0.1070896	0.12589533	-0.8506237
1880	0.10658823	0.12565266	0.84827672
1740	0.10641922	0.12556858	0.84749878

© 2024 Zhang X et al. *JAMA Pediatrics.*

785	-0.1089639	0.12882725	-0.8458137
1137	-0.1089639	0.12882725	-0.8458137
1259	-0.1089639	0.12882725	-0.8458137
583	-0.1062695	0.12577158	-0.8449402
1774	0.10613627	0.12579226	0.8437425
1617	-0.1061955	0.12596054	-0.8430853
1111	-0.1057786	0.12573035	-0.8413136
390	-0.1059671	0.1261117	-0.8402641
1110	-0.1059913	0.12618985	-0.8399355
771	-0.1070979	0.12768722	-0.8387517
1569	0.10533019	0.12574621	0.83764105
1637	-0.1056154	0.12616052	-0.8371507
1720	0.105132	0.12604978	0.83405147
565	-0.1054034	0.12649049	-0.8332911
11	0.10594526	0.1271487	0.83323908
229	-0.104623	0.12572489	-0.832158
1427	0.10651161	0.12815339	0.83112596
1249	-0.1045294	0.12589899	-0.8302636
1710	-0.1075435	0.12964775	-0.8295053
339	-0.1041612	0.12557285	-0.8294883
406	-0.1041612	0.12557285	-0.8294883
548	-0.1041777	0.12594377	-0.8271763
380	0.10387736	0.12563759	0.82680156
1860	0.10572751	0.12794216	0.82636961
877	0.10702349	0.12959735	0.82581544
1266	-0.1039123	0.12594493	-0.8250618
286	-0.1035096	0.12564729	-0.8238111
348	-0.1027796	0.12567495	-0.817821
334	-0.1026812	0.12557297	-0.8177014
1799	0.10663014	0.13050075	0.81708453
1858	0.10258324	0.12557963	0.816878
552	-0.1027488	0.1258444	-0.8164746
752	-0.1030349	0.12619924	-0.8164466
588	-0.1039338	0.12733183	-0.8162436
1405	-0.1032857	0.12659207	-0.8158941
784	-0.1030865	0.12646679	-0.8151272
898	0.10610157	0.13033481	0.81406936
1780	0.10224334	0.1256694	0.81358977
1158	-0.1023682	0.12593578	-0.81286
363	-0.1020719	0.12557373	-0.8128443
1376	-0.1021485	0.1257881	-0.8120682
719	0.10223057	0.12602471	0.81119461

© 2024 Zhang X et al. *JAMA Pediatrics.*

1797	0.10444213	0.12878582	0.81097541
1513	-0.1018303	0.12561112	-0.8106791
1620	-0.1020785	0.12629531	-0.8082521
139	-0.1022048	0.12654836	-0.8076345
1234	-0.1017822	0.12609614	-0.8071795
899	-0.1012203	0.12557243	-0.8060707
1886	0.10117057	0.12567417	0.80502277
130	0.10176113	0.12644001	0.80481753
1069	0.10107616	0.12561959	0.80462101
1820	0.10102882	0.12556991	0.80456231
323	0.10102657	0.12558325	0.80445897
1930	0.10101601	0.12580438	0.802961
988	0.10104179	0.12592663	0.80238622
1150	-0.1006831	0.12556875	-0.8018167
839	0.1016583	0.12686099	0.80133618
410	-0.1005927	0.12556403	-0.8011264
1794	0.10080431	0.12598756	0.80011323
1396	-0.1019199	0.12746139	-0.7996142
316	0.10050653	0.12601042	0.79760487
1855	0.1008754	0.12654714	0.79713699
1730	0.09990729	0.12557889	0.79557392
492	0.10091921	0.12690351	0.79524367
546	-0.1002578	0.12609735	-0.7950822
1703	-0.1102718	0.13878497	-0.7945514
389	-0.0996324	0.12557442	-0.793413
542	-0.1000235	0.12618757	-0.7926576
1039	0.09987959	0.12600663	0.79265347
993	-0.0994601	0.12624807	-0.7878149
329	0.09903034	0.12577123	0.78738467
1777	0.09877066	0.12558101	0.78650954
115	0.09896708	0.12595717	0.78572014
118	-0.0990112	0.12650641	-0.7826573
618	0.09903709	0.12695516	0.78009506
1461	-0.0984749	0.1263893	-0.7791395
502	-0.0981839	0.12604137	-0.7789819
594	-0.0982545	0.12618907	-0.7786293
571	-0.0978182	0.12572952	-0.7780054
1661	0.09774671	0.12632988	0.77374179
587	-0.0972111	0.1256427	-0.7737109
831	-0.0975545	0.12621494	-0.7729231
1256	-0.0972376	0.12590237	-0.7723258
1138	-0.1030104	0.13356618	-0.7712308

© 2024 Zhang X et al. *JAMA Pediatrics.*

647	-0.0978112	0.1268736	-0.7709339
1194	-0.0974139	0.12659874	-0.7694694
1633	0.09661347	0.12586271	0.76760994
958	-0.0963596	0.12562147	-0.7670635
1350	0.09659602	0.12612295	0.76588773
1399	-0.0961362	0.12565833	-0.76506
1941	0.09769793	0.12800988	0.76320615
1272	-0.0961886	0.12603985	-0.7631604
1887	0.0958845	0.12568949	0.76286809
1426	0.09589527	0.12616303	0.76009011
838	0.0983047	0.12933976	0.76005009
134	0.09976488	0.13126905	0.76000306
1300	0.09564179	0.12588952	0.75972799
1366	-0.0957789	0.12618533	-0.7590339
452	0.09539808	0.12573902	0.75869914
972	-0.0954385	0.1258227	-0.7585159
1813	0.09751641	0.12861978	0.75817588
1190	-0.0955627	0.12609463	-0.7578648
1716	-0.0953726	0.12590346	-0.7575061
800	0.09592528	0.12675545	0.75677438
1001	0.09502989	0.12574538	0.7557326
1713	-0.0948805	0.12577889	-0.7543435
464	0.09492629	0.125844	0.75431715
1485	0.0954177	0.1265849	0.75378426
1876	0.09481726	0.12579589	0.753739
1904	0.09524259	0.1263912	0.75355399
702	-0.0962552	0.12780168	-0.7531609
1203	-0.0955133	0.12711392	-0.7513994
1236	-0.0946033	0.12590341	-0.7513961
822	0.09721189	0.12963406	0.74989462
1651	0.09491957	0.1274207	0.74493057
881	-0.0935197	0.12574814	-0.7437061
921	0.09378415	0.12621438	0.74305437
1180	-0.0928514	0.12560005	-0.7392624
543	0.09307744	0.12593344	0.73910033
1044	0.09287956	0.12568166	0.73900648
558	-0.0928138	0.12560675	-0.7389235
1735	-0.0957044	0.12968592	-0.7379707
1940	0.09263714	0.12565976	0.73720605
1700	-0.0963417	0.13073646	-0.7369154
1664	-0.0925324	0.12561783	-0.736618
415	-0.0924689	0.12571061	-0.7355697

© 2024 Zhang X et al. *JAMA Pediatrics*.

698	-0.0925641	0.12587637	-0.7353572
623	-0.0926109	0.1259656	-0.7352079
28	-0.0929981	0.12661802	-0.7344776
1102	0.09309142	0.1271956	0.73187612
1286	-0.0923691	0.1264064	-0.7307313
738	-0.0921719	0.12617072	-0.7305334
863	0.09183094	0.12583515	0.72977176
1027	-0.0917091	0.12581252	-0.7289349
1187	-0.0923091	0.12671472	-0.7284797
947	-0.0941027	0.12935634	-0.7274688
547	-0.0916144	0.12610959	-0.7264666
1992	0.09207887	0.12682831	0.72601199
1957	0.09111441	0.1255876	0.72550484
1269	-0.0921967	0.12710553	-0.7253555
198	-0.0921618	0.12776842	-0.7213192
1523	-0.0905005	0.12571151	-0.7199065
335	-0.0903322	0.12559121	-0.7192559
1451	0.09029885	0.1255635	0.71914893
1114	-0.0903339	0.12566617	-0.71884
1665	-0.0903028	0.12563626	-0.7187638
906	-0.0903803	0.12580458	-0.7184181
915	0.09143706	0.12728079	0.71838856
1657	0.091457	0.12734539	0.71818065
1486	0.090804	0.126564	0.71745521
267	-0.0901069	0.12571274	-0.7167679
1151	-0.0917578	0.12804326	-0.7166156
1527	-0.0900735	0.1256951	-0.7166027
92	0.09043242	0.12622302	0.71644953
1541	-0.0902292	0.12601098	-0.7160421
572	-0.0899518	0.12606586	-0.7135299
1896	0.08976654	0.12581012	0.71350808
1115	0.08970477	0.12578424	0.71316384
1274	0.09030285	0.12673508	0.71253243
43	-0.0945005	0.13270622	-0.7121032
1244	-0.089451	0.12564485	-0.7119356
1063	0.09008922	0.12669647	0.71106337
1060	0.09002555	0.12678405	0.71007004
354	0.08913214	0.12572805	0.70892802
445	0.08886539	0.12556651	0.70771567
1759	0.08908851	0.1260168	0.70695741
436	-0.0898432	0.12720286	-0.7062985
391	-0.0893188	0.12662953	-0.7053549

© 2024 Zhang X et al. *JAMA Pediatrics.*

336	-0.0885048	0.12558804	-0.7047228
777	-0.0884381	0.12596764	-0.70207
236	-0.088975	0.12682373	-0.7015641
342	0.08802905	0.12559708	0.70088453
405	0.08802905	0.12559708	0.70088453
1263	-0.0885456	0.12658607	-0.6994891
9	0.08809386	0.12619864	0.69805708
1798	0.09146147	0.13105133	0.6979057
293	-0.0876082	0.12569464	-0.696992
16	0.08846206	0.12695526	0.69679712
734	-0.0874358	0.12581535	-0.6949533
1727	0.08722806	0.12565249	0.69420079
206	-0.0883905	0.12736653	-0.6939852
1630	-0.0873655	0.1261336	-0.6926428
460	-0.0876999	0.12676786	-0.6918152
1989	0.0869858	0.12574646	0.69175549
1223	-0.0881915	0.12763236	-0.690981
509	-0.0881232	0.1275381	-0.6909561
1397	-0.0878177	0.12711427	-0.6908564
1986	0.08686862	0.12574336	0.69084065
1233	-0.0870835	0.12608068	-0.6906964
1335	0.08722033	0.12635783	0.69026457
756	0.08702904	0.12652667	0.68783156
634	0.08750769	0.12729113	0.68746099
31	-0.086341	0.12564919	-0.687159
1121	0.0860728	0.12562763	0.68514225
1898	-0.0869488	0.12692976	-0.6850151
360	-0.086076	0.12570117	-0.6847671
1729	0.08574577	0.12570818	0.68210182
1529	-0.0856969	0.12579116	-0.6812635
23	-0.0855752	0.12562407	-0.6812004
1116	-0.0854413	0.12557313	-0.6804105
431	-0.0854366	0.12557792	-0.6803477
1628	0.08564205	0.12591654	0.68014933
172	0.0864121	0.12706053	0.68008609
1955	0.08537291	0.12559396	0.67975333
1956	0.08536863	0.12559304	0.67972419
121	0.08549675	0.12588249	0.67917909
722	0.08911285	0.13120772	0.67917384
1811	0.08673284	0.12792661	0.67798909
1241	-0.0856815	0.12641167	-0.6777975
671	-0.0853705	0.12595471	-0.6777877

© 2024 Zhang X et al. *JAMA Pediatrics.*

1670	0.08596177	0.12701071	0.67680726
568	-0.0852928	0.12609	-0.676444
1081	0.08688647	0.12853227	0.67598956
1739	-0.0850387	0.1258718	-0.675598
562	-0.0848234	0.12559561	-0.6753693
1389	-0.0847397	0.12577817	-0.6737237
1672	0.08528368	0.1265897	0.67370159
694	-0.0845447	0.125794	-0.6720882
10	-0.0853802	0.12704377	-0.6720536
394	-0.0843445	0.12559917	-0.6715372
1018	-0.0848349	0.12657493	-0.6702342
1704	-0.0917169	0.13696756	-0.6696249
345	0.08412344	0.12568038	0.6693443
304	0.08394045	0.12558738	0.66838286
1741	0.08391937	0.12557427	0.6682848
1611	0.08386175	0.12620137	0.66450743
642	-0.0871407	0.13120892	-0.6641372
1472	0.0837325	0.12662967	0.66123917
430	-0.0828416	0.12556994	-0.6597251
1680	-0.0865228	0.1312413	-0.6592647
968	-0.0832722	0.12632829	-0.6591726
4	-0.0840861	0.12771404	-0.6583938
258	-0.082766	0.12571343	-0.6583703
1298	-0.0836535	0.12712051	-0.6580641
577	-0.0828464	0.12603069	-0.6573508
1980	0.08248685	0.1255914	0.65678743
181	-0.0828875	0.12635811	-0.6559726
595	0.08386875	0.12789439	0.65576562
119	-0.0826032	0.12597666	-0.6557021
188	-0.0830262	0.12698151	-0.6538448
1950	0.08786868	0.13492689	0.6512318
1530	-0.0818521	0.12579249	-0.6506915
1579	-0.0816738	0.12614347	-0.6474672
1728	0.08112465	0.12556738	0.64606467
374	-0.0809656	0.12561263	-0.6445659
212	0.08100257	0.12587348	0.6435237
651	-0.082182	0.12775237	-0.6432917
397	-0.0812879	0.1263997	-0.6431016
1736	0.08246372	0.128351	0.642486
1562	0.08090841	0.12606041	0.64182254
1070	0.08055441	0.12587933	0.63993359
154	0.08057999	0.12621618	0.63842837

© 2024 Zhang X et al. *JAMA Pediatrics*.

755	0.08044958	0.12601822	0.63839641
932	0.08112028	0.12710628	0.63820826
163	-0.0816751	0.12799811	-0.6380961
1388	-0.0803401	0.12592979	-0.6379757
59	-0.0819599	0.12888026	-0.6359385
927	-0.0808431	0.12721946	-0.6354616
524	-0.0806248	0.12688838	-0.6353991
1516	-0.0797709	0.12594682	-0.6333698
521	-0.0806588	0.12750217	-0.632607
448	0.07967204	0.12594899	0.63257386
496	0.08013318	0.12672903	0.63231903
592	-0.0812211	0.12890269	-0.6300965
677	-0.0797444	0.12667544	-0.6295175
821	-0.0790123	0.12602035	-0.6269805
782	-0.079034	0.1260585	-0.6269628
1347	0.07903568	0.12606549	0.62694142
264	0.08014568	0.12849942	0.62370458
3	-0.0787771	0.12646911	-0.6228962
721	-0.0782406	0.12561722	-0.6228492
693	0.07828103	0.12571318	0.62269543
1572	0.07836177	0.12600253	0.62190629
1270	-0.0782391	0.12581343	-0.6218661
122	0.07840787	0.12621606	0.62121949
1733	0.07815037	0.12586868	0.62088809
238	-0.0786266	0.12668701	-0.6206366
19	-0.0779092	0.12589478	-0.6188441
123	0.07815713	0.12630891	0.61877762
556	-0.0778842	0.12602092	-0.6180259
827	-0.0783488	0.12687866	-0.6175099
854	-0.0777548	0.12600874	-0.6170589
112	-0.0776848	0.12592265	-0.6169246
701	-0.0777891	0.12618093	-0.6164887
1731	0.07761523	0.12602497	0.61587183
973	-0.0774875	0.12583341	-0.6157946
717	-0.0780279	0.12672089	-0.6157459
1450	0.07715174	0.12557396	0.61439283
297	-0.0770844	0.12569196	-0.6132803
1022	0.07691347	0.12570542	0.61185485
142	-0.0767989	0.12580352	-0.6104668
1897	0.07681333	0.12584117	0.61039909
1830	0.07662711	0.12557071	0.61023077
986	-0.0776214	0.12764435	-0.6081066

© 2024 Zhang X et al. *JAMA Pediatrics.*

1985	0.07632066	0.12556889	0.60779913
90	-0.0764387	0.12613019	-0.6060302
1502	-0.0761436	0.12564654	-0.6060144
280	0.07784345	0.12859835	0.60532232
1214	-0.0760008	0.12587938	-0.6037587
1094	0.07682703	0.12735991	0.60322773
1117	-0.0759016	0.12589014	-0.6029196
963	-0.0761825	0.12639509	-0.6027332
285	0.0769845	0.12774381	0.6026476
384	-0.0756682	0.12584937	-0.6012598
1697	-0.0756772	0.12588242	-0.6011734
262	-0.0756013	0.12577389	-0.6010891
171	0.07575825	0.12638715	0.59941421
149	-0.075234	0.12561753	-0.5989134
1051	0.07694282	0.12851777	0.598694
1954	0.07514407	0.12559766	0.59829192
174	-0.075215	0.12641722	-0.594974
712	-0.0752417	0.12666948	-0.5940002
959	-0.0746939	0.12579839	-0.5937585
758	-0.0745728	0.12566834	-0.59341
63	-0.0755235	0.12730161	-0.5932642
158	0.07500652	0.12654867	0.59270886
214	-0.0746025	0.12620786	-0.5911083
1589	-0.0743005	0.12570948	-0.5910494
967	-0.0742142	0.12563059	-0.5907334
1313	-0.0742518	0.12570298	-0.5906928
1744	0.07450607	0.1267818	0.58767164
1418	-0.0746123	0.12700453	-0.5874772
580	-0.0745446	0.12704937	-0.5867369
1953	0.0736846	0.125597	0.58667483
609	-0.073919	0.12617211	-0.5858587
617	0.07474624	0.12761896	0.58569856
1090	-0.0737211	0.12615861	-0.5843526
1126	-0.0734049	0.1257606	-0.5836875
1342	-0.0748003	0.12830036	-0.5830096
346	-0.0732065	0.12558083	-0.5829434
537	0.07364809	0.12634989	0.58289002
911	0.07840273	0.13450825	0.58288419
674	-0.0732675	0.12576238	-0.5825867
1678	0.07326457	0.12577759	0.58249305
1674	0.07427324	0.1275225	0.58243247
1035	-0.0732337	0.1259853	-0.5812876

© 2024 Zhang X et al. *JAMA Pediatrics.*

253	-0.0729702	0.12565147	-0.5807349
387	-0.0733565	0.126771	-0.578654
788	0.07383894	0.12767565	0.5783322
686	0.07312236	0.12663535	0.57742451
378	-0.0727882	0.12607771	-0.5773281
1222	-0.0750464	0.13008515	-0.5769024
856	0.07284994	0.12680642	0.57449722
300	-0.0720986	0.12562731	-0.5739087
167	0.07269754	0.12671793	0.57369575
50	-0.072468	0.12643877	-0.573147
1369	-0.0721684	0.1259449	-0.5730159
681	-0.0719477	0.1255748	-0.5729472
1499	-0.0742414	0.12972684	-0.5722903
1802	0.07219042	0.12614721	0.57227122
463	-0.0719405	0.12577387	-0.5719832
1372	-0.0718815	0.12572439	-0.5717384
1454	0.0722495	0.12671542	0.57017135
332	-0.0717478	0.12641464	-0.5675591
1299	0.07343018	0.12940589	0.5674408
72	-0.071248	0.12565186	-0.5670269
1371	-0.0712081	0.12565569	-0.5666925
1419	-0.0715526	0.12641614	-0.5660086
1334	-0.0711556	0.12601231	-0.564672
1245	-0.071551	0.12682136	-0.5641877
1564	-0.0706482	0.12609883	-0.5602606
1285	-0.0704348	0.12576883	-0.560034
18	0.07205873	0.12880705	0.55943152
117	-0.0709381	0.12696926	-0.5587026
364	-0.070056	0.12559324	-0.5578009
790	-0.069976	0.12557974	-0.557224
1320	0.06987282	0.12569592	0.5558877
175	-0.072528	0.13066383	-0.5550735
1801	0.06970688	0.12563381	0.55484173
217	-0.070179	0.12650642	-0.5547465
125	-0.0702444	0.12666972	-0.5545474
1065	-0.0701726	0.12674775	-0.5536397
843	-0.0699729	0.12642825	-0.553459
1106	0.06950839	0.12594121	0.55191139
1972	0.06986841	0.1270932	0.54974151
1682	0.06992014	0.12729965	0.54925634
155	-0.0699555	0.12745759	-0.5488535
935	-0.0692049	0.12611159	-0.5487596

© 2024 Zhang X et al. *JAMA Pediatrics.*

1565	0.06899864	0.12604711	0.5474036
1535	0.06951263	0.12698906	0.54739064
1087	-0.0690687	0.1267593	-0.544881
1315	-0.0685369	0.12587487	-0.5444845
22	-0.0683946	0.12563195	-0.5444047
1265	-0.0686978	0.12622597	-0.5442448
1695	-0.0684697	0.12584707	-0.5440705
1294	0.06832929	0.12583927	0.54298866
1686	-0.0683796	0.12610429	-0.5422468
1725	0.06891684	0.12729096	0.5414119
1566	-0.0684762	0.12653874	-0.5411481
302	0.06796195	0.12587064	0.53993487
1349	0.06823518	0.12638872	0.53988347
1445	0.06754533	0.12570896	0.53731519
807	-0.067879	0.12638868	-0.5370657
783	-0.0690296	0.12880837	-0.5359093
26	-0.0673828	0.12583164	-0.5354994
1215	-0.0672498	0.12564888	-0.5352203
1515	-0.0676727	0.12664783	-0.5343379
507	-0.067666	0.12677404	-0.5337527
781	-0.0672285	0.12623467	-0.5325677
1246	-0.0671504	0.12623513	-0.5319469
416	-0.0666464	0.1255766	-0.5307232
1280	-0.0666121	0.12561669	-0.530281
1375	-0.0666194	0.12563529	-0.53026
1284	0.067009	0.12645208	0.52991612
1374	0.06667763	0.12605285	0.52896569
519	-0.0664064	0.1256828	-0.5283652
518	-0.0663901	0.12613153	-0.526356
1240	0.06616986	0.1259157	0.52550919
1139	-0.0673485	0.12818747	-0.5253909
1681	-0.0659627	0.12572689	-0.5246503
1447	0.0663989	0.1265841	0.52454378
483	-0.0660843	0.12615185	-0.5238474
846	-0.0659398	0.12604668	-0.523138
1390	-0.0657379	0.12575574	-0.5227427
1582	-0.0667407	0.12775676	-0.5224048
1684	-0.0655633	0.12564729	-0.5218042
1075	-0.0679651	0.13037007	-0.5213247
1577	-0.0677311	0.12996725	-0.52114
1693	-0.0656672	0.1261476	-0.5205588
726	-0.0665342	0.12785719	-0.5203791

© 2024 Zhang X et al. *JAMA Pediatrics.*

541	-0.0654131	0.1258017	-0.5199702
420	0.06534633	0.12587012	0.51915682
645	-0.0651135	0.12617024	-0.5160769
240	0.06479618	0.12568638	0.51553856
243	0.06524907	0.12659482	0.5154166
1108	-0.0648449	0.12581469	-0.5154002
1543	-0.0650968	0.12667232	-0.5138988
573	-0.0645478	0.12564639	-0.5137257
228	0.06481852	0.12638035	0.51288448
811	-0.0646114	0.12607059	-0.5125017
1578	-0.0654338	0.12778135	-0.5120766
792	-0.0642817	0.12568515	-0.51145
608	-0.0643501	0.12583266	-0.5113942
25	-0.0654485	0.12798494	-0.5113762
1145	-0.0642233	0.12566871	-0.5110524
1127	0.06420238	0.12572379	0.51066213
314	-0.0640182	0.12586692	-0.5086183
865	0.06721478	0.13223421	0.50830097
527	-0.0647117	0.12754499	-0.5073637
1355	0.06528834	0.12886234	0.50665182
288	-0.0635537	0.12566635	-0.5057333
1304	0.06376131	0.12616439	0.50538276
159	0.06466026	0.12796722	0.50528773
691	-0.0638557	0.12654185	-0.5046213
216	0.06341253	0.12584596	0.50389004
683	-0.0636234	0.1265438	-0.5027774
1055	0.06325703	0.12587993	0.50251883
1895	0.06320135	0.12581987	0.50231614
1894	0.06314581	0.12574174	0.50218651
1555	0.06324434	0.12619262	0.50117309
1691	-0.0629282	0.12566135	-0.5007764
1640	-0.0638952	0.12771974	-0.5002766
861	-0.063233	0.1265127	-0.4998158
825	-0.0631616	0.12640987	-0.4996569
1705	-0.0628719	0.12614933	-0.4983928
135	0.06314863	0.12670489	0.49839144
1206	-0.0624354	0.12585258	-0.4960996
1235	-0.0629774	0.12743466	-0.4941936
1281	0.06272103	0.12702188	0.4937813
1411	-0.0623223	0.12630153	-0.4934407
1292	-0.0625442	0.12682848	-0.4931397
1683	-0.0631088	0.12803324	-0.4929092

© 2024 Zhang X et al. *JAMA Pediatrics*.

385	-0.0619045	0.12569651	-0.4924918
774	-0.0621013	0.12635608	-0.4914783
1129	0.06179493	0.12576777	0.49134151
637	0.0618715	0.12593828	0.49128428
1227	-0.0619229	0.12608752	-0.4911101
762	-0.0633717	0.12919913	-0.4904966
682	-0.0615889	0.12579833	-0.4895844
816	-0.0615253	0.1257228	-0.4893724
1721	-0.0614515	0.12560724	-0.4892354
493	0.06214231	0.12703481	0.48917545
1218	-0.0616876	0.12654239	-0.4874855
864	0.06221995	0.12769222	0.48726502
485	0.06168415	0.1266107	0.48719537
1301	-0.0616008	0.12659836	-0.4865843
1483	-0.0611134	0.12576767	-0.4859233
1639	0.06359686	0.13096946	0.48558541
929	0.06081397	0.12559243	0.48421686
1836	0.0620896	0.12824447	0.48415025
201	0.06164011	0.12732312	0.48412348
409	-0.0610086	0.12608196	-0.4838801
727	-0.0617766	0.12795122	-0.4828137
1333	0.06216162	0.12880955	0.48258551
313	0.06062337	0.12607591	0.48084812
73	-0.0607799	0.12656781	-0.4802158
1479	-0.0606838	0.12641473	-0.4800374
987	-0.0608158	0.12681791	-0.4795519
1988	0.06015514	0.12562334	0.47885324
1410	-0.0600302	0.12567943	-0.4776455
1091	0.06052021	0.12711453	0.47610776
832	-0.0597227	0.12574449	-0.4749525
6	0.05979657	0.12629902	0.47345233
964	-0.0594935	0.12576256	-0.473062
170	-0.0593198	0.1259119	-0.4711212
917	-0.0590973	0.12565092	-0.4703292
739	-0.0591426	0.12622459	-0.4685503
1717	-0.0590589	0.12605463	-0.4685182
1421	0.05877681	0.12557352	0.46806691
1644	-0.0587222	0.12566588	-0.4672881
438	-0.0588236	0.12596935	-0.4669678
611	0.05874519	0.12585678	0.46676222
1436	-0.059087	0.12664076	-0.4665715
457	-0.0589101	0.12627563	-0.4665199

© 2024 Zhang X et al. *JAMA Pediatrics.*

852	-0.0586089	0.12568359	-0.4663213
980	-0.0587668	0.12603961	-0.4662569
1437	-0.058613	0.12582011	-0.4658478
1003	0.05868463	0.12600668	0.46572636
549	-0.0586557	0.12603233	-0.4654023
270	0.05920516	0.12746339	0.46448757
1394	-0.0586186	0.12641665	-0.4636939
761	-0.0582659	0.12648883	-0.460641
365	-0.0576917	0.12558332	-0.45939
1788	0.05789371	0.12641281	0.45797347
230	0.05779724	0.12631854	0.45755154
76	-0.0575847	0.125913	-0.457337
1557	0.05753434	0.12598645	0.45667085
1043	0.05704423	0.12558946	0.45421191
1747	0.05703686	0.12557544	0.45420394
1364	0.05732575	0.12629436	0.45390584
75	-0.0569999	0.12591477	-0.4526862
168	-0.0581109	0.1285495	-0.452051
213	-0.0571748	0.12670167	-0.4512551
736	-0.0570628	0.1268878	-0.4497107
835	0.05708065	0.1270872	0.44914553
48	-0.0565871	0.12625503	-0.4481965
625	0.05698378	0.12716722	0.44810116
1104	0.06054335	0.13579991	0.44582759
146	-0.0569342	0.12816267	-0.4442336
1078	-0.0574489	0.12938081	-0.4440296
131	0.05562194	0.12567971	0.44256893
1793	0.05569417	0.12586029	0.4425079
79	-0.0560832	0.12675968	-0.4424373
352	0.05561709	0.12579922	0.44210997
989	-0.0556193	0.12582359	-0.4420417
582	0.05566061	0.12599583	0.44176547
814	0.05550093	0.1256486	0.44171543
1242	-0.0556855	0.12646485	-0.4403236
602	-0.0554298	0.12589607	-0.4402821
1559	0.05577955	0.12672636	0.44015742
743	-0.0558172	0.12711845	-0.4390963
120	0.05716807	0.13037199	0.43849965
369	-0.0553234	0.12618813	-0.4384197
1463	0.05503753	0.12583895	0.4373648
1838	0.05630026	0.12888631	0.4368211
498	-0.0551652	0.12634738	-0.4366149

© 2024 Zhang X et al. *JAMA Pediatrics*.

231	-0.0548293	0.12569905	-0.4361947
892	-0.0546776	0.12569606	-0.4349982
1609	0.0546968	0.1257668	0.43490647
1179	-0.0550384	0.1265802	-0.4348108
1840	0.05501713	0.12671074	0.43419469
1470	0.05635103	0.12983744	0.43401217
836	-0.0543907	0.1259601	-0.4318086
754	0.05453604	0.12656486	0.43089403
58	-0.0549546	0.12756299	-0.4308039
1947	0.05409176	0.12559701	0.43067711
88	-0.0555115	0.12897753	-0.4303967
666	0.05418379	0.1260131	0.42998534
1927	0.05402416	0.12565409	0.42994349
166	-0.0549842	0.12820412	-0.4288802
862	-0.054128	0.1264359	-0.4281063
705	0.05455483	0.12750854	0.42785237
715	-0.0540062	0.12623791	-0.4278127
441	-0.0542008	0.12672982	-0.427688
1159	0.0538863	0.12603662	0.42754483
672	-0.0536085	0.12562754	-0.4267253
824	0.05419663	0.12750487	0.4250554
1606	-0.0538935	0.12680714	-0.4250034
1037	-0.0533796	0.12578073	-0.4243859
1605	-0.0535207	0.12631773	-0.423699
152	-0.0536185	0.12657444	-0.4236122
37	-0.0533753	0.12611145	-0.4232391
1666	-0.0539555	0.12757923	-0.4229176
636	-0.053341	0.12655966	-0.4214694
1809	0.05334591	0.12667955	0.42110912
38	-0.0529332	0.12599921	-0.420107
1224	-0.053007	0.12624347	-0.4198792
657	0.05252905	0.12578234	0.41761863
370	0.05239553	0.12557771	0.41723588
631	0.05389948	0.12925438	0.4170031
723	0.0524388	0.1257772	0.41691816
1943	0.05232596	0.12559317	0.41663064
895	-0.0536748	0.12883301	-0.4166233
1767	0.05353673	0.12874946	0.41582101
750	-0.0523308	0.12589759	-0.4156618
830	-0.0525974	0.1265657	-0.415574
1491	-0.0526897	0.12687501	-0.4152883
189	-0.0529861	0.12766797	-0.4150308

© 2024 Zhang X et al. *JAMA Pediatrics.*

1590	0.05239549	0.12644414	0.41437656
24	-0.0524195	0.12700593	-0.4127328
1675	0.05321629	0.12924096	0.41176022
193	0.05172201	0.12573115	0.41136991
1053	0.05255006	0.12816992	0.41000309
373	-0.0514453	0.12562736	-0.4095073
557	-0.0514146	0.1258539	-0.4085263
680	-0.0518879	0.12716874	-0.4080241
1734	-0.0523643	0.12853298	-0.4073998
833	0.05143561	0.12640865	0.40689944
488	-0.0515197	0.12698184	-0.4057249
1208	0.05166252	0.1275436	0.40505771
1211	0.05166252	0.1275436	0.40505771
1140	-0.0508163	0.12571662	-0.4042133
14	0.05100548	0.12619983	0.40416438
148	0.05081605	0.12579829	0.4039487
328	0.05088682	0.12598457	0.4039131
510	-0.0513744	0.1277747	-0.4020704
799	0.05063828	0.12597141	0.40198234
1353	-0.0505153	0.12663693	-0.3988985
321	0.0501611	0.12577518	0.39881562
730	-0.0504405	0.12650419	-0.3987262
52	-0.0512326	0.12855981	-0.3985118
349	-0.050389	0.1267428	-0.397569
806	-0.0503681	0.12689971	-0.3969129
844	0.04984472	0.12564625	0.39670677
1834	0.04951912	0.12579826	0.39363917
153	-0.0495753	0.12623972	-0.3927076
1507	0.0495799	0.12625801	0.39268718
299	-0.0492733	0.12561381	-0.3922605
1435	-0.0491934	0.12561917	-0.3916074
367	-0.0491709	0.12558406	-0.3915381
45	0.04962513	0.12691013	0.39102577
426	0.04907501	0.1255839	0.39077467
1821	0.05285344	0.13539681	0.39035954
1047	0.04873171	0.12573459	0.38757599
1576	-0.0488625	0.12642306	-0.3864997
1318	0.04889551	0.12671857	0.38585909
1654	-0.0484648	0.12587234	-0.3850312
924	0.04894494	0.12725785	0.38461237
310	-0.0485232	0.12640202	-0.3838797
1297	-0.0482754	0.12577151	-0.3838339

© 2024 Zhang X et al. *JAMA Pediatrics.*

260	0.0487086	0.12703998	0.38341156
182	-0.0481919	0.12577621	-0.3831557
1690	-0.0482701	0.12628644	-0.3822269
1428	0.04872496	0.12790895	0.38093473
599	0.05078333	0.13332843	0.38088895
905	0.04935177	0.12968044	0.3805645
616	0.04788465	0.1259742	0.38011473
1273	0.04777499	0.12574158	0.37994582
1826	0.04768166	0.12561688	0.37958007
60	0.04768898	0.12565262	0.37953035
1634	-0.0480557	0.12665819	-0.3794128
1563	-0.0476365	0.12567195	-0.3790542
1553	-0.0478711	0.12650905	-0.3784008
261	0.04755149	0.12571064	0.37826144
371	0.04750247	0.12559661	0.37821464
1216	-0.0513504	0.13590809	-0.3778321
1255	-0.0513504	0.13590809	-0.3778321
27	-0.0473931	0.12575608	-0.3768652
303	-0.0471822	0.12561348	-0.3756144
991	-0.0481361	0.12863376	-0.3742106
1358	0.04709172	0.12601931	0.37368654
1367	-0.0472041	0.12639954	-0.3734519
356	0.04689329	0.12559655	0.37336447
366	-0.0466024	0.12558392	-0.371086
1172	0.04823036	0.12997085	0.37108596
268	0.0472669	0.12760055	0.37042867
1698	-0.046561	0.125981	-0.3695876
1277	0.04634507	0.12579629	0.36841367
1291	0.04634507	0.12579629	0.36841367
700	-0.0463362	0.12602003	-0.3676891
550	-0.0462373	0.12636325	-0.3659078
196	0.04724221	0.12912437	0.36586594
244	0.04591982	0.12557633	0.36567257
744	-0.0462809	0.12656694	-0.3656631
185	-0.0461166	0.12613427	-0.3656153
29	-0.0465827	0.12794865	-0.3640732
1136	0.04606894	0.12658062	0.36394941
1623	-0.0456854	0.12572002	-0.3633898
1213	-0.0463638	0.12804891	-0.362079
1416	-0.0462748	0.12795437	-0.361651
713	-0.0463754	0.12902642	-0.3594259
357	-0.0450716	0.12567094	-0.3586474

© 2024 Zhang X et al. *JAMA Pediatrics.*

400	-0.0450023	0.12565225	-0.3581494
1377	-0.0449176	0.1256941	-0.3573562
1449	-0.0450858	0.12622121	-0.3571965
1968	0.04486364	0.1255996	0.3571957
1258	-0.0449125	0.12586841	-0.356821
1627	0.04514981	0.12656679	0.35672713
1455	0.04586783	0.12899064	0.35559038
1247	-0.0451151	0.12689717	-0.355525
478	0.04465687	0.12569065	0.35529192
44	-0.0469797	0.1325662	-0.3543865
1503	-0.0454728	0.12905066	-0.3523636
162	0.04454105	0.12641494	0.35234008
165	0.04451986	0.12640241	0.35220735
1283	-0.0442806	0.1263556	-0.3504446
882	-0.0442216	0.126672	-0.3491032
1417	0.04443758	0.12768251	0.34803182
1998	0.0436809	0.12566729	0.34759161
70	-0.0434968	0.12592059	-0.3454302
627	0.04327372	0.12581713	0.34394143
710	-0.0434253	0.12641337	-0.343518
1845	0.04337634	0.12652374	0.34283164
251	-0.0430269	0.12561606	-0.3425272
1567	-0.0433351	0.12671997	-0.3419755
241	0.04402441	0.12895615	0.34139055
1314	-0.0428944	0.12583028	-0.3408912
1689	-0.0428204	0.12563468	-0.340833
1652	-0.0437159	0.12844911	-0.3403362
344	0.0428517	0.12618687	0.33958923
759	-0.0428029	0.12615462	-0.339289
1225	-0.0430264	0.1271963	-0.3382679
205	0.04251508	0.12603216	0.33733516
1591	-0.0427353	0.12672534	-0.3372274
1089	0.04615846	0.13697571	0.33698283
661	0.04232065	0.12588769	0.33617785
39	-0.0421518	0.12570312	-0.3353283
179	0.04223103	0.12624386	0.33451951
1420	0.04195567	0.12557715	0.33410272
192	0.04228771	0.12661594	0.3339841
591	-0.0420895	0.12605649	-0.3338936
320	0.04211257	0.12624343	0.33358228
330	0.04208515	0.12647117	0.33276477
916	-0.041771	0.12577088	-0.3321196

© 2024 Zhang X et al. *JAMA Pediatrics*.

1466	-0.0418243	0.12633294	-0.3310639
1909	0.04239178	0.12827015	0.33048828
757	0.04176378	0.12637829	0.3304664
1494	0.04153926	0.12570668	0.3304459
797	-0.0429124	0.13015406	-0.3297043
684	-0.0414732	0.12589171	-0.3294355
1438	0.04170766	0.12663258	0.32935966
978	-0.0417406	0.12677819	-0.3292416
1317	0.04136229	0.12574582	0.32893572
379	0.04145891	0.12632248	0.32819896
68	-0.0410155	0.12565628	-0.3264102
1381	-0.0409906	0.1257195	-0.3260477
1645	-0.0409624	0.1256628	-0.325971
1323	-0.040989	0.12578156	-0.3258748
780	-0.0414687	0.12740478	-0.3254879
232	-0.0408401	0.12558347	-0.3252029
753	-0.0409318	0.12608403	-0.3246392
124	0.04118641	0.12701802	0.32425644
412	-0.040674	0.12581053	-0.3232957
1217	0.04127248	0.12786203	0.32278918
724	-0.0405763	0.12580329	-0.3225379
55	-0.0406231	0.12601556	-0.3223656
271	0.04102899	0.12737157	0.32212049
237	0.04077401	0.12662717	0.32200049
442	-0.0404648	0.1259454	-0.3212884
289	0.0402777	0.12599088	0.31968744
890	-0.0400538	0.12562834	-0.3188274
281	0.04018093	0.12624871	0.31826803
368	-0.0398401	0.12558671	-0.3172318
848	-0.0398348	0.12569671	-0.3169121
343	0.03981066	0.12569496	0.31672437
487	0.03977519	0.12566338	0.31652174
141	-0.0397769	0.12586998	-0.316016
769	-0.0399734	0.1265113	-0.3159668
1013	-0.0402731	0.12753437	-0.3157822
1289	0.03990929	0.12660242	0.31523321
20	-0.0403843	0.12816567	-0.3150949
763	0.04007648	0.12736206	0.3146658
1469	-0.0397854	0.12652648	-0.3144432
888	-0.0394643	0.12570317	-0.3139485
1687	-0.0398734	0.12789449	-0.311768
886	-0.0398734	0.12789449	-0.311768

© 2024 Zhang X et al. *JAMA Pediatrics.*

992	-0.039539	0.12733099	-0.3105212
658	-0.039516	0.12743234	-0.3100936
1209	0.03921991	0.1266934	0.30956554
1166	0.03887312	0.1256137	0.30946564
1702	-0.0388529	0.1257181	-0.309048
760	0.03939625	0.12754973	0.30886976
1595	-0.0387872	0.12568471	-0.3086069
1531	-0.0387957	0.12585116	-0.3082665
358	0.0386976	0.12568201	0.3079009
1446	-0.0389304	0.12650537	-0.3077368
47	-0.0385582	0.12588845	-0.3062882
35	-0.038557	0.12602901	-0.3059376
71	-0.038557	0.12602901	-0.3059376
598	0.03950629	0.12920324	0.30576857
1430	-0.0384111	0.12576128	-0.3054283
81	-0.0387722	0.1269941	-0.3053069
897	-0.0383111	0.12569993	-0.3047824
1107	0.0395538	0.12993373	0.30441515
1662	0.03821486	0.12567923	0.30406666
1542	-0.037968	0.12599087	-0.3013555
1807	0.03844816	0.12765274	0.3011934
269	0.03782545	0.12575629	0.30078381
581	-0.038531	0.12843341	-0.300008
1547	-0.0377754	0.12598632	-0.299837
1642	-0.0377862	0.12616707	-0.2994937
945	0.03788666	0.12672054	0.29897805
311	0.03752216	0.12562364	0.29868706
199	0.03788631	0.12687227	0.29861769
351	0.03744399	0.12564598	0.29801181
1015	-0.0374644	0.1259678	-0.2974126
1832	0.03791605	0.1275697	0.29721825
858	-0.0380395	0.12832315	-0.2964354
318	-0.0368922	0.12562178	-0.2936766
685	-0.0368941	0.12564753	-0.2936315
708	-0.0370408	0.12643309	-0.2929679
589	0.03872987	0.13237587	0.29257495
1038	0.0370074	0.12665741	0.29218503
308	0.03698059	0.12686571	0.29149398
1062	0.03686711	0.12653118	0.29136776
462	-0.0367796	0.12627938	-0.2912559
1386	-0.0365855	0.12575509	-0.2909263
1971	0.03654194	0.125668	0.29078155

© 2024 Zhang X et al. *JAMA Pediatrics*.

1587	-0.0365838	0.1258185	-0.2907665
67	-0.0364493	0.12569227	-0.2899884
1290	0.03659531	0.12626985	0.28981829
140	0.03675531	0.12686408	0.28972196
500	0.03669162	0.12667775	0.28964535
66	-0.0363303	0.12560967	-0.2892315
1406	-0.0372871	0.12907631	-0.2888762
1271	-0.0362325	0.12607539	-0.2873872
51	-0.0361476	0.12582662	-0.2872814
1212	-0.0361262	0.12645047	-0.2856942
439	0.0362707	0.12696071	0.28568447
630	0.03614852	0.12673409	0.28523121
1442	-0.0357992	0.12618166	-0.2837113
787	-0.0354168	0.12559387	-0.2819949
643	0.03541623	0.12594919	0.28119461
659	0.03644765	0.1304459	0.27940818
375	-0.0350365	0.1255838	-0.2789886
1792	0.03549147	0.12737566	0.27863617
948	-0.0349639	0.12607285	-0.2773308
427	0.03479541	0.12559977	0.27703407
1042	-0.0354428	0.1279732	-0.2769549
869	-0.0351469	0.1269165	-0.2769293
1580	-0.0348091	0.12613548	-0.275966
1431	-0.0346357	0.12577964	-0.2753679
423	-0.0346616	0.12609311	-0.2748888
1597	0.03469104	0.12631528	0.27463849
1045	-0.0348951	0.12706893	-0.2746152
994	-0.0349341	0.12755457	-0.2738756
953	-0.0344803	0.12624226	-0.273128
1052	0.03469581	0.12710765	0.27296394
1751	0.03735218	0.13767087	0.27131504
1432	-0.034127	0.12581891	-0.2712388
350	0.03414584	0.12593056	0.27114812
138	-0.0343152	0.12684828	-0.2705216
1433	-0.0341093	0.12654015	-0.2695533
1612	0.03375317	0.12567973	0.26856495
704	0.0339806	0.12685951	0.2678601
545	-0.0337171	0.126296	-0.2669692
1398	-0.0338399	0.12711512	-0.2662142
1066	-0.0346408	0.13044638	-0.265556
955	0.03347466	0.12633681	0.26496363
127	0.03332134	0.12638286	0.26365396

© 2024 Zhang X et al. *JAMA Pediatrics.*

1863	-0.0329177	0.125802	-0.2616628
355	0.03288182	0.12579727	0.26138739
273	-0.0331481	0.12683559	-0.2613466
151	-0.0329519	0.12613418	-0.261245
1171	0.03391057	0.13085987	0.25913648
1701	-0.0386044	0.14978093	-0.2577391
1016	-0.0324381	0.12587542	-0.2576997
1423	-0.0324481	0.12592194	-0.2576845
664	0.03247761	0.12649155	0.25675716
626	-0.0332951	0.13049466	-0.2551455
480	-0.0324285	0.12720579	-0.2549292
1443	-0.0321088	0.12606573	-0.2546986
970	-0.0321099	0.12611569	-0.2546067
1629	0.03202655	0.12583201	0.25451829
1477	0.03213421	0.12638367	0.25425923
421	-0.0319185	0.12567891	-0.2539687
1026	0.03191337	0.12586798	0.25354634
1568	0.03211525	0.12675937	0.25335604
411	0.03182528	0.12569014	0.2532043
372	-0.031681	0.12564178	-0.252153
1917	-0.0320306	0.12729275	-0.2516291
887	-0.0318311	0.12660072	-0.2514292
1536	-0.0319923	0.12772554	-0.2504768
116	-0.0312385	0.12585413	-0.2482117
1232	-0.0314353	0.1266691	-0.2481689
1373	-0.0323752	0.13091698	-0.2472956
703	-0.0316499	0.1281336	-0.2470068
663	0.03130931	0.12692217	0.24668119
1365	-0.0309444	0.12578324	-0.246014
913	-0.0308946	0.12599066	-0.2452135
428	-0.0307383	0.12578057	-0.2443805
248	-0.0307332	0.12586923	-0.2441674
1100	0.03142105	0.12904028	0.24349802
690	-0.0305457	0.12609365	-0.242246
823	-0.0306845	0.1266752	-0.2422296
1588	-0.0306437	0.1278175	-0.2397455
429	0.03005928	0.12563605	0.23925679
640	-0.0312371	0.13056888	-0.2392387
966	-0.0300549	0.1257155	-0.2390707
347	-0.0299774	0.12603454	-0.2378505
901	-0.0297048	0.12558742	-0.236527
1659	0.02984791	0.12630042	0.23632473

© 2024 Zhang X et al. *JAMA Pediatrics*.

1444	-0.0296077	0.12558619	-0.2357559
1643	-0.0296367	0.1263428	-0.2345736
1636	0.02951015	0.12597964	0.23424542
1379	-0.0295561	0.12716463	-0.2324238
749	0.02925991	0.12648284	0.23133501
834	-0.029102	0.1259239	-0.2311079
2004	0.02980163	0.1291269	0.23079332
233	-0.029505	0.12791213	-0.2306663
65	0.02940488	0.12758694	0.23046934
449	-0.0291156	0.12764131	-0.2281046
200	-0.0286595	0.12577637	-0.2278605
815	-0.0285654	0.12607539	-0.2265742
1170	0.02838751	0.12569188	0.22585
649	-0.0284215	0.12701383	-0.2237667
946	-0.0284019	0.12714233	-0.2233867
1148	-0.028152	0.12609527	-0.2232601
1923	0.02871647	0.12923125	0.22220998
910	-0.0287522	0.13001297	-0.2211486
465	-0.0278934	0.12629005	-0.2208674
1712	-0.0277001	0.12581764	-0.2201606
1380	-0.0276775	0.12601928	-0.2196294
638	-0.0277888	0.12699149	-0.2188245
655	-0.0277888	0.12699149	-0.2188245
160	0.02751771	0.12632228	0.21783738
1571	0.02904524	0.13351695	0.21753969
341	0.02744743	0.12648141	0.21700761
461	-0.0279065	0.12953671	-0.2154334
1839	0.02758523	0.1281143	0.21531735
962	0.02706318	0.1265128	0.21391652
399	-0.0268506	0.12583856	-0.2133737
1452	0.02671824	0.12579854	0.21238915
1321	-0.0265208	0.12586778	-0.2107034
84	-0.0264652	0.12652821	-0.2091646
1677	0.02635796	0.12604585	0.20911406
775	0.02635417	0.12670781	0.20799169
331	0.02789603	0.13413184	0.20797474
984	-0.0263895	0.12692478	-0.2079146
884	-0.0259976	0.12581915	-0.2066267
949	0.02603651	0.12624708	0.20623454
1492	0.02588989	0.12643501	0.20476834
1614	0.02552501	0.12576436	0.20295905
628	-0.0254983	0.12599767	-0.2023712

© 2024 Zhang X et al. *JAMA Pediatrics.*

1322	-0.0254343	0.12585343	-0.2020945
1490	0.02535014	0.12653912	0.20033439
1699	-0.0255543	0.12790164	-0.1997962
952	-0.0252269	0.1262759	-0.1997758
180	-0.0249216	0.12606159	-0.197694
950	0.02490926	0.12628512	0.19724616
1135	-0.0250007	0.12729647	-0.1963971
1558	-0.024565	0.12569456	-0.1954339
1050	-0.0248169	0.1269938	-0.1954184
1504	0.02501791	0.1288052	0.19423061
40	-0.0244416	0.1262799	-0.1935511
30	-0.0243069	0.12582225	-0.1931848
386	-0.0241461	0.12607095	-0.1915275
1123	0.0241302	0.12634144	0.19099195
173	-0.0242717	0.12730547	-0.1906576
1	0.02391982	0.12601889	0.18981136
1049	0.02369015	0.12562024	0.18858547
242	0.02364356	0.12578038	0.1879749
596	-0.0239164	0.12739694	-0.1877314
1287	0.02362498	0.12622065	0.18717205
1585	-0.0234365	0.12614697	-0.1857871
1023	0.02343688	0.12714757	0.18432814
1024	0.02343688	0.12714757	0.18432814
402	-0.0230666	0.12565195	-0.1835752
660	-0.0230602	0.12599791	-0.1830209
957	0.02331877	0.12759573	0.18275514
818	0.02334669	0.12805172	0.18232236
292	0.02282507	0.12559779	0.18173146
470	-0.0229053	0.12618722	-0.181518
1400	0.02291854	0.12696619	0.18050903
1046	-0.0227081	0.12585908	-0.1804245
309	0.02264199	0.12586304	0.17989384
1471	0.02257939	0.12563749	0.17971856
891	-0.0229056	0.12820182	-0.1786685
136	0.02273775	0.12750971	0.17832171
1489	-0.0223628	0.12579918	-0.1777659
1408	-0.0223984	0.12621602	-0.177461
1719	-0.0229486	0.12945996	-0.1772644
1495	0.02210648	0.12633241	0.17498664
1783	0.02240407	0.1286558	0.17413962
326	0.02175514	0.1256018	0.17320719
1641	0.02175378	0.12616775	0.1724195

© 2024 Zhang X et al. *JAMA Pediatrics.*

563	0.0216543	0.12634816	0.17138599
1434	0.02154996	0.12647472	0.17038944
102	-0.0214848	0.12620926	-0.1702312
555	-0.0221958	0.13131837	-0.1690227
1404	-0.0214147	0.12729975	-0.1682223
699	0.02095938	0.12591	0.16646319
985	-0.0210665	0.12663657	-0.166354
778	0.02101903	0.12689487	0.16564131
995	-0.0208128	0.12620693	-0.1649104
1724	-0.0206252	0.12594273	-0.1637665
1601	0.02088774	0.12779336	0.16344933
561	0.02052077	0.12575544	0.16318001
1157	-0.0203848	0.12612546	-0.161623
624	0.02073158	0.12872401	0.1610545
54	-0.0202641	0.12586416	-0.1609999
983	0.02049912	0.12748404	0.16079755
951	-0.0203075	0.12630108	-0.1607862
654	-0.020071	0.12560651	-0.159793
1279	0.02036273	0.12769009	0.15946989
676	-0.0200754	0.12619459	-0.1590833
644	-0.019953	0.12558559	-0.1588794
1392	-0.0199348	0.1257248	-0.1585587
1505	0.02001639	0.12645056	0.15829418
1344	0.01987253	0.12644981	0.15715742
620	0.0198656	0.12642685	0.1571312
1017	-0.020377	0.12975321	-0.157044
889	-0.0197348	0.12586526	-0.1567928
1711	-0.020523	0.13138549	-0.1562041
249	0.01971729	0.12636408	0.15603558
1019	-0.0195231	0.12566192	-0.1553625
195	-0.0196245	0.12642847	-0.155222
1723	-0.0193391	0.12577957	-0.1537541
1119	-0.0193115	0.1256626	-0.1536772
1891	-0.0192816	0.12573051	-0.1533565
401	-0.0192682	0.12565072	-0.1533469
137	-0.0196187	0.12836349	-0.1528368
204	-0.0200171	0.13190234	-0.1517573
1124	-0.0190812	0.12585005	-0.1516187
772	-0.0191254	0.12621997	-0.1515242
1621	-0.0190481	0.12601527	-0.1511574
41	-0.0186436	0.12679409	-0.1470384
926	-0.0200774	0.13659885	-0.1469805

© 2024 Zhang X et al. *JAMA Pediatrics*.

766	-0.0184574	0.12626403	-0.146181
296	0.01834529	0.12563458	0.146021
1707	-0.0183302	0.12560962	-0.1459298
164	-0.0181676	0.12615099	-0.1440149
422	-0.0179292	0.12566091	-0.1426792
804	0.01792937	0.12648893	0.14174655
845	-0.0178713	0.1267624	-0.1409823
1488	0.01779547	0.12650586	0.14066915
1679	-0.0174314	0.12660525	-0.1376827
1393	-0.0170494	0.12570383	-0.1356313
597	-0.0173411	0.12804011	-0.1354352
64	-0.0170659	0.1260347	-0.1354065
1422	0.01698246	0.12604477	0.13473354
1378	0.01663016	0.12579156	0.13220406
584	0.01716822	0.1301356	0.13192563
1275	-0.0166051	0.12640001	-0.1313695
450	-0.0165368	0.12661169	-0.1306107
714	0.01632483	0.12611362	0.12944544
1607	-0.0161733	0.12569254	-0.1286737
1120	-0.0162485	0.1270531	-0.1278874
1833	0.01575179	0.12622926	0.12478717
615	0.01574617	0.12632727	0.12464582
810	-0.0158573	0.12755247	-0.1243202
2	-0.0155464	0.1257513	-0.1236282
407	-0.0154574	0.12578313	-0.1228897
678	-0.0154064	0.12567251	-0.1225919
1692	-0.0153619	0.12583109	-0.1220832
1305	0.0153856	0.12613574	0.12197653
74	0.01530867	0.12591637	0.12157804
1185	0.01530184	0.12669863	0.1207735
176	-0.0151301	0.12559417	-0.1204685
255	0.01513491	0.12589459	0.12021887
1326	0.01515233	0.12607618	0.12018394
590	-0.0150432	0.12611874	-0.1192785
1231	-0.0151505	0.12816152	-0.1182143
791	-0.0147536	0.12615186	-0.1169513
860	-0.0147075	0.12613159	-0.1166046
2008	-0.0147479	0.12659704	-0.1164951
1370	0.01466773	0.12601487	0.11639679
15	0.01492734	0.12851997	0.11614802
506	-0.0147478	0.1274701	-0.1156958
1784	0.01417909	0.12558909	0.11290067

© 2024 Zhang X et al. *JAMA Pediatrics*.

809	0.01375618	0.12565791	0.10947327
83	-0.0136633	0.12604294	-0.1084017
1653	-0.0136171	0.1257795	-0.1082617
1487	0.01368887	0.12646575	0.1082417
1596	-0.013609	0.12589882	-0.1080945
497	0.01341985	0.12577214	0.10669967
1847	-0.0136586	0.12871468	-0.106115
1441	0.01332652	0.12559662	0.10610575
1385	-0.0135492	0.12828073	-0.1056217
1356	0.01316067	0.12563172	0.10475598
272	-0.0132638	0.12692759	-0.1044987
1267	0.01339223	0.12879565	0.10398048
1025	-0.0131552	0.12688348	-0.1036797
194	-0.0129389	0.12646906	-0.102309
1722	0.0129935	0.12764247	0.10179605
981	-0.0128513	0.12627245	-0.1017744
1448	0.0127283	0.12601178	0.10100884
1824	0.01274391	0.12637018	0.10084586
274	0.01249209	0.12600716	0.0991379
847	0.01215332	0.12622402	0.09628372
254	0.01207173	0.12601616	0.09579507
1067	0.01192492	0.12608802	0.09457613
184	0.0118765	0.12637274	0.0939799
876	0.01183665	0.12598663	0.09395161
1586	-0.0117004	0.12798104	-0.091423
458	-0.0113914	0.12572124	-0.0906082
1061	-0.0113365	0.12651068	-0.0896088
398	0.01122183	0.12618239	0.08893337
1742	0.01118095	0.12572428	0.0889323
1795	-0.0110748	0.12751813	-0.0868485
1345	-0.0110503	0.12814839	-0.0862303
396	0.01050745	0.12559828	0.08365922
908	-0.0104915	0.1282447	-0.0818084
960	-0.0104915	0.1282447	-0.0818084
635	0.01031361	0.1264558	0.08155904
144	0.01035391	0.12760896	0.0811378
324	0.01033621	0.12756446	0.08102738
376	-0.0100866	0.12649702	-0.0797381
1057	0.00990477	0.1257354	0.07877469
33	0.00975331	0.12595032	0.07743779
633	0.0097594	0.12611371	0.07738572
919	-0.0096237	0.12604226	-0.0763531

© 2024 Zhang X et al. *JAMA Pediatrics.*

1147	0.00972376	0.1278548	0.07605311
1278	0.00947908	0.12642679	0.07497681
1608	0.00947638	0.1264597	0.07493596
859	-0.0093964	0.12600831	-0.0745698
1268	-0.0093639	0.12669401	-0.0739097
1152	0.00932988	0.1264853	0.07376258
1164	0.00922687	0.12594612	0.07326042
190	0.00915929	0.12595508	0.07271871
408	-0.0091112	0.12563367	-0.072522
57	0.00894231	0.12602687	0.07095557
1307	0.00892345	0.12629083	0.07065793
914	-0.0088792	0.1262731	-0.0703171
1732	0.00852976	0.12655922	0.06739736
111	0.00842134	0.12588098	0.06689924
578	0.00822634	0.12608205	0.06524591
1462	0.00820913	0.12625066	0.06502249
150	-0.0081653	0.12654227	-0.0645262
501	0.00809017	0.12558936	0.06441765
1009	-0.0080181	0.12575827	-0.0637582
1056	-0.007965	0.12690802	-0.0627618
1303	-0.0079155	0.12667447	-0.0624866
377	-0.0076867	0.1259569	-0.0610263
1685	-0.0074954	0.12575545	-0.0596028
459	-0.0074757	0.12590205	-0.059377
1474	0.00717242	0.12601128	0.05691883
918	0.00708316	0.12575689	0.05632425
1864	0.0069909	0.12572158	0.05560621
322	-0.0069738	0.1255814	-0.0555318
143	-0.0069999	0.12625852	-0.0554413
1467	0.00689129	0.12752199	0.05404002
484	0.00681232	0.12622551	0.05396947
2005	0.00671965	0.12567155	0.05346991
1460	-0.0069253	0.13042458	-0.0530981
425	-0.0065779	0.12628393	-0.0520882
1626	0.00650041	0.12585877	0.05164845
841	0.00645478	0.12575725	0.0513273
1282	0.00636142	0.12584446	0.05054986
936	0.00628941	0.12590646	0.04995305
1676	0.00630687	0.12629041	0.04993943
605	0.00630445	0.12656707	0.04981116
169	-0.0062506	0.12728871	-0.0491055
1360	-0.0059499	0.12567009	-0.047345

© 2024 Zhang X et al. *JAMA Pediatrics.*

1293	-0.0059255	0.12563816	-0.0471635
1718	-0.0059238	0.12576084	-0.0471033
187	0.00596156	0.12791909	0.04660411
517	-0.0058618	0.126226	-0.0464386
692	0.00583465	0.12576697	0.04639254
1099	-0.005785	0.12863787	-0.0449713
197	-0.0056633	0.12663398	-0.0447216
319	-0.0056132	0.12562752	-0.0446813
208	0.00558143	0.12754892	0.04375917
1808	-0.0060103	0.13767281	-0.0436565
307	0.00535828	0.12558551	0.04266639
1425	-0.005343	0.12559434	-0.0425417
21	-0.0053264	0.12594271	-0.0422924
1424	-0.0052426	0.12559435	-0.0417421
476	0.00515497	0.12745969	0.04044393
1594	-0.0049753	0.12579621	-0.0395505
1484	-0.0048191	0.1258849	-0.0382814
36	0.00475651	0.12568837	0.03784363
86	-0.0049488	0.13202586	-0.0374839
1183	0.00484013	0.13001427	0.03722766
554	0.00467659	0.12590554	0.03714363
317	0.00459876	0.12576831	0.0365653
1439	0.00457493	0.12590533	0.03633625
614	0.00445478	0.1255886	0.03547124
382	-0.0044467	0.126035	-0.0352815
849	0.00443148	0.12576041	0.03523744
245	0.00445859	0.12925983	0.03449321
1030	-0.0043005	0.12582395	-0.0341786
433	0.00409675	0.12606912	0.03249605
203	-0.0040494	0.12659326	-0.0319872
1054	0.00396311	0.12632779	0.03137163
1975	0.00442735	0.14394094	0.03075811
632	-0.0038959	0.12682197	-0.0307195
971	-0.0039	0.12777474	-0.0305226
434	0.00381347	0.12638197	0.03017417
937	-0.0035785	0.12627418	-0.0283394
1604	0.00361961	0.12850313	0.02816746
1481	-0.0035278	0.12560873	-0.0280858
650	-0.0034768	0.12837195	-0.0270835
977	0.00336753	0.12631242	0.02666036
826	-0.0032675	0.12640662	-0.025849
656	-0.0032697	0.12978919	-0.0251927

© 2024 Zhang X et al. *JAMA Pediatrics.*

954	-0.0031327	0.12725427	-0.0246176
1497	-0.0030498	0.12619915	-0.0241662
1658	0.00266411	0.12590869	0.0211591
662	0.00265637	0.12578627	0.02111815
7	0.00260302	0.12564461	0.02071731
404	-0.002571	0.12569415	-0.0204544
456	-0.0025132	0.12591828	-0.0199592
1551	0.00244956	0.12685536	0.01930987
673	-0.0023924	0.12722931	-0.0188041
393	0.00224421	0.12564061	0.01786216
893	0.00200272	0.1285735	0.01557649
295	-0.0019222	0.12559593	-0.0153048
709	0.00186399	0.12596706	0.01479741
301	-0.001545	0.12565798	-0.0122954
575	-0.0014378	0.12673821	-0.011345
874	-0.0014256	0.12720774	-0.0112072
1995	0.00131098	0.1259369	0.01040978
1040	0.0013119	0.12631514	0.01038595
161	-0.0013134	0.12841168	-0.0102283
491	0.00123324	0.12576276	0.00980608
239	0.00120465	0.12745268	0.00945171
1237	0.00108666	0.12701325	0.00855552
883	-0.0010721	0.1256967	-0.0085291
298	0.00077717	0.12574735	0.0061804
808	-0.0007582	0.12816191	-0.0059163
1276	-0.0006313	0.13094747	-0.0048214
1694	-0.0005698	0.12598075	-0.0045229
1844	-0.0004849	0.12564633	-0.0038589
1167	-0.0003546	0.12577448	-0.0028191

	estimate	zval	pval	ci.lb	ci.ub	Q	Qp	tau2	I2	H2
1	0.08494859	104.850706	0	0.08186839	0.08807986	2238147.1	0	0.01576211	99.9264828	1360.22508
2	0.08495976	104.85684	0	0.08187935	0.08809124	2238132.97	0	0.01576235	99.9264777	1360.13178
3	0.08497733	104.878343	0	0.08189694	0.08810879	2238113.68	0	0.01575926	99.9264715	1360.01668
4	0.08497834	104.880998	0	0.08189799	0.08810976	2238132.3	0	0.0157588	99.9264705	1359.99765
5	0.08499744	104.918544	0	0.08191752	0.0881284	2237912.97	0	0.01575078	99.9264299	1359.24749
6	0.08493848	104.849744	0	0.08185861	0.08806942	2238135.46	0	0.01576049	99.9264767	1360.11394
7	0.08495461	104.853158	0	0.08187428	0.08808602	2238144.85	0	0.01576247	99.9264613	1359.82834
8	0.08499074	104.90355	0	0.08191062	0.08812191	2238077.63	0	0.01575415	99.926448	1359.58187
9	0.08493041	104.851771	0	0.08185088	0.08806101	2238111.09	0	0.01575829	99.9264662	1359.9186
10	0.08497894	104.881424	0	0.08189859	0.08811037	2238124.13	0	0.0157587	99.9264696	1359.98245

© 2024 Zhang X et al. *JAMA Pediatrics.*

11	0.08492578	104.854994	0	0.0818465	0.08805611	2238126.1	0	0.01575653	99.9264596	1359.79703
12	0.08492295	104.85639	0	0.08184382	0.08805314	2238104.01	0	0.01575548	99.926454	1359.69318
13	0.08491497	104.865265	0	0.08183637	0.08804461	2238124.55	0	0.01575148	99.9264366	1359.37091
14	0.08494094	104.849557	0	0.08186098	0.08807198	2238138.31	0	0.01576101	99.9264788	1360.15171
15	0.08495128	104.853814	0	0.08187109	0.08808256	2238148.7	0	0.01576199	99.9264856	1360.277
16	0.0849306	104.852319	0	0.08185108	0.08806119	2238131.45	0	0.01575826	99.9264675	1359.94308
17	0.08491519	104.865345	0	0.08183659	0.08804484	2238127.38	0	0.01575155	99.9264369	1359.37802
18	0.08493578	104.852193	0	0.08185608	0.08806655	2238144.13	0	0.01575953	99.9264741	1360.06547
19	0.08497729	104.877792	0	0.08189688	0.08810876	2238053.66	0	0.01575935	99.9264685	1359.96157
20	0.08496634	104.865105	0	0.08188595	0.08809781	2238144.79	0	0.0157614	99.9264827	1360.22457
21	0.08495686	104.854839	0	0.08187649	0.0880883	2238145.98	0	0.01576243	99.9264834	1360.23732
22	0.0849747	104.873652	0	0.08189427	0.08810621	2237742.17	0	0.01576008	99.9264488	1359.59621
23	0.08497954	104.881207	0	0.08189915	0.08811099	2237521.12	0	0.0157587	99.9264412	1359.45575
24	0.08496987	104.868287	0	0.08188945	0.08810137	2238137.79	0	0.01576096	99.9264802	1360.17701
25	0.08497319	104.873466	0	0.0818928	0.08810465	2238139.31	0	0.01576014	99.9264768	1360.11515
26	0.08497436	104.873321	0	0.08189392	0.08810586	2238056.14	0	0.01576014	99.9264709	1360.00501
27	0.08496875	104.865862	0	0.0818883	0.08810028	2238074.73	0	0.01576133	99.9264734	1360.05275
28	0.08498121	104.884908	0	0.08190087	0.08811261	2238108.51	0	0.01575802	99.926466	1359.91492
29	0.08496807	104.866882	0	0.08188767	0.08809954	2238143.32	0	0.01576116	99.9264816	1360.20302
30	0.08496223	104.859029	0	0.0818818	0.08809373	2238128.9	0	0.01576217	99.9264798	1360.17128
31	0.08497974	104.881572	0	0.08189936	0.08811119	2237704.17	0	0.01575863	99.9264498	1359.61575
32	0.08498849	104.898584	0	0.0819083	0.08811973	2238068.55	0	0.01575521	99.9264524	1359.66437
33	0.08495259	104.852318	0	0.08187231	0.08808396	2238148.72	0	0.01576238	99.9264833	1360.23479
34	0.08499304	104.908633	0	0.08191298	0.08812414	2238067.1	0	0.01575304	99.9264427	1359.48512
35	0.08496621	104.863193	0	0.08188577	0.08809773	2238127.56	0	0.01576169	99.9264809	1360.19111
36	0.084954	104.852844	0	0.08187368	0.0880854	2238147.51	0	0.01576246	99.9264722	1360.03056
37	0.08497035	104.868135	0	0.08188991	0.08810187	2238118.56	0	0.01576099	99.9264783	1360.1418
38	0.08497026	104.867917	0	0.08188981	0.08810178	2238111.08	0	0.01576102	99.9264776	1360.12886
39	0.08496729	104.864106	0	0.08188683	0.08809882	2238061.31	0	0.01576158	99.9264705	1359.9978
40	0.08496221	104.859405	0	0.0818818	0.08809371	2238141.82	0	0.01576211	99.9264842	1360.25256
41	0.08496054	104.858369	0	0.08188015	0.08809201	2238145.96	0	0.01576217	99.9264856	1360.27709
42	0.08491422	104.864015	0	0.08183561	0.08804387	2238017	0	0.0157514	99.9264336	1359.31568
43	0.08497926	104.88636	0	0.08189903	0.08811054	2238142.76	0	0.01575792	99.9264669	1359.932
44	0.08496728	104.869535	0	0.08188698	0.08809865	2238146.9	0	0.01576074	99.9264801	1360.17517
45	0.08494149	104.850162	0	0.08186153	0.08807252	2238144.19	0	0.01576102	99.9264803	1360.17992
46	0.08499022	104.904181	0	0.08191013	0.08812135	2238124.48	0	0.01575412	99.9264489	1359.59946
47	0.08496624	104.8631	0	0.08188579	0.08809776	2238117.97	0	0.01576171	99.9264793	1360.16025
48	0.08497121	104.869367	0	0.08189078	0.08810273	2238122.51	0	0.0157608	99.926478	1360.13793
49	0.08498586	104.896545	0	0.0819057	0.08811707	2238135.73	0	0.01575581	99.926457	1359.74857
50	0.08497559	104.875644	0	0.08189518	0.08810706	2238117.33	0	0.01575974	99.9264737	1360.05725
51	0.08496557	104.86233	0	0.08188512	0.08809709	2238113.96	0	0.01576181	99.9264783	1360.14294
52	0.0849692	104.868724	0	0.08188881	0.08810066	2238143.71	0	0.01576089	99.9264804	1360.18242

© 2024 Zhang X et al. *JAMA Pediatrics.*

53	0.08498733	104.896394	0	0.08190712	0.0881186	2238087.9	0	0.01575568	99.926455	1359.71262
54	0.08496108	104.858045	0	0.08188067	0.08809257	2238135.51	0	0.01576226	99.9264813	1360.19795
55	0.0849668	104.863822	0	0.08188635	0.08809832	2238125.06	0	0.01576161	99.9264804	1360.18192
56	0.08499768	104.918968	0	0.08191777	0.08812864	2237679.98	0	0.01575067	99.9264255	1359.16611
57	0.08495282	104.8525	0	0.08187253	0.08808419	2238148.71	0	0.01576238	99.9264841	1360.24956
58	0.08497044	104.869459	0	0.08189003	0.08810192	2238140.23	0	0.01576078	99.9264797	1360.16778
59	0.08497736	104.880368	0	0.08189702	0.08810875	2238138.6	0	0.01575894	99.9264714	1360.01538
60	0.08494177	104.849115	0	0.08186176	0.08807284	2238077.28	0	0.01576123	99.9264601	1359.80548
61	0.08499869	104.922036	0	0.08191882	0.08812958	2238022.28	0	0.01575001	99.9264283	1359.21761
62	0.08498899	104.90145	0	0.08190887	0.08812017	2238124.91	0	0.0157547	99.9264516	1359.64884
63	0.08497615	104.877213	0	0.08189576	0.0881076	2238131.86	0	0.01575948	99.9264735	1360.053
64	0.08496016	104.857414	0	0.08187976	0.08809164	2238142.09	0	0.01576229	99.9264838	1360.2435
65	0.08494723	104.851599	0	0.08186711	0.08807843	2238148.03	0	0.01576177	99.9264843	1360.253
66	0.08496566	104.862237	0	0.0818852	0.08809719	2237873.13	0	0.01576182	99.9264306	1359.26047
67	0.08496568	104.862329	0	0.08188522	0.0880972	2238072.06	0	0.01576181	99.9264701	1359.99157
68	0.08496697	104.863714	0	0.08188652	0.0880985	2238015.2	0	0.01576163	99.9264625	1359.85095
69	0.08498654	104.902101	0	0.08190652	0.08811761	2238141.65	0	0.01575488	99.9264528	1359.67151
70	0.08496762	104.864675	0	0.08188717	0.08809915	2238115.05	0	0.0157615	99.9264788	1360.15199
71	0.08496621	104.863193	0	0.08188577	0.08809773	2238127.56	0	0.01576169	99.9264809	1360.19111
72	0.0849755	104.874844	0	0.08189507	0.088107	2237823.15	0	0.01575987	99.926455	1359.71203
73	0.0849723	104.871073	0	0.08189187	0.0881038	2238128.5	0	0.01576053	99.9264776	1360.12927
74	0.08495102	104.851551	0	0.08187076	0.08808235	2238148.46	0	0.01576231	99.9264825	1360.21973
75	0.08497142	104.869343	0	0.08189097	0.08810294	2238095.62	0	0.0157608	99.9264755	1360.09111
76	0.08497158	104.869558	0	0.08189114	0.0881031	2238094.44	0	0.01576077	99.9264753	1360.0877
77	0.08499455	104.911943	0	0.08191454	0.0881256	2238040.5	0	0.01575229	99.9264389	1359.41337
78	0.08499967	104.924009	0	0.08191983	0.08813054	2237695.54	0	0.01574951	99.926421	1359.08313
79	0.08497095	104.869444	0	0.08189052	0.08810245	2238133.93	0	0.01576079	99.9264791	1360.15702
80	0.08490315	104.878759	0	0.08182535	0.08803197	2237991.87	0	0.01574478	99.9264036	1358.76142
81	0.0849661	104.863886	0	0.08188568	0.0880976	2238141.95	0	0.01576158	99.926483	1360.23002
82	0.08492168	104.858171	0	0.08184264	0.08805177	2238123.92	0	0.01575481	99.9264518	1359.65235
83	0.0849592	104.856648	0	0.08187881	0.08809067	2238143.72	0	0.01576234	99.9264841	1360.24904
84	0.08496275	104.860111	0	0.08188234	0.08809424	2238143.02	0	0.01576203	99.9264845	1360.25759
85	0.08491282	104.8657	0	0.08183431	0.08804238	2238036.27	0	0.01575064	99.9264307	1359.26183
86	0.0849566	104.859484	0	0.08187638	0.08808789	2238148.58	0	0.01576169	99.9264845	1360.257
87	0.08489836	104.887573	0	0.081821	0.08802675	2238073.7	0	0.01574131	99.9263888	1358.4885
88	0.08497025	104.870366	0	0.08188988	0.0881017	2238143.7	0	0.01576064	99.9264794	1360.16232
89	0.08500609	104.941543	0	0.08192653	0.08813666	2237743.3	0	0.01574541	99.926404	1358.76876
90	0.0849768	104.877224	0	0.08189639	0.08810827	2238095.31	0	0.01575946	99.9264712	1360.01208
91	0.08498563	104.892224	0	0.08190535	0.08811696	2237586.28	0	0.01575651	99.9264429	1359.48832
92	0.08492976	104.852081	0	0.08185026	0.08806032	2238110.32	0	0.01575808	99.9264653	1359.90168
93	0.08498914	104.900081	0	0.08190897	0.08812036	2238078.74	0	0.0157549	99.9264513	1359.64376
94	0.08499529	104.913322	0	0.08191529	0.08812633	2237928.32	0	0.01575196	99.9264353	1359.34691

© 2024 Zhang X et al. *JAMA Pediatrics.*

95	0.08500617	104.942236	0	0.08192664	0.08813673	2237979.4	0	0.01574528	99.9264062	1358.81001
96	0.08499211	104.905715	0	0.081912	0.08812326	2237179.78	0	0.01575363	99.9264259	1359.17352
97	0.08500471	104.938209	0	0.0819251	0.08813533	2238011.12	0	0.01574624	99.926411	1358.89868
98	0.08499517	104.913739	0	0.08191519	0.08812619	2238072.5	0	0.01575191	99.9264378	1359.39302
99	0.08499747	104.91911	0	0.08191756	0.08812841	2238045.17	0	0.01575069	99.9264317	1359.28117
100	0.08502439	105.001579	0	0.08194594	0.0881538	2236589.37	0	0.01573057	99.9263291	1357.3888
101	0.08500173	104.929467	0	0.08192198	0.08813251	2237768.66	0	0.01574825	99.9264167	1359.0042
102	0.08496139	104.858608	0	0.08188098	0.08809288	2238142.28	0	0.01576219	99.9264843	1360.25396
103	0.08500412	104.935914	0	0.08192447	0.0881348	2237608.87	0	0.01574673	99.926408	1358.8442
104	0.08499496	104.91246	0	0.08191495	0.08812602	2237848.25	0	0.01575215	99.9264345	1359.33348
105	0.08498892	104.899617	0	0.08190874	0.08812015	2238080.2	0	0.015755	99.9264518	1359.65263
106	0.08499218	104.908563	0	0.08191215	0.08812325	2238122.66	0	0.01575317	99.9264445	1359.51794
107	0.08498897	104.89972	0	0.08190879	0.08812019	2238079.88	0	0.01575498	99.9264517	1359.65065
108	0.08501625	104.973056	0	0.08193726	0.08814623	2237587.57	0	0.01573774	99.9263682	1358.10973
109	0.08499399	104.91109	0	0.08191397	0.08812505	2238083.73	0	0.01575251	99.9264407	1359.44737
110	0.085014	104.967465	0	0.08193492	0.08814407	2238067.26	0	0.01573928	99.9263795	1358.31648
111	0.08495297	104.85245	0	0.08187267	0.08808434	2238148.67	0	0.0157624	99.9264823	1360.21675
112	0.08497722	104.877702	0	0.08189681	0.08810869	2238061.57	0	0.01575936	99.926469	1359.97076
113	0.08499423	104.912065	0	0.08191423	0.08812527	2238099.85	0	0.01575232	99.9264401	1359.43723
114	0.08500634	104.944107	0	0.08192685	0.08813684	2238087.53	0	0.01574495	99.9264059	1358.80538
115	0.08492721	104.853153	0	0.08184783	0.08805765	2238070.53	0	0.01575722	99.9264596	1359.7959
116	0.08496418	104.860924	0	0.08188374	0.0880957	2238123.6	0	0.01576197	99.9264798	1360.1698
117	0.08497499	104.875211	0	0.08189459	0.08810646	2238129.98	0	0.01575983	99.9264748	1360.07861
118	0.08498292	104.887861	0	0.08190261	0.08811429	2238098.48	0	0.01575743	99.9264631	1359.86072
119	0.08497857	104.879915	0	0.08189818	0.08811003	2238064.78	0	0.01575895	99.9264678	1359.94782
120	0.0849402	104.852908	0	0.08186036	0.08807111	2238146.96	0	0.01576035	99.9264781	1360.13942
121	0.08493103	104.851247	0	0.08185146	0.08806166	2238078.37	0	0.01575853	99.9264646	1359.88872
122	0.08493317	104.850758	0	0.08185352	0.0880639	2238120.53	0	0.01575914	99.9264702	1359.99259
123	0.08493328	104.850804	0	0.08185362	0.088064	2238124.25	0	0.01575916	99.9264706	1360.00033
124	0.08494387	104.850448	0	0.08186383	0.08807498	2238146.15	0	0.0157614	99.9264822	1360.215
125	0.0849749	104.874816	0	0.08189449	0.08810637	2238125.24	0	0.01575989	99.9264748	1360.07788
126	0.08499723	104.917831	0	0.0819173	0.08812821	2237629.55	0	0.01575093	99.9264256	1359.16927
127	0.08494597	104.850291	0	0.08186586	0.08807717	2238146.16	0	0.01576178	99.926483	1360.22967
128	0.08499706	104.918126	0	0.08191714	0.08812802	2238048.96	0	0.01575091	99.9264328	1359.3012
129	0.08500126	104.928461	0	0.08192149	0.08813205	2237947.61	0	0.01574851	99.9264204	1359.07197
130	0.08492663	104.853891	0	0.0818473	0.08805703	2238111.27	0	0.01575695	99.9264607	1359.81644
131	0.08493951	104.849146	0	0.08185958	0.0880705	2238072.24	0	0.01576079	99.9264642	1359.88095
132	0.08491764	104.860847	0	0.08183882	0.08804751	2238084.64	0	0.01575309	99.9264428	1359.4856
133	0.08491906	104.860778	0	0.08184019	0.08804898	2238125.15	0	0.01575357	99.9264462	1359.54856
134	0.08492922	104.856328	0	0.08184987	0.08805964	2238143.3	0	0.01575728	99.9264639	1359.8762
135	0.08493764	104.850176	0	0.08185781	0.08806854	2238139.04	0	0.01576024	99.9264765	1360.10891
136	0.08494906	104.852111	0	0.08186889	0.08808031	2238148.42	0	0.01576196	99.9264851	1360.26859

© 2024 Zhang X et al. *JAMA Pediatrics*.

137	0.08496067	104.859765	0	0.08188032	0.0880921	2238147.45	0	0.01576196	99.9264854	1360.2741
138	0.08496489	104.862485	0	0.08188447	0.08809639	2238142.47	0	0.01576176	99.9264837	1360.24263
139	0.08498378	104.889486	0	0.08190349	0.08811513	2238097.84	0	0.01575711	99.9264616	1359.83417
140	0.08494508	104.850512	0	0.081865	0.08807624	2238146.61	0	0.0157616	99.926483	1360.22917
141	0.08496658	104.863464	0	0.08188613	0.08809811	2238114.41	0	0.01576166	99.9264787	1360.14976
142	0.08497701	104.877276	0	0.0818966	0.08810849	2238019.85	0	0.01575944	99.9264669	1359.9312
143	0.08495732	104.855427	0	0.08187696	0.08808876	2238146.94	0	0.01576238	99.9264854	1360.27427
144	0.08495249	104.853648	0	0.08187225	0.08808381	2238148.73	0	0.01576216	99.9264861	1360.28697
145	0.08499045	104.90402	0	0.08191035	0.0881216	2238116.56	0	0.01575412	99.9264487	1359.59567
146	0.08497083	104.870451	0	0.08189044	0.0881023	2238141.81	0	0.01576063	99.9264791	1360.15824
147	0.08500542	104.939843	0	0.08192584	0.08813602	2237900.21	0	0.01574582	99.9264077	1358.83827
148	0.08494091	104.849217	0	0.08186094	0.08807195	2238119.81	0	0.01576105	99.926474	1360.06327
149	0.08497663	104.876534	0	0.08189621	0.08810812	2237545.89	0	0.01575957	99.9264391	1359.4176
150	0.08495763	104.85589	0	0.08187728	0.08808907	2238147.29	0	0.01576234	99.926486	1360.28439
151	0.08496462	104.861607	0	0.08188418	0.08809613	2238135.25	0	0.01576188	99.9264825	1360.22098
152	0.08497031	104.868471	0	0.08188987	0.08810181	2238132.44	0	0.01576094	99.9264795	1360.165
153	0.08496926	104.866885	0	0.08188881	0.08810078	2238127.17	0	0.01576118	99.9264798	1360.16949
154	0.08493256	104.850961	0	0.08185293	0.08806325	2238118.73	0	0.01575896	99.9264693	1359.97704
155	0.08497457	104.875001	0	0.08189418	0.08810603	2238135.19	0	0.01575987	99.9264754	1360.08834
156	0.08491343	104.865662	0	0.0818349	0.08804301	2238096.72	0	0.01575086	99.9264331	1359.30656
157	0.08498672	104.894723	0	0.08190648	0.08811802	2238030.75	0	0.01575601	99.9264548	1359.70839
158	0.08493425	104.850722	0	0.08185456	0.08806501	2238131.91	0	0.0157594	99.9264723	1360.03188
159	0.08493757	104.85122	0	0.08185777	0.08806843	2238143.9	0	0.01576007	99.9264765	1360.10863
160	0.0849476	104.850657	0	0.08186744	0.08807884	2238147.18	0	0.01576197	99.9264837	1360.2432
161	0.0849557	104.856114	0	0.08187541	0.08808706	2238148.53	0	0.01576213	99.9264862	1360.28828
162	0.08494281	104.849829	0	0.0818628	0.08807391	2238143.25	0	0.01576131	99.9264809	1360.19119
163	0.08497759	104.88002	0	0.08189724	0.088109	2238134.96	0	0.01575899	99.9264714	1360.01564
164	0.08496046	104.857763	0	0.08188006	0.08809194	2238143.02	0	0.01576226	99.9264844	1360.25497
165	0.08494282	104.849819	0	0.0818628	0.08807391	2238143.18	0	0.01576132	99.9264809	1360.1908
166	0.08497029	104.869797	0	0.0818899	0.08810176	2238142.31	0	0.01576073	99.9264796	1360.16716
167	0.08493496	104.850683	0	0.08185524	0.08806575	2238135.4	0	0.01575957	99.9264734	1360.05134
168	0.08497106	104.871051	0	0.08189067	0.08810251	2238142.51	0	0.01576054	99.9264788	1360.15178
169	0.08495707	104.856116	0	0.08187674	0.08808848	2238148.08	0	0.01576225	99.9264864	1360.2918
170	0.08497207	104.870207	0	0.08189162	0.08810359	2238091.52	0	0.01576066	99.9264748	1360.0785
171	0.08493398	104.850661	0	0.0818543	0.08806474	2238128.11	0	0.01575935	99.9264717	1360.02084
172	0.08493122	104.852155	0	0.08185167	0.08806183	2238133.5	0	0.01575844	99.9264684	1359.96044
173	0.08496205	104.860113	0	0.08188166	0.08809352	2238145.96	0	0.01576199	99.9264852	1360.26956
174	0.08497636	104.876792	0	0.08189595	0.08810783	2238114.39	0	0.01575954	99.9264727	1360.03865
175	0.0849743	104.877152	0	0.08189398	0.08810569	2238143.45	0	0.01575955	99.9264744	1360.07083
176	0.08495965	104.85662	0	0.08187924	0.08809114	2237961.25	0	0.01576238	99.9263637	1358.02516
177	0.0849174	104.860775	0	0.08183859	0.08804727	2238044.92	0	0.01575302	99.9264413	1359.45853
178	0.08492391	104.854902	0	0.0818447	0.08805418	2234957.6	0	0.015756	99.9263173	1357.17079

© 2024 Zhang X et al. *JAMA Pediatrics*.

179	0.08494343	104.849749	0	0.08186339	0.08807455	2238142.74	0	0.01576144	99.926481	1360.19194
180	0.08496237	104.859366	0	0.08188195	0.08809387	2238138.4	0	0.01576213	99.9264832	1360.23308
181	0.08497851	104.880139	0	0.08189813	0.08810995	2238105.04	0	0.01575892	99.9264697	1359.98283
182	0.08496897	104.866144	0	0.08188852	0.0881005	2238080.38	0	0.01576129	99.9264742	1360.06725
183	0.08491939	104.859299	0	0.08184046	0.08804937	2238094.68	0	0.01575391	99.9264467	1359.55868
184	0.08495201	104.85239	0	0.08187175	0.08808335	2238148.72	0	0.0157623	99.9264854	1360.27412
185	0.08496831	104.865662	0	0.08188787	0.08809983	2238125.93	0	0.01576136	99.9264801	1360.17567
186	0.08499337	104.908507	0	0.08191329	0.08812448	2235024.8	0	0.01575301	99.9263798	1358.32231
187	0.08495371	104.854538	0	0.08187345	0.08808505	2238148.69	0	0.01576216	99.9264862	1360.28915
188	0.08497832	104.880354	0	0.08189795	0.08810975	2238124.28	0	0.0157589	99.9264705	1359.99864
189	0.08496988	104.868836	0	0.08188947	0.08810136	2238141.13	0	0.01576088	99.9264801	1360.17677
190	0.08495276	104.852407	0	0.08187247	0.08808413	2238148.71	0	0.01576239	99.9264834	1360.2364
191	0.08490207	104.881455	0	0.0818244	0.08803078	2238084.39	0	0.0157439	99.9264009	1358.71218
192	0.08494349	104.850066	0	0.08186345	0.0880746	2238144.88	0	0.0157614	99.9264817	1360.20573
193	0.08494063	104.849159	0	0.08186067	0.08807167	2238105.54	0	0.01576101	99.9264705	1359.99795
194	0.08495897	104.856828	0	0.0818786	0.08809043	2238146.28	0	0.0157623	99.9264856	1360.2784
195	0.08496085	104.858322	0	0.08188045	0.08809233	2238144.49	0	0.0157622	99.9264851	1360.26804
196	0.0849426	104.852011	0	0.08186265	0.08807362	2238147.24	0	0.01576094	99.9264808	1360.18799
197	0.08495693	104.855473	0	0.08187659	0.08808836	2238147.75	0	0.01576234	99.9264861	1360.28759
198	0.08498052	104.884664	0	0.0819002	0.0881119	2238129.88	0	0.0157581	99.9264672	1359.93806
199	0.08494477	104.850463	0	0.0818647	0.08807591	2238146.44	0	0.01576155	99.9264828	1360.22546
200	0.08496346	104.860152	0	0.08188303	0.08809498	2238117.85	0	0.01576206	99.9264777	1360.13127
201	0.08493823	104.850618	0	0.08185839	0.08806914	2238142.83	0	0.0157603	99.9264773	1360.12377
202	0.0849109	104.869461	0	0.08183256	0.08804027	2238112.85	0	0.01574934	99.9264264	1359.18392
203	0.08495648	104.855134	0	0.08187614	0.0880879	2238147.9	0	0.01576235	99.9264861	1360.28737
204	0.08496048	104.862332	0	0.08188021	0.08809182	2238148.16	0	0.01576155	99.9264838	1360.2452
205	0.08494332	104.849556	0	0.08186327	0.08807444	2238139.8	0	0.01576144	99.9264798	1360.17032
206	0.08497965	104.882858	0	0.08189931	0.08811105	2238127.29	0	0.01575844	99.9264686	1359.96368
207	0.08491353	104.864968	0	0.08183497	0.08804313	2238051.46	0	0.01575101	99.9264327	1359.29876
208	0.0849538	104.85429	0	0.08187353	0.08808515	2238148.68	0	0.01576221	99.9264863	1360.29079
209	0.08489106	104.902979	0	0.0818144	0.08801873	2238099.87	0	0.01573545	99.9263618	1357.99174
210	0.08491696	104.861176	0	0.08183818	0.0880468	2238035.67	0	0.0157528	99.9264401	1359.43667
211	0.08498646	104.894731	0	0.08190622	0.08811774	2238096.17	0	0.01575603	99.9264569	1359.74595
212	0.08493231	104.850756	0	0.08185269	0.08806301	2238084.4	0	0.01575893	99.9264662	1359.91944
213	0.08497126	104.869808	0	0.08189084	0.08810276	2238132.65	0	0.01576073	99.9264787	1360.15068
214	0.08497626	104.876461	0	0.08189585	0.08810773	2238103.74	0	0.01575959	99.9264723	1360.03099
215	0.08499967	104.923978	0	0.08191983	0.08813054	2237631.49	0	0.01574952	99.92642	1359.06522
216	0.08493732	104.849498	0	0.08185749	0.08806823	2238108.14	0	0.01576028	99.9264718	1360.02334
217	0.08497493	104.874727	0	0.08189452	0.08810641	2238121.17	0	0.01575991	99.9264746	1360.0739
218	0.0850139	104.966317	0	0.08193479	0.088144	2238018.3	0	0.01573949	99.92638	1358.32621
219	0.08499978	104.924204	0	0.08191994	0.08813065	2237384.62	0	0.01574946	99.926416	1358.99074
220	0.08500407	104.935786	0	0.08192441	0.08813474	2237673.82	0	0.01574677	99.926409	1358.86226

© 2024 Zhang X et al. *JAMA Pediatrics*.

221	0.08499336	104.909146	0	0.08191331	0.08812446	2238038.72	0	0.01575291	99.9264416	1359.46374
222	0.08498965	104.900857	0	0.08190949	0.08812087	2238038.68	0	0.01575471	99.9264495	1359.61027
223	0.0849915	104.904461	0	0.08191137	0.08812267	2237748.29	0	0.01575391	99.9264394	1359.42376
224	0.08499008	104.901854	0	0.08190993	0.08812128	2238050.86	0	0.0157545	99.9264489	1359.59887
225	0.08501457	104.968247	0	0.0819355	0.08814464	2237981.46	0	0.01573899	99.9263773	1358.27762
226	0.08499959	104.923906	0	0.08191975	0.08813046	2237843.55	0	0.01574955	99.9264234	1359.12789
227	0.08498775	104.897957	0	0.08190757	0.08811899	2238114.12	0	0.01575539	99.9264545	1359.70189
228	0.08493708	104.849984	0	0.08185726	0.08806796	2238134.27	0	0.01576015	99.9264754	1360.0897
229	0.08498484	104.890764	0	0.08190454	0.08811618	2237832.91	0	0.01575681	99.9264516	1359.64897
230	0.08493905	104.849714	0	0.08185916	0.08807001	2238136.83	0	0.01576061	99.9264774	1360.1256
231	0.08497086	104.868435	0	0.08189041	0.08810239	2238012.48	0	0.01576094	99.9264673	1359.9399
232	0.08496694	104.863611	0	0.08188648	0.08809847	2236445.52	0	0.01576165	99.9262036	1355.07941
233	0.08496342	104.861888	0	0.08188303	0.08809488	2238146.03	0	0.01576179	99.9264845	1360.25675
234	0.08490098	104.883035	0	0.08182339	0.0880296	2238074.36	0	0.01574319	99.9263974	1358.64829
235	0.08492031	104.861033	0	0.08184141	0.08805027	2238135.19	0	0.01575394	99.9264482	1359.58584
236	0.08498002	104.883031	0	0.08189967	0.08811143	2238117.63	0	0.01575839	99.926468	1359.95176
237	0.08494392	104.850129	0	0.08186387	0.08807504	2238145.27	0	0.01576146	99.926482	1360.21164
238	0.08497721	104.878342	0	0.08189682	0.08810866	2238120.62	0	0.01575926	99.9264719	1360.02408
239	0.08495501	104.854911	0	0.08187472	0.08808638	2238148.55	0	0.01576224	99.9264864	1360.29264
240	0.08493688	104.849428	0	0.08185706	0.08806778	2238046.68	0	0.0157602	99.9264627	1359.85372
241	0.08494343	104.85196	0	0.08186346	0.08807449	2238147.42	0	0.01576109	99.9264815	1360.20114
242	0.08494864	104.850522	0	0.08186844	0.08807992	2238145.31	0	0.01576214	99.9264782	1360.14158
243	0.08493702	104.850173	0	0.08185721	0.0880679	2238137.13	0	0.01576011	99.9264757	1360.09439
244	0.08494226	104.849077	0	0.08186224	0.08807335	2220918.81	0	0.01576133	99.9209415	1264.88631
245	0.08495414	104.85585	0	0.0818739	0.08808545	2238148.69	0	0.01576201	99.9264858	1360.28071
246	0.08500112	104.928235	0	0.08192135	0.08813192	2237990.29	0	0.01574857	99.9264213	1359.0886
247	0.08492064	104.858197	0	0.08184164	0.0880507	2238096.82	0	0.01575448	99.9264493	1359.60704
248	0.08496404	104.860793	0	0.0818836	0.08809555	2238125.48	0	0.01576199	99.9264802	1360.17705
249	0.0849498	104.851434	0	0.08186959	0.0880811	2238148.3	0	0.01576217	99.9264848	1360.2621
250	0.08483599	105.075798	0	0.08176626	0.08795653	2238046.9	0	0.01567256	99.9260668	1352.57166
251	0.08496755	104.864332	0	0.08188709	0.08809908	2237857.56	0	0.01576155	99.9264398	1359.43033
252	0.08481572	105.163579	0	0.08174924	0.08793292	2237792.83	0	0.01564144	99.9259193	1349.87924
253	0.08497598	104.875573	0	0.08189556	0.08810748	2237809.79	0	0.01575974	99.9264544	1359.70146
254	0.08495194	104.852055	0	0.08187167	0.08808329	2238148.71	0	0.01576234	99.9264838	1360.24471
255	0.08495106	104.851553	0	0.08187081	0.0880824	2238148.46	0	0.01576231	99.9264821	1360.21362
256	0.08489378	104.912564	0	0.0818173	0.08802125	2238136.71	0	0.01573533	99.9263616	1357.98734
257	0.08499015	104.901806	0	0.08190999	0.08812135	2238009.46	0	0.0157545	99.9264479	1359.58028
258	0.08497872	104.879924	0	0.08189832	0.08811017	2237913.28	0	0.01575895	99.9264601	1359.80682
259	0.08486662	104.964602	0	0.0817926	0.08799157	2238066.41	0	0.015712	99.9262521	1355.97051
260	0.08494177	104.85028	0	0.0818618	0.08807281	2238144.79	0	0.01576105	99.9264806	1360.18507
261	0.08494182	104.849167	0	0.08186181	0.0880729	2238108.3	0	0.01576123	99.9264698	1359.98474
262	0.08497668	104.876745	0	0.08189627	0.08810817	2238004.99	0	0.01575953	99.9264662	1359.91946

© 2024 Zhang X et al. *JAMA Pediatrics*.

263	0.08490352	104.878353	0	0.0818257	0.08803236	2238024.58	0	0.015745	99.9264051	1358.78891
264	0.08493347	104.852535	0	0.08185386	0.08806415	2238142.23	0	0.01575894	99.9264713	1360.01375
265	0.08499668	104.921871	0	0.08191688	0.08812751	2238131.86	0	0.01575039	99.9264318	1359.28304
266	0.08487853	104.936639	0	0.08180328	0.08800474	2238117.81	0	0.01572355	99.9263064	1356.97048
267	0.08498078	104.883383	0	0.08190041	0.0881122	2237878.32	0	0.01575829	99.9264572	1359.75268
268	0.08494229	104.850769	0	0.08186232	0.08807333	2238146.09	0	0.01576107	99.926481	1360.19287
269	0.0849446	104.849497	0	0.08186451	0.08807577	2238132.14	0	0.01576167	99.926475	1360.08122
270	0.08493894	104.85067	0	0.08185909	0.08806988	2238143.78	0	0.01576044	99.926478	1360.13717
271	0.08494397	104.850753	0	0.08186394	0.08807508	2238146.68	0	0.01576137	99.9264823	1360.21691
272	0.08495904	104.85726	0	0.08187867	0.08809048	2238147.07	0	0.01576224	99.926486	1360.28534
273	0.08496457	104.862145	0	0.08188415	0.08809606	2238142.73	0	0.0157618	99.9264839	1360.24584
274	0.08495182	104.851991	0	0.08187155	0.08808317	2238148.69	0	0.01576234	99.9264837	1360.24276
275	0.08487324	104.942325	0	0.08179834	0.08799909	2237598.89	0	0.01571955	99.9262847	1356.56983
276	0.08492456	104.854938	0	0.08184533	0.08805486	2238083.38	0	0.01575619	99.9264561	1359.73247
277	0.08492125	104.858769	0	0.08184224	0.08805131	2238126.57	0	0.01575458	99.9264509	1359.63505
278	0.08500112	104.927586	0	0.08192134	0.08813194	2219702.43	0	0.01574867	99.9261561	1354.2071
279	0.08490381	104.878475	0	0.08182598	0.08803266	2238075.97	0	0.01574511	99.9264063	1358.81211
280	0.08493414	104.852422	0	0.0818545	0.08806484	2238142.85	0	0.01575911	99.9264722	1360.02916
281	0.08494402	104.849826	0	0.08186396	0.08807515	2238143.53	0	0.01576152	99.9264814	1360.19995
282	0.08489197	104.90161	0	0.08181523	0.08801971	2238106.51	0	0.01573612	99.926365	1358.04993
283	0.08489923	104.886324	0	0.0818218	0.08802769	2238085.03	0	0.0157419	99.9263916	1358.54122
284	0.08488116	104.922592	0	0.08180541	0.08800789	2237947.88	0	0.0157272	99.9263222	1357.26163
285	0.08493409	104.851748	0	0.08185443	0.08806482	2238140.72	0	0.01575921	99.9264724	1360.03304
286	0.08498456	104.890174	0	0.08190426	0.08811592	2237566.45	0	0.01575693	99.926443	1359.49001
287	0.08490808	104.873076	0	0.08182995	0.08803725	2238107.97	0	0.01574769	99.9264187	1359.04174
288	0.08497333	104.871721	0	0.08189288	0.08810484	2237916.65	0	0.01576041	99.9264603	1359.81008
289	0.08494394	104.849598	0	0.08186388	0.08807508	2238140.22	0	0.01576155	99.9264799	1360.17212
290	0.08498739	104.895636	0	0.08190715	0.08811868	2231901.3	0	0.01575579	99.9262802	1356.48768
291	0.08499043	104.901974	0	0.08191027	0.08812164	2230492.15	0	0.01575444	99.9262675	1356.2533
292	0.08494885	104.850438	0	0.08186864	0.08808014	2238115.75	0	0.01576219	99.9263925	1358.55635
293	0.08498008	104.882182	0	0.08189971	0.08811152	2237853.88	0	0.01575852	99.9264566	1359.74104
294	0.08490185	104.880221	0	0.08182415	0.08803058	2236821.49	0	0.015744	99.9263834	1358.38971
295	0.0849559	104.853908	0	0.08187554	0.08808733	2238108.55	0	0.01576249	99.926375	1358.23501
296	0.08495013	104.850944	0	0.08186989	0.08808145	2238144.26	0	0.01576229	99.9264558	1359.72714
297	0.08497713	104.877367	0	0.08189671	0.08810861	2237901.81	0	0.01575942	99.9264601	1359.80633
298	0.08495513	104.853557	0	0.08187479	0.08808655	2238146.47	0	0.01576247	99.926478	1360.13642
299	0.08496931	104.866419	0	0.08188886	0.08810085	2237775.57	0	0.01576125	99.9264372	1359.38195
300	0.08497575	104.875194	0	0.08189532	0.08810724	2237675.35	0	0.01575981	99.9264457	1359.54009
301	0.08495579	104.853892	0	0.08187544	0.08808722	2238141.27	0	0.01576248	99.926466	1359.91518
302	0.08493603	104.849736	0	0.08185625	0.08806689	2238105.01	0	0.01575996	99.9264709	1360.00592
303	0.08496872	104.865705	0	0.08188826	0.08810026	2237794.25	0	0.01576136	99.9264367	1359.37408
304	0.08493136	104.850864	0	0.08185178	0.08806202	2237168.92	0	0.01575868	99.9263915	1358.53881

© 2024 Zhang X et al. *JAMA Pediatrics.*

305	0.08499499	104.912267	0	0.08191497	0.08812605	2228040.33	0	0.01575217	99.9262476	1355.88775
306	0.08501131	104.957145	0	0.08193203	0.0881416	2237678.98	0	0.01574165	99.9263866	1358.44811
307	0.08495383	104.852659	0	0.08187351	0.08808523	2238127.38	0	0.01576247	99.9261377	1353.87022
308	0.08494502	104.850502	0	0.08186494	0.08807617	2238146.57	0	0.01576159	99.9264829	1360.22844
309	0.08494893	104.850692	0	0.08186873	0.08808022	2238146.66	0	0.01576216	99.9264808	1360.18936
310	0.08496893	104.866622	0	0.08188849	0.08810044	2238132.01	0	0.01576122	99.9264804	1360.1822
311	0.08494466	104.849395	0	0.08186457	0.08807584	2238085.76	0	0.0157617	99.9264472	1359.56739
312	0.08491571	104.862075	0	0.08183699	0.08804548	2237601.28	0	0.01575223	99.926426	1359.17652
313	0.08493818	104.849586	0	0.08185832	0.08806912	2238128.92	0	0.01576045	99.9264755	1360.09119
314	0.0849734	104.871994	0	0.08189296	0.08810491	2238073.86	0	0.01576037	99.9264727	1360.03891
315	0.08489467	104.892676	0	0.08181758	0.08802277	2236405.95	0	0.0157388	99.926359	1357.9383
316	0.08492679	104.853436	0	0.08184744	0.08805721	2238077.46	0	0.01575706	99.9264593	1359.79175
317	0.08495405	104.852938	0	0.08187373	0.08808545	2238147.99	0	0.01576245	99.926479	1360.15646
318	0.08496581	104.862415	0	0.08188536	0.08809734	2237945.05	0	0.0157618	99.9264457	1359.54021
319	0.08495695	104.854632	0	0.08187658	0.0880884	2238126.72	0	0.01576247	99.9264514	1359.64579
320	0.08494347	104.849752	0	0.08186343	0.08807459	2238142.78	0	0.01576144	99.926481	1360.19237
321	0.08494109	104.8492	0	0.08186111	0.08807214	2238117.48	0	0.01576109	99.9264733	1360.04979
322	0.08495734	104.854863	0	0.08187696	0.08808879	2236648.9	0	0.01576247	99.9244007	1322.7634
323	0.08492645	104.853275	0	0.0818471	0.08805686	2236817.78	0	0.01575699	99.926392	1358.54824
324	0.08495249	104.853613	0	0.08187225	0.08808382	2238148.73	0	0.01576217	99.9264861	1360.28711
325	0.08492459	104.854452	0	0.08184534	0.0880549	2236776.79	0	0.01575627	99.9263959	1358.61979
326	0.08494916	104.850549	0	0.08186894	0.08808046	2238126.07	0	0.01576221	99.9264092	1358.86599
327	0.08490901	104.870239	0	0.08183076	0.08803829	2237973.86	0	0.01574849	99.9264199	1359.06258
328	0.08494093	104.849375	0	0.08186096	0.08807197	2238132.92	0	0.01576103	99.9264774	1360.12689
329	0.08492711	104.853053	0	0.08184773	0.08805755	2238001.5	0	0.01575721	99.9264557	1359.72387
330	0.08494352	104.849949	0	0.08186348	0.08807463	2238144.31	0	0.01576142	99.9264815	1360.20276
331	0.08494835	104.856892	0	0.08186835	0.08807943	2238148.59	0	0.01576112	99.9264819	1360.20912
332	0.08497539	104.875337	0	0.08189498	0.08810687	2238116.98	0	0.0157598	99.9264739	1360.06083
333	0.085012	104.958983	0	0.08193274	0.08814225	2230060.53	0	0.01574117	99.9263106	1357.04794
334	0.08498436	104.889736	0	0.08190406	0.08811573	2234537.66	0	0.01575702	99.9263401	1357.59108
335	0.08498089	104.883473	0	0.08190052	0.08811232	2236671.55	0	0.01575826	99.9264054	1358.79575
336	0.08498038	104.88259	0	0.0819	0.08811181	2236491.26	0	0.01575843	99.9263958	1358.61902
337	0.08491794	104.86016	0	0.08183909	0.08804784	2238020.07	0	0.01575329	99.9264417	1359.46656
338	0.08492376	104.855266	0	0.08184456	0.08805401	2238022.25	0	0.0157559	99.9264523	1359.66101
339	0.08498478	104.890522	0	0.08190448	0.08811613	2234573.04	0	0.01575686	99.9263437	1357.65685
340	0.08490975	104.868865	0	0.08183144	0.0880391	2234756.57	0	0.01574899	99.9263583	1357.92545
341	0.08494764	104.850802	0	0.08186748	0.08807888	2238147.47	0	0.01576196	99.9264841	1360.24938
342	0.08493019	104.851357	0	0.08185066	0.08806079	2237426.35	0	0.0157583	99.9264165	1359.001
343	0.08494402	104.849358	0	0.08186395	0.08807517	2238119.75	0	0.0157616	99.9264696	1359.98171
344	0.08494325	104.84968	0	0.08186321	0.08807436	2238141.93	0	0.01576141	99.9264806	1360.18554
345	0.08493135	104.850949	0	0.08185176	0.088062	2237961.27	0	0.01575866	99.9264555	1359.72151
346	0.08497607	104.875648	0	0.08189565	0.08810757	2235652.48	0	0.01575973	99.9263204	1357.22856

© 2024 Zhang X et al. *JAMA Pediatrics.*

347	0.0849638	104.8607	0	0.08188337	0.08809531	2238134.46	0	0.01576199	99.9264823	1360.21745
348	0.08498434	104.889787	0	0.08190404	0.0881157	2237709.26	0	0.01575701	99.926448	1359.58196
349	0.08496937	104.867447	0	0.08188894	0.08810088	2238136.16	0	0.01576109	99.9264805	1360.18289
350	0.08494567	104.849845	0	0.08186556	0.08807687	2238142.43	0	0.0157618	99.9264803	1360.1803
351	0.08494469	104.849418	0	0.08186459	0.08807586	2238106.55	0	0.0157617	99.9264595	1359.79425
352	0.08493954	104.849246	0	0.08185962	0.08807053	2238112.84	0	0.01576078	99.9264728	1360.04098
353	0.08498595	104.894702	0	0.08190574	0.08811722	2238122.76	0	0.01575609	99.9264579	1359.7651
354	0.08492993	104.851585	0	0.08185041	0.08806051	2237999.3	0	0.0157582	99.9264579	1359.76567
355	0.08494601	104.849805	0	0.08186588	0.08807722	2238139.64	0	0.01576186	99.9264776	1360.13006
356	0.08494198	104.849078	0	0.08186197	0.08807307	2237894.48	0	0.01576128	99.9263991	1358.67992
357	0.08496812	104.865035	0	0.08188766	0.08809965	2238018.67	0	0.01576145	99.9264653	1359.90141
358	0.08494434	104.849393	0	0.08186425	0.0880755	2238118.54	0	0.01576165	99.926468	1359.95172
359	0.0849049	104.877036	0	0.08182699	0.08803383	2238087.09	0	0.01574578	99.9264096	1358.87295
360	0.08497965	104.881458	0	0.08189927	0.08811109	2237875.62	0	0.01575865	99.9264578	1359.76388
361	0.0849923	104.906179	0	0.0819122	0.08812345	2237426.27	0	0.01575353	99.926431	1359.26802
362	0.0849872	104.8953	0	0.08190696	0.0881185	2237028.77	0	0.01575587	99.9264255	1359.1665
363	0.08498419	104.889415	0	0.08190388	0.08811556	2234698.91	0	0.01575708	99.9263446	1357.67422
364	0.08497518	104.874323	0	0.08189475	0.08810669	2237008.46	0	0.01575996	99.9264001	1358.69742
365	0.0849717	104.86943	0	0.08189124	0.08810323	2236190.1	0	0.01576079	99.9262972	1356.79999
366	0.08496857	104.865492	0	0.08188811	0.0881001	2236452.06	0	0.01576139	99.9262569	1356.05974
367	0.08496929	104.866367	0	0.08188883	0.08810083	2236441.06	0	0.01576126	99.926274	1356.37401
368	0.08496665	104.863299	0	0.0818862	0.08809819	2237056.57	0	0.01576169	99.9262971	1356.79951
369	0.08497088	104.868874	0	0.08189044	0.08810239	2238120.72	0	0.01576088	99.9264781	1360.13944
370	0.0849404	104.84903	0	0.08186045	0.08807144	2235851.99	0	0.01576098	99.9259144	1349.78931
371	0.08494181	104.849069	0	0.0818618	0.08807289	2237888.09	0	0.01576125	99.9263997	1358.69012
372	0.08496434	104.860899	0	0.08188389	0.08809586	2238036.65	0	0.01576198	99.9264586	1359.77899
373	0.08496993	104.867185	0	0.08188947	0.08810146	2237855.75	0	0.01576114	99.9264489	1359.59833
374	0.08497825	104.879077	0	0.08189785	0.08810971	2237416.78	0	0.0157591	99.9264345	1359.33323
375	0.08496529	104.861833	0	0.08188484	0.08809682	2236672.68	0	0.01576187	99.9261762	1354.57683
376	0.08495817	104.856246	0	0.08187781	0.08808962	2238146.9	0	0.01576233	99.9264858	1360.28186
377	0.08495752	104.855317	0	0.08187715	0.08808897	2238145.24	0	0.01576242	99.9264835	1360.23933
378	0.08497579	104.875648	0	0.08189538	0.08810728	2238094.52	0	0.01575974	99.9264722	1360.03036
379	0.08494367	104.849842	0	0.08186362	0.08807479	2238143.63	0	0.01576146	99.9264814	1360.19927
380	0.08492566	104.853805	0	0.08184635	0.08805602	2237715.66	0	0.01575668	99.9264397	1359.42929
381	0.0849931	104.908064	0	0.08191302	0.08812422	2237772.61	0	0.01575312	99.9264369	1359.37811
382	0.08495661	104.854747	0	0.08187625	0.08808804	2238146.78	0	0.01576242	99.9264844	1360.25459
383	0.08491817	104.859687	0	0.0818393	0.08804809	2237764.03	0	0.01575344	99.9264345	1359.3332
384	0.08497668	104.876799	0	0.08189626	0.08810816	2238043.55	0	0.01575953	99.9264685	1359.96091
385	0.08497286	104.871091	0	0.08189241	0.08810437	2237980.23	0	0.01576052	99.9264652	1359.90125
386	0.08496215	104.859173	0	0.08188173	0.08809365	2238139.04	0	0.01576214	99.9264833	1360.23589
387	0.08497573	104.876132	0	0.08189533	0.08810719	2238125.56	0	0.01575966	99.9264739	1360.06055
388	0.08501049	104.954295	0	0.08193115	0.08814083	2192919.85	0	0.01574231	99.9259347	1350.16054

© 2024 Zhang X et al. *JAMA Pediatrics*.

389	0.08498351	104.888141	0	0.08190319	0.08811489	2234768.68	0	0.01575734	99.926343	1357.64474
390	0.08498503	104.891463	0	0.08190475	0.08811636	2238051.56	0	0.01575669	99.9264583	1359.77246
391	0.08498019	104.883158	0	0.08189984	0.0881116	2238111.68	0	0.01575836	99.9264676	1359.94449
392	0.0850017	104.929282	0	0.08192194	0.08813248	2237558.04	0	0.01574829	99.9264139	1358.95176
393	0.08495471	104.853215	0	0.08187438	0.08808613	2238144.21	0	0.01576248	99.9264595	1359.79501
394	0.0849792	104.880626	0	0.08189881	0.08811066	2237069.69	0	0.01575881	99.926419	1359.04673
395	0.08500103	104.927329	0	0.08192123	0.08813185	2220792.26	0	0.01574873	99.9261711	1354.48308
396	0.08495236	104.851904	0	0.08187207	0.08808373	2238148.73	0	0.01576242	99.9263913	1358.53534
397	0.08497805	104.879432	0	0.08189767	0.0881095	2238108.61	0	0.01575906	99.9264704	1359.99629
398	0.08495219	104.852314	0	0.08187191	0.08808354	2238148.72	0	0.01576233	99.9264849	1360.26415
399	0.08496294	104.859709	0	0.08188251	0.08809445	2238127.36	0	0.0157621	99.92648	1360.17389
400	0.0849681	104.865	0	0.08188764	0.08809963	2237986.91	0	0.01576146	99.9264607	1359.81787
401	0.08496082	104.857637	0	0.0818804	0.08809232	2238098.95	0	0.0157623	99.9264631	1359.86157
402	0.0849619	104.858582	0	0.08188147	0.0880934	2238086.8	0	0.01576222	99.9264633	1359.8643
403	0.08489322	104.89699	0	0.08181631	0.08802114	2238058.78	0	0.01573744	99.9263707	1358.15443
404	0.08495608	104.85411	0	0.08187573	0.08808752	2238142.74	0	0.01576247	99.9264731	1360.04685
405	0.08493019	104.851357	0	0.08185066	0.08806079	2237426.35	0	0.0157583	99.9264165	1359.001
406	0.08498478	104.890522	0	0.08190448	0.08811613	2234573.04	0	0.01575686	99.9263437	1357.65685
407	0.08495973	104.856845	0	0.08187933	0.08809121	2238135.54	0	0.01576235	99.9264793	1360.16095
408	0.08495794	104.855342	0	0.08187755	0.0880894	2238119.96	0	0.01576245	99.9264557	1359.72396
409	0.0849725	104.870929	0	0.08189206	0.08810401	2238108.85	0	0.01576055	99.926476	1360.10021
410	0.08498378	104.88864	0	0.08190346	0.08811516	2226680.54	0	0.01575724	99.9260604	1352.45568
411	0.0849463	104.849779	0	0.08186616	0.08807752	2238132.56	0	0.01576191	99.9264702	1359.99372
412	0.08496685	104.863706	0	0.0818864	0.08809837	2238104.12	0	0.01576163	99.926477	1360.11922
413	0.08492317	104.856595	0	0.08184404	0.08805337	2238118.15	0	0.01575552	99.9264547	1359.70679
414	0.08498739	104.895751	0	0.08190716	0.08811868	2237705.14	0	0.01575578	99.9264446	1359.51987
415	0.08498144	104.884535	0	0.08190108	0.08811285	2237863.1	0	0.01575806	99.9264561	1359.7315
416	0.08497423	104.872916	0	0.08189379	0.08810574	2233438.02	0	0.01576021	99.9261339	1353.80104
417	0.08501243	104.96038	0	0.08193321	0.08814266	2234018.62	0	0.01574083	99.926348	1357.73611
418	0.0850117	104.958059	0	0.08193243	0.08814197	2233721.48	0	0.0157414	99.9263467	1357.71284
419	0.08500551	104.939619	0	0.08192592	0.08813612	2234746.26	0	0.01574584	99.9263692	1358.12731
420	0.08493678	104.849601	0	0.08185697	0.08806767	2238108.73	0	0.01576015	99.9264717	1360.02146
421	0.0849644	104.860993	0	0.08188395	0.08809592	2238077.4	0	0.01576197	99.9264688	1359.96622
422	0.08496044	104.857324	0	0.08188002	0.08809193	2238109	0	0.01576233	99.9264663	1359.92019
423	0.08496511	104.862072	0	0.08188467	0.08809662	2238133.01	0	0.01576183	99.926482	1360.21176
424	0.08490284	104.880332	0	0.08182511	0.0880316	2238089.03	0	0.01574441	99.9264033	1358.75637
425	0.0849572	104.855365	0	0.08187684	0.08808864	2238147.08	0	0.01576238	99.9264855	1360.27592
426	0.08494135	104.849042	0	0.08186136	0.08807242	2237453.54	0	0.01576116	99.926279	1356.4647
427	0.08494544	104.849516	0	0.08186532	0.08807664	2238041.05	0	0.01576181	99.9264058	1358.80263
428	0.08496405	104.860731	0	0.08188361	0.08809557	2238115.24	0	0.015762	99.9264776	1360.12939
429	0.0849468	104.849855	0	0.08186664	0.08807803	2238122.25	0	0.01576197	99.9264559	1359.72795
430	0.08497879	104.879923	0	0.08189839	0.08811025	2227984.1	0	0.01575894	99.9259659	1350.72956

© 2024 Zhang X et al. *JAMA Pediatrics*.

431	0.08497952	104.881132	0	0.08189913	0.08811097	2235170.77	0	0.01575871	99.9263299	1357.40329
432	0.08498595	104.893084	0	0.08190569	0.08811727	2237989.33	0	0.01575634	99.9264549	1359.71057
433	0.08495419	104.853277	0	0.08187388	0.08808559	2238148.4	0	0.01576241	99.9264846	1360.25854
434	0.08495428	104.853589	0	0.08187397	0.08808566	2238148.51	0	0.01576237	99.9264858	1360.28079
435	0.08491878	104.859226	0	0.08183988	0.08804875	2237945.83	0	0.01575372	99.9264412	1359.456
436	0.08498011	104.8835	0	0.08189977	0.08811151	2238124.45	0	0.01575831	99.9264679	1359.95089
437	0.0849899	104.90089	0	0.08190973	0.08812113	2236862.79	0	0.01575468	99.9264203	1359.07125
438	0.08497192	104.870049	0	0.08189147	0.08810343	2238100.51	0	0.01576069	99.9264757	1360.09447
439	0.08494523	104.85062	0	0.08186515	0.08807639	2238146.83	0	0.01576161	99.9264831	1360.23161
440	0.08500109	104.927781	0	0.08192131	0.0881319	2237777.41	0	0.01574865	99.9264185	1359.03777
441	0.08497043	104.868759	0	0.08189	0.08810193	2238134.38	0	0.01576089	99.9264795	1360.16549
442	0.08496676	104.863728	0	0.08188632	0.08809829	2238120.71	0	0.01576162	99.9264797	1360.16916
443	0.08500635	104.94218	0	0.08192681	0.08813691	2237477.69	0	0.01574524	99.9264001	1358.6975
444	0.08499235	104.906286	0	0.08191225	0.0881235	2237473.4	0	0.01575351	99.9264319	1359.28562
445	0.08492994	104.851442	0	0.08185042	0.08806053	2229557.63	0	0.01575822	99.9258625	1348.84486
446	0.08500732	104.94506	0	0.08192782	0.08813783	2237641.73	0	0.01574456	99.9263991	1358.67848
447	0.08499364	104.909805	0	0.0819136	0.08812473	2238039.16	0	0.01575277	99.9264409	1359.45195
448	0.08493272	104.85068	0	0.08185308	0.08806343	2238098.99	0	0.01575904	99.9264679	1359.94959
449	0.08496334	104.861598	0	0.08188295	0.08809481	2238145.73	0	0.01576183	99.9264846	1360.25852
450	0.08495997	104.857741	0	0.08187958	0.08809143	2238145.92	0	0.01576223	99.9264856	1360.27746
451	0.08500064	104.926515	0	0.08192084	0.08813147	2237658.85	0	0.01574893	99.926418	1359.02818
452	0.08492814	104.852466	0	0.08184871	0.08805863	2237987.67	0	0.01575758	99.9264558	1359.72739
453	0.08499677	104.916717	0	0.08191682	0.08812776	2237677.92	0	0.01575118	99.9264275	1359.20341
454	0.0849923	104.908284	0	0.08191225	0.08812338	2238116.53	0	0.0157532	99.9264445	1359.51788
455	0.08498639	104.894752	0	0.08190616	0.08811768	2238103.2	0	0.01575604	99.9264571	1359.75004
456	0.08495606	104.854289	0	0.08187571	0.08808749	2238146.74	0	0.01576244	99.9264831	1360.23193
457	0.08497186	104.870232	0	0.08189142	0.08810337	2238121.51	0	0.01576066	99.9264775	1360.12743
458	0.08495858	104.855894	0	0.08187819	0.08809005	2238135.2	0	0.01576241	99.9264758	1360.09653
459	0.08495747	104.855229	0	0.08187709	0.08808892	2238144.74	0	0.01576242	99.9264828	1360.22553
460	0.08497969	104.882428	0	0.08189933	0.08811111	2238116.99	0	0.0157585	99.9264684	1359.96036
461	0.08496278	104.86258	0	0.08188244	0.0880942	2238147.27	0	0.01576165	99.9264841	1360.25048
462	0.08496567	104.862823	0	0.08188523	0.08809718	2238136.09	0	0.01576173	99.9264825	1360.21998
463	0.08497566	104.875181	0	0.08189523	0.08810715	2238016.22	0	0.01575981	99.9264675	1359.94276
464	0.08492832	104.852461	0	0.08184888	0.08805882	2238048.54	0	0.01575763	99.9264597	1359.79855
465	0.08496318	104.860317	0	0.08188276	0.08809468	2238140.51	0	0.01576202	99.9264839	1360.24537
466	0.08499833	104.92095	0	0.08191844	0.08812925	2237985.28	0	0.01575024	99.9264287	1359.22612
467	0.08500104	104.927385	0	0.08192125	0.08813186	2234448.77	0	0.01574872	99.9263702	1358.14584
468	0.08501027	104.953779	0	0.08193092	0.08814062	2237297.64	0	0.01574245	99.9263863	1358.4419
469	0.08499319	104.908254	0	0.08191311	0.0881243	2237750.4	0	0.01575308	99.9264363	1359.3665
470	0.08496179	104.858945	0	0.08188138	0.08809328	2238141.44	0	0.01576216	99.9264841	1360.24966
471	0.08500685	104.943637	0	0.08192733	0.08813739	2237513.47	0	0.0157449	99.926399	1358.678
472	0.08498736	104.896478	0	0.08190715	0.08811863	2238089.58	0	0.01575567	99.926455	1359.71216

© 2024 Zhang X et al. *JAMA Pediatrics*.

473	0.08500875	104.94984	0	0.08192935	0.08813917	2237972.49	0	0.01574346	99.9263978	1358.65504
474	0.08500623	104.94168	0	0.08192668	0.08813681	2233989.56	0	0.01574535	99.926359	1357.93959
475	0.08498887	104.898757	0	0.08190867	0.08812012	2237486.76	0	0.01575514	99.9264369	1359.37803
476	0.08495392	104.854282	0	0.08187365	0.08808527	2238148.67	0	0.01576223	99.9264863	1360.29122
477	0.08500637	104.942267	0	0.08192683	0.08813693	2237554	0	0.01574522	99.9264009	1358.7127
478	0.08494264	104.849202	0	0.08186261	0.08807375	2238108.19	0	0.01576138	99.9264681	1359.95456
479	0.08500644	104.942434	0	0.0819269	0.088137	2237449.28	0	0.01574518	99.9263995	1358.68653
480	0.08496432	104.862196	0	0.08188391	0.0880958	2238144.25	0	0.01576178	99.9264841	1360.25009
481	0.08498726	104.896986	0	0.08190706	0.0881185	2238115.7	0	0.0157556	99.9264554	1359.71999
482	0.08500355	104.934286	0	0.08192387	0.08813425	2237494.65	0	0.01574711	99.9264082	1358.84623
483	0.0849739	104.872935	0	0.08189347	0.0881054	2238108.61	0	0.01576021	99.9264749	1360.07906
484	0.08495343	104.852987	0	0.08187313	0.08808481	2238148.64	0	0.01576237	99.9264852	1360.27115
485	0.08493803	104.850051	0	0.08185818	0.08806894	2238138.74	0	0.01576034	99.9264768	1360.11504
486	0.08500673	104.943622	0	0.08192722	0.08813727	2237900.76	0	0.01574493	99.9264037	1358.76361
487	0.08494403	104.849332	0	0.08186395	0.08807518	2238109.22	0	0.0157616	99.9264642	1359.88241
488	0.08496963	104.867965	0	0.08188921	0.08810113	2238137.91	0	0.01576101	99.9264804	1360.18081
489	0.08499758	104.920471	0	0.08191771	0.08812849	2238101.67	0	0.01575045	99.9264316	1359.27903
490	0.08483861	105.061769	0	0.08176838	0.08795966	2237946.96	0	0.01567683	99.9260866	1352.93397
491	0.084955	104.853491	0	0.08187467	0.08808642	2238146.85	0	0.01576246	99.9264788	1360.15245
492	0.08492708	104.854019	0	0.08184773	0.08805749	2238124.67	0	0.01575705	99.9264618	1359.83809
493	0.08493801	104.850405	0	0.08185818	0.08806892	2238141.52	0	0.01576029	99.926477	1360.11906
494	0.08490066	104.88459	0	0.08182313	0.08802922	2238103.15	0	0.0157428	99.9263961	1358.62294
495	0.08488759	104.909662	0	0.08181124	0.08801493	2238083.85	0	0.01573265	99.9263487	1357.7485
496	0.08493286	104.851286	0	0.08185324	0.08806356	2238132.21	0	0.01575898	99.9264706	1360.00102
497	0.08495154	104.851663	0	0.08187127	0.08808289	2238148.55	0	0.01576236	99.9264788	1360.15219
498	0.08497079	104.8689	0	0.08189036	0.0881023	2238126.61	0	0.01576087	99.9264787	1360.14987
499	0.08491292	104.869985	0	0.08183453	0.08804235	2238134.38	0	0.01575002	99.92643	1359.25005
500	0.08494507	104.850355	0	0.08186499	0.08807623	2238146.26	0	0.01576162	99.9264829	1360.22687
501	0.08495305	104.852245	0	0.08187275	0.08808443	2238146.12	0	0.01576245	99.926293	1356.7232
502	0.08498289	104.887419	0	0.08190257	0.08811428	2238051.13	0	0.0157575	99.9264616	1359.83397
503	0.08501002	104.953073	0	0.08193066	0.08814038	2237513.1	0	0.01574263	99.9263892	1358.49694
504	0.08500406	104.935734	0	0.08192441	0.08813474	2237576.58	0	0.01574677	99.9264078	1358.83976
505	0.08498712	104.89842	0	0.08190697	0.08811831	2238132.31	0	0.01575539	99.9264549	1359.71078
506	0.08495941	104.858003	0	0.08187905	0.08809085	2238147.4	0	0.01576215	99.926486	1360.28467
507	0.08497415	104.873823	0	0.08189374	0.08810563	2238128.62	0	0.01576007	99.9264757	1360.09534
508	0.08498952	104.901825	0	0.08190939	0.0881207	2238114.68	0	0.01575458	99.9264508	1359.63378
509	0.08497951	104.882768	0	0.08189917	0.08811091	2238129.27	0	0.01575846	99.9264688	1359.96714
510	0.08496941	104.868348	0	0.08188901	0.08810089	2238141.87	0	0.01576095	99.9264805	1360.18377
511	0.0850066	104.942791	0	0.08192706	0.08813715	2236677.1	0	0.01574509	99.92639	1358.5119
512	0.08500109	104.929135	0	0.08192135	0.08813186	2238084.06	0	0.01574843	99.926422	1359.10225
513	0.08499947	104.924304	0	0.08191964	0.08813033	2238052.86	0	0.01574951	99.9264265	1359.18447
514	0.08502423	105.001317	0	0.08194578	0.08815365	2237645.92	0	0.01573066	99.9263366	1357.52566

© 2024 Zhang X et al. *JAMA Pediatrics*.

515	0.08500641	104.942728	0	0.08192688	0.08813696	2237919.65	0	0.01574514	99.9264049	1358.78577
516	0.08498735	104.896863	0	0.08190714	0.0881186	2238108.06	0	0.01575561	99.9264553	1359.71684
517	0.084957	104.855177	0	0.08187664	0.08808843	2238147.08	0	0.01576239	99.9264853	1360.27281
518	0.08497399	104.873049	0	0.08189356	0.08810549	2238106.83	0	0.01576019	99.9264747	1360.07538
519	0.08497413	104.872864	0	0.08189369	0.08810564	2237937.21	0	0.01576022	99.9264623	1359.84625
520	0.08499327	104.908351	0	0.0819132	0.08812439	2237252.17	0	0.01575305	99.9264259	1359.17356
521	0.08497748	104.879451	0	0.08189712	0.08810891	2238131.78	0	0.01575908	99.9264717	1360.02027
522	0.08499409	104.910325	0	0.08191405	0.08812518	2237691.14	0	0.01575262	99.9264333	1359.31159
523	0.08499087	104.903054	0	0.08191073	0.08812206	2237665.78	0	0.01575421	99.9264386	1359.40865
524	0.08497769	104.87927	0	0.08189731	0.08810913	2238123.86	0	0.0157591	99.9264714	1360.01418
525	0.08499597	104.914763	0	0.08191599	0.08812699	2237674.53	0	0.01575162	99.9264292	1359.23459
526	0.08499691	104.917243	0	0.08191697	0.08812789	2237919.74	0	0.01575108	99.9264313	1359.27341
527	0.08497311	104.872999	0	0.08189271	0.08810458	2238137.41	0	0.01576022	99.926477	1360.11853
528	0.08498861	104.898127	0	0.0819084	0.08811987	2232199.24	0	0.01575527	99.9262974	1356.80372
529	0.084993	104.907694	0	0.08191292	0.08812413	2236547.31	0	0.01575319	99.9264115	1358.90724
530	0.0849862	104.894071	0	0.08190596	0.0881175	2238087.58	0	0.01575616	99.9264572	1359.75182
531	0.08499375	104.909479	0	0.08191369	0.08812485	2237487.03	0	0.0157528	99.9264299	1359.24816
532	0.08499963	104.924058	0	0.08191979	0.0881305	2237877.16	0	0.01574951	99.9264238	1359.13492
533	0.08498996	104.90143	0	0.0819098	0.08812117	2238016.57	0	0.01575459	99.9264484	1359.58998
534	0.08499103	104.904113	0	0.08191091	0.0881222	2238070.46	0	0.01575403	99.9264472	1359.56834
535	0.08498985	104.902124	0	0.08190972	0.08812104	2238104.28	0	0.01575449	99.9264501	1359.62168
536	0.08499264	104.909544	0	0.08191263	0.0881237	2238121.56	0	0.01575296	99.9264435	1359.49884
537	0.08493457	104.850472	0	0.08185486	0.08806535	2238128.48	0	0.01575951	99.9264724	1360.03327
538	0.08500037	104.926096	0	0.08192057	0.08813121	2237938.67	0	0.01574905	99.9264227	1359.11457
539	0.08498673	104.895136	0	0.0819065	0.08811802	2238086.09	0	0.01575594	99.9264561	1359.73298
540	0.08500302	104.932769	0	0.08192331	0.08813374	2237158.9	0	0.01574747	99.9264051	1358.78954
541	0.08497381	104.872517	0	0.08189337	0.08810532	2238049.08	0	0.01576028	99.9264706	1360.00048
542	0.08498334	104.888362	0	0.08190303	0.08811471	2238071.48	0	0.01575732	99.9264616	1359.8338
543	0.08492888	104.852251	0	0.08184942	0.08805941	2238075.8	0	0.01575782	99.926462	1359.84164
544	0.08500305	104.93287	0	0.08192335	0.08813377	2237292.48	0	0.01574744	99.9264068	1358.82114
545	0.08496481	104.861938	0	0.08188438	0.08809632	2238137.93	0	0.01576184	99.926483	1360.23022
546	0.08498345	104.888479	0	0.08190314	0.08811482	2238058.11	0	0.01575729	99.926461	1359.82289
547	0.08498104	104.884172	0	0.08190068	0.08811245	2238072.95	0	0.01575814	99.926465	1359.89749
548	0.08498461	104.89052	0	0.08190432	0.08811596	2238013.21	0	0.01575687	99.9264578	1359.76314
549	0.08497185	104.870017	0	0.08189141	0.08810337	2238107.34	0	0.01576069	99.9264763	1360.10607
550	0.0849683	104.865839	0	0.08188786	0.08809981	2238132.51	0	0.01576133	99.9264809	1360.19002
551	0.08490034	104.883032	0	0.08182277	0.08802893	2237896.25	0	0.0157429	99.9263937	1358.58024
552	0.08498426	104.88977	0	0.08190396	0.08811561	2237970.23	0	0.01575702	99.9264569	1359.74683
553	0.0849866	104.894301	0	0.08190635	0.0881179	2237949.97	0	0.01575609	99.9264528	1359.67029
554	0.08495403	104.853044	0	0.08187371	0.08808542	2238148.31	0	0.01576243	99.9264829	1360.22704
555	0.08496109	104.86242	0	0.0818808	0.08809246	2238148	0	0.01576158	99.9264839	1360.24689
556	0.08497724	104.877819	0	0.08189683	0.08810871	2238080.22	0	0.01575934	99.92647	1359.98877

© 2024 Zhang X et al. *JAMA Pediatrics*.

557	0.08496986	104.867302	0	0.08188941	0.08810139	2238094.07	0	0.01576112	99.9264759	1360.09743
558	0.08498158	104.884692	0	0.08190122	0.088113	2237180.93	0	0.01575803	99.9264281	1359.2149
559	0.08500582	104.941242	0	0.08192627	0.08813639	2237985.11	0	0.01574551	99.9264073	1358.83132
560	0.08499075	104.90318	0	0.08191061	0.08812193	2238026.82	0	0.01575421	99.926447	1359.56461
561	0.08494952	104.850813	0	0.0818693	0.08808083	2238146.46	0	0.01576222	99.9264774	1360.12544
562	0.08497934	104.880849	0	0.08189895	0.08811079	2236934.77	0	0.01575877	99.9264129	1358.93327
563	0.08494926	104.851217	0	0.08186906	0.08808054	2238148.09	0	0.01576212	99.9264845	1360.25769
564	0.08502361	104.999049	0	0.08194512	0.08815307	2237694	0	0.01573124	99.9263395	1357.58039
565	0.08498469	104.891148	0	0.08190442	0.08811603	2238091.64	0	0.01575677	99.9264599	1359.80288
566	0.08498668	104.894398	0	0.08190643	0.08811799	2237868.94	0	0.01575606	99.9264503	1359.62444
567	0.08500527	104.939359	0	0.08192568	0.08813588	2237873.98	0	0.01574594	99.9264079	1358.84151
568	0.08497928	104.881176	0	0.0818989	0.08811072	2238079.26	0	0.01575872	99.9264676	1359.94465
569	0.08499308	104.908021	0	0.081913	0.0881242	2237782.82	0	0.01575313	99.9264372	1359.38277
570	0.08499268	104.907109	0	0.08191259	0.08812381	2237762.58	0	0.01575333	99.9264376	1359.3892
571	0.08498293	104.887216	0	0.0819026	0.08811432	2237872.09	0	0.01575753	99.9264551	1359.71291
572	0.08498059	104.883365	0	0.08190023	0.08811201	2238068.91	0	0.0157583	99.9264655	1359.90568
573	0.08497362	104.872105	0	0.08189317	0.08810513	2237847.15	0	0.01576035	99.9264551	1359.71397
574	0.08498808	104.89718	0	0.08190786	0.08811935	2237797.45	0	0.01575548	99.9264462	1359.54954
575	0.08495575	104.854778	0	0.08187542	0.08808715	2238148.24	0	0.01576234	99.9264863	1360.29014
576	0.08499248	104.908066	0	0.08191242	0.08812357	2238104.06	0	0.01575321	99.9264443	1359.51371
577	0.08497862	104.880039	0	0.08189823	0.08811007	2238074.19	0	0.01575893	99.9264682	1359.95506
578	0.08495303	104.852652	0	0.08187274	0.0880844	2238148.69	0	0.01576238	99.9264845	1360.25746
579	0.08500182	104.929608	0	0.08192207	0.0881326	2237543.96	0	0.01574821	99.9264134	1358.94228
580	0.08497596	104.876721	0	0.08189557	0.08810742	2238129.41	0	0.01575956	99.9264737	1360.05699
581	0.08496579	104.864728	0	0.08188541	0.08809725	2238145.42	0	0.01576144	99.926483	1360.22937
582	0.08493957	104.849411	0	0.08185966	0.08807056	2238129.54	0	0.01576076	99.9264763	1360.10579
583	0.08498527	104.891643	0	0.08190499	0.08811661	2237895.75	0	0.01575663	99.9264531	1359.67613
584	0.08495078	104.854863	0	0.08187063	0.08808201	2238148.69	0	0.01576177	99.9264847	1360.26142
585	0.08499978	104.924207	0	0.08191994	0.08813065	2237396.38	0	0.01574946	99.9264162	1358.99405
586	0.08498534	104.89228	0	0.08190508	0.08811666	2238080.34	0	0.01575653	99.9264585	1359.77709
587	0.0849828	104.886901	0	0.08190246	0.08811419	2237582.99	0	0.01575759	99.9264444	1359.51497
588	0.08498391	104.890368	0	0.08190364	0.08811523	2238119.55	0	0.01575696	99.9264617	1359.83578
589	0.08494539	104.854863	0	0.08186543	0.08807643	2238148.27	0	0.01576098	99.9264812	1360.19589
590	0.08495959	104.857017	0	0.08187919	0.08809106	2238143.92	0	0.01576232	99.9264844	1360.25632
591	0.0849672	104.86431	0	0.08188675	0.08809872	2238125.83	0	0.01576154	99.9264805	1360.18252
592	0.08497715	104.880063	0	0.08189682	0.08810855	2238138.83	0	0.015759	99.9264717	1360.02029
593	0.08498921	104.899556	0	0.08190903	0.08812045	2237810.16	0	0.01575497	99.9264448	1359.5229
594	0.08498285	104.887463	0	0.08190253	0.08811423	2238074.02	0	0.0157575	99.9264624	1359.8494
595	0.08493224	104.852454	0	0.08185266	0.08806288	2238139.62	0	0.01575865	99.9264698	1359.98606
596	0.08496194	104.860091	0	0.08188156	0.08809341	2238146.16	0	0.01576199	99.9264852	1360.27027
597	0.08496008	104.859008	0	0.08187973	0.08809151	2238147.49	0	0.01576204	99.9264857	1360.27927
598	0.0849447	104.852333	0	0.08186469	0.08807578	2238147.82	0	0.01576125	99.9264822	1360.21554

© 2024 Zhang X et al. *JAMA Pediatrics*.

599	0.08494247	104.855153	0	0.08186262	0.0880734	2238147.92	0	0.01576043	99.9264787	1360.14921
600	0.08501025	104.9537	0	0.0819309	0.0881406	2237100.68	0	0.01574247	99.9263843	1358.40578
601	0.08499264	104.909544	0	0.08191263	0.0881237	2238121.56	0	0.01575296	99.9264435	1359.49884
602	0.08497098	104.868758	0	0.08189053	0.0881025	2238095.06	0	0.01576089	99.9264756	1360.0935
603	0.08501567	104.970914	0	0.08193663	0.08814569	2236653.97	0	0.01573825	99.9263625	1358.00421
604	0.08488856	104.907872	0	0.08181212	0.08801598	2238091.2	0	0.01573343	99.9263523	1357.81643
605	0.08495358	104.853356	0	0.08187329	0.08808495	2238148.66	0	0.01576233	99.926486	1360.28491
606	0.08490016	104.885698	0	0.08182268	0.08802867	2238106.72	0	0.01574241	99.9263943	1358.59023
607	0.08502488	105.006664	0	0.08194656	0.08815416	2238075.64	0	0.01572959	99.9263344	1357.48594
608	0.0849735	104.872109	0	0.08189307	0.08810501	2238063.28	0	0.01576035	99.9264718	1360.02292
609	0.08497608	104.876159	0	0.08189567	0.08810756	2238101.81	0	0.01575965	99.9264724	1360.0326
610	0.08498753	104.896479	0	0.08190731	0.08811881	2238053.04	0	0.01575565	99.9264539	1359.69179
611	0.08493866	104.849355	0	0.08185877	0.08806962	2238116.13	0	0.01576058	99.9264735	1360.05343
612	0.08498752	104.896273	0	0.08190729	0.08811879	2238012.08	0	0.01575568	99.9264529	1359.67332
613	0.08498933	104.901911	0	0.08190921	0.0881205	2238121.93	0	0.01575459	99.926451	1359.63788
614	0.08495409	104.852806	0	0.08187376	0.08808549	2238130.55	0	0.01576247	99.926272	1356.33657
615	0.08495092	104.851857	0	0.08187068	0.08808224	2238148.58	0	0.01576225	99.926485	1360.26708
616	0.08494178	104.849389	0	0.08186178	0.08807285	2238134.81	0	0.01576119	99.9264781	1360.13843
617	0.08493467	104.851494	0	0.08185499	0.08806543	2238140.79	0	0.01575938	99.9264731	1360.0472
618	0.08492763	104.853755	0	0.08184826	0.08805807	2238126.5	0	0.01575725	99.9264628	1359.8557
619	0.08487467	104.938917	0	0.08179962	0.08800068	2237874.6	0	0.01572094	99.9262927	1356.71786
620	0.08494977	104.851472	0	0.08186956	0.08808106	2238148.32	0	0.01576215	99.9264849	1360.26419
621	0.08498882	104.898982	0	0.08190863	0.08812006	2238013.11	0	0.01575511	99.9264506	1359.6295
622	0.08492423	104.857081	0	0.08184508	0.08805445	2238134.43	0	0.01575576	99.9264565	1359.73968
623	0.08498137	104.884637	0	0.08190102	0.08811278	2238043.85	0	0.01575805	99.9264635	1359.86827
624	0.08494972	104.853331	0	0.08186956	0.08808095	2238148.6	0	0.01576186	99.926485	1360.26657
625	0.08493949	104.850393	0	0.0818596	0.08807045	2238143.37	0	0.01576059	99.9264785	1360.14721
626	0.08496408	104.86458	0	0.08188375	0.08809549	2238147.21	0	0.0157614	99.9264831	1360.23059
627	0.08494306	104.849348	0	0.08186302	0.08807418	2238131	0	0.01576143	99.9264763	1360.10626
628	0.08496254	104.859469	0	0.08188212	0.08809405	2238136.43	0	0.01576212	99.9264826	1360.22233
629	0.08498606	104.894063	0	0.08190582	0.08811735	2238103.03	0	0.01575618	99.9264577	1359.76166
630	0.08494523	104.850432	0	0.08186515	0.08807639	2238146.48	0	0.01576164	99.926483	1360.22955
631	0.08494082	104.852038	0	0.08186094	0.08807178	2238146.72	0	0.0157606	99.9264792	1360.1594
632	0.08495643	104.855293	0	0.0818761	0.08808785	2238148.07	0	0.01576232	99.9264863	1360.29071
633	0.0849526	104.852458	0	0.08187231	0.08808396	2238148.72	0	0.01576236	99.9264846	1360.25954
634	0.084931	104.852432	0	0.08185147	0.0880616	2238135.16	0	0.01575834	99.9264681	1359.95482
635	0.08495245	104.852676	0	0.08187218	0.0880838	2238148.73	0	0.01576231	99.9264857	1360.27872
636	0.08497023	104.868366	0	0.0818898	0.08810174	2238132.33	0	0.01576095	99.9264796	1360.16591
637	0.08493779	104.849514	0	0.08185794	0.08806872	2238120.09	0	0.01576038	99.9264739	1360.06108
638	0.08496306	104.860792	0	0.08188266	0.08809454	2238144.62	0	0.01576194	99.9264847	1360.26095
639	0.08499669	104.916575	0	0.08191674	0.08812768	2237791.85	0	0.01575122	99.9264296	1359.24316
640	0.08496353	104.864096	0	0.08188321	0.08809493	2238147.37	0	0.01576145	99.9264833	1360.23505

© 2024 Zhang X et al. *JAMA Pediatrics*.

641	0.08499649	104.916156	0	0.08191653	0.08812749	2237867.67	0	0.01575132	99.9264314	1359.27563
642	0.08497791	104.883057	0	0.08189764	0.08810925	2238142.16	0	0.0157585	99.9264696	1359.98114
643	0.08494532	104.849789	0	0.08186521	0.0880765	2238142.09	0	0.01576175	99.9264804	1360.18064
644	0.08496102	104.857753	0	0.0818806	0.08809252	2237500.55	0	0.0157623	99.9261838	1354.71596
645	0.08497362	104.872559	0	0.08189319	0.08810512	2238110.81	0	0.01576027	99.9264753	1360.08626
646	0.08498887	104.90619	0	0.08190888	0.08811989	2238140.05	0	0.01575398	99.9264486	1359.59373
647	0.08498243	104.887283	0	0.08190212	0.0881138	2238113.36	0	0.01575756	99.9264642	1359.8813
648	0.08499402	104.910226	0	0.08191397	0.08812511	2237835.55	0	0.01575264	99.9264363	1359.36674
649	0.08496323	104.860975	0	0.08188283	0.08809472	2238144.55	0	0.01576192	99.9264846	1360.25958
650	0.08495629	104.856463	0	0.08187599	0.08808766	2238148.45	0	0.01576213	99.9264862	1360.28829
651	0.08497781	104.880175	0	0.08189746	0.08810923	2238133.23	0	0.01575895	99.9264712	1360.01117
652	0.08492539	104.857089	0	0.08184619	0.08805565	2238138.57	0	0.0157561	99.9264582	1359.77167
653	0.08491846	104.861313	0	0.08183962	0.08804834	2238124.19	0	0.01575329	99.9264449	1359.52421
654	0.08496105	104.857798	0	0.08188063	0.08809255	2238009.13	0	0.01576229	99.9264224	1359.1102
655	0.08496306	104.860792	0	0.08188266	0.08809454	2238144.62	0	0.01576194	99.9264847	1360.26095
656	0.08495621	104.857529	0	0.08187594	0.08808754	2238148.55	0	0.01576196	99.9264856	1360.2773
657	0.08494041	104.849205	0	0.08186046	0.08807144	2238114.94	0	0.01576096	99.926473	1360.04444
658	0.08496623	104.864388	0	0.08188582	0.08809772	2238143.4	0	0.01576151	99.926483	1360.22898
659	0.08494571	104.853486	0	0.08186569	0.08807679	2238148.19	0	0.01576124	99.9264823	1360.21674
660	0.08496186	104.858842	0	0.08188144	0.08809335	2238138.04	0	0.01576218	99.9264829	1360.22745
661	0.08494334	104.849437	0	0.0818633	0.08807447	2238135.8	0	0.01576147	99.9264781	1360.1396
662	0.0849546	104.853271	0	0.08187427	0.08808601	2238147.54	0	0.01576246	99.9264799	1360.17196
663	0.08494661	104.850891	0	0.08186649	0.08807781	2238147.45	0	0.01576179	99.9264839	1360.24637
664	0.08494623	104.85044	0	0.08186611	0.08807742	2238146.63	0	0.0157618	99.9264833	1360.2359
665	0.08491301	104.866675	0	0.08183452	0.08804254	2238111.35	0	0.01575056	99.926432	1359.28673
666	0.08494	104.849409	0	0.08186006	0.088071	2238131.47	0	0.01576085	99.9264769	1360.11603
667	0.08500083	104.928302	0	0.08192107	0.08813161	2238079.1	0	0.01574862	99.9264228	1359.11651
668	0.08499189	104.906058	0	0.0819118	0.08812303	2238072.48	0	0.01575361	99.9264454	1359.53435
669	0.08491063	104.869972	0	0.08183232	0.08803998	2238115.16	0	0.01574916	99.9264257	1359.16936
670	0.08499406	104.910349	0	0.08191402	0.08812515	2237865.45	0	0.01575262	99.9264368	1359.37581
671	0.08497935	104.881183	0	0.08189897	0.0881108	2238054.9	0	0.01575872	99.9264664	1359.92292
672	0.08497054	104.867955	0	0.08189008	0.08810206	2237838.79	0	0.01576102	99.9264487	1359.59575
673	0.08495601	104.855352	0	0.08187569	0.0880874	2238148.33	0	0.01576227	99.9264864	1360.29289
674	0.08497603	104.875739	0	0.08189561	0.08810752	2238004.04	0	0.01575971	99.9264665	1359.92512
675	0.0849896	104.900431	0	0.08190942	0.08812083	2237895.65	0	0.01575479	99.9264463	1359.55004
676	0.084961	104.858252	0	0.08188059	0.08809248	2238142.7	0	0.01576222	99.9264844	1360.25539
677	0.08497752	104.878825	0	0.08189714	0.08810897	2238119.62	0	0.01575918	99.9264715	1360.01612
678	0.08495972	104.856745	0	0.08187932	0.08809121	2238119.83	0	0.01576237	99.9264691	1359.9728
679	0.08499149	104.904693	0	0.08191138	0.08812266	2237985.37	0	0.01575387	99.9264446	1359.5202
680	0.08496969	104.868193	0	0.08188927	0.08810118	2238139.08	0	0.01576098	99.9264803	1360.18047
681	0.08497572	104.875111	0	0.08189529	0.08810722	2232464.38	0	0.01575982	99.9261128	1353.41363
682	0.08497274	104.871017	0	0.08189229	0.08810425	2238057.13	0	0.01576053	99.9264716	1360.01946

© 2024 Zhang X et al. *JAMA Pediatrics*.

683	0.0849731	104.872144	0	0.08189267	0.08810459	2238126.32	0	0.01576035	99.9264767	1360.11324
684	0.08496706	104.86401	0	0.08188661	0.08809858	2238114.62	0	0.01576159	99.9264788	1360.15116
685	0.08496581	104.862433	0	0.08188536	0.08809734	2238020.41	0	0.0157618	99.9264603	1359.81039
686	0.08493481	104.85065	0	0.08185509	0.0880656	2238134.16	0	0.01575954	99.9264731	1360.04672
687	0.08498935	104.900177	0	0.08190917	0.08812057	2238032.88	0	0.01575486	99.92645	1359.61911
688	0.08499497	104.912424	0	0.08191495	0.08812602	2237795.56	0	0.01575215	99.9264336	1359.31532
689	0.08499057	104.902543	0	0.08191042	0.08812177	2237911.39	0	0.01575433	99.9264448	1359.5238
690	0.08496395	104.8609	0	0.08188352	0.08809546	2238135.74	0	0.01576197	99.9264827	1360.22345
691	0.08497316	104.872233	0	0.08189274	0.08810466	2238126.14	0	0.01576033	99.9264766	1360.11189
692	0.0849537	104.852742	0	0.08187338	0.08808509	2238148.26	0	0.01576244	99.9264789	1360.15459
693	0.08493303	104.850378	0	0.08185337	0.08806376	2238023.43	0	0.01575916	99.9264611	1359.82402
694	0.08497918	104.880764	0	0.0818988	0.08811063	2237991.31	0	0.01575879	99.9264636	1359.87113
695	0.08499637	104.916261	0	0.08191642	0.08812736	2238029.41	0	0.01575132	99.9264343	1359.32911
696	0.08500207	104.931282	0	0.08192236	0.08813281	2238056.03	0	0.0157479	99.9264192	1359.04946
697	0.0849933	104.908435	0	0.08191322	0.08812441	2237472.4	0	0.01575303	99.9264304	1359.25692
698	0.0849814	104.884603	0	0.08190105	0.08811281	2238014.06	0	0.01575805	99.9264623	1359.84588
699	0.08494941	104.850903	0	0.0818692	0.08808071	2238147.41	0	0.01576219	99.9264819	1360.20835
700	0.0849684	104.865666	0	0.08188795	0.08809992	2238119.83	0	0.01576136	99.9264793	1360.16092
701	0.08497715	104.877826	0	0.08189675	0.08810862	2238098.25	0	0.01575935	99.926471	1360.0075
702	0.08498161	104.886599	0	0.08190131	0.08811297	2238128.67	0	0.01575773	99.9264655	1359.90586
703	0.08496397	104.86261	0	0.08188359	0.08809543	2238145.99	0	0.0157617	99.9264841	1360.25046
704	0.08494586	104.850664	0	0.08186576	0.08807703	2238147.03	0	0.0157617	99.9264835	1360.23795
705	0.08494025	104.850646	0	0.08186034	0.08807123	2238144.77	0	0.0157607	99.9264793	1360.16038
706	0.08499074	104.903057	0	0.0819106	0.08812193	2237996.46	0	0.01575423	99.9264464	1359.55307
707	0.08501358	104.964855	0	0.08193445	0.08814372	2237950.96	0	0.01573981	99.9263808	1358.34199
708	0.08496572	104.863003	0	0.08188528	0.08809723	2238138.26	0	0.01576171	99.9264828	1360.22593
709	0.08495482	104.853558	0	0.0818745	0.08808623	2238147.96	0	0.01576243	99.9264837	1360.24313
710	0.0849675	104.864959	0	0.08188707	0.08809902	2238134.95	0	0.01576145	99.9264816	1360.20299
711	0.08500466	104.937569	0	0.08192504	0.0881353	2237833.49	0	0.01574635	99.9264093	1358.86671
712	0.08497628	104.876887	0	0.08189588	0.08810774	2238122.27	0	0.01575953	99.9264731	1360.0463
713	0.0849678	104.867432	0	0.08188742	0.08809925	2238145.05	0	0.01576107	99.9264814	1360.19948
714	0.08495074	104.851601	0	0.0818705	0.08808206	2238148.48	0	0.01576226	99.9264842	1360.25133
715	0.0849705	104.868428	0	0.08189006	0.08810201	2238123.84	0	0.01576094	99.9264787	1360.14932
716	0.08500815	104.948157	0	0.08192872	0.0881386	2237996.09	0	0.01574387	99.9264	1358.69484
717	0.08497703	104.878093	0	0.08189664	0.08810849	2238121.81	0	0.01575931	99.9264722	1360.0289
718	0.08498629	104.8958	0	0.0819061	0.08811754	2238126.88	0	0.01575589	99.9264571	1359.74997
719	0.08492631	104.853734	0	0.08184698	0.0880567	2238077.09	0	0.01575688	99.9264586	1359.77823
720	0.08502173	104.992165	0	0.0819431	0.08815133	2237669.39	0	0.01573297	99.9263473	1357.72337
721	0.08497748	104.877855	0	0.08189707	0.08810896	2237511.4	0	0.01575933	99.9264384	1359.40558
722	0.084932	104.855114	0	0.08185251	0.08806255	2238144.47	0	0.01575818	99.9264681	1359.95386
723	0.08494044	104.8492	0	0.08186048	0.08807147	2238114.23	0	0.01576096	99.9264728	1360.04098
724	0.08496682	104.863671	0	0.08188637	0.08809835	2238102.86	0	0.01576164	99.9264768	1360.11522

© 2024 Zhang X et al. *JAMA Pediatrics*.

725	0.08498842	104.897904	0	0.08190821	0.08811968	2237840.93	0	0.01575532	99.9264469	1359.56131
726	0.08497352	104.873825	0	0.08189313	0.08810498	2238138.5	0	0.01576008	99.9264765	1360.10908
727	0.0849722	104.872084	0	0.08189181	0.08810367	2238140.08	0	0.01576037	99.9264779	1360.1346
728	0.08500625	104.942397	0	0.08192671	0.0881368	2237967.21	0	0.01574523	99.9264059	1358.80376
729	0.08500712	104.944262	0	0.08192761	0.08813765	2234381.33	0	0.01574473	99.9263623	1357.99946
730	0.08496944	104.867331	0	0.081889	0.08810095	2238132.89	0	0.01576111	99.9264802	1360.17744
731	0.08498712	104.895113	0	0.08190688	0.08811842	2235535.71	0	0.0157559	99.9263825	1358.37283
732	0.08498617	104.893235	0	0.0819059	0.08811749	2236918.58	0	0.0157563	99.9264221	1359.10434
733	0.0850023	104.932682	0	0.08192262	0.08813301	2238091.43	0	0.01574763	99.9264184	1359.0357
734	0.08497999	104.882123	0	0.08189961	0.08811142	2237996.53	0	0.01575853	99.9264631	1359.86143
735	0.08499595	104.914697	0	0.08191597	0.08812697	2237605.25	0	0.01575164	99.926428	1359.2122
736	0.08497119	104.869861	0	0.08189076	0.08810268	2238134.98	0	0.01576072	99.9264789	1360.15402
737	0.08499732	104.918224	0	0.0819174	0.08812829	2237887.35	0	0.01575085	99.9264297	1359.24503
738	0.08498117	104.88445	0	0.08190082	0.08811257	2238079.87	0	0.01575809	99.9264651	1359.89891
739	0.08497194	104.870294	0	0.0818915	0.08810345	2238119.18	0	0.01576065	99.9264772	1360.12297
740	0.08498819	104.898916	0	0.08190802	0.08811941	2238114.66	0	0.01575519	99.9264536	1359.68543
741	0.08501059	104.956649	0	0.08193132	0.08814087	2238073.58	0	0.01574192	99.9263918	1358.54337
742	0.08491349	104.865216	0	0.08183494	0.08804308	2238074.49	0	0.01575096	99.926433	1359.30453
743	0.08497078	104.869533	0	0.08189036	0.08810228	2238137.47	0	0.01576077	99.9264794	1360.16214
744	0.08496827	104.865975	0	0.08188783	0.08809978	2238135.84	0	0.01576131	99.9264812	1360.19655
745	0.08499164	104.904625	0	0.08191151	0.08812281	2235182.49	0	0.01575387	99.9263815	1358.35344
746	0.08498673	104.894355	0	0.08190648	0.08811804	2237026.48	0	0.01575606	99.9264255	1359.16576
747	0.08499044	104.902039	0	0.08191028	0.08812165	2236984.23	0	0.01575443	99.926423	1359.12017
748	0.08499998	104.924678	0	0.08192015	0.08813084	2237004.08	0	0.01574935	99.9264097	1358.87545
749	0.08494713	104.85066	0	0.08186699	0.08807835	2238147.19	0	0.0157619	99.9264838	1360.24471
750	0.08497011	104.867648	0	0.08188966	0.08810163	2238100.11	0	0.01576106	99.9264765	1360.10856
751	0.08489281	104.897655	0	0.08181593	0.0880207	2238050.24	0	0.01573714	99.9263692	1358.12708
752	0.08498417	104.889916	0	0.08190388	0.08811552	2238068.86	0	0.015757	99.9264602	1359.80779
753	0.08496687	104.863963	0	0.08188642	0.08809839	2238128.01	0	0.01576159	99.9264809	1360.1901
754	0.08494003	104.849872	0	0.08186011	0.08807102	2238140.98	0	0.01576078	99.9264788	1360.15117
755	0.08493252	104.850805	0	0.08185289	0.08806322	2238105.7	0	0.01575897	99.9264682	1359.95671
756	0.08493084	104.851865	0	0.0818513	0.08806145	2238124.61	0	0.01575839	99.9264676	1359.9441
757	0.08494359	104.84988	0	0.08186355	0.08807471	2238143.9	0	0.01576144	99.9264814	1360.20063
758	0.08497643	104.876265	0	0.08189601	0.08810792	2237858.37	0	0.01575962	99.9264576	1359.75954
759	0.08496738	104.864599	0	0.08188694	0.0880989	2238129.22	0	0.0157615	99.9264809	1360.19039
760	0.08494446	104.85097	0	0.08186442	0.08807558	2238147.06	0	0.01576142	99.9264826	1360.22275
761	0.08497162	104.8701	0	0.08189119	0.08810313	2238128.25	0	0.01576068	99.9264782	1360.14
762	0.0849723	104.873216	0	0.08189193	0.08810373	2238142.83	0	0.01576019	99.9264773	1360.12401
763	0.08494424	104.850783	0	0.0818642	0.08807535	2238146.79	0	0.01576142	99.9264825	1360.2203
764	0.08499772	104.919563	0	0.08191782	0.08812866	2238018.46	0	0.01575057	99.9264307	1359.26324
765	0.08498966	104.903049	0	0.08190956	0.08812081	2238125.69	0	0.01575437	99.9264501	1359.62102
766	0.08496054	104.857919	0	0.08188014	0.08809201	2238143.87	0	0.01576224	99.9264848	1360.26259

© 2024 Zhang X et al. *JAMA Pediatrics*.

767	0.0850175	104.983367	0	0.08193876	0.08814721	2238116.04	0	0.01573573	99.9263634	1358.01965
768	0.08490479	104.882295	0	0.08182705	0.08803356	2238132.6	0	0.01574493	99.9264064	1358.81332
769	0.08496652	104.86394	0	0.08188609	0.08809803	2238137.92	0	0.01576159	99.9264824	1360.21861
770	0.08499394	104.910928	0	0.08191392	0.08812501	2238080.58	0	0.01575255	99.9264408	1359.44893
771	0.08498461	104.891975	0	0.08190436	0.08811591	2238123.11	0	0.01575665	99.9264604	1359.81184
772	0.08496073	104.858043	0	0.08188032	0.08809221	2238143.29	0	0.01576223	99.9264846	1360.25877
773	0.08500557	104.940383	0	0.081926	0.08813616	2237947.94	0	0.01574571	99.9264078	1358.83916
774	0.08497273	104.871474	0	0.0818923	0.08810423	2238122.05	0	0.01576046	99.9264768	1360.11474
775	0.08494797	104.85109	0	0.08186781	0.08807921	2238147.85	0	0.01576196	99.9264845	1360.25691
776	0.0849864	104.894112	0	0.08190615	0.0881177	2238041.1	0	0.01575614	99.9264557	1359.72404
777	0.08498021	104.882626	0	0.08189984	0.08811164	2238052.28	0	0.01575844	99.9264653	1359.90203
778	0.08494948	104.851756	0	0.08186929	0.08808076	2238148.4	0	0.01576207	99.9264852	1360.27057
779	0.08489967	104.883877	0	0.08182214	0.08802821	2237446.35	0	0.01574247	99.9263859	1358.43624
780	0.08496677	104.864961	0	0.08188636	0.08809826	2238142.89	0	0.01576143	99.9264826	1360.22232
781	0.08497419	104.873427	0	0.08189377	0.08810569	2238112.6	0	0.01576013	99.9264749	1360.07885
782	0.08497755	104.878337	0	0.08189715	0.08810901	2238083.87	0	0.01575925	99.9264698	1359.98615
783	0.08497392	104.875157	0	0.08189355	0.08810535	2238141.06	0	0.01575987	99.9264757	1360.09469
784	0.08498407	104.889941	0	0.08190378	0.08811541	2238092.4	0	0.01575701	99.926461	1359.82305
785	0.08498459	104.892862	0	0.08190436	0.08811586	2238131.59	0	0.01575651	99.9264601	1359.80535
786	0.08490827	104.871579	0	0.08183009	0.08803748	2238048.27	0	0.01574799	99.926419	1359.04719
787	0.0849654	104.861953	0	0.08188495	0.08809693	2237615.17	0	0.01576186	99.9263753	1358.23948
788	0.08493494	104.851473	0	0.08185525	0.08806571	2238141.22	0	0.01575945	99.9264735	1360.05327
789	0.08489825	104.888242	0	0.08182091	0.08802661	2238089.72	0	0.01574116	99.9263883	1358.47898
790	0.08497517	104.874285	0	0.08189473	0.08810667	2235323.7	0	0.01575997	99.9262868	1356.60974
791	0.0849595	104.856978	0	0.08187911	0.08809097	2238144.3	0	0.01576232	99.9264846	1360.25977
792	0.08497353	104.872017	0	0.08189309	0.08810504	2237951.89	0	0.01576036	99.9264632	1359.86323
793	0.08490916	104.874687	0	0.08183104	0.08803832	2238131.92	0	0.01574787	99.92642	1359.06506
794	0.08489432	104.89851	0	0.08181741	0.08802223	2238119.46	0	0.01573774	99.9263727	1358.19161
795	0.08498948	104.900344	0	0.08190931	0.08812071	2237997.59	0	0.01575482	99.9264489	1359.59933
796	0.08491096	104.874205	0	0.08183276	0.08804019	2238136.77	0	0.01574863	99.9264237	1359.13244
797	0.08496667	104.867033	0	0.08188632	0.08809809	2238146.29	0	0.01576111	99.9264817	1360.20494
798	0.08486116	104.985146	0	0.08178792	0.0879853	2238097.03	0	0.01570525	99.9262206	1355.39165
799	0.084941	104.849365	0	0.08186103	0.08807204	2238132.59	0	0.01576105	99.9264774	1360.12554
800	0.08492842	104.853172	0	0.081849	0.08805891	2238124.54	0	0.01575755	99.926464	1359.87823
801	0.0849077	104.872304	0	0.08182956	0.08803687	2238040.18	0	0.01574765	99.9264174	1359.01617
802	0.08490617	104.875068	0	0.08182817	0.08803521	2238085.28	0	0.01574661	99.9264134	1358.94288
803	0.08500495	104.93835	0	0.08192534	0.08813557	2237805.29	0	0.01574617	99.9264081	1358.84485
804	0.08495032	104.85174	0	0.08187009	0.08808162	2238148.49	0	0.01576219	99.9264852	1360.26952
805	0.08489302	104.897787	0	0.08181613	0.0880209	2238075.22	0	0.01573722	99.9263698	1358.13844
806	0.08496933	104.867528	0	0.0818889	0.08810083	2238137.66	0	0.01576108	99.9264806	1360.18504
807	0.08497433	104.873751	0	0.08189391	0.08810581	2238118.94	0	0.01576007	99.9264751	1360.08337
808	0.08495555	104.855819	0	0.08187526	0.08808691	2238148.53	0	0.01576216	99.9264863	1360.28986

© 2024 Zhang X et al. *JAMA Pediatrics*.

809	0.08495144	104.851519	0	0.08187117	0.08808279	2238148.19	0	0.01576237	99.9264656	1359.90741
810	0.08495971	104.85831	0	0.08187935	0.08809115	2238147.34	0	0.01576212	99.9264859	1360.28314
811	0.08497351	104.872318	0	0.08189307	0.08810501	2238103.81	0	0.01576031	99.9264749	1360.07894
812	0.08498582	104.892921	0	0.08190555	0.08811714	2238031.11	0	0.01575638	99.9264564	1359.73725
813	0.08498577	104.8927	0	0.0819055	0.08811709	2237979.55	0	0.01575642	99.9264549	1359.71064
814	0.08493953	104.849118	0	0.08185961	0.08807053	2238040.89	0	0.0157608	99.9264574	1359.75535
815	0.0849634	104.860343	0	0.08188297	0.0880949	2238136.53	0	0.01576203	99.9264828	1360.2264
816	0.08497274	104.870956	0	0.0818923	0.08810426	2238011.15	0	0.01576054	99.9264678	1359.94852
817	0.08499078	104.902763	0	0.08191063	0.08812198	2236584.53	0	0.01575427	99.9264133	1358.94181
818	0.08494895	104.852511	0	0.08186879	0.08808018	2238148.47	0	0.01576188	99.9264849	1360.26551
819	0.0849868	104.894442	0	0.08190655	0.08811811	2185978.05	0	0.01575604	99.9249525	1332.48983
820	0.08498603	104.892924	0	0.08190576	0.08811736	2186138.03	0	0.01575636	99.9248901	1331.38153
821	0.08497755	104.878316	0	0.08189715	0.08810902	2238078.43	0	0.01575925	99.9264696	1359.98088
822	0.08492925	104.85507	0	0.08184986	0.08805971	2238141.5	0	0.01575748	99.9264647	1359.89128
823	0.08496391	104.861348	0	0.08188349	0.0880954	2238142.6	0	0.01576189	99.9264841	1360.25037
824	0.08494035	104.850641	0	0.08186044	0.08807133	2238144.83	0	0.01576072	99.9264793	1360.16208
825	0.08497301	104.871906	0	0.08189258	0.0881045	2238123.04	0	0.01576039	99.9264766	1360.11126
826	0.08495627	104.854835	0	0.08187592	0.08808769	2238147.82	0	0.01576238	99.9264859	1360.28248
827	0.08497707	104.878278	0	0.08189668	0.08810852	2238124.89	0	0.01575928	99.9264722	1360.02965
828	0.08500187	104.930995	0	0.08192216	0.08813261	2238072.07	0	0.01574798	99.9264198	1359.06084
829	0.08489982	104.883519	0	0.08182229	0.08802838	2236422.05	0	0.01574259	99.926373	1358.1981
830	0.08497003	104.868109	0	0.08188959	0.08810153	2238132.81	0	0.01576099	99.9264798	1360.16948
831	0.08498264	104.887112	0	0.08190232	0.08811402	2238077.88	0	0.01575757	99.9264629	1359.85752
832	0.08497223	104.870277	0	0.08189178	0.08810375	2238034.19	0	0.01576065	99.9264698	1359.98472
833	0.08494087	104.849732	0	0.08186091	0.0880719	2238140.76	0	0.01576097	99.9264793	1360.16102
834	0.08496357	104.86038	0	0.08188313	0.08809508	2238130.67	0	0.01576203	99.9264813	1360.19821
835	0.08493944	104.850329	0	0.08185956	0.0880704	2238143.06	0	0.01576059	99.9264785	1360.14618
836	0.08497067	104.868416	0	0.08189023	0.08810219	2238105.35	0	0.01576095	99.9264768	1360.11461
837	0.08500751	104.946307	0	0.08192805	0.08813799	2238005.45	0	0.01574432	99.9264021	1358.73465
838	0.08492884	104.855042	0	0.08184946	0.08805928	2238140.73	0	0.01575738	99.9264642	1359.88149
839	0.08492685	104.854114	0	0.08184751	0.08805725	2238123.48	0	0.01575697	99.9264614	1359.83057
840	0.0849854	104.893451	0	0.08190517	0.08811669	2238121.05	0	0.01575634	99.926459	1359.78508
841	0.08495352	104.852638	0	0.08187321	0.08808491	2238148.36	0	0.01576244	99.9264784	1360.14485
842	0.08476372	105.466818	0	0.08170779	0.08787005	2237068.74	0	0.01553985	99.9254349	1341.11056
843	0.0849749	104.874613	0	0.08189448	0.08810638	2238118.8	0	0.01575993	99.9264745	1360.07238
844	0.08494115	104.849091	0	0.08186117	0.08807221	2238062.09	0	0.01576112	99.9264577	1359.76105
845	0.08496033	104.858164	0	0.08187994	0.08809179	2238146.03	0	0.01576219	99.9264856	1360.2779
846	0.08497389	104.872833	0	0.08189346	0.08810539	2238099.89	0	0.01576023	99.9264743	1360.0682
847	0.08495193	104.852225	0	0.08187166	0.08808327	2238148.71	0	0.01576232	99.926485	1360.26609
848	0.08496663	104.863369	0	0.08188618	0.08809816	2238064.28	0	0.01576168	99.9264701	1359.99178
849	0.08495409	104.852958	0	0.08187377	0.08808549	2238147.91	0	0.01576245	99.9264786	1360.14912
850	0.08492396	104.857892	0	0.08184484	0.08805415	2238136.97	0	0.01575555	99.9264557	1359.72424

© 2024 Zhang X et al. *JAMA Pediatrics*.

851	0.08499033	104.90262	0	0.0819102	0.08812152	2238075.39	0	0.01575435	99.9264488	1359.59731
852	0.08497193	104.869825	0	0.08189148	0.08810345	2237976.04	0	0.01576072	99.9264645	1359.8867
853	0.08499454	104.911898	0	0.08191453	0.0881256	2238036.98	0	0.0157523	99.9264388	1359.41281
854	0.08497721	104.877759	0	0.0818968	0.08810868	2238078.52	0	0.01575935	99.9264699	1359.98774
855	0.08488531	104.912637	0	0.08180913	0.08801248	2237903.15	0	0.015731	99.9263395	1357.57916
856	0.08493494	104.85076	0	0.08185522	0.08806573	2238136.29	0	0.01575956	99.9264734	1360.05203
857	0.08499996	104.925426	0	0.08192015	0.0881308	2238036.48	0	0.01574924	99.926425	1359.15722
858	0.08496568	104.864517	0	0.08188529	0.08809714	2238145.35	0	0.01576147	99.9264831	1360.23142
859	0.084958	104.855707	0	0.08187763	0.08808946	2238145.06	0	0.0157624	99.926484	1360.24784
860	0.08495949	104.856951	0	0.0818791	0.08809096	2238144.16	0	0.01576232	99.9264845	1360.25798
861	0.084973	104.87198	0	0.08189257	0.08810449	2238125.82	0	0.01576038	99.9264768	1360.11448
862	0.08497048	104.868577	0	0.08189005	0.08810199	2238129.51	0	0.01576092	99.9264792	1360.15845
863	0.0849292	104.852015	0	0.08184972	0.08805975	2238052.31	0	0.01575794	99.9264609	1359.82075
864	0.08493817	104.850926	0	0.08185834	0.08806906	2238143.75	0	0.01576024	99.9264772	1360.12174
865	0.08493802	104.854444	0	0.08185831	0.08806881	2238146.86	0	0.01575967	99.9264751	1360.08271
866	0.0849868	104.894442	0	0.08190655	0.08811811	2185978.05	0	0.01575604	99.9249525	1332.48983
867	0.08498603	104.892923	0	0.08190576	0.08811735	2193017.8	0	0.01575636	99.9250941	1335.00872
868	0.0849868	104.894443	0	0.08190655	0.08811811	2180229.85	0	0.01575604	99.9247901	1329.61272
869	0.08496511	104.86277	0	0.08188469	0.08809661	2238142.57	0	0.01576172	99.9264836	1360.24081
870	0.08490135	104.882361	0	0.08182372	0.08803	2238071.01	0	0.01574345	99.9263986	1358.66986
871	0.08499507	104.912454	0	0.08191505	0.08812612	2233417.53	0	0.01575213	99.9263502	1357.77651
872	0.08489989	104.883531	0	0.08182235	0.08802845	2237526.42	0	0.01574262	99.9263876	1358.46734
873	0.08491394	104.867185	0	0.08183543	0.08804349	2238129.07	0	0.01575082	99.9264336	1359.31635
874	0.08495574	104.855162	0	0.08187543	0.08808713	2238148.38	0	0.01576228	99.9264864	1360.29295
875	0.08490816	104.871138	0	0.08182997	0.08803738	2237782.36	0	0.01574802	99.9264142	1358.95874
876	0.084952	104.852061	0	0.08187173	0.08808336	2238148.71	0	0.01576235	99.9264836	1360.23996
877	0.08492659	104.856458	0	0.08184733	0.08805691	2238139.7	0	0.01575654	99.9264603	1359.80983
878	0.08491643	104.863514	0	0.08183773	0.08804618	2238124.14	0	0.01575226	99.9264401	1359.43717
879	0.08500606	104.941269	0	0.0819265	0.08813664	2236936.33	0	0.01574545	99.9263945	1358.59422
880	0.08499307	104.907948	0	0.08191299	0.08812419	2237612.61	0	0.01575314	99.9264337	1359.31779
881	0.08498172	104.885058	0	0.08190137	0.08811313	2237916.88	0	0.01575796	99.926458	1359.76722
882	0.08496767	104.865372	0	0.08188724	0.08809918	2238137.9	0	0.01576139	99.9264818	1360.20682
883	0.08495566	104.85384	0	0.08187531	0.08808708	2238144.14	0	0.01576247	99.9264735	1360.05313
884	0.08496271	104.859468	0	0.08188227	0.08809421	2238126.66	0	0.01576213	99.9264796	1360.16599
885	0.08499819	104.921944	0	0.08191835	0.08812908	2238098.99	0	0.01575011	99.92643	1359.24924
886	0.08496625	104.864785	0	0.08188585	0.08809772	2238144.41	0	0.01576145	99.9264829	1360.22718
887	0.08496424	104.861614	0	0.08188382	0.08809574	2238141.79	0	0.01576187	99.9264839	1360.24591
888	0.08496653	104.863258	0	0.08188607	0.08809806	2238069.76	0	0.01576169	99.926471	1360.00673
889	0.08496093	104.857916	0	0.08188052	0.08809242	2238136.01	0	0.01576227	99.9264814	1360.19927
890	0.08496671	104.863393	0	0.08188625	0.08809824	2237949.1	0	0.01576168	99.9264506	1359.63063
891	0.08496158	104.860423	0	0.08188122	0.08809302	2238147.05	0	0.01576192	99.9264852	1360.26945
892	0.08497082	104.868378	0	0.08189037	0.08810235	2238009.73	0	0.01576095	99.926467	1359.93424

© 2024 Zhang X et al. *JAMA Pediatrics*.

893	0.0849548	104.855689	0	0.08187453	0.08808614	2238148.63	0	0.0157621	99.9264861	1360.2868
894	0.08499454	104.911947	0	0.08191453	0.08812559	2238044.21	0	0.01575229	99.9264389	1359.41478
895	0.0849698	104.869676	0	0.08188941	0.08810125	2238143.77	0	0.01576075	99.9264798	1360.17098
896	0.08499661	104.91668	0	0.08191666	0.08812759	2237991.69	0	0.01575121	99.9264332	1359.308
897	0.0849662	104.8629	0	0.08188575	0.08809773	2238071.25	0	0.01576174	99.9264708	1360.00358
898	0.08492716	104.856698	0	0.08184789	0.08805749	2238141.26	0	0.01575666	99.9264609	1359.82167
899	0.08498395	104.888968	0	0.08190364	0.08811533	2234314.97	0	0.01575717	99.9263299	1357.40197
900	0.08500064	104.92652	0	0.08192084	0.08813147	2237652.34	0	0.01574893	99.9264179	1359.02631
901	0.08496378	104.860304	0	0.08188334	0.08809531	2237392.43	0	0.01576205	99.9262828	1356.53579
902	0.0849958	104.914385	0	0.08191581	0.08812682	2237763.34	0	0.01575171	99.9264312	1359.27108
903	0.08489273	104.897192	0	0.08181583	0.08802062	2237980.8	0	0.01573717	99.9263686	1358.11642
904	0.0849879	104.896669	0	0.08190768	0.08811918	2212315.21	0	0.01575558	99.9257495	1346.79293
905	0.08494214	104.852413	0	0.08186222	0.08807313	2238147.29	0	0.01576079	99.9264801	1360.17664
906	0.08498081	104.88353	0	0.08190045	0.08811224	2237980.74	0	0.01575826	99.9264615	1359.83283
907	0.08490805	104.871408	0	0.08182987	0.08803726	2237915.34	0	0.01574793	99.9264163	1358.99727
908	0.0849582	104.857702	0	0.08187787	0.0880896	2238148.08	0	0.0157621	99.926486	1360.28558
909	0.08491935	104.858509	0	0.0818404	0.08804935	2235895	0	0.01575401	99.9263748	1358.23113
910	0.08496295	104.863108	0	0.08188262	0.08809436	2238147.37	0	0.01576158	99.9264838	1360.24502
911	0.0849358	104.856416	0	0.08185622	0.08806645	2238146.75	0	0.01575888	99.9264715	1360.01615
912	0.08490429	104.876597	0	0.0818264	0.08803322	2237707.35	0	0.0157456	99.9264028	1358.74772
913	0.08496406	104.860924	0	0.08188363	0.08809558	2238132.21	0	0.01576197	99.9264818	1360.20797
914	0.08495785	104.855817	0	0.08187748	0.08808929	2238146.58	0	0.01576237	99.9264854	1360.27394
915	0.0849299	104.852895	0	0.08185042	0.08806044	2238133.66	0	0.01575799	99.9264665	1359.92402
916	0.08496716	104.864027	0	0.08188671	0.08809869	2238092.69	0	0.01576159	99.9264753	1360.08755
917	0.08497208	104.869993	0	0.08189163	0.0881036	2237899.86	0	0.0157607	99.9264577	1359.76161
918	0.08495334	104.852542	0	0.08187304	0.08808473	2238148.47	0	0.01576243	99.9264784	1360.14406
919	0.08495807	104.855782	0	0.08187769	0.08808952	2238145.25	0	0.01576239	99.9264843	1360.2528
920	0.08488262	104.91899	0	0.08180671	0.0880095	2237927.98	0	0.01572856	99.9263284	1357.37513
921	0.0849288	104.85253	0	0.08184935	0.08805932	2238106.56	0	0.01575775	99.9264637	1359.87325
922	0.08490665	104.872996	0	0.08182857	0.08803577	2233942.3	0	0.01574713	99.9263442	1357.66676
923	0.0849941	104.910384	0	0.08191406	0.08812519	2237788.05	0	0.01575261	99.9264352	1359.34648
924	0.08494175	104.850459	0	0.08186179	0.08807279	2238145.26	0	0.01576102	99.9264806	1360.18521
925	0.08490365	104.880811	0	0.0818259	0.08803243	2238118.58	0	0.01574468	99.926405	1358.78779
926	0.08496013	104.865341	0	0.08187996	0.08809137	2238148.4	0	0.01576107	99.9264817	1360.20517
927	0.08497763	104.879455	0	0.08189726	0.08810907	2238128.77	0	0.01575907	99.9264715	1360.0168
928	0.08492367	104.855802	0	0.0818445	0.08805391	2238102.35	0	0.01575579	99.9264553	1359.71675
929	0.084938	104.849196	0	0.08185813	0.08806894	2237640.4	0	0.01576047	99.9263915	1358.53942
930	0.08492282	104.863802	0	0.08184391	0.08805279	2238143.35	0	0.0157543	99.9264501	1359.62183
931	0.08499679	104.916819	0	0.08191684	0.08812778	2237793.01	0	0.01575116	99.9264294	1359.2393
932	0.08493272	104.851645	0	0.08185311	0.0880634	2238135.92	0	0.01575889	99.9264706	1359.99999
933	0.08499442	104.912781	0	0.08191444	0.08812545	2238106.13	0	0.01575218	99.9264396	1359.42741
934	0.08498973	104.90204	0	0.08190959	0.08812091	2238109.4	0	0.01575452	99.9264504	1359.62637

© 2024 Zhang X et al. *JAMA Pediatrics*.

935	0.08497478	104.874176	0	0.08189436	0.08810627	2238102.1	0	0.01576	99.9264736	1360.05657
936	0.08495357	104.852792	0	0.08187326	0.08808496	2238148.51	0	0.01576242	99.9264828	1360.22639
937	0.08495636	104.854783	0	0.08187601	0.08808778	2238147.59	0	0.01576239	99.9264855	1360.27648
938	0.08498618	104.894013	0	0.08190594	0.08811748	2238086.05	0	0.01575618	99.9264572	1359.75186
939	0.08498741	104.896521	0	0.0819072	0.08811868	2238085.6	0	0.01575565	99.9264549	1359.70911
940	0.08498692	104.895395	0	0.08190669	0.0881182	2238076.16	0	0.01575588	99.9264556	1359.72281
941	0.08498738	104.896388	0	0.08190716	0.08811865	2238080.25	0	0.01575568	99.9264548	1359.70833
942	0.08500558	104.94052	0	0.08192601	0.08813616	2237978.59	0	0.01574568	99.926408	1358.84407
943	0.08499248	104.907261	0	0.0819124	0.0881236	2238059.28	0	0.01575334	99.9264439	1359.50663
944	0.08489543	104.892949	0	0.08181832	0.08802355	2238071.03	0	0.01573912	99.9263786	1358.30055
945	0.08494474	104.850333	0	0.08186467	0.08807589	2238146.13	0	0.01576157	99.9264827	1360.22354
946	0.08496321	104.86106	0	0.08188281	0.08809469	2238144.9	0	0.01576191	99.9264847	1360.26019
947	0.08498041	104.885764	0	0.08190013	0.08811176	2238137.42	0	0.01575794	99.9264668	1359.92999
948	0.0849652	104.862147	0	0.08188476	0.08809671	2238132.15	0	0.01576182	99.9264819	1360.20849
949	0.08494801	104.850716	0	0.08186784	0.08807926	2238147.3	0	0.01576202	99.9264837	1360.24276
950	0.08494833	104.850849	0	0.08186815	0.08807959	2238147.56	0	0.01576205	99.926484	1360.24761
951	0.08496105	104.85839	0	0.08188065	0.08809253	2238143.51	0	0.0157622	99.9264847	1360.26156
952	0.08496243	104.859604	0	0.08188202	0.08809393	2238141.46	0	0.01576209	99.9264841	1360.25067
953	0.08496503	104.862121	0	0.0818846	0.08809654	2238136.64	0	0.01576182	99.9264827	1360.22487
954	0.08495621	104.855506	0	0.08187589	0.08808761	2238148.29	0	0.01576227	99.9264864	1360.29279
955	0.08494592	104.850242	0	0.08186581	0.08807712	2238145.97	0	0.01576178	99.9264829	1360.22722
956	0.08499267	104.906921	0	0.08191257	0.08812381	2234265.09	0	0.01575336	99.9263629	1358.01116
957	0.08494891	104.852128	0	0.08186875	0.08808015	2238148.41	0	0.01576194	99.926485	1360.26723
958	0.08498257	104.886467	0	0.08190223	0.08811397	2237391.5	0	0.01575768	99.9264371	1359.38091
959	0.08497642	104.876362	0	0.081896	0.0881079	2238022.88	0	0.0157596	99.9264675	1359.94202
960	0.0849582	104.857702	0	0.08187787	0.0880896	2238148.08	0	0.0157621	99.926486	1360.28558
961	0.0849222	104.856858	0	0.08184311	0.08805235	2238095.66	0	0.01575518	99.9264523	1359.66268
962	0.08494775	104.850861	0	0.08186759	0.08807899	2238147.56	0	0.01576196	99.9264842	1360.25121
963	0.08497663	104.877198	0	0.08189623	0.0881081	2238112.68	0	0.01575947	99.9264723	1360.0313
964	0.08497216	104.870199	0	0.08189171	0.08810368	2238045.79	0	0.01576066	99.9264708	1360.00369
965	0.08500139	104.928778	0	0.08192163	0.08813218	2237931.37	0	0.01574843	99.9264198	1359.06134
966	0.08496387	104.860493	0	0.08188342	0.08809538	2238100.89	0	0.01576203	99.9264736	1360.05606
967	0.08497634	104.876098	0	0.08189592	0.08810783	2237682.67	0	0.01575965	99.9264469	1359.56197
968	0.08497863	104.880305	0	0.08189825	0.08811007	2238102.96	0	0.01575889	99.9264694	1359.97848
969	0.08499832	104.9204	0	0.08191842	0.08812926	2234735.53	0	0.01575033	99.9263758	1358.24888
970	0.08496439	104.861354	0	0.08188395	0.0880959	2238135.32	0	0.01576191	99.9264826	1360.22155
971	0.08495641	104.856065	0	0.0818761	0.0880878	2238148.36	0	0.0157622	99.9264863	1360.29149
972	0.08498222	104.88602	0	0.08190188	0.08811362	2237977.73	0	0.01575777	99.9264598	1359.80101
973	0.08497719	104.877588	0	0.08189678	0.08810867	2238032.69	0	0.01575938	99.9264674	1359.94187
974	0.08500288	104.932436	0	0.08192317	0.08813361	2237424.64	0	0.01574755	99.926409	1358.86177
975	0.0849916	104.904855	0	0.08191148	0.08812276	2237953.55	0	0.01575383	99.9264438	1359.50387
976	0.08501135	104.957255	0	0.08193207	0.08814164	2237620.83	0	0.01574162	99.9263859	1358.43504

© 2024 Zhang X et al. *JAMA Pediatrics*.

977	0.0849544	104.853602	0	0.08187409	0.08808579	2238148.45	0	0.01576238	99.9264856	1360.27794
978	0.08496697	104.864655	0	0.08188654	0.08809847	2238139.74	0	0.01576149	99.9264824	1360.21766
979	0.08490796	104.871677	0	0.08182979	0.08803716	2237983.89	0	0.01574786	99.9264172	1359.01411
980	0.08497188	104.870062	0	0.08189144	0.08810339	2238107.86	0	0.01576069	99.9264763	1360.10654
981	0.08495896	104.856653	0	0.08187858	0.08809042	2238145.6	0	0.01576233	99.9264852	1360.2704
982	0.08491983	104.859151	0	0.08184089	0.08804983	2238107.48	0	0.01575407	99.9264479	1359.58026
983	0.08494968	104.852315	0	0.0818695	0.08808094	2238148.52	0	0.01576201	99.9264853	1360.27297
984	0.08496268	104.860378	0	0.08188228	0.08809416	2238144.73	0	0.01576199	99.9264848	1360.26369
985	0.08496123	104.858828	0	0.08188083	0.08809271	2238144.99	0	0.01576214	99.9264852	1360.2708
986	0.08497661	104.878198	0	0.08189624	0.08810804	2238133.97	0	0.01575931	99.9264728	1360.04137
987	0.08497224	104.871204	0	0.08189182	0.08810373	2238132.56	0	0.01576051	99.9264778	1360.13405
988	0.0849266	104.853476	0	0.08184726	0.08805701	2238060.2	0	0.015757	99.9264582	1359.77041
989	0.08497105	104.868789	0	0.0818906	0.08810258	2238079.04	0	0.01576089	99.9264741	1360.06408
990	0.08501641	104.973624	0	0.08193743	0.08814638	2237643.17	0	0.0157376	99.9263681	1358.10695
991	0.08496835	104.867764	0	0.08188796	0.08809981	2238144.31	0	0.01576103	99.9264811	1360.19464
992	0.08496626	104.864331	0	0.08188584	0.08809774	2238143.09	0	0.01576152	99.926483	1360.22881
993	0.08498316	104.888077	0	0.08190285	0.08811453	2238078.98	0	0.01575738	99.9264621	1359.84342
994	0.08496495	104.863133	0	0.08188455	0.08809643	2238144.61	0	0.01576166	99.9264837	1360.2432
995	0.0849612	104.858441	0	0.08188079	0.08809269	2238142.53	0	0.0157622	99.9264844	1360.25501
996	0.08491347	104.865213	0	0.08183492	0.08804306	2238072.44	0	0.01575095	99.9264329	1359.30313
997	0.08499319	104.908091	0	0.08191311	0.08812431	2234147.34	0	0.0157531	99.9263613	1357.9825
998	0.08489006	104.902843	0	0.08181342	0.08801769	2238010.89	0	0.01573497	99.9263588	1357.93471
999	0.08489251	104.897045	0	0.08181563	0.08802041	2237683.24	0	0.01573708	99.9263652	1358.05388
1000	0.08483791	105.063553	0	0.08176776	0.08795888	2237821.16	0	0.01567599	99.9260823	1352.85527
1001	0.08492825	104.852415	0	0.08184881	0.08805875	2237994.46	0	0.01575762	99.9264564	1359.7367
1002	0.08489184	104.89844	0	0.08181502	0.08801967	2237754.45	0	0.01573654	99.9263635	1358.02168
1003	0.08493872	104.849477	0	0.08185883	0.08806968	2238127.47	0	0.01576058	99.9264756	1360.09192
1004	0.08490455	104.87824	0	0.0818267	0.08803344	2238105.05	0	0.01574546	99.9264084	1358.85022
1005	0.08488569	104.912366	0	0.08180948	0.08801288	2238007.77	0	0.01573124	99.9263414	1357.61531
1006	0.08489192	104.899069	0	0.08181511	0.08801974	2238018.91	0	0.01573648	99.9263658	1358.06527
1007	0.08499233	104.906278	0	0.08191223	0.08812347	2237669.08	0	0.01575351	99.9264362	1359.36441
1008	0.08499647	104.916309	0	0.08191651	0.08812746	2237985.09	0	0.0157513	99.9264334	1359.31258
1009	0.08495762	104.85522	0	0.08187725	0.08808908	2238141.06	0	0.01576244	99.9264785	1360.1461
1010	0.0850184	104.98031	0	0.08193955	0.08814825	2237457.73	0	0.01573593	99.9263591	1357.94074
1011	0.08501161	104.957988	0	0.08193234	0.08814188	2237472.34	0	0.01574143	99.9263836	1358.39307
1012	0.08499305	104.912771	0	0.08191311	0.08812403	2238133.15	0	0.01575239	99.9264411	1359.45503
1013	0.08496642	104.864679	0	0.08188601	0.0880979	2238143.53	0	0.01576147	99.9264828	1360.22645
1014	0.08491932	104.860723	0	0.08184044	0.08804925	2238127.32	0	0.01575366	99.9264467	1359.55781
1015	0.08496592	104.86282	0	0.08188547	0.08809744	2238125.31	0	0.01576174	99.9264805	1360.18414
1016	0.08496452	104.861279	0	0.08188407	0.08809603	2238124.06	0	0.01576193	99.92648	1360.17465
1017	0.08496076	104.860934	0	0.08188044	0.08809215	2238147.83	0	0.01576179	99.9264848	1360.26279
1018	0.08497897	104.881073	0	0.0818986	0.0881104	2238113.04	0	0.01575875	99.9264693	1359.97694

© 2024 Zhang X et al. *JAMA Pediatrics*.

1019	0.08496089	104.857708	0	0.08188047	0.08809239	2238105.03	0	0.0157623	99.9264664	1359.92305
1020	0.08499096	104.904703	0	0.08191086	0.0881221	2238107.26	0	0.01575395	99.9264477	1359.57706
1021	0.08500184	104.929754	0	0.08192209	0.08813262	2237743.08	0	0.01574818	99.9264161	1358.9921
1022	0.08493342	104.85025	0	0.08185374	0.08806417	2238021.24	0	0.01575928	99.9264609	1359.82065
1023	0.08494883	104.851734	0	0.08186866	0.08808008	2238148.31	0	0.01576199	99.926485	1360.267
1024	0.08494883	104.851734	0	0.08186866	0.08808008	2238148.31	0	0.01576199	99.926485	1360.267
1025	0.08495901	104.857202	0	0.08187864	0.08809045	2238147.03	0	0.01576224	99.926486	1360.28513
1026	0.0849463	104.849931	0	0.08186616	0.08807751	2238142.44	0	0.01576189	99.9264797	1360.16799
1027	0.08498118	104.884176	0	0.08190083	0.0881126	2237982.13	0	0.01575813	99.9264612	1359.82651
1028	0.0850202	104.986494	0	0.08194146	0.08814992	2237161.14	0	0.01573438	99.9263499	1357.77131
1029	0.08501179	104.95856	0	0.08193254	0.08814206	2237458.78	0	0.01574129	99.9263829	1358.37936
1030	0.08495657	104.854544	0	0.08187621	0.08808801	2238145.15	0	0.01576245	99.9264812	1360.19577
1031	0.08499577	104.914334	0	0.08191578	0.0881268	2237776.36	0	0.01575172	99.9264314	1359.27639
1032	0.08490965	104.869769	0	0.08183137	0.08803897	2238054.43	0	0.01574881	99.9264229	1359.11807
1033	0.08499496	104.91242	0	0.08191494	0.08812601	2237818.38	0	0.01575215	99.926434	1359.32352
1034	0.0849978	104.919234	0	0.08191788	0.08812875	2237635.89	0	0.01575061	99.9264245	1359.14784
1035	0.08497595	104.875804	0	0.08189553	0.08810743	2238081.75	0	0.01575971	99.9264713	1360.01379
1036	0.08501312	104.962888	0	0.08193394	0.0881433	2237712.25	0	0.01574025	99.9263806	1358.33834
1037	0.08497043	104.867957	0	0.08188998	0.08810196	2238069.97	0	0.01576102	99.9264732	1360.04813
1038	0.08494498	104.850322	0	0.0818649	0.08807613	2238146.15	0	0.01576161	99.9264828	1360.22545
1039	0.08492697	104.85333	0	0.08184761	0.0880574	2238077.81	0	0.01575713	99.9264596	1359.79679
1040	0.08495498	104.853947	0	0.08187466	0.08808638	2238148.27	0	0.01576239	99.9264857	1360.27868
1041	0.08492182	104.858463	0	0.08184278	0.08805191	2238128.86	0	0.01575481	99.926452	1359.65571
1042	0.08496503	104.863551	0	0.08188463	0.08809649	2238145.26	0	0.0157616	99.9264836	1360.24069
1043	0.08493908	104.849095	0	0.08185917	0.08807006	2237606.37	0	0.01576071	99.9263711	1358.162
1044	0.08492884	104.852062	0	0.08184937	0.08805937	2237923	0	0.01575783	99.9264523	1359.661
1045	0.08496502	104.862799	0	0.0818846	0.08809651	2238143.26	0	0.01576171	99.9264837	1360.24258
1046	0.08496177	104.858648	0	0.08188135	0.08809327	2238133.05	0	0.0157622	99.9264809	1360.1915
1047	0.08494149	104.849175	0	0.0818615	0.08807255	2238112.23	0	0.01576117	99.9264714	1360.01543
1048	0.08489794	104.887304	0	0.08182058	0.08802632	2237956.53	0	0.01574116	99.9263866	1358.44911
1049	0.08494861	104.850375	0	0.08186841	0.08807989	2238131.8	0	0.01576216	99.9264445	1359.51799
1050	0.08496224	104.860024	0	0.08188184	0.08809371	2238145.24	0	0.01576202	99.926485	1360.26753
1051	0.08493436	104.852299	0	0.0818547	0.08806508	2238142.84	0	0.01575918	99.9264725	1360.03496
1052	0.08494569	104.850834	0	0.08186561	0.08807686	2238147.22	0	0.01576165	99.9264834	1360.23748
1053	0.08494095	104.851178	0	0.08186104	0.08807194	2238146.05	0	0.01576076	99.9264797	1360.1693
1054	0.08495424	104.853519	0	0.08187393	0.08808562	2238148.5	0	0.01576238	99.9264856	1360.27843
1055	0.08493738	104.849519	0	0.08185754	0.08806829	2238112.83	0	0.01576029	99.9264726	1360.03695
1056	0.08495756	104.856145	0	0.08187722	0.08808899	2238147.71	0	0.01576229	99.9264862	1360.28967
1057	0.08495254	104.852109	0	0.08187225	0.08808391	2238148.72	0	0.01576241	99.9264769	1360.1168
1058	0.0849968	104.916786	0	0.08191685	0.08812779	2237690.16	0	0.01575117	99.9264276	1359.20626
1059	0.08491792	104.860063	0	0.08183907	0.08804783	2237967.32	0	0.0157533	99.9264402	1359.43795
1060	0.0849301	104.852397	0	0.0818506	0.08806066	2238128.22	0	0.01575812	99.9264667	1359.9275

© 2024 Zhang X et al. *JAMA Pediatrics*.

1061	0.08495852	104.856518	0	0.08187815	0.08808997	2238146.7	0	0.01576231	99.9264858	1360.28114
1062	0.084945	104.85022	0	0.08186491	0.08807616	2238145.84	0	0.01576163	99.9264827	1360.22315
1063	0.08493004	104.852347	0	0.08185054	0.08806061	2238126.59	0	0.01575811	99.9264665	1359.92494
1064	0.08498636	104.893995	0	0.08190611	0.08811767	2238029.76	0	0.01575616	99.9264554	1359.71948
1065	0.08497485	104.874817	0	0.08189444	0.08810633	2238126.84	0	0.01575989	99.9264749	1360.07991
1066	0.08496444	104.864904	0	0.08188411	0.08809585	2238147.1	0	0.01576137	99.9264829	1360.22748
1067	0.08495198	104.852137	0	0.08187171	0.08808333	2238148.71	0	0.01576234	99.9264843	1360.2545
1068	0.08492386	104.854978	0	0.08184466	0.08805413	2237554.55	0	0.01575597	99.9264319	1359.28566
1069	0.08492645	104.853304	0	0.08184711	0.08805686	2237612.5	0	0.01575699	99.9264343	1359.32909
1070	0.08493244	104.850715	0	0.08185281	0.08806314	2238086.37	0	0.01575897	99.9264665	1359.92464
1071	0.08492064	104.857518	0	0.08184162	0.08805072	2237844.62	0	0.01575459	99.9264408	1359.44984
1072	0.08491482	104.86334	0	0.08183617	0.08804452	2238013.5	0	0.01575172	99.9264349	1359.3396
1073	0.08491213	104.866251	0	0.08183366	0.08804165	2237944.63	0	0.0157503	99.9264271	1359.19643
1074	0.08501939	104.983698	0	0.08194059	0.08814916	2237411.39	0	0.01573508	99.9263549	1357.86414
1075	0.08497319	104.875358	0	0.08189286	0.08810459	2238143.72	0	0.01575985	99.9264758	1360.09642
1076	0.0849083	104.874247	0	0.0818302	0.08803744	2238124.37	0	0.0157476	99.9264186	1359.03932
1077	0.08490818	104.872459	0	0.08183003	0.08803737	2238096.18	0	0.01574782	99.9264191	1359.04915
1078	0.08497068	104.871221	0	0.08189031	0.08810211	2238143.98	0	0.01576051	99.9264788	1360.15192
1079	0.08489643	104.889908	0	0.08181919	0.08802467	2237913.76	0	0.01574006	99.9263811	1358.34717
1080	0.08492282	104.863802	0	0.08184391	0.08805279	2238143.35	0	0.0157543	99.9264501	1359.62183
1081	0.08493164	104.853185	0	0.08185211	0.08806223	2238141	0	0.01575839	99.9264688	1359.96646
1082	0.084868	104.95736	0	0.08179372	0.08799321	2237744.92	0	0.01571399	99.9262597	1356.11125
1083	0.08486298	104.979685	0	0.08178952	0.08798735	2238100.5	0	0.01570731	99.9262303	1355.56941
1084	0.08488184	104.920414	0	0.08180601	0.08800866	2237740.31	0	0.01572791	99.926324	1357.29454
1085	0.08492188	104.856969	0	0.0818428	0.08805202	2238081.64	0	0.01575506	99.9264513	1359.64331
1086	0.08489203	104.898155	0	0.08181519	0.08801987	2237824.82	0	0.01573667	99.9263648	1358.04585
1087	0.08497454	104.874376	0	0.08189413	0.08810602	2238127.64	0	0.01575997	99.9264753	1360.08678
1088	0.0848965	104.892829	0	0.08181935	0.08802466	2238110.58	0	0.01573964	99.9263815	1358.35363
1089	0.08494425	104.857856	0	0.08186442	0.08807515	2238148.3	0	0.01576033	99.9264782	1360.14155
1090	0.08497603	104.876071	0	0.08189561	0.08810751	2238100.95	0	0.01575966	99.9264724	1360.03271
1091	0.08493849	104.850422	0	0.08185864	0.08806941	2238142.31	0	0.01576038	99.9264775	1360.12852
1092	0.08491542	104.862683	0	0.08183674	0.08804516	2238012.16	0	0.01575204	99.9264362	1359.36405
1093	0.08491334	104.865348	0	0.08183481	0.08804293	2238071.29	0	0.01575088	99.9264326	1359.29709
1094	0.08493401	104.851459	0	0.08185434	0.08806474	2238139.03	0	0.01575923	99.9264723	1360.03203
1095	0.08489936	104.884946	0	0.08182188	0.08802786	2237985.51	0	0.01574217	99.9263916	1358.54045
1096	0.08490069	104.88905	0	0.08182329	0.08802912	2238130.63	0	0.01574214	99.9263933	1358.57219
1097	0.08488839	104.906369	0	0.08181192	0.08801586	2238007.84	0	0.01573357	99.9263523	1357.8152
1098	0.08485161	105.014392	0	0.08177956	0.08797454	2238053.42	0	0.01569406	99.9261679	1354.42395
1099	0.08495691	104.857094	0	0.08187661	0.08808828	2238148.39	0	0.01576209	99.926486	1360.28576
1100	0.08494686	104.852666	0	0.08186678	0.08807801	2238148.23	0	0.01576155	99.9264836	1360.24064
1101	0.08490582	104.875294	0	0.08182783	0.08803484	2238069.26	0	0.01574643	99.9264123	1358.92279
1102	0.0849294	104.853054	0	0.08184994	0.08805992	2238132.22	0	0.01575783	99.9264657	1359.90965

© 2024 Zhang X et al. *JAMA Pediatrics*.

1103	0.08487925	104.927211	0	0.0818037	0.08800577	2237923.33	0	0.01572541	99.9263138	1357.1055
1104	0.08494054	104.856804	0	0.08186081	0.08807135	2238147.79	0	0.01575981	99.9264758	1360.09697
1105	0.08500063	104.926504	0	0.08192082	0.08813146	2237710.49	0	0.01574894	99.9264188	1359.04268
1106	0.08493561	104.849882	0	0.08185585	0.08806645	2238111.46	0	0.01575984	99.9264715	1360.01636
1107	0.0849448	104.85292	0	0.08186481	0.08807587	2238147.97	0	0.01576118	99.926482	1360.21072
1108	0.08497365	104.872297	0	0.08189321	0.08810516	2238055.77	0	0.01576031	99.9264712	1360.0111
1109	0.08491348	104.866185	0	0.08183497	0.08804305	2238113.2	0	0.01575081	99.9264332	1359.30864
1110	0.084985	104.891472	0	0.08190472	0.08811633	2238063.56	0	0.01575669	99.9264587	1359.77974
1111	0.08498516	104.891382	0	0.08190487	0.0881165	2237837.67	0	0.01575669	99.9264514	1359.6447
1112	0.08492464	104.854547	0	0.0818454	0.08805495	2237946.1	0	0.01575627	99.9264501	1359.62108
1113	0.08498625	104.893829	0	0.081906	0.08811756	2238042.59	0	0.0157562	99.926456	1359.72946
1114	0.08498086	104.883486	0	0.08190049	0.08811229	2237753.66	0	0.01575826	99.9264518	1359.65293
1115	0.08492979	104.851697	0	0.08185028	0.08806036	2238035.85	0	0.01575814	99.9264604	1359.8108
1116	0.08497952	104.881133	0	0.08189913	0.08811097	2233270.38	0	0.01575871	99.9262402	1355.75293
1117	0.08497673	104.876914	0	0.08189632	0.08810821	2238056.44	0	0.01575951	99.9264692	1359.97374
1118	0.08500079	104.926992	0	0.08192099	0.08813161	2237800.88	0	0.01574883	99.9264197	1359.05882
1119	0.08496083	104.857657	0	0.08188041	0.08809233	2238105.99	0	0.0157623	99.9264666	1359.92655
1120	0.08495986	104.858016	0	0.08187948	0.08809131	2238146.81	0	0.01576218	99.9264859	1360.28243
1121	0.08493077	104.851138	0	0.08185121	0.0880614	2237787.79	0	0.01575848	99.9264422	1359.47562
1122	0.08501463	104.967555	0	0.08193554	0.08814473	2237040.03	0	0.01573908	99.9263694	1358.1312
1123	0.08494856	104.85097	0	0.08186837	0.08807982	2238147.76	0	0.01576206	99.9264842	1360.25215
1124	0.08496075	104.857747	0	0.08188034	0.08809224	2238135.86	0	0.01576228	99.9264811	1360.19418
1125	0.08498912	104.899852	0	0.08190895	0.08812035	2238059.26	0	0.01575494	99.926451	1359.63772
1126	0.08497607	104.875797	0	0.08189565	0.08810756	2238002.24	0	0.0157597	99.9264664	1359.92271
1127	0.08493706	104.849432	0	0.08185724	0.08806797	2238073.36	0	0.01576023	99.9264666	1359.92547
1128	0.08500113	104.92769	0	0.08192135	0.08813195	2236912.9	0	0.01574865	99.926406	1358.80596
1129	0.08493776	104.849373	0	0.08185791	0.08806869	2238095.22	0	0.01576039	99.9264698	1359.98598
1130	0.08499573	104.914482	0	0.08191575	0.08812675	2237971.57	0	0.01575171	99.926435	1359.34134
1131	0.08499927	104.923219	0	0.08191942	0.08813016	2237931.03	0	0.01574971	99.9264255	1359.1666
1132	0.08499513	104.913587	0	0.08191514	0.08812615	2238068.98	0	0.01575194	99.9264378	1359.39439
1133	0.08501836	104.980062	0	0.0819395	0.08814821	2237121.61	0	0.01573598	99.9263567	1357.89624
1134	0.08501065	104.956442	0	0.08193137	0.08814094	2238055.74	0	0.01574193	99.9263916	1358.54141
1135	0.08496225	104.86029	0	0.08188186	0.08809372	2238145.83	0	0.01576198	99.9264851	1360.26806
1136	0.08494242	104.849936	0	0.08186242	0.08807349	2238143.74	0	0.01576122	99.9264809	1360.18988
1137	0.08498459	104.892862	0	0.08190436	0.08811586	2238131.59	0	0.01575651	99.9264601	1359.80535
1138	0.08498106	104.890051	0	0.08190088	0.0881123	2238142.52	0	0.01575723	99.9264637	1359.87324
1139	0.08497365	104.874271	0	0.08189327	0.0881051	2238139.61	0	0.01576001	99.9264762	1360.10468
1140	0.08496973	104.867014	0	0.08188927	0.08810126	2238042.9	0	0.01576116	99.92647	1359.98915
1141	0.08500555	104.940858	0	0.08192599	0.08813612	2238040.57	0	0.01574564	99.9264086	1358.85411
1142	0.08501097	104.955817	0	0.08193166	0.08814129	2233767.28	0	0.01574194	99.9263486	1357.74831
1143	0.08498839	104.898262	0	0.08190819	0.08811963	2238055.12	0	0.01575527	99.9264524	1359.66283
1144	0.08500367	104.934708	0	0.081924	0.08813436	2237725.8	0	0.01574702	99.9264108	1358.89518

© 2024 Zhang X et al. *JAMA Pediatrics*.

1145	0.08497352	104.871986	0	0.08189307	0.08810503	2237918.67	0	0.01576037	99.9264606	1359.81523
1146	0.08501222	104.960898	0	0.08193301	0.08814242	2238019.09	0	0.01574081	99.9263861	1358.43911
1147	0.08495267	104.85394	0	0.08187243	0.08808399	2238148.73	0	0.01576214	99.9264861	1360.28663
1148	0.08496328	104.860246	0	0.08188285	0.08809478	2238137.24	0	0.01576204	99.926483	1360.2297
1149	0.08501306	104.96257	0	0.08193388	0.08814325	2237422.95	0	0.01574031	99.9263782	1358.29383
1150	0.0849838	104.888687	0	0.08190349	0.08811518	2232712.91	0	0.01575723	99.9262725	1356.34581
1151	0.0849803	104.884516	0	0.08189998	0.08811168	2238132.12	0	0.01575814	99.9264675	1359.94286
1152	0.08495273	104.85284	0	0.08187246	0.08808409	2238148.72	0	0.01576232	99.9264858	1360.28053
1153	0.08501002	104.953091	0	0.08193067	0.08814039	2237496.46	0	0.01574262	99.926389	1358.49337
1154	0.0850112	104.957809	0	0.08193194	0.08814146	2238032.1	0	0.01574157	99.9263897	1358.50635
1155	0.08501253	104.96074	0	0.08193331	0.08814275	2236492.02	0	0.01574075	99.9263713	1358.16562
1156	0.0849917	104.905727	0	0.08191161	0.08812284	2238079.33	0	0.01575368	99.9264459	1359.54358
1157	0.08496109	104.858274	0	0.08188068	0.08809258	2238141.79	0	0.01576222	99.926484	1360.24899
1158	0.08498411	104.88957	0	0.08190381	0.08811547	2238014.4	0	0.01575706	99.9264586	1359.77786
1159	0.08494009	104.849427	0	0.08186015	0.0880711	2238132.57	0	0.01576086	99.9264771	1360.12104
1160	0.08501059	104.954611	0	0.08193126	0.08814093	2229244.32	0	0.01574223	99.9263034	1356.91537
1161	0.08499482	104.911947	0	0.0819148	0.08812588	2237306.78	0	0.01575225	99.9264245	1359.14888
1162	0.08499561	104.913989	0	0.08191562	0.08812664	2237825.69	0	0.0157518	99.9264327	1359.29927
1163	0.08498608	104.893951	0	0.08190584	0.08811738	2238094.77	0	0.01575619	99.9264575	1359.75828
1164	0.08495274	104.852389	0	0.08187245	0.08808411	2238148.71	0	0.01576239	99.9264833	1360.23434
1165	0.08484685	105.027128	0	0.08177533	0.08796922	2233027.15	0	0.01568856	99.9261253	1353.64263
1166	0.08494428	104.849325	0	0.08186419	0.08807544	2238060.75	0	0.01576165	99.9264369	1359.377
1167	0.08495545	104.853778	0	0.08187511	0.08808687	2238146.31	0	0.01576246	99.9264794	1360.16316
1168	0.08492341	104.855497	0	0.08184424	0.08805365	2238013.72	0	0.01575576	99.9264514	1359.6447
1169	0.08487047	104.964315	0	0.08179631	0.08799557	2238122.9	0	0.01571449	99.926264	1356.19033
1170	0.08494728	104.850031	0	0.08186711	0.08807853	2238137.48	0	0.01576202	99.926471	1360.0067
1171	0.08494643	104.853965	0	0.08186641	0.08807753	2238148.32	0	0.01576128	99.9264825	1360.22084
1172	0.08494249	104.852662	0	0.08186257	0.08807349	2238147.46	0	0.01576082	99.9264803	1360.17948
1173	0.08492128	104.856864	0	0.08184222	0.08805141	2230192.56	0	0.01575489	99.9261586	1354.25461
1174	0.08498591	104.892861	0	0.08190564	0.08811724	2237858.7	0	0.01575638	99.9264511	1359.63888
1175	0.08500252	104.933962	0	0.08192287	0.08813319	2238105.6	0	0.01574739	99.9264175	1359.01829
1176	0.08500398	104.936025	0	0.08192434	0.08813465	2237985.15	0	0.01574675	99.926413	1358.93494
1177	0.08501355	104.963996	0	0.08193439	0.08814371	2236433.88	0	0.01573995	99.9263675	1358.09672
1178	0.08499146	104.904741	0	0.08191134	0.08812262	2238023.9	0	0.01575387	99.9264455	1359.53606
1179	0.0849707	104.868977	0	0.08189027	0.08810221	2238131.81	0	0.01576086	99.9264792	1360.15845
1180	0.08498159	104.88471	0	0.08190124	0.08811301	2237005.88	0	0.01575802	99.926421	1359.08315
1181	0.08499618	104.915524	0	0.08191621	0.08812719	2237951.44	0	0.01575147	99.9264336	1359.31535
1182	0.08498955	104.900488	0	0.08190937	0.08812077	2238001.23	0	0.01575478	99.9264489	1359.59865
1183	0.08495405	104.856387	0	0.08187383	0.08808534	2238148.7	0	0.01576192	99.9264854	1360.2742
1184	0.0849962	104.915409	0	0.08191623	0.08812721	2237833	0	0.01575148	99.9264315	1359.27714
1185	0.08495107	104.852232	0	0.08187083	0.08808238	2238148.65	0	0.01576221	99.9264856	1360.27796
1186	0.0849147	104.865326	0	0.08183612	0.08804434	2238122.02	0	0.01575138	99.926436	1359.36107

© 2024 Zhang X et al. *JAMA Pediatrics*.

1187	0.08498098	104.884589	0	0.08190064	0.08811238	2238112.38	0	0.01575808	99.9264664	1359.9231
1188	0.08499381	104.910234	0	0.08191378	0.08812489	2238044.19	0	0.01575267	99.9264406	1359.44618
1189	0.08499077	104.903552	0	0.08191065	0.08812195	2238072.34	0	0.01575415	99.9264478	1359.57926
1190	0.08498214	104.886107	0	0.08190181	0.08811353	2238064.91	0	0.01575776	99.9264632	1359.86319
1191	0.08499369	104.909685	0	0.08191364	0.08812478	2237988.64	0	0.01575278	99.92644	1359.4341
1192	0.08499312	104.908508	0	0.08191306	0.08812423	2238022.09	0	0.01575305	99.9264418	1359.46845
1193	0.08500314	104.934859	0	0.08192349	0.08813381	2238086.88	0	0.01574711	99.926416	1358.99044
1194	0.08498244	104.887067	0	0.08190212	0.08811381	2238104.26	0	0.01575759	99.926464	1359.87765
1195	0.08499611	104.915059	0	0.08191613	0.08812712	2237555.57	0	0.01575155	99.9264268	1359.18976
1196	0.08498831	104.899382	0	0.08190814	0.08811952	2238118.31	0	0.01575511	99.9264533	1359.67989
1197	0.0849958	104.914323	0	0.08191581	0.08812683	2237564.02	0	0.01575172	99.9264275	1359.2041
1198	0.08500592	104.942016	0	0.08192638	0.08813647	2238047.28	0	0.01574537	99.9264074	1358.83286
1199	0.08498911	104.900286	0	0.08190894	0.08812032	2238096.39	0	0.01575487	99.9264516	1359.64975
1200	0.08499682	104.916837	0	0.08191687	0.0881278	2237707.27	0	0.01575115	99.9264279	1359.21098
1201	0.08500525	104.939099	0	0.08192566	0.08813587	2237625.79	0	0.01574598	99.926405	1358.78864
1202	0.0850002	104.92522	0	0.08192038	0.08813106	2236305.79	0	0.01574922	99.9263987	1358.67226
1203	0.0849817	104.886178	0	0.08190138	0.08811307	2238120.1	0	0.01575779	99.9264654	1359.90464
1204	0.08499335	104.908634	0	0.08191328	0.08812446	2237760.49	0	0.01575299	99.9264362	1359.36437
1205	0.08500414	104.936608	0	0.08192451	0.0881348	2238009.38	0	0.01574662	99.9264127	1358.93001
1206	0.08497296	104.871369	0	0.08189252	0.08810447	2238073.23	0	0.01576047	99.9264729	1360.04216
1207	0.08484167	105.051103	0	0.08177103	0.08796315	2238022.78	0	0.0156809	99.9261059	1353.2884
1208	0.08494106	104.850672	0	0.08186113	0.08807206	2238145.33	0	0.01576086	99.92648	1360.17385
1209	0.08494436	104.850249	0	0.0818643	0.0880755	2238145.8	0	0.01576152	99.9264824	1360.21847
1210	0.08490327	104.880664	0	0.08182553	0.08803204	2238110.59	0	0.01574454	99.9264042	1358.77362
1211	0.08494106	104.850672	0	0.08186113	0.08807206	2238145.33	0	0.01576086	99.92648	1360.17385
1212	0.08496546	104.862743	0	0.08188503	0.08809697	2238138.85	0	0.01576174	99.926483	1360.2294
1213	0.08496799	104.86687	0	0.08188759	0.08809946	2238143.58	0	0.01576116	99.9264816	1360.20361
1214	0.08497676	104.876953	0	0.08189635	0.08810824	2238053.03	0	0.0157595	99.9264689	1359.96965
1215	0.08497438	104.873191	0	0.08189394	0.08810588	2237838.53	0	0.01576016	99.9264552	1359.71597
1216	0.08496774	104.872419	0	0.08188751	0.08809904	2238147.31	0	0.0157603	99.9264781	1360.13891
1217	0.08494399	104.851155	0	0.08186397	0.08807509	2238147.1	0	0.01576132	99.9264822	1360.21567
1218	0.08497256	104.871403	0	0.08189213	0.08810406	2238127.44	0	0.01576047	99.9264773	1360.12375
1219	0.08498532	104.893656	0	0.0819051	0.0881166	2238125.8	0	0.01575632	99.926459	1359.78574
1220	0.08492399	104.85547	0	0.08184479	0.08805425	2238095.6	0	0.01575594	99.9264556	1359.72298
1221	0.0849892	104.899797	0	0.08190902	0.08812043	2238014.7	0	0.01575494	99.9264499	1359.61669
1222	0.08497513	104.877921	0	0.08189481	0.08810653	2238142.4	0	0.0157594	99.9264737	1360.0578
1223	0.08497949	104.882817	0	0.08189915	0.08811089	2238130.14	0	0.01575845	99.9264688	1359.96731
1224	0.08497022	104.868077	0	0.08188977	0.08810173	2238124.8	0	0.015761	99.9264789	1360.15436
1225	0.08496724	104.865314	0	0.08188682	0.08809873	2238141.74	0	0.01576139	99.9264823	1360.2163
1226	0.08490125	104.88232	0	0.08182363	0.0880299	2238058.64	0	0.01574341	99.9263983	1358.66371
1227	0.08497275	104.871281	0	0.08189231	0.08810426	2238108.28	0	0.01576049	99.9264758	1360.09592
1228	0.08499056	104.902606	0	0.08191041	0.08812175	2237974.08	0	0.01575433	99.9264463	1359.55069

© 2024 Zhang X et al. *JAMA Pediatrics*.

1229	0.0850041	104.935632	0	0.08192444	0.08813478	1905303.26	0	0.01574678	99.9223387	1287.64278
1230	0.08499144	104.904295	0	0.08191131	0.08812261	2237674.81	0	0.01575394	99.9264379	1359.39524
1231	0.08495947	104.858616	0	0.08187913	0.08809089	2238147.73	0	0.01576206	99.9264858	1360.28143
1232	0.08496412	104.861551	0	0.0818837	0.08809561	2238142.34	0	0.01576187	99.926484	1360.24828
1233	0.08497978	104.882008	0	0.08189941	0.08811122	2238075.42	0	0.01575856	99.9264668	1359.92993
1234	0.08498387	104.889265	0	0.08190357	0.08811523	2238055.5	0	0.01575713	99.9264603	1359.80914
1235	0.08497267	104.872297	0	0.08189226	0.08810414	2238137.28	0	0.01576033	99.9264775	1360.12754
1236	0.08498196	104.885612	0	0.08190161	0.08811336	2238020.11	0	0.01575786	99.9264618	1359.83721
1237	0.08495504	104.854568	0	0.08187474	0.08808643	2238148.48	0	0.0157623	99.9264864	1360.29224
1238	0.08499576	104.914734	0	0.08191579	0.08812678	2238019.64	0	0.01575166	99.9264356	1359.35396
1239	0.08499868	104.921994	0	0.08191882	0.08812958	2238017.02	0	0.01575002	99.9264282	1359.21667
1240	0.08493656	104.849678	0	0.08185675	0.08806744	2238113.01	0	0.01576009	99.9264722	1360.03055
1241	0.08497927	104.881425	0	0.0818989	0.0881107	2238105.38	0	0.01575868	99.9264687	1359.96467
1242	0.08497091	104.869149	0	0.08189047	0.08810242	2238129.24	0	0.01576083	99.9264788	1360.15201
1243	0.08500505	104.939083	0	0.08192546	0.08813565	2237996.87	0	0.01574603	99.9264098	1358.87743
1244	0.08498062	104.883055	0	0.08190025	0.08811205	2237656.34	0	0.01575835	99.9264477	1359.57597
1245	0.08497521	104.875406	0	0.08189481	0.08810668	2238127.43	0	0.01575979	99.9264745	1360.07275
1246	0.08497417	104.873396	0	0.08189374	0.08810566	2238112.69	0	0.01576013	99.9264749	1360.07934
1247	0.08496788	104.865794	0	0.08188745	0.08809938	2238139.46	0	0.01576133	99.9264818	1360.2065
1248	0.08498717	104.895247	0	0.08190693	0.08811847	2237200.38	0	0.01575588	99.9264304	1359.25757
1249	0.08498473	104.890708	0	0.08190444	0.08811607	2237994.51	0	0.01575683	99.926457	1359.74878
1250	0.08500832	104.948164	0	0.08192888	0.08813877	2237817.71	0	0.01574383	99.9263978	1358.655
1251	0.08498834	104.897989	0	0.08190813	0.08811959	2238019.17	0	0.01575532	99.9264516	1359.64898
1252	0.08499335	104.908875	0	0.08191329	0.08812445	2237977.36	0	0.01575296	99.9264405	1359.44406
1253	0.08499228	104.907062	0	0.0819122	0.0881234	2238081.43	0	0.0157534	99.9264446	1359.52036
1254	0.08498914	104.899816	0	0.08190896	0.08812037	2238048.2	0	0.01575494	99.9264507	1359.6328
1255	0.08496774	104.872419	0	0.08188751	0.08809904	2238147.31	0	0.0157603	99.9264781	1360.13891
1256	0.08498269	104.886932	0	0.08190236	0.08811408	2238013.58	0	0.01575759	99.9264606	1359.81463
1257	0.08500899	104.949882	0	0.08192957	0.08813941	2237186.36	0	0.01574339	99.926389	1358.4934
1258	0.08496803	104.865104	0	0.08188758	0.08809956	2238106.94	0	0.01576144	99.9264776	1360.1303
1259	0.08498459	104.892862	0	0.08190436	0.08811586	2238131.59	0	0.01575651	99.9264601	1359.80535
1260	0.08499988	104.924438	0	0.08192004	0.08813075	2237228.89	0	0.01574941	99.9264134	1358.94271
1261	0.08500404	104.935621	0	0.08192438	0.08813472	2237427.84	0	0.0157468	99.926406	1358.80605
1262	0.08491496	104.86358	0	0.08183632	0.08804466	2238077.72	0	0.01575174	99.9264366	1359.37086
1263	0.08497999	104.882788	0	0.08189964	0.08811141	2238110.68	0	0.01575843	99.9264678	1359.94921
1264	0.08499375	104.910878	0	0.08191373	0.08812481	2238098.57	0	0.01575258	99.9264413	1359.45866
1265	0.0849746	104.874015	0	0.08189418	0.08810609	2238110.74	0	0.01576003	99.9264743	1360.06948
1266	0.08498453	104.89038	0	0.08190424	0.08811588	2238014.21	0	0.0157569	99.9264579	1359.76583
1267	0.08495172	104.854231	0	0.08187151	0.08808299	2238148.72	0	0.01576198	99.9264856	1360.27701
1268	0.08495796	104.856255	0	0.0818776	0.0880894	2238147.33	0	0.01576231	99.9264861	1360.28644
1269	0.08498079	104.884589	0	0.08190046	0.08811218	2238121.69	0	0.0157581	99.9264669	1359.93126
1270	0.08497741	104.877913	0	0.081897	0.08810888	2238021.19	0	0.01575932	99.9264666	1359.92696

© 2024 Zhang X et al. *JAMA Pediatrics.*

1271	0.08496555	104.862523	0	0.08188511	0.08809707	2238131.3	0	0.01576178	99.9264817	1360.20487
1272	0.08498234	104.886414	0	0.08190201	0.08811373	2238054.23	0	0.0157577	99.9264625	1359.8509
1273	0.08494176	104.849191	0	0.08186176	0.08807284	2238115.46	0	0.01576122	99.9264721	1360.02791
1274	0.08493	104.8524	0	0.0818505	0.08806056	2238127.21	0	0.01575809	99.9264665	1359.92417
1275	0.08496001	104.857593	0	0.08187961	0.08809147	2238145.18	0	0.01576226	99.9264853	1360.27162
1276	0.0849555	104.857961	0	0.08187527	0.08808679	2238148.64	0	0.01576182	99.926485	1360.2673
1277	0.08494218	104.84926	0	0.08186217	0.08807327	2238125.56	0	0.01576129	99.926475	1360.08145
1278	0.08495269	104.852768	0	0.08187241	0.08808404	2238148.72	0	0.01576232	99.9264856	1360.27859
1279	0.08494974	104.852504	0	0.08186955	0.08808099	2238148.55	0	0.01576199	99.9264853	1360.27274
1280	0.08497421	104.872919	0	0.08189377	0.08810572	2237624.56	0	0.01576021	99.9264394	1359.42311
1281	0.08493785	104.850413	0	0.08185802	0.08806875	2238141.29	0	0.01576025	99.9264768	1360.11574
1282	0.08495355	104.852728	0	0.08187324	0.08808494	2238148.47	0	0.01576243	99.9264816	1360.20391
1283	0.08496775	104.865197	0	0.08188731	0.08809927	2238133.46	0	0.01576142	99.9264813	1360.19738
1284	0.08493648	104.850143	0	0.08185669	0.08806734	2238134.37	0	0.01576	99.9264749	1360.07958
1285	0.08497523	104.874549	0	0.0818948	0.08810673	2238017.48	0	0.01575993	99.9264678	1359.94801
1286	0.08498112	104.884577	0	0.08190078	0.08811253	2238098.98	0	0.01575808	99.9264658	1359.91223
1287	0.08494869	104.850912	0	0.0818685	0.08807996	2238147.66	0	0.01576209	99.9264839	1360.24659
1288	0.08492477	104.854469	0	0.08184552	0.08805509	2237954.5	0	0.01575632	99.9264506	1359.63104
1289	0.08494415	104.850142	0	0.0818641	0.08807529	2238145.39	0	0.0157615	99.9264822	1360.21427
1290	0.08494503	104.850007	0	0.08186494	0.0880762	2238144.83	0	0.01576167	99.9264821	1360.21377
1291	0.08494218	104.84926	0	0.08186217	0.08807327	2238125.56	0	0.01576129	99.926475	1360.08145
1292	0.08497272	104.87186	0	0.0818923	0.08810421	2238131.92	0	0.0157604	99.9264773	1360.12501
1293	0.08495704	104.854702	0	0.08187666	0.08808849	2238130.09	0	0.01576247	99.9264583	1359.77191
1294	0.08493592	104.849732	0	0.08185614	0.08806677	2238099.29	0	0.01575994	99.9264701	1359.99133
1295	0.08498972	104.900694	0	0.08190955	0.08812094	2237897.32	0	0.01575473	99.9264461	1359.54672
1296	0.08498894	104.89897	0	0.08190875	0.08812019	2237780.2	0	0.0157551	99.9264445	1359.51679
1297	0.084969	104.86617	0	0.08188854	0.08810053	2238078.54	0	0.01576129	99.926474	1360.06309
1298	0.08497844	104.880667	0	0.08189808	0.08810987	2238126.18	0	0.01575884	99.9264704	1359.99595
1299	0.08493559	104.852703	0	0.08185591	0.08806634	2238144.68	0	0.0157594	99.9264736	1360.05652
1300	0.08492813	104.852596	0	0.08184871	0.08805862	2238061.06	0	0.01575756	99.9264602	1359.80824
1301	0.08497252	104.871397	0	0.08189209	0.08810402	2238128.65	0	0.01576047	99.9264774	1360.12569
1302	0.08491847	104.860494	0	0.08183961	0.08804838	2238108.48	0	0.01575342	99.926445	1359.52623
1303	0.08495756	104.855947	0	0.08187721	0.08808899	2238147.5	0	0.01576232	99.9264861	1360.28711
1304	0.08493732	104.849768	0	0.08185749	0.08806822	2238129.74	0	0.01576024	99.926475	1360.08252
1305	0.08495101	104.851734	0	0.08187076	0.08808234	2238148.56	0	0.01576228	99.9264844	1360.25489
1306	0.08498544	104.892077	0	0.08190517	0.08811677	2237986.35	0	0.01575655	99.9264557	1359.72383
1307	0.08495284	104.852731	0	0.08187256	0.0880842	2238148.71	0	0.01576235	99.9264854	1360.27334
1308	0.08488788	104.906524	0	0.08181142	0.08801532	2237515.08	0	0.01573328	99.9263466	1357.71092
1309	0.08488789	104.909408	0	0.08181152	0.08801525	2238091.99	0	0.01573285	99.9263496	1357.76669
1310	0.08484538	105.035928	0	0.08177417	0.08796744	2238004.12	0	0.0156861	99.9261303	1353.73554
1311	0.08489658	104.889617	0	0.08181933	0.08802484	2237906.78	0	0.01574017	99.9263816	1358.35533
1312	0.08490936	104.871126	0	0.08183113	0.08803863	2238104.54	0	0.01574849	99.9264224	1359.10869

© 2024 Zhang X et al. *JAMA Pediatrics*.

1313	0.08497633	104.876139	0	0.08189591	0.08810782	2237935.07	0	0.01575964	99.9264622	1359.84522
1314	0.08496747	104.864424	0	0.08188702	0.088099	2238104.02	0	0.01576153	99.9264772	1360.12177
1315	0.08497467	104.873809	0	0.08189424	0.08810617	2238067.06	0	0.01576006	99.9264714	1360.01548
1316	0.08501751	104.97768	0	0.08193861	0.0881474	2237875.05	0	0.01573662	99.9263656	1358.06009
1317	0.08494359	104.849345	0	0.08186353	0.08807473	2238126.33	0	0.01576153	99.9264737	1360.05782
1318	0.08494165	104.850009	0	0.08186168	0.0880727	2238143.62	0	0.01576107	99.9264804	1360.18058
1319	0.08492331	104.855351	0	0.08184414	0.08805355	2237258.91	0	0.01575575	99.9264193	1359.0527
1320	0.08493543	104.849713	0	0.08185567	0.08806627	2238037.37	0	0.01575983	99.9264623	1359.84657
1321	0.08496285	104.859643	0	0.08188242	0.08809435	2238129.86	0	0.01576211	99.9264807	1360.18686
1322	0.08496254	104.859345	0	0.08188211	0.08809405	2238130.01	0	0.01576214	99.9264805	1360.1836
1323	0.08496694	104.863786	0	0.08188649	0.08809847	2238097.17	0	0.01576162	99.9264759	1360.09887
1324	0.08500908	104.95063	0	0.08192968	0.08813948	2237922.84	0	0.01574325	99.9263963	1358.62793
1325	0.08499963	104.923876	0	0.08191978	0.0881305	2237645.12	0	0.01574954	99.9264203	1359.07094
1326	0.08495107	104.85171	0	0.08187082	0.0880824	2238148.56	0	0.01576229	99.926484	1360.24895
1327	0.08501656	104.973876	0	0.08193758	0.08814653	2236585.52	0	0.01573751	99.9263589	1357.93704
1328	0.08499821	104.920875	0	0.08191832	0.08812912	2238032.85	0	0.01575028	99.9264297	1359.2433
1329	0.0850119	104.958807	0	0.08193265	0.08814216	2236917.43	0	0.01574122	99.9263773	1358.2767
1330	0.08500067	104.926757	0	0.08192088	0.0881315	2237862.21	0	0.01574889	99.9264208	1359.08029
1331	0.08499576	104.914296	0	0.08191577	0.08812679	2237756.5	0	0.01575173	99.9264311	1359.27023
1332	0.08491012	104.868715	0	0.0818318	0.08803949	2237936.99	0	0.01574916	99.9264221	1359.10309
1333	0.08493848	104.851791	0	0.08185867	0.08806936	2238145.49	0	0.01576017	99.9264771	1360.12102
1334	0.08497536	104.874942	0	0.08189494	0.08810685	2238088.84	0	0.01575986	99.9264723	1360.03162
1335	0.08493072	104.851773	0	0.08185118	0.08806133	2238119.32	0	0.01575837	99.9264671	1359.93559
1336	0.08481862	105.149476	0	0.08175163	0.08793634	2237799.05	0	0.01564626	99.9259421	1350.295
1337	0.08487142	104.950392	0	0.08179681	0.08799696	2238067.76	0	0.01571721	99.9262764	1356.41829
1338	0.08492283	104.857022	0	0.08184372	0.088053	2238121.32	0	0.01575535	99.9264541	1359.69499
1339	0.08487613	104.9362	0	0.08180095	0.08800226	2238027.83	0	0.01572222	99.9262996	1356.84501
1340	0.08489241	104.897969	0	0.08181555	0.08802027	2238004.58	0	0.01573689	99.9263676	1358.09755
1341	0.08491561	104.862991	0	0.08183692	0.08804534	2238086.48	0	0.01575205	99.9264382	1359.40138
1342	0.08497563	104.877243	0	0.08189526	0.08810706	2238138.25	0	0.01575949	99.9264739	1360.06091
1343	0.08499173	104.906888	0	0.08191167	0.08812284	2238115.34	0	0.0157535	99.9264459	1359.54262
1344	0.08494977	104.851492	0	0.08186956	0.08808106	2238148.33	0	0.01576215	99.9264849	1360.26493
1345	0.08495836	104.857742	0	0.08187803	0.08808976	2238148.02	0	0.01576211	99.926486	1360.28569
1346	0.08491877	104.859167	0	0.08183986	0.08804873	2237845.37	0	0.01575372	99.926438	1359.3973
1347	0.08493294	104.850706	0	0.08185329	0.08806366	2238111.31	0	0.01575909	99.9264692	1359.97354
1348	0.0848666	104.965938	0	0.08179261	0.0879915	2238084.71	0	0.01571178	99.9262511	1355.95319
1349	0.08493611	104.850158	0	0.08185634	0.08806696	2238132.58	0	0.01575991	99.9264744	1360.0696
1350	0.08492796	104.852884	0	0.08184855	0.08805844	2238096.4	0	0.01575747	99.926462	1359.841
1351	0.08489507	104.894517	0	0.08181802	0.08802313	2238095.91	0	0.01573871	99.926377	1358.27065
1352	0.08469737	105.995859	0	0.08165885	0.08778575	2236998.53	0	0.01536975	99.9246108	1326.44982
1353	0.08496943	104.867431	0	0.081889	0.08810094	2238134.84	0	0.01576109	99.9264803	1360.18041
1354	0.08491428	104.863591	0	0.08183566	0.08804396	2237588.76	0	0.01575149	99.9264233	1359.12682

© 2024 Zhang X et al. *JAMA Pediatrics*.

1355	0.08493764	104.851926	0	0.08185786	0.08806849	2238145.15	0	0.01575997	99.9264762	1360.10423
1356	0.08495161	104.851574	0	0.08187134	0.08808296	2238148.18	0	0.01576238	99.9264543	1359.69829
1357	0.08489581	104.890592	0	0.08181862	0.08802402	2237058.22	0	0.01573967	99.9263696	1358.13523
1358	0.08494201	104.849439	0	0.08186201	0.08807309	2238136.75	0	0.01576123	99.9264787	1360.14969
1359	0.08500708	104.94454	0	0.08192757	0.08813759	2237856.48	0	0.0157447	99.9264022	1358.73537
1360	0.08495704	104.854734	0	0.08187667	0.08808849	2238136.71	0	0.01576246	99.926469	1359.97027
1361	0.08490384	104.880978	0	0.08182609	0.08803262	2238121.97	0	0.01574474	99.9264053	1358.79328
1362	0.084915	104.863574	0	0.08183635	0.0880447	2238080.85	0	0.01575175	99.9264367	1359.37357
1363	0.08501314	104.962731	0	0.08193396	0.08814333	2237009.27	0	0.01574026	99.9263742	1358.21889
1364	0.08493917	104.849685	0	0.08185928	0.08807014	2238136.67	0	0.01576064	99.9264774	1360.12676
1365	0.08496411	104.860791	0	0.08188367	0.08809562	2238115.36	0	0.01576199	99.9264777	1360.1309
1366	0.08498216	104.886222	0	0.08190183	0.08811355	2238076.85	0	0.01575774	99.9264636	1359.87027
1367	0.08496856	104.86618	0	0.08188812	0.08810007	2238132.7	0	0.01576128	99.9264807	1360.1876
1368	0.08486282	104.974298	0	0.08178921	0.08798734	2237981.4	0	0.01570802	99.9262331	1355.62073
1369	0.08497566	104.87534	0	0.08189524	0.08810715	2238076.34	0	0.01575979	99.9264713	1360.01245
1370	0.0849512	104.851717	0	0.08187095	0.08808254	2238148.57	0	0.0157623	99.9264836	1360.24118
1371	0.08497549	104.874829	0	0.08189506	0.08810699	2237837.99	0	0.01575987	99.926456	1359.7298
1372	0.08497565	104.875138	0	0.08189523	0.08810715	2237973.93	0	0.01575982	99.9264648	1359.89284
1373	0.08496379	104.864607	0	0.08188347	0.08809518	2238147.39	0	0.01576138	99.926483	1360.22963
1374	0.08493645	104.849812	0	0.08185666	0.08806732	2238122.73	0	0.01576004	99.9264735	1360.05334
1375	0.0849742	104.87293	0	0.08189376	0.08810571	2237777.55	0	0.0157602	99.9264506	1359.63038
1376	0.08498411	104.889455	0	0.08190381	0.08811548	2237928.45	0	0.01575708	99.9264557	1359.72438
1377	0.08496807	104.864998	0	0.08188761	0.0880996	2238044.85	0	0.01576146	99.9264689	1359.9687
1378	0.08495063	104.851281	0	0.08187038	0.08808196	2238148.02	0	0.0157623	99.9264794	1360.16283
1379	0.08496353	104.861384	0	0.08188312	0.08809501	2238144.73	0	0.01576187	99.9264845	1360.25745
1380	0.08496315	104.860063	0	0.08188272	0.08809466	2238135.58	0	0.01576206	99.9264825	1360.22089
1381	0.08496695	104.863747	0	0.0818865	0.08809848	2238074.64	0	0.01576163	99.9264723	1360.0309
1382	0.08500018	104.925718	0	0.08192037	0.08813102	2237985.28	0	0.01574915	99.9264238	1359.13555
1383	0.08501289	104.962013	0	0.08193369	0.08814308	2237459.16	0	0.01574045	99.9263792	1358.31121
1384	0.08499878	104.92175	0	0.0819189	0.08812969	2237737	0	0.01575004	99.9264238	1359.13529
1385	0.08495903	104.858362	0	0.0818787	0.08809044	2238147.89	0	0.01576207	99.9264859	1360.28253
1386	0.0849657	104.862412	0	0.08188525	0.08809723	2238098.99	0	0.0157618	99.9264755	1360.09013
1387	0.08499154	104.904561	0	0.08191142	0.08812271	2237771.38	0	0.01575389	99.9264399	1359.43196
1388	0.08497796	104.878881	0	0.08189756	0.08810942	2238058.29	0	0.01575915	99.9264681	1359.95404
1389	0.08497925	104.880851	0	0.08189886	0.08811069	2237978.91	0	0.01575877	99.926463	1359.85923
1390	0.08497392	104.872627	0	0.08189348	0.08810543	2238023.14	0	0.01576026	99.9264686	1359.964
1391	0.08498759	104.896355	0	0.08190736	0.08811887	2237982.87	0	0.01575566	99.926452	1359.65665
1392	0.084961	104.857856	0	0.08188058	0.0880925	2238123.33	0	0.01576228	99.9264755	1360.09148
1393	0.08496019	104.85715	0	0.08187978	0.08809167	2238124.35	0	0.01576234	99.9264737	1360.05842
1394	0.08497174	104.870194	0	0.08189131	0.08810324	2238126.27	0	0.01576067	99.9264779	1360.13567
1395	0.08499204	104.906094	0	0.08191194	0.08812318	2238034.17	0	0.01575358	99.9264444	1359.51664
1396	0.0849833	104.889352	0	0.08190302	0.08811464	2238122.49	0	0.01575717	99.9264628	1359.85509

© 2024 Zhang X et al. *JAMA Pediatrics*.

1397	0.08497959	104.882549	0	0.08189924	0.088111	2238124.06	0	0.01575849	99.9264687	1359.96523
1398	0.08496472	104.86253	0	0.08188431	0.08809621	2238143.67	0	0.01576175	99.9264839	1360.24585
1399	0.08498249	104.886357	0	0.08190215	0.08811389	2237681.34	0	0.0157577	99.9264482	1359.58612
1400	0.08494896	104.851628	0	0.08186878	0.08808022	2238148.29	0	0.01576202	99.926485	1360.26727
1401	0.08501576	104.971735	0	0.08193675	0.08814577	2237865.16	0	0.01573809	99.9263722	1358.18286
1402	0.08498551	104.897337	0	0.08190538	0.08811668	2238139.22	0	0.01575573	99.9264567	1359.74285
1403	0.08499832	104.921789	0	0.08191846	0.08812921	2238083.02	0	0.01575011	99.9264297	1359.24462
1404	0.08496126	104.859407	0	0.08188088	0.08809272	2238146.39	0	0.01576206	99.9264855	1360.27495
1405	0.08498406	104.890043	0	0.08190378	0.08811541	2238099.08	0	0.01575699	99.9264612	1359.82599
1406	0.08496535	104.864774	0	0.08188498	0.08809679	2238146.17	0	0.01576142	99.926483	1360.22956
1407	0.08488548	104.914097	0	0.08180933	0.08801261	2238074.72	0	0.01573086	99.9263402	1357.59324
1408	0.08496165	104.85884	0	0.08188123	0.08809314	2238141.98	0	0.01576217	99.9264842	1360.25272
1409	0.08498596	104.892885	0	0.08190569	0.08811729	2237609.37	0	0.01575637	99.9264433	1359.49631
1410	0.08497233	104.870362	0	0.08189188	0.08810385	2237962.4	0	0.01576064	99.9264635	1359.8687
1411	0.0849728	104.871534	0	0.08189237	0.08810431	2238119.87	0	0.01576045	99.9264766	1360.11079
1412	0.0850079	104.948157	0	0.08192847	0.08813834	2238064.74	0	0.01574393	99.926401	1358.71416
1413	0.08488288	104.917935	0	0.08180694	0.08800981	2237728.74	0	0.01572886	99.9263283	1357.37352
1414	0.08500227	104.931325	0	0.08192256	0.08813302	2237987.13	0	0.01574785	99.926418	1359.0277
1415	0.08490756	104.873798	0	0.08182947	0.08803669	2238107.31	0	0.01574737	99.9264173	1359.01427
1416	0.08496798	104.866787	0	0.08188758	0.08809946	2238143.39	0	0.01576118	99.9264816	1360.20419
1417	0.08494309	104.850902	0	0.08186309	0.08807416	2238146.57	0	0.0157612	99.9264816	1360.20415
1418	0.084976	104.876734	0	0.0818956	0.08810745	2238128.77	0	0.01575956	99.9264736	1360.05604
1419	0.08497534	104.875257	0	0.08189493	0.08810682	2238117.18	0	0.01575981	99.9264739	1360.06212
1420	0.08494339	104.849178	0	0.08186333	0.08807452	2227173.34	0	0.01576152	99.9219342	1280.97012
1421	0.08493858	104.849121	0	0.08185868	0.08806954	2212758.45	0	0.0157606	99.9220057	1282.14473
1422	0.08495055	104.851462	0	0.08187031	0.08808187	2238148.37	0	0.01576226	99.9264837	1360.24213
1423	0.08496451	104.861315	0	0.08188407	0.08809603	2238127.4	0	0.01576193	99.9264808	1360.18881
1424	0.08495684	104.854532	0	0.08187647	0.08808829	2238076.9	0	0.01576248	99.9263622	1357.99807
1425	0.08495687	104.854552	0	0.0818765	0.08808832	2238075.98	0	0.01576248	99.9263622	1357.99803
1426	0.08492818	104.852803	0	0.08184876	0.08805866	2238100.66	0	0.01575754	99.9264625	1359.85096
1427	0.08492608	104.855623	0	0.08184681	0.08805641	2238134.75	0	0.01575652	99.92646	1359.80402
1428	0.08494195	104.850999	0	0.08186199	0.08807298	2238146.26	0	0.01576097	99.9264807	1360.1865
1429	0.08492563	104.859714	0	0.0818465	0.08805582	2238143.02	0	0.01575576	99.9264569	1359.74681
1430	0.08496622	104.862971	0	0.08188577	0.08809775	2238096.95	0	0.01576173	99.9264755	1360.09041
1431	0.08496515	104.86185	0	0.0818847	0.08809667	2238108.54	0	0.01576187	99.926477	1360.11786
1432	0.084965	104.861728	0	0.08188455	0.08809652	2238115.84	0	0.01576188	99.9264784	1360.14498
1433	0.08496488	104.862217	0	0.08188446	0.08809638	2238140.53	0	0.0157618	99.9264835	1360.23808
1434	0.0849493	104.851338	0	0.0818691	0.08808058	2238148.19	0	0.01576211	99.9264848	1360.26248
1435	0.08496929	104.866395	0	0.08188883	0.08810082	2237822.39	0	0.01576125	99.9264428	1359.48581
1436	0.08497181	104.870477	0	0.08189138	0.08810331	2238130.76	0	0.01576062	99.9264781	1360.13983
1437	0.0849719	104.869895	0	0.08189145	0.08810342	2238071.73	0	0.01576071	99.9264732	1360.04743
1438	0.08494365	104.8501	0	0.08186361	0.08807477	2238145.08	0	0.01576142	99.9264818	1360.20827

© 2024 Zhang X et al. *JAMA Pediatrics*.

1439	0.08495406	104.85306	0	0.08187374	0.08808545	2238148.3	0	0.01576243	99.9264829	1360.22703
1440	0.08498638	104.893636	0	0.08190612	0.0881177	2235030.73	0	0.01575621	99.9263654	1358.05778
1441	0.08495156	104.851521	0	0.08187129	0.08808291	2238146.79	0	0.01576238	99.9263822	1358.36773
1442	0.08496541	104.862466	0	0.08188497	0.08809693	2238134.64	0	0.01576178	99.9264823	1360.21658
1443	0.08496439	104.861319	0	0.08188396	0.08809591	2238133.94	0	0.01576192	99.9264823	1360.21592
1444	0.08496376	104.860276	0	0.08188331	0.08809528	2237267	0	0.01576205	99.926248	1355.89558
1445	0.0849361	104.849585	0	0.08185631	0.08806697	2238055.12	0	0.01576	99.9264643	1359.88404
1446	0.08496623	104.863618	0	0.0818858	0.08809774	2238138.29	0	0.01576163	99.9264826	1360.22199
1447	0.08493669	104.850217	0	0.0818569	0.08806756	2238136.51	0	0.01576003	99.9264753	1360.08734
1448	0.08495175	104.851964	0	0.08187149	0.0880831	2238148.68	0	0.01576233	99.9264837	1360.24323
1449	0.08496801	104.865376	0	0.08188756	0.08809952	2238129.72	0	0.0157614	99.9264807	1360.1868
1450	0.08493331	104.850172	0	0.08185363	0.08806405	2235274.42	0	0.01575926	99.926192	1354.86749
1451	0.08492953	104.851629	0	0.08185003	0.0880601	2170612.59	0	0.01575809	99.9221041	1283.76481
1452	0.08494777	104.850262	0	0.08186759	0.08807902	2238143.97	0	0.01576206	99.9264786	1360.14755
1453	0.08490007	104.886757	0	0.08182261	0.08802854	2238116.15	0	0.01574221	99.9263935	1358.57489
1454	0.08493508	104.850651	0	0.08185536	0.08806588	2238135.56	0	0.01575961	99.9264735	1360.05418
1455	0.08494294	104.851936	0	0.08186298	0.08807398	2238147.29	0	0.01576101	99.9264811	1360.19401
1456	0.08498695	104.895916	0	0.08190673	0.08811821	2238104.27	0	0.0157558	99.926456	1359.73077
1457	0.08500736	104.945575	0	0.08192788	0.08813785	2237946.26	0	0.01574447	99.9264021	1358.73509
1458	0.08491707	104.860695	0	0.08183826	0.08804692	2237527.39	0	0.01575291	99.926426	1359.17491
1459	0.08485374	105.003952	0	0.08178131	0.08797706	2237916.72	0	0.0156972	99.9261821	1354.68477
1460	0.08495716	104.858664	0	0.0818769	0.08808849	2238148.48	0	0.01576187	99.9264852	1360.2706
1461	0.08498282	104.887585	0	0.08190251	0.0881142	2238091.9	0	0.01575748	99.926463	1359.86029
1462	0.08495304	104.8528	0	0.08187275	0.08808441	2238148.7	0	0.01576236	99.9264853	1360.27169
1463	0.08493971	104.849272	0	0.08185979	0.08807071	2238118.98	0	0.01576081	99.9264741	1360.06495
1464	0.08492328	104.855653	0	0.08184411	0.08805351	2238037.79	0	0.01575569	99.9264521	1359.65895
1465	0.08489563	104.891481	0	0.08181847	0.08802381	2237952.64	0	0.01573944	99.9263788	1358.30365
1466	0.08496707	104.864401	0	0.08188663	0.08809859	2238134.37	0	0.01576153	99.9264817	1360.20553
1467	0.08495344	104.85407	0	0.08187318	0.08808479	2238148.7	0	0.01576221	99.9264863	1360.29005
1468	0.08499364	104.91025	0	0.08191361	0.08812472	2238082.43	0	0.0157527	99.9264415	1359.46232
1469	0.08496647	104.863892	0	0.08188603	0.08809798	2238138.17	0	0.01576159	99.9264825	1360.21967
1470	0.0849403	104.852495	0	0.08186044	0.08807122	2238146.8	0	0.01576043	99.9264785	1360.14568
1471	0.08494893	104.850498	0	0.08186872	0.08808022	2238138.73	0	0.01576219	99.9264572	1359.75208
1472	0.08493181	104.851571	0	0.08185223	0.08806247	2238128.77	0	0.01575868	99.9264691	1359.97236
1473	0.08499406	104.910283	0	0.08191402	0.08812515	2237758.14	0	0.01575263	99.9264347	1359.33705
1474	0.08495332	104.852748	0	0.08187302	0.0880847	2238148.63	0	0.0157624	99.926484	1360.24856
1475	0.08498706	104.895249	0	0.08190682	0.08811835	2237971.46	0	0.01575589	99.9264526	1359.66774
1476	0.08499752	104.922518	0	0.08191771	0.08812836	2238125.82	0	0.01575014	99.9264306	1359.25997
1477	0.08494631	104.850368	0	0.08186618	0.08807751	2238146.43	0	0.01576182	99.9264832	1360.23325
1478	0.08499505	104.912507	0	0.08191503	0.0881261	2237470.77	0	0.01575213	99.9264273	1359.19917
1479	0.08497232	104.870964	0	0.08189188	0.08810382	2238124.86	0	0.01576054	99.9264773	1360.12485
1480	0.08487009	104.955584	0	0.08179569	0.08799544	2238087.82	0	0.01571559	99.926269	1356.28118

© 2024 Zhang X et al. *JAMA Pediatrics*.

1481	0.08495636	104.854217	0	0.08187599	0.0880878	2238120.86	0	0.01576248	99.9264267	1359.18905
1482	0.08491179	104.867493	0	0.08183337	0.08804126	2238089.36	0	0.01574998	99.9264289	1359.22897
1483	0.08497261	104.870819	0	0.08189217	0.08810413	2238044.02	0	0.01576056	99.9264706	1359.99974
1484	0.08495671	104.854694	0	0.08187635	0.08808815	2238145.67	0	0.01576244	99.9264826	1360.22133
1485	0.08492849	104.852994	0	0.08184907	0.08805898	2238120.82	0	0.0157576	99.926464	1359.87777
1486	0.08492979	104.852352	0	0.0818503	0.08806035	2238123.2	0	0.01575804	99.926466	1359.91539
1487	0.08495151	104.852233	0	0.08187126	0.08808284	2238148.68	0	0.01576226	99.9264855	1360.27515
1488	0.08495036	104.85177	0	0.08187013	0.08808166	2238148.5	0	0.01576219	99.9264852	1360.27021
1489	0.08496168	104.858515	0	0.08188126	0.08809318	2238129.18	0	0.01576222	99.9264793	1360.16147
1490	0.08494824	104.851031	0	0.08186806	0.08807949	2238147.82	0	0.01576201	99.9264844	1360.256
1491	0.08496998	104.868309	0	0.08188955	0.08810148	2238136.58	0	0.01576096	99.92648	1360.1745
1492	0.08494807	104.850893	0	0.0818679	0.08807932	2238147.63	0	0.01576201	99.9264842	1360.25173
1493	0.08490987	104.869054	0	0.08183156	0.08803922	2237943.35	0	0.01574901	99.9264215	1359.09335
1494	0.08494353	104.849305	0	0.08186347	0.08807467	2238119.24	0	0.01576152	99.9264706	1360.00028
1495	0.08494913	104.851157	0	0.08186893	0.08808041	2238148.02	0	0.01576212	99.9264844	1360.25607
1496	0.08499875	104.921556	0	0.08191887	0.08812967	2237213.7	0	0.01575007	99.9264155	1358.98243
1497	0.08495621	104.854622	0	0.08187586	0.08808763	2238147.55	0	0.01576241	99.9264853	1360.27183
1498	0.08498961	104.901046	0	0.08190945	0.08812082	2238075.62	0	0.01575469	99.9264503	1359.62522
1499	0.08497503	104.877492	0	0.0818947	0.08810643	2238141.97	0	0.01575947	99.926474	1360.0632
1500	0.08500668	104.943728	0	0.08192717	0.08813721	2237982.25	0	0.01574492	99.9264046	1358.7808
1501	0.08499131	104.904791	0	0.0819112	0.08812246	2238076.49	0	0.01575388	99.9264467	1359.55908
1502	0.08497688	104.87694	0	0.08189646	0.08810836	2237764.98	0	0.0157595	99.9264522	1359.6603
1503	0.08496755	104.867167	0	0.08188717	0.088099	2238145.19	0	0.01576111	99.9264815	1360.20267
1504	0.08494857	104.852984	0	0.08186844	0.08807977	2238148.47	0	0.01576175	99.9264845	1360.25757
1505	0.08494973	104.851477	0	0.08186952	0.08808102	2238148.32	0	0.01576215	99.9264849	1360.26469
1506	0.08498814	104.898101	0	0.08190795	0.08811939	2238088.99	0	0.01575532	99.9264535	1359.68383
1507	0.08494136	104.849614	0	0.08186139	0.08807241	2238139.86	0	0.01576108	99.9264794	1360.16216
1508	0.08499599	104.915107	0	0.08191602	0.088127	2237971.44	0	0.01575157	99.9264343	1359.3299
1509	0.08500025	104.925316	0	0.08192043	0.0881311	2234622.25	0	0.0157492	99.9263732	1358.20195
1510	0.08501453	104.967069	0	0.08193542	0.08814463	2049229.9	0	0.01573919	99.9247287	1328.52758
1511	0.08499345	104.911296	0	0.08191346	0.08812449	2238119.51	0	0.01575256	99.9264416	1359.46459
1512	0.08498829	104.897502	0	0.08190808	0.08811956	2236646.01	0	0.0157554	99.9264149	1358.9714
1513	0.08498411	104.889288	0	0.0819038	0.08811547	2237181.63	0	0.01575711	99.92643	1359.24879
1514	0.08499157	104.904642	0	0.08191145	0.08812274	2237783.9	0	0.01575387	99.9264401	1359.43598
1515	0.08497419	104.873774	0	0.08189378	0.08810567	2238126.25	0	0.01576007	99.9264756	1360.09278
1516	0.08497779	104.878632	0	0.08189739	0.08810926	2238063.39	0	0.01575919	99.9264685	1359.962
1517	0.08498828	104.897693	0	0.08190807	0.08811955	2237921.25	0	0.01575537	99.9264492	1359.60443
1518	0.0849882	104.897576	0	0.08190799	0.08811946	2237960.93	0	0.0157554	99.9264504	1359.62619
1519	0.08499169	104.904821	0	0.08191157	0.08812286	2237457.94	0	0.01575383	99.9264326	1359.29765
1520	0.08499047	104.902233	0	0.08191031	0.08812167	2237802.38	0	0.0157544	99.9264425	1359.48028
1521	0.08500202	104.93092	0	0.0819223	0.08813277	2238034.61	0	0.01574797	99.9264192	1359.04961
1522	0.08498937	104.899813	0	0.08190919	0.08812061	2237555.23	0	0.01575491	99.9264381	1359.3994

© 2024 Zhang X et al. *JAMA Pediatrics*.

1523	0.08498089	104.883573	0	0.08190052	0.08811231	2237874.2	0	0.01575825	99.926457	1359.74791
1524	0.08499504	104.912644	0	0.08191503	0.08812609	2237849.9	0	0.01575211	99.9264344	1359.33092
1525	0.08500513	104.938747	0	0.08192552	0.08813575	2237651.95	0	0.01574606	99.9264057	1358.80135
1526	0.08498637	104.894212	0	0.08190612	0.08811767	2238068.17	0	0.01575612	99.9264564	1359.73795
1527	0.08498077	104.883364	0	0.08190041	0.0881122	2237842.11	0	0.01575829	99.9264557	1359.72393
1528	0.08500503	104.938451	0	0.08192542	0.08813566	2237588.42	0	0.01574613	99.9264052	1358.79217
1529	0.08497951	104.8813	0	0.08189913	0.08811095	2237985.73	0	0.01575869	99.9264631	1359.86084
1530	0.08497843	104.879527	0	0.08189803	0.08810989	2237998.5	0	0.01575902	99.9264646	1359.88968
1531	0.08496631	104.863149	0	0.08188586	0.08809784	2238113.44	0	0.0157617	99.9264785	1360.14573
1532	0.08499184	104.906064	0	0.08191175	0.08812298	2238081.27	0	0.01575361	99.9264456	1359.53842
1533	0.08499424	104.911294	0	0.08191422	0.08812531	2238050.53	0	0.01575244	99.9264397	1359.42932
1534	0.08499686	104.917496	0	0.08191693	0.08812783	2238032.87	0	0.01575104	99.9264331	1359.30722
1535	0.08493593	104.850692	0	0.08185618	0.08806676	2238139.02	0	0.01575979	99.9264747	1360.07569
1536	0.08496412	104.862428	0	0.08188373	0.08809559	2238145.41	0	0.01576174	99.9264842	1360.25106
1537	0.08499491	104.912232	0	0.08191489	0.08812596	2237692.77	0	0.01575219	99.9264317	1359.28194
1538	0.08498559	104.892153	0	0.08190531	0.08811693	2237560.23	0	0.01575652	99.9264421	1359.47367
1539	0.0849947	104.911659	0	0.08191468	0.08812577	2237135.84	0	0.01575231	99.9264214	1359.09151
1540	0.08499435	104.910942	0	0.08191431	0.08812542	2237758.44	0	0.01575248	99.9264342	1359.32646
1541	0.08498069	104.883489	0	0.08190033	0.08811211	2238058.61	0	0.01575827	99.9264649	1359.89562
1542	0.08496605	104.862988	0	0.08188561	0.08809757	2238126.15	0	0.01576172	99.9264807	1360.18686
1543	0.08497347	104.872771	0	0.08189305	0.08810496	2238128.16	0	0.01576024	99.9264764	1360.10816
1544	0.08498849	104.897973	0	0.08190828	0.08811975	2237588.44	0	0.0157553	99.9264401	1359.43574
1545	0.08500176	104.92942	0	0.081922	0.08813254	2237505.17	0	0.01574825	99.926413	1358.93539
1546	0.08500649	104.942593	0	0.08192695	0.08813704	2237524.64	0	0.01574515	99.9264002	1358.7001
1547	0.084966	104.862927	0	0.08188555	0.08809752	2238126.08	0	0.01576173	99.9264807	1360.18668
1548	0.08498573	104.893025	0	0.08190548	0.08811704	2238077.69	0	0.01575637	99.9264578	1359.76321
1549	0.08501175	104.958384	0	0.08193248	0.08814201	2237384.82	0	0.01574133	99.9263823	1358.36932
1550	0.0849939	104.911111	0	0.08191388	0.08812495	2238094.49	0	0.01575252	99.926441	1359.4522
1551	0.08495466	104.85421	0	0.08187436	0.08808605	2238148.53	0	0.01576232	99.9264863	1360.29088
1552	0.08490687	104.87282	0	0.08182878	0.08803601	2237599.92	0	0.01574724	99.9264078	1358.84027
1553	0.08496872	104.866466	0	0.08188829	0.08810023	2238134.26	0	0.01576124	99.9264808	1360.18853
1554	0.08491528	104.863864	0	0.08183664	0.08804498	2238108.45	0	0.01575181	99.9264377	1359.39121
1555	0.08493747	104.84977	0	0.08185764	0.08806838	2238130.95	0	0.01576027	99.9264753	1360.0878
1556	0.08491994	104.858082	0	0.08184096	0.08804998	2237741.82	0	0.01575427	99.9264363	1359.36654
1557	0.08493904	104.849435	0	0.08185914	0.08807001	2238127.46	0	0.01576065	99.9264757	1360.09449
1558	0.08496232	104.859	0	0.08188188	0.08809382	2238106.26	0	0.01576218	99.926472	1360.02548
1559	0.08493972	104.850017	0	0.08185981	0.0880707	2238141.69	0	0.0157607	99.9264786	1360.14846
1560	0.08491827	104.860121	0	0.08183941	0.08804818	2238074.1	0	0.01575341	99.9264439	1359.50609
1561	0.08491371	104.864417	0	0.08183513	0.08804334	2237943.19	0	0.01575116	99.9264307	1359.26322
1562	0.08493241	104.850881	0	0.08185278	0.0880631	2238108.88	0	0.01575893	99.9264684	1359.95935
1563	0.08496884	104.865894	0	0.08188838	0.08810037	2238008.61	0	0.01576133	99.926465	1359.89613
1564	0.08497519	104.874764	0	0.08189477	0.08810668	2238099.27	0	0.01575989	99.9264731	1360.04631

© 2024 Zhang X et al. *JAMA Pediatrics*.

1565	0.08493579	104.849934	0	0.08185602	0.08806664	2238120.24	0	0.01575988	99.9264727	1360.0383
1566	0.08497445	104.874051	0	0.08189403	0.08810593	2238123.19	0	0.01576002	99.9264752	1360.08519
1567	0.08496742	104.865119	0	0.08188699	0.08809892	2238138.69	0	0.01576142	99.926482	1360.21098
1568	0.08494637	104.850696	0	0.08186625	0.08807756	2238147.16	0	0.01576178	99.9264837	1360.24235
1569	0.08492529	104.854129	0	0.08184601	0.08805564	2237959.89	0	0.01575653	99.9264515	1359.64703
1570	0.08492022	104.858087	0	0.08184123	0.08805026	2238025.49	0	0.01575436	99.9264463	1359.55182
1571	0.084948	104.856346	0	0.08186799	0.08807908	2238148.56	0	0.01576115	99.926482	1360.21168
1572	0.08493311	104.850598	0	0.08185345	0.08806384	2238106.71	0	0.01575915	99.9264689	1359.96916
1573	0.08492445	104.854583	0	0.08184521	0.08805475	2237635.12	0	0.01575621	99.9264356	1359.35395
1574	0.08491953	104.858792	0	0.08184059	0.08804953	2238051.26	0	0.01575403	99.9264458	1359.54234
1575	0.08500398	104.935608	0	0.08192433	0.08813466	2237765.54	0	0.01574681	99.9264104	1358.88789
1576	0.08496902	104.866748	0	0.08188858	0.08810053	2238132.24	0	0.0157612	99.9264804	1360.18157
1577	0.08497324	104.875117	0	0.0818929	0.08810465	2238143.29	0	0.01575988	99.9264759	1360.09896
1578	0.08497324	104.873374	0	0.08189285	0.08810471	2238138.44	0	0.01576015	99.9264768	1360.11507
1579	0.08497825	104.879537	0	0.08189786	0.0881097	2238090.25	0	0.01575903	99.9264693	1359.9769
1580	0.08496514	104.862144	0	0.0818847	0.08809666	2238134.11	0	0.01576182	99.9264822	1360.21565
1581	0.08499319	104.908215	0	0.08191312	0.08812431	2237565.03	0	0.01575308	99.9264325	1359.29556
1582	0.08497361	104.873863	0	0.08189321	0.08810507	2238137.96	0	0.01576007	99.9264764	1360.10779
1583	0.0849238	104.855187	0	0.0818446	0.08805406	2237993.61	0	0.01575592	99.9264511	1359.63959
1584	0.08491511	104.866346	0	0.08183654	0.08804473	2238132.17	0	0.01575136	99.9264362	1359.36457
1585	0.08496195	104.859049	0	0.08188153	0.08809344	2238140.68	0	0.01576215	99.9264838	1360.24528
1586	0.08495854	104.857747	0	0.08187821	0.08808996	2238147.92	0	0.01576212	99.926486	1360.28602
1587	0.08496569	104.862455	0	0.08188524	0.08809722	2238112.11	0	0.01576179	99.926478	1360.1373
1588	0.08496374	104.862126	0	0.08188335	0.0880952	2238145.75	0	0.01576177	99.9264843	1360.25427
1589	0.08497634	104.876162	0	0.08189592	0.08810783	2237944.77	0	0.01575964	99.9264628	1359.85603
1590	0.0849406	104.849761	0	0.08186066	0.08807162	2238140.73	0	0.01576091	99.9264791	1360.15783
1591	0.08496725	104.864933	0	0.08188682	0.08809876	2238138.96	0	0.01576145	99.9264821	1360.21329
1592	0.08492439	104.854774	0	0.08184516	0.08805468	2237995.3	0	0.01575616	99.926452	1359.65633
1593	0.08490505	104.876465	0	0.08182712	0.088034	2238070.08	0	0.01574593	99.92641	1358.88069
1594	0.08495676	104.85465	0	0.0818764	0.0880882	2238144.31	0	0.01576245	99.9264803	1360.1789
1595	0.08496634	104.863036	0	0.08188588	0.08809787	2238058.55	0	0.01576172	99.9264687	1359.96498
1596	0.0849592	104.856521	0	0.0818788	0.08809067	2238141.48	0	0.01576236	99.9264824	1360.21932
1597	0.08494558	104.85015	0	0.08186547	0.08807676	2238145.58	0	0.01576174	99.9264826	1360.2224
1598	0.08501156	104.957874	0	0.08193229	0.08814184	2237570.98	0	0.01574146	99.9263847	1358.41348
1599	0.08499824	104.921495	0	0.08191838	0.08812914	2238078.38	0	0.01575017	99.9264299	1359.24832
1600	0.08489012	104.901975	0	0.08181346	0.08801778	2237780.71	0	0.01573513	99.9263574	1357.90893
1601	0.0849496	104.852537	0	0.08186942	0.08808085	2238148.54	0	0.01576197	99.9264853	1360.27143
1602	0.08490366	104.877722	0	0.08182582	0.08803253	2237891.7	0	0.01574516	99.9264037	1358.76477
1603	0.08491567	104.862373	0	0.08183696	0.08804542	2237992.12	0	0.01575217	99.9264362	1359.36476
1604	0.08495436	104.855374	0	0.0818741	0.08808569	2238148.66	0	0.0157621	99.9264861	1360.28678
1605	0.08497034	104.868299	0	0.0818899	0.08810186	2238126.85	0	0.01576096	99.926479	1360.15622
1606	0.08497033	104.868692	0	0.0818899	0.08810183	2238135.41	0	0.0157609	99.9264797	1360.16811

© 2024 Zhang X et al. *JAMA Pediatrics*.

1607	0.08495994	104.856938	0	0.08187953	0.08809143	2238123.56	0	0.01576235	99.9264724	1360.03361
1608	0.08495269	104.852797	0	0.08187241	0.08808404	2238148.72	0	0.01576232	99.9264857	1360.27964
1609	0.08493979	104.849207	0	0.08185986	0.0880708	2238108.45	0	0.01576084	99.9264718	1360.02177
1610	0.08498814	104.897383	0	0.08190792	0.0881194	2237912.46	0	0.01575544	99.9264492	1359.60469
1611	0.08493162	104.851286	0	0.08185203	0.08806227	2238115.15	0	0.01575868	99.926468	1359.95159
1612	0.08494575	104.849647	0	0.08186562	0.08807695	2238127.58	0	0.01576184	99.9264684	1359.95927
1613	0.08499815	104.920701	0	0.08191826	0.08812907	2238028.4	0	0.01575032	99.9264298	1359.24509
1614	0.0849481	104.850335	0	0.08186791	0.08807937	2238143.89	0	0.0157621	99.9264773	1360.12375
1615	0.08499778	104.919828	0	0.08191788	0.08812871	2238037.3	0	0.01575052	99.9264308	1359.26474
1616	0.08498864	104.898633	0	0.08190844	0.08811989	2238021.63	0	0.01575518	99.9264511	1359.63954
1617	0.08498516	104.891593	0	0.08190488	0.0881165	2238014.55	0	0.01575665	99.926457	1359.74791
1618	0.08502364	104.999506	0	0.08194516	0.08815309	2237877.05	0	0.01573116	99.9263404	1357.59612
1619	0.0849959	104.915813	0	0.08191596	0.08812689	2238087.72	0	0.01575147	99.926436	1359.36075
1620	0.08498386	104.88942	0	0.08190357	0.08811522	2238080.43	0	0.01575711	99.926461	1359.82331
1621	0.08496072	104.857867	0	0.08188032	0.08809221	2238140.77	0	0.01576226	99.9264834	1360.23753
1622	0.08500799	104.947166	0	0.08192853	0.08813846	2237815.61	0	0.01574407	99.9263988	1358.67437
1623	0.08496828	104.865268	0	0.08188782	0.08809981	2238061.35	0	0.01576142	99.9264714	1360.01494
1624	0.08499703	104.917255	0	0.08191708	0.08812801	2237238.4	0	0.01575105	99.9264193	1359.0529
1625	0.08500232	104.930997	0	0.08192259	0.08813307	2237687.04	0	0.01574789	99.926414	1358.95451
1626	0.08495351	104.852719	0	0.0818732	0.0880849	2238148.5	0	0.01576242	99.9264819	1360.20992
1627	0.08494267	104.849944	0	0.08186266	0.08807376	2238143.93	0	0.01576127	99.926481	1360.19333
1628	0.084931	104.851287	0	0.08185144	0.08806163	2238084.95	0	0.01575852	99.926465	1359.89737
1629	0.08494626	104.849891	0	0.08186613	0.08807748	2238141.47	0	0.01576189	99.9264788	1360.1523
1630	0.08497984	104.882151	0	0.08189947	0.08811127	2238081.88	0	0.01575854	99.926467	1359.93352
1631	0.08500109	104.929135	0	0.08192135	0.08813186	2238084.06	0	0.01574843	99.926422	1359.10225
1632	0.08500292	104.932611	0	0.08192321	0.08813364	2237667.93	0	0.01574751	99.9264121	1358.91988
1633	0.08492784	104.852727	0	0.08184843	0.08805832	2238051.19	0	0.01575746	99.9264593	1359.79112
1634	0.08496874	104.866614	0	0.08188831	0.08810025	2238136.18	0	0.01576121	99.9264809	1360.19131
1635	0.0850123	104.960379	0	0.08193308	0.08814253	2237790.74	0	0.01574087	99.9263842	1358.40364
1636	0.084947	104.8502	0	0.08186684	0.08807823	2238145.15	0	0.01576195	99.9264816	1360.20452
1637	0.08498491	104.891274	0	0.08190463	0.08811624	2238059.96	0	0.01575673	99.9264587	1359.78065
1638	0.08499961	104.92451	0	0.08191979	0.08813047	2238034.19	0	0.01574945	99.9264259	1359.17414
1639	0.08493864	104.853444	0	0.08185888	0.08806948	2238146.69	0	0.01575995	99.9264763	1360.10601
1640	0.08497284	104.872767	0	0.08189244	0.08810431	2238138.57	0	0.01576026	99.9264773	1360.12333
1641	0.08494921	104.851049	0	0.08186901	0.0880805	2238147.87	0	0.01576214	99.9264839	1360.24659
1642	0.08496597	104.86305	0	0.08188553	0.08809749	2238133.03	0	0.01576171	99.9264819	1360.20905
1643	0.08496366	104.860826	0	0.08188324	0.08809516	2238140.37	0	0.01576196	99.9264838	1360.24365
1644	0.08497197	104.869859	0	0.08189152	0.08810349	2237942.23	0	0.01576072	99.9264614	1359.83028
1645	0.08496696	104.863701	0	0.0818865	0.08809849	2238025.62	0	0.01576163	99.9264642	1359.88197
1646	0.08500634	104.942158	0	0.0819268	0.08813691	2237455.92	0	0.01574525	99.9263999	1358.69312
1647	0.08487214	104.946279	0	0.08179739	0.08799783	2237956.91	0	0.01571828	99.9262808	1356.4988
1648	0.08498775	104.896998	0	0.08190754	0.08811902	2238062.47	0	0.01575554	99.9264537	1359.68817

© 2024 Zhang X et al. *JAMA Pediatrics*.

1649	0.08491498	104.863126	0	0.08183632	0.08804469	2237999.59	0	0.01575181	99.9264349	1359.34016
1650	0.0849152	104.864278	0	0.08183657	0.08804488	2238115.43	0	0.01575171	99.9264374	1359.38658
1651	0.08492898	104.853439	0	0.08184955	0.08805948	2238133.59	0	0.01575766	99.926465	1359.8973
1652	0.08496719	104.86628	0	0.0818868	0.08809865	2238144.7	0	0.01576124	99.926482	1360.21195
1653	0.08495921	104.856428	0	0.08187881	0.08809068	2238137.13	0	0.01576238	99.9264793	1360.16029
1654	0.08496903	104.866293	0	0.08188858	0.08810056	2238102.15	0	0.01576127	99.9264769	1360.11729
1655	0.08492108	104.857986	0	0.08184205	0.08805116	2238105.74	0	0.01575465	99.9264504	1359.62683
1656	0.08487359	104.942727	0	0.08179869	0.08799944	2238005.33	0	0.0157197	99.9262877	1356.62657
1657	0.08492992	104.852939	0	0.08185044	0.08806046	2238134.21	0	0.01575799	99.9264665	1359.92457
1658	0.0849546	104.853374	0	0.08187427	0.088086	2238147.98	0	0.01576244	99.926483	1360.22878
1659	0.08494694	104.850456	0	0.0818668	0.08807816	2238146.67	0	0.0157619	99.9264833	1360.23596
1660	0.08499282	104.907879	0	0.08191275	0.08812394	2238034.81	0	0.01575319	99.9264427	1359.48499
1661	0.08492772	104.853185	0	0.08184833	0.08805818	2238109.51	0	0.01575736	99.9264623	1359.84676
1662	0.08494448	104.849412	0	0.08186439	0.08807564	2238118.74	0	0.01576167	99.9264676	1359.94487
1663	0.08500628	104.942	0	0.08192673	0.08813685	2237526.2	0	0.01574529	99.9264009	1358.71159
1664	0.0849815	104.884554	0	0.08190114	0.08811291	2237378.71	0	0.01575805	99.9264362	1359.36334
1665	0.08498086	104.883466	0	0.0819005	0.08811229	2237590.67	0	0.01575827	99.9264448	1359.52366
1666	0.08497016	104.869121	0	0.08188975	0.08810164	2238140.55	0	0.01576084	99.9264799	1360.17237
1667	0.08501833	104.980024	0	0.08193947	0.08814818	2237336.89	0	0.015736	99.9263584	1357.92872
1668	0.08501315	104.962807	0	0.08193397	0.08814333	2237280	0	0.01574025	99.9263766	1358.26429
1669	0.0849176	104.860133	0	0.08183876	0.08804749	2236556.89	0	0.01575318	99.9263978	1358.65551
1670	0.08493133	104.852071	0	0.08185178	0.08806195	2238133.15	0	0.01575848	99.9264686	1359.96315
1671	0.08491977	104.858624	0	0.08184081	0.08804978	2238060.04	0	0.01575413	99.9264466	1359.55588
1672	0.08493136	104.85171	0	0.0818518	0.08806199	2238127.11	0	0.01575855	99.9264684	1359.95954
1673	0.08490368	104.878559	0	0.08182586	0.08803252	2238069.18	0	0.01574504	99.9264059	1358.80445
1674	0.08493477	104.85139	0	0.08185508	0.08806553	2238140.51	0	0.01575942	99.9264733	1360.04981
1675	0.084941	104.852029	0	0.08186111	0.08807197	2238146.78	0	0.01576064	99.9264794	1360.16249
1676	0.08495358	104.853119	0	0.08187328	0.08808495	2238148.63	0	0.01576237	99.9264855	1360.27532
1677	0.0849479	104.850511	0	0.08186772	0.08807915	2238146.59	0	0.01576204	99.9264827	1360.22334
1678	0.08493449	104.850009	0	0.08185477	0.08806528	2238073.66	0	0.01575957	99.9264665	1359.92504
1679	0.08496022	104.857942	0	0.08187983	0.08809169	2238145.71	0	0.01576222	99.9264855	1360.27607
1680	0.08497774	104.882809	0	0.08189747	0.08810908	2238142.28	0	0.01575855	99.9264698	1359.98519
1681	0.08497399	104.872705	0	0.08189355	0.0881055	2237998.94	0	0.01576024	99.9264668	1359.93045
1682	0.08493591	104.850951	0	0.08185617	0.08806673	2238140.67	0	0.01575975	99.9264747	1360.07592
1683	0.08497254	104.872612	0	0.08189215	0.088104	2238140.06	0	0.01576028	99.9264775	1360.12766
1684	0.0849739	104.87251	0	0.08189346	0.08810541	2237843.74	0	0.01576028	99.9264552	1359.71474
1685	0.08495748	104.855112	0	0.0818771	0.08808893	2238141.37	0	0.01576244	99.9264783	1360.14352
1686	0.08497455	104.873837	0	0.08189413	0.08810605	2238102.42	0	0.01576006	99.9264739	1360.06079
1687	0.08496625	104.864785	0	0.08188585	0.08809772	2238144.41	0	0.01576145	99.9264829	1360.22718
1688	0.08491236	104.865717	0	0.08183387	0.0880419	2237115.7	0	0.01575046	99.9264092	1358.86542
1689	0.08496749	104.864277	0	0.08188703	0.08809902	2237952.88	0	0.01576156	99.9264541	1359.69472
1690	0.08496888	104.86647	0	0.08188844	0.0881004	2238129.45	0	0.01576124	99.9264802	1360.17787

© 2024 Zhang X et al. *JAMA Pediatrics*.

1691	0.08497315	104.871473	0	0.08189271	0.08810467	2237907.3	0	0.01576045	99.9264594	1359.79383
1692	0.0849597	104.856861	0	0.0818793	0.08809118	2238138.14	0	0.01576235	99.9264809	1360.19077
1693	0.08497378	104.872768	0	0.08189335	0.08810528	2238108.75	0	0.01576024	99.926475	1360.08109
1694	0.08495551	104.853991	0	0.08187517	0.08808693	2238147.51	0	0.01576244	99.9264839	1360.24616
1695	0.08497466	104.87377	0	0.08189423	0.08810616	2238058.97	0	0.01576006	99.9264709	1360.00538
1696	0.08498955	104.900488	0	0.08190938	0.08812077	2237998.92	0	0.01575478	99.9264488	1359.59755
1697	0.08497667	104.876814	0	0.08189625	0.08810815	2238054.63	0	0.01575952	99.9264691	1359.9728
1698	0.08496847	104.865717	0	0.08188802	0.08809999	2238116.79	0	0.01576135	99.9264789	1360.15336
1699	0.08496234	104.860862	0	0.08188196	0.08809379	2238146.52	0	0.01576189	99.9264849	1360.26568
1700	0.08498046	104.886924	0	0.08190021	0.08811177	2238140.13	0	0.01575775	99.9264661	1359.91641
1701	0.08496297	104.875533	0	0.081883	0.08809401	2238148.37	0	0.01575965	99.9264752	1360.08507
1702	0.08496635	104.863078	0	0.0818859	0.08809788	2238079.82	0	0.01576171	99.9264725	1360.03567
1703	0.08498082	104.893174	0	0.08190074	0.08811196	2238144.56	0	0.01575676	99.9264616	1359.83432
1704	0.08497712	104.885889	0	0.08189696	0.08810835	2238145.24	0	0.0157581	99.9264678	1359.94914
1705	0.084973	104.871675	0	0.08189256	0.0881045	2238111.69	0	0.01576042	99.9264758	1360.09704
1706	0.0849132	104.873132	0	0.0818349	0.08804255	2238139.67	0	0.01574964	99.9264284	1359.21968
1707	0.08496056	104.857379	0	0.08188014	0.08809205	2238037.06	0	0.01576233	99.9264291	1359.23384
1708	0.08488244	104.918803	0	0.08180654	0.08800933	2237507.48	0	0.01572849	99.9263249	1357.31151
1709	0.08490128	104.881372	0	0.08182363	0.08802996	2237803.65	0	0.01574357	99.9263955	1358.61192
1710	0.08498384	104.892115	0	0.08190362	0.08811511	2238135.39	0	0.01575669	99.926461	1359.82307
1711	0.08496065	104.862094	0	0.08188037	0.08809201	2238148.08	0	0.0157616	99.926484	1360.24891
1712	0.08496319	104.859922	0	0.08188275	0.0880947	2238124.42	0	0.01576208	99.9264793	1360.16127
1713	0.08498209	104.885737	0	0.08190174	0.08811349	2237945.13	0	0.01575783	99.9264587	1359.78071
1714	0.08500164	104.929392	0	0.08192189	0.08813242	2237905.15	0	0.01574828	99.9264188	1359.04235
1715	0.08499669	104.916656	0	0.08191674	0.08812768	2237889.05	0	0.01575121	99.9264313	1359.27383
1716	0.08498217	104.885995	0	0.08190183	0.08811357	2238018.35	0	0.01575778	99.9264614	1359.83078
1717	0.08497196	104.870179	0	0.08189152	0.08810347	2238108.81	0	0.01576067	99.9264764	1360.10708
1718	0.08495703	104.854804	0	0.08187666	0.08808848	2238142.78	0	0.01576245	99.9264787	1360.14973
1719	0.08496147	104.861323	0	0.08188113	0.08809288	2238147.6	0	0.01576177	99.9264847	1360.26083
1720	0.08492549	104.854258	0	0.08184621	0.08805584	2238076.57	0	0.01575656	99.9264573	1359.75473
1721	0.08497275	104.870873	0	0.0818923	0.08810427	2237548.36	0	0.01576055	99.9264289	1359.22882
1722	0.08495176	104.853325	0	0.08187154	0.08808307	2238148.72	0	0.01576213	99.9264859	1360.28401
1723	0.08496083	104.857754	0	0.08188041	0.08809232	2238131.01	0	0.01576229	99.9264788	1360.15259
1724	0.08496118	104.858193	0	0.08188076	0.08809267	2238138.13	0	0.01576224	99.9264825	1360.22092
1725	0.08493619	104.85089	0	0.08185644	0.08806702	2238140.89	0	0.01575982	99.926475	1360.08196
1726	0.08491384	104.864489	0	0.08183526	0.08804347	2238029.72	0	0.0157512	99.926433	1359.30455
1727	0.08493044	104.851295	0	0.0818509	0.08806106	2237883.32	0	0.01575838	99.9264496	1359.61243
1728	0.08493216	104.850547	0	0.08185254	0.08806286	2216333.26	0	0.01575893	99.9246067	1326.37865
1729	0.08493089	104.851155	0	0.08185133	0.08806152	2237991.71	0	0.01575851	99.9264578	1359.76409
1730	0.08492677	104.853083	0	0.08184741	0.0880572	2236531	0	0.01575711	99.9263752	1358.23732
1731	0.08493333	104.850548	0	0.08185367	0.08806407	2238109.62	0	0.01575921	99.9264694	1359.97798
1732	0.08495296	104.853018	0	0.08187268	0.08808432	2238148.71	0	0.01576231	99.9264859	1360.28309

© 2024 Zhang X et al. *JAMA Pediatrics.*

1733	0.08493312	104.85048	0	0.08185346	0.08806386	2238088.37	0	0.01575917	99.9264672	1359.9382
1734	0.08496951	104.869084	0	0.08188912	0.08810097	2238143.48	0	0.01576084	99.9264802	1360.17774
1735	0.08498071	104.886528	0	0.08190044	0.08811204	2238138.02	0	0.0157578	99.9264662	1359.91877
1736	0.08493278	104.852634	0	0.0818532	0.08806344	2238141.41	0	0.01575876	99.9264705	1359.99756
1737	0.08491038	104.868179	0	0.08183203	0.08803978	2237611.79	0	0.01574934	99.9264162	1358.99491
1738	0.08491491	104.862923	0	0.08183625	0.08804463	2237651.94	0	0.01575182	99.926426	1359.17636
1739	0.08497929	104.88101	0	0.08189891	0.08811074	2238030.23	0	0.01575875	99.9264654	1359.90315
1740	0.08492489	104.854236	0	0.08184563	0.08805522	2235150.01	0	0.01575639	99.926318	1357.1843
1741	0.08493136	104.850852	0	0.08185178	0.08806202	2235705.95	0	0.01575868	99.9262734	1356.36299
1742	0.08495218	104.851921	0	0.08187189	0.08808354	2238148.72	0	0.01576239	99.926476	1360.09969
1743	0.08487334	104.942421	0	0.08179844	0.08799919	2237842.41	0	0.0157196	99.9262863	1356.60012
1744	0.08493447	104.850858	0	0.08185477	0.08806523	2238135.36	0	0.01575943	99.9264728	1360.04075
1745	0.08487835	104.928535	0	0.08180287	0.08800479	2233662.04	0	0.01572469	99.926282	1356.52018
1746	0.08492122	104.856916	0	0.08184216	0.08805133	2210903.92	0	0.01575486	99.9254575	1341.51687
1747	0.08493907	104.849083	0	0.08185916	0.08807006	2233512.63	0	0.01576071	99.9255564	1343.29928
1748	0.08488221	104.925636	0	0.08180651	0.08800888	2238113.91	0	0.01572732	99.926324	1357.29409
1749	0.0849114	104.866798	0	0.08183297	0.08804087	2211959.66	0	0.01574994	99.9258713	1349.00536
1750	0.08489455	104.893006	0	0.08181748	0.08802264	2237444.77	0	0.01573869	99.9263698	1358.13875
1751	0.08494645	104.858744	0	0.08186657	0.08807741	2238148.5	0	0.01576055	99.9264793	1360.16091
1752	0.08487731	104.931341	0	0.08180194	0.08800363	2237076.14	0	0.01572365	99.9263001	1356.85436
1753	0.08490399	104.878441	0	0.08182616	0.08803285	2238087.11	0	0.01574519	99.9264069	1358.82229
1754	0.08490193	104.881013	0	0.08182425	0.08803064	2238041.99	0	0.01574391	99.9264003	1358.70158
1755	0.08489868	104.885503	0	0.08182124	0.08802714	2237126.16	0	0.01574177	99.926379	1358.30812
1756	0.0849201	104.857844	0	0.0818411	0.08805015	2199048.08	0	0.01575436	99.9251242	1335.54495
1757	0.08491727	104.860828	0	0.08183847	0.08804713	2238025.73	0	0.01575296	99.9264405	1359.44401
1758	0.08490719	104.872354	0	0.08182907	0.08803635	2237486.53	0	0.01574744	99.9264066	1358.81797
1759	0.08493006	104.851773	0	0.08185054	0.08806064	2238094.62	0	0.0157582	99.9264647	1359.89036
1760	0.08490022	104.882793	0	0.08182265	0.08802882	2114249.92	0	0.01574288	99.9247787	1329.40991
1761	0.08490494	104.875491	0	0.08182699	0.08803393	2236832.13	0	0.01574604	99.9263906	1358.52276
1762	0.08492246	104.85596	0	0.08184333	0.08805265	2236262.41	0	0.0157554	99.9263799	1358.32439
1763	0.08490911	104.869774	0	0.08183085	0.08803841	2237455.16	0	0.0157486	99.9264104	1358.88721
1764	0.08489425	104.896424	0	0.08181728	0.08802222	2238100.38	0	0.01573802	99.9263738	1358.21245
1765	0.08490684	104.872782	0	0.08182875	0.08803597	2236914.1	0	0.01574724	99.9263959	1358.6193
1766	0.08489553	104.891341	0	0.08181837	0.0880237	2237757.88	0	0.01573941	99.9263764	1358.26086
1767	0.08494081	104.85164	0	0.08186091	0.08807178	2238146.44	0	0.01576066	99.9264794	1360.16308
1768	0.08489063	104.901658	0	0.08181394	0.08801831	2238010.98	0	0.01573544	99.9263609	1357.97472
1769	0.08489943	104.88447	0	0.08182194	0.08802795	2237827.92	0	0.01574227	99.92639	1358.51184
1770	0.08489152	104.900645	0	0.08181477	0.08801927	2238067.79	0	0.01573604	99.9263643	1358.0362
1771	0.08488935	104.90536	0	0.08181281	0.08801688	2238072.46	0	0.01573422	99.9263559	1357.88119
1772	0.0849019	104.881886	0	0.08182424	0.08803058	2238089.22	0	0.01574376	99.9264003	1358.70131
1773	0.08491488	104.86284	0	0.08183622	0.0880446	2203995.17	0	0.01575182	99.925573	1343.59867
1774	0.08492508	104.854303	0	0.08184581	0.08805541	2237994.5	0	0.01575644	99.9264529	1359.67282

© 2024 Zhang X et al. *JAMA Pediatrics*.

1775	0.08490811	104.871087	0	0.08182992	0.08803734	2237399.11	0	0.01574801	99.9264072	1358.82771
1776	0.08490686	104.872961	0	0.08182877	0.08803598	2237833.68	0	0.01574721	99.9264118	1358.91316
1777	0.0849271	104.852897	0	0.08184772	0.08805754	2236693	0	0.01575723	99.9263825	1358.37262
1778	0.08490839	104.876101	0	0.08183033	0.08803747	2238132.43	0	0.01574735	99.9264176	1359.02043
1779	0.08490336	104.879322	0	0.08182558	0.08803218	2238082.75	0	0.01574479	99.9264049	1358.78694
1780	0.08492614	104.853534	0	0.08184681	0.08805653	2237847.64	0	0.01575686	99.9264466	1359.5569
1781	0.08491869	104.859569	0	0.0818398	0.08804864	2238052.43	0	0.01575363	99.9264442	1359.51189
1782	0.08487826	104.933962	0	0.08180294	0.08800454	2238101.44	0	0.01572381	99.9263075	1356.99013
1783	0.08494926	104.853106	0	0.08186911	0.08808048	2238148.55	0	0.01576183	99.9264849	1360.2639
1784	0.08495132	104.851405	0	0.08187105	0.08808266	2238142.69	0	0.01576237	99.9262935	1356.73285
1785	0.08490692	104.87335	0	0.08182884	0.08803604	2238034.13	0	0.01574718	99.9264151	1358.97472
1786	0.08491823	104.861614	0	0.08183942	0.0880481	2238124.94	0	0.01575317	99.9264443	1359.51441
1787	0.08491104	104.867253	0	0.08183263	0.08804048	2233771.59	0	0.01574973	99.9263368	1357.52914
1788	0.08493904	104.849793	0	0.08185916	0.08807001	2238138.12	0	0.0157606	99.9264776	1360.12933
1789	0.08489052	104.901278	0	0.08181382	0.08801822	2237866.49	0	0.01573544	99.9263596	1357.94985
1790	0.08487477	104.938646	0	0.08179971	0.08800079	2237875.02	0	0.01572104	99.9262932	1356.72665
1791	0.08489778	104.887723	0	0.08182044	0.08802615	2237990.56	0	0.01574102	99.9263864	1358.44521
1792	0.08494551	104.851017	0	0.08186544	0.08807667	2238147.36	0	0.01576159	99.9264833	1360.23564
1793	0.08493953	104.849298	0	0.08185961	0.08807052	2238120.4	0	0.01576077	99.9264744	1360.07055
1794	0.0849267	104.853472	0	0.08184735	0.08805711	2238073.17	0	0.01575703	99.926459	1359.78518
1795	0.0849584	104.857259	0	0.08187805	0.08808982	2238147.78	0	0.01576219	99.9264862	1360.28834
1796	0.0849165	104.862232	0	0.08183776	0.08804629	2238097.55	0	0.01575248	99.9264404	1359.4424
1797	0.08492693	104.855623	0	0.08184764	0.08805729	2238137.99	0	0.01575676	99.9264612	1359.82723
1798	0.08493133	104.855251	0	0.08185187	0.08806185	2238144.08	0	0.01575799	99.9264672	1359.93731
1799	0.08492709	104.856863	0	0.08184782	0.08805741	2238141.43	0	0.01575662	99.9264607	1359.81803
1800	0.08489927	104.884511	0	0.08182178	0.08802778	2237232.12	0	0.01574219	99.926382	1358.36413
1801	0.08493546	104.849654	0	0.0818557	0.0880663	2237929.8	0	0.01575985	99.9264489	1359.59905
1802	0.08493492	104.850215	0	0.08185518	0.08806572	2238122.59	0	0.01575963	99.9264722	1360.02917
1803	0.08491888	104.859435	0	0.08183998	0.08804884	2238060.31	0	0.01575372	99.9264448	1359.5231
1804	0.08490854	104.872114	0	0.08183037	0.08803775	2238101.02	0	0.01574802	99.9264201	1359.06751
1805	0.08491851	104.859342	0	0.08183962	0.08804846	2237649.22	0	0.01575361	99.9264315	1359.27676
1806	0.08491575	104.861983	0	0.08183703	0.08804553	2237152.69	0	0.01575226	99.9264141	1358.95623
1807	0.08494474	104.8511	0	0.08186469	0.08807586	2238147.24	0	0.01576145	99.9264828	1360.22589
1808	0.08495673	104.863485	0	0.08187662	0.0880879	2238148.64	0	0.01576108	99.9264818	1360.20696
1809	0.08494039	104.84996	0	0.08186046	0.0880714	2238142.15	0	0.01576084	99.9264792	1360.15949
1810	0.08491532	104.862612	0	0.08183663	0.08804506	2237906.86	0	0.01575201	99.9264333	1359.31121
1811	0.08493145	104.852769	0	0.08185192	0.08806206	2238139.03	0	0.01575841	99.9264687	1359.96528
1812	0.08488487	104.914629	0	0.08180876	0.08801196	2238038.55	0	0.01573045	99.926338	1357.55267
1813	0.08492876	104.854515	0	0.08184937	0.08805921	2238139	0	0.01575744	99.9264643	1359.88461
1814	0.08489272	104.897334	0	0.08181583	0.08802061	2238000.56	0	0.01573714	99.9263687	1358.11781
1815	0.08489604	104.890066	0	0.08181882	0.08802427	2163135.85	0	0.01573985	99.9255334	1342.88313
1816	0.08490929	104.869545	0	0.08183101	0.0880386	2237469.7	0	0.01574871	99.926411	1358.89944

© 2024 Zhang X et al. *JAMA Pediatrics*.

1817	0.08490937	104.869325	0	0.08183109	0.0880387	2171309.55	0	0.01574877	99.9251403	1335.83225
1818	0.08485417	105.00526	0	0.08178176	0.08797746	2238054.94	0	0.0156973	99.9261831	1354.70379
1819	0.08488998	104.903656	0	0.08181337	0.08801758	2238058.49	0	0.0157348	99.9263584	1357.92873
1820	0.08492644	104.853267	0	0.0818471	0.08805686	2235226.46	0	0.01575699	99.9263075	1356.99024
1821	0.08494235	104.856588	0	0.08186254	0.08807322	2238148.03	0	0.01576019	99.9264776	1360.12903
1822	0.08484783	105.02732	0	0.08177628	0.08797023	2238027.91	0	0.01568927	99.9261453	1354.01016
1823	0.08486746	104.960746	0	0.0817933	0.08799256	2238027.73	0	0.01571313	99.9262572	1356.06406
1824	0.08495177	104.852273	0	0.08187151	0.0880831	2238148.7	0	0.01576229	99.9264853	1360.273
1825	0.08491334	104.865438	0	0.08183481	0.08804292	2238078.5	0	0.01575087	99.9264327	1359.29894
1826	0.08494176	104.849085	0	0.08186176	0.08807284	2238015.32	0	0.01576123	99.9264405	1359.44446
1827	0.08485193	105.015726	0	0.0817799	0.08797483	2238083.07	0	0.01569409	99.9261681	1354.42862
1828	0.08489911	104.886822	0	0.08182169	0.08802754	2238092.77	0	0.01574177	99.9263911	1358.53161
1829	0.08488646	104.910603	0	0.08181017	0.08801373	2238005.78	0	0.01573192	99.9263446	1357.67317
1830	0.08493346	104.850124	0	0.08185377	0.08806421	2231761.87	0	0.01575931	99.9258411	1348.45537
1831	0.08489227	104.897928	0	0.08181541	0.08802013	2237939.93	0	0.01573683	99.9263666	1358.08011
1832	0.08494487	104.851055	0	0.08186482	0.088076	2238147.24	0	0.01576148	99.9264829	1360.22777
1833	0.08495091	104.851771	0	0.08187067	0.08808223	2238148.56	0	0.01576226	99.9264847	1360.26165
1834	0.08494128	104.849223	0	0.08186129	0.08807233	2238121.6	0	0.01576112	99.9264743	1360.06908
1835	0.08490865	104.870466	0	0.08183042	0.08803791	2237759.73	0	0.01574831	99.9264151	1358.97378
1836	0.08493835	104.851353	0	0.08185853	0.08806924	2238144.81	0	0.01576021	99.9264772	1360.12246
1837	0.08490241	104.882421	0	0.08182475	0.08803109	2238114.17	0	0.0157439	99.9264013	1358.71973
1838	0.08494009	104.851756	0	0.08186022	0.08807103	2238146.24	0	0.0157605	99.9264787	1360.14985
1839	0.08494779	104.852188	0	0.08186767	0.08807899	2238148.28	0	0.01576176	99.9264844	1360.25576
1840	0.08493993	104.849997	0	0.08186002	0.08807092	2238141.83	0	0.01576074	99.9264788	1360.15196
1841	0.08490449	104.876144	0	0.08182657	0.08803343	2236158.63	0	0.01574575	99.9263791	1358.30909
1842	0.08488708	104.909609	0	0.08181075	0.0880144	2238039.03	0	0.0157324	99.9263471	1357.71934
1843	0.08490098	104.882515	0	0.08182338	0.08802961	2238035.83	0	0.01574326	99.9263973	1358.64537
1844	0.08495549	104.853692	0	0.08187514	0.08808692	2238141.42	0	0.01576248	99.926462	1359.84178
1845	0.08494316	104.849954	0	0.08186314	0.08807427	2238144.21	0	0.01576136	99.9264814	1360.19931
1846	0.08492409	104.85486	0	0.08184487	0.08805437	2237799	0	0.01575606	99.9264429	1359.48768
1847	0.08495903	104.858712	0	0.08187871	0.08809043	2238148	0	0.01576201	99.9264857	1360.27952
1848	0.08492413	104.855178	0	0.08184492	0.0880544	2238072.83	0	0.01575602	99.926455	1359.71108
1849	0.08490468	104.876008	0	0.08182676	0.08803364	2237686.71	0	0.01574585	99.9264036	1358.76145
1850	0.08492451	104.855385	0	0.0818453	0.08805479	2238112.71	0	0.0157561	99.9264571	1359.75051
1851	0.08490807	104.871853	0	0.08182991	0.08803728	2238049.58	0	0.01574787	99.9264185	1359.03774
1852	0.08489464	104.89327	0	0.08181757	0.08802272	2237927.21	0	0.0157387	99.9263751	1358.23541
1853	0.08490706	104.872864	0	0.08182896	0.08803619	2237961.02	0	0.01574731	99.9264144	1358.96193
1854	0.0849035	104.880915	0	0.08182576	0.08803227	2238117.37	0	0.0157446	99.9264046	1358.78063
1855	0.08492693	104.853807	0	0.08184758	0.08805734	2238115.98	0	0.01575704	99.9264613	1359.82876
1856	0.08491085	104.869489	0	0.08183252	0.08804023	2238112.36	0	0.01574932	99.9264263	1359.18201
1857	0.08489941	104.885685	0	0.08182195	0.08802789	2238071.95	0	0.01574208	99.9263923	1358.55325
1858	0.084926	104.853542	0	0.08184668	0.08805639	2236583.41	0	0.01575682	99.9263816	1358.35523

© 2024 Zhang X et al. *JAMA Pediatrics*.

1859	0.08492356	104.855464	0	0.08184438	0.08805381	2238044.57	0	0.01575581	99.9264529	1359.67258
1860	0.0849262	104.85538	0	0.08184692	0.08805654	2238133.74	0	0.01575659	99.9264603	1359.80912
1861	0.08487683	104.940027	0	0.08180174	0.08800288	2238113.25	0	0.01572205	99.9262994	1356.8403
1862	0.08489703	104.888604	0	0.08181973	0.08802533	2237757.51	0	0.01574054	99.9263815	1358.3533
1863	0.08496466	104.861366	0	0.08188422	0.08809618	2238115.22	0	0.01576192	99.9264781	1360.13852
1864	0.08495337	104.852525	0	0.08187306	0.08808475	2238148.38	0	0.01576244	99.9264759	1360.09899
1865	0.08489027	104.901539	0	0.08181359	0.08801795	2237646.56	0	0.01573528	99.9263568	1357.8978
1866	0.0848826	104.919075	0	0.0818067	0.08800948	2237936.47	0	0.01572853	99.9263283	1357.37424
1867	0.0849024	104.880761	0	0.08182469	0.08803113	2238077.63	0	0.01574415	99.9264019	1358.73145
1868	0.08485494	105.000695	0	0.08178237	0.08797839	2237994.76	0	0.01569855	99.9261887	1354.80683
1869	0.08488794	104.906956	0	0.0818115	0.08801537	2237943.63	0	0.01573325	99.9263502	1357.77721
1870	0.08487266	104.947265	0	0.08179793	0.08799835	2238076.2	0	0.01571845	99.9262823	1356.52615
1871	0.08491151	104.867354	0	0.0818331	0.08804097	2238048.98	0	0.0157499	99.9264276	1359.20601
1872	0.0848939	104.896229	0	0.08181694	0.08802187	2238082.13	0	0.01573789	99.926373	1358.19728
1873	0.0848979	104.88978	0	0.08182061	0.0880262	2238106.18	0	0.01574076	99.9263866	1358.44843
1874	0.08488382	104.915899	0	0.08180779	0.08801084	2237840.37	0	0.01572969	99.926333	1357.45927
1875	0.08484929	105.022993	0	0.08177756	0.08797187	2238053.54	0	0.01569103	99.9261536	1354.16304
1876	0.08492833	104.852415	0	0.08184889	0.08805883	2238028.25	0	0.01575764	99.9264585	1359.7761
1877	0.08489814	104.886346	0	0.08182074	0.08802655	2232294.93	0	0.0157414	99.926318	1357.18316
1878	0.08489218	104.901573	0	0.08181543	0.08801993	2238110.25	0	0.01573623	99.9263655	1358.05997
1879	0.08489612	104.894253	0	0.08181902	0.08802422	2238116.11	0	0.01573925	99.9263797	1358.32077
1880	0.08492488	104.854313	0	0.08184562	0.08805521	2237767.63	0	0.01575638	99.926442	1359.47083
1881	0.08491376	104.867975	0	0.08183529	0.08804328	2238131.69	0	0.01575063	99.9264328	1359.30176
1882	0.08490489	104.876451	0	0.08182697	0.08803384	2238049.21	0	0.01574587	99.9264094	1358.86911
1883	0.08489703	104.888269	0	0.08181973	0.08802535	2137887.8	0	0.01574059	99.9252148	1337.16338
1884	0.08489964	104.884175	0	0.08182213	0.08802817	2237871.24	0	0.01574241	99.9263912	1358.53359
1885	0.08487875	104.928258	0	0.08180325	0.08800522	2237863.12	0	0.01572496	99.9263113	1357.05986
1886	0.08492645	104.853352	0	0.08184711	0.08805686	2237865.62	0	0.01575698	99.9264477	1359.57769
1887	0.08492798	104.852509	0	0.08184855	0.08805846	2237923.28	0	0.01575753	99.9264519	1359.65385
1888	0.08487656	104.934166	0	0.08180131	0.08800277	2237935.23	0	0.01572278	99.9263017	1356.88271
1889	0.08486951	104.960081	0	0.08179526	0.0879947	2238106.84	0	0.01571454	99.9262642	1356.19268
1890	0.0849209	104.857193	0	0.08184186	0.08805099	2236022.26	0	0.01575472	99.9263753	1358.2399
1891	0.08496082	104.857702	0	0.0818804	0.08809231	2238125.32	0	0.01576229	99.926476	1360.10085
1892	0.08502195	104.992758	0	0.08194334	0.08815155	2237230.53	0	0.01573281	99.9263434	1357.65177
1893	0.08491016	104.868864	0	0.08183183	0.08803952	2238012.06	0	0.01574915	99.9264235	1359.13019
1894	0.08493737	104.849402	0	0.08185753	0.08806829	2238083.84	0	0.01576031	99.9264681	1359.95352
1895	0.08493738	104.849468	0	0.08185754	0.08806829	2238104.22	0	0.0157603	99.9264713	1360.01236
1896	0.08492978	104.851722	0	0.08185027	0.08806036	2238047.55	0	0.01575814	99.9264612	1359.82555
1897	0.0849335	104.850342	0	0.08185382	0.08806425	2238084.79	0	0.01575928	99.9264672	1359.93686
1898	0.08497942	104.882114	0	0.08189906	0.08811084	2238121.2	0	0.01575857	99.9264689	1359.9691
1899	0.08492307	104.856952	0	0.08184395	0.08805326	2238123.78	0	0.01575543	99.9264546	1359.70398
1900	0.08489955	104.883981	0	0.08182204	0.08802809	2236586.14	0	0.0157424	99.9263745	1358.22504

© 2024 Zhang X et al. *JAMA Pediatrics*.

1901	0.08491741	104.860552	0	0.08183859	0.08804728	2237963.71	0	0.01575305	99.9264391	1359.4175
1902	0.08488513	104.912623	0	0.08180895	0.08801229	2237572.18	0	0.0157309	99.9263364	1357.52168
1903	0.08483147	105.092062	0	0.08176237	0.08795136	2237953.58	0	0.01566632	99.9260371	1352.02924
1904	0.08492846	104.852849	0	0.08184903	0.08805896	2238114.42	0	0.01575761	99.9264636	1359.87162
1905	0.08490206	104.88169	0	0.08182439	0.08803075	2238091.17	0	0.01574386	99.9264008	1358.71018
1906	0.08491676	104.863317	0	0.08183805	0.08804653	2238125.88	0	0.01575241	99.9264409	1359.4504
1907	0.08490392	104.877107	0	0.08182605	0.08803281	2237543.78	0	0.01574536	99.9263993	1358.68269
1908	0.08489108	104.906736	0	0.08181453	0.08801864	2238123.78	0	0.01573489	99.9263594	1357.94744
1909	0.08494376	104.851453	0	0.08186375	0.08807484	2238147.24	0	0.01576123	99.926482	1360.21032
1910	0.08490803	104.87186	0	0.08182987	0.08803724	2238043.71	0	0.01574786	99.9264183	1359.03437
1911	0.08489653	104.891214	0	0.08181934	0.08802475	2238084.02	0	0.01573991	99.9263824	1358.37068
1912	0.08492302	104.85692	0	0.0818439	0.0880532	2238122.38	0	0.01575542	99.9264545	1359.70192
1913	0.0849056	104.875131	0	0.08182762	0.08803462	2238013.11	0	0.01574637	99.9264111	1358.90034
1914	0.08489108	104.900853	0	0.08181435	0.08801881	2238025.41	0	0.01573579	99.9263627	1358.00733
1915	0.08486198	104.984209	0	0.08178869	0.08798618	2238105.39	0	0.01570595	99.9262239	1355.45249
1916	0.08484652	105.034877	0	0.08177524	0.08796865	2238070.83	0	0.01568713	99.9261354	1353.82824
1917	0.08496419	104.862145	0	0.08188379	0.08809567	2238144.56	0	0.01576178	99.9264842	1360.25133
1918	0.08491333	104.86464	0	0.08183477	0.08804294	2237514.9	0	0.01575099	99.9264199	1359.06319
1919	0.08490416	104.877127	0	0.08182628	0.08803306	2237970.92	0	0.01574546	99.9264063	1358.81219
1920	0.08491583	104.862113	0	0.08183711	0.0880456	2237937.93	0	0.01575227	99.9264352	1359.3454
1921	0.0849142	104.863839	0	0.08183559	0.08804386	2237918.09	0	0.01575142	99.9264312	1359.2723
1922	0.08483606	105.071137	0	0.08176619	0.08795674	2237809.48	0	0.01567333	99.9260697	1352.62559
1923	0.08494761	104.853024	0	0.08186752	0.08807878	2238148.37	0	0.01576161	99.9264839	1360.24621
1924	0.08488472	104.914005	0	0.0818086	0.08801183	2237909.6	0	0.01573047	99.9263371	1357.5353
1925	0.08491284	104.866152	0	0.08183435	0.08804239	2238086.62	0	0.01575058	99.9264315	1359.27799
1926	0.08489923	104.884947	0	0.08182175	0.08802772	2237911.14	0	0.01574211	99.9263904	1358.51785
1927	0.08493996	104.849106	0	0.08186002	0.08807097	2238054.35	0	0.01576088	99.9264593	1359.79046
1928	0.08489251	104.897227	0	0.08181563	0.0880204	2237849.39	0	0.01573706	99.9263668	1358.08245
1929	0.08489849	104.887061	0	0.0818211	0.0880269	2238058.41	0	0.01574145	99.9263892	1358.49665
1930	0.08492656	104.853401	0	0.08184721	0.08805697	2238016.14	0	0.015757	99.9264558	1359.7268
1931	0.08490784	104.883056	0	0.08183001	0.0880367	2238139.6	0	0.01574606	99.9264117	1358.91237
1932	0.0848742	104.942977	0	0.08179929	0.08800007	2238076.89	0	0.01572004	99.9262897	1356.66285
1933	0.08489099	104.901253	0	0.08181428	0.08801871	2238042.76	0	0.01573568	99.9263624	1358.00132
1934	0.08489359	104.895052	0	0.08181661	0.08802158	2237802.02	0	0.01573792	99.9263702	1358.14542
1935	0.08488578	104.912709	0	0.08180958	0.08801296	2238048.46	0	0.01573123	99.9263417	1357.62115
1936	0.08492202	104.857302	0	0.08184294	0.08805215	2238110.73	0	0.01575505	99.9264524	1359.66321
1937	0.08491681	104.867738	0	0.08183822	0.08804644	2238139.87	0	0.01575174	99.9264382	1359.40066
1938	0.08488074	104.922641	0	0.08180501	0.08800745	2235487.31	0	0.01572695	99.9263036	1356.91804
1939	0.08491551	104.863747	0	0.08183685	0.08804522	2238111.76	0	0.0157519	99.9264382	1359.40088
1940	0.0849289	104.852014	0	0.08184943	0.08805943	2237873.71	0	0.01575786	99.9264491	1359.60254
1941	0.08492845	104.85418	0	0.08184906	0.08805891	2238136.5	0	0.01575741	99.9264641	1359.87939
1942	0.08488085	104.925855	0	0.08180521	0.08800747	2238085.97	0	0.01572652	99.9263201	1357.222

© 2024 Zhang X et al. *JAMA Pediatrics.*

1943	0.08494043	104.849043	0	0.08186047	0.08807146	2237772.38	0	0.01576099	99.9263877	1358.46874
1944	0.08491516	104.862571	0	0.08183648	0.0880449	2236213.2	0	0.01575196	99.9263892	1358.49545
1945	0.08491578	104.862315	0	0.08183707	0.08804554	2238013.3	0	0.01575222	99.926437	1359.37884
1946	0.08488886	104.908616	0	0.08181243	0.08801628	2238108.26	0	0.01573347	99.9263527	1357.8229
1947	0.08493992	104.849058	0	0.08185998	0.08807094	2237816.23	0	0.01576089	99.9264047	1358.78273
1948	0.08491682	104.862525	0	0.08183808	0.08804661	2238116.46	0	0.01575255	99.9264412	1359.45758
1949	0.08490628	104.873501	0	0.08182822	0.08803537	2065931.32	0	0.0157469	99.9235903	1308.73515
1950	0.08493357	104.857265	0	0.08185409	0.08806412	2238146.28	0	0.01575823	99.9264685	1359.96053
1951	0.08490985	104.869282	0	0.08183154	0.08803919	2238016.09	0	0.01574896	99.9264228	1359.11652
1952	0.08491869	104.859384	0	0.08183979	0.08804864	2237992.04	0	0.01575366	99.9264424	1359.47885
1953	0.08493431	104.849903	0	0.08185459	0.0880651	2237583.18	0	0.01575954	99.926413	1358.93501
1954	0.08493389	104.850019	0	0.08185419	0.08806466	2237579.54	0	0.01575943	99.9264148	1358.96888
1955	0.08493095	104.851032	0	0.08185139	0.08806159	2237379.73	0	0.01575855	99.9264103	1358.88544
1956	0.08493096	104.851031	0	0.08185139	0.08806159	2237354.63	0	0.01575855	99.9264083	1358.84931
1957	0.0849293	104.851755	0	0.08184981	0.08805986	2237105.94	0	0.01575801	99.9263976	1358.65148
1958	0.08489271	104.89831	0	0.08181585	0.08802057	2238071.92	0	0.01573699	99.9263687	1358.11818
1959	0.08487205	104.94708	0	0.08179733	0.08799772	2238011.13	0	0.0157181	99.9262803	1356.48969
1960	0.08489202	104.899976	0	0.08181523	0.0880198	2238080.09	0	0.01573639	99.926366	1358.06846
1961	0.08487774	104.934363	0	0.08180245	0.08800399	2238091.85	0	0.01572344	99.9263057	1356.95781
1962	0.08489683	104.892219	0	0.08181965	0.08802502	2238110.87	0	0.01573989	99.9263826	1358.37501
1963	0.08487912	104.930831	0	0.08180368	0.08800552	2238093.13	0	0.01572478	99.926312	1357.07281
1964	0.08487998	104.927802	0	0.08180442	0.08800651	2238081.48	0	0.01572574	99.9263164	1357.15362
1965	0.08487596	104.937484	0	0.08180083	0.08800206	2238061.75	0	0.01572193	99.9262985	1356.82368
1966	0.08485925	104.987923	0	0.08178616	0.08798324	2238059.32	0	0.01570353	99.9262123	1355.23993
1967	0.08491464	104.864296	0	0.08183603	0.0880443	2238098.65	0	0.01575151	99.9264361	1359.3619
1968	0.08494256	104.849118	0	0.08186253	0.08807367	2237947.73	0	0.01576138	99.9264091	1358.86339
1969	0.08490706	104.872534	0	0.08182895	0.0880362	2237447.77	0	0.01574736	99.9264056	1358.79956
1970	0.08487233	104.947441	0	0.08179761	0.087998	2238059.83	0	0.01571822	99.9262811	1356.50476
1971	0.08494495	104.849482	0	0.08186485	0.08807613	2238118.88	0	0.01576174	99.9264658	1359.9107
1972	0.08493587	104.850791	0	0.08185612	0.08806669	2238139.58	0	0.01575976	99.9264746	1360.0746
1973	0.08489888	104.885572	0	0.08182143	0.08802734	2237922.65	0	0.01574185	99.9263894	1358.49942
1974	0.08487564	104.937858	0	0.08180053	0.08800171	2238045.08	0	0.01572168	99.9262972	1356.80051
1975	0.08495433	104.865791	0	0.08187437	0.08808535	2238148.72	0	0.01576049	99.9264791	1360.15688
1976	0.08490395	104.876938	0	0.08182607	0.08803285	2235520.06	0	0.0157454	99.9263683	1358.11014
1977	0.08486987	104.953847	0	0.08179542	0.08799526	2238037.93	0	0.01571572	99.9262693	1356.28717
1978	0.08489343	104.895634	0	0.08181647	0.0880214	2237940.83	0	0.01573775	99.9263709	1358.1583
1979	0.08487839	104.929123	0	0.08180292	0.08800482	2237843.11	0	0.01572462	99.9263096	1357.02837
1980	0.08493178	104.850707	0	0.08185217	0.08806246	2237337.32	0	0.01575881	99.926403	1358.75166
1981	0.08489772	104.887421	0	0.08182037	0.08802609	2237811.26	0	0.01574104	99.9263843	1358.40649
1982	0.08489666	104.889311	0	0.0818194	0.08802494	2237810.63	0	0.01574026	99.9263808	1358.34188
1983	0.08490859	104.87052	0	0.08183037	0.08803786	2237719.48	0	0.01574828	99.9264142	1358.95753
1984	0.0849076	104.879945	0	0.08182969	0.08803654	2238136.77	0	0.01574644	99.9264135	1358.94433

© 2024 Zhang X et al. *JAMA Pediatrics*.

1985	0.08493354	104.850096	0	0.08185386	0.0880643	2216710.07	0	0.01575933	99.9243702	1322.23048
1986	0.08493058	104.851313	0	0.08185104	0.0880612	2238019.18	0	0.01575841	99.9264598	1359.7997
1987	0.0848951	104.891901	0	0.08181797	0.08802324	2236928.46	0	0.01573913	99.926366	1358.06777
1988	0.08493819	104.8492	0	0.08185832	0.08806914	2237954.77	0	0.01576051	99.9264449	1359.52575
1989	0.08493055	104.851329	0	0.081851	0.08806117	2238021.02	0	0.0157584	99.9264599	1359.80186
1990	0.08488835	104.906606	0	0.08181188	0.08801581	2238022.38	0	0.01573352	99.9263521	1357.81256
1991	0.0850199	104.985981	0	0.08194115	0.08814962	2237882.34	0	0.01573456	99.9263561	1357.88593
1992	0.08492954	104.852688	0	0.08185006	0.08806007	2238127.92	0	0.01575792	99.9264658	1359.91189
1993	0.084922	104.857533	0	0.08184293	0.08805212	2238117.09	0	0.01575501	99.9264524	1359.66417
1994	0.08499136	104.904442	0	0.08191124	0.08812253	2238000.81	0	0.01575393	99.9264452	1359.5312
1995	0.08495498	104.853626	0	0.08187465	0.08808639	2238147.79	0	0.01576244	99.9264834	1360.23657
1996	0.08491129	104.86794	0	0.0818329	0.08804072	2238078.84	0	0.01574972	99.9264275	1359.20318
1997	0.08499826	104.920527	0	0.08191836	0.08812918	2237863.78	0	0.01575032	99.9264271	1359.1958
1998	0.08494291	104.849206	0	0.08186287	0.08807403	2238100.55	0	0.01576143	99.9264644	1359.88607
1999	0.08491353	104.864618	0	0.08183497	0.08804315	2237944.25	0	0.01575106	99.9264304	1359.25631
2000	0.0850157	104.971476	0	0.08193668	0.08814571	2237836.63	0	0.01573815	99.9263722	1358.1833
2001	0.08501195	104.959679	0	0.08193272	0.08814219	2237962.96	0	0.01574107	99.9263868	1358.45157
2002	0.08499807	104.920067	0	0.08191817	0.08812901	2237863.55	0	0.01575043	99.9264275	1359.2041
2003	0.08498778	104.896855	0	0.08190757	0.08811905	2238021.96	0	0.01575556	99.9264527	1359.66907
2004	0.08494731	104.852855	0	0.08186722	0.08807847	2238148.32	0	0.01576159	99.9264838	1360.24425
2005	0.08495344	104.852523	0	0.08187313	0.08808483	2238148.1	0	0.01576245	99.9264692	1359.97478
2006	0.08498673	104.894373	0	0.08190647	0.08811803	2237457.04	0	0.01575606	99.9264381	1359.40008
2007	0.0849898	104.900823	0	0.08190963	0.08812103	2237837.29	0	0.0157547	99.9264445	1359.51706
2008	0.08495947	104.857327	0	0.08187909	0.08809093	2238146.25	0	0.01576226	99.9264857	1360.27949

eTable 7. Sensitivity analysis performed by using a built-in function.

	rstudent	dffits	cook.d	cov.r	tau2.del	QE.del	hat	weight	inf
1	0.18981136	0.00409377	1.68E-05	1.00100055	0.01576211	2238147.1	0.0005043	0.05043013	
2	-0.1236282	-0.0029579	8.75E-06	1.00101805	0.01576235	2238132.97	0.00050646	0.05064599	
3	-0.6228962	-0.0140496	0.00019745	1.00081923	0.01575926	2238113.68	0.00050062	0.05006243	
4	-0.6583938	-0.0146902	0.00021586	1.00078079	0.0157588	2238132.3	0.00049089	0.04908902	
5	-1.1939997	-0.0267488	0.00071535	1.00029442	0.01575078	2237912.97	0.00050433	0.05043306	
6	0.47345233	0.01047544	0.00010978	1.00089711	0.01576049	2238135.46	0.00050201	0.05020133	
7	0.02071731	0.00028914	8.36E-08	1.00102637	0.01576247	2238144.85	0.00050733	0.05073251	
8	-1.0074825	-0.0225191	0.00050711	1.00049979	0.01575415	2238077.63	0.00049935	0.04993544	
9	0.69805708	0.01556725	0.0002424	1.00076127	0.01575829	2238111.09	0.00050274	0.05027437	
10	-0.6720536	-0.0150695	0.00022715	1.00077989	0.0157587	2238124.13	0.00049608	0.04960847	
11	0.83323908	0.0184959	0.00034215	1.00064387	0.01575653	2238126.1	0.0004952	0.04951991	
12	0.90790299	0.02028045	0.00041133	1.00058319	0.01575548	2238104.01	0.00050012	0.05001192	
13	1.14836344	0.02532591	0.0006413	1.00031764	0.01575148	2238124.55	0.00048396	0.04839557	
14	0.40416438	0.00891877	7.96E-05	1.0009304	0.01576101	2238138.31	0.00050282	0.050282	
15	0.11614802	0.00239083	5.72E-06	1.00097372	0.01576199	2238148.7	0.00048484	0.0484842	

© 2024 Zhang X et al. *JAMA Pediatrics*.

16	0.69679712	0.01544652	0.00023866	1.00075309	0.01575826	2238131.45	0.00049676	0.04967636	
17	1.14551323	0.02518259	0.00063406	1.00031886	0.01575155	2238127.38	0.00048092	0.04809235	
18	0.55943152	0.01217817	0.00014836	1.00081788	0.01575953	2238144.13	0.00048261	0.04826093	
19	-0.6188441	-0.014024	0.00019674	1.00082927	0.01575935	2238053.66	0.00050521	0.05052089	
20	-0.3150949	-0.0071137	5.06E-05	1.00093958	0.0157614	2238144.79	0.00048751	0.04875113	
21	-0.0422924	-0.0011273	1.27E-06	1.0010213	0.01576243	2238145.98	0.00050492	0.05049226	
22	-0.5444047	-0.012392	0.00015362	1.00087715	0.01576008	2237742.17	0.00050735	0.05073504	
23	-0.6812004	-0.0154449	0.00023861	1.0007912	0.0157587	2237521.12	0.00050737	0.05073697	
24	-0.4127328	-0.0093439	8.73E-05	1.0009214	0.01576096	2238137.79	0.00049645	0.04964504	
25	-0.5113762	-0.0114372	0.00013086	1.00086248	0.01576014	2238139.31	0.00048885	0.04888528	
26	-0.5354994	-0.0121735	0.00014825	1.00087929	0.01576014	2238056.14	0.00050574	0.05057419	
27	-0.3768652	-0.0086359	7.46E-05	1.00095418	0.01576133	2238074.73	0.00050639	0.05063885	
28	-0.7344776	-0.0165003	0.00027233	1.0007407	0.01575802	2238108.51	0.00049941	0.04994077	
29	-0.3640732	-0.0082039	6.73E-05	1.00092647	0.01576116	2238143.32	0.00048916	0.0489161	
30	-0.1931848	-0.0045173	2.04E-05	1.00100602	0.01576217	2238128.9	0.00050588	0.05058825	
31	-0.687159	-0.0155745	0.00024263	1.00078669	0.01575863	2237704.17	0.00050716	0.05071645	
32	-0.9411069	-0.0210969	0.00044511	1.00056742	0.01575521	2238068.55	0.0005012	0.05012014	
33	0.07743779	0.00156477	2.45E-06	1.00101806	0.01576238	2238148.72	0.00050486	0.05048598	
34	-1.0733073	-0.0239708	0.00057456	1.0004305	0.01575304	2238067.1	0.00049953	0.04995296	
35	-0.3059376	-0.0070323	4.95E-05	1.00097453	0.01576169	2238127.56	0.00050421	0.0504207	
36	0.03784363	0.00067504	4.56E-07	1.001025	0.01576246	2238147.51	0.00050697	0.05069711	
37	-0.4232391	-0.0096455	9.31E-05	1.00093004	0.01576099	2238118.56	0.00050353	0.05035252	
38	-0.420107	-0.0095844	9.19E-05	1.00093304	0.01576102	2238111.08	0.00050442	0.05044245	
39	-0.3353283	-0.0077095	5.95E-05	1.00097003	0.01576158	2238061.31	0.00050682	0.05068237	
40	-0.1935511	-0.0045085	2.03E-05	1.00099868	0.01576211	2238141.82	0.00050222	0.05022168	
41	-0.1470384	-0.0034538	1.19E-05	1.00099833	0.01576217	2238145.96	0.00049815	0.04981496	
42	1.14680843	0.02579785	0.00066541	1.00033249	0.0157514	2238017	0.00050349	0.05034855	
43	-0.7121032	-0.0152672	0.00023314	1.00068946	0.01575792	2238142.76	0.0004546	0.04546031	
44	-0.3543865	-0.0077072	5.94E-05	1.00086632	0.01576074	2238146.9	0.00045564	0.04556378	
45	0.39102577	0.00857475	7.36E-05	1.00092542	0.01576102	2238144.19	0.0004972	0.04972025	
46	-1.0095155	-0.0221894	0.00049237	1.00048147	0.01575412	2238124.48	0.00048292	0.04829215	
47	-0.3062882	-0.0070482	4.97E-05	1.0009766	0.01576171	2238117.97	0.00050534	0.05053352	
48	-0.4481965	-0.0101901	0.00010388	1.00091693	0.0157608	2238122.51	0.00050237	0.05023734	
49	-0.8938618	-0.0194345	0.00037774	1.00057461	0.01575581	2238135.73	0.00047077	0.04707684	
50	-0.573147	-0.0129498	0.00016776	1.00084976	0.01575974	2238117.33	0.00050088	0.050088	
51	-0.2872814	-0.0066261	4.39E-05	1.00098325	0.01576181	2238113.96	0.00050584	0.05058356	
52	-0.3985118	-0.008918	7.96E-05	1.00090492	0.01576089	2238143.71	0.00048451	0.0484509	
53	-0.9095127	-0.0203674	0.00041487	1.00059528	0.01575568	2238087.9	0.00049962	0.04996229	
54	-0.1609999	-0.0037938	1.44E-05	1.00101101	0.01576226	2238135.51	0.00050555	0.0505548	
55	-0.3223656	-0.0074003	5.48E-05	1.00096953	0.01576161	2238125.06	0.00050431	0.05043122	
56	-1.19897	-0.0269044	0.00072369	1.0002893	0.01575067	2237679.98	0.00050606	0.05060623	
57	0.07095557	0.00141814	2.01E-06	1.00101737	0.01576238	2238148.71	0.00050425	0.0504246	

© 2024 Zhang X et al. *JAMA Pediatrics*.

58	-0.4308039	-0.0097008	9.41E-05	1.00090584	0.01576078	2238140.23	0.00049211	0.04921144	
59	-0.6359385	-0.0140681	0.00019797	1.00078095	0.01575894	2238138.6	0.00048204	0.04820439	
60	0.37953035	0.00839862	7.06E-05	1.00094876	0.01576123	2238077.28	0.00050722	0.05072204	
61	-1.234103	-0.0275381	0.00075815	1.00024288	0.01575001	2238022.28	0.00050088	0.050088	
62	-0.972452	-0.0214146	0.0004586	1.0005188	0.0157547	2238124.91	0.00048417	0.04841662	
63	-0.5932642	-0.0133044	0.00017706	1.00082659	0.01575948	2238131.86	0.0004941	0.04940994	
64	-0.1354065	-0.0032149	1.03E-05	1.00101199	0.01576229	2238142.09	0.00050418	0.05041806	
65	0.23046934	0.00495052	2.45E-05	1.00096716	0.01576177	2238148.03	0.00049196	0.04919594	
66	-0.2892315	-0.0066816	4.47E-05	1.00098608	0.01576182	2237873.13	0.00050759	0.05075868	
67	-0.2899884	-0.0066941	4.48E-05	1.00098456	0.01576181	2238072.06	0.00050692	0.05069187	
68	-0.3264102	-0.0075126	5.65E-05	1.00097374	0.01576163	2238015.2	0.0005072	0.05072037	
69	-0.9556087	-0.0198672	0.00039472	1.00047685	0.01575488	2238141.65	0.00043136	0.04313606	
70	-0.3454302	-0.0079218	6.28E-05	1.0009632	0.0157615	2238115.05	0.00050507	0.05050703	
71	-0.3059376	-0.0070323	4.95E-05	1.00097453	0.01576169	2238127.56	0.00050421	0.0504207	
72	-0.5670269	-0.0128951	0.00016634	1.00086398	0.01575987	2237823.15	0.00050718	0.05071828	
73	-0.4802158	-0.0108754	0.00011832	1.00089744	0.01576053	2238128.5	0.00049988	0.04998827	
74	0.12157804	0.00255912	6.55E-06	1.00101372	0.01576231	2238148.46	0.00050513	0.050513	
75	-0.4526862	-0.0103182	0.00010651	1.00091983	0.0157608	2238095.62	0.00050509	0.05050949	
76	-0.457337	-0.0104222	0.00010867	1.0009177	0.01576077	2238094.44	0.00050511	0.05051079	
77	-1.1148958	-0.0249241	0.00062114	1.00038554	0.01575229	2238040.5	0.00050111	0.05011126	
78	-1.2560982	-0.0281588	0.00079269	1.00021709	0.01574951	2237695.54	0.00050583	0.05058336	
79	-0.4424373	-0.0100212	0.00010047	1.00091223	0.01576079	2238133.93	0.00049838	0.04983773	
80	1.45484864	0.03279713	0.00107502	0.99991792	0.01574478	2237991.87	0.00050176	0.0501758	
81	-0.3053069	-0.0069636	4.85E-05	1.00095998	0.01576158	2238141.95	0.00049656	0.0496562	
82	0.95062728	0.02108594	0.00044463	1.00053383	0.01575481	2238123.92	0.00049242	0.04924155	
83	-0.1084017	-0.0026093	6.81E-06	1.00101505	0.01576234	2238143.72	0.00050412	0.05041162	
84	-0.2091646	-0.0048475	2.35E-05	1.00099166	0.01576203	2238143.02	0.00050024	0.0500243	
85	1.18677492	0.02668113	0.00071173	1.00028351	0.01575064	2238036.27	0.00050235	0.05023473	
86	-0.0374839	-0.0009645	9.31E-07	1.00092952	0.01576169	2238148.58	0.0004594	0.04594033	
87	1.60182321	0.03582154	0.00128217	0.99969231	0.01574131	2238073.7	0.00049224	0.04922363	
88	-0.4303967	-0.0095839	9.19E-05	1.00088628	0.01576064	2238143.7	0.00048137	0.04813658	
89	-1.4415129	-0.0322133	0.00103713	0.99996005	0.01574541	2237743.3	0.00050493	0.05049331	
90	-0.6060302	-0.0137128	0.0001881	1.00083418	0.01575946	2238095.31	0.00050333	0.05033267	
91	-0.8541558	-0.0192888	0.00037212	1.00065428	0.01575651	2237586.28	0.00050705	0.05070492	
92	0.71644953	0.0159812	0.00025546	1.00074744	0.01575808	2238110.32	0.00050254	0.05025425	
93	-0.9610653	-0.02151	0.0004627	1.00054674	0.0157549	2238078.74	0.00049994	0.04999374	
94	-1.1322301	-0.0253893	0.00064453	1.00036811	0.01575196	2237928.32	0.00050444	0.05044404	
95	-1.4500212	-0.032268	0.00104064	0.99994789	0.01574528	2237979.4	0.0005008	0.05008009	
96	-1.0384501	-0.0233815	0.00054667	1.00047494	0.01575363	2237179.78	0.00050718	0.05071834	
97	-1.4093956	-0.0313424	0.00098186	1.00000701	0.01574624	2238011.12	0.00049963	0.04996312	
98	-1.1363209	-0.0253152	0.00064077	1.00035874	0.01575191	2238072.5	0.00049795	0.04979472	
99	-1.2004055	-0.0267684	0.00071639	1.00028401	0.01575069	2238045.17	0.00049977	0.04997711	

© 2024 Zhang X et al. *JAMA Pediatrics*.

100	-1.9675847	-0.0437881	0.00191454	0.99903679	0.01573057	2236589.37	0.00050648	0.05064754	
101	-1.3159363	-0.0294603	0.00086758	1.00013782	0.01574825	2237768.66	0.00050523	0.05052286	
102	-0.1702312	-0.0039895	1.59E-05	1.00100398	0.01576219	2238142.28	0.00050278	0.05027822	
103	-1.3838531	-0.030973	0.00095888	1.00004372	0.01574673	2237608.87	0.00050582	0.05058195	
104	-1.1219667	-0.0251864	0.00063427	1.00038067	0.01575215	2237848.25	0.00050539	0.05053887	
105	-0.9546796	-0.0213678	0.00045661	1.00055286	0.015755	2238080.2	0.00049987	0.04998669	
106	-1.0677091	-0.0234288	0.00054888	1.00042152	0.01575317	2238122.66	0.00048213	0.04821262	
107	-0.9561061	-0.0213996	0.00045797	1.0005515	0.01575498	2238079.88	0.00049988	0.04998827	
108	-1.7348532	-0.0386397	0.00149148	0.99948195	0.01573774	2237587.57	0.00050486	0.05048554	
109	-1.1035121	-0.0245687	0.00060356	1.00039504	0.01575251	2238083.73	0.00049685	0.04968543	
110	-1.6984215	-0.037214	0.00138362	0.99956133	0.01573928	2238067.26	0.00048811	0.04881088	
111	0.06689924	0.00132826	1.77E-06	1.00102006	0.0157624	2238148.67	0.00050542	0.05054175	
112	-0.6169246	-0.0139782	0.00019545	1.00083011	0.01575936	2238061.57	0.00050499	0.05049857	
113	-1.1148954	-0.0247207	0.00061104	1.00037933	0.01575232	2238099.85	0.00049296	0.0492957	
114	-1.4742841	-0.0323709	0.00104729	0.99991419	0.01574495	2238087.53	0.00048776	0.04877646	
115	0.78572014	0.01758944	0.00030945	1.00069629	0.01575722	2238070.53	0.00050464	0.05046405	
116	-0.2482117	-0.0057496	3.31E-05	1.00099336	0.01576197	2238123.6	0.00050562	0.05056196	
117	-0.5587026	-0.012576	0.00015821	1.0008509	0.01575983	2238129.98	0.0004967	0.04967023	
118	-0.7826573	-0.0175799	0.00030912	1.00070493	0.01575743	2238098.48	0.00050027	0.05002716	
119	-0.6557021	-0.0148345	0.00022013	1.0008042	0.01575895	2238064.78	0.00050454	0.05045394	
120	0.43849965	0.00938785	8.82E-05	1.00085748	0.01576035	2238146.96	0.00047111	0.04711064	
121	0.67917909	0.01517716	0.00023041	1.00077871	0.01575853	2238078.37	0.00050528	0.05052818	
122	0.62121949	0.01382437	0.00019117	1.00081384	0.01575914	2238120.53	0.00050263	0.05026316	
123	0.61877762	0.01375903	0.00018937	1.00081422	0.01575916	2238124.25	0.00050189	0.05018928	
124	0.32425644	0.00707126	5.00E-05	1.00094866	0.0157614	2238146.15	0.00049637	0.04963693	
125	-0.5545474	-0.012514	0.00015666	1.00085725	0.01575989	2238125.24	0.00049906	0.04990583	
126	-1.1858121	-0.0266192	0.00070844	1.00030555	0.01575093	2237629.55	0.00050627	0.0506268	
127	0.26365396	0.00574237	3.30E-05	1.00097701	0.01576178	2238146.16	0.00050139	0.05013876	
128	-1.1888948	-0.0265117	0.00070273	1.00029788	0.01575091	2238048.96	0.00049961	0.04996145	
129	-1.3054074	-0.0291634	0.0008502	1.00015134	0.01574851	2237947.61	0.00050298	0.05029777	
130	0.80481753	0.01795517	0.00032244	1.00067524	0.01575695	2238111.27	0.00050078	0.05007824	
131	0.44256893	0.00982586	9.66E-05	1.00092093	0.01576079	2238072.24	0.00050699	0.05069873	
132	1.05534916	0.0236354	0.0005586	1.00043417	0.01575309	2238084.64	0.00050036	0.0500356	
133	1.02798685	0.02274044	0.00051711	1.00045255	0.01575357	2238125.15	0.00048858	0.04885805	
134	0.76000306	0.01631876	0.00026635	1.00065996	0.01575728	2238143.3	0.0004646	0.04646016	
135	0.49839144	0.01100364	0.00012113	1.00087878	0.01576024	2238139.04	0.00049879	0.04987919	
136	0.17832171	0.00379199	1.44E-05	1.0009795	0.01576196	2238148.42	0.00049256	0.04925618	
137	-0.1528368	-0.003537	1.25E-05	1.00097326	0.01576196	2238147.45	0.00048603	0.04860251	
138	-0.2705216	-0.006199	3.84E-05	1.00097213	0.01576176	2238142.47	0.00049771	0.04977109	
139	-0.8076345	-0.0181256	0.0003286	1.00068433	0.01575711	2238097.84	0.00049993	0.04999296	
140	0.28972196	0.00630537	3.98E-05	1.00096204	0.0157616	2238146.61	0.00049758	0.04975818	
141	-0.316016	-0.007267	5.28E-05	1.00097382	0.01576166	2238114.41	0.00050548	0.05054821	

© 2024 Zhang X et al. *JAMA Pediatrics.*

142	-0.6104668	-0.0138476	0.00019182	1.00083574	0.01575944	2238019.85	0.00050595	0.05059457	
143	-0.0554413	-0.0014186	2.01E-06	1.00101564	0.01576238	2238146.94	0.0005024	0.05023956	
144	0.0811378	0.00162884	2.65E-06	1.00099144	0.01576216	2238148.73	0.0004918	0.04918014	
145	-1.0097355	-0.0223341	0.00049881	1.00048729	0.01575412	2238116.56	0.00048903	0.04890302	
146	-0.4442336	-0.0099493	9.90E-05	1.00089163	0.01576063	2238141.81	0.00048751	0.04875113	
147	-1.4249507	-0.0317949	0.00101039	0.99998448	0.01574582	2237900.21	0.0005032	0.05031992	
148	0.4039487	0.00894191	8.00E-05	1.00093645	0.01576105	2238119.81	0.00050604	0.05060396	
149	-0.5989134	-0.0136105	0.00018531	1.00084538	0.01575957	2237545.89	0.00050745	0.05074506	
150	-0.0645262	-0.0016181	2.62E-06	1.00101061	0.01576234	2238147.29	0.00050014	0.05001413	
151	-0.261245	-0.0060278	3.64E-05	1.00098563	0.01576188	2238135.25	0.00050337	0.05033718	
152	-0.4236122	-0.009618	9.25E-05	1.0009231	0.01576094	2238132.44	0.00049984	0.04998432	
153	-0.3927076	-0.0089553	8.02E-05	1.00094075	0.01576118	2238127.17	0.00050251	0.05025074	
154	0.63842836	0.01421405	0.0002021	1.00080255	0.01575896	2238118.73	0.00050262	0.05026249	
155	-0.5488535	-0.0123105	0.0001516	1.00084984	0.01575987	2238135.19	0.0004929	0.04929018	
156	1.17717746	0.02629772	0.00069143	1.00029154	0.01575086	2238096.72	0.00049615	0.04961529	
157	-0.8881987	-0.0199803	0.00039926	1.00061978	0.01575601	2238030.75	0.00050378	0.05037819	
158	0.59270886	0.01314459	0.00017284	1.00082751	0.0157594	2238131.91	0.0005	0.04999989	
159	0.50528773	0.01104998	0.00012215	1.00085797	0.01576007	2238143.9	0.00048899	0.04889861	
160	0.21783738	0.00471441	2.22E-05	1.00098965	0.01576197	2238147.18	0.00050188	0.05018752	
161	-0.0102283	-0.0003957	1.57E-07	1.000983	0.01576213	2238148.53	0.00048566	0.04856649	
162	0.35234008	0.00773649	5.99E-05	1.00094777	0.01576131	2238143.25	0.00050112	0.05011182	
163	-0.6380961	-0.0142131	0.00020207	1.00079035	0.01575899	2238134.96	0.00048872	0.04887176	
164	-0.1440149	-0.0034046	1.16E-05	1.00100896	0.01576226	2238143.02	0.00050325	0.05032495	
165	0.35220735	0.00773425	5.98E-05	1.000948	0.01576132	2238143.18	0.00050122	0.05012177	
166	-0.4288802	-0.0096093	9.24E-05	1.00089763	0.01576073	2238142.31	0.0004872	0.04871988	
167	0.57369575	0.01269863	0.00016131	1.00083677	0.01575957	2238135.4	0.00049867	0.04986683	
168	-0.452051	-0.0100899	0.00010184	1.00088286	0.01576054	2238142.51	0.00048458	0.04845764	
169	-0.0491055	-0.0012649	1.60E-06	1.00099956	0.01576225	2238148.08	0.00049428	0.04942844	
170	-0.4711212	-0.0107299	0.00011518	1.0009112	0.01576066	2238091.52	0.00050511	0.05051135	
171	0.59941421	0.01331282	0.00017729	1.00082547	0.01575935	2238128.11	0.00050128	0.05012771	
172	0.68008609	0.01505758	0.00022679	1.00076366	0.01575844	2238133.5	0.00049595	0.04959458	
173	-0.1906576	-0.0044066	1.94E-05	1.0009832	0.01576199	2238145.96	0.00049415	0.04941461	
174	-0.594974	-0.0134362	0.00018059	1.00083713	0.01575954	2238114.39	0.00050104	0.05010445	
175	-0.5550735	-0.0121389	0.0001474	1.0008055	0.01575955	2238143.45	0.00046898	0.04689806	
176	-0.1204685	-0.0028908	8.36E-06	1.00102097	0.01576238	2237961.25	0.00050773	0.05077301	
177	1.05894982	0.02378445	0.00056566	1.0004327	0.01575302	2238044.92	0.00050319	0.05031885	
178	0.87473666	0.01967612	0.00038719	1.0006231	0.015756	2234957.6	0.00050774	0.05077434	
179	0.33451951	0.00734503	5.40E-05	1.00095678	0.01576144	2238142.74	0.00050248	0.05024826	
180	-0.197694	-0.0046093	2.13E-05	1.00100133	0.01576213	2238138.4	0.00050396	0.050396	
181	-0.6559726	-0.0147954	0.00021897	1.00079919	0.01575892	2238105.04	0.00050149	0.05014944	
182	-0.3831557	-0.0087753	7.70E-05	1.00095145	0.01576129	2238080.38	0.00050622	0.0506225	
183	1.00726665	0.02252997	0.00050759	1.00048477	0.01575391	2238094.68	0.00049982	0.04998193	

© 2024 Zhang X et al. *JAMA Pediatrics.*

184	0.0939799	0.00193119	3.73E-06	1.00100968	0.0157623	2238148.72	0.00050148	0.05014844	
185	-0.3656153	-0.0083586	6.99E-05	1.0009527	0.01576136	2238125.93	0.00050335	0.05033544	
186	-1.0738423	-0.0241761	0.00058444	1.00043681	0.01575301	2235024.8	0.00050763	0.05076253	
187	0.04660411	0.00086028	7.40E-07	1.00098911	0.01576216	2238148.69	0.00048942	0.04894173	
188	-0.6538448	-0.0146753	0.00021543	1.00079279	0.0157589	2238124.28	0.00049658	0.04965778	
189	-0.4150308	-0.0093453	8.74E-05	1.00091094	0.01576088	2238141.13	0.00049131	0.04913076	
190	0.07271871	0.00145853	2.13E-06	1.00101839	0.01576239	2238148.71	0.00050482	0.05048218	
191	1.49891377	0.03347517	0.00111988	0.99985361	0.0157439	2238084.39	0.00049202	0.04920218	
192	0.3339841	0.00731184	5.35E-05	1.00095135	0.0157614	2238144.88	0.00049953	0.04995296	
193	0.41136991	0.00911477	8.31E-05	1.00093425	0.01576101	2238105.54	0.00050658	0.05065792	
194	-0.102309	-0.0024638	6.07E-06	1.00100884	0.0157623	2238146.28	0.00050072	0.050072	
195	-0.155222	-0.0036471	1.33E-05	1.0010029	0.0157622	2238144.49	0.00050104	0.05010387	
196	0.36586594	0.00787528	6.20E-05	1.00090347	0.01576094	2238147.24	0.00048028	0.0480279	
197	-0.0447216	-0.0011744	1.38E-06	1.0010101	0.01576234	2238147.75	0.00049942	0.04994166	
198	-0.7213192	-0.0160628	0.00025808	1.000737	0.0157581	2238129.88	0.00049045	0.04904512	
199	0.29861769	0.00650437	4.23E-05	1.00095918	0.01576155	2238146.44	0.00049752	0.04975161	
200	-0.2278605	-0.0052969	2.81E-05	1.00099945	0.01576206	2238117.85	0.00050625	0.05062484	
201	0.48412348	0.01063118	0.00011306	1.00087746	0.0157603	2238142.83	0.00049396	0.04939573	
202	1.25751339	0.02789735	0.00077803	1.00018858	0.01574934	2238112.85	0.0004883	0.04883002	
203	-0.0319872	-0.0008902	7.93E-07	1.00101115	0.01576235	2238147.9	0.00049974	0.04997386	
204	-0.1517573	-0.0034146	1.17E-05	1.00092191	0.01576155	2238148.16	0.00046026	0.04602613	
205	0.33733516	0.00742069	5.51E-05	1.000959	0.01576144	2238139.8	0.00050417	0.05041739	
206	-0.6939852	-0.0155131	0.00024072	1.00076098	0.01575844	2238127.29	0.00049356	0.04935637	
207	1.16800173	0.0262355	0.00068817	1.00030596	0.01575101	2238051.46	0.0005017	0.05017024	
208	0.04375917	0.00079909	6.39E-07	1.00099502	0.01576221	2238148.68	0.00049227	0.04922664	
209	1.8351802	0.04044246	0.00163377	0.99931078	0.01573545	2238099.87	0.00047591	0.04759102	
210	1.07092836	0.02406435	0.00057904	1.00041971	0.0157528	2238035.67	0.00050346	0.05034642	
211	-0.8851222	-0.0198132	0.00039261	1.00061644	0.01575603	2238096.17	0.00049879	0.04987919	
212	0.6435237	0.01436823	0.00020651	1.00080352	0.01575893	2238084.4	0.00050537	0.05053668	
213	-0.4512551	-0.0102215	0.00010452	1.00090911	0.01576073	2238132.65	0.00049883	0.04988324	
214	-0.5911083	-0.0133728	0.00017889	1.00084223	0.01575959	2238103.74	0.00050271	0.05027113	
215	-1.2557439	-0.0281579	0.00079264	1.00021765	0.01574952	2237631.49	0.00050608	0.05060833	
216	0.50389004	0.01120257	0.00012555	1.00088795	0.01576028	2238108.14	0.00050563	0.05056312	
217	-0.5547465	-0.0125348	0.00015718	1.00085935	0.01575991	2238121.17	0.00050035	0.0500349	
218	-1.6822359	-0.0371506	0.00137891	0.99958198	0.01573949	2238018.3	0.00049573	0.04957312	
219	-1.2582198	-0.0282282	0.0007966	1.00021469	0.01574946	2237384.62	0.00050665	0.05066473	
220	-1.3825971	-0.0309371	0.00095666	1.00004549	0.01574677	2237673.82	0.00050555	0.05055467	
221	-1.0802981	-0.0241744	0.00058436	1.00042459	0.01575291	2238038.72	0.0005016	0.05015977	
222	-0.9729143	-0.0218311	0.00047662	1.00053804	0.01575471	2238038.68	0.0005027	0.05026982	
223	-1.0220125	-0.022997	0.00052885	1.00049152	0.01575391	2237748.29	0.00050629	0.05062937	
224	-0.9859579	-0.0221019	0.00048851	1.00052419	0.0157545	2238050.86	0.00050191	0.05019147	
225	-1.6976944	-0.0375796	0.00141089	0.99955339	0.01573899	2237981.46	0.00049823	0.04982305	

© 2024 Zhang X et al. *JAMA Pediatrics*.

226	-1.2550135	-0.028108	0.00078983	1.00021808	0.01574955	2237843.55	0.00050486	0.05048643	
227	-0.9278266	-0.0206301	0.00042563	1.00057047	0.01575539	2238114.12	0.00049287	0.0492874	
228	0.51288448	0.01135876	0.00012907	1.00087567	0.01576015	2238134.27	0.00050136	0.05013564	
229	-0.832158	-0.0187896	0.00035311	1.00067262	0.01575681	2237832.91	0.0005065	0.05064951	
230	0.45755154	0.01011493	0.00010235	1.00090457	0.01576061	2238136.83	0.00050186	0.05018619	
231	-0.4361947	-0.0099674	9.94E-05	1.00093046	0.01576094	2238012.48	0.00050684	0.05068362	
232	-0.3252029	-0.00749	5.61E-05	1.00097527	0.01576165	2236445.52	0.00050779	0.05077931	
233	-0.2306663	-0.0052675	2.78E-05	1.00096576	0.01576179	2238146.03	0.00048946	0.04894593	
234	1.52682855	0.03416475	0.00116644	0.99981035	0.01574319	2238074.36	0.00049361	0.04936141	
235	1.00573185	0.02194721	0.00048167	1.0004628	0.01575394	2238135.19	0.00047578	0.04757831	
236	-0.7015641	-0.0157472	0.00024804	1.00076215	0.01575839	2238117.63	0.0004978	0.04977992	
237	0.32200048	0.00704194	4.96E-05	1.00095529	0.01576146	2238145.27	0.00049944	0.04994429	
238	-0.6206366	-0.0139752	0.00019537	1.00081784	0.01575926	2238120.62	0.0004989	0.04989024	
239	0.00945171	3.72E-05	1.39E-09	1.00099771	0.01576224	2238148.55	0.00049301	0.04930118	
240	0.51553856	0.01148108	0.00013187	1.00088395	0.0157602	2238046.68	0.00050691	0.05069144	
241	0.34139055	0.0073452	5.40E-05	1.00091456	0.01576109	2238147.42	0.00048154	0.04815386	
242	0.1879749	0.00405976	1.65E-05	1.00100473	0.01576214	2238145.31	0.00050622	0.05062188	
243	0.5154166	0.01139678	0.00012993	1.00087135	0.01576011	2238137.13	0.00049966	0.04996563	
244	0.36567257	0.00808942	6.55E-05	1.0009554	0.01576133	2220918.81	0.00050784	0.05078406	
245	0.03449321	0.00058768	3.46E-07	1.00096927	0.01576201	2238148.69	0.0004793	0.04793029	
246	-1.3031323	-0.0290762	0.00084513	1.00015398	0.01574857	2237990.29	0.00050169	0.05016883	
247	0.9721345	0.02173842	0.00047257	1.0005209	0.01575448	2238096.82	0.00050011	0.05001118	
248	-0.2441674	-0.0056582	3.20E-05	1.00099412	0.01576199	2238125.48	0.0005055	0.05054986	
249	0.15603558	0.0033243	1.11E-05	1.00100136	0.01576217	2238148.3	0.00050155	0.05015489	
250	3.4684064	0.07537963	0.00565596	0.99536565	0.01567256	2238046.9	0.00045084	0.04508371	*
251	-0.3425272	-0.0078763	6.21E-05	1.00096889	0.01576155	2237857.56	0.00050753	0.05075262	
252	3.89019385	0.08831433	0.00774587	0.99346193	0.01564144	2237792.83	0.0004873	0.04873041	*
253	-0.5807349	-0.0132011	0.00017433	1.00085592	0.01575974	2237809.79	0.00050718	0.05071818	
254	0.09579507	0.00197701	3.91E-06	1.00101521	0.01576234	2238148.71	0.00050433	0.05043306	
255	0.12021887	0.00252891	6.40E-06	1.00101425	0.01576231	2238148.46	0.00050531	0.05053052	
256	2.09691714	0.03871921	0.001498	0.99916089	0.01573533	2238136.71	0.00033404	0.03340437	
257	-0.9860782	-0.0221434	0.00049034	1.00052589	0.0157545	2238009.46	0.00050367	0.05036748	
258	-0.6583703	-0.0149253	0.00022283	1.00080577	0.01575895	2237913.28	0.00050666	0.05066559	
259	2.53570902	0.05592605	0.00311997	0.99784394	0.015712	2238066.41	0.00047077	0.04707684	
260	0.38341156	0.00839544	7.05E-05	1.00092658	0.01576105	2238144.79	0.00049619	0.04961867	
261	0.37826144	0.00836607	7.00E-05	1.00094839	0.01576123	2238108.3	0.00050675	0.05067519	
262	-0.6010891	-0.0136419	0.00018617	1.00084195	0.01575953	2238004.99	0.00050619	0.05061874	
263	1.44681251	0.03256331	0.00105976	0.99993013	0.015745	2238024.58	0.00050024	0.0500243	
264	0.62370458	0.01363561	0.00018598	1.00078354	0.01575894	2238142.23	0.00048491	0.04849074	
265	-1.2337678	-0.0262705	0.00068999	1.00022188	0.01575039	2238131.86	0.00045597	0.04559738	
266	2.31238226	0.0483744	0.00233627	0.99851904	0.01572355	2238117.81	0.00042592	0.0425918	
267	-0.7167679	-0.0162258	0.00026334	1.00076456	0.01575829	2237878.32	0.00050664	0.05066404	

© 2024 Zhang X et al. *JAMA Pediatrics*.

268	0.37042867	0.00806947	6.51E-05	1.00092348	0.01576107	2238146.09	0.00049183	0.04918332	
269	0.30078381	0.00660973	4.37E-05	1.00097561	0.01576167	2238132.14	0.0005064	0.05063979	
270	0.46448757	0.01018035	0.00010368	1.00088508	0.01576044	2238143.78	0.00049287	0.0492874	
271	0.32212049	0.00700433	4.91E-05	1.00094411	0.01576137	2238146.68	0.00049361	0.04936141	
272	-0.1044987	-0.002503	6.27E-06	1.00100135	0.01576224	2238147.07	0.0004971	0.04971032	
273	-0.2613466	-0.0059957	3.60E-05	1.00097475	0.0157618	2238142.73	0.00049781	0.04978118	
274	0.0991379	0.00205236	4.21E-06	1.00101499	0.01576234	2238148.69	0.0005044	0.05044026	
275	2.27201403	0.05173281	0.00267046	0.99834721	0.01571955	2237598.89	0.00050343	0.05034306	
276	0.86042546	0.01926157	0.00037106	1.00063034	0.01575619	2238083.38	0.00050315	0.05031505	
277	0.96503027	0.0213576	0.00045616	1.00051724	0.01575458	2238126.57	0.00048999	0.04899888	
278	-1.2952395	-0.0290777	0.00084522	1.00016639	0.01574867	2219702.43	0.00050779	0.05077906	
279	1.44647958	0.03237919	0.00104783	0.99993129	0.01574511	2238075.97	0.00049487	0.04948736	
280	0.60532232	0.01321681	0.00017474	1.00079368	0.01575911	2238142.85	0.00048417	0.04841662	
281	0.31826803	0.00697849	4.87E-05	1.0009623	0.01576152	2238143.53	0.00050245	0.05024467	
282	1.81768124	0.03986611	0.00158762	0.99934783	0.01573612	2238106.51	0.0004716	0.04716021	
283	1.5812752	0.03527122	0.00124313	0.99972659	0.0157419	2238085.03	0.00048995	0.04899492	
284	2.06701905	0.04671166	0.00217832	0.99881811	0.0157272	2237947.88	0.00049791	0.04979107	
285	0.6026476	0.01324478	0.00017548	1.00080601	0.01575921	2238140.72	0.00049067	0.04906735	
286	-0.8238111	-0.0186157	0.00034661	1.00068056	0.01575693	2237566.45	0.00050713	0.05071251	
287	1.33645777	0.02967599	0.00088032	1.00008575	0.01574769	2238107.97	0.00048825	0.04882527	
288	-0.5057333	-0.0115248	0.00013287	1.00089746	0.01576041	2237916.65	0.00050708	0.05070831	
289	0.31968744	0.00702451	4.94E-05	1.00096577	0.01576155	2238140.22	0.00050451	0.0504508	
290	-0.9036326	-0.0204023	0.0004163	1.00061041	0.01575579	2231901.3	0.00050776	0.05077637	
291	-0.9900498	-0.0223213	0.00049825	1.00052609	0.01575444	2230492.15	0.00050777	0.05077665	
292	0.18173146	0.00392428	1.54E-05	1.0010089	0.01576219	2238115.75	0.00050769	0.05076947	
293	-0.696992	-0.0157878	0.00024932	1.00077914	0.01575852	2237853.88	0.00050679	0.05067938	
294	1.48271875	0.03361485	0.00112923	0.99987448	0.015744	2236821.49	0.00050709	0.05070948	
295	-0.0153048	-0.0005228	2.73E-07	1.00102755	0.01576249	2238108.55	0.00050772	0.05077193	
296	0.146021	0.00311642	9.72E-06	1.00101468	0.01576229	2238144.26	0.0005074	0.05074001	
297	-0.6132803	-0.0139228	0.00019391	1.00083545	0.01575942	2237901.81	0.00050684	0.05068444	
298	0.0061804	-3.83E-05	1.47E-09	1.00102501	0.01576247	2238146.47	0.0005065	0.05064953	
299	-0.3922605	-0.0089907	8.09E-05	1.00095031	0.01576125	2237775.57	0.00050753	0.05075348	
300	-0.5739087	-0.0130513	0.0001704	1.00086029	0.01575981	2237675.35	0.00050738	0.05073792	
301	-0.0122954	-0.0004547	2.07E-07	1.00102655	0.01576248	2238141.27	0.00050722	0.05072172	
302	0.53993487	0.0120176	0.00014448	1.00086783	0.01575996	2238105.01	0.00050542	0.05054226	
303	-0.3756144	-0.0086179	7.43E-05	1.00095683	0.01576136	2237794.25	0.00050754	0.05075408	
304	0.66838285	0.01496676	0.00022407	1.00079004	0.01575868	2237168.92	0.00050767	0.05076657	
305	-1.1200423	-0.025203	0.00063511	1.00038467	0.01575217	2228040.33	0.00050777	0.05077702	
306	-1.5921539	-0.0355146	0.00126029	0.99972558	0.01574165	2237678.98	0.00050481	0.05048116	
307	0.04266638	0.00078423	6.15E-07	1.00102644	0.01576247	2238127.38	0.0005078	0.0507803	
308	0.29149398	0.00634501	4.03E-05	1.00096148	0.01576159	2238146.57	0.00049757	0.04975688	
309	0.17989384	0.00387491	1.50E-05	1.00100501	0.01576216	2238146.66	0.00050555	0.05055539	

© 2024 Zhang X et al. *JAMA Pediatrics*.

310	-0.3838797	-0.0087472	7.65E-05	1.00094179	0.01576122	2238132.01	0.00050122	0.05012177	
311	0.29868706	0.00656907	4.32E-05	1.00097835	0.0157617	2238085.76	0.00050747	0.05074698	
312	1.10189361	0.02485541	0.00061772	1.00038747	0.01575223	2237601.28	0.00050688	0.05068816	
313	0.48084812	0.01066105	0.0001137	1.0008966	0.01576045	2238128.92	0.00050379	0.05037921	
314	-0.5086183	-0.0115705	0.00013393	1.00089312	0.01576037	2238073.86	0.00050547	0.05054654	
315	1.67958783	0.038161	0.00145484	0.99955052	0.0157388	2236405.95	0.0005071	0.05071018	
316	0.79760487	0.01785223	0.00031876	1.00068589	0.01575706	2238077.46	0.00050421	0.05042087	
317	0.0365653	0.00064593	4.17E-07	1.00102375	0.01576245	2238147.99	0.00050633	0.05063258	
318	-0.2936766	-0.0067807	4.60E-05	1.00098458	0.0157618	2237945.05	0.00050749	0.0507488	
319	-0.0446813	-0.0011843	1.40E-06	1.00102634	0.01576247	2238126.72	0.00050746	0.05074632	
320	0.33358228	0.00732393	5.37E-05	1.00095712	0.01576144	2238142.78	0.00050249	0.05024861	
321	0.39881562	0.00882728	7.80E-05	1.00093897	0.01576109	2238117.48	0.00050623	0.05062269	
322	-0.0555318	-0.0014292	2.04E-06	1.00102662	0.01576247	2236648.9	0.00050784	0.05078364	
323	0.80445897	0.01806919	0.00032655	1.00068483	0.01575699	2236817.78	0.00050764	0.05076447	
324	0.08102738	0.0016269	2.65E-06	1.00099214	0.01576217	2238148.73	0.00049215	0.04921451	
325	0.85605516	0.01924696	0.0003705	1.00063989	0.01575627	2236776.79	0.00050762	0.05076221	
326	0.17320719	0.00373149	1.39E-05	1.00101048	0.01576221	2238126.07	0.00050766	0.0507663	
327	1.29061108	0.0290914	0.00084601	1.00015099	0.01574849	2237973.86	0.00050361	0.05036135	
328	0.4039131	0.00892809	7.97E-05	1.00093369	0.01576103	2238132.92	0.00050454	0.05045421	
329	0.78738467	0.01765328	0.0003117	1.00069699	0.01575721	2238001.5	0.00050613	0.05061344	
330	0.33276477	0.00729261	5.32E-05	1.00095395	0.01576142	2238144.31	0.00050068	0.05006756	
331	0.20797474	0.00423999	1.80E-05	1.00087967	0.01576112	2238148.59	0.00044506	0.0445064	
332	-0.5675591	-0.0128283	0.00016462	1.00085331	0.0157598	2238116.98	0.00050107	0.0501073	
333	-1.6073998	-0.0359492	0.00129128	0.99969883	0.01574117	2230060.53	0.00050766	0.05076636	
334	-0.8177014	-0.0184909	0.00034198	1.00068652	0.01575702	2234537.66	0.00050773	0.05077287	
335	-0.7192559	-0.016297	0.00026566	1.0007642	0.01575826	2236671.55	0.00050762	0.05076214	
336	-0.7047228	-0.0159736	0.00025523	1.00077488	0.01575843	2236491.26	0.00050765	0.05076525	
337	1.04314408	0.02344767	0.00054976	1.00045084	0.01575329	2238020.07	0.00050421	0.05042087	
338	0.88098101	0.01977097	0.00039093	1.00061436	0.0157559	2238022.25	0.00050529	0.05052899	
339	-0.8294883	-0.0187531	0.00035174	1.00067653	0.01575686	2234573.04	0.00050772	0.05077245	
340	1.26522128	0.02862407	0.00081907	1.00018611	0.01574899	2234756.57	0.00050763	0.05076341	
341	0.21700761	0.00469005	2.20E-05	1.00098735	0.01576196	2238147.47	0.00050061	0.05006113	
342	0.70088452	0.01570586	0.00024674	1.00076653	0.0157583	2237426.35	0.00050758	0.05075751	
343	0.31672437	0.00697363	4.87E-05	1.00097133	0.0157616	2238119.75	0.00050689	0.05068902	
344	0.33958923	0.00746261	5.57E-05	1.00095585	0.01576141	2238141.93	0.00050294	0.05029362	
345	0.6693443	0.01497762	0.00022439	1.00078819	0.01575866	2237961.27	0.00050691	0.05069135	
346	-0.5829434	-0.0132579	0.00017584	1.00085556	0.01575973	2235652.48	0.00050775	0.05077525	
347	-0.2378505	-0.0055092	3.04E-05	1.00099304	0.01576199	2238134.46	0.00050417	0.05041723	
348	-0.817821	-0.0184785	0.00034152	1.00068532	0.01575701	2237709.26	0.0005069	0.05069043	
349	-0.397569	-0.0090271	8.15E-05	1.00093138	0.01576109	2238136.16	0.00049852	0.04985197	
350	0.27114812	0.0059316	3.52E-05	1.00098191	0.0157618	2238142.43	0.000505	0.05049998	
351	0.29801181	0.00655265	4.30E-05	1.00097821	0.0157617	2238106.55	0.00050729	0.05072892	

© 2024 Zhang X et al. *JAMA Pediatrics.*

352	0.44210997	0.00980625	9.62E-05	1.00091939	0.01576078	2238112.84	0.00050602	0.05060233	
353	-0.878805	-0.0194952	0.00038011	1.00061092	0.01575609	2238122.76	0.00048979	0.04897888	
354	0.70892802	0.01587264	0.00025201	1.00075898	0.0157582	2237999.3	0.00050651	0.0506514	
355	0.26138739	0.00571715	3.27E-05	1.00098677	0.01576186	2238139.64	0.00050607	0.05060736	
356	0.37336447	0.00826252	6.83E-05	1.00095207	0.01576128	2237894.48	0.00050768	0.05076752	
357	-0.3586474	-0.0082339	6.78E-05	1.0009623	0.01576145	2238018.67	0.00050708	0.05070794	
358	0.3079009	0.00677462	4.59E-05	1.00097446	0.01576165	2238118.54	0.000507	0.05069964	
359	1.41869295	0.03169121	0.00100381	0.99997184	0.01574578	2238087.09	0.00049315	0.0493147	
360	-0.6847671	-0.0155148	0.00024078	1.00078773	0.01575865	2237875.62	0.00050675	0.05067455	
361	-1.0443009	-0.0235057	0.0005525	1.00046845	0.01575353	2237426.27	0.00050695	0.05069492	
362	-0.8986679	-0.0202848	0.00041152	1.00061458	0.01575587	2237028.77	0.00050741	0.05074056	
363	-0.8128443	-0.0183828	0.00033799	1.0006906	0.01575708	2234698.91	0.00050772	0.05077247	
364	-0.5578009	-0.0126951	0.00016123	1.00087011	0.01575996	2237008.46	0.00050766	0.05076597	
365	-0.45939	-0.0104959	0.00011021	1.00092155	0.01576079	2236190.1	0.00050777	0.05077665	
366	-0.371086	-0.0085185	7.26E-05	1.000959	0.01576139	2236452.06	0.00050778	0.0507781	
367	-0.3915381	-0.0089767	8.06E-05	1.00095105	0.01576126	2236441.06	0.00050778	0.05077758	
368	-0.3172318	-0.007311	5.35E-05	1.00097782	0.01576169	2237056.57	0.00050777	0.05077682	
369	-0.4384197	-0.0099778	9.96E-05	1.00092229	0.01576088	2238120.72	0.00050291	0.05029092	
370	0.41723588	0.00925883	8.58E-05	1.00093397	0.01576098	2235851.99	0.00050782	0.05078183	
371	0.37821464	0.00837248	7.01E-05	1.00095014	0.01576125	2237888.09	0.00050767	0.05076738	
372	-0.252153	-0.005848	3.42E-05	1.00099572	0.01576198	2238036.65	0.00050733	0.05073322	
373	-0.4095073	-0.0093759	8.79E-05	1.00094305	0.01576114	2237855.75	0.00050742	0.05074215	
374	-0.6445659	-0.0146295	0.00021409	1.0008163	0.0157591	2237416.78	0.00050748	0.05074751	
375	-0.2789886	-0.0064532	4.17E-05	1.00098943	0.01576187	2236672.68	0.0005078	0.05077977	
376	-0.0797381	-0.0019588	3.84E-06	1.00101032	0.01576233	2238146.9	0.0005005	0.05004993	
377	-0.0610263	-0.0015478	2.40E-06	1.00102022	0.01576242	2238145.24	0.00050481	0.05048082	
378	-0.5773281	-0.0130803	0.00017115	1.00085217	0.01575974	2238094.52	0.00050376	0.05037551	
379	0.32819896	0.00719817	5.18E-05	1.00095779	0.01576146	2238143.63	0.00050186	0.05018575	
380	0.82680156	0.01857106	0.00034494	1.00066513	0.01575668	2237715.66	0.0005072	0.05071954	
381	-1.0678741	-0.0240066	0.00057628	1.00044204	0.01575312	2237772.61	0.00050605	0.0506054	
382	-0.0352815	-0.0009689	9.39E-07	1.00102003	0.01576242	2238146.78	0.00050418	0.05041823	
383	1.03429028	0.02330181	0.00054295	1.00046265	0.01575344	2237764.03	0.00050665	0.05066503	
384	-0.6012598	-0.0136375	0.00018604	1.00084084	0.01575953	2238043.55	0.00050558	0.05055796	
385	-0.4924918	-0.0112261	0.00012608	1.00090379	0.01576052	2237980.23	0.00050684	0.05068429	
386	-0.1915275	-0.0044709	2.00E-05	1.00100238	0.01576214	2238139.04	0.00050389	0.05038856	
387	-0.578654	-0.0130374	0.00017003	1.00084213	0.01575966	2238125.56	0.00049825	0.04982534	
388	-1.5638483	-0.0349972	0.00122388	0.99977003	0.01574231	2192919.85	0.00050781	0.05078125	
389	-0.793413	-0.0179503	0.00032228	1.00070666	0.01575734	2234768.68	0.00050773	0.05077274	
390	-0.8402641	-0.0189112	0.00035769	1.0006616	0.01575669	2238051.56	0.00050339	0.05033867	
391	-0.7053549	-0.0158553	0.00025146	1.00076178	0.01575836	2238111.68	0.00049933	0.04993275	
392	-1.3138022	-0.0294405	0.00086642	1.00014096	0.01574829	2237558.04	0.00050617	0.05061657	
393	0.01786216	0.00022479	5.06E-08	1.00102651	0.01576248	2238144.21	0.00050736	0.05073575	

© 2024 Zhang X et al. *JAMA Pediatrics*.

394	-0.6715372	-0.0152326	0.0002321	1.00079823	0.01575881	2237069.69	0.00050757	0.05075746	
395	-1.2924481	-0.029016	0.00084164	1.00017011	0.01574873	2220792.26	0.00050779	0.05077876	
396	0.08365922	0.00170908	2.92E-06	1.00102321	0.01576242	2238148.73	0.0005077	0.0507698	
397	-0.6431016	-0.0145053	0.00021047	1.00080715	0.01575906	2238108.61	0.00050117	0.05011684	
398	0.08893337	0.00182046	3.32E-06	1.00101324	0.01576233	2238148.72	0.000503	0.05030011	
399	-0.2133737	-0.0049694	2.47E-05	1.00100167	0.0157621	2238127.36	0.00050575	0.0505749	
400	-0.3581494	-0.008224	6.77E-05	1.00096276	0.01576146	2237986.91	0.00050723	0.05072306	
401	-0.1533469	-0.0036286	1.32E-05	1.00101563	0.0157623	2238098.95	0.00050727	0.05072703	
402	-0.1835752	-0.0043078	1.86E-05	1.00101056	0.01576222	2238086.8	0.00050726	0.05072577	
403	1.74467166	0.03907558	0.00152533	0.99945091	0.01573744	2238058.78	0.00049227	0.04922664	
404	-0.0204544	-0.0006382	4.08E-07	1.00102589	0.01576247	2238142.74	0.00050692	0.05069249	
405	0.70088452	0.01570586	0.00024674	1.00076653	0.0157583	2237426.35	0.00050758	0.05075751	
406	-0.8294883	-0.0187531	0.00035174	1.00067653	0.01575686	2234573.04	0.00050772	0.05077245	
407	-0.1228897	-0.0029406	8.65E-06	1.00101762	0.01576235	2238135.54	0.0005062	0.05062032	
408	-0.072522	-0.001811	3.28E-06	1.00102477	0.01576245	2238119.96	0.00050741	0.05074127	
409	-0.4838801	-0.0109995	0.00012104	1.00090256	0.01576055	2238108.85	0.00050375	0.05037468	
410	-0.8011264	-0.0181235	0.00032852	1.00070044	0.01575724	2226680.54	0.00050781	0.05078083	
411	0.2532043	0.00553683	3.07E-05	1.00099071	0.01576191	2238132.56	0.00050694	0.05069391	
412	-0.3232957	-0.0074335	5.53E-05	1.00097239	0.01576163	2238104.12	0.00050596	0.05059595	
413	0.90481626	0.02013977	0.00040564	1.00058196	0.01575552	2238118.15	0.00049662	0.04966248	
414	-0.9046303	-0.0204036	0.00041635	1.00060824	0.01575578	2237705.14	0.00050673	0.05067254	
415	-0.7355697	-0.0166444	0.00027711	1.00075057	0.01575806	2237863.1	0.00050665	0.05066503	
416	-0.5307232	-0.0120917	0.00014627	1.00088546	0.01576021	2233438.02	0.0005078	0.05078022	
417	-1.6202855	-0.0362259	0.00131121	0.99967749	0.01574083	2234018.62	0.00050749	0.05074864	
418	-1.5989936	-0.0357594	0.0012777	0.99971283	0.0157414	2233721.48	0.00050752	0.05075186	
419	-1.4212383	-0.0318483	0.00101378	0.99998976	0.01574584	2234746.26	0.00050751	0.05075053	
420	0.51915682	0.01154652	0.00013337	1.0008794	0.01576015	2238108.73	0.00050543	0.05054327	
421	-0.2539687	-0.005887	3.47E-05	1.00099467	0.01576197	2238077.4	0.00050703	0.05070318	
422	-0.1426792	-0.0033885	1.15E-05	1.00101703	0.01576233	2238109	0.00050719	0.05071886	
423	-0.2748888	-0.0063348	4.01E-05	1.00098261	0.01576183	2238133.01	0.0005037	0.05036984	
424	1.47865012	0.03298982	0.00108768	0.99988426	0.01574441	2238089.03	0.00049127	0.04912736	
425	-0.0520882	-0.0013432	1.81E-06	1.00101539	0.01576238	2238147.08	0.00050219	0.05021932	
426	0.39077467	0.00865813	7.50E-05	1.00094521	0.01576116	2237453.54	0.00050777	0.0507774	
427	0.27703407	0.00608004	3.70E-05	1.00098537	0.01576181	2238041.05	0.00050767	0.05076665	
428	-0.2443805	-0.0056671	3.21E-05	1.00099547	0.015762	2238115.24	0.00050621	0.05062125	
429	0.23925679	0.00522365	2.73E-05	1.00099524	0.01576197	2238122.25	0.00050738	0.05073782	
430	-0.6597251	-0.0149727	0.00022425	1.00080668	0.01575894	2227984.1	0.00050782	0.05078153	
431	-0.6803477	-0.0154316	0.0002382	1.00079237	0.01575871	2235170.77	0.00050774	0.05077434	
432	-0.8652798	-0.019496	0.00038014	1.00064183	0.01575634	2237989.33	0.00050498	0.05049766	
433	0.03249605	0.00055337	3.06E-07	1.00101905	0.01576241	2238148.4	0.00050391	0.05039088	
434	0.03017417	0.0005004	2.51E-07	1.0010141	0.01576237	2238148.51	0.00050141	0.05014133	
435	1.01824781	0.02291221	0.00052496	1.00047889	0.01575372	2237945.83	0.00050566	0.0505657	

© 2024 Zhang X et al. *JAMA Pediatrics*.

436	-0.7062985	-0.0158042	0.00024984	1.00075429	0.01575831	2238124.45	0.00049483	0.04948317	
437	-0.9754968	-0.0219904	0.00048359	1.00054044	0.01575468	2236862.79	0.0005074	0.05074001	
438	-0.4669678	-0.0106323	0.00011309	1.00091235	0.01576069	2238100.51	0.00050465	0.05046532	
439	0.28568447	0.00621026	3.86E-05	1.00096179	0.01576161	2238146.83	0.00049682	0.04968242	
440	-1.2976944	-0.0290587	0.00084412	1.0001623	0.01574865	2237777.41	0.00050524	0.05052362	
441	-0.427688	-0.0096964	9.41E-05	1.00091911	0.01576089	2238134.38	0.00049862	0.04986158	
442	-0.3212884	-0.0073804	5.45E-05	1.00097096	0.01576162	2238120.71	0.00050488	0.05048752	
443	-1.4474766	-0.0323806	0.00104791	0.99995109	0.01574524	2237477.69	0.00050607	0.05060709	
444	-1.0456442	-0.0235339	0.00055382	1.00046694	0.01575351	2237473.4	0.00050688	0.05068846	
445	0.70771567	0.01586534	0.00025177	1.00076184	0.01575822	2229557.63	0.00050782	0.05078199	
446	-1.4762997	-0.0329928	0.00108787	0.99990787	0.01574456	2237641.73	0.00050541	0.05054087	
447	-1.0885115	-0.0243525	0.00059299	1.00041543	0.01575277	2238039.16	0.00050148	0.05014844	
448	0.63257386	0.01411115	0.00019919	1.00080986	0.01575904	2238098.99	0.00050476	0.05047641	
449	-0.2281046	-0.0052224	2.73E-05	1.00097043	0.01576183	2238145.73	0.00049154	0.04915418	
450	-0.1306107	-0.0030925	9.57E-06	1.00100341	0.01576223	2238145.92	0.00049959	0.04995892	
451	-1.2837891	-0.0287714	0.00082752	1.00018097	0.01574893	2237658.85	0.00050591	0.05059065	
452	0.75869914	0.01700436	0.00028921	1.00072057	0.01575758	2237987.67	0.00050641	0.05064059	
453	-1.172805	-0.0263286	0.00069306	1.00032125	0.01575118	2237677.92	0.00050614	0.05061416	
454	-1.0654556	-0.0235002	0.00055223	1.00042819	0.0157532	2238116.53	0.00048709	0.04870925	
455	-0.884538	-0.0197728	0.00039101	1.00061527	0.01575604	2238103.2	0.00049741	0.04974091	
456	-0.0199592	-0.0006256	3.92E-07	1.00102224	0.01576244	2238146.74	0.00050512	0.0505119	
457	-0.4665199	-0.0105962	0.00011233	1.00090815	0.01576066	2238121.51	0.00050221	0.0502205	
458	-0.0906082	-0.0022164	4.92E-06	1.00102195	0.01576241	2238135.2	0.0005067	0.05067043	
459	-0.059377	-0.0015115	2.29E-06	1.0010212	0.01576242	2238144.74	0.00050525	0.05052488	
460	-0.6918152	-0.015539	0.00024152	1.00076968	0.0157585	2238116.99	0.00049824	0.0498242	
461	-0.2154334	-0.0048676	2.37E-05	1.00094492	0.01576165	2238147.27	0.00047724	0.04772439	
462	-0.2912559	-0.0066905	4.48E-05	1.00097507	0.01576173	2238136.09	0.00050221	0.0502209	
463	-0.5719832	-0.012993	0.00016888	1.00085943	0.01575981	2238016.22	0.0005062	0.05061965	
464	0.75431715	0.01689051	0.00028535	1.00072283	0.01575763	2238048.54	0.00050556	0.05055623	
465	-0.2208674	-0.0051184	2.62E-05	1.00099291	0.01576202	2238140.51	0.00050213	0.05021331	
466	-1.2216779	-0.0273108	0.00074569	1.00025926	0.01575024	2237985.28	0.00050256	0.05025564	
467	-1.2930813	-0.0290241	0.00084211	1.0001692	0.01574872	2234448.77	0.00050758	0.0507583	
468	-1.5598569	-0.0348554	0.001214	0.99977718	0.01574245	2237297.64	0.00050623	0.05062334	
469	-1.0702855	-0.0240622	0.00057895	1.00043947	0.01575308	2237750.4	0.00050615	0.05061466	
470	-0.181518	-0.0042428	1.80E-05	1.00100238	0.01576216	2238141.44	0.00050296	0.05029571	
471	-1.4620259	-0.0326962	0.00106841	0.99992934	0.0157449	2237513.47	0.00050594	0.05059354	
472	-0.9105561	-0.0203853	0.0004156	1.00059402	0.01575567	2238089.58	0.00049937	0.04993723	
473	-1.5252916	-0.0339001	0.00114845	0.99983404	0.01574346	2237972.49	0.0005004	0.05003975	
474	-1.4419317	-0.0323058	0.00104309	0.9999592	0.01574535	2233989.56	0.00050756	0.0507559	
475	-0.9465011	-0.0213391	0.00045539	1.00056873	0.01575514	2237486.76	0.00050703	0.05070293	
476	0.04044393	0.00072585	5.27E-07	1.00099658	0.01576223	2238148.67	0.00049296	0.0492957	
477	-1.4484303	-0.0323942	0.0010488	0.99994969	0.01574522	2237554	0.00050584	0.05058411	

© 2024 Zhang X et al. *JAMA Pediatrics.*

478	0.35529192	0.00784708	6.16E-05	1.00095763	0.01576138	2238108.19	0.00050692	0.05069179	
479	-1.4499807	-0.0324378	0.00105161	0.99994735	0.01574518	2237449.28	0.00050614	0.05061373	
480	-0.2549292	-0.0058356	3.41E-05	1.00097072	0.01576178	2238144.25	0.00049492	0.04949152	
481	-0.9138138	-0.0203175	0.00041284	1.00058304	0.0157556	2238115.7	0.00049259	0.04925907	
482	-1.3668276	-0.0306101	0.00093656	1.00006785	0.01574711	2237494.65	0.00050621	0.05062112	
483	-0.5238474	-0.011883	0.00014126	1.00088111	0.01576021	2238108.61	0.00050318	0.05031777	
484	0.05396947	0.00103484	1.07E-06	1.0010154	0.01576237	2238148.64	0.00050266	0.05026584	
485	0.48719537	0.01075951	0.00011581	1.00088583	0.01576034	2238138.74	0.00049954	0.04995381	
486	-1.4630694	-0.0326229	0.00106364	0.99992822	0.01574493	2237900.76	0.00050295	0.05029511	
487	0.31652174	0.00697076	4.86E-05	1.00097188	0.0157616	2238109.22	0.00050715	0.05071454	
488	-0.4057249	-0.0091905	8.45E-05	1.00092461	0.01576101	2238137.91	0.00049664	0.04966404	
489	-1.2161623	-0.0268392	0.00072018	1.00025941	0.01575045	2238101.67	0.00048967	0.04896666	
490	3.28134047	0.0737242	0.00540993	0.99566291	0.01567683	2237946.96	0.00048221	0.0482208	*
491	0.00980608	4.34E-05	1.88E-09	1.0010247	0.01576246	2238146.85	0.00050637	0.0506371	
492	0.79524367	0.01767361	0.00031241	1.00067816	0.01575705	2238124.67	0.00049713	0.04971318	
493	0.48917545	0.01076845	0.000116	1.00087891	0.01576029	2238141.52	0.0004962	0.04962035	
494	1.55111574	0.03436804	0.00118035	0.99977684	0.0157428	2238103.15	0.00048381	0.04838132	
495	1.92224145	0.04264223	0.00181601	0.99914165	0.01573265	2238083.85	0.00048137	0.04813658	
496	0.63231903	0.0140191	0.00019659	1.00080006	0.01575898	2238132.21	0.00049856	0.04985626	
497	0.10669967	0.00222636	4.96E-06	1.00101793	0.01576236	2238148.55	0.00050629	0.05062919	
498	-0.4366149	-0.0099249	9.85E-05	1.00092077	0.01576087	2238126.61	0.00050164	0.0501641	
499	1.23602798	0.02661936	0.00070842	1.00020295	0.01575002	2238134.38	0.00046054	0.04605406	
500	0.28964535	0.0063127	3.99E-05	1.0009649	0.01576162	2238146.26	0.00049905	0.04990487	
501	0.06441765	0.00127494	1.63E-06	1.00102499	0.01576245	2238146.12	0.00050777	0.05077712	
502	-0.7789819	-0.0175636	0.00030854	1.00071306	0.0157575	2238051.13	0.00050397	0.05039749	
503	-1.5534844	-0.0346974	0.00120303	0.99978761	0.01574263	2237513.1	0.0005057	0.05057045	
504	-1.3819559	-0.0309349	0.00095652	1.00004643	0.01574677	2237576.58	0.00050594	0.05059385	
505	-0.9252486	-0.0202291	0.00040925	1.00055399	0.01575539	2238132.31	0.00047653	0.04765305	
506	-0.1156958	-0.0027399	7.51E-06	1.00099169	0.01576215	2238147.4	0.00049287	0.0492874	
507	-0.5337527	-0.0120433	0.00014509	1.00086723	0.01576007	2238128.62	0.00049824	0.0498242	
508	-0.9807642	-0.0217467	0.00047293	1.00051797	0.01575458	2238114.68	0.000491	0.04909965	
509	-0.6909561	-0.0154256	0.00023801	1.00076103	0.01575846	2238129.27	0.00049224	0.04922363	
510	-0.4020704	-0.009052	8.20E-05	1.00091465	0.01576095	2238141.87	0.00049049	0.04904886	
511	-1.4532003	-0.0325371	0.00105805	0.99994243	0.01574509	2236677.1	0.00050703	0.05070288	
512	-1.3144359	-0.0290563	0.00084397	1.00013645	0.01574843	2238084.06	0.00049253	0.04925328	
513	-1.2599212	-0.0280325	0.00078559	1.00020905	0.01574951	2238052.86	0.0004983	0.04982988	
514	-1.9687603	-0.0436901	0.00190601	0.99903992	0.01573066	2237645.92	0.0005036	0.05035979	
515	-1.4542742	-0.0324191	0.0010504	0.99994138	0.01574514	2237919.65	0.00050261	0.05026079	
516	-0.9137407	-0.0203749	0.00041517	1.00058647	0.01575561	2238108.06	0.00049545	0.04954519	
517	-0.0464386	-0.0012173	1.48E-06	1.00101657	0.01576239	2238147.08	0.00050266	0.05026551	
518	-0.526356	-0.0119408	0.00014264	1.00088006	0.01576019	2238106.83	0.00050334	0.05033394	
519	-0.5283652	-0.0120288	0.00014475	1.00088525	0.01576022	2237937.21	0.00050694	0.0506944	

© 2024 Zhang X et al. *JAMA Pediatrics*.

520	-1.0717459	-0.0241168	0.00058158	1.00043867	0.01575305	2237252.17	0.00050709	0.05070873	
521	-0.632607	-0.0141482	0.00020023	1.00079997	0.01575908	2238131.78	0.00049253	0.04925328	
522	-1.0960488	-0.0246358	0.00060686	1.00041091	0.01575262	2237691.14	0.0005063	0.05062997	
523	-1.0037934	-0.0226002	0.00051077	1.0005108	0.01575421	2237665.78	0.0005066	0.05066003	
524	-0.6353991	-0.0142789	0.00020395	1.00080592	0.0157591	2238123.86	0.00049731	0.04973137	
525	-1.1497319	-0.0258207	0.0006666	1.00034886	0.01575162	2237674.53	0.00050622	0.05062152	
526	-1.178825	-0.0264138	0.00069755	1.00031286	0.01575108	2237919.74	0.00050431	0.05043122	
527	-0.5073637	-0.0113888	0.00012975	1.00087046	0.01576022	2238137.41	0.00049224	0.04922363	
528	-0.9382496	-0.0211711	0.00044825	1.00057755	0.01575527	2232199.24	0.00050775	0.05077538	
529	-1.0636068	-0.0239446	0.00057331	1.00044792	0.01575319	2236547.31	0.00050743	0.05074258	
530	-0.8764555	-0.0196499	0.00038617	1.00062592	0.01575616	2238087.58	0.0005002	0.05001998	
531	-1.085715	-0.0244191	0.00059624	1.00042291	0.0157528	2237487.03	0.00050679	0.05067942	
532	-1.256737	-0.0281355	0.00079137	1.0002157	0.01574951	2237877.16	0.00050449	0.05044872	
533	-0.9809753	-0.0220266	0.00048518	1.00053084	0.01575459	2238016.57	0.00050349	0.05034925	
534	-1.0151648	-0.0227033	0.00051544	1.00049257	0.01575403	2238070.46	0.00050002	0.05000218	
535	-0.9865138	-0.0219566	0.0004821	1.00051626	0.01575449	2238104.28	0.00049479	0.04947895	
536	-1.0806231	-0.0237197	0.00056258	1.00040834	0.01575296	2238121.56	0.00048261	0.04826093	
537	0.58289002	0.01294314	0.00016758	1.00083613	0.01575951	2238128.48	0.00050158	0.05015783	
538	-1.2794457	-0.0286035	0.0008179	1.00018577	0.01574905	2237938.67	0.00050336	0.05033569	
539	-0.8918301	-0.019988	0.00039956	1.00061205	0.01575594	2238086.09	0.00050014	0.05001413	
540	-1.3507877	-0.030274	0.00091612	1.00009037	0.01574747	2237158.9	0.00050679	0.05067883	
541	-0.5199702	-0.0118299	0.00014	1.00088808	0.01576028	2238049.08	0.00050599	0.05059872	
542	-0.7926576	-0.017846	0.00031854	1.00070045	0.01575732	2238071.48	0.0005028	0.05028012	
543	0.73910033	0.01653261	0.00027339	1.00073367	0.01575782	2238075.8	0.00050485	0.05048499	
544	-1.3518942	-0.0302934	0.0009173	1.0000888	0.01574744	2237292.48	0.00050662	0.05066219	
545	-0.2669692	-0.0061476	3.78E-05	1.0009816	0.01576184	2238137.93	0.00050208	0.050208	
546	-0.7950822	-0.0179125	0.00032092	1.00069948	0.01575729	2238058.11	0.00050352	0.05035205	
547	-0.7264666	-0.0163896	0.00026868	1.00075264	0.01575814	2238072.95	0.00050345	0.05034498	
548	-0.8271763	-0.0186464	0.00034775	1.00067453	0.01575687	2238013.21	0.00050474	0.05047368	
549	-0.4654023	-0.010592	0.00011224	1.00091218	0.01576069	2238107.34	0.00050415	0.05041487	
550	-0.3659078	-0.0083497	6.97E-05	1.00094915	0.01576133	2238132.51	0.00050153	0.05015292	
551	1.52933738	0.03457073	0.00119429	0.99980268	0.0157429	2237896.25	0.00050364	0.05036422	
552	-0.8164746	-0.0184236	0.00033949	1.00068461	0.01575702	2237970.23	0.00050554	0.05055395	
553	-0.8832149	-0.0199031	0.00039618	1.00062636	0.01575609	2237949.97	0.00050546	0.05054563	
554	0.03714363	0.00065843	4.34E-07	1.00102149	0.01576243	2238148.31	0.00050522	0.05052209	
555	-0.1690227	-0.0038018	1.45E-05	1.00092762	0.01576158	2238148	0.00046437	0.04643683	
556	-0.6180259	-0.0139917	0.00019583	1.00082813	0.01575934	2238080.22	0.0005042	0.05041972	
557	-0.4085263	-0.0093368	8.72E-05	1.00094007	0.01576112	2238094.07	0.00050559	0.05055941	
558	-0.7389235	-0.016733	0.00028006	1.00074926	0.01575803	2237180.93	0.00050749	0.0507488	
559	-1.4400415	-0.0320448	0.00102631	0.99996259	0.01574551	2237985.11	0.00050065	0.050065	
560	-1.0039903	-0.0225223	0.00050726	1.00050692	0.01575421	2238026.82	0.00050292	0.05029213	
561	0.16318001	0.00350081	1.23E-05	1.00100984	0.01576222	2238146.46	0.00050642	0.05064224	

© 2024 Zhang X et al. *JAMA Pediatrics*.

562	-0.6753693	-0.0153184	0.00023472	1.00079562	0.01575877	2236934.77	0.0005076	0.0507602	
563	0.17138599	0.00366952	1.35E-05	1.00099891	0.01576212	2238148.09	0.00050167	0.05016742	
564	-1.9513255	-0.043295	0.00187176	0.99907558	0.01573124	2237694	0.00050323	0.05032285	
565	-0.8332911	-0.0187004	0.00034976	1.00066358	0.01575677	2238091.64	0.00050038	0.05003768	
566	-0.885036	-0.019957	0.00039833	1.00062555	0.01575606	2237868.94	0.00050614	0.05061409	
567	-1.4198722	-0.0316983	0.00100426	0.99999186	0.01574594	2237873.98	0.00050367	0.05036705	
568	-0.676444	-0.0152818	0.0002336	1.00078867	0.01575872	2238079.26	0.00050362	0.05036246	
569	-1.0673179	-0.0239932	0.00057563	1.00044261	0.01575313	2237782.82	0.00050601	0.05060059	
570	-1.0558663	-0.0237428	0.00056369	1.00045522	0.01575333	2237762.58	0.00050614	0.05061366	
571	-0.7780054	-0.0175856	0.00030932	1.00071736	0.01575753	2237872.09	0.00050648	0.05064807	
572	-0.7135299	-0.0161082	0.00025954	1.00076267	0.0157583	2238068.91	0.0005038	0.05038043	
573	-0.5137257	-0.0117052	0.00013707	1.00089358	0.01576035	2237847.15	0.00050724	0.05072422	
574	-0.9244338	-0.0208359	0.00043417	1.00058921	0.01575548	2237797.45	0.00050637	0.05063747	
575	-0.011345	-0.0004281	1.83E-07	1.00100913	0.01576234	2238148.24	0.00049859	0.04985946	
576	-1.0642684	-0.023614	0.00055759	1.00043451	0.01575321	2238104.06	0.0004929	0.04929018	
577	-0.6573508	-0.0148648	0.00022103	1.00080241	0.01575893	2238074.19	0.00050411	0.05041059	
578	0.06524591	0.00128926	1.66E-06	1.00101693	0.01576238	2238148.69	0.0005038	0.05038043	
579	-1.3173007	-0.0295185	0.00087101	1.00013623	0.01574821	2237543.96	0.0005062	0.05061965	
580	-0.5867369	-0.0131869	0.00017395	1.00083373	0.01575956	2238129.41	0.00049607	0.04960675	
581	-0.300008	-0.0067675	4.58E-05	1.00094017	0.01576144	2238145.42	0.00048548	0.04854802	
582	0.44176547	0.00978336	9.58E-05	1.00091668	0.01576076	2238129.54	0.00050444	0.05044433	
583	-0.8449402	-0.0190664	0.00036358	1.00066111	0.01575663	2237895.75	0.00050611	0.05061131	
584	0.13192563	0.00270703	7.33E-06	1.00094767	0.01576177	2238148.69	0.00047286	0.04728606	
585	-1.2582514	-0.0282284	0.00079661	1.00021464	0.01574946	2237396.38	0.00050663	0.05066286	
586	-0.8509412	-0.0191091	0.00036521	1.00064978	0.01575653	2238080.34	0.00050137	0.05013668	
587	-0.7737109	-0.0175023	0.0003064	1.00072178	0.01575759	2237582.99	0.00050718	0.05071834	
588	-0.8162436	-0.0182025	0.00033139	1.00066897	0.01575696	2238119.55	0.00049379	0.04937878	
589	0.29257495	0.00611018	3.73E-05	1.00088271	0.01576098	2238148.27	0.00045696	0.04569563	
590	-0.1192785	-0.0028514	8.13E-06	1.00101264	0.01576232	2238143.92	0.00050351	0.05035089	
591	-0.3338936	-0.0076554	5.86E-05	1.00096509	0.01576154	2238125.83	0.00050398	0.05039823	
592	-0.6300965	-0.0139387	0.00019434	1.00078428	0.015759	2238138.83	0.00048188	0.04818776	
593	-0.9569746	-0.0215542	0.00046461	1.00055758	0.01575497	2237810.16	0.00050622	0.05062198	
594	-0.7786293	-0.0175351	0.00030754	1.00071167	0.0157575	2238074.02	0.00050279	0.05027949	
595	0.65576562	0.01441618	0.00020788	1.00077018	0.01575865	2238139.62	0.0004895	0.04895012	
596	-0.1877314	-0.0043385	1.88E-05	1.00098233	0.01576199	2238146.16	0.00049344	0.0493436	
597	-0.1354352	-0.0031625	1.00E-05	1.00098055	0.01576204	2238147.49	0.00048849	0.0488488	
598	0.30576857	0.0065471	4.29E-05	1.00092267	0.01576125	2238147.82	0.0004797	0.04797015	
599	0.38088895	0.00795185	6.33E-05	1.00084202	0.01576043	2238147.92	0.00045043	0.04504309	
600	-1.5589509	-0.0348459	0.00121334	0.99977849	0.01574247	2237100.68	0.00050654	0.05065373	
601	-1.0806231	-0.0237197	0.00056258	1.00040834	0.01575296	2238121.56	0.00048261	0.04826093	
602	-0.4402821	-0.0100429	0.0001009	1.00092574	0.01576089	2238095.06	0.00050525	0.0505248	
603	-1.7147386	-0.0382708	0.00146318	0.99951572	0.01573825	2236653.97	0.00050675	0.05067458	

© 2024 Zhang X et al. *JAMA Pediatrics*.

604	1.90035199	0.04202858	0.00176421	0.99918741	0.01573343	2238091.2	0.00047868	0.04786832	
605	0.04981116	0.00093943	8.83E-07	1.00101018	0.01576233	2238148.66	0.00049995	0.04999451	
606	1.56951453	0.03468413	0.00120214	0.99974969	0.01574241	2238106.72	0.0004811	0.04811022	
607	-2.0438269	-0.0440979	0.00194178	0.99894594	0.01572959	2238075.64	0.00047653	0.04765305	
608	-0.5113942	-0.0116356	0.00013544	1.00089216	0.01576035	2238063.28	0.00050574	0.05057403	
609	-0.5858587	-0.01326	0.00017589	1.00084585	0.01575965	2238101.81	0.000503	0.05029982	
610	-0.912454	-0.0204928	0.00041999	1.00059605	0.01575565	2238053.04	0.00050258	0.0502584	
611	0.46676222	0.01036023	0.00010738	1.00090675	0.01576058	2238116.13	0.00050555	0.05055539	
612	-0.9104729	-0.0204814	0.00041953	1.00059977	0.01575568	2238012.08	0.00050417	0.05041739	
613	-0.9799253	-0.0216283	0.0004678	1.00051406	0.01575459	2238121.93	0.00048649	0.04864877	
614	0.03547124	0.00062192	3.87E-07	1.00102673	0.01576247	2238130.55	0.00050778	0.05077782	
615	0.12464582	0.00262023	6.87E-06	1.00100672	0.01576225	2238148.58	0.00050184	0.05018441	
616	0.38011472	0.00839076	7.04E-05	1.00094368	0.01576119	2238134.81	0.00050463	0.05046303	
617	0.58569856	0.01287829	0.0001659	1.00081786	0.01575938	2238140.79	0.00049164	0.04916401	
618	0.78009506	0.01732464	0.0003002	1.00068987	0.01575725	2238126.5	0.00049673	0.04967331	
619	2.24232779	0.05082525	0.00257785	0.99842928	0.01572094	2237874.6	0.00049923	0.04992274	
620	0.1571312	0.00334732	1.12E-05	1.00100018	0.01576215	2238148.32	0.00050105	0.05010502	
621	-0.9479724	-0.0213048	0.00045392	1.00056378	0.01575511	2238013.11	0.00050386	0.05038622	
622	0.88551616	0.01947003	0.00037912	1.00058545	0.01575576	2238134.43	0.00048491	0.04849074	
623	-0.7352079	-0.0166025	0.00027571	1.00074782	0.01575805	2238043.85	0.0005046	0.05045993	
624	0.1610545	0.003377	1.14E-05	1.00096413	0.01576186	2238148.6	0.0004833	0.0483301	
625	0.44810116	0.00983644	9.68E-05	1.00089693	0.01576059	2238143.37	0.00049518	0.04951793	
626	-0.2551455	-0.0056891	3.24E-05	1.0009224	0.0157614	2238147.21	0.00047025	0.04702496	
627	0.34394143	0.00758256	5.75E-05	1.0009599	0.01576143	2238131	0.0005059	0.05059	
628	-0.2023712	-0.0047165	2.23E-05	1.00100142	0.01576212	2238136.43	0.00050447	0.05044717	
629	-0.8746447	-0.01956	0.00038264	1.00062436	0.01575618	2238103.03	0.00049766	0.04976596	
630	0.28523121	0.00621092	3.86E-05	1.00096538	0.01576164	2238146.48	0.00049861	0.04986052	
631	0.4170031	0.0089941	8.09E-05	1.0008816	0.0157606	2238146.72	0.0004793	0.04793029	
632	-0.0307195	-0.00086	7.40E-07	1.00100753	0.01576232	2238148.07	0.00049794	0.04979351	
633	0.07738572	0.0015618	2.44E-06	1.00101543	0.01576236	2238148.72	0.00050355	0.05035505	
634	0.68746099	0.01519615	0.00023098	1.00075572	0.01575834	2238135.16	0.00049415	0.04941461	
635	0.08155904	0.00165159	2.73E-06	1.00100957	0.01576231	2238148.73	0.00050083	0.05008254	
636	-0.4214694	-0.0095715	9.17E-05	1.00092423	0.01576095	2238132.33	0.00049996	0.04999606	
637	0.49128428	0.01090891	0.00011905	1.00089323	0.01576038	2238120.09	0.00050489	0.05048926	
638	-0.2188245	-0.0050438	2.55E-05	1.00098242	0.01576194	2238144.62	0.00049659	0.04965935	
639	-1.171087	-0.0262767	0.00069034	1.00032299	0.01575122	2237791.85	0.0005056	0.05056048	
640	-0.2392387	-0.0053422	2.86E-05	1.00092502	0.01576145	2238147.37	0.00046972	0.0469716	
641	-1.1661075	-0.0261518	0.00068379	1.00032859	0.01575132	2237867.67	0.00050502	0.05050237	
642	-0.6641372	-0.0144187	0.00020795	1.00073631	0.0157585	2238142.16	0.00046506	0.04650607	
643	0.28119461	0.00615754	3.79E-05	1.00097867	0.01576175	2238142.09	0.00050485	0.05048488	
644	-0.1588794	-0.003755	1.41E-05	1.00101583	0.0157623	2237500.55	0.0005078	0.05077969	
645	-0.5160769	-0.0117083	0.00013714	1.00088496	0.01576027	2238110.81	0.00050303	0.05030329	

© 2024 Zhang X et al. *JAMA Pediatrics*.

646	-1.0201367	-0.0213357	0.00045521	1.00042687	0.01575398	2238140.05	0.00043738	0.04373792	
647	-0.7709339	-0.0172705	0.00029833	1.00071006	0.01575756	2238113.36	0.00049738	0.0497382	
648	-1.0946781	-0.0245883	0.00060453	1.00041188	0.01575264	2237835.55	0.00050559	0.05055935	
649	-0.2237667	-0.0051527	2.66E-05	1.000981	0.01576192	2238144.55	0.00049642	0.04964181	
650	-0.0270835	-0.0007674	5.89E-07	1.00098343	0.01576213	2238148.45	0.00048597	0.04859659	
651	-0.6432917	-0.0143546	0.00020612	1.00079008	0.01575895	2238133.23	0.0004906	0.04906	
652	0.85912916	0.01873821	0.00035116	1.00059927	0.0157561	2238138.57	0.00047759	0.04775885	
653	1.0447975	0.02312028	0.00053452	1.00043509	0.01575329	2238124.19	0.00048867	0.04886721	
654	-0.159793	-0.0037748	1.43E-05	1.00101535	0.01576229	2238009.13	0.00050763	0.05076274	
655	-0.2188245	-0.0050438	2.55E-05	1.00098242	0.01576194	2238144.62	0.00049659	0.04965935	
656	-0.0251927	-0.000716	5.13E-07	1.0009621	0.01576196	2238148.55	0.0004754	0.04753959	
657	0.41761863	0.00925266	8.56E-05	1.00093075	0.01576096	2238114.94	0.00050617	0.0506165	
658	-0.3100936	-0.007045	4.97E-05	1.00095195	0.01576151	2238143.4	0.00049315	0.0493147	
659	0.27940818	0.00591152	3.50E-05	1.00091279	0.01576124	2238148.19	0.0004706	0.04705971	
660	-0.1830209	-0.0042831	1.84E-05	1.00100512	0.01576218	2238138.04	0.00050447	0.05044717	
661	0.33617785	0.00740288	5.48E-05	1.00096162	0.01576147	2238135.8	0.00050533	0.05053336	
662	0.02111815	0.00029804	8.89E-08	1.00102404	0.01576246	2238147.54	0.00050618	0.05061813	
663	0.24668119	0.00533856	2.85E-05	1.00097326	0.01576179	2238147.45	0.00049713	0.04971318	
664	0.25675716	0.00558259	3.12E-05	1.00097722	0.0157618	2238146.63	0.00050053	0.0500526	
665	1.1949443	0.02656226	0.0007054	1.00026718	0.01575056	2238111.35	0.00049103	0.04910317	
666	0.42998534	0.00951561	9.06E-05	1.00092183	0.01576085	2238131.47	0.00050431	0.05043075	
667	-1.3050319	-0.0288891	0.0008343	1.00014915	0.01574862	2238079.1	0.00049381	0.04938123	
668	-1.0403422	-0.023244	0.00054026	1.00046576	0.01575361	2238072.48	0.00049944	0.04994429	
669	1.26726351	0.02806787	0.00078757	1.00017559	0.01574916	2238115.16	0.0004866	0.04866002	
670	-1.0961405	-0.0246147	0.00060583	1.00041004	0.01575262	2237865.45	0.00050535	0.05053499	
671	-0.6777877	-0.0153282	0.00023502	1.00078943	0.01575872	2238054.9	0.00050471	0.05047078	
672	-0.4267253	-0.0097613	9.53E-05	1.00093569	0.01576102	2237838.79	0.00050742	0.05074162	
673	-0.0188041	-0.0005917	3.50E-07	1.0010013	0.01576227	2238148.33	0.00049475	0.04947471	
674	-0.5825867	-0.0132307	0.00017511	1.00085332	0.01575971	2238004.04	0.00050629	0.05062859	
675	-0.9685192	-0.0217973	0.00047514	1.00054553	0.01575479	2237895.65	0.00050562	0.05056233	
676	-0.1590833	-0.0037406	1.40E-05	1.00100602	0.01576222	2238142.7	0.0005029	0.05029001	
677	-0.6295175	-0.014173	0.00020094	1.00081238	0.01575918	2238119.62	0.00049899	0.04989908	
678	-0.1225919	-0.0029366	8.63E-06	1.00101945	0.01576237	2238119.83	0.0005071	0.05070962	
679	-1.0242125	-0.0229941	0.00052872	1.00048704	0.01575387	2237985.37	0.00050403	0.05040316	
680	-0.4080241	-0.0092276	8.52E-05	1.00092097	0.01576098	2238139.08	0.00049518	0.04951793	
681	-0.5729472	-0.0130353	0.00016998	1.00086158	0.01575982	2232464.38	0.0005078	0.05078044	
682	-0.4895844	-0.011152	0.00012442	1.00090379	0.01576053	2238057.13	0.00050602	0.05060225	
683	-0.5027774	-0.0113783	0.00012952	1.00088659	0.01576035	2238126.32	0.00050007	0.0500067	
684	-0.3294355	-0.0075659	5.73E-05	1.00096911	0.01576159	2238114.62	0.00050531	0.05053052	
685	-0.2936315	-0.0067782	4.60E-05	1.00098419	0.0157618	2238020.41	0.00050728	0.05072798	
686	0.57742451	0.01279093	0.00016366	1.00083564	0.01575954	2238134.16	0.00049932	0.04993185	
687	-0.9638628	-0.0216385	0.00046825	1.00054735	0.01575486	2238032.88	0.00050304	0.05030386	

© 2024 Zhang X et al. *JAMA Pediatrics.*

688	-1.1215899	-0.0251872	0.00063431	1.00038138	0.01575215	2237795.56	0.00050576	0.05057561	
689	-0.9965635	-0.0224123	0.00050231	1.00051696	0.01575433	2237911.39	0.00050535	0.05053484	
690	-0.242246	-0.0056049	3.14E-05	1.00099106	0.01576197	2238135.74	0.0005037	0.05036984	
691	-0.5046213	-0.0114194	0.00013045	1.00088568	0.01576033	2238126.14	0.00050008	0.0500082	
692	0.04639254	0.00086729	7.53E-07	1.00102327	0.01576244	2238148.26	0.00050634	0.05063363	
693	0.62269543	0.01391285	0.00019363	1.00081942	0.01575916	2238023.43	0.00050666	0.0506665	
694	-0.6720882	-0.0152211	0.00023175	1.00079539	0.01575879	2237991.31	0.000506	0.05060017	
695	-1.1670051	-0.0260723	0.00067965	1.00032507	0.01575132	2238029.41	0.0005012	0.05011959	
696	-1.3370498	-0.0296756	0.0008803	1.00010732	0.0157479	2238056.03	0.00049681	0.04968091	
697	-1.0727337	-0.0241328	0.00058235	1.00043737	0.01575303	2237472.4	0.00050684	0.05068396	
698	-0.7353572	-0.0166177	0.00027622	1.00074876	0.01575805	2238014.06	0.00050532	0.05053155	
699	0.16646319	0.00357073	1.28E-05	1.00100677	0.01576219	2238147.41	0.00050518	0.05051776	
700	-0.3676891	-0.0084126	7.08E-05	1.00095366	0.01576136	2238119.83	0.00050427	0.05042683	
701	-0.6164887	-0.0139396	0.00019438	1.000827	0.01575935	2238098.25	0.00050292	0.05029183	
702	-0.7531609	-0.0167555	0.00028081	1.00071329	0.01575773	2238128.67	0.00049018	0.04901844	
703	-0.2470068	-0.0056178	3.16E-05	1.00095865	0.0157617	2238145.99	0.00048776	0.04877646	
704	0.2678601	0.00581563	3.38E-05	1.0009685	0.0157617	2238147.03	0.00049762	0.04976209	
705	0.42785237	0.00935789	8.76E-05	1.00090124	0.0157607	2238144.77	0.00049253	0.04925328	
706	-1.0027659	-0.0225174	0.00050704	1.00050916	0.01575423	2237996.46	0.00050391	0.05039107	
707	-1.6659309	-0.036954	0.00136437	0.99960609	0.01573981	2237950.96	0.0005	0.04999989	
708	-0.2929679	-0.0067203	4.52E-05	1.0009722	0.01576171	2238138.26	0.00050099	0.05009866	
709	0.01479741	0.00015577	2.43E-08	1.00102127	0.01576243	2238147.96	0.00050473	0.05047272	
710	-0.343518	-0.0078477	6.16E-05	1.00095638	0.01576145	2238134.95	0.00050114	0.0501135	
711	-1.4013594	-0.0313112	0.00097991	1.00001861	0.01574635	2237833.49	0.0005043	0.05042951	
712	-0.5940002	-0.0133877	0.00017929	1.0008344	0.01575953	2238122.27	0.00049905	0.04990487	
713	-0.3594259	-0.0080327	6.46E-05	1.0009127	0.01576107	2238145.05	0.00048101	0.04810132	
714	0.12944544	0.00273234	7.47E-06	1.00100947	0.01576226	2238148.48	0.00050355	0.05035482	
715	-0.4278127	-0.0097376	9.49E-05	1.00092621	0.01576094	2238123.84	0.00050251	0.05025145	
716	-1.509378	-0.0335198	0.00112285	0.99985888	0.01574387	2237996.09	0.00049942	0.04994166	
717	-0.6157459	-0.0138633	0.00019225	1.00082045	0.01575931	2238121.81	0.00049864	0.04986368	
718	-0.8924045	-0.0197109	0.00038856	1.0005941	0.01575589	2238126.88	0.00048579	0.04857863	
719	0.81119461	0.0181592	0.00032981	1.00067435	0.01575688	2238077.09	0.00050409	0.05040884	
720	-1.8958043	-0.0421063	0.00177058	0.99918402	0.01573297	2237669.39	0.0005037	0.05037005	
721	-0.6228492	-0.0141446	0.00020014	1.00083037	0.01575933	2237511.4	0.00050745	0.05074453	
722	0.67917384	0.01456415	0.00021216	1.00071659	0.01575818	2238144.47	0.00046506	0.04650607	
723	0.41691816	0.00923716	8.54E-05	1.00093114	0.01576096	2238114.23	0.00050621	0.05062066	
724	-0.3225379	-0.0074169	5.50E-05	1.00097275	0.01576164	2238102.86	0.00050602	0.05060179	
725	-0.9343287	-0.0210503	0.00044315	1.00057949	0.01575532	2237840.93	0.00050613	0.05061344	
726	-0.5203791	-0.0116465	0.00013569	1.00085961	0.01576008	2238138.5	0.00048983	0.04898292	
727	-0.4828137	-0.0108133	0.00011697	1.00087694	0.01576037	2238140.08	0.00048912	0.04891176	
728	-1.4514722	-0.0323145	0.00104364	0.9999457	0.01574523	2237967.21	0.00050126	0.0501261	
729	-1.4675769	-0.0328693	0.00107974	0.99992071	0.01574473	2234381.33	0.00050752	0.05075189	

© 2024 Zhang X et al. *JAMA Pediatrics*.

730	-0.3987262	-0.0090701	8.23E-05	1.00093443	0.01576111	2238132.89	0.0005004	0.05004044	
731	-0.8961706	-0.0202343	0.00040947	1.00061719	0.0157559	2235535.71	0.00050766	0.05076564	
732	-0.869172	-0.0196306	0.00038541	1.0006415	0.0157563	2236918.58	0.00050747	0.05074683	
733	-1.3537892	-0.0298225	0.00088903	1.00008342	0.01574763	2238091.43	0.00048958	0.04895843	
734	-0.6949533	-0.0157272	0.00024741	1.0007791	0.01575853	2237996.53	0.00050582	0.05058215	
735	-1.1489813	-0.0258094	0.00066602	1.00034989	0.01575164	2237605.25	0.00050642	0.05064244	
736	-0.4497107	-0.0101721	0.00010351	1.00090715	0.01576072	2238134.98	0.00049737	0.04973684	
737	-1.1902982	-0.0266768	0.0007115	1.00029915	0.01575085	2237887.35	0.00050469	0.05046931	
738	-0.7305334	-0.0164717	0.00027139	1.00074889	0.01575809	2238079.87	0.00050296	0.050296	
739	-0.4685503	-0.0106457	0.00011338	1.00090792	0.01576065	2238119.18	0.00050261	0.05026113	
740	-0.9410193	-0.0209052	0.00043706	1.00055742	0.01575519	2238114.66	0.00049224	0.04922363	
741	-1.5972339	-0.0350598	0.00122827	0.99972638	0.01574192	2238073.58	0.00048881	0.0488808	
742	1.17129357	0.02625911	0.0006894	1.00030079	0.01575096	2238074.49	0.00049975	0.04997467	
743	-0.4390963	-0.0099186	9.84E-05	1.00090857	0.01576077	2238137.47	0.00049557	0.04955651	
744	-0.3656631	-0.0083305	6.94E-05	1.00094619	0.01576131	2238135.84	0.00049991	0.0499914	
745	-1.0246063	-0.0230849	0.0005329	1.00049007	0.01575387	2235182.49	0.00050763	0.05076328	
746	-0.8852481	-0.0199869	0.00039953	1.00062692	0.01575606	2237026.48	0.00050742	0.05074183	
747	-0.9907343	-0.0223271	0.00049851	1.00052495	0.01575443	2236984.23	0.00050734	0.05073398	
748	-1.2634465	-0.0283545	0.00080373	1.00020806	0.01574935	2237004.08	0.00050704	0.05070385	
749	0.23133501	0.0050118	2.51E-05	1.00098392	0.0157619	2238147.19	0.0005006	0.05005983	
750	-0.4156618	-0.0094929	9.02E-05	1.00093643	0.01576106	2238100.11	0.00050524	0.05052413	
751	1.75385906	0.03933326	0.00154548	0.99943334	0.01573714	2238050.24	0.00049346	0.04934617	
752	-0.8164466	-0.018371	0.00033755	1.00068081	0.015757	2238068.86	0.0005027	0.05026982	
753	-0.3246392	-0.007447	5.55E-05	1.00096774	0.01576159	2238128.01	0.00050376	0.05037634	
754	0.43089403	0.00949516	9.02E-05	1.00091339	0.01576078	2238140.98	0.00049991	0.0499914	
755	0.63839641	0.01423552	0.00020271	1.0008051	0.01575897	2238105.7	0.00050421	0.0504207	
756	0.68783156	0.01529589	0.00023403	1.00076465	0.01575839	2238124.61	0.00050014	0.05001413	
757	0.3304664	0.00724611	5.25E-05	1.00095616	0.01576144	2238143.9	0.00050141	0.05014133	
758	-0.59341	-0.0134822	0.00018183	1.00084806	0.01575962	2237858.37	0.00050704	0.05070415	
759	-0.339289	-0.0077697	6.04E-05	1.00096176	0.0157615	2238129.22	0.0005032	0.05031965	
760	0.30886976	0.00669923	4.49E-05	1.00094579	0.01576142	2238147.06	0.00049224	0.04922363	
761	-0.460641	-0.0104475	0.00010919	1.00090785	0.01576068	2238128.25	0.00050051	0.05005126	
762	-0.4904966	-0.0108746	0.0001183	1.00085651	0.01576019	2238142.83	0.0004797	0.04797015	
763	0.3146658	0.00683833	4.68E-05	1.00094671	0.01576142	2238146.79	0.00049369	0.04936891	
764	-1.2057105	-0.0269278	0.00072494	1.0002784	0.01575057	2238018.46	0.00050137	0.0501372	
765	-0.9938085	-0.0218369	0.00047686	1.00049633	0.01575437	2238125.69	0.00048237	0.04823701	
766	-0.146181	-0.0034498	1.19E-05	1.00100684	0.01576224	2238143.87	0.00050235	0.05023473	
767	-1.8804743	-0.0394263	0.00155283	0.99930061	0.01573573	2238116.04	0.00044831	0.04483104	
768	1.49653362	0.03175622	0.00100794	0.99987032	0.01574493	2238132.6	0.00044457	0.04445711	
769	-0.3159668	-0.0072282	5.23E-05	1.000964	0.01576159	2238137.92	0.00050036	0.0500363	
770	-1.101605	-0.0245402	0.00060216	1.00039759	0.01575255	2238080.58	0.0004974	0.04973956	
771	-0.8387517	-0.0186439	0.00034765	1.00064672	0.01575665	2238123.11	0.00049103	0.04910317	

© 2024 Zhang X et al. *JAMA Pediatrics*.

772	-0.1515242	-0.0035706	1.28E-05	1.00100677	0.01576223	2238143.29	0.0005027	0.05026982	
773	-1.4308311	-0.0318876	0.00101628	0.99997596	0.01574571	2237947.94	0.00050205	0.05020511	
774	-0.4914783	-0.0111442	0.00012424	1.00089491	0.01576046	2238122.05	0.00050156	0.05015587	
775	0.20799169	0.00447985	2.01E-05	1.00098585	0.01576196	2238147.85	0.00049882	0.04988223	
776	-0.8792048	-0.0197751	0.0003911	1.00062752	0.01575614	2238041.1	0.00050347	0.05034737	
777	-0.70207	-0.0158662	0.0002518	1.00077214	0.01575844	2238052.28	0.0005046	0.05045954	
778	0.16564131	0.00352592	1.24E-05	1.00099134	0.01576207	2238148.4	0.00049735	0.04973548	
779	1.54389873	0.03499846	0.00122399	0.99977842	0.01574247	2237446.35	0.0005063	0.05063029	
780	-0.3254879	-0.0073869	5.46E-05	1.00094753	0.01576143	2238142.89	0.00049336	0.04933583	
781	-0.5325677	-0.012069	0.00014572	1.00087528	0.01576013	2238112.6	0.00050251	0.05025145	
782	-0.6269628	-0.0141861	0.00020131	1.00082196	0.01575925	2238083.87	0.00050389	0.05038934	
783	-0.5359093	-0.011898	0.00014161	1.000839	0.01575987	2238141.06	0.00048261	0.04826093	
784	-0.8151272	-0.0183028	0.00033505	1.00067904	0.01575701	2238092.4	0.00050057	0.05005722	
785	-0.8458137	-0.0186314	0.00034718	1.00062956	0.01575651	2238131.59	0.00048237	0.04823701	
786	1.3155445	0.02956049	0.00087349	1.0001165	0.01574799	2238048.27	0.00050017	0.05001707	
787	-0.2819949	-0.0065201	4.25E-05	1.00098842	0.01576186	2237615.17	0.00050772	0.05077157	
788	0.5783322	0.01270785	0.00016154	1.00082151	0.01575945	2238141.22	0.00049121	0.04912052	
789	1.61230367	0.03589234	0.00128724	0.99967821	0.01574116	2238089.72	0.00048771	0.04877145	
790	-0.557224	-0.0126836	0.00016093	1.00087063	0.01575997	2235323.7	0.00050777	0.05077691	
791	-0.1169513	-0.0027984	7.84E-06	1.00101237	0.01576232	2238144.3	0.00050324	0.05032443	
792	-0.51145	-0.0116507	0.00013579	1.00089422	0.01576036	2237951.89	0.00050693	0.05069296	
793	1.34618182	0.02899363	0.00084033	1.00006802	0.01574787	2238131.92	0.0004594	0.04594033	
794	1.77703895	0.03838277	0.00147185	0.9994351	0.01573774	2238119.46	0.0004579	0.04579024	
795	-0.9666491	-0.0217239	0.00047195	1.00054579	0.01575482	2237997.59	0.00050414	0.05041402	
796	1.31855318	0.02786023	0.00077596	1.00009882	0.01574863	2238136.77	0.00044252	0.04425197	
797	-0.3297043	-0.0073187	5.36E-05	1.00090667	0.01576111	2238146.29	0.00047271	0.04727081	
798	2.79601596	0.05938653	0.00351737	0.99738779	0.01570525	2238097.03	0.00043548	0.04354811	
799	0.40198234	0.00888535	7.90E-05	1.00093471	0.01576105	2238132.59	0.00050465	0.05046481	
800	0.75677437	0.01682498	0.00028314	1.00071052	0.01575755	2238124.54	0.00049831	0.04983101	
801	1.33081427	0.02992108	0.00089491	1.00009581	0.01574765	2238040.18	0.00050057	0.05005656	
802	1.3814718	0.03088426	0.00095339	1.00002473	0.01574661	2238085.28	0.00049435	0.04943525	
803	-1.4092851	-0.0314933	0.00099133	1.0000072	0.01574617	2237805.29	0.00050455	0.05045516	
804	0.14174655	0.00300063	9.01E-06	1.00100167	0.01576219	2238148.49	0.00050056	0.0500559	
805	1.75712292	0.03920635	0.00153555	0.99943322	0.01573722	2238075.22	0.00048849	0.0488488	
806	-0.3969129	-0.0090012	8.11E-05	1.00092934	0.01576108	2238137.66	0.00049729	0.04972861	
807	-0.5370657	-0.012154	0.00014777	1.00087072	0.01576007	2238118.94	0.00050129	0.05012878	
808	-0.0059163	-0.0003015	9.10E-08	1.00098684	0.01576216	2238148.53	0.00048756	0.04875625	
809	0.10947327	0.00229082	5.25E-06	1.00101944	0.01576237	2238148.19	0.00050721	0.05072141	
810	-0.1243202	-0.0029291	8.58E-06	1.00098943	0.01576212	2238147.34	0.00049224	0.04922363	
811	-0.5125017	-0.0116381	0.0001355	1.00088822	0.01576031	2238103.81	0.00050383	0.05038305	
812	-0.8622215	-0.019409	0.00037676	1.00064327	0.01575638	2238031.11	0.00050398	0.05039842	
813	-0.8598174	-0.0193786	0.00037558	1.0006469	0.01575642	2237979.55	0.00050517	0.05051732	

© 2024 Zhang X et al. *JAMA Pediatrics*.

814	0.44171543	0.0098089	9.63E-05	1.00092179	0.0157608	2238040.89	0.00050724	0.05072389	
815	-0.2265742	-0.0052551	2.76E-05	1.00099501	0.01576203	2238136.53	0.00050385	0.05038464	
816	-0.4893724	-0.0111541	0.00012446	1.00090498	0.01576054	2238011.15	0.00050663	0.05066315	
817	-1.0003151	-0.0225423	0.00050816	1.00051525	0.01575427	2236584.53	0.00050746	0.05074617	
818	0.18232236	0.00386532	1.49E-05	1.00097047	0.01576188	2238148.47	0.00048839	0.04883945	
819	-0.8867468	-0.0200284	0.00040119	1.00062606	0.01575604	2185978.05	0.00050783	0.05078338	
820	-0.8649125	-0.019543	0.00038198	1.00064576	0.01575636	2186138.03	0.00050783	0.05078341	
821	-0.6269805	-0.0141909	0.00020144	1.00082245	0.01575925	2238078.43	0.0005042	0.05041988	
822	0.74989462	0.01630071	0.00026577	1.0006843	0.01575748	2238141.5	0.00047641	0.04764084	
823	-0.2422296	-0.005578	3.11E-05	1.00098199	0.01576189	2238142.6	0.00049908	0.04990774	
824	0.4250554	0.00929565	8.64E-05	1.00090251	0.01576072	2238144.83	0.00049256	0.04925618	
825	-0.4996569	-0.0113211	0.00012822	1.00089005	0.01576039	2238123.04	0.00050113	0.05011294	
826	-0.025849	-0.0007544	5.69E-07	1.00101427	0.01576238	2238147.82	0.00050122	0.05012177	
827	-0.6175099	-0.0138848	0.00019285	1.00081732	0.01575928	2238124.89	0.0004974	0.04973956	
828	-1.3344087	-0.0295508	0.00087292	1.0001104	0.01574798	2238072.07	0.00049455	0.04945527	
829	1.53822537	0.03489954	0.00121709	0.99978714	0.01574259	2236422.05	0.00050722	0.05072214	
830	-0.415574	-0.00944	8.92E-05	1.00092662	0.01576099	2238132.81	0.00049991	0.0499914	
831	-0.7729231	-0.0174051	0.000303	1.0007159	0.01575757	2238077.88	0.00050259	0.05025909	
832	-0.4749525	-0.01083	0.00011734	1.00091177	0.01576065	2238034.19	0.00050646	0.05064602	
833	0.40689944	0.0089659	8.04E-05	1.00092616	0.01576097	2238140.76	0.00050116	0.05011573	
834	-0.2311079	-0.0053632	2.88E-05	1.00099637	0.01576203	2238130.67	0.00050506	0.05050607	
835	0.44914553	0.00986598	9.74E-05	1.00089758	0.01576059	2238143.06	0.0004958	0.04958036	
836	-0.4318086	-0.0098486	9.70E-05	1.00092855	0.01576095	2238105.35	0.00050474	0.05047356	
837	-1.4913514	-0.0331147	0.00109591	0.99988642	0.01574432	2238005.45	0.00049907	0.04990679	
838	0.76005009	0.01656243	0.00027437	1.00067984	0.01575738	2238140.73	0.00047858	0.04785773	
839	0.80133618	0.0178171	0.0003175	1.00067362	0.01575697	2238123.48	0.00049746	0.04974629	
840	-0.8613666	-0.019148	0.00036669	1.00062818	0.01575634	2238121.05	0.00049151	0.04915087	
841	0.0513273	0.00097852	9.58E-07	1.00102314	0.01576244	2238148.36	0.00050641	0.05064146	
842	5.25626537	0.12167956	0.01460944	0.98713812	0.01553985	2237068.74	0.00049543	0.04954328	*
843	-0.553459	-0.012514	0.00015666	1.00086113	0.01575993	2238118.8	0.00050097	0.05009691	
844	0.39670677	0.00878838	7.73E-05	1.00094179	0.01576112	2238062.09	0.00050727	0.05072682	
845	-0.1409823	-0.0033197	1.10E-05	1.00099967	0.01576219	2238146.03	0.0004984	0.04983995	
846	-0.523138	-0.0118772	0.00014112	1.00088296	0.01576023	2238099.89	0.00050402	0.0504019	
847	0.09628372	0.00198502	3.94E-06	1.00101182	0.01576232	2238148.71	0.00050267	0.05026684	
848	-0.3169121	-0.0072973	5.33E-05	1.00097622	0.01576168	2238064.28	0.00050688	0.05068786	
849	0.03523744	0.00061605	3.80E-07	1.00102394	0.01576245	2238147.91	0.00050639	0.05063896	
850	0.89864602	0.01964257	0.00038586	1.0005667	0.01575555	2238136.97	0.000479	0.04789964	
851	-0.9953035	-0.022261	0.00049556	1.0005126	0.01575435	2238075.39	0.0004998	0.04997952	
852	-0.4663213	-0.0106424	0.00011331	1.00091681	0.01576072	2237976.04	0.00050695	0.05069538	
853	-1.1143843	-0.0249183	0.00062085	1.00038628	0.0157523	2238036.98	0.00050133	0.05013302	
854	-0.6170589	-0.0139715	0.00019527	1.0008289	0.01575935	2238078.52	0.0005043	0.05042951	
855	1.94744909	0.04408211	0.00194042	0.99905763	0.015731	2237903.15	0.00050067	0.05006693	

© 2024 Zhang X et al. *JAMA Pediatrics*.

856	0.57449722	0.01270789	0.00016154	1.00083512	0.01575956	2238136.29	0.00049797	0.04979715	
857	-1.272394	-0.0283406	0.00080294	1.00019353	0.01574924	2238036.48	0.00049954	0.04995381	
858	-0.2964354	-0.006695	4.48E-05	1.00094282	0.01576147	2238145.35	0.00048632	0.04863165	
859	-0.0745698	-0.0018511	3.43E-06	1.00101854	0.0157624	2238145.06	0.0005044	0.05043953	
860	-0.1166046	-0.0027912	7.79E-06	1.00101274	0.01576232	2238144.16	0.00050341	0.05034063	
861	-0.4998158	-0.0113154	0.00012809	1.00088853	0.01576038	2238125.82	0.00050031	0.0500314	
862	-0.4281063	-0.0097286	9.47E-05	1.00092319	0.01576092	2238129.51	0.00050094	0.05009397	
863	0.72977176	0.01633326	0.00026684	1.00074199	0.01575794	2238052.31	0.00050564	0.05056433	
864	0.48726502	0.01067096	0.00011391	1.00087082	0.01576024	2238143.75	0.0004911	0.04911015	
865	0.50830097	0.01076201	0.00011586	1.00080193	0.01575967	2238146.86	0.0004579	0.04579024	
866	-0.8867468	-0.0200284	0.00040119	1.00062606	0.01575604	2185978.05	0.00050783	0.05078338	
867	-0.8648996	-0.0195427	0.00038197	1.00064577	0.01575636	2193017.8	0.00050783	0.05078328	
868	-0.8867603	-0.0200287	0.0004012	1.00062605	0.01575604	2180229.85	0.00050783	0.05078347	
869	-0.2769293	-0.0063379	4.02E-05	1.00096935	0.01576172	2238142.57	0.00049717	0.04971743	
870	1.51547387	0.03393509	0.00115083	0.99982758	0.01574345	2238071.01	0.00049444	0.04944422	
871	-1.1222879	-0.0252506	0.00063751	1.00038201	0.01575213	2233417.53	0.00050769	0.05076869	
872	1.53816925	0.03485994	0.00121433	0.99978767	0.01574262	2237526.42	0.00050612	0.05061183	
873	1.18672337	0.02597618	0.00067463	1.00026861	0.01575082	2238129.07	0.00047629	0.04762853	
874	-0.0112072	-0.0004228	1.79E-07	1.0010017	0.01576228	2238148.38	0.00049492	0.04949152	
875	1.31136034	0.0296299	0.00087759	1.00012358	0.01574802	2237782.36	0.00050575	0.0505755	
876	0.09395161	0.00193595	3.75E-06	1.00101588	0.01576235	2238148.71	0.00050457	0.05045676	
877	0.82581544	0.01798293	0.00032343	1.00062572	0.01575654	2238139.7	0.00047665	0.04766517	
878	1.10447996	0.02440142	0.00059536	1.00036867	0.01575226	2238124.14	0.00048626	0.04862589	
879	-1.438062	-0.032199	0.0010362	0.999965	0.01574545	2236936.33	0.00050687	0.05068741	
880	-1.0665676	-0.0239904	0.0005755	1.00044394	0.01575314	2237612.61	0.00050659	0.05065906	
881	-0.7437061	-0.0168204	0.00028299	1.00074394	0.01575796	2237916.88	0.00050634	0.05063444	
882	-0.3491032	-0.0079555	6.33E-05	1.00095055	0.01576139	2238137.9	0.00049909	0.0499087	
883	-0.0085291	-0.0003697	1.37E-07	1.00102593	0.01576247	2238144.14	0.0005069	0.05069043	
884	-0.2066267	-0.0048189	2.32E-05	1.00100339	0.01576213	2238126.66	0.00050591	0.0505906	
885	-1.2333601	-0.027227	0.00074113	1.0002387	0.01575011	2238098.99	0.00049018	0.04901844	
886	-0.311768	-0.0070558	4.98E-05	1.00094458	0.01576145	2238144.41	0.00048958	0.04895843	
887	-0.2514292	-0.0057863	3.35E-05	1.00098089	0.01576187	2238141.79	0.00049966	0.04996646	
888	-0.3139485	-0.0072305	5.23E-05	1.00097707	0.01576169	2238069.76	0.00050683	0.05068269	
889	-0.1567928	-0.0036994	1.37E-05	1.00101165	0.01576227	2238136.01	0.00050554	0.05055395	
890	-0.3188274	-0.0073443	5.40E-05	1.00097666	0.01576168	2237949.1	0.00050743	0.0507431	
891	-0.1786685	-0.0041106	1.69E-05	1.00097163	0.01576192	2238147.05	0.00048725	0.04872516	
892	-0.4349982	-0.0099409	9.89E-05	1.00093104	0.01576095	2238009.73	0.00050686	0.05068606	
893	0.01557649	0.00017325	3.00E-08	1.00098026	0.0157621	2238148.63	0.00048444	0.04844412	
894	-1.1148918	-0.0249181	0.00062084	1.00038536	0.01575229	2238044.21	0.00050087	0.0500874	
895	-0.4166233	-0.0092942	8.64E-05	1.00089393	0.01576075	2238143.77	0.00048245	0.04824503	
896	-1.1720914	-0.0262247	0.00068761	1.00031999	0.01575121	2237991.69	0.00050275	0.05027502	
897	-0.3047824	-0.0070253	4.94E-05	1.00097999	0.01576174	2238071.25	0.00050685	0.05068546	

© 2024 Zhang X et al. *JAMA Pediatrics*.

898	0.81406936	0.0176232	0.00031062	1.00062801	0.01575666	2238141.26	0.00047127	0.04712732	
899	-0.8060707	-0.0182323	0.00033248	1.00069626	0.01575717	2234314.97	0.00050774	0.05077381	
900	-1.2838322	-0.0287731	0.00082762	1.00018092	0.01574893	2237652.34	0.00050593	0.05059318	
901	-0.236527	-0.0054997	3.03E-05	1.00100043	0.01576205	2237392.43	0.00050777	0.05077741	
902	-1.1451875	-0.0257111	0.00066096	1.00035397	0.01575171	2237763.34	0.00050585	0.05058549	
903	1.74572146	0.03938888	0.00154984	0.9994411	0.01573717	2237980.8	0.0004995	0.04994951	
904	-0.9181373	-0.0207258	0.0004296	1.00059686	0.01575558	2212315.21	0.00050782	0.05078232	
905	0.3805645	0.00816476	6.67E-05	1.00089026	0.01576079	2238147.29	0.00047616	0.04761612	
906	-0.7184181	-0.0162505	0.00026415	1.00076224	0.01575826	2237980.74	0.0005059	0.05058995	
907	1.31584023	0.02969686	0.00088156	1.00011717	0.01574793	2237915.34	0.00050454	0.05045408	
908	-0.0818084	-0.0019754	3.90E-06	1.00098283	0.0157621	2238148.08	0.00048693	0.0486931	
909	1.00063431	0.02255411	0.00050868	1.00049939	0.01575401	2235895	0.00050765	0.05076535	
910	-0.2211486	-0.0049732	2.47E-05	1.00093685	0.01576158	2238147.37	0.00047375	0.04737486	
911	0.58288419	0.01216379	0.000148	1.00073754	0.01575888	2238146.75	0.00044252	0.04425197	
912	1.41755778	0.03207189	0.00102805	0.99997296	0.0157456	2237707.35	0.0005058	0.05057974	
913	-0.2452135	-0.005676	3.22E-05	1.00099195	0.01576197	2238132.21	0.00050452	0.05045231	
914	-0.0703171	-0.0017516	3.07E-06	1.00101456	0.01576237	2238146.58	0.00050228	0.0502279	
915	0.71838856	0.01589254	0.00025263	1.0007336	0.01575799	2238133.66	0.00049422	0.04942156	
916	-0.3321196	-0.0076334	5.83E-05	1.00097007	0.01576159	2238092.69	0.00050628	0.05062775	
917	-0.4703292	-0.0107348	0.00011528	1.00091536	0.0157607	2237899.86	0.00050722	0.05072169	
918	0.05632425	0.00109111	1.19E-06	1.00102283	0.01576243	2238148.47	0.00050642	0.05064174	
919	-0.0763531	-0.0018906	3.58E-06	1.00101787	0.01576239	2238145.25	0.00050412	0.05041231	
920	2.0243844	0.04578913	0.0020933	0.99890422	0.01572856	2237927.98	0.00049919	0.04991904	
921	0.74305437	0.01658563	0.00027515	1.00072735	0.01575775	2238106.56	0.0005026	0.05026011	
922	1.35026248	0.03058095	0.00093478	1.00007004	0.01574713	2233942.3	0.00050765	0.05076494	
923	-1.0966888	-0.0246398	0.00060706	1.00040985	0.01575261	2237788.05	0.00050588	0.0505883	
924	0.38461237	0.00840818	7.07E-05	1.00092293	0.01576102	2238145.26	0.00049449	0.04944866	
925	1.48072084	0.0324761	0.0010541	0.99988491	0.01574468	2238118.58	0.00047486	0.04748648	
926	-0.1469805	-0.0031931	1.02E-05	1.00086025	0.01576107	2238148.4	0.00042912	0.04291192	
927	-0.6354616	-0.0142428	0.00020292	1.00080171	0.01575907	2238128.77	0.00049473	0.04947258	
928	0.88726773	0.01982505	0.00039307	1.00060309	0.01575579	2238102.35	0.00050076	0.05007638	
929	0.48421686	0.01077811	0.00011621	1.00090181	0.01576047	2237640.4	0.00050768	0.05076826	
930	0.98120341	0.02036271	0.00041464	1.00044019	0.0157543	2238143.35	0.00043063	0.04306277	
931	-1.1739498	-0.0263395	0.00069364	1.00031953	0.01575116	2237793.01	0.00050559	0.05055872	
932	0.63820826	0.01411019	0.00019916	1.00079146	0.01575889	2238135.92	0.0004956	0.04956024	
933	-1.1233719	-0.0248442	0.00061716	1.00036803	0.01575218	2238106.13	0.00049053	0.04905259	
934	-0.9846555	-0.0218794	0.00047872	1.00051639	0.01575452	2238109.4	0.00049315	0.0493147	
935	-0.5487596	-0.0124412	0.00015484	1.00086808	0.01576	2238102.1	0.00050349	0.05034925	
936	0.04995305	0.00094664	8.97E-07	1.0010208	0.01576242	2238148.51	0.00050521	0.05052132	
937	-0.0283394	-0.0008112	6.58E-07	1.00101635	0.01576239	2238147.59	0.00050227	0.05022713	
938	-0.8757431	-0.0196381	0.0003857	1.0006268	0.01575618	2238086.05	0.0005004	0.05003975	
939	-0.9114763	-0.0204162	0.00041686	1.00059378	0.01575565	2238085.6	0.00049989	0.04998905	

© 2024 Zhang X et al. *JAMA Pediatrics*.

940	-0.8961774	-0.0201039	0.00040421	1.00060931	0.01575588	2238076.16	0.00050114	0.0501135	
941	-0.9099707	-0.0203961	0.00041604	1.00059593	0.01575568	2238080.25	0.00050053	0.05005326	
942	-1.4326	-0.0318928	0.00101661	0.99997344	0.01574568	2237978.59	0.00050099	0.05009925	
943	-1.0561214	-0.0236154	0.00055766	1.00044991	0.01575334	2238059.28	0.00050048	0.05004792	
944	1.68549027	0.03768056	0.00141851	0.99955446	0.01573912	2238071.03	0.00049107	0.04910667	
945	0.29897805	0.00652006	4.25E-05	1.00096137	0.01576157	2238146.13	0.00049871	0.04987099	
946	-0.2233867	-0.0051389	2.64E-05	1.0009791	0.01576191	2238144.9	0.00049541	0.04954136	
947	-0.7274688	-0.0159974	0.00025598	1.00071477	0.01575794	2238137.42	0.00047847	0.04784707	
948	-0.2773308	-0.0063904	4.09E-05	1.00098224	0.01576182	2238132.15	0.00050386	0.05038602	
949	0.20623454	0.00445608	1.99E-05	1.00099346	0.01576202	2238147.3	0.00050248	0.05024754	
950	0.19724616	0.00425266	1.81E-05	1.0009948	0.01576205	2238147.56	0.00050217	0.05021733	
951	-0.1607862	-0.0037753	1.43E-05	1.00100406	0.0157622	2238143.51	0.00050205	0.05020511	
952	-0.1997758	-0.0046477	2.16E-05	1.00099753	0.01576209	2238141.46	0.00050225	0.05022481	
953	-0.273128	-0.0062878	3.96E-05	1.00098077	0.01576182	2238136.64	0.00050251	0.05025074	
954	-0.0246176	-0.0007208	5.20E-07	1.00100082	0.01576227	2238148.29	0.00049455	0.04945527	
955	0.26496363	0.00577387	3.34E-05	1.00097736	0.01576178	2238145.97	0.00050175	0.05017534	
956	-1.0539386	-0.0237361	0.00056338	1.00045869	0.01575336	2234265.09	0.00050767	0.05076738	
957	0.18275514	0.00388818	1.51E-05	1.00097734	0.01576194	2238148.41	0.0004919	0.04918966	
958	-0.7670635	-0.0173574	0.00030135	1.00072729	0.01575768	2237391.5	0.00050736	0.05073578	
959	-0.5937585	-0.0134759	0.00018166	1.0008461	0.0157596	2238022.88	0.00050599	0.05059923	
960	-0.0818084	-0.0019754	3.90E-06	1.00098283	0.0157621	2238148.08	0.00048693	0.0486931	
961	0.92793116	0.02075352	0.00043074	1.00056524	0.01575518	2238095.66	0.00050102	0.05010157	
962	0.21391652	0.00461952	2.14E-05	1.00098757	0.01576196	2238147.56	0.00050036	0.0500363	
963	-0.6027332	-0.0136107	0.00018531	1.00083271	0.01575947	2238112.68	0.00050122	0.05012177	
964	-0.473062	-0.0107862	0.00011639	1.00091242	0.01576066	2238045.79	0.00050631	0.05063149	
965	-1.3087923	-0.0292477	0.00085512	1.00014691	0.01574843	2237931.37	0.00050332	0.05033191	
966	-0.2390707	-0.005551	3.08E-05	1.00099779	0.01576203	2238100.89	0.00050674	0.05067381	
967	-0.5907334	-0.0134265	0.00018033	1.0008502	0.01575965	2237682.67	0.00050735	0.05073475	
968	-0.6591726	-0.0148699	0.00022118	1.00079743	0.01575889	2238102.96	0.00050173	0.05017303	
969	-1.2154076	-0.0273083	0.00074556	1.00026969	0.01575033	2234735.53	0.00050759	0.0507592	
970	-0.2546067	-0.0058803	3.46E-05	1.00098764	0.01576191	2238135.32	0.00050352	0.05035205	
971	-0.0305226	-0.000848	7.19E-07	1.00099257	0.0157622	2238148.36	0.00049053	0.04905259	
972	-0.7585159	-0.0171395	0.00029383	1.00073166	0.01575777	2237977.73	0.00050574	0.05057381	
973	-0.6157946	-0.013963	0.00019503	1.000832	0.01575938	2238032.69	0.0005057	0.05057034	
974	-1.3473627	-0.0301872	0.00091088	1.00009506	0.01574755	2237424.64	0.00050641	0.05064082	
975	-1.026528	-0.0230592	0.00053172	1.00048519	0.01575383	2237953.55	0.00050464	0.05046393	
976	-1.5929766	-0.0355442	0.00126239	0.99972405	0.01574162	2237620.83	0.00050514	0.05051427	
977	0.02666036	0.00042179	1.78E-07	1.00101535	0.01576238	2238148.45	0.00050197	0.05019666	
978	-0.3292416	-0.0075076	5.64E-05	1.00095568	0.01576149	2238139.74	0.00049825	0.04982534	
979	1.32014478	0.02975379	0.00088494	1.00011096	0.01574786	2237983.89	0.00050313	0.05031341	
980	-0.4662569	-0.0106104	0.00011263	1.00091167	0.01576069	2238107.86	0.00050409	0.05040902	
981	-0.1017744	-0.0024559	6.03E-06	1.00101203	0.01576233	2238145.6	0.00050228	0.05022828	

© 2024 Zhang X et al. *JAMA Pediatrics.*

982	0.99715269	0.02224964	0.00049504	1.00049295	0.01575407	2238107.48	0.00049756	0.04975556	
983	0.16079755	0.00340255	1.16E-05	1.00098303	0.01576201	2238148.52	0.00049276	0.0492762	
984	-0.2079146	-0.0048041	2.31E-05	1.00098574	0.01576199	2238144.73	0.00049712	0.04971175	
985	-0.166354	-0.003889	1.51E-05	1.00099786	0.01576214	2238144.99	0.00049939	0.04993901	
986	-0.6081066	-0.0135943	0.00018486	1.00081331	0.01575931	2238133.97	0.00049144	0.04914422	
987	-0.4795519	-0.010839	0.00011753	1.00089423	0.01576051	2238132.56	0.00049791	0.04979107	
988	0.80238622	0.01797287	0.00032308	1.00068282	0.015757	2238060.2	0.00050488	0.05048785	
989	-0.4420417	-0.0100881	0.00010181	1.00092601	0.01576089	2238079.04	0.00050583	0.05058306	
990	-1.7400503	-0.0387401	0.00149923	0.99947305	0.0157376	2237643.17	0.00050451	0.0504508	
991	-0.3742106	-0.0083812	7.03E-05	1.000913	0.01576103	2238144.31	0.00048396	0.04839557	
992	-0.3105212	-0.0070602	4.99E-05	1.00095333	0.01576152	2238143.09	0.00049393	0.04939334	
993	-0.7878149	-0.0177302	0.00031442	1.00070368	0.01575738	2238078.98	0.00050232	0.05023209	
994	-0.2738756	-0.0062379	3.89E-05	1.00096051	0.01576166	2238144.61	0.00049221	0.0492206	
995	-0.1649104	-0.0038705	1.50E-05	1.0010049	0.0157622	2238142.53	0.0005028	0.05028012	
996	1.17153951	0.02627035	0.00068999	1.00030063	0.01575095	2238072.44	0.00049996	0.04999606	
997	-1.0686544	-0.0240623	0.00057895	1.00044259	0.0157531	2234147.34	0.00050767	0.05076745	
998	1.82434129	0.04107839	0.00168542	0.9993013	0.01573497	2238010.89	0.00049669	0.04966869	
999	1.74232071	0.03952322	0.0015604	0.99944135	0.01573708	2237683.24	0.00050485	0.05048454	
1000	3.26691712	0.07417297	0.00547518	0.99562069	0.01567599	2237821.16	0.00049221	0.0492206	*
1001	0.7557326	0.01693595	0.00028689	1.00072285	0.01575762	2237994.46	0.00050636	0.05063558	
1002	1.76185213	0.03994931	0.00159418	0.99940666	0.01573654	2237754.45	0.00050422	0.05042201	
1003	0.46572636	0.01032462	0.00010664	1.0009051	0.01576058	2238127.47	0.00050435	0.05043504	
1004	1.43786279	0.0319073	0.00101754	0.99994477	0.01574546	2238105.05	0.00048649	0.04864877	
1005	1.94759132	0.04384659	0.0019198	0.99906727	0.01573124	2238007.77	0.00049534	0.04953365	
1006	1.77281126	0.03989727	0.00159005	0.99939566	0.01573648	2238018.91	0.00049669	0.04966869	
1007	-1.0454205	-0.0235199	0.00055316	1.00046683	0.01575351	2237669.08	0.00050649	0.05064942	
1008	-1.1677284	-0.0261348	0.00068291	1.00032535	0.0157513	2237985.09	0.00050299	0.05029894	
1009	-0.0637582	-0.001612	2.60E-06	1.00102329	0.01576244	2238141.06	0.00050641	0.05064064	
1010	-1.7965123	-0.0400026	0.00159837	0.99936973	0.01573593	2237457.73	0.00050525	0.05052496	
1011	-1.5994257	-0.0357058	0.00127388	0.99971315	0.01574143	2237472.34	0.00050572	0.05057192	
1012	-1.1181264	-0.0239773	0.00057485	1.0003517	0.01575239	2238133.15	0.00046109	0.04610894	
1013	-0.3157822	-0.0071648	5.14E-05	1.00094867	0.01576147	2238143.53	0.00049236	0.04923562	
1014	1.02236392	0.02257463	0.0005096	1.00045675	0.01575366	2238127.32	0.00048688	0.04868766	
1015	-0.2974126	-0.0068452	4.69E-05	1.00097807	0.01576174	2238125.31	0.0005047	0.05046992	
1016	-0.2576997	-0.0059611	3.56E-05	1.00099061	0.01576193	2238124.06	0.00050545	0.0505447	
1017	-0.157044	-0.0035889	1.29E-05	1.00095193	0.01576179	2238147.83	0.00047566	0.04756551	
1018	-0.6702342	-0.0150854	0.00022764	1.00078689	0.01575875	2238113.04	0.00049977	0.04997711	
1019	-0.1553625	-0.0036735	1.35E-05	1.00101514	0.0157623	2238105.03	0.00050718	0.05071795	
1020	-1.0203358	-0.0226553	0.00051326	1.0004804	0.01575395	2238107.26	0.00049296	0.0492957	
1021	-1.318998	-0.0295322	0.00087182	1.00013372	0.01574818	2237743.08	0.00050538	0.05053827	
1022	0.61185485	0.01366726	0.00018685	1.00082656	0.01575928	2238021.24	0.00050673	0.05067313	
1023	0.18432814	0.00393644	1.55E-05	1.00098394	0.01576199	2238148.31	0.00049538	0.04953752	

© 2024 Zhang X et al. *JAMA Pediatrics*.

1024	0.18432814	0.00393644	1.55E-05	1.00098394	0.01576199	2238148.31	0.00049538	0.04953752	
1025	-0.1036797	-0.0024857	6.18E-06	1.00100213	0.01576224	2238147.03	0.00049745	0.04974495	
1026	0.25354634	0.00553696	3.07E-05	1.00098782	0.01576189	2238142.44	0.00050551	0.05055054	
1027	-0.7289349	-0.0164834	0.00027177	1.00075434	0.01575813	2237982.13	0.00050583	0.05058316	
1028	-1.8473597	-0.0411407	0.00169044	0.99927362	0.01573438	2237161.14	0.00050593	0.05059291	
1029	-1.6046784	-0.035822	0.00128217	0.99970448	0.01574129	2237458.78	0.00050575	0.05057479	
1030	-0.0341786	-0.000946	8.95E-07	1.00102349	0.01576245	2238145.15	0.00050588	0.05058777	
1031	-1.144572	-0.0256958	0.00066017	1.00035465	0.01575172	2237776.36	0.00050579	0.05057875	
1032	1.27712216	0.02868341	0.00082246	1.00016773	0.01574881	2238054.43	0.00050017	0.05001707	
1033	-1.1215124	-0.0251819	0.00063405	1.00038136	0.01575215	2237818.38	0.00050561	0.05056122	
1034	-1.2020304	-0.0269758	0.00072753	1.0002856	0.01575061	2237635.89	0.00050621	0.05062076	
1035	-0.5812876	-0.0131781	0.00017372	1.00085108	0.01575971	2238081.75	0.00050449	0.05044941	
1036	-1.6451061	-0.0366586	0.00134267	0.99963795	0.01574025	2237712.25	0.00050437	0.05043715	
1037	-0.4243859	-0.0096969	9.41E-05	1.00093444	0.01576102	2238069.97	0.00050618	0.05061799	
1038	0.29218503	0.00637071	4.06E-05	1.00096444	0.01576161	2238146.15	0.00049921	0.04992089	
1039	0.79265347	0.01774018	0.00031477	1.00069004	0.01575713	2238077.81	0.00050424	0.05042412	
1040	0.01038595	5.69E-05	3.24E-09	1.00101574	0.01576239	2238148.27	0.00050195	0.05019451	
1041	0.95026006	0.02099545	0.00044083	1.00053	0.01575481	2238128.86	0.00048858	0.04885805	
1042	-0.2769549	-0.0062848	3.95E-05	1.00095342	0.0157616	2238145.26	0.00048899	0.04889861	
1043	0.45421191	0.0100971	0.00010199	1.00091674	0.01576071	2237606.37	0.00050771	0.05077143	
1044	0.73900648	0.01656346	0.00027441	1.0007367	0.01575783	2237923	0.00050688	0.05068766	
1045	-0.2746152	-0.0062788	3.94E-05	1.00096766	0.01576171	2238143.26	0.00049598	0.04959808	
1046	-0.1804245	-0.0042297	1.79E-05	1.0010078	0.0157622	2238133.05	0.00050559	0.05055872	
1047	0.38757599	0.00857547	7.36E-05	1.00094425	0.01576117	2238112.23	0.00050656	0.05065566	
1048	1.59826984	0.03608906	0.00130136	0.99969235	0.01574116	2237956.53	0.00050179	0.05017855	
1049	0.18858547	0.00407852	1.66E-05	1.00100717	0.01576216	2238131.8	0.00050751	0.05075122	
1050	-0.1954184	-0.0045237	2.05E-05	1.00098713	0.01576202	2238145.24	0.00049658	0.04965778	
1051	0.598694	0.01307771	0.00017108	1.00079869	0.01575918	2238142.84	0.00048478	0.04847761	
1052	0.27296394	0.00591872	3.50E-05	1.00096328	0.01576165	2238147.22	0.00049568	0.04956764	
1053	0.41000309	0.00891352	7.95E-05	1.00089955	0.01576076	2238146.05	0.00048746	0.04874599	
1054	0.03137163	0.00052739	2.78E-07	1.00101492	0.01576238	2238148.5	0.00050184	0.05018441	
1055	0.50251883	0.01116851	0.00012478	1.00088819	0.01576029	2238112.83	0.00050536	0.05053584	
1056	-0.0627618	-0.0015736	2.48E-06	1.00100489	0.01576229	2238147.71	0.00049726	0.04972584	
1057	0.07877469	0.00159729	2.55E-06	1.00102143	0.01576241	2238148.72	0.00050659	0.05065899	
1058	-1.173602	-0.026345	0.00069393	1.00032026	0.01575117	2237690.16	0.00050609	0.0506094	
1059	1.04247885	0.02345715	0.00055021	1.00045252	0.0157533	2237967.32	0.00050527	0.05052695	
1060	0.71007004	0.0157668	0.00024865	1.00074551	0.01575812	2238128.22	0.0004981	0.04981027	
1061	-0.0896088	-0.0021791	4.75E-06	1.00100932	0.01576231	2238146.7	0.00050039	0.05003906	
1062	0.29136776	0.00635855	4.05E-05	1.00096662	0.01576163	2238145.84	0.00050021	0.0500207	
1063	0.71106337	0.01580008	0.0002497	1.00074581	0.01575811	2238126.59	0.00049879	0.04987919	
1064	-0.8777926	-0.0197523	0.0003902	1.00062932	0.01575616	2238029.76	0.0005039	0.05039011	
1065	-0.5536397	-0.0124862	0.00015596	1.0008567	0.01575989	2238126.84	0.00049844	0.04984436	

© 2024 Zhang X et al. *JAMA Pediatrics*.

1066	-0.265556	-0.0059163	3.50E-05	1.00092055	0.01576137	2238147.1	0.0004706	0.04705971	
1067	0.09457613	0.00194857	3.80E-06	1.00101418	0.01576234	2238148.71	0.00050376	0.05037551	
1068	0.8762929	0.01970321	0.00038826	1.00062114	0.01575597	2237554.55	0.00050731	0.05073061	
1069	0.804621	0.01806767	0.0003265	1.00068429	0.01575699	2237612.5	0.00050735	0.05073507	
1070	0.63993359	0.01428603	0.00020415	1.00080585	0.01575897	2238086.37	0.00050532	0.05053211	
1071	0.96598133	0.02173709	0.00047251	1.00053388	0.01575459	2237844.62	0.00050654	0.05065432	
1072	1.12990046	0.02541776	0.00064596	1.00035262	0.01575172	2238013.5	0.00050373	0.05037322	
1073	1.20304477	0.02711699	0.00073515	1.00026469	0.0157503	2237944.63	0.00050473	0.0504726	
1074	-1.8247842	-0.0406238	0.00164831	0.99931704	0.01573508	2237411.39	0.00050534	0.05053391	
1075	-0.5213247	-0.0114398	0.00013091	1.0008264	0.01575985	2238143.72	0.00047111	0.04711064	
1076	1.3494897	0.02953668	0.00087208	1.00006607	0.0157476	2238124.37	0.00047432	0.0474316	
1077	1.32751327	0.02961416	0.00087666	1.00009869	0.01574782	2238096.18	0.00049287	0.0492874	
1078	-0.4440296	-0.0098498	9.71E-05	1.00087504	0.01576051	2238143.98	0.00047836	0.04783632	
1079	1.63863788	0.03704782	0.00137133	0.99962447	0.01574006	2237913.76	0.00050262	0.05026215	
1080	0.98120341	0.02036271	0.00041464	1.00044019	0.0157543	2238143.35	0.00043063	0.04306277	
1081	0.67598956	0.01479491	0.00021895	1.000749	0.01575839	2238141	0.00048464	0.04846433	
1082	2.42063941	0.05505573	0.00302351	0.9979982	0.01571399	2237744.92	0.00050099	0.05009925	
1083	2.7512213	0.05822955	0.00338209	0.99751349	0.01570731	2238100.5	0.00043279	0.04327852	
1084	2.03774526	0.04628044	0.00213835	0.99886777	0.01572791	2237740.31	0.0005031	0.05031036	
1085	0.93573705	0.02095856	0.00043929	1.00055921	0.01575506	2238081.64	0.00050234	0.05023435	
1086	1.75813367	0.03983273	0.0015849	0.9994143	0.01573667	2237824.82	0.00050345	0.05034522	
1087	-0.544881	-0.0122911	0.00015113	1.00086139	0.01575997	2238127.64	0.00049836	0.0498355	
1088	1.68571091	0.03700239	0.001368	0.99956964	0.01573964	2238110.58	0.0004736	0.04736037	
1089	0.33698283	0.00683138	4.67E-05	1.00081181	0.01576033	2238148.3	0.00042674	0.04267424	
1090	-0.5843526	-0.013228	0.00017504	1.00084693	0.01575966	2238100.95	0.00050311	0.05031064	
1091	0.47610776	0.0104686	0.00010963	1.00088432	0.01576038	2238142.31	0.00049558	0.04955838	
1092	1.11296985	0.02503518	0.00062668	1.00037244	0.01575204	2238012.16	0.0005039	0.0503903	
1093	1.17495948	0.02635007	0.00069418	1.00029654	0.01575088	2238071.29	0.00050003	0.05000293	
1094	0.60322773	0.01329742	0.00017688	1.00081053	0.01575923	2238139.03	0.00049364	0.04936392	
1095	1.56037127	0.03519307	0.00123762	0.99975434	0.01574217	2237985.51	0.00050106	0.05010616	
1096	1.62334755	0.03434752	0.00117897	0.99969219	0.01574214	2238130.63	0.00044089	0.04408916	
1097	1.87102217	0.0421341	0.00177302	0.99921406	0.01573357	2238007.84	0.00049634	0.04963365	
1098	2.98143177	0.06545186	0.00426904	0.99671767	0.01569406	2238053.42	0.00046316	0.04631631	
1099	-0.0449713	-0.0011589	1.34E-06	1.00097887	0.01576209	2238148.39	0.00048396	0.04839557	
1100	0.24349802	0.00518337	2.69E-05	1.00094233	0.01576155	2238148.23	0.00048092	0.04809235	
1101	1.38762842	0.03110681	0.00096717	1.00001619	0.01574643	2238069.26	0.00049697	0.04969728	
1102	0.73187612	0.01620661	0.00026271	1.00072457	0.01575783	2238132.22	0.00049487	0.04948736	
1103	2.11831948	0.04792461	0.00229265	0.99870714	0.01572541	2237923.33	0.00049853	0.04985305	
1104	0.44582759	0.00917106	8.41E-05	1.00078725	0.01575981	2238147.79	0.00043416	0.04341578	
1105	-1.2836926	-0.0287628	0.00082703	1.00018101	0.01574894	2237710.49	0.00050568	0.05056787	
1106	0.55191139	0.0122824	0.00015091	1.00086	0.01575984	2238111.46	0.00050485	0.05048521	
1107	0.30441515	0.00648148	4.20E-05	1.00091272	0.01576118	2238147.97	0.00047432	0.0474316	

© 2024 Zhang X et al. *JAMA Pediatrics*.

1108	-0.5154002	-0.0117267	0.00013757	1.00089031	0.01576031	2238055.77	0.00050588	0.05058839	
1109	1.18217161	0.02626133	0.00068952	1.00028225	0.01575081	2238113.2	0.00049056	0.04905631	
1110	-0.8399355	-0.0188922	0.00035697	1.00066106	0.01575669	2238063.56	0.00050276	0.0502763	
1111	-0.8413136	-0.0189921	0.00036076	1.00066469	0.01575669	2237837.67	0.00050645	0.0506447	
1112	0.8555241	0.01921076	0.0003691	1.00063876	0.01575627	2237946.1	0.00050635	0.05063497	
1113	-0.8750856	-0.0196835	0.00038749	1.00063118	0.0157562	2238042.59	0.00050345	0.05034498	
1114	-0.71884	-0.0162779	0.00026504	1.0007636	0.01575826	2237753.66	0.00050702	0.05070155	
1115	0.71316384	0.01596195	0.00025485	1.00075514	0.01575814	2238035.85	0.00050606	0.05060595	
1116	-0.6804105	-0.0154336	0.00023826	1.00079239	0.01575871	2233270.38	0.00050778	0.05077822	
1117	-0.6029196	-0.01367	0.00018693	1.00083927	0.01575951	2238056.44	0.00050525	0.05052513	
1118	-1.2890993	-0.0288654	0.00083293	1.00017367	0.01574883	2237800.88	0.00050509	0.05050949	
1119	-0.1536772	-0.0036356	1.32E-05	1.00101539	0.0157623	2238105.99	0.00050717	0.05071741	
1120	-0.1278874	-0.0030206	9.13E-06	1.00099679	0.01576218	2238146.81	0.00049612	0.04961189	
1121	0.68514225	0.01534356	0.00023549	1.00077761	0.01575848	2237787.79	0.00050733	0.05073339	
1122	-1.6854688	-0.037617	0.00141369	0.99956727	0.01573908	2237040.03	0.00050641	0.05064115	
1123	0.19099195	0.00411027	1.69E-05	1.00099521	0.01576206	2238147.76	0.00050173	0.05017257	
1124	-0.1516187	-0.0035838	1.29E-05	1.00101268	0.01576228	2238135.86	0.00050566	0.05056623	
1125	-0.9587918	-0.0214974	0.00046216	1.00055085	0.01575494	2238059.26	0.00050168	0.05016836	
1126	-0.5836875	-0.0132554	0.00017577	1.00085268	0.0157597	2238002.24	0.0005063	0.05063	
1127	0.51066213	0.01136702	0.00012926	1.00088606	0.01576023	2238073.36	0.00050661	0.05066138	
1128	-1.2964562	-0.0290837	0.00084557	1.00016453	0.01574865	2236912.9	0.00050706	0.05070609	
1129	0.49134151	0.01092483	0.0001194	1.00089563	0.01576039	2238095.22	0.00050626	0.05062643	
1130	-1.1460436	-0.0256705	0.00065887	1.00035135	0.01575171	2237971.57	0.00050351	0.05035136	
1131	-1.2473828	-0.0279081	0.00077864	1.00022737	0.01574971	2237931.03	0.00050372	0.05037154	
1132	-1.1345299	-0.0252877	0.00063938	1.00036112	0.01575194	2238068.98	0.00049841	0.04984106	
1133	-1.793668	-0.039974	0.00159608	0.99937391	0.01573598	2237121.61	0.0005061	0.05061034	
1134	-1.5929573	-0.0350998	0.00123107	0.99973109	0.01574193	2238055.74	0.00049253	0.04925328	
1135	-0.1963971	-0.0045342	2.06E-05	1.00098226	0.01576198	2238145.83	0.00049422	0.04942156	
1136	0.36394941	0.00798763	6.38E-05	1.00094093	0.01576122	2238143.74	0.0004998	0.04998033	
1137	-0.8458137	-0.0186314	0.00034718	1.00062956	0.01575651	2238131.59	0.00048237	0.04823701	
1138	-0.7712308	-0.0164072	0.00026924	1.00064078	0.01575723	2238142.52	0.00044875	0.04487469	
1139	-0.5253909	-0.0117259	0.00013755	1.00085262	0.01576001	2238139.61	0.0004873	0.04873041	
1140	-0.4042133	-0.0092507	8.56E-05	1.00094391	0.01576116	2238042.9	0.0005067	0.05067014	
1141	-1.4374743	-0.0318724	0.00101531	0.99996662	0.01574564	2238040.57	0.000497	0.04970021	
1142	-1.5782298	-0.0353033	0.00124536	0.99974685	0.01574194	2233767.28	0.00050752	0.05075232	
1143	-0.9371832	-0.0210312	0.00044234	1.00057229	0.01575527	2238055.12	0.0005022	0.05021971	
1144	-1.3714623	-0.0306847	0.00094113	1.00006122	0.01574702	2237725.8	0.0005053	0.05053036	
1145	-0.5110524	-0.0116434	0.00013562	1.00089466	0.01576037	2237918.67	0.00050706	0.05070625	
1146	-1.6322741	-0.0360885	0.0013013	0.999665	0.01574081	2238019.09	0.00049634	0.04963365	
1147	0.07605311	0.00151331	2.29E-06	1.00098807	0.01576214	2238148.73	0.00048991	0.04899093	
1148	-0.2232601	-0.0051801	2.68E-05	1.00099544	0.01576204	2238137.24	0.00050369	0.05036877	
1149	-1.6412956	-0.0366253	0.00134024	0.99964337	0.01574031	2237422.95	0.00050576	0.05057641	

© 2024 Zhang X et al. *JAMA Pediatrics*.

1150	-0.8018167	-0.0181381	0.00032906	1.00069982	0.01575723	2232712.91	0.00050777	0.05077697	
1151	-0.7166156	-0.0159253	0.00025368	1.0007372	0.01575814	2238132.12	0.00048835	0.04883475	
1152	0.07376258	0.00147653	2.18E-06	1.00100978	0.01576232	2238148.72	0.00050059	0.05005918	
1153	-1.5536217	-0.0347023	0.00120337	0.99978737	0.01574262	2237496.46	0.00050576	0.05057588	
1154	-1.6040486	-0.0354445	0.00125534	0.9997116	0.01574157	2238032.1	0.00049547	0.04954709	
1155	-1.6239043	-0.0362863	0.00131558	0.99967178	0.01574075	2236492.02	0.00050695	0.05069504	
1156	-1.0357971	-0.0231265	0.00053482	1.00046984	0.01575368	2238079.33	0.00049869	0.04986891	
1157	-0.161623	-0.0037996	1.44E-05	1.00100672	0.01576222	2238141.79	0.00050345	0.05034522	
1158	-0.81286	-0.01833	0.00033605	1.00068664	0.01575706	2238014.4	0.00050481	0.0504807	
1159	0.42754483	0.00945868	8.95E-05	1.00092259	0.01576086	2238132.57	0.00050412	0.05041197	
1160	-1.566875	-0.0350594	0.00122823	0.9997652	0.01574223	2229244.32	0.00050769	0.05076877	
1161	-1.1160163	-0.0250942	0.00062964	1.00038871	0.01575225	2237306.78	0.00050698	0.05069755	
1162	-1.1404024	-0.0255961	0.00065507	1.00035933	0.0157518	2237825.69	0.00050549	0.05054897	
1163	-0.8740541	-0.0195778	0.00038334	1.00062684	0.01575619	2238094.77	0.00049923	0.04992274	
1164	0.07326042	0.00147081	2.16E-06	1.00101849	0.01576239	2238148.71	0.00050489	0.05048937	
1165	2.9782247	0.06849148	0.00467158	0.99641872	0.01568856	2233027.15	0.00050702	0.05070161	*
1166	0.30946564	0.00681365	4.64E-05	1.00097501	0.01576165	2238060.75	0.00050755	0.05075484	
1167	-0.0028191	-0.0002408	5.80E-08	1.00102465	0.01576246	2238146.31	0.00050628	0.05062766	
1168	0.89036609	0.01998702	0.00039952	1.00060582	0.01575576	2238013.72	0.00050539	0.05053946	
1169	2.74682301	0.05347294	0.00285415	0.9978957	0.01571449	2238122.9	0.00036737	0.03673683	
1170	0.22585	0.00491834	2.42E-05	1.0009977	0.01576202	2238137.48	0.00050693	0.05069286	
1171	0.25913648	0.00545297	2.97E-05	1.0009124	0.01576128	2238148.32	0.00046762	0.04676229	
1172	0.37108596	0.0079392	6.31E-05	1.00088981	0.01576082	2238147.46	0.00047403	0.04740347	
1173	0.94714211	0.02133284	0.00045511	1.00055407	0.01575489	2230192.56	0.0005078	0.05077954	
1174	-0.8629222	-0.0194687	0.00037908	1.00064556	0.01575638	2237858.7	0.00050628	0.0506275	
1175	-1.3693154	-0.0299584	0.00089714	1.00006161	0.01574739	2238105.6	0.00048307	0.04830747	
1176	-1.3862991	-0.0308837	0.00095336	1.00003991	0.01574675	2237985.15	0.00050115	0.05011462	
1177	-1.6533565	-0.0369326	0.00136279	0.99962205	0.01573995	2236433.88	0.00050695	0.05069507	
1178	-1.0244194	-0.0229718	0.0005277	1.0004857	0.01575387	2238023.9	0.00050285	0.05028542	
1179	-0.4348108	-0.0098663	9.74E-05	1.00091818	0.01576086	2238131.81	0.0004998	0.04997952	
1180	-0.7392624	-0.0167414	0.00028034	1.00074908	0.01575802	2237005.88	0.00050754	0.05075421	
1181	-1.1585175	-0.0259547	0.00067353	1.00033688	0.01575147	2237951.44	0.00050388	0.05038759	
1182	-0.9685504	-0.0217637	0.00047368	1.00054379	0.01575478	2238001.23	0.00050404	0.05040352	
1183	0.03722766	0.0006448	4.16E-07	1.00095794	0.01576192	2238148.7	0.00047375	0.04737486	
1184	-1.1573146	-0.0259665	0.00067415	1.0003393	0.01575148	2237833	0.00050537	0.05053683	
1185	0.1207735	0.00252635	6.39E-06	1.00100134	0.01576221	2238148.65	0.0004989	0.04989024	
1186	1.15329235	0.02549079	0.00064967	1.00031334	0.01575138	2238122.02	0.00048603	0.04860251	
1187	-0.7284797	-0.0163553	0.00026756	1.00074401	0.01575808	2238112.38	0.00049865	0.04986473	
1188	-1.0937744	-0.0244595	0.00059821	1.00040928	0.01575267	2238044.19	0.00050112	0.05011182	
1189	-1.0077328	-0.0225378	0.00050795	1.00050011	0.01575415	2238072.34	0.00049994	0.04999374	
1190	-0.7578648	-0.0170879	0.00029206	1.00072903	0.01575776	2238064.91	0.00050356	0.05035573	
1191	-1.087492	-0.0243788	0.00059428	1.00041821	0.01575278	2237988.64	0.0005035	0.05034972	

© 2024 Zhang X et al. *JAMA Pediatrics.*

1192	-1.0725027	-0.024024	0.00057711	1.00043391	0.01575305	2238022.09	0.00050249	0.05024897	
1193	-1.3769967	-0.0303497	0.00092071	1.00005196	0.01574711	2238086.88	0.00049038	0.04903758	
1194	-0.7694694	-0.0172758	0.00029852	1.00071428	0.01575759	2238104.26	0.00049955	0.04995467	
1195	-1.1532851	-0.0259073	0.00067108	1.00034486	0.01575155	2237555.57	0.00050653	0.05065341	
1196	-0.9464987	-0.0209811	0.00044023	1.00054997	0.01575511	2238118.31	0.00049018	0.04901844	
1197	-1.1445402	-0.025714	0.00066111	1.0003552	0.01575172	2237564.02	0.00050653	0.05065339	
1198	-1.4496036	-0.0321078	0.00103035	0.99994904	0.01574537	2238047.28	0.0004961	0.04961018	
1199	-0.962586	-0.0214854	0.00046164	1.00054234	0.01575487	2238096.39	0.00049724	0.04972445	
1200	-1.1741948	-0.0263563	0.00069452	1.0003195	0.01575115	2237707.27	0.00050602	0.05060245	
1201	-1.4165715	-0.031688	0.00100361	0.99999662	0.01574598	2237625.79	0.00050566	0.05056599	
1202	-1.2694096	-0.0284945	0.00081168	1.00020039	0.01574922	2236305.79	0.00050734	0.05073389	
1203	-0.7513994	-0.0168079	0.00028257	1.00072233	0.01575779	2238120.1	0.00049551	0.04955088	
1204	-1.0750285	-0.0241657	0.00058394	1.00043419	0.01575299	2237760.49	0.00050609	0.05060883	
1205	-1.3927199	-0.0309864	0.0009597	1.00003076	0.01574662	2238009.38	0.00049991	0.0499914	
1206	-0.4960996	-0.0112925	0.00012757	1.00089974	0.01576047	2238073.23	0.00050558	0.05055841	
1207	3.24634325	0.07177169	0.00512912	0.99590185	0.0156809	2238022.78	0.00046762	0.04676229	*
1208	0.40505771	0.00884619	7.83E-05	1.00091049	0.01576086	2238145.33	0.00049227	0.04922664	
1209	0.30956554	0.00675912	4.57E-05	1.00095841	0.01576152	2238145.8	0.00049892	0.04989222	
1210	1.48086702	0.03271728	0.0010698	0.99988312	0.01574454	2238110.59	0.00048179	0.04817937	
1211	0.40505771	0.00884619	7.83E-05	1.00091049	0.01576086	2238145.33	0.00049227	0.04922664	
1212	-0.2856942	-0.0065573	4.30E-05	1.00097404	0.01576174	2238138.85	0.00050085	0.05008498	
1213	-0.362079	-0.0081535	6.65E-05	1.00092573	0.01576116	2238143.58	0.00048839	0.04883945	
1214	-0.6037587	-0.0136898	0.00018747	1.0008389	0.0157595	2238053.03	0.00050534	0.05053375	
1215	-0.5352203	-0.0121852	0.00014853	1.00088199	0.01576016	2237838.53	0.00050722	0.05072161	
1216	-0.3778321	-0.0079998	6.40E-05	1.00081686	0.0157603	2238147.31	0.00043348	0.04334778	
1217	0.32278918	0.0069929	4.89E-05	1.00093665	0.01576132	2238147.1	0.00048983	0.04898292	
1218	-0.4874855	-0.011039	0.00012191	1.00089425	0.01576047	2238127.44	0.00050008	0.0500082	
1219	-0.8620541	-0.0190964	0.00036472	1.00062328	0.01575632	2238125.8	0.00048811	0.04881088	
1220	0.87753135	0.01962456	0.00038517	1.00061326	0.01575594	2238095.6	0.00050181	0.05018127	
1221	-0.959032	-0.0215466	0.00046428	1.00055285	0.01575494	2238014.7	0.00050373	0.0503728	
1222	-0.5769024	-0.0126643	0.00016043	1.00080085	0.0157594	2238142.4	0.00047316	0.04731617	
1223	-0.690981	-0.0154147	0.00023768	1.0007599	0.01575845	2238130.14	0.00049151	0.04915087	
1224	-0.4198792	-0.0095604	9.14E-05	1.00092953	0.015761	2238124.8	0.00050247	0.05024718	
1225	-0.3382679	-0.0076822	5.90E-05	1.00094643	0.01576139	2238141.74	0.00049498	0.04949771	
1226	1.51530998	0.03399513	0.0011549	0.99982722	0.01574341	2238058.64	0.00049629	0.0496287	
1227	-0.4911101	-0.0111601	0.0001246	1.0008989	0.01576049	2238108.28	0.0005037	0.05037005	
1228	-0.9970293	-0.0224026	0.00050188	1.00051558	0.01575433	2237974.08	0.00050445	0.05044504	
1229	-1.3803871	-0.0309582	0.00095796	1.00004882	0.01574678	1905303.26	0.00050784	0.05078397	
1230	-1.019957	-0.022957	0.00052702	1.00049392	0.01575394	2237674.81	0.00050654	0.05065387	
1231	-0.1182143	-0.0027797	7.73E-06	1.00098075	0.01576206	2238147.73	0.00048756	0.04875625	
1232	-0.2481689	-0.0057105	3.26E-05	1.00098064	0.01576187	2238142.34	0.00049912	0.04991249	
1233	-0.6906964	-0.0155994	0.00024341	1.00077885	0.01575856	2238075.42	0.00050369	0.05036941	

© 2024 Zhang X et al. *JAMA Pediatrics.*

1234	-0.8071795	-0.0181808	0.0003306	1.0006896	0.01575713	2238055.5	0.00050353	0.05035252	
1235	-0.4941936	-0.0111086	0.00012345	1.00087852	0.01576033	2238137.28	0.00049309	0.04930932	
1236	-0.7513961	-0.0169704	0.00028806	1.00073623	0.01575786	2238020.11	0.00050509	0.0505092	
1237	0.00855551	1.68E-05	2.81E-10	1.00100464	0.0157623	2238148.48	0.00049643	0.04964342	
1238	-1.1488628	-0.0256908	0.00065991	1.00034692	0.01575166	2238019.64	0.00050188	0.05018752	
1239	-1.2336188	-0.0275352	0.00075799	1.00024363	0.01575002	2238017.02	0.00050116	0.05011628	
1240	0.52550919	0.01168635	0.00013662	1.00087528	0.01576009	2238113.01	0.00050506	0.05050645	
1241	-0.6777975	-0.0152726	0.00023332	1.00078375	0.01575868	2238105.38	0.00050106	0.05010616	
1242	-0.4403236	-0.0099979	0.0001	1.00091743	0.01576083	2238129.24	0.00050071	0.05007074	
1243	-1.4181656	-0.0315556	0.00099525	0.99999441	0.01574603	2237996.87	0.00050031	0.0500314	
1244	-0.7119356	-0.016127	0.00026015	1.00076893	0.01575835	2237656.34	0.00050719	0.05071904	
1245	-0.5641877	-0.0127122	0.00016166	1.00084978	0.01575979	2238127.43	0.00049786	0.04978615	
1246	-0.5319469	-0.0120552	0.00014538	1.00087561	0.01576013	2238112.69	0.00050251	0.05025109	
1247	-0.355525	-0.0080835	6.54E-05	1.00094493	0.01576133	2238139.46	0.00049731	0.04973137	
1248	-0.8978762	-0.0202657	0.00041074	1.00061522	0.01575588	2237200.38	0.00050733	0.05073273	
1249	-0.8302636	-0.0187215	0.00035055	1.00067238	0.01575683	2237994.51	0.00050509	0.05050949	
1250	-1.5073013	-0.0336258	0.00112996	0.99986077	0.01574383	2237817.71	0.00050397	0.05039656	
1251	-0.9342481	-0.0209996	0.00044102	1.0005769	0.01575532	2238019.17	0.00050379	0.0503786	
1252	-1.0774632	-0.024166	0.00058395	1.00042961	0.01575296	2237977.36	0.00050385	0.05038523	
1253	-1.0528718	-0.0234882	0.00055167	1.00045127	0.0157534	2238081.43	0.00049811	0.04981145	
1254	-0.9586268	-0.0215083	0.00046263	1.00055175	0.01575494	2238048.2	0.00050236	0.05023623	
1255	-0.3778321	-0.0079998	6.40E-05	1.00081686	0.0157603	2238147.31	0.00043348	0.04334778	
1256	-0.7723258	-0.0174353	0.00030405	1.00071991	0.01575759	2238013.58	0.00050509	0.0505092	
1257	-1.5226862	-0.0340474	0.00115843	0.99983605	0.01574339	2237186.36	0.00050648	0.0506483	
1258	-0.356821	-0.0081799	6.69E-05	1.00095995	0.01576144	2238106.94	0.00050549	0.05054877	
1259	-0.8458137	-0.0186314	0.00034718	1.00062956	0.01575651	2238131.59	0.00048237	0.04823701	
1260	-1.2608011	-0.0282907	0.00080012	1.00021142	0.01574941	2237228.89	0.00050685	0.05068459	
1261	-1.3806743	-0.0309187	0.00095552	1.00004828	0.0157468	2237427.84	0.00050633	0.05063341	
1262	1.13007759	0.02532737	0.00064138	1.00034977	0.01575174	2238077.72	0.00050001	0.05000065	
1263	-0.6994891	-0.0157311	0.00024753	1.00076648	0.01575843	2238110.68	0.00049967	0.04996729	
1264	-1.1001993	-0.024418	0.00059618	1.00039621	0.01575258	2238098.57	0.00049369	0.04936891	
1265	-0.5442448	-0.0123295	0.00015207	1.00086902	0.01576003	2238110.74	0.00050258	0.05025806	
1266	-0.8250618	-0.0185993	0.00034599	1.0006763	0.0157569	2238014.21	0.00050473	0.05047284	
1267	0.10398048	0.00211817	4.49E-06	1.00097098	0.01576198	2238148.72	0.00048277	0.04827664	
1268	-0.0739097	-0.0018254	3.33E-06	1.0010076	0.01576231	2238147.33	0.00049894	0.04989419	
1269	-0.7253555	-0.0162359	0.00026367	1.00074174	0.0157581	2238121.69	0.00049558	0.04955838	
1270	-0.6218661	-0.0141005	0.00019889	1.00082842	0.01575932	2238021.19	0.00050586	0.05058622	
1271	-0.2873872	-0.0066151	4.38E-05	1.00097935	0.01576178	2238131.3	0.00050384	0.05038384	
1272	-0.7631604	-0.0172129	0.00029635	1.00072554	0.0157577	2238054.23	0.00050399	0.05039934	
1273	0.37994582	0.00840219	7.06E-05	1.00094725	0.01576122	2238115.46	0.0005065	0.05065018	
1274	0.71253243	0.01582846	0.0002506	1.00074428	0.01575809	2238127.21	0.00049849	0.04984872	
1275	-0.1313695	-0.0031149	9.71E-06	1.00100668	0.01576226	2238145.18	0.00050127	0.05012664	

© 2024 Zhang X et al. *JAMA Pediatrics*.

1276	-0.0048214	-0.0002679	7.18E-08	1.0009453	0.01576182	2238148.64	0.00046701	0.04670115	
1277	0.36841367	0.00813757	6.62E-05	1.00095099	0.01576129	2238125.56	0.00050606	0.05060631	
1278	0.07497681	0.00150435	2.26E-06	1.00101061	0.01576232	2238148.72	0.00050106	0.05010559	
1279	0.15946989	0.00336782	1.13E-05	1.00098008	0.01576199	2238148.55	0.00049117	0.04911708	
1280	-0.530281	-0.012078	0.00014593	1.00088514	0.01576021	2237624.56	0.00050748	0.05074779	
1281	0.4937813	0.01087295	0.00011827	1.00087676	0.01576025	2238141.29	0.0004963	0.04963035	
1282	0.05054986	0.00096046	9.23E-07	1.00102177	0.01576243	2238148.47	0.00050571	0.05057119	
1283	-0.3504446	-0.0080057	6.41E-05	1.00095484	0.01576142	2238133.46	0.00050159	0.05015929	
1284	0.52991612	0.01173672	0.0001378	1.00086543	0.01576	2238134.37	0.00050078	0.05007824	
1285	-0.560034	-0.012727	0.00016204	1.00086642	0.01575993	2238017.48	0.00050624	0.05062407	
1286	-0.7307313	-0.0164452	0.00027051	1.00074596	0.01575808	2238098.98	0.00050108	0.05010844	
1287	0.18717205	0.00402812	1.62E-05	1.00099789	0.01576209	2238147.66	0.00050269	0.05026883	
1288	0.8520621	0.01913082	0.00036604	1.00064178	0.01575632	2237954.5	0.0005063	0.05062991	
1289	0.31523321	0.00689122	4.75E-05	1.00095793	0.0157615	2238145.39	0.00049964	0.04996396	
1290	0.28981829	0.00633649	4.02E-05	1.0009711	0.01576167	2238144.83	0.00050228	0.05022828	
1291	0.36841367	0.00813757	6.62E-05	1.00095099	0.01576129	2238125.56	0.00050606	0.05060631	
1292	-0.4931397	-0.011139	0.00012413	1.00088745	0.0157604	2238131.92	0.00049782	0.04978242	
1293	-0.0471635	-0.0012401	1.54E-06	1.00102607	0.01576247	2238130.09	0.00050738	0.05073771	
1294	0.54298866	0.01208984	0.00014622	1.00086653	0.01575994	2238099.29	0.00050567	0.0505674	
1295	-0.9720481	-0.0218748	0.00047852	1.00054198	0.01575473	2237897.32	0.00050559	0.05055935	
1296	-0.9490802	-0.0213825	0.00045724	1.00056549	0.0157551	2237780.2	0.00050637	0.05063742	
1297	-0.3838339	-0.0087908	7.73E-05	1.00095126	0.01576129	2238078.54	0.00050626	0.05062627	
1298	-0.6580641	-0.0147521	0.00021769	1.00078829	0.01575884	2238126.18	0.00049549	0.04954899	
1299	0.5674408	0.01229897	0.00015131	1.00080583	0.0157594	2238144.68	0.00047815	0.0478146	
1300	0.75972799	0.0170075	0.00028932	1.00071803	0.01575756	2238061.06	0.00050519	0.05051942	
1301	-0.4865843	-0.0110141	0.00012136	1.0008939	0.01576047	2238128.65	0.00049964	0.04996396	
1302	1.0365087	0.02311415	0.00053424	1.00045083	0.01575342	2238108.48	0.00049637	0.04963693	
1303	-0.0624866	-0.0015707	2.47E-06	1.00100861	0.01576232	2238147.5	0.0004991	0.04990965	
1304	0.50538276	0.01120835	0.00012568	1.00088267	0.01576024	2238129.74	0.00050308	0.05030784	
1305	0.12197653	0.00256392	6.58E-06	1.00101014	0.01576228	2238148.56	0.00050337	0.05033718	
1306	-0.8506237	-0.0191736	0.00036768	1.00065488	0.01575655	2237986.35	0.00050512	0.05051153	
1307	0.07065793	0.00140886	1.99E-06	1.00101315	0.01576235	2238148.71	0.00050214	0.05021372	
1308	1.86851044	0.04245957	0.00180044	0.99920492	0.01573328	2237515.08	0.00050529	0.05052939	
1309	1.92126975	0.04245046	0.00179975	0.99915013	0.01573285	2238091.99	0.00047759	0.04775885	
1310	3.11550187	0.06941242	0.00479859	0.99623423	0.0156861	2238004.12	0.00047566	0.04756551	*
1311	1.63420214	0.03695244	0.00136428	0.99963183	0.01574017	2237906.78	0.0005028	0.05028012	
1312	1.29731914	0.02887131	0.00083326	1.00013827	0.01574849	2238104.54	0.00049089	0.04908902	
1313	-0.5906928	-0.0134178	0.0001801	1.00084925	0.01575964	2237935.07	0.00050676	0.05067626	
1314	-0.3408912	-0.007826	6.13E-05	1.00096616	0.01576153	2238104.02	0.0005058	0.05057974	
1315	-0.5444845	-0.0123696	0.00015306	1.00087373	0.01576006	2238067.06	0.00050539	0.05053916	
1316	-1.7775369	-0.039439	0.00155373	0.99940909	0.01573662	2237875.05	0.00050143	0.05014287	
1317	0.32893572	0.00724717	5.25E-05	1.00096635	0.01576153	2238126.33	0.00050648	0.05064775	

© 2024 Zhang X et al. *JAMA Pediatrics*.

1318	0.38585909	0.00847139	7.18E-05	1.00093032	0.01576107	2238143.62	0.00049871	0.04987099	
1319	0.8913382	0.02005019	0.00040205	1.00060741	0.01575575	2237258.91	0.00050747	0.05074707	
1320	0.5558877	0.01239647	0.00015373	1.00086105	0.01575983	2238037.37	0.00050683	0.05068256	
1321	-0.2107034	-0.0049084	2.41E-05	1.00100177	0.01576211	2238129.86	0.00050551	0.05055142	
1322	-0.2020945	-0.0047159	2.23E-05	1.00100377	0.01576214	2238130.01	0.00050563	0.05056306	
1323	-0.3258748	-0.007493	5.62E-05	1.00097199	0.01576162	2238097.17	0.00050619	0.05061925	
1324	-1.5321157	-0.0341028	0.00116221	0.99982285	0.01574325	2237922.84	0.00050198	0.05019837	
1325	-1.2546038	-0.0281315	0.00079115	1.00021911	0.01574954	2237645.12	0.00050604	0.05060388	
1326	0.12018394	0.00252473	6.38E-06	1.00101133	0.01576229	2238148.56	0.00050385	0.05038484	
1327	-1.7404088	-0.0388339	0.00150648	0.99947003	0.01573751	2236585.52	0.00050676	0.05067637	
1328	-1.2208326	-0.0272336	0.00074149	1.00025922	0.01575028	2238032.85	0.0005004	0.05003975	
1329	-1.606395	-0.035892	0.00128718	0.9997011	0.01574122	2236917.43	0.00050667	0.05066665	
1330	-1.2865951	-0.0287938	0.00082881	1.00017678	0.01574889	2237862.21	0.00050452	0.0504519	
1331	-1.1441334	-0.0256888	0.00065981	1.00035524	0.01575173	2237756.5	0.00050589	0.05058929	
1332	1.25867952	0.02838615	0.00080552	1.00019351	0.01574916	2237936.99	0.00050453	0.05045326	
1333	0.48258551	0.01047583	0.00010978	1.00085803	0.01576017	2238145.49	0.00048261	0.04826093	
1334	-0.564672	-0.0128054	0.00016404	1.00086042	0.01575986	2238088.84	0.00050428	0.05042825	
1335	0.69026457	0.0153713	0.00023634	1.00076496	0.01575837	2238119.32	0.00050148	0.05014793	
1336	3.80905888	0.08646328	0.00742672	0.99376318	0.01564626	2237799.05	0.00048781	0.04878145	*
1337	2.39131474	0.05288568	0.00279077	0.99817183	0.01571721	2238067.76	0.00047445	0.04744549	
1338	0.91598625	0.0203585	0.0004145	1.00056964	0.01575535	2238121.32	0.00049498	0.04949771	
1339	2.22619718	0.04990144	0.0024853	0.9984985	0.01572222	2238027.83	0.00048858	0.04885805	
1340	1.75705396	0.03958846	0.00156556	0.9994225	0.01573689	2238004.58	0.00049798	0.04979836	
1341	1.11311333	0.02492081	0.00062097	1.00036875	0.01575205	2238086.48	0.00049919	0.04991904	
1342	-0.5830096	-0.0129757	0.00016842	1.00081974	0.01575949	2238138.25	0.00048643	0.04864309	
1343	-1.0476233	-0.0231449	0.00053567	1.00044805	0.0157535	2238115.34	0.00048844	0.04884414	
1344	0.15715742	0.00334733	1.12E-05	1.00099981	0.01576215	2238148.33	0.00050087	0.05008679	
1345	-0.0862303	-0.0020746	4.31E-06	1.00098397	0.01576211	2238148.02	0.00048766	0.04876641	
1346	1.01806	0.02292434	0.00052551	1.00047978	0.01575372	2237845.37	0.00050638	0.05063817	
1347	0.62694142	0.01397049	0.00019524	1.00081206	0.01575909	2238111.31	0.00050383	0.05038325	
1348	2.56919772	0.05594118	0.00312181	0.99781823	0.01571178	2238084.71	0.00045881	0.04588139	
1349	0.53988347	0.01196763	0.00014328	1.00086075	0.01575991	2238132.58	0.00050128	0.05012825	
1350	0.76588773	0.01711599	0.00029302	1.00071047	0.01575747	2238096.4	0.00050332	0.05033217	
1351	1.71041318	0.0379037	0.00143535	0.99952032	0.01573871	2238095.91	0.00048237	0.04823701	
1352	7.08514395	0.16475131	0.02650826	0.97652658	0.01536975	2236998.53	0.00048671	0.04867117	*
1353	-0.3988985	-0.0090643	8.22E-05	1.0009324	0.01576109	2238134.84	0.00049935	0.04993544	
1354	1.14123909	0.02575593	0.00066326	1.00034138	0.01575149	2237588.76	0.00050683	0.05068291	
1355	0.50665182	0.01100422	0.00012114	1.00084538	0.01575997	2238145.15	0.00048221	0.0482208	
1356	0.10475598	0.00218478	4.78E-06	1.00102043	0.01576238	2238148.18	0.00050743	0.05074263	
1357	1.64883265	0.03743505	0.0014001	0.99960406	0.01573967	2237058.22	0.0005067	0.05067036	
1358	0.37368654	0.00824256	6.80E-05	1.00094556	0.01576123	2238136.75	0.00050427	0.05042699	
1359	-1.4719058	-0.0328391	0.00107776	0.99991484	0.0157447	2237856.48	0.00050364	0.05036444	

© 2024 Zhang X et al. *JAMA Pediatrics*.

1360	-0.047345	-0.0012438	1.55E-06	1.00102554	0.01576246	2238136.71	0.00050712	0.05071189	
1361	1.48206869	0.03235863	0.0010465	0.99988395	0.01574474	2238121.97	0.0004706	0.04705971	
1362	1.12936697	0.0253023	0.00064011	1.00035035	0.01575175	2238080.85	0.00049966	0.04996563	
1363	-1.6422527	-0.0366734	0.00134376	0.99964122	0.01574026	2237009.27	0.00050652	0.05065173	
1364	0.45390584	0.01003455	0.00010073	1.00090666	0.01576064	2238136.67	0.00050206	0.05020552	
1365	-0.246014	-0.0057036	3.25E-05	1.00099502	0.01576199	2238115.36	0.00050619	0.05061908	
1366	-0.7590339	-0.0171015	0.00029253	1.00072708	0.01575774	2238076.85	0.00050283	0.05028325	
1367	-0.3734519	-0.0085152	7.25E-05	1.0009458	0.01576128	2238132.7	0.00050124	0.05012395	
1368	2.59366031	0.05834045	0.00339402	0.9976139	0.01570802	2237981.4	0.00048876	0.04887629	
1369	-0.5730159	-0.0129982	0.00016901	1.0008565	0.01575979	2238076.34	0.00050482	0.05048207	
1370	0.11639679	0.00244063	5.96E-06	1.00101281	0.0157623	2238148.57	0.00050434	0.05043398	
1371	-0.5666925	-0.0128872	0.00016614	1.00086413	0.01575987	2237837.99	0.00050715	0.0507152	
1372	-0.5717384	-0.0129927	0.00016887	1.00086024	0.01575982	2237973.93	0.0005066	0.05065957	
1373	-0.2472956	-0.0055011	3.03E-05	1.0009183	0.01576138	2238147.39	0.00046722	0.04672174	
1374	0.52896569	0.01175201	0.00013816	1.00087146	0.01576004	2238122.73	0.00050396	0.05039637	
1375	-0.53026	-0.0120757	0.00014588	1.00088489	0.0157602	2237777.55	0.00050733	0.05073274	
1376	-0.8120682	-0.0183341	0.0003362	1.0006889	0.01575708	2237928.45	0.00050599	0.05059945	
1377	-0.3573562	-0.0082035	6.73E-05	1.00096241	0.01576146	2238044.85	0.00050689	0.05068926	
1378	0.13220406	0.00280111	7.85E-06	1.00101425	0.0157623	2238148.02	0.00050613	0.05061337	
1379	-0.2324238	-0.0053384	2.85E-05	1.00097674	0.01576187	2238144.73	0.00049524	0.04952387	
1380	-0.2196294	-0.0051021	2.60E-05	1.00099745	0.01576206	2238135.58	0.0005043	0.05042966	
1381	-0.3260477	-0.0075006	5.63E-05	1.00097289	0.01576163	2238074.64	0.00050669	0.0506693	
1382	-1.2753952	-0.0284793	0.00081081	1.0001906	0.01574915	2237985.28	0.00050211	0.05021128	
1383	-1.6363155	-0.0365127	0.00133202	0.99965183	0.01574045	2237459.16	0.00050567	0.05056676	
1384	-1.2307442	-0.0275963	0.00076136	1.00024944	0.01575004	2237737	0.00050571	0.05057142	
1385	-0.1056217	-0.0024995	6.25E-06	1.00098023	0.01576207	2238147.89	0.00048666	0.04866561	
1386	-0.2909263	-0.0067117	4.51E-05	1.00098331	0.0157618	2238098.99	0.00050641	0.05064115	
1387	-1.023271	-0.0230226	0.00053003	1.0004901	0.01575389	2237771.38	0.0005062	0.05061952	
1388	-0.6379757	-0.0144458	0.00020875	1.00081652	0.01575915	2238058.29	0.00050492	0.05049215	
1389	-0.6737237	-0.0152594	0.00023292	1.00079446	0.01575877	2237978.91	0.00050613	0.05061286	
1390	-0.5227427	-0.0118962	0.00014157	1.00088725	0.01576026	2238023.14	0.00050636	0.05063569	
1391	-0.9120002	-0.0205282	0.00042145	1.0005991	0.01575566	2237982.87	0.00050481	0.05048105	
1392	-0.1585587	-0.0037434	1.40E-05	1.00101363	0.01576228	2238123.33	0.00050667	0.05066713	
1393	-0.1356313	-0.0032289	1.04E-05	1.00101729	0.01576234	2238124.35	0.00050684	0.05068423	
1394	-0.4636939	-0.0105214	0.00011074	1.00090746	0.01576067	2238126.27	0.00050108	0.05010844	
1395	-1.0417049	-0.0233389	0.00054469	1.00046689	0.01575358	2238034.17	0.00050224	0.05022364	
1396	-0.7996142	-0.0178193	0.00031759	1.00068105	0.01575717	2238122.49	0.00049279	0.04927902	
1397	-0.6908564	-0.0154752	0.00023955	1.00076617	0.01575849	2238124.06	0.00049553	0.04955276	
1398	-0.2662142	-0.0060902	3.71E-05	1.0009692	0.01576175	2238143.67	0.00049562	0.0495621	
1399	-0.76506	-0.0173077	0.00029962	1.00072844	0.0157577	2237681.34	0.00050706	0.05070606	
1400	0.18050903	0.00385644	1.49E-05	1.00098749	0.01576202	2238148.29	0.00049679	0.0496794	
1401	-1.7256192	-0.0383293	0.00146766	0.99950121	0.01573809	2237865.16	0.00050199	0.05019922	

© 2024 Zhang X et al. *JAMA Pediatrics*.

1402	-0.8969846	-0.0192126	0.00036916	1.00055527	0.01575573	2238139.22	0.00045696	0.04569563	
1403	-1.2314468	-0.0273048	0.00074537	1.00024314	0.01575011	2238083.02	0.00049451	0.04945087	
1404	-0.1682223	-0.0039093	1.53E-05	1.0009872	0.01576206	2238146.39	0.00049419	0.04941925	
1405	-0.8158941	-0.0183016	0.00033501	1.00067707	0.01575699	2238099.08	0.00049958	0.04995807	
1406	-0.2888762	-0.00649	4.21E-05	1.00093398	0.01576142	2238146.17	0.00048065	0.04806511	
1407	1.97744893	0.04397601	0.00193117	0.99903183	0.01573086	2238074.72	0.00048323	0.0483226	
1408	-0.177461	-0.004151	1.72E-05	1.00100264	0.01576217	2238141.98	0.00050273	0.05027276	
1409	-0.8637705	-0.0195015	0.00038036	1.00064572	0.01575637	2237609.37	0.000507	0.05069982	
1410	-0.4776455	-0.0108959	0.00011877	1.0009114	0.01576064	2237962.4	0.00050698	0.05069846	
1411	-0.4934407	-0.0111928	0.00012533	1.0008947	0.01576045	2238119.87	0.00050199	0.05019922	
1412	-1.5127316	-0.0333598	0.00111218	0.99985584	0.01574393	2238064.74	0.00049247	0.04924744	
1413	2.00882539	0.04562306	0.00207815	0.99892747	0.01572886	2237728.74	0.00050337	0.05033694	
1414	-1.3365668	-0.0298047	0.00088797	1.00010895	0.01574785	2237987.13	0.00050151	0.05015143	
1415	1.35131814	0.03000684	0.00090004	1.00006568	0.01574737	2238107.31	0.00048811	0.04881088	
1416	-0.361651	-0.0081502	6.65E-05	1.00092724	0.01576118	2238143.39	0.00048912	0.04891176	
1417	0.34803182	0.00756507	5.73E-05	1.00093057	0.0157612	2238146.57	0.00049121	0.04912052	
1418	-0.5874772	-0.013208	0.00017451	1.00083388	0.01575956	2238128.77	0.00049642	0.04964181	
1419	-0.5660086	-0.0127938	0.00016374	1.00085418	0.01575981	2238117.18	0.00050106	0.05010616	
1420	0.33410272	0.00737379	5.44E-05	1.00096713	0.01576152	2227173.34	0.00050784	0.050784	
1421	0.46806691	0.01041294	0.00010847	1.00091021	0.0157606	2212758.45	0.00050784	0.050784	
1422	0.13473353	0.00285276	8.14E-06	1.00100981	0.01576226	2238148.37	0.0005041	0.05040989	
1423	-0.2576845	-0.0059585	3.55E-05	1.00098988	0.01576193	2238127.4	0.00050507	0.05050731	
1424	-0.0417421	-0.0011185	1.25E-06	1.001027	0.01576248	2238076.9	0.00050773	0.05077318	
1425	-0.0425417	-0.0011365	1.29E-06	1.00102697	0.01576248	2238075.98	0.00050773	0.05077318	
1426	0.76009011	0.01697898	0.00028835	1.00071462	0.01575754	2238100.66	0.000503	0.0503004	
1427	0.83112596	0.0183039	0.00033508	1.00063554	0.01575652	2238134.75	0.00048746	0.04874599	
1428	0.38093473	0.00828421	6.87E-05	1.00091499	0.01576097	2238146.26	0.00048946	0.04894593	
1429	0.87646807	0.01858816	0.00034555	1.00055203	0.01575576	2238143.02	0.00045124	0.04512375	
1430	-0.3054283	-0.0070362	4.95E-05	1.00097884	0.01576173	2238096.95	0.00050636	0.05063593	
1431	-0.2753679	-0.0063617	4.05E-05	1.00098737	0.01576187	2238108.54	0.00050622	0.05062158	
1432	-0.2712388	-0.0062672	3.93E-05	1.00098789	0.01576188	2238115.84	0.0005059	0.05059	
1433	-0.2695533	-0.006193	3.84E-05	1.00097713	0.0157618	2238140.53	0.00050014	0.05001413	
1434	0.17038944	0.00364365	1.33E-05	1.00099709	0.01576211	2238148.19	0.00050067	0.05006693	
1435	-0.3916074	-0.0089757	8.06E-05	1.00095049	0.01576125	2237822.39	0.00050749	0.05074915	
1436	-0.4665715	-0.0105664	0.00011169	1.00090291	0.01576062	2238130.76	0.00049931	0.04993095	
1437	-0.4658478	-0.0106201	0.00011283	1.00091505	0.01576071	2238071.73	0.00050585	0.05058529	
1438	0.32935966	0.00720699	5.20E-05	1.0009527	0.01576142	2238145.08	0.0004994	0.04993989	
1439	0.03633625	0.00064026	4.10E-07	1.00102154	0.01576243	2238148.3	0.00050522	0.05052226	
1440	-0.8751038	-0.0197669	0.00039078	1.00063645	0.01575621	2235030.73	0.00050769	0.05076933	
1441	0.10610575	0.00221582	4.91E-06	1.00102084	0.01576238	2238146.79	0.00050771	0.05077103	
1442	-0.2837113	-0.0065273	4.26E-05	1.00097876	0.01576178	2238134.64	0.00050299	0.05029894	
1443	-0.2546986	-0.0058847	3.46E-05	1.0009884	0.01576192	2238133.94	0.00050392	0.05039203	

© 2024 Zhang X et al. *JAMA Pediatrics*.

1444	-0.2357559	-0.0054825	3.01E-05	1.00100064	0.01576205	2237267	0.00050778	0.05077842	
1445	0.53731519	0.01197342	0.00014342	1.00087156	0.01576	2238055.12	0.00050673	0.05067259	
1446	-0.3077368	-0.0070453	4.97E-05	1.00096666	0.01576163	2238138.29	0.00050041	0.05004112	
1447	0.52454378	0.01160348	0.00013469	1.00086656	0.01576003	2238136.51	0.00049974	0.04997386	
1448	0.10100884	0.00209439	4.39E-06	1.00101471	0.01576233	2238148.68	0.00050437	0.05043655	
1449	-0.3571965	-0.008165	6.67E-05	1.00095446	0.0157614	2238129.72	0.00050266	0.05026618	
1450	0.61439283	0.01373922	0.00018883	1.00082665	0.01575926	2235274.42	0.00050779	0.05077932	
1451	0.71914893	0.0161263	0.00026012	1.0007533	0.01575809	2170612.59	0.00050784	0.05078399	
1452	0.21238914	0.00461034	2.13E-05	1.00099918	0.01576206	2238143.97	0.00050607	0.05060697	
1453	1.58627385	0.03474504	0.00120637	0.99972835	0.01574221	2238116.15	0.00047255	0.04725542	
1454	0.57017135	0.01261946	0.0001593	1.00083889	0.01575961	2238135.56	0.00049869	0.04986891	
1455	0.35559038	0.00765656	5.86E-05	1.00090908	0.01576101	2238147.29	0.00048128	0.04812785	
1456	-0.9009746	-0.0201219	0.00040493	1.00059972	0.0157558	2238104.27	0.00049679	0.0496794	
1457	-1.4829618	-0.0330178	0.00108952	0.99989853	0.01574447	2237946.26	0.0005017	0.05016977	
1458	1.06429287	0.02399823	0.00057587	1.00043013	0.01575291	2237527.39	0.00050706	0.05070573	
1459	2.83640873	0.06410497	0.00409507	0.99694147	0.0156972	2237916.72	0.00049131	0.04913076	
1460	-0.0530981	-0.001317	1.74E-06	1.00095191	0.01576187	2238148.48	0.00047077	0.04707684	
1461	-0.7791395	-0.0175185	0.00030696	1.00070903	0.01575748	2238091.9	0.0005012	0.05012014	
1462	0.06502249	0.00128275	1.65E-06	1.00101423	0.01576236	2238148.7	0.00050246	0.05024575	
1463	0.4373648	0.00969572	9.40E-05	1.00092101	0.01576081	2238118.98	0.0005057	0.05057045	
1464	0.89457543	0.02007185	0.00040292	1.00060123	0.01575569	2238037.79	0.00050483	0.0504832	
1465	1.66244794	0.03754922	0.00140864	0.99958494	0.01573944	2237952.64	0.00050139	0.05013927	
1466	-0.3310639	-0.0075752	5.74E-05	1.00096182	0.01576153	2238134.37	0.00050178	0.05017764	
1467	0.05404002	0.00102763	1.06E-06	1.00099485	0.01576221	2238148.7	0.00049247	0.04924744	
1468	-1.0931044	-0.0243505	0.00059289	1.0004069	0.0157527	2238082.43	0.00049726	0.04972584	
1469	-0.3144432	-0.0071934	5.18E-05	1.00096425	0.01576159	2238138.17	0.00050024	0.0500243	
1470	0.43401217	0.00932751	8.70E-05	1.00086651	0.01576043	2238146.8	0.000475	0.04749992	
1471	0.17971856	0.00387761	1.50E-05	1.00100866	0.01576219	2238138.73	0.00050737	0.05073735	
1472	0.66123917	0.01468292	0.00021565	1.00078196	0.01575868	2238128.77	0.00049934	0.04993365	
1473	-1.0954721	-0.0246167	0.00060592	1.00041135	0.01575263	2237758.14	0.00050604	0.05060367	
1474	0.05691883	0.00110263	1.22E-06	1.00101866	0.0157624	2238148.63	0.00050437	0.05043715	
1475	-0.8965353	-0.0201912	0.00040773	1.00061376	0.01575589	2237971.46	0.00050509	0.0505093	
1476	-1.2410008	-0.0268007	0.00071811	1.00021964	0.01575014	2238125.82	0.00046917	0.04691671	
1477	0.25425923	0.00553104	3.06E-05	1.00097957	0.01576182	2238146.43	0.00050138	0.05013824	
1478	-1.1227647	-0.025238	0.00063687	1.00038077	0.01575213	2237470.77	0.00050675	0.05067535	
1479	-0.4800374	-0.0108848	0.00011853	1.0008997	0.01576054	2238124.86	0.0005011	0.05010957	
1480	2.46480081	0.05372387	0.00287985	0.99805716	0.01571559	2238087.82	0.00046054	0.04605406	
1481	-0.0280858	-0.0008107	6.58E-07	1.00102715	0.01576248	2238120.86	0.00050762	0.05076156	
1482	1.2220149	0.02733197	0.00074684	1.00023691	0.01574998	2238089.36	0.00049675	0.04967484	
1483	-0.4859233	-0.011073	0.00012266	1.00090605	0.01576056	2238044.02	0.00050627	0.05062704	
1484	-0.0382814	-0.0010377	1.08E-06	1.00102238	0.01576244	2238145.67	0.00050539	0.05053872	
1485	0.75378426	0.01677994	0.00028163	1.00071478	0.0157576	2238120.82	0.00049966	0.04996563	

© 2024 Zhang X et al. *JAMA Pediatrics*.

1486	0.71745521	0.01596106	0.00025482	1.00074266	0.01575804	2238123.2	0.00049984	0.04998352	
1487	0.1082417	0.00224966	5.06E-06	1.0010066	0.01576226	2238148.68	0.00050075	0.05007451	
1488	0.14066915	0.00297609	8.86E-06	1.00100157	0.01576219	2238148.5	0.00050042	0.05004249	
1489	-0.1777658	-0.0041722	1.74E-05	1.00100924	0.01576222	2238129.18	0.00050607	0.05060697	
1490	0.20033439	0.00431375	1.86E-05	1.00099015	0.01576201	2238147.82	0.00050016	0.0500156	
1491	-0.4152883	-0.0094103	8.86E-05	1.00092224	0.01576096	2238136.58	0.00049748	0.04974762	
1492	0.20476834	0.00441676	1.95E-05	1.00099082	0.01576201	2238147.63	0.00050098	0.05009808	
1493	1.26593445	0.0285483	0.00081474	1.00018395	0.01574901	2237943.35	0.00050439	0.05043879	
1494	0.3304459	0.00728357	5.31E-05	1.00096643	0.01576152	2238119.24	0.00050679	0.05067931	
1495	0.17498664	0.00375086	1.41E-05	1.00099849	0.01576212	2238148.02	0.0005018	0.05017991	
1496	-1.2285271	-0.0275798	0.00076045	1.00025282	0.01575007	2237213.7	0.00050691	0.05069086	
1497	-0.0241662	-0.0007182	5.16E-07	1.00101764	0.01576241	2238147.55	0.00050287	0.05028696	
1498	-0.9742675	-0.021804	0.00047543	1.00053392	0.01575469	2238075.62	0.00050008	0.0500082	
1499	-0.5722903	-0.0125998	0.00015881	1.00080782	0.01575947	2238141.97	0.00047578	0.04757831	
1500	-1.4650973	-0.0325893	0.00106145	0.99992556	0.01574492	2237982.25	0.00050054	0.05005392	
1501	-1.0237787	-0.0228749	0.00052325	1.0004829	0.01575388	2238076.49	0.00049924	0.04992366	
1502	-0.6060144	-0.0137658	0.00018956	1.0008406	0.0157595	2237764.98	0.00050721	0.05072137	
1503	-0.3523636	-0.0078771	6.21E-05	1.00091478	0.01576111	2238145.19	0.00048083	0.04808333	
1504	0.19423061	0.00410612	1.69E-05	1.00095683	0.01576175	2238148.47	0.00048269	0.04826881	
1505	0.15829418	0.00337283	1.14E-05	1.00099961	0.01576215	2238148.32	0.00050086	0.05008619	
1506	-0.9332504	-0.0208789	0.00043596	1.00057248	0.01575532	2238088.99	0.00049907	0.04990679	
1507	0.39268718	0.0086558	7.50E-05	1.00093434	0.01576108	2238139.86	0.00050236	0.05023585	
1508	-1.1535034	-0.0258336	0.00066727	1.00034254	0.01575157	2237971.44	0.00050346	0.05034642	
1509	-1.2704531	-0.0285243	0.00081338	1.00019912	0.0157492	2234622.25	0.00050758	0.05075791	
1510	-1.6801399	-0.0375525	0.00140885	0.9995753	0.01573919	2049229.9	0.00050783	0.05078346	
1511	-1.1033449	-0.0242316	0.00058712	1.00038468	0.01575256	2238119.51	0.00048345	0.04834495	
1512	-0.9295094	-0.0209717	0.00043985	1.00058566	0.0157554	2236646.01	0.0005075	0.05074962	
1513	-0.8106791	-0.0183291	0.00033602	1.000692	0.01575711	2237181.63	0.00050742	0.05074231	
1514	-1.0242984	-0.023044	0.00053102	1.00048897	0.01575387	2237783.9	0.00050614	0.05061355	
1515	-0.5343379	-0.0120684	0.0001457	1.00086864	0.01576007	2238126.25	0.00049924	0.04992366	
1516	-0.6333698	-0.0143414	0.00020574	1.00081929	0.01575919	2238063.39	0.00050479	0.05047863	
1517	-0.9310865	-0.0209662	0.00043962	1.00058192	0.01575537	2237921.25	0.00050555	0.05055461	
1518	-0.9292083	-0.0209148	0.00043746	1.00058317	0.0157554	2237960.93	0.00050507	0.05050712	
1519	-1.0268948	-0.0231198	0.00053451	1.00048699	0.01575383	2237457.94	0.00050694	0.05069368	
1520	-0.9927934	-0.0223464	0.00049937	1.00052163	0.0157544	2237802.38	0.00050615	0.05061452	
1521	-1.3327277	-0.0296441	0.00087844	1.00011361	0.01574797	2238034.61	0.00049893	0.04989321	
1522	-0.9607862	-0.0216535	0.0004689	1.00055458	0.01575491	2237555.23	0.00050691	0.05069101	
1523	-0.7199065	-0.0162958	0.00026562	1.00076226	0.01575825	2237874.2	0.00050665	0.05066491	
1524	-1.1241851	-0.0252348	0.00063671	1.0003781	0.01575211	2237849.9	0.00050537	0.0505366	
1525	-1.4130179	-0.0316066	0.00099846	1.00000179	0.01574606	2237651.95	0.00050556	0.05055558	
1526	-0.879689	-0.0197568	0.00039038	1.00062527	0.01575612	2238068.17	0.000502	0.05020007	
1527	-0.7166027	-0.0162244	0.0002633	1.0007649	0.01575829	2237842.11	0.00050678	0.05067828	

© 2024 Zhang X et al. *JAMA Pediatrics*.

1528	-1.4099114	-0.0315465	0.00099468	1.0000063	0.01574613	2237588.42	0.00050582	0.05058235	
1529	-0.6812635	-0.0154256	0.00023802	1.00078906	0.01575869	2237985.73	0.00050602	0.05060212	
1530	-0.6506915	-0.0147449	0.00021748	1.00080991	0.01575902	2237998.5	0.00050602	0.05060212	
1531	-0.3082665	-0.0070946	5.04E-05	1.00097656	0.0157617	2238113.44	0.00050563	0.05056348	
1532	-1.0400604	-0.0232125	0.0005388	1.00046505	0.01575361	2238081.27	0.00049836	0.0498355	
1533	-1.1067932	-0.0247314	0.00061158	1.00039424	0.01575244	2238050.53	0.00050053	0.0500526	
1534	-1.1815653	-0.026383	0.00069593	1.00030744	0.01575104	2238032.87	0.00050084	0.05008376	
1535	0.54739064	0.01208032	0.00014599	1.0008484	0.01575979	2238139.02	0.00049655	0.04965462	
1536	-0.2504768	-0.0057129	3.27E-05	1.00096394	0.01576174	2238145.41	0.00049089	0.04908902	
1537	-1.1193391	-0.0251493	0.00063241	1.00038433	0.01575219	2237692.77	0.00050623	0.05062324	
1538	-0.8531449	-0.0192671	0.00037128	1.00065521	0.01575652	2237560.23	0.00050709	0.0507086	
1539	-1.1125452	-0.0250211	0.00062598	1.00039281	0.01575231	2237135.84	0.00050713	0.0507126	
1540	-1.1035719	-0.0247951	0.00061473	1.00040217	0.01575248	2237758.44	0.00050601	0.05060104	
1541	-0.7160421	-0.016171	0.00026157	1.00076149	0.01575827	2238058.61	0.00050424	0.05042428	
1542	-0.3013555	-0.006932	4.81E-05	1.00097652	0.01576172	2238126.15	0.00050451	0.05045135	
1543	-0.5138988	-0.0116131	0.00013492	1.0008791	0.01576024	2238128.16	0.00049905	0.04990487	
1544	-0.9356892	-0.0210965	0.00044509	1.00057905	0.0157553	2237588.44	0.0005069	0.05069017	
1545	-1.3152591	-0.0294765	0.00086854	1.00013902	0.01574825	2237505.17	0.0005063	0.05063003	
1546	-1.4516489	-0.0324678	0.00105356	0.99994488	0.01574515	2237524.64	0.00050593	0.05059272	
1547	-0.299837	-0.0068983	4.76E-05	1.00097705	0.01576173	2238126.08	0.00050455	0.05045502	
1548	-0.862009	-0.0193555	0.00037469	1.00064026	0.01575637	2238077.69	0.00050146	0.05014592	
1549	-1.6029365	-0.0357911	0.00127996	0.99970723	0.01574133	2237384.82	0.00050595	0.05059546	
1550	-1.1033262	-0.0245109	0.00060072	1.00039354	0.01575252	2238094.49	0.00049468	0.04946829	
1551	0.01930987	0.00025663	6.59E-08	1.0010069	0.01576232	2238148.53	0.00049767	0.04976725	
1552	1.34582015	0.03044002	0.00092619	1.00007608	0.01574724	2237599.92	0.00050637	0.05063699	
1553	-0.3784008	-0.0086178	7.43E-05	1.0009423	0.01576124	2238134.26	0.00050037	0.05003699	
1554	1.12776843	0.02512406	0.00063113	1.00034822	0.01575181	2238108.45	0.00049408	0.04940759	
1555	0.50117309	0.01111071	0.0001235	1.00088449	0.01576027	2238130.95	0.00050285	0.05028542	
1556	0.98514392	0.02218144	0.00049202	1.00051464	0.01575427	2237741.82	0.00050683	0.05068289	
1557	0.45667085	0.01012133	0.00010248	1.00090978	0.01576065	2238127.46	0.00050451	0.05045149	
1558	-0.1954339	-0.0045726	2.09E-05	1.00100763	0.01576218	2238106.26	0.00050691	0.05069121	
1559	0.44015742	0.00969155	9.40E-05	1.00090686	0.0157607	2238141.69	0.00049864	0.04986368	
1560	1.03660291	0.0232393	0.00054004	1.00045558	0.01575341	2238074.1	0.00050166	0.050166	
1561	1.15915727	0.02611902	0.00068207	1.0003187	0.01575116	2237943.19	0.00050499	0.05049857	
1562	0.64182253	0.01430847	0.00020479	1.00080227	0.01575893	2238108.88	0.00050387	0.05038681	
1563	-0.3790542	-0.0086908	7.56E-05	1.00095462	0.01576133	2238008.61	0.00050707	0.05070673	
1564	-0.5602606	-0.0126984	0.00016131	1.00086177	0.01575989	2238099.27	0.00050359	0.05035912	
1565	0.5474036	0.01217007	0.00014816	1.00086115	0.01575988	2238120.24	0.000504	0.05040044	
1566	-0.5411481	-0.01223	0.00014963	1.00086644	0.01576002	2238123.19	0.0005001	0.05000969	
1567	-0.3419755	-0.0077941	6.08E-05	1.00095229	0.01576142	2238138.69	0.00049871	0.04987099	
1568	0.25335604	0.00549486	3.02E-05	1.00097401	0.01576178	2238147.16	0.00049841	0.04984106	
1569	0.83764105	0.01880216	0.00035357	1.00065454	0.01575653	2237959.89	0.00050631	0.0506314	

© 2024 Zhang X et al. *JAMA Pediatrics.*

1570	0.97968331	0.02200655	0.00048429	1.00051788	0.01575436	2238025.49	0.00050453	0.0504534	
1571	0.21753969	0.00446228	1.99E-05	1.00088585	0.01576115	2238148.56	0.00044918	0.04491771	
1572	0.62190629	0.01386322	0.00019225	1.00081617	0.01575915	2238106.71	0.00050434	0.05043383	
1573	0.86015321	0.01933344	0.00037383	1.00063574	0.01575621	2237635.12	0.00050725	0.05072469	
1574	0.99977178	0.02244124	0.00050361	1.00049614	0.01575403	2238051.26	0.00050347	0.05034689	
1575	-1.3809058	-0.0308833	0.00095334	1.00004784	0.01574681	2237765.54	0.000505	0.05050038	
1576	-0.3864997	-0.008804	7.75E-05	1.00094046	0.0157612	2238132.24	0.00050105	0.05010502	
1577	-0.52114	-0.0114717	0.00013164	1.00083161	0.01575988	2238143.29	0.00047403	0.04740347	
1578	-0.5120766	-0.0114711	0.00013163	1.00086487	0.01576015	2238138.44	0.00049041	0.04904136	
1579	-0.6474672	-0.0146319	0.00021416	1.00080755	0.01575903	2238090.25	0.00050321	0.05032072	
1580	-0.275966	-0.0063567	4.04E-05	1.00098165	0.01576182	2238134.11	0.00050336	0.05033594	
1581	-1.0699432	-0.0240674	0.0005792	1.00044031	0.01575308	2237565.03	0.00050669	0.05066859	
1582	-0.5224048	-0.0117003	0.00013695	1.00085991	0.01576007	2238137.96	0.0004906	0.04906	
1583	0.8794382	0.01974514	0.00038991	1.00061637	0.01575592	2237993.61	0.00050577	0.05057695	
1584	1.1582554	0.02523546	0.00063672	1.00029875	0.01575136	2238132.17	0.00047224	0.04722425	
1585	-0.1857871	-0.0043397	1.88E-05	1.00100224	0.01576215	2238140.68	0.00050328	0.05032781	
1586	-0.091423	-0.0021922	4.81E-06	1.00098612	0.01576212	2238147.92	0.00048894	0.04889419	
1587	-0.2907665	-0.0067046	4.50E-05	1.00098236	0.01576179	2238112.11	0.0005059	0.05059004	
1588	-0.2397455	-0.0054719	3.00E-05	1.00096511	0.01576177	2238145.75	0.00049018	0.04901844	
1589	-0.5910494	-0.0134251	0.00018029	1.00084894	0.01575964	2237944.77	0.00050671	0.050671	
1590	0.41437656	0.00913189	8.34E-05	1.00092243	0.01576091	2238140.73	0.00050087	0.0500874	
1591	-0.3372274	-0.0076882	5.91E-05	1.00095382	0.01576145	2238138.96	0.00049867	0.04986683	
1592	0.86305225	0.01937263	0.00037535	1.00063135	0.01575616	2237995.3	0.00050583	0.05058321	
1593	1.4097998	0.03159635	0.00099782	0.99998453	0.01574593	2238070.08	0.00049648	0.04964825	
1594	-0.0395505	-0.0010671	1.14E-06	1.00102378	0.01576245	2238144.31	0.0005061	0.05061012	
1595	-0.3086069	-0.0071119	5.06E-05	1.00097904	0.01576172	2238058.55	0.00050698	0.05069769	
1596	-0.1080945	-0.0026056	6.79E-06	1.00101741	0.01576236	2238141.48	0.00050527	0.05052728	
1597	0.27463849	0.00599257	3.59E-05	1.00097493	0.01576174	2238145.58	0.00050192	0.05019234	
1598	-1.5985872	-0.0356748	0.00127167	0.99971474	0.01574146	2237570.98	0.00050536	0.05053591	
1599	-1.2280259	-0.0272561	0.00074271	1.00024783	0.01575017	2238078.38	0.00049545	0.04954519	
1600	1.80991702	0.04103947	0.00168222	0.99931858	0.01573513	2237780.71	0.00050374	0.05037364	
1601	0.16344933	0.0034536	1.19E-05	1.00097782	0.01576197	2238148.54	0.00049038	0.04903758	
1602	1.43729384	0.03247495	0.00105402	0.99994384	0.01574516	2237891.7	0.00050422	0.0504225	
1603	1.10565011	0.02488161	0.00061902	1.00038129	0.01575217	2237992.12	0.00050446	0.05044561	
1604	0.02816746	0.00045073	2.03E-07	1.00098097	0.0157621	2238148.66	0.00048497	0.04849725	
1605	-0.423699	-0.0096398	9.30E-05	1.00092682	0.01576096	2238126.85	0.00050188	0.05018796	
1606	-0.4250034	-0.0096309	9.28E-05	1.00091913	0.0157609	2238135.41	0.00049801	0.04980076	
1607	-0.1286737	-0.0030728	9.45E-06	1.00101838	0.01576235	2238123.56	0.00050693	0.0506934	
1608	0.07493596	0.00150309	2.26E-06	1.00101009	0.01576232	2238148.72	0.00050079	0.05007947	
1609	0.43490647	0.00964549	9.31E-05	1.0009232	0.01576084	2238108.45	0.00050629	0.05062862	
1610	-0.9268524	-0.0208745	0.00043578	1.00058608	0.01575544	2237912.46	0.00050565	0.05056498	
1611	0.66450743	0.01480652	0.0002193	1.00078504	0.01575868	2238115.15	0.00050273	0.0502734	

© 2024 Zhang X et al. *JAMA Pediatrics.*

1612	0.26856494	0.00588469	3.46E-05	1.00098659	0.01576184	2238127.58	0.00050702	0.0507021	
1613	-1.2188285	-0.0271977	0.00073954	1.00026185	0.01575032	2238028.4	0.00050069	0.05006947	
1614	0.20295905	0.00439859	1.94E-05	1.00100183	0.0157621	2238143.89	0.00050635	0.05063464	
1615	-1.2087562	-0.0269644	0.00072691	1.00027405	0.01575052	2238037.3	0.00050024	0.0500243	
1616	-0.9430625	-0.0211914	0.00044911	1.00056827	0.01575518	2238021.63	0.00050364	0.05036356	
1617	-0.8430853	-0.0189965	0.00036093	1.00066075	0.01575665	2238014.55	0.0005046	0.05045954	
1618	-1.9584503	-0.0433144	0.00187344	0.99906726	0.01573116	2237877.05	0.00050007	0.0500067	
1619	-1.1610496	-0.0257802	0.00066451	1.00032806	0.01575147	2238087.72	0.00049498	0.04949771	
1620	-0.8082521	-0.0181757	0.00033042	1.00068655	0.01575711	2238080.43	0.00050194	0.05019364	
1621	-0.1511574	-0.0035685	1.27E-05	1.0010101	0.01576226	2238140.77	0.00050434	0.05043352	
1622	-1.4975567	-0.0334145	0.00111582	0.99987575	0.01574407	2237815.61	0.00050404	0.0504037	
1623	-0.3633898	-0.0083368	6.95E-05	1.0009598	0.01576142	2238061.35	0.00050668	0.05066821	
1624	-1.1791465	-0.0264899	0.00070158	1.00031408	0.01575105	2237238.4	0.00050695	0.0506954	
1625	-1.3322526	-0.0298316	0.00088957	1.00011573	0.01574789	2237687.04	0.00050564	0.05056402	
1626	0.05164845	0.0009851	9.71E-07	1.00102147	0.01576242	2238148.5	0.0005056	0.05055966	
1627	0.35672713	0.00782604	6.13E-05	1.00094386	0.01576127	2238143.93	0.00049991	0.0499914	
1628	0.68014933	0.01519511	0.00023096	1.0007776	0.01575852	2238084.95	0.00050501	0.05050078	
1629	0.25451829	0.00556045	3.09E-05	1.00098812	0.01576189	2238141.47	0.00050579	0.05057948	
1630	-0.6926428	-0.015636	0.00024455	1.00077682	0.01575854	2238081.88	0.00050327	0.05032704	
1631	-1.3144359	-0.0290563	0.00084397	1.00013645	0.01574843	2238084.06	0.00049253	0.04925328	
1632	-1.3493672	-0.0302096	0.00091223	1.00009215	0.01574751	2237667.93	0.00050568	0.05056764	
1633	0.76760994	0.01719045	0.00029557	1.00071204	0.01575746	2238051.19	0.00050541	0.05054065	
1634	-0.3794128	-0.0086299	7.45E-05	1.0009397	0.01576121	2238136.18	0.00049919	0.04991904	
1635	-1.6226726	-0.0361443	0.00130531	0.99967595	0.01574087	2237790.74	0.00050372	0.05037196	
1636	0.23424542	0.00509681	2.60E-05	1.00099108	0.01576195	2238145.15	0.00050461	0.0504611	
1637	-0.8371507	-0.018835	0.00035481	1.00066375	0.01575673	2238059.96	0.000503	0.05029982	
1638	-1.2621166	-0.0281235	0.0007907	1.00020687	0.01574945	2238034.19	0.00049983	0.04998273	
1639	0.48558541	0.01037029	0.00010758	1.00082849	0.01575995	2238146.69	0.0004668	0.04668036	
1640	-0.5002766	-0.0112172	0.00012587	1.00087162	0.01576026	2238138.57	0.00049089	0.04908902	
1641	0.1724195	0.00369778	1.37E-05	1.00100158	0.01576214	2238147.87	0.00050311	0.0503112	
1642	-0.2994937	-0.0068806	4.74E-05	1.00097436	0.01576171	2238133.03	0.0005031	0.05031036	
1643	-0.2345736	-0.0054222	2.94E-05	1.00098898	0.01576196	2238140.37	0.00050171	0.05017117	
1644	-0.4672881	-0.0106655	0.0001138	1.0009166	0.01576072	2237942.23	0.0005071	0.05070967	
1645	-0.325971	-0.0075024	5.63E-05	1.00097379	0.01576163	2238025.62	0.00050715	0.05071511	
1646	-1.4472293	-0.032377	0.00104768	0.99995145	0.01574525	2237455.92	0.00050613	0.05061271	
1647	2.32251057	0.05243196	0.00274301	0.99825871	0.01571828	2237956.91	0.0004946	0.04945964	
1648	-0.9193805	-0.0206324	0.00042573	1.00058882	0.01575554	2238062.47	0.00050193	0.05019278	
1649	1.12503161	0.02531704	0.00064086	1.00035861	0.01575181	2237999.59	0.00050415	0.05041504	
1650	1.13365243	0.02517815	0.00063384	1.00033929	0.01575171	2238115.43	0.000491	0.04909965	
1651	0.74493057	0.01647134	0.00027136	1.00071216	0.01575766	2238133.59	0.00049312	0.04931202	
1652	-0.3403362	-0.0076511	5.86E-05	1.0009274	0.01576124	2238144.7	0.00048536	0.04853554	
1653	-0.1082617	-0.002612	6.83E-06	1.00101932	0.01576238	2238137.13	0.00050623	0.05062334	

© 2024 Zhang X et al. *JAMA Pediatrics.*

1654	-0.3850312	-0.0088104	7.77E-05	1.00094927	0.01576127	2238102.15	0.00050545	0.05054506	
1655	0.9613805	0.02146449	0.00046074	1.00053015	0.01575465	2238105.74	0.00049873	0.04987305	
1656	2.29177157	0.05151015	0.00264769	0.99834378	0.0157197	2238005.33	0.0004906	0.04906	
1657	0.71818065	0.01587984	0.00025223	1.00073301	0.01575799	2238134.21	0.00049371	0.04937139	
1658	0.0211591	0.00029884	8.94E-08	1.00102205	0.01576244	2238147.98	0.0005052	0.0505196	
1659	0.23632473	0.00513105	2.63E-05	1.00098553	0.0157619	2238146.67	0.00050205	0.05020469	
1660	-1.0644223	-0.0238336	0.00056801	1.00044227	0.01575319	2238034.81	0.00050198	0.05019751	
1661	0.77374179	0.0172661	0.00029818	1.00070186	0.01575736	2238109.51	0.00050167	0.05016695	
1662	0.30406666	0.00668798	4.48E-05	1.00097575	0.01576167	2238118.74	0.00050702	0.05070195	
1663	-1.4457252	-0.0323378	0.00104515	0.99995371	0.01574529	2237526.2	0.00050594	0.05059372	
1664	-0.736618	-0.0166801	0.0002783	1.00075088	0.01575805	2237378.71	0.0005074	0.05073993	
1665	-0.7187638	-0.0162801	0.00026511	1.00076402	0.01575827	2237590.67	0.00050726	0.05072572	
1666	-0.4229176	-0.0095257	9.08E-05	1.00090894	0.01576084	2238140.55	0.00049199	0.04919907	
1667	-1.7937534	-0.0399578	0.00159479	0.99937433	0.015736	2237336.89	0.00050564	0.0505642	
1668	-1.6432173	-0.0366796	0.00134421	0.99963988	0.01574025	2237280	0.0005061	0.05061023	
1669	1.0491725	0.02366334	0.00055992	1.00044726	0.01575318	2236556.89	0.00050755	0.05075457	
1670	0.67680726	0.01498963	0.00022475	1.00076655	0.01575848	2238133.15	0.00049634	0.04963365	
1671	0.99353307	0.02229085	0.00049688	1.00050223	0.01575413	2238060.04	0.0005031	0.05031008	
1672	0.67370159	0.01496906	0.00022413	1.00077384	0.01575855	2238127.11	0.00049965	0.04996479	
1673	1.4485304	0.03246123	0.00105314	0.99992814	0.01574504	2238069.18	0.00049595	0.04959458	
1674	0.58243247	0.01281481	0.00016427	1.00082106	0.01575942	2238140.51	0.00049239	0.0492386	
1675	0.41176022	0.00887946	7.89E-05	1.00088395	0.01576064	2238146.78	0.0004794	0.04794036	
1676	0.04993943	0.00094399	8.92E-07	1.0010146	0.01576237	2238148.63	0.00050214	0.05021412	
1677	0.20911406	0.00452782	2.05E-05	1.00099599	0.01576204	2238146.59	0.00050408	0.05040832	
1678	0.58249305	0.01299255	0.00016886	1.00084402	0.01575957	2238073.66	0.00050616	0.05061587	
1679	-0.1376827	-0.0032504	1.06E-05	1.0010026	0.01576222	2238145.71	0.00049964	0.04996396	
1680	-0.6592647	-0.0143111	0.00020486	1.00073898	0.01575855	2238142.28	0.00046483	0.04648323	
1681	-0.5246503	-0.0119416	0.00014266	1.00088663	0.01576024	2237998.94	0.00050659	0.05065891	
1682	0.54925634	0.01209295	0.00014629	1.0008432	0.01575975	2238140.67	0.00049412	0.04941228	
1683	-0.4929092	-0.0110278	0.00012166	1.00087097	0.01576028	2238140.06	0.00048849	0.0488488	
1684	-0.5218042	-0.0118856	0.00014132	1.00088928	0.01576028	2237843.74	0.00050723	0.05072327	
1685	-0.0596028	-0.0015186	2.31E-06	1.00102357	0.01576244	2238141.37	0.00050643	0.05064294	
1686	-0.5422468	-0.012297	0.00015127	1.0008718	0.01576006	2238102.42	0.00050355	0.05035527	
1687	-0.311768	-0.0070558	4.98E-05	1.00094458	0.01576145	2238144.41	0.00048958	0.04895843	
1688	1.19375213	0.02697131	0.00072728	1.00027765	0.01575046	2237115.7	0.00050724	0.05072375	
1689	-0.340833	-0.0078372	6.15E-05	1.00096919	0.01576156	2237952.88	0.00050738	0.05073759	
1690	-0.3822269	-0.0087185	7.60E-05	1.00094415	0.01576124	2238129.45	0.00050214	0.05021372	
1691	-0.5007764	-0.0114145	0.00013034	1.00090009	0.01576045	2237907.3	0.00050712	0.05071247	
1692	-0.1220832	-0.0029213	8.54E-06	1.00101694	0.01576235	2238138.14	0.00050582	0.05058169	
1693	-0.5205588	-0.0118102	0.00013953	1.00088292	0.01576024	2238108.75	0.00050321	0.05032125	
1694	-0.0045229	-0.0002784	7.76E-08	1.0010213	0.01576244	2238147.51	0.00050462	0.05046174	
1695	-0.5440705	-0.0123631	0.0001529	1.00087435	0.01576006	2238058.97	0.00050562	0.05056153	

© 2024 Zhang X et al. *JAMA Pediatrics*.

1696	-0.9685778	-0.0217655	0.00047376	1.00054383	0.01575478	2237998.92	0.00050409	0.05040937	
1697	-0.6011734	-0.0136319	0.00018589	1.00084045	0.01575952	2238054.63	0.00050531	0.05053139	
1698	-0.3695876	-0.0084577	7.16E-05	1.00095354	0.01576135	2238116.79	0.00050458	0.05045809	
1699	-0.1997962	-0.0045869	2.11E-05	1.00097235	0.01576189	2238146.52	0.00048954	0.04895428	
1700	-0.7369154	-0.0160298	0.00025701	1.00069325	0.01575775	2238140.13	0.00046841	0.04684101	
1701	-0.2577391	-0.0049875	2.49E-05	1.00069995	0.01575965	2238148.37	0.00035684	0.03568397	
1702	-0.309048	-0.0071198	5.07E-05	1.00097838	0.01576171	2238079.82	0.00050671	0.05067072	
1703	-0.7945514	-0.0162575	0.00026434	1.00057833	0.01575676	2238144.56	0.0004156	0.04156034	
1704	-0.6696249	-0.0139206	0.00019382	1.00067275	0.0157581	2238145.24	0.00042674	0.04267424	
1705	-0.4983928	-0.0113167	0.00012812	1.00089436	0.01576042	2238111.69	0.0005032	0.05032046	
1706	1.27378263	0.02643939	0.00069887	1.00014636	0.01574964	2238139.67	0.00042755	0.04275505	
1707	-0.1459298	-0.0034631	1.20E-05	1.00101739	0.01576233	2238037.06	0.0005076	0.05076033	
1708	2.01776809	0.04590022	0.00210342	0.99890566	0.01572849	2237507.48	0.00050489	0.05048861	
1709	1.50171148	0.03397794	0.00115373	0.99984562	0.01574357	2237803.65	0.00050489	0.05048861	
1710	-0.8295053	-0.0181618	0.0003299	1.00063486	0.01575669	2238135.39	0.00047629	0.04762853	
1711	-0.1562041	-0.0035243	1.24E-05	1.00092856	0.0157616	2238148.08	0.00046389	0.04638941	
1712	-0.2201606	-0.0051224	2.63E-05	1.00100053	0.01576208	2238124.42	0.00050592	0.05059167	
1713	-0.7543435	-0.0170528	0.00029086	1.00073541	0.01575783	2237945.13	0.00050609	0.05060925	
1714	-1.3153718	-0.0294054	0.00086436	1.0001382	0.01574828	2237905.15	0.00050377	0.05037675	
1715	-1.1719756	-0.0262745	0.00069022	1.00032139	0.01575121	2237889.05	0.00050476	0.05047629	
1716	-0.7575061	-0.017106	0.00029268	1.00073151	0.01575778	2238018.35	0.00050509	0.05050892	
1717	-0.4685182	-0.0106596	0.00011367	1.00091038	0.01576067	2238108.81	0.00050397	0.05039693	
1718	-0.0471033	-0.0012374	1.53E-06	1.00102407	0.01576245	2238142.78	0.00050639	0.05063862	
1719	-0.1772644	-0.0040385	1.63E-05	1.00095309	0.01576177	2238147.6	0.00047781	0.0477814	
1720	0.83405147	0.01867532	0.00034882	1.0006545	0.01575656	2238076.57	0.00050388	0.05038778	
1721	-0.4892354	-0.0111614	0.00012463	1.00090671	0.01576055	2237548.36	0.00050757	0.05075654	
1722	0.10179605	0.00208729	4.36E-06	1.00098885	0.01576213	2238148.72	0.00049154	0.04915418	
1723	-0.1537541	-0.0036338	1.32E-05	1.00101349	0.01576229	2238131.01	0.00050623	0.05062298	
1724	-0.1637665	-0.0038534	1.49E-05	1.0010093	0.01576224	2238138.13	0.00050492	0.05049162	
1725	0.5414119	0.01191786	0.00014209	1.00084771	0.01575982	2238140.89	0.00049419	0.04941925	
1726	1.15786663	0.02603585	0.00067774	1.00031892	0.0157512	2238029.72	0.00050292	0.05029183	
1727	0.69420079	0.01554678	0.00024177	1.00077074	0.01575838	2237883.32	0.00050713	0.05071296	
1728	0.64606467	0.01446089	0.00020918	1.00080573	0.01575893	2216333.26	0.00050784	0.05078356	
1729	0.68210182	0.01526459	0.00023307	1.00077879	0.01575851	2237991.71	0.00050668	0.05066844	
1730	0.79557392	0.01786708	0.00031929	1.00069233	0.01575711	2236531	0.00050768	0.05076839	
1731	0.61587183	0.01372393	0.00018841	1.00081978	0.01575921	2238109.62	0.00050416	0.05041605	
1732	0.06739736	0.00133322	1.78E-06	1.00100911	0.01576231	2238148.71	0.00050001	0.05000065	
1733	0.62088809	0.01385474	0.00019201	1.00081857	0.01575917	2238088.37	0.00050541	0.05054131	
1734	-0.4073998	-0.0091144	8.31E-05	1.0009018	0.01576084	2238143.48	0.00048471	0.04847099	
1735	-0.7379707	-0.0161831	0.00026195	1.00070373	0.0157578	2238138.02	0.00047604	0.04760362	
1736	0.64248599	0.01406945	0.000198	1.00077338	0.01575876	2238141.41	0.00048603	0.04860251	
1737	1.24909249	0.02822324	0.00079631	1.00020683	0.01574934	2237611.79	0.00050656	0.05065591	

© 2024 Zhang X et al. *JAMA Pediatrics*.

1738	1.12398865	0.02535817	0.00064294	1.0003617	0.01575182	2237651.94	0.00050674	0.05067371	
1739	-0.675598	-0.0152896	0.00023384	1.00079199	0.01575875	2238030.23	0.00050537	0.05053744	
1740	0.84749878	0.01905379	0.0003631	1.00064769	0.01575639	2235150.01	0.00050774	0.05077441	
1741	0.6682848	0.01496608	0.00022405	1.00079027	0.01575868	2235705.95	0.00050777	0.05077719	
1742	0.0889323	0.00182644	3.34E-06	1.00102065	0.01576239	2238148.72	0.00050668	0.05066791	
1743	2.27723863	0.05166775	0.00266379	0.99834662	0.0157196	2237842.41	0.00049988	0.04998827	
1744	0.58767164	0.01300712	0.00016924	1.00082753	0.01575943	2238135.36	0.00049816	0.04981612	
1745	2.12436732	0.04849582	0.00234745	0.9986711	0.01572469	2233662.04	0.00050737	0.05073727	
1746	0.9490107	0.0213763	0.00045697	1.00055225	0.01575486	2210903.92	0.00050783	0.0507829	
1747	0.45420394	0.01009803	0.00010201	1.00091695	0.01576071	2233512.63	0.00050783	0.05078279	
1748	2.15516753	0.04604286	0.00211678	0.99877279	0.01572732	2238113.91	0.00044506	0.0445064	
1749	1.21956171	0.02757973	0.00076044	1.00024543	0.01574994	2211959.66	0.00050782	0.05078179	
1750	1.68452159	0.03823291	0.00146033	0.99954287	0.01573869	2237444.77	0.000506	0.05059966	
1751	0.27131504	0.00544144	2.96E-05	1.00082139	0.01576055	2238148.5	0.00042245	0.04224454	
1752	2.15597314	0.04915519	0.00241157	0.99860516	0.01572365	2237076.14	0.00050581	0.05058144	
1753	1.44472307	0.03226304	0.00104033	0.99993415	0.01574519	2238087.11	0.00049256	0.04925618	
1754	1.49336379	0.03356598	0.00112596	0.99986031	0.01574391	2238041.99	0.00049839	0.04983884	
1755	1.57024842	0.03562193	0.00126793	0.99973556	0.01574177	2237126.16	0.00050675	0.05067467	
1756	0.9799009	0.02208336	0.00048768	1.00052106	0.01575436	2199048.08	0.00050783	0.05078325	
1757	1.06188	0.02386798	0.00056964	1.00043009	0.01575296	2238025.73	0.00050389	0.05038915	
1758	1.33675606	0.03023975	0.00091405	1.00008879	0.01574744	2237486.53	0.00050664	0.05066396	
1759	0.70695741	0.01579169	0.00024944	1.00075695	0.0157582	2238094.62	0.00050419	0.05041939	
1760	1.52640524	0.03464787	0.00119962	0.99980577	0.01574288	2114249.92	0.00050783	0.05078344	
1761	1.39780001	0.03166089	0.0010019	1.00000189	0.01574604	2236832.13	0.00050718	0.05071774	
1762	0.91472238	0.02058845	0.00042392	1.00058552	0.0157554	2236262.41	0.00050766	0.0507657	
1763	1.28381756	0.02902735	0.00084229	1.00016109	0.0157486	2237455.16	0.00050679	0.0506789	
1764	1.73967605	0.03842703	0.00147521	0.99947402	0.01573802	2238100.38	0.000479	0.04789964	
1765	1.3456907	0.03046178	0.00092751	1.00007639	0.01574724	2236914.1	0.00050719	0.05071884	
1766	1.66012875	0.03761758	0.00141377	0.99958631	0.01573941	2237757.88	0.00050462	0.05046174	
1767	0.41582101	0.00900271	8.11E-05	1.00088903	0.01576066	2238146.44	0.00048307	0.04830747	
1768	1.80829092	0.04071858	0.00165608	0.99933072	0.01573544	2238010.98	0.00049688	0.04968841	
1769	1.55320392	0.03514717	0.0012344	0.99976429	0.01574227	2237827.92	0.00050443	0.05044274	
1770	1.79717179	0.04015474	0.00161062	0.99936074	0.01573604	2238067.79	0.00048942	0.04894173	
1771	1.86255998	0.04152843	0.00172252	0.99924478	0.01573422	2238072.46	0.00048677	0.04867669	
1772	1.5060077	0.03358594	0.0011273	0.99984329	0.01574376	2238089.22	0.00049056	0.04905631	
1773	1.12360611	0.02537672	0.00064388	1.00036293	0.01575182	2203995.17	0.00050783	0.05078269	
1774	0.8437425	0.01893435	0.00035856	1.00064869	0.01575644	2237994.5	0.00050594	0.05059403	
1775	1.3112505	0.02965881	0.0008793	1.000124	0.01574801	2237399.11	0.00050682	0.05068228	
1776	1.34777372	0.03045156	0.00092689	1.00007317	0.01574721	2237833.68	0.00050527	0.05052687	
1777	0.78650954	0.01765998	0.00031194	1.00069984	0.01575723	2236693	0.00050767	0.05076706	
1778	1.37419016	0.02948402	0.00086897	1.00003172	0.01574735	2238132.43	0.00045564	0.04556378	
1779	1.46119544	0.03265921	0.00106601	0.99990982	0.01574479	2238082.75	0.00049323	0.0493227	

© 2024 Zhang X et al. *JAMA Pediatrics*.

1780	0.81358977	0.01826504	0.00033367	1.00067614	0.01575686	2237847.64	0.00050694	0.05069443	
1781	1.02324225	0.02296998	0.0005276	1.00047124	0.01575363	2238052.43	0.00050319	0.05031858	
1782	2.23827868	0.04854944	0.00235295	0.99856671	0.01572381	2238101.44	0.0004579	0.04579024	
1783	0.17413962	0.00366737	1.35E-05	1.00096288	0.01576183	2238148.55	0.00048381	0.04838132	
1784	0.11290067	0.00236938	5.62E-06	1.00102014	0.01576237	2238142.69	0.00050777	0.05077708	
1785	1.35203978	0.03041067	0.00092441	1.00006653	0.01574718	2238034.13	0.00050071	0.05007137	
1786	1.05202328	0.023261	0.00054104	1.00042666	0.01575317	2238124.94	0.00048776	0.04877646	
1787	1.22971843	0.02780963	0.00077316	1.00023238	0.01574973	2233771.59	0.00050769	0.05076907	
1788	0.45797347	0.010117	0.0001024	1.00090302	0.0157606	2238138.12	0.00050111	0.05011126	
1789	1.801043	0.04078553	0.0016615	0.99933668	0.01573544	2237866.49	0.00050254	0.05025425	
1790	2.23952615	0.0507609	0.00257135	0.99843566	0.01572104	2237875.02	0.00049924	0.04992366	
1791	1.60477837	0.03618869	0.00130855	0.99968246	0.01574102	2237990.56	0.00050042	0.05004181	
1792	0.27863617	0.00603319	3.64E-05	1.0009576	0.01576159	2238147.36	0.00049359	0.04935889	
1793	0.4425079	0.00981057	9.63E-05	1.00091832	0.01576077	2238120.4	0.00050553	0.05055316	
1794	0.80011323	0.01791251	0.00032092	1.00068405	0.01575703	2238073.17	0.00050439	0.05043908	
1795	-0.0868485	-0.0020994	4.41E-06	1.00099367	0.01576219	2238147.78	0.0004925	0.04925036	
1796	1.08985287	0.02435756	0.00059323	1.00039397	0.01575248	2238097.55	0.00049779	0.04977867	
1797	0.81097541	0.01776587	0.00031568	1.00064579	0.01575676	2238137.99	0.00048269	0.04826881	
1798	0.6979057	0.01499014	0.00022475	1.00070585	0.01575799	2238144.08	0.00046617	0.04661669	
1799	0.81708453	0.01766702	0.00031217	1.00062402	0.01575662	2238141.43	0.00047007	0.04700734	
1800	1.55427707	0.03524984	0.00124161	0.99976156	0.01574219	2237232.12	0.00050665	0.05066461	
1801	0.55484173	0.01237877	0.00015329	1.00086252	0.01575985	2237929.8	0.00050733	0.05073278	
1802	0.57227122	0.01272336	0.00016194	1.00084523	0.01575963	2238122.59	0.0005032	0.05031965	
1803	1.01835475	0.0228503	0.00052212	1.00047607	0.01575372	2238060.31	0.00050282	0.05028232	
1804	1.31911059	0.02938468	0.00086313	1.00010965	0.01574802	2238101.02	0.00049157	0.04915747	
1805	1.02459385	0.0230866	0.00053297	1.00047334	0.01575361	2237649.22	0.00050694	0.05069431	
1806	1.10017358	0.02482667	0.00061629	1.00038978	0.01575226	2237152.69	0.00050732	0.05073164	
1807	0.3011934	0.00652291	4.26E-05	1.00094668	0.01576145	2238147.24	0.00049144	0.04914422	
1808	-0.0436565	-0.0010455	1.09E-06	1.00085468	0.01576108	2238148.64	0.00042245	0.04224454	
1809	0.42110912	0.00926659	8.59E-05	1.00091607	0.01576084	2238142.15	0.00049901	0.04990102	
1810	1.11396935	0.02510064	0.00062996	1.00037256	0.01575201	2237906.86	0.00050561	0.05056147	
1811	0.67798909	0.01490929	0.00022234	1.00075476	0.01575841	2238139.03	0.00048925	0.04892472	
1812	1.9782006	0.04436474	0.00196537	0.99901432	0.01573045	2238038.55	0.00049131	0.04913076	
1813	0.75817588	0.01661312	0.00027605	1.00068893	0.01575744	2238139	0.00048396	0.04839557	
1814	1.74801422	0.03939598	0.0015504	0.99943827	0.01573714	2238000.56	0.00049836	0.0498355	
1815	1.64092154	0.03729395	0.00138958	0.99961676	0.01573985	2163135.85	0.00050783	0.05078265	
1816	1.27893113	0.02891468	0.00083577	1.00016761	0.01574871	2237469.7	0.00050678	0.05067752	
1817	1.27529726	0.0288614	0.0008327	1.00017282	0.01574877	2171309.55	0.00050783	0.05078321	
1818	2.90392726	0.06382809	0.00406057	0.99692176	0.0156973	2238054.94	0.00046483	0.04648323	
1819	1.83793339	0.04112929	0.00168961	0.99928482	0.0157348	2238058.49	0.00049053	0.04905259	
1820	0.80456231	0.01807346	0.00032671	1.00068488	0.01575699	2235226.46	0.00050775	0.05077526	
1821	0.39035954	0.00803137	6.45E-05	1.00081312	0.01576019	2238148.03	0.00043676	0.04367576	

© 2024 Zhang X et al. *JAMA Pediatrics*.

1822	3.06381969	0.06785811	0.00458715	0.99642686	0.01568927	2238027.91	0.0004706	0.04705971	*
1823	2.47806933	0.0553952	0.00306102	0.99792735	0.01571313	2238027.73	0.00048381	0.04838132	
1824	0.10084586	0.00208528	4.35E-06	1.00100898	0.01576229	2238148.7	0.0005015	0.05015044	
1825	1.17601009	0.02635229	0.0006943	1.00029478	0.01575087	2238078.5	0.00049921	0.04992089	
1826	0.37958007	0.0084021	7.06E-05	1.00094928	0.01576123	2238015.32	0.00050751	0.05075095	
1827	3.05042766	0.06524389	0.00424271	0.99669619	0.01569409	2238083.07	0.00043976	0.04397603	
1828	1.58914949	0.03535122	0.00124877	0.99971557	0.01574177	2238092.77	0.00048725	0.04872516	
1829	1.92534839	0.04335713	0.00187725	0.99911015	0.01573192	2238005.78	0.0004958	0.04958036	
1830	0.61023077	0.01364486	0.00018624	1.00082938	0.01575931	2231761.87	0.00050782	0.05078209	
1831	1.75563918	0.03967906	0.00157272	0.99942156	0.01573683	2237939.93	0.00050105	0.05010502	
1832	0.29721825	0.00643843	4.15E-05	1.00094914	0.01576148	2238147.24	0.00049208	0.04920837	
1833	0.12478717	0.00262531	6.90E-06	1.00100826	0.01576226	2238148.56	0.00050262	0.05026249	
1834	0.39363917	0.00870848	7.59E-05	1.00094079	0.01576112	2238121.6	0.00050604	0.0506042	
1835	1.29755584	0.02931776	0.00085921	1.00014236	0.01574831	2237759.73	0.00050592	0.05059204	
1836	0.48415025	0.01055626	0.00011148	1.00086486	0.01576021	2238144.81	0.00048688	0.04868766	
1837	1.51137381	0.03326313	0.00110576	0.99983923	0.0157439	2238114.17	0.00047781	0.0477814	
1838	0.4368211	0.00945751	8.95E-05	1.00087814	0.0157605	2238146.24	0.00048204	0.04820439	
1839	0.21531735	0.00459483	2.11E-05	1.0009627	0.01576176	2238148.28	0.00048791	0.04879136	
1840	0.43419469	0.00955862	9.14E-05	1.0009098	0.01576074	2238141.83	0.00049876	0.04987613	
1841	1.41008504	0.03195069	0.0010203	0.99998391	0.01574575	2236158.63	0.00050739	0.05073944	
1842	1.91489887	0.04296383	0.00184342	0.99913655	0.0157324	2238039.03	0.00049233	0.04923264	
1843	1.51929956	0.03416524	0.00116647	0.99982026	0.01574326	2238035.83	0.00049857	0.04985733	
1844	-0.0038589	-0.0002647	7.01E-08	1.00102675	0.01576248	2238141.42	0.00050731	0.05073114	
1845	0.34283164	0.00751606	5.65E-05	1.0009496	0.01576136	2238144.21	0.00050026	0.05002573	
1846	0.87037395	0.01956104	0.00038268	1.0006261	0.01575606	2237799	0.00050694	0.05069443	
1847	-0.106115	-0.0025014	6.26E-06	1.00097359	0.01576201	2238148	0.00048338	0.04833755	
1848	0.87207943	0.01953692	0.00038174	1.00062051	0.01575602	2238072.83	0.00050368	0.05036813	
1849	1.40671547	0.03182641	0.00101239	0.99998887	0.01574585	2237686.71	0.00050592	0.05059204	
1850	0.86514368	0.0192932	0.00037227	1.00062119	0.0157561	2238112.71	0.00049922	0.04992182	
1851	1.32107809	0.02968173	0.00088066	1.00010896	0.01574787	2238049.58	0.0005	0.04999989	
1852	1.68889431	0.0381783	0.00145617	0.99953887	0.0157387	2237927.21	0.00050194	0.05019408	
1853	1.34451184	0.03032444	0.00091918	1.00007744	0.01574731	2237961.02	0.00050354	0.05035436	
1854	1.48315191	0.03257069	0.00106025	0.99988112	0.0157446	2238117.37	0.00047604	0.04760362	
1855	0.79713699	0.01776611	0.00031569	1.00068045	0.01575704	2238115.98	0.00049994	0.04999374	
1856	1.25840319	0.02792413	0.00077953	1.00018755	0.01574932	2238112.36	0.00048853	0.04885343	
1857	1.57122406	0.03516037	0.00123533	0.99974087	0.01574208	2238071.95	0.0004932	0.04932004	
1858	0.816878	0.01835312	0.00033689	1.0006743	0.01575682	2236583.41	0.00050767	0.05076685	
1859	0.88682209	0.01989248	0.00039575	1.00060826	0.01575581	2238044.57	0.0005047	0.05046968	
1860	0.82636961	0.01822753	0.00033229	1.00064163	0.01575659	2238133.74	0.00048907	0.0489074	
1861	2.33859792	0.04945105	0.00244114	0.99843415	0.01572205	2238113.25	0.00043483	0.04348254	
1862	1.61868719	0.03666845	0.00134342	0.99965654	0.01574054	2237757.51	0.00050479	0.05047875	
1863	-0.2616628	-0.0060535	3.67E-05	1.00099073	0.01576192	2238115.22	0.00050604	0.05060375	

© 2024 Zhang X et al. *JAMA Pediatrics.*

1864	0.05560621	0.00107518	1.16E-06	1.00102345	0.01576244	2238148.38	0.0005067	0.05067023	
1865	1.80367493	0.0409413	0.00167419	0.99932876	0.01573528	2237646.56	0.00050486	0.05048554	
1866	2.02566285	0.04580192	0.00209447	0.99890235	0.01572853	2237936.47	0.00049883	0.04988324	
1867	1.48727346	0.03327007	0.00110621	0.99987074	0.01574415	2238077.63	0.00049376	0.04937632	
1868	2.82733279	0.06334334	0.00399891	0.99701769	0.01569855	2237994.76	0.00048307	0.04830747	
1869	1.87678418	0.04241937	0.00179705	0.99919744	0.01573325	2237943.63	0.00049989	0.04998905	
1870	2.36383413	0.05209576	0.00270826	0.9982463	0.01571845	2238076.2	0.00047144	0.04714384	
1871	1.22429876	0.0275073	0.00075645	1.00023621	0.0157499	2238048.98	0.00050122	0.05012177	
1872	1.73490353	0.03864374	0.00149186	0.99947362	0.01573789	2238082.13	0.00048704	0.0487039	
1873	1.63687158	0.03611825	0.00130348	0.99964458	0.01574076	2238106.18	0.000479	0.04789964	
1874	1.98593128	0.04502585	0.00202422	0.99897757	0.01572969	2237840.37	0.00050189	0.05018928	
1875	3.05480236	0.06692727	0.00446297	0.99652653	0.01569103	2238053.54	0.00046082	0.04608166	
1876	0.75373899	0.01688379	0.00028513	1.00072384	0.01575764	2238028.25	0.00050595	0.05059497	
1877	1.58371465	0.03596499	0.00129243	0.99971297	0.0157414	2232294.93	0.00050765	0.0507648	
1878	1.81864218	0.03973398	0.00157713	0.99935104	0.01573623	2238110.25	0.00046802	0.04680204	
1879	1.7090627	0.03724215	0.00138577	0.99953795	0.01573925	2238116.11	0.00046659	0.04665935	
1880	0.84827672	0.01905882	0.00036329	1.00064612	0.01575638	2237767.63	0.00050706	0.0507064	
1881	1.19873367	0.0260854	0.00068031	1.00025147	0.01575063	2238131.69	0.0004706	0.04705971	
1882	1.41090655	0.03169747	0.00100421	0.99998288	0.01574587	2238049.21	0.00049885	0.04988525	
1883	1.61363644	0.03666316	0.00134303	0.99966302	0.01574059	2137887.8	0.00050783	0.05078311	
1884	1.54828751	0.03501623	0.00122523	0.99977241	0.01574241	2237871.24	0.00050392	0.05039203	
1885	2.12801999	0.04824144	0.00232299	0.99868118	0.01572496	2237863.12	0.00050042	0.05004249	
1886	0.80502277	0.018069	0.00032655	1.00068336	0.01575698	2237865.62	0.00050691	0.05069095	
1887	0.76286808	0.01710602	0.00029268	1.00071782	0.01575753	2237923.28	0.0005068	0.05068037	
1888	2.19490175	0.04962839	0.00245818	0.99854241	0.01572278	2237935.23	0.00049726	0.04972584	
1889	2.55735701	0.05408762	0.00291919	0.99796468	0.01571454	2238106.84	0.00043348	0.04334778	
1890	0.95797792	0.02157792	0.00046562	1.00054312	0.01575472	2236022.26	0.00050766	0.05076604	
1891	-0.1533565	-0.0036264	1.32E-05	1.00101434	0.01576229	2238125.32	0.00050663	0.05066256	
1892	-1.8985616	-0.0422482	0.00178251	0.99917543	0.01573281	2237230.53	0.00050567	0.0505674	
1893	1.25999939	0.02836453	0.00080429	1.00019107	0.01574915	2238012.06	0.0005027	0.05027014	
1894	0.50218651	0.01117311	0.00012489	1.00089033	0.01576031	2238083.84	0.00050647	0.05064712	
1895	0.50231614	0.01116918	0.0001248	1.00088915	0.0157603	2238104.22	0.00050584	0.05058416	
1896	0.71350808	0.01596651	0.00025499	1.00075457	0.01575814	2238047.55	0.00050585	0.0505851	
1897	0.61039909	0.01361956	0.00018555	1.0008257	0.01575928	2238084.79	0.00050564	0.05056378	
1898	-0.6850151	-0.015369	0.00023627	1.00077243	0.01575857	2238121.2	0.00049697	0.04969728	
1899	0.91002014	0.02020203	0.00040815	1.00057395	0.01575543	2238123.78	0.00049391	0.04939093	
1900	1.54575973	0.0350709	0.00122905	0.99977507	0.0157424	2236586.14	0.00050715	0.0507149	
1901	1.0566489	0.02378047	0.00056547	1.00043702	0.01575305	2237963.71	0.00050524	0.05052446	
1902	1.94461435	0.04420047	0.00195082	0.99905565	0.0157309	2237572.18	0.0005048	0.05047978	
1903	3.49442098	0.07826627	0.00609349	0.9950034	0.01566632	2237953.58	0.0004777	0.04777017	*
1904	0.75355399	0.01680035	0.00028231	1.00071716	0.01575761	2238114.42	0.00050119	0.05011904	
1905	1.5023961	0.03348699	0.00112067	0.9998489	0.01574386	2238091.17	0.00049007	0.04900676	

© 2024 Zhang X et al. *JAMA Pediatrics*.

1906	1.09649633	0.02419019	0.0005851	1.00037646	0.01575241	2238125.88	0.00048497	0.04849725	
1907	1.42719098	0.03231057	0.00104339	0.99995869	0.01574536	2237543.78	0.00050633	0.0506329	
1908	1.908046	0.04042772	0.00163267	0.99923947	0.01573489	2238123.78	0.00043976	0.04397603	
1909	0.33048828	0.00714188	5.10E-05	1.00092812	0.01576123	2238147.24	0.00048671	0.04867117	
1910	1.32159654	0.02970649	0.00088213	1.00010836	0.01574786	2238043.71	0.00050043	0.05004317	
1911	1.65923543	0.03697957	0.0013663	0.99960087	0.01573991	2238084.02	0.00048835	0.04883475	
1912	0.91097874	0.02023813	0.00040961	1.00057389	0.01575542	2238122.38	0.00049462	0.04946181	
1913	1.38741078	0.03124348	0.00097568	1.0000167	0.01574637	2238013.11	0.00050148	0.05014793	
1914	1.79774748	0.04043003	0.00163273	0.99935154	0.01573579	2238025.41	0.00049575	0.04957494	
1915	2.82374771	0.05886299	0.00345609	0.99741546	0.01570595	2238105.39	0.00041964	0.04196415	
1916	3.18812806	0.0686827	0.00469973	0.99626779	0.01568713	2238070.83	0.00044506	0.0445064	*
1917	-0.2516291	-0.0057584	3.32E-05	1.00097021	0.01576178	2238144.56	0.00049424	0.04942386	
1918	1.16744024	0.02635869	0.00069464	1.00030987	0.01575099	2237514.9	0.0005069	0.05069023	
1919	1.42564079	0.03215823	0.00103359	0.99996122	0.01574546	2237970.92	0.0005027	0.05027014	
1920	1.10024153	0.02478004	0.00061398	1.00038818	0.01575227	2237937.93	0.00050535	0.05053538	
1921	1.14524659	0.02581027	0.00066606	1.00033561	0.01575142	2237918.09	0.00050536	0.05053622	
1922	3.31717053	0.07535017	0.00564941	0.99545479	0.01567333	2237809.48	0.00049227	0.04922664	*
1923	0.22220997	0.00470795	2.22E-05	1.00094458	0.01576161	2238148.37	0.0004795	0.04795036	
1924	1.96430199	0.04445619	0.00197343	0.99902446	0.01573047	2237909.6	0.00050034	0.05003421	
1925	1.19148018	0.0266666	0.00071095	1.00027517	0.01575058	2238086.62	0.0004978	0.04977992	
1926	1.56074399	0.03527674	0.00124351	0.99975273	0.01574211	2237911.14	0.00050318	0.05031831	
1927	0.42994349	0.00954146	9.11E-05	1.00092713	0.01576088	2238054.35	0.0005072	0.05071974	
1928	1.74529086	0.03952506	0.00156055	0.99943784	0.01573706	2237849.39	0.00050316	0.05031642	
1929	1.59382675	0.03574117	0.00127643	0.9997036	0.01574145	2238058.41	0.00049502	0.0495018	
1930	0.802961	0.01800338	0.00032418	1.00068367	0.015757	2238016.14	0.00050586	0.05058608	
1931	1.48925053	0.0298295	0.00088944	0.99989251	0.01574606	2238139.6	0.00039648	0.03964847	
1932	2.31774026	0.05112078	0.00260807	0.99834638	0.01572004	2238076.89	0.00047255	0.04725542	
1933	1.80399941	0.04048672	0.00163731	0.99934284	0.01573568	2238042.76	0.00049366	0.04936641	
1934	1.71435787	0.03884275	0.00150722	0.99949227	0.01573792	2237802.02	0.00050395	0.05039525	
1935	1.95533618	0.04378791	0.00191469	0.99906184	0.01573123	2238048.46	0.00049011	0.04901067	
1936	0.93567389	0.02086902	0.00043554	1.00055455	0.01575505	2238110.73	0.00049813	0.04981262	
1937	1.14586004	0.02416083	0.00058367	1.00029268	0.01575174	2238139.87	0.00044252	0.04425197	
1938	2.05972724	0.04697807	0.00220313	0.99881213	0.01572695	2235487.31	0.0005071	0.05070995	
1939	1.12280243	0.02498399	0.00062411	1.00035311	0.0157519	2238111.76	0.00049298	0.04929844	
1940	0.73720604	0.01652532	0.00027315	1.00073835	0.01575786	2237873.71	0.00050705	0.05070542	
1941	0.76320615	0.01680451	0.00028245	1.00069167	0.01575741	2238136.5	0.00048858	0.04885805	
1942	2.12698047	0.0469078	0.00219674	0.99875224	0.01572652	2238085.97	0.00047403	0.04740347	
1943	0.41663064	0.00924397	8.55E-05	1.000934	0.01576099	2237772.38	0.00050769	0.05076933	
1944	1.11618399	0.02519988	0.00063495	1.00037143	0.01575196	2236213.2	0.00050756	0.05075634	
1945	1.10321451	0.02481332	0.00061563	1.00038369	0.01575222	2238013.3	0.00050394	0.0503945	
1946	1.91824924	0.041836	0.00174815	0.9991769	0.01573347	2238108.26	0.00046551	0.04655101	
1947	0.43067711	0.00956238	9.15E-05	1.00092764	0.01576089	2237816.23	0.00050766	0.0507659	

© 2024 Zhang X et al. *JAMA Pediatrics.*

1948	1.08718303	0.02415404	0.00058336	1.00039225	0.01575255	2238116.46	0.00049196	0.04919594	
1949	1.36027133	0.03081714	0.00094926	1.00005591	0.0157469	2065931.32	0.00050784	0.05078381	
1950	0.6512318	0.01357288	0.00018426	1.00069445	0.01575823	2238146.28	0.00043976	0.04397603	
1951	1.26890056	0.02856139	0.00081549	1.00017934	0.01574896	2238016.09	0.00050246	0.05024647	
1952	1.02159202	0.02297351	0.00052777	1.0004747	0.01575366	2237992.04	0.00050499	0.05049918	
1953	0.58667483	0.01310619	0.00017183	1.00084388	0.01575954	2237583.18	0.00050762	0.05076157	
1954	0.59829192	0.01337035	0.00017883	1.00083662	0.01575943	2237579.54	0.00050761	0.05076066	
1955	0.67975333	0.01522492	0.00023186	1.0007819	0.01575855	2237379.73	0.00050761	0.05076083	
1956	0.67972419	0.01522436	0.00023185	1.00078193	0.01575855	2237354.63	0.00050762	0.05076157	
1957	0.72550484	0.01626807	0.00026471	1.00074819	0.01575801	2237105.94	0.00050764	0.05076423	
1958	1.76439999	0.03939905	0.00155066	0.9994196	0.01573699	2238071.92	0.00048916	0.0489161	
1959	2.33768619	0.05248298	0.00274839	0.9982425	0.0157181	2238011.13	0.00048912	0.04891176	
1960	1.78891809	0.03983868	0.00158541	0.99937949	0.01573639	2238080.09	0.00048632	0.04863165	
1961	2.23214267	0.04887885	0.00238486	0.99855284	0.01572344	2238091.85	0.00046659	0.04665935	
1962	1.67607372	0.03679292	0.00135258	0.99958526	0.01573989	2238110.87	0.00047375	0.04737486	
1963	2.19158619	0.04800607	0.00230063	0.99863665	0.01572478	2238093.13	0.00046722	0.04672174	
1964	2.14779572	0.04745806	0.00224846	0.99870471	0.01572574	2238081.48	0.00047566	0.04756551	
1965	2.24945897	0.05000549	0.00249571	0.99847208	0.01572193	2238061.75	0.00048047	0.04804663	
1966	2.75267548	0.06060069	0.00366158	0.99731213	0.01570353	2238059.32	0.00046742	0.04674212	
1967	1.14298881	0.02552978	0.00065166	1.00033225	0.01575151	2238098.65	0.00049645	0.04964504	
1968	0.3571957	0.00789581	6.24E-05	1.0009583	0.01576138	2237947.73	0.00050765	0.05076538	
1969	1.34040101	0.03032536	0.00091923	1.00008371	0.01574736	2237447.77	0.0005067	0.05066996	
1970	2.3559308	0.05230517	0.00272995	0.99823898	0.01571822	2238059.83	0.00047836	0.04783632	
1971	0.29078155	0.0063879	4.08E-05	1.00098014	0.01576174	2238118.88	0.00050711	0.05071124	
1972	0.54974151	0.01212332	0.00014703	1.00084568	0.01575976	2238139.58	0.00049573	0.04957312	
1973	1.57079422	0.03549699	0.00125906	0.99973663	0.01574185	2237922.65	0.00050288	0.05028788	
1974	2.24810159	0.05020929	0.00251601	0.99846111	0.01572168	2238045.08	0.00048491	0.04849074	
1975	0.03075811	0.00046947	2.20E-07	1.00078176	0.01576049	2238148.72	0.00038642	0.03864175	
1976	1.42472431	0.03229121	0.00104214	0.99996227	0.0157454	2235520.06	0.0005075	0.05074958	
1977	2.41310207	0.0538657	0.00289478	0.99808754	0.01571572	2238037.93	0.000483	0.04829983	
1978	1.72320313	0.03894202	0.00151492	0.99947928	0.01573775	2237940.83	0.00050128	0.05012825	
1979	2.13703232	0.04847079	0.00234507	0.99866038	0.01572462	2237843.11	0.00050085	0.05008498	
1980	0.65678743	0.01470226	0.00021622	1.00079807	0.01575881	2237337.32	0.00050764	0.05076374	
1981	1.60030285	0.03623015	0.00131154	0.99968743	0.01574104	2237811.26	0.00050438	0.05043849	
1982	1.62953073	0.03689882	0.00136033	0.99963867	0.01574026	2237810.63	0.00050426	0.05042572	
1983	1.29884814	0.02935258	0.00086125	1.00014066	0.01574828	2237719.48	0.0005061	0.0506097	
1984	1.4390989	0.02998029	0.00089845	0.99994896	0.01574644	2238136.77	0.00042912	0.04291192	
1985	0.60779913	0.01358972	0.00018474	1.00083096	0.01575933	2216710.07	0.00050784	0.05078364	
1986	0.69084065	0.01545915	0.00023905	1.00077207	0.01575841	2238019.18	0.0005064	0.05063974	
1987	1.66825665	0.03788755	0.0014341	0.9995705	0.01573913	2236928.46	0.0005068	0.05067994	
1988	0.47885324	0.01065372	0.00011355	1.00090409	0.01576051	2237954.77	0.00050743	0.05074339	
1989	0.69175549	0.01547959	0.00023968	1.00077136	0.0157584	2238021.02	0.00050637	0.05063721	

© 2024 Zhang X et al. *JAMA Pediatrics.*

1990	1.87481064	0.04216148	0.00177532	0.99920902	0.01573352	2238022.38	0.00049496	0.04949565	
1991	-1.8481019	-0.040947	0.0016746	0.99927971	0.01573456	2237882.34	0.00050075	0.05007451	
1992	0.72601199	0.01612102	0.00025995	1.00073319	0.01575792	2238127.92	0.00049775	0.04977489	
1993	0.93817071	0.02088305	0.00043612	1.00054989	0.01575501	2238117.09	0.0004961	0.04961018	
1994	-1.020815	-0.0229106	0.00052489	1.00049024	0.01575393	2238000.81	0.00050366	0.05036597	
1995	0.01040978	5.71E-05	3.26E-09	1.00102185	0.01576244	2238147.79	0.00050497	0.05049694	
1996	1.23416776	0.02764914	0.00076426	1.00022233	0.01574972	2238078.84	0.00049822	0.04982191	
1997	-1.2168447	-0.0272658	0.00074325	1.00026646	0.01575032	2237863.78	0.00050483	0.0504832	
1998	0.34759161	0.00767409	5.89E-05	1.00096086	0.01576143	2238100.55	0.00050711	0.05071082	
1999	1.16407536	0.02623054	0.00068791	1.00031273	0.01575106	2237944.25	0.00050494	0.05049446	
2000	-1.7229215	-0.0382914	0.00146475	0.99950541	0.01573815	2237836.63	0.00050254	0.05025425	
2001	-1.6186523	-0.0359214	0.0012893	0.99968504	0.01574107	2237962.96	0.00049994	0.04999374	
2002	-1.2115723	-0.0271503	0.00073697	1.00027304	0.01575043	2237863.55	0.00050486	0.05048587	
2003	-0.9184552	-0.0206509	0.0004265	1.0005919	0.01575556	2238021.96	0.00050382	0.05038245	
2004	0.23079332	0.00490046	2.40E-05	1.00094413	0.01576159	2238148.32	0.00048028	0.0480279	
2005	0.05346991	0.00102737	1.06E-06	1.00102441	0.01576245	2238148.1	0.00050711	0.05071065	
2006	-0.885329	-0.0199835	0.00039939	1.00062652	0.01575606	2237457.04	0.00050715	0.05071539	
2007	-0.9739652	-0.0219265	0.00048079	1.00054052	0.0157547	2237837.29	0.00050602	0.05060204	
2008	-0.1164951	-0.0027778	7.72E-06	1.00100532	0.01576226	2238146.25	0.00049971	0.04997059	

eTable 8. Univariate meta-regression.

**Country or Region**	Mixed-Effects Model (k = 2006; tau^2 estimator: DL)<br><br>tau^2 (estimated amount of residual heterogeneity): 0.0010 (SE = 0.0002)<br>tau (square root of estimated tau^2 value): 0.0315<br>I^2 (residual heterogeneity / unaccounted variability): 99.63%<br>H^2 (unaccounted variability / sampling variability): 270.35<br>R^2 (amount of heterogeneity accounted for): 66.55%<br><br>Test for Residual Heterogeneity:<br>QE(df = 1853) = 500966.3322, p-val < .0001<br><br>Test of Moderators (coefficients 2:153):<br><br>F(df1 = 152, df2 = 1853) = 8.8895, p-val < .0001<br>Model Results:<br><br>estimate se tval pval ci.lb ci.ub<br>intrcpt 0.0879 0.0398 2.2106 0.0272 0.0099 0.1659 *<br>countryAlgeria -0.0512 0.0515 -0.9928 0.3209 -0.1522 0.0499<br>countryArgentina 0.0296 0.0431 0.6858 0.4929 -0.055 0.1141<br>countryAustralia -0.0271 0.0405 -0.6683 0.504 -0.1064 0.0523<br>countryAustria -0.0494 0.0487 -1.0144 0.3105 -0.1449 0.0461<br>countryBahamas 0.1254 0.0711 1.7627 0.0781 -0.0141 0.2649 .<br>countryBahrain 0.0107 0.0523 0.2041 0.8383 -0.0919 0.1132<br>countryBangladesh -0.003 0.0449 -0.0662 0.9472 -0.0911 0.0851<br>countryBarbados 0.0544 0.0701 0.7758 0.438 -0.0831 0.1919<br>countryBelgium -0.0637 0.0423 -1.5053 0.1324 -0.1467 0.0193<br>countryBenin -0.0732 0.0561 -1.3044 0.1923 -0.1832 0.0369<br>countryBhutan -0.0828 0.0686 -1.2072 0.2275 -0.2173 0.0517<br>countryBolivia -0.0452 0.0487 -0.9274 0.3539 -0.1407 0.0504<br>countryBosnia and Herzegovina 0.02 0.0563 0.3556 0.7222 -0.0905 0.1305<br>countryBotswana -0.0384 0.0698 -0.5502 0.5823 -0.1753 0.0985<br>countryBrazil 0.0018 0.0402 0.0456 0.9636 -0.0771 0.0807<br>countryBrunei Darussalam 0.087 0.0701 1.2416 0.2145 -0.0504 0.2244

© 2024 Zhang X et al. *JAMA Pediatrics.*

	countryBulgaria	-0.0187	0.046	-0.407	0.6841	-0.1088	0.0714	
	countryBurkina Faso	-0.0631	0.0513	-1.2312	0.2184	-0.1637	0.0374	
	countryBurundi	-0.073	0.0684	-1.0677	0.2858	-0.2071	0.0611	
	countryCote d'Ivoire	-0.0695	0.056	-1.24	0.2151	-0.1794	0.0404	
	countryCameroon	-0.064	0.0451	-1.4213	0.1554	-0.1524	0.0243	
	countryCanada	0.0168	0.0407	0.4138	0.679	-0.063	0.0967	
	countryChile	0.0758	0.0433	1.752	0.0799	-0.0091	0.1607	.
	countryChina mainland	-0.0046	0.04	-0.1156	0.908	-0.0831	0.0739	
	countryColombia	-0.0376	0.046	-0.8177	0.4136	-0.1279	0.0526	
	countryComoros	-0.0238	0.0688	-0.346	0.7294	-0.1587	0.1111	
	countryCongo	-0.0679	0.0559	-1.2136	0.2251	-0.1775	0.0418	
	countryCosta Rica	0.0535	0.0683	0.7838	0.4333	-0.0804	0.1875	
	countryCroatia	-0.0227	0.0445	-0.5097	0.6104	-0.1101	0.0647	
	countryCyprus	-0.0221	0.0446	-0.496	0.62	-0.1095	0.0653	
	countryCzech	-0.0367	0.0451	-0.815	0.4152	-0.1251	0.0516	
	countryDenmark	-0.0521	0.043	-1.2127	0.2254	-0.1364	0.0322	
	countryDjibouti	-0.0176	0.0564	-0.3113	0.7556	-0.1283	0.0931	
	countryDominican Republic	0.0347	0.0708	0.4907	0.6237	-0.1041	0.1736	
	countryEast Timor	-0.0756	0.0685	-1.1049	0.2694	-0.2099	0.0586	
	countryEcuador	0.0338	0.0498	0.6795	0.4969	-0.0638	0.1315	
	countryEgypt	0.0445	0.0443	1.0045	0.3153	-0.0423	0.1313	
	countryEl Salvador	0.0022	0.0683	0.0318	0.9747	-0.1318	0.1361	
	countryEstonia	-0.0649	0.0488	-1.3312	0.1833	-0.1605	0.0307	
	countryEthiopia	-0.057	0.0433	-1.3174	0.1879	-0.142	0.0279	
	countryFiji	-0.0261	0.0513	-0.5088	0.611	-0.1268	0.0746	
	countryFinland	-0.0549	0.0445	-1.2352	0.2169	-0.1422	0.0323	
	countryFrance	-0.0452	0.0415	-1.0901	0.2758	-0.1265	0.0361	
	countryFrench Polynesia	0.1329	0.0703	1.8904	0.0589	-0.005	0.2708	.
	countryGabon	-0.0509	0.0685	-0.7421	0.4581	-0.1852	0.0835	
	countryGambia	-0.054	0.0685	-0.7878	0.4309	-0.1883	0.0804	
	countryGeorgia	-0.0071	0.0583	-0.122	0.9029	-0.1215	0.1073	
	countryGermany	-0.0408	0.0408	-1.0003	0.3173	-0.1208	0.0392	
	countryGhana	-0.002	0.0435	-0.0467	0.9628	-0.0874	0.0833	
	countryGreece	-0.0025	0.0405	-0.0606	0.9517	-0.0819	0.0769	
	countryGreenland	-0.0666	0.0563	-1.1833	0.2368	-0.1769	0.0438	
	countryGuatemala	0.1077	0.0775	1.3896	0.1648	-0.0443	0.2597	
	countryGuinea	-0.063	0.0685	-0.9206	0.3574	-0.1973	0.0712	
	countryHonduras	-0.044	0.0687	-0.6415	0.5213	-0.1787	0.0906	
	countryHong Kong	-0.033	0.0432	-0.7628	0.4457	-0.1178	0.0518	
	countryHungary	-0.0211	0.0432	-0.4885	0.6252	-0.1059	0.0637	
	countryIceland	-0.061	0.0486	-1.2555	0.2094	-0.1563	0.0343	
	countryIndia	-0.0256	0.0402	-0.6371	0.5241	-0.1045	0.0533	
	countryIndonesia	0.0241	0.0439	0.5473	0.5842	-0.0621	0.1102	
	countryIran	0.0042	0.0403	0.1049	0.9165	-0.0748	0.0833	
	countryIraq	-0.0308	0.0474	-0.6489	0.5165	-0.1237	0.0622	
	countryIreland	-0.0288	0.0424	-0.6798	0.4967	-0.112	0.0543	
	countryIsrael	-0.0142	0.0443	-0.3201	0.749	-0.101	0.0727	
	countryItaly	0.0021	0.0405	0.0518	0.9587	-0.0774	0.0816	
	countryJamaica	0.0129	0.0702	0.1845	0.8537	-0.1247	0.1506	
	countryJapan	-0.046	0.0425	-1.0825	0.2792	-0.1294	0.0373	
	countryJordan	0.0065	0.0431	0.1517	0.8794	-0.078	0.0911	
	countryKazakhstan	-0.0567	0.056	-1.0121	0.3116	-0.1665	0.0531	
	countryKenya	-0.0315	0.0493	-0.6385	0.5232	-0.1282	0.0652	
	countryKiribati	-0.0139	0.0693	-0.2013	0.8405	-0.1498	0.1219	
	countryKuwait	0.1185	0.0437	2.71	0.0068	0.0327	0.2042	**
	countryKyrgyzstan	-0.0609	0.0684	-0.8901	0.3735	-0.195	0.0733	
	countryLaos	-0.066	0.0686	-0.9623	0.336	-0.2005	0.0685	
	countryLatvia	-0.0527	0.0472	-1.1175	0.2639	-0.1453	0.0398	
	countryLebanon	-0.0168	0.0455	-0.3688	0.7123	-0.1059	0.0724	
	countryLiberia	-0.071	0.0684	-1.0383	0.2993	-0.2052	0.0631	
	countryLibya	0.0148	0.0493	0.3	0.7642	-0.0818	0.1114	
	countryLithuania	-0.04	0.047	-0.8523	0.3942	-0.1321	0.0521	
	countryLuxemburg	-0.0648	0.0684	-0.9477	0.3434	-0.199	0.0693	
	countryMacedonia	-0.0196	0.0488	-0.4013	0.6883	-0.1154	0.0762	
	countryMalawi	-0.0551	0.056	-0.9843	0.3251	-0.1649	0.0547	
	countryMalaysia	0.0225	0.0418	0.5381	0.5906	-0.0595	0.1045	
	countryMali	-0.0659	0.0684	-0.9635	0.3354	-0.2	0.0682	
	countryMalta	0.0403	0.0475	0.8483	0.3964	-0.0529	0.1335	
	countryMauritania	-0.0539	0.0687	-0.7847	0.4327	-0.1885	0.0808	
	countryMauritius	-0.0185	0.0523	-0.3541	0.7233	-0.1211	0.0841	
	countryMexico	0.0798	0.0411	1.9432	0.0521	-0.0007	0.1604	.
	countryMongolia	-0.0698	0.0684	-1.0209	0.3075	-0.204	0.0643	
	countryMontenegro	0.0065	0.0524	0.1246	0.9009	-0.0962	0.1093	
	countryMorocco	-0.0036	0.0473	-0.0752	0.9401	-0.0964	0.0893	
	countryMozambique	-0.0459	0.0684	-0.6716	0.5019	-0.18	0.0882	

© 2024 Zhang X et al. *JAMA Pediatrics.*

	countryMultiple Countries -0.017 0.0423 -0.4022 0.6876 -0.1 0.066<br>countryNamibia -0.067 0.056 -1.1952 0.2322 -0.1768 0.0429<br>countryNepal -0.048 0.0454 -1.0586 0.2899 -0.137 0.041<br>countryNetherlands -0.0507 0.0413 -1.2261 0.2203 -0.1317 0.0304<br>countryNew Zealand 0.0647 0.0441 1.4645 0.1432 -0.0219 0.1512<br>countryNiger -0.053 0.0684 -0.7738 0.4391 -0.1872 0.0813<br>countryNigeria -0.0429 0.0428 -1.0004 0.3172 -0.1269 0.0412<br>countryNorway -0.0632 0.0419 -1.5089 0.1315 -0.1454 0.019<br>countryPakistan 0.0139 0.0435 0.3184 0.7502 -0.0715 0.0992<br>countryPalestine -0.0279 0.0447 -0.6244 0.5324 -0.1156 0.0597<br>countryPeru -0.0156 0.049 -0.318 0.7505 -0.1117 0.0805<br>countryPhilippines -0.0598 0.0684 -0.8747 0.3818 -0.194 0.0743<br>countryPoland -0.0412 0.0408 -1.0095 0.3128 -0.1212 0.0388<br>countryPortugal -0.001 0.0408 -0.0235 0.9813 -0.081 0.0791<br>countryPuerto Rico 0.1901 0.0515 3.6947 0.0002 0.0892 0.291 ***<br>countryQatar 0.0067 0.0521 0.129 0.8974 -0.0955 0.1089<br>countryRepublic of Marshall Islands -0.0368 0.0686 -0.5369 0.5914 -0.1714 0.0977<br>countryRomania -0.0132 0.0435 -0.3037 0.7614 -0.0986 0.0722<br>countryRussia -0.0591 0.047 -1.2579 0.2086 -0.1512 0.033<br>countryRwanda -0.0648 0.0684 -0.9474 0.3435 -0.199 0.0694<br>countrySamoa 0.1052 0.0697 1.509 0.1315 -0.0315 0.242<br>countrySan Marino 0.0342 0.0759 0.4508 0.6522 -0.1146 0.1831<br>countrySaudi Arabia 0.0834 0.0413 2.019 0.0436 0.0024 0.1645 *<br>countrySenegal -0.0798 0.0683 -1.1683 0.2429 -0.2138 0.0542<br>countrySerbia -0.0024 0.0438 -0.0542 0.9567 -0.0883 0.0835<br>countrySeychelles -0.0169 0.0513 -0.3296 0.7418 -0.1175 0.0837<br>countrySeychelles -0.0259 0.0684 -0.3778 0.7056 -0.1601 0.1084<br>countrySierra Leone 0.007 0.0687 0.1024 0.9185 -0.1277 0.1418<br>countrySingapore -0.0201 0.0515 -0.3908 0.696 -0.1212 0.0809<br>countrySlovakia -0.0749 0.056 -1.3373 0.1813 -0.1847 0.0349<br>countrySlovenia -0.0363 0.0432 -0.8394 0.4013 -0.1211 0.0485<br>countrySolomon Islands -0.0612 0.0687 -0.8901 0.3735 -0.1959 0.0736<br>countrySouth Africa -0.0196 0.0415 -0.4723 0.6367 -0.101 0.0618<br>countrySouth Korea -0.0009 0.0413 -0.0226 0.982 -0.082 0.0801<br>countrySpain 0.0094 0.0405 0.2315 0.817 -0.0701 0.0889<br>countrySri Lanka -0.052 0.0489 -1.0624 0.2882 -0.148 0.044<br>countrySudan -0.0145 0.0523 -0.2764 0.7823 -0.117 0.0881<br>countrySuriname 0.027 0.0699 0.387 0.6988 -0.11 0.164<br>countrySweden -0.0541 0.0409 -1.3217 0.1864 -0.1344 0.0262<br>countrySwitzerland -0.0527 0.0435 -1.2115 0.2259 -0.1381 0.0326<br>countrySyria 0.0293 0.0517 0.567 0.5708 -0.0721 0.1307<br>countryTaiwan 0.0359 0.0417 0.8598 0.39 -0.0459 0.1177<br>countryTajikistan -0.073 0.0684 -1.0674 0.2859 -0.2072 0.0611<br>countryTanzania -0.038 0.0453 -0.8393 0.4014 -0.1269 0.0508<br>countryThailand 0.0122 0.0425 0.2868 0.7743 -0.0711 0.0955<br>countryTogo -0.074 0.0561 -1.3193 0.1872 -0.184 0.036<br>countryTonga 0.101 0.0523 1.9326 0.0534 -0.0015 0.2036 .<br>countryTrinidad and Tobago 0.0478 0.0578 0.827 0.4084 -0.0656 0.1612<br>countryTunisia -0.0306 0.0566 -0.5397 0.5895 -0.1416 0.0805<br>countryTurkey -0.0107 0.0405 -0.2645 0.7914 -0.0902 0.0687<br>countryTurkmenistan 0.0201 0.0685 0.2935 0.7692 -0.1143 0.1545<br>countryUganda -0.0748 0.0684 -1.0948 0.2737 -0.2089 0.0592<br>countryUkraine -0.0578 0.047 -1.2299 0.2189 -0.1501 0.0344<br>countryUnited Arab Emirates 0.067 0.0458 1.4634 0.1435 -0.0228 0.1568<br>countryUnited Kingdom of Great Britain and Northern Ireland -0.007 0.0406 -0.1733 0.8625 -<br>0.0866 0.0725<br>countryUnited States of America 0.0976 0.0399 2.4441 0.0146 0.0193 0.176 *<br>countryVanuatu -0.0843 0.0684 -1.2335 0.2176 -0.2184 0.0498<br>countryVietnam -0.0074 0.0428 -0.1723 0.8632 -0.0914 0.0766<br>countryYemen 0.0014 0.0685 0.0198 0.9842 -0.1329 0.1356<br>countryZambia -0.0539 0.0684 -0.7885 0.4305 -0.188 0.0802<br><br>---<br>Signif. codes: 0 ‘***’ 0.001 ‘**’ 0.01 ‘*’ 0.05 ‘.’ 0.1 ‘ ’ 1
**Continent**	Mixed-Effects Model (k = 2006; tau^2 estimator: DL)<br><br>tau^2 (estimated amount of residual heterogeneity): 0.0016 (SE = 0.0003)<br>tau (square root of estimated tau^2 value): 0.0398<br>I^2 (residual heterogeneity / unaccounted variability): 99.81%<br>H^2 (unaccounted variability / sampling variability): 513.86<br>R^2 (amount of heterogeneity accounted for): 46.78%<br><br>Test for Residual Heterogeneity:

© 2024 Zhang X et al. *JAMA Pediatrics*.

	QE(df = 1983) = 1018981.0512, p-val < .0001<br><br>Test of Moderators (coefficients 2:23):<br>F(df1 = 22, df2 = 1983) = 40.1216, p-val < .0001<br><br>Model Results:<br><br>estimate se tval pval ci.lb ci.ub<br>intrcpt 0.0710 0.0159 4.4777 <.0001 0.0399 0.1021 ***<br>continentAustralia and New Zealand 0.0025 0.0176 0.1414 0.8876 -0.0320 0.0370<br>continentCaribbean 0.1211 0.0260 4.6580 <.0001 0.0701 0.1721 ***<br>continentCentral America 0.0926 0.0189 4.8915 <.0001 0.0555 0.1297 ***<br>continentCentral Asia -0.0243 0.0239 -1.0156 0.3099 -0.0711 0.0226<br>continentEastern Africa -0.0254 0.0191 -1.3306 0.1835 -0.0628 0.0120<br>continentEastern Asia 0.0124 0.0164 0.7582 0.4484 -0.0197 0.0446<br>continentEastern Europe -0.0190 0.0172 -1.1038 0.2698 -0.0527 0.0148<br>continentMelanesia -0.0280 0.0317 -0.8818 0.3780 -0.0901 0.0342<br>continentMicronesia -0.0104 0.0253 -0.4118 0.6805 -0.0600 0.0392<br>continentMiddle Africa -0.0466 0.0250 -1.8596 0.0631 -0.0957 0.0025 .<br>continentNorthern Africa 0.0255 0.0200 1.2729 0.2032 -0.0138 0.0648<br>continentNorthern America 0.1035 0.0163 6.3565 <.0001 0.0716 0.1354 ***<br>continentNorthern Europe -0.0201 0.0166 -1.2121 0.2256 -0.0527 0.0124<br>continentPolynesia 0.1247 0.0323 3.8585 0.0001 0.0613 0.1880 ***<br>continentSouth-Eastern Asia 0.0250 0.0167 1.4992 0.1340 -0.0077 0.0578<br>continentSouth America 0.0281 0.0168 1.6764 0.0938 -0.0048 0.0610<br>continentSouthern Africa -0.0056 0.0199 -0.2827 0.7774 -0.0447 0.0335<br>continentSouthern Asia -0.0060 0.0168 -0.3555 0.7223 -0.0390 0.0270<br>continentSouthern Europe 0.0182 0.0163 1.1162 0.2645 -0.0138 0.0503<br>continentWestern Africa -0.0220 0.0186 -1.1838 0.2366 -0.0584 0.0144<br>continentWestern Asia 0.0381 0.0167 2.2855 0.0224 0.0054 0.0708 *<br>continentWestern Europe -0.0284 0.0168 -1.6885 0.0915 -0.0613 0.0046 .<br><br>---<br><br>Signif. codes: 0 '***' 0.001 '**' 0.01 '*' 0.05 '.' 0.1 ' ' 1
**Sample Size**	Mixed-Effects Model (k = 2006; tau^2 estimator: DL)<br><br>tau^2 (estimated amount of residual heterogeneity): 0.0030 (SE = 0.0009)<br>tau (square root of estimated tau^2 value): 0.0545<br>I^2 (residual heterogeneity / unaccounted variability): 99.93%<br>H^2 (unaccounted variability / sampling variability): 1349.65<br>R^2 (amount of heterogeneity accounted for): 0.01%<br><br>Test for Residual Heterogeneity:<br>QE(df = 2004) = 2704705.6597, p-val < .0001<br><br>Test of Moderators (coefficient 2):<br>F(df1 = 1, df2 = 2004) = 8.3299, p-val = 0.0039<br><br>Model Results:<br><br>estimate se tval pval ci.lb ci.ub<br>intrcpt 0.0976 0.0020 49.6732 <.0001 0.0938 0.1015 ***<br>size -0.0118 0.0041 -2.8862 0.0039 -0.0198 -0.0038 **<br>---<br>Signif. codes: 0 '***' 0.001 '**' 0.01 '*' 0.05 '.' 0.1 ' ' 1
**Development Level**	Mixed-Effects Model (k = 2006; tau^2 estimator: DL)<br><br>tau^2 (estimated amount of residual heterogeneity): 0.0017 (SE = 0.0005)<br>tau (square root of estimated tau^2 value): 0.0416<br>I^2 (residual heterogeneity / unaccounted variability): 99.87%<br>H^2 (unaccounted variability / sampling variability): 761.13<br>R^2 (amount of heterogeneity accounted for): 41.86%<br><br>Test for Residual Heterogeneity:<br>QE(df = 2003) = 1524541.2218, p-val < .0001<br><br>Test of Moderators (coefficients 2:3):<br>F(df1 = 2, df2 = 2003) = 17.4872, p-val < .0001<br><br>Model Results:<br><br>estimate se tval pval ci.lb ci.ub

© 2024 Zhang X et al. *JAMA Pediatrics.*

	intrcpt 0.0710 0.0189 3.7553 0.0002 0.0339 0.1081 ***<br>developmentdeveloped 0.0328 0.0191 1.7193 0.0857 -0.0046 0.0702 .<br>developmentdeveloping 0.0133 0.0191 0.6966 0.4861 -0.0241 0.0506<br><br>---<br>Signif. codes: 0 '***' 0.001 '**' 0.01 '*' 0.05 '.' 0.1 ' ' 1
**Diagnostic Reference**	Mixed-Effects Model (k = 2006; tau^2 estimator: DL)<br><br>tau^2 (estimated amount of residual heterogeneity): 0.0033 (SE = 0.0008)<br>tau (square root of estimated tau^2 value): 0.0571<br>I^2 (residual heterogeneity / unaccounted variability): 99.92%<br>H^2 (unaccounted variability / sampling variability): 1262.78<br>R^2 (amount of heterogeneity accounted for): 0.00%<br><br>Test for Residual Heterogeneity:<br>QE(df = 2002) = 2528092.9521, p-val < .0001<br><br>Test of Moderators (coefficients 2:4):<br>F(df1 = 3, df2 = 2002) = 171.0796, p-val < .0001<br><br>Model Results:<br><br>estimate se tval pval ci.lb ci.ub<br>intrcpt 0.1529 0.0033 46.3359 <.0001 0.1464 ***<br>diagnosticIOTF -0.0921 0.0041 -22.4962 <.0001 -0.1001 ***<br>diagnosticNationalReference -0.0476 0.0053 -9.0224 <.0001 -0.0580 ***<br>diagnosticWHO -0.0581 0.0046 -12.6483 <.0001 -0.0672 ***<br><br>---<br><br>Signif. codes: 0 '***' 0.001 '**' 0.01 '*' 0.05 '.' 0.1 ' ' 1
**Sample Source**	Mixed-Effects Model (k = 2006; tau^2 estimator: DL)<br><br>tau^2 (estimated amount of residual heterogeneity): 0.0029 (SE = 0.0008)<br>tau (square root of estimated tau^2 value): 0.0539<br>I^2 (residual heterogeneity / unaccounted variability): 99.92%<br>H^2 (unaccounted variability / sampling variability): 1192.59<br>R^2 (amount of heterogeneity accounted for): 2.37%<br><br>Test for Residual Heterogeneity:<br>QE(df = 2002) = 2387566.4644, p-val < .0001<br><br>Test of Moderators (coefficients 2:4):<br>F(df1 = 3, df2 = 2002) = 19.9431, p-val < .0001<br><br>Model Results:<br><br>estimate se tval pval ci.lb ci.ub<br>intrcpt 0.0992 0.0056 17.8658 <.0001 0.0883 0.1100 ***<br>sourcedatabase -0.0136 0.0063 -2.1734 0.0299 -0.0259 -0.0013 **<br>sourcemedical institution 0.0485 0.0094 5.1769 <.0001 0.0302 0.0669 ***<br>sourceschool -0.0041 0.0060 -0.6722 0.5015 -0.0159 0.0078<br><br>---<br><br>Signif. codes: 0 '***' 0.001 '**' 0.01 '*' 0.05 '.' 0.1 ' ' 1
**Study Design**	Mixed-Effects Model (k = 2006; tau^2 estimator: DL)<br><br>tau^2 (estimated amount of residual heterogeneity): 0.0028 (SE = 0.0008)<br>tau (square root of estimated tau^2 value): 0.0533<br>I^2 (residual heterogeneity / unaccounted variability): 99.91%<br>H^2 (unaccounted variability / sampling variability): 1113.91<br>R^2 (amount of heterogeneity accounted for): 4.38%<br><br>Test for Residual Heterogeneity:<br>QE(df = 2000) = 2227820.9565, p-val < .0001<br><br>Test of Moderators (coefficients 2:6):<br>F(df1 = 5, df2 = 2000) = 0.6899, p-val = 0.6311<br><br>Model Results:<br><br>estimate se tval pval ci.lb ci.ub<br>intrcpt 0.1467 0.0819 1.7924 0.0732 -0.0138 0.3073 .<br>designcohort -0.0434 0.0823 -0.5269 0.5983 -0.2047 0.1180

© 2024 Zhang X et al. *JAMA Pediatrics.*

	designcross-sectional -0.0528 0.0819 -0.6445 0.5193 -0.2133 0.1078<br>designlongitudinal -0.0424 0.0827 -0.5127 0.6082 -0.2045 0.1198<br>designprospective -0.0387 0.0854 -0.4529 0.6507 -0.2061 0.1288<br>designRCT -0.0318 0.0849 -0.3745 0.7081 -0.1982 0.1347<br><br>---<br><br>Signif. codes: 0 ‘***’ 0.001 ‘**’ 0.01 ‘*’ 0.05 ‘.’ 0.1 ‘ ’ 1
**Publication Year**	Mixed-Effects Model (k = 2006; tau^2 estimator: DL)<br><br>tau^2 (estimated amount of residual heterogeneity): 0.0029 (SE = 0.0009)<br>tau (square root of estimated tau^2 value): 0.0542<br>I^2 (residual heterogeneity / unaccounted variability): 99.92%<br>H^2 (unaccounted variability / sampling variability): 1307.27<br>R^2 (amount of heterogeneity accounted for): 1.37%<br><br>Test for Residual Heterogeneity:<br>QE(df = 2004) = 2619765.9043, p-val < .0001<br><br>Test of Moderators (coefficient 2):<br>F(df1 = 1, df2 = 2004) = 5.5007, p-val = 0.0191<br><br>Model Results:<br><br>estimate se tval pval ci.lb ci.ub<br>intrcpt -1.5144 0.6862 -2.2071 0.0274 -2.8601 -0.1687 *<br>publication 0.0008 0.0003 2.3454 0.0191 0.0001 0.0015 *<br><br>---<br><br>Signif. codes: 0 ‘***’ 0.001 ‘**’ 0.01 ‘*’ 0.05 ‘.’ 0.1 ‘ ’ 1

eTable 9. Results of multivariable meta-regression analysis.

```
Multimodel Inference: Final Results
--------------------------

 - Number of fitted models: 512
 - Full formula: ~ publication + design + income + source + country + continent + size + diagnostic + development
 - Coefficient significance test: knha
 - Interactions modeled: no
 - Evaluation criterion: AICc


Best 5 Models
--------------------------


Global model call: metafor::rma(yi = TE, sei = seTE, mods = form, data = glm.data,
    method = method, test = test)
---
Model selection table
    (Intrc) cntnn desgn dvlpm dgnst incom     pblct      size sourc df   logLik    AICc delta weight
498       +     +                 +     + 0.0010230 -0.01518     + 35 2720.679 -5370.1  0.00  0.689
506       +     +           +     +     + 0.0009662 -0.01541     + 36 2720.689 -5368.0  2.05  0.247
434       +     +                 +     +           -0.01450     + 34 2716.727 -5364.2  5.83  0.037
442       +     +           +     +     +           -0.01481     + 35 2717.412 -5363.5  6.53  0.026
```

© 2024 Zhang X et al. *JAMA Pediatrics*.

502 + + + + + 0.0011400 -0.01560 + 40 2719.126 -5356.6 13.50 0.001
Models ranked by AICc(x)

Multimodel Inference Coefficients

	Estimate	Std. Error	z value	Pr(>|z|)
intrcpt	-1.798131e+00	7.803885e-01	2.304148e+00	0.0212143
continentAustralia and New Zealand	-1.355924e-02	1.823520e-02	7.435748e-01	0.4571338
continentCaribbean	7.492982e-02	2.540007e-02	2.949984e+00	0.0031779
continentCentral America	7.210581e-02	1.846149e-02	3.905742e+00	0.0000939
continentCentral Asia	-3.150518e-02	2.410108e-02	1.307210e+00	0.1911413
continentEastern Africa	-2.750799e-02	2.054470e-02	1.338934e+00	0.1805922
continentEastern Asia	-5.775004e-03	1.627332e-02	3.548755e-01	0.7226828
continentEastern Europe	-4.215230e-02	1.767599e-02	2.384721e+00	0.0170921
continentMelanesia	-8.268136e-03	3.120131e-02	2.649933e-01	0.7910147
continentMicronesia	-7.712613e-03	2.577984e-02	2.991722e-01	0.7648086
continentMiddle Africa	-5.172292e-02	2.561254e-02	2.019437e+00	0.0434418
continentNorthern Africa	3.046851e-02	2.070046e-02	1.471876e+00	0.1410544
continentNorthern America	5.772230e-02	1.725304e-02	3.345631e+00	0.0008210
continentNorthern Europe	-3.486453e-02	1.714943e-02	2.032985e+00	0.0420540
continentPolynesia	1.231190e-01	3.136674e-02	3.925144e+00	0.0000867
continentSouth-Eastern Asia	1.886970e-02	1.748900e-02	1.078947e+00	0.2806114
continentSouth America	1.225641e-02	1.653333e-02	7.413149e-01	0.4585026
continentSouthern Africa	-1.094483e-02	1.948373e-02	5.617419e-01	0.5742919
continentSouthern Asia	-1.203825e-02	1.853167e-02	6.496041e-01	0.5159480
continentSouthern Europe	9.596103e-04	1.693679e-02	5.665834e-02	0.9548174
continentWestern Africa	-2.343602e-02	1.995674e-02	1.174342e+00	0.2402583
continentWestern Asia	1.537885e-02	1.664327e-02	9.240284e-01	0.3554715
continentWestern Europe	-4.478948e-02	1.741583e-02	2.571769e+00	0.0101181
diagnosticIOTF	-4.811007e-02	4.674154e-03	1.029279e+01	0.0000000
diagnosticNationalReference	-5.616114e-03	5.419860e-03	1.036210e+00	0.3001041
diagnosticWHO	-1.418388e-02	5.068897e-03	2.798218e+00	0.0051385
incomeHigh-Income	2.959900e-02	6.531320e-03	4.531856e+00	0.0000058
incomeLow-Income	-2.124083e-02	1.378730e-02	1.540608e+00	0.1234122
incomeLower-Middle-Income	-1.220832e-02	8.316055e-03	1.468042e+00	0.1420928
publication	9.439778e-04	3.872274e-04	2.437787e+00	0.0147775
size	-1.519536e-02	3.428513e-03	4.432055e+00	0.0000093
sourcedatabase	-4.307882e-03	5.199371e-03	8.285391e-01	0.4073653
sourcemedical institution	3.221678e-02	7.578126e-03	4.251286e+00	0.0000213
sourceschool	6.940933e-03	4.937378e-03	1.405793e+00	0.1597855
developmentdeveloped	-3.414148e-03	6.519478e-03	5.236843e-01	0.6004981

© 2024 Zhang X et al. *JAMA Pediatrics.*

designcohort	-7.118041e-05	3.032551e-03	2.347212e-02	0.9812737
designcross-sectional	-4.912615e-05	2.597797e-03	1.891070e-02	0.9849123
designlongitudinal	-7.128821e-05	3.041616e-03	2.343761e-02	0.9813012
designprospective	-7.869547e-05	3.249413e-03	2.421837e-02	0.9806784
designRCT	-3.085086e-05	2.405538e-03	1.282493e-02	0.9897675
incomeUpper-Middle-Income	2.414715e-62	4.148737e-32	5.820361e-31	1.0000000
developmentdeveloping	4.391619e-63	1.773359e-32	2.476441e-31	1.0000000
countryAlgeria	-3.899725e-107	2.174712e-54	1.793214e-53	1.0000000
countryArgentina	3.284601e-107	2.118284e-54	1.550595e-53	1.0000000
countryAustralia	-6.202145e-107	2.650231e-54	2.340229e-53	1.0000000
countryAustria	-7.113199e-107	2.715106e-54	2.619861e-53	1.0000000
countryBahamas	1.259312e-106	4.408055e-54	2.856843e-53	1.0000000
countryBahrain	2.269439e-107	2.274190e-54	9.979107e-54	1.0000000
countryBangladesh	2.484211e-107	1.908094e-54	1.301934e-53	1.0000000
countryBarbados	3.672261e-107	2.795981e-54	1.313407e-53	1.0000000
countryBelgium	-6.583480e-107	2.454988e-54	2.681675e-53	1.0000000
countryBenin	-5.513649e-107	2.825003e-54	1.951732e-53	1.0000000
countryBhutan	-9.328194e-107	3.703401e-54	2.518818e-53	1.0000000
countryBolivia	-2.364468e-108	1.913274e-54	1.235823e-54	1.0000000
countryBosnia and Herzegovina	6.170394e-108	2.059942e-54	2.995421e-54	1.0000000
countryBotswana	-5.478154e-107	2.788420e-54	1.964608e-53	1.0000000
countryBrazil	-7.474679e-108	1.836494e-54	4.070081e-54	1.0000000
countryBrunei Darussalam	9.444260e-107	3.546832e-54	2.662731e-53	1.0000000
countryBulgaria	1.589108e-107	1.704389e-54	9.323625e-54	1.0000000
countryBurkina Faso	-4.142064e-107	2.496121e-54	1.659400e-53	1.0000000
countryBurundi	-7.591443e-107	3.326250e-54	2.282283e-53	1.0000000
countryC?te d'Ivoire	-5.675712e-107	2.849104e-54	1.992104e-53	1.0000000
countryCameroon	-5.389408e-107	2.622799e-54	2.054831e-53	1.0000000
countryCanada	-1.002375e-107	2.076559e-54	4.827097e-54	1.0000000
countryChile	8.048676e-107	2.964610e-54	2.714919e-53	1.0000000
countryChina mainland	-2.543782e-107	1.509200e-54	1.685517e-53	1.0000000
countryColombia	-5.037953e-107	2.448918e-54	2.057216e-53	1.0000000
countryComoros	-1.409280e-107	2.580473e-54	5.461325e-54	1.0000000
countryCongo	-4.282784e-107	2.738387e-54	1.563981e-53	1.0000000
countryCosta Rica	3.298045e-107	2.816226e-54	1.171087e-53	1.0000000
countryCroatia	-1.609713e-107	1.681760e-54	9.571595e-54	1.0000000
countryCyprus	-8.997912e-108	1.981184e-54	4.541685e-54	1.0000000
countryCzech	2.961079e-108	1.615074e-54	1.833401e-54	1.0000000
countryDenmark	-4.317938e-107	1.733766e-54	2.490496e-53	1.0000000
countryDjibouti	-1.678148e-108	2.107022e-54	7.964552e-55	1.0000000
countryDominican Republic	1.203667e-107	2.627136e-54	4.581670e-54	1.0000000
countryEast Timor	-1.098665e-106	3.871983e-54	2.837475e-53	1.0000000
countryEcuador	6.208736e-107	2.730375e-54	2.273950e-53	1.0000000
countryEgypt	5.986371e-107	2.327384e-54	2.572146e-53	1.0000000

© 2024 Zhang X et al. *JAMA Pediatrics*.

countryEl Salvador	-5.078835e-108	2.666779e-54	1.904483e-54	1.0000000
countryEstonia	-4.812824e-107	2.017797e-54	2.385188e-53	1.0000000
countryEthiopia	-6.455529e-107	2.455709e-54	2.628784e-53	1.0000000
countryFiji	3.121812e-107	2.494113e-54	1.251672e-53	1.0000000
countryFinland	-3.789437e-107	1.684838e-54	2.249141e-53	1.0000000
countryFrance	-5.097085e-107	2.132261e-54	2.390460e-53	1.0000000
countryFrench Polynesis	8.428928e-107	3.983892e-54	2.115752e-53	1.0000000
countryGabon	-3.261070e-107	2.194450e-54	1.486053e-53	1.0000000
countryGambia	-2.734211e-107	2.868451e-54	9.532011e-54	1.0000000
countryGeorgia	-1.145988e-107	2.373164e-54	4.828944e-54	1.0000000
countryGermany	-6.143963e-107	2.325823e-54	2.641630e-53	1.0000000
countryGhana	3.667409e-107	2.218578e-54	1.653045e-53	1.0000000
countryGreece	2.478358e-107	1.633086e-54	1.517591e-53	1.0000000
countryGreenland	-9.816659e-107	3.730610e-54	2.631382e-53	1.0000000
countryGuatemala	7.497719e-107	3.483684e-54	2.152238e-53	1.0000000
countryGuinea	-3.871146e-107	2.970333e-54	1.303270e-53	1.0000000
countryHonduras	-1.167681e-106	4.234772e-54	2.757365e-53	1.0000000
countryHong Kong	-3.849584e-107	1.816114e-54	2.119682e-53	1.0000000
countryHungary	3.807205e-107	1.883787e-54	2.021038e-53	1.0000000
countryIceland	-4.411477e-107	1.941241e-54	2.272504e-53	1.0000000
countryIndia	4.545695e-108	1.635318e-54	2.779701e-54	1.0000000
countryIndonesia	3.741312e-107	1.656650e-54	2.258360e-53	1.0000000
countryIran	2.990936e-108	1.138155e-54	2.627882e-54	1.0000000
countryIraq	-3.172085e-107	2.231550e-54	1.421471e-53	1.0000000
countryIreland	-1.153521e-107	1.249705e-54	9.230346e-54	1.0000000
countryIsrael	-1.742629e-107	2.009638e-54	8.671359e-54	1.0000000
countryItaly	3.428619e-107	1.765864e-54	1.941610e-53	1.0000000
countryJamaica	-1.533771e-107	2.633810e-54	5.823393e-54	1.0000000
countryJapan	-6.487169e-107	2.320042e-54	2.796142e-53	1.0000000
countryJordan	8.782654e-108	1.935714e-54	4.537164e-54	1.0000000
countryKazakhstan	-9.306910e-107	3.522283e-54	2.642295e-53	1.0000000
countryKenya	-2.760504e-107	2.002336e-54	1.378642e-53	1.0000000
countryKiribati	5.578293e-107	3.293609e-54	1.693672e-53	1.0000000
countryKuwait	1.411475e-106	4.425832e-54	3.189174e-53	1.0000000
countryKyrgyzstan	-8.240695e-107	3.600835e-54	2.288551e-53	1.0000000
countryLaos	-9.775597e-107	3.605311e-54	2.711444e-53	1.0000000
countryLatvia	-4.905384e-107	1.981933e-54	2.475051e-53	1.0000000
countryLebanon	-1.350109e-107	2.019961e-54	6.683838e-54	1.0000000
countryLiberia	-4.876172e-107	3.085347e-54	1.580429e-53	1.0000000
countryLibya	5.399100e-107	2.344808e-54	2.302577e-53	1.0000000
countryLithuania	-4.084519e-107	1.824673e-54	2.238493e-53	1.0000000
countryLuxemburg	-6.231198e-107	3.089131e-54	2.017136e-53	1.0000000
countryMacedonia	-3.257728e-107	2.048899e-54	1.589990e-53	1.0000000
countryMalawi	-5.270441e-107	2.572873e-54	2.048466e-53	1.0000000

© 2024 Zhang X et al. *JAMA Pediatrics.*

countryMalaysia	3.244379e-107	1.509123e-54	2.149845e-53	1.0000000
countryMali	-4.232955e-107	3.008718e-54	1.406897e-53	1.0000000
countryMalta	8.089445e-107	2.858011e-54	2.830446e-53	1.0000000
countryMauritania	-2.596825e-107	2.860075e-54	9.079572e-54	1.0000000
countryMauritius	-5.665532e-108	1.947747e-54	2.908761e-54	1.0000000
countryMexico	4.768312e-107	2.174471e-54	2.192861e-53	1.0000000
countryMongolia	-1.248969e-106	4.278366e-54	2.919266e-53	1.0000000
countryMontenegro	2.006739e-107	1.966771e-54	1.020322e-53	1.0000000
countryMorocco	-3.194582e-108	1.724189e-54	1.852803e-54	1.0000000
countryMozambique	-1.957531e-107	2.605234e-54	7.513840e-54	1.0000000
countryMultiple Countries	-1.795855e-108	1.090489e-54	1.646835e-54	1.0000000
countryNamibia	-8.521038e-107	3.002617e-54	2.837870e-53	1.0000000
countryNepal	-3.502592e-107	2.047167e-54	1.710945e-53	1.0000000
countryNetherlands	-5.161466e-107	2.142044e-54	2.409598e-53	1.0000000
countryNew Zealand	5.720339e-107	2.547303e-54	2.245645e-53	1.0000000
countryNiger	-3.754262e-108	2.765083e-54	1.357739e-54	1.0000000
countryNigeria	-1.034935e-107	1.972583e-54	5.246597e-54	1.0000000
countryNorway	-5.050682e-107	1.854717e-54	2.723155e-53	1.0000000
countryPakistan	5.018682e-107	2.232976e-54	2.247531e-53	1.0000000
countryPalestine	1.017978e-107	2.183929e-54	4.661224e-54	1.0000000
countryPeru	-1.992085e-107	2.156341e-54	9.238269e-54	1.0000000
countryPhilippines	-6.767314e-107	3.004607e-54	2.252313e-53	1.0000000
countryPoland	-1.289890e-107	1.501103e-54	8.592952e-54	1.0000000
countryPortugal	2.320345e-107	1.624837e-54	1.428048e-53	1.0000000
countryPuerto Rico	2.092085e-106	6.208393e-54	3.369768e-53	1.0000000
countryQatar	2.516713e-107	2.292578e-54	1.097765e-53	1.0000000
countryRepublic of Marshall Islands	-3.576529e-107	2.275590e-54	1.571693e-53	1.0000000
countryRomania	2.626938e-107	1.724048e-54	1.523703e-53	1.0000000
countryRussia	-1.468743e-107	1.736096e-54	8.460031e-54	1.0000000
countryRwanda	-6.562548e-107	3.147449e-54	2.085037e-53	1.0000000
countrySamoa	9.719804e-107	4.208056e-54	2.309808e-53	1.0000000
countrySan Marino	3.407320e-107	2.762367e-54	1.233479e-53	1.0000000
countrySaudi Arabia	9.741078e-107	3.321086e-54	2.933100e-53	1.0000000
countrySenegal	-3.749799e-107	2.957469e-54	1.267908e-53	1.0000000
countrySerbia	7.679791e-108	1.604763e-54	4.785624e-54	1.0000000
countrySeychelles	2.916689e-107	2.013349e-54	1.448676e-53	1.0000000
countrySierra Leone	4.929109e-107	3.094904e-54	1.592653e-53	1.0000000
countrySingapore	-1.524071e-107	1.682554e-54	9.058081e-54	1.0000000
countrySlovakia	-2.412659e-107	2.135419e-54	1.129829e-53	1.0000000
countrySlovenia	-1.446611e-107	1.630038e-54	8.874702e-54	1.0000000
countrySolomon Islands	-5.097968e-108	2.870771e-54	1.775819e-54	1.0000000
countrySouth Africa	-4.671333e-108	1.082597e-54	4.314931e-54	1.0000000
countrySouth Korea	-2.040236e-107	1.493185e-54	1.366365e-53	1.0000000
countrySpain	3.879140e-107	1.837796e-54	2.110756e-53	1.0000000

© 2024 Zhang X et al. *JAMA Pediatrics.*

```
countrySri Lanka                                         -3.597479e-107 1.906521e-54 1.886933e-53 1.0000000
countrySudan                                             -8.951779e-108 1.900831e-54 4.709403e-54 1.0000000
countrySuriname                                           1.189616e-107 1.840803e-54 6.462481e-54 1.0000000
countrySweden                                            -5.124962e-107 1.841831e-54 2.782537e-53 1.0000000
countrySwitzerland                                       -6.887082e-107 2.547508e-54 2.703458e-53 1.0000000
countrySyria                                              2.593705e-107 1.679430e-54 1.544395e-53 1.0000000
countryTaiwan                                             2.700572e-107 1.521603e-54 1.774820e-53 1.0000000
countryTajikistan                                        -1.199772e-106 4.362139e-54 2.750422e-53 1.0000000
countryTanzania                                          -7.063471e-107 2.829609e-54 2.496271e-53 1.0000000
countryThailand                                           1.294957e-107 1.283046e-54 1.009283e-53 1.0000000
countryTogo                                              -4.179889e-107 2.206399e-54 1.894439e-53 1.0000000
countryTonga                                              7.253072e-107 3.184564e-54 2.277571e-53 1.0000000
countryTrinidad and Tobago                                2.144157e-107 1.691220e-54 1.267817e-53 1.0000000
countryTunisia                                           -2.908873e-108 1.456282e-54 1.997466e-54 1.0000000
countryTurkey                                            -2.842766e-107 2.011217e-54 1.413456e-53 1.0000000
countryTurkmenistan                                       1.933932e-107 1.927747e-54 1.003208e-53 1.0000000
countryUganda                                            -7.821343e-107 3.368154e-54 2.322145e-53 1.0000000
countryUkraine                                           -2.606776e-107 1.594891e-54 1.634454e-53 1.0000000
countryUnited Arab Emirates                               6.486109e-107 2.702980e-54 2.399614e-53 1.0000000
countryUnited Kingdom of Great Britain and Northern Ireland  1.001531e-107 1.119151e-54 8.949029e-54
1.0000000
countryUnited States of America                           6.214055e-107 2.685970e-54 2.313524e-53 1.0000000
countryVanuatu                                           -3.420602e-107 2.234134e-54 1.531064e-53 1.0000000
countryVietnam                                            4.676372e-108 1.116398e-54 4.188805e-54 1.0000000
countryYemen                                              1.512013e-107 1.868423e-54 8.092456e-54 1.0000000
countryZambia                                            -2.958788e-107 2.679054e-54 1.104415e-53 1.0000000
countryZimbabwe                                          -1.714617e-107 1.600817e-54 1.071089e-53 1.0000000


Predictor Importance
--------------------------



        model    importance
1   continent  1.000000e+00
2  diagnostic  1.000000e+00
3      income  9.999351e-01
4      source  9.999074e-01
5        size  9.994791e-01
6 publication  9.363782e-01
7 development  2.732340e-01
8      design  1.108354e-03
9     country 1.256282e-105
```

© 2024 Zhang X et al. *JAMA Pediatrics*.

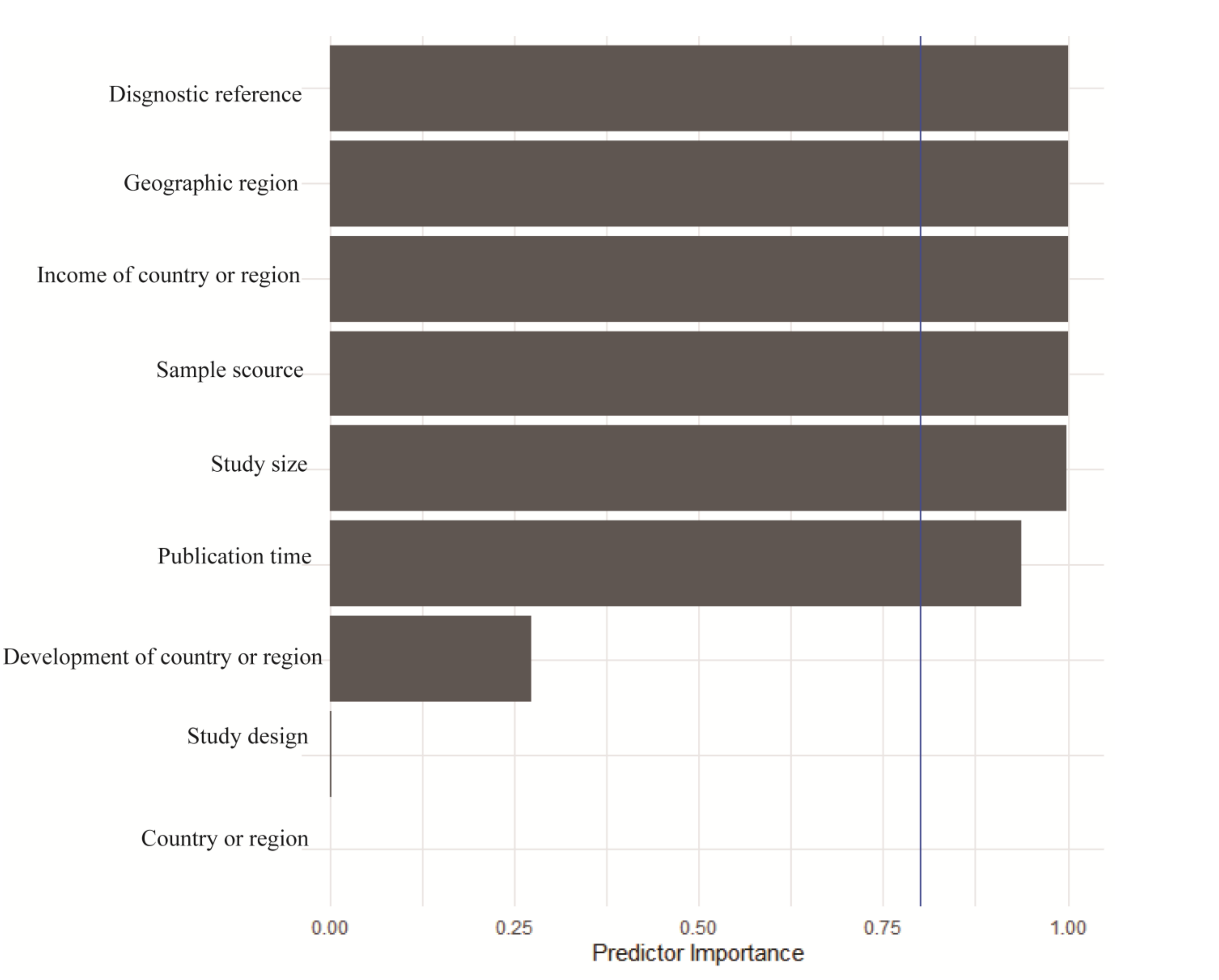

eTable 10. Subgroup analysis for overweight prevalence among children and adolescents.

Country	Number of studies combined: k = 2008<br><br>proportion 95%-CI<br>Random effects model 0.0848 [0.0820; 0.0877]<br><br>Quantifying heterogeneity:<br>tau^2 = 0.0129 [<0.0000; <0.0000]; tau = 0.1137 [<0.0000; <0.0000];<br>I^2 = 99.9% [99.9%; 99.9%]; H = 33.39 [33.28; 33.51]<br><br>Quantifying residual heterogeneity:<br>I^2 = 99.6% [99.5%; 99.6%]; H = 14.99 [14.90; 15.09]<br><br>Test of heterogeneity:<br>Q d.f. p-value<br>2238148.73 2007 0
Geographic region	Number of studies combined: k = 2008<br><br>proportion 95%-CI<br>Random effects model 0.0848 [0.0820; 0.0877]

© 2024 Zhang X et al. *JAMA Pediatrics.*

	Quantifying heterogeneity:<br>tau^2 = 0.0129 [<0.0000; <0.0000]; tau = 0.1137 [<0.0000; <0.0000];<br>I^2 = 99.9% [99.9%; 99.9%]; H = 33.39 [33.28; 33.51]<br><br>Quantifying residual heterogeneity:<br>I^2 = 99.8% [99.8%; 99.8%]; H = 21.31 [21.21; 21.41]<br><br>Test of heterogeneity:<br>Q d.f. p-value<br>2238148.73 2007 0
Development	Number of studies combined: k = 2008<br><br>proportion 95%-CI<br>Random effects model 0.0848 [0.0820; 0.0877]<br><br>Quantifying heterogeneity:<br>tau^2 = 0.0129 [<0.0000; <0.0000]; tau = 0.1137 [<0.0000; <0.0000];<br>I^2 = 99.9% [99.9%; 99.9%]; H = 33.39 [33.28; 33.51]<br><br>Quantifying residual heterogeneity:<br>I^2 = 99.8% [99.8%; 99.8%]; H = 24.53 [24.42; 24.63]<br><br>Test of heterogeneity:<br>Q d.f. p-value<br>2238148.73 2007 0
Country or region income	Number of studies combined: k = 2008<br><br>proportion 95%-CI<br>Random effects model 0.0848 [0.0820; 0.0877]<br><br>Quantifying heterogeneity:<br>tau^2 = 0.0129 [<0.0000; <0.0000]; tau = 0.1137 [<0.0000; <0.0000];<br>I^2 = 99.9% [99.9%; 99.9%]; H = 33.39 [33.28; 33.51]<br><br>Quantifying residual heterogeneity:<br>I^2 = 99.9% [99.9%; 99.9%]; H = 27.13 [27.02; 27.24]<br><br>Test of heterogeneity:<br>Q d.f. p-value<br>2238148.73 2007 0
Sample source	Number of studies combined: k = 2008<br><br>proportion 95%-CI<br>Random effects model 0.0848 [0.0820; 0.0877]<br><br>Quantifying heterogeneity:<br>tau^2 = 0.0129 [<0.0000; <0.0000]; tau = 0.1137 [<0.0000; <0.0000];<br>I^2 = 99.9% [99.9%; 99.9%]; H = 33.39 [33.28; 33.51]<br><br>Quantifying residual heterogeneity:<br>I^2 = 99.9% [99.9%; 99.9%]; H = 29.15 [29.04; 29.25]<br><br>Test of heterogeneity:<br>Q d.f. p-value<br>2238148.73 2007 0
Study design	Number of studies combined: k = 2008

© 2024 Zhang X et al. *JAMA Pediatrics.*

	proportion 95%-CI<br>Random effects model 0.0848 [0.0820; 0.0877]<br><br>Quantifying heterogeneity:<br>tau^2 = 0.0129 [<0.0000; <0.0000]; tau = 0.1137 [<0.0000; <0.0000];<br>I^2 = 99.9% [99.9%; 99.9%]; H = 33.39 [33.28; 33.51]<br><br>Quantifying residual heterogeneity:<br>I^2 = 99.9% [99.9%; 99.9%]; H = 31.49 [31.38; 31.60]<br><br>Test of heterogeneity:<br>Q d.f. p-value<br>2238148.73 2007 0
Diagnostic reference	Number of studies combined: k = 2008<br><br>proportion 95%-CI<br>Random effects model 0.0848 [0.0820; 0.0877]<br><br>Quantifying heterogeneity:<br>tau^2 = 0.0129 [<0.0000; <0.0000]; tau = 0.1137 [<0.0000; <0.0000];<br>I^2 = 99.9% [99.9%; 99.9%]; H = 33.39 [33.28; 33.51]<br><br>Quantifying residual heterogeneity:<br>I^2 = 99.9% [99.9%; 99.9%]; H = 32.06 [31.95; 32.18]<br><br>Test of heterogeneity:<br>Q d.f. p-value<br>2238148.73 2007 0
Sample size	Number of studies combined: k = 2008<br><br>proportion 95%-CI<br>Random effects model 0.0848 [0.0820; 0.0877]<br><br>Quantifying heterogeneity:<br>tau^2 = 0.0129 [<0.0000; <0.0000]; tau = 0.1137 [<0.0000; <0.0000];<br>I^2 = 99.9% [99.9%; 99.9%]; H = 33.39 [33.28; 33.51]<br><br>Quantifying residual heterogeneity:<br>I^2 = 99.9% [99.9%; 99.9%]; H = 33.31 [33.20; 33.42]<br><br>Test of heterogeneity:<br>Q d.f. p-value<br>2238148.73 2007 0
Study period	Number of studies combined: k = 1913<br><br>proportion 95%-CI<br>Random effects model 0.0848 [0.0820; 0.0877]<br><br>Quantifying heterogeneity:<br>tau^2 = 0.0125 [<0.0000; <0.0000]; tau = 0.1118 [<0.0000; <0.0000];<br>I^2 = 99.9% [99.9%; 99.9%]; H = 30.66 [30.55; 30.78]<br><br>Quantifying residual heterogeneity:<br>I^2 = 99.8% [99.8%; 99.8%]; H = 25.65 [25.55; 25.76]<br><br>Test of heterogeneity:<br>Q d.f. p-value

© 2024 Zhang X et al. *JAMA Pediatrics.*

	1797916.95 1912 0

eTable 11. Analysis of risk factors for obesity in children and adolescents.

Age	Number of studies combined: k = 1577<br><br>proportion 95%-CI<br>Random effects model 0.0839 [0.0808; 0.0870]<br><br>Quantifying heterogeneity:<br>tau^2 = 0.0122 [<0.0000; <0.0000]; tau = 0.1105 [<0.0000; <0.0000];<br>I^2 = 99.9% [99.9%; 99.9%]; H = 28.33 [28.21; 28.45]<br><br>Quantifying residual heterogeneity:<br>I^2 = 99.9% [99.9%; 99.9%]; H = 26.91 [26.79; 27.03]<br><br>Test of heterogeneity:<br>Q d.f. p-value<br>1264868.90 1576 0
Gender	Number of studies combined: k = 2163<br><br>proportion 95%-CI<br>Random effects model 0.0841 [0.0814; 0.0868]<br><br>Quantifying heterogeneity:<br>tau^2 = 0.0128 [<0.0000; <0.0000]; tau = 0.1133 [<0.0000; <0.0000];<br>I^2 = 99.8% [99.8%; 99.8%]; H = 21.09 [21.00; 21.19]<br><br>Quantifying residual heterogeneity:<br>I^2 = 99.8% [99.8%; 99.8%]; H = 20.46 [20.37; 20.56]<br><br>Test of heterogeneity:<br>Q d.f. p-value<br>961877.64 2162 0
Ethnicity	Number of studies combined: k = 177<br><br>proportion 95%-CI<br>Random effects model 0.1532 [0.1416; 0.1651]<br><br>Quantifying heterogeneity:<br>tau^2 = 0.0110 [0.0082; 0.0191]; tau = 0.1047 [0.0904; 0.1381];<br>I^2 = 99.7% [99.7%; 99.7%]; H = 19.37 [19.05; 19.70]<br><br>Quantifying residual heterogeneity:<br>I^2 = 99.5% [99.5%; 99.5%]; H = 14.59 [14.29; 14.90]<br><br>Test of heterogeneity:<br>Q d.f. p-value<br>66061.74 176 0
Residential location	Number of studies combined: k = 171<br><br>proportion 95%-CI<br>Random effects model 0.0727 [0.0645; 0.0813]<br><br>Quantifying heterogeneity:<br>tau^2 = 0.0110 [0.0088; 0.0205]; tau = 0.1050 [0.0940; 0.1432];

© 2024 Zhang X et al. *JAMA Pediatrics*.

	I^2 = 99.9% [99.9%; 99.9%]; H = 31.08 [30.71; 31.46]<br><br>Quantifying residual heterogeneity:<br>I^2 = 99.9% [99.9%; 99.9%]; H = 30.84 [30.46; 31.22]<br><br>Test of heterogeneity:<br>Q d.f. p-value<br>164225.61 170 0
School type	Number of studies combined: k = 82<br><br>proportion 95%-CI<br>Random effects model 0.0883 [0.0762; 0.1011]<br><br>Quantifying heterogeneity:<br>tau^2 = 0.0096 [0.0110; 0.0262]; tau = 0.0979 [0.1048; 0.1619];<br>I^2 = 98.8% [98.7%; 98.9%]; H = 9.14 [8.77; 9.53]<br><br>Quantifying residual heterogeneity:<br>I^2 = 98.8% [98.7%; 98.9%]; H = 9.12 [8.74; 9.51]<br><br>Test of heterogeneity:<br>Q d.f. p-value<br>6767.81 81 0
Paternal weight status	Number of studies combined: k = 26<br><br>proportion 95%-CI<br>Random effects model 0.0943 [0.0641; 0.1294]<br><br>Quantifying heterogeneity:<br>tau^2 = 0.0201 [0.0107; 0.0403]; tau = 0.1419 [0.1034; 0.2008];<br>I^2 = 99.2% [99.0%; 99.3%]; H = 10.85 [10.15; 11.60]<br><br>Quantifying residual heterogeneity:<br>I^2 = 99.0% [98.8%; 99.1%]; H = 10.00 [9.30; 10.75]<br><br>Test of heterogeneity:<br>Q d.f. p-value<br>2942.92 25 0
Maternal weight status	Number of studies combined: k = 52<br><br>proportion 95%-CI<br>Random effects model 0.1156 [0.0919; 0.1417]<br><br>Quantifying heterogeneity:<br>tau^2 = 0.0193 [0.0115; 0.0328]; tau = 0.1389 [0.1074; 0.1811];<br>I^2 = 99.6% [99.5%; 99.6%]; H = 15.21 [14.66; 15.79]<br><br>Quantifying residual heterogeneity:<br>I^2 = 99.6% [99.5%; 99.6%]; H = 14.98 [14.42; 15.56]<br><br>Test of heterogeneity:<br>Q d.f. p-value<br>11799.59 51 0
Maternal diabetes	Number of studies combined: k = 12<br><br>proportion 95%-CI<br>Random effects model 0.0952 [0.0563; 0.1424]

© 2024 Zhang X et al. *JAMA Pediatrics.*

	Quantifying heterogeneity:<br>tau^2 = 0.0142 [0.0073; 0.1177]; tau = 0.1192 [0.0855; 0.3430];<br>I^2 = 99.4% [99.3%; 99.5%]; H = 12.75 [11.65; 13.96]<br><br>Quantifying residual heterogeneity:<br>I^2 = 99.4% [99.2%; 99.5%]; H = 12.62 [11.47; 13.88]<br><br>Test of heterogeneity:<br>Q d.f. p-value<br>1789.01 11 0
Gestational diabetes	Number of studies combined: k = 10<br><br>proportion 95%-CI<br>Random effects model 0.1568 [0.1134; 0.2055]<br><br>Quantifying heterogeneity:<br>tau^2 = 0.0093 [0.0041; 0.0468]; tau = 0.0965 [0.0642; 0.2164];<br>I^2 = 98.3% [97.7%; 98.7%]; H = 7.65 [6.65; 8.81]<br><br>Quantifying residual heterogeneity:<br>I^2 = 98.5% [98.0%; 98.8%]; H = 8.07 [6.98; 9.32]<br><br>Test of heterogeneity:<br>Q d.f. p-value<br>526.75 9 < 0.0001
Paternal diabetes	Number of studies combined: k = 4<br><br>proportion 95%-CI<br>Random effects model 0.0672 [0.0393; 0.1017]<br><br>Quantifying heterogeneity:<br>tau^2 = 0.0034 [0.0040; 0.4810]; tau = 0.0582 [0.0633; 0.6936];<br>I^2 = 99.2% [98.8%; 99.4%]; H = 10.96 [9.06; 13.26]<br><br>Quantifying residual heterogeneity:<br>I^2 = 99.1% [98.6%; 99.5%]; H = 10.77 [8.51; 13.63]<br><br>Test of heterogeneity:<br>Q d.f. p-value<br>360.39 3 < 0.0001
Maternal education	Number of studies combined: k = 92<br><br>proportion 95%-CI<br>Random effects model 0.1012 [0.0868; 0.1164]<br><br>Quantifying heterogeneity:<br>tau^2 = 0.0118 [0.0089; 0.0200]; tau = 0.1085 [0.0944; 0.1415];<br>I^2 = 98.3% [98.1%; 98.4%]; H = 7.59 [7.26; 7.93]<br><br>Quantifying residual heterogeneity:<br>I^2 = 98.0% [97.8%; 98.2%]; H = 7.03 [6.71; 7.37]<br><br>Test of heterogeneity:<br>Q d.f. p-value<br>5239.55 91 0
Paternal	Number of studies: k = 63

© 2024 Zhang X et al. *JAMA Pediatrics*.

education	Number of observations: o = 57923<br>Number of events: e = 3640<br><br>proportion 95%-CI<br>Common effect model 0.0548 [0.0529; 0.0567]<br>Random effects model 0.0793 [0.0662; 0.0934]<br><br>Quantifying heterogeneity:<br>tau^2 = 0.0088 [0.0062; 0.0139]; tau = 0.0941 [0.0785; 0.1177]<br>I^2 = 96.1% [95.5%; 96.6%]; H = 5.05 [4.71; 5.42]<br><br>Test of heterogeneity:<br>Q d.f. p-value<br>1580.98 62 < 0.0001
Mother occupation	Number of studies combined: k = 40<br><br>proportion 95%-CI<br>Random effects model 0.0904 [0.0701; 0.1127]<br><br>Quantifying heterogeneity:<br>tau^2 = 0.0119 [0.0063; 0.0221]; tau = 0.1091 [0.0796; 0.1486];<br>I^2 = 98.5% [98.3%; 98.7%]; H = 8.29 [7.77; 8.84]<br><br>Quantifying residual heterogeneity:<br>I^2 = 98.5% [98.2%; 98.6%]; H = 8.05 [7.53; 8.60]<br><br>Test of heterogeneity:<br>Q d.f. p-value<br>2678.54 39 0
Father occupation	Number of studies combined: k = 14<br><br>proportion 95%-CI<br>Random effects model 0.0599 [0.0382; 0.0858]<br><br>Quantifying heterogeneity:<br>tau^2 = 0.0072 [0.0044; 0.0333]; tau = 0.0849 [0.0661; 0.1826];<br>I^2 = 96.3% [95.1%; 97.3%]; H = 5.23 [4.50; 6.07]<br><br>Quantifying residual heterogeneity:<br>I^2 = 96.6% [95.4%; 97.5%]; H = 5.40 [4.64; 6.29]<br><br>Test of heterogeneity:<br>Q d.f. p-value<br>355.54 13 < 0.0001
Parents' marriage	Number of studies combined: k = 8<br><br>proportion 95%-CI<br>Random effects model 0.0121 [0.0088; 0.0158]<br><br>Quantifying heterogeneity:<br>tau^2 = 0.0002 [0.0007; 0.0241]; tau = 0.0152 [0.0264; 0.1551];<br>I^2 = 98.5% [98.0%; 98.9%]; H = 8.15 [6.99; 9.50]<br><br>Quantifying residual heterogeneity:<br>I^2 = 98.4% [97.8%; 98.9%]; H = 7.99 [6.76; 9.44]<br><br>Test of heterogeneity:

© 2024 Zhang X et al. *JAMA Pediatrics*.

	Q d.f. p-value<br>465.02 7 < 0.0001
Caregivers	Number of studies combined: k = 4<br><br>proportion 95%-CI<br>Random effects model 0.0944 [0.0343; 0.1778]<br><br>Quantifying heterogeneity:<br>tau^2 = 0.0123 [0.0035; 0.2683]; tau = 0.1108 [0.0591; 0.5180];<br>I^2 = 87.9% [71.5%; 94.9%]; H = 2.88 [1.87; 4.43]<br><br>Quantifying residual heterogeneity:<br>I^2 = 89.4% [71.4%; 96.1%]; H = 3.08 [1.87; 5.06]<br><br>Test of heterogeneity:<br>Q d.f. p-value<br>24.87 3 < 0.0001
Number of children in the family	Number of studies combined: k = 24<br><br>proportion 95%-CI<br>Random effects model 0.0849 [0.0560; 0.1190]<br><br>Quantifying heterogeneity:<br>tau^2 = 0.0187 [0.0089; 0.0359]; tau = 0.1369 [0.0946; 0.1894];<br>I^2 = 99.4% [99.3%; 99.4%]; H = 12.59 [11.82; 13.41]<br><br>Quantifying residual heterogeneity:<br>I^2 = 99.2% [99.1%; 99.3%]; H = 11.46 [10.70; 12.28]<br><br>Test of heterogeneity:<br>Q d.f. p-value<br>3643.49 23 0
Birth order	Number of studies combined: k = 14<br><br>proportion 95%-CI<br>Random effects model 0.0483 [0.0335; 0.0656]<br><br>Quantifying heterogeneity:<br>tau^2 = 0.0049 [0.0027; 0.0163]; tau = 0.0699 [0.0518; 0.1275];<br>I^2 = 98.5% [98.2%; 98.8%]; H = 8.22 [7.35; 9.20]<br><br>Quantifying residual heterogeneity:<br>I^2 = 98.6% [98.3%; 98.9%]; H = 8.52 [7.61; 9.55]<br><br>Test of heterogeneity:<br>Q d.f. p-value<br>878.86 13 < 0.0001
Socioeconomic status of family	Number of studies combined: k = 93<br><br>proportion 95%-CI<br>Random effects model 0.0740 [0.0668; 0.0816]<br><br>Quantifying heterogeneity:<br>tau^2 = 0.0040 [0.0058; 0.0145]; tau = 0.0631 [0.0761; 0.1205];<br>I^2 = 97.9% [97.6%; 98.1%]; H = 6.84 [6.52; 7.17]

© 2024 Zhang X et al. *JAMA Pediatrics*.

	Quantifying residual heterogeneity:<br>I^2 = 97.9% [97.7%; 98.1%]; H = 6.89 [6.57; 7.23]<br><br>Test of heterogeneity:<br>Q d.f. p-value<br>4300.76 92 0
Way of going to school	Number of studies combined: k = 25<br><br>proportion 95%-CI<br>Random effects model 0.0798 [0.0584; 0.1041]<br><br>Quantifying heterogeneity:<br>tau^2 = 0.0099 [0.0062; 0.0234]; tau = 0.0995 [0.0786; 0.1531];<br>I^2 = 94.0% [92.2%; 95.3%]; H = 4.07 [3.58; 4.63]<br><br>Quantifying residual heterogeneity:<br>I^2 = 93.5% [91.4%; 95.1%]; H = 3.92 [3.41; 4.50]<br><br>Test of heterogeneity:<br>Q d.f. p-value<br>397.76 24 < 0.0001
Breakfast	Number of studies combined: k = 48<br><br>proportion 95%-CI<br>Random effects model 0.0839 [0.0690; 0.1000]<br><br>Quantifying heterogeneity:<br>tau^2 = 0.0084 [0.0053; 0.0149]; tau = 0.0916 [0.0729; 0.1219];<br>I^2 = 97.8% [97.5%; 98.1%]; H = 6.76 [6.32; 7.23]<br><br>Quantifying residual heterogeneity:<br>I^2 = 97.8% [97.4%; 98.0%]; H = 6.67 [6.23; 7.15]<br><br>Test of heterogeneity:<br>Q d.f. p-value<br>2148.92 47 0
Number of meals per day	Number of studies combined: k = 9<br><br>proportion 95%-CI<br>Random effects model 0.0727 [0.0385; 0.1165]<br><br>Quantifying heterogeneity:<br>tau^2 = 0.0120 [0.0060; 0.0582]; tau = 0.1097 [0.0773; 0.2412];<br>I^2 = 95.7% [93.6%; 97.1%]; H = 4.83 [3.95; 5.89]<br><br>Quantifying residual heterogeneity:<br>I^2 = 93.0% [88.4%; 95.7%]; H = 3.77 [2.94; 4.83]<br><br>Test of heterogeneity:<br>Q d.f. p-value<br>186.26 8 < 0.0001
Watching TV while eating	Number of studies combined: k = 12<br><br>proportion 95%-CI<br>Random effects model 0.2310 [0.0952; 0.4033]

© 2024 Zhang X et al. *JAMA Pediatrics.*

	Quantifying heterogeneity:<br>tau^2 = 0.1060 [0.0532; 0.3440]; tau = 0.3255 [0.2306; 0.5865];<br>I^2 = 99.1% [98.9%; 99.3%]; H = 10.57 [9.53; 11.71]<br><br>Quantifying residual heterogeneity:<br>I^2 = 99.1% [98.9%; 99.3%]; H = 10.54 [9.46; 11.74]<br><br>Test of heterogeneity:<br>Q d.f. p-value<br>1228.10 11 < 0.0001
Hours of watching TV per day	Number of studies combined: k = 38<br><br>proportion 95%-CI<br>Random effects model 0.1547 [0.1260; 0.1858]<br><br>Quantifying heterogeneity:<br>tau^2 = 0.0162 [0.0178; 0.0593]; tau = 0.1271 [0.1333; 0.2434];<br>I^2 = 99.2% [99.1%; 99.3%]; H = 11.39 [10.79; 12.01]<br><br>Quantifying residual heterogeneity:<br>I^2 = 99.2% [99.1%; 99.3%]; H = 11.08 [10.49; 11.71]<br><br>Test of heterogeneity:<br>Q d.f. p-value<br>4796.27 37 0
Hours of playing computer per day	Number of studies combined: k = 6<br><br>proportion 95%-CI<br>Random effects model 0.0835 [0.0459; 0.1309]<br><br>Quantifying heterogeneity:<br>tau^2 = 0.0090 [0.0026; 0.0473]; tau = 0.0949 [0.0505; 0.2175];<br>I^2 = 98.6% [98.0%; 99.0%]; H = 8.38 [7.02; 10.01]<br><br>Quantifying residual heterogeneity:<br>I^2 = 94.9% [90.8%; 97.2%]; H = 4.43 [3.30; 5.95]<br><br>Test of heterogeneity:<br>Q d.f. p-value<br>351.30 5 < 0.0001
Passive smoking	Number of studies combined: k = 16<br><br>proportion 95%-CI<br>Random effects model 0.0802 [0.0519; 0.1139]<br><br>Quantifying heterogeneity:<br>tau^2 = 0.0132 [0.0066; 0.0403]; tau = 0.1150 [0.0813; 0.2008];<br>I^2 = 99.6% [99.6%; 99.7%]; H = 16.89 [15.85; 18.00]<br><br>Quantifying residual heterogeneity:<br>I^2 = 99.5% [99.5%; 99.6%]; H = 14.86 [13.82; 15.97]<br><br>Test of heterogeneity:<br>Q d.f. p-value<br>4280.76 15 0
Maternal smoking in	Number of studies combined: k = 44

© 2024 Zhang X et al. *JAMA Pediatrics.*

pregnancy	proportion 95%-CI<br>Random effects model 0.0591 [0.0483; 0.0709]<br><br>Quantifying heterogeneity:<br>tau^2 = 0.0055 [0.0049; 0.0142]; tau = 0.0744 [0.0702; 0.1193];<br>I^2 = 97.4% [97.0%; 97.7%]; H = 6.17 [5.73; 6.65]<br><br>Quantifying residual heterogeneity:<br>I^2 = 97.2% [96.8%; 97.6%]; H = 5.99 [5.55; 6.47]<br><br>Test of heterogeneity:<br>Q d.f. p-value<br>1639.25 43 < 0.0001
Gestational weight gain	Number of studies combined: k = 12<br><br>proportion 95%-CI<br>Random effects model 0.1264 [0.0936; 0.1634]<br><br>Quantifying heterogeneity:<br>tau^2 = 0.0077 [0.0027; 0.0259]; tau = 0.0876 [0.0520; 0.1611];<br>I^2 = 99.1% [98.9%; 99.3%]; H = 10.74 [9.70; 11.89]<br><br>Quantifying residual heterogeneity:<br>I^2 = 99.2% [99.0%; 99.4%]; H = 11.17 [10.02; 12.46]<br><br>Test of heterogeneity:<br>Q d.f. p-value<br>1268.84 11 < 0.0001
Maternal age at birth	Number of studies combined: k = 14<br><br>proportion 95%-CI<br>Random effects model 0.0680 [0.0460; 0.0938]<br><br>Quantifying heterogeneity:<br>tau^2 = 0.0076 [0.0033; 0.0230]; tau = 0.0869 [0.0576; 0.1515];<br>I^2 = 98.9% [98.7%; 99.1%]; H = 9.59 [8.67; 10.61]<br><br>Quantifying residual heterogeneity:<br>I^2 = 98.8% [98.5%; 99.0%]; H = 8.97 [8.04; 10.01]<br><br>Test of heterogeneity:<br>Q d.f. p-value<br>1195.99 13 < 0.0001
Term of delivery	Number of studies combined: k = 28<br><br>proportion 95%-CI<br>Random effects model 0.1059 [0.0897; 0.1232]<br><br>Quantifying heterogeneity:<br>tau^2 = 0.0048 [0.0028; 0.0141]; tau = 0.0690 [0.0527; 0.1187];<br>I^2 = 99.3% [99.2%; 99.4%]; H = 12.06 [11.35; 12.80]<br><br>Quantifying residual heterogeneity:<br>I^2 = 99.3% [99.2%; 99.4%]; H = 12.25 [11.53; 13.01]<br><br>Test of heterogeneity:<br>Q d.f. p-value

© 2024 Zhang X et al. *JAMA Pediatrics*.

	3924.44 27 0
Type of delivery	Number of studies combined: k = 28<br><br>proportion 95%-CI<br>Random effects model 0.0907 [0.0772; 0.1052]<br><br>Quantifying heterogeneity:<br>tau^2 = 0.0038 [0.0027; 0.0096]; tau = 0.0616 [0.0516; 0.0977];<br>I^2 = 95.3% [94.1%; 96.2%]; H = 4.59 [4.10; 5.14]<br><br>Quantifying residual heterogeneity:<br>I^2 = 95.4% [94.2%; 96.3%]; H = 4.67 [4.17; 5.23]<br><br>Test of heterogeneity:<br>Q d.f. p-value<br>569.02 27 < 0.0001
Birth weight	Number of studies combined: k = 49<br><br>proportion 95%-CI<br>Random effects model 0.0914 [0.0777; 0.1061]<br><br>Quantifying heterogeneity:<br>tau^2 = 0.0065 [0.0058; 0.0205]; tau = 0.0809 [0.0760; 0.1430];<br>I^2 = 99.2% [99.1%; 99.3%]; H = 11.06 [10.54; 11.60]<br><br>Quantifying residual heterogeneity:<br>I^2 = 99.0% [98.9%; 99.1%]; H = 10.24 [9.73; 10.78]<br><br>Test of heterogeneity:<br>Q d.f. p-value<br>5866.53 48 0
Duration of breastfeeding	Number of studies combined: k = 17<br><br>proportion 95%-CI<br>Random effects model 0.0887 [0.0675; 0.1124]<br><br>Quantifying heterogeneity:<br>tau^2 = 0.0065 [0.0036; 0.0186]; tau = 0.0804 [0.0600; 0.1362];<br>I^2 = 98.3% [97.8%; 98.6%]; H = 7.58 [6.81; 8.43]<br><br>Quantifying residual heterogeneity:<br>I^2 = 98.3% [97.9%; 98.6%]; H = 7.66 [6.87; 8.55]<br><br>Test of heterogeneity:<br>Q d.f. p-value<br>919.17 16 < 0.0001
Breastfeeding package	Number of studies combined: k = 28<br><br>proportion 95%-CI<br>Random effects model 0.0957 [0.0792; 0.1135]<br><br>Quantifying heterogeneity:<br>tau^2 = 0.0056 [0.0034; 0.0140]; tau = 0.0747 [0.0585; 0.1185];<br>I^2 = 99.1% [99.0%; 99.2%]; H = 10.58 [9.91; 11.30]<br><br>Quantifying residual heterogeneity:<br>I^2 = 99.0% [98.9%; 99.2%]; H = 10.19 [9.50; 10.93]

© 2024 Zhang X et al. *JAMA Pediatrics.*

	Test of heterogeneity:<br>Q d.f. p-value<br>3022.70 27 0
Antibiotic exposure	Number of studies combined: k = 12<br><br>proportion 95%-CI<br>Random effects model 0.0736 [0.0587; 0.0899]<br><br>Quantifying heterogeneity:<br>tau^2 = 0.0021 [0.0023; 0.0364]; tau = 0.0462 [0.0481; 0.1907];<br>I^2 = 99.4% [99.2%; 99.5%]; H = 12.59 [11.49; 13.79]<br><br>Quantifying residual heterogeneity:<br>I^2 = 99.4% [99.3%; 99.5%]; H = 12.94 [11.78; 14.21]<br><br>Test of heterogeneity:<br>Q d.f. p-value<br>1742.56 11 0
Physical activity	Number of studies combined: k = 42<br><br>proportion 95%-CI<br>Random effects model 0.0975 [0.0824; 0.1137]<br><br>Quantifying heterogeneity:<br>tau^2 = 0.0069 [0.0057; 0.0161]; tau = 0.0832 [0.0755; 0.1270];<br>I^2 = 97.2% [96.8%; 97.6%]; H = 6.03 [5.58; 6.51]<br><br>Quantifying residual heterogeneity:<br>I^2 = 97.2% [96.7%; 97.6%]; H = 5.97 [5.52; 6.46]<br><br>Test of heterogeneity:<br>Q d.f. p-value<br>1489.17 41 < 0.0001
Sleep duration	Number of studies combined: k = 24<br><br>proportion 95%-CI<br>Random effects model 0.1070 [0.0772; 0.1411]<br><br>Quantifying heterogeneity:<br>tau^2 = 0.0161 [0.0115; 0.0456]; tau = 0.1267 [0.1073; 0.2136];<br>I^2 = 99.1% [99.0%; 99.2%]; H = 10.61 [9.89; 11.39]<br><br>Quantifying residual heterogeneity:<br>I^2 = 99.0% [98.8%; 99.1%]; H = 9.98 [9.26; 10.77]<br><br>Test of heterogeneity:<br>Q d.f. p-value<br>2591.19 23 0

P value-adjusted analysis for multiple comparisons in risk factors

1. Age (in years)

Age (in years)	0-5	6-12
0-5	—	—
6-12	< 0.001	—
13-18	0.001	< 0.001

© 2024 Zhang X et al. *JAMA Pediatrics.*

0-5 v.s. 6-12	Number of studies combined: k = 1331<br><br>proportion 95%-CI<br>Fixed effect model 0.0932 [0.0931; 0.0934]<br>Random effects model 0.0837 [0.0806; 0.0869]<br><br>Quantifying heterogeneity:<br>tau^2 = 0.0106 [<0.0000; <0.0000]; tau = 0.1031 [<0.0000; <0.0000];<br>I^2 = 99.8% [99.8%; 99.8%]; H = 24.01 [23.88; 24.14]<br><br>Quantifying residual heterogeneity:<br>I^2 = 99.8% [99.8%; 99.8%]; H = 23.88 [23.75; 24.01]<br><br>Test of heterogeneity:<br>Q d.f. p-value<br>766679.43 1330 0
0-5 v.s. 13-18	Number of studies combined: k = 761<br><br>proportion 95%-CI<br>Fixed effect model 0.0732 [0.0731; 0.0733]<br>Random effects model 0.0741 [0.0701; 0.0781]<br><br>Quantifying heterogeneity:<br>tau^2 = 0.0111 [<0.0000; <0.0000]; tau = 0.1055 [<0.0000; <0.0000];<br>I^2 = 99.9% [99.9%; 99.9%]; H = 31.88 [31.70; 32.06]<br><br>Quantifying residual heterogeneity:<br>I^2 = 99.9% [99.9%; 99.9%]; H = 30.38 [30.21; 30.56]<br><br>Test of heterogeneity:<br>Q d.f. p-value<br>772307.95 760 0
6-12 v.s. 13-18	Number of studies combined: k = 1331<br><br>proportion 95%-CI<br>Fixed effect model 0.0932 [0.0931; 0.0934]<br>Random effects model 0.0837 [0.0806; 0.0869]<br><br>Quantifying heterogeneity:<br>tau^2 = 0.0106 [<0.0000; <0.0000]; tau = 0.1031 [<0.0000; <0.0000];<br>I^2 = 99.8% [99.8%; 99.8%]; H = 24.01 [23.88; 24.14]<br><br>Quantifying residual heterogeneity:<br>I^2 = 99.8% [99.8%; 99.8%]; H = 23.88 [23.75; 24.01]<br><br>Test of heterogeneity:<br>Q d.f. p-value<br>766679.43 1330 0

2. Residential location

Residential location	Rural	Urban
Rural	—	—
Urban	0.05	—

© 2024 Zhang X et al. *JAMA Pediatrics*.

Suburban	0.37	0.94

Rural v.s. Urban	Number of studies combined: k = 157<br><br>proportion 95%-CI<br>Fixed effect model 0.0236 [0.0235; 0.0238]<br>Random effects model 0.0721 [0.0637; 0.0810]<br><br>Quantifying heterogeneity:<br>tau^2 = 0.0108 [0.0086; 0.0210]; tau = 0.1039 [0.0928; 0.1448];<br>I^2 = 99.9% [99.9%; 99.9%]; H = 31.35 [30.95; 31.74]<br><br>Quantifying residual heterogeneity:<br>I^2 = 99.9% [99.9%; 99.9%]; H = 31.38 [30.99; 31.78]<br><br>Test of heterogeneity:<br>Q d.f. p-value<br>153289.62 156 0
Rural v.s. Suburban	Number of studies combined: k = 88<br><br>proportion 95%-CI<br>Fixed effect model 0.0234 [0.0232; 0.0237]<br>Random effects model 0.0651 [0.0542; 0.0769]<br><br>Quantifying heterogeneity:<br>tau^2 = 0.0114 [0.0072; 0.0204]; tau = 0.1067 [0.0846; 0.1427];<br>I^2 = 99.8% [99.8%; 99.8%]; H = 24.45 [23.96; 24.95]<br><br>Quantifying residual heterogeneity:<br>I^2 = 99.8% [99.8%; 99.8%]; H = 23.53 [23.04; 24.03]<br><br>Test of heterogeneity:<br>Q d.f. p-value<br>51991.86 87 0
Urban v.s. Suburban	Number of studies combined: k = 97<br><br>proportion 95%-CI<br>Fixed effect model 0.0263 [0.0261; 0.0265]<br>Random effects model 0.0809 [0.0683; 0.0945]<br><br>Quantifying heterogeneity:<br>tau^2 = 0.0142 [0.0088; 0.0261]; tau = 0.1190 [0.0937; 0.1616];<br>I^2 = 99.9% [99.9%; 99.9%]; H = 35.75 [35.23; 36.27]<br><br>Quantifying residual heterogeneity:<br>I^2 = 99.9% [99.9%; 99.9%]; H = 35.43 [34.92; 35.96]<br><br>Test of heterogeneity:<br>Q d.f. p-value<br>122676.91 96 0

3. Maternal education

Maternal education	Less than secondary	Secondary
Less than secondary	—	—
Secondary	0.93	—
Tertiary	0.47	0.36

© 2024 Zhang X et al. *JAMA Pediatrics*.

Less than secondary v.s. Secondary	Number of studies combined: k = 59<br><br>proportion 95%-CI<br>Fixed effect model 0.1083 [0.1059; 0.1107]<br>Random effects model 0.0927 [0.0757; 0.1109]<br><br>Quantifying heterogeneity:<br>tau^2 = 0.0108 [0.0078; 0.0216]; tau = 0.1040 [0.0885; 0.1468];<br>I^2 = 97.8% [97.5%; 98.1%]; H = 6.75 [6.35; 7.17]<br><br>Quantifying residual heterogeneity:<br>I^2 = 97.4% [97.0%; 97.7%]; H = 6.19 [5.80; 6.60]<br><br>Test of heterogeneity:<br>Q d.f. p-value<br>2639.75 58 0
Less than secondary v.s. Tertiary	Number of studies combined: k = 63<br><br>proportion 95%-CI<br>Fixed effect model 0.1230 [0.1205; 0.1256]<br>Random effects model 0.1030 [0.0829; 0.1249]<br><br>Quantifying heterogeneity:<br>tau^2 = 0.0160 [0.0102; 0.0263]; tau = 0.1267 [0.1008; 0.1621];<br>I^2 = 98.5% [98.3%; 98.7%]; H = 8.18 [7.77; 8.61]<br><br>Quantifying residual heterogeneity:<br>I^2 = 98.2% [97.9%; 98.4%]; H = 7.37 [6.97; 7.79]<br><br>Test of heterogeneity:<br>Q d.f. p-value<br>4149.90 62 0
Secondary v.s. Tertiary	Number of studies combined: k = 62<br><br>proportion 95%-CI<br>Fixed effect model 0.1436 [0.1414; 0.1459]<br>Random effects model 0.1076 [0.0917; 0.1245]<br><br>Quantifying heterogeneity:<br>tau^2 = 0.0098 [0.0068; 0.0183]; tau = 0.0991 [0.0826; 0.1351];<br>I^2 = 98.2% [98.0%; 98.4%]; H = 7.46 [7.06; 7.88]<br><br>Quantifying residual heterogeneity:<br>I^2 = 98.2% [98.0%; 98.4%]; H = 7.42 [7.01; 7.84]<br><br>Test of heterogeneity:<br>Q d.f. p-value<br>3393.36 61 0

4. Paternal education

Paternal education	Less than secondary	Secondary
Less than secondary	—	—
Secondary	0.22	—
Tertiary	0.45	0.81

© 2024 Zhang X et al. *JAMA Pediatrics.*

Less than secondary v.s. Secondary	Number of studies combined: k = 43<br><br>proportion 95%-CI<br>Fixed effect model 0.0652 [0.0623; 0.0681]<br>Random effects model 0.0769 [0.0645; 0.0903]<br><br>Quantifying heterogeneity:<br>tau^2 = 0.0051 [0.0039; 0.0118]; tau = 0.0712 [0.0628; 0.1087];<br>I^2 = 92.9% [91.3%; 94.2%]; H = 3.76 [3.39; 4.16]<br><br>Quantifying residual heterogeneity:<br>I^2 = 93.1% [91.5%; 94.4%]; H = 3.80 [3.42; 4.21]<br><br>Test of heterogeneity:<br>Q d.f. p-value<br>592.94 42 < 0.0001
Less than secondary v.s. Tertiary	Number of studies combined: k = 42<br><br>proportion 95%-CI<br>Fixed effect model 0.0497 [0.0475; 0.0519]<br>Random effects model 0.0749 [0.0594; 0.0919]<br><br>Quantifying heterogeneity:<br>tau^2 = 0.0086 [0.0056; 0.0178]; tau = 0.0928 [0.0745; 0.1333];<br>I^2 = 96.6% [96.0%; 97.1%]; H = 5.44 [5.01; 5.91]<br><br>Quantifying residual heterogeneity:<br>I^2 = 96.5% [95.9%; 97.1%]; H = 5.37 [4.93; 5.84]<br><br>Test of heterogeneity:<br>Q d.f. p-value<br>1214.29 41 < 0.0001
Secondary v.s. Tertiary	Number of studies combined: k = 41<br><br>proportion 95%-CI<br>Fixed effect model 0.0532 [0.0511; 0.0553]<br>Random effects model 0.0850 [0.0692; 0.1023]<br><br>Quantifying heterogeneity:<br>tau^2 = 0.0083 [0.0053; 0.0168]; tau = 0.0911 [0.0731; 0.1297];<br>I^2 = 96.9% [96.3%; 97.3%]; H = 5.64 [5.20; 6.12]<br><br>Quantifying residual heterogeneity:<br>I^2 = 96.7% [96.1%; 97.2%]; H = 5.48 [5.04; 5.96]<br><br>Test of heterogeneity:<br>Q d.f. p-value<br>1273.11 40 < 0.0001

5. Socioeconomic status of family

Socioeconomic status of family	Low	Middle
Low	—	—
Middle	0.41	—
High	0.57	0.76

© 2024 Zhang X et al. *JAMA Pediatrics.*

Low v.s. Middle	Number of studies combined: k = 63<br><br>proportion 95%-CI<br>Fixed effect model 0.0436 [0.0427; 0.0445]<br>Random effects model 0.0740 [0.0643; 0.0842]<br><br>Quantifying heterogeneity:<br>tau^2 = 0.0052 [0.0054; 0.0157]; tau = 0.0722 [0.0736; 0.1253];<br>I^2 = 98.2% [98.0%; 98.4%]; H = 7.42 [7.03; 7.84]<br><br>Quantifying residual heterogeneity:<br>I^2 = 98.2% [98.0%; 98.4%]; H = 7.48 [7.08; 7.91]<br><br>Test of heterogeneity:<br>Q d.f. p-value<br>3417.22 62 0
Low v.s. High	Number of studies combined: k = 63<br><br>proportion 95%-CI<br>Fixed effect model 0.0451 [0.0442; 0.0460]<br>Random effects model 0.0719 [0.0625; 0.0819]<br><br>Quantifying heterogeneity:<br>tau^2 = 0.0048 [0.0059; 0.0180]; tau = 0.0695 [0.0768; 0.1341];<br>I^2 = 97.8% [97.6%; 98.1%]; H = 6.80 [6.42; 7.21]<br><br>Quantifying residual heterogeneity:<br>I^2 = 97.9% [97.6%; 98.1%]; H = 6.83 [6.44; 7.24]<br><br>Test of heterogeneity:<br>Q d.f. p-value<br>2867.51 62 0
Middle v.s. High	Number of studies combined: k = 60<br><br>proportion 95%-CI<br>Fixed effect model 0.0455 [0.0446; 0.0463]<br>Random effects model 0.0765 [0.0681; 0.0855]<br><br>Quantifying heterogeneity:<br>tau^2 = 0.0034 [0.0048; 0.0151]; tau = 0.0583 [0.0695; 0.1227];<br>I^2 = 97.4% [97.1%; 97.7%]; H = 6.25 [5.87; 6.66]<br><br>Quantifying residual heterogeneity:<br>I^2 = 97.5% [97.1%; 97.8%]; H = 6.28 [5.90; 6.69]<br><br>Test of heterogeneity:<br>Q d.f. p-value<br>2304.22 59 0

6. Way of going to school

Way of going to school	Walk	Bike
Walk	—	—
Bike	0.87	—
Car	0.08	0.09

© 2024 Zhang X et al. *JAMA Pediatrics*.

Walk v.s. Bike	Number of studies combined: k = 16<br><br>proportion 95%-CI<br>Fixed effect model 0.0554 [0.0500; 0.0609]<br>Random effects model 0.0628 [0.0425; 0.0866]<br><br>Quantifying heterogeneity:<br>tau^2 = 0.0070 [0.0034; 0.0194]; tau = 0.0837 [0.0582; 0.1393];<br>I^2 = 92.2% [89.0%; 94.5%]; H = 3.59 [3.01; 4.28]<br><br>Quantifying residual heterogeneity:<br>I^2 = 92.7% [89.7%; 94.9%]; H = 3.71 [3.11; 4.44]<br><br>Test of heterogeneity:<br>Q d.f. p-value<br>193.12 15 < 0.0001
Walk v.s. Car	Number of studies combined: k = 21<br><br>proportion 95%-CI<br>Fixed effect model 0.0691 [0.0639; 0.0744]<br>Random effects model 0.0837 [0.0594; 0.1116]<br><br>Quantifying heterogeneity:<br>tau^2 = 0.0106 [0.0066; 0.0280]; tau = 0.1030 [0.0811; 0.1673];<br>I^2 = 94.8% [93.2%; 96.0%]; H = 4.38 [3.83; 5.01]<br><br>Quantifying residual heterogeneity:<br>I^2 = 94.2% [92.2%; 95.6%]; H = 4.14 [3.59; 4.78]<br><br>Test of heterogeneity:<br>Q d.f. p-value<br>383.46 20 < 0.0001
Bike v.s. Car	Number of studies combined: k = 13<br><br>proportion 95%-CI<br>Fixed effect model 0.0897 [0.0807; 0.0991]<br>Random effects model 0.0956 [0.0611; 0.1366]<br><br>Quantifying heterogeneity:<br>tau^2 = 0.0122 [0.0061; 0.0397]; tau = 0.1103 [0.0780; 0.1991];<br>I^2 = 93.2% [90.0%; 95.3%]; H = 3.83 [3.17; 4.62]<br><br>Quantifying residual heterogeneity:<br>I^2 = 93.0% [89.5%; 95.3%]; H = 3.77 [3.09; 4.59]<br><br>Test of heterogeneity:<br>Q d.f. p-value<br>175.63 12 < 0.0001

7. Gestational weight gain

Gestational weight gain	Inadequate	Adequate
Inadequate	—	—
Adequate	0.99	—
Excessive	0.37	0.39

Inadequate v.s.	Number of studies combined: k = 8

© 2024 Zhang X et al. *JAMA Pediatrics*.

Adequate	proportion 95%-CI<br>Fixed effect model 0.1380 [0.1336; 0.1424]<br>Random effects model 0.1116 [0.0803; 0.1472]<br><br>Quantifying heterogeneity:<br>tau^2 = 0.0049 [0.0015; 0.0250]; tau = 0.0702 [0.0386; 0.1580];<br>I^2 = 97.7% [96.8%; 98.4%]; H = 6.63 [5.56; 7.89]<br><br>Quantifying residual heterogeneity:<br>I^2 = 97.9% [96.9%; 98.5%]; H = 6.83 [5.67; 8.22]<br><br>Test of heterogeneity:<br>Q d.f. p-value<br>307.51 7 < 0.0001
Inadequate v.s. Excessive	Number of studies combined: k = 8<br><br>proportion 95%-CI<br>Fixed effect model 0.1624 [0.1587; 0.1662]<br>Random effects model 0.1342 [0.0856; 0.1916]<br><br>Quantifying heterogeneity:<br>tau^2 = 0.0116 [0.0030; 0.0505]; tau = 0.1078 [0.0549; 0.2248];<br>I^2 = 99.3% [99.2%; 99.5%]; H = 12.22 [10.88; 13.73]<br><br>Quantifying residual heterogeneity:<br>I^2 = 99.4% [99.2%; 99.5%]; H = 12.42 [10.97; 14.07]<br><br>Test of heterogeneity:<br>Q d.f. p-value<br>1045.97 7 < 0.0001
Adequate v.s. Excessive	Number of studies combined: k = 8<br><br>proportion 95%-CI<br>Fixed effect model 0.1627 [0.1594; 0.1660]<br>Random effects model 0.1342 [0.0923; 0.1825]<br><br>Quantifying heterogeneity:<br>tau^2 = 0.0083 [0.0027; 0.0447]; tau = 0.0910 [0.0516; 0.2114];<br>I^2 = 99.4% [99.2%; 99.5%]; H = 12.53 [11.17; 14.05]<br><br>Quantifying residual heterogeneity:<br>I^2 = 99.4% [99.3%; 99.5%]; H = 13.18 [11.69; 14.85]<br><br>Test of heterogeneity:<br>Q d.f. p-value<br>1099.03 7 < 0.0001

8. Birth weight

Birth weight	Low (≤2499g)	Normal (2500-4000g)
Low (≤2499g)	—	—
Normal (2500-4000g)	0.09	—
High (≥4001g)	0.001	0.06

© 2024 Zhang X et al. *JAMA Pediatrics*.

Low v.s. Normal	Number of studies combined: k = 34<br><br>proportion 95%-CI<br>Fixed effect model 0.1396 [0.1383; 0.1409]<br>Random effects model 0.0775 [0.0633; 0.0931]<br><br>Quantifying heterogeneity:<br>tau^2 = 0.0059 [0.0043; 0.0192]; tau = 0.0765 [0.0659; 0.1386];<br>I^2 = 99.3% [99.2%; 99.3%]; H = 11.70 [11.07; 12.37]<br><br>Quantifying residual heterogeneity:<br>I^2 = 99.2% [99.2%; 99.3%]; H = 11.50 [10.87; 12.18]<br><br>Test of heterogeneity:<br>Q d.f. p-value<br>4517.94 33 0
Low v.s. High	Number of studies combined: k = 32<br><br>proportion 95%-CI<br>Fixed effect model 0.1580 [0.1546; 0.1615]<br>Random effects model 0.0907 [0.0676; 0.1167]<br><br>Quantifying heterogeneity:<br>tau^2 = 0.0130 [0.0084; 0.0320]; tau = 0.1142 [0.0915; 0.1790];<br>I^2 = 98.4% [98.1%; 98.6%]; H = 7.92 [7.35; 8.53]<br><br>Quantifying residual heterogeneity:<br>I^2 = 96.9% [96.3%; 97.5%]; H = 5.71 [5.20; 6.27]<br><br>Test of heterogeneity:<br>Q d.f. p-value<br>1943.51 31 0
Normal v.s. High	Number of studies combined: k = 32<br><br>proportion 95%-CI<br>Fixed effect model 0.1488 [0.1475; 0.1501]<br>Random effects model 0.1071 [0.0896; 0.1260]<br><br>Quantifying heterogeneity:<br>tau^2 = 0.0064 [0.0050; 0.0228]; tau = 0.0799 [0.0709; 0.1509];<br>I^2 = 99.4% [99.3%; 99.5%]; H = 12.85 [12.18; 13.56]<br><br>Quantifying residual heterogeneity:<br>I^2 = 99.3% [99.2%; 99.4%]; H = 12.17 [11.50; 12.88]<br><br>Test of heterogeneity:<br>Q d.f. p-value<br>5121.59 31 0

9. Breastfeeding package

Breastfeeding package	None (full fomula)	Mixed
None (full fomula)	—	—

© 2024 Zhang X et al. *JAMA Pediatrics*.

Mixed	0.85	—
Full (no fomula)	0.17	0.25

None v.s. Mixed	Number of studies combined: k = 20<br><br>proportion 95%-CI<br>Fixed effect model 0.1106 [0.1088; 0.1123]<br>Random effects model 0.1030 [0.0818; 0.1263]<br><br>Quantifying heterogeneity:<br>tau^2 = 0.0062 [0.0034; 0.0186]; tau = 0.0788 [0.0584; 0.1363];<br>I^2 = 99.2% [99.1%; 99.4%]; H = 11.53 [10.71; 12.41]<br><br>Quantifying residual heterogeneity:<br>I^2 = 99.3% [99.1%; 99.4%]; H = 11.57 [10.72; 12.47]<br><br>Test of heterogeneity:<br>Q d.f. p-value<br>2526.46 19 0
None v.s. Full	Number of studies combined: k = 20<br><br>proportion 95%-CI<br>Fixed effect model 0.1032 [0.1009; 0.1054]<br>Random effects model 0.0937 [0.0726; 0.1172]<br><br>Quantifying heterogeneity:<br>tau^2 = 0.0069 [0.0038; 0.0188]; tau = 0.0833 [0.0618; 0.1372];<br>I^2 = 98.9% [98.6%; 99.0%]; H = 9.36 [8.60; 10.19]<br><br>Quantifying residual heterogeneity:<br>I^2 = 98.5% [98.2%; 98.8%]; H = 8.30 [7.55; 9.12]<br><br>Test of heterogeneity:<br>Q d.f. p-value<br>1664.63 19 0
Mixed v.s. Full	Number of studies combined: k = 16<br><br>proportion 95%-CI<br>Fixed effect model 0.0971 [0.0954; 0.0989]<br>Random effects model 0.0893 [0.0704; 0.1103]<br><br>Quantifying heterogeneity:<br>tau^2 = 0.0047 [0.0021; 0.0142]; tau = 0.0683 [0.0459; 0.1191];<br>I^2 = 99.1% [99.0%; 99.3%]; H = 10.74 [9.84; 11.72]<br><br>Quantifying residual heterogeneity:<br>I^2 = 99.1% [98.9%; 99.2%]; H = 10.51 [9.59; 11.52]<br><br>Test of heterogeneity:<br>Q d.f. p-value<br>1730.10 15 0

© 2024 Zhang X et al. *JAMA Pediatrics.*

eTable 12. Analysis of comorbidities for obesity in children and adolescents.

Comorbidity	Number of studies combined: k = 64<br><br>proportion 95%-CI<br>Random effects model 0.2078 [0.1736; 0.2443]<br><br>Quantifying heterogeneity:<br>tau^2 = 0.0300 [0.0266; 0.1038]; tau = 0.1731 [0.1631; 0.3222];<br>I^2 = 99.6% [99.6%; 99.7%]; H = 16.72 [16.20; 17.25]<br><br>Quantifying residual heterogeneity:<br>I^2 = 99.4% [99.4%; 99.5%]; H = 13.36 [12.85; 13.89]<br><br>Test of heterogeneity:<br>Q d.f. p-value<br>17605.01 63 0<br>Results for subgroups (random effects model):<br>k proportion 95%-CI tau^2 tau<br>subgroup = hypertension 26 0.2802 [0.2016; 0.3661] 0.0546 0.2337<br>subgroup = diabetes 3 0.0123 [0.0023; 0.0298] 0.0028 0.0528<br>subgroup = asthma 7 0.1884 [0.1246; 0.2616] 0.0129 0.1135<br>subgroup = depression 2 0.3524 [0.0044; 0.8690] 0.1649 0.4061<br>subgroup = anxiety 2 0.2508 [0.0000; 0.9418] 0.3331 0.5772<br>subgroup = Vitamin D deficiency 8 0.1163 [0.0536; 0.1986] 0.0261 0.1617<br>subgroup = flatfoot 3 0.2608 [0.0669; 0.5219] 0.0533 0.2309<br>subgroup = dental caries 13 0.1788 [0.1261; 0.2383] 0.0173 0.1315<br>Q I^2<br>subgroup = hypertension 2991.55 99.2%<br>subgroup = diabetes 41.15 95.1%<br>subgroup = asthma 256.76 97.7%<br>subgroup = depression 371.04 99.7%<br>subgroup = anxiety 754.92 99.9%<br>subgroup = Vitamin D deficiency 401.62 98.3%<br>subgroup = flatfoot 47.91 95.8%<br>subgroup = dental caries 5128.67 99.8%

eTable 13. Subgroup analysis for overweight in children and adolescents.

Subgroup	Studies	Events	Total	Prevalence (95% CI)	*P* value	I^2
Country					<0·0001	
Albania	2	774	8069	10·94 (4·71–19·36)		98·9%
Algeria	3	398	3837	11·72 (5·97–19·06)		97·1%
Argentina	12	4549	24328	19·33 (17·57–21·16)		86·3%
Australia	51	32624	190767	16·87 (15·87–17·89)		96·8%
Austria	4	1169	8940	12·18 (8·29–16·70)		97·1%
Bahamas	1	319	1308	24·39 (22·1–26·75)		
Bahrain	3	460	3350	14·16 (9·89–19·04)		91·4%
Bangladesh	8	1940	18088	15·22 (11·09–19·87)		98·0%
Barbados	1	266	1504	17·69 (15·80–19·66)		
Belgium	15	3801	39466	9·86 (8·77–11·01)		91·9%
Benin	2	376	3398	11·46 (9·38–13·71)		64·7%
Bhutan	1	138	392	35·20 (30·55–40·01)		
Bolivia	4	1102	7020	15·80 (13·23–18·55)		86·3%
Bosnia and Herzegovina	2	787	6108	13·06 (10·71–15·60)		87·7%
Botswana	1	87	707	12·31 (9·98–14·83)		
Brazil	80	51837	305649	15·93 (14·85–17·05)		98·3%
Brunei Darussalam	1	341	1824	18·70 (16·94–20·52)		
Bulgaria	6	2645	14734	16·85 (11·15–23·45)		99·0%

© 2024 Zhang X et al. *JAMA Pediatrics*.

Burkina Faso	3	301	8431	4·34 (0·88–10·17)	98·3%
Burundi	1	112	3493	3·21 (2·65–3·82)	
Cameroon	7	1291	13385	10·74 (8·04–13·77)	96·2%
Canada	45	631800	3477584	17·35 (16·56–18·15)	97·9%
Chile	11	3982	19447	25·69 (21·90–29·67)	96·3%
China mainland	131	669741	5586169	12·45 (11·68–13·24)	99·9%
Colombia	5	3358	25080	15·47 (12·12–19·16)	97·0%
Comoros	1	266	2699	9·86 (8·76–11·01)	
Congo	2	478	12922	3·70 (3·38–4·03)	0%
Costa Rica	1	68941	347366	19·85 (19·71–19·98)	
Cote d'Ivoire	2	193	4545	5·03 (1·36–10·82)	97·8%
Croatia	8	2960	19623	11·56 (7·89–15·82)	98·6%
Cyprus	8	4159	21867	18·55 (16·28–20·93)	94·3%
Czech	7	5806	48743	14·04 (11·40–16·90)	98·1%
Denmark	12	6314	68426	10·65 (9·15–12·24)	97·2%
Djibouti	1	233	1711	13·62 (12·03–15·28)	
Dominican Republic	1	192	954	20·13 (17·64–22·73)	
East Timor	1	77	1631	4·72 (3·74–5·81)	
Ecuador	3	2309	12664	13·25 (7·60–20·17)	96·4%
Egypt	9	2010	15109	14·36 (7·81–22·48)	99·3%
El Salvador	1	15881	111991	14·18 (13·98–14·39)	
Estonia	4	835	10275	9·28 (7·09–11·74)	92·7%
Ethiopia	11	708	18012	6·92 (3·96–10·61)	98·3%
Fiji	3	2038	12257	16·58 (14·15–19·15)	89·1%
Finland	7	3722	30778	11·85 (10·94–12·79)	80·7%
France	23	19468	162311	12·97 (11·37–14·66)	98·5%
French Polynesis	1	455	1902	23·92 (22·03–25·87)	
Gabon	1	209	3482	6·00 (5·24–6·82)	
Gambia	1	67	3360	1·99 (1·55–2·50)	
Georgia	2	508	3226	15·66 (14·42–16·94)	0%
Germany	36	18019	156459	10·63 (9·36–11·98)	98·5%
Ghana	13	1496	20425	10·91 (7·03–15·50)	98·7%
Greece	58	97060	417432	21·68 (20·65–22·72)	97·8%
Greenland	2	258	1501	17·40 (14·42–20·60)	59·2%
Guatemala	1	68	363	18·73 (14·88–22·92)	
Guinea	1	103	3216	3·20 (2·62–3·84)	
Honduras	1	266	2554	10·42 (9·26–11·63)	
Hong Kong	9	6722	48045	14·51 (12·05–17·16)	98·2%
Hungary	11	5899	43224	14·73 (11·83–17·89)	98·6%
Iceland	4	1752	14284	12·41 (11·07–13·82)	78·1%
India	81	34248	299540	11·75 (10·66–12·90)	98·8%
Indonesia	8	14299	121161	12·37 (10·82–14·00)	90·3%
Iran	76	583443	4600387	13·67 (13·23–14·12)	99·2%
Iraq	4	1260	14922	10·96 (5·32–18·29)	99·2%
Ireland	15	11078	65512	15·10 (12·50–17·90)	98·8%
Israel	8	68521	608056	11·16 (10·64–11·70)	93·4%
Italy	53	57962	281965	20·15 (18·96–21·37)	98·2%
Jamaica	1	166	1061	15·65 (13·52–17·90)	
Japan	10	8005	72729	10·13 (7·82–12·70)	99·0%
Jordan	12	2621	14367	18·44 (16·41–20·55)	90·1%
Kazakhstan	2	632	6388	8·13 (1·47–19·45)	99·4%
Kenya	3	325	2327	13·94 (12·56–15·39)	0%
Kiribati	1	514	1582	32·49 (30·2–34·82)	
Kuwait	11	7719	64261	16·62 (10·78–23·41)	99·6%
Kyrgyzstan	1	429	5958	7·20 (6·56–7·87)	
Laos	1	155	1644	9·43 (8·06–10·89)	
Latvia	5	1470	13122	11·15 (5·94–17·73)	99·0%
Lebanon	7	4543	20131	19·80 (15·70–24·24)	97·5%
Liberia	1	101	3259	3·10 (2·53–3·72)	
Libya	3	410	2019	20·40 (17·31–23·68)	69·3%
Lithuania	5	2423	19529	11·68 (5·94–19·02)	99·5%
Luxemburg	1	402	3904	10·30 (9·36–11·27)	
Macedonia	4	1574	11931	13·66 (10·38–17·31)	96·5%
Malawi	2	633	7134	8·87 (8·22–9·54)	0%
Malaysia	19	18590	122524	14·56 (13·06–16·13)	97·5%
Mali	1	129	4591	2·81 (2·35–3·31)	
Malta	5	1294	6904	18·75 (17·79–19·73)	7·0%
Mauritania	1	424	2028	20·91 (19·16–22·71)	
Mauritius	3	472	2996	15·47 (13·22–17·86)	56·0%
Mexico	36	13178	69610	19·38 (17·13–21·73)	98·1%
Mongolia	1	374	3707	10·09 (9·14–11·08)	
Montenegro	3	1311	6999	19·01 (16·75–21·37)	76·1%
Morocco	4	1081	9594	11·63 (6·52–17·97)	98·4%
Mozambique	1	749	9721	7·70 (7·18–8·24)	
Multiple countries	11	28831	190450	14·80 (12·71–17·01)	99·0%

© 2024 Zhang X et al. *JAMA Pediatrics*.

Namibia	2	178	3781	4·64 (2·88–6·78)		88·2%
Nepal	7	673	7945	9·49 (5·98–13·70)		96·0%
Netherlands	25	32285	252778	12·36 (10·40–14·47)		99·5%
New Zealand	8	39442	216398	20·50 (18·07–23·05)		98·8%
Niger	1	113	5123	2·21 (1·82–2·63)		
Nigeria	12	2658	48540	8·20 (6·03–10·67)		98·4%
Norway	18	6213	53178	12·87 (11·87–13·90)		90·5%
Pakistan	9	842	7882	13·43 (9·19–18·32)		96·7%
Palestine	8	1023	7463	13·62 (12·61–14·65)		34·7%
Peru	2	722	4288	16·76 (14·89–18·72)		65·7%
Philippines	1	487	6162	7·90 (7·24–8·59)		
Poland	37	10473	91015	11·87 (10·74–13·06)		96·3%
Portugal	35	21190	113959	19·45 (17·85–21·09)		97·8%
Puerto Rico	3	341	2066	13·35 (8·24–19·44)		88·0%
Qatar	3	2640	11824	22·67 (20·87–24·53)		74·0%
Republic of Marshall Islands	1	269	3271	8·22 (7·31–9·19)		
Romania	10	7208	55265	12·91 (10·64–15·36)		98·4%
Russia	5	1500	19758	8·01 (5·25–11·28)		98·2%
Rwanda	1	305	4116	7·41 (6·63–8·23)		
Samoa	1	788	2418	32·59 (30·73–34·47)		
San Marino	1	64	303	21·12 (16·70–25·91)		
Saudi Arabia	26	9934	69028	14·44 (12·72–16·25)		97·4%
Senegal	1	79	6062	1·30 (1·03–1·60)		
Serbia	10	5016	32643	15·24 (12·49–18·20)		97·8%
Seychelles	4	4054	30478	13·98 (10·14–18·33)		96·8%
Sierra Leone	1	348	4698	7·41 (6·68–8·17)		
Singapore	1	183	2256	8·11 (7·02–9·28)		
Slovakia	2	451	5078	9·63 (6·13–13·81)		94·4%
Slovenia	11	8703	46166	17·64 (14·56–20·96)		98·8%
Solomon Islands	1	269	1421	18·93 (16·93–21·01)		
South Africa	22	6161	43256	11·65 (9·32–14·21)		98·2%
South Korea	19	539138	5416214	11·52 (11·11–11·94)		98·8%
Spain	52	585922	2803430	19·27 (17·84–20·74)		99·5%
Sri Lanka	2	1389	13875	8·21 (4·61–12·73)		95·6%
Sudan	3	231	2344	10·21 (7·22–13·65)		83·3%
Suriname	1	256	1453	17·62 (15·7–19·62)		
Sweden	31	24053	180310	14·49 (13·38–15·64)		97·7%
Switzerland	10	3762	31991	11·60 (9·49–13·88)		97·2%
Syria	2	361	1838	19·64 (17·85–21·49)		0%
Taiwan	20	10823	68969	14·82 (12·12–17·74)		99·%
Tajikistan	1	175	2822	6·20 (5·34–7·12)		
Tanzania	7	989	12740	9·34 (5·75–13·69)		97·8%
Thailand	13	3944	36999	10·70 (9·26–12·22)		94·7%
Togo	2	88	3862	3·16 (0·62–7·50)		95·3%
Tonga	3	1576	4602	34·47 (29·07–40·09)		92·7%
Trinidad and Tobago	2	433	2699	15·44 (12·83–18·24)		49·9%
Tunisia	2	314	2138	14·72 (13·03–16·49)		22·5%
Turkey	51	17477	149216	12·24 (11·17–13·35)		97·6%
Turkmenistan	1	1514	9768	15·5 (14·79–16·22)		
Uganda	1	198	4212	4·70 (4·08–5·36)		
Ukraine	5	4272	40633	9·59 (6·98–12·55)		98·7%
UAE	7	4298	31845	15·91 (12·13–20·10)		96·7%
UK	46	177299	1258506	16·39 (15·06–17·77)		99·1%
USA	222	1518921	7017160	17·81 (16·61–19·04)		99·9%
Vanuatu	1	129	1119	11·53 (9·72–13·47)		
Vietnam	13	5640	30800	14·69 (10·61–19·31)		99·1%
Zambia	1	631	11677	5·40 (5·00–5·82)		
Zimbabwe	2	301	5379	5·58 (4·98–6·21)		0%
Geographic region					<0·0001	
Southern Europe	240	783043	3743601	19·36 (18·81–19·91)		98·9%
Northern Africa	24	4444	35041	13·77 (10·83–16·99)		98·5%
South America	114	67013	392909	17·07 (16·18–17·98)		98·0%
Australia and New Zealand	59	72066	407165	17·37 (16·53–18·23)		97·4%
Western Europe	120	80841	669618	11·69 (10·98–12·43)		98·8%
Caribbean	9	1717	9592	16·65 (14·40–19·03)		88·3%
Western Asia	142	124140	1012093	14·61 (13·92–15·32)		98·7%
Southern Asia	112	40332	354742	12·18 (11·24–13·15)		98·6%
Western Africa	42	6476	121538	7·45 (6·11–8·92)		98·8%
Southern Africa	25	6426	47744	11·00 (8·71–13·51)		98·4%
South-Eastern Asia	134	627159	4925388	13·39 (13·02–13·77)		99·0%
Eastern Europe	83	38254	318450	12·43 (11·55–13·34)		98·3%
Eastern Africa	32	8987	103955	9·01 (7·23–10·97)		99·1%
Middle Africa	10	1978	29789	8·52 (6·16–11·23)		98·3%
Northern America	269	2150979	10496245	17·75 (16·81–18·71)		99·9%
Eastern Asia	190	1234803	11195833	12·63 (12·12–13·14)		99·8%

© 2024 Zhang X et al. *JAMA Pediatrics*.

Central America	40	98334	531884	18·87 (17·36–20·43)		99·0%
Northern Europe	147	235159	1713920	14·01 (13·39–14·65)		98·8%
Melanesia	5	2436	14797	16·01 (13·70–18·46)		91·5%
Polynesia	5	2819	8922	31·86 (27·04–36·89)		95·9%
Central Asia	12	3739	37676	9·12 (6·49–12·14)		98·8%
Micronesia	10	1806	12316	14·51 (10·30–19·30)		97·9%
Not Applicable	11	28831	190450	14·80 (12·71–17·01)		99·0%
Development					<0·0001	
Developing	966	1663562	13128349	13·37 (13·09–13·65)		99·5%
Developed	858	3929389	23054869	16·41 (15·93–16·91)		99·9%
Not Applicable	11	28831	190450	14·80 (12·71–17·01)		99·0%
Country or region income					<0·0001	
High-Income	1033	4019167	23726591	16·10 (15·66–16·54)		99·9%
Upper-Middle-Income	449	886870	6913008	14·18 (13·70–14·66)		99·7%
Lower-Middle-Income	308	681736	5444532	12·40 (12·05–12·74)		99·1%
Low-Income	34	5178	99087	6·36 (5·13–7·71)		98·5%
Not Applicable	11	28831	190450	14·8 (12·71–17·01)		99·0%
Sample source					0·025	
Database	611	3867079	24515622	14·15 (13·66–14·64)		99·9%
School	960	1086082	6727100	15·16 (14·65–15·67)		99·7%
Community	173	521771	4167843	14·97 (14·35–15·59)		99·6%
Medical institution	91	146849	963103	14·98 (13·53–16·49)		99·7%
Study design					<0·0001	
Cross-sectional	1705	4771295	32527627	14·72 (14·44–15·00)		99·8%
Longitudinal	41	767785	3211812	17·21 (14·08–20·57)		100·0%
Cohort	69	73582	558683	14·80 (13·48–16·17)		99·4%
RCT	11	2394	15171	15·26 (13·31–17·30)		90·2%
Prospective	8	6654	60116	13·11 (10·63–15·82)		96·2%
Case-control	1	72	259	27·80 (22·50–33·43)		
Diagnostic reference					<0·0001	
WHO	420	1048570	6088050	13·94 (13·30–14·60)		99·8%
IOTF	774	1286208	7864087	15·54 (15·19–15·91)		99·4%
CDC	398	1992345	10997958	15·33 (14·53–16·15)		99·9%
National Reference	243	1294659	11423573	12·90 (12·50–13·29)		99·7%
Sample size					0·0002	
≤5000	1441	324666	2188652	15·08 (14·73–15·43)		98·1%
>5000	394	5297115	34185016	13·80 (13·20–14·41)		100·0%

Country	Number of studies combined: k = 1835<br><br>proportion 95%-CI<br>Random effects model 0.1479 [0.1449; 0.1508]<br><br>Quantifying heterogeneity:<br>tau^2 = 0.0079 [<0.0000; <0.0000]; tau = 0.0891 [<0.0000; <0.0000];<br>I^2 = 99.8% [99.8%; 99.8%]; H = 24.73 [24.62; 24.84]<br><br>Quantifying residual heterogeneity:<br>I^2 = 99.7% [99.7%; 99.7%]; H = 18.06 [17.96; 18.17]<br><br>Test of heterogeneity:<br>Q d.f. p-value<br>1121396.80 1834 0
Geographic region	Number of studies combined: k = 1835<br><br>proportion 95%-CI<br>Random effects model 0.1479 [0.1449; 0.1508]<br><br>Quantifying heterogeneity:<br>tau^2 = 0.0079 [<0.0000; <0.0000]; tau = 0.0891 [<0.0000; <0.0000];<br>I^2 = 99.8% [99.8%; 99.8%]; H = 24.73 [24.62; 24.84]<br><br>Quantifying residual heterogeneity:<br>I^2 = 99.7% [99.7%; 99.7%]; H = 18.13 [18.03; 18.23]<br><br>Test of heterogeneity:

© 2024 Zhang X et al. *JAMA Pediatrics*.

	Q d.f. p-value<br>1121396.80 1834 0
Development	Number of studies combined: k = 1835<br><br>proportion 95%-CI<br>Random effects model 0.1479 [0.1449; 0.1508]<br><br>Quantifying heterogeneity:<br>tau^2 = 0.0079 [<0.0000; <0.0000]; tau = 0.0891 [<0.0000; <0.0000];<br>I^2 = 99.8% [99.8%; 99.8%]; H = 24.73 [24.62; 24.84]<br><br>Quantifying residual heterogeneity:<br>I^2 = 99.8% [99.8%; 99.8%]; H = 23.43 [23.32; 23.54]<br><br>Test of heterogeneity:<br>Q d.f. p-value<br>1121396.80 1834 0
Country or region income	Number of studies combined: k = 1835<br><br>proportion 95%-CI<br>Random effects model 0.1479 [0.1449; 0.1508]<br><br>Quantifying heterogeneity:<br>tau^2 = 0.0079 [<0.0000; <0.0000]; tau = 0.0891 [<0.0000; <0.0000];<br>I^2 = 99.8% [99.8%; 99.8%]; H = 24.73 [24.62; 24.84]<br><br>Quantifying residual heterogeneity:<br>I^2 = 99.8% [99.8%; 99.8%]; H = 23.42 [23.31; 23.53]<br><br>Test of heterogeneity:<br>Q d.f. p-value<br>1121396.80 1834 0
Sample source	Number of studies combined: k = 1835<br><br>proportion 95%-CI<br>Random effects model 0.1479 [0.1449; 0.1508]<br><br>Quantifying heterogeneity:<br>tau^2 = 0.0079 [<0.0000; <0.0000]; tau = 0.0891 [<0.0000; <0.0000];<br>I^2 = 99.8% [99.8%; 99.8%]; H = 24.73 [24.62; 24.84]<br><br>Quantifying residual heterogeneity:<br>I^2 = 99.8% [99.8%; 99.8%]; H = 24.43 [24.33; 24.54]<br><br>Test of heterogeneity:<br>Q d.f. p-value<br>1121396.80 1834 0
Study design	Number of studies combined: k = 1835<br><br>proportion 95%-CI<br>Random effects model 0.1479 [0.1449; 0.1508]<br><br>Quantifying heterogeneity:<br>tau^2 = 0.0079 [<0.0000; <0.0000]; tau = 0.0891 [<0.0000; <0.0000];<br>I^2 = 99.8% [99.8%; 99.8%]; H = 24.73 [24.62; 24.84]

© 2024 Zhang X et al. *JAMA Pediatrics.*

	Quantifying residual heterogeneity:<br>I^2 = 99.8% [99.8%; 99.8%]; H = 22.79 [22.68; 22.90]<br><br>Test of heterogeneity:<br>Q d.f. p-value<br>1121396.80 1834 0
Diagnostic reference	Number of studies combined: k = 1835<br><br>proportion 95%-CI<br>Random effects model 0.1479 [0.1449; 0.1508]<br><br>Quantifying heterogeneity:<br>tau^2 = 0.0079 [<0.0000; <0.0000]; tau = 0.0891 [<0.0000; <0.0000];<br>I^2 = 99.8% [99.8%; 99.8%]; H = 24.73 [24.62; 24.84]<br><br>Quantifying residual heterogeneity:<br>I^2 = 99.8% [99.8%; 99.8%]; H = 22.27 [22.16; 22.37]<br><br>Test of heterogeneity:<br>Q d.f. p-value<br>1121396.80 1834 0
Sample size	Number of studies combined: k = 1835<br><br>proportion 95%-CI<br>Random effects model 0.1479 [0.1449; 0.1508]<br><br>Quantifying heterogeneity:<br>tau^2 = 0.0079 [<0.0000; <0.0000]; tau = 0.0891 [<0.0000; <0.0000];<br>I^2 = 99.8% [99.8%; 99.8%]; H = 24.73 [24.62; 24.84]<br><br>Quantifying residual heterogeneity:<br>I^2 = 99.8% [99.8%; 99.8%]; H = 24.72 [24.62; 24.83]<br><br>Test of heterogeneity:<br>Q d.f. p-value<br>1121396.80 1834 0

eTable 14. Subgroup analysis for excess weight prevalence among children and adolescents.

Subgroup	Studies	Events	Total	Prevalence (95% CI)	*P* value	I^2
Country					<0·0001	
Albania	2	1445	8069	19·80 (11·19–30·15)		99·0%
Algeria	3	517	3837	15·35 (7·89–24·73)		97·8%
Argentina	14	7145	25532	29·30 (24·09–34·80)		98·3%
Australia	79	52687	245361	20·50 (18·76–22·29)		99·1%
Austria	4	1484	8940	16·16 (11·23–21·78)		97·6%
Bahamas	1	598	1308	45·72 (43·02–48·42)		
Bahrain	4	1002	4085	26·43 (18·32–35·43)		97·1%
Bangladesh	10	3501	31561	20·76 (13·45–29·18)		99·6%
Barbados	1	480	1504	31·91 (29·58–34·29)		
Belgium	17	4836	40154	13·65 (11·82–15·60)		96·3%
Benin	2	409	3398	12·99 (9·30–17·17)		87·7%
Bhutan	1	140	392	35·71 (31·04–40·53)		
Bolivia	4	1548	7020	20·46 (17·56–23·52)		86·7%
Bosnia and Herzegovina	3	1523	6638	23·21 (16·17–31·10)		97·8%
Botswana	1	122	707	17·26 (14·56–20·13)		
Brazil	123	89891	368457	22·29 (20·54–24·08)		99·4%
Brunei Darussalam	1	660	1824	36·18 (33·99–38·40)		
Bulgaria	7	3952	15617	23·05 (14·83–32·46)		99·4%
Burkina Faso	3	486	8431	6·57 (1·57–14·60)		98·7%
Burundi	1	164	3493	4·70 (4·02–5·42)		
Cambodia	1	21	624	3·37 (2·08–4·94)		

© 2024 Zhang X et al. *JAMA Pediatrics.*

Cameroon	10	1838	15080	12·66 (9·90–15·71)	96·2%
Canada	58	1023092	3518820	27·03 (25·48–28·61)	99·5%
Chile	14	9494	174559	38·76 (22·57–56·33)	99·9%
China mainland	175	1113234	6156139	18·92 (17·71–20·16)	99·9%
Colombia	9	5708	38343	16·66 (13·27–20·36)	98·5%
Comoros	2	659	5144	12·41 (6·20–20·36)	98·4%
Congo	2	764	12922	5·65 (4·10–7·42)	93·7%
Costa Rica	3	118888	349779	33·98 (33·82–34·14)	0%
Cote d'Ivoire	2	264	4545	6·84 (2·10–14·00)	98·1%
Croatia	8	4446	19623	17·72 (10·58–26·22)	99·5%
Cyprus	9	6114	23833	24·84 (22·33–27·44)	94·8%
Czech	7	7294	48743	18·89 (13·43–25·04)	99·5%
Denmark	14	8498	69429	14·19 (11·26–17·40)	99·1%
Djibouti	2	459	3249	13·48 (5·34–24·57)	98·5%
Dominican Republic	1	309	954	32·39 (29·46–35·40)	
East Timor	1	97	1631	5·95 (4·85–7·15)	
Ecuador	6	4274	15145	24·96 (18·5–32·04)	97·8%
Egypt	10	3618	15845	27·08 (19·84–35·00)	99·0%
El Salvador	1	25968	111991	23·19 (22·94–23·44)	
Estonia	4	1023	10275	11·70 (8·38–15·49)	96·3%
Ethiopia	14	1359	28893	8·43 (5·73–11·59)	98·4%
Fiji	3	2722	12257	22·72 (18·96–26·71)	94·3%
Finland	15	11925	80332	14·91 (13·80–16·04)	94·1%
France	27	26351	174549	16·46 (14·69–18·32)	98·7%
French Polynesis	1	875	1902	46·00 (43·77–48·25)	
Gabon	1	338	3482	9·71 (8·75–10·71)	
Gambia	1	181	3360	5·39 (4·65–6·18)	
Georgia	2	789	3226	24·39 (22·92–25·90)	0%
Germany	49	28237	189787	14·34 (12·90–15·84)	98·7%
Ghana	17	2255	22050	14·90 (9·64–21·05)	99·2%
Greece	68	133301	426836	29·93 (28·58–31·29)	98·5%
Greenland	2	292	1501	19·44 (17·46–21·51)	1·7%
Guatemala	1	139	363	38·29 (33·35–43·36)	
Guinea	1	183	3216	5·69 (4·92–6·52)	
Honduras	1	378	2554	14·80 (13·45–16·20)	
Hong Kong	14	20209	258849	16·92 (11·08–23·69)	99·9%
Hungary	11	9028	43224	21·48 (18·54–24·57)	98·2%
Iceland	6	2299	14929	15·53 (13·61–17·56)	86·4%
India	99	55489	507241	16·42 (14·34–18·62)	99·7%
Indonesia	18	25749	190648	21·46 (17·38–25·84)	99·7%
Iran	82	754296	4616406	21·94 (21·24–22·64)	99·6%
Iraq	5	1877	21340	13·80 (6·56–23·16)	99·6%
Ireland	15	15367	65512	21·07 (18·22–24·08)	98·7%
Israel	10	93579	613132	17·64 (15·65–19·72)	99·6%
Italy	63	90822	290473	28·27 (25·67–30·94)	99·6%
Jamaica	2	609	4064	17·84 (6·06–34·08)	99·1%
Japan	28	41276	308956	12·27 (10·82–13·79)	99·2%
Jordan	12	4129	14367	27·94 (24·15–31·89)	96·3%
Kazakhstan	2	873	6388	10·81 (1·10–28·62)	99·7%
Kenya	6	1272	22254	11·62 (5·01–20·48)	99·0%
Kiribati	1	631	1582	39·89 (37·49–42·31)	
Kuwait	11	12225	64261	38·61 (25·25–52·91)	99·8%
Kyrgyzstan	1	590	5958	9·90 (9·16–10·67)	
Laos	2	226	2297	8·28 (3·22–15·38)	95·8%
Latvia	5	2007	13122	14·34 (6·12–25·26)	99·5%
Lebanon	7	5983	20131	27·11 (21·51–33·10)	98·3%
Liberia	1	156	3259	4·79 (4·08–5·55)	
Libya	4	1556	9251	25·05 (14·39–37·52)	98·8%
Lithuania	6	3446	20982	14·37 (6·73–24·26)	99·7%
Luxemburg	1	492	3904	12·60 (11·58–13·66)	
Macedonia	5	5198	21012	23·05 (13·86–33·77)	99·6%
Malawi	2	931	7134	12·20 (8·17–16·91)	96·6%
Malaysia	26	48094	186707	24·20 (22·24–26·22)	98·8%
Maldives	1	20	145	13·79 (8·61–19·93)	
Mali	1	230	4591	5·01 (4·40–5·66)	
Malta	5	2264	6904	31·79 (27·82–35·90)	91·7%
Mauritania	1	493	2028	24·31 (22·47–26·20)	
Mauritius	3	707	2996	22·57 (19·62–25·65)	64·6%
Mexico	41	25762	73398	37·04 (33·06–41·12)	99·1%
Mongolia	1	441	3707	11·90 (10·87–12·96)	
Montenegro	3	1894	6999	28·60 (20·81–37·10)	97·7%
Morocco	7	2481	15537	20·09 (14·91–25·83)	98·4%
Mozambique	2	1854	19144	9·53 (5·59–14·39)	99·1%
Multiple countries	19	44950	233682	17·77 (15·21–20·49)	99·5%
Namibia	2	257	3781	6·76 (5·25–8·44)	74·8%

© 2024 Zhang X et al. *JAMA Pediatrics*.

Nepal	8	920	8213	13·16 (7·55–20·01)		98·1%
Netherlands	39	44296	289663	14·48 (12·48–16·61)		99·5%
New Zealand	10	75820	226167	33·23 (29·75–36·79)		99·3%
Niger	1	292	5123	5·70 (5·08–6·35)		
Nigeria	17	4461	53487	9·78 (7·56–12·24)		98·4%
Norway	23	8234	60392	14·47 (13·41–15·56)		92·1%
Pakistan	14	2467	25793	18·70 (13·13–25·00)		99·1%
Palestine	11	4254	23077	19·23 (16·66–21·94)		94·5%
Peru	6	37377	2351432	14·31 (5·61–26·14)		99·9%
Philippines	1	660	6162	10·71 (9·95–11·50)		
Poland	42	14905	99699	15·70 (14·14–17·32)		97·8%
Portugal	50	36943	144879	26·41 (24·33–28·54)		98·8%
Puerto Rico	5	1721	5325	38·26 (28·34–48·71)		97·6%
Qatar	4	3820	12985	34·80 (28·36–41·54)		97·9%
Republic of Marshall Islands	1	436	3271	13·33 (12·19–14·52)		
Romania	10	13304	55265	20·12 (15·69–24·95)		99·4%
Russia	5	1858	19758	10·56 (6·03–16·17)		99·2%
Rwanda	1	400	4116	9·72 (8·83–10·64)		
Samoa	3	1349	2920	28·59 (7·82–55·85)		99·1%
San Marino	1	101	303	33·33 (28·13–38·75)		
Saudi Arabia	32	19944	73527	30·49 (25·86–35·33)		99·4%
Senegal	1	128	6062	2·11 (1·76–2·49)		
Serbia	10	7051	32643	24·27 (20·01–28·80)		98·7%
Seychelles	4	5813	30478	20·77 (14·16–28·26)		99·1%
Sierra Leone	1	794	4698	16·90 (15·84–17·99)		
Singapore	5	1192	12980	11·08 (6·60–16·54)		98·5%
Slovakia	2	506	5078	10·91 (6·57–16·17)		96·0%
Slovenia	12	11451	47287	22·71 (18·76–26·91)		99·1%
Solomon Islands	1	307	1421	21·60 (19·50–23·78)		
South Africa	30	9114	50516	15·52 (12·46–18·85)		98·9%
South Korea	38	1177293	5671915	18·02 (16·61–19·47)		99·9%
Spain	69	985407	2851054	27·31 (25·18–29·49)		99·7%
Sri Lanka	4	2056	15077	7·27 (2·24–14·82)		98·8%
Sudan	3	399	2344	17·63 (10·30–26·40)		95·9%
Suriname	1	423	1453	29·11 (26·80–31·48)		
Sweden	41	40770	1157287	17·07 (12·29–22·44)		99·9%
Switzerland	12	5038	34823	14·43 (11·68–17·40)		98·2%
Syria	3	1592	7292	24·43 (19·40–29·84)		94·1%
Taiwan	23	30143	105074	27·42 (24·42–30·53)		99·10%
Tajikistan	1	217	2822	7·69 (6·73–8·70)		
Tanzania	7	1494	12740	14·33 (9·28–20·25)		98·4%
Thailand	16	8553	52014	18·41 (14·79–22·32)		99·2%
Togo	2	129	3862	4·52 (1·14–9·95)		96·0%
Tonga	3	2574	4602	53·65 (48·03–59·22)		92·2%
Trinidad and Tobago	3	1021	3789	22·38 (9·24–39·19)		99·0%
Tunisia	2	437	2138	20·41 (18·73–22·15)		0%
Turkey	59	28712	154496	19·11 (17·3–21·00)		98·8%
Turkmenistan	1	2569	9768	26·3 (25·43–27·18)		
Uganda	1	253	4212	6·01 (5·31–6·74)		
Ukraine	5	5678	40633	12·4 (8·44–16·99)		99·3%
UAE	8	9739	32121	27·93 (23·02–33·12)		97·0%
UK	61	349617	1332449	22·01 (19·86–24·23)		99·7%
USA	303	3390634	10493725	33·92 (32·08–35·79)		100·0%
Vanuatu	1	133	1119	11·89 (10·05–13·85)		
Vietnam	21	14139	136193	18·61 (11·83–26·51)		99·8%
Yemen	3	1466	14062	12·05 (5·93–19·94)		99·0%
Zambia	2	1531	21382	6·88 (3·77–10·85)		99·1%
Zimbabwe	3	870	11785	8·93 (5·64–12·88)		97·6%
Geographic region					<0·0001	
Southern Europe	294	1276648	3841708	27·40 (26·49–28·32)		99·6%
Northern Africa	29	9008	48952	22·31 (19·16–25·61)		98·6%
South America	173	154312	2974921	23·60 (20·62–26·72)		99·9%
Australia and New Zealand	89	128507	471528	21·80 (19·90–23·75)		99·6%
Western Europe	157	117524	770124	15·17 (14·29–16·07)		99·1%
Caribbean	13	4738	16944	30·62 (23·77–37·93)		99·0%
Western Asia	166	189379	1051566	24·29 (22·93–25·69)		99·6%
Southern Asia	141	66141	595442	16·63 (14·91–18·42)		99·7%
Western Africa	51	10461	128110	10·47 (8·78–12·28)		99·0%
Southern Africa	33	9493	55004	14·94 (12·02–18·11)		98·9%
South-Eastern Asia	174	853687	5207486	20·73 (20·00–21·47)		99·7%
Eastern Europe	89	56525	328017	17·05 (15·55–18·60)		99·3%
Eastern Africa	43	16272	164280	11·10 (9·09–13·28)		99·4%
Middle Africa	13	2940	31484	11·14 (8·73–13·80)		97·9%
Northern America	363	4414018	14014046	32·78 (31·30–34·28)		100·0%
Eastern Asia	279	2382596	12504640	18·63 (17·79–19·48)		99·9%

© 2024 Zhang X et al. *JAMA Pediatrics*.

Central America	47	171135	538085	35·91 (33·26–38·60)		99·6%
Northern Europe	190	443186	2824709	17·86 (15·31–20·56)		100·0%
Melanesia	5	3162	14797	20·19 (16·33–24·35)		96·5%
Polynesia	7	4798	9424	41·89 (34·20–49·78)		98·2%
Central Asia	12	5743	37676	13·56 (9·12–18·72)		99·4%
Micronesia	13	5321	27930	20·10 (16·47–23·99)		98·0%
Not Applicable	19	44950	233682	17·77 (15·21–20·49)		99·5%
Development					<0·0001	
Developing	1221	2629137	17936675	20·03 (19·33–20·75)		99·9%
Developed	1160	7696457	27720198	24·59 (23·91–25·27)		99·9%
Not Applicable	19	44950	233682	17·77 (15·21–20·49)		99·5%
Country or region income					<0·0001	
High-Income	1360	7849093	29534511	24·34 (23·61–25·08)		100·0%
Upper-Middle-Income	589	1540122	10035109	21·57 (20·41–22·76)		99·9%
Lower-Middle-Income	389	923978	5938641	17·61 (17·00–18·24)		99·7%
Low-Income	43	12400	148612	9·51 (7·92–11·24)		99·2%
Not Applicable	19	44950	233682	17·77 (15·21–20·49)		99·5%
Sample source					<0·0001	
Database	806	7050900	28247286	20·34 (19·49–21·21)		100·0%
School	1225	2259576	9528157	22·90 (22·13–23·68)		99·9%
Community	231	767427	6786477	22·87 (21·10–24·67)		100·0%
Medical institution	138	292640	1328635	25·87 (23·63–28·18)		99·9%
Study design					<0·0001	
Cross-sectional	2174	8764855	39558537	22·25 (21·71–22·80)		99·9%
Longitudinal	125	1268676	3272424	20·11 (16·94–23·47)		100·0%
Cohort	61	159010	1753923	24·79 (20·66–29·17)		100·0%
RCT	21	7577	30032	24·76 (21·01–28·71)		98·2%
Prospective	18	170316	1275380	16·05 (14·21–17·98)		98·9%
Case-control	1	110	259	42·47 (36·50–48·55)		
Diagnostic reference					<0·0001	
WHO	579	1841813	9292341	20·84 (19·31–22·40)		100·0%
IOTF	966	2038500	8716287	20·40 (19·78–21·03)		99·8%
CDC	517	3888205	14685524	28·18 (26·81–29·56)		100·0%
National Reference	338	2602026	13196403	20·94 (19·79–22·12)		100·0%
Sample size					<0·0001	
≤5000	1888	592544	2720290	22·91 (22·38–23·44)		99·1%
>5000	512	9777999	43170265	19·56 (18·41–20·75)		100·0%

Country	Number of studies combined: k = 2400<br><br>proportion 95%-CI<br>Random effects model 0.2219 [0.2163; 0.2275]<br><br>Quantifying heterogeneity:<br>tau^2 = 0.0282 [<0.0000; <0.0000]; tau = 0.1680 [<0.0000; <0.0000];<br>I^2 = 100.0% [100.0%; 100.0%]; H = 45.92 [45.81; 46.03]<br><br>Quantifying residual heterogeneity:<br>I^2 = 99.9% [99.9%; 99.9%]; H = 28.57 [28.47; 28.67]<br><br>Test of heterogeneity:<br>Q d.f. p-value<br>5058406.11 2399 0
Geographic region	Number of studies combined: k = 2400<br><br>proportion 95%-CI<br>Random effects model 0.2219 [0.2163; 0.2275]<br><br>Quantifying heterogeneity:<br>tau^2 = 0.0282 [<0.0000; <0.0000]; tau = 0.1680 [<0.0000; <0.0000];<br>I^2 = 100.0% [100.0%; 100.0%]; H = 45.92 [45.81; 46.03]<br><br>Quantifying residual heterogeneity:<br>I^2 = 99.9% [99.9%; 99.9%]; H = 33.03 [32.93; 33.14]<br><br>Test of heterogeneity:

© 2024 Zhang X et al. *JAMA Pediatrics*.

	Q d.f. p-value<br>5058406.11 2399 0
Country or region income	Number of studies combined: k = 2400<br><br>proportion 95%-CI<br>Random effects model 0.2219 [0.2163; 0.2275]<br><br>Quantifying heterogeneity:<br>tau^2 = 0.0282 [<0.0000; <0.0000]; tau = 0.1680 [<0.0000; <0.0000];<br>I^2 = 100.0% [100.0%; 100.0%]; H = 45.92 [45.81; 46.03]<br><br>Quantifying residual heterogeneity:<br>I^2 = 99.9% [99.9%; 99.9%]; H = 41.61 [41.51; 41.72]<br><br>Test of heterogeneity:<br>Q d.f. p-value<br>5058406.11 2399 0
Development	Number of studies combined: k = 2400<br><br>proportion 95%-CI<br>Random effects model 0.2219 [0.2163; 0.2275]<br><br>Quantifying heterogeneity:<br>tau^2 = 0.0282 [<0.0000; <0.0000]; tau = 0.1680 [<0.0000; <0.0000];<br>I^2 = 100.0% [100.0%; 100.0%]; H = 45.92 [45.81; 46.03]<br><br>Quantifying residual heterogeneity:<br>I^2 = 99.9% [99.9%; 99.9%]; H = 38.98 [38.87; 39.09]<br><br>Test of heterogeneity:<br>Q d.f. p-value<br>5058406.11 2399 0
Sample source	Number of studies combined: k = 2400<br><br>proportion 95%-CI<br>Random effects model 0.2219 [0.2163; 0.2275]<br><br>Quantifying heterogeneity:<br>tau^2 = 0.0282 [<0.0000; <0.0000]; tau = 0.1680 [<0.0000; <0.0000];<br>I^2 = 100.0% [100.0%; 100.0%]; H = 45.92 [45.81; 46.03]<br><br>Quantifying residual heterogeneity:<br>I^2 = 99.9% [99.9%; 99.9%]; H = 41.90 [41.79; 42.00]<br><br>Test of heterogeneity:<br>Q d.f. p-value<br>5058406.11 2399 0
Study design	Number of studies combined: k = 2400<br><br>proportion 95%-CI<br>Random effects model 0.2219 [0.2163; 0.2275]<br><br>Quantifying heterogeneity:<br>tau^2 = 0.0282 [<0.0000; <0.0000]; tau = 0.1680 [<0.0000; <0.0000];<br>I^2 = 100.0% [100.0%; 100.0%]; H = 45.92 [45.81; 46.03]

© 2024 Zhang X et al. *JAMA Pediatrics*.

	Quantifying residual heterogeneity:<br>I^2 = 99.9% [99.9%; 99.9%]; H = 41.62 [41.52; 41.73]<br><br>Test of heterogeneity:<br>Q d.f. p-value<br>5058406.11 2399 0
Diagnostic reference	Number of studies combined: k = 2400<br><br>proportion 95%-CI<br>Random effects model 0.2219 [0.2163; 0.2275]<br><br>Quantifying heterogeneity:<br>tau^2 = 0.0282 [<0.0000; <0.0000]; tau = 0.1680 [<0.0000; <0.0000];<br>I^2 = 100.0% [100.0%; 100.0%]; H = 45.92 [45.81; 46.03]<br><br>Quantifying residual heterogeneity:<br>I^2 = 99.9% [99.9%; 99.9%]; H = 44.41 [44.30; 44.52]<br><br>Test of heterogeneity:<br>Q d.f. p-value<br>5058406.11 2399 0
Sample size	Number of studies combined: k = 2400<br><br>proportion 95%-CI<br>Random effects model 0.2219 [0.2163; 0.2275]<br><br>Quantifying heterogeneity:<br>tau^2 = 0.0282 [<0.0000; <0.0000]; tau = 0.1680 [<0.0000; <0.0000];<br>I^2 = 100.0% [100.0%; 100.0%]; H = 45.92 [45.81; 46.03]<br><br>Quantifying residual heterogeneity:<br>I^2 = 100.0% [100.0%; 100.0%]; H = 45.93 [45.82; 46.04]<br><br>Test of heterogeneity:<br>Q d.f. p-value<br>5058406.11 2399 0

eReferences

1. Ramos Salas X, Buoncristiano M, Williams J, et al. Parental Perceptions of Children's Weight Status in 22 Countries: The WHO European Childhood Obesity Surveillance Initiative: COSI 2015/2017. *Obes Facts* 2021; **14**(6): 658-74.
2. Hyska J, Mersini E, Mone I, Burazeri G. Prevalence and demographic correlates of overweight and obesity among children in a transitional southeastern European population. *J Community Health* 2014; **39**(5): 828-34.
3. Benmohammed K, Valensi P, Nguyen MT, et al. Influence of waist circumference on blood pressure status in non-obese adolescents. *Int J Adolesc Med Health* 2018; **32**(3).
4. Musaiger AO, Al-Mannai M, Tayyem R, et al. Prevalence of Overweight and Obesity among Adolescents in Seven Arab Countries: A Cross-Cultural Study. *J Obes* 2012; **2012**: 981390.
5. Fedala N, Mekimene L, Mokhtari M, Haddam AEM, Fedala NS, Kardjadj M. Prevalence and risk factors of overweight and obesity among schoolchildren and adolescents in Algiers. *Mediterranean Journal of Nutrition and Metabolism* 2017; **10**(2): 105-12.
6. Orden AB, Lamarque MS, Chan D, Mayer MA. Short sleep and low milk intake are associated with obesity in a community of school aged children from Argentina. *Am J Hum Biol* 2019; **31**(3): e23224.

© 2024 Zhang X et al. *JAMA Pediatrics.*

7. Meyer E. Prevalencia de sobrepeso y obesidad en escolares jujeños de diferente nivel altitudinal según las referencias IOTF, CDC y OMS. *Archivos Argentinos de Pediatria* 2013; **111**(6): 516-22.

8. de Moraes AC, Musso C, Graffigna MN, et al. Prevalence of cardiovascular risk factors among Latin American adolescents: a multilevel analysis. *J Hum Hypertens* 2014; **28**(3): 206-9.

9. Hirschler V, Molinari C, Maccallini G, Aranda C. Is albuminuria associated with obesity in school children? *Pediatr Diabetes* 2010; **11**(5): 322-30.

10. Stray-Pedersen M, Helsing RM, Gibbons L, et al. Weight status and hypertension among adolescent girls in Argentina and Norway: data from the ENNyS and HUNT studies. *BMC Public Health* 2009; **9**: 398.

11. Hirschler V, Calcagno ML, Clemente AM, Aranda C, Gonzalez C. Association between school children's overweight and maternal obesity and perception of their children's weight status. *J Pediatr Endocrinol Metab* 2008; **21**(7): 641-9.

12. Hirschler V, Bugna J, Roque M, Gilligan T, Gonzalez C. Does low birth weight predict obesity/overweight and metabolic syndrome in elementary school children? *Arch Med Res* 2008; **39**(8): 796-802.

13. Hirschler V, Gonzalez C, Talgham S, Jadzinsky M. Do mothers of overweight Argentinean preschool children perceive them as such? *Pediatr Diabetes* 2006; **7**(4): 201-4.

14. Hirschler V, Molinari C, Gonzalez C. Maternal misperceptions of children's overweight in an indigenous Argentinean community. *Am J Health Behav* 2015; **39**(3): 338-44.

15. Kovalskys I, Rausch Herscovici C, De Gregorio MJ. Nutritional status of school-aged children of Buenos Aires, Argentina: data using three references. *J Public Health (Oxf)* 2011; **33**(3): 403-11.

16. Tringler M, Rodriguez EM, Aguera D, Molina JD, Canziani GA, Diaz A. High blood pressure, overweight and obesity among rural scholars from the Vela Project: a population-based study from South America. *High Blood Press Cardiovasc Prev* 2012; **19**(1): 41-6.

17. Catalani F, Fraire J, Perez N, Mazzola M, Martinez AM, Mayer MA. Underweight, overweight and obesity prevalence among adolescent school children in the Province of La Pamp Argentina. *Arch Argent Pediatr* 2016; **114**(2): 154-8.

18. Gotthelf SJ, Tempestti CP. Breakfast, nutritional status, and socioeconomic outcome measures among primary school students from the City of Salta: A cross-sectional study. *Arch Argent Pediatr* 2017; **115**(5): 424-31.

19. Rivero MR, De Angelo C, Nunez P, Salas M, Liang S. Intestinal parasitism and nutritional status among indigenous children from the Argentinian Atlantic Forest: Determinants of enteroparasites infections in minority populations. *Acta Trop* 2018; **187**: 248-56.

20. Abbott RA, Lee AJ, Stubbs CO, Davies PS. Accuracy of weight status perception in contemporary Australian children and adolescents. *J Paediatr Child Health* 2010; **46**(6): 343-8.

21. Gibson LY, Byrne SM, Davis EA, Blair E, Jacoby P, Zubrick SR. The role of family and maternal factors in childhood obesity. *Med J Aust* 2007; **186**(11): 591-5.

22. Spinks AB, Macpherson AK, Bain C, McClure RJ. Compliance with the Australian national physical activity guidelines for children: relationship to overweight status. *J Sci Med Sport* 2007; **10**(3): 156-63.

23. Crawford DA, Timperio AF, Salmon JA, et al. Neighbourhood fast food outlets and obesity in children and adults: the CLAN Study. *Int J Pediatr Obes* 2008; **3**(4): 249-56.

24. Franklin J, Denyer G, Steinbeck KS, Caterson ID, Hill AJ. Obesity and risk of low self-esteem: a statewide survey of Australian children. *Pediatrics* 2006; **118**(6): 2481-7.

25. Wolfenden L, Hardy LL, Wiggers J, Milat AJ, Bell C, Sutherland R. Prevalence and socio-demographic associations of overweight and obesity among children attending child-care services in rural and regional Australia. *Nutrition & Dietetics* 2011; **68**(1): 15-20.

26. Van Cauwenberghe E, Jones RA, Hinkley T, Crawford D, Okely AD. Patterns of physical activity and sedentary

© 2024 Zhang X et al. *JAMA Pediatrics*.

behaviour in preschool children. *Int J Behav Nutr Phys Act* 2012; **9**: 138.
27. Wake M, Clifford SA, Patton GC, et al. Morbidity patterns among the underweight, overweight and obese between 2 and 18 years: population-based cross-sectional analyses. *Int J Obes (Lond)* 2013; **37**(1): 86-93.
28. James S, Pezic A, Ponsonby AL, et al. Obesity and asthma at school entry: co-morbidities and temporal trends. *J Paediatr Child Health* 2013; **49**(4): E273-80.
29. Marshall S, Watson J, Burrows T, Guest M, Collins CE. The development and evaluation of the Australian child and adolescent recommended food score: a cross-sectional study. *Nutr J* 2012; **11**: 96.
30. Martin K, Bremner A, Salmon J, Rosenberg M, Giles-Corti B. School and individual-level characteristics are associated with children's moderate to vigorous-intensity physical activity during school recess. *Aust N Z J Public Health* 2012; **36**(5): 469-77.
31. O'Dea JA, Dibley MJ. Obesity increase among low SES Australian schoolchildren between 2000 and 2006: time for preventive interventions to target children from low income schools? *Int J Public Health* 2010; **55**(3): 185-92.
32. O'Dea JA, Chiang H, Peralta LR. Socioeconomic patterns of overweight, obesity but not thinness persist from childhood to adolescence in a 6-year longitudinal cohort of Australian schoolchildren from 2007 to 2012. *BMC Public Health* 2014; **14**: 222.
33. Schofield G, Schofield L, Hinckson EA, Mummery WK. Daily step counts and selected coronary heart disease risk factors in adolescent girls. *J Sci Med Sport* 2009; **12**(1): 148-55.
34. Gopinath B, Baur LA, Burlutsky G, Robaei D, Mitchell P. Socio-economic, familial and perinatal factors associated with obesity in Sydney schoolchildren. *J Paediatr Child Health* 2012; **48**(1): 44-51.
35. Spurrier NJ, Volkmer RE, Abdallah CA, Chong A. South Australian four-year-old Aboriginal children: residence and socioeconomic status influence weight. *Aust N Z J Public Health* 2012; **36**(3): 285-90.
36. Trapp GS, Giles-Corti B, Christian HE, et al. On your bike! a cross-sectional study of the individual, social and environmental correlates of cycling to school. *Int J Behav Nutr Phys Act* 2011; **8**: 123.
37. Waters E, Ashbolt R, Gibbs L, et al. Double disadvantage: the influence of ethnicity over socioeconomic position on childhood overweight and obesity: findings from an inner urban population of primary school children. *Int J Pediatr Obes* 2008; **3**(4): 196-204.
38. Bell S, Yew SSY, Devenish G, Ha D, Do L, Scott J. Duration of Breastfeeding, but Not Timing of Solid Food, Reduces the Risk of Overweight and Obesity in Children Aged 24 to 36 Months: Findings from an Australian Cohort Study. *Int J Environ Res Public Health* 2018; **15**(4).
39. Wen LM, Baur LA, Rissel C, Xu H, Simpson JM. Correlates of body mass index and overweight and obesity of children aged 2 years: findings from the healthy beginnings trial. *Obesity (Silver Spring)* 2014; **22**(7): 1723-30.
40. Hoare E, Crooks N, Hayward J, Allender S, Strugnell C. Associations between combined overweight and obesity, lifestyle behavioural risk and quality of life among Australian regional school children: baseline findings of the Goulburn Valley health behaviours monitoring study. *Health Qual Life Outcomes* 2019; **17**(1): 16.
41. Maher C, Olds TS, Eisenmann JC, Dollman J. Screen time is more strongly associated than physical activity with overweight and obesity in 9- to 16-year-old Australians. *Acta Paediatr* 2012; **101**(11): 1170-4.
42. Hayes AJ, Carrello JP, Kelly PJ, Killedar A, Baur LA. Looking backwards and forwards: tracking and persistence of weight status between early childhood and adolescence. *Int J Obes (Lond)* 2021; **45**(4): 870-8.
43. Miller LJ, Joyce S, Carter S, Yun G. Associations between childhood obesity and the availability of food outlets in the local environment: a retrospective cross-sectional study. *Am J Health Promot* 2014; **28**(6): e137-45.
44. Achat HM, Stubbs JM. Socio-economic and ethnic differences in the prevalence of overweight and obesity among school children. *J Paediatr Child Health* 2014; **50**(10): E77-84.
45. O'Dea JA. Gender, ethnicity, culture and social class influences on childhood obesity among Australian schoolchildren: implications for treatment, prevention and community education. *Health Soc Care Community* 2008;

16(3): 282-90.
46. O'Sullivan TA, Bremner AP, Bremer HK, et al. Dairy product consumption, dietary nutrient and energy density and associations with obesity in Australian adolescents. *J Hum Nutr Diet* 2015; **28**(5): 452-64.
47. Barnes C, Hall A, Nathan N, et al. Efficacy of a school-based physical activity and nutrition intervention on child weight status: Findings from a cluster randomized controlled trial. *Prev Med* 2021; **153**: 106822.
48. Magee CA, Caputi P, Iverson DC. Patterns of health behaviours predict obesity in Australian children. *J Paediatr Child Health* 2013; **49**(4): 291-6.
49. Cox R, Skouteris H, Rutherford L, Fuller-Tyszkiewicz M, Dell' Aquila D, Hardy LL. Television viewing, television content, food intake, physical activity and body mass index: a cross-sectional study of preschool children aged 2-6 years. *Health Promot J Austr* 2012; **23**(1): 58-62.
50. Wickramasinghe VP, Cleghorn GJ, Edmiston KA, Murphy AJ, Abbott RA, Davies PS. Validity of BMI as a measure of obesity in Australian white Caucasian and Australian Sri Lankan children. *Ann Hum Biol* 2005; **32**(1): 60-71.
51. Telford RD, Cunningham RB, Daly RM, et al. Discordance of international adiposity classifications in Australian boys and girls - the LOOK study. *Ann Hum Biol* 2008; **35**(3): 334-41.
52. Seach KA, Dharmage SC, Lowe AJ, Dixon JB. Delayed introduction of solid feeding reduces child overweight and obesity at 10 years. *Int J Obes (Lond)* 2010; **34**(10): 1475-9.
53. Mallan KM, Daniels LA, Nicholson JM. Obesogenic eating behaviors mediate the relationships between psychological problems and BMI in children. *Obesity (Silver Spring)* 2017; **25**(5): 928-34.
54. Jansen PW, Mensah FK, Clifford SA, Tiemeier H, Nicholson JM, Wake M. Development of mental health problems and overweight between ages 4 and 11 years: a population-based longitudinal study of Australian children. *Acad Pediatr* 2013; **13**(2): 159-67.
55. Tai A, Volkmer R, Burton A. Association between asthma symptoms and obesity in preschool (4-5 year old) children. *J Asthma* 2009; **46**(4): 362-5.
56. Hoare E, Millar L, Fuller-Tyszkiewicz M, et al. Associations between obesogenic risk and depressive symptomatology in Australian adolescents: a cross-sectional study. *J Epidemiol Community Health* 2014; **68**(8): 767-72.
57. Bergmeier H, Skouteris H, Horwood S, Hooley M, Richardson B. Child temperament and maternal predictors of preschool children's eating and body mass index. A prospective study. *Appetite* 2014; **74**: 125-32.
58. Hardy LL, King L, Hector D, Lloyd B. Weight status and weight-related behaviors of children commencing school. *Prev Med* 2012; **55**(5): 433-7.
59. Watson A, D'Souza NJ, Timperio A, Cliff DP, Okely AD, Hesketh KD. Longitudinal associations between weight status and academic achievement in primary school children. *Pediatr Obes* 2023; **18**(1): e12975.
60. White PA, Awad YA, Gauvin L, et al. Household income and maternal education in early childhood and risk of overweight and obesity in late childhood: Findings from seven birth cohort studies in six high-income countries. *Int J Obes (Lond)* 2022; **46**(9): 1703-11.
61. Au N. The health care cost implications of overweight and obesity during childhood. *Health Serv Res* 2012; **47**(2): 655-76.
62. Aurangzeb B, Whitten KE, Harrison B, et al. Prevalence of malnutrition and risk of under-nutrition in hospitalized children. *Clin Nutr* 2012; **31**(1): 35-40.
63. Sanigorski AM, Bell AC, Kremer PJ, Swinburn BA. Lunchbox contents of Australian school children: room for improvement. *Eur J Clin Nutr* 2005; **59**(11): 1310-6.
64. Rehor PR, Brownsey E. A preliminary investigation into the prevalence and trends of overweight and obesity of northern Tasmanian primary school children. *Aust N Z J Public Health* 2002; **26**(5): 479-80.
© 2024 Zhang X et al. *JAMA Pediatrics.*

65. Wen LM, Merom D, Rissel C, Simpson JM. Weight status, modes of travel to school and screen time: a cross-sectional survey of children aged 10-13 years in Sydney. *Health Promot J Austr* 2010; **21**(1): 57-63.
66. Scott B, Bolton KA, Strugnell C, Allender S, Marks J. Weight status and obesity-related dietary behaviours among culturally and linguistically diverse (CALD) children in Victoria, Australia. *BMC Pediatr* 2019; **19**(1): 511.
67. Shi Z, Taylor AW, Gill TK, Tuckerman J, Adams R, Martin J. Short sleep duration and obesity among Australian children. *BMC Public Health* 2010; **10**: 609.
68. Jones RA, Okely AD, Caputi P, Cliff DP. Perceived and actual competence among overweight and non-overweight children. *J Sci Med Sport* 2010; **13**(6): 589-96.
69. MacFarlane A, Cleland V, Crawford D, Campbell K, Timperio A. Longitudinal examination of the family food environment and weight status among children. *Int J Pediatr Obes* 2009; **4**(4): 343-52.
70. Jones RA, Okely AD, Gregory P, Cliff DP. Relationships between weight status and child, parent and community characteristics in preschool children. *Int J Pediatr Obes* 2009; **4**(1): 54-60.
71. Haysom L, Williams RE, Hodson EM, et al. Cardiovascular risk factors in Australian indigenous and non-indigenous children: a population-based study. *J Paediatr Child Health* 2009; **45**(1-2): 20-7.
72. Haug E, Rasmussen M, Samdal O, et al. Overweight in school-aged children and its relationship with demographic and lifestyle factors: results from the WHO-Collaborative Health Behaviour in School-aged Children (HBSC) study. *Int J Public Health* 2009; **54 Suppl 2**(Suppl 2): 167-79.
73. Ducher G, Bass SL, Naughton GA, Eser P, Telford RD, Daly RM. Overweight children have a greater proportion of fat mass relative to muscle mass in the upper limbs than in the lower limbs: implications for bone strength at the distal forearm. *Am J Clin Nutr* 2009; **90**(4): 1104-11.
74. Sutherland R, Finch M, Harrison M, Collins C. Higher prevalence of childhood overweight and obesity in association with gender and socioeconomic status in the Hunter region of New South Wales. *Nutrition & Dietetics* 2008; **65**(3): 192-7.
75. Denney-Wilson E, Hardy LL, Dobbins T, Okely AD, Baur LA. Body mass index, waist circumference, and chronic disease risk factors in Australian adolescents. *Arch Pediatr Adolesc Med* 2008; **162**(6): 566-73.
76. Fisher L, Fraser J, Alexander C. Caregivers' inability to identify childhood adiposity: a cross-sectional survey of rural children and their caregivers' attitudes. *Aust J Rural Health* 2006; **14**(2): 56-61.
77. Cretikos MA, Valenti L, Britt HC, Baur LA. General practice management of overweight and obesity in children and adolescents in Australia. *Med Care* 2008; **46**(11): 1163-9.
78. Cleland V, Crawford D, Baur LA, Hume C, Timperio A, Salmon J. A prospective examination of children's time spent outdoors, objectively measured physical activity and overweight. *Int J Obes (Lond)* 2008; **32**(11): 1685-93.
79. Crawford D, Timperio A, Telford A, Salmon J. Parental concerns about childhood obesity and the strategies employed to prevent unhealthy weight gain in children. *Public Health Nutr* 2006; **9**(7): 889-95.
80. Campbell MW, Williams J, Hampton A, Wake M. Maternal concern and perceptions of overweight in Australian preschool-aged children. *Med J Aust* 2006; **184**(6): 274-7.
81. Burke V, Beilin LJ, Durkin K, Stritzke WG, Houghton S, Cameron CA. Television, computer use, physical activity, diet and fatness in Australian adolescents. *Int J Pediatr Obes* 2006; **1**(4): 248-55.
82. Sanigorski AM, Bell AC, Kremer PJ, Swinburn BA. High childhood obesity in an Australian population. *Obesity (Silver Spring)* 2007; **15**(8): 1908-12.
83. McLean K, Wake M, McCallum Z. Overweight in medical paediatric inpatients: detection and parent expectations. *J Paediatr Child Health* 2007; **43**(4): 256-61.
84. Leech RM, McNaughton SA, Timperio A. Clustering of diet, physical activity and sedentary behaviour among Australian children: cross-sectional and longitudinal associations with overweight and obesity. *Int J Obes (Lond)* 2015; **39**(7): 1079-85.

© 2024 Zhang X et al. *JAMA Pediatrics.*

85. Katzmarzyk PT, Barreira TV, Broyles ST, et al. Physical Activity, Sedentary Time, and Obesity in an International Sample of Children. *Med Sci Sports Exerc* 2015; **47**(10): 2062-9.
86. Cochrane T, Davey RC, de Castella FR. Influence of school community and fitness on prevalence of overweight in Australian school children. *Prev Med* 2015; **81**: 433-7.
87. O'Dea JA, Dibley MJ. Prevalence of obesity, overweight and thinness in Australian children and adolescents by socioeconomic status and ethnic/cultural group in 2006 and 2012. *Int J Public Health* 2014; **59**(5): 819-28.
88. Chen S, Binns CW, Maycock B, Zhao Y, Liu Y. Chinese mothers' perceptions of their child's weight and obesity status. *Asia Pac J Clin Nutr* 2014; **23**(3): 452-8.
89. Keating CL, Moodie ML, Swinburn BA. The health-related quality of life of overweight and obese adolescents--a study measuring body mass index and adolescent-reported perceptions. *Int J Pediatr Obes* 2011; **6**(5-6): 434-41.
90. Olds TS, Maher CA, Matricciani L. Sleep duration or bedtime? Exploring the relationship between sleep habits and weight status and activity patterns. *Sleep* 2011; **34**(10): 1299-307.
91. Morley BC, Scully ML, Niven PH, et al. What factors are associated with excess body weight in Australian secondary school students? *Med J Aust* 2012; **196**(3): 189-92.
92. Schultz R. Prevalences of overweight and obesity among children in remote Aboriginal communities in central Australia. *Rural Remote Health* 2012; **12**: 1872.
93. Fredrickson J, Kremer P, Swinburn B, de Silva-Sanigorski A, McCabe M. Biopsychosocial correlates of weight status perception in Australian adolescents. *Body Image* 2013; **10**(4): 552-7.
94. Ruedl G, Greier K, Kirschner W, Kopp M. Factors associated with motor performance among overweight and nonoverweight Tyrolean primary school children. *Wien Klin Wochenschr* 2016; **128**(1-2): 14-9.
95. Furthner D, Ehrenmuller M, Biebl A, et al. Gender differences and the role of parental education, school types and migration on the body mass index of 2930 Austrian school children : A cross-sectional study. *Wien Klin Wochenschr* 2017; **129**(21-22): 786-92.
96. Mihrshahi S, Drayton BA, Bauman AE, Hardy LL. Associations between childhood overweight, obesity, abdominal obesity and obesogenic behaviors and practices in Australian homes. *BMC Public Health* 2017; **18**(1): 44.
97. Furthner D, Ehrenmueller M, Lanzersdorfer R, Halmerbauer G, Schmitt K, Biebl A. Education, school type and screen time were associated with overweight and obesity in 2930 adolescents. *Acta Paediatr* 2018; **107**(3): 517-22.
98. Yngve A, De Bourdeaudhuij I, Wolf A, et al. Differences in prevalence of overweight and stunting in 11-year olds across Europe: The Pro Children Study. *Eur J Public Health* 2008; **18**(2): 126-30.
99. Janssen I, Katzmarzyk PT, Boyce WF, King MA, Pickett W. Overweight and obesity in Canadian adolescents and their associations with dietary habits and physical activity patterns. *J Adolesc Health* 2004; **35**(5): 360-7.
100. Pfeiffer M, Kotz R, Ledl T, Hauser G, Sluga M. Prevalence of flat foot in preschool-aged children. *Pediatrics* 2006; **118**(2): 634-9.
101. Romano E, Ma R, Vancampfort D, et al. The association of cannabis use with fast-food consumption, overweight, and obesity among adolescents aged 12-15 years from 28 countries. *Journal of Substance Use* 2022; **28**(6): 952-61.
102. Musaiger AO, Al-Roomi K, Bader Z. Social, dietary and lifestyle factors associated with obesity among Bahraini adolescents. *Appetite* 2014; **73**: 197-204.
103. Al-Raees GY, Al-Amer MA, Musaiger AO, D'Souza R. Prevalence of overweight and obesity among children aged 2-5 years in Bahrain: a comparison between two reference standards. *Int J Pediatr Obes* 2009; **4**(4): 414-6.
104. Al-Sendi AM, Shetty P, Musaiger AO. Prevalence of overweight and obesity among Bahraini adolescents: a comparison between three different sets of criteria. *Eur J Clin Nutr* 2003; **57**(3): 471-4.
105. A OM, Al-Mannai M, Al-Marzog Q. Overweight and Obesity among Children (10-13 years) in Bahrain:A

comparison between Two International Standards. *Pak J Med Sci* 2014; **30**(3): 497-500.
106. Anam MR, Akter S, Hossain F, et al. Association of sleep duration and sleep quality with overweight/obesity among adolescents of Bangladesh: a multilevel analysis. *BMC Public Health* 2022; **22**(1): 374.
107. Taher MA, Ali MA, Islam MN, et al. Overweight and Obesity among Children of Primary Schools of Mymensingh Municipal Area. *Mymensingh Med J* 2021; **30**(3): 613-24.
108. Sultana S, Saleh F, Ali L. Childhood Obesity in Primary School Children of Middle and Upper-Middle Income Group in the Capital City of Bangladesh. *Food and Nutrition Sciences* 2015; **06**(13): 1185-92.
109. Sultana A, Banik S, Hossain MS, Billah M, Afrin F. The Prevalence of Childhood Overweight and Obesity in the Children of Noakhali City in Bangladesh. *Journal of Research in Obesity* 2016: 1-6.
110. Saha M, Adhikary DK, Parvin I, Sharma YR, Akhter F, Majumder M. Obesity and Its Risk Factors of among School Children in Sylhet, Bangladesh. *J Nepal Health Res Counc* 2018; **16**(2): 205-8.
111. Islam MM, Benzamin M, Roy RR, et al. Overweight, hypertension and microalbuminuria in urban and rural Bangladeshi schoolchildren. *Paediatrica Indonesiana* 2019; **59**(1): 18-26.
112. Sultana N, Afroz S, Tomalika N, Momtaz H, Kabir MH. Prevalence of Childhood Obesity and Undernutrition among Urban School Children in Bangladesh. *J Biosoc Sci* 2019; **51**(2): 244-53.
113. Khan JR, Hossain MB, Mistry SK. Breastfeeding is a protective factor for overweight/obesity among young children in Bangladesh: Findings from a nationwide data. *Children and Youth Services Review* 2020; **119**.
114. Bulbul T, Hoque M. Prevalence of childhood obesity and overweight in Bangladesh: findings from a countrywide epidemiological study. *BMC Pediatr* 2014; **14**: 86.
115. Brug J, van Stralen MM, Te Velde SJ, et al. Differences in weight status and energy-balance related behaviors among schoolchildren across Europe: the ENERGY-project. *PLoS One* 2012; **7**(4): e34742.
116. Ahrens W, Pigeot I, Pohlabeln H, et al. Prevalence of overweight and obesity in European children below the age of 10. *Int J Obes (Lond)* 2014; **38 Suppl 2**: S99-107.
117. Usheva N, Lateva M, Galcheva S, et al. Breastfeeding and Overweight in European Preschoolers: The ToyBox Study. *Nutrients* 2021; **13**(8).
118. Juliusson PB, Brannsether B, Kristiansen H, Hoppenbrouwers K, Bjerknes R, Roelants M. Should children with overweight or obesity be excluded from height references? *Arch Dis Child* 2015; **100**(11): 1044-8.
119. Seghers J, Claessens AL. Bias in self-reported height and weight in preadolescents. *J Pediatr* 2010; **157**(6): 911-6.
120. De Vriendt T, Huybrechts I, Ottevaere C, Van Trimpont I, De Henauw S. Validity of self-reported weight and height of adolescents, its impact on classification into BMI-categories and the association with weighing behaviour. *Int J Environ Res Public Health* 2009; **6**(10): 2696-711.
121. De Coen V, De Bourdeaudhuij I, Verbestel V, Maes L, Vereecken C. Risk factors for childhood overweight: a 30-month longitudinal study of 3- to 6-year-old children. *Public Health Nutr* 2014; **17**(9): 1993-2000.
122. Van Gysel D, Govaere E, Verhamme K, Doli E, De Baets F. Body mass index in Belgian schoolchildren and its relationship with sensitization and allergic symptoms. *Pediatr Allergy Immunol* 2009; **20**(3): 246-53.
123. Deforche B, De Bourdeaudhuij I, D'Hondt E, Cardon G. Objectively measured physical activity, physical activity related personality and body mass index in 6- to 10-yr-old children: a cross-sectional study. *Int J Behav Nutr Phys Act* 2009; **6**: 25.
124. Vissers D, Devoogdt N, Gebruers N, Mertens I, Truijen S, Van Gaal L. Overweight in adolescents: differences per type of education. Does one size fit all? *J Nutr Educ Behav* 2008; **40**(2): 65-71.
125. Huybrechts I, De Bacquer D, Van Trimpont I, De Backer G, De Henauw S. Validity of parentally reported weight and height for preschool-aged children in Belgium and its impact on classification into body mass index categories. *Pediatrics* 2006; **118**(5): 2109-18.

© 2024 Zhang X et al. *JAMA Pediatrics.*

126.Te Velde SJ, Haraldsen E, Vik FN, et al. Associations of commuting to school and work with demographic variables and with weight status in eight European countries: The ENERGY-cross sectional study. *Prev Med* 2017; **99**: 305-12.
127.Manyanga T, El-Sayed H, Doku DT, Randall JR. The prevalence of underweight, overweight, obesity and associated risk factors among school-going adolescents in seven African countries. *BMC Public Health* 2014; **14**: 887.
128.Norbu W, Wangdi U, Dorji D, et al. Obesity prevalence and contributing factors among adolescents in secondary schools in Pemagatshel district, Bhutan. *Int J Adolesc Med Health* 2017; **31**(1).
129.Baya Botti A, Pérez-Cueto FJ, Vasquez Monllor PA, Kolsteren PW. International BMI-for-age references underestimate thinness and overestimate overweight and obesity in Bolivian adolescents. *Nutr Hosp* 2010; **25**(3): 428-36.
130.Perez-Cueto A, Almanza M, Kolsteren PW. Female gender and wealth are associated to overweight among adolescents in La Paz, Bolivia. *Eur J Clin Nutr* 2005; **59**(1): 82-7.
131.Benefice E, Lopez R, Monroy SL, Rodriguez S. Fatness and overweight in women and children from riverine Amerindian communities of the Beni River (Bolivian Amazon). *Am J Hum Biol* 2007; **19**(1): 61-73.
132.Spahic R, Pranjic N. Children's Eating Behaviour Questionnaire: association with BMI in children aged 3-10 years from Bosnia and Herzegovina. *Public Health Nutr* 2019; **22**(18): 3360-7.
133.Hasanbegović S, Mesihović-Dinarević S, Cuplov M, et al. Epidemiology and etiology of obesity in children and youth of Sarajevo Canton. *Bosn J Basic Med Sci* 2010; **10**(2): 140-6.
134.Ramic E, Kapidzic-Durakovic S, Karic E, Batic-Mujanovic O, Alibasic E, Zildzic M. Influence of lifestyle on overweight and obesity in school-age children. *Med Arh* 2009; **63**(5): 280-3.
135.Wrotniak BH, Malete L, Maruapula SD, et al. Association between socioeconomic status indicators and obesity in adolescent students in Botswana, an African country in rapid nutrition transition. *Pediatr Obes* 2012; **7**(2): e9-e13.
136.Alexius SL, Olinto MT, Henn RL, Pattussi MP. The association between self perceptions of psychological well-being and overweight in Brazilian children. *Matern Child Nutr* 2012; **8**(2): 267-74.
137.Andaki ACR, Mendes EL, Tinoco ALA, et al. Waist circumference percentile in children from municipalities of developed and developing countries. *Motriz: Revista de Educação Física* 2018; **23**(spe2).
138.Ataide Lima RP, de Carvalho Pereira D, Pordeus Luna RC, et al. BMI, overweight status and obesity adjusted by various factors in all age groups in the population of a city in Northeastern Brazil. *Int J Environ Res Public Health* 2015; **12**(4): 4422-38.
139.Castilho SD, Nucci LB, Hansen LO, Assuino SR. Prevalence of weight excess according to age group in students from Campinas, SP, Brazil. *Rev Paul Pediatr* 2014; **32**(2): 200-6.
140.Costa Lda C, Silva DA, Almeida Sde S, de Vasconcelos Fde A. Association between inaccurate estimation of body size and obesity in schoolchildren. *Trends Psychiatry Psychother* 2015; **37**(4): 220-6.
141.Ferreira HS, Lucio GM, Assuncao ML, et al. High Blood Pressure among Students in Public and Private Schools in Maceio, Brazil. *PLoS One* 2015; **10**(11): e0142982.
142.Fraiz GM, Crispim SP, Montes GR, et al. Excess Body Weight, Snack Limits and Dental Caries in Brazilian Preschoolers: A Population-Based Study. *Pesquisa Brasileira em Odontopediatria e Clínica Integrada* 2019; **19**(1): 1-9.
143.Justo GF, Callo GQ, Carletti L, Molina MC. Nutritional extremes among school children in a rural Brazilian municipality. *Rural Remote Health* 2012; **12**(4): 2220.
144.Guedes DP, Rocha GD, Silva AJ, Carvalhal IM, Coelho EM. Effects of social and environmental determinants on overweight and obesity among Brazilian schoolchildren from a developing region. *Rev Panam Salud Publica* 2011; **30**(4): 295-302.

© 2024 Zhang X et al. *JAMA Pediatrics.*

145.Kupek E, Lobo AS, Leal DB, Bellisle F, de Assis MA. Dietary patterns associated with overweight and obesity among Brazilian schoolchildren: an approach based on the time-of-day of eating events. *Br J Nutr* 2016; **116**(11): 1954-65.
146.Moreira MA, Cabral PC, Ferreira HS, Lira PI. Overweight and associated factors in children from northeastern Brazil. *J Pediatr (Rio J)* 2012; **88**(4): 347-52.
147.Nobre LN, Silva KC, de Castro Ferreira SE, et al. Early determinants of overweight and obesity at 5 years old in preschoolers from inner of Minas Gerais, Brazil. *Nutr Hosp* 2013; **28**(3): 764-71.
148.Oppitz IN, Cesar JA, Neumann NA. Overweight among children under five years of age in municipalities of the semiarid region. *Rev Bras Epidemiol* 2014; **17**(4): 860-72.
149.Doumid Borges Pretto A, Correa Kaufmann C, Ferreira Dutra G, Pinto Albernaz E. Prevalence of factors related to the bone mass formation of children from a cohort in Southern Brazil. *Nutr Hosp* 2014; **31**(3): 1122-8.
150.Ramalho AA, Mantovani SA, Delfino BM, et al. Nutritional status of children under 5 years of age in the Brazilian Western Amazon before and after the Interoceanic highway paving: a population-based study. *BMC Public Health* 2013; **13**: 1098.
151.Santos LP, Santos IS, Matijasevich A, Barros AJD. Changes in overall and regional body fatness from childhood to early adolescence. *Sci Rep* 2019; **9**(1): 1888.
152.Silva APD, Feilbelmann TCM, Silva DC, et al. Prevalence of overweight and obesity and associated factors in school children and adolescents in a medium-sized Brazilian city. *Clinics (Sao Paulo)* 2018; **73**: e438.
153.Mastroeni MF, Mastroeni S, Czarnobay SA, Ekwaru JP, Loehr SA, Veugelers PJ. Breast-feeding duration for the prevention of excess body weight of mother-child pairs concurrently: a 2-year cohort study. *Public Health Nutr* 2017; **20**(14): 2537-48.
154.Vale D, Andrade M, Dantas NM, Bezerra RA, Lyra CO, Oliveira A. Social Determinants of Obesity and Stunting among Brazilian Adolescents: A Multilevel Analysis. *Nutrients* 2022; **14**(11).
155.Pereira LJ, Vieira FGK, Belchor ALL, et al. Methodological aspects and characteristics of participants in the study on the prevalence of obesity in children and adolescents in Florianopolis, Southern Brazil, 2018-2019: EPOCA study. *Ann Epidemiol* 2023; **77**: 13-23.
156.Lopes AF, Machado TC, Nascimento VG, Bertoli CJ, Leone C. Cesarean Delivery and Risk of Excess Weight Among Brazilian Preschool Children. *Matern Child Health J* 2022; **26**(6): 1305-11.
157.Santos MD, Ferrari G, Drenowatz C, et al. Association between breastfeeding, parents' body mass index and birth weight with obesity indicators in children. *BMC Pediatr* 2022; **22**(1): 604.
158.Coelho SR, Faria JCP, Fonseca FLA, de Souza FIS, Sarni ROS. Is There an Association between Vitamin D Concentrations and Overweight in Children and Adolescents? *J Trop Pediatr* 2022; **68**(3).
159.Cavalcante LFP, Carvalho CA, Padilha LL, Viola P, Silva A, Simoes VMF. Cesarean section and body mass index in children: is there a causal effect? *Cad Saude Publica* 2022; **38**(4): e00344020.
160.Blumenberg C, Martins RC, da Silva SG, et al. Influence of parental physical activity on offspring's nutritional status: an intergenerational study in the 1993 Pelotas birth cohort. *Public Health Nutr* 2021; **25**(8): 1-8.
161.Barbiero SM, Pellanda LC, Cesa CC, Campagnolo P, Beltrami F, Abrantes CC. Overweight, obesity and other risk factors for IHD in Brazilian schoolchildren. *Public Health Nutr* 2009; **12**(5): 710-5.
162.Barbosa MCF, Reis CLB, Lopes C, et al. Assessing the Association Between Nutritional Status, Caries, and Gingivitis in Schoolchildren: A Cross-Sectional Study. *Glob Pediatr Health* 2021; **8**: 2333794x211001237.
163.Barcellos Gemelli IF, Farias EDS, Souza OF. Age at Menarche and Its Association with Excess Weight and Body Fat Percentage in Girls in the Southwestern Region of the Brazilian Amazon. *J Pediatr Adolesc Gynecol* 2016; **29**(5): 482-8.
164.Candido AP, Benedetto R, Castro AP, et al. Cardiovascular risk factors in children and adolescents living in an

© 2024 Zhang X et al. *JAMA Pediatrics.*

urban area of Southeast of Brazil: Ouro Preto Study. *Eur J Pediatr* 2009; **168**(11): 1373-82.
165.Polderman J, Gurgel RQ, Barreto-Filho JAS, et al. Blood pressure and BMI in adolescents in Aracaju, Brazil. *Public Health Nutrition* 2011; **14**(6): 1064-70.
166.de Novaes JF, Priore SE, do Carmo Castro Franceschini S, Lamounier JA. Does the body mass index reflect cardiovascular risk factors in Brazilian children? *J Trop Pediatr* 2013; **59**(1): 43-8.
167.Duncan S, Duncan EK, Fernandes RA, et al. Modifiable risk factors for overweight and obesity in children and adolescents from São Paulo, Brazil. *BMC Public Health* 2011; **11**: 585.
168.Halal CSE, Matijasevich A, Howe LD, Santos IS, Barros FC, Nunes ML. Short Sleep Duration in the First Years of Life and Obesity/Overweight at Age 4 Years: A Birth Cohort Study. *J Pediatr* 2016; **168**: 99-103 e3.
169.Dalamaria T, Pinto WJ, Farias EDS, Souza OF. Internet Addiction among Adolescents in a Western Brazilian Amazonian City. *Rev Paul Pediatr* 2021; **39**: e2019270.
170.Lima TR, Moraes MS, Andrade JHC, Farias JM, Silva DAS. Associated Factors with the Isolated and Simultaneous Presence of Overweight and Abdominal Obesity in Adolescents. *Rev Paul Pediatr* 2020; **38**: e2018332.
171.Ribeiro CCC, Silva M, Nunes AMM, et al. Overweight, obese, underweight, and frequency of sugar consumption as risk indicators for early childhood caries in Brazilian preschool children. *Int J Paediatr Dent* 2017; **27**(6): 532-9.
172.Nogueira LR, Fontanelli MM, Aguiar BS, et al. Is the local food environment associated with excess body weight in adolescents in Sao Paulo, Brazil? *Cad Saude Publica* 2020; **36**(2): e00048619.
173.Todendi PF, Martinez JA, Reuter CP, et al. Biochemical profile, eating habits, and telomere length among Brazilian children and adolescents. *Nutrition* 2020; **71**: 110645.
174.Goldani MZ, Barbieri MA, da Silva AA, Gutierrez MR, Bettiol H, Goldani HA. Cesarean section and increased body mass index in school children: two cohort studies from distinct socioeconomic background areas in Brazil. *Nutr J* 2013; **12**: 104.
175.Caixeta HCV, Amato AA. Factors associated with overweight and abdominal obesity in Brazilian school-aged children: a comprehensive approach. *Arch Endocrinol Metab* 2020; **64**(4): 445-53.
176.Kuschnir FC, da Cunha AL. Association of overweight with asthma prevalence in adolescents in Rio de Janeiro, Brazil. *J Asthma* 2009; **46**(9): 928-32.
177.de Souza Ferreira JE, da Veiga GV. Eating disorder risk behavior in Brazilian adolescents from low socio-economic level. *Appetite* 2008; **51**(2): 249-55.
178.Rodrigues D, Costa D, Gama A, et al. Socioeconomic inequalities in the prevalence of overweight and obesity among Portuguese preschool-aged children: Changes from 2009 to 2016. *Am J Hum Biol* 2022; **34**(1): e23582.
179.Amorim PRD, de Faria RC, Byrne NM, Hills AP. Physical activity and nutritional status of Brazilian children of low socioeconomic status: undernutrition and overweight. *Asia Pac J Clin Nutr* 2006; **15**(2): 217-23.
180.Araújo CL, Dumith SC, Menezes AM, et al. Nutritional status of adolescents: the 11-year follow-up of the 1993 Pelotas (Brazil) birth cohort study. *Cad Saude Publica* 2010; **26**(10): 1895-903.
181.Flores LS, Gaya AR, Petersen RD, Gaya A. Trends of underweight, overweight, and obesity in Brazilian children and adolescents. *J Pediatr (Rio J)* 2013; **89**(5): 456-61.
182.Bispo S, Correia MI, Proietti FA, Xavier CC, Caiaffa WT. Nutritional status of urban adolescents: individual, household and neighborhood factors based on data from The BH Health Study. *Cad Saude Publica* 2015; **31 Suppl 1**: 232-45.
183.Castro TG, Barufaldi LA, Schlüssel MM, Conde WL, Leite MS, Schuch I. Waist circumference and waist circumference to height ratios of Kaingáng indigenous adolescents from the State of Rio Grande do Sul, Brazil. *Cad Saude Publica* 2012; **28**(11): 2053-62.

© 2024 Zhang X et al. *JAMA Pediatrics.*

184.Santana ML, Silva Rde C, Assis AM, et al. Factors associated with body image dissatisfaction among adolescents in public schools students in Salvador, Brazil. *Nutr Hosp* 2013; **28**(3): 747-55.
185.Lock NC, Susin C, Dame-Teixeira N, Maltz M, Alves LS. Sex differences in the association between obesity and gingivitis among 12-year-old South Brazilian schoolchildren. *J Periodontal Res* 2020; **55**(4): 559-66.
186.da Cruz LL, Cardoso LD, Pala D, et al. Metabolic syndrome components can predict C reactive protein concentration in adolescents. *Nutr Hosp* 2013; **28**(5): 1580-6.
187.de Moraes MM, Moreira NF, de Oliveira ASD, Cunha DB, Sichieri R, da Veiga GV. Associations of changes in BMI and body fat percentage with demographic and socioeconomic factors: the ELANA middle school cohort. *Int J Obes (Lond)* 2019; **43**(11): 2282-90.
188.Christofaro D, Andrade SM, Fernandes RA, Cabrera M, Rodriguez-Artalejo F, Mesas AE. Overweight parents are twice as likely to underestimate the weight of their teenage children, regardless of their sociodemographic characteristics. *Acta Paediatr* 2016; **105**(10): e474-9.
189.Coutinho CA, Longui CA, Monte O, Conde W, Kochi C. Measurement of neck circumference and its correlation with body composition in a sample of students in São Paulo, Brazil. *Horm Res Paediatr* 2014; **82**(3): 179-86.
190.de Pinho L, Botelho AC, Caldeira AP. Associated factors of overweight in adolescents from public schools in Northern Minas Gerais State, Brazil. *Rev Paul Pediatr* 2014; **32**(2): 237-43.
191.Guedes DP, Almeida FN, Neto JT, Maia Mde F, Tolentino TM. Low body weight/thinness, overweight and obesity of children and adolescents from a Brazilian region of low economic status. *Rev Paul Pediatr* 2013; **31**(4): 437-43.
192.Rosini N, Moura SA, Rosini RD, Machado MJ, Silva EL. Metabolic Syndrome and Importance of Associated Variables in Children and Adolescents in Guabiruba - SC, Brazil. *Arq Bras Cardiol* 2015; **105**(1): 37-44.
193.Neves FS, Leandro DA, Silva FA, Netto MP, Oliveira RM, Candido AP. Evaluation of the predictive capacity of vertical segmental tetrapolar bioimpedance for excess weight detection in adolescents. *J Pediatr (Rio J)* 2015; **91**(6): 551-9.
194.de Almeida Santana CC, Farah BQ, de Azevedo LB, et al. Associations Between Cardiorespiratory Fitness and Overweight With Academic Performance in 12-Year-Old Brazilian Children. *Pediatr Exerc Sci* 2017; **29**(2): 220-7.
195.Costa MVC, Calderan MF, Cruvinel T. Could orthodontic fixed appliances and excess weight affect gingival health in adolescents? *Am J Orthod Dentofacial Orthop* 2020; **157**(2): 172-7.
196.Nascimento Kluczynik Vieira CE, Cruz Enders B, Silva Coura A, Brandao de Carvalho Lira AL, Campos Muniz Medeiros C, Soares Mariz L. Nursing Diagnosis of overweight and related factors in adolescents. *Invest Educ Enferm* 2015; **33**(3): 509-18.
197.de Araujo TS, Barbosa Filho VC, Gubert FDA, et al. Factors Associated With Body Image Perception Among Brazilian Students From Low Human Development Index Areas. *J Sch Nurs* 2018; **34**(6): 449-57.
198.Carmo CDS, Ribeiro MRC, Teixeira JXP, et al. Added Sugar Consumption and Chronic Oral Disease Burden among Adolescents in Brazil. *J Dent Res* 2018; **97**(5): 508-14.
199.Fradkin C, Valentini NC, Nobre GC, Dos Santos JOL. Obesity and Overweight Among Brazilian Early Adolescents: Variability Across Region, Socioeconomic Status, and Gender. *Front Pediatr* 2018; **6**: 81.
200.Reuter CP, Burgos MS, Barbian CD, Renner JDP, Franke SIR, de Mello ED. Comparison between different criteria for metabolic syndrome in schoolchildren from southern Brazil. *Eur J Pediatr* 2018; **177**(10): 1471-7.
201.Ulbricht L, de Campos MF, Esmanhoto E, Ripka WL. Prevalence of excessive body fat among adolescents of a south Brazilian metropolitan region and State capital, associated risk factors, and consequences. *BMC Public Health* 2018; **18**(1): 312.
202.Ripka WL, Ulbricht L, Gewehr PM. Body composition and prediction equations using skinfold thickness for body fat percentage in Southern Brazilian adolescents. *PLoS One* 2017; **12**(9): e0184854.
© 2024 Zhang X et al. *JAMA Pediatrics.*

203.Tebar WR, Ritti-Dias RM, Farah BQ, Zanuto EF, Vanderlei LCM, Christofaro DGD. High blood pressure and its relationship to adiposity in a school-aged population: body mass index vs waist circumference. *Hypertens Res* 2018; **41**(2): 135-40.
204.Farias EDS, Carvalho WRG, Moraes AM, Santos JPD, Gemelli IFB, Souza OF. Inactive Behavior in Adolescent Students of the Brazilian Western Amazon. *Rev Paul Pediatr* 2019; **37**(3): 345-50.
205.Silva MPD, Pacifico AB, Piola TS, et al. Association between Physical Activity Practice and Clustering of Health Risk Behaviors in Adolescents. *Rev Paul Pediatr* 2020; **38**: e2018247.
206.Schwertner DS, Oliveira R, Koerich M, Motta AF, Pimenta AL, Gioda FR. Prevalence of low back pain in young Brazilians and associated factors: Sex, physical activity, sedentary behavior, sleep and body mass index. *J Back Musculoskelet Rehabil* 2020; **33**(2): 233-44.
207.de Souza Dantas M, Dos Santos MC, Lopes LAF, Guedes DP, Guedes MRA, Oesterreich SA. Clustering of Excess Body Weight-Related Behaviors in a Sample of Brazilian Adolescents. *Nutrients* 2018; **10**(10).
208.Christofaro DG, De Andrade SM, Mesas AE, Fernandes RA, Farias Junior JC. Higher screen time is associated with overweight, poor dietary habits and physical inactivity in Brazilian adolescents, mainly among girls. *Eur J Sport Sci* 2016; **16**(4): 498-506.
209.Werneck AO, Silva DR, Agostinete RR, Fernandes RA, Ronque ERV, Cyrino ES. Social, behavioral and biological correlates of cardiorespiratory fitness according to sex, nutritional status and maturity status among adolescents. A cross-sectional study. *Sao Paulo Med J* 2018; **136**(3): 237-44.
210.de Assis MA, Rolland-Cachera MF, Grosseman S, et al. Obesity, overweight and thinness in schoolchildren of the city of Florianopolis, Southern Brazil. *Eur J Clin Nutr* 2005; **59**(9): 1015-21.
211.Salas MMS, Vargas-Ferreira F, Nascimento GG, Huysmanns MC, Demarco FF. Tooth Erosion Association with Obesity: Findings from a Brazilian Survey in Schoolchildren. *Pesquisa Brasileira em Odontopediatria e Clínica Integrada* 2018; **18**(1): 1-13.
212.Assis MM, Leite MA, Carmo ASD, et al. Food environment, social deprivation and obesity among students from Brazilian public schools. *Public Health Nutr* 2019; **22**(11): 1920-7.
213.Borges Dalmaso S, Grippa Sant'ana P, Pimentel Cordeiro J, et al. Prevalência de Sobrepeso e Obesidade em Escolares do Ensino Fundamental do Vitória/ES. *O Mundo da Saúde* 2019; **43**(1): 061-82.
214.Lima AS, Pitangui ACR, Gomes MRA, Mola R, Araujo RC. Risky sexual behaviors and their association with overweight and obesity among adolescent students: a cross-sectional study. *Einstein (Sao Paulo)* 2019; **17**(3): eAO4782.
215.Porcelli ICdS, Corsi NM, Barata TdJE, et al. Sweetness Taste Preference Levels and Their Relationship to the Nutritional and Dental Caries Patterns among Brazilian Preschool Children. *Pesquisa Brasileira em Odontopediatria e Clínica Integrada* 2019; **19**(1): 1-13.
216.Alves MD, Retondario A, Bricarello LP, et al. Association between dietary patterns and overweight/obesity: a Brazilian national school-based research (ERICA 2013-2014). *J Public Health-Heidelberg* 2020; **28**(2): 163-71.
217.Folmann AG, Wolf VLW, Roman EP, Guerra-Junior G. Prevalence of overweight in adolescents from a Southern Brazilian city according to different anthropometric indexes. *Rev Paul Pediatr* 2020; **39**: e2019277.
218.Hercules E, Peralta M, Henriques-Neto D, et al. The influence of socioeconomic status and age on the prevalence of overweight and obesity among 5 to 10-year-old children in Curitiba, Brazil. *Am J Hum Biol* 2020; **32**(6): e23424.
219.Lucena PN, Bueno NB, Vieira KA, Cabral MJ, Clemente APG, Florencio T. Food insecurity and weight status of socially vulnerable child beneficiaries of a food assistance programme in Maceio, Northeast Brazil. *Public Health Nutr* 2020; **23**(4): 721-6.
220.Rocha S, Rocha HAL, Leite AJM, et al. Environmental, Socioeconomic, Maternal, and Breastfeeding Factors
© 2024 Zhang X et al. *JAMA Pediatrics.*

Associated with Childhood Overweight and Obesity in Ceara, Brazil: A Population-Based Study. *Int J Environ Res Public Health* 2020; **17**(5).
221.Rivera IR, Silva MA, Silva RD, Oliveira BA, Carvalho AC. Physical inactivity, TV-watching hours and body composition in children and adolescents. *Arq Bras Cardiol* 2010; **95**(2): 159-65.
222.Gabriel CG, Corso AC, Caldeira GV, Gimeno SG, Schmitz Bde A, de Vasconcelos Fde A. Overweight and obesity related factors in schoolchildren in Santa Catarina State, Brazil. *Arch Latinoam Nutr* 2010; **60**(4): 332-9.
223.Andreasi V, Michelin E, Rinaldi AE, Burini RC. Physical fitness and associations with anthropometric measurements in 7 to 15-year-old school children. *J Pediatr (Rio J)* 2010; **86**(6): 497-502.
224.Molina Mdel C, de Faria CP, Montero P, Cade NV. Correspondence between children's nutritional status and mothers' perceptions: a population-based study. *Cad Saude Publica* 2009; **25**(10): 2285-90.
225.Fernandes RA, Conterato I, Messias KP, Christofaro DG, de Oliveira AR, Freitas Júnior IF. [Risk factors associated with overweight among adolescents from western Sao Paulo state]. *Rev Esc Enferm USP* 2009; **43**(4): 768-73.
226.Alves JG, Siqueira PP, Figueiroa JN. Overweight and physical inactivity in children living in favelas in the metropolitan region of Recife, Brazil. *J Pediatr (Rio J)* 2009; **85**(1): 67-71.
227.Pelegrini A, Petroski EL, Coqueiro Rda S, Gaya AC. Overweight and obesity in Brazilian schoolchildren aged 10 to 15 years: data from a Brazilian sports project. *Arch Latinoam Nutr* 2008; **58**(4): 343-9.
228.Bertolace Mdo P, Toledo E, Jorge PP, Liberatore Junior Rdel R. Association between obesity and asthma among teenagers. *Sao Paulo Med J* 2008; **126**(5): 285-7.
229.Oliveira AM, Oliveira AC, Almeida MS, Oliveira N, Adan L. Influence of the family nucleus on obesity in children from northeastern Brazil: a cross-sectional study. *BMC Public Health* 2007; **7**: 235.
230.de Souza Valente da Silva L, Valeria da Veiga G, Ramalho RA. Association of serum concentrations of retinol and carotenoids with overweight in children and adolescents. *Nutrition* 2007; **23**(5): 392-7.
231.Granville-Garcia AF, de Menezes VA, de Lira PI. Dental trauma and associated factors in Brazilian preschoolers. *Dent Traumatol* 2006; **22**(6): 318-22.
232.Lima MC, Romaldini CC, Romaldini JH. Frequency of obesity and related risk factors among school children and adolescents in a low-income community. A cross-sectional study. *Sao Paulo Med J* 2015; **133**(2): 125-30.
233.Weinmayr G, Forastiere F, Buchele G, et al. Overweight/obesity and respiratory and allergic disease in children: international study of asthma and allergies in childhood (ISAAC) phase two. *PLoS One* 2014; **9**(12): e113996.
234.Muller Rde M, Tomasi E, Facchini LA, et al. Prevalence of overweight and associated factors in under-five-year-old children in urban population in Brazil. *Rev Bras Epidemiol* 2014; **17**(2): 285-96.
235.De Arruda Moreira M, Coelho Cabral P, Da Silva Ferreira H, Cabral de Lira PI. Prevalence and factors associated with overweight and obesity in children under five in Alagoas, Northeast of Brazil; a population-based study. *Nutr Hosp* 2014; **29**(6): 1320-6.
236.Mendonca KL, Sousa AL, Carneiro CS, et al. Does nutritional status interfere with adolescents' body image perception? *Eat Behav* 2014; **15**(3): 509-12.
237.Crispim PA, Peixoto Mdo R, Jardim PC. Risk factors associated with high blood pressure in two-to five-year-old children. *Arq Bras Cardiol* 2014; **102**(1): 39-46.
238.Menezes RC, Lira PI, Oliveira JS, et al. Prevalence and determinants of overweight in preschool children. *J Pediatr (Rio J)* 2011; **87**(3): 231-7.
239.de Carvalho Cremm E, Marrocos Leite FH, de Abreu DS, de Oliveira MA, Scagliusi FB, Martins PA. Factors associated with overweight in children living in the neighbourhoods of an urban area of Brazil. *Public Health Nutr* 2012; **15**(6): 1056-64.
240.Nascimento VG, Silva JP, Bertoli CJ, Abreu LC, Valenti VE, Leone C. Prevalence of overweight preschool
© 2024 Zhang X et al. *JAMA Pediatrics.*

children in public day care centers: a cross-sectional study. *Sao Paulo Med J* 2012; **130**(4): 225-9.
241.Novaes JF, Lamounier JA, Colosimo EA, Franceschini SC, Priore SE. Breastfeeding and obesity in Brazilian children. *Eur J Public Health* 2012; **22**(3): 383-9.
242.Rosaneli CF, Baena CP, Auler F, et al. Elevated blood pressure and obesity in childhood: a cross-sectional evaluation of 4,609 schoolchildren. *Arq Bras Cardiol* 2014; **103**(3): 238-44.
243.Ferreira Marques CD, Ribeiro Silva Rde C, Machado ME, et al. The prevalence of overweight and obesity in adolescents in Bahia, Brazil. *Nutr Hosp* 2013; **28**(2): 491-6.
244.Reuter CP, Burgos LT, Camargo MD, et al. Prevalence of obesity and cardiovascular risk among children and adolescents in the municipality of Santa Cruz do Sul, Rio Grande do Sul. *Sao Paulo Med J* 2013; **131**(5): 323-30.
245.Andrade FJ, Sales-Peres Ade C, Moura-Grec PG, Mapengo MA, Sales-Peres A, Sales-Peres SH. Nutritional status, tooth wear and quality of life in Brazilian schoolchildren. *Public Health Nutr* 2016; **19**(8): 1479-85.
246.Casonatto J, Fernandes RA, Batista MB, et al. Association between health-related physical fitness and body mass index status in children. *J Child Health Care* 2016; **20**(3): 294-303.
247.Farias Junior JC, Mendonca G. Temporal trend in overweight among adolescents in northeastern Brazil. *Arch Endocrinol Metab* 2016; **60**(1): 21-8.
248.Jardim-Botelho A, Queiroz Gurgel R, Simeone Henriques G, et al. Micronutrient deficiencies in normal and overweight infants in a low socio-economic population in north-east Brazil. *Paediatr Int Child Health* 2016; **36**(3): 198-202.
249.Silva KEdS, Pelegrini A, Pinto AdA, Ronque ERV, Cyrino ES, Filho AdAB. Nutritional status of schoolchildren aged 7-10 years enrolled in public and private schools of Cascavel, Paraná, Brazil. *Revista de Nutrição* 2016; **29**(5): 699-708.
250.Araujo AM, Brandao SA, Araujo MA, Frota KM, Moreira-Araujo RS. Overweight and obesity in preschoolers: Prevalence and relation to food consumption. *Rev Assoc Med Bras (1992)* 2017; **63**(2): 124-33.
251.Lazaro Cuesta L, Rearte A, Rodriguez S, et al. Anthropometric and biochemical assessment of nutritional status and dietary intake in school children aged 6-14 years, Province of Buenos Aires, Argentina. *Arch Argent Pediatr* 2018; **116**(1): e34-e46.
252.Dos Santos FK, Moura Dos Santos MA, Almeida MB, et al. Biological and behavioral correlates of body weight status among rural Northeast Brazilian schoolchildren. *Am J Hum Biol* 2018; **30**(3): e23096.
253.Pivatto BC, Lima LAd. Agreement between the nutritional status of schoolchildren and the perception of their guardians. *Revista de Nutrição* 2018; **31**(2): 175-81.
254.Reuter CP, de Mello ED, da Silva PT, et al. Overweight and Obesity in Schoolchildren: Hierarchical Analysis of Associated Demographic, Behavioral, and Biological Factors. *J Obes* 2018; **2018**: 6128034.
255.Mladenova S, Andreenko E. Prevalence of underweight, overweight, general and central obesity among 8-15-years old Bulgarian children and adolescents (Smolyan region, 2012-2014). *Nutr Hosp* 2015; **31**(6): 2419-27.
256.Gorog K, Pattenden S, Antova T, et al. Maternal smoking during pregnancy and childhood obesity: results from the CESAR Study. *Matern Child Health J* 2011; **15**(7): 985-92.
257.Wijnhoven TM, van Raaij JM, Yngve A, et al. WHO European Childhood Obesity Surveillance Initiative: health-risk behaviours on nutrition and physical activity in 6-9-year-old schoolchildren. *Public Health Nutr* 2015; **18**(17): 3108-24.
258.Roos E, Pajunen T, Ray C, et al. Does eating family meals and having the television on during dinner correlate with overweight? A sub-study of the PRO GREENS project, looking at children from nine European countries. *Public Health Nutr* 2014; **17**(11): 2528-36.
259.Mank I, De Neve JW, Mauti J, et al. Prevalence of Obesity and Anemia Among Early Adolescents in Junior Secondary Schools: A Cross-Sectional Study in Ouagadougou, Burkina Faso. *J Sch Health* 2022; **92**(11): 1081-95.
© 2024 Zhang X et al. *JAMA Pediatrics.*

260.Daboné C, Delisle HF, Receveur O. Poor nutritional status of schoolchildren in urban and peri-urban areas of Ouagadougou (Burkina Faso). *Nutr J* 2011; **10**: 34.
261.Gebremedhin S. Prevalence and differentials of overweight and obesity in preschool children in Sub-Saharan Africa. *BMJ Open* 2015; **5**(12): e009005.
262.Dimaisip-Nabuab J, Duijster D, Benzian H, et al. Nutritional status, dental caries and tooth eruption in children: a longitudinal study in Cambodia, Indonesia and Lao PDR. *BMC Pediatr* 2018; **18**(1): 300.
263.Navti LK, Foudjo BUS. 10-Year Changes in Adiposity in Cameroon School-Age Children: Evidence for Increasing Central Adiposity and Higher Adiposity Levels in Tallest-for-Age Children. *J Obes* 2021; **2021**: 6866911.
264.Navti LK, Ferrari U, Tange E, Bechtold-Dalla Pozza S, Parhofer KG. Contribution of socioeconomic status, stature and birth weight to obesity in Sub-Saharan Africa: cross-sectional data from primary school-age children in Cameroon. *BMC Public Health* 2014; **14**: 320.
265.Choukem SP, Kamdeu-Chedeu J, Leary SD, et al. Overweight and obesity in children aged 3-13 years in urban Cameroon: a cross-sectional study of prevalence and association with socio-economic status. *BMC Obes* 2017; **4**: 7.
266.Chelo D, Mah EM, Chiabi EN, et al. Prevalence and factors associated with hypertension in primary school children, in the centre region of Cameroon. *Transl Pediatr* 2019; **8**(5): 391-7.
267.Dapi LN, Janlert U, Nouedoui C, Stenlund H, Haglin L. Socioeconomic and gender differences in adolescents' nutritional status in urban Cameroon, Africa. *Nutr Res* 2009; **29**(5): 313-9.
268.Navti LK, Ferrari U, Tange E, Parhofer KG, Pozza SB. Height-obesity relationship in school children in Sub-Saharan Africa: results of a cross-sectional study in Cameroon. *BMC Res Notes* 2015; **8**: 98.
269.Wamba PC, Enyong Oben J, Cianflone K. Prevalence of overweight, obesity, and thinness in Cameroon urban children and adolescents. *J Obes* 2013; **2013**: 737592.
270.Navti LK, Atanga MB, Niba LL. Associations of out of school physical activity, sedentary lifestyle and socioeconomic status with weight status and adiposity of Cameroon children. *BMC Obes* 2017; **4**: 35.
271.Van Hulst A, Wills-Ibarra N, Nikiema B, Kakinami L, Pratt KJ, Ball GDC. Associations between family functioning during early to mid-childhood and weight status in childhood and adolescence: findings from a Quebec birth cohort. *Int J Obes (Lond)* 2022; **46**(5): 986-91.
272.Anderson LN, Fatima T, Shah B, et al. Income and neighbourhood deprivation in relation to obesity in urban dwelling children 0-12 years of age: a cross-sectional study from 2013 to 2019. *J Epidemiol Community Health* 2022; **76**(3): 274-80.
273.Heroux M, Iannotti RJ, Currie D, Pickett W, Janssen I. The food retail environment in school neighborhoods and its relation to lunchtime eating behaviors in youth from three countries. *Health Place* 2012; **18**(6): 1240-7.
274.Le H, Engler-Stringer R, Muhajarine N. Walkable home neighbourhood food environment and children's overweight and obesity: Proximity, density or price? *Can J Public Health* 2016; **107**(Suppl 1): 5347.
275.Seliske LM, Pickett W, Boyce WF, Janssen I. Association between the food retail environment surrounding schools and overweight in Canadian youth. *Public Health Nutr* 2009; **12**(9): 1384-91.
276.Ball GDC, Savu A, Kaul P. Changes in the prevalence of overweight, obesity, and severe obesity between 2010 and 2017 in preschoolers: A population-based study. *Pediatr Obes* 2019; **14**(11): e12561.
277.Dubois L, Girard M, Potvin Kent M, Farmer A, Tatone-Tokuda F. Breakfast skipping is associated with differences in meal patterns, macronutrient intakes and overweight among pre-school children. *Public Health Nutr* 2009; **12**(1): 19-28.
278.Chaput JP, Brunet M, Tremblay A. Relationship between short sleeping hours and childhood overweight/obesity: results from the 'Quebec en Forme' Project. *Int J Obes (Lond)* 2006; **30**(7): 1080-5.
279.Chaput JP, Lambert M, Gray-Donald K, et al. Short sleep duration is independently associated with overweight
© 2024 Zhang X et al. *JAMA Pediatrics.*

and obesity in Quebec children. *Can J Public Health* 2011; **102**(5): 369-74.
280.Davidson K, Schroth RJ, Levi JA, Yaffe AB, Mittermuller BA, Sellers EAC. Higher body mass index associated with severe early childhood caries. *BMC Pediatr* 2016; **16**: 137.
281.Brault MC, Turcotte O, Aime A, Cote M, Begin C. Body Mass Index Accuracy in Preadolescents: Can We Trust Self-Report or Should We Seek Parent Report? *J Pediatr* 2015; **167**(2): 366-71.
282.Larsen K, Cook B, Stone MR, Faulkner GE. Food access and children's BMI in Toronto, Ontario: assessing how the food environment relates to overweight and obesity. *Int J Public Health* 2015; **60**(1): 69-77.
283.Azad MB, Bridgman SL, Becker AB, Kozyrskyj AL. Infant antibiotic exposure and the development of childhood overweight and central adiposity. *Int J Obes (Lond)* 2014; **38**(10): 1290-8.
284.Bridgman SL, Azad MB, Persaud RR, et al. Impact of maternal pre-pregnancy overweight on infant overweight at 1 year of age: associations and sex-specific differences. *Pediatr Obes* 2018; **13**(10): 579-89.
285.Cassidy-Bushrow AE, Burmeister C, Havstad S, et al. Prenatal antimicrobial use and early-childhood body mass index. *Int J Obes (Lond)* 2018; **42**(1): 1-7.
286.Shields M, Tremblay MS. Canadian childhood obesity estimates based on WHO, IOTF and CDC cut-points. *Int J Pediatr Obes* 2010; **5**(3): 265-73.
287.Medehouenou TC, Ayotte P, St-Jean A, et al. Overweight and Obesity Prevalence Among School-Aged Nunavik Inuit Children According to Three Body Mass Index Classification Systems. *J Adolesc Health* 2015; **57**(1): 31-6.
288.Wang F, Veugelers PJ. Self-esteem and cognitive development in the era of the childhood obesity epidemic. *Obes Rev* 2008; **9**(6): 615-23.
289.Mai XM, Becker AB, Sellers EA, Liem JJ, Kozyrskyj AL. The relationship of breast-feeding, overweight, and asthma in preadolescents. *J Allergy Clin Immunol* 2007; **120**(3): 551-6.
290.Shi Y, De Groh M, Morrison H. Perinatal and early childhood factors for overweight and obesity in young Canadian children. *Can J Public Health* 2013; **104**(1): e69-74.
291.Walton K, Simpson JR, Darlington G, Haines J. Parenting stress: a cross-sectional analysis of associations with childhood obesity, physical activity, and TV viewing. *BMC Pediatr* 2014; **14**: 244.
292.Oliver LN, Hayes MV. Neighbourhood socio-economic status and the prevalence of overweight Canadian children and youth. *Can J Public Health* 2005; **96**(6): 415-20.
293.Moffat T, Galloway T, Latham J. Stature and adiposity among children in contrasting neighborhoods in the city of Hamilton, Ontario, Canada. *Am J Hum Biol* 2005; **17**(3): 355-67.
294.Ball GD, Marshall JD, McCargar LJ. Physical activity, aerobic fitness, self-perception, and dietary intake in at risk of overweight and normal weight children. *Can J Diet Pract Res* 2005; **66**(3): 162-9.
295.Woodruff SJ, Hanning RM, McGoldrick K, Brown KS. Healthy eating index-C is positively associated with family dinner frequency among students in grades 6-8 from Southern Ontario, Canada. *Eur J Clin Nutr* 2010; **64**(5): 454-60.
296.Twells L, Newhook LA. Can exclusive breastfeeding reduce the likelihood of childhood obesity in some regions of Canada? *Can J Public Health* 2010; **101**(1): 36-9.
297.Menon S, Philipneri A, Ratnasingham S, Manson H. The integrated role of multiple healthy weight behaviours on overweight and obesity among adolescents: a cross-sectional study. *BMC Public Health* 2019; **19**(1): 1157.
298.Shay M, Tomfohr-Madsen L, Tough S. Maternal psychological distress and child weight at 24 months: investigating indirect effects through breastfeeding in the All Our Families cohort. *Can J Public Health* 2020; **111**(4): 543-54.
299.Simen-Kapeu A, Veugelers PJ. Should public health interventions aimed at reducing childhood overweight and obesity be gender-focused? *BMC Public Health* 2010; **10**: 340.
300.Leatherdale ST. The association between overweight and school policies on physical activity: a multilevel
© 2024 Zhang X et al. *JAMA Pediatrics.*

analysis among elementary school youth in the PLAY-On study. *Health Educ Res* 2010; **25**(6): 1061-73.
301. Khalil CB, Johnson-Down L, Egeland GM. Emerging obesity and dietary habits among James Bay Cree youth. *Public Health Nutr* 2010; **13**(11): 1829-37.
302. Ismailov RM, Leatherdale ST. Rural-urban differences in overweight and obesity among a large sample of adolescents in Ontario. *Int J Pediatr Obes* 2010; **5**(4): 351-60.
303. Galloway T, Young TK, Egeland GM. Emerging obesity among preschool-aged Canadian Inuit children: results from the Nunavut Inuit Child Health Survey. *Int J Circumpolar Health* 2010; **69**(2): 151-7.
304. Doan Q, Koehoorn M, Kissoon N. Body mass index and the risk of acute injury in adolescents. *Paediatr Child Health* 2010; **15**(6): 351-6.
305. Cairney J, Hay J, Veldhuizen S, Missiuna C, Mahlberg N, Faught BE. Trajectories of relative weight and waist circumference among children with and without developmental coordination disorder. *CMAJ* 2010; **182**(11): 1167-72.
306. Garcia Bengoechea E, Sabiston CM, Ahmed R, Farnoush M. Exploring links to unorganized and organized physical activity during adolescence: the role of gender, socioeconomic status, weight status, and enjoyment of physical education. *Res Q Exerc Sport* 2010; **81**(1): 7-16.
307. Wahi G, Zorzi A, Macnab A, Panagiotopoulos C. Prevalence of type 2 diabetes, obesity and the metabolic syndrome among Canadian First Nations children in a remote Pacific coast community. *Paediatr Child Health* 2009; **14**(2): 79-83.
308. Vance VA, Woodruff SJ, McCargar LJ, Husted J, Hanning RM. Self-reported dietary energy intake of normal weight, overweight and obese adolescents. *Public Health Nutr* 2009; **12**(2): 222-7.
309. Potestio ML, Patel AB, Powell CD, McNeil DA, Jacobson RD, McLaren L. Is there an association between spatial access to parks/green space and childhood overweight/obesity in Calgary, Canada? *Int J Behav Nutr Phys Act* 2009; **6**: 77.
310. McGavock JM, Torrance BD, McGuire KA, Wozny PD, Lewanczuk RZ. Cardiorespiratory fitness and the risk of overweight in youth: the Healthy Hearts Longitudinal Study of Cardiometabolic Health. *Obesity (Silver Spring)* 2009; **17**(9): 1802-7.
311. Downs SM, Arnold A, Marshall D, McCargar LJ, Raine KD, Willows ND. Associations among the food environment, diet quality and weight status in Cree children in Quebec. *Public Health Nutr* 2009; **12**(9): 1504-11.
312. Spence JC, Cutumisu N, Edwards J, Evans J. Influence of neighbourhood design and access to facilities on overweight among preschool children. *Int J Pediatr Obes* 2008; **3**(2): 109-16.
313. Salvadori M, Sontrop JM, Garg AX, et al. Elevated blood pressure in relation to overweight and obesity among children in a rural Canadian community. *Pediatrics* 2008; **122**(4): e821-7.
314. Edwards J, Evans J, Brown AD. Using routine growth data to determine overweight and obesity prevalence estimates in preschool children in the Capital Health Region of Alberta. *Can J Public Health* 2008; **99**(2): 91-4.
315. Bruner MW, Lawson J, Pickett W, Boyce W, Janssen I. Rural Canadian adolescents are more likely to be obese compared with urban adolescents. *Int J Pediatr Obes* 2008; **3**(4): 205-11.
316. Willows ND, Johnson MS, Ball GD. Prevalence estimates of overweight and obesity in Cree preschool children in northern Quebec according to international and US reference criteria. *Am J Public Health* 2007; **97**(2): 311-6.
317. He M, Evans A. Are parents aware that their children are overweight or obese? Do they care? *Can Fam Physician* 2007; **53**(9): 1493-9.
318. Bassett DR, Jr., Tremblay MS, Esliger DW, Copeland JL, Barnes JD, Huntington GE. Physical activity and body mass index of children in an old order Amish community. *Med Sci Sports Exerc* 2007; **39**(3): 410-5.
319. Ng C, Marshall D, Willows ND. Obesity, adiposity, physical fitness and activity levels in Cree children. *Int J Circumpolar Health* 2006; **65**(4): 322-30.
320. Janssen I, Boyce WF, Simpson K, Pickett W. Influence of individual- and area-level measures of socioeconomic

© 2024 Zhang X et al. *JAMA Pediatrics.*

status on obesity, unhealthy eating, and physical inactivity in Canadian adolescents. *Am J Clin Nutr* 2006; **83**(1): 139-45.
321.Galloway T. Obesity rates among rural Ontario schoolchildren. *Can J Public Health* 2006; **97**(5): 353-6.
322.Rossiter MD, Colapinto CK, Khan MK, et al. Breast, Formula and Combination Feeding in Relation to Childhood Obesity in Nova Scotia, Canada. *Matern Child Health J* 2015; **19**(9): 2048-56.
323.Borghese MM, Tremblay MS, Leduc G, et al. Television viewing and food intake during television viewing in normal-weight, overweight and obese 9- to 11-year-old Canadian children: a cross-sectional analysis. *J Nutr Sci* 2015; **4**: e8.
324.Banerjee AT, Flora PK, Stone M, Faulkner G. Differences in the Prevalence of Overweight Between 10-12-Year-old South Asian and Non-South Asian Children in Toronto, Ontario: Findings from Project BEAT. *J Racial Ethn Health Disparities* 2015; **2**(2): 184-91.
325.Carson V, Stone M, Faulkner G. Patterns of sedentary behavior and weight status among children. *Pediatr Exerc Sci* 2014; **26**(1): 95-102.
326.Leatherdale ST. A cross-sectional examination of school characteristics associated with overweight and obesity among grade 1 to 4 students. *BMC Public Health* 2013; **13**: 982.
327.Delisle Nystrom C, Traversy G, Barnes JD, Chaput JP, Longmuir PE, Tremblay MS. Associations between domains of physical literacy by weight status in 8- to 12-year-old Canadian children. *BMC Public Health* 2018; **18**(Suppl 2): 1043.
328.Pena-Jorquera H, Campos-Nunez V, Sadarangani KP, Ferrari G, Jorquera-Aguilera C, Cristi-Montero C. Breakfast: A Crucial Meal for Adolescents' Cognitive Performance According to Their Nutritional Status. The Cogni-Action Project. *Nutrients* 2021; **13**(4).
329.Duran Aguero S, Haro Rivera P. Association between the amount of sleep and obesity in Chilean schoolchildren. *Arch Argent Pediatr* 2016; **114**(2): 114-9.
330.Olivares S, Kain J, Lera L, Pizarro F, Vio F, Moron C. Nutritional status, food consumption and physical activity among Chilean school children: a descriptive study. *Eur J Clin Nutr* 2004; **58**(9): 1278-85.
331.Cediel G, Corvalan C, Aguirre C, de Romana DL, Uauy R. Serum 25-Hydroxyvitamin D associated with indicators of body fat and insulin resistance in prepubertal chilean children. *Int J Obes (Lond)* 2016; **40**(1): 147-52.
332.Mardones F, Villarroel L, Karzulovic L, et al. Association of perinatal factors and obesity in 6- to 8-year-old Chilean children. *Int J Epidemiol* 2008; **37**(4): 902-10.
333.Delgado Floody PA, Jerez Mayorga D, Caamano-Navarrete F, Carter-Thuillier B, Cofre-Lizama A, Alvarez C. Psychological well-being related to screen time, physical activity after school, and weight status in Chilean schoolchildren. *Nutr Hosp* 2019; **36**(6): 1254-60.
334.Corvalan C, Uauy R, Kain J, Martorell R. Obesity indicators and cardiometabolic status in 4-y-old children. *Am J Clin Nutr* 2010; **91**(1): 166-74.
335.Castillo Valenzuela O, Liberona Zuniga J, Dominguez de Landa A, et al. Consumption of Ready-to-Eat Cereal Is Inversely Associated with Body Mass Index in 6-13 Years Old Chilean Schoolchildren. *Nutr Hosp* 2015; **32**(5): 2301-8.
336.Lizana PA, Simpson C, Yanez L, Saavedra K. Body image and weight status of children from rural areas of Valparaiso, Chile. *Nutr Hosp* 2014; **31**(2): 698-703.
337.Cadenas-Sanchez C, Artero EG, Concha F, Leyton B, Kain J. Anthropometric Characteristics and Physical Fitness Level in Relation to Body Weight Status in Chilean Preschool Children. *Nutr Hosp* 2015; **32**(1): 346-53.
338.Heitzinger K, Velez JC, Parra SG, Barbosa C, Fitzpatrick AL. Caregiver perceptions of child nutritional status in Magallanes, Chile. *Obes Res Clin Pract* 2014; **8**(1): e98-105.
339.Silva JR, Capurro G, Saumann MP, Slachevsky A. Problematic eating behaviors and nutritional status in 7 to 12
© 2024 Zhang X et al. *JAMA Pediatrics.*

year-old Chilean children. *International Journal of Clinical and Health Psychology* 2013; **13**(1): 32-9.
340.Kagawa RM, Fernald LC, Behrman JR. Weight Status and Behavioral Problems among Very Young Children in Chile. *PLoS One* 2016; **11**(9): e0161380.
341.Garcia-Hermoso A, Marina R. Relationship of weight status, physical activity and screen time with academic achievement in adolescents. *Obes Res Clin Pract* 2017; **11**(1): 44-50.
342.Liu X, Chen G, Yan J, Luo J. Weight status and bullying behaviors among Chinese school-aged children. *Child Abuse Negl* 2016; **52**: 11-9.
343.Li L, Shen T, Wen LM, et al. Lifestyle factors associated with childhood obesity: a cross-sectional study in Shanghai, China. *BMC Res Notes* 2015; **8**: 6.
344.Tang J, Gou W, Fu Y, et al. Association between postterm pregnancy and adverse growth outcomes in preschool-age children. *Am J Clin Nutr* 2022; **116**(2): 482-90.
345.Su W, Song Q, Li N, et al. The effect of air pollution and emotional and behavioral problems on preschoolers' overweight and obesity. *Environ Sci Pollut Res Int* 2022; **29**(50): 75587-96.
346.Shi R, Lin C, Li S, Deng L, Lin Z, Xiu L. Obesity is negatively associated with dental caries among children and adolescents in Huizhou: a cross-sectional study. *BMC Oral Health* 2022; **22**(1): 76.
347.Shi J, He L, Yu D, et al. Prevalence and Correlates of Metabolic Syndrome and Its Components in Chinese Children and Adolescents Aged 7-17: The China National Nutrition and Health Survey of Children and Lactating Mothers from 2016-2017. *Nutrients* 2022; **14**(16).
348.Ma Y, Wu H, Shen J, Wang J, Wang J, Hou Y. Correlation between lifestyle patterns and overweight and obesity among Chinese adolescents. *Front Public Health* 2022; **10**: 1027565.
349.Liu N, Li H, Guo Z, et al. Prevalence and factors associated with overweight or obesity among 2- to 6-year-old children in Hunan, China: a cross-sectional study. *Public Health Nutr* 2022; **25**(12): 1-12.
350.Liu F, Lv D, Wang L, et al. Breastfeeding and overweight/obesity among children and adolescents: a cross-sectional study. *BMC Pediatr* 2022; **22**(1): 347.
351.Li W, Yuan J, Wang L, et al. The association between breastfeeding and childhood obesity/underweight: a population-based birth cohort study with repeated measured data. *Int Breastfeed J* 2022; **17**(1): 82.
352.Li S, Zhu Y, Zeng M, et al. Association Between Nutrition Literacy and Overweight/Obesity of Adolescents: A Cross-Sectional Study in Chongqing, China. *Front Nutr* 2022; **9**: 893267.
353.Huang H, Gao Y, Zhu N, et al. The Effects of Breastfeeding for Four Months on Thinness, Overweight, and Obesity in Children Aged 3 to 6 Years: A Retrospective Cohort Study from National Physical Fitness Surveillance of Jiangsu Province, China. *Nutrients* 2022; **14**(19).
354.He Y, Luo B, Zhao L, Liao S. Influences of the COVID-19 Pandemic on Obesity and Weight-Related Behaviors among Chinese Children: A Multi-Center Longitudinal Study. *Nutrients* 2022; **14**(18).
355.Guo Y, Yin X, Sun Y, et al. Research on Environmental Influencing Factors of Overweight and Obesity in Children and Adolescents in China. *Nutrients* 2021; **14**(1).
356.Gong QH, Li SX, Wang SJ, Zhang Y. Self-reported eating speed is associated with overweight among Chinese schoolchildren: a cross-sectional survey. *Eat Weight Disord* 2022; **27**(4): 1297-302.
357.Chen S, Zhang X, Du W, Fan L, Zhang F. Association of insufficient sleep and skipping breakfast with overweight/obesity in children and adolescents: Findings from a cross-sectional provincial surveillance project in Jiangsu. *Pediatr Obes* 2022; **17**(11): e12950.
358.Chen H, Wang LJ, Xin F, Liang G, Chen Y. Associations between sleep duration, sleep quality, and weight status in Chinese children and adolescents. *BMC Public Health* 2022; **22**(1): 1136.
359.Chen G, Chen J, Liu J, Hu Y, Liu Y. Relationship between body mass index and physical fitness of children and adolescents in Xinjiang, China: a cross-sectional study. *BMC Public Health* 2022; **22**(1): 1680.
© 2024 Zhang X et al. *JAMA Pediatrics.*

360.Chang R, Mei H, Zhang Y, Xu K, Yang S, Zhang J. Early childhood body mass index trajectory and overweight/obesity risk differed by maternal weight status. *Eur J Clin Nutr* 2022; **76**(3): 450-5.
361.Zhu J, Tan Y, Lu W, He Y, Yu Z. Current Assessment of Weight, Dietary and Physical Activity Behaviors among Middle and High School Students in Shanghai, China-A 2019 Cross-Sectional Study. *Nutrients* 2021; **13**(12).
362.Zhou S, Lin L, Bao Z, et al. The association of prenatal exposure to particulate matter with infant growth: A birth cohort study in Beijing, China. *Environ Pollut* 2021; **277**: 116792.
363.Zheng J, Gao L, Xue H, et al. Eating-out behaviors, associated factors and associations with obesity in Chinese school children: findings from the childhood obesity study in China mega-cities. *Eur J Nutr* 2021; **60**(6): 3003-12.
364.Zhao S, Li X, Xiang ST, et al. Changes in the age-specific body mass index distribution among urban children between 2002 and 2018 in Changsha, China. *Transl Pediatr* 2021; **10**(3): 502-9.
365.Zhang YQ, Li H, Wu HH, Zong XN. Stunting, wasting, overweight and their coexistence among children under 7 years in the context of the social rapidly developing: Findings from a population-based survey in nine cities of China in 2016. *PLoS One* 2021; **16**(1): e0245455.
366.Zhang Y, Sun J, Zhao M, Magnussen CG, Xi B. Prevalence of thinness, overweight and obesity among Tibetan adolescents aged 12-17 years. *Public Health Nutr* 2021; **24**(13): 4017-22.
367.Zhang L, Chen J, Zhang J, et al. Regional Disparities in Obesity Among a Heterogeneous Population of Chinese Children and Adolescents. *JAMA Netw Open* 2021; **4**(10): e2131040.
368.Yuan J, Jiang X, Zhu T, et al. Caregivers' feeding behaviour, children's eating behaviour and weight status among children of preschool age in China. *J Hum Nutr Diet* 2021; **34**(5): 807-18.
369.You Y, Zhang F, Han J, et al. Breakfast preferences and consumption location among children at a median age of 7 in Shenzhen: Implications for managing overweight and obesity. *Appetite* 2021; **156**: 104851.
370.Xu X, Han YQ, Yu ZW, Cheng F, Lu JK. Difference in overweight/obesity rates between Chinese and Japanese teenagers. *Int J Clin Exp Med* 2021; **14**(2): 1274-82.
371.Wang S, Li W, Qiao Y, et al. Abnormal glucose in pre-school children and the association with obesity/physical activity: A large population-based study. *Diabetes Metab Res Rev* 2021; **37**(7): e3442.
372.Wang L, Xu Z, Li N, et al. The association between overweight and obesity on bone mineral density in 12 to 15 years old adolescents in China. *Medicine (Baltimore)* 2021; **100**(32): e26872.
373.Sun X, Zhao B, Liu J, et al. A 3-year longitudinal study of the association of physical activity and sedentary behaviours with childhood obesity in China: The childhood obesity study in China mega-cities. *Pediatr Obes* 2021; **16**(6): e12753.
374.Min K, Wang J, Liao W, et al. Dietary patterns and their associations with overweight/obesity among preschool children in Dongcheng District of Beijing: a cross-sectional study. *BMC Public Health* 2021; **21**(1): 223.
375.Liu M, Cao B, Liu M, et al. High Prevalence of Obesity but Low Physical Activity in Children Aged 9-11 Years in Beijing. *Diabetes Metab Syndr Obes* 2021; **14**: 3323-35.
376.Zhou S, Ye B, Fu P, et al. Double Burden of Malnutrition: Examining the Growth Profile and Coexistence of Undernutrition, Overweight, and Obesity among School-Aged Children and Adolescents in Urban and Rural Counties in Henan Province, China. *J Obes* 2020; **2020**: 2962138.
377.Zhou S, Cheng Y, Cheng L, et al. Association between convenience stores near schools and obesity among school-aged children in Beijing, China. *BMC Public Health* 2020; **20**(1): 150.
378.Zheng H, Long W, Tan W, Yang C, Cao M, Zhu Y. Anaemia, iron deficiency, iron-deficiency anaemia and their associations with obesity among schoolchildren in Guangzhou, China. *Public Health Nutr* 2020; **23**(10): 1693-702.
379.Xu Y, Mei M, Wang H, Yan Q, He G. Association between Weight Status and Physical Fitness in Chinese Mainland Children and Adolescents: A Cross-Sectional Study. *Int J Environ Res Public Health* 2020; **17**(7).
380.Tang J, Yu Y, Du Y, Ma Y, Zhu H, Liu Z. Association between actual weight status, perceived weight and
© 2024 Zhang X et al. *JAMA Pediatrics.*

depressive, anxious symptoms in Chinese adolescents: a cross-sectional study. *BMC Public Health* 2010; **10**: 594.
381.Shan XY, Xi B, Cheng H, Hou DQ, Wang Y, Mi J. Prevalence and behavioral risk factors of overweight and obesity among children aged 2-18 in Beijing, China. *Int J Pediatr Obes* 2010; **5**(5): 383-9.
382.Zhang YX, Wang SR. Blood pressure level profiles among children and adolescents with various types of obesity: study in a large population in Shandong, China. *Clin Res Cardiol* 2014; **103**(7): 553-9.
383.Abdumijit T, Zhao D, Zhang R. Neighborhood Food Environment and Children's BMI: A New Framework with Structural Equation Modeling. *Nutrients* 2022; **14**(21).
384.Cheng YH, Liao Y, Chen DY, Wang Y, Wu Y. Prevalence of dental caries and its association with body mass index among school-age children in Shenzhen, China. *BMC Oral Health* 2019; **19**(1): 270.
385.Guo Q, Wang B, Cao S, et al. Association between milk intake and childhood growth: results from a nationwide cross-sectional survey. *Int J Obes (Lond)* 2020; **44**(11): 2194-202.
386.Li B, Adab P, Cheng KK. Family and neighborhood correlates of overweight and obesogenic behaviors among Chinese children. *Int J Behav Med* 2014; **21**(4): 700-9.
387.Zheng H, Xu Z, Wang Q, et al. Long-term exposure to ambient air pollution and obesity in school-aged children and adolescents in Jiangsu province of China. *Environ Res* 2021; **195**: 110804.
388.Zhang Z, Dong B, Chen G, et al. Ambient air pollution and obesity in school-aged children and adolescents: A multicenter study in China. *Sci Total Environ* 2021; **771**: 144583.
389.Liu Z, Li Q, Maddison R, et al. A School-Based Comprehensive Intervention for Childhood Obesity in China: A Cluster Randomized Controlled Trial. *Child Obes* 2019; **15**(2): 105-15.
390.Jiang J, Xia X, Greiner T, Wu G, Lian G, Rosenqvist U. The effects of a 3-year obesity intervention in schoolchildren in Beijing. *Child Care Health Dev* 2007; **33**(5): 641-6.
391.Li YP, Hu XQ, Schouten EG, et al. Report on childhood obesity in China (8): effects and sustainability of physical activity intervention on body composition of Chinese youth. *Biomed Environ Sci* 2010; **23**(3): 180-7.
392.Li XH, Lin S, Guo H, et al. Effectiveness of a school-based physical activity intervention on obesity in school children: a nonrandomized controlled trial. *BMC Public Health* 2014; **14**: 1282.
393.Xu F, Ware RS, Leslie E, et al. Effectiveness of a Randomized Controlled Lifestyle Intervention to Prevent Obesity among Chinese Primary School Students: CLICK-Obesity Study. *PLoS One* 2015; **10**(10): e0141421.
394.Meng L, Xu H, Liu A, et al. The costs and cost-effectiveness of a school-based comprehensive intervention study on childhood obesity in China. *PLoS One* 2013; **8**(10): e77971.
395.Ji M, Tang A, Zhang Y, et al. The Relationship between Obesity, Sleep and Physical Activity in Chinese Preschool Children. *Int J Environ Res Public Health* 2018; **15**(3).
396.Cui Z, Huxley R, Wu Y, Dibley MJ. Temporal trends in overweight and obesity of children and adolescents from nine Provinces in China from 1991-2006. *Int J Pediatr Obes* 2010; **5**(5): 365-74.
397.Li Y, Schouten EG, Hu X, Cui Z, Luan D, Ma G. Obesity prevalence and time trend among youngsters in China, 1982-2002. *Asia Pac J Clin Nutr* 2008; **17**(1): 131-7.
398.Zhang M, Guo F, Tu Y, et al. Further increase of obesity prevalence in Chinese children and adolescents--cross-sectional data of two consecutive samples from the city of Shanghai from 2003 to 2008. *Pediatr Diabetes* 2012; **13**(7): 572-7.
399.Ma L, Mai J, Jing J, et al. Empirical change in the prevalence of overweight and obesity in adolescents from 2007 to 2011 in Guangzhou, China. *Eur J Pediatr* 2014; **173**(6): 787-91.
400.Zhang YX, Wang SR. Prevalence and regional distribution of childhood overweight and obesity in Shandong Province, China. *World J Pediatr* 2013; **9**(2): 135-9.
401.Cheng L, Li Q, Hebestreit A, et al. The associations of specific school- and individual-level characteristics with obesity among primary school children in Beijing, China. *Public Health Nutr* 2020; **23**(10): 1838-45.
© 2024 Zhang X et al. *JAMA Pediatrics.*

402.Zhang Y, Zhang X, Li J, Zhong H, Pan CW. Associations of outdoor activity and screen time with adiposity: findings from rural Chinese adolescents with relatively low adiposity risks. *BMC Public Health* 2020; **20**(1): 1769.
403.Xiao P, Dong H, Li H, et al. Adequate 25-hydroxyvitamin D levels are inversely associated with various cardiometabolic risk factors in Chinese children, especially obese children. *BMJ Open Diabetes Res Care* 2020; **8**(1).
404.Andegiorgish AK, Wang J, Zhang X, Liu X, Zhu H. Prevalence of overweight, obesity, and associated risk factors among school children and adolescents in Tianjin, China. *Eur J Pediatr* 2012; **171**(4): 697-703.
405.Yao Y, Ren X, Song X, et al. The relationship between dental caries and obesity among primary school children aged 5 to 14 years. *Nutr Hosp* 2014; **30**(1): 60-5.
406.Zhou X, Dibley MJ, Cheng Y, Ouyang X, Yan H. Validity of self-reported weight, height and resultant body mass index in Chinese adolescents and factors associated with errors in self-reports. *BMC Public Health* 2010; **10**: 190.
407.Li N, Liu E, Guo J, et al. Maternal prepregnancy body mass index and gestational weight gain on offspring overweight in early infancy. *PLoS One* 2013; **8**(10): e77809.
408.Zhang J, Himes JH, Guo Y, et al. Birth weight, growth and feeding pattern in early infancy predict overweight/obesity status at two years of age: a birth cohort study of Chinese infants. *PLoS One* 2013; **8**(6): e64542.
409.Dong GH, Qian Z, Liu MM, et al. Ambient air pollution and the prevalence of obesity in chinese children: The seven northeastern cities study. *Obesity* 2014; **22**(3): 795-800.
410.Zhou P, Li R, Liu K. The Neighborhood Food Environment and the Onset of Child-Hood Obesity: A Retrospective Time-Trend Study in a Mid-sized City in China. *Front Public Health* 2021; **9**: 688767.
411.Zhao Y, Wang L, Xue H, Wang H, Wang Y. Fast food consumption and its associations with obesity and hypertension among children: results from the baseline data of the Childhood Obesity Study in China Mega-cities. *BMC Public Health* 2017; **17**(1): 933.
412.Ma YN, Chen T, Wang D, Liu MM, He QC, Dong GH. Prevalence of overweight and obesity among preschool children from six cities of northeast China. *Arch Med Res* 2011; **42**(7): 633-40.
413.Cao ZQ, Zhu L, Zhang T, Wu L, Wang Y. Blood pressure and obesity among adolescents: a school-based population study in China. *Am J Hypertens* 2012; **25**(5): 576-82.
414.He QQ, Wong TW, Du L, et al. Respiratory health in overweight and obese Chinese children. *Pediatr Pulmonol* 2009; **44**(10): 997-1002.
415.Ying-Xiu Z, Shu-Rong W. Secular trends in body mass index and the prevalence of overweight and obesity among children and adolescents in Shandong, China, from 1985 to 2010. *J Public Health (Oxf)* 2012; **34**(1): 131-7.
416.He F, Guan P, Liu Q, Crabtree D, Peng L, Wang H. The relationship between obesity and body compositions with respect to the timing of puberty in Chongqing adolescents: a cross-sectional study. *BMC Public Health* 2017; **17**(1): 664.
417.Zou Y, Zhang R, Huang L, et al. Serum levels of vitamin D, retinol, zinc, and CRP in relation to obesity among children and adolescents. *Eur J Med Res* 2022; **27**(1): 51.
418.Zheng X, Li R, Wang L, et al. The association of cesarean section with overweight and neurodevelopment of Chinese children aged 1-5 months. *Front Pediatr* 2022; **10**: 940422.
419.Zhang Y, Lou H, Huang Y, et al. Trends of overweight and obesity prevalence in school-aged children among Henan Province from 2000 to 2019. *Front Public Health* 2022; **10**: 1046026.
420.Zhang S, Gao H, Cui Y, et al. Relationship between energy balance-related behaviors and personal and family factors in overweight/obese primary school students aged 10-12 years in China: a cross-sectional study. *BMC Public Health* 2022; **22**(1): 1968.
421.Zeng J, Shen F, Zou ZY, et al. Association of maternal obesity and gestational diabetes mellitus with

overweight/obesity and fatty liver risk in offspring. *World J Gastroenterol* 2022; **28**(16): 1681-91.
422.You Y, Liu R, Zhou H, et al. Effect of Exposure to Paternal Smoking on Overweight and Obesity in Children: Findings from the Children Lifeway Cohort in Shenzhen, Southern China. *Obes Facts* 2022; **15**(4): 609-20.
423.Yang D, Luo C, Feng X, et al. Changes in obesity and lifestyle behaviours during the COVID-19 pandemic in Chinese adolescents: A longitudinal analysis from 2019 to 2020. *Pediatr Obes* 2022; **17**(5): e12874.
424.Xu H, Du S, Liu A, Zhang Q, Ma G. Low Dietary Diversity for Recommended Food Groups Increases the Risk of Obesity among Children: Evidence from a Chinese Longitudinal Study. *Nutrients* 2022; **14**(19).
425.Wu QZ, Xu SL, Tan YW, et al. Exposure to ultrafine particles and childhood obesity: A cross-sectional analysis of the Seven Northeast Cities (SNEC) Study in China. *Sci Total Environ* 2022; **846**: 157524.
426.Wang X, He T, Xu S, et al. Soy Food Intake Associated with Obesity and Hypertension in Children and Adolescents in Guangzhou, Southern China. *Nutrients* 2022; **14**(3).
427.Wang Q, Yang M, Deng X, et al. Explorations on risk profiles for overweight and obesity in 9501 preschool-aged children. *Obes Res Clin Pract* 2022; **16**(2): 106-14.
428.Iwata F, Hara M, Okada T, Harada K, Li S. Body fat ratios in urban Chinese children. *Pediatr Int* 2003; **45**(2): 190-2.
429.Xiong F, Garnett SP, Cowell CT, et al. Waist circumference and waist-to-height ratio in Han Chinese children living in Chongqing, south-west China. *Public Health Nutr* 2011; **14**(1): 20-6.
430.Tan Y, Xin X, Ming Q. Prevalence and characteristics of overweight and obesity among Chinese youth aged 12-18 years: a multistage nationwide survey. *Public Health* 2018; **155**: 152-9.
431.Tan Z, Min J, Xue H, Wang W, Wang Y. Parenting practices and overweight status of junior high school students in China: A nationally representative study of 19,487 students from 112 schools. *Prev Med* 2018; **107**: 1-7.
432.Wang H, Hu R, Du H, Fiona B, Zhong J, Yu M. The relationship between sleep duration and obesity risk among school students: a cross-sectional study in Zhejiang, China. *Nutr Metab (Lond)* 2018; **15**: 48.
433.Zhai J, Xue H, Luo J, Zhang L, Cheng G. Associations between socioeconomic status and overweight among urban children aged 7-12 years in Chengdu, southwest China. *Asia Pac J Clin Nutr* 2018; **27**(3): 617-23.
434.Zhang J, Li X, Hawley N, et al. Trends in the Prevalence of Overweight and Obesity among Chinese School-Age Children and Adolescents from 2010 to 2015. *Child Obes* 2018; **14**(3): 182-8.
435.Zhang J, Wang H, Wang Z, et al. Prevalence and stabilizing trends in overweight and obesity among children and adolescents in China, 2011-2015. *BMC Public Health* 2018; **18**(1): 571.
436.Zhang X, Zhang F, Yang J, et al. Prevalence of overweight and obesity among primary school-aged children in Jiangsu Province, China, 2014-2017. *PLoS One* 2018; **13**(8): e0202681.
437.Cai L, Tao J, Li X, et al. Association between the full range of birth weight and childhood weight status: by gestational age. *Eur J Clin Nutr* 2019; **73**(8): 1141-8.
438.Chen J, Hu C, Zeng G, et al. Trends and Prevalence of Overweight and Obesity among Children Aged 2-7 Years from 2011 to 2017 in Xiamen, China. *Obes Facts* 2019; **12**(4): 476-88.
439.Wu L, Yao R, Zhang Y, et al. The association between digit ratio (2D:4D) and overweight or obesity among Chinese children and adolescents: A cross-sectional study. *Early Hum Dev* 2019; **136**: 14-20.
440.Zhang Y, Liu S, Li Y, Li X, Ren P, Luo F. The Relationships Between Weight Status and Physical Fitness Among Chinese Children and Youth. *Res Q Exerc Sport* 2019; **90**(2): 113-22.
441.Zhao Z, Ding N, Song S, Liu Y, Wen D. Association between depression and overweight in Chinese adolescents: a cross-sectional study. *BMJ Open* 2019; **9**(2): e024177.
442.Zou Y, Xia N, Zou Y, Chen Z, Wen Y. Smartphone addiction may be associated with adolescent hypertension: a cross-sectional study among junior school students in China. *BMC Pediatr* 2019; **19**(1): 310.
443.Duan R, Kou C, Jie J, et al. Prevalence and correlates of overweight and obesity among adolescents in
© 2024 Zhang X et al. *JAMA Pediatrics.*

northeastern China: a cross-sectional study. *BMJ Open* 2020; **10**(7): e036820.
444.Ke D, Lu D, Cai G, Zhang J, Wang X, Suzuki K. Accelerated skeletal maturation is associated with overweight and obesity as early as preschool age: a cross-sectional study. *BMC Pediatr* 2020; **20**(1): 452.
445.Liu S, Lei J, Ma J, et al. Interaction between delivery mode and maternal age in predicting overweight and obesity in 1,123 Chinese preschool children. *Ann Transl Med* 2020; **8**(7): 474.
446.Qian K, Tan L, Li S, et al. Comparison of different BMI cut-offs to screen for child and adolescent obesity in urban China. *Public Health Nutr* 2020; **23**(14): 2485-93.
447.Song Q, Yang Y, Liu X, et al. Breastfeeding Practices and Overweight/Obesity Among Children Under 5 Years of Age: A Multistage Random Sampling Survey in Central and Western China. *Matern Child Health J* 2020; **24**(8): 998-1007.
448.Sun M, Hu X, Li F, Deng J, Shi J, Lin Q. Eating Habits and Their Association with Weight Status in Chinese School-Age Children: A Cross-Sectional Study. *Int J Environ Res Public Health* 2020; **17**(10).
449.Xing X, Hu L, Guo Y, et al. Interactions between ambient air pollution and obesity on lung function in children: The Seven Northeastern Chinese Cities (SNEC) Study. *Sci Total Environ* 2020; **699**: 134397.
450.Fan Y, Li Y, Liu A, Hu X, Ma G, Xu G. Associations between body mass index, weight control concerns and behaviors, and eating disorder symptoms among non-clinical Chinese adolescents. *BMC Public Health* 2010; **10**: 314.
451.Wang X, Hojer B, Guo S, Luo S, Zhou W, Wang Y. Stunting and 'overweight' in the WHO Child Growth Standards - malnutrition among children in a poor area of China. *Public Health Nutr* 2009; **12**(11): 1991-8.
452.Xu YQ, Ji CY. Prevalence of the metabolic syndrome in secondary school adolescents in Beijing, China. *Acta Paediatr* 2008; **97**(3): 348-53.
453.Xu F, Li J, Ware RS, Owen N. Associations of television viewing time with excess body weight among urban and rural high-school students in regional mainland China. *Public Health Nutr* 2008; **11**(9): 891-6.
454.Li M, Dibley MJ, Sibbritt D, Yan H. Factors associated with adolescents' overweight and obesity at community, school and household levels in Xi'an City, China: results of hierarchical analysis. *Eur J Clin Nutr* 2008; **62**(5): 635-43.
455.Shi Z, Lien N, Nirmal Kumar B, Holmboe-Ottesen G. Perceptions of weight and associated factors of adolescents in Jiangsu Province, China. *Public Health Nutr* 2007; **10**(3): 298-305.
456.Liu JM, Ye R, Li S, et al. Prevalence of overweight/obesity in Chinese children. *Arch Med Res* 2007; **38**(8): 882-6.
457.Yan WL, Zheng YJ, Wu J, et al. Ethnic differences in body mass index and prevalence of obesity in school children of Urumqi City, Xinjiang, China. *Biomed Environ Sci* 2006; **19**(6): 469-73.
458.Li M, Dibley MJ, Sibbritt D, Yan H. An assessment of adolescent overweight and obesity in Xi'an City, China. *Int J Pediatr Obes* 2006; **1**(1): 50-8.
459.Jiang J, Rosenqvist U, Wang H, Greiner T, Ma Y, Toschke AM. Risk factors for overweight in 2- to 6-year-old children in Beijing, China. *Int J Pediatr Obes* 2006; **1**(2): 103-8.
460.Zhu L, Chen Y, Ding L, et al. Prevalence of overweight and obesity among secondary school children aged 14 to 18 years (china). *Nutr Hosp* 2015; **31**(5): 2006-10.
461.Yuan ZP, Yang M, Liang L, et al. Possible role of birth weight on general and central obesity in Chinese children and adolescents: a cross-sectional study. *Ann Epidemiol* 2015; **25**(10): 748-52.
462.Xu T, Zhu G, Liu J, Han S. Gender-specific prevalence and associated risk factors of high normal blood pressure and hypertension among multi-ethnic Chinese adolescents aged 8-18 years old. *Blood Press* 2015; **24**(3): 189-95.
463.Xiao Y, Qiao Y, Pan L, et al. Trends in the Prevalence of Overweight and Obesity among Chinese Preschool

Children from 2006 to 2014. *PLoS One* 2015; **10**(8): e0134466.
464.Wu J, Wu H, Wang J, Guo L, Deng X, Lu C. Associations between Sleep Duration and Overweight/Obesity: Results from 66,817 Chinese Adolescents. *Sci Rep* 2015; **5**: 16686.
465.Wan Y, Xu R, Feng H, et al. Is parental body weight related with their children's overweight and obesity in Gao Hang Town, Shanghai? *Asia Pac J Clin Nutr* 2015; **24**(3): 509-14.
466.Piernas C, Wang D, Du S, et al. The double burden of under- and overnutrition and nutrient adequacy among Chinese preschool and school-aged children in 2009-2011. *Eur J Clin Nutr* 2015; **69**(12): 1323-9.
467.Ma L, Zhu Y, Mai J, et al. Secular Trends in Overweight and Obesity among Urban Children in Guangzhou China, 2007-2011. *Iran J Public Health* 2015; **44**(1): 36-42.
468.Cao M, Zhu Y, He B, et al. Association between sleep duration and obesity is age- and gender-dependent in Chinese urban children aged 6-18 years: a cross-sectional study. *BMC Public Health* 2015; **15**: 1029.
469.Wang D, Qian Z, Wang J, et al. Gender-specific differences in associations of overweight and obesity with asthma and asthma-related symptoms in 30 056 children: result from 25 districts of Northeastern China. *J Asthma* 2014; **51**(5): 508-14.
470.Li M, Deng Y, Ren Y, Guo S, He X. Obesity status of middle school students in Xiangtan and its relationship with Internet addiction. *Obesity (Silver Spring)* 2014; **22**(2): 482-7.
471.He L, Ren X, Chen Y, et al. Prevalence of overweight and obesity among primary school children aged 5 to 14 years in Wannan area, China. *Nutr Hosp* 2014; **30**(4): 776-81.
472.Dong J, Guo XL, Lu ZL, et al. Prevalence of overweight and obesity and their associations with blood pressure among children and adolescents in Shandong, China. *BMC Public Health* 2014; **14**: 1080.
473.Dai YL, Fu JF, Liang L, et al. Association between obesity and sexual maturation in Chinese children: a muticenter study. *Int J Obes (Lond)* 2014; **38**(10): 1312-6.
474.Chen TJ, Modin B, Ji CY, Hjern A. Regional, socioeconomic and urban-rural disparities in child and adolescent obesity in China: a multilevel analysis. *Acta Paediatr* 2011; **100**(12): 1583-9.
475.Xu H, Hu X, Zhang Q, et al. The Association of Hypertension with Obesity and Metabolic Abnormalities among Chinese Children. *Int J Hypertens* 2011; **2011**: 987159.
476.Guo X, Zheng L, Li Y, et al. Differences in lifestyle behaviors, dietary habits, and familial factors among normal-weight, overweight, and obese Chinese children and adolescents. *Int J Behav Nutr Phys Act* 2012; **9**: 120.
477.Jia M, Wang C, Zhang Y, et al. Sugary beverage intakes and obesity prevalence among junior high school students in Beijing - a cross-sectional research on SSBs intake. *Asia Pac J Clin Nutr* 2012; **21**(3): 425-30.
478.Li Y, Raychowdhury S, Tedders SH, Lyn R, Lopez-De Fede A, Zhang J. Association between increased BMI and severe school absenteeism among US children and adolescents: findings from a national survey, 2005-2008. *Int J Obes (Lond)* 2012; **36**(4): 517-23.
479.Yi X, Yin C, Chang M, Xiao Y. Prevalence and risk factors of obesity among school-aged children in Xi'an, China. *Eur J Pediatr* 2012; **171**(2): 389-94.
480.Dong B, Ma J, Wang HJ, Wang ZQ. The association of overweight and obesity with blood pressure among Chinese children and adolescents. *Biomed Environ Sci* 2013; **26**(6): 437-44.
481.Chen J, Chen W, Zeng G, Li G. Secular Trends in Growth and Nutritional Outcomes of Children under Five Years Old in Xiamen, China. *Int J Environ Res Public Health* 2016; **13**(11).
482.Lei Y, Yang H, Zhen L. Obesity is a risk factor for allergic rhinitis in children of Wuhan (China). *Asia Pac Allergy* 2016; **6**(2): 101-4.
483.Liu W, Lin R, Liu W, et al. Relationship between weight status and health-related quality of life in Chinese primary school children in Guangzhou: a cross-sectional study. *Health Qual Life Outcomes* 2016; **14**(1): 166.
484.Liu W, Liu W, Lin R, et al. Socioeconomic determinants of childhood obesity among primary school children in
© 2024 Zhang X et al. *JAMA Pediatrics.*

Guangzhou, China. *BMC Public Health* 2016; **16**: 482.
485.Peng R, Li S, Zhang H, et al. Weight Status Is Associated with Blood Pressure, Vital Capacity, Dental Decay, and Visual Acuity among School-Age Children in Chengdu, China. *Ann Nutr Metab* 2016; **69**(3-4): 237-45.
486.Wei X, Peng R, Cao J, et al. Serum vitamin A status is associated with obesity and the metabolic syndrome among school-age children in Chongqing, China. *Asia Pac J Clin Nutr* 2016; **25**(3): 563-70.
487.Zhang P, Wu H, Zhou X, et al. The Association between Family and Parental Factors and Obesity among Children in Nanchang, China. *Front Public Health* 2016; **4**: 162.
488.Xue-Yan Z, Dong-Mei L, Dan-Dan X, Le-Shan Z. Obese Chinese Primary-School Students and Low Self-Esteem: A Cross-Sectional Study. *Iran J Pediatr* 2016; **26**(4): e3777.
489.Cai Y, Zhu X, Wu X. Overweight, obesity, and screen-time viewing among Chinese school-aged children: National prevalence estimates from the 2016 Physical Activity and Fitness in China-The Youth Study. *J Sport Health Sci* 2017; **6**(4): 404-9.
490.Liang J, Zhang Z, Yang W, et al. Association between Cesarean Section and Weight Status in Chinese Children and Adolescents: A National Survey. *Int J Environ Res Public Health* 2017; **14**(12).
491.Liu WJ, Xiong LH, Guo CS, et al. Weight status, cardiorespiratory fitness and high blood pressure relationship among 5-12-year-old Chinese primary school children. *J Hum Hypertens* 2017; **31**(12): 808-14.
492.Zhang Y, Zhang D, Li X, et al. Daily Time-Use Patterns and Obesity and Mental Health among Primary School Students in Shanghai: A Population-Based Cross-Sectional Study. *Sci Rep* 2017; **7**(1): 16200.
493.Zong Y, Xie R, Deng N, et al. Secular trends in overweight and obesity among urban children and adolescents, 2003-2012: A serial cross-sectional study in Guangzhou, China. *Sci Rep* 2017; **7**(1): 12042.
494.Chen JL, Esquivel JH, Guo J, Chesla CA, Tang S. Risk factors for obesity in preschool-aged children in China. *Int Nurs Rev* 2018; **65**(2): 217-24.
495.Dong Y, Zou Z, Yang Z, et al. Prevalence of excess body weight and underweight among 26 Chinese ethnic minority children and adolescents in 2014: a cross-sectional observational study. *BMC Public Health* 2018; **18**(1): 562.
496.Gong QH, Li SX, Li H, Cui J, Xu GZ. Insufficient Sleep Duration and Overweight/Obesity among Adolescents in a Chinese Population. *Int J Environ Res Public Health* 2018; **15**(5).
497.He B, Long W, Li X, Yang W, Chen Y, Zhu Y. Sugar-Sweetened Beverages Consumption Positively Associated with the Risks of Obesity and Hypertriglyceridemia Among Children Aged 7-18 Years in South China. *J Atheroscler Thromb* 2018; **25**(1): 81-9.
498.Li W, Liu Q, Deng X, et al. Association of prepubertal obesity with pubertal development in Chinese girls and boys: A longitudinal study. *Am J Hum Biol* 2018; **30**(6): e23195.
499.Liu Y, Ma Y, Jiang N, Song S, Fan Q, Wen D. Interaction between Parental Education and Household Wealth on Children's Obesity Risk. *Int J Environ Res Public Health* 2018; **15**(8).
500.Rutayisire E, Wu X, Huang K, et al. Dietary patterns are not associated with overweight and obesity in a sample of 8900 Chinese preschool children from four cities. *J Nutr Sci* 2018; **7**: e24.
501.Olaya Contreras P, Bastidas M, Arvidsson D. Colombian Children With Overweight and Obesity Need Additional Motivational Support at School to Perform Health-Enhancing Physical Activity. *J Phys Act Health* 2015; **12**(5): 604-9.
502.Rincon-Pabon D, Urazan-Hernandez Y, Gonzalez-Santamaria J. Association between the time spent watching television and the sociodemographic characteristics with the presence of overweight and obesity in Colombian adolescents (secondary analysis of the ENSIN 2010). *PLoS One* 2019; **14**(5): e0216455.
503.Flannagan KS, Bowman AA, Mora-Plazas M, et al. Micronutrient status and leukocyte telomere length in school-age Colombian children. *Eur J Nutr* 2020; **59**(3): 1055-65.
504.Ramirez-Velez R, Correa-Bautista JE, Villa-Gonzalez E, Martinez-Torres J, Hackney AC, Garcia-Hermoso A.
© 2024 Zhang X et al. *JAMA Pediatrics.*

Effects of preterm birth and fetal growth retardation on life-course cardiovascular risk factors among schoolchildren from Colombia: The FUPRECOL study. *Early Hum Dev* 2017; **106-107**: 53-8.
505.Martinez-Ospina A, Sudfeld CR, Gonzalez SA, Sarmiento OL. School Food Environment, Food Consumption, and Indicators of Adiposity Among Students 7-14 Years in Bogota, Colombia. *J Sch Health* 2019; **89**(3): 200-9.
506.McDonald CM, Baylin A, Arsenault JE, Mora-Plazas M, Villamor E. Overweight is more prevalent than stunting and is associated with socioeconomic status, maternal obesity, and a snacking dietary pattern in school children from Bogota, Colombia. *J Nutr* 2009; **139**(2): 370-6.
507.Gomez LF, Parra DC, Lobelo F, et al. Television viewing and its association with overweight in Colombian children: results from the 2005 National Nutrition Survey: a cross sectional study. *Int J Behav Nutr Phys Act* 2007; **4**: 41.
508.Arango CM, Parra DC, Eyler A, et al. Walking or bicycling to school and weight status among adolescents from Montería, Colombia. *J Phys Act Health* 2011; **8 Suppl 2**: S171-7.
509.Tiruneh SA, Gebremariam AD, Engidaw MT, et al. Overweight and/or obesity and its determinants among under-five children in East African countries: A multilevel analysis using Bayesian approach. *Heliyon* 2021; **7**(12): e08643.
510.Monge-Rojas R, Smith-Castro V, O'Connor TM, Vargas-Quesada R, Reyes-Fernandez B. Association between Fathers' and Mothers' Parenting Styles and the Risk of Overweight/Obesity among Adolescents in San Jose Province, Costa Rica. *Nutrients* 2022; **14**(24).
511.Gamboa-Gamboa T, Fantin R, Cordoba J, Caravaca I, Gomez-Duarte I. Relationship between childhood obesity and socio-economic status among primary school children in Costa Rica. *Public Health Nutr* 2021; **24**(12): 3825-33.
512.Núñez-Rivas HP, Monge-Rojas R, León H, Roselló M. Prevalence of overweight and obesity among Costa Rican elementary school children. *Rev Panam Salud Publica* 2003; **13**(1): 24-32.
513.Fossou AF, Ahui Bitty ML, Coulibaly TJ, Batai NF, Toure MF, Zahe K. Prevalence of obesity in children enrolled in private and public primary schools. *Clin Nutr ESPEN* 2020; **40**: 115-20.
514.Banjari I, Martinovic M, Belojevic G, et al. Poverty and other correlates of obesity and underweight among 7-year-olds from Croatia and Montenegro. *Public Health* 2020; **182**: 64-9.
515.Juresa V, Musil V, Majer M, Ivanković D, Petrović D. Behavioral pattern of overweight and obese school children. *Coll Antropol* 2012; **36 Suppl 1**: 139-46.
516.Janssen I, Katzmarzyk PT, Boyce WF, et al. Comparison of overweight and obesity prevalence in school-aged youth from 34 countries and their relationships with physical activity and dietary patterns. *Obes Rev* 2005; **6**(2): 123-32.
517.Music Milanovic S, Lang Morovic M, Bukal D, Krizan H, Buoncristiano M, Breda J. Regional and sociodemographic determinants of the prevalence of overweight and obesity in children aged 7-9 years in Croatia. *Acta Clin Croat* 2020; **59**(2): 303-11.
518.Bilić-Kirin V, Gmajnić R, Burazin J, Milicić V, Buljan V, Ivanko M. Association between socioeconomic status and obesity in children. *Coll Antropol* 2014; **38**(2): 553-8.
519.Pećin I, Samovojska R, Heinrich B, Zeljković-Vrkić T, Laganović M, Jelaković B. Hypertension, overweight and obesity in adolescents: the CRO-KOP study. *Coll Antropol* 2013; **37**(3): 761-4.
520.Savva SC, Tornaritis M, Chadjigeorgiou C, et al. Prevalence and socio-demographic associations of undernutrition and obesity among preschool children in Cyprus. *Eur J Clin Nutr* 2005; **59**(11): 1259-65.
521.Savva SC, Tornaritis MJ, Chadjigeorgiou C, Kourides YA, Siamounki M, Kafatos A. Prevalence of overweight and obesity among 11-year-old children in Cyprus, 1997-2003. *Int J Pediatr Obes* 2008; **3**(3): 186-92.
522.Savva SC, Kourides YA, Hadjigeorgiou C, Tornaritis MJ. Overweight and obesity prevalence and trends in
© 2024 Zhang X et al. *JAMA Pediatrics.*

children and adolescents in Cyprus 2000-2010. *Obes Res Clin Pract* 2014; **8**(5): e426-34.
523.Lazarou C, Panagiotakos DB, Panayiotou G, Matalas AL. Overweight and obesity in preadolescent children and their parents in Cyprus: prevalence and associated socio-demographic factors - the CYKIDS study. *Obes Rev* 2008; **9**(3): 185-93.
524.Loucaides CA, Jago R. Differences in physical activity by gender, weight status and travel mode to school in Cypriot children. *Prev Med* 2008; **47**(1): 107-11.
525.Loucaides CA, Jago R, Theophanous M. Prevalence and correlates of active traveling to school among adolescents in Cyprus. *Cent Eur J Public Health* 2010; **18**(3): 151-6.
526.Tornaritis MJ, Philippou E, Hadjigeorgiou C, Kourides YA, Panayi A, Savva SC. A study of the dietary intake of Cypriot children and adolescents aged 6-18 years and the association of mother's educational status and children's weight status on adherence to nutritional recommendations. *BMC Public Health* 2014; **14**: 13.
527.Savva SC, Tornaritis MJ, Kolokotroni O, et al. High cardiorespiratory fitness is inversely associated with incidence of overweight in adolescence: a longitudinal study. *Scand J Med Sci Sports* 2014; **24**(6): 982-9.
528.Vazna A, Vignerova J, Brabec M, et al. Influence of COVID-19-Related Restrictions on the Prevalence of Overweight and Obese Czech Children. *Int J Environ Res Public Health* 2022; **19**(19).
529.de Gouw L, Klepp KI, Vignerova J, Lien N, Steenhuis IH, Wind M. Associations between diet and (in)activity behaviours with overweight and obesity among 10-18-year-old Czech Republic adolescents. *Public Health Nutr* 2010; **13**(10A): 1701-7.
530.Brixval CS, Rayce SL, Rasmussen M, Holstein BE, Due P. Overweight, body image and bullying--an epidemiological study of 11- to 15-years olds. *Eur J Public Health* 2012; **22**(1): 126-30.
531.Ajslev TA, Andersen CS, Gamborg M, Sorensen TI, Jess T. Childhood overweight after establishment of the gut microbiota: the role of delivery mode, pre-pregnancy weight and early administration of antibiotics. *Int J Obes (Lond)* 2011; **35**(4): 522-9.
532.Mor A, Antonsen S, Kahlert J, et al. Prenatal exposure to systemic antibacterials and overweight and obesity in Danish schoolchildren: a prevalence study. *Int J Obes (Lond)* 2015; **39**(10): 1450-5.
533.Hoyer BB, Ramlau-Hansen CH, Henriksen TB, et al. Body mass index in young school-age children in relation to organochlorine compounds in early life: a prospective study. *Int J Obes (Lond)* 2014; **38**(7): 919-25.
534.Karlsen M, Grandjean P, Weihe P, Steuerwald U, Oulhote Y, Valvi D. Early-life exposures to persistent organic pollutants in relation to overweight in preschool children. *Reprod Toxicol* 2017; **68**: 145-53.
535.Krue S, Coolidge J. The prevalence of overweight and obesity among Danish school children. *Obes Rev* 2010; **11**(7): 489-91.
536.Rex KF, Larsen NH, Rex H, Niclasen B, Pedersen ML. A national study on weight classes among children in Greenland at school entry. *Int J Circumpolar Health* 2014; **73**.
537.Matthiessen J, Stockmarr A, Fagt S, Knudsen VK, Biltoft-Jensen A. Danish children born to parents with lower levels of education are more likely to become overweight. *Acta Paediatr* 2014; **103**(10): 1083-8.
538.Klakk H, Chinapaw M, Heidemann M, Andersen LB, Wedderkopp N. Effect of four additional physical education lessons on body composition in children aged 8-13 years--a prospective study during two school years. *BMC Pediatr* 2013; **13**: 170.
539.Kjelgaard HH, Holstein BE, Due P, Brixval CS, Rasmussen M. Adolescent Weight Status: Associations With Structural and Functional Dimensions of Social Relations. *J Adolesc Health* 2017; **60**(4): 460-8.
540.El Taguri A, Besmar F, Abdel Monem A, Betilmal I, Ricour C, Rolland-Cachera MF. Stunting is a major risk factor for overweight: results from national surveys in 5 Arab countries. *East Mediterr Health J* 2009; **15**(3): 549-62.
541.Andrade S, Lachat C, Ochoa-Aviles A, et al. A school-based intervention improves physical fitness in Ecuadorian adolescents: a cluster-randomized controlled trial. *Int J Behav Nutr Phys Act* 2014; **11**: 153.
© 2024 Zhang X et al. *JAMA Pediatrics.*

542.Abril V, Manuel-y-keenoy B, Solà R, et al. Prevalence of overweight and obesity among 6-to 9-year-old school children in Cuenca, Ecuador: relationship with physical activity, poverty, and eating habits. *Food Nutr Bull* 2013; **34**(4): 388-401.
543.Freire WB, Silva-Jaramillo KM, Ramirez-Luzuriaga MJ, Belmont P, Waters WF. The double burden of undernutrition and excess body weight in Ecuador. *Am J Clin Nutr* 2014; **100**(6): 1636S-43S.
544.Ortiz J, Van Camp J, Wijaya S, Donoso S, Huybregts L. Determinants of child malnutrition in rural and urban Ecuadorian highlands. *Public Health Nutr* 2014; **17**(9): 2122-30.
545.Casapulla SL, Howe CA, Mora GR, et al. Cardiometabolic risk factors, metabolic syndrome and pre-diabetes in adolescents in the Sierra region of Ecuador. *Diabetol Metab Syndr* 2017; **9**: 24.
546.Walrod J, Seccareccia E, Sarmiento I, et al. Community factors associated with stunting, overweight and food insecurity: a community-based mixed-method study in four Andean indigenous communities in Ecuador. *BMJ Open* 2018; **8**(7): e020760.
547.Abd El-Aty NS, Osman SR, Ahmed ES, GadAllah MA. Overweight and obesity prevalence among upper Egypt primary schools' children using Egyptian and CDC growth charts. *Appl Nurs Res* 2020; **56**: 151346.
548.Abdel Wahed WY, Hassan SK, Eldessouki R. Malnutrition and Its Associated Factors among Rural School Children in Fayoum Governorate, Egypt. *J Environ Public Health* 2017; **2017**: 4783791.
549.Abdelkarim O, Ammar A, Soliman AMA, Hökelmann A. Prevalence of overweight and obesity associated with the levels of physical fitness among primary school age children in Assiut city. *Egyptian Pediatric Association Gazette* 2017; **65**(2): 43-8.
550.Abou-Khadra MK, Ahmed D, Sadek SA, Mansour HH. Sleep patterns, problems, and habits in a sample of Egyptian preschoolers. *Sleep Sci* 2022; **15**(Spec 1): 164-71.
551.El-Sabely AA, Tork HMM, Hussein YE. Comparative Study of Nutritional Status and Dietary Habits of Children from Public and Private Primary Schools in Zagazig City, Egypt. *IOSR-JNHS* 2013; **3**(1): 47-52.
552.Badawi NE-S, Barakat AA, El Sherbini SA, Fawzy HM. Prevalence of overweight and obesity in primary school children in Port Said city. *Egyptian Pediatric Association Gazette* 2013; **61**(1): 31-6.
553.Hassan NE, El-Masry SA, El-Sawaf AE. Waist circumference and central fatness of Egyptian primary-school children. *East Mediterr Health J* 2008; **14**(4): 916-25.
554.Hassan NE, El-Masry SA, Farid T, Khalil A. Influence of Parental and Some Demographic Characteristics on Overweight/Obesity Status among a Sample of Egyptian Children. *Open Access Maced J Med Sci* 2016; **4**(3): 342-7.
555.Talat MA, El Shahat E. Prevalence of overweight and obesity among preparatory school adolescents in Urban Sharkia Governorate, Egypt. *Egyptian Pediatric Association Gazette* 2016; **64**(1): 20-5.
556.Perez W, Melgar P, Garces A, de Marquez AD, Merino G, Siu C. Overweight and obesity of school-age children in El Salvador according to two international systems: a population-based multilevel and spatial analysis. *BMC Public Health* 2020; **20**(1): 687.
557.Worku M, Gizaw Z, Kassahun Belew A, Wagnew A, Hunegnaw MT. Prevalence and Associated Factors of Overweight and Obesity among High School Adolescents in Bahir Dar City, Northwest, Ethiopia: A Cross-Sectional Study. *J Obes* 2021; **2021**: 8846723.
558.Wakayo T, Whiting SJ, Belachew T. Vitamin D Deficiency is Associated with Overweight and/or Obesity among Schoolchildren in Central Ethiopia: A Cross-Sectional Study. *Nutrients* 2016; **8**(4): 190.
559.Abich Y, Mihiret T, Yihunie Akalu T, Gashaw M, Janakiraman B. Flatfoot and associated factors among Ethiopian school children aged 11 to 15 years: A school-based study. *PLoS One* 2020; **15**(8): e0238001.
560.Tadesse Y, Derso T, Alene KA, Wassie MM. Prevalence and factors associated with overweight and obesity among private kindergarten school children in Bahirdar Town, Northwest Ethiopia: cross-sectional study. *BMC Res*

© 2024 Zhang X et al. *JAMA Pediatrics.*

Notes 2017; **10**(1): 22.
561.Sorrie MB, Yesuf ME, GebreMichael TG. Overweight/Obesity and associated factors among preschool children in Gondar City, Northwest Ethiopia: A cross-sectional study. *PLoS One* 2017; **12**(8): e0182511.
562.Desalew A, Mandesh A, Semahegn A. Childhood overweight, obesity and associated factors among primary school children in dire dawa, eastern Ethiopia; a cross-sectional study. *BMC Obes* 2017; **4**: 20.
563.Yetubie M, Haidar J, Kassa H, Fallon F. Socioeconomic and Demographic Factors Affecting Body Mass Index of Adolescents Students Aged 10-19 in Ambo (a Rural Town) in Ethiopia. *Int J Biomed Sci* 2010; **6**(4): 321-6.
564.Gali N, Tamiru D, Tamrat M. The Emerging Nutritional Problems of School Adolescents: Overweight/Obesity and Associated Factors in Jimma Town, Ethiopia. *J Pediatr Nurs* 2017; **35**: 98-104.
565.Yifokr T AT, Getachew Tg BM. Prevalence and Associated Factors of Overweight and/or Obesity among Primary School Children in Bole Sub-City, Addis Ababa, Ethiopia. *Journal of Nutrition & Food Sciences* 2015; **05**(05).
566.Carrillo-Larco RM, Bernabe-Ortiz A, Miranda JJ. Short sleep duration and childhood obesity: cross-sectional analysis in Peru and patterns in four developing countries. *PLoS One* 2014; **9**(11): e112433.
567.Mitiku H, Admassu D, Teklemariam Z, Weldegebreal F, Nigusse A. Nutritional status of school children in eastern Hararghe administrative zone, eastern Ethiopia. *Journal of Public Health* 2018; **27**(1): 111-8.
568.Mekonnen T, Tariku A, Abebe SM. Overweight/obesity among school aged children in Bahir Dar City: cross sectional study. *Ital J Pediatr* 2018; **44**(1): 17.
569.Pengpid S, Peltzer K. Overweight and Obesity and Associated Factors among School-Aged Adolescents in Six Pacific Island Countries in Oceania. *Int J Environ Res Public Health* 2015; **12**(11): 14505-18.
570.Petersen S, Moodie M, Mavoa H, Waqa G, Goundar R, Swinburn B. Relationship between overweight and health-related quality of life in secondary school children in Fiji: results from a cross-sectional population-based study. *Int J Obes (Lond)* 2014; **38**(4): 539-46.
571.Sarkkola C, Viljakainen J, de Oliveira Figueiredo RA, et al. Prevalence of Thinness, Overweight, Obesity, and Central Obesity in Finnish School-Aged Children: A Comparison of National and International Reference Values. *Obes Facts* 2022; **15**(2): 240-7.
572.Eloranta AM, Lindi V, Schwab U, et al. Dietary factors associated with overweight and body adiposity in Finnish children aged 6-8 years: the PANIC Study. *Int J Obes (Lond)* 2012; **36**(7): 950-5.
573.Laitinen J, Jääskeläinen A, Hartikainen AL, et al. Maternal weight gain during the first half of pregnancy and offspring obesity at 16 years: a prospective cohort study. *Bjog* 2012; **119**(6): 716-23.
574.Saari A, Virta LJ, Sankilampi U, Dunkel L, Saxen H. Antibiotic exposure in infancy and risk of being overweight in the first 24 months of life. *Pediatrics* 2015; **135**(4): 617-26.
575.Parikka S, Maki P, Levalahti E, Lehtinen-Jacks S, Martelin T, Laatikainen T. Associations between parental BMI, socioeconomic factors, family structure and overweight in Finnish children: a path model approach. *BMC Public Health* 2015; **15**: 271.
576.Vuorela N, Saha MT, Salo MK. Parents underestimate their child;s overweight. *Acta Paediatr* 2010; **99**(9): 1374-9.
577.Veltsista A, Laitinen J, Sovio U, Roma E, Jarvelin MR, Bakoula C. Relationship between eating behavior, breakfast consumption, and obesity among Finnish and Greek adolescents. *J Nutr Educ Behav* 2010; **42**(6): 417-21.
578.Vanhala M, Korpelainen R, Tapanainen P, et al. Lifestyle risk factors for obesity in 7-year-old children. *Obes Res Clin Pract* 2009; **3**(2): I-II.
579.Fogelholm M, Stigman S, Huisman T, Metsamuuronen J. Physical fitness in adolescents with normal weight and overweight. *Scand J Med Sci Sports* 2008; **18**(2): 162-70.
580.Virtanen M, Kivimaki H, Ervasti J, et al. Fast-food outlets and grocery stores near school and adolescents'
© 2024 Zhang X et al. *JAMA Pediatrics*.

eating habits and overweight in Finland. *Eur J Public Health* 2015; **25**(4): 650-5.
581.Palomaki S, Heikinaro-Johansson P, Huotari P. Cardiorespiratory performance and physical activity in normal weight and overweight Finnish adolescents from 2003 to 2010. *J Sports Sci* 2015; **33**(6): 588-96.
582.Vanhelst J, Baudelet JB, Thivel D, Ovigneur H, Deschamps T. French youth trends in prevalence of overweight, obesity and underweight from 2008 to 2018. *BMC Res Notes* 2022; **15**(1): 208.
583.Roth B, Reynaud R, Negre V, Gentile S, Pauly V, Bernard O. Prevalence of overweight, obesity, and early adiposity rebound in nursery school children in southeastern France. *Arch Pediatr* 2022; **29**(5): 388-94.
584.Vanhelst J, Deken V, Boulic G, Raffin S, Duhamel A, Romon M. Trends in prevalence of childhood overweight and obesity in a community-based programme: The VIF Programme. *Pediatr Obes* 2021; **16**(7): e12761.
585.Luiggi M, Rey O, Travert M, Griffet J. Overweight and obesity by school socioeconomic composition and adolescent socioeconomic status: a school-based study. *BMC Public Health* 2021; **21**(1): 1837.
586.Dupuy M, Godeau E, Vignes C, Ahluwalia N. Socio-demographic and lifestyle factors associated with overweight in a representative sample of 11-15 year olds in France: results from the WHO-Collaborative Health Behaviour in School-aged Children (HBSC) cross-sectional study. *BMC Public Health* 2011; **11**: 442.
587.Thibault H, Carriere C, Langevin C, Kossi Deti E, Barberger-Gateau P, Maurice S. Prevalence and factors associated with overweight and obesity in French primary-school children. *Public Health Nutr* 2013; **16**(2): 193-201.
588.Thibault H, Contrand B, Saubusse E, Baine M, Maurice-Tison S. Risk factors for overweight and obesity in French adolescents: physical activity, sedentary behavior and parental characteristics. *Nutrition* 2010; **26**(2): 192-200.
589.Carriere C, Langevin C, Deti EK, Barberger-Gateau P, Maurice S, Thibault H. Stabilization of overweight prevalence and improvement of dietary habits in French children between 2004 and 2008. *Public Health Nutr* 2015; **18**(10): 1883-9.
590.Salanave B, Peneau S, Rolland-Cachera MF, Hercberg S, Castetbon K. Stabilization of overweight prevalence in French children between 2000 and 2007. *Int J Pediatr Obes* 2009; **4**(2): 66-72.
591.Heude B, Lafay L, Borys JM, et al. Time trend in height, weight, and obesity prevalence in school children from Northern France, 1992-2000. *Diabetes Metab* 2003; **29**(3): 235-40.
592.Legleye S, Beck F, Spilka S, Chau N. Correction of body-mass index using body-shape perception and socioeconomic status in adolescent self-report surveys. *PLoS One* 2014; **9**(5): e96768.
593.Chau N, Chau K, Mayet A, Baumann M, Legleye S, Falissard B. Self-reporting and measurement of body mass index in adolescents: refusals and validity, and the possible role of socioeconomic and health-related factors. *BMC Public Health* 2013; **13**: 815.
594.Jouret B, Ahluwalia N, Cristini C, et al. Factors associated with overweight in preschool-age children in southwestern France. *Am J Clin Nutr* 2007; **85**(6): 1643-9.
595.Pitrou I, Shojaei T, Wazana A, Gilbert F, Kovess-Masfety V. Child overweight, associated psychopathology, and social functioning: a French school-based survey in 6- to 11-year-old children. *Obesity (Silver Spring)* 2010; **18**(4): 809-17.
596.Ruiz M, Goldblatt P, Morrison J, et al. Impact of Low Maternal Education on Early Childhood Overweight and Obesity in Europe. *Paediatr Perinat Epidemiol* 2016; **30**(3): 274-84.
597.Klein-Platat C, Wagner A, Haan MC, Arveiler D, Schlienger JL, Simon C. Prevalence and sociodemographic determinants of overweight in young French adolescents. *Diabetes Metab Res Rev* 2003; **19**(2): 153-8.
598.Rolland-Cachera MF, Castetbon K, Arnault N, et al. Body mass index in 7-9-y-old French children: frequency of obesity, overweight and thinness. *Int J Obes Relat Metab Disord* 2002; **26**(12): 1610-6.
599.Tubert-Jeannin S, Pichot H, Rouchon B, Pereira B, Hennequin M. Common risk indicators for oral diseases and obesity in 12-year-olds: a South Pacific cross sectional study. *BMC Public Health* 2018; **18**(1): 112.
© 2024 Zhang X et al. *JAMA Pediatrics.*

600.Keke LM, Samouda H, Jacobs J, et al. Body mass index and childhood obesity classification systems: A comparison of the French, International Obesity Task Force (IOTF) and World Health Organization (WHO) references. *Rev Epidemiol Sante Publique* 2015; **63**(3): 173-82.
601.Vanhelst J, Baudelet JB, Fardy PS, Beghin L, Mikulovic J, Ulmer Z. Prevalence of overweight, obesity, underweight and normal weight in French youth from 2009 to 2013. *Public Health Nutr* 2017; **20**(6): 959-64.
602.Wang M, Flexeder C, Kilanowski A, et al. Changes in sleep duration and sleep difficulties from adolescence to young adulthood and the risk of obesity: Bidirectional evidence in the GINIplus and LISA studies. *Sleep Med* 2023; **101**: 401-10.
603.Hoebel J, Waldhauer J, Blume M, Schienkiewitz A. Socioeconomic Status, Overweight, and Obesity in Childhood and Adolescence-Secular Trends From the Nationwide German Kiggs Study. *Dtsch Arztebl Int* 2022; **119**(49): 839-45.
604.Zhou Y, von Lengerke T, Dreier M. Comparing different data sources by examining the associations between surrounding greenspace and children's weight status. *Int J Health Geogr* 2021; **20**(1): 24.
605.Sergentanis TN, Bampalitsa SD, Theofilou P, et al. Cyberbullying and Obesity in Adolescents: Prevalence and Associations in Seven European Countries of the EU NET ADB Survey. *Children (Basel)* 2021; **8**(3).
606.Nguyen TH, Gotz S, Kreffter K, Lisak-Wahl S, Dragano N, Weyers S. Neighbourhood deprivation and obesity among 5656 pre-school children-findings from mandatory school enrollment examinations. *Eur J Pediatr* 2021; **180**(6): 1947-54.
607.Liu W, Schwertz R, Welker A, et al. Associations between BMI and visual impairment of 33 407 preschool children in Germany: a pooled cross-sectional study. *Eur J Public Health* 2021; **31**(1): 105-11.
608.Willerhausen B, Blettner M, Kasaj A, Hohenfellner K. Association between body mass index and dental health in 1,290 children of elementary schools in a German city. *Clin Oral Investig* 2007; **11**(3): 195-200.
609.Kesztyüs D, Wirt T, Kobel S, et al. Is central obesity associated with poorer health and health-related quality of life in primary school children? Cross-sectional results from the Baden-Württemberg Study. *BMC Public Health* 2013; **13**: 260.
610.Nagel G, Wabitsch M, Galm C, et al. Determinants of obesity in the Ulm Research on Metabolism, Exercise and Lifestyle in Children (URMEL-ICE). *Eur J Pediatr* 2009; **168**(10): 1259-67.
611.Boneberger A, von Kries R, Milde-Busch A, et al. Association between peer relationship problems and childhood overweight/obesity. *Acta Paediatr* 2009; **98**(12): 1950-5.
612.Kromeyer-Hauschild K, Zellner K. Trends in overweight and obesity and changes in the distribution of body mass index in schoolchildren of Jena, East Germany. *Eur J Clin Nutr* 2007; **61**(3): 404-11.
613.Landsberg B, Plachta-Danielzik S, Lange D, Johannsen M, Seiberl J, Muller MJ. Clustering of lifestyle factors and association with overweight in adolescents of the Kiel Obesity Prevention Study. *Public Health Nutr* 2010; **13**(10A): 1708-15.
614.Toschke AM, Küchenhoff H, Koletzko B, von Kries R. Meal frequency and childhood obesity. *Obes Res* 2005; **13**(11): 1932-8.
615.Toschke AM, Montgomery SM, Pfeiffer U, von Kries R. Early intrauterine exposure to tobacco-inhaled products and obesity. *Am J Epidemiol* 2003; **158**(11): 1068-74.
616.von Kries R, Bolte G, Baghi L, Toschke AM, Group GMES. Parental smoking and childhood obesity--is maternal smoking in pregnancy the critical exposure? *Int J Epidemiol* 2008; **37**(1): 210-6.
617.Raum E, Kupper-Nybelen J, Lamerz A, Hebebrand J, Herpertz-Dahlmann B, Brenner H. Tobacco smoke exposure before, during, and after pregnancy and risk of overweight at age 6. *Obesity (Silver Spring)* 2011; **19**(12): 2411-7.
618.Pei Z, Flexeder C, Fuertes E, et al. Early life risk factors of being overweight at 10 years of age: results of the
© 2024 Zhang X et al. *JAMA Pediatrics.*

German birth cohorts GINIplus and LISAplus. *Eur J Clin Nutr* 2013; **67**(8): 855-62.
619.Kleiser C, Schaffrath Rosario A, Mensink GB, Prinz-Langenohl R, Kurth BM. Potential determinants of obesity among children and adolescents in Germany: results from the cross-sectional KiGGS Study. *BMC Public Health* 2009; **9**: 46.
620.Toschke AM, Ruckinger S, Bohler E, Von Kries R. Adjusted population attributable fractions and preventable potential of risk factors for childhood obesity. *Public Health Nutr* 2007; **10**(9): 902-6.
621.Weyermann M, Rothenbacher D, Brenner H. Duration of breastfeeding and risk of overweight in childhood: a prospective birth cohort study from Germany. *Int J Obes (Lond)* 2006; **30**(8): 1281-7.
622.Hoffmann B, Kobel S, Wartha O, Kettner S, Dreyhaupt J, Steinacker JM. High sedentary time in children is not only due to screen media use: a cross-sectional study. *BMC Pediatr* 2019; **19**(1): 154.
623.Suchert V, Hanewinkel R, Isensee B. Screen time, weight status and the self-concept of physical attractiveness in adolescents. *J Adolesc* 2016; **48**: 11-7.
624.Will B, Zeeb H, Baune BT. Overweight and obesity at school entry among migrant and German children: a cross-sectional study. *BMC Public Health* 2005; **5**: 45.
625.Rapp K, Schick KH, Bode H, Weiland SK. Type of kindergarten and other potential determinants of overweight in pre-school children. *Public Health Nutr* 2005; **8**(6): 642-9.
626.Lamerz A, Kuepper-Nybelen J, Wehle C, et al. Social class, parental education, and obesity prevalence in a study of six-year-old children in Germany. *Int J Obes (Lond)* 2005; **29**(4): 373-80.
627.Graf C, Koch B, Kretschmann-Kandel E, et al. Correlation between BMI, leisure habits and motor abilities in childhood (CHILT-project). *Int J Obes Relat Metab Disord* 2004; **28**(1): 22-6.
628.Graf C, Koch B, Dordel S, et al. Physical activity, leisure habits and obesity in first-grade children. *Eur J Cardiovasc Prev Rehabil* 2004; **11**(4): 284-90.
629.Reich A, Muller G, Gelbrich G, Deutscher K, Godicke R, Kiess W. Obesity and blood pressure--results from the examination of 2365 schoolchildren in Germany. *Int J Obes Relat Metab Disord* 2003; **27**(12): 1459-64.
630.Wurbach A, Zellner K, Kromeyer-Hauschild K. Meal patterns among children and adolescents and their associations with weight status and parental characteristics. *Public Health Nutr* 2009; **12**(8): 1115-21.
631.Warschburger P, Kroller K. Maternal perception of weight status and health risks associated with obesity in children. *Pediatrics* 2009; **124**(1): e60-8.
632.Toschke AM, Thorsteinsdottir KH, von Kries R, Group GMES. Meal frequency, breakfast consumption and childhood obesity. *Int J Pediatr Obes* 2009; **4**(4): 242-8.
633.Sporisević L, Krzelj V, Bajraktarević A, Jahić E. Evaluation of cardiovascular risk in school children. *Bosn J Basic Med Sci* 2009; **9**(3): 182-6.
634.Koller D, Mielck A. Regional and social differences concerning overweight, participation in health check-ups and vaccination. Analysis of data from a whole birth cohort of 6-year old children in a prosperous German city. *BMC Public Health* 2009; **9**: 43.
635.De Toia D, Klein D, Weber S, et al. Relationship between anthropometry and motor abilities at pre-school age. *Obes Facts* 2009; **2**(4): 221-5.
636.Bayer O, Bolte G, Morlock G, Ruckinger S, von Kries R, Group GM-S. A simple assessment of physical activity is associated with obesity and motor fitness in pre-school children. *Public Health Nutr* 2009; **12**(8): 1242-7.
637.Bau AM, Ernert A, Schenk L, et al. Is there a further acceleration in the age at onset of menarche? A cross-sectional study in 1840 school children focusing on age and bodyweight at the onset of menarche. *Eur J Endocrinol* 2009; **160**(1): 107-13.
638.Nagel G, Rapp K, Wabitsch M, et al. Prevalence and cluster of cardiometabolic biomarkers in overweight and obese schoolchildren: results from a large survey in southwest Germany. *Clin Chem* 2008; **54**(2): 317-25.
© 2024 Zhang X et al. *JAMA Pediatrics.*

639.Mauch M, Grau S, Krauss I, Maiwald C, Horstmann T. Foot morphology of normal, underweight and overweight children. *Int J Obes (Lond)* 2008; **32**(7): 1068-75.
640.Jahnke DL, Warschburger PA. Familial transmission of eating behaviors in preschool-aged children. *Obesity (Silver Spring)* 2008; **16**(8): 1821-5.
641.Herpertz-Dahlmann B, Wille N, Holling H, Vloet TD, Ravens-Sieberer U, group Bs. Disordered eating behaviour and attitudes, associated psychopathology and health-related quality of life: results of the BELLA study. *Eur Child Adolesc Psychiatry* 2008; **17 Suppl 1**: 82-91.
642.Willershausen B, Moschos D, Azrak B, Blettner M. Correlation between oral health and body mass index (BMI) in 2071 primary school pupils. *Eur J Med Res* 2007; **12**(7): 295-9.
643.Kobel S, Kettner S, Erkelenz N, Kesztyüs D, Steinacker JM. Does a higher incidence of break times in primary schools result in children being more physically active? *J Sch Health* 2015; **85**(3): 149-54.
644.Brettschneider AK, Schaffrath Rosario A, Kuhnert R, et al. Updated prevalence rates of overweight and obesity in 11- to 17-year-old adolescents in Germany. Results from the telephone-based KiGGS Wave 1 after correction for bias in self-reports. *BMC Public Health* 2015; **15**: 1101.
645.Akoto S, Tandoh MA, Nsiah K, Asamoah-Boakye O, Annaful VT. Lifestyle habits, macronutrient intake, and obesity prevalence among adolescents in rural-periurban community senior high schools in the Ho municipality of Ghana. *Front Nutr* 2022; **9**: 955898.
646.Agaba M, Azupogo F, Brouwer ID. Maternal nutritional status, decision-making autonomy and the nutritional status of adolescent girls: a cross-sectional analysis in the Mion District of Ghana. *J Nutr Sci* 2022; **11**: e97.
647.Aboagye RG, Kugbey N, Ahinkorah BO, et al. Nutritional status of school children in the South Tongu District, Ghana. *PLoS One* 2022; **17**(8): e0269718.
648.Adom T, De Villiers A, Puoane T, Kengne AP. Prevalence and correlates of overweight and obesity among school children in an urban district in Ghana. *BMC Obes* 2019; **6**: 14.
649.Amidu N, Owiredu WKBA, Saaka M, et al. Determinants of childhood obesity among basic school children aged 6 – 12 years in Tamale Metropolis. *Journal of Medical and Biomedical Sciences* 2013; **2**(3): 26-34.
650.Mohammed H, Vuvor F. Prevalence of childhood overweight/obesity in basic school in Accra. *Ghana Med J* 2012; **46**(3): 124-7.
651.Intiful FD, Ogyiri L, Asante M, Mensah AA, Steele-Dadzie RK, Boateng L. Nutritional Status of Boarding and Non-Boarding Children in Selected Schools in the Accra Metropolis. *Journal of Biology, Agriculture and Healthcare* 2013; **3**(7): 156-62.
652.Obirikorang C, Anto E, Ngala R, Gyamfi E. The prevalence OF childhood obesity and lifestyle-associated risk factors using anthropometric measurements among primary schools IN the kumasi metropolis, Ghana. *Innovative Journal of Medical and Health Science* 2015; **4**.
653.Aryeetey R, Lartey A, Marquis GS, Nti H, Colecraft E, Brown P. Prevalence and predictors of overweight and obesity among school-aged children in urban Ghana. *BMC Obes* 2017; **4**: 38.
654.Atsu BK, Guure C, Laar AK. Determinants of overweight with concurrent stunting among Ghanaian children. *BMC Pediatr* 2017; **17**(1): 177.
655.J A-A, M A, Agb A. Obesity and its Correlates among Junior High School Children in the Accra Metropolis. *Journal of Nutrition and Health Sciences* 2017; **4**(2).
656.Kwabla MP, Gyan C, Zotor F. Nutritional status of in-school children and its associated factors in Denkyembour District, eastern region, Ghana: comparing schools with feeding and non-school feeding policies. *Nutr J* 2018; **17**(1): 8.
657.Gyamfi D, Obirikorang C, Acheampong E, et al. Weight management among school-aged children and adolescents: a quantitative assessment in a Ghanaian municipality. *BMC Pediatr* 2019; **19**(1): 376.
© 2024 Zhang X et al. *JAMA Pediatrics.*

658.Ganle JK, Boakye PP, Baatiema L. Childhood obesity in urban Ghana: evidence from a cross-sectional survey of in-school children aged 5-16 years. *BMC Public Health* 2019; **19**(1): 1561.
659.Mogre V, Aneyire E, Gyamfi E. Physical activity and BMI status of school-age children in Tamale, Northern Ghana. *Pakistan Journal of Nutrition* 2013; **12**(5): 484.
660.Makri R, Katsoulis M, Fotiou A, et al. Prevalence of Overweight and Obesity and Associated Diet-Related Behaviours and Habits in a Representative Sample of Adolescents in Greece. *Children (Basel)* 2022; **9**(1).
661.Kostopoulou E, Tsekoura E, Fouzas S, Gkentzi D, Jelastopulu E, Varvarigou A. Association of lifestyle factors with a high prevalence of overweight and obesity in Greek children aged 10-16 years. *Acta Paediatr* 2021; **110**(12): 3356-64.
662.Pikramenou V, Dimitraki D, Zoumpoulakis M, Verykouki E, Kotsanos N. Association between dental caries and body mass in preschool children. *Eur Arch Paediatr Dent* 2016; **17**(3): 171-5.
663.Trikaliotis A, Boka V, Kotsanos N, Karagiannis V, Hassapidou M. Short communication: Dmfs and BMI in preschool Greek children. An epidemiological study. *Eur Arch Paediatr Dent* 2011; **12**(3): 176-8.
664.Grigorakis DA, Georgoulis M, Psarra G, Tambalis KD, Panagiotakos DB, Sidossis LS. Prevalence and lifestyle determinants of central obesity in children. *Eur J Nutr* 2016; **55**(5): 1923-31.
665.Kleanthous K, Dermitzaki E, Papadimitriou DT, Papaevangelou V, Papadimitriou A. Overweight and obesity decreased in Greek schoolchildren from 2009 to 2012 during the early phase of the economic crisis. *Acta Paediatr* 2016; **105**(2): 200-5.
666.Manios Y, Moschonis G, Chrousos GP, et al. The double burden of obesity and iron deficiency on children and adolescents in Greece: the Healthy Growth Study. *J Hum Nutr Diet* 2013; **26**(5): 470-8.
667.Manios Y, Angelopoulos PD, Kourlaba G, et al. Prevalence of obesity and body mass index correlates in a representative sample of Cretan school children. *Int J Pediatr Obes* 2011; **6**(2): 135-41.
668.Tokmakidis SP, Kasambalis A, Christodoulos AD. Fitness levels of Greek primary schoolchildren in relationship to overweight and obesity. *Eur J Pediatr* 2006; **165**(12): 867-74.
669.Risvas G, Papaioannou I, Panagiotakos DB, Farajian P, Bountziouka V, Zampelas A. Perinatal and family factors associated with preadolescence overweight/obesity in Greece: the GRECO study. *J Epidemiol Glob Health* 2012; **2**(3): 145-53.
670.Daraki V, Georgiou V, Papavasiliou S, et al. Metabolic profile in early pregnancy is associated with offspring adiposity at 4 years of age: the Rhea pregnancy cohort Crete, Greece. *PLoS One* 2015; **10**(5): e0126327.
671.Lamprokostopoulou A, Moschonis G, Manios Y, et al. Childhood obesity and leucocyte telomere length. *Eur J Clin Invest* 2019; **49**(12): e13178.
672.Kontogianni MD, Farmaki AE, Vidra N, Sofrona S, Magkanari F, Yannakoulia M. Associations between lifestyle patterns and body mass index in a sample of Greek children and adolescents. *J Am Diet Assoc* 2010; **110**(2): 215-21.
673.Kosti RI, Panagiotakos DB, Mihas CC, et al. Dietary habits, physical activity and prevalence of overweight/obesity among adolescents in Greece: the Vyronas study. *Med Sci Monit* 2007; **13**(10): Cr437-44.
674.Lagiou A, Parava M. Correlates of childhood obesity in Athens, Greece. *Public Health Nutr* 2008; **11**(9): 940-5.
675.Cassimos D, Sidiropoulos H, Batzios S, Balodima V, Christoforidis A. Sociodemographic and dietary risk factors for excess weight in a greek pediatric population living in Kavala, Northern Greece. *Nutr Clin Pract* 2011; **26**(2): 186-91.
676.Krassas GE, Tsametis C, Baleki V, et al. Prevalence of overweight and obesity among children and adolescents in Thessaloniki-Greece and Kayseri-Turkey. *Pediatr Endocrinol Rev* 2004; **1 Suppl 3**: 460-4.
677.Angelopoulos PD, Milionis HJ, Moschonis G, Manios Y. Relations between obesity and hypertension: preliminary data from a cross-sectional study in primary schoolchildren: the children study. *Eur J Clin Nutr* 2006;

60(10): 1226-34.
678.Magkos F, Manios Y, Christakis G, Kafatos AG. Age-dependent changes in body size of Greek boys from 1982 to 2002. *Obesity (Silver Spring)* 2006; **14**(2): 289-94.
679.Magkos F, Piperkou I, Manios Y, et al. Diet, blood lipid profile and physical activity patterns in primary school children from a semi-rural area of Greece. *J Hum Nutr Diet* 2006; **19**(2): 101-12, quiz 13-6.
680.Papadimitriou A, Kounadi D, Konstantinidou M, Xepapadaki P, Nicolaidou P. Prevalence of obesity in elementary schoolchildren living in Northeast Attica, Greece. *Obesity (Silver Spring)* 2006; **14**(7): 1113-7.
681.Manios Y, Costarelli V, Kolotourou M, Kondakis K, Tzavara C, Moschonis G. Prevalence of obesity in preschool Greek children, in relation to parental characteristics and region of residence. *BMC Public Health* 2007; **7**: 178.
682.Kamtsios S, Digelidis N. Physical activity levels, exercise attitudes, self-perceptions and BMI type of 11 to 12-year-old children. *J Child Health Care* 2008; **12**(3): 232-40.
683.Linardakis M, Sarri K, Pateraki MS, Sbokos M, Kafatos A. Sugar-added beverages consumption among kindergarten children of Crete: effects on nutritional status and risk of obesity. *BMC Public Health* 2008; **8**: 279.
684.Hassapidou M, Papadopoulou SK, Frossinis A, Kaklamanos I, Tzotzas T. Sociodemographic, ethnic and dietary factors associated with childhood obesity in Thessaloniki, Northern Greece. *Hormones (Athens)* 2009; **8**(1): 53-9.
685.Kollias AK, Skliros EA, Leotsakos N, Gikas A, Garifallos D. Childhood obesity in relation to parental weight status in Greece. *Hippokratia* 2009; **13**(4): 253.
686.Koroni M, Garagouni-Areou F, Roussi-Vergou CJ, Zafiropoulou M, Piperakis SM. The stigmatization of obesity in children. A survey in Greek elementary schools. *Appetite* 2009; **52**(1): 241-4.
687.Mavrakanas TA, Konsoula G, Patsonis I, Merkouris BP. Childhood obesity and elevated blood pressure in a rural population of northern Greece. *Rural Remote Health* 2009; **9**(2): 1150.
688.Yannakoulia M, Ntalla I, Papoutsakis C, Farmaki AE, Dedoussis GV. Consumption of vegetables, cooked meals, and eating dinner is negatively associated with overweight status in children. *J Pediatr* 2010; **157**(5): 815-20.
689.Hassapidou M, Daskalou E, Tsofliou F, et al. Prevalence of overweight and obesity in preschool children in Thessaloniki, Greece. *Hormones (Athens)* 2015; **14**(4): 615-22.
690.Spathopoulos D, Paraskakis E, Trypsianis G, et al. The effect of obesity on pulmonary lung function of school aged children in Greece. *Pediatr Pulmonol* 2009; **44**(3): 273-80.
691.Antonogeorgos G, Panagiotakos DB, Papadimitriou A, Priftis KN, Anthracopoulos M, Nicolaidou P. Breakfast consumption and meal frequency interaction with childhood obesity. *Pediatr Obes* 2012; **7**(1): 65-72.
692.Nassis GP, Psarra G, Sidossis LS. Central and total adiposity are lower in overweight and obese children with high cardiorespiratory fitness. *Eur J Clin Nutr* 2005; **59**(1): 137-41.
693.Manios Y, Yiannakouris N, Papoutsakis C, et al. Behavioral and physiological indices related to BMI in a cohort of primary schoolchildren in Greece. *Am J Hum Biol* 2004; **16**(6): 639-47.
694.Poulimeneas D, Vlachos D, Maraki MI, et al. Diet quality, overweight and daily monetary allowance of Greek adolescents. *Int J Adolesc Med Health* 2017; **31**(3).
695.Vazquez CE, Cubbin C. Associations between breastfeeding duration and overweight/obese among children aged 5-10: a focus on racial/ethnic disparities in California. *AIMS Public Health* 2019; **6**(4): 355-69.
696.Katsagoni CN, Psarra G, Georgoulis M, et al. High and moderate adherence to Mediterranean lifestyle is inversely associated with overweight, general and abdominal obesity in children and adolescents: The MediLIFE-index. *Nutr Res* 2020; **73**: 38-47.
697.Notara V, Legkou M, Kanellopoulou A, et al. Lack of association between dietary fibres intake and childhood obesity: an epidemiological study among preadolescents in Greece. *Int J Food Sci Nutr* 2020; **71**(5): 635-43.
698.Mirkopoulou D, Grammatikopoulou M, Gerothanasi K, Tagka A, Stylianou C, Hassapidou M. Metabolic indices, energy and macronutrient intake according to weight status in a rural sample of 17-year-old adolescents. *Rural and*
© 2024 Zhang X et al. *JAMA Pediatrics.*

Remote Health 2010.
699.Lazarou C, Soteriades ES. Children's physical activity, TV watching and obesity in Cyprus: the CYKIDS study. *Eur J Public Health* 2010; **20**(1): 70-7.
700.Antonogeorgos G, Papadimitriou A, Panagiotakos DB, Priftis KN, Nikolaidou P. Physical activity patterns and obesity status among 10- to 12-year-old adolescents living in Athens, Greece. *J Phys Act Health* 2010; **7**(5): 633-40.
701.Karatzi K, Protogerou A, Rarra V, Stergiou GS. Home and office blood pressure in children and adolescents: the role of obesity. The Arsakeion School Study. *J Hum Hypertens* 2009; **23**(8): 512-20.
702.Yannakoulia M, Papanikolaou K, Hatzopoulou I, Efstathiou E, Papoutsakis C, Dedoussis GV. Association between family divorce and children's BMI and meal patterns: the GENDAI Study. *Obesity (Silver Spring)* 2008; **16**(6): 1382-7.
703.Hassapidou M, Fotiadou E, Maglara E, Papadopoulou SK. Energy intake, diet composition, energy expenditure, and body fatness of adolescents in northern Greece. *Obesity (Silver Spring)* 2006; **14**(5): 855-62.
704.Vafeiadi M, Georgiou V, Chalkiadaki G, et al. Association of Prenatal Exposure to Persistent Organic Pollutants with Obesity and Cardiometabolic Traits in Early Childhood: The Rhea Mother-Child Cohort (Crete, Greece). *Environ Health Perspect* 2015; **123**(10): 1015-21.
705.Sourani M. Cross-Sectional Study on Childhood Obesity and Central Obesity on a Rural Greek Island. *Acta Endocrinologica (Bucharest)* 2015; **11**(3): 329-36.
706.Patsopoulou A, Tsimtsiou Z, Katsioulis A, Rachiotis G, Malissiova E, Hadjichristodoulou C. Prevalence and Risk Factors of Overweight and Obesity among Adolescents and Their Parents in Central Greece (FETA Project). *Int J Environ Res Public Health* 2015; **13**(1): 83.
707.Kambas A, Venetsanou F, Avloniti A, et al. Pedometer determined physical activity and obesity prevalence of Greek children aged 4-6 years. *Ann Hum Biol* 2015; **42**(3): 231-6.
708.Grammatikopoulou MG, Poulimeneas D, Gounitsioti IS, et al. Prevalence of simple and abdominal obesity in Greek adolescents: the ADONUT study. *Clin Obes* 2014; **4**(6): 303-8.
709.Antonogeorgos G, Papadimitriou A, Panagiotakos DB, Priftis KN, Nicolaidou P. Association of extracurricular sports participation with obesity in Greek children. *J Sports Med Phys Fitness* 2011; **51**(1): 121-7.
710.Athanasopoulos DT, Garopoulou AI, Dragoumanos VP. Childhood obesity and associated factors in a rural Greek island. *Rural Remote Health* 2011; **11**(4): 1641.
711.Farajian P, Risvas G, Karasouli K, et al. Very high childhood obesity prevalence and low adherence rates to the Mediterranean diet in Greek children: the GRECO study. *Atherosclerosis* 2011; **217**(2): 525-30.
712.Michalopoulou M, Gourgoulis V, Kourtessis T, Kambas A, Dimitrou M, Gretziou H. Step counts and body mass index among 9-14 years old greek schoolchildren. *J Sports Sci Med* 2011; **10**(1): 215-21.
713.Jelastopulu E, Kallianezos P, Merekoulias G, Alexopoulos EC, Sapountzi-Krepia D. Prevalence and risk factors of excess weight in school children in West Greece. *Nurs Health Sci* 2012; **14**(3): 372-80.
714.Kyriazis I, Rekleiti M, Saridi M, et al. Prevalence of obesity in children aged 6-12 years in Greece: nutritional behaviour and physical activity. *Arch Med Sci* 2012; **8**(5): 859-64.
715.Tambalis KD, Panagiotakos DB, Kavouras SA, Papoutsakis S, Sidossis LS. Higher prevalence of obesity in Greek children living in rural areas despite increased levels of physical activity. *J Paediatr Child Health* 2013; **49**(9): 769-74.
716.Poulimeneas D, Grammatikopoulou MG, Dimitrakopoulos L, et al. Regional differences in the prevalence of underweight, overweight and obesity among 13-year-old adolescents in Greece. *Int J Pediatr Adolesc Med* 2016; **3**(4): 153-61.
717.Garoufi A, Grammatikos EE, Kollias A, Grammatikos E, Stergiou GS, Soldatou A. Associations between obesity, adverse behavioral patterns and cardiovascular risk factors among adolescent inhabitants of a Greek island. *J*
© 2024 Zhang X et al. *JAMA Pediatrics.*

Pediatr Endocrinol Metab 2017; **30**(4): 445-54.
718.Koulouvaris P, Tsolakis C, Tsekouras YE, Donti O, Papagelopoulos PJ. Obesity and physical fitness indices of children aged 5-12 years living on remote and isolated islands. *Rural Remote Health* 2018; **18**(2): 4425.
719.Manios Y, Karatzi K, Protogerou AD, et al. Prevalence of childhood hypertension and hypertension phenotypes by weight status and waist circumference: the Healthy Growth Study. *Eur J Nutr* 2018; **57**(3): 1147-55.
720.Alvarado VJ, Mayorga E, Molina S, Solomons NW. Nutritional status of an economically-privileged convenience sample of urban children in Guatemala City. *Int J Food Sci Nutr* 2009; **60 Suppl 5**: 181-91.
721.Mejia Torres RE, Franco Garcia DN, Fontecha Sandoval GA, et al. Prevalence and intensity of soil-transmitted helminthiasis, prevalence of malaria and nutritional status of school going children in honduras. *PLoS Negl Trop Dis* 2014; **8**(10): e3248.
722.Tung JY, Ho FK, Tung KT, et al. Does obesity persist from childhood to adolescence? A 4-year prospective cohort study of chinese students in Hong Kong. *BMC Pediatr* 2021; **21**(1): 60.
723.Huang WY, Wong SHS. Prospective Associations between Weekend Catch-Up Sleep, Physical Activity, and Childhood Obesity. *Child Obes* 2019; **15**(1): 40-7.
724.Wing YK, Li SX, Li AM, Zhang J, Kong AP. The effect of weekend and holiday sleep compensation on childhood overweight and obesity. *Pediatrics* 2009; **124**(5): e994-e1000.
725.Wang J, Li AM, Lam H, Leung GM, Schooling CM. Sleep Duration and Adiposity in Children and Adults: Observational and Mendelian Randomization Studies. *Obesity (Silver Spring)* 2019; **27**(6): 1013-22.
726.Chan NP, Choi KC, Nelson EA, Sung RY, Chan JC, Kong AP. Self-reported body weight and height: an assessment tool for identifying children with overweight/obesity status and cardiometabolic risk factors clustering. *Matern Child Health J* 2013; **17**(2): 282-91.
727.Wang JJ, Gao Y, Lau PWC. Prevalence of overweight in Hong Kong Chinese children: Its associations with family, early-life development and behaviors-related factors. *J Exerc Sci Fit* 2017; **15**(2): 89-95.
728.Wong JPS, Ho SY, Lai MK, Leung GM, Stewart SM, Lam TH. Overweight, obesity, weight-related concerns and behaviours in Hong Kong Chinese children and adolescents. *Acta Paediatrica* 2005; **94**(5): 595-601.
729.Leung TF, Kong AP, Chan IH, et al. Association between obesity and atopy in Chinese schoolchildren. *Int Arch Allergy Immunol* 2009; **149**(2): 133-40.
730.Ko GT, Ozaki R, Wong GW, et al. The problem of obesity among adolescents in Hong Kong: a comparison using various diagnostic criteria. *BMC Pediatr* 2008; **8**: 10.
731.Cheung PC, Ip PL, Lam ST, Bibby H. A study on body weight perception and weight control behaviours among adolescents in Hong Kong. *Hong Kong Med J* 2007; **13**(1): 16-21.
732.Knowles G, Ling FC, Thomas GN, Adab P, McManus AM. Body size dissatisfaction among young Chinese children in Hong Kong: a cross-sectional study. *Public Health Nutr* 2015; **18**(6): 1067-74.
733.Chan R, Chan D, Lau W, Lo D, Li L, Woo J. A cross-sectional study to examine the association between dietary patterns and risk of overweight and obesity in Hong Kong Chinese adolescents aged 10-12 years. *J Am Coll Nutr* 2014; **33**(6): 450-8.
734.Ip P, Ho FK, Louie LH, et al. Childhood Obesity and Physical Activity-Friendly School Environments. *J Pediatr* 2017; **191**: 110-6.
735.Lee RLT, Lee PH, Sze DMY, Chien WT. Anthropometric profile of Hong Kong children and adolescents: the Wellness Population of Youth Study. *J Am Soc Hypertens* 2017; **11**(4): 196-203 e4.
736.Barath A, Boda K, Tichy M, Karoly E, Turi S. International comparison of blood pressure and BMI values in schoolchildren aged 11-16 years. *Acta Paediatr* 2010; **99**(2): 251-5.
737.Bjornara HB, Vik FN, Brug J, et al. The association of breakfast skipping and television viewing at breakfast with weight status among parents of 10-12-year-olds in eight European countries; the ENERGY (EuropeaN Energy
© 2024 Zhang X et al. *JAMA Pediatrics.*

balance Research to prevent excessive weight Gain among Youth) cross-sectional study. *Public Health Nutr* 2014; **17**(4): 906-14.
738.Antal M, Peter S, Biro L, et al. Prevalence of underweight, overweight and obesity on the basis of body mass index and body fat percentage in Hungarian schoolchildren: representative survey in metropolitan elementary schools. *Ann Nutr Metab* 2009; **54**(3): 171-6.
739.Erdei G, Bakacs M, Illes E, et al. Substantial variation across geographic regions in the obesity prevalence among 6-8 years old Hungarian children (COSI Hungary 2016). *BMC Public Health* 2018; **18**(1): 611.
740.Jakab AE, Hidvegi EV, Illyes M, Cziraki A, Bereczki C. Prevalence of Overweight and Obesity in Hungarian Children and Adolescents. *Ann Nutr Metab* 2018; **72**(4): 259-64.
741.Aanesen A, Katzmarzyk PT, Ernstsen L. Breakfast skipping and overweight/obesity in first grade primary school children: A nationwide register-based study in Iceland. *Clin Obes* 2020; **10**(5): e12384.
742.Hrafnkelsson H, Magnusson KT, Sigurdsson EL, Johannsson E. Association of BMI and fasting insulin with cardiovascular disease risk factors in seven-year-old Icelandic children. *Scand J Prim Health Care* 2009; **27**(3): 186-91.
743.Saha J, Chouhan P, Ahmed F, et al. Overweight/Obesity Prevalence among Under-Five Children and Risk Factors in India: A Cross-Sectional Study Using the National Family Health Survey (2015-2016). *Nutrients* 2022; **14**(17).
744.Dabas A, Rastogi V, Khadgawat R, Marwaha RK. Predictive Performance of Different Diagnostic Criteria for Overweight and Obesity Between 2008-2015 in Adolescents. *Indian Pediatr* 2022; **59**(2): 110-3.
745.Arushi P, Dileep G, Devendra S, Hemlata M, Prateek A. Effect of Screen Time, Hours of Physical Activity and Sleep Hours on Overweight and Obesity in School-Going Adolescent Girls of Udaipur. *International Journal of Toxicological and Pharmacological Research* 2022; **12**(6): 17-27.
746.Thomas UM, Narayanappa D, Sujatha MS. Prevalence of overweight and obesity among school children in Mysuru, Karnataka. *J Family Med Prim Care* 2021; **10**(8): 2788-92.
747.Seema S, Rohilla KK, Kalyani VC, Babbar P. Prevalence and contributing factors for adolescent obesity in present era: Cross-sectional Study. *J Family Med Prim Care* 2021; **10**(5): 1890-4.
748.Moitra P, Madan J, Verma P. Independent and combined influences of physical activity, screen time, and sleep quality on adiposity indicators in Indian adolescents. *BMC Public Health* 2021; **21**(1): 2093.
749.Elangovan A, Mungara J, Joseph E. Exploring the relation between body mass index, diet, and dental caries among 6-12-year-old children. *J Indian Soc Pedod Prev Dent* 2012; **30**(4): 293-300.
750.Subramaniam P, Singh D. Association of age specific body mass index, dental caries and socioeconomic status of children and adolescents. *J Clin Pediatr Dent* 2011; **36**(2): 175-9.
751.Shailee F, GM S, KR S. Association between dental caries and body mass index among 12 and 15 years school children in Shimla, Himachal Pradesh. *Journal of Advanced Oral Research* 2013; **4**(1): 6-10.
752.Sutradhar B, Choudhuri D. Nutritional status of adolescent of Tripura-A North eastern state of India. *Biomedicine* 2017; **37**: 274-8.
753.Goyal JP, Kumar N, Parmar I, Shah VB, Patel B. Determinants of Overweight and Obesity in Affluent Adolescent in Surat City, South Gujarat region, India. *Indian J Community Med* 2011; **36**(4): 296-300.
754.Jain S, Pant B, Chopra H, Tiwari R. Obesity among adolescents of affluent public schools in Meerut. *Indian J Public Health* 2010; **54**(3): 158-60.
755.Gautam S, Jeong HS. Childhood Obesity and Its Associated Factors among School Children in Udupi, Karnataka, India. *J Lifestyle Med* 2019; **9**(1): 27-35.
756.Bharati DR, Deshmukh PR, Garg BS. Correlates of overweight & obesity among school going children of Wardha city, Central India. *Indian J Med Res* 2008; **127**(6): 539-43.
© 2024 Zhang X et al. *JAMA Pediatrics.*

757.Kotian MS, S GK, Kotian SS. Prevalence and determinants of overweight and obesity among adolescent school children of South karnataka, India. *Indian J Community Med* 2010; **35**(1): 176-8.
758.Warraich HJ, Javed F, Faraz-Ul-Haq M, Khawaja FB, Saleem S. Prevalence of obesity in school-going children of Karachi. *PLoS One* 2009; **4**(3): e4816.
759.Mushtaq MU, Gull S, Abdullah HM, Shahid U, Shad MA, Akram J. Prevalence and socioeconomic correlates of overweight and obesity among Pakistani primary school children. *BMC Public Health* 2011; **11**: 724.
760.Kumar S, Mahabalaraju D, Anuroopa M. Prevalence of Obesity and Its Infl uencing Factor among Affl uent School Children of Davangere City. *Indian journal of community medicine* 2007; **32**(1): 15-7.
761.Tiwari HC, Dwivedi S, Bali S, Parveen K. Overweight & obesity and it's correlates among school going adolescents of district Allahabad-a cross sectional study. *Indian J Prev Soc Med* 2014; **45**(1-2): 77-82.
762.Bansal S, Kumar R, Arif Husain CM. PREVALENCE OF OBESITY IN 11-15YRS OF URBAN SCHOOL CHILDREN USING BODY MASS INDEX, WAIST CIRCUMFERENCE AND WAIST HEIGHT RATIO. *Int J Acad Med Pharm* 2022; **4**(5): 172-6.
763.Baruah C. Prevalence and Correlates of overweight and obesity among school children in Guwahati city, Assam. *Indian J Community Health* 2018; **30**(1): 85-9.
764.Batson YA, Teelucksingh S, Maharaj RG, Cockburn BN. A cross-sectional study to determine the prevalence of obesity and other risk factors for type 2 diabetes among school children in Trinidad, West Indies. *Paediatr Int Child Health* 2014; **34**(3): 178-83.
765.Khadilkar VV, Khadilkar AV, Cole TJ, Chiplonkar SA, Pandit D. Overweight and obesity prevalence and body mass index trends in Indian children. *Int J Pediatr Obes* 2011; **6**(2-2): e216-24.
766.Sultan K, Habiba T. Prevalence of overweight and obesity in infancy. *Bangladesh Med Res Counc Bull* 2008; **34**(2): 69-70.
767.Gupta DK, Shah P, Misra A, et al. Secular trends in prevalence of overweight and obesity from 2006 to 2009 in urban asian Indian adolescents aged 14-17 years. *PLoS One* 2011; **6**(2): e17221.
768.Sharma A, Sharma K, Mathur KP. Growth pattern and prevalence of obesity in affluent schoolchildren of Delhi. *Public Health Nutr* 2007; **10**(5): 485-91.
769.Ramachandran A, Snehalatha C, Vinitha R, et al. Prevalence of overweight in urban Indian adolescent school children. *Diabetes Res Clin Pract* 2002; **57**(3): 185-90.
770.Bose K, Bisai S, Mukhopadhyay A, Bhadra M. Overweight and obesity among affluent Bengalee schoolgirls of Lake Town, Kolkata, India. *Matern Child Nutr* 2007; **3**(2): 141-5.
771.Mehta M, Bhasin SK, Agrawal K, Dwivedi S. Obesity amongst affluent adolescent girls. *Indian J Pediatr* 2007; **74**(7): 619-22.
772.Laxmaiah A, Nagalla B, Vijayaraghavan K, Nair M. Factors affecting prevalence of overweight among 12- to 17-year-old urban adolescents in Hyderabad, India. *Obesity (Silver Spring)* 2007; **15**(6): 1384-90.
773.Raj M, Sundaram KR, Paul M, Deepa AS, Kumar RK. Obesity in Indian children: time trends and relationship with hypertension. *Natl Med J India* 2007; **20**(6): 288-93.
774.Iyer U, Elayath N, Akolkar A. Magnitude and determinants of overweight and obesity in 6–12 year old school children of Vadodara City. *Curr Pediatr Res* 2011; **15**(2): 105-9.
775.Premanath M, Basavanagowdappa H, Shekar MA, Vikram SB, Narayanappa D. Mysore childhood obesity study. *Indian Pediatr* 2010; **47**(2): 171-3.
776.Marwaha RK, Tandon N, Singh Y, Aggarwal R, Grewal K, Mani K. A study of growth parameters and prevalence of overweight and obesity in school children from delhi. *Indian Pediatr* 2006; **43**(11): 943-52.
777.Kumar HN, Mohanan P, Kotian S, Sajjan BS, Kumar SG. Prevalence of overweight and obesity among preschool children in semi urban South India. *Indian Pediatr* 2008; **45**(6): 497-49.
© 2024 Zhang X et al. *JAMA Pediatrics.*

778.Sood A, Sundararaj P, Sharma S, Kurpad AV, Muthayya S. BMI and body fat percent: affluent adolescent girls in Bangalore City. *Indian Pediatr* 2007; **44**(8): 587-91.
779.Goyal RK, Shah VN, Saboo BD, et al. Prevalence of overweight and obesity in Indian adolescent school going children: its relationship with socioeconomic status and associated lifestyle factors. *J Assoc Physicians India* 2010; **58**: 151-8.
780.Ghosh A. Rural-urban comparison in prevalence of overweight and obesity among children and adolescents of Asian Indian origin. *Asia Pac J Public Health* 2011; **23**(6): 928-35.
781.Mahajan PB, Purty AJ, Singh Z, et al. Study of childhood obesity among school children aged 6 to 12 years in union territory of puducherry. *Indian J Community Med* 2011; **36**(1): 45-50.
782.Cherian AT, Cherian SS, Subbiah S. Prevalence of obesity and overweight in urban school children in Kerala, India. *Indian Pediatr* 2012; **49**(6): 475-7.
783.Thakre SB, Mohane SP, Ughade SM, Thakre SS, Morey S, Humne A. Correlates of overweight and obesity among urban school going children of Nagpur city. *Journal of clinical and diagnostic research* 2011; **5**(8): 1593-7.
784.Prashanth K, Baby KE, Rao KR, et al. Prevalence of obesity among high school children in Dakshina Kannada and Udupi districts. *Journal of Health and Allied Sciences NU* 2011; **1**(04): 16-20.
785.Vohra R, Bhardwaj P, Srivastava JP, Srivastava S, Vohra A. Overweight and obesity among school-going children of Lucknow city. *J Family Community Med* 2011; **18**(2): 59-62.
786.Chakraborty P, Dey S, Pal R, Kar S, Zaman FA, Pal S. Obesity in Kolkata children: Magnitude in relationship to hypertension. *J Nat Sci Biol Med* 2011; **2**(1): 101-6.
787.Sidhu S, Marwah G, Prabhjot. Prevalence of overweight and obesity among the affluent adolescent school children of Amritsar, Punjab. *Coll Antropol* 2005; **29**(1): 53-5.
788.Sidhu S, Kaur N, Kaur R. Overweight and obesity in affluent school children of Punjab. *Ann Hum Biol* 2006; **33**(2): 255-9.
789.Misra A, Shah P, Goel K, et al. The high burden of obesity and abdominal obesity in urban Indian schoolchildren: a multicentric study of 38,296 children. *Ann Nutr Metab* 2011; **58**(3): 203-11.
790.Jagadesan S, Harish R, Miranda P, Unnikrishnan R, Anjana RM, Mohan V. Prevalence of overweight and obesity among school children and adolescents in Chennai. *Indian Pediatr* 2014; **51**(7): 544-9.
791.Kaur S, Sachdev HP, Dwivedi SN, Lakshmy R, Kapil U. Prevalence of overweight and obesity amongst school children in Delhi, India. *Asia Pac J Clin Nutr* 2008; **17**(4): 592-6.
792.Chhabra G, Sodhi M. Impact of dietary habits on prevalence of obesity in children aged 5-17 years. *Int J Health Sci Res* 2012; **2**(1).
793.Chhatwal J, Verma M, Riar SK. Obesity among pre-adolescent and adolescents of a developing country (India). *Asia Pac J Clin Nutr* 2004; **13**(3): 231-5.
794.Saikia D, Ahmed SJ, Saikia H, Sarma R. Body mass index and body fat percentage in assessing obesity: An analytical study among the adolescents of Dibrugarh, Assam. *Indian J Public Health* 2018; **62**(4): 277-81.
795.V A, Shrivastava A. Prevalence of Obesity among School Going Children Attending Paediatrics Outpatient Department of a Private Medical College Hospital at Puducherry. *Journal of Evolution of Medical and Dental Sciences* 2019; **8**(42): 3135-9.
796.Chandra N, Anne B, Venkatesh K, Teja GD, Katkam SK. Prevalence of Childhood Obesity in an Affluent School in Telangana Using the Recent IAP Growth Chart: A Pilot Study. *Indian J Endocrinol Metab* 2019; **23**(4): 428-32.
797.Minhas S, Kunwar R, Sekhon H, Jaiswal P. Study of the anthropometry and prevalence of overweight in school boys in an Urban Area. *Journal of Marine Medical Society* 2019; **21**(1).
798.Mohan B, Verma A, Singh K, et al. Prevalence of sustained hypertension and obesity among urban and rural adolescents: a school-based, cross-sectional study in North India. *BMJ Open* 2019; **9**(9): e027134.
© 2024 Zhang X et al. *JAMA Pediatrics.*

799.Pinni J, Avula JSS, Bandi S. Association of dental caries with socio-demographic and nutritional factors among school children in Guntur district of Andhra Pradesh, India. *Pediatric Dental Journal* 2019; **29**(3): 111-5.
800.Sharma B, Indushekar KR, Saraf BG, Sardana D, Sheoran N, Mavi S. Are dental caries and overweight/obesity interrelated? A cross-sectional study in rural and urban preschool children. *J Indian Soc Pedod Prev Dent* 2019; **37**(3): 224-31.
801.Swaminathan K, Anandan V, H S, Thomas E. Correlation Between Body Mass Index and Dental Caries Among Three- to 12-Year-Old Schoolchildren in India: A Cross-Sectional Study. *Cureus* 2019; **11**(8): e5421.
802.Singh DP, Arya A, Kondepudi KK, Bishnoi M, Boparai RK. Prevalence and associated factors of overweight/obesity among school going children in Chandigarh, India. *Child Care Health Dev* 2020; **46**(5): 571-5.
803.Singh P, Gandhi S, Malhotra RK, Seth A. Impact of Using Different Growth References on Interpretation of Anthropometric Parameters of Children Aged 8-15 Years. *Indian Pediatr* 2020; **57**(2): 124-8.
804.Solanki DK, Walia R, Gautam A, Misra A, Aggarwal AK, Bhansali A. Prevalence of abdominal obesity in non-obese adolescents: a North Indian adolescent study. *J Pediatr Endocrinol Metab* 2020; **33**(7): 853-8.
805.K R. Prevalence of overweight and obesity among high school students of Thiruvananthapuram City Corporation, Kerala, India. *Australasian Medical Journal* 2010: 650-61.
806.Sharma A, Hegde AM. Relationship between body mass index, caries experience and dietary preferences in children. *J Clin Pediatr Dent* 2009; **34**(1): 49-52.
807.Rao S, Apte P. Social class-related gradient in the association of skeletal growth with blood pressure among adolescent boys in India. *Public Health Nutr* 2009; **12**(12): 2256-62.
808.Ghosh JR, Bandyopadhyay AR. Prevalence of thinness and overweight among urban adolescents of West Bengal, India. *J Trop Pediatr* 2009; **55**(5): 340-1.
809.Singh R, Bhansali A, Sialy R, Aggarwal A. Prevalence of metabolic syndrome in adolescents from a north Indian population. *Diabet Med* 2007; **24**(2): 195-9.
810.Senbanjo IO, Adejuyigbe EA. Prevalence of overweight and obesity in Nigerian preschool children. *Nutr Health* 2007; **18**(4): 391-9.
811.Viswanath HMK, Chinta VMK, Udayshankar PM. A Cross Sectional Study to Estimate Overweight & Obesity & its Contributing Factors in Adolescent Govt School Children in Chrompet, Chennai. *Indian Journal of Public Health Research & Development* 2015; **6**(3).
812.Joseph N, Nelliyanil M, Rai S, et al. Fast Food Consumption Pattern and Its Association with Overweight Among High School Boys in Mangalore City of Southern India. *J Clin Diagn Res* 2015; **9**(5): LC13-7.
813.Faizi N, Khan Z, Amir A, Azmi SA. Sleep duration and its effect on nutritional status in adolescents of Aligarh, India. *South African Journal of Child Health* 2015; **9**(1).
814.Basha S, Mohammad RN, Swamy HS, Sexena V. Association between Traumatic Dental Injury, Obesity, and Socioeconomic Status in 6- and 13-Year-Old Schoolchildren. *Soc Work Public Health* 2015; **30**(4): 336-44.
815.Jani R, Mihrshahi S, Mandalika S, Mallan KM. Accuracy of mothers' perceptions of their child's weight status. *Indian Pediatr* 2014; **51**(5): 412-3.
816.Gregori D, Gulati A, Paramesh EC, et al. Cross-regional analysis of multiple factors associated with childhood obesity in India: a national or local challenge? *Indian J Pediatr* 2014; **81 Suppl 1**: 5-16.
817.Ghosh A. Explaining overweight and obesity in children and adolescents of Asian Indian origin: the Calcutta childhood obesity study. *Indian J Public Health* 2014; **58**(2): 125-8.
818.Aroor AR, Airody SK, Mahale R, Sr R, Shetty S, Rao AR. Anthropometry and Prevalence of Common Health Problems among School Going Children in Surathkal, Karnataka. *J Clin Diagn Res* 2014; **8**(12): PC01-5.
819.Honne T, Pentapati K, Kumar N, Acharya S. Relationship between obesity/overweight status, sugar consumption and dental caries among adolescents in South India. *Int J Dent Hyg* 2012; **10**(4): 240-4.
© 2024 Zhang X et al. *JAMA Pediatrics.*

820.Basha S, Hiremath S, Mohamed R. Association Between Obesity, Dental Caries and Socioeconomic Status in 6- and 13-year-old School Children. *Oral health & preventive dentistry* 2012; **10**: 231-41.
821.Chakravathy KP, Thippeswamy HM, Kumar N, Chenna D. Relationship of body mass index and dental caries with oral health related quality of life among adolescents of Udupi district, South India. *Eur Arch Paediatr Dent* 2013; **14**(3): 155-9.
822.Maiti S, De D, Ali KM, Bera TK, Ghosh D, Paul S. Overweight and obesity among early adolescent school girls in urban area of west bengal, India: prevalence assessment using different reference standards. *Int J Prev Med* 2013; **4**(9): 1070-4.
823.Bhargava M, Kandpal SD, Aggarwal P, Sati HC. Overweight and Obesity in School Children of a Hill State in North India: Is the Dichotomy Urban-Rural or Socio-Economic? Results from a Cross-Sectional Survey. *PLoS One* 2016; **11**(5): e0156283.
824.Deepika B, Kalyani S, Manisha S. Comparison of WHO, CDC and IOTF growth references in relation to overweight and obesity in school going adolescents of Delhi, India. *Indian Journal of Public Health Research and Development* 2016; **7**(3): 83-90.
825.Pawar SV, Choksey AS, Jain SS, Surude RG, Rathi PM. Prevalence of Overweight and Obesity in 4 Schools of South Mumbai. *J Clin Diagn Res* 2016; **10**(3): OC01-2.
826.Prasad R, Bazroy J, Singh Z. Prevalence of overweight and obesity among adolescent students in Pondicherry, South India. *International Journal of Nutrition, Pharmacology, Neurological Diseases* 2016; **6**(2).
827.Saikia D, Ahmed SJ, Saikia H, Sarma R. Overweight and obesity in early adolescents and its relation to dietary habit and physical activity: A study in Dibrugarh town. *Clinical Epidemiology and Global Health* 2016; **4**: S22-S8.
828.Bharati S, Pal M, Shome S, Roy P, Dhara P, Bharati P. Influence of socio-economic status and television watching on childhood obesity in Kolkata. *Homo* 2017; **68**(6): 487-94.
829.Choudhary S, Kumar S, Bharati D, Rajak B, Shivani K, Shree V. Problem of Obesity among School Going Adolescent in Rural Practice Area of Indira Gandhi Institute of Medical Sciences, Patna. *International journal of scientific study* 2017; **102**.
830.Eshwar TK, Chudasama RK, Eshwar ST, Thakrar D. Prevalence of obesity and overweight and their comparison by three growth standards among affluent school students aged 8-18 years in Rajkot. *Indian J Public Health* 2017; **61**(1): 51-4.
831.Ganie MA, Bhat GA, Wani IA, et al. Prevalence, risk factors and consequences of overweight and obesity among schoolchildren: a cross-sectional study in Kashmir, India. *J Pediatr Endocrinol Metab* 2017; **30**(2): 203-9.
832.Haq I, Raja M, Ahmad M. A comparison of the 2015 Indian Academy of Pediatrics, International Obesity Task Force and World Health Organization growth references among 5–18-year-old children. *Annals of Tropical Medicine and Public Health* 2017; **10**(6).
833.Kumar S, Kroon J, Lalloo R, Kulkarni S, Johnson NW. Relationship between body mass index and dental caries in children, and the influence of socio-economic status. *Int Dent J* 2017; **67**(2): 91-7.
834.Mishra A, Acharya H. Factors influencing obesity among school-going children in Sambalpur district of Odisha. *Journal of Medical Society* 2017; **31**(3).
835.Shah DP, Maiya AG. Correlation between academic performance and obesity in school-children from Anand district. *International Journal of Physiotherapy* 2017: 222-8.
836.Greene-Cramer B, Harrell MB, Hoelscher DM, et al. Association between parent and child weight status among private school children in Delhi, India. *Glob Health Promot* 2018; **25**(2): 67-74.
837.Andriani H. Birth weight and childhood obesity: effect modification by residence and household wealth. *Emerg Themes Epidemiol* 2021; **18**(1): 6.
838.Sarintohe E, Larsen JK, Burk WJ, Vink JM. The Prevalence of Overweight Status among Early Adolescents
© 2024 Zhang X et al. *JAMA Pediatrics.*

from Private Schools in Indonesia: Sex-Specific Patterns Determined by School Urbanization Level. *Nutrients* 2022; **14**(5).
839.Windiani IGAT, Agustini NKW, Adnyana IGANS, Soetjiningsih S, Murti NLSP. The association between nutritional status and risk of developmental disorder in children in Denpasar Bali Indonesia. *Open Access Macedonian Journal of Medical Sciences* 2021; **9**(B): 687-91.
840.Aditya CJ, Sekartini R. Association between obesity and behavioral/emotional disorders in primary school-aged children: a cross-sectional study. *Medical Journal of Indonesia* 2017; **26**(1): 70-5.
841.Agustina R, Meilianawati, Fenny, et al. Psychosocial, Eating Behavior, and Lifestyle Factors Influencing Overweight and Obesity in Adolescents. *Food Nutr Bull* 2021; **42**(1_suppl): S72-S91.
842.Collins AE, Pakiz B, Rock CL. Factors associated with obesity in Indonesian adolescents. *Int J Pediatr Obes* 2008; **3**(1): 58-64.
843.Rachmi CN, Agho KE, Li M, Baur LA. Stunting, Underweight and Overweight in Children Aged 2.0-4.9 Years in Indonesia: Prevalence Trends and Associated Risk Factors. *PLoS One* 2016; **11**(5): e0154756.
844.Yuwanita N, Sinuhaji AB, Sembiring T, Supriatmo S, Yudiyanto AR. Obesity and functional constipation in children. *Paediatrica Indonesiana* 2018; **58**(1).
845.Febriani D, Sudarti T. Fast food as drivers for overweight and obesity among urban school children at Jakarta, Indonesia. *Jurnal Gizi dan Pangan* 2019; **14**(2): 99-106.
846.Maehara M, Rah JH, Roshita A, Suryantan J, Rachmadewi A, Izwardy D. Patterns and risk factors of double burden of malnutrition among adolescent girls and boys in Indonesia. *PLoS One* 2019; **14**(8): e0221273.
847.Melinda TF, Sekartini R. Association between obesity and sleep disorders in primary school children: a cross-sectional study. *Medical Journal of Indonesia* 2019; **28**(2): 167-73.
848.Rizkiriani A, Khomsan A, Riyadi H. Obesity and hypertension among adolescents in Jakarta, Indonesia. *Pakistan Journal of Nutrition* 2013; **13**(1): 17-21.
849.Syahrul S, Kimura R, Tsuda A, Susanto T, Saito R, Ahmad F. Prevalence of underweight and overweight among school-aged children and it's association with children's sociodemographic and lifestyle in Indonesia. *International Journal of Nursing Sciences* 2016; **3**(2): 169-77.
850.Yulia C, Khomsan A, Sukandar D, Riyadi H. Nutritional Status, Lipid Profile and Blood Pressure among Primary Schoolchildren in Urban West Java, Indonesia. *Pakistan Journal of Nutrition* 2017; **16**(10): 770-5.
851.Kalsum U, Nainggolan S, Ng N. Pizza and Hamburger Consumption to Overweight among Adolescents in Jambi City. *Kesmas: National Public Health Journal* 2018; **13**(2).
852.Moradi G, Mostafavi F, Azadi N, Esmaeilnasab N, Nouri B. Evaluation of screen time activities and their relationship with physical activity, overweight and socioeconomic status in children 10-12 years of age in Sanandaj, Iran: A cross-sectional study in 2015. *Med J Islam Repub Iran* 2016; **30**: 448.
853.Soheilipour F, Pishgahroudsari M, Pazouki A. Overweight and Obesity Prevalence in Iranian Children Aged 8 - 12 Years; A Study in Tehran. *Journal of Comprehensive Pediatrics* 2022; **13**(3).
854.Barati L, Radgoodarzi M, Vakili M, Tabatabaizadeh M. Overweight and obesity: worldwide risk factors for pediatric hypertension. *ARYA Atheroscler* 2022; **18**(1): 1-6.
855.Mohammadi S, Mohammadi MA, Dadkhah B. Dental caries prevalence among elementary school students and its relationship with body mass index and oral hygiene in Ardabil in 2019. *J Indian Soc Pedod Prev Dent* 2021; **39**(2): 147-53.
856.Entezarmahdi R, Houshiarrad A, Gheibi S, et al. Dietary and Socio-Economic Predictors of Obesity Among 2-5-Year Old in Northwest Iran. *Int J Prev Med* 2021; **12**: 116.
857.Jari M, Qorbani M, Moafi M, et al. Association of 25-hydroxy Vitamin D levels with indexes of general and abdominal obesity in Iranian adolescents: The CASPIAN-III study. *J Res Med Sci* 2015; **20**(2): 122-6.
© 2024 Zhang X et al. *JAMA Pediatrics.*

858.Bagherian A, Sadeghi M. Association between dental caries and age-specific body mass index in preschool children of an Iranian population. *Indian J Dent Res* 2013; **24**(1): 66-70.
859.Sadeghi M, Lynch CD, Arsalan A. Is there a correlation between dental caries and body mass index-for-age among adolescents in Iran? *Community Dent Health* 2011; **28**(2): 174-7.
860.Shahraki T, Shahraki M, Omrani Mehr S. Association between body mass index and caries frequency among zahedan elementary school children. *Int J High Risk Behav Addict* 2013; **2**(3): 122-5.
861.Agha-Alinejad H, Farzad B, Salari M, Kamjoo S, Harbaugh BL, Peeri M. Prevalence of overweight and obesity among Iranian preschoolers: Interrelationship with physical fitness. *J Res Med Sci* 2015; **20**(4): 334-41.
862.Javedan GA, Salehiniya H, Soheilipour F. Prevalence of obesity and overweight in preschool children in northwest of Tehran, Iran. *Journal of Krishna Institute of Medical Sciences University* 2016; **5**(4): 58-63.
863.Fatemeh T, Mohammad-Mehdi HT, Toba K, Afsaneh N, Sharifzadeh G. Prevalence of overweight and obesity in preschool children (2-5 year-olds) in Birjand, Iran. *BMC Res Notes* 2012; **5**: 529.
864.Ahmadi A, Gharipour M, Nouri F, Kelishadi R, Sadeghi M, Sarrafzadegan N. Association between adolescence obesity and metabolic syndrome: Evidence from Isfahan Healthy Heart Program. *Indian J Endocrinol Metab* 2014; **18**(4): 569-73.
865.Badeli H, Hassankhani A, Naeemi Z, et al. Prevalence of Hypertension and Obesity-related Hypertension in Urban School-aged Children in Rasht. *Iranian journal of kidney diseases* 2016; **10**: 364-8.
866.Bahreini N, Noor MI, Koon PB, et al. Weight status among Iranian adolescents: Comparison of four different criteria. *Journal of research in medical sciences: the official journal of Isfahan University of Medical Sciences* 2013; **18**(8): 641.
867.Bahreynian M, Kelishadi R, Qorbani M, et al. Weight disorders and anthropometric indices according to socioeconomic status of living place in Iranian children and adolescents: The CASPIAN-IV study. *Journal of research in medical sciences: The official journal of Isfahan University of Medical Sciences* 2015; **20**(5): 440.
868.Basiratnia M, Derakhshan D, Ajdari S, Saki F. Prevalence of childhood obesity and hypertension in south of Iran. *Iranian journal of kidney diseases* 2013; **7**(4): 282.
869.Maddah M, Nikooyeh B. Factors associated with overweight in children in Rasht, Iran: gender, maternal education, skipping breakfast and parental obesity. *Public Health Nutr* 2010; **13**(2): 196-200.
870.Kelishadi R, Ardalan G, Gheiratmand R, et al. Thinness, overweight and obesity in a national sample of Iranian children and adolescents: CASPIAN Study. *Child Care Health Dev* 2008; **34**(1): 44-54.
871.Fatemeh T, Mohammad-Mehdi H-T, Toba K, Afsaneh N, Sharifzadeh G, Committee SR. Prevalence of overweight and obesity in preschool children (2–5 year-olds) in Birjand, Iran. *BMC Research Notes* 2012; **5**: 1-6.
872.Motlagh ME, Kelishadi R, Amirkhani MA, et al. Double burden of nutritional disorders in young Iranian children: findings of a nationwide screening survey. *Public Health Nutr* 2011; **14**(4): 605-10.
873.Ziaoddini H, Kelishadi R, Kamsari F, Mirmoghtadaee P, Poursafa P. First nationwide survey of prevalence of weight disorders in Iranian children at school entry. *World J Pediatr* 2010; **6**(3): 223-7.
874.Ayatollahi SM, Mostajabi F. Prevalence of obesity among schoolchildren in Iran. *Obes Rev* 2007; **8**(4): 289-91.
875.Khodaverdi F, Alhani F, Kazemnejad A, Khodaverdi Z. The relationship between obesity and quality of life in school children. *Iranian journal of public health* 2011; **40**(2): 96.
876.Kajbaf TZ, Asar S, Alipoor MR. Relationship between obesity and asthma symptoms among children in Ahvaz, Iran: a cross sectional study. *Italian journal of pediatrics* 2011; **37**: 1-5.
877.Vafa M, Moslehi N, Afshari S, Hossini A, Eshraghian M. Relationship between breastfeeding and obesity in childhood. *Journal of health, population, and nutrition* 2012; **30**(3): 303.
878.Zarrati M, Shidfar F, Moradof M, et al. Relationship between Breast Feeding and Obesity in Children with Low Birth Weight. *Iran Red Crescent Med J* 2013; **15**(8): 676-82.
© 2024 Zhang X et al. *JAMA Pediatrics.*

879.Hajian-Tilaki K, Heidari B. Prevalences of overweight and obesity and their association with physical activity pattern among Iranian adolescents aged 12-17 years. *Public Health Nutr* 2012; **15**(12): 2246-52.
880.Gaeini A, Kashef M, Samadi A, Fallahi A. Prevalence of underweight, overweight and obesity in preschool children of Tehran, Iran. *Journal of research in medical sciences: the official journal of Isfahan University of Medical Sciences* 2011; **16**(6): 821.
881.Montazerifar F, Karajibani M, Rakhshani F, Hashemi M. Prevalence of underweight, overweight and obesity among high-school girls in Sistan va Baluchistan. *EMHJ-Eastern Mediterranean Health Journal, 15 (5), 1293-1300, 2009* 2009.
882.Heidari K, Minasian V, Marandi SM, Kelishadi R, Khalighinejad P, Davari H. Prevalence of Obesity and Overweight in 12-14-year-old Students in Isfahan-Iran. *International Journal of Preventive Medicine* 2014; **5**(Suppl 2): S120.
883.Hajian-Tilaki KO, Sajjadi P, Razavi A. Prevalence of overweight and obesity and associated risk factors in urban primary-school children in Babol, Islamic Republic of Iran. *East Mediterr Health J* 2011; **17**(2): 109-14.
884.Gargari BP, Behzad MH, Ghassabpour S, Ayat A. Prevalence of overweight and obesity among high-school girls in Tabriz, Iran, in 2001. *Food Nutr Bull* 2004; **25**(3): 288-91.
885.Tabesh H, Hosseiny SM, Kompani F, et al. Prevalence and trend of overweight and obesity among schoolchildren in Ahvaz, Southwest of Iran. *Glob J Health Sci* 2013; **6**(2): 35-41.
886.Shafaghi K, Shariff ZM, Taib MN, Rahman HA, Mobarhan MG, Jabbari H. Parental body mass index is associated with adolescent overweight and obesity in Mashhad, Iran. *Asia Pac J Clin Nutr* 2014; **23**(2): 225-31.
887.Moayeri H, Bidad K, Aghamohammadi A, et al. Overweight and obesity and their associated factors in adolescents in Tehran, Iran, 2004-2005. *Eur J Pediatr* 2006; **165**(7): 489-93.
888.Behzadnia S, Vahidshahi K, Hamzeh Hosseini S, Anvari S, Ehteshami S. Obesity and related factors in 7-12 year-old elementary school students during 2009-2010 in Sari, Iran. *Med Glas (Zenica)* 2012; **9**(1): 86-90.
889.Maddah M, Nikooyeh B. Obesity among Iranian adolescent girls: location of residence and parental obesity. *J Health Popul Nutr* 2010; **28**(1): 61-6.
890.Nouri Saeidlou S, Babaei F, Ayremlou P. Malnutrition, overweight, and obesity among urban and rural children in north of west Azerbijan, Iran. *J Obes* 2014; **2014**: 541213.
891.Kavehmanesh Z, Saburi A, Maavaiyan A. Comparison of body mass index on children with functional constipation and healthy controls. *J Family Med Prim Care* 2013; **2**(3): 222-6.
892.Hajian-Tilaki K, Heidari B. Childhood Obesity, Overweight, Socio-Demographic and Life Style Determinants among Preschool Children in Babol, Northern Iran. *Iran J Public Health* 2013; **42**(11): 1283-91.
893.Mirhosseini NZ, Shahar S, Ghayour-Mobarhan M, Parizadeh MR, Yusoff NA, Shakeri MT. Body fat distribution and its association with cardiovascular risk factors in adolescent Iranian girls. *Iran J Pediatr* 2012; **22**(2): 197-204.
894.Heshmat R, Kelishadi R, Motamed-Gorji N, et al. Association between body mass index and perceived weight status with self-rated health and life satisfaction in Iranian children and adolescents: the CASPIAN-III study. *Qual Life Res* 2015; **24**(1): 263-72.
895.Sadeghi M, Alizadeh F. Association between Dental Caries and Body Mass Index-For-Age among 6-11-Year-Old Children in Isfahan in 2007. *J Dent Res Dent Clin Dent Prospects* 2007; **1**(3): 119-24.
896.Zekavat OR, Makarem AR, Shayan Z, Shojaee M, Karami MY. Obesity and overweight among primary school children in Iran. *Minerva Pediatr* 2014; **66**(6): 579-84.
897.Ejtahed HS, Heshmat R, Motlagh ME, et al. Association of parental obesity with cardiometabolic risk factors in their children: The CASPIAN-V study. *PLoS One* 2018; **13**(4): e0193978.
898.Salehi-Abargouei A, Abdollahzad H, Bameri Z, Esmaillzadeh A. Underweight, overweight and obesity among zaboli adolescents: a comparison between international and iranians' national criteria. *Int J Prev Med* 2013; **4**(5):
© 2024 Zhang X et al. *JAMA Pediatrics.*

523-30.
899.Maddah M. Overweight among rural girls in Iran: a terrifying prospects of cardiometabolic disorders. *Int J Cardiol* 2009; **132**(3): 442-4.
900.Taheri F, Kazemi T, Chahkandi T, Namakin K, Zardast M, Bijari B. Prevalence of overweight, obesity and central obesity among elementary school children in Birjand, east of Iran, 2012. *J Res Health Sci* 2013; **13**(2): 157-61.
901.Zakeri M, Sedaghat M, Motlagh ME, Tayari Ashtiani R, Ardalan G. BMI correlation with psychiatric problems among 10-18 years Iranian students. *Acta Med Iran* 2012; **50**(3): 177-84.
902.Jari M, Qorbani M, Motlagh ME, Heshmat R, Ardalan G, Kelishadi R. Association of Overweight and Obesity with Mental Distress in Iranian Adolescents: The CASPIAN-III Study. *Int J Prev Med* 2014; **5**(3): 256-61.
903.Sedaghat F, Naja F, Darand M, Beyzai B, Rashidkhani B. Adherence to a Mediterranean dietary pattern and overweight and obesity among female adolescents in Iran. *Int J Adolesc Med Health* 2017; **31**(2).
904.Rafraf M, Gargari BP, Safaiyan A. Prevalence of prehypertension and hypertension among adolescent high school girls in Tabriz, Iran. *Food and nutrition bulletin* 2010; **31**(3): 461-5.
905.Maddah M, Shahraki T, Shahraki M. Underweight and overweight among children in Zahedan, south-east Iran. *Public Health Nutr* 2010; **13**(10): 1519-21.
906.Maddah M. Association of parental diabetes with overweight in Iranian children and adolescents. *International journal of cardiology* 2010; **144**(1): 126-8.
907.Salem Z, Vazirinejad R. Prevalence of obesity and metabolic syndrome in adolescent girls in South East of Iran. *Pak J Med Sci* 2009; **25**(2): 196-200.
908.Maddah M, Rashidi A, Mohammadpour B, Vafa R, Karandish M. In-school snacking, breakfast consumption, and sleeping patterns of normal and overweight Iranian high school girls: a study in urban and rural areas in Guilan, Iran. *J Nutr Educ Behav* 2009; **41**(1): 27-31.
909.Kelishadi R, Cook SR, Adibi A, et al. Association of the components of the metabolic syndrome with non-alcoholic fatty liver disease among normal-weight, overweight and obese children and adolescents. *Diabetol Metab Syndr* 2009; **1**: 29.
910.Fallahzadeh H, Golestan M, Rezvanian T, Ghasemian Z. Breast-feeding history and overweight in 11 to 13-year-old children in Iran. *World J Pediatr* 2009; **5**(1): 36-41.
911.Azita F, Asghar Z, Gholam-Reza S. Relationship of body mass index with serum lipids in elementary school students. *The Indian Journal of Pediatrics* 2009; **76**: 729-31.
912.Bidad K, Anari S, Tavasoli S, et al. Dietary intakes of adolescent girls in relation to weight status. 2008.
913.Rashidi A, Mohammadpour-Ahranjani B, Karandish M, et al. Obese and female adolescents skip breakfast more than their non-obese and male peers. *Open Medicine* 2007; **2**(4): 481-7.
914.Mozaffari H, Nabaei B. Obesity and related risk factors. *Indian J Pediatr* 2007; **74**(3): 265-7.
915.Maddah M. Overweight and obesity among Iranian female adolescents in Rasht: more overweight in the lower social group. *Public Health Nutr* 2007; **10**(5): 450-3.
916.Tabriz AA, Sohrabi MR, Parsay S, et al. Relation of intelligence quotient and body mass index in preschool children: a community-based cross-sectional study. *Nutr Diabetes* 2015; **5**(8): e176.
917.Ghadimi R, Asgharzadeh E, Sajjadi P. Obesity among Elementary Schoolchildren: A Growing Concern in the North of Iran, 2012. *Int J Prev Med* 2015; **6**: 99.
918.Jalali-Farahani S, Chin YS, Amiri P, Mohd Taib MN. Body mass index (BMI)-for-age and health-related quality of life (HRQOL) among high school students in Tehran. *Child Care Health Dev* 2014; **40**(5): 731-9.
919.Hatami M, Taib MN, Jamaluddin R, et al. Dietary factors as the major determinants of overweight and obesity among Iranian adolescents. A cross-sectional study. *Appetite* 2014; **82**: 194-201.
920.Ahmadi A, Moazen M, Mosallaei Z, Mohammadbeigi A, Amin-lari F. Nutrient intake and growth indices for
© 2024 Zhang X et al. *JAMA Pediatrics.*

children at kindergartens in Shiraz, Iran. *perspective* 2014; **1**: 3.
921.Motlagh ME, Kelishadi R, Ziaoddini H, et al. Secular trends in the national prevalence of overweight and obesity during 2007-2009 in 6-year-old Iranian children. *J Res Med Sci* 2011; **16**(8): 979-84.
922.Hatami M, Mohd Taib MN, Jamaluddin R, Abu Saad H, Djazayery A. Body change techniques in Iranian adolescents. Relationship to sex and body weight status. *Appetite* 2013; **60**(1): 27-32.
923.Hassanzadeh-Rostami Z, Kavosi E, Nasihatkon A. Overweight and obesity among preschool children from Fars province of Iran: prevalence and associated factors. *Journal of research in health sciences* 2016; **16**(1): 26.
924.Keykhaei F, Shahraki M, Sargolhosseinzadeh E, Shahraki T, Dashipour A. Correlation of Body Mass Index and Physical Activity Among 7- to 11-Year Children at Zahedan, Iran. *Food Nutr Bull* 2016; **37**(3): 364-74.
925.Nouri Saeidlou S, Rezaiegoyjeloo F, Ayremlou P, Babaie F. Changes of Overweight and Obesity Prevalence Among School Children in North West of Iran After 3 Years Follow-up (2009-2011): A Longitudinal Study. *Int J Prev Med* 2016; **7**: 79.
926.Salehiniya H, Yazdani K, Barekati H, Asadi Lari M. The Prevalence of Overweight and Obesity in Children Under 5 Years in Tehran, Iran, in 2012: A Population-Based Study. *Res Cardiovasc Med* 2016; **5**(1): e30425.
927.Darabiyan P, Baraz S, Elahi N, et al. AN INVESTIGATION OF PREVALENCE OF OBESITY AND ITS RELATED DEMOGRAPHIC FACTORS IN STUDENTS AGED 12-16 YEARS OLD IN AHVAZ IN 2017. *Indo American Journal of Pharmaceutical Sciences* 2018; **5**(1): 413-9.
928.Ghobadi S, Totosy de Zepetnek JO, Hemmatdar Z, et al. Association between overweight/obesity and eating habits while watching television among primary-school children in the city of Shiraz, Iran. *Public Health Nutr* 2018; **21**(3): 571-9.
929.Heshmat R, Hemati Z, Payab M, et al. Prevalence of different metabolic phenotypes of obesity in Iranian children and adolescents: the CASPIAN V study. *J Diabetes Metab Disord* 2018; **17**(2): 211-21.
930.Motlagh M, Shirvani S, Hassanzadeh-Rostami Z, Taheri M, Ghadimi R. Assessment of overweight and obesity in Iranian adolescents: optimal cut-off values of anthropometric indices. *East Mediterr Health J* 2018; **24**(10): 975-87.
931.Parastar S, Ebrahimpour K, Hashemi M, et al. Association of urinary concentrations of four chlorophenol pesticides with cardiometabolic risk factors and obesity in children and adolescents. *Environ Sci Pollut Res Int* 2018; **25**(5): 4516-23.
932.Abood GM, Kadhem RH, Mohan JB. Prevalence and determinants of overweight and obesity among public primary school students in AL-Nasiriya city at 2018-2019. *Current Pediatric Research* 2021; **25**(8): 805-12.
933.Lafta RK, Kadhim MJ. Childhood obesity in Iraq: prevalence and possible risk factors. *Ann Saudi Med* 2005; **25**(5): 389-93.
934.Amin SS. Obesity prevalence in primary school children in Baghdad City. *Research Journal of Pharmacy and Technology* 2019; **12**(1): 339-41.
935.Lafta R, Al Saffar A, Eisa S, Hayyawi A, Abdulhameed F. Obesity in children: A sample from Baghdad. *Qatar Medical Journal* 2007; **2007**(1): 8.
936.Musaiger AO, Al-Mannai M, Al-Haifi AR, et al. Prevalence of overweight and obesity among adolescents in eight Arab countries: comparison between two international standards (ARABEAT-2). *Nutr Hosp* 2016; **33**(5): 567.
937.Reulbach U, Ladewig EL, Nixon E, O'Moore M, Williams J, O'Dowd T. Weight, body image and bullying in 9-year-old children. *J Paediatr Child Health* 2013; **49**(4): E288-93.
938.Quinn L, Brady B, Ball E, Keaver L. An exploration into the influence of birthweight and breastfeeding status on the weight status of 3-year-olds in an Irish cohort. *Irish Journal of Medical Science (1971-)* 2023; **192**(1): 199-205.
939.Moore Heslin A, O'Donnell A, Kehoe L, et al. Adolescent overweight and obesity in Ireland-Trends and sociodemographic associations between 1990 and 2020. *Pediatr Obes* 2023; **18**(2): e12988.
© 2024 Zhang X et al. *JAMA Pediatrics.*

940.Barron C, Comiskey C, Saris J. Prevalence rates and comparisons of obesity levels in Ireland. *British journal of nursing* 2009; **18**(13): 799-803.
941.Dowd KP, Kirwan RP, Hannigan A, Purtill H, O'Gorman CS. The association between maternal perceptions of own weight status and weight status of her child: results from a national cohort study. *Arch Dis Child* 2016; **101**(1): 28-32.
942.Evans DS, Glacken M, Goggin D. Childhood obesity: the extent of the problem among 6-year-old Irish national school children. *Child Care Health Dev* 2011; **37**(3): 352-9.
943.Whelton H, Harrington J, Crowley E, Kelleher V, Cronin M, Perry IJ. Prevalence of overweight and obesity on the island of Ireland: results from the North South Survey of Children's Height, Weight and Body Mass Index, 2002. *BMC Public Health* 2007; **7**: 187.
944.O'Neill JL, McCarthy SN, Burke SJ, et al. Prevalence of overweight and obesity in Irish school children, using four different definitions. *Eur J Clin Nutr* 2007; **61**(6): 743-51.
945.Kelly D, Kelly A, O'Dowd T, Hayes CB. Antibiotic use in early childhood and risk of obesity: longitudinal analysis of a national cohort. *World J Pediatr* 2019; **15**(4): 390-7.
946.McMaster C, Cullen L, Raymond N. Overweight and obesity in Irish primary schools: retrospective cohort study. *Child: care, health and development* 2005; **31**(5): 499-506.
947.Belton S, Brady P, Meegan S, Woods C. Pedometer step count and BMI of Irish primary school children aged 6-9 years. *Prev Med* 2010; **50**(4): 189-92.
948.Perry CP, Keane E, Layte R, Fitzgerald AP, Perry IJ, Harrington JM. The use of a dietary quality score as a predictor of childhood overweight and obesity. *BMC Public Health* 2015; **15**: 581.
949.Pinhas-Hamiel O, Bar-Zvi E, Boyko V, Reichman B, Lerner-Geva L. Prevalence of overweight in kindergarten children in the centre of Israel - association with lifestyle habits. *Child Care Health Dev* 2009; **35**(2): 147-52.
950.Bar Dayan Y, Elishkevits K, Grotto I, et al. The prevalence of obesity and associated morbidity among 17-year-old Israeli conscripts. *Public Health* 2005; **119**(5): 385-9.
951.Soskolne V, Cohen-Dar M, Obeid S, Cohen N, Rudolf MCJ. Risk and Protective Factors for Child Overweight/Obesity Among Low Socio-Economic Populations in Israel: A Cross Sectional Study. *Front Endocrinol (Lausanne)* 2018; **9**: 456.
952.Loewenberg Weisband Y, Kaufman-Shriqui V, Wolff Sagy Y, Krieger M, Abu Ahmad W, Manor O. Area-level socioeconomic disparity trends in nutritional status among 5-6-year-old children in Israel. *Arch Dis Child* 2020; **105**(11): 1049-54.
953.Meyerovitch J, Goldman RD, Avner-Cohen H, MBS FA. Primary care screening for childhood obesity: a population-based analysis. *children* 2007; **15**: 70.
954.Goldberg S, Werbeloff N, Fruchter E, Portuguese S, Davidson M, Weiser M. IQ and obesity in adolescence: a population-based, cross-sectional study. *Pediatr Obes* 2014; **9**(6): 419-26.
955.Kaufman-Shriqui V, Fraser D, Friger M, et al. Factors associated with childhood overweight and obesity among acculturated and new immigrants. *Ethnicity & Disease* 2013; **23**(3): 329-35.
956.Ram E, Marcus O, Joubran S, Abdo B, Asal NR. Prevalence of obesity among Arab school children in Nazareth, Israel: comparison with national (Jewish) and international data. *Pediatr Obes* 2013; **8**(6): 428-38.
957.Galfo M, Censi L, D'Addezio L, Melini F, Martone D. Overweight/obesity and lifestyle factors among Italian adolescents: the ALIADO study. *Minerva Pediatr (Torino)* 2022; **74**(3): 251-8.
958.Ashi H, Campus G, Klingberg G, Forslund HB, Lingstrom P. Childhood obesity in relation to sweet taste perception and dental caries - a cross-sectional multicenter study. *Food Nutr Res* 2019; **63**.
959.Costacurta M, Di Renzo L, Bianchi A, Fabiocchi F, De Lorenzo A, Docimo R. Obesity and dental caries in paediatric patients. A cross-sectional study. *Eur J Paediatr Dent* 2011; **12**(2): 112-6.
© 2024 Zhang X et al. *JAMA Pediatrics.*

960.Barba G, Troiano E, Russo P, Venezia A, Siani A. Inverse association between body mass and frequency of milk consumption in children. *Br J Nutr* 2005; **93**(1): 15-9.
961.Barba G, Troiano E, Russo P, Strazzullo P, Siani A. Body mass, fat distribution and blood pressure in Southern Italian children: results of the ARCA project. *Nutr Metab Cardiovasc Dis* 2006; **16**(4): 239-48.
962.Fioravanti S, Cesaroni G, Badaloni C, Michelozzi P, Forastiere F, Porta D. Traffic-related air pollution and childhood obesity in an Italian birth cohort. *Environ Res* 2018; **160**: 479-86.
963.Ceschia A, Giacomini S, Santarossa S, et al. Deleterious effects of obesity on physical fitness in pre-pubertal children. *Eur J Sport Sci* 2016; **16**(2): 271-8.
964.Toselli S, Brasili P, Spiga F. Body image, body dissatisfaction and weight status in children from Emilia-Romagna (Italy): comparison between immigrant and native-born. *Ann Hum Biol* 2014; **41**(1): 23-8.
965.Caserta CA, Pendino GM, Alicante S, et al. Body mass index, cardiovascular risk factors, and carotid intima-media thickness in a pediatric population in southern Italy. *J Pediatr Gastroenterol Nutr* 2010; **51**(2): 216-20.
966.Genovesi S, Antolini L, Giussani M, et al. Hypertension, prehypertension, and transient elevated blood pressure in children: association with weight excess and waist circumference. *Am J Hypertens* 2010; **23**(7): 756-61.
967.Bertoncello C, Cazzaro R, Ferraresso A, Mazzer R, Moretti G. Prevalence of overweight and obesity among school-aged children in urban, rural and mountain areas of the Veneto Region, Italy. *Public Health Nutr* 2008; **11**(9): 887-90.
968.Albertini A, Tripodi A, Fabbri A, et al. Prevalence of obesity in 6- and 9-year-old children living in Central-North Italy. Analysis of determinants and indicators of risk of overweight. *Obes Rev* 2008; **9**(1): 4-10.
969.Maffeis C, Consolaro A, Cavarzere P, et al. Prevalence of overweight and obesity in 2-to 6-year-old Italian children. Wiley Online Library; 2006.
970.Parrino C, Rossetti P, Baratta R, et al. Secular trends in the prevalence of overweight and obesity in Sicilian schoolchildren aged 11-13 years during the last decade. *PLoS One* 2012; **7**(4): e34551.
971.Parrino C, Vinciguerra F, La Spina N, et al. Influence of early-life and parental factors on childhood overweight and obesity. *J Endocrinol Invest* 2016; **39**(11): 1315-21.
972.Lombardo FL, Spinelli A, Lazzeri G, et al. Severe obesity prevalence in 8- to 9-year-old Italian children: a large population-based study. *Eur J Clin Nutr* 2015; **69**(5): 603-8.
973.Cibella F, Cuttitta G, La Grutta S, Melis MR, Bucchieri S, Viegi G. A cross-sectional study assessing the relationship between BMI, asthma, atopy, and eNO among schoolchildren. *Ann Allergy Asthma Immunol* 2011; **107**(4): 330-6.
974.Genovesi S, Giussani M, Pieruzzi F, et al. Results of blood pressure screening in a population of school-aged children in the province of Milan: role of overweight. *Journal of hypertension* 2005; **23**(3): 493-7.
975.Valerio G, Scalfi L, De Martìno C, Franzese A, Tenore A, Contaldo F. Comparison between different methods to assess the prevalence of obesity in a sample of Italian children. *Journal of Pediatric Endocrinology and Metabolism* 2003; **16**(2): 211-6.
976.Pecoraro P, Guida B, Caroli M, et al. Body mass index and skinfold thickness versus bioimpedance analysis: fat mass prediction in children. *Acta Diabetol* 2003; **40 Suppl 1**: S278-81.
977.Collo A, Ferro A, Belci P, et al. Nutritional behavior in Italian and immigrant children. *Minerva Pediatr* 2019; **71**(6): 481-7.
978.Toselli S, Argnani L, Canducci E, Ricci E, Gualdi-Russo E. Food habits and nutritional status of adolescents in Emilia-Romagna, Italy. *Nutricion hospitalaria* 2010; **25**(4): 613-21.
979.Greco E, Meo F, Cedrone F. Gender differences in childhood bmi z-score, alimentary behaviour and lifestyle in a sample of 9-11 children. *Clin Ter* 2020; **171**(5): e425-e30.
980.Binkin N, Fontana G, Lamberti A, et al. A national survey of the prevalence of childhood overweight and obesity
© 2024 Zhang X et al. *JAMA Pediatrics.*

in Italy. *Obes Rev* 2010; **11**(1): 2-10.
981.Rapa A, Monzani A, Moia S, et al. Subclinical hypothyroidism in children and adolescents: a wide range of clinical, biochemical, and genetic factors involved. *J Clin Endocrinol Metab* 2009; **94**(7): 2414-20.
982.Caserta CA, Pendino GM, Amante A, et al. Cardiovascular risk factors, nonalcoholic fatty liver disease, and carotid artery intima-media thickness in an adolescent population in southern Italy. *Am J Epidemiol* 2010; **171**(11): 1195-202.
983.Bonaccorsi G, Baggiani L, Bassetti A, et al. Body composition assessment in a sample of eight-year-old children. *Nutrition* 2009; **25**(10): 1020-8.
984.Turconi G, Maccarini L, Bazzano R, Roggi C. Overweight and blood pressure: results from the examination of a selected group of adolescents in northern Italy. *Public Health Nutr* 2008; **11**(9): 905-13.
985.Maffeis C, Grezzani A, Perrone L, Del Giudice EM, Saggese G, Tato L. Could the savory taste of snacks be a further risk factor for overweight in children? *Journal of pediatric gastroenterology and nutrition* 2008; **46**(4): 429-37.
986.Lazzeri G, Rossi S, Pammolli A, Pilato V, Pozzi T, Giacchi MV. Underweight and overweight among children and adolescents in Tuscany (Italy). Prevalence and short-term trends. *J Prev Med Hyg* 2008; **49**(1): 13-21.
987.Guida B, Pietrobelli A, Trio R, et al. Body mass index and bioelectrical vector distribution in 8-year-old children. *Nutr Metab Cardiovasc Dis* 2008; **18**(2): 133-41.
988.Gualdi-Russo E, Albertini A, Argnani L, Celenza F, Nicolucci M, Toselli S. Weight status and body image perception in Italian children. *Journal of Human Nutrition and Dietetics* 2008; **21**(1): 39-45.
989.Fuiano N, Rapa A, Monzani A, et al. Prevalence and risk factors for overweight and obesity in a population of Italian schoolchildren: a longitudinal study. *Journal of endocrinological investigation* 2008; **31**: 979-84.
990.Vidal E, Carlin E, Driul D, Tomat M, Tenore A. A comparison study of the prevalence of overweight and obese Italian preschool children using different reference standards. *Eur J Pediatr* 2006; **165**(10): 696-700.
991.Menghetti E, Strisciuglio P, Spagnolo A, et al. Hypertension and obesity in Italian school children: The role of diet, lifestyle and family history. *Nutr Metab Cardiovasc Dis* 2015; **25**(6): 602-7.
992.Lazzeri G, Panatto D, Pammolli A, et al. Trends in overweight and obesity prevalence in Tuscan schoolchildren (2002-2012). *Public Health Nutr* 2015; **18**(17): 3078-85.
993.Geremia R, Cimadon HM, de Souza WB, Pellanda LC. Childhood overweight and obesity in a region of Italian immigration in Southern Brazil: a cross-sectional study. *Ital J Pediatr* 2015; **41**: 28.
994.Lazzeri G, Giacchi MV, Spinelli A, et al. Overweight among students aged 11–15 years and its relationship with breakfast, area of residence and parents' education: results from the Italian HBSC 2010 cross-sectional study. *Nutrition journal* 2014; **13**(1): 1-10.
995.Bo S, De Carli L, Venco E, et al. Impact of snacking pattern on overweight and obesity risk in a cohort of 11- to 13-year-old adolescents. *J Pediatr Gastroenterol Nutr* 2014; **59**(4): 465-71.
996.Sacchetti R, Ceciliani A, Garulli A, et al. Physical fitness of primary school children in relation to overweight prevalence and physical activity habits. *J Sports Sci* 2012; **30**(7): 633-40.
997.Bracale R, Milani L, Ferrara E, et al. Childhood obesity, overweight and underweight: a study in primary schools in Milan. *Eat Weight Disord* 2013; **18**(2): 183-91.
998.Petracci E, Cavrini G. The effect of weight status, lifestyle, and body image perception on health-related quality of life in children: a quantile approach. *Qual Life Res* 2013; **22**(9): 2607-15.
999.Pileggi C, Lotito F, Bianco A, Nobile CG, Pavia M. Relationship between Chronic Short Sleep Duration and Childhood Body Mass Index: A School-Based Cross-Sectional Study. *PLoS One* 2013; **8**(6): e66680.
1000. Vijaykumar S, Guna Shekhar M, Vijayakumar R. Traumatic Dental Injuries and Its Relation to Overweight among Indian School Children Living in an Urban Area. *J Clin Diagn Res* 2013; **7**(11): 2631-3.
1001. Galfo M, D'Addezio L, Censi L, Roccaldo R, Martone D. Overweight and Obesity in Italian Adolescents:
© 2024 Zhang X et al. *JAMA Pediatrics.*

Examined Prevalence and Socio-Demographic Factors. *Cent Eur J Public Health* 2016; **24**(4): 262-7.
1002. Grassi T, De Donno A, Bagordo F, et al. Socio-Economic and Environmental Factors Associated with Overweight and Obesity in Children Aged 6-8 Years Living in Five Italian Cities (the MAPEC_LIFE Cohort). *Int J Environ Res Public Health* 2016; **13**(10).
1003. Fox K, Gordon-Strachan G, Johnson A, Ashley D. Jamaican Youth Health Status 2005. *The West Indian medical journal* 2009; **58**: 533-8.
1004. Sun Y, Sekine M, Kagamimori S. Lifestyle and overweight among Japanese adolescents: the Toyama Birth Cohort Study. *J Epidemiol* 2009; **19**(6): 303-10.
1005. Tsujiguchi H, Hori D, Kambayashi Y, et al. Relationship between screen time and nutrient intake in Japanese children and adolescents: a cross-sectional observational study. *Environ Health Prev Med* 2018; **23**(1): 34.
1006. Yaguchi-Tanaka Y, Tabuchi T. Skipping Breakfast and Subsequent Overweight/Obesity in Children: A Nationwide Prospective Study of 2.5- to 13-year-old Children in Japan. *J Epidemiol* 2021; **31**(7): 417-25.
1007. Tani Y, Isumi A, Doi S, Fujiwara T. Associations of Caregiver Cooking Skills with Child Dietary Behaviors and Weight Status: Results from the A-CHILD Study. *Nutrients* 2021; **13**(12).
1008. Watanabe E, Lee JS, Kawakubo K. Associations of maternal employment and three-generation families with pre-school children's overweight and obesity in Japan. *Int J Obes (Lond)* 2011; **35**(7): 945-52.
1009. Yoshitake N, Okuda M, Sasaki S, Kunitsugu I, Hobara T. Validity of self-reported body mass index of Japanese children and adolescents. *Pediatr Int* 2012; **54**(3): 397-401.
1010. Oishi K, Aoki T, Harada T, et al. Association of Neighborhood Food Environment and Physical Activity Environment With Obesity: A Large-Scale Cross-Sectional Study of Fifth- to Ninth-Grade Children in Japan. *Inquiry* 2021; **58**: 469580211055626.
1011. Kachi Y, Otsuka T, Kawada T. Socioeconomic Status and Overweight: A Population-Based Cross-Sectional Study of Japanese Children and Adolescents. *J Epidemiol* 2015; **25**(7): 463-9.
1012. Nakano T, Sei M, Ewis AA, Munakata H, Onishi C, Nakahori Y. Tracking overweight and obesity in Japanese children; a six years longitudinal study. *J Med Invest* 2010; **57**(1-2): 114-23.
1013. Wang H, Sekine M, Chen X, Kanayama H, Yamagami T, Kagamimori S. Sib-size, birth order and risk of overweight in junior high school students in Japan: results of the Toyama Birth Cohort Study. *Prev Med* 2007; **44**(1): 45-51.
1014. Okabe Y, Itazawa T, Adachi Y, et al. Association of overweight with asthma symptoms in Japanese school children. *Pediatr Int* 2011; **53**(2): 192-8.
1015. Suzuki K, Ando D, Sato M, Tanaka T, Kondo N, Yamagata Z. The association between maternal smoking during pregnancy and childhood obesity persists to the age of 9-10 years. *J Epidemiol* 2009; **19**(3): 136-42.
1016. Yamashita M, Yorifuji T, Matsumoto N, Kubo T, Tsukahara H. Early childhood exposure to maternal smoking and obesity: A nationwide longitudinal survey in Japan. *Clin Obes* 2023; **13**(3): e12572.
1017. Shirasawa T, Ochiai H, Yoshimoto T, et al. Effects of eating dinner alone on overweight in Japanese adolescents: a cross-sectional survey. *BMC Pediatr* 2018; **18**(1): 36.
1018. Tomata Y, Tanno K, Zhang S, et al. Subjective Household Economic Status and Obesity in Toddlers: A Cross-Sectional Study of Daycare Centers in Japan. *J Epidemiol* 2019; **29**(1): 33-7.
1019. Wada K, Yamakawa M, Konishi K, et al. Associations of Cell Phone Use and Screen Viewing with Overweight in Children. *Child Obes* 2019; **15**(7): 417-25.
1020. Ochiai H, Shirasawa T, Nishimura R, et al. Changes in overweight/obesity and central obesity status from preadolescence to adolescence: a longitudinal study among schoolchildren in Japan. *BMC Public Health* 2020; **20**(1): 241.

© 2024 Zhang X et al. *JAMA Pediatrics.*

1021. Sasaki A, Yorifuji T, Iwase T, Komatsu H, Takao S. Is there any association between TV viewing and obesity in preschool children in Japan? *Acta Medica Okayama* 2010; **64**(2): 137-42.
1022. Okuda M, Sugiyama S, Kunitsugu I, et al. Use of body mass index and percentage overweight cutoffs to screen Japanese children and adolescents for obesity-related risk factors. *J Epidemiol* 2010; **20**(1): 46-53.
1023. Ochiai H, Shirasawa T, Nishimura R, et al. Relationship of body mass index to percent body fat and waist circumference among schoolchildren in Japan--the influence of gender and obesity: a population-based cross-sectional study. *BMC Public Health* 2010; **10**: 493.
1024. Shirasawa T, Ochiai H, Nanri H, et al. Trends of Underweight and Overweight/Obesity Among Japanese Schoolchildren From 2003 to 2012, Defined by Body Mass Index and Percentage Overweight Cutoffs. *J Epidemiol* 2015; **25**(7): 482-8.
1025. Shinozaki K, Okuda M, Sasaki S, Kunitsugu I, Shigeta M. Dietary Fiber Consumption Decreases the Risks of Overweight and Hypercholesterolemia in Japanese Children. *Ann Nutr Metab* 2015; **67**(1): 58-64.
1026. Ochiai H, Shirasawa T, Nishimura R, et al. Waist-to-height ratio is more closely associated with alanine aminotransferase levels than body mass index and waist circumference among population-based children: a cross-sectional study in Japan. *BMC Pediatr* 2015; **15**: 59.
1027. Murakami K, Miyake Y, Sasaki S, Tanaka K, Arakawa M. Dietary glycemic index and glycemic load in relation to risk of overweight in Japanese children and adolescents: the Ryukyus Child Health Study. *Int J Obes (Lond)* 2011; **35**(7): 925-36.
1028. Itoi A, Yamada Y, Watanabe Y, Kimura M. Physical activity, energy intake, and obesity prevalence among urban and rural schoolchildren aged 11-12 years in Japan. *Appl Physiol Nutr Metab* 2012; **37**(6): 1189-99.
1029. Mizuta A, Fujiwara T, Ojima T. Association between economic status and body mass index among adolescents: a community-based cross-sectional study in Japan. *BMC Obes* 2016; **3**: 47.
1030. Ochiai H, Shirasawa T, Nanri H, Nishimura R, Hoshino H, Kokaze A. Relationship between Eating Quickly and Overweight : A Cohort Study of Schoolchildren in Japan. *Acta Med Okayama* 2018; **72**(2): 121-8.
1031. Abu Baker NN, Daradkeh SM. Prevalence of overweight and obesity among adolescents in Irbid governorate, Jordan. *Eastern Mediterranean Health Journal* 2010; **16**(06): 657-62.
1032. Hamaideh SH, Al-Khateeb RY, Al-Rawashdeh AB. Overweight and obesity and their correlates among Jordanian adolescents. *J Nurs Scholarsh* 2010; **42**(4): 387-94.
1033. Ibrahim AI, Hawamdeh ZM, Al-Smadi JT, Ammari BA. Prevalence of overweight and obesity in urban and semi-urban Jordanian children aged 3-6 years. *Child Care Health Dev* 2008; **34**(4): 464-9.
1034. Khader Y, Irshaidat O, Khasawneh M, Amarin Z, Alomari M, Batieha A. Overweight and obesity among school children in Jordan: prevalence and associated factors. *Matern Child Health J* 2009; **13**(3): 424-31.
1035. Al-Akour NA, Khader YS, Khassawneh MY, Bawadi H. Health-related quality of life of adolescents with overweight or obesity in the north of Jordan. *Child Care Health Dev* 2012; **38**(2): 237-43.
1036. Al-Domi HA, Faqih A, Jaradat Z, Al-Dalaeen A, Jaradat S, Amarneh B. Physical activity, sedentary behaviors and dietary patterns as risk factors of obesity among Jordanian schoolchildren. *Diabetes Metab Syndr* 2019; **13**(1): 189-94.
1037. Al-Kloub MI, Al-Hassan MA, Froelicher ES. Predictors of obesity in school-aged Jordanian adolescents. *Int J Nurs Pract* 2010; **16**(4): 397-405.
1038. Tayyem RF, Al-Hazzaa HM, Abu-Mweis SS, Bawadi HA, Hammad SS, Musaiger AO. Dietary habits and physical activity levels in Jordanian adolescents attending private versus public schools. *East Mediterr Health J* 2014; **20**(7): 416-23.
1039. A OM, Al-Mannai M, Tayyem R. Prevalence of Overweight and Obesity among Female Adolescents in Jordan: A comparison between Two International Reference Standards. *Pak J Med Sci* 2013; **29**(2): 678-9.

© 2024 Zhang X et al. *JAMA Pediatrics.*

1040. Zayed AA, Beano AM, Haddadin FI, et al. Prevalence of short stature, underweight, overweight, and obesity among school children in Jordan. *BMC Public Health* 2016; **16**(1): 1040.
1041. Facchini F, Fiori G, Bedogni G, et al. Prevalence of overweight and cardiovascular risk factors in rural and urban children from Central Asia: the Kazakhstan health and nutrition examination survey. *Am J Hum Biol* 2007; **19**(6): 809-20.
1042. Adamo KB, Sheel AW, Onywera V, Waudo J, Boit M, Tremblay MS. Child obesity and fitness levels among Kenyan and Canadian children from urban and rural environments: a KIDS-CAN Research Alliance Study. *Int J Pediatr Obes* 2011; **6**(2-2): e225-32.
1043. Kyallo F, Makokha A, Mwangi AM. Overweight and obesity among public and private primary school children in Nairobi, Kenya. *Health* 2013; **05**(08): 85-90.
1044. Muthuri SK, Wachira LJ, Onywera VO, Tremblay MS. Correlates of objectively measured overweight/obesity and physical activity in Kenyan school children: results from ISCOLE-Kenya. *BMC Public Health* 2014; **14**: 436.
1045. Gewa CA. Childhood overweight and obesity among Kenyan pre-school children: association with maternal and early child nutritional factors. *Public Health Nutr* 2010; **13**(4): 496-503.
1046. Abdelalim A, Ajaj N, Al-Tmimy A, et al. Childhood obesity and academic achievement among male students in public primary schools in Kuwait. *Med Princ Pract* 2012; **21**(1): 14-9.
1047. Alqaoud N, Al-Taiar A. Trends of stunting, underweight and overweight among children aged < 5 years in Kuwait: findings from Kuwait Nutritional Surveillance System (2007-2019). *East Mediterr Health J* 2022; **28**(6): 407-17.
1048. Al-Haifi AR, Al-Awadhi BA, Al-Dashti YA, et al. Prevalence of overweight and obesity among Kuwaiti adolescents and the perception of body weight by parents or friends. *PLoS One* 2022; **17**(1): e0262101.
1049. Badr HE, Lakha SF, Pennefather P. Differences in physical activity, eating habits and risk of obesity among Kuwaiti adolescent boys and girls: a population-based study. *Int J Adolesc Med Health* 2017; **31**(1).
1050. El-Ghaziri M, Boodai S, Young D, Reilly JJ. Impact of using national v. international definitions of underweight, overweight and obesity: an example from Kuwait. *Public Health Nutr* 2011; **14**(11): 2074-8.
1051. El-Bayoumy I, Shady I, Lotfy H. Prevalence of obesity among adolescents (10 to 14 years) in Kuwait. *Asia Pac J Public Health* 2009; **21**(2): 153-9.
1052. AlRodhan Y, AlAbdeen Y, Saleh E, et al. Obesity and maternal perception: a cross-sectional study of children aged 6 to 8 years in Kuwait. *East Mediterr Health J* 2019; **25**(7): 465-72.
1053. Al-Qaoud N, Prakash P. Breastfeeding and obesity among Kuwaiti preschool children. *Med Princ Pract* 2009; **18**(2): 111-7.
1054. Al-Refaee FA, Al-Qattan SA, Jaber SM, Al-Mutairi AA, Al-Dhafiri SS, Nassar MF. The rising tide of overweight among Kuwaiti children: study from Al-Adan Hospital, Kuwait. *Med Princ Pract* 2013; **22**(6): 600-2.
1055. Musaiger AO, Al-Mannai M, Al-Lalla O, et al. Obesity among adolescents in five Arab countries; relative to gender and age. *Nutr Hosp* 2013; **28**(6): 1922-5.
1056. Karklina H, Apinis P, Kalnina L, et al. Analysis of body composition of 9- and 10-year-old children in Latvia. *Medicina (Kaunas)* 2011; **47**(10): 573-8.
1057. Nasreddine L, Naja F, Akl C, et al. Dietary, lifestyle and socio-economic correlates of overweight, obesity and central adiposity in Lebanese children and adolescents. *Nutrients* 2014; **6**(3): 1038-62.
1058. Chacar H, Salameh P. Public schools adolescents' obesity and growth curves in Lebanon. *Le Journal médical libanais The Lebanese medical journal* 2011; **59**: 80-8.
1059. Jabre P, Sikias P, Khater-Menassa B, Baddoura R, Awada H. Overweight children in Beirut: prevalence estimates and characteristics. *Child Care Health Dev* 2005; **31**(2): 159-65.
© 2024 Zhang X et al. *JAMA Pediatrics.*

1060. Fazah A, Jacob C, Moussa E, El-Hage R, Youssef H, Delamarche P. Activity, inactivity and quality of life among Lebanese adolescents. *Pediatr Int* 2010; **52**(4): 573-8.
1061. Chakar H, Salameh PR. Adolescent obesity in Lebanese private schools. *Eur J Public Health* 2006; **16**(6): 648-51.
1062. Nasreddine L, Hwalla N, Saliba A, Akl C, Naja F. Prevalence and Correlates of Preschool Overweight and Obesity Amidst the Nutrition Transition: Findings from a National Cross-Sectional Study in Lebanon. *Nutrients* 2017; **9**(3).
1063. Smetanina N, Albaviciute E, Babinska V, et al. Prevalence of overweight/obesity in relation to dietary habits and lifestyle among 7-17 years old children and adolescents in Lithuania. *BMC Public Health* 2015; **15**: 1001.
1064. Grazuleviciene R, Andrusaityte S, Uzdanaviciute I, Kudzyte J, Kevalas R, Nieuwenhuijsen MJ. The impact of tobacco smoke exposure on wheezing and overweight in 4-6-year-old children. *Biomed Res Int* 2014; **2014**: 240757.
1065. Raufi A, Konstantinova MK. Prevalence of Overweight and Obesity in Children: Variation in Different Ethnicities, Age, and Sex in North Macedonia. *Pril (Makedon Akad Nauk Umet Odd Med Nauki)* 2022; **43**(2): 23-31.
1066. Gontarev S, Kalac R, Velickovska L, Stojmanovska D, Misovski A, Milenkovski J. Health-related physical fitness of normal, stunted and overweight children aged 6-14 years in Macedonia. *Nutr Hosp* 2018; **35**(5): 1208-14.
1067. Myrtaj N, Maliqi A, Gontarev S, Kalac R, Georgiev G, Stojanoska BB. Antropometría y Composición Corporal de Adolescentes en Macedonia. *International journal of morphology* 2018; **36**(4): 1398-406.
1068. Zulfarina MS, Sharif R, Sharkawi AM, Mokhtar SA, Shuid AN, Naina-Mohamed I. Quality of energy intake in Malaysian adolescents: prevalence, characteristics, determinants and impact of implausible reporters. *Public Health Nutr* 2023; **26**(1): 122-31.
1069. Lai WK, Mohd Sidik S, Lekhraj R, Gan WY, Ismail SIF. Prevalence and Predictors of Overweight and Obesity Among Adolescents in Seremban, Negeri Sembilan, Malaysia. *Cureus* 2022; **14**(1): e21795.
1070. Al-Sadat N, Majid HA, Sim PY, et al. Vitamin D deficiency in Malaysian adolescents aged 13 years: findings from the Malaysian Health and Adolescents Longitudinal Research Team study (MyHeARTs). *BMJ Open* 2016; **6**(8): e010689.
1071. Khor GL, Chee WS, Shariff ZM, et al. High prevalence of vitamin D insufficiency and its association with BMI-for-age among primary school children in Kuala Lumpur, Malaysia. *BMC Public Health* 2011; **11**: 95.
1072. Adeyemi AJ, Rohani JM, AbdulRani MR, Schumacher U. Comparing body composition measures among Malaysian primary school children. *Cogent Medicine* 2014; **1**(1).
1073. Ahmad A, Zulaily N, Abdul Manan NS, et al. Body weight status of school adolescents in Terengganu, Malaysia: a population baseline study. *BMC Public Health* 2017; **17**(1): 9.
1074. Tee ES, Nurliyana AR, Norimah AK, et al. Breakfast consumption among Malaysian primary and secondary school children and relationship with body weight status - Findings from the MyBreakfast Study. *Asia Pac J Clin Nutr* 2018; **27**(2): 421-32.
1075. Kee CC, Lim KH, Sumarni MG, et al. Validity of self-reported weight and height: a cross-sectional study among Malaysian adolescents. *BMC Med Res Methodol* 2017; **17**(1): 85.
1076. Chee Cheong K, Yoon Ling C, Kuang Hock L, et al. Association between Availability of Neighborhood Fast Food Outlets and Overweight Among 5(-)18 Year-Old Children in Peninsular Malaysia: A Cross-Sectional Study. *Int J Environ Res Public Health* 2019; **16**(4).
1077. Moy FM, Gan CY, Zaleha MK. Body mass status of school children and adolescents in Kuala Lumpur, Malaysia. *Asia Pac J Clin Nutr* 2004; **13**(4): 324-9.
1078. Alagappan M, Rampal L, Zalilah MS. Prevalence of overweight/obesity and its associated factors among secondary school students in semi urban area in Malaysia. *Med J Malaysia* 2019; **74**(6): 513-20.

© 2024 Zhang X et al. *JAMA Pediatrics.*

1079. Whye Lian C. Obesity-associated Hypertension among Primary School Children in Sarawak: A Cross-sectional Study. *Medicine & Health* 2019; **14**(2): 121-31.
1080. Tan AKG, Yen ST, Fang X, Chiang FS. Body weight and physical activity of adolescents in Malaysia. *Int Health* 2019; **11**(2): 150-8.
1081. Muhammad NA, Omar K, Shah SA. Parental perception of their children's weight status, and its association with their nutrition and obesity knowledge. *Asia Pac J Clin Nutr* 2008; **17**(4): 597.
1082. Zalilah M, Mirnalini K, Khor G, Merlin A, Bahaman A, Norimah K. Estimates and distribution of body mass index in a sample of Malaysian adolescents. *Med J Malaysia* 2006; **61**(1): 48-58.
1083. Sumarni Mohd G, Muhammad Amir K, Ibrahim Md S, Mohd Rodi I, Izzuna Mudla MG, Nurziyana I. Obesity among schoolchildren in Kuala Selangor: a cross-sectional study. *Trop Biomed* 2006; **23**(2): 148-54.
1084. Woon FC, Chin YS, Mohd Nasir MT. Association between behavioural factors and BMI-for-age among early adolescents in Hulu Langat district, Selangor, Malaysia. *Obes Res Clin Pract* 2015; **9**(4): 346-56.
1085. Loh DA, Moy FM, Zaharan NL, Mohamed Z. Disparities in health-related quality of life among healthy adolescents in a developing country - the impact of gender, ethnicity, socio-economic status and weight status. *Child Care Health Dev* 2015; **41**(6): 1216-26.
1086. Kaartina S, Chin YS, Fara Wahida R, et al. Adolescent self-report and parent proxy-report of health-related quality of life: an analysis of validity and reliability of PedsQL 4.0 among a sample of Malaysian adolescents and their parents. *Health Qual Life Outcomes* 2015; **13**: 44.
1087. Zainuddin AA, Manickam MA, Baharudin A, et al. Self-perception of body weight status and weight control practices among adolescents in Malaysia. *Asia Pac J Public Health* 2014; **26**(5 Suppl): 18S-26S.
1088. Su TT, Sim PY, Nahar AM, et al. Association between self-reported physical activity and indicators of body composition in Malaysian adolescents. *Prev Med* 2014; **67**: 100-5.
1089. Rezali FW, Chin YS, Mohd Yusof BN. Obesity-related behaviors of Malaysian adolescents: a sample from Kajang district of Selangor state. *Nutr Res Pract* 2012; **6**(5): 458-65.
1090. Naidu BM, Mahmud SZ, Ambak R, et al. Overweight among primary school-age children in Malaysia. *Asia Pac J Clin Nutr* 2013; **22**(3): 408-15.
1091. Poh BK, Wong JE, Norimah AK, Deurenberg P. Differences in Body Build in Children of Different Ethnic Groups and their Impact on the Prevalence of Stunting, Thinness, Overweight, and Obesity. *Food Nutr Bull* 2016; **37**(1): 3-13.
1092. Shariff ZM, Lin KG, Sariman S, et al. Higher Dietary Energy Density is Associated with Stunting but not Overweight and Obesity in a Sample of Urban Malaysian Children. *Ecol Food Nutr* 2016; **55**(4): 378-89.
1093. Partap U, Young EH, Allotey P, Sandhu MS, Reidpath DD. The Use of Different International References to Assess Child Anthropometric Status in a Malaysian Population. *J Pediatr* 2017; **190**: 63-8 e1.
1094. Cazzaniga E, Orlando A, Terenzio A, et al. Health Status and Nutritional Habits in Maldives Pediatric Population: A Cross-Sectional Study. *Int J Environ Res Public Health* 2022; **19**(23).
1095. Decelis A, Jago R, Fox KR. Physical activity, screen time and obesity status in a nationally representative sample of Maltese youth with international comparisons. *BMC Public Health* 2014; **14**: 664.
1096. Decelis A, Fox K, Jago R. Prevalence of obesity among 10-11-year-old Maltese children using four established standards. *Pediatr Obes* 2013; **8**(5): e54-8.
1097. Caleyachetty R, Rudnicka AR, Echouffo-Tcheugui JB, Siegel KR, Richards N, Whincup PH. Prevalence of overweight, obesity and thinness in 9-10 year old children in Mauritius. *Global Health* 2012; **8**: 28.
1098. Fokeena WB, Jeewon R. Is there an association between socioeconomic status and body mass index among adolescents in Mauritius? *ScientificWorldJournal* 2012; **2012**: 750659.
1099. Shamah-Levy T, Cuevas-Nasu L, Gaona-Pineda EB, Valenzuela-Bravo DG, Mendez Gomez-Humaran I,

© 2024 Zhang X et al. *JAMA Pediatrics.*

Avila-Arcos MA. Childhood obesity in Mexico: Influencing factors and prevention strategies. *Front Public Health* 2022; **10**: 949893.
1100. Brambila-Paz C, Hernandez-Angeles DF, Silverio-Murillo A, Rodriguez-Tirado A. Family Factors Affecting the Transition of Children from Normal Weight to Obesity in Mexico. *Child Obes* 2022; **18**(2): 112-9.
1101. Aburto TC, Cantoral A, Hernandez-Barrera L, Carriquiry AL, Rivera JA. Usual Dietary Energy Density Distribution Is Positively Associated with Excess Body Weight in Mexican Children. *J Nutr* 2015; **145**(7): 1524-30.
1102. Aguilera-Galaviz LA, Hernández-Vázquez B, Frausto-Esparza S, Díaz-Rosas CY, Gaitán-Fonseca C. Nutritional and oral health conditions in high school students. *Odovtos-International Journal of Dental Sciences* 2019; **21**(2): 83-93.
1103. Garcia Perez A, Barrera Ortega CC, Gonzalez-Aragon Pineda AE, Villanueva Gutierrez T, Perez Perez NG, Calderon Uriostegui D. An inverse relationship between obesity and dental caries in Mexican schoolchildren: a cross-sectional study. *Public Health* 2020; **180**: 163-7.
1104. Irigoyen-Camacho ME, Sanchez-Perez L, Molina-Frechero N, Velazquez-Alva C, Zepeda-Zepeda M, Borges-Yanez A. The relationship between body mass index and body fat percentage and periodontal status in Mexican adolescents. *Acta Odontol Scand* 2014; **72**(1): 48-57.
1105. Lara-Capi C, Cagetti MG, Cocco F, Lingstrom P, Garcia-Godoy F, Campus G. Effect of body weight and behavioural factors on caries severity in Mexican rural and urban adolescents. *Int Dent J* 2018; **68**(3): 190-6.
1106. Patino-Marin N, Zavala-Alonso NV, Martinez-Castanon GA, et al. Association between dental hygiene, gingivitis and overweight or the risk of overweight in primary teeth of 4- and 5-year-old preschoolers in Mexico. *Int J Dent Hyg* 2018; **16**(3): 411-8.
1107. Sanchez-Perez L, Irigoyen ME, Zepeda M. Dental caries, tooth eruption timing and obesity: a longitudinal study in a group of Mexican schoolchildren. *Acta Odontol Scand* 2010; **68**(1): 57-64.
1108. Serrano-Pina R, Aguilar-Ayala FJ, Scougall-Vilchis RJ, Trujillo-Guiza ML, Mendieta-Zeron H. Prevalence of Obesity in Elementary School Children and its Association with Dental Caries. *Oral Health Prev Dent* 2020; **18**(1): 35-42.
1109. Vazquez-Nava F, Vazquez-Rodriguez EM, Saldivar-Gonzalez AH, Lin-Ochoa D, Martinez-Perales GM, Joffre-Velazquez VM. Association between obesity and dental caries in a group of preschool children in Mexico. *J Public Health Dent* 2010; **70**(2): 124-30.
1110. Avila-Ortiz MN, Castro-Sanchez AE, Zambrano-Moreno A. Mexican mothers' perceptions of their child's body weight. *Health Soc Care Community* 2017; **25**(2): 569-77.
1111. Bacardi-Gascon M, Jimenez-Cruz A, Jones E, Velasquez Perez I, Loaiza Martinez JA. Trends of overweight and obesity among children in Tijuana, Mexico. *Ecol Food Nutr* 2009; **48**(3): 226-36.
1112. Bacardi-Gascon M, Perez-Morales ME, Jimenez-Cruz A. A six month randomized school intervention and an 18-month follow-up intervention to prevent childhood obesity in Mexican elementary schools. *Nutr Hosp* 2012; **27**(3): 755-62.
1113. Lopez-Gonzalez D, Partida-Gaytan A, Wells JC, et al. Obesogenic Lifestyle and Its Influence on Adiposity in Children and Adolescents, Evidence from Mexico. *Nutrients* 2020; **12**(3).
1114. Del-Rio-Navarro BE, Velazquez-Monroy O, Lara-Esqueda A, et al. Obesity and metabolic risks in children. *Arch Med Res* 2008; **39**(2): 215-21.
1115. Gómez-Díaz RA, Martínez-Hernández AJ, Aguilar-Salinas CA, et al. Percentile distribution of the waist circumference among Mexican pre-adolescents of a primary school in Mexico City. *Diabetes Obes Metab* 2005; **7**(6): 716-21.
1116. Brewis A. Biocultural aspects of obesity in young Mexican schoolchildren. *Am J Hum Biol* 2003; **15**(3): 446-60.

© 2024 Zhang X et al. *JAMA Pediatrics.*

1117. Vergara-Castaneda A, Castillo-Martinez L, Colin-Ramirez E, Orea-Tejeda A. Overweight, obesity, high blood pressure and lifestyle factors among Mexican children and their parents. *Environ Health Prev Med* 2010; **15**(6): 358-66.
1118. Jimenez-Cruz A, Bacardi-Gascon M, Pichardo-Osuna A, Mandujano-Trujillo Z, Castillo-Ruiz O. Infant and toddlers' feeding practices and obesity amongst low-income families in Mexico. *Asia Pac J Clin Nutr* 2010; **19**(3): 316-23.
1119. Varela-Silva MI, Azcorra H, Dickinson F, Bogin B, Frisancho AR. Influence of maternal stature, pregnancy age, and infant birth weight on growth during childhood in Yucatan, Mexico: a test of the intergenerational effects hypothesis. *Am J Hum Biol* 2009; **21**(5): 657-63.
1120. Macías-Rosales R, Vásquez-Garibay EM, Larrosa-Haro A, Rojo-Chávez M, Bernal-Virgen A, Romo-Rubio H. Secondary malnutrition and overweight in a pediatric referral hospital: associated factors. *J Pediatr Gastroenterol Nutr* 2009; **48**(2): 226-32.
1121. Flores-Huerta S, Klunder-Klunder M, Reyes de la Cruz L, Santos JI. Increase in body mass index and waist circumference is associated with high blood pressure in children and adolescents in Mexico city. *Arch Med Res* 2009; **40**(3): 208-15.
1122. Ortiz-Hernandez L, Lopez Olmedo NP, Genis Gomez MT, Melchor Lopez DP, Valdes Flores J. Application of body mass index to schoolchildren of Mexico City. *Ann Nutr Metab* 2008; **53**(3-4): 205-14.
1123. Basaldua N, Chiquete E. Common predictors of excessive adiposity in children from a region with high prevalence of overweight. *Ann Nutr Metab* 2008; **52**(3): 227-32.
1124. Villa-Caballero L, Caballero-Solano V, Chavarria-Gamboa M, et al. Obesity and socioeconomic status in children of Tijuana. *Am J Prev Med* 2006; **30**(3): 197-203.
1125. RODRÍGUEZ-FUENTES G, ARCEGA-CABRERA F, FARGHER LF. Plasma and erythrocyte cholinesterase activities in children from Yucatan, Mexico: relationship with anthropometry and obesity. *Asian J Pharm Clin Res* 2015; **8**(3): 224-8.
1126. Lopez-Barron RG, Jimenez-Cruz A, Bacardi-Gascon M. Modifiable environmental obesity risk factors among elementary school children in a Mexico-us border city. *Nutr Hosp* 2015; **31**(5): 2047-53.
1127. Flores YN, Shaibi GQ, Morales LS, et al. Perceived health status and cardiometabolic risk among a sample of youth in Mexico. *Qual Life Res* 2015; **24**(8): 1887-97.
1128. Datta Banik S, Castillo T, Rodriguez L, Dickinson F. Body fatness in relation to physical activity and selected socioeconomic parameters of adolescents aged 15-17 years in Merida, Yucatan. *Ann Hum Biol* 2014; **41**(6): 497-505.
1129. Datta Banik S, Andrade Olalde AC, Rodriguez L, Dickinson F. The effect of socioeconomic indicators and macronutrient intake rate on body composition in adolescents 12 to 16 years old in Merida, Yucatan. *Anthropol Anz* 2014; **71**(4): 347-68.
1130. Barrera LH, Rothenberg SJ, Barquera S, Cifuentes E. The Toxic Food Environment Around Elementary Schools and Childhood Obesity in Mexican Cities. *Am J Prev Med* 2016; **51**(2): 264-70.
1131. Chavez Caraza KL, Rodriguez de Ita J, Santos Guzman J, Segovia Aguirre JG, Altamirano Montealvo DC, Matias Barrios VM. Altered perception of the nutritional status of preschoolers by their parents: A risk factor for overweight and obesity. *Arch Argent Pediatr* 2016; **114**(3): 237-142.
1132. Meza Peña C, Pompa Guajardo EG. An approach to the study of obesity and depression in a sample of Mexican adolescents in northern Mexico. *CIENCIA ergo sum* 2018; **25**(3): 1-9.
1133. Ramirez-Jimenez R, Martinez-Salazar MF, Almenares-Lopez D, Yanez-Estrada L, Monroy-Noyola A. Relationship Between Paraoxonase-1 and Butyrylcholinesterase Activities and Nutritional Status in Mexican Children. *Metab Syndr Relat Disord* 2018; **16**(2): 90-6.
© 2024 Zhang X et al. *JAMA Pediatrics.*

1134. Martinovic M, Belojevic G, Evans GW, et al. Prevalence of and contributing factors for overweight and obesity among Montenegrin schoolchildren. *Eur J Public Health* 2015; **25**(5): 833-9.
1135. Mehdad S, Benaich S, Hamdouchi AE, et al. Association between overweight and anemia in Moroccan adolescents: a cross-sectional study. *Pan Afr Med J* 2022; **41**: 156.
1136. el Moussaoui S, Kaoutar K, Chetoui A, et al. Prevalence of overweight/obesity and associated factors in children underfive years – of age in Marrakesh, Morocco. 2022.
1137. Achouri I, Aboussaleh Y, Sbaibi R, Ahami A. Anthropometry, food consumption and iron deficiency anemia, among primary school children (6-15 years) in Kenitra city (North-Western Morocco). *Pan Afr Med J* 2021; **38**: 374.
1138. Cherkaoui Dekkaki I, Mouane N, Ettair S, Meskini T, Bouklouze A, Barkat A. Prevalence of obesity and overweight in children: a study in government primary schools in Rabat, Morocco. *Arch Med Res* 2011; **42**(8): 703-8.
1139. El Kabbaoui M, Chda A, Bousfiha A, Aarab L, Bencheikh R, Tazi A. Prevalence of and risk factors for overweight and obesity among adolescents in Morocco. *East Mediterr Health J* 2018; **24**(6): 512-21.
1140. Qiao Y, Ma J, Wang Y, et al. Birth weight and childhood obesity: a 12-country study. *Int J Obes Suppl* 2015; **5**(Suppl 2): S74-9.
1141. Bishwajit G, Yaya S. Overweight and obesity among under-five children in South Asia. *Child and Adolescent Obesity* 2020; **3**(1): 105-21.
1142. Stival C, Lugo A, Barone L, et al. Prevalence and Correlates of Overweight, Obesity and Physical Activity in Italian Children and Adolescents from Lombardy, Italy. *Nutrients* 2022; **14**(11).
1143. Moschonis G, Siopis G, Anastasiou C, et al. Prevalence of Childhood Obesity by Country, Family Socio-Demographics, and Parental Obesity in Europe: The Feel4Diabetes Study. *Nutrients* 2022; **14**(9).
1144. Ferrari G, Drenowatz C, Kovalskys I, et al. Walking and cycling, as active transportation, and obesity factors in adolescents from eight countries. *BMC Pediatr* 2022; **22**(1): 510.
1145. Collings PJ, Grontved A, Jago R, et al. Cross-sectional and prospective associations of sleep duration and bedtimes with adiposity and obesity risk in 15 810 youth from 11 international cohorts. *Pediatr Obes* 2022; **17**(4): e12873.
1146. Ho FK, Rao N, Tung KTS, et al. Association of Early Nutritional Status With Child Development in the Asia Pacific Region. *JAMA Netw Open* 2021; **4**(12): e2139543.
1147. Peltzer K, Pengpid S. Overweight and obesity and associated factors among school-aged adolescents in Ghana and Uganda. *Int J Environ Res Public Health* 2011; **8**(10): 3859-70.
1148. Vrijheid M, Fossati S, Maitre L, et al. Early-Life Environmental Exposures and Childhood Obesity: An Exposome-Wide Approach. *Environ Health Perspect* 2020; **128**(6): 67009.
1149. Roy M, Haszard JJ, Savage JS, et al. Bedtime, body mass index and obesity risk in preschool-aged children. *Pediatr Obes* 2020; **15**(9): e12650.
1150. Ekelund U, Sardinha LB, Anderssen SA, et al. Associations between objectively assessed physical activity and indicators of body fatness in 9- to 10-y-old European children: a population-based study from 4 distinct regions in Europe (the European Youth Heart Study). *Am J Clin Nutr* 2004; **80**(3): 584-90.
1151. McCabe MP, Ricciardelli L, Waqa G, Goundar R, Fotu K. Body image and body change strategies among adolescent males and females from Fiji, Tonga and Australia. *Body Image* 2009; **6**(4): 299-303.
1152. te Velde SJ, De Bourdeaudhuij I, Thorsdottir I, et al. Patterns in sedentary and exercise behaviors and associations with overweight in 9-14-year-old boys and girls--a cross-sectional study. *BMC Public Health* 2007; **7**: 16.
1153. Blaženčić-Mladenović V, Glibetic M, Tepšić J, et al. How much are children and their parents objective about the children's eating behaviour and body composition? *European Eating Disorders Review* 2006; **14**(4): 248-55.

1154. Olaya B, Moneta MV, Pez O, et al. Country-level and individual correlates of overweight and obesity among primary school children: a cross-sectional study in seven European countries. *BMC Public Health* 2015; **15**: 475.
1155. Tsitsika AK, Andrie EK, Psaltopoulou T, et al. Association between problematic internet use, socio-demographic variables and obesity among European adolescents. *Eur J Public Health* 2016; **26**(4): 617-22.
1156. Qiao Y, Zhang T, Liu H, et al. Joint association of birth weight and physical activity/sedentary behavior with obesity in children ages 9-11 years from 12 countries. *Obesity (Silver Spring)* 2017; **25**(6): 1091-7.
1157. Acharya B, Chauhan H, Bala I, Kaphle H, Thapa S. Body image satisfaction, weight perception and knowledge of obesity among adolescents in Kaski district, Nepal. *TAF Preventive Medicine Bulletin* 2016; **15**(5).
1158. Piryani S, Baral KP, Pradhan B, Poudyal AK, Piryani RM. Overweight and its associated risk factors among urban school adolescents in Nepal: a cross-sectional study. *BMJ Open* 2016; **6**(5): e010335.
1159. Bhattarai S, Bhusal CK. Prevalence and associated factors of malnutrition among school going adolescents of Dang district, Nepal. *AIMS Public Health* 2019; **6**(3): 291-306.
1160. Karki A, Shrestha A, Subedi N. Prevalence and associated factors of childhood overweight/obesity among primary school children in urban Nepal. *BMC Public Health* 2019; **19**(1): 1055.
1161. Koirala M, Khatri RB, Khanal V, Amatya A. Prevalence and factors associated with childhood overweight/obesity of private school children in Nepal. *Obes Res Clin Pract* 2015; **9**(3): 220-7.
1162. Gurung TR, Vl NG. Overweight and Obesity among the Adolescent School Students in Belgaum City. *JNMA J Nepal Med Assoc* 2014; **52**(194): 791-5.
1163. Pandey A, Sapkota S. Prevalence and knowledge on obesity among school going adolescents of Kaski, Nepal. *Journal of Nepal Paediatric Society* 2018; **38**(2): 63-8.
1164. de Greeff JW, Hartman E, Mullender-Wijnsma MJ, Bosker RJ, Doolaard S, Visscher C. Effect of Physically Active Academic Lessons on Body Mass Index and Physical Fitness in Primary School Children. *J Sch Health* 2016; **86**(5): 346-52.
1165. Veldhuis L, Vogel I, Renders CM, et al. Behavioral risk factors for overweight in early childhood; the 'Be active, eat right' study. *Int J Behav Nutr Phys Act* 2012; **9**: 74.
1166. Jabakhanji SB, Boland F, Ward M, Biesma R. Prevalence of early childhood obesity in Ireland: Differences over time, between sexes and across child growth criteria. *Pediatr Obes* 2022; **17**(11): e12953.
1167. Vinke PC, Luitjens M, Blijleven KA, Navis G, Kromhout D, Corpeleijn E. Nutrition beyond the first 1000 days: diet quality and 7-year change in BMI and overweight in 3-year old children from the Dutch GECKO Drenthe birth cohort. *J Dev Orig Health Dis* 2021; **12**(6): 933-9.
1168. Croezen S, Visscher TL, Ter Bogt NC, Veling ML, Haveman-Nies A. Skipping breakfast, alcohol consumption and physical inactivity as risk factors for overweight and obesity in adolescents: results of the E-MOVO project. *Eur J Clin Nutr* 2009; **63**(3): 405-12.
1169. Drukker M, Wojciechowski F, Feron FJ, Mengelers R, Van Os J. A community study of psychosocial functioning and weight in young children and adolescents. *Int J Pediatr Obes* 2009; **4**(2): 91-7.
1170. Jansen PW, Roza SJ, Jaddoe VW, et al. Children's eating behavior, feeding practices of parents and weight problems in early childhood: results from the population-based Generation R Study. *Int J Behav Nutr Phys Act* 2012; **9**: 130.
1171. de Wilde JA, van Dommelen P, Middelkoop BJ, Verkerk PH. Trends in overweight and obesity prevalence in Dutch, Turkish, Moroccan and Surinamese South Asian children in the Netherlands. *Arch Dis Child* 2009; **94**(10): 795-800.
1172. de Jong E, Visscher TL, HiraSing RA, Heymans MW, Seidell JC, Renders CM. Association between TV viewing, computer use and overweight, determinants and competing activities of screen time in 4- to 13-year-old children. *Int J Obes (Lond)* 2013; **37**(1): 47-53.

© 2024 Zhang X et al. *JAMA Pediatrics.*

1173. Jansen W, van de Looij-Jansen PM, Ferreira I, de Wilde EJ, Brug J. Differences in measured and self-reported height and weight in Dutch adolescents. *Ann Nutr Metab* 2006; **50**(4): 339-46.
1174. Steur M, Smit HA, Schipper CM, et al. Predicting the risk of newborn children to become overweight later in childhood: the PIAMA birth cohort study. *Int J Pediatr Obes* 2011; **6**(2-2): e170-8.
1175. Akbulut G, Yildirim M, Sanlier N, et al. Comparison of energy balance-related behaviours and measures of body composition between Turkish adolescents in Turkey and Turkish immigrant adolescents in the Netherlands. *Public Health Nutr* 2014; **17**(12): 2692-9.
1176. Mbakwa CA, Scheres L, Penders J, Mommers M, Thijs C, Arts IC. Early Life Antibiotic Exposure and Weight Development in Children. *J Pediatr* 2016; **176**: 105-13 e2.
1177. Jansen W, van de Looij-Jansen PM, de Wilde EJ, Brug J. Feeling fat rather than being fat may be associated with psychological well-being in young dutch adolescents. *J Adolesc Health* 2008; **42**(2): 128-36.
1178. Scholtens S, Wijga AH, Seidell JC, et al. Overweight and changes in weight status during childhood in relation to asthma symptoms at 8 years of age. *J Allergy Clin Immunol* 2009; **123**(6): 1312-8 e2.
1179. Timmermans SH, Mommers M, Gubbels JS, et al. Maternal smoking during pregnancy and childhood overweight and fat distribution: the KOALA Birth Cohort Study. *Pediatr Obes* 2014; **9**(1): e14-25.
1180. Vrijkotte TGM, Oostvogels A, Stronks K, Roseboom TJ, Hof MHP. Growth patterns from birth to overweight at age 5-6 years of children with various backgrounds in socioeconomic status and country of origin: the ABCD study. *Pediatr Obes* 2020; **15**(8): e12635.
1181. de Wilde JA, Eilander M, Middelkoop BJC. Effect of neighbourhood socioeconomic status on overweight and obesity in children 2-15 years of different ethnic groups. *Eur J Public Health* 2019; **29**(4): 796-801.
1182. Oude Luttikhuis HG, Stolk RP, Sauer PJ. How do parents of 4- to 5-year-old children perceive the weight of their children? *Acta Paediatr* 2010; **99**(2): 263-7.
1183. Jansen W, Mackenbach JP, Joosten-van Zwanenburg E, Brug J. Weight status, energy-balance behaviours and intentions in 9-12-year-old inner-city children. *J Hum Nutr Diet* 2010; **23**(1): 85-96.
1184. Bossink-Tuna HN, L'Hoir MP, Beltman M, Boere-Boonekamp MM. Parental perception of weight and weight-related behaviour in 2- to 4-year-old children in the eastern part of the Netherlands. *Eur J Pediatr* 2009; **168**(3): 333-9.
1185. Snoek HM, van Strien T, Janssens JM, Engels RC. Emotional, external, restrained eating and overweight in Dutch adolescents. *Scand J Psychol* 2007; **48**(1): 23-32.
1186. Scholtens S, Brunekreef B, Visscher TL, et al. Reported versus measured body weight and height of 4-year-old children and the prevalence of overweight. *Eur J Public Health* 2007; **17**(4): 369-74.
1187. Jansen W, Brug J. Parents often do not recognize overweight in their child, regardless of their socio-demographic background. *Eur J Public Health* 2006; **16**(6): 645-7.
1188. Wijtzes AI, Bouthoorn SH, Jansen W, et al. Sedentary behaviors, physical activity behaviors, and body fat in 6-year-old children: the generation R study. *Int J Behav Nutr Phys Act* 2014; **11**: 96.
1189. Bere E, Seiler S, Eikemo TA, Oenema A, Brug J. The association between cycling to school and being overweight in Rotterdam (The Netherlands) and Kristiansand (Norway). *Scand J Med Sci Sports* 2011; **21**(1): 48-53.
1190. Schwiebbe L, van Rest J, Verhagen E, Visser RW, Holthe JK, Hirasing RA. Childhood obesity in the Caribbean. *West Indian Med J* 2011; **60**(4): 442-5.
1191. de Jong E, Stocks T, Visscher TL, HiraSing RA, Seidell JC, Renders CM. Association between sleep duration and overweight: the importance of parenting. *Int J Obes (Lond)* 2012; **36**(10): 1278-84.
1192. Veldwijk J, Proper KI, Hoeven-Mulder HB, Bemelmans WJ. The prevalence of physical, sexual and mental abuse among adolescents and the association with BMI status. *BMC Public Health* 2012; **12**: 840.
1193. Willeboordse M, van den Bersselaar DL, van de Kant KD, Muris JW, van Schayck OC, Dompeling E. Sex

© 2024 Zhang X et al. *JAMA Pediatrics.*

differences in the relationship between asthma and overweight in Dutch children: a survey study. *PLoS One* 2013; **8**(10): e77574.
1194. Leppers I, Tiemeier H, Swanson SA, et al. Agreement between Weight Status and Perceived Body Size and the Association with Body Size Satisfaction in Children. *Obesity (Silver Spring)* 2017; **25**(11): 1956-64.
1195. Ridder MAM, Koning M, Visscher TLS, Hirasing RA, Seidell JC, Renders CM. Energy Balance-Related Behavior and Anthropometric Measures Among Adolescents Across Three Educational Levels: A Cross-Sectional Study in Dutch Schools. *Health Educ Behav* 2018; **45**(3): 349-58.
1196. Utter J, Scragg R, Schaaf D. Associations between television viewing and consumption of commonly advertised foods among New Zealand children and young adolescents. *Public Health Nutr* 2006; **9**(5): 606-12.
1197. Hobbs M, Biddle SJH, Kingsnorth AP, et al. Investigating the Association Between Child Television Viewing and Measured Child Adiposity Outcomes in a Large Nationally Representative Sample of New Zealanders: A Cross-Sectional Study. *J Phys Act Health* 2021; **18**(5): 524-32.
1198. Aung YM, Jelleyman T, Ameratunga S, Tin Tin S. Body mass index and dental caries in New Zealand pre-school children: A population-based study. *J Paediatr Child Health* 2021; **57**(9): 1432-7.
1199. Chelimo C, Camargo CA, Jr., Morton SMB, Grant CC. Association of Repeated Antibiotic Exposure Up to Age 4 Years With Body Mass at Age 4.5 Years. *JAMA Netw Open* 2020; **3**(1): e1917577.
1200. Rockell JE, Green TJ, Skeaff CM, et al. Season and ethnicity are determinants of serum 25-hydroxyvitamin D concentrations in New Zealand children aged 5-14 y. *J Nutr* 2005; **135**(11): 2602-8.
1201. Gordon FK, Ferguson EL, Toafa V, et al. High levels of childhood obesity observed among 3- to 7-year-old New Zealand Pacific children is a public health concern. *J Nutr* 2003; **133**(11): 3456-60.
1202. Utter J, Denny S, Crengle S, et al. Overweight among New Zealand adolescents: associations with ethnicity and deprivation. *Int J Pediatr Obes* 2010; **5**(6): 461-6.
1203. Quigg R, Gray A, Reeder AI, Holt A, Waters DL. Using accelerometers and GPS units to identify the proportion of daily physical activity located in parks with playgrounds in New Zealand children. *Prev Med* 2010; **50**(5-6): 235-40.
1204. Rajput N, Tuohy P, Mishra S, Smith A, Taylor B. Overweight and obesity in 4-5-year-old children in New Zealand: results from the first 4 years (2009-2012) of the B4School Check programme. *J Paediatr Child Health* 2015; **51**(3): 334-43.
1205. Adeomi AA, Adelusi IO, Adedeji PO, Awofeso AE, Oroleye OO, Gbadegesin DL. Nutritional status and Cardiometabolic health among adolescents; findings from southwestern Nigeria. *BMC Nutr* 2019; **5**: 45.
1206. Adetunji AE, Adeniran KA, Olomu SC, et al. Socio-demographic factors associated with overweight and obesity among primary school children in semi-urban areas of mid-western Nigeria. *PLoS One* 2019; **14**(4): e0214570.
1207. Maruf FA, Aronu U, Chukwuegbu K, Aronu AE. Influence of gender on prevalence of overweight and obesity in Nigerian schoolchildren and adolescents. *Tanzan J Health Res* 2013; **15**(4): 247-51.
1208. Ene-Obong H, Ibeanu V, Onuoha N, Ejekwu A. Prevalence of overweight, obesity, and thinness among urban school-aged children and adolescents in southern Nigeria. *Food Nutr Bull* 2012; **33**(4): 242-50.
1209. Omigbodun OO, Adediran KI, Akinyemi JO, Omigbodun AO, Adedokun BO, Esan O. Gender and rural-urban differences in the nutritional status of in-school adolescents in south-western Nigeria. *J Biosoc Sci* 2010; **42**(5): 653-76.
1210. Opara D, Ikpeme E, Ekanem U. Prevalence of stunting, underweight and obesity in school aged children in Uyo, Nigeria. *Pakistan Journal of Nutrition* 2010; **9**(5): 459-66.
1211. Senbanjo IO, Oshikoya KA. Physical activity and body mass index of school children and adolescents in Abeokuta, Southwest Nigeria. *World J Pediatr* 2010; **6**(3): 217-22.
© 2024 Zhang X et al. *JAMA Pediatrics.*

1212. Goon DT, Toriola AL, Shaw BS. Screening for body-weight disorders in Nigerian children using contrasting definitions. *Obes Rev* 2010; **11**(7): 508-15.

1213. Fetuga MB, Ogunlesi TA, Adekanmbi AF, Alabi AD. Growth pattern of schoolchildren in Sagamu, Nigeria using the CDC standards and 2007 WHO standards. *Indian Pediatr* 2011; **48**(7): 523-8.

1214. Wariri O, Akhimienho KI, Alhassan JAK, et al. Population and Individual-Level Double Burden of Malnutrition Among Adolescents in Two Emerging Cities in Northern and Southern Nigeria: A Comparative Cross-Sectional Study. *Ann Glob Health* 2020; **86**(1): 153.

1215. Mezie-Okoye M, Alex-Hart B. Overweight and Obesity among Preschool Children in Port Harcourt, Nigeria. *Pakistan Journal of Nutrition* 2015; **14**: 209-13.

1216. Nwizu SE, Njokanma OF, Okoromah CA, David NA. Relationship between bioelectrical impedance analysis and body mass index in adolescent urban Nigerians. *West Afr J Med* 2011; **30**(2): 99-103.

1217. Musa DI, Toriola AL, Monyeki MA, Lawal B. Prevalence of childhood and adolescent overweight and obesity in Benue State, Nigeria. *Trop Med Int Health* 2012; **17**(11): 1369-75.

1218. Oduwole AA, Ladapo TA, Fajolu IB, Ekure EN, Adeniyi OF. Obesity and elevated blood pressure among adolescents in Lagos, Nigeria: a cross-sectional study. *BMC Public Health* 2012; **12**: 616.

1219. Sadoh WE, Sadoh AE, Onyiriuka AN. Physical activity, body mass index and blood pressure in primary school pupils attending private schools. *Afr Health Sci* 2016; **16**(4): 947-53.

1220. Igbokwe O, Adimorah G, Ikefuna A, et al. Socio-demographic determinants of malnutrition among primary school aged children in Enugu, Nigeria. *Pan Afr Med J* 2017; **28**: 248.

1221. Aadland E, Holmoy OK, Nilsen AKO. The multivariate physical activity signature associated with body mass index in young children. *Prev Med* 2021; **145**: 106437.

1222. Aadland E, Nilsen AKO, Haugland ES, Vabo KB, Aadland KN. The multivariate physical activity signatures associated with body mass index and waist-to-height ratio in 3-5-year-old Norwegian children. *Prev Med Rep* 2022; **29**: 101930.

1223. Donkor HM, Grundt JH, Juliusson PB, et al. Social and somatic determinants of underweight, overweight and obesity at 5 years of age: a Norwegian regional cohort study. *BMJ Open* 2017; **7**(8): e014548.

1224. Ovrebo B, Bergh IH, Stea TH, et al. Overweight, obesity, and thinness among a nationally representative sample of Norwegian adolescents and changes from childhood: Associations with sex, region, and population density. *PLoS One* 2021; **16**(8): e0255699.

1225. Andersen LF, Lillegaard IT, Øverby N, Lytle L, Klepp KI, Johansson L. Overweight and obesity among Norwegian schoolchildren: changes from 1993 to 2000. *Scand J Public Health* 2005; **33**(2): 99-106.

1226. Grydeland M, Bjelland M, Anderssen SA, et al. Effects of a 20-month cluster randomised controlled school-based intervention trial on BMI of school-aged boys and girls: the HEIA study. *Br J Sports Med* 2014; **48**(9): 768-73.

1227. Bernhardsen GP, Stensrud T, Nystad W, Dalene KE, Kolle E, Ekelund U. Early life risk factors for childhood obesity-Does physical activity modify the associations? The MoBa cohort study. *Scand J Med Sci Sports* 2019; **29**(10): 1636-46.

1228. Oellingrath IM, Svendsen MV, Brantsaeter AL. Eating patterns and overweight in 9- to 10-year-old children in Telemark County, Norway: a cross-sectional study. *Eur J Clin Nutr* 2010; **64**(11): 1272-9.

1229. Juliusson PB, Eide GE, Roelants M, Waaler PE, Hauspie R, Bjerknes R. Overweight and obesity in Norwegian children: prevalence and socio-demographic risk factors. *Acta Paediatr* 2010; **99**(6): 900-5.

1230. Kolle E, Steene-Johannessen J, Holme I, Andersen LB, Anderssen SA. Secular trends in adiposity in Norwegian 9-year-olds from 1999-2000 to 2005. *BMC Public Health* 2009; **9**: 389.

1231. Gebremariam MK, Andersen LF, Bjelland M, et al. Are weight-related attitudes and behaviours associated with the accuracy of BMI derived from self-reported weight and height among 13-year-olds? *Scand J Public Health*

© 2024 Zhang X et al. *JAMA Pediatrics.*

2015; **43**(2): 130-7.
1232. Fasting MH, Oien T, Storro O, Nilsen TI, Johnsen R, Vik T. Maternal smoking cessation in early pregnancy and offspring weight status at four years of age. A prospective birth cohort study. *Early Hum Dev* 2009; **85**(1): 19-24.
1233. Hestetun I, Svendsen MV, Oellingrath IM. Associations between overweight, peer problems, and mental health in 12-13-year-old Norwegian children. *Eur Child Adolesc Psychiatry* 2015; **24**(3): 319-26.
1234. Vik FN, Overby NC, Lien N, Bere E. Number of meals eaten in relation to weight status among Norwegian adolescents. *Scand J Public Health* 2010; **38**(5 Suppl): 13-8.
1235. Bjelland M, Lien N, Bergh IH, et al. Overweight and waist circumference among Norwegian 11-year-olds and associations with reported parental overweight and waist circumference: The HEIA study. *Scand J Public Health* 2010; **38**(5 Suppl): 19-27.
1236. Groholt EK, Stigum H, Nordhagen R. Overweight and obesity among adolescents in Norway: cultural and socio-economic differences. *J Public Health (Oxf)* 2008; **30**(3): 258-65.
1237. Brannsether B, Eide GE, Roelants M, Bjerknes R, Juliusson PB. Interrelationships between anthropometric variables and overweight in childhood and adolescence. *Am J Hum Biol* 2014; **26**(4): 502-10.
1238. Tanveer M, Hohmann A, Roy N, Zeba A, Tanveer U, Siener M. The Current Prevalence of Underweight, Overweight, and Obesity Associated with Demographic Factors among Pakistan School-Aged Children and Adolescents-An Empirical Cross-Sectional Study. *Int J Environ Res Public Health* 2022; **19**(18).
1239. Bekhwani AR, Khan M. Various Risk Factors of Overweight and Obesity among Children Aged 5-16 Years. *J Coll Physicians Surg Pak* 2022; **32**(6): 763-7.
1240. Ahmed J, Laghari A, Naseer M, Mehraj V. Prevalence of and factors associated with obesity among Pakistani schoolchildren: a school-based, cross-sectional study. *East Mediterr Health J* 2013; **19**(3): 242-7.
1241. Mansoori N, Nisar N, Shahid N, Mubeen SM, Ahsan S. Prevalence of obesity and its risk factors among school children in Karachi, Pakistan. *Trop Doct* 2018; **48**(4): 266-9.
1242. Anwar A, Anwar F, Joiya H, et al. Prevalence of obesity among the school-going children of Lahore and associated factors. *Journal of Ayub Medical College, Abbottabad : JAMC* 2010; **22**: 27-32.
1243. Hashmi A, Ahmed J, Saleem K. Prevalence of Obesity and Factors Leading to Obesity among High School Students of Pakistan. *Journal of Medicine* 2013; **14**.
1244. Jafar TH, Qadri Z, Islam M, Hatcher J, Bhutta ZA, Chaturvedi N. Rise in childhood obesity with persistently high rates of undernutrition among urban school-aged Indo-Asian children. *Arch Dis Child* 2008; **93**(5): 373-8.
1245. Ramzan M, Ali I, Khan A. Body mass status of school children of Dera Ismail Khan, Pakistan. *Journal of Ayub Medical College, Abbottabad : JAMC* 2008; **20**: 119-21.
1246. Aziz S, Noorulain W, Zaidi UE, Hossain K, Siddiqui IA. Prevalence of overweight and obesity among children and adolescents of affluent schools in Karachi. *J Pak Med Assoc* 2009; **59**(1): 35-8.
1247. ul Haq I, Siddiqu TS, Jan MA. Prevalence of obesity in school children of Hazara division. *Journal of Ayub Medical College Abbottabad* 2010; **22**(4): 50-2.
1248. Aziz S, Noor-Ul-Ain W, Majeed R, et al. Growth centile charts (anthropometric measurement) of Pakistani pediatric population. *J Pak Med Assoc* 2012; **62**(4): 367-77.
1249. Hydrie MZ, Basit A, Hakeem R, Ahmedani M, Masood M. Comparison of fasting glucose, Insulin levels and lipid profile in normal and overweight children. *Journal of Postgraduate Medical Institute* 2005; **19**: 8-13.
1250. Basit A, Hakeem R, Hydrie MZ, Ahmedani MY, Masood Q. Relationship among fatness, blood lipids, and insulin resistance in Pakistani children. *J Health Popul Nutr* 2005; **23**(1): 34-43.
1251. Ishaque A, Ahmad F, Zehra N, Amin H. Frequency of and factors leading to obesity and overweight in school children. *J Ayub Med Coll Abbottabad* 2012; **24**(2): 34-8.
1252. Salah RO, Ghandour R, Husseini A. Prevalence of overweight, obesity, and associated factors among
© 2024 Zhang X et al. *JAMA Pediatrics.*

adolescents in the occupied Palestinian territory: a cross-sectional study. *Lancet* 2021; **398 Suppl 1**: S46.
1253. Abudayya A, Thoresen M, Abed Y, Holmboe-Ottesen G. Overweight, stunting, and anemia are public health problems among low socioeconomic groups in school adolescents (12-15 years) in the North Gaza Strip. *Nutrition Research* 2007; **27**(12): 762-71.
1254. AbuKishk N, Gilbert H, Seita A, Mukherjee J, Rohloff PJ. Under-five malnutrition among Palestine refugee children living in camps in Jordan: a mixed-methods study. *BMJ Glob Health* 2021; **6**(8).
1255. Badrasawi M, Abu.Snouber L, Al-Tamimi M, Badrasawi K. Prevalence, Risk Factors and Psychosocial Status of Obese and Overweight Adolescents in Hebron City, Palestine. *International Journal of Nutrition, Pharmacology, Neurological Diseases* 2019; **9**(2).
1256. Al-Lahham S, Jaradat N, Altamimi M, et al. Prevalence of underweight, overweight and obesity among Palestinian school-age children and the associated risk factors: a cross sectional study. *BMC Pediatr* 2019; **19**(1): 483.
1257. Mikki N, Abdul-Rahim HF, Awartani F, Holmboe-Ottesen G. Prevalence and sociodemographic correlates of stunting, underweight, and overweight among Palestinian school adolescents (13-15 years) in two major governorates in the West Bank. *BMC Public Health* 2009; **9**: 485.
1258. Al Sabbah H, Vereecken C, Abdeen Z, Coats E, Maes L. Associations of overweight and of weight dissatisfaction among Palestinian adolescents: findings from the national study of Palestinian schoolchildren (HBSC-WBG2004). *J Hum Nutr Diet* 2009; **22**(1): 40-9.
1259. Massad S, Deckelbaum RJ, Gebre-Medhin M, et al. Double Burden of Undernutrition and Obesity in Palestinian Schoolchildren: A Cross-Sectional Study. *Food Nutr Bull* 2016; **37**(2): 144-52.
1260. Bartkowiak J, Spitzer E, Kurmann R, et al. The impact of obesity on left ventricular hypertrophy and diastolic dysfunction in children and adolescents. *Sci Rep* 2021; **11**(1): 13022.
1261. Echevarria-Castro N, Matayoshi-Perez A, Alvarado GF. Association between Maternal Depressive Symptoms with Overweight/Obesity among Children Aged 0-5 Years According to the 2016 Demographic and Family Health Survey. *Child Obes* 2020; **16**(5): 307-15.
1262. Preston EC, Ariana P, Penny ME, Frost M, Plugge E. Prevalence of childhood overweight and obesity and associated factors in Peru. *Rev Panam Salud Publica* 2015; **38**(6): 472-8.
1263. Hernández-Vásquez A, Bendezú-Quispe G, Santero M, Azañedo D. Prevalence of Childhood Obesity by Sex and Regions in Peru, 2015. *Rev Esp Salud Publica* 2016; **90**: e1-e10.
1264. Suligowska K, Buczny J. Obesity in Polish Children and Parents' Perception of Their Children's Weight Status: The Results of the SOPKARD-Junior Study. *Int J Environ Res Public Health* 2022; **19**(8).
1265. Potempa-Jeziorowska M, Jonczyk P, Świętochowska E, Kucharzewski M. Do Young Polish Children Struggle with Overweight and Obesity? *Health Problems of Civilization* 2022; **16**(1): 37-47.
1266. Kryst L, Zeglen M, Badzinska J, Kowal M, Woronkowicz A. Changes in the adiposity level and prevalence of overweight/obesity among children from Krakow (Poland) within the last decade (from 2010 to 2020). *J Biosoc Sci* 2023; **55**(3): 564-73.
1267. Bryl E, Hanc T, Szczesniewska P, Dutkiewicz A, Dmitrzak-Weglarz M, Slopien A. The relation between prenatal stress, overweight and obesity in children diagnosed according to BMI and percentage fat tissue. *Eat Weight Disord* 2022; **27**(7): 2759-73.
1268. Basiak-Rasala A, Gorna S, Krajewska J, et al. Nutritional habits according to age and BMI of 6-17-year-old children from the urban municipality in Poland. *J Health Popul Nutr* 2022; **41**(1): 17.
1269. Wyszynska J, Matlosz P, Szybisty A, Deren K, Mazur A, Herbert J. The association of actigraphic sleep measures and physical activity with excess weight and adiposity in kindergarteners. *Sci Rep* 2021; **11**(1): 2298.
1270. Wieniawski P, Werner B. Epidemiology of Obesity and Hypertension in School Adolescents Aged 15-17

from the Region of Central Poland-A Cross-Sectional Study. *Int J Environ Res Public Health* 2021; **18**(5).
1271. Matlosz P, Wyszynska J, Asif M, et al. Prevalence of Overweight, Obesity, Abdominal Obesity, and Obesity-Related Risk Factors in Polish Preschool Children: A Cross-Sectional Study. *J Clin Med* 2021; **10**(4).
1272. Zeglen M, Kryst L, Kowal M, Woronkowicz A, Sobiecki J. Changes in the prevalence of overweight/obesity and adiposity among pre-school children in Krakow, Poland, from 2008 to 2018. *J Biosoc Sci* 2020; **52**(6): 895-906.
1273. Szczyrska J, Jankowska A, Brzezinski M, Jankowski M, Metelska P, Szlagatys-Sidorkiewicz A. Prevalence of Overweight and Obesity in 6-7-Year-Old Children-A Result of 9-Year Analysis of Big City Population in Poland. *Int J Environ Res Public Health* 2020; **17**(10).
1274. Tomaszewski P, Zmijewski P, Milde K, Sienkiewicz-Dianzenza E. Weight-height relationships and central obesity in 7-year-old to 10-year-old Polish urban children: a comparison of different BMI and WHtR standards. *J Physiol Anthropol* 2015; **34**: 34.
1275. Bac A, Wozniacka R, Matusik S, Golec J, Golec E. Prevalence of overweight and obesity in children aged 6-13 years-alarming increase in obesity in Cracow, Poland. *Eur J Pediatr* 2012; **171**(2): 245-51.
1276. Baran J, Weres A, Baran R, Czenczek-Lewandowska E, Leszczak J, Wyszynska J. Preterm Birth and the Type of Birth and Their Impact on the Incidence of Overweight and Obesity in Children. *Int J Environ Res Public Health* 2022; **19**(19).
1277. Kulaga Z, Litwin M, Tkaczyk M, et al. Polish 2010 growth references for school-aged children and adolescents. *Eur J Pediatr* 2011; **170**(5): 599-609.
1278. Malecka-Tendera E, Klimek K, Matusik P, Olszanecka-Glinianowicz M, Lehingue Y. Obesity and overweight prevalence in Polish 7- to 9-year-old children. *Obes Res* 2005; **13**(6): 964-8.
1279. Kowal M, Kryst L, Woronkowicz A, Sobiecki J. Long-term changes in body composition and prevalence of overweight and obesity in girls (aged 3-18 years) from Krakow (Poland) from 1983, 2000 and 2010. *Ann Hum Biol* 2014; **41**(5): 415-27.
1280. Chrzanowska M, Koziel S, Ulijaszek SJ. Changes in BMI and the prevalence of overweight and obesity in children and adolescents in Cracow, Poland, 1971-2000. *Econ Hum Biol* 2007; **5**(3): 370-8.
1281. Zadzinska E, Rosset I. Pre-natal and perinatal factors affecting body mass index in pre-pubertal Polish children. *Ann Hum Biol* 2013; **40**(6): 477-84.
1282. Zatonski T, Pazdro-Zastawny K, Kolator M, et al. A study on health and the association between overweight/obesity and otorhinolaryngological diseases in 6- to 17-year-old children from Wroclaw, Poland. *Arch Med Sci* 2022; **18**(2): 413-21.
1283. Wozniacka R, Bac A, Kowal M, Matusik S. DIFFERENCES IN THE PREVALENCE OF OVERWEIGHT AND OBESITY IN 5- to 14-YEAR-OLD CHILDREN IN KRAKOW, POLAND, USING THREE NATIONAL BMI CUT-OFFS. *J Biosoc Sci* 2018; **50**(3): 365-79.
1284. Baran J, Weres A, Czenczek-Lewandowska E, et al. Early Eating Patterns and Overweight and Obesity in a Sample of Preschool Children in South-East Poland. *Int J Environ Res Public Health* 2019; **16**(17).
1285. Jodkowska M, Oblacinska A, Tabak I. Overweight and obesity among adolescents in Poland: gender and regional differences. *Public Health Nutr* 2010; **13**(10A): 1688-92.
1286. Zawodniak-Szałapska M, Stawerska R, Lewiński A. The prevalence of obesity of children (aged 13-15) and the significance of selected obesity risk factors. *Archives of Medical Science* 2007; **3**(4): 376-82.
1287. Klimek-Piotrowska W, Koziej M, Holda MK, et al. Anthropometry and body composition of adolescents in cracow, poland. *PLoS One* 2015; **10**(3): e0122274.
1288. Dlugosz A, Niedzwiedzka E, Dlugosz T, Wadolowska L. Socio-economic status as an environmental factor - incidence of underweight, overweight and obesity in adolescents from less-urbanized regions of Poland. *Ann Agric Environ Med* 2015; **22**(3): 518-23.

© 2024 Zhang X et al. *JAMA Pediatrics.*

1289. Stankiewicz M, Pieszko M, Sliwinska A, et al. Obesity and diet awareness among Polish children and adolescents in small towns and villages. *Cent Eur J Public Health* 2014; **22**(1): 12-6.

1290. Kowalkowska J, Wadolowska L, Weronika Wuenstel J, Słowińska MA, Niedźwiedzka E. Socioeconomic status and overweight prevalence in polish adolescents: the impact of single factors and a complex index of socioeconomic status in respect to age and sex. *Iran J Public Health* 2014; **43**(7): 913-25.

1291. Januszek-Trzciakowska A, Malecka-Tendera E, Klimek K, Matusik P. Obesity risk factors in a representative group of Polish prepubertal children. *Arch Med Sci* 2014; **10**(5): 880-5.

1292. Golec J, Kmiotek EK, Czechowska D, et al. Analysis of body composition among children and adolescents - a cross-sectional study of the Polish population and comparison of body fat measurement methods. *J Pediatr Endocrinol Metab* 2014; **27**(7-8): 603-9.

1293. Rutkowski B, Czarniak P, Krol E, Szczesniak P, Zdrojewski T. Overweight, obesity, hypertension and albuminuria in Polish adolescents--results of the Sopkard 15 study. *Nephrol Dial Transplant* 2013; **28 Suppl 4**: iv204-11.

1294. Czyz SH, Toriola AL, Starosciak W, Lewandowski M, Paul Y, Oyeyemi AL. Physical Fitness, Physical Activity, Sedentary Behavior, or Diet-What Are the Correlates of Obesity in Polish School Children? *Int J Environ Res Public Health* 2017; **14**(6).

1295. Kantanista A, Krol-Zielinska M, Borowiec J, Osinski W. Is Underweight Associated with more Positive Body Image? Results of a Cross-Sectional Study in Adolescent Girls and Boys. *Span J Psychol* 2017; **20**: E8.

1296. Paciencia I, Cavaleiro Rufo J, Mendes F, et al. A cross-sectional study of the impact of school neighbourhood on children obesity and body composition. *Eur J Pediatr* 2021; **180**(2): 535-45.

1297. Nazareth M, Pinto E, Severo M, Lopes C, Rego C. Association between parental and offspring BMI: results from EPACI Portugal 2012. *Public Health Nutr* 2021; **24**(10): 2798-807.

1298. Abreu S, Agostinis-Sobrinho C, Santos R, et al. Association of Dairy Product Consumption with Metabolic and Inflammatory Biomarkers in Adolescents: A Cross-Sectional Analysis from the LabMed Study. *Nutrients* 2019; **11**(10).

1299. Abreu S, Santos R, Moreira C, et al. Relationship of milk intake and physical activity to abdominal obesity among adolescents. *Pediatr Obes* 2014; **9**(1): 71-80.

1300. Frias-Bulhosa J, Barbosa P, Gomes E, Vieira MR, Manso MC. Association between body mass index and caries among 13-year-old population in Castelo de Paiva, Portugal. *Revista Portuguesa de Estomatologia, Medicina Dentária e Cirurgia Maxilofacial* 2015; **56**(1): 3-8.

1301. Abreu S, Santos R, Moreira C, Santos PC, Mota J, Moreira P. Food consumption, physical activity and socio-economic status related to BMI, waist circumference and waist-to-height ratio in adolescents. *Public Health Nutr* 2014; **17**(8): 1834-49.

1302. Padez C, Mourão I, Moreira P, Rosado V. Prevalence and risk factors for overweight and obesity in Portuguese children. *Acta Paediatr* 2005; **94**(11): 1550-7.

1303. Araujo J, Severo M, Ramos E. Sleep duration and adiposity during adolescence. *Pediatrics* 2012; **130**(5): e1146-54.

1304. Abreu S, Santos R, Moreira C, et al. Milk intake is inversely related to body mass index and body fat in girls. *Eur J Pediatr* 2012; **171**(10): 1467-74.

1305. Silva-Santos S, Santos A, Vale S, Mota J. Motor fitness and preschooler children obesity status. *J Sports Sci* 2017; **35**(17): 1704-8.

1306. Sardinha LB, Santos R, Vale S, et al. Prevalence of overweight and obesity among Portuguese youth: a study in a representative sample of 10-18-year-old children and adolescents. *Int J Pediatr Obes* 2011; **6**(2-2): e124-8.

© 2024 Zhang X et al. *JAMA Pediatrics.*

1307. Seabra A, Mendonca D, Maia J, et al. Gender, weight status and socioeconomic differences in psychosocial correlates of physical activity in schoolchildren. *J Sci Med Sport* 2013; **16**(4): 320-6.
1308. Rito A, Wijnhoven TM, Rutter H, et al. Prevalence of obesity among Portuguese children (6-8 years old) using three definition criteria: COSI Portugal, 2008. *Pediatr Obes* 2012; **7**(6): 413-22.
1309. Antunes AM, Maia JA, Stasinopoulos MD, et al. Gross motor coordination and weight status of Portuguese children aged 6-14 years. *Am J Hum Biol* 2015; **27**(5): 681-9.
1310. Ferreira RJ, Marques-Vidal PM. Prevalence and determinants of obesity in children in public schools of Sintra, Portugal. *Obesity (Silver Spring)* 2008; **16**(2): 497-500.
1311. Marques-Vidal P, Ferreira R, Oliveira JM, Paccaud F. Is thinness more prevalent than obesity in Portuguese adolescents? *Clin Nutr* 2008; **27**(4): 531-6.
1312. Cabral M, Araujo J, Teixeira J, et al. Vitamin D levels and cardiometabolic risk factors in Portuguese adolescents. *Int J Cardiol* 2016; **220**: 501-7.
1313. Ribeiro AI, Santos AC, Vieira VM, Barros H. Hotspots of childhood obesity in a large metropolitan area: does neighbourhood social and built environment play a part? *Int J Epidemiol* 2020; **49**(3): 934-43.
1314. Mota J, Fidalgo F, Silva R, et al. Relationships between physical activity, obesity and meal frequency in adolescents. *Ann Hum Biol* 2008; **35**(1): 1-10.
1315. Minghelli B, Nunes C, Oliveira R. Body mass index and waist circumference to define thinness, overweight and obesity in Portuguese adolescents: Comparison between CDC, IOTF, WHO references. *Pediatric endocrinology reviews : PER* 2014; **12**: 35-41.
1316. Albuquerque D, Nóbrega C, Samouda H, Manco L. Assessment of obesity and abdominal obesity among Portuguese children. *Acta Med Port* 2012; **25**(3): 169-73.
1317. Padez C, Fernandes T, Mourao I, Moreira P, Rosado V. Prevalence of overweight and obesity in 7-9-year-old Portuguese children: trends in body mass index from 1970-2002. *Am J Hum Biol* 2004; **16**(6): 670-8.
1318. Vale S, Santos R, Soares-Miranda L, Mota J. The relationship of cardiorespiratory fitness, birth weight and parental BMI on adolescents' obesity status. *Eur J Clin Nutr* 2010; **64**(6): 622-7.
1319. Gama A, Rosado-Marques V, Machado-Rodrigues AM, Nogueira H, MourAo I, Padez C. Prevalence of overweight and obesity in 3-to-10-year-old children: assessment of different cut-off criteria WHO-IOTF. *An Acad Bras Cienc* 2020; **92**(2): e20190449.
1320. Pereira SA, Seabra AT, Silva RG, Katzmarzyk PT, Beunen GP, Maia JA. Prevalence of overweight, obesity and physical activity levels in children from Azores Islands. *Ann Hum Biol* 2010; **37**(5): 682-91.
1321. Padez C, Mourao I, Moreira P, Rosado V. Long sleep duration and childhood overweight/obesity and body fat. *Am J Hum Biol* 2009; **21**(3): 371-6.
1322. Fonseca H, Matos MG, Guerra A, Pedro JG. Are overweight and obese adolescents different from their peers? *Int J Pediatr Obes* 2009; **4**(3): 166-74.
1323. Rebelo D, Teixeira J, Marques-Vidal P, Oliveira JM. Obesity markers and blood pressure in a sample of Portuguese children and adolescents. *Eur J Cardiovasc Prev Rehabil* 2008; **15**(1): 73-7.
1324. Aires L, Silva P, Santos R, Santos M, Ribeiro J, Mota J. Association of physical fitness and Body Mass Index in youth. *Minerva pediatrica* 2008; **60**: 397-405.
1325. Moreira P, Padez C, Mourao-Carvalhal I, Rosado V. Maternal weight gain during pregnancy and overweight in Portuguese children. *Int J Obes (Lond)* 2007; **31**(4): 608-14.
1326. Ribeiro J, Santos P, Duarte J, Mota J. Association between overweight and early sexual maturation in Portuguese boys and girls. *Ann Hum Biol* 2006; **33**(1): 55-63.
1327. Mota J, Flores L, Flores L, Ribeiro JC, Santos MP. Relationship of single measures of cardiorespiratory fitness and obesity in young schoolchildren. *Am J Hum Biol* 2006; **18**(3): 335-41.
© 2024 Zhang X et al. *JAMA Pediatrics.*

1328. Vale S, Trost SG, Rego C, Abreu S, Mota J. Physical Activity, Obesity Status, and Blood Pressure in Preschool Children. *J Pediatr* 2015; **167**(1): 98-102.
1329. Minghelli B, Oliveira R, Nunes C. Association of obesity with chronic disease and musculoskeletal factors. *Rev Assoc Med Bras (1992)* 2015; **61**(4): 347-54.
1330. Pedrosa C, Correia F, Seabra D, Oliveira BM, Simoes-Pereira C, Vaz-de-Almeida MD. Prevalence of overweight and obesity among 7-9-year-old children in Aveiro, Portugal: comparison between IOTF and CDC references. *Public Health Nutr* 2011; **14**(1): 14-9.
1331. Valente H, Teixeira V, Padrao P, et al. Sugar-sweetened beverage intake and overweight in children from a Mediterranean country. *Public Health Nutr* 2011; **14**(1): 127-32.
1332. Vasques C, Mota M, Correia T, Lopes V. Prevalence of overweight/obesity and its association with sedentary behavior in children. *Rev Port Cardiol* 2012; **31**(12): 783-8.
1333. Nogueira H, Ferrao M, Gama A, Mourao I, Rosado Marques V, Padez C. Perceptions of neighborhood environments and childhood obesity: evidence of harmful gender inequities among Portuguese children. *Health Place* 2013; **19**: 69-73.
1334. Marques A, de Matos MG. Trends in prevalence of overweight and obesity: are Portuguese adolescents still increasing weight? *Int J Public Health* 2016; **61**(1): 49-56.
1335. Marques G, Matos M, Afonso C, et al. Childhood obesity and reduction of hours of sleep: results from cross-sectional study. *O Mundo da Saúde* 2018; **42**(4): 811-22.
1336. Santiago H, Pagán D, Acevedo A. Obesity and Hypertension in School Children of Puerto Rico. *P R Health Sci J* 2021; **40**(1): 45-9.
1337. Rivera W, Rodriguez-Figueroa L, Calderón G. Prevalence of Childhood Obesity in a Representative Sample of Elementary School Children in Puerto Rico by Socio-Demographic Characteristics, 2008. *Puerto Rico health sciences journal* 2010; **29**: 357-63.
1338. Nazario Rodriguez IJ, Figueroa WI, Rosado J, Parrilla Idel C. Perception of parents regarding their children's weight. *Bol Asoc Med P R* 2008; **100**(2): 33-8.
1339. Elias-Boneta AR, Toro MJ, Garcia O, Torres R, Palacios C. High prevalence of overweight and obesity among a representative sample of Puerto Rican children. *BMC Public Health* 2015; **15**: 219.
1340. Torres R, Serrano M, Pérez CM, Palacios C. Physical environment, diet quality, and body weight in a group of 12-year-old children from four public schools in Puerto Rico. *P R Health Sci J* 2014; **33**(1): 14-21.
1341. Cheema S, Abraham A, El-Nahas KG, et al. Assessment of Overweight, Obesity, Central Obesity, and Type 2 Diabetes among Adolescents in Qatar: A Cross-Sectional Study. *Int J Environ Res Public Health* 2022; **19**(21).
1342. Kerkadi A, Sadig AH, Bawadi H, et al. The Relationship between Lifestyle Factors and Obesity Indices among Adolescents in Qatar. *Int J Environ Res Public Health* 2019; **16**(22).
1343. Bener A, Kamal A. Growth Patterns of Qatari School Children and Adolescents Aged 6-18 Years. *Journal of health, population, and nutrition* 2005; **23**: 250-8.
1344. Bener A. Prevalence of obesity, overweight, and underweight in Qatari adolescents. *Food Nutr Bull* 2006; **27**(1): 39-45.
1345. Passmore E, Smith T. Dual Burden of Stunting and Obesity Among Elementary School Children on Majuro, Republic of Marshall Islands. *Hawaii J Health Soc Welf* 2019; **78**(8): 262-6.
1346. Barbu CG, Teleman MD, Albu AI, et al. Obesity and eating behaviors in school children and adolescents - data from a cross sectional study from Bucharest, Romania. *BMC Public Health* 2015; **15**: 206.
1347. Pop TL, Maniu D, Rajka D, et al. Prevalence of Underweight, Overweight and Obesity in School-Aged Children in the Urban Area of the Northwestern Part of Romania. *Int J Environ Res Public Health* 2021; **18**(10).
1348. Valean C, Tatar S, Nanulescu M, Leucuta A, Ichim G. Prevalence of obesity and overweight among school

© 2024 Zhang X et al. *JAMA Pediatrics.*

children in Cluj-Napoca. *Acta Endocrinologica (Buc)* 2009; **5**(2): 213-9.
1349. Mocanu V. Prevalence of overweight and obesity in urban elementary school children in northeastern Romania: its relationship with socioeconomic status and associated dietary and lifestyle factors. *Biomed Res Int* 2013; **2013**: 537451.
1350. Emandi A. Impact of increased body mass on growth patterns in school children. *Acta Endocrinologica (Bucharest)* 2012; **8**(4): 551-63.
1351. Eliza C, Balgradean M. Hypertension in Romanian Children and Adolescents: A Cross-Sectional Survey. *Mædica* 2013; **8**: 5-10.
1352. Khasnutdinova SL, Grjibovski AM. Prevalence of stunting, underweight, overweight and obesity in adolescents in Velsk district, north-west Russia: a cross-sectional study using both international and Russian growth references. *Public Health* 2010; **124**(7): 392-7.
1353. Choy CC, Howe CJ, Soti-Ulberg C, et al. Is overweight or obesity associated with anemia in children? Follow-up of Samoans in the Ola Tuputupua'e "Growing Up" study. *Obes Res Clin Pract* 2022; **16**(2): 144-50.
1354. Choy CC, Desai MM, Park JJ, et al. Child, maternal and household-level correlates of nutritional status: a cross-sectional study among young Samoan children. *Public Health Nutr* 2017; **20**(7): 1235-47.
1355. Abdellatif H, Hebbal MI. Dental Caries and Its Association with Body Mass Index among School Children of Riyadh, Saudi Arabia. *J Pharm Bioallied Sci* 2020; **12**(Suppl 1): S176-s81.
1356. Mohamed RN, Basha S, Al-Thomali Y, AlZahrani FS, Ashour AA, Almutair NE. Association Between Early Childhood Caries and Obesity among Preschool Children. *Oral Health Prev Dent* 2022; **20**(1): 113-8.
1357. Abu El Qomsan MA, Alasqah MN, Alqahtani FA, Alobaydaa MA, Alharbi MM, Kola Z. Intricate Evaluation of Association between Dental Caries and Obesity among the Children in Al-Kharj City (Saudi Arabia). *J Contemp Dent Pract* 2017; **18**(1): 29-33.
1358. Alghamdi AA, Almahdy A. Association Between Dental Caries and Body Mass Index in Schoolchildren Aged Between 14 and 16 Years in Riyadh, Saudi Arabia. *J Clin Med Res* 2017; **9**(12): 981-6.
1359. Bhayat A, Ahmad MS, Fadel HT. Association between body mass index, diet and dental caries in Grade 6 boys in Medina, Saudi Arabia. *East Mediterr Health J* 2016; **22**(9): 687-93.
1360. Farsi DJ, Elkhodary HM. The prevalence of overweight/obesity in high school adolescents in Jeddah and the association of obesity association with dental caries. *Ann Saudi Med* 2017; **37**(2): 114-21.
1361. Farsi DJ, Elkhodary HM, Merdad LA, et al. Prevalence of obesity in elementary school children and its association with dental caries. *Saudi Med J* 2016; **37**(12): 1387-94.
1362. Quadri MF, Hakami BM, Hezam AA, et al. Relation between Dental Caries and Body Mass Index-for-age among Schoolchildren of Jazan City, Kingdom of Saudi Arabia. *J Contemp Dent Pract* 2017; **18**(4): 277-82.
1363. Ahmad Bahathig A, Abu Saad H, Md Yusop NB, Mohd Shukri NH, El-Din MME. Relationship between Physical Activity, Sedentary Behavior, and Anthropometric Measurements among Saudi Female Adolescents: A Cross-Sectional Study. *Int J Environ Res Public Health* 2021; **18**(16).
1364. Amin TT, Al-Sultan AI, Ali A. Overweight and obesity and their relation to dietary habits and socio-demographic characteristics among male primary school children in Al-Hassa, Kingdom of Saudi Arabia. *Eur J Nutr* 2008; **47**(6): 310-8.
1365. Bajamal E, Robbins LB, Ling J, Smith B, Pfeiffer KA, Sharma D. Physical Activity Among Female Adolescents in Jeddah, Saudi Arabia: A Health Promotion Model-Based Path Analysis. *Nurs Res* 2017; **66**(6): 473-82.
1366. Bawazeer NM, Al-Daghri NM, Valsamakis G, et al. Sleep duration and quality associated with obesity among Arab children. *Obesity (Silver Spring)* 2009; **17**(12): 2251-3.
1367. Abalkhail BA, Shawky S, Soliman NK. Validity of self-reported weight and height among Saudi school
© 2024 Zhang X et al. *JAMA Pediatrics.*

children and adolescents. *Saudi Med J* 2002; **23**(7): 831-7.
1368. Al-Attas OS, Al-Daghri N, Bamakhramah A, Shaun Sabico S, McTernan P, Huang TT. Telomere length in relation to insulin resistance, inflammation and obesity among Arab youth. *Acta Paediatr* 2010; **99**(6): 896-9.
1369. Al Alwan I, Al Fattani A, Longford N. The effect of parental socioeconomic class on children's body mass indices. *J Clin Res Pediatr Endocrinol* 2013; **5**(2): 110-5.
1370. Al-Almaie SM. Prevalence of obesity and overweight among Saudi adolescents in Eastern Saudi Arabia. *Saudi Med J* 2005; **26**(4): 607-11.
1371. Mustafa AEM, Assery AAA, Asiri FMA, Alfarhan NM, Alqarni AM, Alqahtani FMS. Childhood obesity and its relation with dietary habits among children in Aseer region, Southern Saudi Arabia. *J Family Med Prim Care* 2021; **10**(10): 3760-4.
1372. Al-Agha AE, Nizar FS, Nahhas AM. The association between body mass index and duration spent on electronic devices in children and adolescents in Western Saudi Arabia. *Saudi Med J* 2016; **37**(4): 436-9.
1373. Al-Musharaf S, Al-Othman A, Al-Daghri NM, et al. Vitamin D deficiency and calcium intake in reference to increased body mass index in children and adolescents. *Eur J Pediatr* 2012; **171**(7): 1081-6.
1374. Alselaim N, Malaekah H, Saade M, et al. Does obesity impact the pattern and outcome of trauma in children? *J Pediatr Surg* 2012; **47**(7): 1404-9.
1375. Washi SA, Ageib MB. Poor diet quality and food habits are related to impaired nutritional status in 13- to 18-year-old adolescents in Jeddah. *Nutr Res* 2010; **30**(8): 527-34.
1376. Al-Hussaini A, Bashir MS, Khormi M, et al. Overweight and obesity among Saudi children and adolescents: Where do we stand today? *Saudi J Gastroenterol* 2019; **25**(4): 229-35.
1377. Fakeeh MI, Shanawaz M, Azeez FK, Arar IA. Overweight and obesity among the boys of primary public schools of Baish City in Jazan Province, Saudi Arabia: A cross-sectional study. *Indian J Public Health* 2019; **63**(4): 330-3.
1378. El Mouzan MI, Foster PJ, Al Herbish AS, et al. Prevalence of overweight and obesity in Saudi children and adolescents. *Ann Saudi Med* 2010; **30**(3): 203-8.
1379. Al-Dossary SS, Sarkis PE, Hassan A, Ezz El Regal M, Fouda AE. Obesity in Saudi children: a dangerous reality. *East Mediterr Health J* 2010; **16**(9): 1003-8.
1380. Al-Saeed WY, Al-Dawood KM, Bukhari IA, Bahnassy A. Prevalence and socioeconomic risk factors of obesity among urban female students in Al-Khobar city, Eastern Saudi Arabia, 2003. *Obes Rev* 2007; **8**(2): 93-9.
1381. Al-Muhaimeed AA, Dandash K, Ismail MS, Saquib N. Prevalence and correlates of overweight status among Saudi school children. *Ann Saudi Med* 2015; **35**(4): 275-81.
1382. Akinpelu A, Oyewole O, Odole A, Tella B. Nutritional status of Nigerian children from urban community using different reference cut-offs. *African Journal of Biomedical Research* 2014; **17**(2): 61-7.
1383. El Mouzan MI, Al Herbish AS, Al Salloum AA, Al Omar AA, Qurachi MM. Regional variation in prevalence of overweight and obesity in Saudi children and adolescents. *Saudi J Gastroenterol* 2012; **18**(2): 129-32.
1384. Al-Dahi AS, Albalawi FA, Al Alwani SS, Al Balawi MM. Prevalence of overweight and obesity among Saudi primary school students in Tabuk, Saudi Arabia 2015. *Bangladesh J Med Sci 2016; 15 (3): 329* 2016; **334**.
1385. Susnjevic S, Milijasevic D, Maric D, Niciforovic-Surkovic O, Mijatovic-Jovanovic V, Ukropina S. Association between flat foot prevalence and nutritional status in schoolchildren. *Srpski arhiv za celokupno lekarstvo* 2022; **150**(1-2): 59-63.
1386. Djordjic V, Radisavljevic S, Milanovic I, et al. WHO European Childhood Obesity Surveillance Initiative in Serbia: a prevalence of overweight and obesity among 6-9-year-old school children. *J Pediatr Endocrinol Metab* 2016; **29**(9): 1025-30.
1387. Rada R, Tatjana P, Miroslav S, Jelena B. Nutritional status and prevalence of abdominal obesity in
© 2024 Zhang X et al. *JAMA Pediatrics.*

adolescents aged 11 to 15 years in Vojvodina (the Republic of Serbia). *Progress in Nutrition* 2019.
1388. Bukara-Radujković G, Zdravković D. Physical activity as an important determinant in developing childhood obesity. *Med Pregl* 2009; **62**(3-4): 107-13.
1389. Markovic D, Ristic-Medic D, Vucic V, et al. Association between being overweight and oral health in Serbian schoolchildren. *Int J Paediatr Dent* 2015; **25**(6): 409-17.
1390. Rakic R, Bozic-Krstic V, Pavlica T. Relationship between overweight, obesity and socioeconomic factors of adolescents in Vojvodina, Serbia. *Homo* 2011; **62**(4): 307-13.
1391. Srdic B, Obradovic B, Dimitric G, Stokic E, Babovic SS. Relationship between body mass index and body fat in children-Age and gender differences. *Obes Res Clin Pract* 2012; **6**(2): e91-e174.
1392. Janić SR, Milanović I, Živković M, Mirkov D. Prevalence of overweight and obesity among Belgrade youth: A study in a representative sample of 9–14-year-old children and adolescents. *Anthropological Notebooks* 2013; **19**(3).
1393. Bjelanovic J, Velicki R, Popovic M, Bjelica A, Jevtic M. Prevalence and some risk factors of childhood obesity. *Progress in Nutrition* 2017; **19**(2): 138-45.
1394. Halasi S, Lepes J, Dordic V, et al. Relationship between obesity and health-related quality of life in children aged 7-8 years. *Health Qual Life Outcomes* 2018; **16**(1): 149.
1395. Chiolero A, Madeleine G, Gabriel A, Burnier M, Paccaud F, Bovet P. Prevalence of elevated blood pressure and association with overweight in children of a rapidly developing country. *J Hum Hypertens* 2007; **21**(2): 120-7.
1396. Bovet P, Auguste R, Burdette H. Strong inverse association between physical fitness and overweight in adolescents: a large school-based survey. *Int J Behav Nutr Phys Act* 2007; **4**: 24.
1397. Bovet P, Chiolero A, Madeleine G, Paccaud F. Prevalence of overweight and underweight in public and private schools in the Seychelles. *Int J Pediatr Obes* 2010; **5**(3): 274-8.
1398. Yeo SC, Jos AM, Erwin C, et al. Associations of sleep duration on school nights with self-rated health, overweight, and depression symptoms in adolescents: problems and possible solutions. *Sleep Med* 2019; **60**: 96-108.
1399. Fu WP, Lee HC, Ng CJ, et al. Screening for childhood obesity: international vs population-specific definitions. Which is more appropriate? *Int J Obes Relat Metab Disord* 2003; **27**(9): 1121-6.
1400. Pwint MK, Lee YS, Wong TY, Saw SM. Prevalence of overweight and obesity in Chinese preschoolers in Singapore. *Ann Acad Med Singap* 2013; **42**(2): 66-72.
1401. Sabanayagam C, Shankar A, Chong YS, Wong TY, Saw SM. Breast-feeding and overweight in Singapore school children. *Pediatr Int* 2009; **51**(5): 650-6.
1402. Deurenberg-Yap M, Niti M, Foo LL, Ng SA, Loke KY. Diagnostic accuracy of anthropometric indices for obesity screening among Asian adolescents. *Ann Acad Med Singap* 2009; **38**(1): 3-6.
1403. Planinsec J, Matejek C. Differences in physical activity between non-overweight, overweight and obese children. *Coll Antropol* 2004; **28**(2): 747-54.
1404. Planinsec J, Fosnaric S. Body mass index and triceps skinfold thickness in prepubertal children in Slovenia. *Coll Antropol* 2009; **33**(2): 341-5.
1405. Sedej K, Kotnik P, Avbelj Stefanija M, et al. Decreased prevalence of hypercholesterolaemia and stabilisation of obesity trends in 5-year-old children: possible effects of changed public health policies. *Eur J Endocrinol* 2014; **170**(2): 293-300.
1406. Sedej K, Lusa L, Battelino T, Kotnik P. Stabilization of Overweight and Obesity in Slovenian Adolescents and Increased Risk in Those Entering Non-Grammar Secondary Schools. *Obes Facts* 2016; **9**(4): 241-50.
1407. Symington EA, Gericke GJ, Nel JH, Labadarios D. The relationship between stunting and overweight among children from South Africa: Secondary analysis of the National Food Consumption Survey--Fortification
© 2024 Zhang X et al. *JAMA Pediatrics.*

Baseline I. *S Afr Med J* 2015; **106**(1): 65-9.
1408. Kirsten AP, Marais D, Schübl C. The influence of socio-demographic factors on the nutritional status of children in the Stellenbosch area, Western Cape. *South African Journal of Clinical Nutrition* 2013; **26**(3): 124-31.
1409. Nomatshila SC, Mabunda SA, Puoane T, Apalata TR. Prevalence of Obesity and Associated Risk Factors among Children and Adolescents in the Eastern Cape Province. *Int J Environ Res Public Health* 2022; **19**(5).
1410. Engwa GA, Anye C, Nkeh-Chungag BN. Association between obesity and lung function in South African adolescents of African Ancestry. *BMC Pediatr* 2022; **22**(1): 109.
1411. Abrahams Z, de Villiers A, Steyn NP, et al. What's in the lunchbox? Dietary behaviour of learners from disadvantaged schools in the Western Cape, South Africa. *Public Health Nutr* 2011; **14**(10): 1752-8.
1412. Armstrong M, Lambert M, Sharwood KA, Lambert E. Obesity and overweight in South African primary school children - The Health of the Nation Study. *South African medical journal = Suid-Afrikaanse tydskrif vir geneeskunde* 2006; **96**: 439-44.
1413. Baard M. Body mass index and associated physical activity levels in 7 - 10-year-old children in primary schools in Port Elizabeth. *South African Journal of Sports Medicine* 2014; **26**(4): 115-8.
1414. Moselakgomo V, Monyeki MA, Toriola A. Relationship between physical activity and risk factors of body weight disorders among south African primary school children. *Biomed Res* 2015; **26**(4): 730-8.
1415. Oldewage-Theron WH, Egal AA. Nutrition knowledge and nutritional status of primary school children in QwaQwa. *South African Journal of Clinical Nutrition* 2010; **23**(3).
1416. Pedro TM, Kahn K, Pettifor J, Tollman S, Norris S. Under-and overnutrition and evidence of metabolic disease risk in rural black South African children and adolescents. *South African Journal of Clinical Nutrition* 2014; **27**(4): 194-200.
1417. Pienaar AE. Prevalence of overweight and obesity among primary school children in a developing country: NW-CHILD longitudinal data of 6-9-yr-old children in South Africa. *BMC Obes* 2015; **2**: 2.
1418. Puckree T, Naidoo P, Pillay P, Naidoo T. Underweight and overweight in primary school children in eThekwini district in KwaZulu-Natal, South Africa. *African Journal of Primary Health Care & Family Medicine* 2011; **3**(1).
1419. Tathiah N, Moodley I, Mubaiwa V, Denny L, Taylor M. South Africa's nutritional transition: overweight, obesity, underweight and stunting in female primary school learners in rural KwaZulu-Natal, South Africa. *S Afr Med J* 2013; **103**(10): 718-23.
1420. Truter L, Pienaar AE, Du Toit D. The relationship of overweight and obesity to the motor performance of children living in South Africa. *South African Family Practice* 2014; **54**(5): 429-35.
1421. Wiles N, Green J, Veldman F. Tuck-shop purchasing practices of Grade 4 learners in Pietermaritzburg and childhood overweight and obesity. *South African Journal of Clinical Nutrition* 2013; **26**(1): 37-42.
1422. Negash S, Agyemang C, Matsha TE, Peer N, Erasmus RT, Kengne AP. Differential prevalence and associations of overweight and obesity by gender and population group among school learners in South Africa: a cross-sectional study. *BMC Obes* 2017; **4**: 29.
1423. Kimani-Murage EW, Kahn K, Pettifor JM, Tollman SM, Klipstein-Grobusch K, Norris SA. Predictors of adolescent weight status and central obesity in rural South Africa. *Public Health Nutr* 2011; **14**(6): 1114-22.
1424. Reddy SP, Resnicow K, James S, et al. Rapid increases in overweight and obesity among South African adolescents: comparison of data from the South African National Youth Risk Behaviour Survey in 2002 and 2008. *American journal of public health* 2012; **102**(2): 262-8.
1425. Moselakgomo KV, Van Staden M. Diagnostic comparison of Centers for Disease Control and Prevention and International Obesity Task Force criteria for obesity classification in South African children. *Afr J Prim Health Care Fam Med* 2017; **9**(1): e1-e7.

© 2024 Zhang X et al. *JAMA Pediatrics.*

1426. Modjadji P, Madiba S. The double burden of malnutrition in a rural health and demographic surveillance system site in South Africa: a study of primary schoolchildren and their mothers. *BMC Public Health* 2019; **19**(1): 1087.
1427. Pretorius SS, Neophytou N, Watson ED. Anthropometric profiles of 8-11 year old children from a low-income setting in South Africa. *BMC Public Health* 2019; **19**(1): 314.
1428. Monyeki KD, Kemper HC, Makgae PJ. The association of fat patterning with blood pressure in rural South African children: the Ellisras Longitudinal Growth and Health Study. *Int J Epidemiol* 2006; **35**(1): 114-20.
1429. Kruger R, Kruger H, Macintyre U. The determinants of overweight and obesity among 10-to 15-year-old schoolchildren in the North West Province, South Africa–the THUSA BANA (Transition and Health during Urbanisation of South Africans; BANA, children) study. *Public health nutrition* 2006; **9**(3): 351-8.
1430. Nkeh-Chungag BN, Sekokotla AM, Sewani-Rusike C, Namugowa A, Iputo JE. Prevalence of hypertension and pre-hypertension in 13-17 year old adolescents living in Mthatha - South Africa: a cross-sectional study. *Cent Eur J Public Health* 2015; **23**(1): 59-64.
1431. Meko LN, Slabber-Stretch M, Walsh CM, Kruger SH, Nel M. School environment, socioeconomic status and weight of children in Bloemfontein, South Africa. *Afr J Prim Health Care Fam Med* 2015; **7**(1).
1432. Van Niekerk S-M, Grimmer K, Louw Q. The prevalence of underweight, overweight and obesity in a multiracial group of urban adolescent schoolchildren in the Cape Metropole area of Cape Town. *South African Journal of Clinical Nutrition* 2014; **27**(1): 18-24.
1433. Toriola AL, Moselakgomo V, Shaw B, Goon D. Overweight, obesity and underweight in rural black South African children. *South African Journal of Clinical Nutrition* 2012; **25**(2): 57-61.
1434. Craig E, Reilly JJ, Bland R. Risk factors for overweight and overfatness in rural South African children and adolescents. *J Public Health (Oxf)* 2016; **38**(1): 24-33.
1435. Choo J, Yang HM, Jae SY, Kim HJ, You J, Lee J. Effects of the Healthy Children, Healthy Families, Healthy Communities Program for Obesity Prevention among Vulnerable Children: A Cluster-Randomized Controlled Trial. *Int J Environ Res Public Health* 2020; **17**(8).
1436. Park HK, Lim JS. Change of obesity prevalence and lifestyle patterns before and during COVID-19 among Korean adolescents. *Ann Pediatr Endocrinol Metab* 2022; **27**(3): 183-91.
1437. Kim SY, Choi SW, Park J, Ryu SY, Han MA, Park SY. Comparing the nutritional status of children and adolescents from North Korean defector families and South Korean families. *BMJ Open* 2021; **11**(10): e052059.
1438. Park S, Choi BY, Wang Y, Colantuoni E, Gittelsohn J. School and neighborhood nutrition environment and their association with students' nutrition behaviors and weight status in Seoul, South Korea. *J Adolesc Health* 2013; **53**(5): 655-62 e12.
1439. Bae JH, Choi JH. Gender disparities in childhood obesity and household food insecurity. *Nutrition* 2021; **87-88**: 111190.
1440. Baek S-I, So W-Y. Association between weight status and recreational drug abuse in Korean adolescents. *Open Medicine* 2012; **7**(2): 224-9.
1441. Kim J-H, So W-Y. Association between frequency of breakfast eating and obesity in Korean adolescents. *Iranian journal of public health* 2012; **41**(6): 50.
1442. Byun W, Dowda M, Pate RR. Associations between screen-based sedentary behavior and cardiovascular disease risk factors in Korean youth. *J Korean Med Sci* 2012; **27**(4): 388-94.
1443. Lee SH, Kim SM, Park HS, et al. Serum 25-hydroxyvitamin D levels, obesity and the metabolic syndrome among Korean children. *Nutr Metab Cardiovasc Dis* 2013; **23**(8): 785-91.
1444. Lee HA, Kim YJ, Lee H, et al. Association of vitamin D concentrations with adiposity indices among preadolescent children in Korea. *J Pediatr Endocrinol Metab* 2013; **26**(9-10): 849-54.
© 2024 Zhang X et al. *JAMA Pediatrics.*

1445. Bae J, Joung H, Kim JY, Kwon KN, Kim Y, Park SW. Validity of self-reported height, weight, and body mass index of the Korea Youth Risk Behavior Web-based Survey questionnaire. *J Prev Med Public Health* 2010; **43**(5): 396-402.

1446. Lee B, Chung SJ, Lee SK, Yoon J. Validation of self-reported height and weight in fifth-grade Korean children. *Nutr Res Pract* 2013; **7**(4): 326-9.

1447. Choo J, Kim HJ, Park S. Neighborhood Environments: Links to Health Behaviors and Obesity Status in Vulnerable Children. *West J Nurs Res* 2017; **39**(8): 1169-91.

1448. Lee HJ, Kim SH, Jin MH, Lee JS. Variability in sociodemographic factors and obesity in Korean children: a cross-sectional analysis of Korea National Health and Nutrition Examination survey data (2007-2015). *Ann Epidemiol* 2020; **43**: 51-7.

1449. Lee HH, Park HA, Kang JH, et al. Factors related to body mass index and body mass index change in korean children: preliminary results from the obesity and metabolic disorders cohort in childhood. *Korean J Fam Med* 2012; **33**(3): 134-43.

1450. Kim E, Hwang JY, Woo EK, Kim SS, Jo SA, Jo I. Body mass index cutoffs for underweight, overweight, and obesity in South Korean schoolgirls. *Obesity research* 2005; **13**(9): 1510-4.

1451. Sunwoo JS, Yang KI, Kim JH, Koo DL, Kim D, Hong SB. Sleep duration rather than sleep timing is associated with obesity in adolescents. *Sleep Med* 2020; **68**: 184-9.

1452. Yoon HK, Kim GS, Kim S. Parental Factors Associated with Obesity in Korean Adolescents. *Int J Environ Res Public Health* 2020; **17**(14).

1453. Lee YJ, Shin YH, Kim JK, Shim JY, Kang DR, Lee HR. Metabolic syndrome and its association with white blood cell count in children and adolescents in Korea: the 2005 Korean National Health and Nutrition Examination Survey. *Nutr Metab Cardiovasc Dis* 2010; **20**(3): 165-72.

1454. Kim IK, Lee HJ, Kang JH, Song J. Effect of parental overweight and serum leptin levels on the manifestation of overweight in 7-year-old Korean children. *Public Health Nutr* 2010; **13**(3): 384-92.

1455. Lee G, Ha Y, Vann JJ, Choi E. Weight perception and dieting behavior among Korean adolescents. *J Sch Nurs* 2009; **25**(6): 427-35.

1456. Chang Y, Woo HY, Sung E, et al. Prevalence of acanthosis nigricans in relation to anthropometric measures: community-based cross-sectional study in Korean pre-adolescent school children. *Pediatr Int* 2008; **50**(5): 667-73.

1457. Ryu SY, Kweon SS, Park HC, Shin JH, Rhee JA. Obesity and the metabolic syndrome in Korean adolescents. *J Korean Med Sci* 2007; **22**(3): 513-7.

1458. Yoo S, Lee SY, Kim KN, Sung E. Obesity in Korean pre-adolescent school children: comparison of various anthropometric measurements based on bioelectrical impedance analysis. *Int J Obes (Lond)* 2006; **30**(7): 1086-90.

1459. Lee K, Kwon ER, Park TJ, Park MS, Lenders CM. Parental overweight as an indicator of childhood overweight: how sensitive? *Asia Pac J Clin Nutr* 2006; **15**(2): 196-200.

1460. Yoo S, Kim H, Lee J. Abnormal weight status and associated characteristics of low-income Korean children. *Asia Pac J Public Health* 2015; **27**(2): NP1093-105.

1461. Lee G, Ham OK. Factors Affecting Underweight and Obesity Among Elementary School Children in South Korea. *Asian Nurs Res (Korean Soc Nurs Sci)* 2015; **9**(4): 298-304.

1462. Kong IG, Lee HJ, Kim SY, Sim S, Choi HG. Physical Activity, Study Sitting Time, Leisure Sitting Time, and Sleep Time Are Differently Associated With Obesity in Korean Adolescents: A Population-Based Study. *Medicine (Baltimore)* 2015; **94**(44): e1965.

1463. Noh JW, Kim YE, Park J, Oh IH, Kwon YD. Impact of parental socioeconomic status on childhood and adolescent overweight and underweight in Korea. *J Epidemiol* 2014; **24**(3): 221-9.

© 2024 Zhang X et al. *JAMA Pediatrics.*

1464. Oh IH, Cho Y, Park SY, et al. Relationship between socioeconomic variables and obesity in Korean adolescents. *J Epidemiol* 2011; **21**(4): 263-70.
1465. Yoo S, Kim HB, Lee SY, et al. Association between obesity and the prevalence of allergic diseases, atopy, and bronchial hyperresponsiveness in Korean adolescents. *Int Arch Allergy Immunol* 2011; **154**(1): 42-8.
1466. Kim B, Lee CY, Kim HS, Ko IS, Park CG, Kim GS. Ecological risk factors of childhood obesity in Korean elementary school students. *West J Nurs Res* 2012; **34**(7): 952-72.
1467. Choi Y, You Y, Go KA, et al. The prevalence of obesity and the level of adherence to the Korean Dietary Action Guides in Korean preschool children. *Nutr Res Pract* 2013; **7**(3): 207-15.
1468. Kim GH, Shin SW, Lee J, et al. Red meat and chicken consumption and its association with high blood pressure and obesity in South Korean children and adolescents: a cross-sectional analysis of KSHES, 2011-2015. *Nutr J* 2017; **16**(1): 31.
1469. Cho WK, Han K, Ahn MB, et al. Metabolic risk factors in Korean adolescents with severe obesity: Results from the Korea National Health and Nutrition Examination Surveys (K-NHANES) 2007-2014. *Diabetes Res Clin Pract* 2018; **138**: 169-76.
1470. Ara I, Moreno LA, Leiva MT, Gutin B, Casajús JA. Adiposity, physical activity, and physical fitness among children from Aragón, Spain. *Obesity (Silver Spring)* 2007; **15**(8): 1918-24.
1471. Sanchez-Cruz JJ, Jimenez-Moleon JJ, Fernandez-Quesada F, Sanchez MJ. Prevalence of child and youth obesity in Spain in 2012. *Rev Esp Cardiol (Engl Ed)* 2013; **66**(5): 371-6.
1472. Gulias-Gonzalez R, Martinez-Vizcaino V, Garcia-Prieto JC, Diez-Fernandez A, Olivas-Bravo A, Sanchez-Lopez M. Excess of weight, but not underweight, is associated with poor physical fitness in children and adolescents from Castilla-La Mancha, Spain. *Eur J Pediatr* 2014; **173**(6): 727-35.
1473. Iguacel I, Escartin L, Fernandez-Alvira JM, et al. Early life risk factors and their cumulative effects as predictors of overweight in Spanish children. *Int J Public Health* 2018; **63**(4): 501-12.
1474. Lopez-Gil JF, Sanchez-Miguel PA, Tapia-Serrano MA, Garcia-Hermoso A. Skipping breakfast and excess weight among young people: the moderator role of moderate-to-vigorous physical activity. *Eur J Pediatr* 2022; **181**(8): 3195-204.
1475. Lasarte-Velillas JJ, Lamiquiz-Moneo I, Lasarte-Sanz I, et al. Prevalence of overweight and obesity in Aragon and variations according to health determinants. *An Pediatr (Engl Ed)* 2023; **98**(3): 157-64.
1476. de Bont J, Bennett M, Leon-Munoz LM, Duarte-Salles T. The prevalence and incidence rate of overweight and obesity among 2.5 million children and adolescents in Spain. *Rev Esp Cardiol (Engl Ed)* 2022; **75**(4): 300-7.
1477. Cartanya-Hueso A, Lidon-Moyano C, Martin-Sanchez JC, Gonzalez-Marron A, Perez-Martin H, Martinez-Sanchez JM. Association between recreational screen time and excess weight and obesity assessed with three sets of criteria in Spanish residents aged 2-14 years. *An Pediatr (Engl Ed)* 2022; **97**(5): 333-41.
1478. Cabeza JF, Aristizabal-Duque CH, Sanchez IMB, et al. Relationship between overweight and obesity and cardiac dimensions and function in a paediatric population. *Eur J Pediatr* 2022; **181**(5): 1943-9.
1479. Aragon-Martin R, Gomez-Sanchez MDM, Martinez-Nieto JM, et al. Independent and Combined Association of Lifestyle Behaviours and Physical Fitness with Body Weight Status in Schoolchildren. *Nutrients* 2022; **14**(6).
1480. Vega-Ramirez L, Perez-Canaveras RM, De Juan Herrero J. There Is an Inverse Correlation between Basic Motor Skills and Overweight in Schoolchildren Aged 8 to 12. *Children (Basel)* 2021; **8**(12).
1481. Barja-Fernandez S, Aguilera CM, Martinez-Silva I, et al. 25-Hydroxyvitamin D levels of children are inversely related to adiposity assessed by body mass index. *J Physiol Biochem* 2018; **74**(1): 111-8.
1482. Adelantado-Renau M, Jimenez-Pavon D, Beltran-Valls MR, Ponce-Gonzalez JG, Chiva-Bartoll O, Moliner-Urdiales D. Fitness and academic performance in adolescents. The mediating role of leptin: DADOS study. *Eur J Pediatr* 2018; **177**(10): 1555-63.

© 2024 Zhang X et al. *JAMA Pediatrics.*

1483. Salcedo Aguilar F, Martinez-Vizcaino V, Sanchez Lopez M, et al. Impact of an after-school physical activity program on obesity in children. *J Pediatr* 2010; **157**(1): 36-42 e3.
1484. Marcos-Pasero H, Aguilar-Aguilar E, de la Iglesia R, et al. Association of calcium and dairy product consumption with childhood obesity and the presence of a Brain Derived Neurotropic Factor-Antisense (BDNF-AS) polymorphism. *Clin Nutr* 2019; **38**(6): 2616-22.
1485. Baile JI, Guevara RM, Gonzalez-Calderon MJ, Urchaga JD. The Relationship between Weight Status, Health-Related Quality of Life, and Life Satisfaction in a Sample of Spanish Adolescents. *Int J Environ Res Public Health* 2020; **17**(9).
1486. Bawaked RA, Fernandez-Barres S, Navarrete-Munoz EM, et al. Impact of lifestyle behaviors in early childhood on obesity and cardiometabolic risk in children: Results from the Spanish INMA birth cohort study. *Pediatr Obes* 2020; **15**(3): e12590.
1487. Ajejas Bazan MJ, Jimenez-Trujillo MI, Warnberg J, Dominguez Fernandez S, Lopez-de-Andres A, Perez-Farinos N. Prevalence of childhood overweight/obesity in Spain 1993-2011 and associated risk factors in 2011 Prevalencia de sobrepeso y obesidad infa. *Nutr Hosp* 2018; **35**(1): 84-9.
1488. de Bont J, Casas M, Barrera-Gomez J, et al. Ambient air pollution and overweight and obesity in school-aged children in Barcelona, Spain. *Environ Int* 2019; **125**: 58-64.
1489. Tamayo-Ortiz M, Tellez-Rojo MM, Rothenberg SJ, et al. Exposure to PM(2.5) and Obesity Prevalence in the Greater Mexico City Area. *Int J Environ Res Public Health* 2021; **18**(5).
1490. Martin JJ, Hernandez LS, Gonzalez MG, Mendez CP, Rey Galan C, Guerrero SM. Trends in childhood and adolescent obesity prevalence in Oviedo (Asturias, Spain) 1992-2006. *Acta Paediatr* 2008; **97**(7): 955-8.
1491. Llargues E, Franco R, Recasens A, et al. Assessment of a school-based intervention in eating habits and physical activity in school children: the AVall study. *J Epidemiol Community Health* 2011; **65**(10): 896-901.
1492. Santiago S, Zazpe I, Marti A, Cuervo M, Martinez JA. Gender differences in lifestyle determinants of overweight prevalence in a sample of Southern European children. *Obes Res Clin Pract* 2013; **7**(5): e391-400.
1493. Riano-Galan I, Fernandez-Somoano A, Rodriguez-Dehli C, Valvi D, Vrijheid M, Tardon A. Proatherogenic Lipid Profile in Early Childhood: Association with Weight Status at 4 Years and Parental Obesity. *J Pediatr* 2017; **187**: 153-7 e2.
1494. Montero P. Nutritional assessment and diet quality of visually impaired Spanish children. *Ann Hum Biol* 2005; **32**(4): 498-512.
1495. Perez-Bonaventura I, Granero R, Ezpeleta L. The relationship between weight status and emotional and behavioral problems in Spanish preschool children. *J Pediatr Psychol* 2015; **40**(4): 455-63.
1496. Garcia-Marcos L, Arnedo Pena A, Busquets-Monge R, et al. How the presence of rhinoconjunctivitis and the severity of asthma modify the relationship between obesity and asthma in children 6-7 years old. *Clin Exp Allergy* 2008; **38**(7): 1174-8.
1497. Garcia-Marcos L, Valverde-Molina J, Ortega ML, Sanchez-Solis M, Martinez-Torres AE, Castro-Rodríguez JA. Percent body fat, skinfold thickness or body mass index for defining obesity or overweight, as a risk factor for asthma in schoolchildren: which one to use in epidemiological studies? *Matern Child Nutr* 2008; **4**(4): 304-10.
1498. Vazquez FL, Diaz O, Pomar C. Prevalence of overweight and obesity among preadolescent schoolchildren in Galicia, Spain. *Child Care Health Dev* 2010; **36**(3): 392-5.
1499. Albaladejo R, Villanueva R, Astasio P, Ortega P, Santos J, Regidor E. Sports facilities, socio-economic context and overweight among the childhood population in two southern European cities: a cross sectional study. *BMC Pediatr* 2019; **19**(1): 307.
1500. Scholz A, Navarrete-Munoz EM, Garcia-de-la-Hera M, et al. Association between trans fatty acid intake and overweight including obesity in 4 to 5-year-old children from the INMA study. *Pediatr Obes* 2019; **14**(9): e12528.
© 2024 Zhang X et al. *JAMA Pediatrics.*

1501. Esteban-Vasallo MD, Galan I, Ortiz-Pinto MA, et al. Accuracy of anthropometric measurements and weight status perceptions reported by parents of 4-year-old children. *Public Health Nutr* 2020; **23**(4): 589-98.
1502. Martinez-Gomez D, Eisenmann JC, Warnberg J, et al. Associations of physical activity, cardiorespiratory fitness and fatness with low-grade inflammation in adolescents: the AFINOS Study. *Int J Obes (Lond)* 2010; **34**(10): 1501-7.
1503. Bibiloni Mdel M, Martinez E, Llull R, Juarez MD, Pons A, Tur JA. Prevalence and risk factors for obesity in Balearic Islands adolescents. *Br J Nutr* 2010; **103**(1): 99-106.
1504. Larranaga N, Amiano P, Arrizabalaga JJ, Bidaurrazaga J, Gorostiza E. Prevalence of obesity in 4-18-year-old population in the Basque Country, Spain. *Obes Rev* 2007; **8**(4): 281-7.
1505. Ayala GX, Mickens L, Galindo P, Elder JP. Acculturation and body image perception among Latino youth. *Ethn Health* 2007; **12**(1): 21-41.
1506. Rojo L, Ruiz E, Dominguez JA, Calaf M, Livianos L. Comorbidity between obesity and attention deficit/hyperactivity disorder: population study with 13-15-year-olds. *Int J Eat Disord* 2006; **39**(6): 519-22.
1507. Morales-Suarez-Varela M, Rubio-Lopez N, Ruso C, et al. Anthropometric Status and Nutritional Intake in Children (6-9 Years) in Valencia (Spain): The ANIVA Study. *Int J Environ Res Public Health* 2015; **12**(12): 16082-95.
1508. Gutierrez-Zornoza M, Sanchez-Lopez M, Garcia-Hermoso A, Gonzalez-Garcia A, Chillon P, Martinez-Vizcaino V. Active commuting to school, weight status, and cardiometabolic risk in children from rural areas: the Cuenca study. *Health Educ Behav* 2015; **42**(2): 231-9.
1509. Alonso-Fernandez N, Jimenez-Garcia R, Alonso-Fernandez L, Hernandez-Barrera V, Palacios-Cena D. Health factors related to physical activity among children and adolescents: results from Spanish National Health Surveys 2006 and 2011/12. *J Spec Pediatr Nurs* 2015; **20**(3): 193-202.
1510. Veses AM, Martínez-Gómez D, Gómez-Martínez S, et al. Physical fitness, overweight and the risk of eating disorders in adolescents. The AVENA and AFINOS studies. *Pediatr Obes* 2014; **9**(1): 1-9.
1511. Milla Tobarra M, Martinez-Vizcaino V, Lahoz Garcia N, Garcia-Prieto JC, Arias-Palencia NM, Garcia-Hermoso A. The relationship between beverage intake and weight status in children: the Cuenca study. *Nutr Hosp* 2014; **30**(4): 818-24.
1512. Nova E, Martínez-Gómez D, Gómez-Martínez S, et al. Influence of health behaviours on the incidence of infection and allergy in adolescents: the AFINOS cross-sectional study. *BMC Public Health* 2014; **14**: 19.
1513. Dadvand P, Villanueva CM, Font-Ribera L, et al. Risks and benefits of green spaces for children: a cross-sectional study of associations with sedentary behavior, obesity, asthma, and allergy. *Environ Health Perspect* 2014; **122**(12): 1329-35.
1514. Rodríguez-Ramírez S, Mundo-Rosas V, García-Guerra A, Shamah-Levy T. Dietary patterns are associated with overweight and obesity in Mexican school-age children. *Arch Latinoam Nutr* 2011; **61**(3): 270-8.
1515. Cerrillo I, Fernández-Pachón MS, Ortega Mde L, et al. Two methods to determine the prevalence of overweight and obesity in 8-9 year-old-children in Seville, Spain. *Nutr Hosp* 2012; **27**(2): 463-8.
1516. Martinez-Vizcaino V, Solera Martinez M, Notario Pacheco B, et al. Trends in excess of weight, underweight and adiposity among Spanish children from 2004 to 2010: the Cuenca Study. *Public Health Nutr* 2012; **15**(12): 2170-4.
1517. Navalpotro L, Regidor E, Ortega P, Martinez D, Villanueva R, Astasio P. Area-based socioeconomic environment, obesity risk behaviours, area facilities and childhood overweight and obesity: socioeconomic environment and childhood overweight. *Prev Med* 2012; **55**(2): 102-7.
1518. Jimenez-Ormeno E, Aguado X, Delgado-Abellan L, Mecerreyes L, Alegre LM. Foot morphology in normal-weight, overweight, and obese schoolchildren. *Eur J Pediatr* 2013; **172**(5): 645-52.
1519. Laguna M, Ruiz JR, Gallardo C, Garcia-Pastor T, Lara MT, Aznar S. Obesity and physical activity patterns

© 2024 Zhang X et al. *JAMA Pediatrics.*

in children and adolescents. *J Paediatr Child Health* 2013; **49**(11): 942-9.
1520. Morales PF, Sanchez-Lopez M, Moya-Martinez P, et al. Health-related quality of life, obesity, and fitness in schoolchildren: the Cuenca study. *Qual Life Res* 2013; **22**(7): 1515-23.
1521. Moreno G, Johnson-Shelton D, Boles S. Prevalence and prediction of overweight and obesity among elementary school students. *J Sch Health* 2013; **83**(3): 157-63.
1522. Perez-Farinos N, Lopez-Sobaler AM, Dal Re MA, et al. The ALADINO study: a national study of prevalence of overweight and obesity in Spanish children in 2011. *Biomed Res Int* 2013; **2013**: 163687.
1523. Garcia-Garcia E, Vazquez-Lopez MA, Garcia-Fuentes E, et al. Thyroid Function and Thyroid Autoimmunity in Relation to Weight Status and Cardiovascular Risk Factors in Children and Adolescents: A Population-Based Study. *J Clin Res Pediatr Endocrinol* 2016; **8**(2): 157-62.
1524. Alvarez Zallo N, Aguinaga-Ontoso I, Alvarez-Alvarez I, Guillen-Grima F, Azcona San Julian C. The influence of gender and atopy in the relationship between obesity and asthma in childhood. *Allergol Immunopathol (Madr)* 2017; **45**(3): 227-33.
1525. Martin-Espinosa N, Diez-Fernandez A, Sanchez-Lopez M, et al. Prevalence of high blood pressure and association with obesity in Spanish schoolchildren aged 4-6 years old. *PLoS One* 2017; **12**(1): e0170926.
1526. Arias Ramos N, Calvo Sánchez MD, Fernández-Villa T, Ovalle Perandones MA, Fernández García D, Marqués-Sánchez P. Social exclusion of the adolescent with overweight: study of sociocentric social networks in the classroom. *Pediatr Obes* 2018; **13**(10): 614-20.
1527. Rathnayake KM, Satchithananthan A, Mahamithawa S, Jayawardena R. Early life predictors of preschool overweight and obesity: a case-control study in Sri Lanka. *BMC Public Health* 2013; **13**: 994.
1528. Wickramasinghe VP, Arambepola C, Bandara DM, et al. Validity of newly-developed BMI and waist cut-off values for Sri Lankan children. *Ann Hum Biol* 2013; **40**(3): 280-5.
1529. Warnakulasuriya LS, Fernando MAM, Adikaram AVN, et al. Assessment of Nutritional Status in Sri Lankan Children: Validity of Current Anthropometry Cutoffs? *Asia Pac J Public Health* 2019; **31**(7): 633-42.
1530. Wickramasinghe VP, Lamabadusuriya SP, Cleghorn GJ, Davies PS. Validity of currently used cutoff values of body mass index as a measure of obesity in Sri Lankan children. *Ceylon Med J* 2009; **54**(4): 114-9.
1531. Salman Z, Kirk GD, Deboer MD. High Rate of Obesity-Associated Hypertension among Primary Schoolchildren in Sudan. *Int J Hypertens* 2010; **2011**: 629492.
1532. Nagwa MA, Elhussein AM, Azza M, Abdulhadi NH. Alarming high prevalence of overweight/obesity among Sudanese children. *Eur J Clin Nutr* 2011; **65**(3): 409-11.
1533. Wallby T, Lagerberg D, Magnusson M. Relationship Between Breastfeeding and Early Childhood Obesity: Results of a Prospective Longitudinal Study from Birth to 4 Years. *Breastfeed Med* 2017; **12**: 48-53.
1534. Hamano T, Li X, Sundquist J, Sundquist K. Association between Childhood Obesity and Neighbourhood Accessibility to Fast-Food Outlets: A Nationwide 6-Year Follow-Up Study of 944,487 Children. *Obes Facts* 2017; **10**(6): 559-68.
1535. Onnestam L, Vad OH, Andersson T, Jolesjo A, Sandegard J, Bengtsson Bostrom K. Maternal body mass index in early pregnancy is associated with overweight and obesity in children up to 16 years of age. *PLoS One* 2022; **17**(10): e0275542.
1536. Faldt A, Nejat S, Edvinsson Sollander S, Durbeej N, Holmgren A. Increased incidence of overweight and obesity among preschool Swedish children during the COVID-19 pandemic. *Eur J Public Health* 2023; **33**(1): 127-31.
1537. Sjoberg RL, Nilsson KW, Leppert J. Obesity, shame, and depression in school-aged children: a population-based study. *Pediatrics* 2005; **116**(3): e389-92.
1538. Norberg C, Hallstrom Stalin U, Matsson L, Thorngren-Jerneck K, Klingberg G. Body mass index (BMI) and

dental caries in 5-year-old children from southern Sweden. *Community Dent Oral Epidemiol* 2012; **40**(4): 315-22.
1539. Nilsen BB, Yngve A, Monteagudo C, Tellström R, Scander H, Werner B. Reported habitual intake of breakfast and selected foods in relation to overweight status among seven- to nine-year-old Swedish children. *Scand J Public Health* 2017; **45**(8): 886-94.
1540. Moraeus L, Lissner L, Sjoberg A. Stable prevalence of obesity in Swedish schoolchildren from 2008 to 2013 but widening socio-economic gap in girls. *Acta Paediatr* 2014; **103**(12): 1277-84.
1541. Garmy P, Clausson EK, Nyberg P, Jakobsson U. Overweight and television and computer habits in Swedish school-age children and adolescents: a cross-sectional study. *Nurs Health Sci* 2014; **16**(2): 143-8.
1542. Sjoberg A, Lissner L, Albertsson-Wikland K, Marild S. Recent anthropometric trends among Swedish school children: evidence for decreasing prevalence of overweight in girls. *Acta Paediatr* 2008; **97**(1): 118-23.
1543. Marild S, Bondestam M, Bergstrom R, Ehnberg S, Hollsing A, Albertsson-Wikland K. Prevalence trends of obesity and overweight among 10-year-old children in western Sweden and relationship with parental body mass index. *Acta Paediatr* 2004; **93**(12): 1588-95.
1544. Mangrio E, Lindstrom M, Rosvall M. Early life factors and being overweight at 4 years of age among children in Malmo, Sweden. *BMC Public Health* 2010; **10**: 764.
1545. Koch FS, Sepa A, Ludvigsson J. Psychological stress and obesity. *J Pediatr* 2008; **153**(6): 839-44.
1546. Stenhammar C, Olsson G, Bahmanyar S, et al. Family stress and BMI in young children. *Acta Paediatr* 2010; **99**(8): 1205-12.
1547. Bornhorst C, Wijnhoven TM, Kunesova M, et al. WHO European Childhood Obesity Surveillance Initiative: associations between sleep duration, screen time and food consumption frequencies. *BMC Public Health* 2015; **15**: 442.
1548. Magnusson MB, Hulthén L, Kjellgren KI. Obesity, dietary pattern and physical activity among children in a suburb with a high proportion of immigrants. *J Hum Nutr Diet* 2005; **18**(3): 187-94.
1549. Erling A, Hwang CP. Body-esteem in Swedish 10-year-old children. *Percept Mot Skills* 2004; **99**(2): 437-44.
1550. Litsfeldt S, Ward TM, Hagell P, Garmy P. Association Between Sleep Duration, Obesity, and School Failure Among Adolescents. *J Sch Nurs* 2020; **36**(6): 458-63.
1551. Lager AC, Fossum B, Rorvall G, Bremberg SG. Children's overweight and obesity: local and national monitoring using electronic health records. *Scand J Public Health* 2009; **37**(2): 201-5.
1552. Guinhouya CB, Apete GK, Hubert H. Diagnostic quality of Actigraph-based physical activity cut-offs for children: what overweight/obesity references can tell? *Pediatr Int* 2009; **51**(4): 568-73.
1553. Bergstrom E, Blomquist HK. Is the prevalence of overweight and obesity declining among 4-year-old Swedish children? *Acta Paediatr* 2009; **98**(12): 1956-8.
1554. Blomquist HK, Bergstrom E. Obesity in 4-year-old children more prevalent in girls and in municipalities with a low socioeconomic level. *Acta Paediatr* 2007; **96**(1): 113-6.
1555. Moraeus L, Lissner L, Olsson L, Sjoberg A. Age and time effects on children's lifestyle and overweight in Sweden. *BMC Public Health* 2015; **15**: 355.
1556. Lindkvist M, Ivarsson A, Silfverdal SA, Eurenius E. Associations between toddlers' and parents' BMI, in relation to family socio-demography: a cross-sectional study. *BMC Public Health* 2015; **15**: 1252.
1557. Vaezghasemi M, Lindkvist M, Ivarsson A, Eurenius E. Overweight and lifestyle among 13-15 year olds: a cross-sectional study in northern Sweden. *Scand J Public Health* 2012; **40**(3): 221-8.
1558. Khanolkar AR, Sovio U, Bartlett JW, Wallby T, Koupil I. Socioeconomic and early-life factors and risk of being overweight or obese in children of Swedish- and foreign-born parents. *Pediatr Res* 2013; **74**(3): 356-63.
1559. de Munter JS, Friedl A, Lind S, et al. Stability in the prevalence of Swedish children who were overweight or obese in 2003 and 2011. *Acta Paediatr* 2016; **105**(10): 1173-80.
© 2024 Zhang X et al. *JAMA Pediatrics.*

1560. Winkvist A, Hulten B, Kim JL, et al. Dietary intake, leisure time activities and obesity among adolescents in Western Sweden: a cross-sectional study. *Nutr J* 2016; **15**: 41.
1561. Aeberli I, Ammann RS, Knabenhans M, Molinari L, Zimmermann MB. Decrease in the prevalence of paediatric adiposity in Switzerland from 2002 to 2007. *Public Health Nutr* 2010; **13**(6): 806-11.
1562. Aeberli I, Henschen I, Molinari L, Zimmermann MB. Stabilization of the prevalence of childhood obesity in Switzerland. *Swiss Med Wkly* 2010; **140**: w13046.
1563. Jeannot E, Mahler P, Elia N, Cerruti B, Chastonnay P. Sociodemographic and Economic Determinants of Overweight and Obesity for Public-school Children in Geneva State, Switzerland: A Cross-sectional Study. *Int J Prev Med* 2015; **6**: 39.
1564. Lasserre AM, Chiolero A, Cachat F, Paccaud F, Bovet P. Overweight in Swiss children and associations with children's and parents' characteristics. *Obesity (Silver Spring)* 2007; **15**(12): 2912-9.
1565. Zimmermann MB, Gübeli C, Püntener C, Molinari L. Overweight and obesity in 6-12 year old children in Switzerland. *Swiss Med Wkly* 2004; **134**(35-36): 523-8.
1566. Kochli S, Endes K, Bartenstein T, et al. Lung function, obesity and physical fitness in young children: The EXAMIN YOUTH study. *Respir Med* 2019; **159**: 105813.
1567. Aeberli I, Gut-Knabenhans M, Kusche-Ammann RS, Molinari L, Zimmermann MB. A composite score combining waist circumference and body mass index more accurately predicts body fat percentage in 6- to 13-year-old children. *Eur J Nutr* 2013; **52**(1): 247-53.
1568. Bonvin A, Barral J, Kakebeeke TH, et al. Weight status and gender-related differences in motor skills and in child care - based physical activity in young children. *BMC Pediatr* 2012; **12**: 23.
1569. Nasreddine L, Mehio-Sibai A, Mrayati M, Adra N, Hwalla N. Adolescent obesity in Syria: prevalence and associated factors. *Child Care Health Dev* 2010; **36**(3): 404-13.
1570. Chen YH, Chiou HY, Wang HY, Chung KH. Sex differences in the associations between psychological symptoms and tumor necrosis factor-alpha levels among obese and nonobese children aged 6-13 in Taiwan. *J Affect Disord* 2023; **320**: 241-6.
1571. Yen CE, Lin YY, Hu SW. Anthropometric Status, Diet, and Dental Caries among Schoolchildren. *Int J Environ Res Public Health* 2021; **18**(13).
1572. Wang SG, Yang SS, Chang SJ. Association Between Obesity and Lower Urinary Tract Symptoms Among Children and Adolescents: A Community-Based Study. *Front Pediatr* 2021; **9**: 609057.
1573. Chen YC, Chen PC, Hsieh WS, Portnov BA, Chen YA, Lee YL. Environmental factors associated with overweight and obesity in taiwanese children. *Paediatr Perinat Epidemiol* 2012; **26**(6): 561-71.
1574. Chu NF, Pan WH. Prevalence of obesity and its comorbidities among schoolchildren in Taiwan. *Asia Pac J Clin Nutr* 2007; **16 Suppl 2**: 601-7.
1575. Liou YM, Liou TH, Chang LC. Obesity among adolescents: sedentary leisure time and sleeping as determinants. *J Adv Nurs* 2010; **66**(6): 1246-56.
1576. Ting WH, Huang CY, Tu YK, Chien KL. Association between weight status and depressive symptoms in adolescents: role of weight perception, weight concern, and dietary restraint. *Eur J Pediatr* 2012; **171**(8): 1247-55.
1577. Tsai HJ, Tsai AC. The association of BMI and sedentary time with respiratory symptoms and asthma in 5th grade schoolchildren in Kaohsiung, Taiwan. *J Asthma* 2009; **46**(1): 9-15.
1578. Chen YC, Fan HY, Yang C, Hsieh RH, Pan WH, Lee YL. Assessing causality between childhood adiposity and early puberty: A bidirectional Mendelian randomization and longitudinal study. *Metabolism* 2019; **100**: 153961.
1579. Lai SW, Ng KC, Lin HF, Chen HL. Association between obesity and hyperlipidemia among children. *Yale J Biol Med* 2001; **74**(4): 205-10.
1580. Yen CF, Hsiao RC, Yen JY, et al. Body weight statuses and their sociodemographic correlates among
© 2024 Zhang X et al. *JAMA Pediatrics.*

adolescents in southern Taiwan: results using two sets of cutoff references. *J Adolesc Health* 2010; **46**(1): 62-9.
1581. Pu C, Chou YJ. Health ratings for underweight, overweight and obese adolescents: disparities between adolescent's own report and the parent's report. *Asia Pac J Clin Nutr* 2010; **19**(2): 180-7.
1582. Chang JH, Wang SH, Kuo CL, Shen HC, Hong YW, Lin LC. Prevalence of flexible flatfoot in Taiwanese school-aged children in relation to obesity, gender, and age. *Eur J Pediatr* 2010; **169**(4): 447-52.
1583. Chen JP, Chung MJ, Wang MJ. Flatfoot prevalence and foot dimensions of 5- to 13-year-old children in Taiwan. *Foot Ankle Int* 2009; **30**(4): 326-32.
1584. Chen LJ, Fox KR, Haase AM. Body shape dissatisfaction and obesity among Taiwanese adolescents. *Asia Pac J Clin Nutr* 2008; **17**(3): 457-60.
1585. Chang SJ, Chiang IN, Lin CD, Hsieh CH, Yang SS. Obese children at higher risk for having overactive bladder symptoms: a community-based study. *Neurourol Urodyn* 2015; **34**(2): 123-7.
1586. Lee JI, Yen CF. Associations between body weight and depression, social phobia, insomnia, and self-esteem among Taiwanese adolescents. *Kaohsiung J Med Sci* 2014; **30**(12): 625-30.
1587. Hsieh PL, Chen ML, Huang CM, Chen WC, Li CH, Chang LC. Physical activity, body mass index, and cardiorespiratory fitness among school children in Taiwan: a cross-sectional study. *Int J Environ Res Public Health* 2014; **11**(7): 7275-85.
1588. Chen KC, Tung LC, Tung CH, Yeh CJ, Yang JF, Wang CH. An investigation of the factors affecting flatfoot in children with delayed motor development. *Res Dev Disabil* 2014; **35**(3): 639-45.
1589. Chiang PH, Huang LY, Lo YT, Lee MS, Wahlqvist ML. Bidirectionality and gender differences in emotional disturbance associations with obesity among Taiwanese schoolchildren. *Res Dev Disabil* 2013; **34**(10): 3504-16.
1590. Liao Y, Chang SH, Miyashita M, et al. Associations between health-related physical fitness and obesity in Taiwanese youth. *J Sports Sci* 2013; **31**(16): 1797-804.
1591. Chen CM, Lou MF, Gau BS. Parental Body Mass Index Is Associated With Adolescent Obesity in Taiwan. *Res Nurs Health* 2016; **39**(6): 399-405.
1592. Chang S-H, Zhang Y, Lin C-T, et al. Childhood overweight/obesity and social inequality in peri-urban regions of Taipei. *Habitat International* 2018; **73**: 1-5.
1593. Mosha MV, Msuya SE, Kasagama E, Ayieko P, Todd J, Filteau S. Prevalence and correlates of overweight and obesity among primary school children in Kilimanjaro, Tanzania. *PLoS One* 2021; **16**(4): e0249595.
1594. Mosha TC, Fungo S. Prevalence of overweight and obesity among children aged 6-12 years in Dodoma and Kinondoni municipalities, Tanzania. *Tanzan J Health Res* 2010; **12**(1): 6-16.
1595. Mpembeni RN, Muhihi AJ, Maghembe M, et al. Overweight, obesity and perceptions about body weight among primary schoolchildren in Dar es Salaam, Tanzania. *Tanzan J Health Res* 2014; **16**(4): 304-11.
1596. Mwaikambo SA, Leyna GH, Killewo J, Simba A, Puoane T. Why are primary school children overweight and obese? A cross sectional study undertaken in Kinondoni district, Dar-es-salaam. *BMC Public Health* 2015; **15**: 1269.
1597. Pangani IN, Kiplamai FK, Kamau JW, Onywera VO. Prevalence of Overweight and Obesity among Primary School Children Aged 8-13 Years in Dar es Salaam City, Tanzania. *Adv Prev Med* 2016; **2016**: 1345017.
1598. Chomba H, Martin HD, Kimywe J. Prevalence and Predictors of Obesity among 7- to 17-Year-Old Schoolchildren in Urban Arusha, Tanzania. *J Nutr Metab* 2019; **2019**: 3106597.
1599. Shinsugi C, Mizumoto A. Associations of Nutritional Status with Full Immunization Coverage and Safe Hygiene Practices among Thai Children Aged 12-59 Months. *Nutrients* 2021; **14**(1).
1600. Rerksuppaphol L, Rerksuppaphol S. Prevalence and Risk Factors of Hypertension in Schoolchildren from Central Thailand: A Cross-Sectional Study. *Int J Prev Med* 2021; **12**: 28.
1601. Weraarchakul W, Weraarchakul W. Relationship between nutritional status and dental caries in elementary
© 2024 Zhang X et al. *JAMA Pediatrics.*

students, Samliam Municipal School, Khon Kaen Province, Thailand. *J Med Assoc Thail* 2017; **100**(8): 232-9.
1602. Nonboonyawat T, Pusanasuwannasri W, Chanrat N, et al. Prevalence and associates of obesity and overweight among school-age children in a rural community of Thailand. *Korean J Pediatr* 2019; **62**(5): 179-86.
1603. Pawloski LR, Ruchiwit M, Pakapong Y. A cross-sectional examination of growth indicators from Thai adolescent girls: evidence of obesity among Thai youth? *Ann Hum Biol* 2008; **35**(4): 378-85.
1604. Manandhar S, Suksaroj TT, Rattanapan C. The Association between Green Space and the Prevalence of Overweight/Obesity among Primary School Children. *Int J Occup Environ Med* 2019; **10**(1): 1-10.
1605. Sengmeuang P, Kukongviriyapan U, Pasurivong O, Jones C, Khrisanapant W. Prevalence of obesity among Thai schoolchildren: a survey in Khon Kaen, Northeast Thailand. *Asian Biomedicine* 2010; **4**(6): 965-70.
1606. Rerksuppaphol S, Rerksuppaphol L. Prevalence of overweight and obesity among school children in suburb Thailand defined by the International Obesity Task Force Standard. [corrected]. *J Med Assoc Thai* 2010; **93 Suppl 2**: S27-31.
1607. Narksawat K, Tonmukayakul U, Boonthum A. Association between nutritional status and dental caries in permanent dentition among primary schoolchildren aged 12-14 years, Thailand. *Southeast Asian J Trop Med Public Health* 2009; **40**(2): 338-44.
1608. Rerksuppaphol S, Rerksuppaphol L. Association of obesity with the prevalence of hypertension in school children from central Thailand. *J Res Health Sci* 2015; **15**(1): 17-21.
1609. Sukhonthachit P, Aekplakorn W, Hudthagosol C, Sirikulchayanonta C. The association between obesity and blood pressure in Thai public school children. *BMC Public Health* 2014; **14**: 729.
1610. Firestone R, Punpuing S, Peterson KE, Acevedo-Garcia D, Gortmaker SL. Child overweight and undernutrition in Thailand: is there an urban effect? *Soc Sci Med* 2011; **72**(9): 1420-8.
1611. Jitnarin N, Kosulwat V, Rojroongwasinkul N, Boonpraderm A, Haddock CK, Poston WS. Prevalence of overweight and obesity in Thai population: results of the National Thai Food Consumption Survey. *Eat Weight Disord* 2011; **16**(4): e242-9.
1612. Rojroongwasinkul N, Kijboonchoo K, Wimonpeerapattana W, et al. SEANUTS: the nutritional status and dietary intakes of 0.5-12-year-old Thai children. *Br J Nutr* 2013; **110 Suppl 3**: S36-44.
1613. Thasanasuwan W, Srichan W, Kijboonchoo K, et al. Low Sleeping Time, High TV Viewing Time, and Physical Inactivity in School Are Risk Factors for Obesity in Pre-Adolescent Thai Children. *J Med Assoc Thai* 2016; **99**(3): 314-21.
1614. Sagbo H, Ekouevi DK, Ranjandriarison DT, et al. Prevalence and factors associated with overweight and obesity among children from primary schools in urban areas of Lome, Togo. *Public Health Nutr* 2018; **21**(6): 1048-56.
1615. Smith BJ, Phongsavan P, Havea D, Halavatau V, Chey T. Body mass index, physical activity and dietary behaviours among adolescents in the Kingdom of Tonga. *Public Health Nutr* 2007; **10**(2): 137-44.
1616. Simeon DT, Rattan RD, Panchoo K, Kungeesingh KV, Ali AC, Abdool PS. Body image of adolescents in a multi-ethnic Caribbean population. *Eur J Clin Nutr* 2003; **57**(1): 157-62.
1617. Mumena WA, Francis-Granderson I, Phillip LE, Gray-Donald K. Rapid increase of overweight and obesity among primary school-aged children in the Caribbean; high initial BMI is the most significant predictor. *BMC Obes* 2018; **5**: 4.
1618. Boukthir S, Essaddam L, Mazigh Mrad S, et al. Prevalence and risk factors of overweight and obesity in elementary schoolchildren in the metropolitan region of Tunis, Tunisia. *Tunis Med* 2011; **89**(1): 50-4.
1619. Ercan S, Dallar YB, Onen S, Engiz O. Prevalence of obesity and associated risk factors among adolescents in Ankara, Turkey. *J Clin Res Pediatr Endocrinol* 2012; **4**(4): 204-7.
1620. Salman H, Gursoy Koca T, Dereci S, Akcam M. Comparison of Body Composition and Body Mass Index in

© 2024 Zhang X et al. *JAMA Pediatrics.*

the Determination of Obesity in Schoolchildren. *Turk Arch Pediatr* 2022; **57**(5): 506-10.
1621. Meydanlioglu A, Akcan A, Oncel S, et al. Prevalence of obesity and hypertension in children and determination of associated factors by CHAID analysis. *Arch Pediatr* 2022; **29**(1): 30-5.
1622. Gunalan E, Bakir BO, Bali R, Tanriover O, Gemici B. Evaluation of body mass index and related lifestyle factors among 14-17-year-old Turkish adolescents. *North Clin Istanb* 2021; **8**(3): 226-35.
1623. Arslan HN, Dundar C, Terzi O. Prevalence of overweight and obesity among school children and parents: a cross-sectional study. *Rural Remote Health* 2021; **21**(4): 6773.
1624. Acar Tek N, Sanlier N, Turkozu D. The prevalence of abdominal obesity is remarkable for underweight and normal weight adolescent girls. *Turk J Med Sci* 2017; **47**(4): 1191-7.
1625. Saglam H, Tarim O. Prevalence and correlates of obesity in schoolchildren from the city of Bursa, Turkey. *J Clin Res Pediatr Endocrinol* 2008; **1**(2): 80-8.
1626. Sur H, Kolotourou M, Dimitriou M, et al. Biochemical and behavioral indices related to BMI in schoolchildren in urban Turkey. *Prev Med* 2005; **41**(2): 614-21.
1627. Gundogdu Z. Relationship between BMI and blood pressure in girls and boys. *Public Health Nutr* 2008; **11**(10): 1085-8.
1628. Turkkahraman D, Bircan I, Tosun O, Saka O. Prevalence and risk factors of obesity in school children in Antalya, Turkey. *Saudi medical journal* 2006; **27**: 1028-33.
1629. Nur N, Cetinkaya S, Yilmaz A, Ayvaz A, Bulut MO, Sümer H. Prevalence of hypertension among high school students in a middle Anatolian province of Turkey. *J Health Popul Nutr* 2008; **26**(1): 88-94.
1630. Bayat M, Erdem E, Barik O, Başer M, Taşci S. Blood pressure, height, weight and body mass index of primary school students in a low socio-economic district in Turkey. *Int Nurs Rev* 2009; **56**(3): 375-80.
1631. Discigil G, Tekin N, Soylemez A. Obesity in Turkish children and adolescents: prevalence and non-nutritional correlates in an urban sample. *Child Care Health Dev* 2009; **35**(2): 153-8.
1632. Etiler N, Cizmecioglu FM, Hatun S, Hamzaoglu O. Nutritional status of students in Kocaeli, Turkey: a population-based study. *Pediatr Int* 2011; **53**(2): 231-5.
1633. Ozmen D, Ozmen E, Ergin D, et al. The association of self-esteem, depression and body satisfaction with obesity among Turkish adolescents. *BMC Public Health* 2007; **7**: 80.
1634. Simsek E, Akpinar S, Bahcebasi T, Senses DA, Kocabay K. The prevalence of overweight and obese children aged 6-17 years in the West Black Sea region of Turkey. *Int J Clin Pract* 2008; **62**(7): 1033-8.
1635. Yuca SA, Yilmaz C, Cesur Y, Dogan M, Kaya A, Basaranoglu M. Prevalence of overweight and obesity in children and adolescents in eastern Turkey. *J Clin Res Pediatr Endocrinol* 2010; **2**(4): 159-63.
1636. Pirincci E, Durmus B, Gundogdu C, Acik Y. Prevalence and risk factors of overweight and obesity among urban school children in Elazig city, Eastern Turkey, 2007. *Ann Hum Biol* 2010; **37**(1): 44-56.
1637. Duzova A, Yalcinkaya F, Baskin E, Bakkaloglu A, Soylemezoglu O. Prevalence of hypertension and decreased glomerular filtration rate in obese children: results of a population-based field study. *Nephrol Dial Transplant* 2013; **28 Suppl 4**: iv166-71.
1638. Demirci H, Nuhoglu C, Ursavas IS, Isildak S, Basaran EO, Kilic MY. Obesity and asymptomatic hypertension among children aged 6-13 years living in Bursa, Turkey. *Fam Pract* 2013; **30**(6): 629-33.
1639. Polat M, Yikilkan H, Aypak C, Gorpelioglu S. The relationship between BMI and blood pressure in children aged 7-12 years in Ankara, Turkey. *Public Health Nutr* 2014; **17**(11): 2419-24.
1640. Gokler ME, Bugrul N, Metintas S, Kalyoncu C. Adolescent obesity and associated cardiovascular risk factors of rural and urban life (Eskisehir, Turkey). *Cent Eur J Public Health* 2015; **23**(1): 20-5.
1641. Agirbasli M, Adabag S, Ciliv G. Secular trends of blood pressure, body mass index, lipids and fasting glucose among children and adolescents in Turkey. *Clin Obes* 2011; **1**(4-6): 161-7.
© 2024 Zhang X et al. *JAMA Pediatrics.*

1642. Manios Y, Kolotourou M, Moschonis G, et al. Macronutrient intake, physical activity, serum lipids and increased body weight in primary schoolchildren in Istanbul. *Pediatr Int* 2005; **47**(2): 159-66.
1643. Yardim MS, Ozcebe LH, Araz OM, et al. Prevalence of childhood obesity and related parental factors across socioeconomic strata in Ankara, Turkey. *East Mediterr Health J* 2019; **25**(6): 374-84.
1644. Canan F, Yildirim O, Ustunel TY, et al. The relationship between internet addiction and body mass index in Turkish adolescents. *Cyberpsychol Behav Soc Netw* 2014; **17**(1): 40-5.
1645. Dundar C, Oz H. Obesity-related factors in Turkish school children. *ScientificWorldJournal* 2012; **2012**: 353485.
1646. Gültekin T, Akin G, Ozer BK. Gender differences in fat patterning in children living in Ankara. *Anthropol Anz* 2005; **63**(4): 427-37.
1647. Uçkun-Kitapçi A, Teziç T, Firat S, et al. Obesity and type 2 diabetes mellitus: a population-based study of adolescents. *J Pediatr Endocrinol Metab* 2004; **17**(12): 1633-40.
1648. Oner N, Vatansever U, Sari A, et al. Prevalence of underweight, overweight and obesity in Turkish adolescents. *Swiss Med Wkly* 2004; **134**(35-36): 529-33.
1649. ArdiÇ C, Omar E. Obesity frequency and related risk factors in primary school children. *The European Research Journal* 2019; **5**(3): 467-72.
1650. Asut O, Abuduxike G, Acar-Vaizoglu S, Cali S. Relationships between screen time, internet addiction and other lifestyle behaviors with obesity among secondary school students in the Turkish Republic of Northern Cyprus. *Turk J Pediatr* 2019; **61**(4): 568-79.
1651. Çelmeli G, Çürek Y, Arslan Gülten Z, et al. Remarkable Increase in the Prevalence of Overweight and Obesity Among School Age Children in Antalya, Turkey, Between 2003 and 2015. *J Clin Res Pediatr Endocrinol* 2019; **11**(1): 76-81.
1652. Comba A, Demir E, Baris Eren N. Nutritional status and related factors of schoolchildren in Corum, Turkey. *Public Health Nutr* 2019; **22**(1): 122-31.
1653. Deniz S, Oguzoncul AF. The prevalence of obesity and related factors among primary and secondary school students. *Niger J Clin Pract* 2019; **22**(12): 1685-92.
1654. Karakus B, Save D, Ates M, Kolasayin M, Tuncekin İ. Prevalence of obesity and overweight among primary school children in a district of Istanbul, Turkey. *Marmara Medical Journal* 2019; **32**(2): 76-80.
1655. Arikan S, Kamuk YU, Revan S. The Prevalence of Overweight and Obesity among Students between the Ages of 6 and 15 years in Konya. *Cyprus Journal of Medical Sciences* 2020; **5**(3): 234-8.
1656. Esenay FI, Yigit R, Erdogan S. Turkish mothers' perceptions of their children's weight. *J Spec Pediatr Nurs* 2010; **15**(2): 144-53.
1657. Ucar S, Dizdarer C, Darcan S, et al. Prevalence of obesity and overweight among children in İzmir, Turkey: Effects of nutritional and socio-economic factors. *Obesity and Metabolism* 2009; **5**: 99-106.
1658. Garipagaoglu M, Budak N, Sut N, Akdikmen O, Oner N, Bundak R. Obesity risk factors in Turkish children. *J Pediatr Nurs* 2009; **24**(4): 332-7.
1659. Dinç G, Saatli G, Baydur H, Ozcan C. Hypertension and overweight among Turkish adolescents in a city in Aegean region of Turkey: a strong relationship in a population with a relatively low prevalence of overweight. *Anadolu Kardiyol Derg* 2009; **9**(6): 450-6.
1660. ÖNsÜZ FM, DemİR F. Prevalence of hypertension and its association with obesity among schoolchildren aged 6–15 living in Sakarya Province in Turkey. *Turkish Journal of Medical Sciences* 2015; **45**: 907-12.
1661. Meseri R, Mermer G, Ergin I, Hassoy H. Evaluation of obesity prevalence and nutritional knowledge in adolescents in a semi urban area of Turkey. *Progress in Nutrition* 2015; **17**.
1662. Inal S, Canbulat Şahiner N, Bozkurt G. The effects of healthy lifestyle behaviors of mothers on obesity in
© 2024 Zhang X et al. *JAMA Pediatrics.*

preschool children. *JPMA The Journal of the Pakistan Medical Association* 2015; **65**: 1079-84.
1663. Vehapoglu A, Yazici M, Demir AD, Turkmen S, Nursoy M, Ozkaya E. Early infant feeding practice and childhood obesity: the relation of breast-feeding and timing of solid food introduction with childhood obesity. *J Pediatr Endocrinol Metab* 2014; **27**(11-12): 1181-7.
1664. CABAR H, ÖZEREN G, Altay B. Nutritional habits and obesity: Primary school students in Sinop, the northernmost point of Turkey. *Journal of Experimental and Clinical Medicine* 2014; **31**(1): 13-7.
1665. Ayyildiz TK, Kurtuncu M, Kulakci H, Celik S. Factors affecting the prevalence of obesity among primary school students in Turkey. *Iran Red Crescent Med J* 2014; **16**(12): e17785.
1666. Çalışır H, Karaçam Z. The prevalence of overweight and obesity in primary schoolchildren and its correlation with sociodemographic factors in Aydin, Turkey. *International Journal of Nursing Practice* 2011; **17**(2): 166-73.
1667. Cinar AB, Murtomaa H. Interrelation between obesity, oral health and life-style factors among Turkish school children. *Clin Oral Investig* 2011; **15**(2): 177-84.
1668. Akca SO, Uysal G, Aysegul Buyukgonenc L. Obesity in Nursery School Children in Corum, Turkey. *Iran Red Crescent Med J* 2016; **18**(10): e27734.
1669. Geckil E, Aslan S, Derya Ister E, Kucukkelpçe Simsek D, Sahin T. Prevalence and Risk Factors of Obesity and Overweight in Elementary School-Age (5 to 15 Years Old) Children in South-eastern Turkey. *Iranian Journal of Pediatrics* 2017; **27**(2).
1670. Gül A, Özer S, Yılmaz R, et al. Prevalence of Proteinuria in School-Aged Turkish Children, and Its Association with Obesity and Hypertension. *The Journal of Pediatric Research* 2017: 195-9.
1671. Eker HH, Tasdemir M, Mercan S, et al. Obesity in adolescents and the risk factors. *Turk J Phys Med Rehabil* 2018; **64**(1): 37-45.
1672. Haney MO. The relationship between BMI and blood pressure in school-age children in Izmir, Turkey. *PROGRESS IN NUTRITION* 2018; **20**(3): 372-7.
1673. Deren K, Wyszynska J, Nyankovskyy S, et al. Assessment of body mass index in a pediatric population aged 7-17 from Ukraine according to various international criteria-A cross-sectional study. *PLoS One* 2020; **15**(12): e0244300.
1674. Deren K, Nyankovskyy S, Nyankovska O, et al. The prevalence of underweight, overweight and obesity in children and adolescents from Ukraine. *Sci Rep* 2018; **8**(1): 3625.
1675. AlBlooshi A, Shaban S, AlTunaiji M, et al. Increasing obesity rates in school children in United Arab Emirates. *Obes Sci Pract* 2016; **2**(2): 196-202.
1676. Cheikh Ismail L, Al Dhaheri AS, Ibrahim S, et al. Nutritional status and adequacy of feeding Practices in Infants and Toddlers 0-23.9 months living in the United Arab Emirates (UAE): findings from the feeding Infants and Toddlers Study (FITS) 2020. *BMC Public Health* 2022; **22**(1): 319.
1677. Abduelkarem AR, Sharif SI, Bankessli FG, Kamal SA, Kulhasan NM, Hamrouni AM. Obesity and its associated risk factors among school-aged children in Sharjah, UAE. *PLoS One* 2020; **15**(6): e0234244.
1678. Abdullatif M, AlAbady K, Altheeb A, Rishmawi F, Jaradat H, Farooq S. Prevalence of Overweight, Obesity, and Dietary Behaviors among Adolescents in Dubai Schools: A Complex Design Survey 2019. *Dubai Medical Journal* 2021; **5**(1): 1-9.
1679. Aburawi EH, Al Hamad S, Yasin J, Almekhaini LA, Souid AK. Dyslipidemia, subclinical inflammation, hepatic cholestasis and endothelial dysfunction in schoolchildren with excess fat: A study from the United Arab Emirates. *PLoS One* 2019; **14**(1): e0210316.
1680. bin Zaal AA, Musaiger AO, D'Souza R. Dietary habits associated with obesity among adolescents in Dubai, United Arab Emirates. *Nutr Hosp* 2009; **24**(4): 437-44.
© 2024 Zhang X et al. *JAMA Pediatrics.*

1681. Fatima Q, Kashif M, Ghazi L. PREVALENCE OF OVERWEIGHT AND OBESITY AMONG PRIMARY SCHOOL CHILDREN IN ABU DHABI. *INDO AMERICAN JOURNAL OF PHARMACEUTICAL SCIENCES* 2018; **5**(12): 14337-44.
1682. Adab P, Pallan MJ, Lancashire ER, et al. Effectiveness of a childhood obesity prevention programme delivered through schools, targeting 6 and 7 year olds: cluster randomised controlled trial (WAVES study). *BMJ* 2018; **360**: k211.
1683. Sweeting H, Wright C, Minnis H. Psychosocial correlates of adolescent obesity, 'slimming down' and 'becoming obese'. *J Adolesc Health* 2005; **37**(5): 409.
1684. Fraser LK, Clarke GP, Cade JE, Edwards KL. Fast food and obesity: a spatial analysis in a large United Kingdom population of children aged 13-15. *Am J Prev Med* 2012; **42**(5): e77-85.
1685. Gilliland JA, Rangel CY, Healy MA, et al. Linking childhood obesity to the built environment: a multi-level analysis of home and school neighbourhood factors associated with body mass index. *Can J Public Health* 2012; **103**(9 Suppl 3): eS15-21.
1686. Uerlich MF, Baker SR, Day PF, Brown L, Vettore MV. Common Determinants of Dental Caries and Obesity in Children: A Multi-Ethnic Nested Birth Cohort Study in the United Kingdom. *Int J Environ Res Public Health* 2021; **18**(23).
1687. Ralphs E, Pembrey L, West J, Santorelli G. Association between mode of delivery and body mass index at 4-5 years in White British and Pakistani children: the Born in Bradford birth cohort. *BMC Public Health* 2021; **21**(1): 987.
1688. Adab P, Pallan MJ, Cade J, et al. Preventing childhood obesity, phase II feasibility study focusing on South Asians: BEACHeS. *BMJ Open* 2014; **4**(4): e004579.
1689. Bartle NC, Hill C, Webber L, van Jaarsveld CH, Wardle J. Emergence and persistence of overweight and obesity in 7- to 11-year-old children. *Obes Facts* 2013; **6**(5): 415-23.
1690. Pearce A, Li L, Abbas J, et al. Is childcare associated with the risk of overweight and obesity in the early years? Findings from the UK Millennium Cohort Study. *Int J Obes (Lond)* 2010; **34**(7): 1160-8.
1691. Harding S, Teyhan A, Maynard MJ, Cruickshank JK. Ethnic differences in overweight and obesity in early adolescence in the MRC DASH study: the role of adolescent and parental lifestyle. *Int J Epidemiol* 2008; **37**(1): 162-72.
1692. Bonuck K, Chervin RD, Howe LD. Sleep-disordered breathing, sleep duration, and childhood overweight: a longitudinal cohort study. *J Pediatr* 2015; **166**(3): 632-9.
1693. Steele RM, van Sluijs EM, Cassidy A, Griffin SJ, Ekelund U. Targeting sedentary time or moderate- and vigorous-intensity activity: independent relations with adiposity in a population-based sample of 10-y-old British children. *Am J Clin Nutr* 2009; **90**(5): 1185-92.
1694. Basterfield L, Jones AR, Parkinson KN, et al. Physical activity, diet and BMI in children aged 6-8 years: a cross-sectional analysis. *BMJ Open* 2014; **4**(6): e005001.
1695. Griffiths C, Frearson A, Taylor A, Radley D, Cooke C. A cross sectional study investigating the association between exposure to food outlets and childhood obesity in Leeds, UK. *Int J Behav Nutr Phys Act* 2014; **11**: 138.
1696. Green MA, Hobbs M, Ding D, et al. The Association between Fast Food Outlets and Overweight in Adolescents Is Confounded by Neighbourhood Deprivation: A Longitudinal Analysis of the Millennium Cohort Study. *International Journal of Environmental Research and Public Health* 2021; **18**(24).
1697. Scott FI, Horton DB, Mamtani R, et al. Administration of Antibiotics to Children Before Age 2 Years Increases Risk for Childhood Obesity. *Gastroenterology* 2016; **151**(1): 120-9 e5.
1698. Clemente DBP, Maitre L, Bustamante M, et al. Obesity is associated with shorter telomeres in 8 year-old children. *Sci Rep* 2019; **9**(1): 18739.

1699. Griffiths LJ, Dezateux C, Hill A. Is obesity associated with emotional and behavioural problems in children? Findings from the Millennium Cohort Study. *Int J Pediatr Obes* 2011; **6**(2-2): e423-32.
1700. Tiffin PA, Arnott B, Moore HJ, Summerbell CD. Modelling the relationship between obesity and mental health in children and adolescents: findings from the Health Survey for England 2007. *Child Adolesc Psychiatry Ment Health* 2011; **5**: 31.
1701. Mendez MA, Torrent M, Ferrer C, Ribas-Fitó N, Sunyer J. Maternal smoking very early in pregnancy is related to child overweight at age 5-7 y. *Am J Clin Nutr* 2008; **87**(6): 1906-13.
1702. Harrison F, Bentham G, Jones AP, Cassidy A, van Sluijs EM, Griffin SJ. School level correlates with adiposity in 9-10 year old children. *Health Place* 2011; **17**(3): 710-6.
1703. Taylor SJ, Viner R, Booy R, et al. Ethnicity, socio-economic status, overweight and underweight in East London adolescents. *Ethn Health* 2005; **10**(2): 113-28.
1704. Cecil JE, Watt P, Murrie IS, et al. Childhood obesity and socioeconomic status: a novel role for height growth limitation. *Int J Obes (Lond)* 2005; **29**(10): 1199-203.
1705. Fletcher ES, Rugg-Gunn AJ, Matthews JN, et al. Changes over 20 years in macronutrient intake and body mass index in 11- to 12-year-old adolescents living in Northumberland. *Br J Nutr* 2004; **92**(2): 321-33.
1706. Webster-Gandy J, Warren J, Henry CJ. Sexual dimorphism in fat patterning in a sample of 5 to 7-year-old children in Oxford. *Int J Food Sci Nutr* 2003; **54**(6): 467-71.
1707. Whitaker KL, Jarvis MJ, Beeken RJ, Boniface D, Wardle J. Comparing maternal and paternal intergenerational transmission of obesity risk in a large population-based sample. *Am J Clin Nutr* 2010; **91**(6): 1560-7.
1708. Thomas NE, Williams DRR, Rowe DA, Davies B, Baker JS. Evidence of secular changes in physical activity and fitness, but not adiposity and diet, in Welsh 12–13 year olds. *Health Education Journal* 2010; **69**(4): 381-9.
1709. Skidmore P, Welch A, van Sluijs E, et al. Impact of neighbourhood food environment on food consumption in children aged 9-10 years in the UK SPEEDY (Sport, Physical Activity and Eating behaviour: Environmental Determinants in Young people) study. *Public Health Nutr* 2010; **13**(7): 1022-30.
1710. Jennings A, Davies GJ, Costarelli V, Dettmar PW. Bowel habit, diet and body weight in preadolescent children. *J Hum Nutr Diet* 2010; **23**(5): 511-9.
1711. Edwards KL, Cade JE, Ransley JK, Clarke GP. A cross-sectional study examining the pattern of childhood obesity in Leeds: affluence is not protective. *Arch Dis Child* 2010; **95**(2): 94-9.
1712. Williamson DA, Champagne CM, Han H, et al. Increased obesity in children living in rural communities of Louisiana. *Int J Pediatr Obes* 2009; **4**(3): 160-5.
1713. Webber L, Hill C, Saxton J, Van Jaarsveld CH, Wardle J. Eating behaviour and weight in children. *Int J Obes (Lond)* 2009; **33**(1): 21-8.
1714. Standley R, Sullivan V, Wardle J. Self-perceived weight in adolescents: over-estimation or under-estimation? *Body Image* 2009; **6**(1): 56-9.
1715. Nelson NM, Woods CB. Obesogenic environments: Are neighbourhood environments that limit physical activity obesogenic? *Health Place* 2009; **15**(4): 917-24.
1716. McCullough N, Muldoon O, Dempster M. Self-perception in overweight and obese children: a cross-sectional study. *Child Care Health Dev* 2009; **35**(3): 357-64.
1717. Fox CL, Farrow CV. Global and physical self-esteem and body dissatisfaction as mediators of the relationship between weight status and being a victim of bullying. *J Adolesc* 2009; **32**(5): 1287-301.
1718. Fairclough SJ, Boddy LM, Hackett AF, Stratton G. Associations between children's socioeconomic status, weight status, and sex, with screen-based sedentary behaviours and sport participation. *Int J Pediatr Obes* 2009;
© 2024 Zhang X et al. *JAMA Pediatrics.*

4(4): 299-305.
1719. van Sluijs EM, Skidmore PM, Mwanza K, et al. Physical activity and dietary behaviour in a population-based sample of British 10-year old children: the SPEEDY study (Sport, Physical activity and Eating behaviour: environmental Determinants in Young people). *BMC Public Health* 2008; **8**: 388.
1720. Harding S, Maynard MJ, Cruickshank K, Teyhan A. Overweight, obesity and high blood pressure in an ethnically diverse sample of adolescents in Britain: the Medical Research Council DASH study. *Int J Obes (Lond)* 2008; **32**(1): 82-90.
1721. Routh K, Rao JN, Denley J. A simple, and potentially low-cost method for measuring the prevalence of childhood obesity. *Child Care Health Dev* 2006; **32**(2): 239-45.
1722. Mutunga M, Gallagher AM, Boreham C, et al. Socioeconomic differences in risk factors for obesity in adolescents in Northern Ireland. *Int J Pediatr Obes* 2006; **1**(2): 114-9.
1723. Solmi F, Morris S. Association between childhood obesity and use of regular medications in the UK: longitudinal cohort study of children aged 5-11 years. *BMJ Open* 2015; **5**(6): e007373.
1724. Jackson SE, Johnson F, Croker H, Wardle J. Weight perceptions in a population sample of English adolescents: cause for celebration or concern? *Int J Obes (Lond)* 2015; **39**(10): 1488-93.
1725. Falconer CL, Park MH, Croker H, et al. Can the relationship between ethnicity and obesity-related behaviours among school-aged children be explained by deprivation? A cross-sectional study. *BMJ Open* 2014; **4**(1): e003949.
1726. Pallan MJ, Hiam LC, Duda JL, Adab P. Body image, body dissatisfaction and weight status in South Asian children: a cross-sectional study. *BMC Public Health* 2011; **11**: 21.
1727. Coulthard JD, Pot GK. The timing of the evening meal: how is this associated with weight status in UK children? *Br J Nutr* 2016; **115**(9): 1616-22.
1728. Beynon C, Fone D. Risk factors for childhood obesity: a data analysis of the Welsh Health Survey. *Nurs Child Young People* 2017; **29**(6): 38-44.
1729. Hudda MT, Nightingale CM, Donin AS, et al. Patterns of childhood body mass index (BMI), overweight and obesity in South Asian and black participants in the English National child measurement programme: effect of applying BMI adjustments standardising for ethnic differences in BMI-body fatness associations. *Int J Obes (Lond)* 2018; **42**(4): 662-70.
1730. Wang J, Iannotti RJ, Luk JW. Bullying victimization among underweight and overweight U.S. youth: differential associations for boys and girls. *J Adolesc Health* 2010; **47**(1): 99-101.
1731. Chiasson MA, Scheinmann R, Hartel D, et al. Predictors of Obesity in a Cohort of Children Enrolled in WIC as Infants and Retained to 3 Years of Age. *J Community Health* 2016; **41**(1): 127-33.
1732. Ehrenthal DB, Wu P, Trabulsi J. Differences in the Protective Effect of Exclusive Breastfeeding on Child Overweight and Obesity by Mother's Race. *Matern Child Health J* 2016; **20**(9): 1971-9.
1733. Taveras EM, Rifas-Shiman SL, Scanlon KS, Grummer-Strawn LM, Sherry B, Gillman MW. To what extent is the protective effect of breastfeeding on future overweight explained by decreased maternal feeding restriction? *Pediatrics* 2006; **118**(6): 2341-8.
1734. Weden MM, Brownell P, Rendall MS. Prenatal, perinatal, early life, and sociodemographic factors underlying racial differences in the likelihood of high body mass index in early childhood. *Am J Public Health* 2012; **102**(11): 2057-67.
1735. Laurson KR, Lee JA, Gentile DA, Walsh DA, Eisenmann JC. Concurrent Associations between Physical Activity, Screen Time, and Sleep Duration with Childhood Obesity. *ISRN Obes* 2014; **2014**: 204540.
1736. Vehrs PR, Fellingham GW, McAferty A, Kelsey L. Trends in BMI Percentile and Body Fat Percentage in Children 12 to 17 Years of Age. *Children (Basel)* 2022; **9**(5).
© 2024 Zhang X et al. *JAMA Pediatrics*.

1737. Vazquez CE, McBride MJ, Hess KE, Cubbin C, Bearman SK, Calzada EJ. Examining School and Neighborhood Effects of Socioeconomic Status on Childhood Obesity in the U.S. *Int J Environ Res Public Health* 2022; **19**(10).
1738. Leung CW, Laraia BA, Kelly M, et al. The influence of neighborhood food stores on change in young girls' body mass index. *Am J Prev Med* 2011; **41**(1): 43-51.
1739. Shier V, An R, Sturm R. Is there a robust relationship between neighbourhood food environment and childhood obesity in the USA? *Public Health* 2012; **126**(9): 723-30.
1740. Salcido A, Robles EH, Chaudhary K, et al. Association of ADHD and Obesity in Hispanic Children on the US-Mexico Border: A Retrospective Analysis. *Front Integr Neurosci* 2021; **15**: 749907.
1741. Odusanya AO, Reid KS, Amutah-Onukagha N, et al. Association between sociodemographic characteristics and age stopped breastfeeding on early childhood weight status. *Obes Res Clin Pract* 2022; **16**(3): 214-9.
1742. Mayne SL, Kelleher S, Hannan C, et al. Neighborhood Greenspace and Changes in Pediatric Obesity During COVID-19. *Am J Prev Med* 2023; **64**(1): 33-41.
1743. Bader MD, Schwartz-Soicher O, Jack D, et al. More neighborhood retail associated with lower obesity among New York City public high school students. *Health Place* 2013; **23**: 104-10.
1744. Burdette HL, Whitaker RC. Neighborhood playgrounds, fast food restaurants, and crime: relationships to overweight in low-income preschool children. *Prev Med* 2004; **38**(1): 57-63.
1745. Carroll-Scott A, Gilstad-Hayden K, Rosenthal L, et al. Disentangling neighborhood contextual associations with child body mass index, diet, and physical activity: the role of built, socioeconomic, and social environments. *Soc Sci Med* 2013; **95**: 106-14.
1746. Hunt KJ, Ferguson PL, Neelon B, et al. The association between maternal pre-pregnancy BMI, gestational weight gain and child adiposity: A racial-ethnically diverse cohort of children. *Pediatr Obes* 2022; **17**(8): e12911.
1747. Fyfe-Johnson AL, Reid MM, Jiang L, et al. Social Determinants of Health and Body Mass Index in American Indian/Alaska Native Children. *Child Obes* 2023; **19**(5): 341-52.
1748. Florez KR, Day SE, Huang TT, Konty KJ, D'Agostino EM. Latino Children's Obesity Risk Varies by Place of Birth: Findings from New York City Public School Youth, 2006-2017. *Child Obes* 2022; **18**(5): 291-300.
1749. Davis B, Carpenter C. Proximity of fast-food restaurants to schools and adolescent obesity. *Am J Public Health* 2009; **99**(3): 505-10.
1750. Cardona Salazar DK, Caplin J, Whyms P, et al. Nutrition, obesity, and dental development in young adolescents in Chicago. *Am J Hum Biol* 2022; **34**(6): e23721.
1751. Galvez MP, Hong L, Choi E, Liao L, Godbold J, Brenner B. Childhood obesity and neighborhood food-store availability in an inner-city community. *Acad Pediatr* 2009; **9**(5): 339-43.
1752. Bejarano G, Brayton RP, Ranjit N, Hoelscher DM, Brown D, Knell G. Weight status and meeting the physical activity, sleep, and screen-time guidelines among Texas children: results from a population based, cross-sectional analysis. *BMC Pediatr* 2022; **22**(1): 428.
1753. Gorski Findling MT, Wolfson JA, Rimm EB, Bleich SN. Differences in the Neighborhood Retail Food Environment and Obesity Among US Children and Adolescents by SNAP Participation. *Obesity (Silver Spring)* 2018; **26**(6): 1063-71.
1754. Zhang L, Gordy XZ, Kolbo J, Johnson J. Prevalence and subgroup comparisons of obesity and severe obesity among Mississippi public school students. *Prev Med Rep* 2021; **23**: 101389.
1755. Kepper M, Tseng TS, Volaufova J, Scribner R, Nuss H, Sothern M. Pre-school obesity is inversely associated with vegetable intake, grocery stores and outdoor play. *Pediatr Obes* 2016; **11**(5): e6-8.
1756. Stine F, Collier DN, Fang X, Dew KR, Lazorick S. Impact of Body Mass Index, Socioeconomic Status, and
© 2024 Zhang X et al. *JAMA Pediatrics.*

Bedtime Technology Use on Sleep Duration in Adolescents. *Clin Pediatr (Phila)* 2021; **60**(13): 520-7.
1757. Reilly J, Zhu L, Hunt MJO, Hovarter R, Flood MB. Comparison of Rural Childhood BMI Percentiles: Prevalence and Trends in a Midwest County, 2008-2016. *J Sch Nurs* 2021; **37**(4): 298-305.
1758. Liu GC, Wilson JS, Qi R, Ying J. Green neighborhoods, food retail and childhood overweight: differences by population density. *Am J Health Promot* 2007; **21**(4 Suppl): 317-25.
1759. Imoisili O, Dooyema C, Kompaniyets L, et al. Prevalence of Overweight and Obesity Among Children Enrolled in Head Start, 2012-2018. *Am J Health Promot* 2021; **35**(3): 334-43.
1760. Mellor JM, Dolan CB, Rapoport RB. Child body mass index, obesity, and proximity to fast food restaurants. *Int J Pediatr Obes* 2011; **6**(1): 60-8.
1761. Ohri-Vachaspati P, DeLia D, DeWeese RS, Crespo NC, Todd M, Yedidia MJ. The relative contribution of layers of the Social Ecological Model to childhood obesity. *Public Health Nutr* 2015; **18**(11): 2055-66.
1762. Oreskovic NM, Kuhlthau KA, Romm D, Perrin JM. Built environment and weight disparities among children in high- and low-income towns. *Acad Pediatr* 2009; **9**(5): 315-21.
1763. Salois MJ. The built environment and obesity among low-income preschool children. *Health Place* 2012; **18**(3): 520-7.
1764. Sanchez BN, Sanchez-Vaznaugh EV, Uscilka A, Baek J, Zhang L. Differential associations between the food environment near schools and childhood overweight across race/ethnicity, gender, and grade. *Am J Epidemiol* 2012; **175**(12): 1284-93.
1765. Tang X, Ohri-Vachaspati P, Abbott JK, et al. Associations between food environment around schools and professionally measured weight status for middle and high school students. *Child Obes* 2014; **10**(6): 511-7.
1766. Wasserman JA, Suminski R, Xi J, Mayfield C, Glaros A, Magie R. A multi-level analysis showing associations between school neighborhood and child body mass index. *Int J Obes (Lond)* 2014; **38**(7): 912-8.
1767. Halfon N, Larson K, Slusser W. Associations between obesity and comorbid mental health, developmental, and physical health conditions in a nationally representative sample of US children aged 10 to 17. *Acad Pediatr* 2013; **13**(1): 6-13.
1768. Hurt L, Pinto CD, Watson J, Grant M, Gielner J. Diagnosis and screening for obesity-related conditions among children and teens receiving Medicaid--Maryland, 2005-2010. *MMWR Morb Mortal Wkly Rep* 2014; **63**(14): 305-8.
1769. Sacheck J, Goodman E, Chui K, Chomitz V, Must A, Economos C. Vitamin D deficiency, adiposity, and cardiometabolic risk in urban schoolchildren. *J Pediatr* 2011; **159**(6): 945-50.
1770. Turer CB, Lin H, Flores G. Prevalence of vitamin D deficiency among overweight and obese US children. *Pediatrics* 2013; **131**(1): e152-61.
1771. Moore CE, Liu Y. Low serum 25-hydroxyvitamin D concentrations are associated with total adiposity of children in the United States: National Health and Examination Survey 2005 to 2006. *Nutr Res* 2016; **36**(1): 72-9.
1772. Lee JM, Appugliese D, Kaciroti N, Corwyn RF, Bradley RH, Lumeng JC. Weight status in young girls and the onset of puberty. *Pediatrics* 2007; **119**(3): e624-30.
1773. Acharya K, Feese M, Franklin F, Kabagambe EK. Body mass index and dietary intake among Head Start children and caregivers. *J Am Diet Assoc* 2011; **111**(9): 1314-21.
1774. Adachi-Mejia AM, Longacre MR, Gibson JJ, Beach ML, Titus-Ernstoff LT, Dalton MA. Children with a TV in their bedroom at higher risk for being overweight. *Int J Obes (Lond)* 2007; **31**(4): 644-51.
1775. Adams A, Prince R. Correlates of physical activity in young American Indian children: lessons learned from the Wisconsin Nutrition and Growth Study. *J Public Health Manag Pract* 2010; **16**(5): 394-400.
1776. Adams AK, Quinn RA, Prince RJ. Low recognition of childhood overweight and disease risk among Native-American caregivers. *Obes Res* 2005; **13**(1): 146-52.
© 2024 Zhang X et al. *JAMA Pediatrics.*

1777. Adams AK, Tomayko EJ, K AC, et al. Predictors of Overweight and Obesity in American Indian Families With Young Children. *J Nutr Educ Behav* 2019; **51**(2): 190-8.
1778. Adams AL, Kessler JI, Deramerian K, et al. Associations between childhood obesity and upper and lower extremity injuries. *Inj Prev* 2013; **19**(3): 191-7.
1779. Ogden CL, Fryar CD, Hales CM, Carroll MD, Aoki Y, Freedman DS. Differences in Obesity Prevalence by Demographics and Urbanization in US Children and Adolescents, 2013-2016. *JAMA* 2018; **319**(23): 2410-8.
1780. Davis AM, Bennett KJ, Befort C, Nollen N. Obesity and related health behaviors among urban and rural children in the United States: data from the National Health And Nutrition Examination Survey 2003-2004 and 2005-2006. *J Pediatr Psychol* 2011; **36**(6): 669-76.
1781. Lewis RD, Meyer MC, Lehman SC, et al. Prevalence and degree of childhood and adolescent overweight in rural, urban, and suburban Georgia. *J Sch Health* 2006; **76**(4): 126-32.
1782. Singh GK, Kogan MD, Van Dyck PC, Siahpush M. Racial/ethnic, socioeconomic, and behavioral determinants of childhood and adolescent obesity in the United States: analyzing independent and joint associations. *Ann Epidemiol* 2008; **18**(9): 682-95.
1783. Davis AM, Boles RE, James RL, et al. Health behaviors and weight status among urban and rural children. *Rural Remote Health* 2008; **8**(2): 810.
1784. Agazzi H, Armstrong K, Bradley-Klug KL. BMI and physical activity among at-risk sixth- and ninth-grade students, Hillsborough County, Florida, 2005-2006. *Prev Chronic Dis* 2010; **7**(3): A48.
1785. DeBoer MD, Agard HE, Scharf RJ. Milk intake, height and body mass index in preschool children. *Arch Dis Child* 2015; **100**(5): 460-5.
1786. Govindan M, Gurm R, Mohan S, et al. Gender differences in physiologic markers and health behaviors associated with childhood obesity. *Pediatrics* 2013; **132**(3): 468-74.
1787. Wojcicki JM, Holbrook K, Lustig RH, et al. Chronic maternal depression is associated with reduced weight gain in latino infants from birth to 2 years of age. *PLoS One* 2011; **6**(2): e16737.
1788. Beck AL, Tschann J, Butte NF, Penilla C, Greenspan LC. Association of beverage consumption with obesity in Mexican American children. *Public Health Nutr* 2014; **17**(2): 338-44.
1789. Badon SE, Quesenberry CP, Xu F, Avalos LA, Hedderson MM. Gestational weight gain, birthweight and early-childhood obesity: between- and within-family comparisons. *Int J Epidemiol* 2020; **49**(5): 1682-90.
1790. Bai Y, Chen S, Laurson KR, Kim Y, Saint-Maurice PF, Welk GJ. The Associations of Youth Physical Activity and Screen Time with Fatness and Fitness: The 2012 NHANES National Youth Fitness Survey. *PLoS One* 2016; **11**(1): e0148038.
1791. Giammattei J, Blix G, Marshak HH, Wollitzer AO, Pettitt DJ. Television watching and soft drink consumption: associations with obesity in 11- to 13-year-old schoolchildren. *Arch Pediatr Adolesc Med* 2003; **157**(9): 882-6.
1792. Barlow SE, Chang JJ. Is parental aggravation associated with childhood overweight? An analysis of the National Survey of Children's Health 2003. *Acta Paediatr* 2007; **96**(9): 1360-2.
1793. Barrera CM, Perrine CG, Li R, Scanlon KS. Age at Introduction to Solid Foods and Child Obesity at 6 Years. *Child Obes* 2016; **12**(3): 188-92.
1794. Barroso CS, Roncancio A, Hinojosa MB, Reifsnider E. The association between early childhood overweight and maternal factors. *Child Obes* 2012; **8**(5): 449-54.
1795. Baxter SD, Guinn CH, Tebbs JM, Royer JA. There is no relationship between academic achievement and body mass index among fourth-grade, predominantly African-American children. *J Acad Nutr Diet* 2013; **113**(4): 551-7.
1796. Baxter SD, Royer JA, Hardin JW, Guinn CH, Devlin CM. The relationship of school absenteeism with body

mass index, academic achievement, and socioeconomic status among fourth-grade children. *J Sch Health* 2011; **81**(7): 417-23.
1797. Geier AB, Foster GD, Womble LG, et al. The relationship between relative weight and school attendance among elementary schoolchildren. *Obesity (Silver Spring)* 2007; **15**(8): 2157-61.
1798. Shore SM, Sachs ML, Lidicker JR, Brett SN, Wright AR, Libonati JR. Decreased scholastic achievement in overweight middle school students. *Obesity (Silver Spring)* 2008; **16**(7): 1535-8.
1799. Pan L, Sherry B, Park S, Blanck HM. The association of obesity and school absenteeism attributed to illness or injury among adolescents in the United States, 2009. *J Adolesc Health* 2013; **52**(1): 64-9.
1800. Kim JS, Alderete TL, Chen Z, et al. Longitudinal associations of in utero and early life near-roadway air pollution with trajectories of childhood body mass index. *Environ Health* 2018; **17**(1): 64.
1801. Seicean A, Redline S, Seicean S, et al. Association between short sleeping hours and overweight in adolescents: results from a US Suburban High School survey. *Sleep Breath* 2007; **11**(4): 285-93.
1802. Lumeng JC, Somashekar D, Appugliese D, Kaciroti N, Corwyn RF, Bradley RH. Shorter sleep duration is associated with increased risk for being overweight at ages 9 to 12 years. *Pediatrics* 2007; **120**(5): 1020-9.
1803. Miller AL, Kaciroti N, Lebourgeois MK, Chen YP, Sturza J, Lumeng JC. Sleep timing moderates the concurrent sleep duration-body mass index association in low-income preschool-age children. *Acad Pediatr* 2014; **14**(2): 207-13.
1804. Silva GE, Goodwin JL, Parthasarathy S, et al. Longitudinal association between short sleep, body weight, and emotional and learning problems in Hispanic and Caucasian children. *Sleep* 2011; **34**(9): 1197-205.
1805. Fiorito LM, Ventura AK, Mitchell DC, Smiciklas-Wright H, Birch LL. Girls' dairy intake, energy intake, and weight status. *J Am Diet Assoc* 2006; **106**(11): 1851-5.
1806. Charvet A, Huffman FG. Beverage Intake and Its Effect on Body Weight Status among WIC Preschool-Age Children. *J Obes* 2019; **2019**: 3032457.
1807. Haidar A, Ranjit N, Archer N, Hoelscher DM. Parental and peer social support is associated with healthier physical activity behaviors in adolescents: a cross-sectional analysis of Texas School Physical Activity and Nutrition (TX SPAN) data. *BMC Public Health* 2019; **19**(1): 640.
1808. Hu EY, Ramachandran S, Bhattacharya K, Nunna S. Obesity Among High School Students in the United States: Risk Factors and Their Population Attributable Fraction. *Prev Chronic Dis* 2018; **15**: E137.
1809. Drake KM, Beach ML, Longacre MR, et al. Influence of sports, physical education, and active commuting to school on adolescent weight status. *Pediatrics* 2012; **130**(2): e296-304.
1810. Suglia SF, Duarte CS, Chambers EC, Boynton-Jarrett R. Social and behavioral risk factors for obesity in early childhood. *J Dev Behav Pediatr* 2013; **34**(8): 549-56.
1811. Drake KM, Longacre MR, Dalton MA, et al. Two-method measurement for adolescent obesity epidemiology: reducing the bias in self-report of height and weight. *J Adolesc Health* 2013; **53**(3): 322-7.
1812. Pérez A, Gabriel K, Nehme EK, Mandell DJ, Hoelscher DM. Measuring the bias, precision, accuracy, and validity of self-reported height and weight in assessing overweight and obesity status among adolescents using a surveillance system. *Int J Behav Nutr Phys Act* 2015; **12 Suppl 1**(Suppl 1): S2.
1813. Ghosh-Dastidar MB, Haas AC, Nicosia N, Datar A. Accuracy of BMI correction using multiple reports in children. *BMC Obes* 2016; **3**(1): 37.
1814. Dalton WT, 3rd, Wang L, Southerland JL, Schetzina KE, Slawson DL. Self-reported versus actual weight and height data contribute to different weight misperception classifications. *South Med J* 2014; **107**(6): 348-55.
1815. Berkowitz RI, Stallings VA, Maislin G, Stunkard AJ. Growth of children at high risk of obesity during the first 6 y of life: implications for prevention. *Am J Clin Nutr* 2005; **81**(1): 140-6.
1816. Gillman MW, Rifas-Shiman SL, Kleinman K, Oken E, Rich-Edwards JW, Taveras EM. Developmental
© 2024 Zhang X et al. *JAMA Pediatrics.*

origins of childhood overweight: potential public health impact. *Obesity (Silver Spring)* 2008; **16**(7): 1651-6.
1817. Hinkle SN, Sharma AJ, Swan DW, Schieve LA, Ramakrishnan U, Stein AD. Excess gestational weight gain is associated with child adiposity among mothers with normal and overweight prepregnancy weight status. *J Nutr* 2012; **142**(10): 1851-8.
1818. Kubo A, Ferrara A, Laurent CA, et al. Associations Between Maternal Pregravid Obesity and Gestational Diabetes and the Timing of Pubarche in Daughters. *Am J Epidemiol* 2016; **184**(1): 7-14.
1819. Lindberg SM, Adams AK, Prince RJ. Early predictors of obesity and cardiovascular risk among American Indian children. *Matern Child Health J* 2012; **16**(9): 1879-86.
1820. Olson CM, Demment MM, Carling SJ, Strawderman MS. Associations Between Mothers' and Their Children's Weights at 4 Years of Age. *Child Obes* 2010; **6**(4): 201-7.
1821. Wojcicki JM, Young MB, Perham-Hester KA, de Schweinitz P, Gessner BD. Risk factors for obesity at age 3 in Alaskan children, including the role of beverage consumption: results from Alaska PRAMS 2005-2006 and its three-year follow-up survey, CUBS, 2008-2009. *PLoS One* 2015; **10**(3): e0118711.
1822. Bider-Canfield Z, Martinez MP, Wang X, et al. Maternal obesity, gestational diabetes, breastfeeding and childhood overweight at age 2 years. *Pediatr Obes* 2017; **12**(2): 171-8.
1823. Pham MT, Brubaker K, Pruett K, Caughey AB. Risk of childhood obesity in the toddler offspring of mothers with gestational diabetes. *Obstet Gynecol* 2013; **121**(5): 976-82.
1824. Ohri-Vachaspati P, Lloyd K, Delia D, Tulloch D, Yedidia MJ. A closer examination of the relationship between children's weight status and the food and physical activity environment. *Prev Med* 2013; **57**(3): 162-7.
1825. Han J, Schwartz AE, Elbel B. Does Proximity to Fast Food Cause Childhood Obesity? Evidence from Public Housing. *Reg Sci Urban Econ* 2020; **84**.
1826. Jia P, Xue H, Cheng X, Wang Y. Effects of school neighborhood food environments on childhood obesity at multiple scales: a longitudinal kindergarten cohort study in the USA. *BMC Med* 2019; **17**(1): 99.
1827. Elbel B, Tamura K, McDermott ZT, Wu E, Schwartz AE. Childhood Obesity and the Food Environment: A Population-Based Sample of Public School Children in New York City. *Obesity (Silver Spring)* 2020; **28**(1): 65-72.
1828. Reis WP, Ghamsary M, Galustian C, et al. Childhood Obesity: Is the Built Environment More Important Than the Food Environment? *Clin Med Insights Pediatr* 2020; **14**: 1179556520932123.
1829. Ohri-Vachaspati P, Acciai F, Lloyd K, et al. Evidence That Changes in Community Food Environments Lead to Changes in Children's Weight: Results from a Longitudinal Prospective Cohort Study. *J Acad Nutr Diet* 2021; **121**(3): 419-34 e9.
1830. Bailey LC, Forrest CB, Zhang P, Richards TM, Livshits A, DeRusso PA. Association of antibiotics in infancy with early childhood obesity. *JAMA Pediatr* 2014; **168**(11): 1063-9.
1831. Ville AP, Heyman MB, Medrano R, Wojcicki JM. Early Antibiotic Exposure and Risk of Childhood Obesity in Latinos. *Child Obes* 2017; **13**(3): 231-5.
1832. Theall KP, Chaparro MP, Denstel K, Bilfield A, Drury SS. Childhood obesity and the associated roles of neighborhood and biologic stress. *Prev Med Rep* 2019; **14**: 100849.
1833. Wojcicki JM, Heyman MB, Elwan D, Lin J, Blackburn E, Epel E. Early exclusive breastfeeding is associated with longer telomeres in Latino preschool children. *Am J Clin Nutr* 2016; **104**(2): 397-405.
1834. Zhu H, Bhagatwala J, Pollock NK, et al. High sodium intake is associated with short leukocyte telomere length in overweight and obese adolescents. *Int J Obes (Lond)* 2015; **39**(8): 1249-53.
1835. Harley KG, Aguilar Schall R, Chevrier J, et al. Prenatal and postnatal bisphenol A exposure and body mass index in childhood in the CHAMACOS cohort. *Environ Health Perspect* 2013; **121**(4): 514-20.
1836. Huh SY, Rifas-Shiman SL, Zera CA, et al. Delivery by caesarean section and risk of obesity in preschool age children: a prospective cohort study. *Arch Dis Child* 2012; **97**(7): 610-6.
© 2024 Zhang X et al. *JAMA Pediatrics*.

1837. Wang L, Alamian A, Southerland J, Wang K, Anderson J, Stevens M. Cesarean section and the risk of overweight in grade 6 children. *Eur J Pediatr* 2013; **172**(10): 1341-7.
1838. Peck T, Scharf RJ, Conaway MR, DeBoer MD. Viewing as little as 1 hour of TV daily is associated with higher change in BMI between kindergarten and first grade. *Obesity (Silver Spring)* 2015; **23**(8): 1680-6.
1839. Miles R, Wang Y, Johnson SB. Neighborhood Built and Social Environments and Change in Weight Status over the Summer in Low-Income Elementary School Children. *Int J Environ Res Public Health* 2018; **15**(6).
1840. Kjaer TW, Faurholt-Jepsen D, Mehta KM, et al. Shorter preschool, leukocyte telomere length is associated with obesity at age 9 in Latino children. *Clin Obes* 2018; **8**(2): 88-94.
1841. DuBose KD, Stewart EE, Charbonneau SR, Mayo MS, Donnelly JE. Prevalence of the metabolic syndrome in elementary school children. *Acta Paediatr* 2006; **95**(8): 1005-11.
1842. de Heer F, Morera O, Warren M, Chaudhari L, de Heer HD. At risk or not: comparing normative and criterion-referenced body mass index standards among Mexican American children. *Arch Latinoam Nutr* 2013; **63**(2): 126-33.
1843. Oken E, Huh SY, Taveras EM, Rich-Edwards JW, Gillman MW. Associations of maternal prenatal smoking with child adiposity and blood pressure. *Obes Res* 2005; **13**(11): 2021-8.
1844. Braun JM, Daniels JL, Poole C, et al. Prenatal environmental tobacco smoke exposure and early childhood body mass index. *Paediatr Perinat Epidemiol* 2010; **24**(6): 524-34.
1845. Mosli RH, Miller AL, Peterson KE, et al. Birth order and sibship composition as predictors of overweight or obesity among low-income 4- to 8-year-old children. *Pediatr Obes* 2016; **11**(1): 40-6.
1846. Block JP, Bailey LC, Gillman MW, et al. Early Antibiotic Exposure and Weight Outcomes in Young Children. *Pediatrics* 2018; **142**(6).
1847. Assari S, Caldwell CH. Gender and Ethnic Differences in the Association Between Obesity and Depression Among Black Adolescents. *J Racial Ethn Health Disparities* 2015; **2**(4): 481-93.
1848. Boutelle KN, Hannan P, Fulkerson JA, Crow SJ, Stice E. Obesity as a prospective predictor of depression in adolescent females. *Health Psychol* 2010; **29**(3): 293-8.
1849. Kubzansky LD, Gilthorpe MS, Goodman E. A prospective study of psychological distress and weight status in adolescents/young adults. *Ann Behav Med* 2012; **43**(2): 219-28.
1850. McClure AC, Tanski SE, Kingsbury J, Gerrard M, Sargent JD. Characteristics associated with low self-esteem among US adolescents. *Acad Pediatr* 2010; **10**(4): 238-44 e2.
1851. Roberts RE, Hao DT. Obesity has few effects on future psychosocial functioning of adolescents. *Eat Behav* 2013; **14**(2): 128-36.
1852. Borges G, Benjet C, Medina-Mora ME, Miller M. Body mass index and its relationship to mental disorders in the Mexican Adolescent Mental Health Survey. *Salud Publica Mex* 2010; **52**(2): 103-10.
1853. Vannucci A, Ohannessian CM. Body Mass Index z-Scores and Weight Status Predict Conduct Disorder Symptoms in Adolescents. *J Adolesc Health* 2017; **61**(5): 657-60.
1854. Adams AK, Harvey HE, Prince RJ. Association of maternal smoking with overweight at age 3 y in American Indian children. *Am J Clin Nutr* 2005; **82**(2): 393-8.
1855. Hack M, Schluchter M, Andreias L, et al. Change in prevalence of chronic conditions between childhood and adolescence among extremely low-birth-weight children. *Jama* 2011; **306**(4): 394-401.
1856. Vohr BR, Heyne R, Bann CM, et al. Extreme Preterm Infant Rates of Overweight and Obesity at School Age in the SUPPORT Neuroimaging and Neurodevelopmental Outcomes Cohort. *J Pediatr* 2018; **200**: 132-9 e3.
1857. Wood CT, Linthavong O, Perrin EM, et al. Antecedents of Obesity Among Children Born Extremely Preterm. *Pediatrics* 2018; **142**(5).
1858. Cottrell L, Neal WA, Ice C, Perez MK, Piedimonte G. Metabolic abnormalities in children with asthma. *Am J*
© 2024 Zhang X et al. *JAMA Pediatrics*.

Respir Crit Care Med 2011; **183**(4): 441-8.
1859. Vangeepuram N, Teitelbaum SL, Galvez MP, Brenner B, Doucette J, Wolff MS. Measures of obesity associated with asthma diagnosis in ethnic minority children. *J Obes* 2011; **2011**: 517417.
1860. Kwon HL, Ortiz B, Swaner R, et al. Childhood asthma and extreme values of body mass index: the Harlem Children's Zone Asthma Initiative. *J Urban Health* 2006; **83**(3): 421-33.
1861. Musaad SM, Patterson T, Ericksen M, et al. Comparison of anthropometric measures of obesity in childhood allergic asthma: central obesity is most relevant. *J Allergy Clin Immunol* 2009; **123**(6): 1321-7 e12.
1862. Anderson J, Hayes D, Chock L. Characteristics of overweight and obesity at age two and the association with breastfeeding in Hawai'i Women, Infants, and Children (WIC) participants. *Matern Child Health J* 2014; **18**(10): 2323-31.
1863. Taveras EM, Gillman MW, Pena MM, Redline S, Rifas-Shiman SL. Chronic sleep curtailment and adiposity. *Pediatrics* 2014; **133**(6): 1013-22.
1864. Scharf RJ, DeBoer MD. Sleep timing and longitudinal weight gain in 4- and 5-year-old children. *Pediatr Obes* 2015; **10**(2): 141-8.
1865. Storfer-Isser A, Patel SR, Babineau DC, Redline S. Relation between sleep duration and BMI varies by age and sex in youth age 8-19. *Pediatr Obes* 2012; **7**(1): 53-64.
1866. Bell JF, Zimmerman FJ. Shortened nighttime sleep duration in early life and subsequent childhood obesity. *Arch Pediatr Adolesc Med* 2010; **164**(9): 840-5.
1867. Roberts RE, Duong HT. Perceived weight, not obesity, increases risk for major depression among adolescents. *J Psychiatr Res* 2013; **47**(8): 1110-7.
1868. Anderson SE, Murray DM, Johnson CC, et al. Obesity and depressed mood associations differ by race/ethnicity in adolescent girls. *Int J Pediatr Obes* 2011; **6**(1): 69-78.
1869. Lohman BJ, Stewart S, Gundersen C, Garasky S, Eisenmann JC. Adolescent overweight and obesity: links to food insecurity and individual, maternal, and family stressors. *J Adolesc Health* 2009; **45**(3): 230-7.
1870. Foster GD, Sherman S, Borradaile KE, et al. A policy-based school intervention to prevent overweight and obesity. *Pediatrics* 2008; **121**(4): e794-802.
1871. Chomitz VR, McGowan RJ, Wendel JM, et al. Healthy Living Cambridge Kids: a community-based participatory effort to promote healthy weight and fitness. *Obesity (Silver Spring)* 2010; **18 Suppl 1**: S45-53.
1872. Shriver LH, Harrist AW, Hubbs-Tait L, Topham G, Page M, Barrett A. Weight status, physical activity, and fitness among third-grade rural children. *J Sch Health* 2011; **81**(9): 536-44.
1873. Eagle TF, Gurm R, Goldberg CS, et al. Health status and behavior among middle-school children in a midwest community: what are the underpinnings of childhood obesity? *Am Heart J* 2010; **160**(6): 1185-9.
1874. Lee JM, Kaciroti N, Appugliese D, Corwyn RF, Bradley RH, Lumeng JC. Body mass index and timing of pubertal initiation in boys. *Arch Pediatr Adolesc Med* 2010; **164**(2): 139-44.
1875. Kunin-Batson AS, Crain AL, Gunnar MR, et al. Household Income, Cortisol, and Obesity During Early Childhood: A Prospective Longitudinal Study. *J Pediatr* 2023; **252**: 76-82.
1876. Wu AJ, Aris IM, Hivert MF, et al. Association of Changes in Obesity Prevalence With the COVID-19 Pandemic in Youth in Massachusetts. *JAMA Pediatr* 2022; **176**(2): 198-201.
1877. Arcan C, Hannan PJ, Himes JH, et al. American Indian Parents' Assessment of and Concern About Their Kindergarten Child's Weight Status, South Dakota, 2005-2006. *Preventing Chronic Disease* 2012.
1878. Armstrong B, Janicke DM. Differentiating the effects of maternal and peer encouragement to diet on child weight control attitudes and behaviors. *Appetite* 2012; **59**(3): 723-9.
1879. Stettler N, Elliott MR, Kallan MJ, Auerbach SB, Kumanyika SK. High prevalence of overweight among pediatric users of community health centers. *Pediatrics* 2005; **116**(3): e381-8.
© 2024 Zhang X et al. *JAMA Pediatrics.*

1880. Rappaport EB, Robbins JM. Overweight in Southeastern Pennsylvania children: 2002 household health survey data. *Public Health Rep* 2005; **120**(5): 525-31.
1881. Kim J, Must A, Fitzmaurice GM, et al. Relationship of physical fitness to prevalence and incidence of overweight among schoolchildren. *Obes Res* 2005; **13**(7): 1246-54.
1882. Kersey M, Lipton R, Sanchez-Rosado M, Kumar J, Thisted R, Lantos JD. Breast-feeding history and overweight in Latino preschoolers. *Ambul Pediatr* 2005; **5**(6): 355-8.
1883. Flynn MA, Hall K, Noack A, et al. Promotion of healthy weights at preschool public health vaccination clinics in Calgary: an obesity surveillance program. *Can J Public Health* 2005; **96**(6): 421-6.
1884. Thorpe LE, List DG, Marx T, May L, Helgerson SD, Frieden TR. Childhood obesity in New York City elementary school students. *Am J Public Health* 2004; **94**(9): 1496-500.
1885. Patrick K, Norman GJ, Calfas KJ, et al. Diet, physical activity, and sedentary behaviors as risk factors for overweight in adolescence. *Arch Pediatr Adolesc Med* 2004; **158**(4): 385-90.
1886. Nelson JA, Chiasson MA, Ford V. Childhood overweight in a New York City WIC population. *Am J Public Health* 2004; **94**(3): 458-62.
1887. Mirza NM, Kadow K, Palmer M, Solano H, Rosche C, Yanovski JA. Prevalence of overweight among inner city Hispanic-American children and adolescents. *Obes Res* 2004; **12**(8): 1298-310.
1888. Hoelscher DM, Day RS, Lee ES, et al. Measuring the prevalence of overweight in Texas schoolchildren. *Am J Public Health* 2004; **94**(6): 1002-8.
1889. Drobac S, Brickman W, Smith T, Binns HJ. Evaluation of a type 2 diabetes screening protocol in an urban pediatric clinic. *Pediatrics* 2004; **114**(1): 141-8.
1890. Davy BM, Harrell K, Stewart J, King DS. Body weight status, dietary habits, and physical activity levels of middle school-aged children in rural Mississippi. *South Med J* 2004; **97**(6): 571-7.
1891. Trost SG, Sirard JR, Dowda M, Pfeiffer KA, Pate RR. Physical activity in overweight and nonoverweight preschool children. *Int J Obes Relat Metab Disord* 2003; **27**(7): 834-9.
1892. Jimenez-Cruz A, Bacardi-Gascon M, Spindler AA. Obesity and hunger among Mexican-Indian migrant children on the US-Mexico border. *Int J Obes Relat Metab Disord* 2003; **27**(6): 740-7.
1893. Friedlander SL, Larkin EK, Rosen CL, Palermo TM, Redline S. Decreased quality of life associated with obesity in school-aged children. *Arch Pediatr Adolesc Med* 2003; **157**(12): 1206-11.
1894. Eisenmann JC, Arnall DA, Kanuho V, McArel H. Growth status and obesity of Hopi children. *Am J Hum Biol* 2003; **15**(6): 741-5.
1895. Demerath E, Muratova V, Spangler E, Li J, Minor VE, Neal WA. School-based obesity screening in rural Appalachia. *Prev Med* 2003; **37**(6 Pt 1): 553-60.
1896. Rinderknecht K, Smith C. Body-image perceptions among urban Native American youth. *Obes Res* 2002; **10**(5): 315-27.
1897. Neumark-Sztainer D, Story M, Hannan PJ, Croll J. Overweight status and eating patterns among adolescents: where do youths stand in comparison with the healthy people 2010 objectives? *Am J Public Health* 2002; **92**(5): 844-51.
1898. Yang Y, Jiang Y, Xu Y, Mzayek F, Levy M. A cross-sectional study of the influence of neighborhood environment on childhood overweight and obesity: Variation by age, gender, and environment characteristics. *Prev Med* 2018; **108**: 23-8.
1899. Hidalgo-Mendez J, Power TG, Fisher JO, O'Connor TM, Hughes SO. Child weight status and accuracy of perceived child weight status as predictors of Latina mothers' feeding practices and styles. *Appetite* 2019; **142**: 104387.
1900. Tschamler JM, Conn KM, Cook SR, Halterman JS. Underestimation of children's weight status: views of
© 2024 Zhang X et al. *JAMA Pediatrics.*

parents in an urban community. *Clin Pediatr (Phila)* 2010; **49**(5): 470-6.
1901. Thundiyil JG, Christiano-Smith D, Greenberger S, Cramm K, Latimer-Pierson J, Modica RF. Trimming the fat: identification of risk factors associated with obesity in a pediatric emergency department. *Pediatr Emerg Care* 2010; **26**(10): 709-15.
1902. Amram O, Amiri S, Lutz RB, Crowley A, Monsivais P. The Association Between Obesity, Socio-Economic Status, and Neighborhood Environment: A Multi-Level Analysis of Spokane Public Schools. *J Community Health* 2020; **45**(1): 41-7.
1903. Strickman-Stein N, Gervais MD, Ludwig DA, Messiah SE, Lipshultz SE, Miller TL. Body mass index as a function of length of United States residency among Haitian immigrant children. *Ethn Dis* 2010; **20**(1): 22-8.
1904. Shankaran S, Bann CM, Bauer CR, et al. Prenatal cocaine exposure and BMI and blood pressure at 9 years of age. *Journal of Hypertension* 2010; **28**(6): 1166-75.
1905. Shabbir S, Kwan D, Wang MC, Shih M, Simon PA. Asians and Pacific Islanders and the growing childhood obesity epidemic. *Ethn Dis* 2010; **20**(2): 129-35.
1906. Rodriguez R, Mowrer J, Romo J, Aleman A, Weffer SE, Ortiz RM. Ethnic and gender disparities in adolescent obesity and elevated systolic blood pressure in a rural US population. *Clin Pediatr (Phila)* 2010; **49**(9): 876-84.
1907. Meininger JC, Brosnan CA, Eissa MA, et al. Overweight and central adiposity in school-age children and links with hypertension. *J Pediatr Nurs* 2010; **25**(2): 119-25.
1908. Hennessy E, Hughes SO, Goldberg JP, Hyatt RR, Economos CD. Parent behavior and child weight status among a diverse group of underserved rural families. *Appetite* 2010; **54**(2): 369-77.
1909. Gungor DE, Paul IM, Birch LL, Bartok CJ. Risky vs rapid growth in infancy: refining pediatric screening for childhood overweight. *Arch Pediatr Adolesc Med* 2010; **164**(12): 1091-7.
1910. Greves Grow HM, Cook AJ, Arterburn DE, Saelens BE, Drewnowski A, Lozano P. Child obesity associated with social disadvantage of children's neighborhoods. *Soc Sci Med* 2010; **71**(3): 584-91.
1911. Farhat T, Iannotti RJ, Simons-Morton BG. Overweight, obesity, youth, and health-risk behaviors. *Am J Prev Med* 2010; **38**(3): 258-67.
1912. Elder JP, Arredondo EM, Campbell N, et al. Individual, family, and community environmental correlates of obesity in Latino elementary school children. *J Sch Health* 2010; **80**(1): 20-30; quiz 53-5.
1913. Dammann K, Smith C. Food-related attitudes and behaviors at home, school, and restaurants: perspectives from racially diverse, urban, low-income 9- to 13-year-old children in Minnesota. *J Nutr Educ Behav* 2010; **42**(6): 389-97.
1914. Bethell C, Simpson L, Stumbo S, Carle AC, Gombojav N. National, state, and local disparities in childhood obesity. *Health Aff (Millwood)* 2010; **29**(3): 347-56.
1915. Beets MW, Foley JT. Comparison of 3 different analytic approaches for determining risk-related active and sedentary behavioral patterns in adolescents. *J Phys Act Health* 2010; **7**(3): 381-92.
1916. Bayles B. Perceptions of childhood obesity on the Texas-Mexico border. *Public Health Nurs* 2010; **27**(4): 320-8.
1917. Baranowski JC, Baranowski T, Beltran A, et al. 6-n-Propylthiouracil sensitivity and obesity status among ethnically diverse children. *Public Health Nutr* 2010; **13**(10): 1587-92.
1918. Babey SH, Hastert TA, Wolstein J, Diamant AL. Income disparities in obesity trends among California adolescents. *Am J Public Health* 2010; **100**(11): 2149-55.
1919. Villa-Caballero L, Arredondo EM, Campbell N, Elder JP. Family history of diabetes, parental body mass index predict obesity in Latino children. *Diabetes Educ* 2009; **35**(6): 959-65.
1920. Valente TW, Fujimoto K, Chou CP, Spruijt-Metz D. Adolescent affiliations and adiposity: a social network
© 2024 Zhang X et al. *JAMA Pediatrics.*

analysis of friendships and obesity. *J Adolesc Health* 2009; **45**(2): 202-4.
1921. Vader AM, Walters ST, Harris TR, Hoelscher DM. Television viewing and snacking behaviors of fourth- and eighth-grade schoolchildren in Texas. *Prev Chronic Dis* 2009; **6**(3): A89.
1922. Treuth MS, Baggett CD, Pratt CA, et al. A longitudinal study of sedentary behavior and overweight in adolescent girls. *Obesity (Silver Spring)* 2009; **17**(5): 1003-8.
1923. Trent M, Jennings JM, Waterfield G, Lyman LM, Thomas H. Finding targets for obesity intervention in urban communities: school-based health centers and the interface with affected youth. *J Urban Health* 2009; **86**(4): 571-83.
1924. Smith DT, Bartee RT, Dorozynski CM, Carr LJ. Prevalence of overweight and influence of out-of-school seasonal periods on body mass index among American Indian schoolchildren. *Prev Chronic Dis* 2009; **6**(1): A20.
1925. Santos JL, Kain J, Dominguez-Vasquez P, et al. Maternal anthropometry and feeding behavior toward preschool children: association with childhood body mass index in an observational study of Chilean families. *Int J Behav Nutr Phys Act* 2009; **6**: 93.
1926. Rundle A, Goldstein IF, Mellins RB, Ashby-Thompson M, Hoepner L, Jacobson JS. Physical activity and asthma symptoms among New York City Head Start Children. *J Asthma* 2009; **46**(8): 803-9.
1927. Raynor HA, Jelalian E, Vivier PM, Hart CN, Wing RR. Parent-reported eating and leisure-time activity selection patterns related to energy balance in preschool- and school-aged children. *J Nutr Educ Behav* 2009; **41**(1): 19-26.
1928. Nsiah-Kumi PA, Ariza AJ, Mikhail LM, Feinglass J, Binns HJ. Family history and parents' beliefs about consequences of childhood overweight and their influence on children's health behaviors. *Acad Pediatr* 2009; **9**(1): 53-9.
1929. Montgomery-Reagan K, Bianco JA, Heh V, Rettos J, Huston RS. Prevalence and correlates of high body mass index in rural Appalachian children aged 6-11 years. *Rural Remote Health* 2009; **9**(4): 1234.
1930. Martyn-Nemeth P, Penckofer S, Gulanick M, Velsor-Friedrich B, Bryant FB. The relationships among self-esteem, stress, coping, eating behavior, and depressive mood in adolescents. *Res Nurs Health* 2009; **32**(1): 96-109.
1931. Lim S, Zoellner JM, Lee JM, et al. Obesity and sugar-sweetened beverages in African-American preschool children: a longitudinal study. *Obesity (Silver Spring)* 2009; **17**(6): 1262-8.
1932. Kubik MY, Davey C, Fulkerson JA, Sirard J, Story M, Arcan C. Alternative high school students: prevalence and correlates of overweight. *Am J Health Behav* 2009; **33**(5): 600-9.
1933. Krukowski RA, West DS, Philyaw Perez A, Bursac Z, Phillips MM, Raczynski JM. Overweight children, weight-based teasing and academic performance. *Int J Pediatr Obes* 2009; **4**(4): 274-80.
1934. Hillman JB, Corathers SD, Wilson SE. Pediatricians and screening for obesity with body mass index: does level of training matter? *Public Health Rep* 2009; **124**(4): 561-7.
1935. Harnack L, Lytle L, Himes JH, Story M, Taylor G, Bishop D. Low awareness of overweight status among parents of preschool-aged children, Minnesota, 2004-2005. *Prev Chronic Dis* 2009; **6**(2): A47.
1936. Harbaugh BL, Bounds W, Kolbo J, Molaison E, Zhang L. Prevalence estimates of overweight in Head Start preschoolers. *J Pediatr Nurs* 2009; **24**(5): 350-9.
1937. Franzini L, Elliott MN, Cuccaro P, et al. Influences of physical and social neighborhood environments on children's physical activity and obesity. *Am J Public Health* 2009; **99**(2): 271-8.
1938. Forman J, Halford JC, Summe H, MacDougall M, Keller KL. Food branding influences ad libitum intake differently in children depending on weight status. Results of a pilot study. *Appetite* 2009; **53**(1): 76-83.
1939. Duncan DT, Johnson RM, Molnar BE, Azrael D. Association between neighborhood safety and overweight status among urban adolescents. *BMC Public Health* 2009; **9**: 289.
1940. Dorsey K, Herrin J, Krumholz H, Irwin M. The utility of shorter epochs in direct motion monitoring. *Res Q*
© 2024 Zhang X et al. *JAMA Pediatrics.*

Exerc Sport 2009; **80**(3): 460-8.
1941. Dixon D, Meng H, Goldberg R, Schneiderman N, Delamater A. Stress and body mass index each contributes independently to tumor necrosis factor-alpha production in prepubescent Latino children. *J Pediatr Nurs* 2009; **24**(5): 378-88.
1942. De La OA, Jordan KC, Ortiz K, et al. Do parents accurately perceive their child's weight status? *J Pediatr Health Care* 2009; **23**(4): 216-21.
1943. Chen JL. Household income, maternal acculturation, maternal education level and health behaviors of Chinese-American children and mothers. *J Immigr Minor Health* 2009; **11**(3): 198-204.
1944. Asante PA, Cox J, Sonneville K, Samuels RC, Taveras EM. Overweight prevention in pediatric primary care: a needs assessment of an urban racial/ethnic minority population. *Clin Pediatr (Phila)* 2009; **48**(8): 837-43.
1945. Anderson SE, Whitaker RC. Prevalence of obesity among US preschool children in different racial and ethnic groups. *Arch Pediatr Adolesc Med* 2009; **163**(4): 344-8.
1946. West DS, Raczynski JM, Phillips MM, Bursac Z, Heath Gauss C, Montgomery BE. Parental recognition of overweight in school-age children. *Obesity (Silver Spring)* 2008; **16**(3): 630-6.
1947. Stovitz SD, Steffen LM, Boostrom A. Participation in physical activity among normal- and overweight Hispanic and non-Hispanic white adolescents. *J Sch Health* 2008; **78**(1): 19-25.
1948. Steele JS, Buchi KF. Medical and mental health of children entering the utah foster care system. *Pediatrics* 2008; **122**(3): e703-9.
1949. Pollack KM, Xie D, Arbogast KB, Durbin DR. Body mass index and injury risk among US children 9-15 years old in motor vehicle crashes. *Inj Prev* 2008; **14**(6): 366-71.
1950. Margellos-Anast H, Shah AM, Whitman S. Prevalence of obesity among children in six Chicago communities: findings from a health survey. *Public Health Rep* 2008; **123**(2): 117-25.
1951. Laurson K, Eisenmann JC, Moore S. Lack of association between television viewing, soft drinks, physical activity and body mass index in children. *Acta Paediatr* 2008; **97**(6): 795-800.
1952. Kapoor M, Laham SG, Sawyer JR. Children at risk identified in an urban scoliosis school screening program: a new model. *J Pediatr Orthop B* 2008; **17**(6): 281-7.
1953. Kalich KA, Chomitz V, Peterson KE, McGowan R, Houser RF, Must A. Comfort and utility of school-based weight screening: the student perspective. *BMC Pediatr* 2008; **8**: 9.
1954. Irigoyen M, Glassman ME, Chen S, Findley SE. Early onset of overweight and obesity among low-income 1- to 5-year olds in New York City. *J Urban Health* 2008; **85**(4): 545-54.
1955. Eisenmann JC, Bartee RT, Smith DT, Welk GJ, Fu Q. Combined influence of physical activity and television viewing on the risk of overweight in US youth. *Int J Obes (Lond)* 2008; **32**(4): 613-8.
1956. Eichner JE, Moore WE, Perveen G, Kobza CE, Abbott KE, Stephens AL. Overweight and obesity in an ethnically diverse rural school district: the Healthy Kids Project. *Obesity (Silver Spring)* 2008; **16**(2): 501-4.
1957. Wald ER, Ewing LJ, Cluss P, et al. Parental perception of children's weight in a paediatric primary care setting. *Child Care Health Dev* 2007; **33**(6): 738-43.
1958. Roseman MG, Yeung WK, Nickelsen J. Examination of weight status and dietary behaviors of middle school students in Kentucky. *J Am Diet Assoc* 2007; **107**(7): 1139-45.
1959. Metallinos-Katsaras ES, Freedson PS, Fulton JE, Sherry B. The association between an objective measure of physical activity and weight status in preschoolers. *Obesity (Silver Spring)* 2007; **15**(3): 686-94.
1960. Martin KS, Ferris AM. Food insecurity and gender are risk factors for obesity. *J Nutr Educ Behav* 2007; **39**(1): 31-6.
1961. Malaty HM, Abudayyeh S, Fraley K, Graham DY, Gilger MA, Hollier DR. Recurrent abdominal pain in school children: effect of obesity and diet. *Acta Paediatr* 2007; **96**(4): 572-6.
© 2024 Zhang X et al. *JAMA Pediatrics.*

1962. Lynch WC, Heil DP, Wagner E, Havens MD. Ethnic differences in BMI, weight concerns, and eating behaviors: comparison of Native American, White, and Hispanic adolescents. *Body Image* 2007; **4**(2): 179-90.

1963. Hernández-Valero MA, Wilkinson AV, Forman MR, et al. Maternal BMI and country of birth as indicators of childhood obesity in children of Mexican origin. *Obesity (Silver Spring)* 2007; **15**(10): 2512-9.

1964. Eisenmann J, Arnall D, Kanuho V, Interpretter C, Coast J. Obesity and pulmonary function in Navajo and Hopi children. *Ethnicity & disease* 2007; **17**: 14-8.

1965. Akridge M, Hilgers KK, Silveira AM, Scarfe W, Scheetz JP, Kinane DF. Childhood obesity and skeletal maturation assessed with Fishman's hand-wrist analysis. *Am J Orthod Dentofacial Orthop* 2007; **132**(2): 185-90.

1966. Zephier E, Himes JH, Story M, Zhou X. Increasing prevalences of overweight and obesity in Northern Plains American Indian children. *Arch Pediatr Adolesc Med* 2006; **160**(1): 34-9.

1967. Nelson JA, Carpenter K, Chiasson MA. Diet, activity, and overweight among preschool-age children enrolled in the Special Supplemental Nutrition Program for Women, Infants, and Children (WIC). *Prev Chronic Dis* 2006; **3**(2): A49.

1968. Pobutsky AM, Hirokawa R, Zou L, Huang T, Rosen L, Wood B. Overweight and at-risk for overweight among Hawai'i public school students entering kindergarten, 2002-2003. *Hawaii Med J* 2006; **65**(10): 283-7.

1969. Mulvaney SA, Kaemingk KL, Goodwin JL, Quan SF. Parent-rated behavior problems associated with overweight before and after controlling for sleep disordered breathing. *BMC Pediatr* 2006; **6**: 34.

1970. Trappmann JL, Jimenez EY, Keane PC, Cohen DA, Davis SM. Cross-Sectional Relationships Between Household Food Insecurity and Child BMI, Feeding Behaviors, and Public Assistance Utilization Among Head Start Children From Predominantly Hispanic and American Indian Communities in the CHILE Study. *J Hunger Environ Nutr* 2015; **10**(4): 439-55.

1971. Trapp CM, Burke G, Gorin AA, et al. The relationship between dietary patterns, body mass index percentile, and household food security in young urban children. *Child Obes* 2015; **11**(2): 148-55.

1972. Tomayko EJ, Flood TL, Tandias A, Hanrahan LP. Linking electronic health records with community-level data to understand childhood obesity risk. *Pediatr Obes* 2015; **10**(6): 436-41.

1973. Scherrer PD, Mallory MD, Cravero JP, et al. The impact of obesity on pediatric procedural sedation-related outcomes: results from the Pediatric Sedation Research Consortium. *Paediatr Anaesth* 2015; **25**(7): 689-97.

1974. Park SH, Kim MJ, Park CG, McCreary L, Patil C, Norr KF. Family Factors and Body Mass Index Among Korean-American Preschoolers. *J Pediatr Nurs* 2015; **30**(6): e101-11.

1975. Nobari TZ, Wang MC, Whaley SE. Asian American mothers' perception of their children's weight: a comparison with other racial/ethnic groups in Los Angeles. *Ethn Dis* 2015; **25**(1): 52-7.

1976. Nagata JM, Hagan MJ, Heyman MB, Wojcicki JM. No Association between Obesity and Behavior in Low-income, Preschool Latino Children. *J Health Care Poor Underserved* 2015; **26**(2): 410-20.

1977. Moreno JP, Johnston CA, Chen TA, et al. Seasonal variability in weight change during elementary school. *Obesity (Silver Spring)* 2015; **23**(2): 422-8.

1978. Lumeng JC, Kaciroti N, Sturza J, et al. Changes in body mass index associated with head start participation. *Pediatrics* 2015; **135**(2): e449-56.

1979. Li Y, Robinson LE, Carter WM, Gupta R. Childhood obesity and community food environments in Alabama's Black Belt region. *Child Care Health Dev* 2015; **41**(5): 668-76.

1980. Jin Y, Jones-Smith JC. Associations between family income and children's physical fitness and obesity in California, 2010-2012. *Prev Chronic Dis* 2015; **12**: E17.

1981. Jackson JA, Smit E, Manore MM, John D, Gunter K. The Family-Home Nutrition Environment and Dietary Intake in Rural Children. *Nutrients* 2015; **7**(12): 9707-20.

1982. Gunter KB, Nader PA, John DH. Physical activity levels and obesity status of Oregon Rural Elementary

© 2024 Zhang X et al. *JAMA Pediatrics.*

School children. *Prev Med Rep* 2015; **2**: 478-82.
1983. Ansari A, Pettit K, Gershoff E. Combating Obesity in Head Start: Outdoor Play and Change in Children's Body Mass Index. *J Dev Behav Pediatr* 2015; **36**(8): 605-12.
1984. Weedn AE, Hale JJ, Thompson DM, Darden PM. Trends in obesity prevalence and disparities among low-income children in Oklahoma, 2005-2010. *Child Obes* 2014; **10**(4): 318-25.
1985. Nichols M, Stein AD, Wold JL. Health status of children of migrant farm workers: Farm Worker Family Health Program, Moultrie, Georgia. *Am J Public Health* 2014; **104**(2): 365-70.
1986. Nguyen JV, Robbins JM, Houck KL, et al. Severe obesity and high blood pressure among children, Philadelphia health centers, 2010. *J Prim Care Community Health* 2014; **5**(2): 152-5.
1987. Lo JC, Maring B, Chandra M, et al. Prevalence of obesity and extreme obesity in children aged 3-5 years. *Pediatr Obes* 2014; **9**(3): 167-75.
1988. Cui W, Zack MM, Wethington H. Health-related quality of life and body mass index among US adolescents. *Qual Life Res* 2014; **23**(7): 2139-50.
1989. Dammann KW, Smith C, Richards R. Low-income minority and homeless mothers' perceptions of their 9-13 year-old children's weight status, diet, and health. *Matern Child Health J* 2011; **15**(1): 106-14.
1990. Florin TA, Shults J, Stettler N. Perception of overweight is associated with poor academic performance in US adolescents. *J Sch Health* 2011; **81**(11): 663-70.
1991. Hill EE, Eisenmann JC, Gentile D, Holmes ME, Walsh D. The association between morning cortisol and adiposity in children varies by weight status. *J Pediatr Endocrinol Metab* 2011; **24**(9-10): 709-13.
1992. Isasi CR, Whiffen A, Campbell E, Florez Y, Freeman K, Wylie-Rosett J. High prevalence of obesity among inner-city adolescent boys in the Bronx, New York: forgetting our boys. *Prev Chronic Dis* 2011; **8**(1): A23.
1993. Mulasi-Pokhriyal U, Smith C. Investigating health and diabetes perceptions among Hmong American children, 9-18 years of age. *J Immigr Minor Health* 2011; **13**(3): 470-7.
1994. Nervik D, Martin K, Rundquist P, Cleland J. The relationship between body mass index and gross motor development in children aged 3 to 5 years. *Pediatr Phys Ther* 2011; **23**(2): 144-8.
1995. Pérez A, Hoelscher DM, Springer AE, et al. Physical activity, watching television, and the risk of obesity in students, Texas, 2004-2005. *Prev Chronic Dis* 2011; **8**(3): A61.
1996. Spruyt K, Molfese DL, Gozal D. Sleep duration, sleep regularity, body weight, and metabolic homeostasis in school-aged children. *Pediatrics* 2011; **127**(2): e345-52.
1997. Xanthopoulos MS, Borradaile KE, Hayes S, et al. The impact of weight, sex, and race/ethnicity on body dissatisfaction among urban children. *Body Image* 2011; **8**(4): 385-9.
1998. Gamble A, Waddell D, Ford MA, Bentley JP, Woodyard CD, Hallam JS. Obesity and health risk of children in the Mississippi Delta. *J Sch Health* 2012; **82**(10): 478-83.
1999. Weedn AE, Ang SC, Zeman CL, Darden PM. Obesity prevalence in low-income preschool children in Oklahoma. *Clin Pediatr (Phila)* 2012; **51**(10): 917-22.
2000. Dodd AH, Briefel R, Cabili C, Wilson A, Crepinsek MK. Disparities in consumption of sugar-sweetened and other beverages by race/ethnicity and obesity status among United States schoolchildren. *J Nutr Educ Behav* 2013; **45**(3): 240-9.
2001. Hayes HM, Eisenmann JC, Pfeiffer K, Carlson JJ. Weight status, physical activity, and vascular health in 9- to 12-year-old children. *J Phys Act Health* 2013; **10**(2): 205-10.
2002. Loth KA, MacLehose RF, Fulkerson JA, Crow S, Neumark-Sztainer D. Food-related parenting practices and adolescent weight status: a population-based study. *Pediatrics* 2013; **131**(5): e1443-50.
2003. Novotny R, Oshiro CE, Wilkens LR. Prevalence of Childhood Obesity among Young Multiethnic Children from a Health Maintenance Organization in Hawaii. *Child Obes* 2013; **9**(1): 35-42.

© 2024 Zhang X et al. *JAMA Pediatrics.*

2004. Nunez-Gaunaurd A, Moore JG, Roach KE, Miller TL, Kirk-Sanchez NJ. Motor proficiency, strength, endurance, and physical activity among middle school children who are healthy, overweight, and obese. *Pediatr Phys Ther* 2013; **25**(2): 130-8; discussion 9.
2005. Pan L, May AL, Wethington H, Dalenius K, Grummer-Strawn LM. Incidence of obesity among young U.S. children living in low-income families, 2008-2011. *Pediatrics* 2013; **132**(6): 1006-13.
2006. Novotny R, Li F, Fialkowski MK, et al. Prevalence of obesity and acanthosis nigricans among young children in the children's healthy living program in the United States Affiliated Pacific. *Medicine (Baltimore)* 2016; **95**(37): e4711.
2007. Stiefel EC, Field L, Replogle W, McIntyre L, Igboechi O, Savoie FH, 3rd. The Prevalence of Obesity and Elevated Blood Pressure in Adolescent Student Athletes From the State of Mississippi. *Orthop J Sports Med* 2016; **4**(2): 2325967116629368.
2008. Zeller MH, Becnel J, Reiter-Purtill J, Peugh J, Wu YP. Associations Among Excess Weight Status and Tobacco, Alcohol, and Illicit Drug Use in a Large National Sample of Early Adolescent Youth. *Prev Sci* 2016; **17**(4): 483-92.
2009. Novotny R, Li F, Leon Guerrero R, et al. Dual burden of malnutrition in US Affiliated Pacific jurisdictions in the Children's Healthy Living Program. *BMC Public Health* 2017; **17**(1): 483.
2010. Sadeghi B, Schaefer S, Tseregounis IE, et al. Prevalence and Perception of Childhood Obesity in California's Farmworker Communities. *J Community Health* 2017; **42**(2): 377-84.
2011. DuBose KD, Gross McMillan A, Wood AP, Sisson SB. Joint Relationship Between Physical Activity, Weight Status, and Motor Skills in Children Aged 3 to 10 Years. *Percept Mot Skills* 2018; **125**(3): 478-92.
2012. Beal T, Le TD, Trinh HT, et al. Child Overweight or Obesity Is Associated with Modifiable and Geographic Factors in Vietnam: Implications for Program Design and Targeting. *Nutrients* 2020; **12**(5).
2013. Le GB, Dinh DX. Prevalence and associated factors of overweight and obesity among primary school children: a cross-sectional study in Thanhhoa City, Vietnam. *BMJ Open* 2022; **12**(4): e058504.
2014. Nguyen T, Sokal-Gutierrez K, Lahiff M, Fernald L, Ivey SL. Early childhood factors associated with obesity at age 8 in Vietnamese children: The Young Lives Cohort Study. *BMC Public Health* 2021; **21**(1): 301.
2015. Dieu HT, Dibley MJ, Sibbritt D, Hanh TT. Prevalence of overweight and obesity in preschool children and associated socio-demographic factors in Ho Chi Minh City, Vietnam. *Int J Pediatr Obes* 2007; **2**(1): 40-50.
2016. Phan HD, Nguyen TNP, Bui PL, et al. Overweight and obesity among Vietnamese school-aged children: National prevalence estimates based on the World Health Organization and International Obesity Task Force definition. *PLoS One* 2020; **15**(10): e0240459.
2017. Chuc DV, Hung NX, Trang VT, Linh DV, Khue PM. Nutritional Status of Children Aged 12 to 36 Months in a Rural District of Hungyen Province, Vietnam. *Biomed Res Int* 2019; **2019**: 6293184.
2018. Huynh G, Huynh QHN, Nguyen NHT, Do QT, Khanh Tran V. Malnutrition among 6-59-Month-Old Children at District 2 Hospital, Ho Chi Minh City, Vietnam: Prevalence and Associated Factors. *Biomed Res Int* 2019; **2019**: 6921312.
2019. Pham TTP, Matsushita Y, Dinh LTK, et al. Prevalence and associated factors of overweight and obesity among schoolchildren in Hanoi, Vietnam. *BMC Public Health* 2019; **19**(1): 1478.
2020. Tang KH, Nguyen HH, Dibley MJ, Sibbritt DW, Phan NT, Tran TM. Factors associated with adolescent overweight/obesity in Ho Chi Minh city. *Int J Pediatr Obes* 2010; **5**(5): 396-403.
2021. Mai TMT, Pham NO, Tran TMH, et al. The double burden of malnutrition in Vietnamese school-aged children and adolescents: a rapid shift over a decade in Ho Chi Minh City. *Eur J Clin Nutr* 2020; **74**(10): 1448-56.
2022. Pham NK, Sepehri A, Le TM, Tran VT. Correlates of body mass index among primary school children in Ho Chi Minh City, Vietnam. *Public Health* 2020; **181**: 65-72.
© 2024 Zhang X et al. *JAMA Pediatrics.*

2023. Nguyen TH, Tang HK, Kelly P, van der Ploeg HP, Dibley MJ. Association between physical activity and metabolic syndrome: a cross sectional survey in adolescents in Ho Chi Minh City, Vietnam. *BMC Public Health* 2010; **10**: 141.
2024. Dieu HT, Dibley MJ, Sibbritt DW, Hanh TT. Trends in overweight and obesity in pre-school children in urban areas of Ho Chi Minh City, Vietnam, from 2002 to 2005. *Public Health Nutr* 2009; **12**(5): 702-9.
2025. Tang HK, Dibley MJ, Sibbritt D, Tran HM. Gender and socio-economic differences in BMI of secondary high school students in Ho Chi Minh city. *Asia Pac J Clin Nutr* 2007; **16**(1): 74-83.
2026. Hong TK, Dibley MJ, Sibbritt D, Binh PN, Trang NH, Hanh TT. Overweight and obesity are rapidly emerging among adolescents in Ho Chi Minh City, Vietnam, 2002-2004. *Int J Pediatr Obes* 2007; **2**(4): 194-201.
2027. Trang NH, Hong TK, HP VDP, Hardy LL, Kelly PJ, Dibley MJ. Longitudinal physical activity changes in adolescents: Ho Chi Minh City Youth Cohort. *Med Sci Sports Exerc* 2012; **44**(8): 1481-9.
2028. Nguyen PV, Hong TK, Hoang T, Nguyen DT, Robert AR. High prevalence of overweight among adolescents in Ho Chi Minh City, Vietnam. *BMC Public Health* 2013; **13**: 141.
2029. Hoang NTD, Orellana L, Le TD, et al. Anthropometric Status among 6(-)9-Year-Old School Children in Rural Areas in Hai Phong City, Vietnam. *Nutrients* 2018; **10**(10).
2030. Ngan HTD, Tuyen LD, Phu PV, Nambiar S. Childhood overweight and obesity amongst primary school children in Hai Phong City, Vietnam. *Asia Pac J Clin Nutr* 2018; **27**(2): 399-405.
2031. Badi MA, Garcia-Triana BE, Suarez-Martinez R. Overweight/obesity and hypertension in schoolchildren aged 6-16 years, Aden Governorate, Yemen, 2009. *East Mediterr Health J* 2012; **18**(7): 718-22.
2032. Raja'a YA, Bin Mohanna MA. Overweight and obesity among schoolchildren in Sana'a City, Yemen. *Ann Nutr Metab* 2005; **49**(5): 342-5.
2033. Kambondo G, Sartorius B. Risk Factors for Obesity and Overfat among Primary School Children in Mashonaland West Province, Zimbabwe. *Int J Environ Res Public Health* 2018; **15**(2).

© 2024 Zhang X et al. *JAMA Pediatrics.*
