# Supplementary material for: Global Prevalence of Overweight and Obesity in Children and Adolescents: A Systematic Review and Meta-Analysis
Source: JAMA Pediatr. 2024 Jun 10;178(8):800–13. doi: 10.1001/jamapediatrics.2024.1576 (PMC11165417; doi:10.1001/jamapediatrics.2024.1576)
Supplement: Supplement 2. — Data sharing statement [file jamapediatr-e241576-s002.pdf]

Data Sharing Statement

Zhang. Global Prevalence of Overweight and Obesity in Children and Adolescents. *JAMA Pediatr*. Published June 10, 2024. doi:10.1001/jamapediatrics.2024.1576

Data
Data available: No
